# Bis(trifluoromethylthio)carbene as a toolbox for versatile carbene transformations

**DOI:** 10.1038/s41467-026-76124-z

**Published:** 2026-07-29

**Authors:** Jun Sun, Qin-Yuan Ni, Yu-Cheng Gu, Jin-Hong Lin, Ji-Chang Xiao

**Affiliations:** 1 State Key Laboratory of Fluorine and Nitrogen Chemistry and Advanced Materials, Shanghai Institute of Organic Chemistry, University of Chinese Academy of Sciences, Chinese Academy of Sciences, Shanghai, China; 2 Syngenta Jealott’s Hill International Research Centre, Bracknell, UK; 3 Department of Chemistry, Shanghai University, Shanghai, China

**Keywords:** Reactive precursors, Synthetic chemistry methodology

## Abstract

Carbenes are highly valued for their broad utility across diverse fields, and their chemistry has consequently been the subject of extensive research. Taming the high reactivity of these divalent carbon intermediates to achieve controllable selectivity for diverse transformations remains a fundamental challenge. This often necessitates bespoke, fine-tuned conditions for each reaction type, limiting generality and efficiency. Here, we report the rational design of a versatile, electrophilic carbene—bis(trifluoromethylthio)carbene (:C(SCF_3_)_2_)—generated from a scalable reagent (BrCH(SCF_3_)_2_, >30 g/batch). We show that this single carbene species efficiently mediates nearly all classic free carbene reactions, including cyclopropanation, skeletal editing, X–H insertion, sulfur ylide-based [2,3]-σ rearrangement/1,2-migration, and nitrogen ylide-initiated [4+1] cyclization, under one set of mild conditions with high chemoselectivity. This versatility may be attributed to the synergistic design of the –SCF_3_ substituent, which concurrently provides strong electron withdrawal, a polarizable sulfur atom, and a bulky trifluoromethyl group. Our work establishes a unified synthetic platform that extends beyond specific reaction development, suggesting a potential paradigm for controlling reactive intermediates through rational substituent design, which may help streamline the synthesis of functional molecules.

## Abstract (web summary)

Here, the authors report the design of a versatile, electrophilic carbene—bis(trifluoromethylthio)carbene (:C(SCF_3_)_2_)—generated from a scalable reagent (BrCH(SCF_3_)_2_, >30 g/batch). The authors show that this single carbene species efficiently mediates nearly all classic free carbene reactions, including cyclopropanation and X–H insertion, under one set of mild conditions with high chemoselectivity.

## Introduction

Organic reactions essentially involve the cleavage and recombination of chemical bonds such as carbon-carbon (C–C) or carbon-heteroatom (C–X) bonds. During the bond-cleavage process, reactive intermediates such as carbocations^1^, carbanions^2^, radicals^3^, and carbenes^4^ are often generated. Carbenes, neutral divalent carbon species with only six valence electrons, can form two new bonds from a single carbon center, participating in diverse transformations such as [2+1] cycloadditions^5,6^, X–H (X = C, N, O, S) bond insertions^7^, ylide formation and subsequent transformations^8–10^, and transition-metal-catalyzed carbene transfer^11,12^. The reactivity profile of a carbene (which can range from nucleophilic to electrophilic) is critically dependent on its substituents. For some carbenes that exhibit electrophilic character, overly strong electrophilicity can lead to indiscriminate reactions with alkenyl groups, X-H bonds, or heteroatoms, resulting in poor chemoselectivity^13,14^. Therefore, transition metal/ligand systems have been commonly employed to adjust the reactivity of carbenes^15,16^. Fine-tuning reaction conditions can also suppress competing reactions and enhance kinetic selectivity^17,18^. Nonetheless, the universality of these strategies is limited by complex experimental procedures or a narrow substrate scope.

**Fig. 1:**
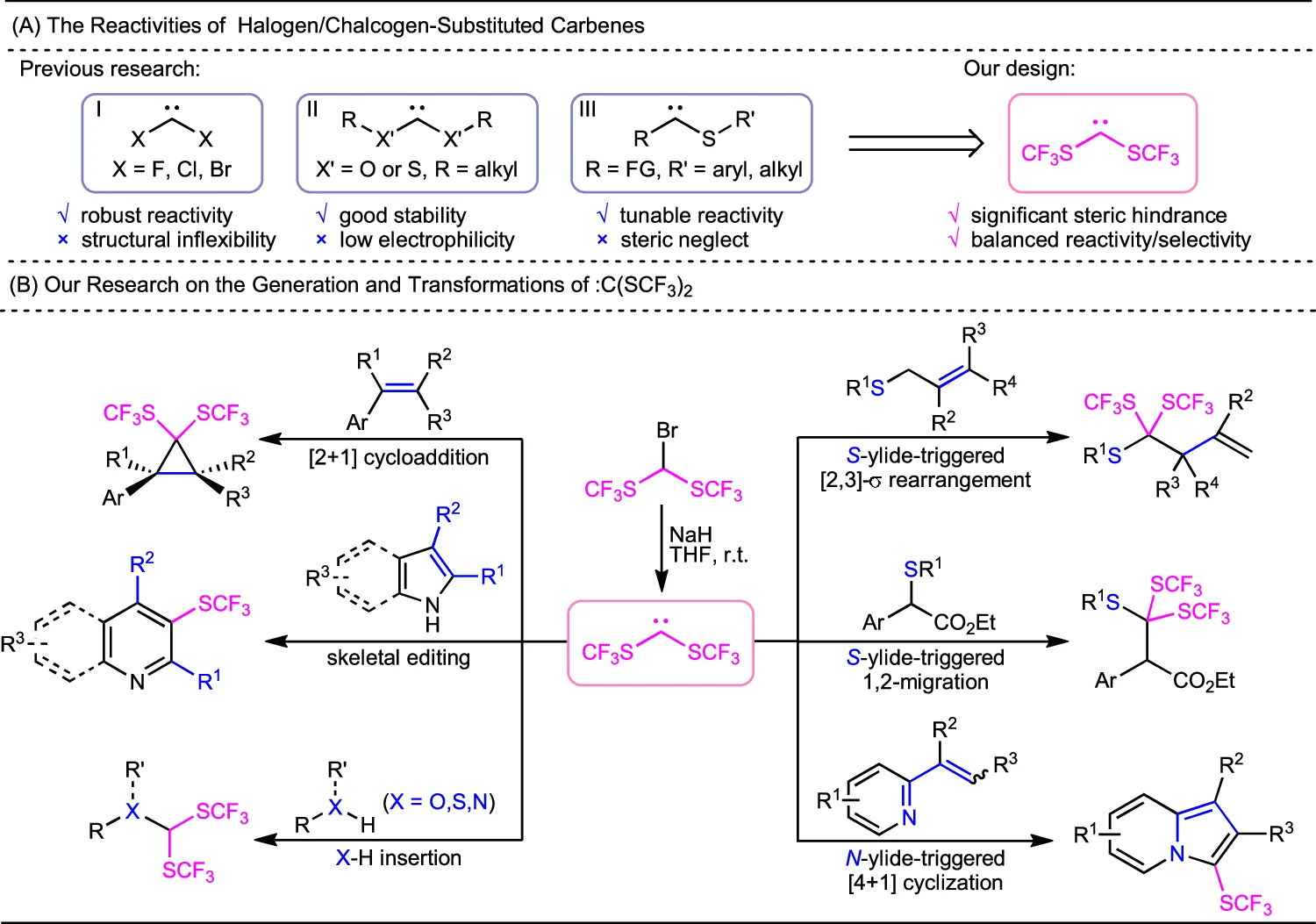
The reactivities of bis(trifluoromethylthio)carbene. **A** Previous research on halogen/chalcogen-substituted carbenes and our design strategy for bis(trifluoromethylthio)carbene (:C(SCF_3_)_2_), FG = functional groups (e.g., SO_2_Ph, CN, P(O)(OEt)_2_, alkyne, SiPh_2_^*t*^Bu, H). **B** Our research on the generation and transformations of bis(trifluoromethylthio)carbene, THF = tetrahydrofuran.

The reactivity of carbenes largely depends on their structure. However, studies on the relationship between the electronic and steric effects of substituents on the carbene carbon and the reactivity of carbenes have been limitedly explored. Previous work by Moss correlated stronger electron-donating resonance (*σ*_R⁺_) or electron-withdrawing inductive (*σ*_I_) effects of substituents with enhanced carbene’s selectivity^19^, while Arduengo et al. demonstrated that bulky substituent confers carbene’s persistence by increasing kinetic barriers^20^. Accordingly, we envisioned that synergistic modulation of both electronic structure and steric hindrance on the substituents could precisely tune carbene reactivity while improving reaction generality. In the search for such synergistic modulation, halogen atoms, with their classic dual electronic character, offer an initial prototype. While halogen atoms impart dihalocarbenes with moderate stability and robust reactivity via their dual inductive withdrawal and conjugative donation^21–23^, their monocoordinate nature prevents further substitution, limiting opportunities for reactivity fine-tuning (Fig. 1A, Previous research, I). Chalcogens may offer inductive withdrawal; meanwhile, their strong conjugation stabilizes bis(alkylthio)carbenes well, which often diminishes electrophilicity^24,25^ (Fig. 1A, Previous research, II). Therefore, enhancing the chalcogen’s inductive withdrawal while mitigating its conjugative donation represents a potential strategy to fine-tune carbene properties. Such electronic modulation is conceptually related to the Hard and Soft Acids and Bases (HSAB) theory^26^, and tuning the polarizability of the chalcogen atom’s electron cloud may therefore improve the selectivity of carbene reactions. Recently, Sharma et al. have systematically demonstrated the tunable reactivity of sulfenylcarbenes through electronic modulation^27,28^; however, steric effects have received less attention in those studies, leaving this aspect underexplored (Fig. 1A, Previous research, III).

In light of the above, we identified the trifluoromethylthio group (–SCF_3_) as a promising substituent for carbene reactivity control. The –SCF_3_ group is strongly electron-withdrawing (*σ*_m_ = 0.40, *σ*_p_ = 0.50)^29^, which is conducive to maintaining a carbene’s electrophilicity. Its sulfur atom, with a highly polarizable electron cloud, enhances thermodynamic stability via conjugation, potentially favoring reactions at soft sites in certain cases. The trifluoromethyl moiety contributes significant steric hindrance^30,31^, promoting regiodiscrimination and chemoselectivity across sterically distinct sites. On the other hand, the –SCF_3_ motif has proven valuable in drug discovery and agrochemistry, with numerous bioactive molecules bearing this group showing promising therapeutic potential^32–36^, and agrochemicals such as Flupentiofenox and Vaniliprole already in practical use^37^. Given this background, we proposed that the bis(trifluoromethylthio)carbene (:C(SCF_3_)_2_), leveraging the synergistic electronic and steric effects of its two –SCF_3_ groups, could enable efficient construction of a range of SCF_3_-containing molecules. (Fig. 1A, Our design).

In 1994, Hass et al. reported the generation of bis(trifluoromethylthio)carbene via low-temperature photolysis of bis(trifluoromethylthio)ketene. While this photochemical method is effective, its application was limited by the relatively laborious synthesis of the precursor^38^. We reason that an alternative route to this carbene can rely on base-promoted *α*-elimination, requiring bis(trifluoromethylthio)halomethane as precursor. However, direct double substitution on polyhalomethanes suffers from poor efficiency and generates complex mixtures^39^. Instead, we turned to an existing bis(trifluoromethylthio) building block. Building upon our previously established hundred-gram-scale synthesis of bis(trifluoromethylthio)thiocarbonate ((CF_3_S)_2_C=S)^40^, we successfully prepared the key reagent, bis(trifluoromethylthio)bromomethane (BrCH(SCF_3_)_2_, >30 g per batch). This reagent cleanly releases the target carbene :C(SCF_3_)_2_ upon treatment with base under ambient temperature, without the need for photoexcitation or transition metal catalysts. Once generated, this carbene exhibits diverse reactivity under unified conditions: it effectively undergoes [2+1] cycloaddition with aryl alkenes, drives regioselective ring-expansion editing of indoles/pyrroles, inserts into aryl X–H bonds, efficiently coordinates with thioethers to trigger subsequent [2,3]-σ rearrangement or 1,2-migration, and coordinates with the nitrogen atom of 2-vinylpyridines to undergo [4+1] cyclization. The synergistic effects of the strong electron-withdrawing nature of the –SCF_3_ group, the highly polarizable sulfur atom, and the steric bulk of the trifluoromethyl group appear to modulate the carbene’s electrophilic reactivity and chemical selectivity, allowing transformations of diverse substrates under identical reaction conditions. This readily accessible carbene intermediate can facilitate a range of common types of free carbene reactions, providing a synthetic pathway for constructing functional molecules containing the –SCF_3_ group (Fig. 1B).

## Results

### Preparation of the carbene precursor

**Fig. 2:**
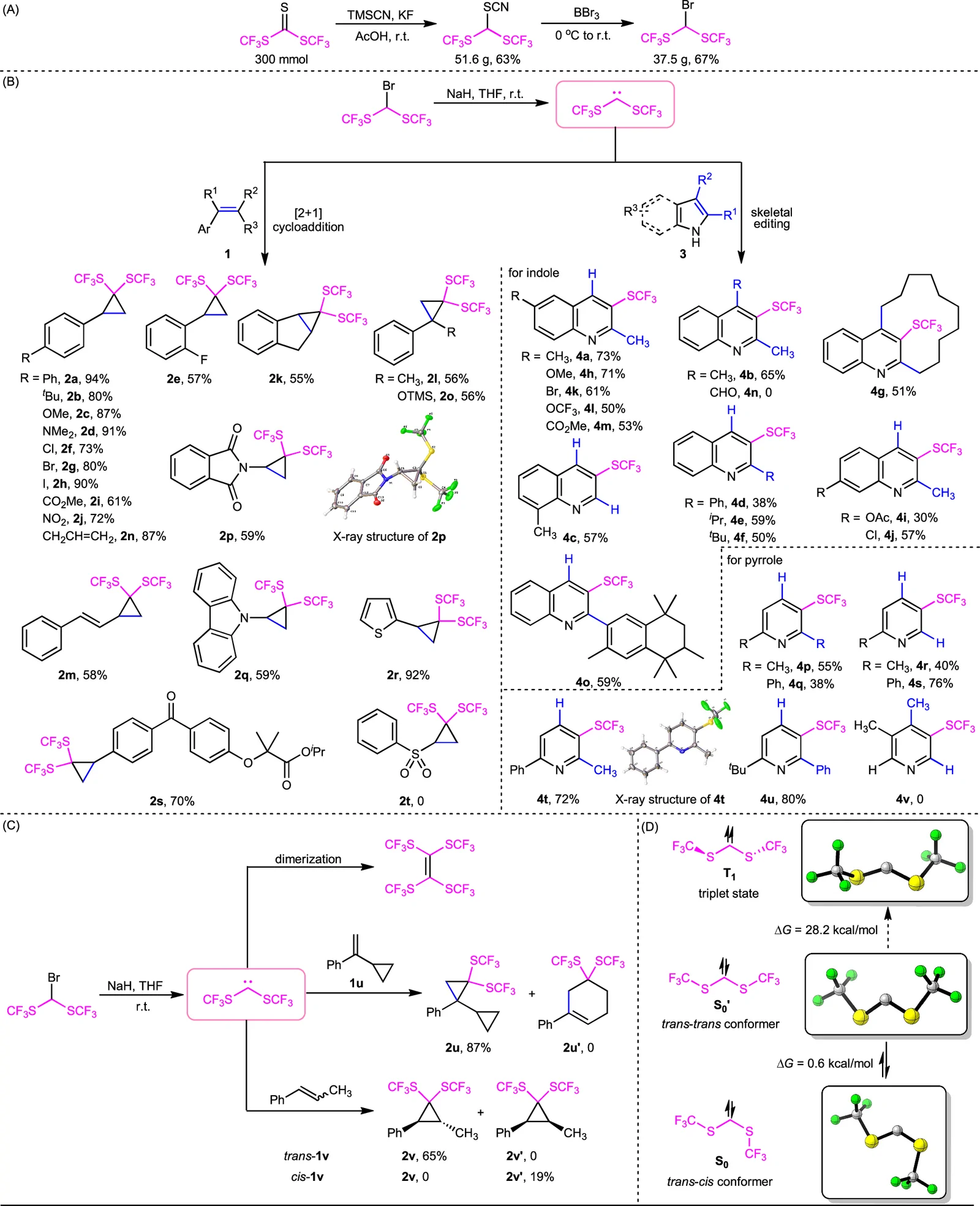
The preparation of BrCH(SCF_3_)_2_, [2+1] Cycloaddition, skeletal editing induced by :C(SCF_3_)_2_ and experimental and theoretical features of :C(SCF_3_)_2_. **A** The preparation of BrCH(SCF_3_)_2_, TMS = trimethylsilyl. **B** [2+1] Cycloaddition and skeletal editing induced by :C(SCF_3_)_2_. Typical reaction conditions: substrates **1** or **3** (0.5 mmol, 1.0 equiv.), BrCH(SCF_3_)_2_ (1.5 mmol, 3.0 equiv.), NaH (3.0 mmol, 6.0 equiv.), THF (7.5 mL), N_2_, r.t., 24 h. **C** Experimental evidence for the generation of :C(SCF_3_)_2_ and its concerted cyclopropanation pathway. **D** Theoretical calculations on the electronic structure and steric configuration of :C(SCF_3_)_2_.

We initiated the synthesis of the carbene precursor bis(trifluoromethylthio)bromomethane (BrCH(SCF_3_)_2_) from bis(trifluoromethylthio)thiocarbonate ((CF_3_S)_2_C=S)^40^ via a two-step addition-substitution sequence. By gradually adding trimethylsilyl cyanide (TMSCN) dropwise to the thiocarbonate, the addition intermediate 1-thiocyanato-1,1-bis(trifluoromethylthio)methane ((CF_3_S)_2_CHSCN) was obtained (63%, >50 g/batch), avoiding the use of highly toxic potassium cyanide (KCN)^41^. All operations were performed in a well-ventilated fume hood due to HCN generation. Subsequently, using boron tribromide (BBr_3_) as the brominating agent, efficient substitution of the thiocyanato group was achieved, yielding the final product BrCH(SCF_3_)_2_ (67%, >30 g per batch) (Fig. 2A).

### Exploration of carbene transformations

We systematically explored the generation of bis(trifluoromethylthio)carbene (:C(SCF_3_)_2_) and its cyclopropanation with the use of 4-phenylstyrene as a model substrate (Supplementary Table 1, see Supplementary Information for details). Through screening, THF (tetrahydrofuran) was identified as the suitable solvent and NaH as the optimal base (Supplementary Table 1, entry 20). A 2:1 ratio of NaH to BrCH(SCF_3_)_2_ was used to ensure complete carbene generation, and NaH was weighed under nitrogen due to its moisture sensitivity. Electron-rich styrenes reacted more efficiently, delivering the desired cyclopropanes in higher yields (Fig. 2B, left, **2a**-**2d**); electron-deficient counterparts also participated smoothly, affording the products in moderate to good yields (**2e**-**2f, 2i**-**2j**). Even more sterically hindered 1,2-disubstituted (**2k**) and 1,1-disubstituted styrenes (**2l**) underwent conversion with moderate yields. In conjugated dienes, the carbene preferentially attacked the less hindered terminal alkene. The initial formation of the bis(trifluoromethylthio)cyclopropane structure, due to its significant steric hindrance^40^, prevented further cycloaddition, even with an excess of carbene precursor (**2m**). For non-conjugated alkenes, cyclopropanation selectively occured at the phenyl-substituted double bond, while the alkyl-substituted double bond seemed less reactive toward the bis(trifluoromethylthio)carbene, enabling controlled mono-addition (**2n**). Furthermore, olefins substituted with O/N heteroatoms or a thienyl group were all successfully transformed (**2o-2r**). The single-crystal structure of compound **2p** unambiguously confirmed the structure of the cyclopropanation product (CCDC 2516953 contains the supplementary crystallographic data of compound **2p**). The cyclopropanation of a fenofibrate derivative (a cholesterol-lowering drug) further demonstrates the potential of this reaction for late-stage functionalization of bioactive molecules (**2s**). Strongly electron‑deficient alkene **1t** gave no conversion, as electron‑withdrawing substituent disfavors electrophilic cycloaddition.

Without the presence of an olefin substrate in the above reaction, tetrakis(trifluoromethylthio)ethylene ((CF_3_S)_2_C=C(SCF_3_)_2_), formed via carbene dimerization, can be detected, which confirms the in situ formation of :C(SCF_3_)_2_. When a radical clock substrate cyclopropyl ethylene (**1u**) was employed, only the direct cyclopropanation product **2u** was obtained, with no observation of the radical-induced ring-expansion side product **2u'**, which implies that the reaction does not proceed via a radical path. Furthermore, both *cis*- and *trans*-alkenes (**1v**) yielded a single configuration product with >99% stereoretention (Fig. 2C). These results collectively demonstrate that the :C(SCF_3_)_2_ generated in this system is a singlet carbene, and thus its cyclopropanation with alkenes proceeds via a concerted mechanism rather than a radical pathway. Theoretical calculations at (U)DLPNO-CCSD(T)^42^/def2-QZVPP^43^//(U)M06-2X^44^/def2-QZVP^43^ level (Fig. 2D, Supplementary Table 3, see Supplementary Information for computational details and Source Data for Cartesian coordinates and raw computational data) also demonstrate that the ground state of :C(SCF_3_)_2_ is a singlet, with all C and S atoms coplanar. The Gibbs free energy of the *trans*-*cis* conformer (**S**_**0**_) is 0.6 kcal/mol lower than that of the *trans*-*trans* conformer (**S**_**0**_**'**). In the singlet *trans*-*cis* conformer (**S**_**0**_), the average bond length of the central C-S bond is 1.63 Å, significantly shorter than the corresponding average bond length (1.81 Å) in bis(trifluoromethylthio)methane (CH_2_(SCF_3_)_2_), indicating that the C-S bond in this carbene :C(SCF_3_)_2_ has substantial double bond character. Additionally, a large singlet-triplet gap of 28.8 kcal/mol (**T**_**1**_ vs. **S**_**0**_) makes it unfavorable for this carbene to participate in subsequent reactions as a triplet.

Skeletal editing reactions triggered by carbene-involved cyclopropanation hold unique appeal in organic synthesis. As early as 1881, Ciamician et al. discovered that pyrrole could be converted into 3-chloropyridine in a chloroform/potassium *tert*-butoxide reaction system^45^. In recent years, chloro/bromo-estercarbenes^46^, arylchlorocarbenes^47^, bromofluorocarbene^48^, and functionalized sulfenylcarbene^27^ have been employed for indole/pyrrole skeletal editing. The common reaction process involves a [2+1] cycloaddition of carbene (or metal carbene) with a heterocycle, yielding a fused azabicyclic intermediate, which could subsequently undergo a base-induced ring opening to afford 3-functionalized quinoline/pyridine products. This strategy enables the precise conversion of the heterocyclic core from indole/pyrrole to quinoline/pyridine while preserving the existing functional groups, thereby circumventing the multi-step procedures of traditional de novo synthesis. However, when unsymmetrical pyrrole substrates are employed, the high reactivity of carbene often leads to poor regioselectivity in the initial cyclopropanation step, potentially generating two pyridine isomers with similar polarity^47,48^, which complicates subsequent purification.

Therefore, we were interested in exploring the skeletal editing reactions involving :C(SCF_3_)_2_ to investigate its electrophilicity and selectivity. It was found that without modifying the above optimized conditions for generating this carbene in the olefin cyclopropanation, the skeletal editing reactions involving :C(SCF_3_)_2_ can be successfully achieved, enabling the regioselective and efficient one-step synthesis of 3-trifluoromethylthioquinoline/pyridine compounds. For indole substrates, this strategy exhibited excellent tolerance to steric hindrance. Numerous 2-alkyl/aryl- and 2,3-macrocyclic substituents would not inhibit product formation (Fig. 2B, right, **4a-4g**). The reaction was largely insensitive to the electronic effects on the indole benzene ring, as substrates with electron-donating or electron-withdrawing groups afforded the target products in moderate to excellent yields (**4h-4m**). Notably, indole **3n** with a strong electron-withdrawing substituent at the C3 position did not afford the corresponding quinoline product, presumably because the reduced electron density on the pyrrole ring hampers the cyclopropanation step that initiates the skeletal editing. The successful transformation of an indole derivative of tonalide (**4o**) highlights the potential of this reaction for the late-stage derivatization of complex molecules. Regarding pyrrole substrates, the reaction tolerated various alkyl- or aryl-substituted pyrroles bearing substituents at the 2- and/or 5-positions (**4p-4u**). Notably, single-crystal X-ray diffraction analysis of compound **4t** confirmed that the skeletal editing of unsymmetrical pyrroles occurs regiospecifically at the less hindered position (**4r-4u**) (CCDC 2516954 contains the supplementary crystallographic data of compound **4t**). This stands in sharp contrast to some literature reports where two isomeric 3-functionalized pyridines are formed simultaneously^47,48^. This high regioselectivity is presumably due to the substantial steric bulk of the bis(trifluoromethylthio)methylene group, which kinetically favors the attack of the :C(SCF_3_)_2_ carbene at the less hindered site of the pyrrole ring. However, 3,4-dimethylpyrrole (**3v**) reacted poorly, likely due to steric hindrance at the C3 position of the pyrrole impeding the initial cycloaddition with the bis(trifluoromethylthio)carbene.

It can be seen that the incorporation of trifluoromethylthio group strikes an effective balance between the electrophilic reactivity and selectivity of the carbene. Furthermore, the :C(SCF_3_)_2_-involved skeletal editing of indoles/pyrroles establishes an efficient route to valuable SCF_3_-functionalized molecules. Given the unique physicochemical properties of the –SCF_3_ group, the resulting 3-(trifluoromethylthio)quinolines/pyridines are promising target molecules for pharmaceutical development. However, existing synthetic methods typically require pre-functionalized substrates or highly reactive reagents^49,50^. In contrast, this :C(SCF_3_)_2_-involved skeletal editing of indoles/pyrroles offers a significantly more streamlined and efficient method for synthesizing 3-(trifluoromethylthio)quinolines/pyridines.

**Fig. 3:**
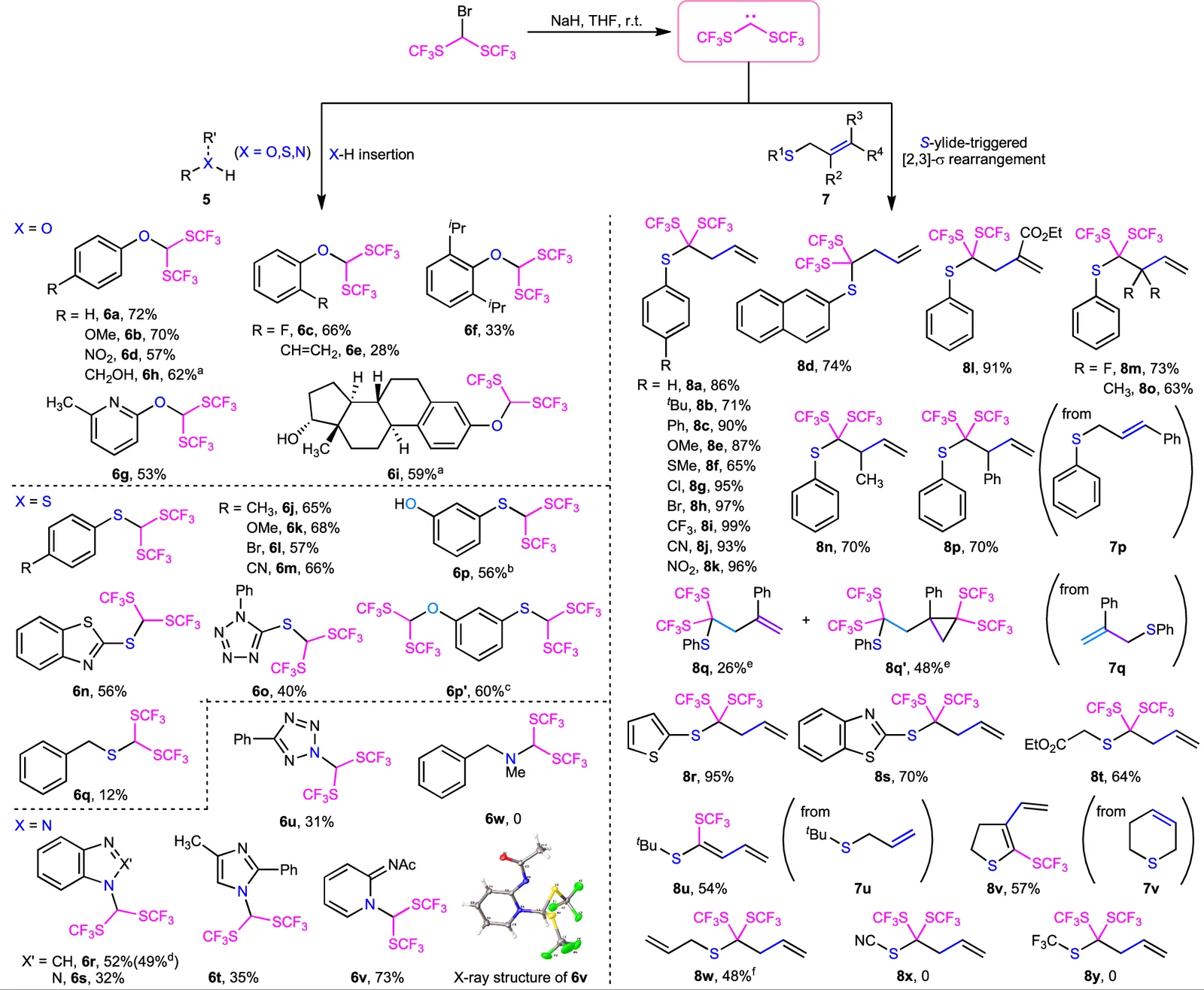
X-H Insertion induced by :C(SCF_3_)_2_ and [2,3]-σ rearrangement triggered by *S*-ylide from :C(SCF_3_)_2_. Typical reaction conditions for X-H insertions: substrates **5** (0.5 mmol, 1.0 equiv.), BrCH(SCF_3_)_2_ (1.25 mmol, 2.5 equiv.), NaH (1.0 mmol, 2.0 equiv.), THF (7.5 mL), N_2_, r.t., 24 h. Typical reaction conditions for [2,3]-σ rearrangement triggered by *S*-ylide: substrates **7** (0.5 mmol, 1.0 equiv.), BrCH(SCF_3_)_2_ (1.0 mmol, 2.0 equiv.), NaH (2.0 mmol, 4.0 equiv.), THF (7.5 mL), N_2_, r.t., 24 h. ^a^BrCH(SCF_3_)_2_ (2.5 mmol, 5.0 equiv.), NaH (2.0 mmol, 4.0 equiv.). ^b^BrCH(SCF_3_)_2_ (0.75 mmol, 1.5 equiv.), NaH (0.6 mmol, 1.2 equiv.), ^c^BrCH(SCF_3_)_2_ (1.5 mmol, 3.0 equiv.), NaH (1.25 mmol, 2.5 equiv.). ^d^Sodium salt of **5r** (instead of **5r**), BrCD(SCF_3_)_2_ (61% D, 0.75 mmol, 1.5 equiv.); no NaH was used. ^e^BrCH(SCF_3_)_2_ (2.5 mmol, 5.0 equiv.), NaH (5.0 mmol, 10.0 equiv.). ^f^BrCH(SCF_3_)_2_ (2.0 mmol, 4.0 equiv.), NaH (4.0 mmol, 8.0 equiv.).

The insertion of carbenes into X-H bonds (X = O, S, N)^7^ represents one of the classic reaction modes of carbenes. We found that NaH could still be employed under identical conditions in THF for X–H insertion, where it was used in slightly lower equivalent relative to BrCH(SCF_3_)_2_ to avoid potential base-induced product decomposition. This reaction exhibits broad substrate applicability and excellent regioselectivity (Fig. 3, left). Phenols and thiophenols with different electronic properties were well-tolerated (**6a-6d, 6j-6m**). 2-Vinylphenol (**5e**) gave only the O–H insertion product in low yield without any competing cyclopropanation; together with the similarly low yield of product **6f**, this indicates that steric hindrance *ortho* to the phenolic O–H bond reduces insertion efficiency. In substrates containing multiple insertion sites, high selectivity was observed: an *ortho*-methyl group shielded the N atom, leading to exclusive O–H insertion (**6g**). When both phenolic and alcoholic O–H bonds were present in substrate, only the phenoxy oxygen reacted, even with a doubled amount of the precursor/base (**6h**). Consequently, estradiol could be selectively functionalized at its phenolic hydroxy group via carbene insertion, illustrating the potential of this reaction for late-stage modification of bioactive molecules (**6i**). In heteroaromatic thiols, the carbene showed a preference for reaction at sulfur over nitrogen (**6n,** **6o**). For 3-mercaptophenol (**5p**), The carbene reacted preferentially at the sulfur atom: exclusive S–H insertion (**6p**) was obtained in moderate yield with a modest excess of the precursor, while double insertion (**6p'**) occurred with more than two equivalents. *N*-aromatic heterocycles, including imidazoles and azoles, were viable substrates (**6r-6u**). When multiple reaction sites were present, the large steric hindrance of the carbene appeared to dominate the regioselectivity, leading to exclusive reaction at the sterically less hindered nitrogen atom (**6t, 6u**). A deuterium-labeling experiment using BrCD(SCF_3_)_2_ (61% D) and the sodium salt of substrate **5r** in the absence of NaH gave **6r** in yield comparable to the standard conditions, but with complete loss of deuterium (Supplementary Fig. 1, see Supplementary Information for details), ruling out a direct substitution pathway. The bulky bis(trifluoromethylthio)methyl carbon center strongly disfavors nucleophilic attack. Furthermore, single-crystal diffraction confirmed that for substrate **5v**, the carbene reacted selectively with the more nucleophilic pyridine nitrogen atom, affording a product featuring double bond migration (CCDC 2516955 contains the supplementary crystallographic data of compound **6v**). Benzyl mercaptan (**5q**) gave low yield, and benzyl methyl amine (**5w**) did not afford desired product. These results show that :C(SCF_3_)_2_ is poorly compatible with aliphatic thiols and amines.

Carbenes with optimal balance of electrophilicity and chemoselectivity may also coordinate with specific heteroatom to form ylides, which are crucial reactive intermediates in organic chemistry, capable of undergoing a series of subsequent transformations. For instance, the [2,3]-σ rearrangement (Doyle−Kirmse reaction) of the ylides, which proceeds through a concerted five-membered ring transition state to simultaneously achieving the cleavage of old bonds and the reorganization of new bonds, has emerged as a powerful strategy for constructing complex architectures^8,10,51,52^. Therefore, we investigated the generation of ylides from :C(SCF_3_)_2_ and their subsequent [2,3]-σ rearrangement (Doyle−Kirmse reaction) (Supplementary Table 2, see Supplementary Information for details). Under the identical carbene generation conditions described above, the O and N heteroatoms in ethers or amines failed to effectively capture the carbene, while terminal alkenyl groups in allyl ether/amine were also unable to undergo cycloaddition with the carbene, resulting in the formation of a large amount of tetakis(trifluoromethylthio)ethylene byproduct via carbene dimerization (Supplementary Table 2, entries 1, 2). In contrast, the S atom in allylic sulfide could efficiently coordinate with the carbene to form sulfur ylides, which underwent [2,3]-σ rearrangement to yield the target product in 88% crude yield (Supplementary Table 2, entry 3). It should be noted that the terminal alkenyl group remained intact even in the presence of excess :C(SCF_3_)_2_. Additionally, in the absence of NaH, BrCH(SCF_3_)_2_ did not react with sulfides (Supplementary Table 2, entry 4), which excludes the traditional ylide generation pathway involving sulfonium salt formation and subsequent deprotonation.

The [2,3]-σ rearrangement reaction of the sulfur ylides from allyl sulfides with :C(SCF_3_)_2_ exhibits excellent substrate generality (Fig. 3, right). While all phenyl allyl sulfides underwent complete conversion, the isolated yields were influenced by substituent effects. Electron‑donating groups (**8b-8f**) led to products that were sensitive to silica gel and partially decomposed during purification, resulting in lower isolated yields. In contrast, electron‑withdrawing substituents (**8g-8l**) significantly enhanced product stability, affording the corresponding rearrangement products in excellent isolated yields. Allyl substrates with substitution at the terminal position reacted smoothly (**8m-8o**). Strikingly, the reaction forged adjacent all-carbon quaternary centers in a sterically congested system (**8o**). For conjugated styrenyl systems, the reaction favored rearrangement over cyclopropanation in these cases (**8p**-**8q**). Furthermore, because the alkenyl group remains conjugated to the benzene ring in product **8q**, the excess carbene in the reaction leads to a cascade cyclopropanation, affording product **8q'**. The reaction generality was further demonstrated with sulfide substrates substituted by heteroaromatic rings including thiophene, thiazole as well as alkyl allyl sulfides bearing electron-withdrawing groups (**8r-8t**). In the case of a *tert*-butyl substituted sulfide, despite the significant steric repulsion, ylide formation occurred over the carbene’s tendency to undergo cyclopropanation with the sterically less hindered terminal alkenyl group. The resulting [2,3]-σ rearrangement proceeded, followed by elimination of HSCF_3_, likely driven by the pronounced steric repulsion between the *tert*-butyl and bis(trifluoromethylthio)methylene groups, to furnish a conjugated diene product (**8u**). A six-membered cyclic allylic sulfide also participated in the reaction; ring contraction yielded a five-membered ring, and the steric strain associated with the incorporated bis(trifluoromethylthio)methylene group prompted HSCF_3_ elimination, leading to a cyclopentene derivative (**8v**). Diallyl sulfide underwent only a single-site reaction even with a doubled amounts of BrCH(SCF_3_)_2_ and NaH, indicating the steric influence of the carbene (**8w**). For allyl sulfides **7x** and **7y**, the strongly electron-withdrawing substituent on the sulfur atom severely diminishes its ability to coordinate with the carbene, preventing ylide formation and subsequent rearrangement.

**Fig. 4:**
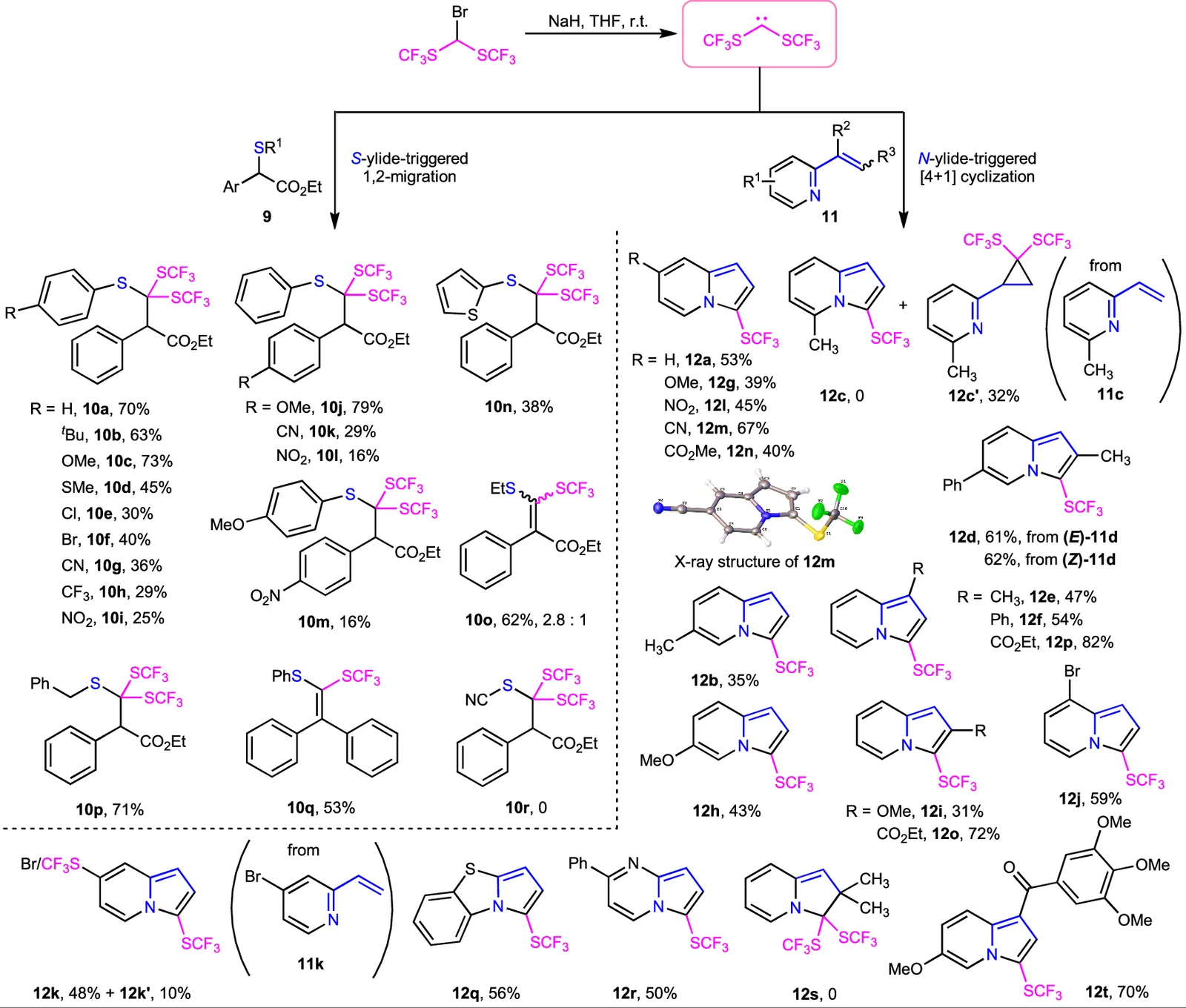
1,2-Migration and [4+1] cyclization triggered by *S*/*N*-ylide from :C(SCF_3_)_2_. Typical reaction conditions for 1,2-migration triggered by *S*-ylide: substrates **9** (0.5 mmol, 1.0 equiv.), BrCH(SCF_3_)_2_ (1.0 mmol, 2.0 equiv.), NaH (2.0 mmol, 4.0 equiv.), THF (7.5 mL), N_2_, r.t., 24 h. Typical reaction conditions for [4+1] cyclization triggered by *N*-ylide: substrates **11** (0.5 mmol, 1.0 equiv.), BrCH(SCF_3_)_2_ (1.5 mmol, 3.0 equiv.), NaH (3.0 mmol, 6.0 equiv.), THF (7.5 mL), N_2_, r.t., 24 h.

In addition to allyl sulfides, the sulfur ylides generated from benzyl sulfides with carbene can also undergo [2,3]-σ rearrangement (Sommelet-Hauser rearrangement), which involves a unique dearomatization intermediate, ultimately restoring aromaticity to yield *ortho*-disubstituted benzenes^53^. Given that the significant steric encumbrance of :C(SCF_3_)_2_ might impede the dearomatization step, we introduced an ester substituent at the benzylic position, aiming to stabilize the dearomatization intermediate. However, instead of the anticipated product, the reaction unexpectedly proceeded predominantly via a sulfur-ylide-triggered 1,2-benzyl migration (Stevens rearrangement) (Fig. 4, left, **10a**). Similar to the aforementioned [2,3]-σ rearrangement, this reaction does not require pre-synthesized sulfonium salt precursors and can proceed efficiently without external heating, photoirradiation, or metal catalysis, showing distinct advantages over conventional methods^54–56^.

For secondary benzylic aryl sulfides substituted with an ester group at the benzylic position, the electronic effects of the substituents significantly influence the efficiency of the sulfur-ylide-triggered benzyl 1,2-migration reaction (Stevens rearrangement) (Fig. 4, left). Substrates bearing electron-neutral or electron-donating groups all undergo conversion with moderate to good yields (**10a-10d, 10j**), while strongly electron-withdrawing groups significantly diminished yields (**10e-10i, 10k-10l**), and low yield caused by an electron-withdrawing group on the benzyl moiety could not be compensated for by an electron-donating group on the phenylthio group (**10m**). The reaction tolerated heteroaromatic (**10n**) and alkyl sulfide motifs, with an ethyl derivative eliminating HSCF_3_ upon workup (**10o**). For dibenzyl sulfides, only the ester-substituted benzyl group underwent exclusive sulfur-ylide-triggered 1,2-migration (**10p**). This indicates that steric influence is subordinate in this process and that a secondary benzylic carbocation, stabilized by concurrent conjugation from both the phenyl and ester groups, is requisite for sufficient migratory aptitude. The expected sulfur-ylide-triggered 1,2-migration also occurred with diphenylmethyl phenyl sulfide. However, subsequent purification by column chromatography resulted in the elimination of HSCF_3_, yielding the olefin product (**10q**). Thiocyanide **9r** did not undergo this reaction, as the strong electron-withdrawing cyano group on sulfur suppresses ylide formation.

When two highly reactive benzyl aryl sulfides (**9c,** **9j**) were simultaneously added to the reaction system, besides the normal sulfur-ylide-triggered 1,2-migration products (**10c,** **10j**) from the respective sulfides, crossover products (**10a**, **10s**) resulting from intermolecular benzyl group exchange (Supplementary Fig. 2a, see Supplementary Information for details) were also detected. This indicates that the cleavage of the C–S bond and the formation of the C–C bond are proceeded via a stepwise mechanism. Furthermore, the reaction of **9a** was quenched by 2,2,6,6-tetramethylpiperidine-1-oxyl (TEMPO), and the corresponding benzyl-TEMPO adduct was detected by HRMS, confirming a radical intermediate (Supplementary Fig. 2b, see Supplementary Information for details). These results collectively demonstrate that this sulfur-ylide-triggered 1,2-migration reaction, distinct from the concerted mechanism of [2,3]-σ rearrangement, likely proceeds through a stepwise process involving homolysis of the secondary benzylic C–S bond, generation of radical species, and their subsequent recombination.

The balanced electrophilicity and chemoselectivity of bis(trifluoromethylthio)carbene enable its efficient capture by the sulfur atom in sulfides to form sulfur ylides. A dichotomy in reactivity was observed with tertiary amines, where nitrogen ylide formation was inefficient (Supplementary Table 2, entry 2), possibly due to the significant steric bulk of the carbene. This selectivity prompted us to investigate the less hindered nitrogen atom in pyridine derivatives. Using the standard carbene-generation conditions, we employed 2-vinylpyridine (Fig. 4, right, **11a**) to concurrently assess competition between [2+1] cycloaddition and nitrogen-ylide formation. Surprisingly, the reaction delivered an indolizine product (**12a**) via a [4+1] cyclization. We propose that the electron-deficient nature of the alkenyl group, imposed by the pyridyl group, disfavors cyclopropanation. Instead, the carbene preferentially attacks the pyridyl nitrogen, generating a ylide that undergoes subsequent cyclization and HSCF_3_ elimination to furnish the 3-SCF_3_ indolizine framework. Control experiments confirmed that no reaction between 2-vinylpyridine and BrCH(SCF_3_)_2_ occurred in the absence of NaH. This result unambiguously rules out the pathway involving deprotonation of a pyridinium salt to generate the nitrogen ylide.

Exploration on steric influence showed that reactions proceeded smoothly when methyl substituents were distal to the nitrogen atom (Fig. 4, right, **12b, 12d-12e**). However, an *ortho*-methyl group on the pyridine suppresses ylide formation due to steric hindrance and prevents indolizine production (**12c**). Its electron-donating effect also reduces the pyridine’s electron deficiency, which enables the carbene to instead undergo [2+1] cyclization at the less hindered vinyl group, forming cyclopropane **12c′**. This result highlights the high sensitivity of :C(SCF_3_)_2_-pyridine ylide formation to steric hindrance. Electronic effects exerted a less pronounced influence; substrates bearing either electron-donating or electron-withdrawing groups on the pyridine ring or alkenyl moiety were converted in moderate to excellent yields (**12f-12p**). Notably, The reaction of substrate **11k** yielded not only the anticipated cyclization product but also a side product, 3,7-bis(trifluoromethylthio)indolizine (**12k′**), wherein the bromine atom was substituted by an –SCF_3_ group. Owing to the electron-rich nature of the indolizine core, the formation of **12k′** is likely attributable to a nucleophilic aromatic substitution by the trifluoromethylthio anion (SCF_3_^-^) on either the pyridinium ylide intermediate or the starting material **11k**. The structure of the 3-(trifluoromethylthio)indolizine framework was firmly validated by single-crystal X-ray diffraction analysis of compound **12m** (CCDC 2516956 contains the supplementary crystallographic data of compound **12m**). The reaction scope could extend to various heterocycles, including benzothiazole and pyrimidine (**12q**-**12r**). For the phenyl-substituted pyrimidine, regioselectivity was observed, with the carbene’s attack dictated by the phenyl group’s position, resulting in functionalization at the nitrogen farther from the substituent (**12r**). The substrate **11s** did not react, presumably because the steric hindrance precludes the formation of ylide or subsequent cyclization. A 3-SCF_3_ substituted derivative **12t**, based on an indolizine scaffold with known anticancer activity^57^, was successfully synthesized, underscoring the potential of this reaction for the late-stage diversification of bioactive molecules.

**Fig. 5:**
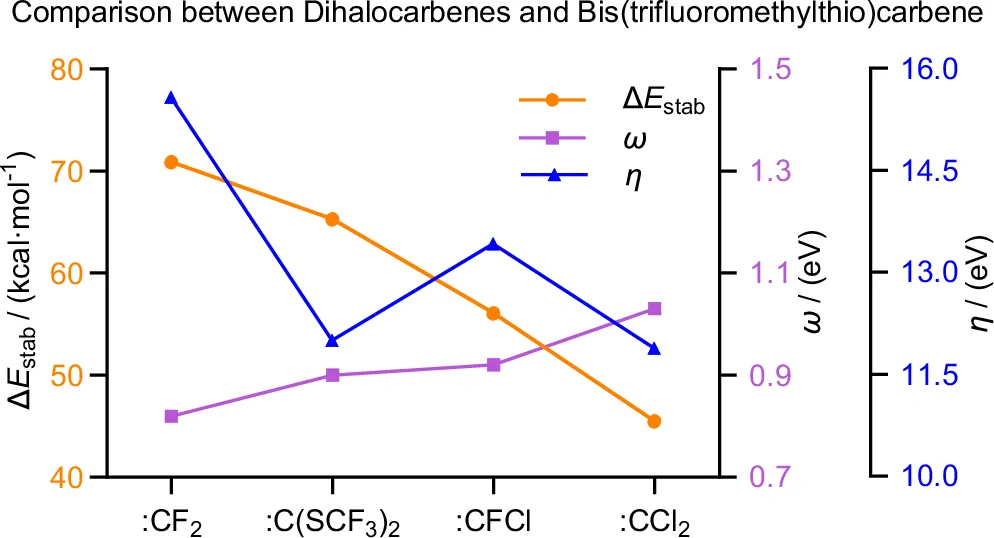
Computed properties of bis(trifluoromethylthio)carbene compared with dihalocarbenes. Theoretical comparison of stabilization energies (Δ*E*_stab_), global electrophilicities (*ω*), and absolute hardness (*η*). Δ*E*_stab_ is defined as the −Δ*E* for the isodesmic reaction :CH_2_ (singlet) + CH_3_X + CH_3_Y → :CXY (singlet) + 2 CH_4_, calculated at the B3LYP/6-311 + + G(2 d,p) level with a fine integration grid. *ω* and *η* are derived from frontier molecular orbital energies at HF/6-31 G(d,p)//MP2/6-31 G(d,p): *ω* = (*E*_LU_ + *E*_HO_)^2^/[8(*E*_LU_ − *E*_HO_)] and *η* = *E*_LU_ − *E*_HO_, where *E*_LU_ and *E*_HO_ are the lowest unoccupied (LU) and highest occupied (HO) molecular orbital energies, respectively. Data presentation: Δ*E*_stab_ (orange), *ω* (purple), and *η* (blue) are plotted as line graphs for direct comparison across the carbene series.

In the aforementioned series of reactions, including cyclopropanation, skeletal editing, X–H insertion, rearrangement, migration, and cyclization, the conditions for generating :C(SCF_3_)_2_ are largely identical. That is, once this carbene is generated, it can undergo all these types of reactions. Such broad-scope reactivity is relatively rare in carbene chemistry. This versatility may be related to the finely balanced electrophilicity and chemoselectivity imparted by the trifluoromethylthio groups on the carbene. By theoretical calculations, we compared bis(trifluoromethylthio)carbene (based on the *trans*-*cis* conformer **S**_**0**_) with ground state dihalocarbenes (:CF_2_, :CFCl, :CCl_2_) in terms of the parameters such as stabilization energies relative to singlet :CH_2_ (Δ*E*_stab_)^58^, global electrophilicities (*ω*)^58,59^, and absolute hardness (*η*)^58,59^, The Δ*E*_stab_ values were calculated as the *−*Δ*E* of the isodesmic reaction :CH_2_ + CH_3_X + CH_3_Y → :CXY + 2 CH_4_ at B3LYP^60^/6-311 + + G(2 d,p)^61–65^ level, while *ω* and *η* were computed at the HF/6-31 G(d,p)^63,66–68^//MP2/6-31 G(d,p) level using the relationships: *ω* = (*E*_LU_ + *E*_HO_)^2^/[8(*E*_LU_ − *E*_HO_)] and *η* = *E*_LU_ − *E*_HO_, where *E*_LU_ and *E*_HO_ denote the energies of the lowest unoccupied and highest occupied molecular orbitals, respectively (Fig. 5, Supplementary Table 4, see Supplementary Information for computational details and Source Data for Cartesian coordinates and raw computational data). The results indicate that for dihalocarbenes, the stabilization energy and absolute hardness decrease in the order :CF_2_ > :CFCl > :CCl_2_, while the global electrophilicity increases sequentially. The thermodynamic stabilization energy and global electrophilicity of :C(SCF_3_)_2_ lie between those of :CF_2_ and :CFCl, suggesting sufficient stability and good electrophilicity of :C(SCF_3_)_2_. On the other hand, its absolute hardness is relatively low, comparable to :CCl_2_, which may contribute to chemoselectivity of :C(SCF_3_)_2_. Furthermore, the bulky trifluoromethyl groups provide additional kinetic persistence to the carbene, improving its ability for regiodiscrimination among reaction sites with differing steric environments. It is the synergistic interplay of the above effects that endows the :C(SCF_3_)_2_ with its well-balanced electrophilicity and chemoselectivity, ultimately leading to the diverse reactivity superior to that of dihalocarbenes, as demonstrated above.

A diverse set of reactions including alkene [2+1] cycloaddition, indole/pyrrole skeletal editing, aryl X–H insertion, sulfur-ylide-triggered [2,3]-σ rearrangement/1,2-migration, and nitrogen-ylide-triggered [4+1] cyclization have been conducted on a gram scale, illustrating the potential utility of this approach (Supplementary Fig. 3, see Supplementary Information for details).

### Proposed mechanistic rationale

**Fig. 6:**
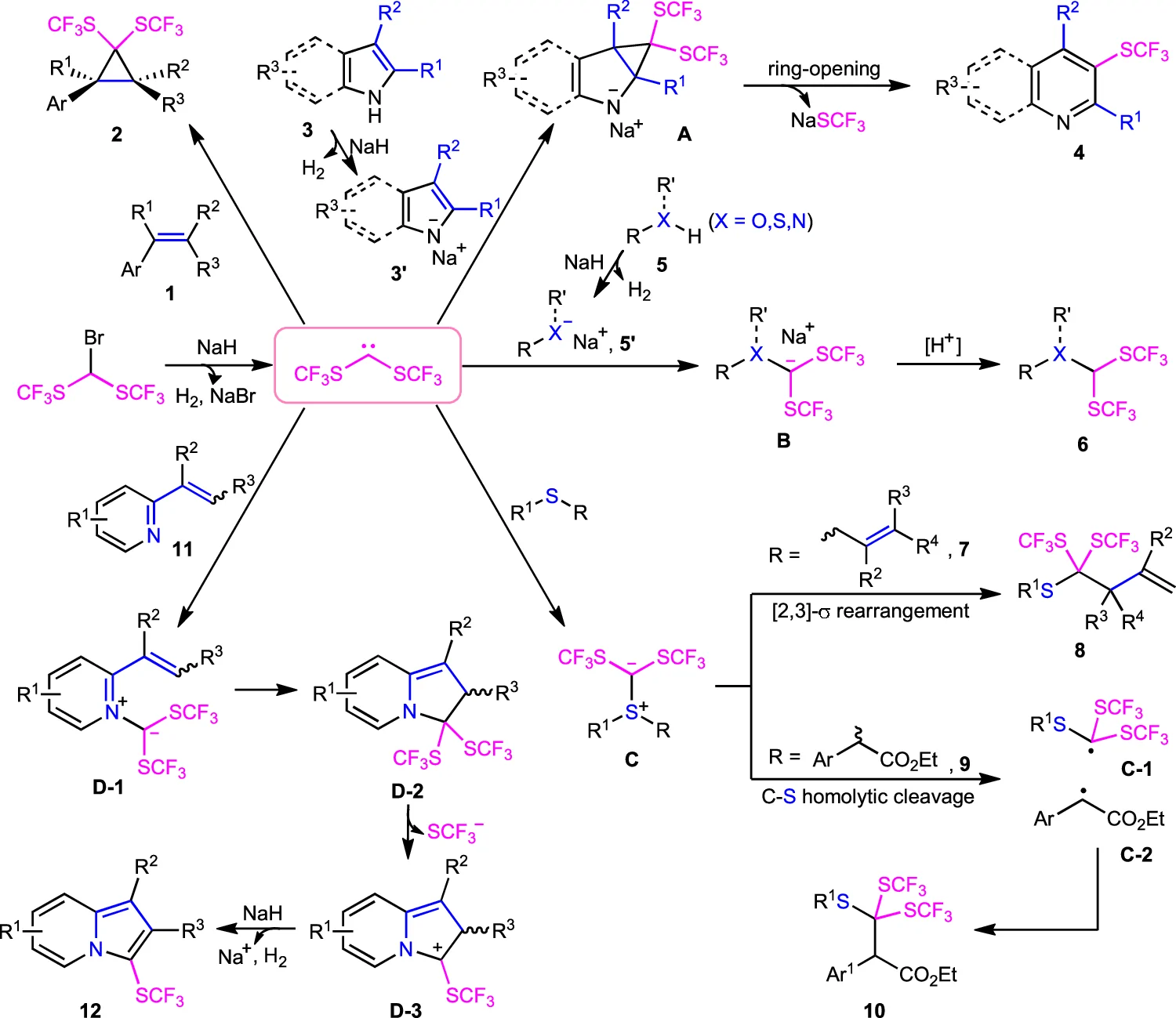
Possible mechanism of the generation and reactivities of :C(SCF_3_)_2_. The carbene is generated via NaH-promoted *α*-elimination from BrCH(SCF_3_)_2_, and subsequently undergoes four distinct reaction pathways, including cyclopropanation, skeletal editing, X–H insertion and ylide-involved transformations.

Based on a comprehensive analysis of experimental data and relevant literature reports, we propose the following mechanism for the generation and subsequent transformations of :C(SCF_3_)_2_ (Fig. 6). Initially, NaH acts as a base to deprotonate BrCH(SCF_3_)_2_, forming a carbanion intermediate. This carbanion then undergoes *α*-elimination of bromide to yield singlet carbene :C(SCF_3_)_2_, which engages in distinct pathways with various substrates: (i) Cyclopropanation: Aryl alkene substrates **1** react with the carbene via a concerted cyclopropanation mechanism, directly affording cyclopropane products **2**. (ii) Skeletal Editing: Indole/pyrrole substrates **3** are first deprotonated by NaH to generate anions **3′**, which subsequently undergoes cyclopropanation with the carbene to form intermediate **A**. This intermediate then undergoes ring-opening followed by elimination of NaSCF_3_ to yield products **4**. (iii) X–H Insertion: Phenol, thiophenol, and other N–H heteroaromatic substrates **5** are deprotonated to anions **5′**, which trap the carbene to form anionic intermediate **B**. Subsequent protonation of **B** delivers the insertion products **6**. (iv) Ylide-involved transformations: Thioethers coordinate with the carbene to form sulfur ylide **C**. The subsequent pathway diverges: For allylic thioethers **7**, ylide **C** undergoes a [2,3]-σ rearrangement to give products **8**, for secondary benzyl thioethers **9**, ylide **C** proceeds via homolytic cleavage of the C–S bond, generating radical pair **C-1** and **C-2**. Their recombination yields the 1,2-migration (Stevens rearrangement) products **10**. 2-Vinylpyridine substrates **11** react with the carbene to form nitrogen ylide intermediate **D-1**, it undergoes a 6π-electrocyclization to furnish intermediate **D-2**, The steric congestion of the –C(SCF_3_)_2_– moiety and the electron donating indolizine nitrogen then promote SCF_3_^-^ departure to form a nitrogen stabilized carbocation **D-3**, followed by rapid deprotonation promoted by NaH to yield **12**.

## Discussion

The rational design of substituents to synergistically modulate electronic and steric effects represents a powerful strategy for controlling the reactivity and selectivity of elusive reaction intermediates. This study demonstrates that installing trifluoromethylthio (–SCF_3_) groups onto a carbene carbon realises this design principle effectively. The strong electron-withdrawing inductive nature, highly polarizable sulfur atom, and bulky trifluoromethyl moiety within the –SCF_3_ group work in concert, bestowing the resulting bis(trifluoromethylthio)carbene (:C(SCF_3_)_2_) with a precisely balanced profile of high electrophilicity and chemoselectivity. This designed carbene, shown by theoretical calculations to possess a singlet ground state with sufficient stability and pronounced electrophilicity, serves as a versatile toolbox. Combined with significant steric hindrance, bis(trifluoromethylthio)carbene (:C(SCF_3_)_2_) can facilitate a range of free carbene transformations—including cyclopropanation, skeletal editing, X–H insertion, sulfur ylide-based [2,3]-σ rearrangement/1,2-migration, and nitrogen ylide-initiated [4+1] cyclization—with high chemoselectivity under a single set of mild conditions from a scalable precursor ( > 30 g/batch). The gram-scale feasibility of these diverse reactions suggests the practical utility of this approach, offering a streamlined synthetic pathway for constructing functional molecules, highlighting the potential of such rational substituent design in taming other reactive species for synthetic and applied sciences.

## Methods

Unless otherwise noted, all reagents and solvents were obtained commercially and used without further purification. The ^1^H, ^13^C and ^19^F NMR spectra were recorded on 400 MHz NMR spectrometers (400 MHz for ^1^H, 101 MHz for ^13^C and 376 MHz for ^19^F, respectively). All reactions were monitored by TLC or ^19^F NMR. Flash column chromatography was carried out using 300-400 mesh silica gel at medium pressure. High resolution mass spectrometry (HRMS) was performed on a Waters Premier GC-TOF MS instrument with electron impact (EI) ionization mode, or on a Thermo Scientific Q Exactive HF Orbitrap-FTMS instrument with electrospray ionization (ESI) mode.

### Procedure for the preparation of BrCH(SCF_3_)_2_

Bis(trifluoromethyl) carbonotrithioate (73.8 g, 300 mmol, 1.0 equiv.) was added to a stirred mixture of KF (31.4 g, 540 mmol, 1.8 equiv.) in AcOH (300 mL). Then TMSCN (56.3 mL, 450 mmol, 1.5 equiv.) was added dropwise at room temperature, and the mixture was stirred for 3 h at room temperature until the red color of the solution faded (*Caution*: the toxic HCN gas would be generated in the reaction; a well-ventilated fume hood was necessary for all operation). The resulting mixture was diluted with DCM (200 mL) and water (1 L). The organic phase was separated and the water phase was extracted 3 times with DCM (100 mL×3). All organic solutions were combined and washed with saturated NaHCO_3_ aqueous solution until the aqueous washings reached a pH of 8–9. The organic phase was dried over anhydrous Na_2_SO_4_, and the solvent was removed under vacuum. The residue was distilled in a reduced pressure to obtain (thiocyanatomethylene)bis((trifluoromethyl)sulfane) (51.6 g, 189 mmol, 63%) as a yellow liquid, b.p. 63-65 ^o^C/6.6 mbar.

Boron tribromide (9.2 mL, 95 mmol, 0.5 equiv.) was added dropwise to (thiocyanatomethylene)bis((trifluoromethyl)sulfane) (51.6 g, 189 mmol, 1.0 equiv.) at 0 ^o^C under a N_2_ atmosphere, The mixture was stirred for 24 h at room temperature. Subsequently, the flask was connected to a cold trap cooled to −196 °C. All volatile components from the reaction mixture were transferred to the cold trap under reduced pressure at room temperature. The cold trap was then brought back to ambient temperature under atmospheric pressure. The collected liquid was combined with 100 mL of saturated NaHCO_3_ aqueous solution and stirred vigorously for 2 hours, separated the organic phase, dried over anhydrous Na_2_SO_4_ to obtain (bromomethylene)bis((trifluoromethyl)sulfane) (37.5 g, 127 mmol, 67%) as a yellow liquid.

### General procedure for the synthesis of products 2, 4, 6, 8, 10, 12

A 25 mL round-bottom flask was charged with the corresponding substrates **1,** **3,** **5,** **7,** **9**, or **11** (0.5 mmol, 1.0 equiv.), NaH, and dry THF (7.5 mL) under a N_2_ atmosphere. BrCH(SCF_3_)_2_ was then added. The resulting mixture was stirred at room temperature for 24 h under N_2_. The reaction was then quenched by dilution with ethyl acetate (20 mL) and saturated aqueous NH_4_Cl solution (30 mL). The organic phase was separated, and the aqueous phase was extracted with ethyl acetate (3 × 20 mL). The combined organic extracts were dried over anhydrous Na_2_SO_4_, concentrated under reduced pressure, and the residue was purified by column chromatography to afford the desired products.

### Reagent loadings

For the synthesis of products **2,** **4,** **12**: NaH (72.0 mg, 3.0 mmol, 6.0 equiv.), BrCH(SCF_3_)_2_ (442.5 mg, 1.5 mmol, 3.0 equiv.).

For the synthesis of product **6**: NaH (24.0 mg, 1.0 mmol, 2.0 equiv.), BrCH(SCF_3_)_2_ (368.8 mg, 1.25 mmol, 2.5 equiv.) **(**with modified quantities of NaH and BrCH(SCF_3_)_2_ in the synthesis of products **6h,** **6i,** **6p** and **6p′**, see the Supplementary Information for details.).

For the synthesis of products **8,** **10**: NaH (48.0 mg, 2.0 mmol, 4.0 equiv.), BrCH(SCF_3_)_2_ (295.0 mg, 1.0 mmol, 2.0 equiv.) (with modified quantities of NaH and BrCH(SCF_3_)_2_ in the synthesis of product **8q,** **8q′** and **8w**, see the Supplementary Information for details.).

## Supplementary information


Supplementary Information
Transparent Peer Review file


## Source data


Source Data


## Data Availability

The X-ray crystal structure data generated in this study have been deposited in the Cambridge Crystallographic Data Centre (CCDC) under deposition numbers CCDC 2516953, 2516954, 2516955 and 2516956 for compounds **2p**, **4t**, **6v** and **12m**, respectively. These data can be obtained free of charge from The Cambridge Crystallographic Data Centre via https://www.ccdc.cam.ac.uk/structures/. Additional experimental and calculation details, materials, methods, and characterization data are provided in Supplementary Information. The Cartesian coordinates and raw computational data for the optimized structures are available in Source Data. Source data are provided with this paper.
