# Supplementary material for: Bis(trifluoromethylthio)carbene as a toolbox for versatile carbene transformations
Source: Nat Commun. 2026 Jul 29;17:9214. doi: 10.1038/s41467-026-76124-z (PMC13522504; doi:10.1038/s41467-026-76124-z)
Supplement: Supplementary file 1 [file 41467_2026_76124_MOESM1_ESM.pdf]

Supplementary Information

Bis(trifluoromethylthio)carbene as a Toolbox for Versatile Carbene Transformations

Jun Sun[1], Qin-Yuan Ni[1], Yu-Cheng Gu[2], Jin-Hong Lin[1,3*] & Ji-Chang Xiao[1*]

[1]State Key Laboratory of Fluorine and Nitrogen Chemistry and Advanced Materials, Shanghai Institute of Organic Chemistry, University of Chinese Academy of Sciences, Chinese Academy of Sciences, 345 Lingling Road, Shanghai 200032, China.

[2]Syngenta Jealott's Hill International Research Centre; Bracknell, RG42 6EY, UK.

[3]Department of Chemistry, Shanghai University, 99 Shangda Road, Shanghai, 200444, China.

*Corresponding authors. Emails: jlin@shu.edu.cn; jchxiao@sioc.ac.cn

Table of Contents

1 General information 1

2 Supplementary experimental tables and figures 2

3 Procedure for the preparation of $BrC(SCF_3)_2H$ 5

4 Procedure for the generation of $(CF_3S)_2C=C(SCF_3)_2$ 8

5 Procedures for the synthesis of substrates 9

6 Procedures for the synthesis of products 43

7 Gram-scale experiments 117

8 Crystal data and structure refinement 120

9 Computational Details 125

10 Copies of ^{1}H NMR, ^{19}F NMR and ^{13}C NMR Spectra 128

1 General information

The ^{1}H, ^{13}C and ^{19}F NMR spectra were recorded on 400MHz NMR spectrometers (400 MHz for ^{1}H, 101MHz for ^{13}C and 376 MHz for ^{19}F respectively). The chemical shifts (δ) for ^{1}H and ^{13}C are given in ppm relative to residual signals of solvents ($CHCl_3$ at 7.26 ppm for ^{1}H NMR and 77.0 ppm for ^{13}C NMR. DMSO at 2.50 ppm for ^{1}H NMR and 39.52 for ^{13}C NMR. CH_3CN at 1.94 for ^{1}H NMR and 118.26 for ^{13}C NMR. $(CH_3)_2CO$ at 2.05 for ^{1}H NMR and 206.26 for ^{13}C NMR.) while the chemical shifts (δ) for ^{19}F NMR are given in ppm relative to trichlorofluoromethane (CCl_3F) as standard. Coupling constants (*J*) are reported in Hz. The following abbreviations were used to explain the multiplicities: s = singlet, d = doublet, t= triplet, q = quartet, sept = septet, m = multiplet, br = broad. All reactions were monitored by TLC or ^{19}F NMR. Flash column chromatography was carried out using 300-400 mesh silica gel at medium pressure. High resolution mass spectrometry (HRMS) was performed on a Waters Premier GC-TOF MS instrument with electron impact (EI) ionization mode, or on a Thermo Scientific Q Exactive HF Orbitrap-FTMS instrument with electrospray ionization (ESI) mode. Sodium hydride (NaH) was purified from 60% dispersion in mineral oil. Under N_2, the dispersion was suspended in anhydrous THF and stirred to dissolve the oil. The suspension was then allowed to settle, and the supernatant was decanted via syringe; this washing was repeated three times. The remaining solid was evacuated to remove residual solvents, affording pure NaH as a powder. Unless otherwise noted, all reagents and solvents were obtained commercially and used without further purification.

Unless otherwise specified, all of the density functional theory (DFT) and (post-)Hartree-Fock [(post-)HF] calculations were carried out with the Gaussian 09 D.01 program package[1]. The geometries of all structures were optimized to their local minima at gas phase which were confirmed by frequency calculations performed at the same theoretical levels described below (zero imaginary frequencies). If necessary, higher-quality single point energy was computed based on their optimized minima. The optimized structures were visualized with CYLview software[2].

2 Supplementary experimental tables and figures

Supplementary Table 1. Optimization of the [2+1] cycloaddition of alkenes induced by :C(SCF_3)$_2$

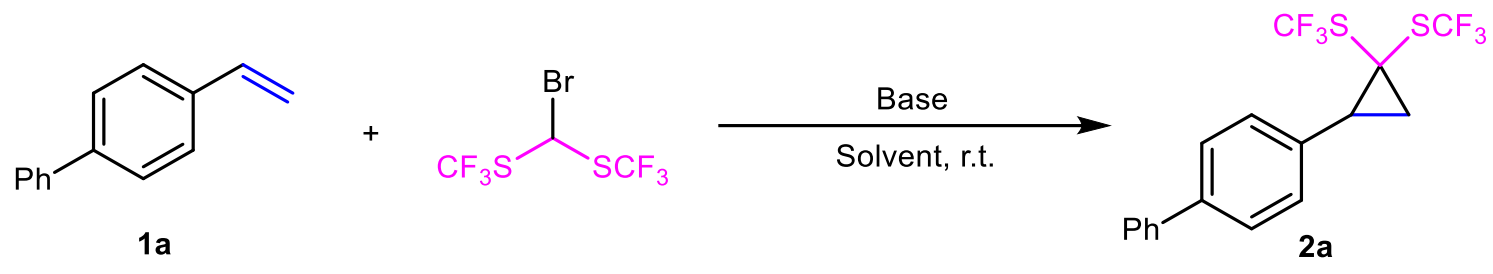

entry	solvent	base	yield of **2a**(%)[a]
1[b]	THF	NaH	64
2[b]	1,4-Dioxane	NaH	26
3[b]	$PhCH_3$	NaH	16
4[b]	DCM	NaH	13
5[b]	DCE	NaH	9
6[b]	EtOAc	NaH	48
7[b]	CH_3CN	NaH	15
8[b]	DMF	NaH	0
9[c]	THF	NaH	74
10[d]	THF	NaH	79
11[e]	THF	NaH	73
12[f]	THF	CH_3OK	0
13[f]	THF	^{t}BuOK	0
14[f]	THF	NaOH	44
15[f]	THF	NaH	87
16[f]	THF	^{n}BuLi	0
17[f]	THF	Na_2CO_3	0
18[f]	THF	Et_3N	37
19[f]	THF	DMAP	0
20[g]	THF	NaH	90

Reaction conditions: **1a** (0.1 mmol, 1.0 equiv.), BrCH(SCF_3)$_2$ and NaH in a solvent (1.5 mL) at a N_2 atmosphere. [a]Yields were determined by the analysis of the crude ^{19}F NMR spectroscopy using 0.1 mmol $PhOCF_3$ as an internal standard; [b]Molar ratio of **1a** : BrCH(SCF_3)$_2$: NaH = 1.0 : 1.8 : 3.6,

reaction time was 8 h; [c]Molar ratio of **1a** : $BrCH(SCF_3)_2$: NaH = 1.0 : 2.4 : 4.8, reaction time was 8 h; [d]Molar ratio of **1a** : $BrCH(SCF_3)_2$: NaH = 1.0 : 3.0 : 6.0, reaction time was 8 h; [e]Molar ratio of **1a** : $BrCH(SCF_3)_2$: NaH = 1.0 : 3.6 : 7.2, reaction time was 8 h; [f]Molar ratio of **1a** : $BrCH(SCF_3)_2$: NaH = 1.0 : 3.0 : 6.0, reaction time was 12 h; [g]Molar ratio of **1a** : $BrCH(SCF_3)_2$: NaH = 1.0 : 3.0 : 6.0, reaction time was 24 h.

Supplementary Table 2. Optimization of the [2,3]-σ rearrangement of sulfur ylide from allyl sulfides with :$C(SCF_3)_2$

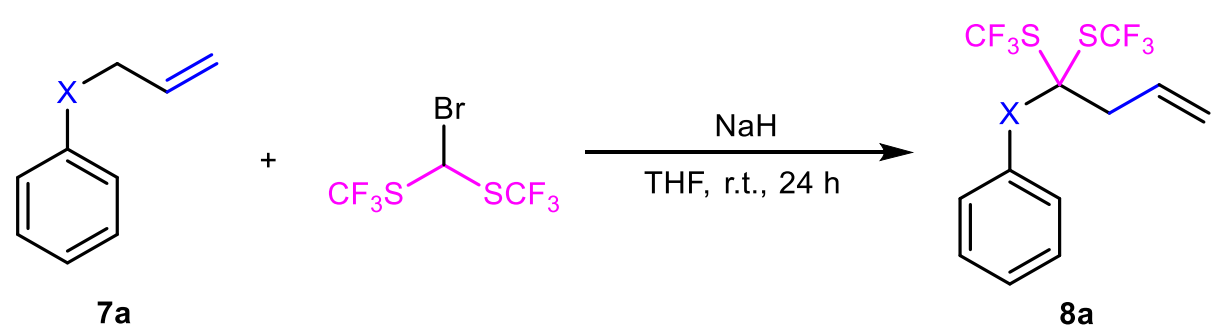

entry	X	yield of **8a**(%)[a]
1	O	0(N.D.)
2	NMe	0(N.D.)
3	S	88
4[b]	S	0(N.R.)

Reaction conditions: **7a** (0.5 mmol, 1.0 equiv.), $BrC(SCF_3)_2H$ (1.0 mmol, 2.0 equiv.), NaH (2.0 mmol, 4.0 equiv.), THF (7.5 mL), N_2, r.t., 24 h. [a]Yields were determined by the analysis of the crude ^{19}F NMR spectroscopy using $PhOCF_3$ as an internal standard; [b]NaH was not added to the reaction.

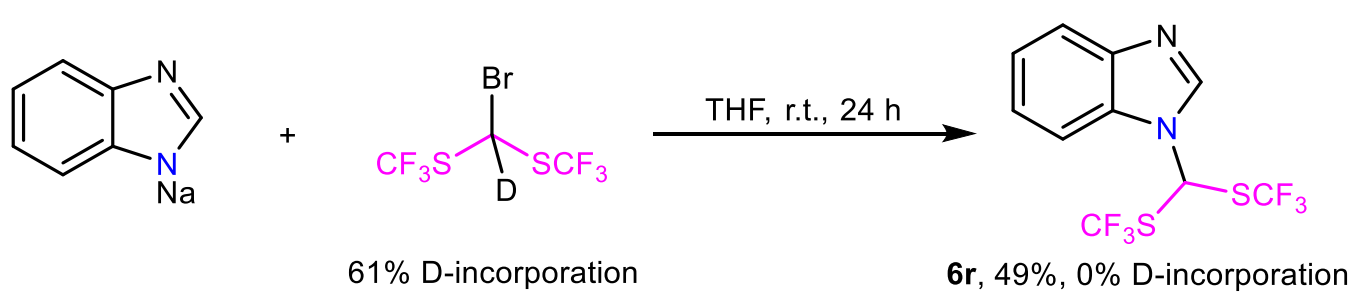

Supplementary Figure 1. Deuterium-labeling experiment of $BrC(SCF_3)_2D$ with the sodium salt of substrate 5r. Reaction condition: the sodium salt of **5r** (0.5 mmol, 1.0 equiv.), $BrC(SCF_3)_2D$ (61% D, 0.75 mmol, 1.5 equiv.), THF (7.5 mL), N_2, r.t., 24 h.

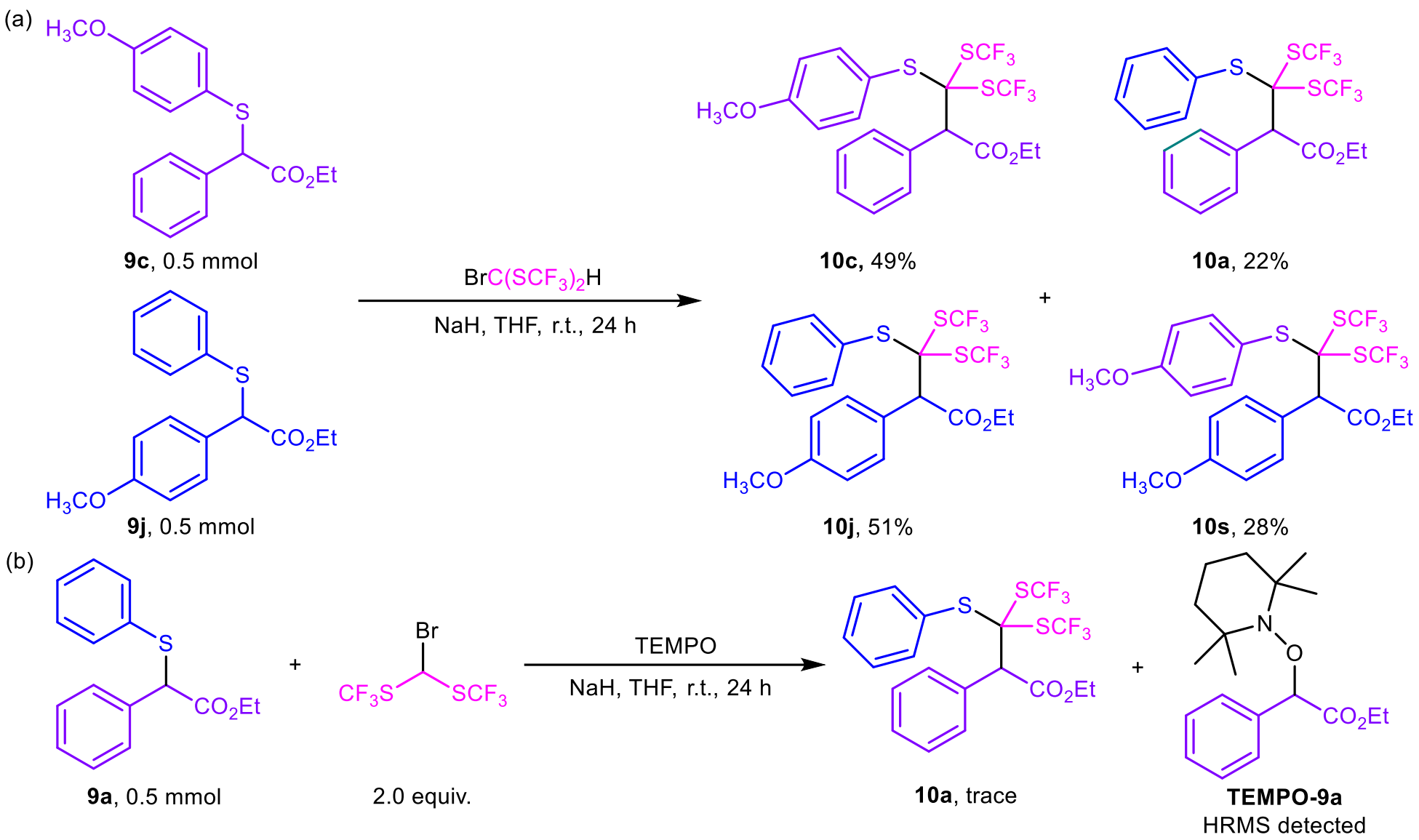

Supplementary Figure 2. Mechanism study on the 1,2-migration of sulfur ylide from *sec*-benzyl sulfides with :C(SCF_3)$_2$. Reaction conditions: (a) *sec*-benzyl sulfide **9c** and **9j** (each 0.5 mmol, 1.0 equiv.), BrC(SCF_3)$_2$H (2.0 mmol, 4.0 equiv.), NaH (4.0 mmol, 8.0 equiv.), THF (7.5 mL), N_2, r.t., 24 h. (b) *sec*-benzyl sulfide **9a** (0.5 mmol, 1.0 equiv.), TEMPO (1.0 mmol, 2.0 equiv.), BrC(SCF_3)$_2$H (1.0 mmol, 2.0 equiv.), NaH (2.0 mmol, 4.0 equiv.), THF (7.5 mL), N_2, r.t., 24 h.

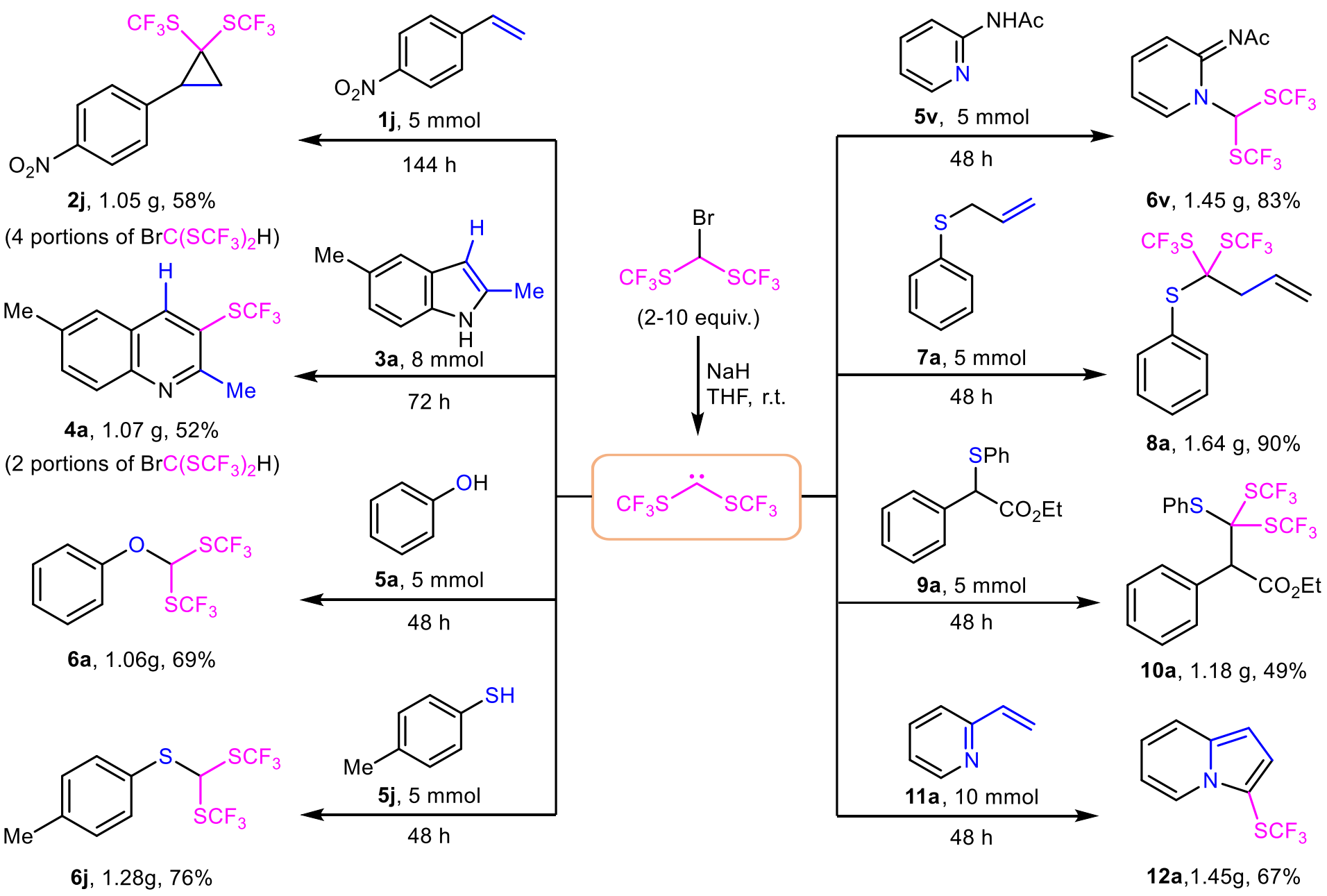

Supplementary Figure 3. Gram-scale experiments. Reaction conditions: substrate **1j**, **3a**, **5a**, **5j**, **5v**, **7a**, **9a** or **11a** (5~10 mmol, 1.0 equiv.), BrC(SCF_3)$_2$H (10~50 mmol, 2~10 equiv.), NaH (10~100 mmol, 2~20 equiv.), THF (40~75 mL), N_2, r.t., 48~144 h.

3 Procedure for the preparation of $BrC(SCF_3)_2H$

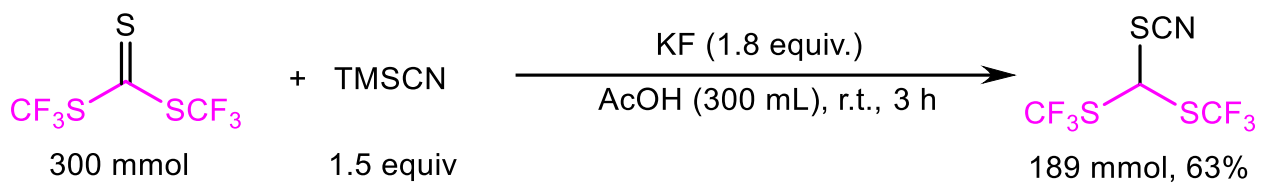

Bis(trifluoromethyl) carbonotrithioate (73.8 g, 300 mmol, 1.0 equiv.) was added to a stirred mixture of KF (31.4 g, 540 mmol, 1.8 equiv.) in AcOH (300 mL). Then TMSCN (56.3 mL, 450 mmol, 1.5 equiv.) was added dropwise at room temperature, and the mixture was stirred for 3 h at room temperature until the red color of the solution faded (**Caution**: the toxic HCN gas would be generated in the reaction, a well-ventilated fume hood was necessary for all operation). The resulting mixture was diluted with DCM (200 mL) and water (1 L). The organic phase was separated and the water phase was extracted 3 times with DCM (100 mL×3). All organic solutions were combined and washed with saturated $NaHCO_3$ aqueous solution until the aqueous washings reached a pH of 8–9. The organic phase was dried over anhydrous Na_2SO_4, and the solvent was removed under vacuum. The residue was distilled in a reduced pressure to obtain (thiocyanatomethylene)bis((trifluoromethyl)sulfane) (51.6 g, 189 mmol, 63%) as a yellow liquid, b.p. 63-65 °C/6.6 mbar.

(Thiocyanatomethylene)bis((trifluoromethyl)sulfane)

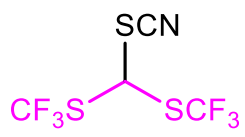

^{1}H NMR (400 MHz, $CDCl_3$) δ 5.97 (s, 1H).

^{19}F NMR (376 MHz, $CDCl_3$) δ -40.97 (s, 6F).

^{13}C NMR (101 MHz, $CDCl_3$) δ 128.1 (q, J = 312.4 Hz), 107.2, 50.0 (sept, J = 3.0 Hz).

HRMS (ESI) m/z: $[M\text{-}SCN]^+$ Calcd for $C_3HF_6S_2$ 214.9418; Found 214.9416.

IR (KBr) 2960, 2164, 1741, 1151, 1113, 1094, 759, 591, 474 cm^{-1}

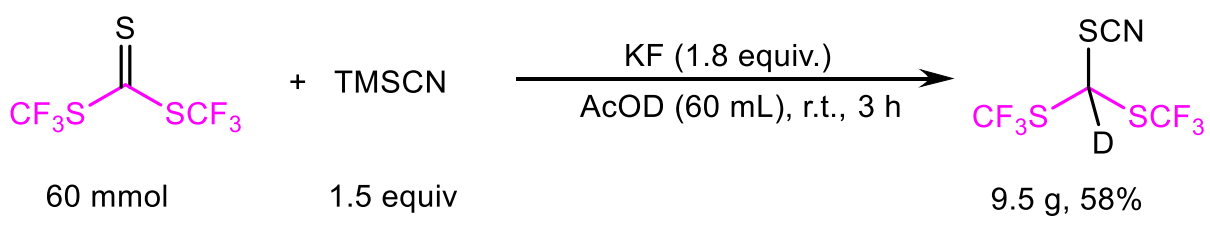

Bis(trifluoromethyl) carbonotrithioate (14.8 g, 60 mmol, 1.0 equiv.) was added to a stirred solution of dry KF (6.3 g, 108 mmol, 1.8 equiv.) in AcOD (60 mL) under a N_2

atmosphere, then TMSCN (11.3 mL, 90 mmol, 1.5 equiv.) was added dropwise under room temperature, the mixture was stirred for 3 h at room temperature until the red color of the solution faded (**Caution**: the toxic DCN gas would be generated in the reaction, a well-ventilated fume hood was necessary for all operation). the resulting mixture was diluted with DCM (40 mL) and water (200 mL). separated the organic phase and extracted water phase 3 times with DCM (40 mL×3), combined all organic solution and washed it with saturated $NaHCO_3$ aqueous solution until water phase keep to pH 8-9, separated the organic phase and dried over anhydrous Na_2SO_4, removed the solvent under vacuum, then the residue was distilled in reduced pressure to obtain (thiocyanatomethylene-*d*)bis((trifluoromethyl)sulfane) (9.5 g, 34.8 mmol, 58%, 61% D) as a yellow liquid, b.p. 63-65 °C/6.6 mbar.

(Thiocyanatomethylene-*d*)bis((trifluoromethyl)sulfane) (61% D)

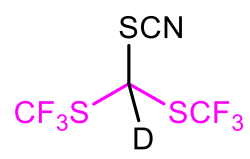

^{19}F NMR (376 MHz, $CDCl_3$) δ -40.99 (s, 6F).

^{13}C NMR (101 MHz, $CDCl_3$) δ 128.1 (q, J = 312.3 Hz), 107.3, 49.9 (m).

HRMS (FI) m/z: $[M]^+$ Calcd for $C_4DNF_6S_3$ 273.9233; Found 273.9237.

IR (KBr) 3005, 2989, 2959, 2208, 2164, 1275, 1260, 1151, 1101, 897, 766, 750, 704, 473 cm^{-1}

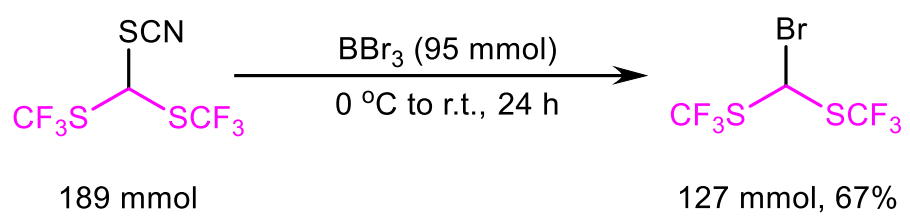

Boron tribromide (9.2 mL, 95 mmol, 0.5 equiv.) was added dropwise to (thiocyanatomethylene)bis((trifluoromethyl)sulfane) (51.6 g, 189 mmol, 1.0 equiv.) at 0 °C under a N_2 atmosphere, The mixture was stirred for 24 h at room temperature. Subsequently, the flask was connected to a cold trap cooled to -196 °C. All volatile components from the reaction mixture were transferred to the cold trap under reduced pressure at room temperature. The cold trap was then brought back to ambient temperature under atmospheric pressure. The collected liquid was combined with 100 mL of saturated $NaHCO_3$ aqueous solution and stirred vigorously for 2 hours, separated

the organic phase, dried over anhydrous Na_2SO_4 to obtain (bromomethylene)bis((trifluoromethyl)sulfane) (37.5 g, 127 mmol, 67%) as a yellow liquid.

(Bromomethylene)bis((trifluoromethyl)sulfane)

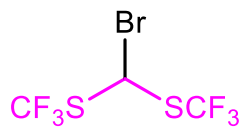

^{1}H NMR (400 MHz, $CDCl_3$) δ 6.38 (s, 1H).

^{19}F NMR (376 MHz, $CDCl_3$) δ -42.19 (s, 6F).

^{13}C NMR (101 MHz, $CDCl_3$) δ 128.6 (q, J = 311.7 Hz), 39.3 (sept, J = 3.5 Hz).

HRMS (ESI) m/z: $[M\text{-}Br]^+$ Calcd for $C_3HF_6S_2$ 214.9418; Found 214.9417.

IR (KBr) 3013, 2963, 2925, 1261, 1181, 1163, 1140, 1108, 1023, 801, 759, 729 cm^{-1}

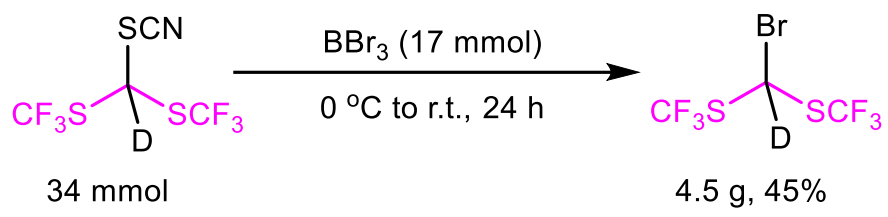

Boron tribromide (1.65 mL, 17 mmol, 0.5 equiv.) was added dropwise to (thiocyanatomethylene-*d*)bis((trifluoromethyl)sulfane) (9.3 g, 34 mmol, 61% deuterated) at 0 ºC under N_2 atmosphere, the mixture was stirred for 24 h at room temperature. Subsequently, the flask was connected to a cold trap cooled to -196 °C, and all volatile components were collected in the trap under vacuum, which was then returned to room temperature. The collected liquid were washed with 20 mL saturated $NaHCO_3$ aqueous solution and dried over anhydrous Na_2SO_4 to obtain (bromomethylene-*d*)bis((trifluoromethyl)sulfane) (4.5 g, 15.2 mmol, 45%, 61% D) as a yellow liquid.

(Bromomethylene-*d*)bis((trifluoromethyl)sulfane) (61% D)

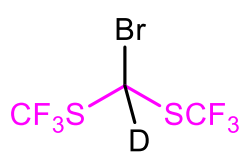

^{19}F NMR (376 MHz, $CDCl_3$) δ -42.19 (s, 6F).

^{13}C NMR (101 MHz, $CDCl_3$) δ 128.6 (q, J = 311.8 Hz), 39.2 (m).

HRMS (FI) m/z: $[M]^+$ Calcd for $C_3DBrF_6S_2$ 294.8665; Found 294.8669.

IR (KBr) 3005, 1275, 1260, 1164, 1112, 1015, 796, 765, 750, 702 cm^{-1}

4 Procedure for the generation of $(CF_3S)_2C{=}C(SCF_3)_2$

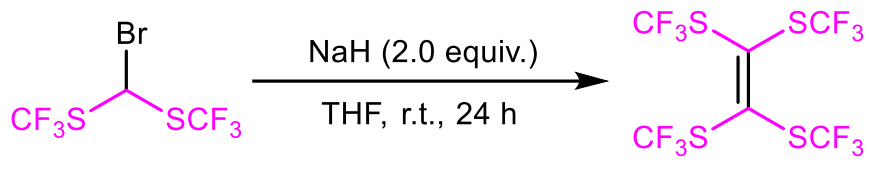

A 25 mL bottom flask was charged with NaH (96.0 mg, 4.0 mmol, 2.0 equiv.) and dry THF (7.5 mL) under a N_2 atmosphere. $BrCH(SCF_3)_2$ (590 mg, 2.0 mmol, 1.0 equiv.) was added. The resulting mixture was stirred at room temperature for 24 h under a N_2 atmosphere. The resulting mixture was diluted with pentane (30 mL) and water (30 mL). The organic phase was separated and the aqueous phase was extracted 3 times with pentane (10 mL×3). All organic solutions were combined and then dried over anhydrous Na_2SO_4. The solvent was removed under vacuum and the residue was subjected to flash column chromatography with an eluent of pentane ($R_f = 0.88$) to give 1,1,2,2-tetrakis((trifluoromethyl)thio)ethene (166.9 mg, 0.39 mmol, 39%) as a yellow oil.

1,1,2,2-Tetrakis((trifluoromethyl)thio)ethene

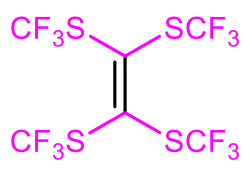

^{19}F NMR (376 MHz, $CDCl_3$) δ -39.40 (s, 12F).

^{13}C NMR (101 MHz, $CDCl_3$) δ 138.8 – 138.5 (m), 127.7 (q, J = 313.7 Hz).

HRMS (ESI) m/z: $[M]^+$ Calcd for $C_6F_{12}S_4$ 427.8686; Found 427.8692.

IR (KBr) 2958, 2923, 2853, 2725, 2673, 1460, 1377, 1302, 1174, 1099, 961, 852, 758, 722, 485 cm^{-1}

5 Procedures for the synthesis of substrates

The synthesis of alkenes 1n, 1s and 1u

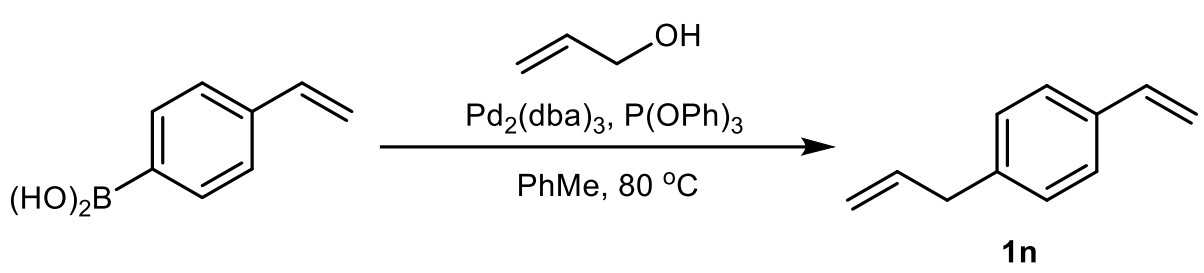

A 50 mL two-necked bottom flask was charged with $Pd_2(dba)_3$ (9.2 mg, 10 µmol, 0.1 mol%), 4-vinylphenyl boronic acid (1.78 g, 12 mmol, 1.2 equiv.) and toluene (13 mL) under an Ar atmosphere. Triphenylphosphite (5.0 µL, 20 µmol, 0.2 mol%) and allyl alcohol (580 mg, 10 mmol, 1 equiv.) were added and the mixture was stirred at 80 °C for 6 h. After being cooled to rt, the reaction mixture was diluted with *tert*-butyl methyl ether (100 mL) and washed with brine (60 mL), The organic layer was dried over Na_2SO_4. The solvent was removed under vacuum and the residue was subjected to flash column chromatography on silica gel with an eluent of petroleum ether (R_f = 0.75) to give substrate **1n** (763.2 mg, 53% yield) as a colorless oil.

1-Allyl-4-vinylbenzene (1n)[3]

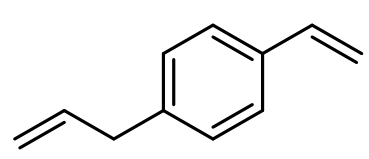

^{1}H NMR (400 MHz, $CDCl_3$ δ 7.33 (d, J = 7.7 Hz, 2H), 7.13 (d, J = 7.8 Hz, 2H), 6.68 (dd, J = 17.6, 10.9 Hz, 1H), 5.94 (ddt, J = 17.1, 10.4, 6.9 Hz, 1H), 5.70 (d, J = 17.6 Hz, 1H), 5.18 (d, J = 10.9 Hz, 1H), 5.12 – 4.96 (m, 2H), 3.36 (d, J = 6.7 Hz, 2H).

^{13}C NMR (101 MHz, $CDCl_3$) δ 139.9, 137.5, 136.8, 135.7, 128.9, 126.5, 116.0, 113.3, 40.1.

LRMS (EI) m/z: $[M]^+$ Calcd for $C_{11}H_{12}$ 144.1; Found 144.1.

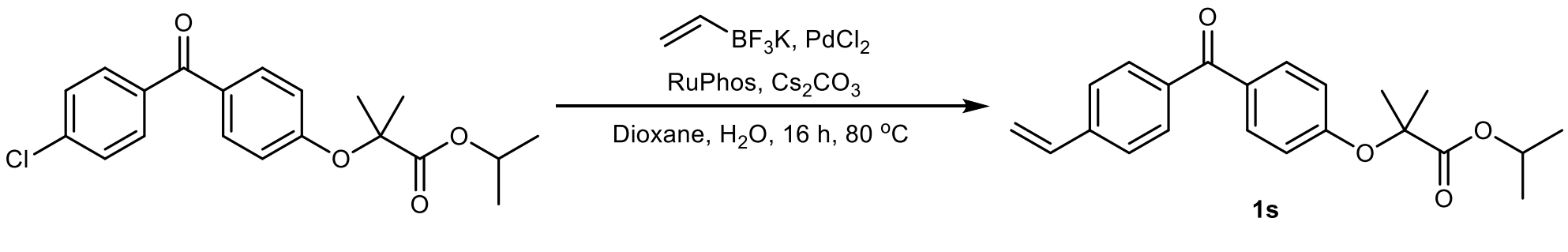

A 250 mL two-neck round-bottom flask was charged with fenofibrate (9.02 g, 25 mmol, 1.0 equiv.), potassium vinyltrifluoroborate (5.02 g, 37.5 mmol, 1.5 equiv.), palladium

(II) chloride (221.7 mg, 1.25 mmol, 5 mol%), RuPhos (1.17 g, 2.5 mmol, 10 mol%) and cesium carbonate (24.4 g, 75 mmol, 3.0 equiv.) under a N_2 atmosphere. Dioxane (100 mL) and distilled water (15 mL) were added, the mixture was stirred at 85 ºC for 16 h. Water (200 mL) was added and the mixture was extracted with Et_2O (100 mL× 3). The combined organic layers were dried over Na_2SO_4. The solvent was removed under vacuum and the residue was subjected to flash column chromatography to give substrate **1s**.

Isopropyl 2-methyl-2-(4-(4-vinylbenzoyl)phenoxy)propanoate (1s)

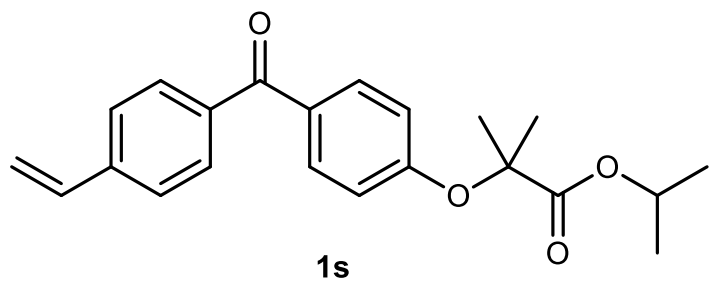

1s

The residue was purified by column chromatography on silica gel with an eluent of petroleum ether/EA (10:1, R_f = 0.54) to afford product **1s** (5.91g, 67% yield) as a white solid.

^{1}H NMR (400 MHz, $CDCl_3$) δ 7.78 – 7.69 (m, 4H), 7.51 – 7.45 (m, 2H), 6.89 – 6.83 (m, 2H), 6.76 (dd, J = 17.6, 10.9 Hz, 1H), 5.86 (d, J = 17.6, 1H), 5.37 (d, J = 10.9 Hz, 1H), 5.08 (sept, J = 6.3 Hz, 1H), 1.65 (s, 6H), 1.19 (d, J = 6.3 Hz, 6H).

^{13}C NMR (101 MHz, $CDCl_3$) δ 195.2, 173.3, 159.7, 141.3, 137.4, 136.2, 132.1, 130.9, 130.4, 126.1, 117.4, 116.5, 79.6, 69.5, 25.6, 21.7.

LRMS (EI) m/z: $[M]^+$ Calcd for $C_{22}H_{24}O_4$ 352.2; Found 352.1.

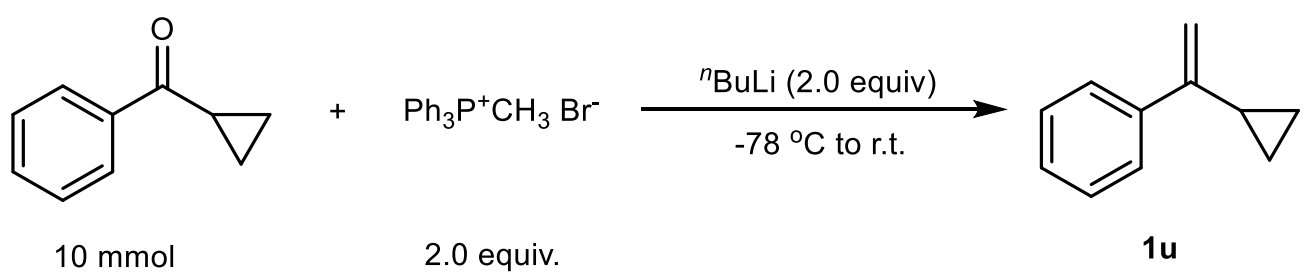

A 250 mL round bottom flask was charged with methyltriphenylphosphonium bromide (7.2 g, 20 mmol, 2.0 equiv) and anhydrous THF (60 mL), and the mixture was cooled to -78 °C under an argon atmosphere. ^{n}BuLi (8.0 mL, 2.5 M, 20 mmol) was added dropwise within 45 min, after which the cooling bath was removed. To this mixture was added the solution of cyclopropyl phenyl ketone (1.46 g, 10.0 mmol, 1.0 equiv) in anhydrous THF (6 mL) and the reaction was stirred for 1 h. Brine (30 mL) was added

and the phases were separated. The aqueous phase was extracted with pentane (3 × 80 mL), and the combined organic layers were dried over Na_2SO_4. The solvent was removed under vacuum, and the residue was subjected to flash column chromatography on silica gel with an eluent of pentane petroleum ether (R_f = 0.69) to afford substrate **1u** (1.28 g, 89% yield) as a colorless oil.

(1-Cyclopropylvinyl)benzene (1u)[4]

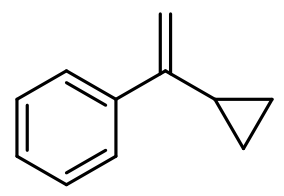

1u

^{1}H NMR (400 MHz, $CDCl_3$) δ 7.68 – 7.60 (m, 2H), 7.42 – 7.35 (m, 2H), 7.35 – 7.29 (m, 1H), 5.32 (d, J = 1.1 Hz, 1H), 4.98 (d, J = 1.3 Hz, 1H), 1.75 – 1.63 (m, 1H), 0.92 – 0.84 (m, 2H), 0.68 – 0.59 (m, 2H).

^{13}C NMR (101 MHz, $CDCl_3$) δ 149.6, 141.9, 128.3, 127.6, 126.3, 109.2, 15.8, 6.9.

LRMS (EI) m/z: $[M]^+$ Calcd for $C_{11}H_{12}$ 144.1; Found 144.1.

The synthesis of indoles 3g, 3o and pyroles 3q, 3t, 3u

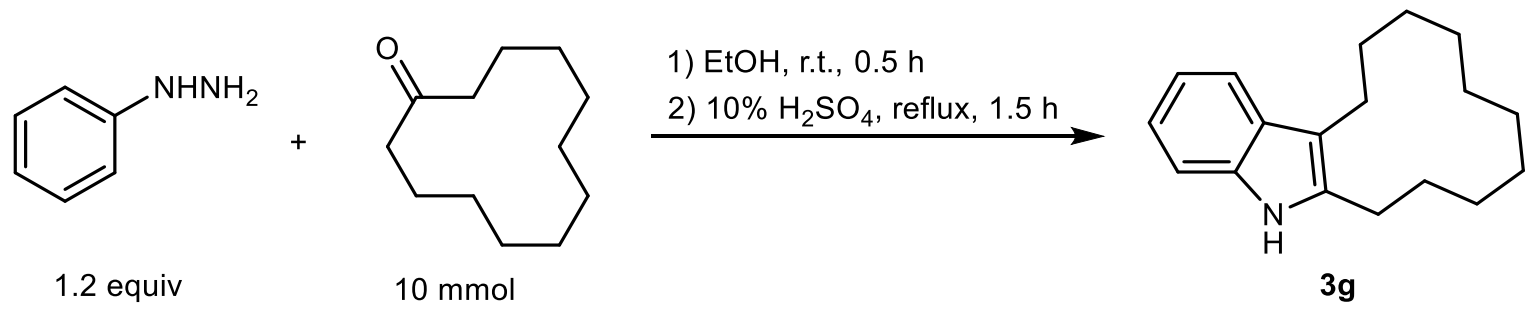

A 100 mL round-bottom flask was charged with a solution of cyclododecanone (1.82 g, 10.0 mmol, 1.0 equiv) in 10 ml of ethanol. Distilled phenylhydrazine (1.30 g, 12.0 mmol, 1.2 equiv) was added, and the mixture was stirred at room temperature for 0.5 h. After evaporation of the solvent, the crude phenylhydrazone was stirred at reflux temperature in 50 ml of 10% H_2SO_4 for 1.5 h. The obtained mixture was extracted with DCM (50 mL × 3) . The combined organic layers were dried over Na_2SO_4. The solvent was removed under vacuum, and the residue was subjected to flash column chromatography on silica gel with an eluent of petroleum ether/EA (20:1, R_f = 0.55), followed by recrystallization from hexane to afford substrate **3g** (968.7 mg, 38% yield) as a white solid.

6,7,8,9,10,11,12,13,14,15-Decahydro-5*H*-cyclododeca[*b*]indole (3g)[5]

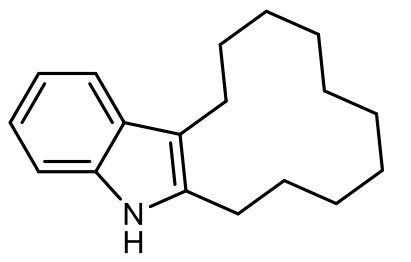

3g

^{1}H NMR (400 MHz, $CDCl_3$) δ 7.53 (d, *J* = 7.4 Hz, 1H), 7.45 (s, 1H), 7.18 (d, *J* = 7.7 Hz, 1H), 7.14 – 7.01 (m, 2H), 2.71 (t, *J* = 6.8 Hz, 2H), 2.65 (t, *J* = 7.2 Hz, 2H), 1.84 – 1.74 (m, 2H), 1.73 – 1.62 (m, 2H), 1.51 – 1.38 (m, 4H), 1.37 – 1.26 (m, 6H), 1.28 – 1.16 (m, 2H).

^{13}C NMR (101 MHz, $CDCl_3$) δ 136.0, 135.9, 128.8, 121.1, 119.0, 118.9, 112.4, 110.4, 27.9, 27.5, 25.03, 25.00, 24.2, 22.8, 22.7, 22.3, 21.4.

LRMS (EI) m/z: $[M]^+$ Calcd for $C_{18}H_{25}N$ 255.2; Found 255.2.

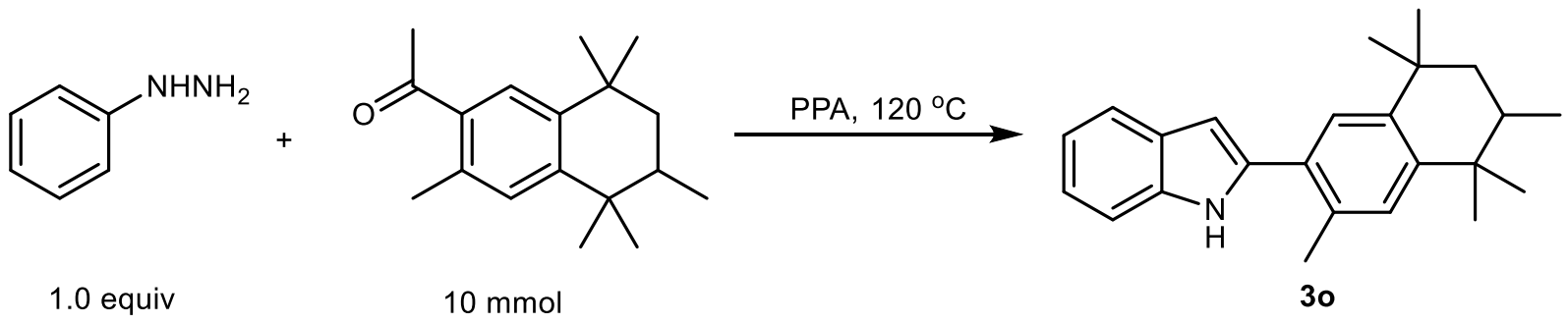

A 100 mL round-bottom flask was charged with tonalid (2.58 g, 10 mmol, 1.0 equiv), phenylhydrazine (1.08 g, 10 mmol, 1.0 equiv), and polyphosphoric acid (PPA, 10.0 mL). The reaction mixture was stirred at 120 °C for 5 h. The reaction mixture was poured into cold water (70 mL) and extracted with EA (100 mL×3). The combined organic layers were dried over anhydrous Na_2SO_4. The solvent was removed under vacuum, and the residue was subjected to flash column chromatography on silica gel with an eluent of petroleum ether/EA (20:1, R_f = 0.49), followed by recrystallization from hexane to afford substrate **3o** (762.0 mg, 23% yield) as a white solid.

2-(3,5,5,6,8,8-Hexamethyl-5,6,7,8-tetrahydronaphthalen-2-yl)-1*H*-indole (3o)[6]

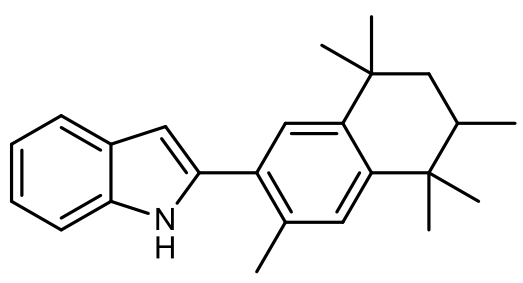

3o

^{1}H NMR (400 MHz, $CDCl_3$) δ 8.19 (s, 1H), 7.76 (d, *J* = 7.8 Hz, 1H), 7.52 (s, 1H), 7.50 (d, *J* = 8.3 Hz, 1H), 7.42 (s, 1H), 7.35 – 7.26 (m, 1H), 7.29 – 7.20 (m, 1H), 6.72 (s, 1H), 2.57 (s, 3H), 2.10 – 1.98 (m, 1H), 1.80 (t, *J* = 13.2 Hz, 1H), 1.57 – 1.49 (m, 4H), 1.47 (s, 3H), 1.43 (s, 3H), 1.25 (s, 3H), 1.14 (d, *J* = 6.7 Hz, 3H).

^{13}C NMR (101 MHz, $CDCl_3$) δ 146.3, 142.9, 138.1, 136.2, 133.3, 130.2, 129.7, 129.1, 127.4, 122.0, 120.6, 120.2, 110.9, 102.8, 43.9, 37.8, 34.8, 34.3, 32.7, 32.3, 28.8, 25.2, 21.0, 17.1.

LRMS (EI) m/z: $[M]^+$ Calcd for $C_{24}H_{29}N$ 331.2; Found 331.3.

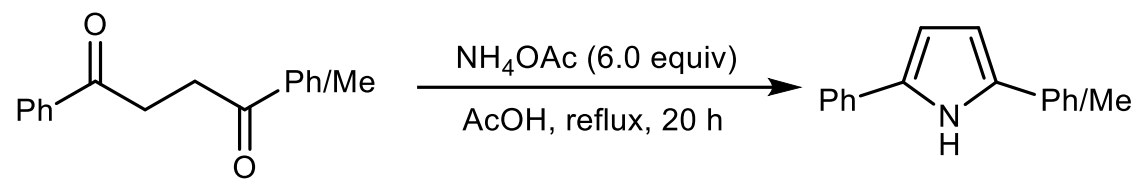

A 250 mL round-bottom flask was charged with 1,4-diphenylbutane-1,4-dione or 1-phenylpentane-1,4-dione (10 mmol, 1.0 equiv), ammonium acetate (4.62 g, 60 mmol, 6.0 equiv) and acetic acid (70 mL), and the resulting solution was refluxed for 20 h. After being cooled to room temperature, the reaction mixture was poured into ice-water. The formed solid was collected and washed with H_2O and dried to give substrate **3q** or **3t**.

2,5-Diphenyl-1*H*-pyrrole (3q)[7]

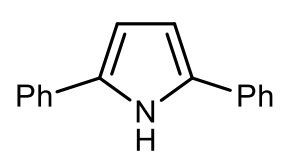

3q

3q (2.08 g, 95% yield) was obtained as a brown solid.

^{1}H NMR (400 MHz, $CDCl_3$) δ 8.54 (s, 1H), 7.50 (d, *J* = 7.6 Hz, 4H), 7.36 (t, *J* = 7.5 Hz, 4H), 7.20 (t, *J* = 7.2 Hz, 2H), 6.56 (s, 2H).

^{13}C NMR (101 MHz, $CDCl_3$) δ 133.3, 132.6, 129.1, 126.5, 124.0, 108.1.

LRMS (EI) m/z: $[M]^+$ Calcd for $C_{16}H_{13}N$ 219.1; Found 219.1.

2-Methyl-5-phenyl-1*H*-pyrrole (3t)[7]

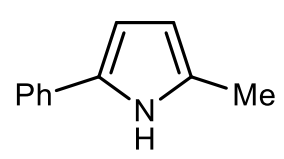

3q

3t (1.02 g, 65% yield) was obtained as a brown solid.

^{1}H NMR (400 MHz, $CDCl_3$) δ 8.14 (s, 1H), 7.50 – 7.43 (m, 2H), 7.37 (t, J = 7.6 Hz, 2H), 7.24 – 7.17 (m, 1H), 6.45 (t, J = 3.0 Hz, 1H), 6.01 (t, J = 2.9 Hz, 1H), 2.36 (s, 3H).

^{13}C NMR (101 MHz, $CDCl_3$) δ 133.1, 131.0, 129.2, 129.0, 125.8, 123.5, 108.1, 106.4, 13.4.

LRMS (EI) m/z: $[M]^+$ Calcd for $C_{11}H_{11}N$ 157.1; Found 157.1.

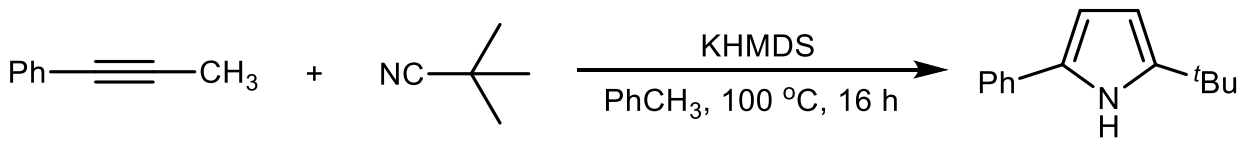

3u

An oven-dried 100 mL vial equipped with a stir bar was charged with prop-1-yn-1-ylbenzene (1.16 g, 10 mmol, 1.0 equiv) under a nitrogen atmosphere in a glovebox. A solution of Pivalonitrile (996 mg, 12 mmol, 1.2 equiv) in dry toluene (30 mL) was added to the reaction vial followed by addition of KHMDS (1.0 mol/L in THF, 30 mL, 30 mmol, 3.0 equiv) by syringe at room temperature to give a homogeneous, clear solution. Upon addition of the base, the color of the reaction mixture turned to brown. The vial was sealed and stirred at 100 °C for 16 h. The vial was removed from the bath and cooled to room temperature. The reaction mixture was quenched with saturated NH_4Cl solution. The resulting solution was passed through a short pad of silica gel and eluted with ethyl acetate (100 mL × 3). The combined organic layers were dried over anhydrous Na_2SO_4. The solvent was removed under vacuum and the residue was subjected to flash column chromatography to give substrate **3u**.

2-(*tert*-Butyl)-5-phenyl-1*H*-pyrrole (3u)[8]

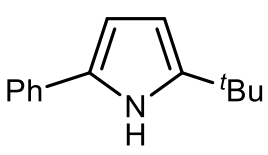

3u

The residue was purified by column chromatography on silica gel with an eluent of petroleum ether/EA (30:1, R_f = 0.57) to afford substrate **3u** (895.2 mg, 48% yield) as a yellow oil.

^{1}H NMR (400 MHz, $CDCl_3$) δ 8.11 (s, 1H), 7.47 – 7.39 (m, 2H), 7.37 – 7.29 (m, 2H), 7.19 – 7.11 (m, 1H), 6.40 (t, J = 3.1 Hz, 1H), 5.99 (t, J = 3.0 Hz, 1H), 1.33 (s, 9H).

^{13}C NMR (101 MHz, $CDCl_3$) δ 143.4, 133.3, 130.6, 129.0, 125.9, 123.7, 105.9, 104.7, 31.7, 30.8.

LRMS (EI) m/z: $[M]^+$ Calcd for $C_{14}H_{17}N$ 199.1; Found 199.2.

The synthesis of allyl sufides 7b-7u

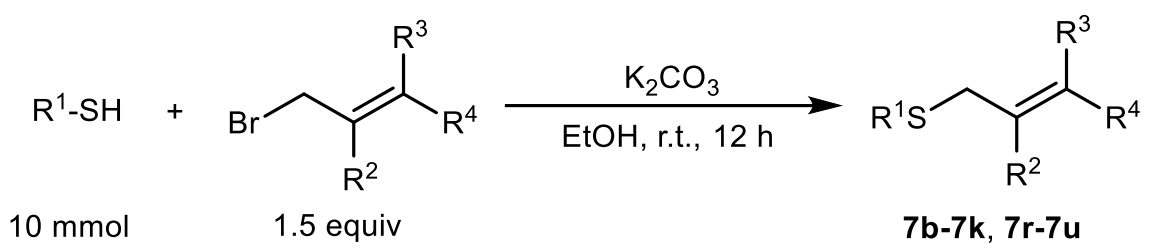

A 100 mL round-bottom flask was charged with thiol (10 mmol, 1.0 equiv), K_2CO_3 (4.14 g, 30 mmol, 3 equiv.) and EtOH (20 mL) at room temperature. Allyl bromide (15 mmol, 1.5 equiv) was added to this solution and the reaction was allowed to stir at room temperature for 12 h. Then the solvent was removed under vacuum, and water (50 mL) was added to the residue. The obtained aqueous phase was extracted with DCM (3 × 50 mL), and the combined organic layers were dried over Na_2SO_4. The solvent was removed under vacuum and the residue was subjected to flash column chromatography to give substrate **7b-7k**, **7q-7t**.

Allyl(4-(*tert*-butyl)phenyl)sulfane (7b)[9]

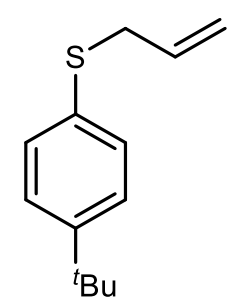

7b

The residue was purified by column chromatography on silica gel with an eluent of petroleum ether (R_f = 0.33) to afford substrate **7b** (1.51 g, 73% yield) as a colorless oil.

^{1}H NMR (400 MHz, $CDCl_3$) δ 7.37 (s, 4H), 6.03 – 5.89 (m, 1H), 5.25 – 5.18 (m, 1H), 5.17 – 5.09 (m, 1H), 3.59 (d, J = 6.8 Hz, 2H), 1.38 (s, 9H).

^{13}C NMR (101 MHz, $CDCl_3$) δ 149.5, 134.0, 132.6, 130.0, 125.9, 117.6, 37.6, 34.6, 31.4.

LRMS (EI) m/z: $[M]^+$ Calcd for $C_{13}H_{18}S$ 206.1; Found 206.2.

[1,1'-Biphenyl]-4-yl(allyl)sulfane (7c)

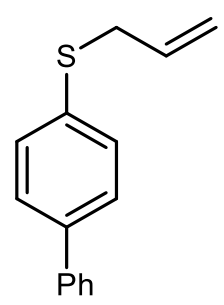

7c

The residue was purified by column chromatography on silica gel with an eluent of petroleum ether (R_f = 0.26) to afford substrate **7c** (1.85 g, 82% yield) as a white solid, m.p. 72-74 ºC.

^{1}H NMR (400 MHz, $CDCl_3$) δ 7.64 – 7.58 (m, 2H), 7.58 – 7.52 (m, 2H), 7.51 – 7.41 (m, 4H), 7.41 – 7.34 (m, 1H), 6.01 – 5.89 (m, 1H), 5.27 – 5.17 (m, 1H), 5.18 – 5.10 (m, 1H), 3.62 (d, J = 6.9 Hz, 2H).

^{13}C NMR (101 MHz, $CDCl_3$) δ 140.6, 139.3, 135.3, 133.7, 130.2, 129.0, 127.6, 127.5, 127.1, 118.0, 37.3.

HRMS (FI) m/z: $[M]^+$ Calcd for $C_{15}H_{14}S$ 226.0811; Found 226.0815.

IR (KBr) 3082, 3056, 3027, 2979, 2919, 1636, 1600, 1479, 1447, 1435, 1425, 1397, 1339, 1265, 1228, 1129, 1095, 1075, 1006, 987, 920, 823, 753, 739, 697, 686, 593, 545, 501, 471 cm^{-1}

Allyl(naphthalen-2-yl)sulfane (7d)[9]

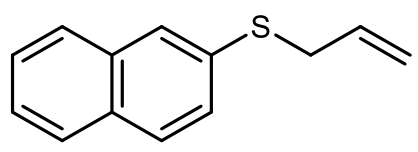

7d

The residue was purified by column chromatography on silica gel with an eluent of petroleum ether (R_f = 0.41) to afford substrate **7d** (1.63 g, 82% yield) as a colorless oil.

^{1}H NMR (400 MHz, $CDCl_3$) δ 7.85 – 7.78 (m, 2H), 7.78 (d, J = 8.4 Hz, 2H), 7.55 – 7.42 (m, 3H), 6.03 – 5.91 (m, 1H), 5.26 – 5.19 (m, 1H), 5.17 – 5.09 (m, 1H), 3.69 (d, J = 6.9 Hz, 2H).

^{13}C NMR (101 MHz, $CDCl_3$) δ 133.9, 133.6, 132.0, 128.4, 127.91, 127.87, 127.8, 127.3, 126.6, 125.9, 118.0, 37.2.

LRMS (EI) m/z: $[M]^+$ Calcd for $C_{13}H_{12}S$ 200.1; Found 200.1.

Allyl(4-methoxyphenyl)sulfane (7e)[10]

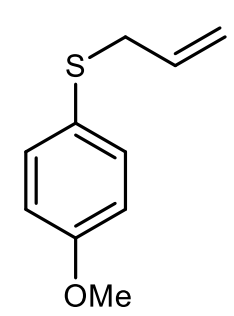

7e

The residue was purified by column chromatography on silica gel with an eluent of petroleum ether/EA (20:1, R_f = 0.50) to afford substrate **7e** (1.33 g, 74% yield) as a colorless oil.

^{1}H NMR (400 MHz, $CDCl_3$) δ 7.38 – 7.33 (m, 2H), 6.88 – 6.81 (m, 2H), 5.92 – 5.80 (m, 1H), 5.05 – 4.99 (m, 1H), 5.01 – 4.97 (m, 1H), 3.77 (s, 3H), 3.44 (d, *J* = 7.1 Hz, 2H).

^{13}C NMR (101 MHz, $CDCl_3$) δ 159.1, 134.1, 133.9, 125.9, 117.3, 114.5, 55.3, 39.3.

LRMS (EI) m/z: $[M]^+$ Calcd for $C_{10}H_{12}OS$ 180.1; Found 180.1.

Allyl(4-(methylthio)phenyl)sulfane (7f)

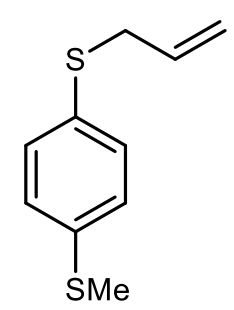

7f

The residue was purified by column chromatography on silica gel with an eluent of petroleum ether (R_f = 0.24) to afford substrate **7f** (1.57 g, 80% yield) as a colorless oil.

^{1}H NMR (400 MHz, $CDCl_3$) δ 7.28 – 7.22 (m, 2H), 7.17 – 7.11 (m, 2H), 5.89 – 5.77 (m, 1H), 5.12 – 5.00 (m, 2H), 3.47 (d, *J* = 6.9 Hz, 2H), 2.42 (s, 3H).

^{13}C NMR (101 MHz, $CDCl_3$) δ 136.9, 133.6, 132.1, 131.0, 127.0, 117.6, 37.8, 15.9.

HRMS (FI) m/z: $[M]^+$ Calcd for $C_{10}H_{12}S_2$ 196.0375; Found 196.0380.

IR (KBr) 3079, 3009, 2978, 2918, 2857, 2835, 1890, 1844, 1635, 1575, 1477, 1435, 1405, 1387, 1339, 1319, 1298, 1260, 1227, 1186, 1106, 1087, 1011, 987, 968, 918, 870, 807, 749, 734, 595, 536, 493, 422 cm^{-1}

Allyl(4-chlorophenyl)sulfane (7g)[10]

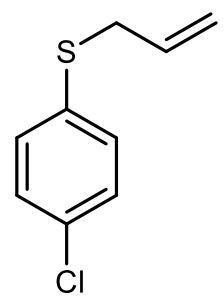

7g

The residue was purified by column chromatography on silica gel with an eluent of petroleum ether (R_f = 0.48) to afford substrate **7g** (1.25 g, 68% yield) as a colorless oil.

^{1}H NMR (400 MHz, $CDCl_3$) δ 7.27 – 7.20 (m, 4H), 5.83 (ddt, J = 16.9, 10.0, 6.9 Hz, 1H), 5.14 – 5.03 (m, 2H), 3.50 (d, J = 6.8 Hz, 2H).

^{13}C NMR (101 MHz, $CDCl_3$) δ 134.5, 133.4, 132.4, 131.4, 129.1, 118.1, 37.6.

LRMS (EI) m/z: $[M]^+$ Calcd for C_9H_9ClS 184.0; Found 184.1.

Allyl(4-bromophenyl)sulfane (7h)[11]

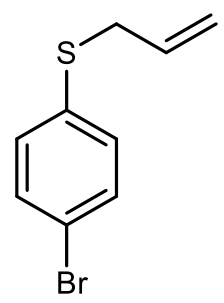

7h

The residue was purified by column chromatography on silica gel with an eluent of petroleum ether (R_f = 0.53) to afford substrate **7h** (1.77 g, 78% yield) as a colorless oil.

^{1}H NMR (400 MHz, $CDCl_3$) δ 7.42 – 7.36 (m, 2H), 7.22 – 7.16 (m, 2H), 5.92 – 5.79 (m, 1H), 5.17 – 5.06 (m, 2H), 3.61 – 3.45 (m, 2H).

^{13}C NMR (101 MHz, $CDCl_3$) δ 135.2, 133.4, 131.9, 131.5, 120.2, 118.1, 37.3.

LRMS (EI) m/z: $[M]^+$ Calcd for C_9H_9BrS 228.0; Found 228.0.

Allyl(4-(trifluoromethyl)phenyl)sulfane (7i)[12]

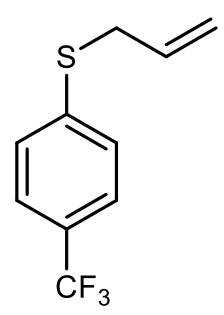

7i

The residue was purified by column chromatography on silica gel with an eluent of petroleum ether (R_f = 0.46) to afford substrate **7i** (1.69 g, 78% yield) as a colorless oil.

^{1}H NMR (400 MHz, $CDCl_3$) δ 7.52 (d, *J* = 8.3 Hz, 2H), 7.38 (d, *J* = 8.2 Hz, 2H), 5.90 (ddt, *J* = 16.8, 10.0, 6.7 Hz, 1H), 5.25 (dq, *J* = 17.0, 1.4 Hz, 1H), 5.16 (dq, *J* = 10.1, 1.2 Hz, 1H), 3.63 (d, *J* = 6.7 Hz, 2H).

^{19}F NMR (376 MHz, $CDCl_3$) δ -62.48 (s, 3F).

^{13}C NMR (101 MHz, $CDCl_3$) δ 141.9, 132.9, 128.2, 127.8 (q, *J* = 32.6 Hz), 125.7 (q, *J* = 3.8 Hz), 124.4 (q, *J* = 271.1 Hz), 118.6, 36.0.

LRMS (EI) m/z: $[M]^+$ Calcd for $C_{10}H_9F_3S$ 218.0; Found 218.1.

4-(Allylthio)benzonitrile (7j)[13]

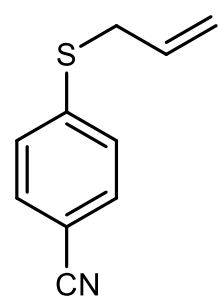

7j

The residue was purified by column chromatography on silica gel with an eluent of petroleum ether/EA (20:1, R_f = 0.33) to afford substrate **7j** (1.21 g, 69% yield) as a colorless oil.

^{1}H NMR (400 MHz, $CDCl_3$) δ 7.53 – 7.44 (m, 2H), 7.33 – 7.24 (m, 2H), 5.84 (ddt, *J* = 16.7, 10.1, 6.6 Hz, 1H), 5.26 (dq, *J* = 16.9, 1.4 Hz, 1H), 5.15 (dq, *J* = 10.0, 1.2 Hz, 1H), 3.61 (dt, *J* = 6.6, 1.4 Hz, 2H).

^{13}C NMR (101 MHz, $CDCl_3$) δ 144.1, 132.23, 132.17, 127.4, 118.9, 118.8, 108.4, 35.2.

LRMS (EI) m/z: $[M]^+$ Calcd for $C_{10}H_9NS$ 175.0; Found 175.1.

Allyl(4-nitrophenyl)sulfane (7k)[10]

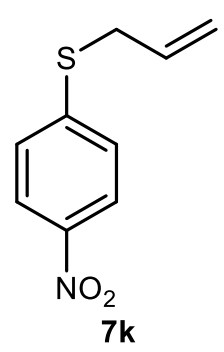

7k

The residue was purified by column chromatography on silica gel with an eluent of petroleum ether/EA (20:1, R_f = 0.43) to afford substrate **7k** (1.16 g, 59% yield) as a yellow solid.

^{1}H NMR (400 MHz, $CDCl_3$) δ 8.07 (d, *J* = 9.0 Hz, 2H), 7.30 (d, *J* = 8.6 Hz, 2H), 5.86 (ddt, *J* = 16.7, 10.0, 6.5 Hz, 1H), 5.35 – 5.26 (m, 1H), 5.18 (d, *J* = 10.1 Hz, 1H), 3.66 (d, *J* = 6.6 Hz, 2H).

^{13}C NMR (101 MHz, $CDCl_3$) δ 147.0, 145.2, 132.0, 126.8, 123.9, 119.1, 35.2.

LRMS (EI) m/z: $[M]^+$ Calcd for $C_9H_9NO_2S$ 195.0; Found 195.1.

2-(Allylthio)thiophene (7r)[11]

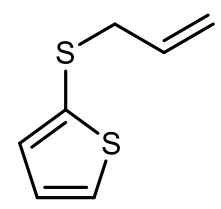

7r

The residue was purified by column chromatography on silica gel with an eluent of petroleum ether (R_f = 0.61) to afford substrate **7r** (1.01 g, 65% yield) as a yellow oil.

^{1}H NMR (400 MHz, $CDCl_3$) δ 7.38 – 7.31 (m, 1H), 7.17 – 7.09 (m, 1H), 7.02 – 6.94 (m, 1H), 5.97 – 5.80 (m, 1H), 5.09 – 5.03 (m, 1H), 5.05 – 4.94 (m, 1H), 3.41 (d, *J* = 7.3 Hz, 2H).

^{13}C NMR (101 MHz, $CDCl_3$) δ 134.3, 133.8, 133.7, 129.6, 127.6, 118.0, 42.2.

LRMS (EI) m/z: $[M]^+$ Calcd for $C_7H_8S_2$ 156.0; Found 156.0.

2-(Allylthio)benzo[*d*]thiazole (7s)[14]

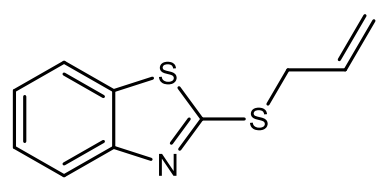

7s

The residue was purified by column chromatography on silica gel with an eluent of petroleum ether/EA (20:1, R_f = 0.41) to afford substrate **7s** (1.19 g, 57% yield) as a yellow oil.

^{1}H NMR (400 MHz, $CDCl_3$) δ 7.89 (d, *J* = 8.1 Hz, 1H), 7.74 (d, *J* = 7.9 Hz, 1H), 7.46 – 7.37 (m, 1H), 7.32 – 7.26 (m, 1H), 6.03 (ddt, *J* = 16.9, 10.0, 7.0 Hz, 1H), 5.43 – 5.34 (m, 1H), 5.25 – 5.17 (m, 1H), 4.00 (d, *J* = 6.9 Hz, 2H).

^{13}C NMR (101 MHz, $CDCl_3$) δ 166.3, 153.3, 135.4, 132.4, 126.2, 124.4, 121.7, 121.1, 119.3, 36.3.

LRMS (EI) m/z: $[M]^+$ Calcd for $C_{10}H_9NS_2$ 207.0; Found 207.1.

Ethyl 2-(allylthio)acetate (7t)[15]

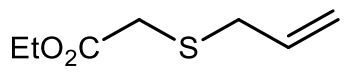

7t

The residue was purified by column chromatography on silica gel with an eluent of petroleum ether/EA (20:1, R_f = 0.47) to afford substrate **7t** (1.03 g, 64% yield) as a colorless oil.

^{1}H NMR (400 MHz, $CDCl_3$) δ 5.76 – 5.63 (m, 1H), 5.13 – 5.05 (m, 2H), 4.11 (q, J = 7.1 Hz, 2H), 3.18 (d, J = 7.3 Hz, 2H), 3.09 (s, 2H), 1.22 (t, J = 7.1 Hz, 3H).

^{13}C NMR (101 MHz, $CDCl_3$) δ 170.5, 133.0, 118.3, 61.2, 35.0, 31.8, 14.2.

LRMS (EI) m/z: $[M]^+$ Calcd for $C_7H_{12}O_2S$ 160.1; Found 160.1.

Allyl(*tert*-butyl)sulfane (7u)[16]

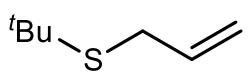

7u

The residue was purified by column chromatography on silica gel with an eluent of petroleum ether (R_f = 0.41) to afford substrate **7u** (783.2 mg, 60% yield) as a colorless oil.

^{1}H NMR (400 MHz, $CDCl_3$) δ 5.85 (ddt, J = 17.0, 10.0, 7.0 Hz, 1H), 5.16 (dq, J = 17.0, 1.4 Hz, 1H), 5.04 – 4.99 (m, 1H), 3.19 (dt, J = 7.1, 1.3 Hz, 2H), 1.31 (s, 9H).

^{13}C NMR (101 MHz, $CDCl_3$) δ 135.6, 116.8, 42.7, 32.2, 31.2.

LRMS (EI) m/z: $[M]^+$ Calcd for $C_7H_{14}S$ 130.1; Found 130.1.

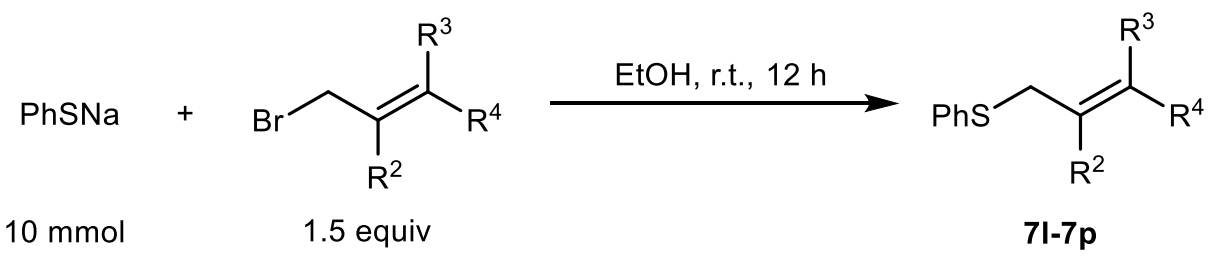

A 100 mL round-bottom flask was charged with sodium thiophenolate (1.32 g, 10 mmol, 1.0 equiv) and EtOH (20 mL) at room temperature. Allyl bromide (15 mmol, 1.5 equiv) was added to this solution and the reaction was allowed to stir at room temperature for 12 h. Then the solvent was removed under vacuum, and water (50 mL) was added to the residue. The obtained aqueous phase was extracted with DCM (3 × 50 mL), and the combined organic layers were dried over Na_2SO_4. The solvent was removed under vacuum and the residue was subjected to flash column chromatography to give

substrate **7l-7p** (3-bromo-3,3-difluoroprop-1-ene was used for the synthesis of substrate **7m**)

Ethyl 2-((phenylthio)methyl)acrylate (7l)[17]

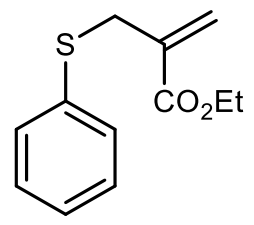

7l

The residue was purified by column chromatography on silica gel with an eluent of petroleum ether/EA (20:1, R_f = 0.43) to afford substrate **7l** (1.42 g, 64% yield) as a colorless oil.

^{1}H NMR (400 MHz, $CDCl_3$) δ 7.36 – 7.31 (m, 2H), 7.29 – 7.21 (m, 2H), 7.20 – 7.14 (m, 1H), 6.14 (s, 1H), 5.52 (s, 1H), 4.22 (q, *J* = 7.1 Hz, 2H), 3.75 (s, 2H), 1.28 (t, *J* = 7.2 Hz, 3H).

^{13}C NMR (101 MHz, $CDCl_3$) δ 165.9, 136.5, 135.5, 130.7, 128.8, 126.7, 126.5, 60.9, 35.6, 14.2.

LRMS (EI) m/z: $[M]^+$ Calcd for $C_{12}H_{14}O_2S$ 222.1; Found 222.1.

(3,3-Difluoroallyl)(phenyl)sulfane (7m)[18]

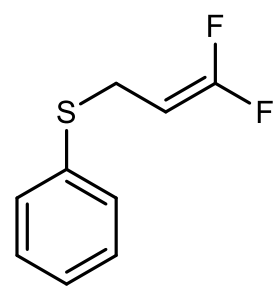

7m

The residue was purified by column chromatography on silica gel with an eluent of petroleum ether (R_f = 0.34) to afford substrate **7m** (1.33 g, 72% yield) as a colorless oil.

^{1}H NMR (400 MHz, $CDCl_3$) δ 7.40 – 7.32 (m, 2H), 7.33 – 7.25 (m, 2H), 7.25 – 7.19 (m, 1H), 4.35 (dtd, *J* = 24.0, 8.1, 1.6 Hz, 1H), 3.48 (dt, *J* = 8.2, 1.6 Hz, 2H).

^{19}F NMR (376 MHz, $CDCl_3$) δ -86.43 (d, *J* = 38.7 Hz, 1F), -88.45 (dd, *J* = 38.5, 24.0 Hz, 1F).

^{13}C NMR (101 MHz, $CDCl_3$) δ 157.3 (dd, *J* = 290.2, 288.8 Hz), 135.0, 130.8, 129.2, 127.1, 76.5 (dd, *J* = 25.0, 18.8 Hz), 27.6 (d, *J* = 6.1 Hz).

LRMS (EI) m/z: $[M]^+$ Calcd for $C_9H_8F_2S$ 186.0; Found 186.1.

But-2-en-1-yl(phenyl)sulfane (7n)[19]

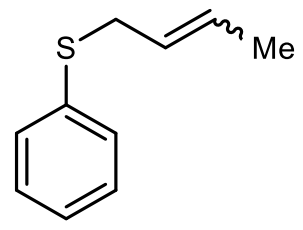

7n

The residue was purified by column chromatography on silica gel with an eluent of petroleum ether (R_f = 0.44) to afford substrate **7n** (1.23 g, 75% yield, *E*/*Z* =1:0.22) as a colorless oil.

^{1}H NMR (400 MHz, $CDCl_3$) δ 7.41 – 7.34 (m, 2H, both *E*- and *Z*-isomers), 7.33 – 7.27 (m, 2H, both *E*- and *Z*-isomers), 7.24 – 7.16 (m, 1H, both *E*- and *Z*-isomers), 5.67 – 5.50 (m, 2H, both *E*- and *Z*-isomers), 3.61 (d, *J* = 6.2 Hz, 2H, *Z*-isomer), 3.55 (d, *J* = 6.1 Hz, 2H, *E*-isomer), 1.68 (d, *J* = 5.4 Hz, 3H, *E*-isomer), 1.60 (d, *J* = 6.1 Hz, 3H, *Z*-isomer).

^{13}C NMR (101 MHz, $CDCl_3$) *E*-isomer: δ 130.1, 129.7, 129.1, 128.9, 126.3, 126.1, 36.5, 17.9. *Z*-isomer: δ 136.6, 129.7, 128.9, 127.8, 126.3, 125.6, 31.0, 12.8.

LRMS (EI) m/z: $[M]^+$ Calcd for $C_{10}H_{12}S$ 164.1; Found 164.1.

(3-Methylbut-2-en-1-yl)(phenyl)sulfane (7o)[20]

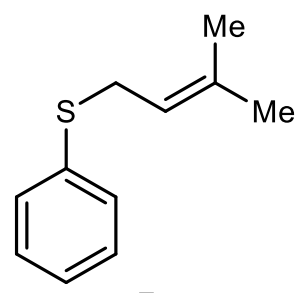

7o

The residue was purified by column chromatography on silica gel with an eluent of petroleum ether (R_f = 0.27) to afford substrate **7o** (1.25 g, 70% yield) as a colorless oil.

^{1}H NMR (400 MHz, $CDCl_3$) δ 7.41 – 7.35 (m, 2H), 7.33 – 7.27 (m, 2H), 7.24 – 7.18 (m, 1H), 5.40 – 5.32 (m, 1H), 3.59 (d, *J* = 7.7 Hz, 2H), 1.76 (s, 3H), 1.63 (s, 3H).

^{13}C NMR (101 MHz, $CDCl_3$) δ 137.0, 136.4, 129.8, 128.8, 126.0, 119.5, 32.3, 25.8, 17.8.

LRMS (EI) m/z: $[M]^+$ Calcd for $C_{11}H_{14}S$ 178.1; Found 178.1.

Cinnamyl(phenyl)sulfane (7p)[21]

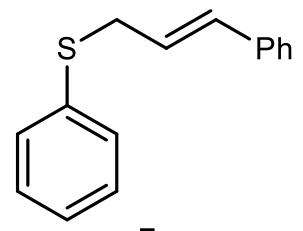

7p

The residue was purified by column chromatography on silica gel with an eluent of petroleum ether (R_f = 0.20) to afford substrate **7p** (1.81 g, 80% yield) as a white solid.

^{1}H NMR (400 MHz, $CDCl_3$) δ 7.40 (d, *J* = 7.6 Hz, 2H), 7.36 – 7.26 (m, 6H), 7.26 – 7.17 (m, 2H), 6.44 (d, *J* = 15.7 Hz, 1H), 6.27 (dt, *J* = 15.1, 7.1 Hz, 1H), 3.73 (d, *J* = 7.1 Hz, 2H).

^{13}C NMR (101 MHz, $CDCl_3$) δ 136.9, 136.0, 132.9, 130.4, 129.0, 128.7, 127.7, 126.52, 126.48, 125.2, 37.2.

LRMS (EI) m/z: $[M]^+$ Calcd for $C_{15}H_{14}S$ 226.1; Found 226.1.

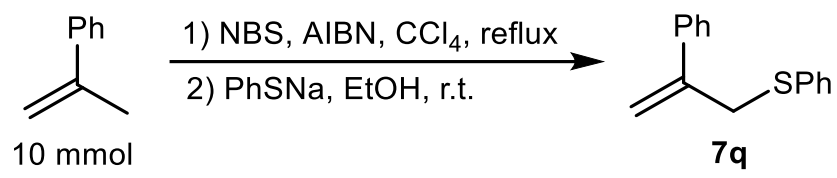

A two-neck 100 mL round-bottom flask equipped with a condenser was charged with AIBN (82.1 mg, 0.5 mmol, 5 mol%), NBS (1.96 g, 11 mmol, 1.1 equiv) and CCl_4 (25 mL) at room temperature. Isopropenylbenzene (1.3 mL, 10 mmol, 1.0 equiv) was added to this solution and the reaction was stirred at reflux temperature overnight. Then the solution was cooled to room temperature, and the solvent was removed under vacuum. EtOH (25 mL) was added to dissolve the residue, and sodium thiophenolate (1.32 g, 10 mmol, 1.0 equiv) was added. The mixture was stirred at room temperature for 12 h, and then the solvent was removed under vacuum. Water (50 mL) was added to the residue. The obtained aqueous phase was extracted with DCM (3 × 50 mL), and the combined organic layers were dried over Na_2SO_4. The solvent was removed under vacuum and the residue was subjected to flash column chromatography to give substrate **7q**.

Phenyl(2-phenylallyl)sulfane (7q)[22]

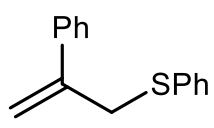

7q

The residue was purified by column chromatography on silica gel with an eluent of petroleum ether (R_f = 0.15) to afford substrate **7q** (1.78 g, 79% yield) as a yellow oil.

^{1}H NMR (400 MHz, $CDCl_3$) δ 7.62 – 7.55 (m, 2H), 7.52 – 7.42 (m, 5H), 7.42 – 7.36 (m, 2H), 7.36 – 7.29 (m, 1H), 5.53 (s, 1H), 5.32 (q, J = 0.9 Hz, 1H), 4.11 (d, J = 0.9 Hz, 2H).

^{13}C NMR (101 MHz, $CDCl_3$) δ 143.3, 139.7, 136.4, 130.5, 129.0, 128.6, 128.0, 126.6, 126.4, 115.8, 39.6.

LRMS (EI) m/z: $[M]^+$ Calcd for $C_{15}H_{14}S$ 226.1; Found 226.1.

The synthesis of *sec*-benzyl sulfides 9a-9q

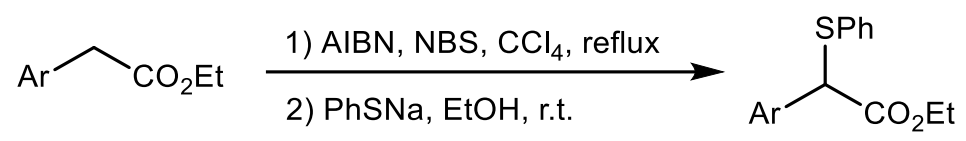

9a, **9j-9l**

A two-neck 100 mL round-bottom flask equipped with a condenser was charged with AIBN (82.1 mg, 0.5 mmol, 5 mol%), NBS (1.96 g, 11 mmol, 1.1 equiv) and CCl_4 (25 mL) at room temperature. Ethyl arylacetate (10 mmol, 1.0 equiv) was added to this solution and the reaction was allowed to stir at reflux temperature overnight. Then the solution was cooled to room temperature, and the solvent was removed under vacuum. EtOH (25 mL) was added to dissolve the residue, and sodium thiophenolate (1.32 g, 10 mmol, 1.0 equiv) was added. The mixture was stirred at room temperature for 12 h. The solvent was removed under vacuum, and water (50 mL) was added to the residue. The obtained aqueous phase was extracted with DCM (3 × 50 mL), and the combined organic layers were dried over Na_2SO_4. The solvent was removed under vacuum and the residue was subjected to flash column chromatography to give substrate **9a**, **9j-9l**.

Ethyl 2-phenyl-2-(phenylthio)acetate (9a)[23]

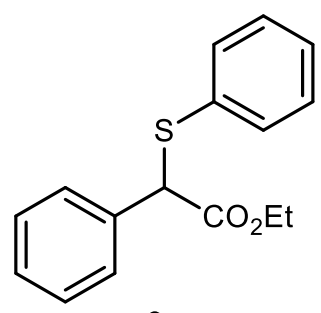

9a

The residue was purified by column chromatography on silica gel with an eluent of petroleum ether/EA (40:1, R_f = 0.54) to afford substrate **9a** (1.44 g, 53% yield) as a colorless oil.

^{1}H NMR (400 MHz, $CDCl_3$) δ 7.48 – 7.42 (m, 2H), 7.40 – 7.35 (m, 2H), 7.34 – 7.27 (m, 3H), 7.27 – 7.22 (m, 3H), 4.90 (s, 1H), 4.19 – 4.03 (m, 2H), 1.15 (t, J = 7.1 Hz, 3H).

^{13}C NMR (101 MHz, $CDCl_3$) δ 170.6, 135.8, 134.0, 132.8, 129.1, 128.8, 128.7, 128.4, 128.1, 61.9, 56.5, 14.1.

LRMS (EI) m/z: $[M]^+$ Calcd for $C_{16}H_{16}O_2S$ 272.1; Found 272.1.

Ethyl 2-(4-methoxyphenyl)-2-(phenylthio)acetate (9j)[24]

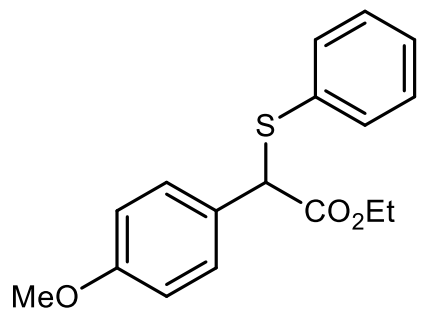

9j

The residue was purified by column chromatography on silica gel with an eluent of petroleum ether/EA (20:1, R_f = 0.23) to afford substrate **9j** (1.88 g, 62% yield) as a colorless oil.

^{1}H NMR (400 MHz, $CDCl_3$) δ 7.41 – 7.35 (m, 4H), 7.28 – 7.22 (m, 3H), 6.87 – 6.81 (m, 2H), 4.88 (s, 1H), 4.19 – 4.01 (m, 2H), 3.76 (s, 3H), 1.14 (t, J = 7.2 Hz, 3H).

^{13}C NMR (101 MHz, $CDCl_3$) δ 170.7, 159.7, 134.1, 132.7, 129.8, 129.0, 128.0, 127.7, 114.2, 61.7, 55.7, 55.4, 14.1.

LRMS (EI) m/z: $[M]^+$ Calcd for $C_{17}H_{18}O_3S$ 302.1; Found 302.1.

Ethyl 2-(4-cyanophenyl)-2-(phenylthio)acetate (9k)

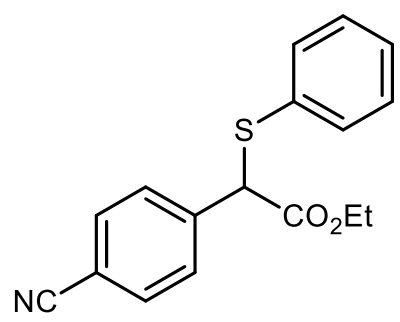

9k

The residue was purified by column chromatography on silica gel with an eluent of petroleum ether/EA (20:1, R_f = 0.29) to afford substrate **9k** (1.18 g, 40% yield) as a colorless oil.

^{1}H NMR (400 MHz, $CDCl_3$) δ 7.57 (d, J = 8.2 Hz, 2H), 7.52 (d, J = 8.2 Hz, 2H), 7.39 – 7.29 (m, 2H), 7.29 – 7.22 (m, 3H), 4.91 (s, 1H), 4.20 – 4.07 (m, 2H), 1.17 (t, J = 7.2 Hz, 3H).

^{13}C NMR (101 MHz, $CDCl_3$) δ 169.3, 141.1, 133.3, 132.4, 132.2, 129.4, 129.1, 128.6, 118.4, 112.0, 62.1, 55.9, 13.9.

HRMS (FI) m/z: $[M]^+$ Calcd for $C_{17}H_{15}NO_2S$ 297.0818; Found 297.0822.

IR (KBr) 3059, 2982, 2937, 2905, 2229, 1732, 1606, 1582, 1502, 1474, 1439, 1415, 1392, 1368, 1333, 1300, 1277, 1214, 1149, 1115, 1095, 1068, 1023, 961, 839, 744, 692, 556, 495, 419 cm^{-1}

Ethyl 2-(4-nitrophenyl)-2-(phenylthio)acetate (9l)[24]

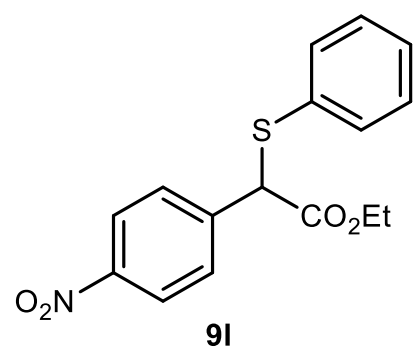

9l

The residue was purified by column chromatography on silica gel with an eluent of petroleum ether/EA (10:1, R_f = 0.40) to afford substrate **9l** (1.01 g, 32% yield) as a yellow oil.

^{1}H NMR (400 MHz, $CDCl_3$) δ 8.18 – 8.12 (m, 2H), 7.62 – 7.56 (m, 2H), 7.38 – 7.33 (m, 2H), 7.33 – 7.23 (m, 3H), 4.94 (s, 1H), 4.24 – 4.10 (m, 2H), 1.20 (t, J = 7.2 Hz, 3H).

^{13}C NMR (101 MHz, $CDCl_3$) δ 169.4, 147.7, 143.4, 133.7, 132.4, 129.7, 129.3, 128.9, 123.8, 62.4, 56.0, 14.1.

LRMS (EI) m/z: $[M]^+$ Calcd for $C_{16}H_{15}NO_4S$ 317.1; Found 317.1.

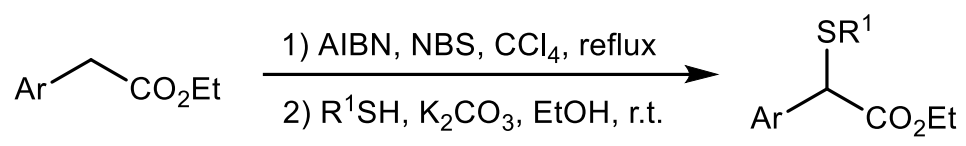

A two-neck 100 mL round-bottom flask equipped with a condenser was charged with AIBN (82.1 mg, 0.5 mmol, 5 mol%), NBS (1.96 g, 11 mmol, 1.1 equiv) and CCl_4 (25 mL) at room temperature. Ethyl arylacetate (10 mmol, 1.0 equiv) was added to this solution and the reaction was allowed to stir at reflux temperature overnight. Then the solution was cooled to room temperature, and the solvent was removed under vacuum. EtOH (25 mL) was added to dissolve the residue. Thiol (10 mmol, 1.0 equiv) and K_2CO_3 (4.14 g, 30 mmol, 3 equiv.) were added. The mixture was stirred at room temperature for 12 h. The solvent was removed under vacuum, and water (50 mL) was

added to the residue. The obtained aqueous phase was extracted with DCM (3 × 50 mL), and the combined organic layers were dried over Na_2SO_4. The solvent was removed under vacuum and the residue was subjected to flash column chromatography to give substrate **9b-9i**, **9m-9p**.

Ethyl 2-((4-(*tert*-butyl)phenyl)thio)-2-phenylacetate (9b)[25]

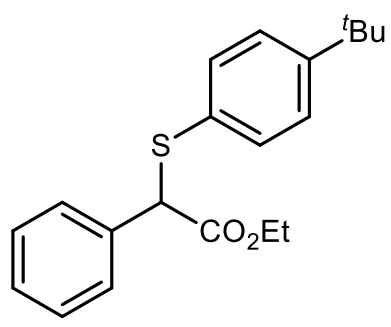

9b

The residue was purified by column chromatography on silica gel with an eluent of petroleum ether/EA (20:1, R_f = 0.54) to afford substrate **9b** (1.59 g, 48% yield) as a colorless oil. **^{1}H NMR** (400 MHz, $CDCl_3$) δ 7.51 – 7.46 (m, 2H), 7.37 – 7.27 (m, 7H), 4.88 (s, 1H), 4.21 – 4.04 (m, 2H), 1.30 (s, 9H), 1.15 (t, J = 7.1 Hz, 3H).

^{13}C NMR (101 MHz, $CDCl_3$) δ 170.8, 151.6, 136.0, 132.9, 130.5, 128.8, 128.7, 128.4, 126.2, 61.8, 56.7, 34.8, 31.4, 14.1.

LRMS (EI) m/z: $[M]^+$ Calcd for $C_{20}H_{24}O_2S$ 328.1; Found 328.2.

Ethyl 2-((4-methoxyphenyl)thio)-2-phenylacetate (9c)[25]

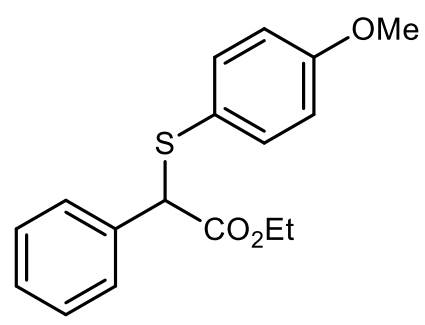

9c

The residue was purified by column chromatography on silica gel with an eluent of petroleum ether/EA (20:1, R_f = 0.29) to afford substrate **9c** (1.67 g, 55% yield) as a colorless oil.

^{1}H NMR (400 MHz, $CDCl_3$) δ 7.43 – 7.37 (m, 2H), 7.35 – 7.30 (m, 2H), 7.30 – 7.21 (m, 3H), 6.79 – 6.74 (m, 2H), 4.76 (s, 1H), 4.16 – 4.01 (m, 2H), 3.70 (s, 3H), 1.13 (t, J = 7.2 Hz, 3H).

^{13}C NMR (101 MHz, $CDCl_3$) δ 170.5, 160.2, 136.1, 135.9, 128.6, 128.5, 128.1, 123.7, 114.4, 61.5, 57.4, 55.2, 14.0.

LRMS (EI) m/z: $[M]^+$ Calcd for $C_{17}H_{18}O_3S$ 302.1; Found 302.1.

Ethyl 2-((4-(methylthio)phenyl)thio)-2-phenylacetate (9d)

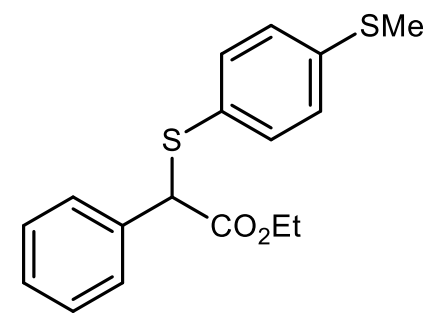

9d

The residue was purified by column chromatography on silica gel with an eluent of petroleum ether/EA (40:1, R_f = 0.41) to afford substrate **9d** (1.41 g, 44% yield) as a colorless oil.

^{1}H NMR (400 MHz, $CDCl_3$) δ 7.48 – 7.42 (m, 2H), 7.35 – 7.27 (m, 5H), 7.15 – 7.09 (m, 2H), 4.86 (s, 1H), 4.21 – 4.04 (m, 2H), 2.42 (s, 3H), 1.17 (t, J = 7.1 Hz, 3H).

^{13}C NMR (101 MHz, $CDCl_3$) δ 170.3, 139.4, 135.7, 133.8, 129.5, 128.6, 128.6, 128.3, 126.5, 61.7, 56.7, 15.4, 14.0.

HRMS (FI) m/z: $[M]^+$ Calcd for $C_{17}H_{18}O_2S_2$ 318.0743; Found 318.0750.

IR (KBr) 3063, 3029, 2980, 2921, 1897, 1731, 1601, 1576, 1497, 1475, 1455, 1435, 1388, 1368, 1339, 1306, 1279, 1211, 1144, 1105, 1076, 1025, 968, 957, 916, 874, 813, 747, 727, 696, 576, 502, 422 cm^{-1}

Ethyl 2-((4-chlorophenyl)thio)-2-phenylacetate (9e)[25]

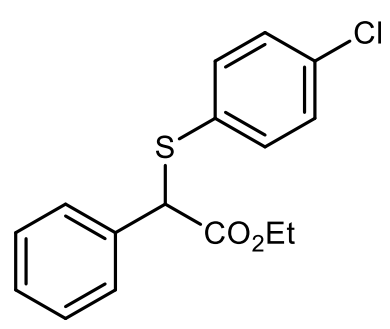

9e

The residue was purified by column chromatography on silica gel with an eluent of petroleum ether/EA (40:1, R_f = 0.45) to afford substrate **9e** (1.35 g, 44% yield) as a colorless oil.

^{1}H NMR (400 MHz, $CDCl_3$) δ 7.45 – 7.39 (m, 2H), 7.31 – 7.23 (m, 5H), 7.21 – 7.15 (m, 2H), 4.88 (s, 1H), 4.17 – 4.02 (m, 2H), 1.12 (t, J = 7.1 Hz, 3H).

^{13}C NMR (101 MHz, $CDCl_3$) δ 170.0, 135.4, 134.2, 134.1, 132.2, 129.1, 128.7, 128.5, 128.4, 61.8, 56.4, 14.0.

LRMS (EI) m/z: $[M]^+$ Calcd for $C_{16}H_{15}ClO_2S$ 306.0; Found 306.1.

Ethyl 2-((4-bromophenyl)thio)-2-phenylacetate (9f)[26]

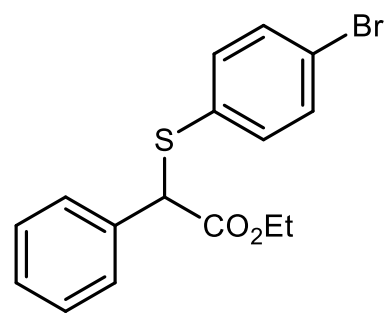

9f

The residue was purified by column chromatography on silica gel with an eluent of petroleum ether/EA (40:1, R_f = 0.36) to afford substrate **9f** (1.63 g, 47% yield) as a colorless oil.

^{1}H NMR (400 MHz, $CDCl_3$) δ 7.46 – 7.41 (m, 2H), 7.41 – 7.36 (m, 2H), 7.36 – 7.27 (m, 3H), 7.25 – 7.20 (m, 2H), 4.88 (s, 1H), 4.23 – 4.05 (m, 2H), 1.18 (t, J = 7.1 Hz, 3H).

^{13}C NMR (101 MHz, $CDCl_3$) δ 170.2, 135.5, 134.4, 133.0, 132.2, 128.9, 128.7, 128.6, 122.5, 62.0, 56.4, 14.2.

LRMS (EI) m/z: $[M]^+$ Calcd for $C_{16}H_{15}BrO_2S$ 350.0; Found 350.1.

Ethyl 2-((4-cyanophenyl)thio)-2-phenylacetate (9g)

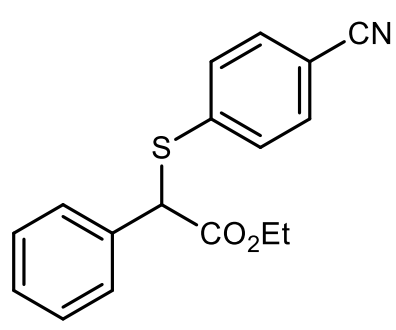

9g

The residue was purified by column chromatography on silica gel with an eluent of petroleum ether/EA (10:1, R_f = 0.34) to afford substrate **9g** (1.13 g, 38% yield) as a white solid, m.p. 59-61 ºC.

^{1}H NMR (400 MHz, $CDCl_3$) δ 7.53 – 7.45 (m, 4H), 7.39 – 7.29 (m, 5H), 5.06 (s, 1H), 4.25 – 4.09 (m, 2H), 1.19 (t, J = 7.1 Hz, 3H).

^{13}C NMR (101 MHz, $CDCl_3$) δ 169.7, 142.0, 134.6, 132.5, 129.8, 129.1, 128.9, 128.6, 118.6, 110.3, 62.4, 54.8, 14.1.

HRMS (FI) m/z: $[M]^+$ Calcd for $C_{17}H_{15}NO_2S$ 297.0818; Found 297.0821.

IR (KBr) 3063, 3030, 2981, 2936, 2226, 1738, 1592, 1507, 1488, 1455, 1397, 1367, 1339, 1302, 1271, 1212, 1146, 1087, 1018, 957, 918, 876, 820, 782, 726, 696, 595, 543, 499, 419 cm^{-1}

Ethyl 2-phenyl-2-((4-(trifluoromethyl)phenyl)thio)acetate (9h)

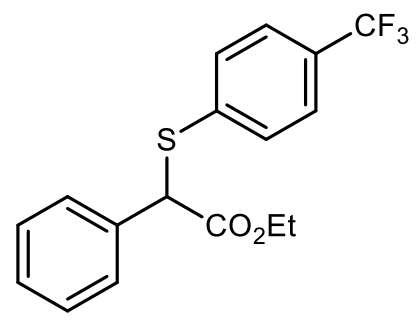

9h

The residue was purified by column chromatography on silica gel with an eluent of petroleum ether/EA (40:1, R_f = 0.32) to afford substrate **9h** (1.39 g, 41% yield) as a colorless oil.

^{1}H NMR (400 MHz, $CDCl_3$) δ 7.54 – 7.47 (m, 4H), 7.43 (d, *J* = 8.2 Hz, 2H), 7.39 – 7.30 (m, 3H), 5.03 (s, 1H), 4.24 – 4.09 (m, 2H), 1.19 (t, *J* = 7.1 Hz, 3H).

^{19}F NMR (376 MHz, $CDCl_3$) δ -62.66 (s, 3F).

^{13}C NMR (101 MHz, $CDCl_3$) δ 170.1, 139.6 (q, *J* = 1.3 Hz), 135.1, 130.9, 129.4 (q, *J* = 32.7 Hz), 129.0, 128.8, 128.6, 125.9 (q, *J* = 3.8 Hz), 124.1 (q, *J* = 272.0 Hz), 62.2, 55.5, 14.1.

HRMS (FI) m/z: $[M]^+$ Calcd for $C_{17}H_{15}F_3O_2S$ 340.0739; Found 340.0737.

IR (KBr) 3065, 3032, 2983, 2938, 1738, 1606, 1572, 1496, 1455, 1400, 1368, 1327, 1281, 1213, 1168, 1127, 1095, 1063, 1030, 1015, 951, 872, 836, 779, 726, 696, 594, 498 cm^{-1}

Ethyl 2-((4-nitrophenyl)thio)-2-phenylacetate (9i)[27]

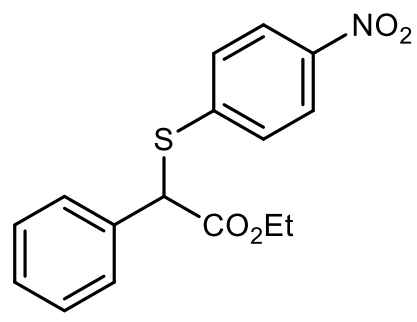

9i

The residue was purified by column chromatography on silica gel with an eluent of petroleum ether/EA (20:1, R_f = 0.33) to afford substrate **9i** (1.04 g, 33% yield) as a white solid.

^{1}H NMR (400 MHz, $CDCl_3$) δ 8.14 – 8.07 (m, 2H), 7.53 – 7.47 (m, 2H), 7.43 – 7.31 (m, 5H), 5.12 (s, 1H), 4.27 – 4.11 (m, 2H), 1.21 (t, J = 7.1 Hz, 3H).
^{13}C NMR (101 MHz, $CDCl_3$) δ 169.6, 146.3, 144.7, 134.5, 129.2, 129.0, 128.6, 124.1, 62.5, 54.7, 14.2.
LRMS (EI) m/z: $[M]^+$ Calcd for $C_{16}H_{15}NO_4S$ 317.1; Found 317.1.

Ethyl 2-((4-methoxyphenyl)thio)-2-(4-nitrophenyl)acetate (9m)

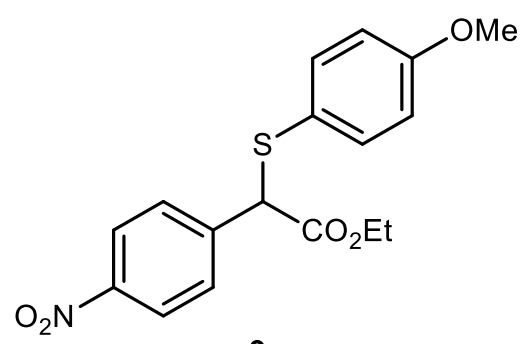

9m

The residue was purified by column chromatography on silica gel with an eluent of petroleum ether/EA (20:1, R_f = 0.25) to afford substrate **9m** (855.1 mg, 25% yield) as a yellow oil.
^{1}H NMR (400 MHz, $CDCl_3$) δ 8.16 – 8.11 (m, 2H), 7.56 – 7.51 (m, 2H), 7.30 – 7.25 (m, 2H), 6.82 – 6.76 (m, 2H), 4.79 (s, 1H), 4.22 – 4.12 (m, 2H), 3.78 (s, 3H), 1.22 (t, J = 7.2 Hz, 3H).
^{13}C NMR (101 MHz, $CDCl_3$) δ 169.5, 160.8, 147.6, 143.6, 136.8, 129.8, 123.7, 122.4, 114.8, 62.2, 56.7, 55.5, 14.1.
HRMS (FI) m/z: $[M]^+$ Calcd for $C_{17}H_{17}NO_5S$ 347.0822; Found 347.0828.
IR (KBr) 3109, 3078, 2981, 2940, 2839, 1732, 1592, 1569, 1520, 1494, 1463, 1418, 1406, 1393, 1347, 1287, 1248, 1173, 1147, 1108, 1027, 858, 831, 799, 732, 699, 646, 626, 526, 501 cm^{-1}

Ethyl 2-phenyl-2-(thiophen-2-ylthio)acetate (9n)[25]

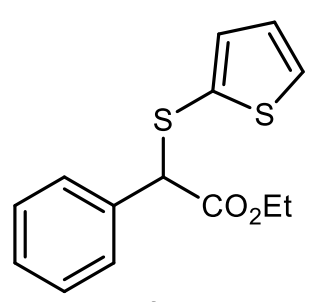

9n

The residue was purified by column chromatography on silica gel with an eluent of petroleum ether/EA (50:1, R_f = 0.60) to afford substrate **9n** (1.44 g, 52% yield) as a

yellow oil.

^{1}H NMR (400 MHz, $CDCl_3$) δ 7.45 – 7.39 (m, 2H), 7.40 – 7.34 (m, 1H), 7.35 – 7.28 (m, 3H), 7.10 – 7.04 (m, 1H), 6.98 – 6.91 (m, 1H), 4.80 (s, 1H), 4.45 – 3.79 (m, 2H), 1.22 (t, *J* = 7.1 Hz, 3H).

^{13}C NMR (101 MHz, $CDCl_3$) δ 170.1, 136.3, 135.4, 131.3, 131.2, 128.72, 128.69, 128.5, 127.6, 61.9, 59.2, 14.1.

LRMS (EI) m/z: $[M]^+$ Calcd for $C_{14}H_{14}O_2S_2$ 278.0; Found 278.1.

Ethyl 2-(ethylthio)-2-phenylacetate (9o)[28]

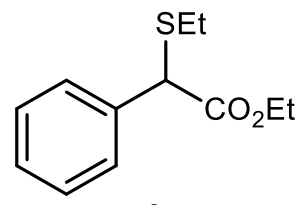

9o

The residue was purified by column chromatography on silica gel with an eluent of petroleum ether/EA (40:1, R_f = 0.50) to afford substrate **9o** (1.70 g, 76% yield) as a colorless oil.

^{1}H NMR (400 MHz, $CDCl_3$) δ 7.50 – 7.45 (m, 2H), 7.37 – 7.28 (m, 3H), 4.59 (s, 1H), 4.27 – 4.12 (m, 2H), 2.64 – 2.45 (m, 2H), 1.30 – 1.19 (m, 6H).

^{13}C NMR (101 MHz, $CDCl_3$) δ 171.1, 136.5, 128.8, 128.6, 128.2, 61.8, 52.2, 26.1, 14.28, 14.26.

LRMS (EI) m/z: $[M]^+$ Calcd for $C_{12}H_{16}O_2S$ 224.1; Found 224.1.

Ethyl 2-(benzylthio)-2-phenylacetate (9p)

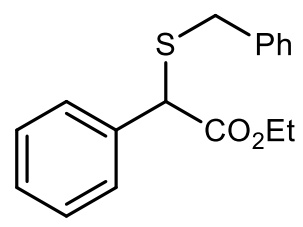

9p

The residue was purified by column chromatography on silica gel with an eluent of petroleum ether/EA (20:1, R_f = 0.28) to afford substrate **9p** (1.29 g, 45% yield) as a colorless oil.

^{1}H NMR (400 MHz, $CDCl_3$) δ 7.44 – 7.38 (m, 2H), 7.35 – 7.21 (m, 8H), 4.40 (s, 1H), 4.22 – 4.07 (m, 2H), 3.76 (d, *J* = 13.4 Hz, 1H), 3.61 (d, *J* = 13.4 Hz, 1H), 1.22 (t, *J* = 7.1 Hz, 3H).

^{13}C NMR (101 MHz, $CDCl_3$) δ 170.8, 137.3, 136.1, 129.2, 128.8, 128.73, 128.66, 128.3, 127.4, 61.8, 51.8, 36.3, 14.2.

HRMS (FI) m/z: $[M]^+$ Calcd for $C_{17}H_{18}O_2S$ 286.1022; Found 286.1025.

IR (KBr) 3085, 3062, 3029, 2981, 2935, 1801, 1732, 1601, 1584, 1495, 1454, 1417, 1393, 1367, 1338, 1306, 1280, 1238, 1212, 1146, 1095, 1072, 1027, 953, 916, 875, 766, 731, 696, 564, 500, 472 cm^{-1}

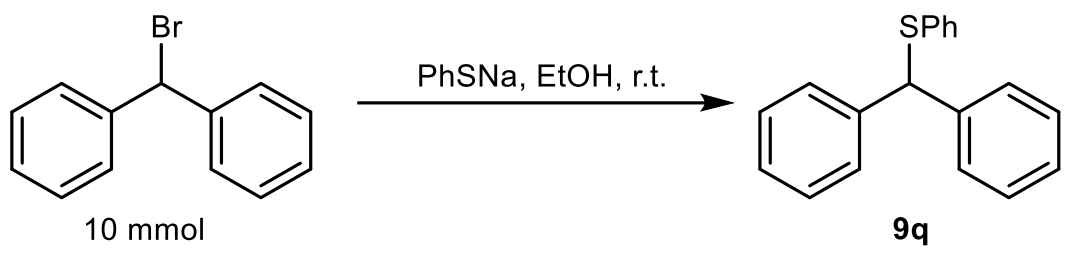

A 100 mL round-bottom flask was charged with (bromomethylene)dibenzene (10 mmol, 1.0 equiv) and EtOH (20 mL) at room temperature. Sodium thiophenolate (1.45 g, 11 mmol, 1.1 equiv) was added to this solution and the reaction was allowed to stir at room temperature for 12 h. Then the solvent was removed under vacuum, and water (50 mL) was added to the residue. The obtained aqueous phase was extracted with DCM (3 × 50 mL), and the combined organic layers were dried over Na_2SO_4. The solvent was removed under vacuum, and the residue was was purified by column chromatography on silica gel with an eluent of petroleum ether/EA (50:1, R_f = 0.30) to afford substrate **9q** (1.55 g, 56% yield) as a white solid.

Benzhydryl(phenyl)sulfane (9q)[29]

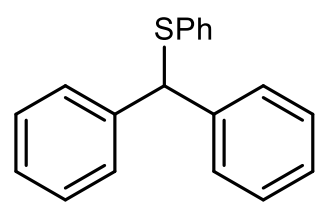

9q

^{1}H NMR (400 MHz, $CDCl_3$) δ 7.44 – 7.38 (m, 4H), 7.33 – 7.24 (m, 4H), 7.26 – 7.17 (m, 4H), 7.18 – 7.09 (m, 3H), 5.54 (s, 1H).

^{13}C NMR (101 MHz, $CDCl_3$) δ 141.2, 136.3, 130.7, 128.9, 128.7, 128.6, 127.4, 126.8, 57.6.

LRMS (EI) m/z: $[M]^+$ Calcd for $C_{19}H_{16}S$ 276.1; Found 276.1.

The synthesis of 2-vinyl pyridines 11d, **11f-11i**, **11l-11n**, **11p-11s**

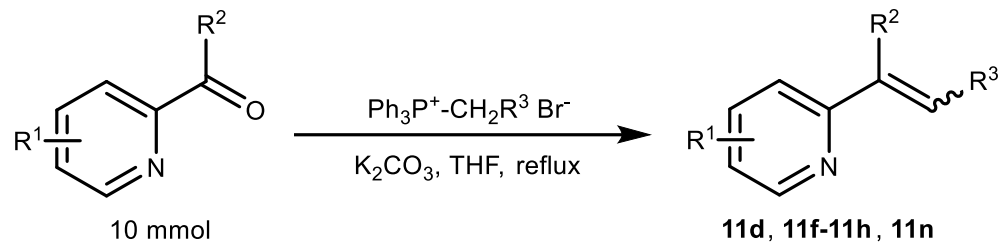

A two-neck 250 mL round-bottom flask equipped with a condenser was charged with alkyltriphenylphosphonium bromide (15 mmol, 1.5 equiv), K_2CO_3 (2.76 g, 20 mmol, 2.0 equiv) and dry THF (30 mL) at room temperature under a N_2 atmosphere. Pyridine-2-carbaldehyde (10 mmol, 1.0 equiv) was added to this solution and the reaction was allowed to stir at reflux temperature for 16 h. Then the solution was cooled to room temperature and water (100 mL) was added. The obtained mixture was extracted with DCM (3 × 100 mL), and the combined organic layers were dried over Na_2SO_4. The solvent was removed under vacuum and the residue was subjected to flash column chromatography to give substrate **11d**, **11f-11h**, **11n**.

5-Phenyl-2-(prop-1-en-1-yl)pyridine (11d)

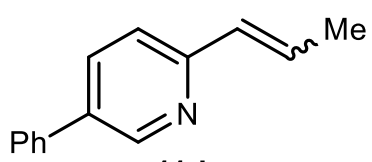

11d

The residue was purified by column chromatography on silica gel with an eluent of petroleum ether/EA (10:1) to afford substrates **(*Z*)-11d** (370.5 mg, 19% yield, $R_f = 0.34$) as a colorless oil and **(*E*)-11d** (819.3 mg, 42% yield, $R_f = 0.32$) as a white solid (m.p. 42-44 °C).

For **(*Z*)-11d**:

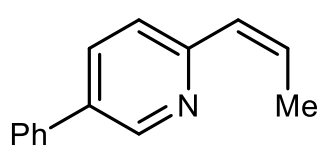

(*Z*)-11d

^{1}H NMR (400 MHz, $CDCl_3$) δ 8.87 (d, J = 2.4 Hz, 1H), 7.84 (dd, J = 8.1, 2.4 Hz, 1H), 7.64 – 7.56 (m, 2H), 7.52 – 7.43 (m, 2H), 7.43 – 7.36 (m, 1H), 7.33 (d, J = 8.1 Hz, 1H), 6.54 (dq, J = 11.8, 1.9 Hz, 1H), 6.05 (dq, J = 11.8, 7.3 Hz, 1H), 2.16 (dd, J = 7.3, 1.9 Hz, 3H).

^{13}C NMR (101 MHz, $CDCl_3$) δ 155.8, 147.7, 137.9, 134.4, 133.9, 131.6, 129.3, 129.2, 128.0, 127.1, 123.9, 15.3.

HRMS (ESI) m/z: $[M+H]^+$ Calcd for $C_{14}H_{14}N$ 196.1121; Found 196.1123.

IR (KBr) 3060, 3024, 2910, 2857, 1642, 1593, 1545, 1475, 1448, 1420, 1364, 1331, 1308, 1264, 1221, 1205, 1188, 1141, 1076, 1029, 1004, 994, 933, 911, 851, 789, 755, 726, 696, 641, 562, 511 cm^{-1}

For **(*E*)-11d**:

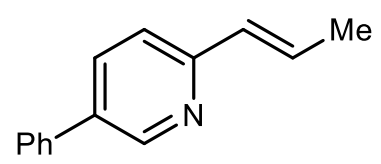

(*E*)-11d

^{1}H NMR (400 MHz, $CDCl_3$) δ 8.77 (d, *J* = 2.4 Hz, 1H), 7.80 (dd, *J* = 8.2, 2.4 Hz, 1H), 7.63 – 7.54 (m, 2H), 7.51 – 7.42 (m, 2H), 7.42 – 7.34 (m, 1H), 7.30 (d, *J* = 8.2 Hz, 1H), 6.79 (dq, *J* = 15.4, 6.7 Hz, 1H), 6.56 (dq, *J* = 15.6, 1.7 Hz, 1H), 1.96 (dd, *J* = 6.7, 1.7 Hz, 3H).

^{13}C NMR (101 MHz, $CDCl_3$) δ 155.1, 147.9, 138.0, 134.9, 134.5, 131.1, 131.0, 129.2, 128.0, 127.0, 120.8, 18.7.

HRMS (ESI) m/z: $[M+H]^+$ Calcd for $C_{14}H_{14}N$ 196.1121; Found 196.1124.

IR (KBr) 3058, 3027, 2957, 2930, 2909, 2874, 2854, 1654, 1591, 1580, 1553, 1474, 1448, 1370, 1333, 1301, 1276, 1203, 1142, 1077, 1046, 1029, 1003, 994, 965, 911, 853, 758, 695, 645, 557, 504, 459, 406 cm^{-1}

2-(1-Phenylvinyl)pyridine (11f)[30]

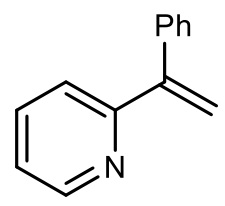

11f

The residue was purified by column chromatography on silica gel with an eluent of petroleum ether/EA (10:1, R_f = 0.38) to afford substrate **11f** (1.35 g, 75% yield) as a brown solid.

^{1}H NMR (400 MHz, $CDCl_3$) δ 8.66 – 8.59 (m, 1H), 7.61 – 7.52 (m, 1H), 7.38 – 7.26 (m, 5H), 7.23 (d, *J* = 7.9 Hz, 1H), 7.19 – 7.11 (m, 1H), 5.99 (s, 1H), 5.59 (s, 1H).

^{13}C NMR (101 MHz, $CDCl_3$) δ 158.5, 149.4, 149.2, 140.4, 136.3, 128.4, 128.3, 127.9, 122.8, 122.4, 117.7.

LRMS (EI) m/z: $[M]^+$ Calcd for $C_{13}H_{11}N$ 181.1; Found 181.1.

4-Methoxy-2-vinylpyridine (11g)[31]

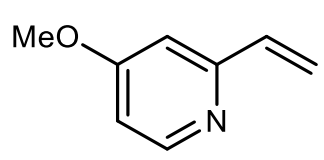

11g

The residue was purified by column chromatography on silica gel with an eluent of petroleum ether/EA (5:1, R_f = 0.20) to afford substrate **11g** (809.7 mg, 60% yield) as a yellow oil.

^{1}H NMR (400 MHz, $CDCl_3$) δ 8.32 (d, J = 5.7 Hz, 1H), 6.76 (s, 1H), 6.69 (dd, J = 17.4, 10.9 Hz, 1H), 6.64 – 6.55 (m, 1H), 6.12 (d, J = 17.4 Hz, 1H), 5.39 (d, J = 10.8 Hz, 1H), 3.75 (s, 3H).

^{13}C NMR (101 MHz, $CDCl_3$) δ 166.2, 157.3, 150.7, 137.0, 118.3, 108.6, 107.4, 55.1.

LRMS (EI) m/z: $[M]^+$ Calcd for C_8H_9NO 135.1; Found 135.1.

5-Methoxy-2-vinylpyridine (11h)

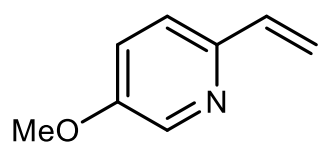

11h

The residue was purified by column chromatography on silica gel with an eluent of petroleum ether/EA (5:1, R_f = 0.39) to afford substrates **11h** (781.4 mg, 58% yield) as a yellow oil.

^{1}H NMR (400 MHz, $CDCl_3$) δ 8.23 (d, J = 3.0 Hz, 1H), 7.24 (d, J = 8.6 Hz, 1H), 7.10 (dd, J = 8.8, 2.8 Hz, 1H), 6.73 (dd, J = 17.5, 10.9 Hz, 1H), 5.99 (dd, J = 17.5, 1.3 Hz, 1H), 5.31 (dd, J = 10.9, 1.3 Hz, 1H), 3.80 (s, 3H).

^{13}C NMR (101 MHz, $CDCl_3$) δ 155.0, 148.7, 137.2, 136.4, 121.6, 120.8, 115.8, 55.7.

HRMS (FI) m/z: $[M]^+$ Calcd for C_8H_9NO 135.0679; Found 135.0677.

IR (KBr) 3099, 3062, 3011, 2967, 2939, 2839, 1629, 1591, 1567, 1492, 1479, 1443, 1411, 1388, 1298, 1270, 1226, 1209, 1181, 1138, 1124, 1036, 1024, 990, 916, 836, 778, 721, 636, 576, 534, 419 cm^{-1}

Methyl 2-vinylisonicotinate (11n)

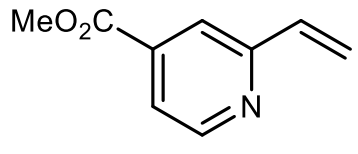

11n

The residue was purified by column chromatography on silica gel with an eluent of

petroleum ether/EA (10:1, R_f = 0.21) to afford substrate **11n** (315.7 mg, 19% yield) as a colorless oil.

^{1}H NMR (400 MHz, $CDCl_3$) δ 8.59 (d, J = 5.0 Hz, 1H), 7.76 (s, 1H), 7.57 (dd, J = 4.9, 1.6 Hz, 1H), 6.76 (dd, J = 17.5, 10.8 Hz, 1H), 6.18 (dd, J = 17.4, 1.2 Hz, 1H), 5.44 (dd, J = 10.7, 1.2 Hz, 1H), 3.84 (s, 3H).

^{13}C NMR (101 MHz, $CDCl_3$) δ 165.5, 156.7, 150.2, 137.8, 136.3, 121.4, 120.3, 119.4, 52.6.

HRMS (FI) m/z: $[M]^+$ Calcd for $C_9H_9NO_2$ 163.0628; Found 163.0626.

IR (KBr) 3059, 3020, 2953, 2848, 1732, 1596, 1557, 1473, 1439, 1419, 1370, 1292, 1264, 1236, 1205, 1113, 984, 861, 793, 776, 758, 686, 419 cm^{-1}

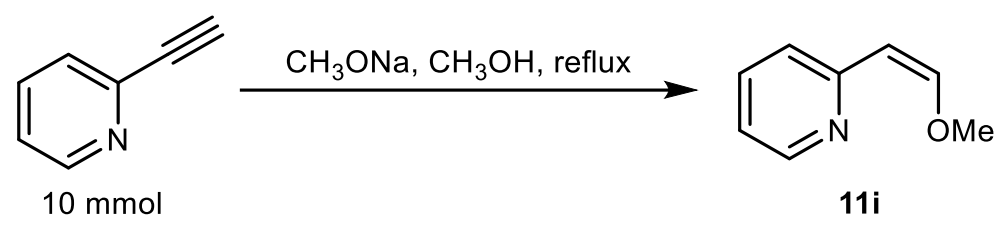

A two-neck 100 mL round-bottom flask equipped with a condenser was charged with a solution of CH_3ONa in CH_3OH (50 mL, 1.0 M, 5.0 equiv) at room temperature. 2-Ethynylpyridine (1.03 g, 10 mmol, 1.0 equiv) was added to this solution and the reaction was allowed to stir at reflux temperature for 12 h. Then the solution was cooled to room temperature, and solvent was removed under vacuum. Then saturated NH_4Cl solution (30 mL) was added to the residue. The obtained aqueous phase was extracted with DCM (3 × 50 mL), and the combined organic layers were dried over Na_2SO_4. The solvent was removed under vacuum and the residue was subjected to flash column chromatography to give substrate **11i**.

(*Z*)-2-(2-methoxyvinyl)pyridine (11i)[32]

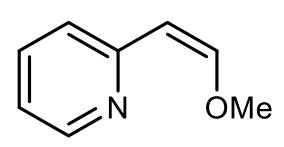

11i

The residue was purified by column chromatography on silica gel with an eluent of petroleum ether/EA (2:1, R_f = 0.24) to afford substrate **11i** (532.2 mg, 39% yield) as a yellow oil.

^{1}H NMR (400 MHz, $CDCl_3$) δ 8.49 (d, J = 5.0 Hz, 1H), 7.87 (d, J = 8.1 Hz, 1H), 7.63 – 7.54 (m, 1H), 7.04 – 6.96 (m, 1H), 6.33 (d, J = 7.2 Hz, 1H), 5.50 (d, J = 7.2 Hz, 1H), 3.82 (s, 3H).

^{13}C NMR (101 MHz, $CDCl_3$) δ 155.2, 151.1, 149.1, 136.2, 123.8, 120.6, 107.0, 61.3.

LRMS (EI) m/z: $[M]^+$ Calcd for C_8H_9NO 135.1; Found 135.1.

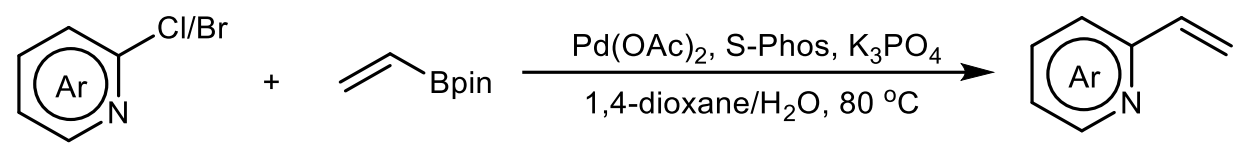

A 100 mL round-bottom flask was charged with chloro/bromoheterocycle (10 mmol, 1.0 equiv), vinylboronic acid pinacol ester (1.12 mL, 11 mmol, 1.1 equiv), $Pd(OAc)_2$ (89.8 mg, 0.4 mmol, 4 mol%), S-Phos (328.4 mg, 0.8 mmol, 8 mol%) and K_3PO_4 (6.37 g, 30 mmol, 3.0 equiv) under a N_2 atmosphere. 1,4-Dioxane (40 mL) and water (0.9 mL, 50 mmol, 5.0 equiv) were added and the reaction was stirred at 80 °C for 4 h. The mixture was then cooled to rt, filtered through Celite®, and concentrated in vacuo. The residue was subjected to flash column chromatography to give substrate **11l-11m**, **11q-11r** (For the synthesis of substrate **11l**, **11m** and **11q**, bromoheterocycles were used as starting materials. For the synthesis of substrate **11r**, chloroheterocycle was used as starting material).

4-Nitro-2-vinylpyridine (11l)

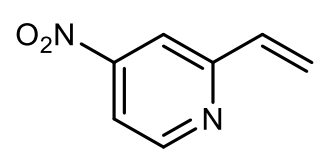

11l

The residue was purified by column chromatography on silica gel with an eluent of petroleum ether/EA (15:1, R_f = 0.38) to afford substrate **11l** (1.02 g, 68% yield) as a brown solid, m.p. 47-49 ºC.

^{1}H NMR (400 MHz, $CDCl_3$) δ 8.81 (d, J = 5.3 Hz, 1H), 7.98 (d, J = 2.1 Hz, 1H), 7.82 (dd, J = 5.3, 2.1 Hz, 1H), 6.87 (dd, J = 17.4, 10.8 Hz, 1H), 6.34 (dd, J = 17.4, 0.9 Hz, 1H), 5.64 (d, J = 10.8 Hz, 1H).

^{13}C NMR (101 MHz, $CDCl_3$) δ 158.8, 154.5, 151.7, 135.4, 121.6, 114.7, 113.5.

HRMS (FI) m/z: $[M]^+$ Calcd for $C_7H_6N_2O_2$ 150.0424; Found 150.0422.

IR (KBr) 3087, 2925, 2873, 1634, 1605, 1565, 1538, 1464, 1419, 1397, 1353, 1295, 1241, 1211, 1097, 1036, 988, 939, 895, 846, 804, 769, 736, 687, 418 cm^{-1}

2-Vinylisonicotinonitrile (11m)[33]

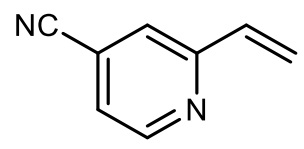

11m

The residue was purified by column chromatography on silica gel with an eluent of petroleum ether/EA (10:1, R_f = 0.40) to afford substrate **11m** (924.3 mg, 71% yield) as a brown solid.

^{1}H NMR (400 MHz, $CDCl_3$) δ 8.72 (d, *J* = 5.0, 1H), 7.52 (s, 1H), 7.36 (dd, *J* = 5.0, 1.5 Hz, 1H), 6.80 (dd, *J* = 17.4, 10.8 Hz, 1H), 6.30 (dd, *J* = 17.4, 1.0 Hz, 1H), 5.62 (dd, *J* = 10.8, 1.0 Hz, 1H).

^{13}C NMR (101 MHz, $CDCl_3$) δ 157.1, 150.6, 135.4, 123.7, 122.9, 121.3, 121.1, 116.7.

LRMS (EI) m/z: $[M]^+$ Calcd for $C_8H_6N_2$ 130.1; Found 130.1.

2-Vinylbenzo[*d*]thiazole (11q)[34]

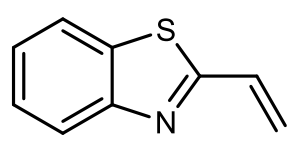

11q

The residue was purified by column chromatography on silica gel with an eluent of petroleum ether/EA (5:1, R_f = 0.59) to afford substrate **11q** (1.12 g, 70% yield) as a yellow oil.

^{1}H NMR (400 MHz, $CDCl_3$) δ 8.00 (d, *J* = 8.0 Hz, 1H), 7.87 – 7.77 (m, 1H), 7.45 (ddd, *J* = 8.2, 7.2, 1.3 Hz, 1H), 7.36 (ddd, *J* = 8.2, 7.2, 1.2 Hz, 1H), 7.04 (dd, *J* = 17.5, 10.9 Hz, 1H), 6.17 (d, *J* = 17.5 Hz, 1H), 5.75 (d, *J* = 10.8 Hz, 1H).

^{13}C NMR (101 MHz, $CDCl_3$) δ 167.4, 153.8, 134.4, 131.6, 126.4, 125.7, 123.4, 123.3, 121.7.

LRMS (EI) m/z: $[M]^+$ Calcd for C_9H_7NS 161.0; Found 161.1.

4-Phenyl-2-vinylpyrimidine (11r)[35]

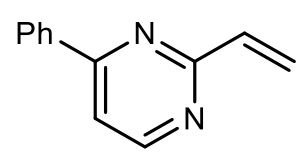

11r

The residue was purified by column chromatography on silica gel with an eluent of petroleum ether/EA (10:1, R_f = 0.22) to afford substrate **11r** (1.23 g, 68% yield) as a yellow oil.

^{1}H NMR (400 MHz, $CDCl_3$) δ 8.63 (d, J = 5.3 Hz, 1H), 8.11 – 8.02 (m, 2H), 7.49 – 7.40 (m, 3H), 7.41 (d, J = 5.3 Hz, 1H), 6.96 (dd, J = 17.3, 10.5 Hz, 1H), 6.74 (dd, J = 17.3, 1.9 Hz, 1H), 5.74 (dd, J = 10.5, 1.9 Hz, 1H).

^{13}C NMR (101 MHz, $CDCl_3$) δ 164.3, 163.7, 157.5, 136.9, 136.7, 130.9, 128.9, 127.1, 123.7, 114.6.

LRMS (EI) m/z: $[M]^+$ Calcd for $C_{12}H_{10}N_2$ 182.1; Found 182.1.

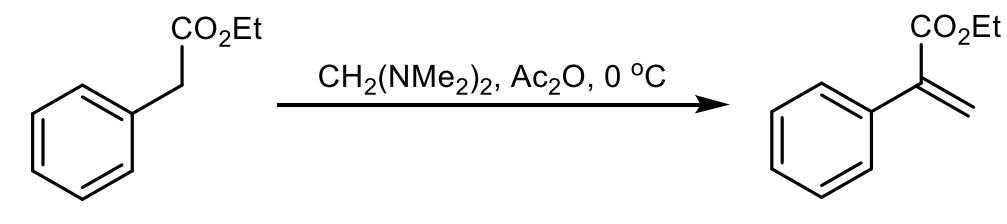

A 25 mL round-bottom flask was charged with ethyl phenylacetate (1.6 mL, 10 mmol, 1.0 equiv) and bisdimethylaminomethane (5.46 mL, 40 mmol, 4.0 equiv) at 0 °C. Acetic anhydride (4.73 mL, 50 mmol, 5.0 equiv) was added slowly and the reaction was vigorously stirred at 0 °C for 10 min. Ethyl ether and water were added. The organic phase was separated. The obtained aqueous phase was extracted with DCM (3 × 50 mL), and the combined organic layers were dried over Na_2SO_4. The solvent was removed under vacuum and the residue was subjected to flash column chromatography to give substrate **11p**.

Ethyl 2-(pyridin-2-yl)acrylate (11p)[36]

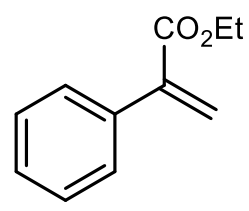

11p

The residue was purified by column chromatography on silica gel with an eluent of petroleum ether/EA (5:1, R_f = 0.30) to afford substrate **11p** (471.3 mg, 27% yield) as a yellow oil.

^{1}H NMR (400 MHz, $CDCl_3$) δ 8.57 – 8.53 (m, 1H), 7.62 (td, J = 7.7, 1.8 Hz, 1H), 7.56 (dt, J = 7.9, 1.2 Hz, 1H), 7.16 (ddd, J = 7.4, 4.8, 1.4 Hz, 1H), 6.41 (d, J = 1.5 Hz, 1H), 6.40 (d, J = 1.6 Hz, 1H), 4.26 (q, J = 7.1 Hz, 2H), 1.28 (t, J = 7.1 Hz, 3H).
^{13}C NMR (101 MHz, $CDCl_3$) δ 166.5, 154.0, 149.3, 140.7, 136.3, 128.8, 123.1, 123.0, 61.1, 14.2.
LRMS (EI) m/z: $[M]^+$ Calcd for $C_{10}H_{11}NO_2$ 177.1; Found 177.1.

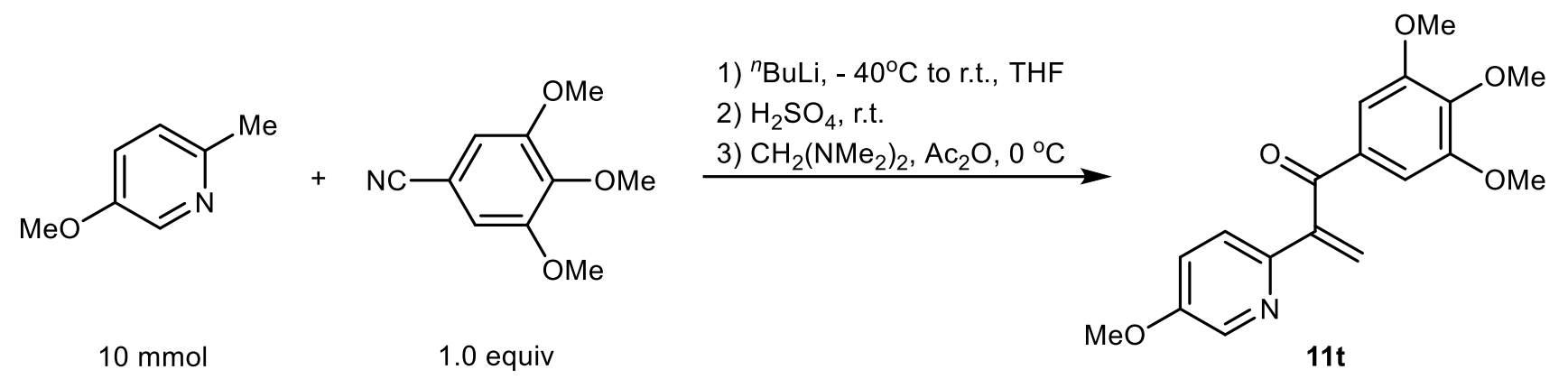

A two-neck 100 mL round-bottom flask was charged with 5-methoxy-2-picoline (1.22 mL, 10 mmol, 1.0 equiv) and dry THF (10 mL) under N_2 atmosphere. After the solution was cooled to -10°C, a solution of *n*-butyl lithium (12.5 mL, 20 mmol, 1.6M in *n*-hexane, 2.0 equiv) was slowly added under efficient stirring. The external cooling bath was removed and the mixture was warmed to room temperature and stirred for 10 min. The mixture was again cooled to -40°C, and then 3,4,5-trimethoxybenzonitrile (1.93 g, 10 mmol, 1.0 equiv) was added in one portion under N_2 atmosphere. The mixture was stirred for 2 days at room temperature.

The mixture was acidified with sulfuric acid (60% aqueous solution) at 0 °C until a pH of 1 was reached. After the resulting biphasic mixture was stirred for 1 day, saturated aqueous KOH solution was added to neutralize the solution. During this procedure large amounts of potassium sulfate precipitated. Water was added to dissolve this precipitate. The mixture was extracted with DCM (3 × 100 mL), and the combined organic layers were dried over Na_2SO_4. The solvent was removed under vacuum, resulted in a dark brown slurry.

A 25 mL round-bottom flask was charged with the obtained slurry and bisdimethylaminomethane (5.46 mL, 40 mmol, 4.0 equiv) at 0 °C. Acetic anhydride (4.73 mL, 50 mmol, 5.0 equiv) was added slowly and the reaction was vigorously stirred at 0 °C for 10 min. Ethyl ether and water were added. The organic phase was

separated. The obtained aqueous phase was extracted with DCM (3 × 50 mL), and the combined organic layers were dried over Na_2SO_4. The solvent was removed under vacuum and the residue was subjected to flash column chromatography to give substrate **11t**.

2-(5-Methoxypyridin-2-yl)-1-(3,4,5-trimethoxyphenyl)prop-2-en-1-one (11t)

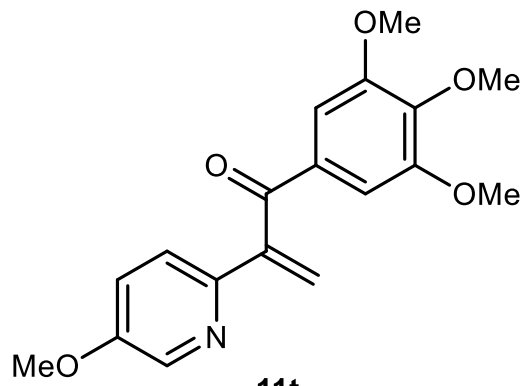

11t

The residue was purified by column chromatography on silica gel with an eluent of petroleum ether/EA (1:1, R_f = 0.30) to afford substrate **11t** (1.84 g, 56% yield) as a brown solid, m.p. 38-40 ºC.

^{1}H NMR (400 MHz, $CDCl_3$) δ 8.26 (d, J = 3.0 Hz, 1H), 7.38 (d, J = 8.7 Hz, 1H), 7.18 (s, 2H), 7.13 (dd, J = 8.7, 3.0 Hz, 1H), 6.40 (s, 1H), 5.64 (s, 1H), 3.87 (s, 3H), 3.82 (s, 3H), 3.81 (s, 6H).

^{13}C NMR (101 MHz, $CDCl_3$) δ 196.3, 155.4, 153.0, 147.2, 147.0, 142.8, 137.6, 132.2, 122.2, 120.6, 107.5, 61.0, 56.3, 55.7.

HRMS (DART) m/z: $[M+H]^+$ Calcd for $C_{18}H_{20}NO_5$ 330.1336; Found 330.1336.

IR (KBr) 3073, 3002, 2940, 2837, 1665, 1583, 1501, 1491, 1480, 1461, 1414, 1336, 1315, 1289, 1269, 1231, 1166, 1126, 1052, 1027, 1004, 923, 887, 867, 840, 783, 727, 672, 635, 568, 525, 420 cm^{-1}

6 Procedures for the synthesis of products

The procedure for [2+1] cycloaddition of alkenes with :C(SCF_3)$_2$ to obtain products 2a-2s, 2u-2v, 2v', 12c'

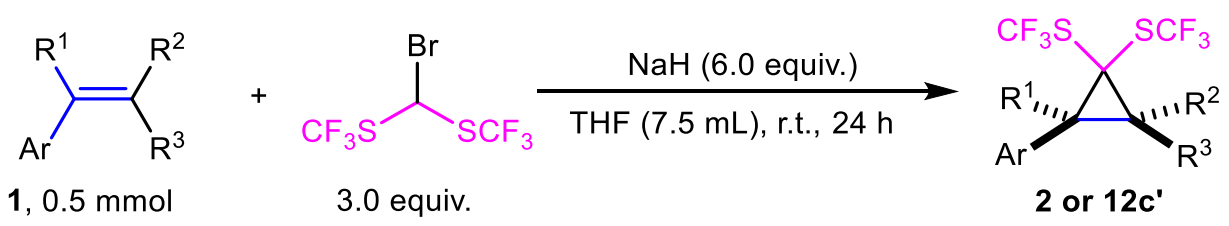

A 25 mL bottom flask was charged with alkenes **1** (0.5 mmol, 1.0 equiv.), NaH (72.0 mg, 3.0 mmol, 6.0 equiv.) and dry THF (7.5 mL) under a N_2 atmosphere, BrCH(SCF_3)$_2$

(442.5 mg, 1.5 mmol, 3.0 equiv.) was then added. The resulting mixture was stirred at room temperature for 24 h under a N_2 atmosphere. The resulting mixture was then diluted with ethyl acetate (20 mL) and saturated NH_4Cl solution (30 mL). The organic phase was separated and the aqueous phase was extracted 3 times. All organic solutions were combined and then dried over anhydrous Na_2SO_4. The solvent was removed under vacuum and the residue was subjected to column chromatography to give final products **2a-2s, 2u-2v, 2v', 12c'**.

(2-([1,1'-Biphenyl]-4-yl)cyclopropane-1,1-diyl)bis((trifluoromethyl)sulfane) (2a)

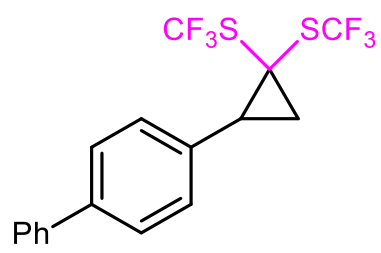

2a

The residue was purified by column chromatography on silica gel with an eluent of petroleum ether (R_f = 0.60) to afford product **2a** (185.2 mg, 94% yield) as a brown oil.

^{1}H NMR (400 MHz, $CDCl_3$) δ 7.70 – 7.63 (m, 4H), 7.53 (t, *J* = 7.6 Hz, 2H), 7.45 (d, *J* = 7.0 Hz, 1H), 7.41 (d, *J* = 8.1 Hz, 2H), 3.38 (t, *J* = 8.7 Hz, 1H), 2.32 (t, *J* = 7.3 Hz, 1H), 2.18 (dd, *J* = 9.4, 6.6 Hz, 1H).

^{19}F NMR (376 MHz, $CDCl_3$) δ -40.02 (s, 3F), -40.80 (s, 3F).

^{13}C NMR (101 MHz, $CDCl_3$) δ 141.3, 140.5, 132.8, 130.64 (q, *J* = 311.0 Hz), 130.60 (q, *J* = 310.8 Hz), 129.6, 129.1, 127.8, 127.5, 127.3, 37.2, 32.1 (sept, *J* = 2.3 Hz), 25.0.

HRMS (FI) m/z: $[M]^+$ Calcd for $C_{17}H_{12}F_6S_2$ 394.0279; Found 394.0283.

IR (KBr) 3059, 3033, 2927, 1602, 1522, 1489, 1438, 1405, 1164, 1126, 1101, 1064, 1008, 916, 841, 779, 758, 728, 697, 596, 579, 531, 504 cm^{-1}

(2-(4-(*tert*-Butyl)phenyl)cyclopropane-1,1-diyl)bis((trifluoromethyl)sulfane) (2b)

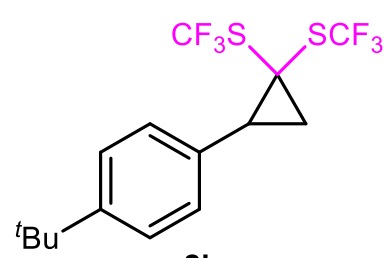

2b

The residue was purified by column chromatography on silica gel with an eluent of petroleum ether (R_f = 0.77) to afford product **2b** (149.6 mg, 80% yield) as a yellow oil.

^{1}H NMR (400 MHz, $CDCl_3$) δ 7.36 (d, *J* = 8.4 Hz, 2H), 7.18 (d, *J* = 8.3 Hz, 2H), 3.22 (t, *J* = 8.7 Hz, 1H), 2.17 (t, *J* = 7.3 Hz, 1H), 2.05 (dd, *J* = 9.5, 6.5 Hz, 1H), 1.31 (s, 9H).

^{19}F NMR (376 MHz, $CDCl_3$) δ -40.17 (s, 3F), -40.97 (s, 3F).

^{13}C NMR (101 MHz, $CDCl_3$) δ 151.6, 130.9, 130.70 (q, J = 310.4 Hz), 130.66 (q, J = 310.7 Hz), 128.9, 125.8, 37.2, 34.8, 31.5, 32.0 (sept, J = 2.0 Hz), 25.1.

HRMS (FI) m/z: $[M]^+$ Calcd for $C_{15}H_{16}F_6S_2$ 374.0592; Found 374.0589.

IR (KBr) 3035, 2966, 2907, 2871, 1516, 1478, 1467, 1439, 1395, 1365, 1269, 1164, 1129, 1101, 1063, 1018, 918, 835, 756, 584, 527 cm^{-1}

(2-(4-Methoxyphenyl)cyclopropane-1,1-diyl)bis((trifluoromethyl)sulfane) (2c)

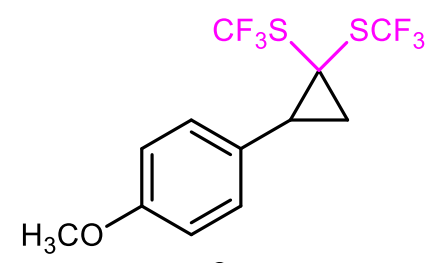

2c

The residue was purified by column chromatography on silica gel with an eluent of petroleum ether (R_f = 0.60) to afford product **2c** (151.5 mg, 87% yield) as a yellow oil.

^{1}H NMR (400 MHz, $CDCl_3$) δ 7.19 (d, J = 8.7 Hz, 2H), 6.90 (d, J = 8.8 Hz, 2H), 3.83 (s, 3H), 3.24 (t, J = 8.7 Hz, 1H), 2.17 (t, J = 7.3 Hz, 1H), 2.08 (dd, J = 9.5, 6.7 Hz, 1H).

^{19}F NMR (376 MHz, $CDCl_3$) δ -40.15 (s, 3F), -40.97 (s, 3F).

^{13}C NMR (101 MHz, $CDCl_3$) δ 159.7, 130.6 (q, J = 310.6 Hz), 130.3, 125.9, 114.2, 55.4, 36.9, 32.0 (sept, J = 2.0 Hz), 25.1.

HRMS (FI) m/z: $[M]^+$ Calcd for $C_{12}H_{10}OF_6S_2$ 348.0072; Found 348.0077.

IR (KBr) 3008, 2961, 2936, 2840, 1612, 1583, 1517, 1465, 1440, 1364, 1305, 1256, 1162, 1124, 1102, 1064, 1036, 917, 834, 806, 782, 756, 729, 693, 578, 505 cm^{-1}

4-(2,2-Bis((trifluoromethyl)thio)cyclopropyl)-*N*,*N*-dimethylaniline (2d)

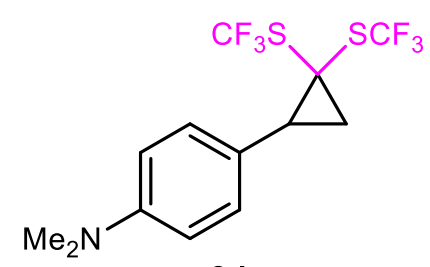

2d

The residue was purified by column chromatography on silica gel with an eluent of petroleum ether (R_f = 0.31) to afford product **2d** (164.3 mg, 91% yield) as a brown oil.

^{1}H NMR (400 MHz, $CDCl_3$) δ 7.17 (d, J = 7.8 Hz, 2H), 6.74 (d, J = 7.8 Hz, 2H), 3.25 (t, J = 8.8 Hz, 1H), 3.01 (s, 6H), 2.19 (t, J = 7.3 Hz, 1H), 2.12 – 2.01 (m, 1H).

^{19}F NMR (376 MHz, $CDCl_3$) δ -40.10 (s, 3F), -40.96 (s, 3F).

^{13}C NMR (101 MHz, $CDCl_3$) δ 150.4, 130.8 (q, J = 310.2 Hz), 130.7 (q, J = 310.6 Hz), 129.9, 121.2, 112.3, 40.5, 37.1. 32.1 (sept, J = 1.8 Hz), 25.0.

HRMS (FI) m/z: $[M]^+$ Calcd for $C_{13}H_{13}NF_6S_2$ 361.0388; Found 361.0392.

IR (KBr) 2891, 2866, 2807, 1615, 1526, 1482, 1438, 1359, 1225, 1202, 1159, 1100, 1064, 948, 918, 819, 776, 756, 676, 576, 554, 505 cm^{-1}

(2-(2-Fluorophenyl)cyclopropane-1,1-diyl)bis((trifluoromethyl)sulfane) (2e)

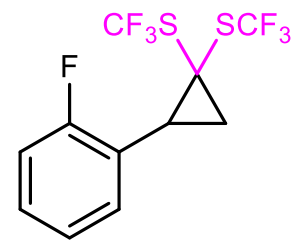

2e

The residue was purified by column chromatography on silica gel with an eluent of petroleum ether (R_f = 0.74) to afford product **2e** (95.8 mg, 57% yield) as a yellow oil.

^{1}H NMR (400 MHz, $CDCl_3$) δ 7.40 – 7.30 (m, 1H), 7.20 – 7.07 (m, 3H), 3.29 (t, J = 8.7 Hz, 1H), 2.24 (t, J = 7.3 Hz, 1H), 2.16 (dd, J = 9.4, 6.7 Hz, 1H).

^{19}F NMR (376 MHz, $CDCl_3$) δ -40.21 (s, 3F), -40.90 (d, J = 10.8 Hz, 3F), -115.10 – -115.30 (m, 1F).

^{13}C NMR (101 MHz, $CDCl_3$) δ 162.6 (d, J = 248.6 Hz), 130.37 (q, J = 310.5 Hz), 130.35 (d, J = 3.2 Hz), 130.32 (q, J = 310.7 Hz), 130.27 (d, J = 8.4 Hz), 124.3 (d, J = 3.7 Hz), 121.6 (d, J = 14.3 Hz), 115.9 (d, J = 21.4 Hz), 31.7 (d, J = 3.6 Hz), 30.0, 24.7.

HRMS (FI) m/z: $[M]^+$ Calcd for $C_{11}H_7F_7S_2$ 335.9872; Found 335.9876.

IR (KBr) 2928, 2857, 1586, 1498, 1457, 1372, 1250, 1211, 1166, 1135, 1100, 1065, 1034, 920, 824, 757, 707, 514 cm^{-1}

(2-(4-Chlorophenyl)cyclopropane-1,1-diyl)bis((trifluoromethyl)sulfane) (2f)

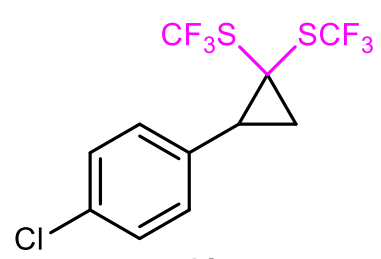

2f

The residue was purified by column chromatography on silica gel with an eluent of petroleum ether (R_f = 0.46) to afford product **2f** (128.7 mg, 73% yield) as a yellow oil.

^{1}H NMR (400 MHz, $CDCl_3$) δ 7.34 (d, J = 8.3 Hz, 2H), 7.20 (d, J = 8.4 Hz, 2H), 3.24 (t, J = 8.6 Hz, 1H), 2.18 (t, J = 7.4 Hz, 1H), 2.12 (dd, J = 9.5, 6.7 Hz, 1H).

^{19}F NMR (376 MHz, $CDCl_3$) δ -40.15 (s, 3F), -40.94 (s, 3F).

^{13}C NMR (101 MHz, $CDCl_3$) δ 134.5, 132.4, 130.53 (q, J = 310.6 Hz), 130.48, 130.4 (q, J = 310.6 Hz), 129.1, 36.8, 32.0 (sept, J = 2.1 Hz), 25.0.

HRMS (FI) m/z: $[M]^+$ Calcd for $C_{11}H_7ClF_6S_2$ 351.9576; Found 351.9578.

IR (KBr) 1599, 1496, 1439, 1403, 136 26, 1101, 1062, 1016, 917, 831, 782, 756, 713, 642, 577, 545, 502 cm^{-1}

(2-(4-Bromophenyl)cyclopropane-1,1-diyl)bis((trifluoromethyl)sulfane) (2g)

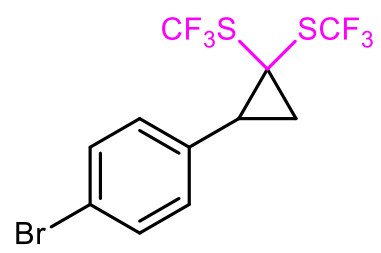

2g

The residue was purified by column chromatography on silica gel with an eluent of petroleum ether (R_f = 0.49) to afford product **2g** (158.4 mg, 80% yield) as a yellow oil.

^{1}H NMR (400 MHz, $CDCl_3$) δ 7.50 (d, J = 8.4 Hz, 2H), 7.14 (d, J = 8.2 Hz, 2H), 3.22 (t, J = 8.7 Hz, 1H), 2.18 (t, J = 7.4 Hz, 1H), 2.12 (dd, J = 9.4, 6.7 Hz, 1H).

^{19}F NMR (376 MHz, $CDCl_3$) δ -40.12 (s, 3F), -40.92 (s, 3F).

^{13}C NMR (101 MHz, $CDCl_3$) δ 132.7, 131.8, 130.6, 130.3 (q, J = 310.5 Hz), 130.2 (q, J = 310.4 Hz), 122.4, 36.7, 31.7 (sept, J = 2.0 Hz), 24.7.

HRMS (FI) m/z: $[M]^+$ Calcd for $C_{11}H_7BrF_6S_2$ 395.9071; Found 395.9075.

IR (KBr) 1593, 1492, 1438, 1398, 1364, 1300, 1164, 1129, 1100, 1077, 1062, 1012, 951, 916, 828, 780, 756, 708, 617, 576, 543, 505, 487 cm^{-1}

(2-(4-Iodophenyl)cyclopropane-1,1-diyl)bis((trifluoromethyl)sulfane) (2h)

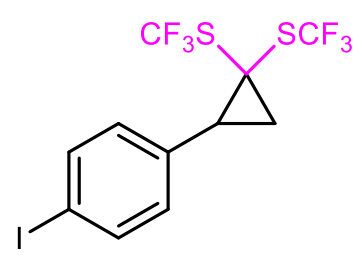

2h

The residue was purified by column chromatography on silica gel with an eluent of petroleum ether (R_f = 0.71) to afford product **2h** (199.8 mg, 90% yield) as a brown oil.

^{1}H NMR (400 MHz, $CDCl_3$) δ 7.70 (d, J = 7.5 Hz, 2H), 7.00 (d, J = 7.6 Hz, 2H), 3.20 (t, J = 8.7 Hz, 1H), 2.18 (t, J = 7.5 Hz, 1H), 2.15 – 2.06 (m, 1H).

^{19}F NMR (376 MHz, $CDCl_3$) δ -40.09 (s, 3F), -40.88 (s, 3F).

^{13}C NMR (101 MHz, $CDCl_3$) δ 137.9, 133.5, 131.0, 130.5 (q, J = 310.7 Hz), 130.4 (q, J = 310.6 Hz), 94.2, 37.0, 31.9 (sept, J = 2.1 Hz), 24.8.

HRMS (FI) m/z: $[M]^+$ Calcd for $C_{11}H_7F_6IS_2$ 443.8933; Found 443.8932.

IR (KBr) 1589, 1488, 1439, 1395, 1365, 1164, 1126, 1100, 1066, 1056, 1008, 952, 915, 824, 778, 756, 706, 608, 576, 543, 505, 484 cm^{-1}

Methyl 4-(2,2-bis((trifluoromethyl)thio)cyclopropyl)benzoate (2i)

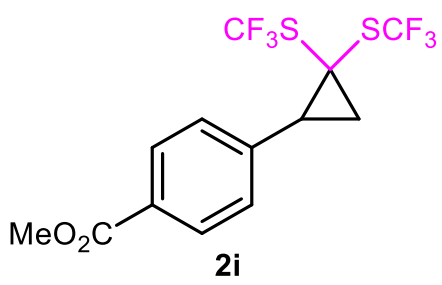

2i

The residue was purified by column chromatography on silica gel with an eluent of petroleum ether/EA (20:1, R_f = 0.36) to afford product **2i** (114.7 mg, 61% yield) as a yellow oil.

^{1}H NMR (400 MHz, $CDCl_3$) δ 8.03 (d, J = 8.1 Hz, 2H), 7.32 (d, J = 8.0 Hz, 2H), 3.91 (s, 3H), 3.29 (t, J = 8.7 Hz, 1H), 2.24 (t, J = 7.4 Hz, 1H), 2.14 (dd, J = 9.3, 6.7 Hz, 1H).

^{19}F NMR (376 MHz, $CDCl_3$) δ -40.20 (s, 3F), -40.92 (s, 3F).

^{13}C NMR (101 MHz, $CDCl_3$) δ 166.7, 138.8, 130.5 (q, J = 310.7 Hz), 130.28 (q, J = 310.4 Hz), 130.26, 130.0, 129.2, 52.3, 37.2, 32.1 (sept, J = 2.2 Hz), 24.9.

HRMS (FI) m/z: $[M]^+$ Calcd for $C_{13}H_{10}O_2F_6S_2$ 376.0021; Found 376.0025.

IR (KBr) 3003, 2956, 2848, 1727, 1613, 1575, 1513, 1437, 1410, 1362, 1312, 1282, 1245, 1164, 1126, 1101, 1063, 1020, 968, 917, 863, 828, 790, 756, 747, 701, 578, 546, 504 cm^{-1}

(2-(4-Nitrophenyl)cyclopropane-1,1-diyl)bis((trifluoromethyl)sulfane) (2j)

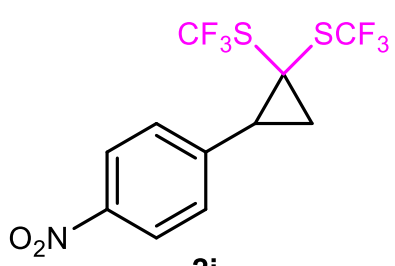

2j

The residue was purified by column chromatography on silica gel with an eluent of petroleum ether to petroleum ether/EA (50:1, R_f = 0.23) to afford product **2j** (130.7 mg, 72% yield) as a yellow oil.

^{1}H NMR (400 MHz, $CDCl_3$) δ 8.22 (d, J = 8.8 Hz, 2H), 7.43 (d, J = 8.8 Hz, 2H), 3.33 (t, J = 8.6 Hz, 1H), 2.33 – 2.19 (m, 2H).

^{19}F NMR (376 MHz, $CDCl_3$) δ -40.09 (s, 3F), -40.85 (s, 3F).

^{13}C NMR (101 MHz, $CDCl_3$) δ 147.9, 141.0, 130.4 (q, J = 310.7 Hz), 130.13, 130.06 (q, J = 310.8 Hz), 124.0, 36.9, 32.3 (sept, J = 2.4 Hz), 25.1.

HRMS (FI) m/z: $[M]^+$ Calcd for $C_{11}H_7O_2NF_6S_2$ 362.9817; Found 362.9811.

IR (KBr) 3116, 3086, 2929, 2857, 1606, 1525, 1497, 1441, 1350, 1298, 1166, 1129, 1100, 1064, 1016, 953, 917, 856, 786, 757, 740, 694, 625, 576, 544, 504 cm^{-1}

(1,1a,6,6a-tetrahydrocyclopropa[*a*]indene-1,1-diyl)bis((trifluoromethyl)sulfane) (2k)

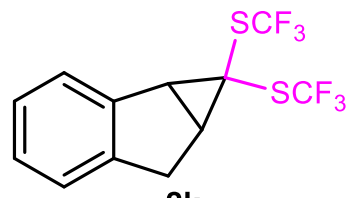

2k

The residue was purified by column chromatography on silica gel with an eluent of petroleum ether (R_f = 0.77) to afford product **2k** (90.8 mg, 55% yield) as a brown oil.

^{1}H NMR (400 MHz, $CDCl_3$) δ 7.35 (d, J = 7.4 Hz, 1H), 7.28 – 7.21 (m, 1H), 7.21 – 7.11 (m, 2H), 3.46 (d, J = 6.7 Hz, 1H), 3.33 (dd, J = 18.3, 6.9 Hz, 1H), 3.05 (d, J = 18.3 Hz, 1H), 2.78 (t, J = 6.8 Hz, 1H).

^{19}F NMR (376 MHz, $CDCl_3$) δ -38.96 (s, 3F), -39.82 (s, 3F).

^{13}C NMR (101 MHz, $CDCl_3$) δ 144.6, 138.5, 130.4 (q, J = 311.3 Hz), 130.3 (q, J = 310.9 Hz), 128.6, 126.8, 125.8, 124.5, 44.1, 37.3 (sept, J = 1.9 Hz), 35.9, 33.8.

HRMS (FI) m/z: $[M]^+$ Calcd for $C_{12}H_8F_6S_2$ 329.9966; Found 329.9963.

IR (KBr) 3076,3055, 3031, 2920, 2848, 1481, 1464, 1431, 1310, 1162, 1135, 1108, 1015, 969, 855, 838, 813, 787, 756, 719, 507, 489, 438 cm^{-1}

(2-Methyl-2-phenylcyclopropane-1,1-diyl)bis((trifluoromethyl)sulfane) (2l)

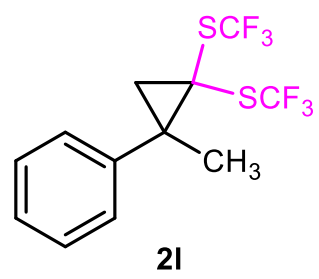

2l

The residue was purified by column chromatography on silica gel with an eluent of petroleum ether (R_f = 0.79) to afford product **2l** (93.0 mg, 56% yield) as a brown oil.

^{1}H NMR (400 MHz, $CDCl_3$) δ 7.38 – 7.27 (m, 3H), 7.23 (d, J = 7.1 Hz, 2H), 2.24 (d, J = 6.5 Hz, 1H), 1.72 (s, 3H), 1.64 (d, J = 6.5 Hz, 1H).

^{19}F NMR (376 MHz, $CDCl_3$) δ -40.01 (s, 3F), -40.18 (s, 3F).

^{13}C NMR (101 MHz, $CDCl_3$) δ 141.1, 130.4 (q, *J* = 310.8 Hz), 130.1 (q, *J* = 310.8 Hz), 129.2, 128.2, 128.1, 39.7, 37.1 (sept, *J* = 2.0 Hz), 31.0, 25.6 (q, *J* = 1.7 Hz).

HRMS (FI) m/z: $[M]^+$ Calcd for $C_{12}H_{10}F_6S_2$ 332.0123; Found 332.0116.

IR (KBr) 3086, 3064, 3030, 2993, 2934, 2879, 1602, 1583, 1498, 1447, 1434, 1382, 1321, 1255, 1162, 1123, 1073, 1063, 1028, 871, 786, 757, 700, 604, 540, 502 cm^{-1}

(*E*)-(2-Styrylcyclopropane-1,1-diyl)bis((trifluoromethyl)sulfane) (2m)

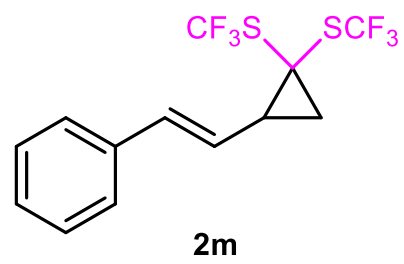

2m

The residue was purified by column chromatography on silica gel with an eluent of petroleum ether (R_f = 0.63) to afford product **2m** (99.8 mg, 58% yield) as a yellow oil.

^{1}H NMR (400 MHz, $CDCl_3$) δ 7.39 – 7.30 (m, 4H), 7.29 – 7.24 (m, 1H), 6.70 (d, *J* = 15.8 Hz, 1H), 5.97 (dd, *J* = 15.8, 8.5 Hz, 1H), 2.72 (q, *J* = 8.4 Hz, 1H), 2.06 (dd, *J* = 9.1, 6.5 Hz, 1H), 1.83 (t, *J* = 7.0 Hz, 1H).

^{19}F NMR (376 MHz, $CDCl_3$) δ -40.07 (s, 3F), -40.63 (s, 3F).

^{13}C NMR (101 MHz, $CDCl_3$) δ 136.4, 135.6, 130.5 (q, *J* = 311.0 Hz), 130.4 (q, *J* = 310.8 Hz), 128.9, 128.3, 126.6, 124.5, 35.8, 31.9 (sept, *J* = 2.0 Hz), 27.2.

HRMS (FI) m/z: $[M]^+$ Calcd for $C_{13}H_{10}F_6S_2$ 344.0123; Found 344.0118.

IR (KBr) 3030, 2928, 2857, 1495, 1437, 1166, 1130, 1094, 1032, 962, 938, 914, 852, 821, 776, 756, 743, 692, 488 cm^{-1}

(2-(4-Allylphenyl)cyclopropane-1,1-diyl)bis((trifluoromethyl)sulfane) (2n)

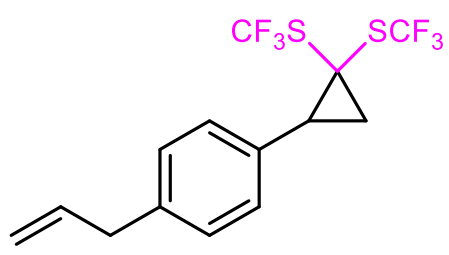

2n

The residue was purified by column chromatography on silica gel with an eluent of petroleum ether (R_f = 0.66) to afford product **2n** (155.7 mg, 87% yield) as a yellow oil.

^{1}H NMR (400 MHz, $CDCl_3$) δ 7.20 (s, 4H), 5.98 (ddt, *J* = 19.0, 9.5, 6.7 Hz, 1H), 5.15 – 5.06 (m, 2H), 3.41 (d, *J* = 6.7 Hz, 2H), 3.26 (t, *J* = 8.7 Hz, 1H), 2.20 (t, *J* = 7.3 Hz, 1H), 2.08 (dd, *J* = 9.5, 6.6 Hz, 1H).

^{19}F NMR (376 MHz, $CDCl_3$) δ -40.18 (s, 3F), -40.96 (s, 3F).

^{13}C NMR (101 MHz, $CDCl_3$) δ 140.5, 137.2, 131.7, 130.6 (q, J = 310.3 Hz), 129.2, 129.0, 116.4, 40.1, 37.2, 31.9 (sept, J = 2.2 Hz), 25.0.

HRMS (FI) m/z: $[M]^+$ Calcd for $C_{14}H_{12}F_6S_2$ 358.0279; Found 358.0286.

IR (KBr) 3086, 3031, 2982, 2907, 1640, 1516, 1437, 1416, 1162, 1126, 1101, 1063, 1021, 993, 917, 842, 819, 778, 756, 578, 553, 504 cm^{-1}

Trimethyl(1-phenyl-2,2-bis((trifluoromethyl)thio)cyclopropoxy)silane (2o)

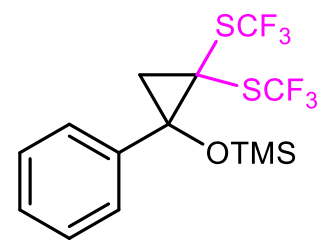

2o

The residue was purified by column chromatography on silica gel with an eluent of petroleum ether (R_f = 0.75) to afford product **2o** (113.7 mg, 56% yield) as a brown oil.

^{1}H NMR (400 MHz, $CDCl_3$) δ 7.43 – 7.34 (m, 5H), 2.52 (d, J = 7.7 Hz, 1H), 2.01 (d, J = 7.7 Hz, 1H), 0.00 (s, 9H).

^{19}F NMR (376 MHz, $CDCl_3$) δ -39.40 (s, 3F), -40.31 (s, 3F).

^{13}C NMR (101 MHz, $CDCl_3$) δ 137.9, 130.4 (q, J = 311.2 Hz), 130.3 (q, J = 310.7 Hz), 129.2, 128.7, 128.4, 69.5, 37.9 (sept, J = 1.8 Hz), 31.6, 0.7.

HRMS (FI) m/z: $[M]^+$ Calcd for $C_{14}H_{16}OF_6S_2Si$ 406.0311; Found 406.0317.

IR (KBr) 3066, 3035, 2962, 2905, 1496, 1450, 1418, 1314, 1295, 1254, 1166, 1121, 1069, 1029, 990, 921, 907, 852, 756, 698, 674, 610, 529, 505, 449 cm^{-1}

2-(2,2-Bis((trifluoromethyl)thio)cyclopropyl)isoindoline-1,3-dione (2p)

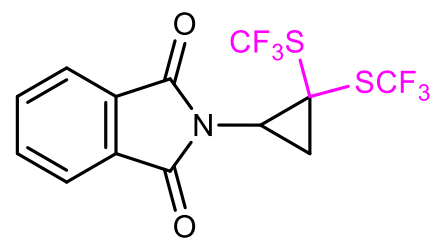

2p

The residue was purified by column chromatography on silica gel with an eluent of petroleum ether/EA (20:1, R_f = 0.23) to afford product **2p** (114.2 mg, 59% yield) as a brown solid, m.p. 96-98 °C.

^{1}H NMR (400 MHz, $CDCl_3$) δ 7.92 – 7.83 (m, 2H), 7.82 – 7.73 (m, 2H), 3.64 (dd, J = 8.5, 6.2 Hz, 1H), 2.88 (t, J = 7.1 Hz, 1H), 2.29 (t, J = 8.3 Hz, 1H).

^{19}F NMR (376 MHz, $CDCl_3$) δ -40.26 (s, 3F), -40.38 (s, 3F).

^{13}C NMR (101 MHz, $CDCl_3$) δ 167.8, 134.9, 131.4, 130.0 (q, J = 310.8 Hz), 129.9 (q, J = 311.1 Hz), 124.0, 39.1, 30.4 (sept, J = 2.3 Hz), 24.2.

HRMS (FI) m/z: $[M]^+$ Calcd for $C_{13}H_7O_2NF_6S_2$ 386.9817; Found 386.9815.

IR (KBr) 3488, 3125, 3104, 3048, 1783, 1725, 1609, 1466, 1436, 1400, 1288, 1228, 1173, 1121, 1078, 1014, 990, 944, 881, 863, 834, 795, 757, 716, 702, 570, 530 cm^{-1}

9-(2,2-Bis((trifluoromethyl)thio)cyclopropyl)-9*H*-carbazole (2q)

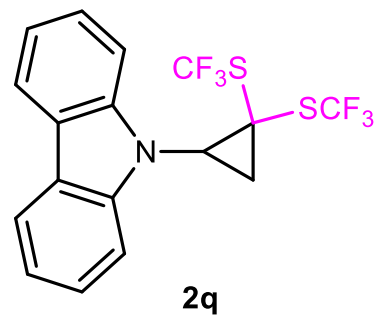

2q

The residue was purified by column chromatography on silica gel with an eluent of petroleum ether (R_f = 0.43) to afford product **2q** (120.1 mg, 59% yield) as a brown solid, m.p. 94-96 ºC.

^{1}H NMR (400 MHz, $CDCl_3$) δ 8.13 (d, J = 7.7 Hz, 2H), 7.63 – 7.42 (m, 4H), 7.42 – 7.29 (m, 2H), 4.18 (t, J = 6.9 Hz, 1H), 2.79 (t, J = 6.6 Hz, 1H), 2.65 (t, J = 7.5 Hz, 1H).

^{19}F NMR (376 MHz, $CDCl_3$) δ -39.40 (s, 3F), -40.14 (s, 3F).

^{13}C NMR (101 MHz, $CDCl_3$) δ 141.0, 140.6, 130.2 (q, J = 310.8 Hz), 130.0 (q, J = 311.2 Hz), 126.8, 126.4, 124.0, 123.9, 121.1, 120.9, 120.7, 110.1, 109.4, 43.3, 32.2 (sept, J = 2.1 Hz), 28.2.

HRMS (FI) m/z: $[M]^+$ Calcd for $C_{17}H_{11}NF_6S_2$ 407.0232; Found 407.0229.

IR (KBr) 3051, 3029, 2936, 1626, 1600, 1491, 1481, 1454, 1431, 1373, 1348, 1332, 1315, 1228, 1162, 1123, 1092, 1078, 1056, 1029, 1005, 975, 928, 899, 845, 748, 724, 631, 614, 555, 520, 437, 417 cm^{-1}

2-(2,2-Bis((trifluoromethyl)thio)cyclopropyl)thiophene (2r)

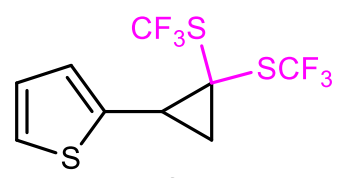

2r

The residue was purified by column chromatography on silica gel with an eluent of petroleum ether (R_f = 0.74) to afford product **2r** (149.0 mg, 92% yield) as a brown oil.

^{1}H NMR (400 MHz, $CDCl_3$) δ 7.30 – 7.18 (m, 1H), 6.98 (dd, J = 5.2, 3.5 Hz, 1H), 6.93 (d, J = 3.0 Hz, 1H), 3.29 (t, J = 8.5 Hz, 1H), 2.24 – 2.09 (m, 2H).

^{19}F NMR (376 MHz, $CDCl_3$) δ -40.10 (s, 3F), -40.70 (s, 3F).

^{13}C NMR (101 MHz, $CDCl_3$) δ 137.6, 130.53 (q, J = 310.3 Hz), 130.50 (q, J = 311.0 Hz), 127.9, 127.4, 126.1, 32.9 (sept, J = 2.3 Hz), 32.4, 27.1.

HRMS (FI) m/z: $[M]^+$ Calcd for $C_9H_6F_6S_3$ 323.9530; Found 323.9527.

IR (KBr) 3112, 3077, 2928, 2857, 1739, 1709, 1601, 1432, 1414, 1166, 1129, 1100, 1078, 1059, 893, 851, 831, 783, 756, 702, 535, 505, 480 cm^{-1}

Isopropyl 2-(4-(4-(2,2-bis((trifluoromethyl)thio)cyclopropyl)benzoyl)phenoxy)-2-methylpropanoate (2s)

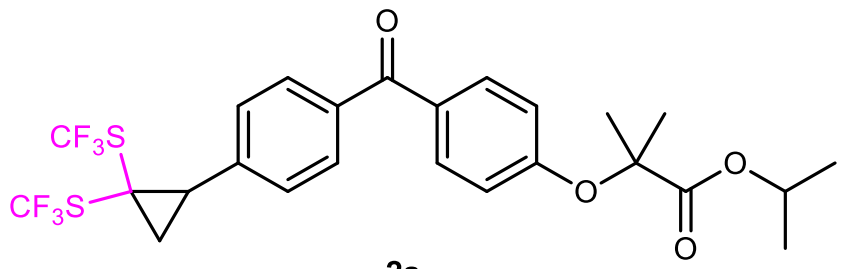

2s

The residue was purified by column chromatography on silica gel with an eluent of petroleum ether/EA (20:1, R_f = 0.20) to afford product **2s** (198.1 mg, 70% yield) as a yellow oil.

^{1}H NMR (400 MHz, $CDCl_3$) δ 7.75 – 7.68 (m, 4H), 7.33 (d, J = 7.9 Hz, 2H), 6.88 – 6.80 (m, 2H), 5.06 (sept, J = 6.2 Hz, 1H), 3.29 (t, J = 8.7 Hz, 1H), 2.25 (t, J = 7.4 Hz, 1H), 2.14 (dd, J = 9.4, 6.7 Hz, 1H), 1.64 (s, 6H), 1.17 (d, J = 6.3 Hz, 6H).

^{19}F NMR (376 MHz, $CDCl_3$) δ -40.13 (s, 3F), -40.89 (s, 3F).

^{13}C NMR (101 MHz, $CDCl_3$) δ 194.8, 173.2, 159.9, 138.0, 137.7, 132.1, 130.5, 130.4 (q, J = 310.6 Hz), 130.3 (q, J = 310.6 Hz), 130.1, 129.0, 117.4, 79.5, 69.4, 37.1, 32.0 (sept, J = 2.2 Hz), 25.4, 24.9, 21.6.

HRMS (ESI) m/z: $[M]^+$ Calcd for $C_{25}H_{24}O_4F_6S_2$ 566.1015; Found 566.1012.

IR (KBr) 3046, 2985, 2940, 2879, 1732, 1657, 1600, 1576, 1504, 1467, 1419, 1385, 1376, 1315, 1281, 1252, 1148, 1102, 1063, 1019, 973, 930, 850, 787, 756, 689, 672, 634, 621, 610, 541, 505 cm^{-1}

(1-Phenyl-[1,1'-bi(cyclopropane)]-2,2-diyl)bis((trifluoromethyl)sulfane) (2u)

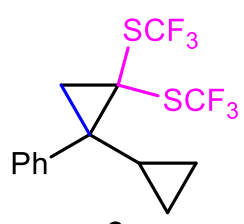

2u

The residue was purified by column chromatography on silica gel with an eluent of petroleum ether (R_f = 0.65) to afford product **2u** (107.9 mg, 65% yield) as a yellow oil.

^{1}H NMR (400 MHz, $CDCl_3$) δ 7.42 – 7.30 (m, 3H), 7.27 – 7.13 (m, 2H), 2.08 (d, *J* = 6.7 Hz, 1H), 1.67 (d, *J* = 6.7 Hz, 1H), 1.58 – 1.47 (m, 1H), 0.73 – 0.650 (m, 1H), 0.60 – 0.50 (m, 1H), 0.32 – 0.16 (m, 2H).

^{19}F NMR (376 MHz, $CDCl_3$) δ -39.54 (s, 3F), -40.04 (s, 3F).

^{13}C NMR (101 MHz, $CDCl_3$) δ 139.5, 130.3 (q, *J* = 311.6 Hz), 130.2 (q, *J* = 310.1 Hz), 129.0, 128.9, 128.1, 45.2, 37.1 (sept, *J* = 2.0 Hz), 28.5, 17.6 (q, *J* = 1.8 Hz), 5.9, 3.8.

HRMS (FI) m/z: $[M]^+$ Calcd for $C_{14}H_{12}F_6S_2$ 358.0279; Found 358.0283.

IR (KBr) 3087, 3064, 3029, 2927, 1602, 1495, 1448, 1429, 1299, 1257, 1227, 1162, 1116, 1085, 1038, 1026, 965, 932, 892, 824, 790, 756, 701, 652, 546, 519, 454 cm^{-1}

(*trans*-2-Methyl-3-phenylcyclopropane-1,1-diyl)bis((trifluoromethyl)sulfane) (2v)

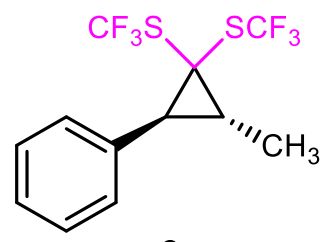

2v

The residue was purified by column chromatography on silica gel with an eluent of petroleum ether (R_f = 0.81) to afford product **2v** (107.9 mg, 65% yield) as a yellow oil.

^{1}H NMR (400 MHz, $CDCl_3$) δ 7.37 – 7.27 (m, 3H), 7.27 – 7.20 (m, 2H), 2.94 (d, *J* = 8.2 Hz, 1H), 2.50 – 2.36 (m, 1H), 1.53 (d, *J* = 6.3 Hz, 3H).

^{19}F NMR (376 MHz, $CDCl_3$) δ -40.04 (s, 3F), -40.25 (s, 3F).

^{13}C NMR (101 MHz, $CDCl_3$) δ 134.4, 130.93 (q, *J* = 310.2 Hz), 130.89 (q, *J* = 310.3 Hz), 129.0, 128.8, 128.3, 44.1, 37.8 (sept, *J* = 1.5 Hz), 32.5, 15.4.

HRMS (FI) m/z: $[M]^+$ Calcd for $C_{12}H_{10}F_6S_2$ 332.0123; Found 332.0120.

IR (KBr) 3071, 3036, 2968, 2934, 2885, 1603, 1499, 1458, 1385, 1163, 1123, 1101, 1076, 1053, 1030, 917, 894, 842, 783, 756, 739, 695, 615, 579, 540, 515, 479 cm^{-1}

(*cis*-2-Methyl-3-phenylcyclopropane-1,1-diyl)bis((trifluoromethyl)sulfane) (2v')

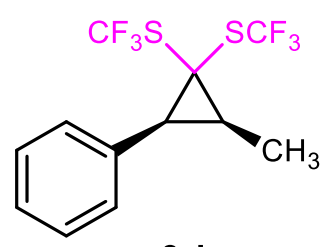

2v'

The residue was purified by column chromatography on silica gel with an eluent of petroleum ether (R_f = 0.83) to afford product **2v'** (31.5 mg, 19% yield) as a yellow oil.

^{1}H NMR (400 MHz, $CDCl_3$) δ 7.41 – 7.28 (m, 3H), 7.21 (d, J = 6.8 Hz, 2H), 3.25 (d, J = 10.1 Hz, 1H), 2.28 – 2.15 (m, 1H), 1.39 (d, J = 6.5 Hz, 3H).

^{19}F NMR (376 MHz, $CDCl_3$) δ -38.33 (s, 3F), -40.71 (s, 3F).

^{13}C NMR (101 MHz, $CDCl_3$) δ 132.6, 130.6 (q, J = 311.2 Hz), 130.0 (q, J = 311.2 Hz), 129.7, 129.0, 128.1, 39.0, 35.4 (sept, J = 1.8 Hz), 30.4, 12.0 (q, J = 1.8 Hz).

HRMS (FI) m/z: $[M]^+$ Calcd for $C_{12}H_{10}F_6S_2$ 332.0123; Found 332.0124.

IR (KBr) 3064, 3033, 2938, 1604, 1499, 1460, 1447, 1389, 1164, 1115, 1099, 1049, 829, 783, 756, 731, 700, 582, 463 cm^{-1}

2-(2,2-Bis((trifluoromethyl)thio)cyclopropyl)-6-methylpyridine (12c')

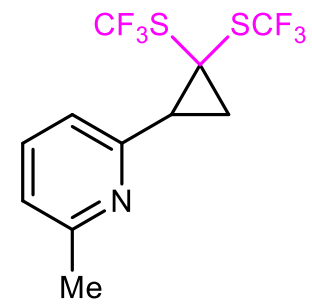

12c'

The residue was purified by column chromatography on silica gel with an eluent of petroleum ether/EA (20:1, R_f = 0.44) to afford product **12c'** (53.3 mg, 32% yield) as a brown oil.

^{1}H NMR (400 MHz, $CDCl_3$) δ 7.55 (t, J = 7.7 Hz, 1H), 7.14 (d, J = 7.6 Hz, 1H), 7.07 (d, J = 7.6 Hz, 1H), 3.33 (t, J = 8.4 Hz, 1H), 2.65 (t, J = 7.0 Hz, 1H), 2.53 (s, 3H), 2.09 (dd, J = 8.6, 6.8 Hz, 1H).

^{19}F NMR (376 MHz, $CDCl_3$) δ -40.41 (s, 3F), -40.83 (s, 3F).

^{13}C NMR (101 MHz, $CDCl_3$) δ 158.5, 152.4, 136.7, 130.6 (q, J = 310.7 Hz), 130.4 (q, J = 310.6 Hz), 122.6, 121.8, 38.4, 32.1 (sept, J = 2.1 Hz), 24.7, 24.2.

HRMS (FI) m/z: $[M]^+$ Calcd for $C_{11}H_9NF_6S_2$ 333.0075; Found 333.0074.

IR (KBr) 3069, 2929, 2857, 1592, 1577, 1461, 1378, 1267, 1164, 1129, 1105, 1077, 994, 848, 805, 770, 756, 741, 624, 589, 551, 505, 418 cm^{-1}

The procedure for skeletal editing of indoles/pyrroles with $:C(SCF_3)_2$ to obtain products 4a-4m, 4o-4u

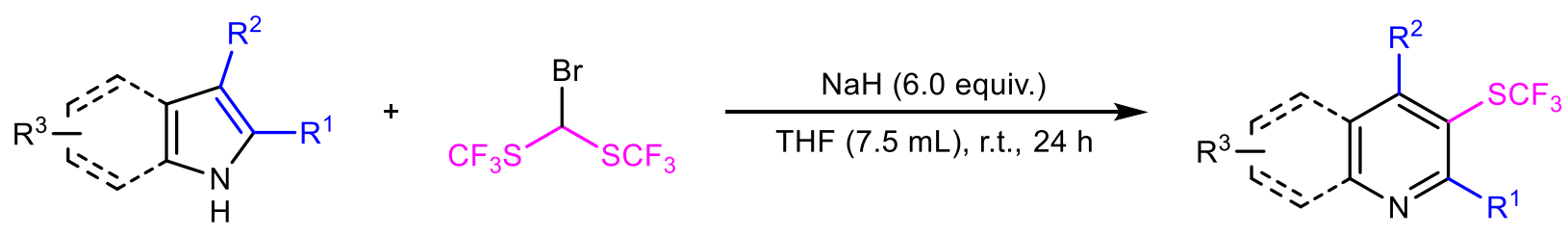

A 25 mL bottom flask was charged with indole/pyrrole **3** (0.5 mmol, 1.0 equiv.), NaH (72.0 mg, 3.0 mmol, 6.0 equiv.) and dry THF (7.5 mL) under a N_2 atmosphere. $BrCH(SCF_3)_2$ (442.5 mg, 1.5 mmol, 3.0 equiv.) was then added. The resulting mixture was stirred at room temperature for 24 h under a N_2 atmosphere. The resulting mixture was then diluted with ethyl acetate (20 mL) and saturated NH_4Cl solution (30 mL). The organic phase was separated and the aqueous phase was extracted 3 times. All organic solutions were combined and then dried over anhydrous Na_2SO_4. The solvent was removed under vacuum and the residue was subjected to column chromatography to give final products **4**.

2,6-Dimethyl-3-((trifluoromethyl)thio)quinoline (4a)

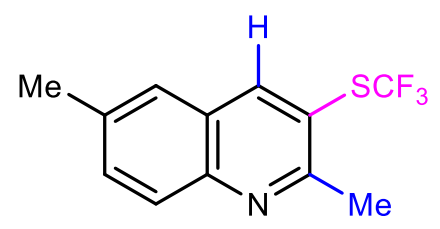

4a

The residue was purified by column chromatography on silica gel with an eluent of petroleum ether/EA (10:1, R_f = 0.53) to afford product **4a** (93.8 mg, 73% yield) as a yellow solid, m.p. 106-108 ºC.

^{1}H NMR (400 MHz, $CDCl_3$) δ 8.33 (s, 1H), 7.90 (d, J = 8.6 Hz, 1H), 7.56 (d, J = 8.6 Hz, 1H), 7.50 (s, 1H), 2.89 (s, 3H), 2.49 (s, 3H).

^{19}F NMR (376 MHz, $CDCl_3$) δ -42.44 (s, 3F).

^{13}C NMR (101 MHz, $CDCl_3$) δ 160.7, 147.2, 146.3, 136.9, 134.1, 129.6 (q, J = 308.9 Hz), 128.5, 127.1, 126.6, 118.2 (q, J = 1.9 Hz), 24.6, 21.6.

HRMS (FI) m/z: $[M]^+$ Calcd for $C_{12}H_{10}NF_3S$ 257.0481; Found 257.0480.

IR (KBr) 3055, 3001, 1626, 1582, 1558, 1489, 1434, 1376, 1223, 1151, 1135, 1112, 1031, 1000, 931, 908, 831, 754, 734, 686, 600, 570, 550, 478 cm^{-1}

2,4-Dimethyl-3-((trifluoromethyl)thio)quinoline (4b)

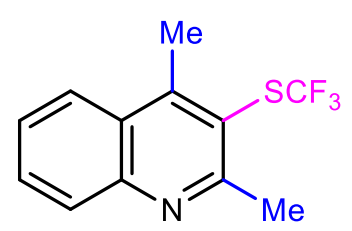

4b

The residue was purified by column chromatography on silica gel with an eluent of petroleum ether/EA (10:1, R_f = 0.43) to afford product **4b** (83.5 mg, 65% yield) as a

yellow solid, m.p. 77-79 ºC.

^{1}H NMR (400 MHz, $CDCl_3$) δ 8.02 – 7.95 (m, 2H), 7.73 (ddd, *J* = 8.4, 6.9, 1.4 Hz, 1H), 7.52 (ddd, *J* = 8.3, 6.9, 1.3 Hz, 1H), 2.96 (s, 3H), 2.95 (s, 3H).

^{19}F NMR (376 MHz, $CDCl_3$) δ -41.88 (s, 3F).

^{13}C NMR (101 MHz, $CDCl_3$) δ 162.5, 153.8, 147.7, 131.3, 129.9 (q, *J* = 309.8 Hz), 129.6, 127.0, 126.7, 125.2, 118.1 (q, *J* = 1.7 Hz), 26.3, 17.9.

HRMS (FI) m/z: $[M]^+$ Calcd for $C_{12}H_{10}NF_3S$ 257.0481; Found 257.0485.

IR (KBr) 3081, 3050, 3026, 2976, 2926, 1649, 1612, 1568, 1557, 1522, 1495, 1435, 1388, 1371, 1329, 1313, 1218, 1150, 1137, 1111, 1024, 1000, 964, 873, 784, 759, 680, 647, 619, 562, 443 cm^{-1}

8-Methyl-3-((trifluoromethyl)thio)quinoline (4c)

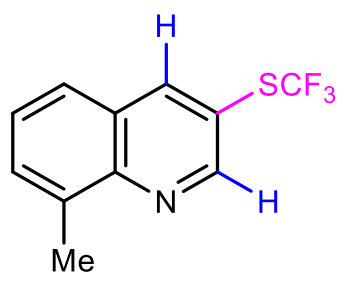

4c

The residue was purified by column chromatography on silica gel with an eluent of petroleum ether/EA (50:1, R_f = 0.47) to afford product **4c** (69.3 mg, 57% yield) as a brown solid, m.p. 60-62 ºC.

^{1}H NMR (400 MHz, $CDCl_3$) δ 9.04 (d, *J* = 2.2 Hz, 1H), 8.46 (d, *J* = 2.2 Hz, 1H), 7.67 (d, *J* = 8.2 Hz, 1H), 7.65 (d, *J* = 8.2 Hz, 1H)., 7.50 (t, *J* = 7.6 Hz, 1H), 2.81 (s, 3H).

^{19}F NMR (376 MHz, $CDCl_3$) δ -42.43 (s, 3F).

^{13}C NMR (101 MHz, $CDCl_3$) δ 153.6, 147.6, 145.0, 137.8, 132.0, 129.5 (q, *J* = 308.8 Hz), 128.0, 127.8, 126.3, 118.2 (q, *J* = 2.0 Hz), 18.1.

HRMS (FI) m/z: $[M]^+$ Calcd for $C_{11}H_8NF_3S$ 243.0324; Found 243.0319.

IR (KBr) 3036, 2962, 2926, 1609, 1575, 1483, 1462, 1434, 1396, 1381, 1367, 1360, 1316, 1153, 1117, 1100, 978, 919, 829, 778, 757, 735, 645, 628, 561, 507, 496 cm^{-1}

2-Phenyl-3-((trifluoromethyl)thio)quinoline (4d)[37]

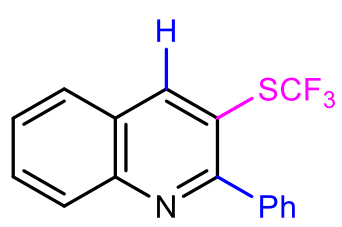

4d

The residue was purified by column chromatography on silica gel with an eluent of petroleum ether/EA (10:1, R_f = 0.44) to afford product **4d** (58.0 mg, 38% yield) as a brown solid.

^{1}H NMR (400 MHz, $CDCl_3$) δ 8.67 (s, 1H), 8.21 (d, J = 8.6 Hz, 1H), 7.90 (d, J = 8.2 Hz, 1H), 7.84 (ddd, J = 9.0, 7.1, 1.9 Hz, 1H), 7.69 – 7.59 (m, 3H), 7.56 – 7.45 (m, 3H).

^{19}F NMR (376 MHz, $CDCl_3$) δ -42.03 (s, 3F).

^{13}C NMR (101 MHz, $CDCl_3$) δ 162.1, 148.3, 146.1, 139.5, 131.9, 129.83, 129.75, 129.4 (q, J = 309.4 Hz), 129.1, 128.3, 127.82, 127.75, 127.3, 118.1 (q, J = 1.9 Hz).

LRMS (EI) m/z: $[M]^+$ Calcd for $C_{16}H_{10}NF_3S$ 305.0; Found 305.1.

2-Isopropyl-3-((trifluoromethyl)thio)quinoline (4e)

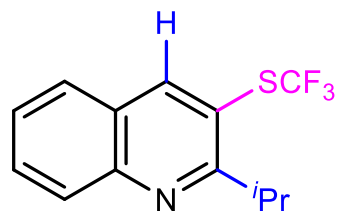

4e

The residue was purified by column chromatography on silica gel with an eluent of petroleum ether/EA (100:1, R_f = 0.45) to afford product **4e** (80.0 mg, 59% yield) as a yellow solid, m.p. 52-54 °C.

^{1}H NMR (400 MHz, $CDCl_3$) δ 8.50 (s, 1H), 8.11 (d, J = 8.5 Hz, 1H), 7.84 – 7.73 (m, 2H), 7.54 (t, J = 7.5 Hz, 1H), 4.03 (sept, J = 6.8 Hz, 1H), 1.41 (d, J = 6.8 Hz, 6H).

^{19}F NMR (376 MHz, $CDCl_3$) δ -42.47 (s, 3F).

^{13}C NMR (101 MHz, $CDCl_3$) δ 169.0, 149.0, 146.8, 131.5, 129.6 (q, J = 308.9 Hz). 129.4, 127.8, 126.92, 126.86, 117.7 (q, J = 1.7 Hz), 33.0, 22.4.

HRMS (FI) m/z: $[M]^+$ Calcd for $C_{13}H_{12}NF_3S$ 271.0637; Found 271.0639.

IR (KBr) 3061, 2968, 2930, 2873, 1619, 1581, 1557, 1485, 1470, 1458, 1403, 1374, 1361, 1346, 1197, 1162, 1126, 1107, 994, 957, 920, 862, 790, 755, 731, 626, 478 cm^{-1}

2-(*tert*-Butyl)-3-((trifluoromethyl)thio)quinoline (4f)

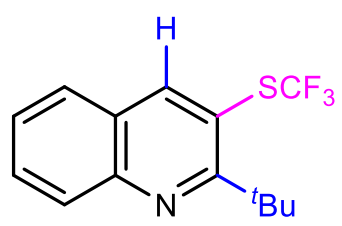

4f

The residue was purified by column chromatography on silica gel with an eluent of petroleum ether (R_f = 0.50) to afford product **4f** (71.3 mg, 50% yield) as a yellow oil.

^{1}H NMR (400 MHz, $CDCl_3$) δ 8.50 (s, 1H), 8.09 (d, *J* = 8.5 Hz, 1H), 7.83 – 7.72 (m, 2H), 7.60 – 7.51 (m, 1H), 1.64 (s, 9H).

^{19}F NMR (376 MHz, $CDCl_3$) δ -41.64 (s, 3F).

^{13}C NMR (101 MHz, $CDCl_3$) δ 168.3, 147.5, 147.0, 131.2, 129.7, 129.6 (q, *J* = 309.3 Hz). 127.4, 127.1, 126.5, 117.8 (q, *J* = 1.9 Hz), 40.6, 30.4.

HRMS (ESI) m/z: $[M+H]^+$ Calcd for $C_{14}H_{15}NF_3S$ 286.0872; Found 286.0874.

IR (KBr) 3060, 2972, 2931, 2908, 2870, 1620, 1578, 1554, 1481, 1462, 1400, 1366, 1304, 1201, 1159, 1136, 1104, 986, 956, 921, 961, 789, 755, 731, 621, 479 cm^{-1}

13-((Trifluoromethyl)thio)-1(2,4)-quinolinacycloundecaphane (4g)

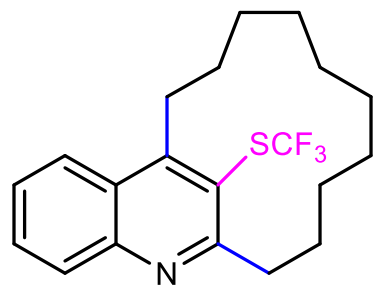

4g

The residue was purified by column chromatography on silica gel with an eluent of petroleum ether/EA (20:1, R_f = 0.55), followed by HPLC purification (Material: 5μm C18, Dimensions: 20×250mm, CH_3CN, flow rate 16.0 mL/min, λ = 220 nm, 25 °C, t_R = 9.708 min) to afford product **4g** (93.6 mg, 51% yield) as a yellow oil.

^{1}H NMR (400 MHz, $CDCl_3$) δ 8.07 (d, *J* = 9.0 Hz, 2H), 7.74 (ddd, *J* = 8.3, 6.8, 1.3 Hz, 1H), 7.54 (ddd, *J* = 8.2, 6.8, 1.3 Hz, 1H), 3.99 – 3.86 (m, 2H), 3.52 (dt, *J* = 13.7, 4.5 Hz, 1H), 3.05 (ddd, *J* = 12.7, 8.1, 4.0 Hz, 1H), 2.25 – 2.04 (m, 2H), 1.95 – 1.78 (m, 1H), 1.71 – 1.57 (m, 1H), 1.36 – 1.16 (m, 4H), 1.07 – 0.91 (m, 2H), 0.91 – 0.80 (m, 1H), 0.80 – 0.64 (m, 3H), 0.22 – 0.03 (m, 2H).

^{19}F NMR (376 MHz, $CDCl_3$) δ -43.31 (s, 3F).

^{13}C NMR (101 MHz, $CDCl_3$) δ 166.4, 157.6, 148.3, 131.2, 130.0, 129.5 (q, *J* = 309.7 Hz), 126.6, 126.4, 125.4, 118.9 (q, *J* = 1.7 Hz), 38.1, 30.6, 28.7, 27.6, 27.22, 27.20, 26.8, 26.5, 26.1, 25.8.

HRMS (ESI) m/z: $[M+H]^+$ Calcd for $C_{20}H_{25}NF_3S$ 368.1654; Found 368.1651.

IR (KBr) 3071, 2931, 2859, 1613, 1563, 1549, 1491, 1460, 1445, 1389, 1357, 1338, 1307, 1214, 1158, 1127, 1107, 1065, 1023, 961, 885, 868, 760, 718, 638, 448 cm^{-1}

6-Methoxy-2-methyl-3-((trifluoromethyl)thio)quinoline (4h)

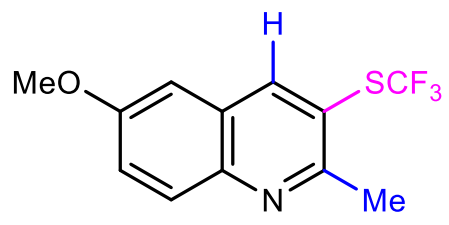

4h

The residue was purified by column chromatography on silica gel with an eluent of petroleum ether/EA (10:1, R_f = 0.37) to afford product **4h** (96.9 mg, 71% yield) as a yellow solid, m.p. 76-78 ºC.

^{1}H NMR (400 MHz, $CDCl_3$) δ 8.31 (s, 1H), 7.89 (d, J = 9.2 Hz, 1H), 7.38 (dd, J = 9.2, 2.8 Hz, 1H), 6.98 (d, J = 2.8 Hz, 1H), 3.87 (s, 3H), 2.86 (s, 3H).

^{19}F NMR (376 MHz, $CDCl_3$) δ -42.36 (s, 3F).

^{13}C NMR (101 MHz, $CDCl_3$) δ 158.9, 158.0, 145.4, 144.8, 130.2, 129.6 (q, J = 313.1 Hz), 128.1, 124.6, 118.4 (q, J = 2.9 Hz), 104.9, 55.7, 24.3.

HRMS (EI) m/z: $[M]^+$ Calcd for $C_{12}H_{10}ONF_3S$ 273.0430; Found 273.0425.

IR (KBr) 3014, 2963, 2939, 2837, 1622, 1578, 1563, 1490, 1464, 1455, 1404, 1377, 1366, 1341, 1296, 1259, 1230, 1224, 1164, 1131, 1115, 1032, 1017, 966, 951, 916, 840, 832, 755, 688, 673, 601, 481, 434 cm^{-1}

2-Methyl-3-((trifluoromethyl)thio)quinolin-7-yl acetate (4i)

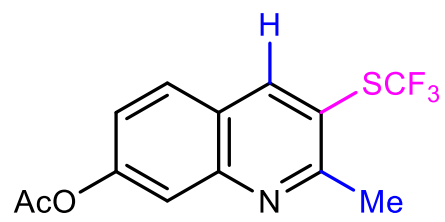

4i

The residue was purified by column chromatography on silica gel with an eluent of petroleum ether/EA (5:1, R_f = 0.30) to afford product **4i** (45.2 mg, 30% yield) as a yellow solid, m.p. 112-114 ºC.

^{1}H NMR (400 MHz, $CDCl_3$) δ 8.44 (s, 1H), 8.06 (d, J = 9.1 Hz, 1H), 7.57 (d, J = 2.6 Hz, 1H), 7.52 (dd, J = 9.1, 2.5 Hz, 1H), 2.93 (s, 3H), 2.36 (s, 3H).

^{19}F NMR (376 MHz, $CDCl_3$) δ -42.17 (s, 3F).

^{13}C NMR (101 MHz, $CDCl_3$) δ 169.4, 161.5, 148.8, 146.6, 146.4, 130.4, 129.5 (q, J = 309.3 Hz), 127.3, 127.1, 119.3 (q, J = 1.6 Hz), 118.5, 24.7, 21.3.

HRMS (EI) m/z: $[M]^+$ Calcd for $C_{13}H_{10}O_2NF_3S$ 301.0379; Found 301.0376.

IR (KBr) 3119, 3054, 3032, 1748, 1620, 1583, 1563, 1488, 1397, 1372, 1342, 1295, 1268, 1247, 1227, 1200, 1150, 1128, 1112, 1018, 985, 963, 937, 913, 875, 848, 754,

676, 656, 582, 509, 482, 435 cm^{-1}

7-Chloro-2-methyl-3-((trifluoromethyl)thio)quinoline (4j)

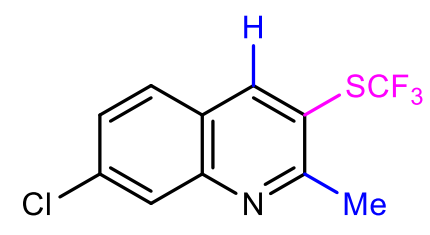

4j

The residue was purified by column chromatography on silica gel with an eluent of petroleum ether/EA (30:1, R_f = 0.26) to afford product **4j** (78.9 mg, 57% yield) as a yellow solid, m.p. 102-104 ºC.

^{1}H NMR (400 MHz, $CDCl_3$) δ 8.41 (s, 1H), 8.00 (d, J = 2.1 Hz, 1H), 7.70 (d, J = 8.7 Hz, 1H), 7.47 (dd, J = 8.7, 2.0 Hz, 1H), 2.90 (s, 3H).

^{19}F NMR (376 MHz, $CDCl_3$) δ -42.25 (s, 3F).

^{13}C NMR (101 MHz, $CDCl_3$) δ 163.0, 148.9, 146.5, 137.9, 129.5 (q, J = 309.3 Hz), 129.0, 128.12, 128.05, 125.4, 118.8 (q, J = 2.0 Hz), 24.8.

HRMS (FI) m/z: $[M]^+$ Calcd for $C_{11}H_7NClF_3S$ 276.9934; Found 276.9933.

IR (KBr) 3047, 3020, 1610, 1577, 1552, 1475, 1439, 1394, 1377, 1360, 1327, 1220, 1155, 1140, 1129, 1112, 1070, 1019, 967, 926, 908, 873, 808, 770, 757, 732, 687, 675, 630, 474, 443 cm^{-1}

6-Bromo-2-methyl-3-((trifluoromethyl)thio)quinoline (4k)

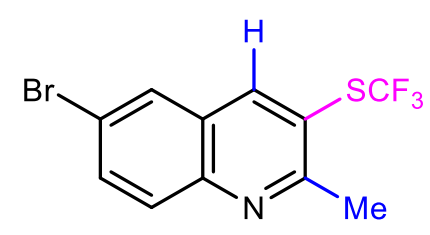

4k

The residue was purified by column chromatography on silica gel with an eluent of petroleum ether/EA (20:1, R_f = 0.41) to afford product **4k** (97.9 mg, 61% yield) as a yellow solid, m.p. 120-122 ºC.

^{1}H NMR (400 MHz, $CDCl_3$) δ 8.33 (s, 1H), 7.90 (d, J = 2.1 Hz, 1H), 7.86 (d, J = 9.0 Hz, 1H), 7.78 (dd, J = 9.0, 2.1 Hz, 1H), 2.88 (s, 3H).

^{19}F NMR (376 MHz, $CDCl_3$) δ -42.09 (s, 3F).

^{13}C NMR (101 MHz, $CDCl_3$) δ 162.1, 147.1, 145.5, 135.2, 130.6, 129.8, 129.5 (q, J = 309.3 Hz), 128.1, 120.8, 119.7 (q, J = 1.8 Hz), 24.8.

HRMS (EI) m/z: $[M]^+$ Calcd for $C_{11}H_7NBrF_3S$ 320.9429; Found 320.9436.

IR (KBr) 3037, 3007, 1603, 1578, 1553, 1474, 1438, 1376, 1357, 1328, 1282, 1221, 1153, 1136, 1111, 1105, 1064, 1014, 1006, 962, 929, 911, 875, 829, 817, 760, 740, 721, 675, 646, 568, 544, 483, 431 cm^{-1}

2-Methyl-6-(trifluoromethoxy)-3-((trifluoromethyl)thio)quinoline (4l)

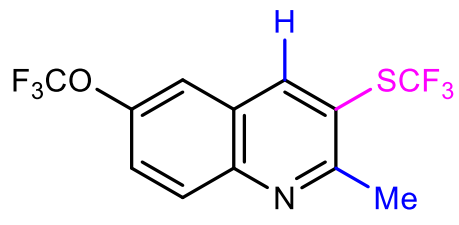

4l

The residue was purified by column chromatography on silica gel with an eluent of petroleum ether/EA (20:1, R_f = 0.52) to afford product **4l** (81.8 mg, 50% yield) as a yellow solid, m.p. 93-95 ºC.

^{1}H NMR (400 MHz, $CDCl_3$) δ 8.46 (s, 1H), 8.07 (d, J = 8.8 Hz, 1H), 7.65 – 7.57 (m, 2H), 2.93 (s, 3H).

^{19}F NMR (376 MHz, $CDCl_3$) δ -42.12 (s, 3F), -58.01 (s, 3F).

^{13}C NMR (101 MHz, $CDCl_3$) δ 162.3, 147.3 (q, J = 1.8 Hz), 146.8, 146.3, 131.3, 129.5 (q, J = 309.2 Hz), 127.2, 125.8, 120.7 (q, J = 258.4 Hz), 120.1 (q, J = 1.9 Hz), 117.7 (q, J = 1.1 Hz), 24.8.

HRMS (ESI) m/z: $[M+H]^+$ Calcd for $C_{12}H_8ONF_6S$ 328.0225; Found 328.0221.

IR (KBr) 3064, 3047, 1625, 1588, 1560, 1489, 1449, 1387, 1381, 1361, 1338, 1282, 1221, 1205, 1181, 1157, 1124, 1103, 1033, 1018, 976, 962, 937, 909, 889, 843, 831, 786, 756, 735, 714, 691, 671, 631, 587, 554, 506, 486, 431 cm^{-1}

Methyl 2-methyl-3-((trifluoromethyl)thio)quinoline-6-carboxylate (4m)

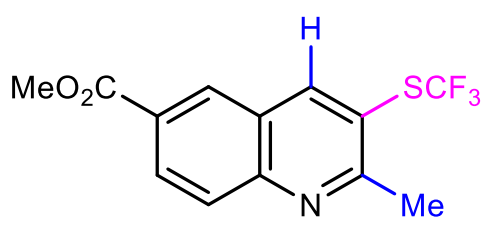

4m

The residue was purified by column chromatography on silica gel with an eluent of petroleum ether/EA (5:1, R_f = 0.50) to afford product **4m** (79.8 mg, 53% yield) as a yellow solid, m.p. 162-164 ºC.

^{1}H NMR (400 MHz, $CDCl_3$) δ 8.59 – 8.54 (m, 2H), 8.35 (dd, J = 8.8, 1.9 Hz, 1H), 8.06 (d, J = 8.8 Hz, 1H), 3.98 (s, 3H), 2.95 (s, 3H).

^{19}F NMR (376 MHz, $CDCl_3$) δ -42.15 (s, 3F).

^{13}C NMR (101 MHz, $CDCl_3$) δ 166.3, 164.2, 150.3, 147.9, 131.3, 131.0, 129.5 (q, J = 309.3 Hz), 129.2, 128.6, 126.3, 119.6 (q, J = 1.9 Hz), 52.7, 25.0.

HRMS (EI) m/z: $[M]^+$ Calcd for $C_{13}H_{10}O_2NF_3S$ 301.0379; Found 301.0376.

IR (KBr) 3059, 3029, 3002, 2954, 1715, 1622, 1557, 1489, 1447, 1376, 1301, 1281, 1262, 1188, 1158, 1114, 1016, 990, 958, 861, 800, 788, 754, 674, 650, 512, 483 cm^{-1}

2-(3,5,5,6,8,8-Hexamethyl-5,6,7,8-tetrahydronaphthalen-2-yl)-3-((trifluoromethyl)thio)quinoline (4o)

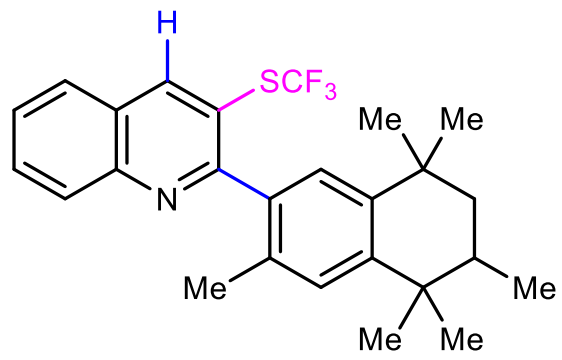

4o

The residue was purified by column chromatography on silica gel with an eluent of petroleum ether/EA (20:1, R_f = 0.49), followed by HPLC purification (Material: 5μm C18, Dimensions: 20×250mm, CH_3CN, flow rate 16.0 mL/min, λ = 220 nm, 25 °C, t_R = 7.735 min) to afford product **4o** (130.7 mg, 59% yield) as a yellow oil.

^{1}H NMR (400 MHz, $CDCl_3$) δ 8.64 (s, 1H), 8.21 (d, J = 8.5 Hz, 1H), 7.90 (d, J = 8.1 Hz, 1H), 7.81 (t, J = 7.5 Hz, 1H), 7.62 (t, J = 7.6 Hz, 1H), 7.30 (s, 1H), 7.19 (s, 1H), 2.14 (s, 3H), 2.01 – 1.89 (m, 1H), 1.70 (t, J = 13.2 Hz, 1H), 1.45 – 1.36 (m, 4H), 1.30 (s, 3H), 1.28 (s, 3H), 1.14 (s, 3H), 1.03 (d, J = 6.7 Hz, 3H).

^{19}F NMR (376 MHz, $CDCl_3$) δ -41.53 (s, 3F).

^{13}C NMR (101 MHz, $CDCl_3$) δ 162.8, 148.0, 146.7, 144.2, 142.0, 136.1, 132.6, 131.5, 129.7, 129.5 (q, J = 309.3 Hz), 128.9, 127.7, 127.5, 127.3, 119.9 (q, J = 1.7 Hz), 43.9, 37.8, 34.7, 34.2, 32.5, 32.1, 28.7, 25.1, 19.5, 17.0.

HRMS (ESI) m/z: $[M+H]^+$ Calcd for $C_{26}H_{29}NF_3S$ 444.1967; Found 444.1963.

IR (KBr) 3057, 3023, 2964, 2928, 2880, 1616, 1577, 1552, 1503, 1482, 1455, 1404, 1390, 1364, 1301, 1260, 1239, 1197, 1160, 1131, 1108, 1087, 1035, 1011, 998, 951, 919, 896, 881, 861, 847, 792, 757, 742, 647, 604, 523, 479, 468 cm^{-1}

2,6-Dimethyl-3-((trifluoromethyl)thio)pyridine (4p)

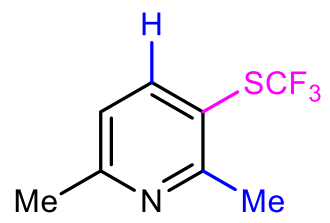

4p

The residue was purified by column chromatography on silica gel with an eluent of petroleum ether/EA (10:1, R_f = 0.28) to afford product **4p** (56.9 mg, 55% yield) as a brown oil.

^{1}H NMR (400 MHz, $CDCl_3$) δ 7.80 (d, J = 7.9 Hz, 1H), 7.05 (d, J = 7.9 Hz, 1H), 2.74 (s, 3H), 2.56 (s, 3H).

^{19}F NMR (376 MHz, $CDCl_3$) δ -42.64 (s, 3F).

^{13}C NMR (101 MHz, $CDCl_3$) δ 163.1, 161.3, 146.1, 129.7 (q, J = 308.8 Hz), 122.0, 117.3 (q, J = 2.0 Hz), 24.7, 23.9.

HRMS (FI) m/z: $[M]^+$ Calcd for $C_8H_8NF_3S$ 207.0324; Found 207.0328.

IR (KBr) 2958, 2927, 2857, 1712, 1644, 1579, 1434, 1261, 1144, 1103, 1047, 1017, 821, 797, 756, 735, 578, 486, 419 cm^{-1}

2,6-Diphenyl-3-((trifluoromethyl)thio)pyridine (4q)

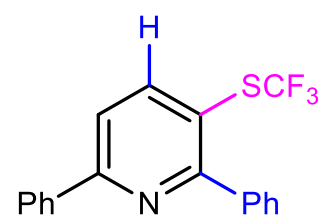

4q

The residue was purified by column chromatography on silica gel with an eluent of petroleum ether (R_f = 0.59), followed by HPLC purification (Material: 5μm C18, Dimensions: 20×250mm, CH_3CN/H_2O = 90:10, flow rate 16.0 mL/min, λ = 220 nm, 25 °C, t_R = 5.271 min) to afford product **4q** (62.9 mg, 38% yield) as a white solid, m.p. 76-78 °C.

^{1}H NMR (400 MHz, $CDCl_3$) δ 8.21 – 8.11 (m, 3H), 7.79 (d, J = 8.2 Hz, 1H), 7.73 – 7.66 (m, 2H), 7.57 – 7.43 (m, 6H).

^{19}F NMR (376 MHz, $CDCl_3$) δ -41.83 (s, 3F).

^{13}C NMR (101 MHz, $CDCl_3$) δ 163.1, 158.6, 145.7, 139.5, 138.0, 130.13, 130.05, 129.5 (q, J = 310.3 Hz), 129.0, 128.1, 127.5, 119.3, 118.5 (q, J = 1.7 Hz).

HRMS (ESI) m/z: $[M+H]^+$ Calcd for $C_{18}H_{13}NF_3S$ 332.0715; Found 332.0712.

IR (KBr) 3090, 3061, 3033, 1563, 1549, 1494, 1451, 1424, 1373, 1312, 1279, 1160,

1118, 1101, 1075, 1031, 1017, 969,0 919, 834, 793, 770, 747, 695, 673, 631, 615, 573, 492, 481 cm^{-1}

2-Methyl-5-((trifluoromethyl)thio)pyridine (4r)

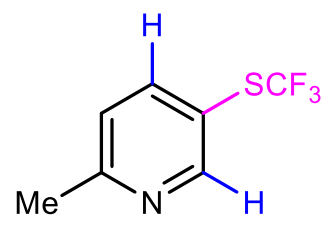

4r

The residue was purified by column chromatography on silica gel with an eluent of petroleum ether/EA (10:1, R_f = 0.28) to afford product **4r** (38.6 mg, 40% yield) as a brown oil.

^{1}H NMR (400 MHz, $CDCl_3$) δ 8.71 (d, J = 2.4 Hz, 1H), 7.85 (dd, J = 8.2, 2.3 Hz, 1H), 7.24 (d, J = 8.1 Hz, 1H), 2.61 (s, 3H).

^{19}F NMR (376 MHz, $CDCl_3$) δ -42.90 (s, 3F).

^{13}C NMR (101 MHz, $CDCl_3$) δ 161.7, 155.6, 144.1, 129.4 (q, J = 308.5 Hz), 124.3, 118.8 (q, J = 2.2 Hz), 24.6.

HRMS (EI) m/z: $[M]^+$ Calcd for $C_7H_6NF_3S$ 193.0168; Found 193.0167.

IR (KBr) 2918, 2848, 1712, 1579, 1578, 1464, 1440, 1397, 1260, 1162, 1131, 1102, 1047, 756, 742 cm^{-1}

2-Phenyl-5-((trifluoromethyl)thio)pyridine (4s)[38]

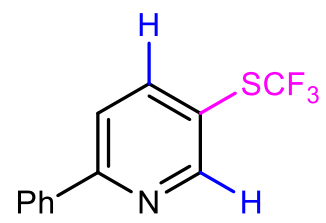

4s

The residue was purified by column chromatography on silica gel with an eluent of petroleum ether/EA (20:1, R_f = 0.58) to afford product **4s** (96.9 mg, 76% yield) as a brown solid.

^{1}H NMR (400 MHz, $CDCl_3$) δ 8.90 (d, J = 2.3 Hz, 1H), 8.04 (dd, J = 8.0, 1.7 Hz, 2H), 8.00 (dd, J = 8.3, 2.3 Hz, 1H), 7.77 (d, J = 8.3 Hz, 1H), 7.53 – 7.45 (m, 3H).

^{19}F NMR (376 MHz, $CDCl_3$) δ -42.57 (s, 3F).

^{13}C NMR (101 MHz, $CDCl_3$) δ 159.6, 155.9, 144.4, 138.0, 130.2, 129.3 (q, J = 308.8 Hz). 129.1, 127.4, 121.0, 120.0 (q, J = 2.0 Hz).

LRMS (EI) m/z: $[M]^+$ Calcd for $C_{12}H_8NF_3S$ 255.0; Found 255.1.

2-Methyl-6-phenyl-3-((trifluoromethyl)thio)pyridine (4t)

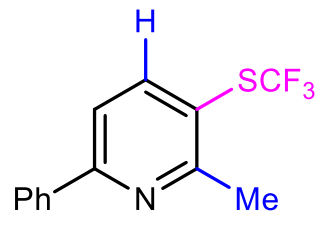

4t

The residue was purified by column chromatography on silica gel with an eluent of petroleum ether/EA (20:1, R_f = 0.43) to afford product **4t** (96.8 mg, 72% yield) as a brown solid, m.p. 55-57 ºC.

^{1}H NMR (400 MHz, $CDCl_3$) δ 8.08 – 8.02 (m, 2H), 7.97 (d, J = 8.1 Hz, 1H), 7.61 (d, J = 8.1 Hz, 1H), 7.54 – 7.44 (m, 3H), 2.87 (s, 3H).

^{19}F NMR (376 MHz, $CDCl_3$) δ -42.30 (s, 3F).

^{13}C NMR (101 MHz, $CDCl_3$) δ 163.5, 159.2, 146.4, 138.3, 130.0, 129.7 (q, J = 309.2 Hz), 129.0, 127.4, 118.8, 118.6 (q, J = 2.0 Hz), 24.2.

HRMS (ESI) m/z: $[M+H]^+$ Calcd for $C_{13}H_{11}NF_3S$ 270.0559; Found 270.0557.

IR (KBr) 3090, 3064, 3037, 2932, 1570, 1554, 1495, 1430, 1383, 1304, 1280, 1159, 1131, 1108, 1084, 1044, 1030, 835, 780, 756, 739, 708, 691, 665, 487 cm^{-1}

6-(*tert*-Butyl)-2-phenyl-3-((trifluoromethyl)thio)pyridine (4u)

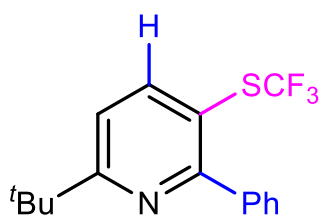

4u

The residue was purified by column chromatography on silica gel with an eluent of petroleum ether/EA (50:1, R_f = 0.24) to afford product **4u** (124.4 mg, 80% yield) as a yellow oil.

^{1}H NMR (400 MHz, $CDCl_3$) δ 8.06 (d, J = 8.3 Hz, 1H), 7.73 – 7.67 (m, 2H), 7.55 – 7.47 (m, 3H), 7.41 (d, J = 8.2 Hz, 1H), 1.47 (s, 9H).

^{19}F NMR (376 MHz, $CDCl_3$) δ -41.99 (s, 3F).

^{13}C NMR (101 MHz, $CDCl_3$) δ 171.6, 161.9, 145.4, 139.8, 130.2, 129.6 (q, J = 309.2 Hz), 128.8, 128.0, 118.5, 116.6 (q, J = 1.8 Hz), 38.1, 30.2.

HRMS (ESI) m/z: $[M+H]^+$ Calcd for $C_{16}H_{17}NF_3S$ 312.1028; Found 312.1023.

IR (KBr) 3090, 3061, 3036, 2965, 2906, 2869, 1567, 1548, 1496, 1479, 1459, 1449, 1424, 1376, 1359, 1261, 1227, 1201, 1158, 1129, 1105, 1032, 1021, 916, 870, 835, 795,

761, 697, 681, 628, 546, 501, 472 cm^{-1}

The procedure for X-H insertion of phenols, thiophenols and *N*-aromatic heterocycles with :C(SCF_3)$_2$ to obtain products 6a-6v

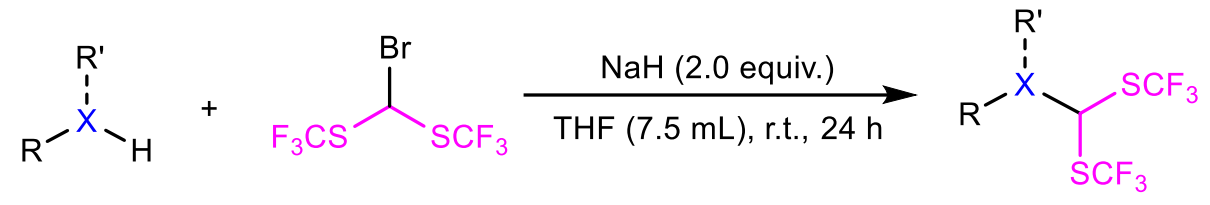

5, 0.5 mmol 2.5 equiv. **6**

A 25 mL bottom flask was charged with phenols, thiophenols or *N*-aromatic heterocycles **5** (0.5 mmol, 1.0 equiv.), NaH (24.0 mg, 1.0 mmol, 2.0 equiv.) and dry THF (7.5 mL) under a N_2 atmosphere. BrCH(SCF_3)$_2$ (368.8 mg, 1.25 mmol, 2.5 equiv.) was then added. The resulting mixture was stirred at room temperature for 24 h under a N_2 atmosphere. The resulting mixture was then diluted with ethyl acetate (20 mL) and saturated NH_4Cl solution (30 mL). The organic phase was separated and the aqueous phase was extracted 3 times. All organic solutions were combined and then dried over anhydrous Na_2SO_4. The solvent was removed under vacuum and the residue was subjected to column chromatography to give final products **6a-6g, 6j-6o, 6q-6v**. For the products **6h** and **6i**, the reagent loadings were modified as follows: BrCH(SCF_3)$_2$ (737.5 mg, 2.5 mmol, 5.0 equiv.) and NaH (48.0 mg, 2.0 mmol, 4.0 equiv.). For the product **6p**, the reagent loadings were modified as follows: BrCH(SCF_3)$_2$ (221.3 mg, 0.75 mmol, 1.5 equiv.) and NaH (14.4 mg, 0.6 mmol, 1.2 equiv.). For the product **6p′**, the reagent loadings were modified as follows: BrCH(SCF_3)$_2$ (442.5 mg, 1.5 mmol, 3.0 equiv.) and NaH (30.0 mg, 1.25 mmol, 2.5 equiv.).

(Phenoxymethylene)bis((trifluoromethyl)sulfane) (6a)

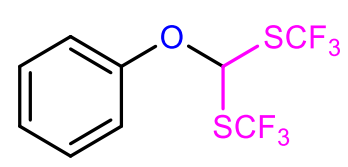

6a

The residue was purified by column chromatography on silica gel with an eluent of petroleum ether (R_f = 0.82) to afford product **6a** (110.9 mg, 72% yield) as a yellow oil.

^{1}H NMR (400 MHz, $CDCl_3$) δ 7.40 (t, *J* = 7.9 Hz, 2H), 7.21 (t, *J* = 7.4 Hz, 1H), 7.14 (d, *J* = 8.2 Hz, 2H), 6.94 (s, 1H).

^{19}F NMR (376 MHz, $CDCl_3$) δ -39.86 (s, 6F).

^{13}C NMR (101 MHz, $CDCl_3$) δ 155.2, 130.2, 128.9 (q, J = 310.4 Hz), 125.2, 118.5, 83.9 (sept, J = 2.9 Hz).

HRMS (FI) m/z: $[M]^+$ Calcd for $C_9H_6OF_6S_2$ 307.9759; Found 307.9757.

IR (KBr) 2930, 1592, 1489, 1306, 1271, 1205, 1155, 1120, 1096, 1039, 997, 907, 836, 758, 730, 688, 419 cm^{-1}

((4-Methoxyphenoxy)methylene)bis((trifluoromethyl)sulfane) (6b)

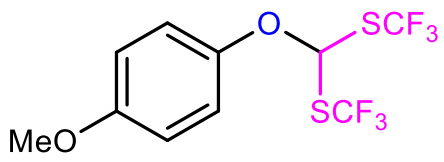

6b

The residue was purified by column chromatography on silica gel with an eluent of petroleum ether (R_f = 0.47) to afford product **6b** (118.3 mg, 70% yield) as a yellow oil.

^{1}H NMR (400 MHz, $CDCl_3$) δ 7.13 – 7.06 (m, 2H), 6.93 – 6.87 (m, 2H), 6.81 (s, 1H), 3.80 (s, 3H).

^{19}F NMR (376 MHz, $CDCl_3$) δ -39.85 (s, 6F).

^{13}C NMR (101 MHz, $CDCl_3$) δ 157.3, 148.9, 128.9 (q, J = 310.5 Hz), 120.6, 115.1, 85.3 (sept, J = 2.9 Hz), 55.7.

HRMS (FI) m/z: $[M]^+$ Calcd for $C_{10}H_8O_2F_6S_2$ 337.9864; Found 337.9860.

IR (KBr) 3006, 2955, 2917, 2840, 1612, 1593, 1506, 1467, 1443, 1299, 1251, 1197, 1163, 1140, 1122, 1095, 1039, 1019, 859, 849, 826, 804, 757, 733, 690, 528, 472 cm^{-1}

((2-Fluorophenoxy)methylene)bis((trifluoromethyl)sulfane) (6c)

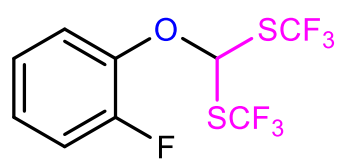

6c

The residue was purified by column chromatography on silica gel with an eluent of petroleum ether (R_f = 0.60) to afford product **6c** (107.6 mg, 66% yield) as a yellow oil.

^{1}H NMR (400 MHz, $CDCl_3$) δ 7.25 – 7.12 (m, 4H), 6.97 (s, 1H).

^{19}F NMR (376 MHz, $CDCl_3$) δ -40.00 (d, J = 3.7 Hz, 6F), -129.47 – -129.61 (m, 1F).

^{13}C NMR (101 MHz, $CDCl_3$) δ 154.6 (d, J = 249.0 Hz), 142.0 (d, J = 11.3 Hz), 128.7 (q, J = 310.4 Hz), 126.8 (d, J = 7.2 Hz), 125.0 (d, J = 3.9 Hz), 122.2, 117.5 (d, J = 18.6 Hz), 85.0 – 84.8 (m).

HRMS (FI) m/z: $[M]^+$ Calcd for $C_9H_5OF_7S_2$ 325.9665; Found 325.9659.

IR (KBr) 2954, 2924, 2855, 1500, 1458, 1379, 1255, 1156, 1102, 1030, 1003, 913, 816, 760, 715, 419 cm^{-1}

((4-Nitrophenoxy)methylene)bis((trifluoromethyl)sulfane) (6d)

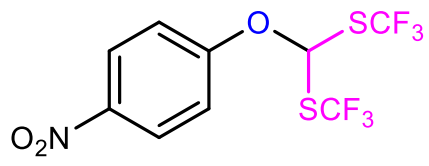

6d

The residue was purified by column chromatography on silica gel with an eluent of petroleum ether/EA (20:1, R_f = 0.54) to afford product **6d** (100.6 mg, 57% yield) as a yellow oil.

^{1}H NMR (400 MHz, $CDCl_3$) δ 8.28 (d, J = 9.3 Hz, 2H), 7.22 (d, J = 9.3 Hz, 2H), 7.03 (s, 1H).

^{19}F NMR (376 MHz, $CDCl_3$) δ -39.75 (s, 6F).

^{13}C NMR (101 MHz, $CDCl_3$) δ 159.2, 144.4, 128.6 (q, J = 310.9 Hz), 126.2, 117.8, 82.4 (sept, J = 2.9 Hz).

HRMS (FI) m/z: $[M]^+$ Calcd for $C_9H_5O_3NF_6S_2$ 352.9610; Found 352.9609.

IR (KBr) 3118, 3088, 2961, 2861, 1615, 1593, 1526, 1493, 1348, 1299, 1226, 1170, 1146, 1116, 1095, 1017, 864, 848, 790, 758, 749, 732, 685, 645, 485, 472, 419 cm^{-1}

((2-Vinylphenoxy)methylene)bis((trifluoromethyl)sulfane) (6e)

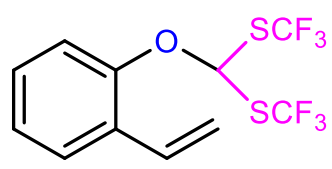

6e

The residue was purified by column chromatography on silica gel with an eluent of petroleum ether (R_f = 0.64) to afford product **6e** (46.8 mg, 28% yield) as a yellow oil.

^{1}H NMR (400 MHz, $CDCl_3$) δ 7.58 (dt, J = 7.7, 1.3 Hz, 1H), 7.31 (ddt, J = 9.1, 7.9, 1.3 Hz, 1H), 7.17 (t, J = 7.5 Hz, 1H), 7.11 (d, J = 8.2 Hz, 1H), 7.05 – 6.94 (m, 2H), 5.77 (dt, J = 17.7, 1.2 Hz, 1H), 5.36 (dt, J = 11.1, 1.1 Hz, 1H).

^{19}F NMR (376 MHz, $CDCl_3$) δ -39.88 (s, 6F).

^{13}C NMR (101 MHz, $CDCl_3$) δ 152.2, 130.6, 129.6, 129.2, 128.8 (q, J = 310.5 Hz), 127.3, 125.0, 116.5, 116.0, 83.7 (sept, J = 3.0 Hz).

HRMS (FI) m/z: $[M]^+$ Calcd for $C_{11}H_8OF_6S_2$ 333.9915; Found 333.9913.

IR (KBr) 3078, 2969, 2927, 2856, 1732, 1629, 1601, 1577, 1484, 1453, 1421, 1291, 1216, 1169, 1142, 1096, 1019, 916, 838, 758, 730 cm^{-1}

((2,6-Diisopropylphenoxy)methylene)bis((trifluoromethyl)sulfane) (6f)

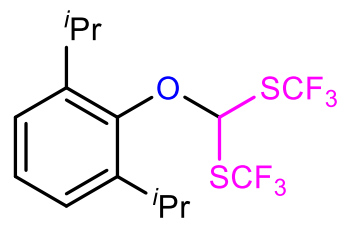

6f

The residue was purified by column chromatography on silica gel with an eluent of petroleum ether (R_f = 0.65) to afford product **6f** (64.7 mg, 33% yield) as a yellow oil.

^{1}H NMR (400 MHz, $CDCl_3$) δ 7.24 – 7.13 (m, 3H), 6.78 (s, 1H), 3.16 (sept, J = 6.8 Hz, 2H), 1.24 (d, J = 6.8 Hz, 12H).

^{19}F NMR (376 MHz, $CDCl_3$) δ -39.87 (s, 6F).

^{13}C NMR (101 MHz, $CDCl_3$) δ 148.5, 142.0, 128.9 (q, J = 310.1 Hz), 127.0, 124.7, 86.3 (sept, J = 2.7 Hz), 27.6, 23.8.

HRMS (FI) m/z: $[M]^+$ Calcd for $C_{15}H_{18}OF_6S_2$ 392.0698; Found 392.0696.

IR (KBr) 2967, 2929, 2875, 1441, 1366, 1326, 1270, 1166, 1143, 1122, 1094, 1047, 1009, 846, 791, 758, 713, 419 cm^{-1}

2-(Bis((trifluoromethyl)thio)methoxy)-6-methylpyridine (6g)

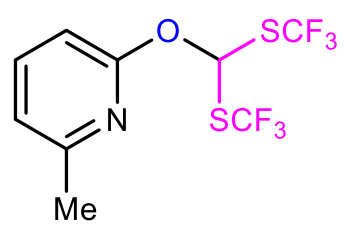

6g

The residue was purified by column chromatography on silica gel with an eluent of petroleum ether/EA (50:1, R_f = 0.47) to afford product **6g** (85.6 mg, 53% yield) as a yellow oil.

^{1}H NMR (400 MHz, $CDCl_3$) δ 7.58 (t, J = 7.8 Hz, 1H), 7.25 (s, 1H), 6.89 (d, J = 7.3 Hz, 1H), 6.68 (d, J = 8.2 Hz, 1H), 2.50 (s, 3H).

^{19}F NMR (376 MHz, $CDCl_3$) δ -42.55 (s, 6F).

^{13}C NMR (101 MHz, $CDCl_3$) δ 158.5, 156.0, 140.6, 129.8 (q, J = 310.3 Hz), 118.6, 108.9, 78.1 (sept, J = 3.6 Hz), 23.8.

HRMS (FI) m/z: $[M]^+$ Calcd for $C_9H_7ONF_6S_2$ 322.9868; Found 322.9862.

IR (KBr) 2930, 1607, 1578, 1457, 1378, 1337, 1287, 1253, 1228, 1127, 1100, 1031, 992, 789, 749, 731, 710, 474, 457 cm^{-1}

(4-(Bis((trifluoromethyl)thio)methoxy)phenyl)methanol (6h)

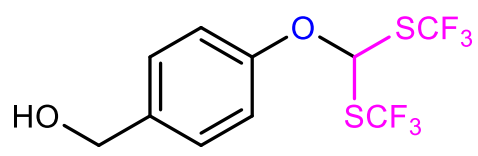

6h

The residue was purified by column chromatography on silica gel with an eluent of petroleum ether/EA (3:1, R_f = 0.27) to afford product **6h** (104.8 mg, 62% yield) as a yellow oil.

^{1}H NMR (400 MHz, $CDCl_3$) δ 7.35 (d, *J* = 8.2 Hz, 2H), 7.10 (d, *J* = 8.3 Hz, 2H), 6.90 (s, 1H), 4.62 (s, 2H), 2.50 (s, 1H).

^{19}F NMR (376 MHz, $CDCl_3$) δ -39.82 (s, 6F).

^{13}C NMR (101 MHz, $CDCl_3$) δ 154.5, 137.8, 128.82, 128.79 (q, *J* = 310.4 Hz), 118.5, 83.8 (sept, *J* = 2.8 Hz), 64.6.

HRMS (FI) m/z: $[M]^+$ Calcd for $C_{10}H_8O_2F_6S_2$ 337.9864; Found 337.9867.

IR (KBr) 3346, 2932, 2879, 1608, 1592, 1507, 1462, 1421, 1306, 1209, 1170, 1142, 1118, 1096, 1025, 1012, 940, 852, 828, 802, 758, 728, 582, 551, 486, 472 cm^{-1}

(8*R*,9*S*,13*S*,14*S*,17*R*)-3-(Bis((trifluoromethyl)thio)methoxy)-13-methyl-7,8,9,11,12,13,14,15,16,17-decahydro-6*H*-cyclopenta[*a*]phenanthren-17-ol (6i)

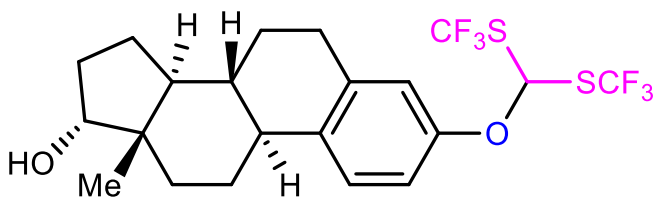

6i

The residue was purified by column chromatography on silica gel with an eluent of petroleum ether/EA (3:1, R_f = 0.27) to afford product **6i** (143.4 mg, 59% yield) as a yellow oil.

^{1}H NMR (400 MHz, $CDCl_3$) δ 7.29 (d, *J* = 8.6 Hz, 1H), 6.93 – 6.88 (m, 2H), 6.83 (d, *J* = 2.7 Hz, 1H), 3.75 (t, *J* = 8.5 Hz, 1H), 2.94 – 2.84 (m, 2H), 2.38 – 2.29 (m, 1H), 2.27 – 2.18 (m, 1H), 2.17 – 2.08 (m, 1H), 2.02 – 1.88 (m, 3H), 1.78 – 1.67 (m, 1H), 1.56 – 1.29 (m, 6H), 1.25 – 1.16 (m, 1H), 0.80 (s, 3H).

^{19}F NMR (376 MHz, $CDCl_3$) δ -39.69 (s, 6F).

^{13}C NMR (101 MHz, $CDCl_3$) δ 153.0, 139.0, 137.4, 128.8 (q, *J* = 310.3 Hz), 127.0, 118.3, 115.2, 83.7 (sept, *J* = 2.9 Hz), 82.0, 50.2, 44.2, 43.4, 38.7, 36.8, 30.7, 29.9, 27.2, 26.3, 23.3, 11.2.

HRMS (FI) m/z: $[M]^+$ Calcd for $C_{21}H_{24}O_2F_6S_2$ 486.1116; Found 486.1123.

IR (KBr) 3413, 2931, 2871, 1768, 1605, 1579, 1494, 1473, 1447, 1435, 1381, 1337, 1310, 1270, 1248, 1213, 1155, 1096, 1054, 1023, 963, 926, 915, 901, 879, 823, 780, 757, 722, 626, 543, 486, 446 cm^{-1}

((*p*-Tolylthio)methylene)bis((trifluoromethyl)sulfane) (6j)

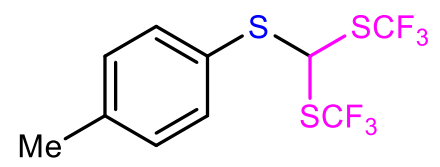

6j

The residue was purified by column chromatography on silica gel with an eluent of petroleum ether (R_f = 0.85) to afford product **6j** (109.9 mg, 65% yield) as a yellow oil.

^{1}H NMR (400 MHz, $CDCl_3$) δ 7.49 (d, *J* = 8.1 Hz, 2H), 7.23 (d, *J* = 7.8 Hz, 2H), 5.65 (s, 1H), 2.40 (s, 3H).

^{19}F NMR (376 MHz, $CDCl_3$) δ -41.34 (s, 6F).

^{13}C NMR (101 MHz, $CDCl_3$) δ 141.3, 135.5, 130.5, 128.8 (q, *J* = 311.3 Hz), 126.8, 53.6 (sept, *J* = 2.2 Hz), 21.6.

HRMS (FI) m/z: $[M]^+$ Calcd for $C_{10}H_8F_6S_3$ 337.9687; Found 337.9692.

IR (KBr) 3029, 2958, 2927, 2856, 1598, 1492, 1401, 1304, 1143, 1116, 1094, 1018, 962, 811, 756, 499, 470 cm^{-1}

(((4-Methoxyphenyl)thio)methylene)bis((trifluoromethyl)sulfane) (6k)

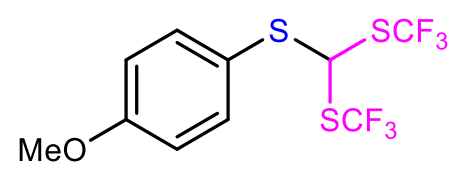

6k

The residue was purified by column chromatography on silica gel with an eluent of petroleum ether (R_f = 0.52) to afford product **6k** (120.2 mg, 68% yield) as a yellow oil.

^{1}H NMR (400 MHz, $CDCl_3$) δ 7.54 (d, *J* = 8.8 Hz, 2H), 6.94 (d, *J* = 8.8 Hz, 2H), 5.59 (s, 1H), 3.85 (s, 3H).

^{19}F NMR (376 MHz, $CDCl_3$) δ -41.34 (s, 6F).

^{13}C NMR (101 MHz, $CDCl_3$) δ 161.9, 137.9, 128.8 (q, *J* = 311.3 Hz), 120.6, 115.2, 55.6, 54.0 (sept, *J* = 2.1 Hz).

HRMS (FI) m/z: $[M]^+$ Calcd for $C_{10}H_8OF_6S_3$ 353.9636; Found 353.9640.

IR (KBr) 3011, 2963, 2943, 2841, 1592, 1571, 1495, 1464, 1442, 1408, 1291, 1254, 1139, 1113, 1094, 1032, 831, 799, 757, 644, 524, 472 cm^{-1}

(((4-Bromophenyl)thio)methylene)bis((trifluoromethyl)sulfane) (6l)

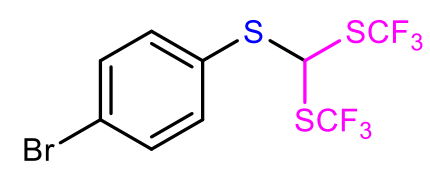

6l

The residue was purified by column chromatography on silica gel with an eluent of petroleum ether (R_f = 0.80) to afford product **6l** (114.6 mg, 57% yield) as a yellow oil.

^{1}H NMR (400 MHz, $CDCl_3$) δ 7.56 (d, J = 8.5 Hz, 2H), 7.45 (d, J = 8.4 Hz, 2H), 5.66 (s, 1H).

^{19}F NMR (376 MHz, $CDCl_3$) δ -41.33 (s, 6F).

^{13}C NMR (101 MHz, $CDCl_3$) δ 136.7, 133.0, 129.3, 128.6 (q, J = 311.5 Hz), 125.6, 53.1 (sept, J = 2.3 Hz).

HRMS (FI) m/z: $[M]^+$ Calcd for $C_9H_5BrF_6S_3$ 401.8635; Found 401.8638.

IR (KBr) 2960, 2926, 1566, 1474, 1387, 1301, 1143, 1115, 1093, 1070, 1010, 820, 757, 731, 509, 483, 470 cm^{-1}

4-((Bis((trifluoromethyl)thio)methyl)thio)benzonitrile (6m)

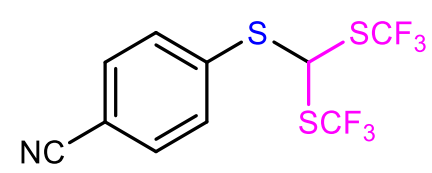

6m

The residue was purified by column chromatography on silica gel with an eluent of petroleum ether/EA (40:1, R_f = 0.48) to afford product **6m** (115.2 mg, 66% yield) as a yellow oil.

^{1}H NMR (400 MHz, $CDCl_3$) δ 7.73 – 7.68 (m, 2H), 7.66 – 7.61 (m, 2H), 5.79 (s, 1H).

^{19}F NMR (376 MHz, $CDCl_3$) δ -41.31 (s, 6F).

^{13}C NMR (101 MHz, $CDCl_3$) δ 137.1, 133.5, 133.2, 128.5 (q, J = 311.6 Hz), 118.0, 113.7, 52.0 (sept, J = 2.5 Hz).

HRMS (FI) m/z: $[M]^+$ Calcd for $C_{10}H_5NF_6S_3$ 348.9483; Found 348.9486.

IR (KBr) 3095, 2958, 2232, 1594, 1485, 1398, 1303, 1265, 1143, 1116, 1093, 1018, 835, 757, 550, 472 cm^{-1}

2-((Bis((trifluoromethyl)thio)methyl)thio)benzo[*d*]thiazole (6n)

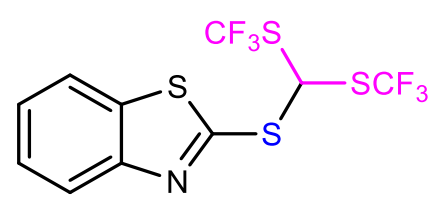

6n

The residue was purified by column chromatography on silica gel with an eluent of petroleum ether (R_f = 0.24) to afford product **6n** (106.7 mg, 56% yield) as a brown oil.

^{1}H NMR (400 MHz, $CDCl_3$) δ 8.02 (d, J = 8.2 Hz, 1H), 7.83 (d, J = 8.0 Hz, 1H), 7.51 (t, J = 7.7 Hz, 1H), 7.40 (t, J = 7.7 Hz, 1H), 6.59 (s, 1H).

^{19}F NMR (376 MHz, $CDCl_3$) δ -42.04 (s, 6F).

^{13}C NMR (101 MHz, $CDCl_3$) δ 160.1, 152.7, 135.9, 129.1 (q, J = 311.5 Hz), 126.8, 125.7, 122.8, 121.5, 49.9 (sept, J = 2.9 Hz).

HRMS (FI) m/z: $[M]^+$ Calcd for $C_{10}H_5NF_6S_4$ 380.9204; Found 380.9202.

IR (KBr) 3067, 2963, 2927, 1465, 1428, 1310, 1239, 1151, 1116, 1094, 1019, 997, 852, 776, 756, 726, 702, 473, 426 cm^{-1}

5-((Bis((trifluoromethyl)thio)methyl)thio)-1-phenyl-1*H*-tetrazole (6o)

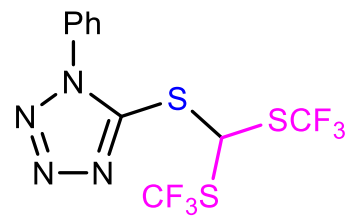

6o

The residue was purified by column chromatography on silica gel with an eluent of petroleum ether/EA (10:1, R_f = 0.51) to afford product **6o** (78.4 mg, 40% yield) as a yellow oil.

^{1}H NMR (400 MHz, $CDCl_3$) δ 7.64 – 7.57 (m, 3H), 7.56 – 7.46 (m, 2H), 6.52 (s, 1H).

^{19}F NMR (376 MHz, $CDCl_3$) δ -41.60 (s, 6F).

^{13}C NMR (101 MHz, $CDCl_3$) δ 150.1, 133.0, 131.1, 130.4, 128.6 (q, J = 311.9 Hz), 124.0, 50.0 (sept, J = 3.0 Hz).

HRMS (ESI) m/z: $[M+H]^+$ Calcd for $C_{10}H_7N_4F_6S_3$ 392.9732; Found 392.9732.

IR (KBr) 3069, 2962, 1596, 1500, 1463, 1417, 1390, 1279, 1245, 1153, 1118, 1094, 1015, 980, 915, 758, 688, 551, 504, 473 cm^{-1}

3-((Bis((trifluoromethyl)thio)methyl)thio)phenol (6p)

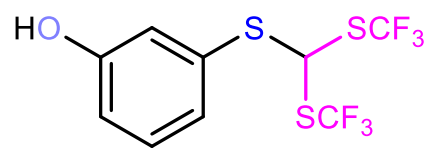

6p

The residue was purified by column chromatography on silica gel with an eluent of petroleum ether/EA (4:1, R_f = 0.44) to afford product **6p** (95.2 mg, 56% yield) as a yellow oil.

^{1}H NMR (400 MHz, $CDCl_3$) δ 7.30 (t, *J* = 7.9 Hz, 1H), 7.16 (ddt, *J* = 7.7, 1.7, 0.9 Hz, 1H), 7.10 – 7.06 (m, 1H), 6.94 (ddt, *J* = 8.2, 2.5, 0.9 Hz, 1H), 5.71 (s, 1H), 5.35 (s, 1H).

^{19}F NMR (376 MHz, $CDCl_3$) δ -41.35 (s, 6F).

^{13}C NMR (101 MHz, $CDCl_3$) δ 156.2, 131.7, 130.9, 128.7 (q, *J* = 311.5 Hz), 127.2, 121.4, 117.8, 53.2 (sept, *J* = 2.5 Hz).

HRMS (FI) m/z: $[M]^+$ Calcd for $C_9H_6OF_6S_3$ 339.9479; Found 339.9478.

IR (KBr) 3617, 3396, 3074, 2930, 2848,1585, 1475, 1438, 1299, 1249, 1165, 1093, 997, 885, 782, 757, 682, 532, 473, 454 cm^{-1}

(((3-(Bis((trifluoromethyl)thio)methoxy)phenyl)thio)methylene)bis((trifluoromethyl)sulfane) (6p′)

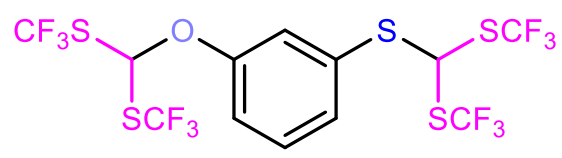

6p'

The residue was purified by column chromatography on silica gel with an eluent of petroleum ether (R_f = 0.67) to afford product **6p′** (166.2 mg, 60% yield) as a yellow oil.

^{1}H NMR (400 MHz, $CDCl_3$) δ 7.49 – 7.40 (m, 2H), 7.38 (t, *J* = 2.0 Hz, 1H), 7.23 (dt, *J* = 7.8, 2.2 Hz, 1H), 6.92 (s, 1H), 5.71 (s, 1H).

^{19}F NMR (376 MHz, $CDCl_3$) δ -39.97 (s, 6F), -41.56 (s, 6F).

^{13}C NMR (101 MHz, $CDCl_3$) δ 155.4, 132.5, 131.3, 131.2, 128.73 (q, *J* = 310.5 Hz), 128.67 (q, *J* = 311.5 Hz), 124.5, 120.7, 83.8 (sept, *J* = 3.0 Hz), 53.2 (sept, *J* = 2.5 Hz).

HRMS (FI) m/z: $[M]^+$ Calcd for $C_{12}H_6OF_{12}S_5$ 553.8825; Found 553.8832.

IR (KBr) 3084, 2972, 2933, 2860, 1578, 1473, 1417, 1302, 1273, 1211, 1147, 1118, 1093, 1019, 995, 882, 785, 758, 733, 682, 473 cm^{-1}

((Benzylthio)methylene)bis((trifluoromethyl)sulfane) (6q)

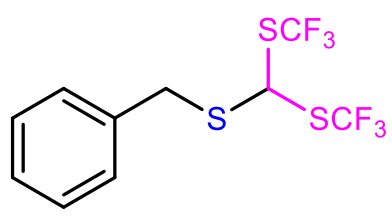

6q

The residue was purified by column chromatography on silica gel with an eluent of petroleum ether (R_f = 0.74), followed by HPLC purification (Material: 5μm C18, Dimensions: 20×250mm, CH_3CN/H_2O = 90:10, flow rate 16.0 mL/min, λ = 220 nm, 25 °C, t_R = 2.879 min) to afford product **6q** (20.4 mg, 12% yield) as a yellow oil.

^{1}H NMR (400 MHz, $CDCl_3$) δ 7.40 – 7.30 (m, 5H), 5.26 (s, 1H), 3.97 (s, 2H).

^{19}F NMR (376 MHz, $CDCl_3$) δ -41.34 (s, 6F).

^{13}C NMR (101 MHz, $CDCl_3$) δ 134.9, 129.3, 129.1, 128.6 (q, J = 311.4 Hz), 128.3, 48.8 (sept, J = 2.7 Hz), 38.0.

HRMS (FI) m/z: $[M]^+$ Calcd for $C_{10}H_8F_6S_3$ 337.9687; Found 337.9683.

IR (KBr) 3033, 2927, 2855, 1496, 1455, 1143, 1116, 1095, 967, 757, 698, 472 cm^{-1}

1-(Bis((trifluoromethyl)thio)methyl)-1*H*-benzo[*d*]imidazole (6r)

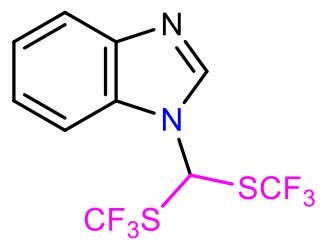

6r

The residue was purified by column chromatography on silica gel with an eluent of petroleum ether/EA (10:1, R_f = 0.22) to afford product **6r** (86.3 mg, 52% yield) as a yellow oil.

^{1}H NMR (400 MHz, $CDCl_3$) δ 8.17 (s, 1H), 7.88 (d, J = 7.1 Hz, 1H), 7.59 (d, J = 7.1 Hz, 1H), 7.47 – 7.35 (m, 2H), 7.27 (s, 1H).

^{19}F NMR (376 MHz, $CDCl_3$) δ -41.07 (s, 6F).

^{13}C NMR (101 MHz, $CDCl_3$) δ 144.8, 141.5, 131.7, 128.4 (q, J = 311.1 Hz), 124.6, 124.2, 121.6, 111.1, 58.4.

HRMS (FI) m/z: $[M]^+$ Calcd for $C_{10}H_6N_2F_6S_2$ 331.9871; Found 331.9876.

IR (KBr) 3088, 3068, 2930, 1615, 1501, 1454, 1345, 1272, 1173, 1144, 1116, 1095, 1012, 888, 783, 758, 742, 616, 473, 424 cm^{-1}

1-(Bis((trifluoromethyl)thio)methyl)-1*H*-benzo[*d*][1,2,3]triazole (6s)

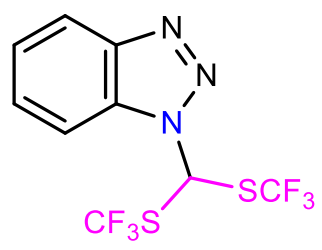

6s

The residue was purified by column chromatography on silica gel with an eluent of petroleum ether/EA (10:1, R_f = 0.50), followed by HPLC purification (Material: 5μm C18, Dimensions: 20×250mm, CH_3CN/H_2O = 80:20, flow rate 16.0 mL/min, λ = 220 nm, 25 °C, t_R = 5.554 min) to afford product **6s** (53.3 mg, 32% yield) as a colorless oil.

^{1}H NMR (400 MHz, $CDCl_3$) δ 8.15 (d, *J* = 8.4 Hz, 1H), 7.82 (s, 1H), 7.72 (d, *J* = 8.4 Hz, 1H), 7.63 (ddd, *J* = 8.3, 7.0, 1.0 Hz, 1H), 7.50 (ddd, *J* = 8.3, 7.0, 1.0 Hz, 1H).

^{19}F NMR (376 MHz, $CDCl_3$) δ -40.99 (s, 6F).

^{13}C NMR (101 MHz, $CDCl_3$) δ 147.2, 130.7, 129.2, 128.3 (q, *J* = 311.2 Hz), 125.5, 121.4, 110.5, 60.3 (sept, *J* = 3.3 Hz).

HRMS (FI) m/z: $[M]^+$ Calcd for $C_9H_5N_3F_6S_2$ 332.9824; Found 332.9828.

IR (KBr) 3070, 2934, 1614, 1594, 1492, 1452, 1389, 1326, 1291, 1270, 1237, 1146, 1119, 1094, 1032, 1005, 925, 799, 787, 758, 744, 655, 619, 475 cm^{-1}

1-(Bis((trifluoromethyl)thio)methyl)-4-methyl-2-phenyl-1*H*-imidazole (6t)

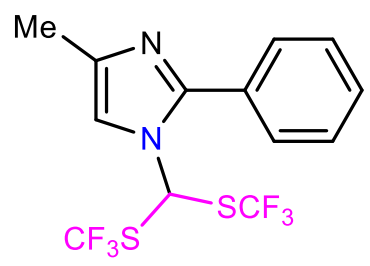

6t

The residue was purified by column chromatography on silica gel with an eluent of petroleum ether/EA (5:1, R_f = 0.30) to afford product **6t** (65.1 mg, 35% yield) as a yellow oil.

^{1}H NMR (400 MHz, $CDCl_3$) δ 7.56 – 7.47 (m, 5H), 7.02 (q, *J* = 1.2 Hz, 1H), 6.94 (s, 1H), 2.27 (d, *J* = 1.1 Hz, 3H).

^{19}F NMR (376 MHz, $CDCl_3$) δ -40.99 (s, 6F).

^{13}C NMR (101 MHz, $CDCl_3$) δ 147.6, 140.9, 130.3, 129.2, 129.0, 128.7, 128.2 (q, *J* = 311.2 Hz), 114.1, 58.9 (sept, *J* = 3.1 Hz), 14.0.

HRMS (FI) m/z: $[M]^+$ Calcd for $C_{13}H_{10}N_2F_6S_2$ 372.0184; Found 372.0187.

IR (KBr) 3033, 3074, 2990, 2958, 2980, 1576, 1475, 1447, 1385, 1371, 1284, 1170, 1142, 1115, 1096, 1043, 1021, 922, 815, 757, 699, 673, 634, 543, 472 cm^{-1}

2-(Bis((trifluoromethyl)thio)methyl)-5-phenyl-2*H*-tetrazole (6u)

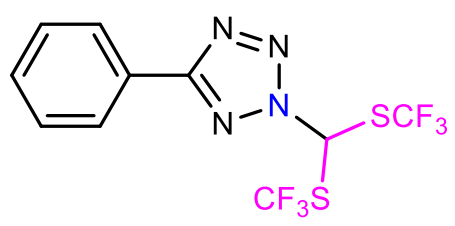

6u

The residue was purified by column chromatography on silica gel with an eluent of petroleum ether/EA (5:1, R_f = 0.34), followed by HPLC purification (Material: 5μm C18, Dimensions: 20×250mm, CH_3CN/H_2O = 80:20, flow rate 16.0 mL/min, λ = 220

nm, 25 °C, t_R = 5.554 min) to afford product **6u** (55.8 mg, 31% yield) as a yellow oil.

^{1}H NMR (400 MHz, $CDCl_3$) δ 7.73 – 7.67 (m, 1H), 7.67 – 7.59 (m, 4H), 7.15 (s, 1H).

^{19}F NMR (376 MHz, $CDCl_3$) δ -41.25 (s, 6F).

^{13}C NMR (101 MHz, $CDCl_3$) δ 154.1, 132.7, 130.0, 129.0, 128.1 (q, J = 311.5 Hz), 122.2, 58.4 (sept, J = 3.4 Hz).

HRMS (FI) m/z: $[M]^+$ Calcd for $C_{10}H_6N_4F_6S_2$ 359.9933; Found 359.9936.

IR (KBr) 3073, 2987, 2927, 1609, 1535, 1472, 1453, 1378, 1318, 1263, 1170, 1142, 1119, 1094, 1033, 1007, 972, 926, 781, 759, 731, 696, 679, 542, 483 cm^{-1}

(*Z*)-*N*-(1-(Bis((trifluoromethyl)thio)methyl)pyridin-2(1*H*)-ylidene)acetamide (6v)

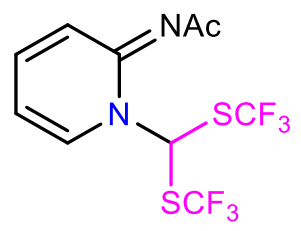

6v

The residue was purified by column chromatography on silica gel with an eluent of petroleum ether/EA (3:1, R_f = 0.42) to afford product **6v** (127.8 mg, 73% yield) as a white solid, m.p. 121-123 °C.

^{1}H NMR (400 MHz, $CDCl_3$) δ 8.26 (d, J = 9.3 Hz, 1H), 7.68 (d, J = 6.8 Hz, 1H), 7.56 (ddd, J = 9.1, 6.8, 1.6 Hz, 1H), 6.56 (t, J = 6.7 Hz, 1H), 6.16 (s, 1H), 2.24 (s, 3H).

^{19}F NMR (376 MHz, $CDCl_3$) δ -44.02 (s, 6F).

^{13}C NMR (101 MHz, $CDCl_3$) δ 179.9, 154.1, 141.2, 134.6, 130.6 (q, J = 311.6 Hz), 120.4, 111.2, 67.5 (sept, J = 3.8 Hz), 28.4.

HRMS (ESI) m/z: $[M+H]^+$ Calcd for $C_{10}H_9ON_2F_6S_2$ 351.0055; Found 351.0055.

IR (KBr) 3152, 3081, 3040, 2970, 1644, 1627, 1613, 1547, 1505, 1453, 1385, 1355, 1292, 1257, 1149, 1123, 1096, 1034, 1011, 950, 909, 866, 853, 781, 769, 744, 725, 699, 606, 560, 545, 472 cm^{-1}

The procedure for Deuterium-labeling experiment of $BrCD(SCF_3)_2$ with the sodium salt of substrate 5r

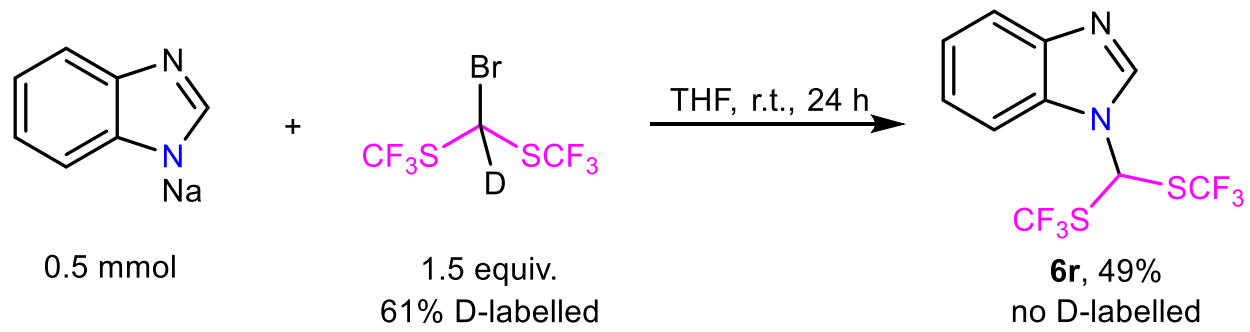

A 25 mL bottom flask was charged with sodium salt of substrate **5r** (70 mg, 0.5 mmol, 1.0 equiv.) and dry THF (7.5 mL) under a N_2 atmosphere. $BrCD(SCF_3)_2$ (221.8 mg, 0.75 mmol, 1.5 equiv.) was then added. The resulting mixture was stirred at room temperature for 24 h under a N_2 atmosphere. The resulting mixture was then diluted with ethyl acetate (20 mL) and saturated NH_4Cl solution (30 mL). The organic phase was separated and the aqueous phase was extracted 3 times. All organic solutions were combined and then dried over anhydrous Na_2SO_4. The solvent was removed under vacuum and the residue was subjected to column chromatography on silica gel with an eluent of petroleum ether/EA (10:1, R_f = 0.22) to afford products **6r** (81.5 mg, 49% yield) as a yellow oil.

The procedure for [2,3]-σ rearrangement (Doyle−Kirmse reaction) of sulfur ylide from allyl sulfides with :C(SCF₃)₂ to obtain products 8a-8w

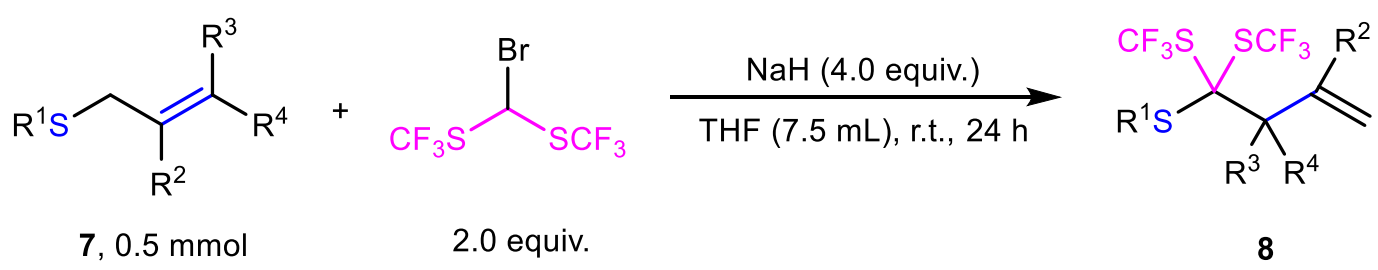

A 25 mL bottom flask was charged with allyl sulfide **7** (0.5 mmol, 1.0 equiv.), NaH (48.0 mg, 2.0 mmol, 4.0 equiv.) and dry THF (7.5 mL) under a N_2 atmosphere. $BrCH(SCF_3)_2$ (295.0 mg, 1.0 mmol, 2.0 equiv.) was then added. The resulting mixture was stirred at room temperature for 24 h under a N_2 atmosphere. The resulting mixture was then diluted with ethyl acetate (20 mL) and saturated NH_4Cl solution (30 mL). The organic phase was separated and the aqueous phase was extracted 3 times. All organic solutions were combined and then dried over anhydrous Na_2SO_4. The solvent was removed under vacuum and the residue was subjected to column chromatography to give final products **8a-8p**, **8r-8v**. For the substrate **7q**, the reagent loadings are shown as follows: $BrCH(SCF_3)_2$ (737.5 mg, 2.5 mmol, 5.0 equiv.), NaH (120.0 mg, 5.0 mmol, 10.0 equiv.) were used in the reactions to afford products **8q** and **8q′**. For the substrate **7w**, the reagent loadings are shown as follows: $BrCH(SCF_3)_2$ (590.0 mg, 2.0 mmol,

4.0 equiv.), NaH (96.0 mg, 4.0 mmol, 8.0 equiv.) were used in the reactions to afford product **8w**.

(1-(Phenylthio)but-3-ene-1,1-diyl)bis((trifluoromethyl)sulfane) (8a)

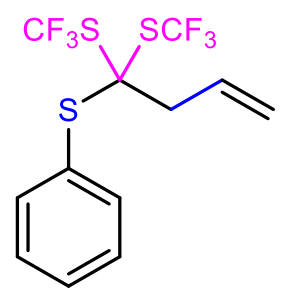

8a

The residue was purified by column chromatography on silica gel with an eluent of petroleum ether (R_f = 0.83) to afford product **8a** (156.5 mg, 86% yield) as a yellow oil.

^{1}H NMR (400 MHz, $CDCl_3$) δ 7.67 (d, *J* = 7.5 Hz, 2H), 7.56 – 7.49 (m, 1H), 7.44 (t, *J* = 7.5 Hz, 2H), 6.15 (ddt, *J* = 16.9, 10.2, 6.7 Hz, 1H), 5.41 – 5.28 (m, 2H), 3.23 (d, *J* = 6.7 Hz, 2H).

^{19}F NMR (376 MHz, $CDCl_3$) δ -37.12 (s, 6F).

^{13}C NMR (101 MHz, $CDCl_3$) δ 138.1, 131.2, 130.5, 129.3, 128.70, 128.69 (q, *J* = 313.1 Hz), 121.5, 71.6, 46.3.

HRMS (FI) m/z: $[M]^+$ Calcd for $C_{12}H_{10}F_6S_3$ 363.9843; Found 363.9841.

IR (KBr) 3083, 3064, 2928, 2857, 1640, 1474, 1440, 1308, 1139, 1111, 1096, 1025, 988, 948, 928, 800, 753, 704, 690, 498, 468 cm^{-1}

(1-((4-(*tert*-Butyl)phenyl)thio)but-3-ene-1,1-diyl)bis((trifluoromethyl)sulfane) (8b)

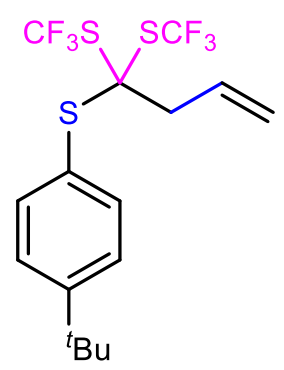

8b

The residue was purified by column chromatography on silica gel with an eluent of petroleum ether (R_f = 0.77), followed by HPLC purification (Material: 5μm C18, Dimensions: 20×250mm, CH_3CN, flow rate 20.0 mL/min, λ = 220 nm, 25 °C, t_R = 7.317 min) to afford product **8b** (149.1 mg, 71% yield) as a yellow oil.

^{1}H NMR (400 MHz, $CDCl_3$) δ 7.55 (d, *J* = 8.4 Hz, 2H), 7.43 (d, *J* = 8.5 Hz, 2H), 6.12 (ddt, *J* = 16.9, 10.0, 6.7 Hz, 1H), 5.37 – 5.26 (m, 2H), 3.19 (d, *J* = 6.8 Hz, 2H), 1.35 (s, 9H).

^{19}F NMR (376 MHz, $CDCl_3$) δ -37.08 (s, 6F).

^{13}C NMR (101 MHz, $CDCl_3$) δ 154.7, 137.9, 130.6, 128.7 (q, J = 313.3 Hz), 126.4, 125.1, 121.3, 71.6, 46.2, 35.1, 31.4.

HRMS (FI) m/z: $[M]^+$ Calcd for $C_{16}H_{18}F_6S_3$ 420.0469; Found 420.0471.

IR (KBr) 3084, 2966, 2905, 2871, 1594, 1489, 1462, 1435, 1399, 1365, 1293, 1268, 1245, 1157, 1139, 1111, 1096, 1014, 989, 948, 927, 833, 754, 560, 474, 436 cm^{-1}

(1-([1,1'-Biphenyl]-4-ylthio)but-3-ene-1,1-diyl)bis((trifluoromethyl)sulfane) (8c)

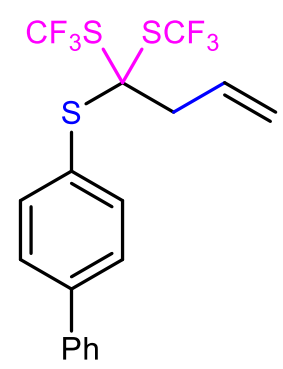

8c

The residue was purified by column chromatography on silica gel with an eluent of petroleum ether (R_f = 0.41) to afford product **8c** (198 mg, 90% yield) as a brown oil.

^{1}H NMR (400 MHz, $CDCl_3$) δ 7.78 – 7.73 (m, 2H), 7.71 – 7.64 (m, 4H), 7.55 – 7.49 (m, 2H), 7.48 – 7.42 (m, 1H), 6.22 (ddt, J = 16.9, 10.2, 6.7 Hz, 1H), 5.47 – 5.35 (m, 2H), 3.32 (d, J = 6.7 Hz, 2H).

^{19}F NMR (376 MHz, $CDCl_3$) δ -36.97 (s, 6F).

^{13}C NMR (101 MHz, $CDCl_3$) δ 144.0, 139.9, 138.5, 130.5, 129.2, 128.7 (q, J = 313.3 Hz), 128.4, 127.9, 127.41, 127.37, 121.5, 71.8, 46.3.

HRMS (FI) m/z: $[M]^+$ Calcd for $C_{18}H_{14}F_6S_3$ 440.0156; Found 440.0163.

IR (KBr) 3068, 3084, 3030, 2986, 1639, 1593, 1478, 1448, 1435, 1417, 1398, 1312, 1293, 1242, 1162, 1140, 1108, 1094, 1019, 1008, 988, 947, 928, 838, 759, 722, 696, 661, 645, 555, 504, 472 cm^{-1}

(1-(Naphthalen-2-ylthio)but-3-ene-1,1-diyl)bis((trifluoromethyl)sulfane) (8d)

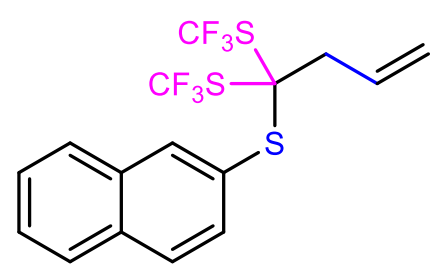

8d

The residue was purified by column chromatography on silica gel with an eluent of petroleum ether (R_f = 0.67), followed by HPLC purification (Material: 5μm C18, Dimensions: 20×250mm, CH_3CN, flow rate 16.0 mL/min, λ = 220 nm, 25 °C, t_R = 6.536

min) to afford product **8d** (153.2 mg, 74% yield) as a brown oil.

^{1}H NMR (400 MHz, $CDCl_3$) δ 8.22 (s, 1H), 7.96 – 7.87 (m, 3H), 7.72 (dd, J = 8.6, 1.9 Hz, 1H), 7.67 – 7.55 (m, 2H), 6.22 (ddt, J = 16.9, 10.2, 6.7 Hz, 1H), 5.52 – 5.30 (m, 2H), 3.29 (d, J = 6.7 Hz, 2H).

^{19}F NMR (376 MHz, $CDCl_3$) δ -36.97 (s, 6F).

^{13}C NMR (101 MHz, $CDCl_3$) δ 138.9, 134.2, 133.5, 133.4, 130.5, 128.9, 128.7 (q, J = 313.3 Hz), 128.6, 128.2, 128.0, 127.1, 125.9, 121.5, 71.9, 46.4.

HRMS (EI) m/z: $[M-HSCF_3]^+$ Calcd for $C_{15}H_{11}F_3S_2$ 312.0249; Found 312.0246.

IR (KBr) 3090, 3058, 2986, 2927, 1860, 1639, 1626, 1585, 1500, 1434, 1417, 1352, 1339, 1293, 1268, 1239, 1162, 1137, 1109, 1096, 1017, 988, 944, 928, 898, 859, 815, 754, 744, 677, 640, 477 cm^{-1}

(1-((4-Methoxyphenyl)thio)but-3-ene-1,1-diyl)bis((trifluoromethyl)sulfane) (8e)

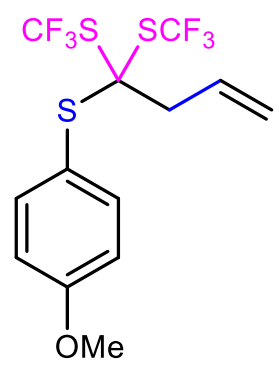

8e

The residue was purified by column chromatography on silica gel with an eluent of petroleum ether (R_f = 0.29) to afford product **8e** (171.4 mg, 87% yield) as a brown oil (a little amount of impurity was contained in the product).

^{1}H NMR (400 MHz, $CDCl_3$) δ 7.56 (d, J = 8.8 Hz, 2H), 6.94 (d, J = 8.8 Hz, 2H), 6.13 (ddt, J = 16.9, 10.3, 6.7 Hz, 1H), 5.39 – 5.28 (m, 2H), 3.85 (s, 3H), 3.21 (d, J = 6.7 Hz, 2H).

^{19}F NMR (376 MHz, $CDCl_3$) δ -37.10 (s, 6F).

^{13}C NMR (101 MHz, $CDCl_3$) δ 162.2, 139.9, 130.6, 128.7 (q, J = 313.2 Hz), 121.3, 119.3, 114.8, 72.0, 55.5, 46.0.

HRMS (FI) m/z: $[M]^+$ Calcd for $C_{13}H_{12}OF_6S_3$ 393.9949; Found 393.9952.

IR (KBr) 3086, 3015, 2967, 2942, 2841, 1639, 1592, 1570, 1494, 1464, 1442, 1291, 1254, 1177, 1139, 1105, 1096, 1032, 989, 949, 928, 831, 800, 755, 647, 531, 472 cm^{-1}

(1-((4-(Methylthio)phenyl)thio)but-3-ene-1,1-diyl)bis((trifluoromethyl)sulfane) (8f)

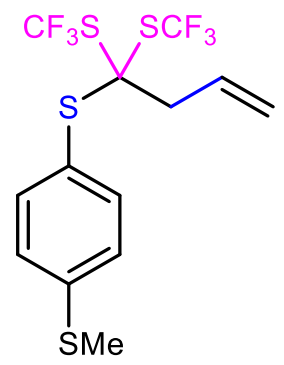

8f

The residue was purified by column chromatography on silica gel with an eluent of petroleum ether (R_f = 0.47), followed by HPLC purification (Material: 5μm C18, Dimensions: 20×250mm, CH_3CN/H_2O = 90:10, flow rate 20.0 mL/min, λ = 220 nm, 25 ºC, t_R = 10.766 min) to afford product **8f** (133.3 mg, 65% yield) as a yellow oil.

^{1}H NMR (400 MHz, $CDCl_3$) δ 7.53 – 7.47 (m, 2H), 7.27 – 7.20 (m, 2H), 6.10 (ddt, *J* = 16.9, 10.2, 6.7 Hz, 1H), 5.37 – 5.27 (m, 2H), 3.18 (d, *J* = 6.7 Hz, 2H), 2.50 (s, 3H).

^{19}F NMR (376 MHz, $CDCl_3$) δ -37.06 (s, 6F).

^{13}C NMR (101 MHz, $CDCl_3$) δ 143.7, 138.3, 130.5, 128.6 (q, *J* = 313.3 Hz), 126.0, 124.0, 121.5, 71.8, 46.1, 15.1.

HRMS (FI) m/z: $[M]^+$ Calcd for $C_{13}H_{12}F_6S_4$ 409.9721; Found 409.9725.

IR (KBr) 3084, 2986, 2924, 2854, 1640, 1577, 1477, 1437, 1392, 1321, 1294, 1243, 1156, 1138, 1096, 1013, 989, 968, 949, 929, 815, 755, 675, 646, 502, 470, 437 cm^{-1}

(1-((4-Chlorophenyl)thio)but-3-ene-1,1-diyl)bis((trifluoromethyl)sulfane) (8g)

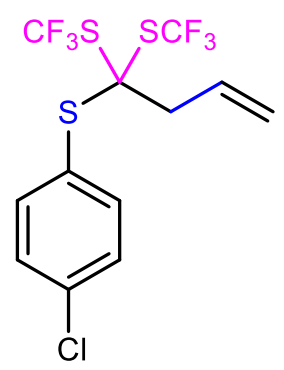

8g

The residue was purified by column chromatography on silica gel with an eluent of petroleum ether (R_f = 0.79) to afford product **8g** (189.1 mg, 95% yield) as a brown oil.

^{1}H NMR (400 MHz, $CDCl_3$) δ 7.60 – 7.53 (m, 2H), 7.44 – 7.38 (m, 2H), 6.10 (ddt, *J* = 16.9, 10.2, 6.7 Hz, 1H), 5.40 – 5.27 (m, 2H), 3.22 (d, *J* = 6.7 Hz, 2H).

^{19}F NMR (376 MHz, $CDCl_3$) δ -37.12 (s, 6F).

^{13}C NMR (101 MHz, $CDCl_3$) δ 139.3, 138.0, 130.3, 129.6, 128.6 (q, J = 313.3 Hz). 127.1, 121.7, 71.6, 46.3.

HRMS (FI) m/z: $[M]^+$ Calcd for $C_{12}H_9ClF_6S_3$ 397.9454; Found 397.9449.

IR (KBr) 3086, 2987, 1640, 1574, 1476, 1436, 1390, 1311, 1294, 1263, 1242, 1146, 1109, 1095, 1015, 988, 949, 929, 826, 755, 706, 674, 646, 555, 503, 469 cm^{-1}

(1-((4-Bromophenyl)thio)but-3-ene-1,1-diyl)bis((trifluoromethyl)sulfane) (8h)

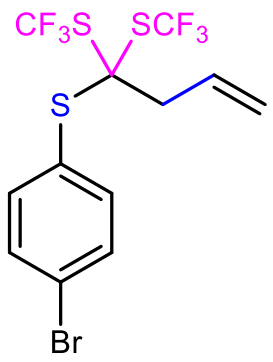

8h

The residue was purified by column chromatography on silica gel with an eluent of petroleum ether (R_f = 0.82) to afford product **8h** (214.4 mg, 97% yield) as a brown oil.

^{1}H NMR (400 MHz, $CDCl_3$) δ 7.57 (d, J = 8.5 Hz, 2H), 7.49 (d, J = 8.5 Hz, 2H), 6.10 (ddt, J = 17.0, 10.2, 6.7 Hz, 1H), 5.40 – 5.29 (m, 2H), 3.22 (d, J = 6.7 Hz, 2H).

^{19}F NMR (376 MHz, $CDCl_3$) δ -37.09 (s, 6F).

^{13}C NMR (101 MHz, $CDCl_3$) δ 139.4, 132.6, 130.2, 128.6 (q, J = 313.4 Hz), 127.7, 126.4, 121.7, 71.5, 46.3.

HRMS (FI) m/z: $[M]^+$ Calcd for $C_{12}H_9BrF_6S_3$ 441.8948; Found 441.8952.

IR (KBr) 3086, 2989, 1640, 1567, 1473, 1435, 1387, 1294, 1143, 1093, 1071, 1011, 988, 949, 929, 821, 755, 731, 646, 510, 467 cm^{-1}

(1-((4-(Trifluoromethyl)phenyl)thio)but-3-ene-1,1-diyl)bis((trifluoromethyl)sulfane) (8i)

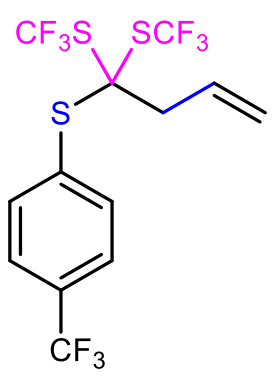

8i

The residue was purified by column chromatography on silica gel with an eluent of petroleum ether (R_f = 0.81) to afford product **8i** (213.8 mg, 99% yield) as a brown oil.

^{1}H NMR (400 MHz, $CDCl_3$) δ 7.78 (d, *J* = 8.1 Hz, 2H), 7.69 (d, *J* = 8.1 Hz, 2H), 6.11 (ddt, *J* = 16.9, 10.2, 6.7 Hz, 1H), 5.41 – 5.29 (m, 2H), 3.25 (d, *J* = 6.7 Hz, 2H).

^{19}F NMR (376 MHz, $CDCl_3$) δ -37.25 (s, 6F), -63.22 (s, 3F).

^{13}C NMR (101 MHz, $CDCl_3$) δ 138.1, 133.3 (q, *J* = 1.4 Hz), 133.1 (q, *J* = 32.9 Hz), 130.1, 128.6 (q, *J* = 314.4 Hz), 126.1 (q, *J* = 3.7 Hz), 123.9 (q, *J* = 272.5 Hz), 121.9, 71.6, 46.5.

HRMS (FI) m/z: $[M]^+$ Calcd for $C_{13}H_9F_9S_3$ 431.9717; Found 431.9712.

IR (KBr) 1608, 1436, 1399, 1325, 1138, 1107, 1094, 1063, 1017, 988, 948, 930, 840, 755, 704, 646, 599, 473 cm^{-1}

4-((1,1-Bis((trifluoromethyl)thio)but-3-en-1-yl)thio)benzonitrile (8j)

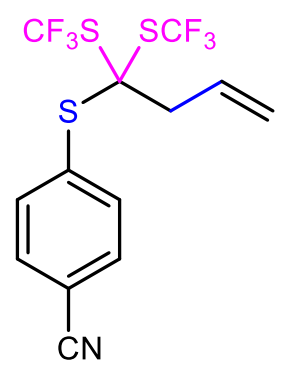

8j

The residue was purified by column chromatography on silica gel with an eluent of petroleum ether/EA (40:1, R_f = 0.33) to afford product **8j** (180.9 mg, 93% yield) as a brown oil.

^{1}H NMR (400 MHz, $CDCl_3$) δ 7.74 (d, *J* = 8.5 Hz, 2H), 7.70 (d, *J* = 8.4 Hz, 2H), 6.05 (ddt, *J* = 16.9, 10.2, 6.7 Hz, 1H), 5.38 – 5.28 (m, 2H), 3.22 (d, *J* = 6.8 Hz, 2H).

^{19}F NMR (376 MHz, $CDCl_3$) δ -37.14 (s, 6F).

^{13}C NMR (101 MHz, $CDCl_3$) δ 137.9, 134.8, 132.6, 129.8, 128.3 (q, *J* = 313.5 Hz), 122.1, 117.9, 114.8, 71.5, 46.5.

HRMS (FI) m/z: $[M]^+$ Calcd for $C_{13}H_9NF_6S_3$ 388.9796; Found 388.9797.

IR (KBr) 3088, 2987, 2232, 1640, 1593, 1485, 1435, 1418, 1397, 1295, 1269, 1242, 1151, 1096, 1019, 989, 948, 931, 836, 799, 755, 674, 647, 590, 551, 475, 456 cm^{-1}

(1-((4-Nitrophenyl)thio)but-3-ene-1,1-diyl)bis((trifluoromethyl)sulfane) (8k)

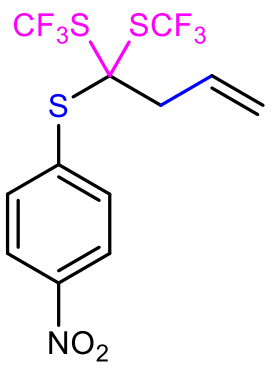

8k

The residue was purified by column chromatography on silica gel with an eluent of petroleum ether/EA (40:1, R_f = 0.44) to afford product **8k** (196.3 mg, 96% yield) as a brown oil.

^{1}H NMR (400 MHz, $CDCl_3$) δ 8.30 – 8.23 (m, 2H), 7.85 – 7.79 (m, 2H), 6.07 (ddt, *J* = 16.9, 10.2, 6.7 Hz, 1H), 5.42 – 5.30 (m, 2H), 3.25 (d, *J* = 6.8 Hz, 2H).

^{19}F NMR (376 MHz, $CDCl_3$) δ -37.10 (s, 6F).

^{13}C NMR (101 MHz, $CDCl_3$) δ 149.3, 138.1, 136.9, 129.8, 128.3 (q, *J* = 313.5 Hz), 124.0, 122.3, 71.6, 46.6.

HRMS (FI) m/z: $[M]^+$ Calcd for $C_{12}H_9O_2NF_6S_3$ 408.9694; Found 408.9701.

IR (KBr) 3100, 2987, 2932, 2859, 1640, 1600, 1580, 1526, 1477, 1435, 1418, 1395, 1346, 1305, 1280, 1242, 1151, 1113, 1093, 1015, 989, 948, 932, 853, 800, 755, 744, 728, 686, 647, 543, 466 cm^{-1}

Ethyl 2-methylene-4-(phenylthio)-4,4-bis((trifluoromethyl)thio)butanoate (8l)

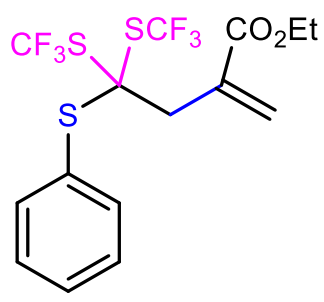

8l

The residue was purified by column chromatography on silica gel with an eluent of petroleum ether/EA (40:1, R_f = 0.38) to afford product **8l** (198.4 mg, 91% yield) as a brown oil.

^{1}H NMR (400 MHz, $CDCl_3$) δ 7.63 (d, *J* = 7.6 Hz, 2H), 7.49 (t, *J* = 7.4 Hz, 1H), 7.41 (t, *J* = 7.5 Hz, 2H), 6.58 (s, 1H), 6.05 (s, 1H), 4.27 (q, *J* = 7.1 Hz, 2H), 3.63 (s, 2H), 1.33 (t, *J* = 7.2 Hz, 3H).

^{19}F NMR (376 MHz, $CDCl_3$) δ -36.69 (s, 6F).

^{13}C NMR (101 MHz, $CDCl_3$) δ 166.9, 138.2, 134.0, 132.2, 131.2, 129.2, 128.6 (q, *J* = 313.7 Hz), 128.3, 72.4, 61.6, 42.1, 14.2.

HRMS (FI) m/z: $[M]^+$ Calcd for $C_{15}H_{14}O_2F_6S_3$ 436.0055; Found 436.0051.

IR (KBr) 3063, 2985, 2941, 2910, 1724, 1631, 1474, 1440, 1417, 1370, 1333, 1300, 1231, 1140, 1109, 1094, 1025, 962, 861, 804, 753, 705, 690, 499, 468 cm^{-1}

(2,2-Difluoro-1-(phenylthio)but-3-ene-1,1-diyl)bis((trifluoromethyl)sulfane) (8m)

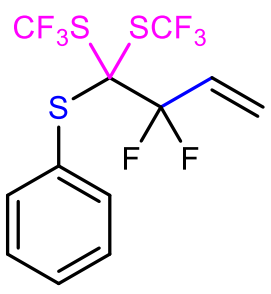

8m

The residue was purified by column chromatography on silica gel with an eluent of petroleum ether (R_f = 0.52) to afford product **8m** (146.1 mg, 73% yield) as a yellow oil.

^{1}H NMR (400 MHz, $CDCl_3$) δ 7.72 – 7.66 (m, 2H), 7.58 – 7.51 (m, 1H), 7.49 – 7.41 (m, 2H), 6.42 (dq, J = 17.1, 11.3 Hz, 1H), 5.98 (dt, J = 17.1, 2.3 Hz, 1H), 5.78 (d, J = 11.0 Hz, 1H).

^{19}F NMR (376 MHz, $CDCl_3$) δ -37.15 (t, J = 9.3 Hz, 6F), -92.59 – -92.80 (m, 2F).

^{13}C NMR (101 MHz, $CDCl_3$) δ 138.7, 131.8, 129.3, 128.2 (t, J = 25.1 Hz), 127.8 (q, J = 314.5 Hz), 126.7, 124.7 (t, J = 8.9 Hz), 118.8 (t, J = 254.0 Hz), 75.5 (t, J = 33.0 Hz).

HRMS (FI) m/z: $[M]^+$ Calcd for $C_{12}H_8F_8S_3$ 399.9655; Found 399.9661.

IR (KBr) 3064, 1645, 1576, 1474, 1442, 1418, 1308, 1220, 1158, 1103, 1084, 1025, 1006, 976, 961, 866, 818, 757, 746, 715, 704, 689, 543, 500, 467 cm^{-1}

(2-Methyl-1-(phenylthio)but-3-ene-1,1-diyl)bis((trifluoromethyl)sulfane) (8n)

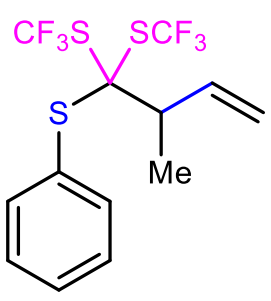

8n

The residue was purified by column chromatography on silica gel with an eluent of petroleum ether (R_f = 0.73), followed by HPLC purification (Material: 5μm C18, Dimensions: 20×250mm, CH_3CN, flow rate 16.0 mL/min, λ = 220 nm, 25 °C, t_R = 5.670 min) to afford product **8n** (132.3 mg, 70% yield) as a yellow oil.

^{1}H NMR (400 MHz, $CDCl_3$) δ 7.73 – 7.67 (m, 2H), 7.53 – 7.45 (m, 1H), 7.46 – 7.37 (m, 2H), 6.08 (ddd, J = 16.4, 10.6, 8.4 Hz, 1H), 5.30 – 5.21 (m, 2H), 3.14 (quin, J = 7.0 Hz, 1H), 1.42 (d, J = 6.7 Hz, 3H).

^{19}F NMR (376 MHz, $CDCl_3$) δ -36.51 (s, 3F), -36.59 (s, 3F).

^{13}C NMR (101 MHz, $CDCl_3$) δ 138.1, 136.6, 131.0, 129.3, 129.2, 128.7 (q, J = 312.8 Hz), 128.5 (q, J = 314.1 Hz), 119.3, 77.8, 50.2, 17.9.

HRMS (FI) m/z: $[M]^+$ Calcd for $C_{13}H_{12}F_6S_3$ 378.0000; Found 378.0001.

IR (KBr) 3082, 3066, 2985, 2939, 1706, 1637, 1583, 1576, 1474, 1455, 1441, 1420, 1377, 1307, 1140, 1105, 1025, 991, 928, 848, 747, 704, 689, 543, 499, 468 cm^{-1}

(2,2-Dimethyl-1-(phenylthio)but-3-ene-1,1-diyl)bis((trifluoromethyl)sulfane) (8o)

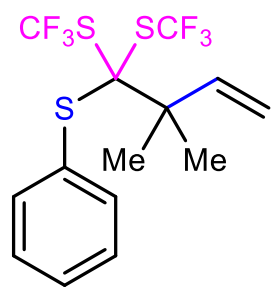

8o

The residue was purified by column chromatography on silica gel with an eluent of petroleum ether (R_f = 0.70), followed by HPLC purification (Material: 5μm C18, Dimensions: 20×250mm, CH_3CN, flow rate 16.0 mL/min, λ = 220 nm, 25 °C, t_R = 6.113 min) to afford product **8o** (123.5 mg, 63% yield) as a yellow oil.

^{1}H NMR (400 MHz, $CDCl_3$) δ 7.71 (d, J = 7.7 Hz, 2H), 7.51 – 7.44 (m, 1H), 7.44 – 7.35 (m, 2H), 6.30 (dd, J = 17.2, 10.8 Hz, 1H), 5.28 – 5.20 (m, 2H), 1.51 (s, 6H).

^{19}F NMR (376 MHz, $CDCl_3$) δ -35.44 (s, 6F).

^{13}C NMR (101 MHz, $CDCl_3$) δ 141.6, 138.5, 130.9, 130.1, 128.9, 128.7 (q, J = 313.3 Hz), 116.3, 82.9, 51.1, 25.0.

HRMS (FI) m/z: $[M]^+$ Calcd for $C_{14}H_{14}F_6S_3$ 392.0156; Found 392.0154.

IR (KBr) 3086, 3064, 2984, 2945, 2879, 1697, 1635, 1575, 1473, 1440, 1415, 1384, 1367, 1307, 1146, 1097, 1025, 1003, 926, 799, 756, 744, 704, 688, 543, 500, 476 cm^{-1}

(2-Phenyl-1-(phenylthio)but-3-ene-1,1-diyl)bis((trifluoromethyl)sulfane) (8p)

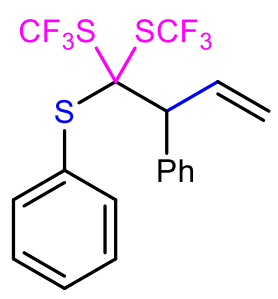

8p

The residue was purified by column chromatography on silica gel with an eluent of petroleum ether (R_f = 0.40), followed by HPLC purification (Material: 5μm C18, Dimensions: 20×250mm, CH_3CN, flow rate 16.0 mL/min, λ = 220 nm, 25 °C, t_R = 5.811

min) to afford product **8p** (154.1 mg, 70% yield) as a yellow oil.

^{1}H NMR (400 MHz, $CDCl_3$) δ 7.65 – 7.54 (m, 4H), 7.50 – 7.44 (m, 1H), 7.43 – 7.35 (m, 5H), 6.55 (ddd, *J* = 17.1, 10.4, 6.7 Hz, 1H), 5.33 (dt, *J* = 10.4, 1.3 Hz, 1H), 5.06 (dt, *J* = 17.0, 1.4 Hz, 1H), 4.35 (dt, *J* = 6.7, 1.6 Hz, 1H).

^{19}F NMR (376 MHz, $CDCl_3$) δ -36.22 (q, *J* = 2.0 Hz, 3F), -36.25 (q, *J* = 2.0 Hz, 3F).

^{13}C NMR (101 MHz, $CDCl_3$) δ 138.1, 136.9, 135.2, 131.5, 131.0, 129.2, 129.0, 128.9, 128.6 (q, *J* = 312.9 Hz), 128.5, 128.4 (q, *J* = 315.1 Hz), 120.4, 76.9, 60.2.

HRMS (EI) m/z: $[M-HSCF_3]^+$ Calcd for $C_{17}H_{13}F_3S_2$ 338.0405; Found 338.0399.

IR (KBr) 3090, 3063, 3031, 1494, 1474, 1455, 1440, 1417, 1307, 1269, 1139, 1101, 1091, 1025, 1002, 990, 929, 849, 796, 755, 722, 699, 689, 503, 473 cm^{-1}

(3-Phenyl-1-(phenylthio)but-3-ene-1,1-diyl)bis((trifluoromethyl)sulfane) (8q)

(2-(1-Phenyl-2,2-bis((trifluoromethyl)thio)cyclopropyl)-1-(phenylthio)ethane-1,1-diyl)bis((trifluoromethyl)sulfane) (8q')

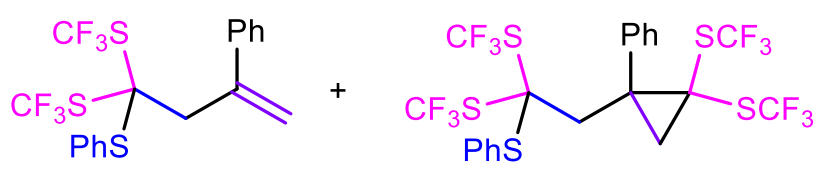

The residue was purified by column chromatography on silica gel with an eluent of petroleum ether (R_f = 0.42), followed by HPLC purification (Material: 5μm C18, Dimensions: 20×250mm, CH_3CN, flow rate 20.0 mL/min, λ = 220 nm, 25 °C) to afford product **8q** (57.2 mg, 26% yield, t_R = 5.691 min) and **8q'** (156.7 mg, 48% yield, t_R = 6.939 min) as yellow oils.

For the product **8q**:

^{1}H NMR (400 MHz, $CDCl_3$) δ 7.56 (d, *J* = 7.6 Hz, 2H), 7.48 (t, *J* = 7.3 Hz, 1H), 7.44 – 7.28 (m, 7H), 5.59 (s, 2H), 3.70 (s, 2H).

^{19}F NMR (376 MHz, $CDCl_3$) δ -36.81 (s, 6F).

^{13}C NMR (101 MHz, $CDCl_3$) δ 142.2, 142.1, 138.0, 131.0, 129.3, 129.1, 128.6 (q, *J* = 313.8 Hz), 128.5, 128.0, 127.3, 122.3, 72.6, 46.9.

HRMS (FI) m/z: $[M]^+$ Calcd for $C_{18}H_{14}F_6S_3$ 440.0156; Found 440.0159.

IR (KBr) 3086, 3059, 3024, 2925, 1494, 1474, 1440, 1309, 1227, 1136, 1111, 1095, 1026, 957, 912, 774, 752, 703, 690, 502, 472, 424 cm^{-1}

For the product **8q'**:

^{1}H NMR (400 MHz, $CDCl_3$) δ 7.56 – 7.51 (m, 2H), 7.50 – 7.46 (m, 1H), 7.45 – 7.31 (m, 7H), 3.66 (d, *J* = 15.3 Hz, 1H), 3.25 (d, *J* = 15.3 Hz, 1H), 2.64 (d, *J* = 7.6 Hz, 1H), 2.25 (d, *J* = 7.6 Hz, 1H).

^{19}F NMR (376 MHz, $CDCl_3$) δ -36.62 (s, 3F), -36.80 (s, 3F), -39.80 (s, 3F), -39.86 (s, 3F).

^{13}C NMR (101 MHz, $CDCl_3$) δ 138.1, 137.4, 131.2, 130.7, 130.2 (q, *J* = 311.1 Hz), 129.58, 129.57 (q, *J* = 311.4 Hz), 129.3, 129.23, 129.19, 129.1, 128.7, 128.5 (q, *J* = 314.0 Hz), 128.3 (q, *J* = 313.9 Hz), 73.2, 48.5, 40.4, 37.1 (sept, *J* = 1.9 Hz), 31.2.

HRMS (FI) m/z: $[M]^+$ Calcd for $C_{21}H_{14}F_{12}S_5$ 653.9502; Found 653.9498.

IR (KBr) 3066, 3034, 2937, 2853, 1602, 1584, 1499, 1474, 1450, 1441, 1428, 1326, 1308, 1295, 1157, 1112, 1057, 1025, 1016, 994, 924, 884, 850, 804, 755, 699, 572, 542, 502, 473, 425 cm^{-1}

2-((1,1-Bis((trifluoromethyl)thio)but-3-en-1-yl)thio)thiophene (8r)

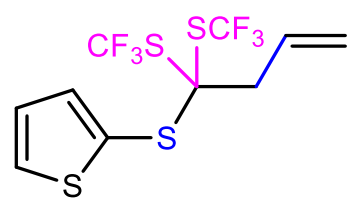

8r

The residue was purified by column chromatography on silica gel with an eluent of petroleum ether (R_f = 0.64) to afford product **8r** (175.8 mg, 95% yield) as a brown oil.

^{1}H NMR (400 MHz, $CDCl_3$) δ 7.63 (dd, *J* = 5.4, 1.3 Hz, 1H), 7.39 (dd, *J* = 3.7, 1.2 Hz, 1H), 7.14 (dd, *J* = 5.4, 3.7 Hz, 1H), 6.11 (ddt, *J* = 17.0, 10.2, 6.7 Hz, 1H), 5.42 – 5.29 (m, 2H), 3.20 (d, *J* = 6.8 Hz, 2H).

^{19}F NMR (376 MHz, $CDCl_3$) δ -36.99 (s, 6F).

^{13}C NMR (101 MHz, $CDCl_3$) δ 140.3, 134.4, 130.2, 128.7 (q, *J* = 313.2 Hz), 128.1, 126.6, 121.8, 72.0, 45.7.

HRMS (FI) m/z: $[M]^+$ Calcd for $C_{10}H_8F_6S_4$ 369.9408; Found 369.9413.

IR (KBr) 3086, 2987, 1640, 1434, 1417, 1399, 1294, 1220, 1142, 1097, 989, 947, 930, 852, 841, 755, 710, 646, 570, 497, 471, 445 cm^{-1}

2-((1,1-Bis((trifluoromethyl)thio)but-3-en-1-yl)thio)benzo[*d*]thiazole (8s)

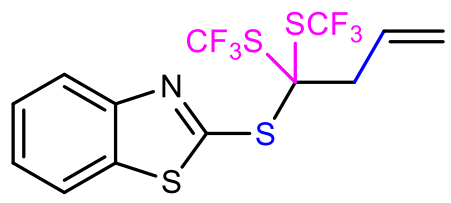

8s

The residue was purified by column chromatography on silica gel with an eluent of petroleum ether/EA (20:1, R_f = 0.58) to afford product **8s** (147.4 mg, 70% yield) as a brown oil.

^{1}H NMR (400 MHz, $CDCl_3$) δ 8.17 – 8.10 (m, 1H), 7.92 – 7.86 (m, 1H), 7.58 – 7.50 (m, 1H), 7.51 – 7.43 (m, 1H), 6.17 (ddt, J = 16.9, 9.8, 6.8 Hz, 1H), 5.41 – 5.33 (m, 2H), 3.45 (d, J = 6.8 Hz, 2H).

^{19}F NMR (376 MHz, $CDCl_3$) δ -36.91 (s, 6F).

^{13}C NMR (101 MHz, $CDCl_3$) δ 157.2, 153.4, 137.8, 130.0, 128.6 (q, J = 313.2 Hz), 126.9, 126.6, 124.2, 122.4, 121.5, 71.4, 46.6.

HRMS (FI) m/z: $[M]^+$ Calcd for $C_{13}H_9NF_6S_4$ 420.9517; Found 420.9521.

IR (KBr) 3086, 3066, 2989, 1639, 1555, 1454, 1432, 1417, 1312, 1238, 1143, 1107, 1095, 1016, 985, 947, 931, 802, 757, 728, 708, 673, 650, 472 cm^{-1}

Ethyl 2-((1,1-bis((trifluoromethyl)thio)but-3-en-1-yl)thio)acetate (8t)

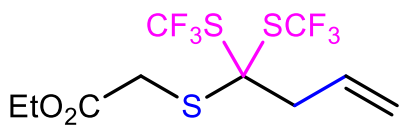

8t

The residue was purified by column chromatography on silica gel with an eluent of petroleum ether/EA (20:1, R_f = 0.37) to afford product **8t** (119.7 mg, 64% yield) as a brown oil.

^{1}H NMR (400 MHz, $CDCl_3$) δ 5.98 (ddt, J = 16.0, 10.5, 6.8 Hz, 1H), 5.35 – 5.27 (m, 2H), 4.20 (q, J = 7.1 Hz, 2H), 3.71 (s, 2H), 3.27 (d, J = 6.8 Hz, 2H), 1.28 (t, J = 7.1 Hz, 3H).

^{19}F NMR (376 MHz, $CDCl_3$) δ -37.47 (s, 6F).

^{13}C NMR (101 MHz, $CDCl_3$) δ 168.6, 130.1, 128.4 (q, J = 313.4 Hz), 121.9, 70.6, 62.3, 47.1, 34.9, 14.2.

HRMS (FI) m/z: $[M]^+$ Calcd for $C_{10}H_{12}O_2F_6S_3$ 373.9898; Found 373.9901.

IR (KBr) 3095, 2987, 2941, 1742, 1640, 1437, 1408, 1368, 1292, 1267, 1143, 1096, 1029, 989, 951, 930, 755, 473 cm^{-1}

(*Z*)-*tert*-Butyl(1-((trifluoromethyl)thio)buta-1,3-dien-1-yl)sulfane (8u)

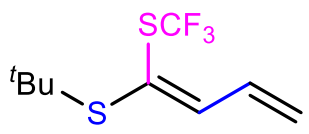

8u

The residue was purified by column chromatography on silica gel with an eluent of petroleum ether (R_f = 0.78) to afford product **8u** (65.3 mg, 54% yield) as a yellow oil.

^{1}H NMR (400 MHz, $CDCl_3$) δ 7.18 (d, *J* = 10.5 Hz, 1H), 6.97 (dt, *J* = 17.1, 10.3 Hz, 1H), 5.57 – 5.49 (m, 1H), 5.47 – 5.41 (m, 1H), 1.39 (s, 9H).

^{19}F NMR (376 MHz, $CDCl_3$) δ -41.81 (s, 3F).

^{13}C NMR (101 MHz, $CDCl_3$) δ 152.1 (q, *J* = 1.6 Hz), 134.3, 129.6 (q, *J* = 310.5 Hz), 124.1, 122.4 (q, *J* = 1.3 Hz), 50.0, 31.5.

HRMS (FI) m/z: $[M]^+$ Calcd for $C_9H_{13}F_3S_2$ 242.0405; Found 242.0402.

IR (KBr) 2964, 2926, 2858, 1462, 1367, 1155, 1144, 1103, 756 cm^{-1}

5-((Trifluoromethyl)thio)-4-vinyl-2,3-dihydrothiophene (8v)

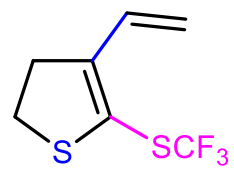

8v

The residue was purified by column chromatography on silica gel with an eluent of pentane (R_f = 0.63) to afford product **8v** (60.4 mg, 57% yield) as a yellow oil.

^{1}H NMR (400 MHz, $CDCl_3$) δ 6.81 (dd, *J* = 17.4, 10.8 Hz, 1H), 5.32 (d, *J* = 10.8 Hz, 1H), 5.21 (d, *J* = 17.3 Hz, 1H), 3.32 (t, *J* = 8.7 Hz, 2H), 3.07 (t, *J* = 8.7 Hz, 2H).

^{19}F NMR (376 MHz, $CDCl_3$) δ -41.22 (s, 3F).

^{13}C NMR (101 MHz, $CDCl_3$) δ 145.4 (q, *J* = 1.1 Hz), 129.8, 128.9 (q, *J* = 311.6 Hz), 121.9 (q, *J* = 1.8 Hz), 117.6, 35.9, 31.2.

HRMS (FI) m/z: $[M]^+$ Calcd for $C_7H_7F_3S_2$ 211.9936; Found 211.9937.

IR (KBr) 3095, 3022, 2938, 2833, 1609, 1539, 1445, 1428, 1414, 1295, 1234, 1160, 1141, 1107, 1026, 988, 960, 906, 755, 670, 572, 535, 453 cm^{-1}

(1-(Allylthio)but-3-ene-1,1-diyl)bis((trifluoromethyl)sulfane) (8w)

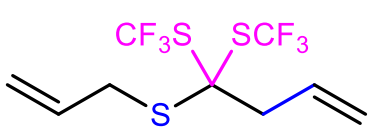

8w

The residue was purified by column chromatography on silica gel with an eluent of pentane (R_f = 0.84), followed by HPLC purification (Material: 5μm C18, Dimensions: 20×250mm, CH_3CN/H_2O = 90:10, flow rate 16.0 mL/min, λ = 220 nm, 25 °C, t_R = 7.588 min) to afford product **8w** (78.7 mg, 48% yield) as a yellow oil.

^{1}H NMR (400 MHz, $CDCl_3$) δ 6.00 (ddt, *J* = 17.1, 10.4, 6.8 Hz, 1H), 5.83 (ddt, *J* = 17.1, 10.0, 7.2 Hz, 1H), 5.34 – 5.26 (m, 3H), 5.19 (d, *J* = 10.0 Hz, 1H), 3.60 (d, *J* = 7.2 Hz, 2H), 3.27 (d, *J* = 6.8 Hz, 2H).

^{19}F NMR (376 MHz, $CDCl_3$) δ -37.52 (s, 6F).

^{13}C NMR (101 MHz, $CDCl_3$) δ 131.8, 130.5, 128.6 (q, *J* = 313.3 Hz), 121.4, 120.1, 71.0, 47.4, 35.8.

HRMS (FI) m/z: $[M]^+$ Calcd for $C_9H_{10}F_6S_3$ 327.9843; Found 327.9847.

IR (KBr) 2958, 2925, 2855, 1733, 1640, 1460, 1436, 1379, 1290, 1157, 1143, 1098, 987, 951, 926, 755 cm^{-1}

The procedure for 1,2-migration (Stevens rearrangement) of sulfur ylide from *sec*-benzyl sulfides with :C(SCF_3)$_2$ to obtain products 10a-10t

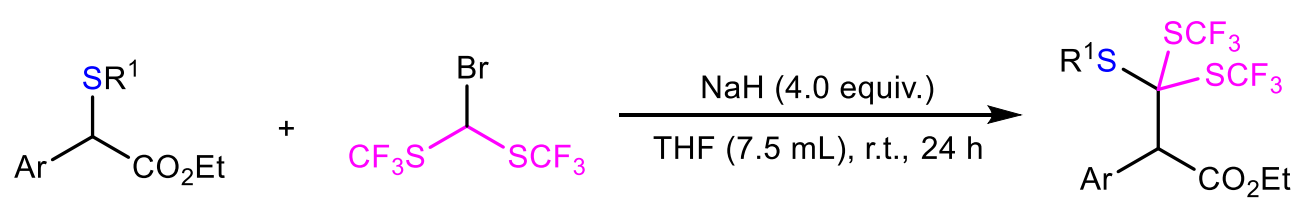

9, 0.5 mmol 2.0 equiv. **10**

A 25 mL bottom flask was charged with *sec*-benzyl sulfide **9** (0.5 mmol, 1.0 equiv.), NaH (48.0 mg, 2.0 mmol, 4.0 equiv.) and dry THF (7.5 mL) under a N_2 atmosphere. $BrCH(SCF_3)_2$ (295.0 mg, 1.0 mmol, 2.0 equiv.) was then added. The resulting mixture was stirred at room temperature for 24 h under a N_2 atmosphere. The resulting mixture was then diluted with ethyl acetate (20 mL) and saturated NH_4Cl solution (30 mL). The organic phase was separated and the aqueous phase was extracted 3 times. All organic solutions were combined and then dried over anhydrous Na_2SO_4. The solvent was removed under vacuum and the residue was subjected to column chromatography to give final products **10**.

Ethyl 2-phenyl-3-(phenylthio)-3,3-bis((trifluoromethyl)thio)propanoate (10a)

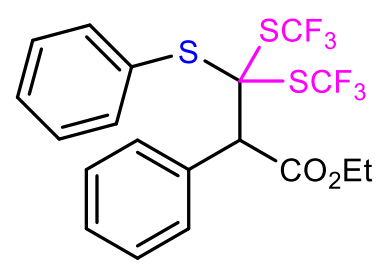

10a

The residue was purified by column chromatography on silica gel with an eluent of petroleum ether/EA (40:1, R_f = 0.41) to afford product **10a** (170.1 mg, 70% yield) as a yellow oil.

^{1}H NMR (400 MHz, $CDCl_3$) δ 7.69 – 7.63 (m, 2H), 7.57 – 7.51 (m, 2H), 7.47 – 7.38 (m, 4H), 7.38 – 7.30 (m, 2H), 4.69 (s, 1H), 4.25 – 4.15 (m, 1H), 4.14 – 4.03 (m, 1H), 1.21 (t, J = 7.1 Hz, 3H).

^{19}F NMR (376 MHz, $CDCl_3$) δ -35.79 (q, J = 3.0 Hz, 3F), -36.60 (q, J = 3.0 Hz, 3F).

^{13}C NMR (101 MHz, $CDCl_3$) δ 168.8, 138.2, 132.4, 131.6, 131.0, 129.5, 129.1, 128.9, 128.527, 128.526 (q, J = 312.9 Hz), 128.49 (q, J = 314.5 Hz), 72.8, 62.1, 61.9, 14.0.

HRMS (ESI) m/z: $[M+Na]^+$ Calcd for $C_{19}H_{16}O_2F_6NaS_3$ 509.0109; Found 509.0103.

IR (KBr) 3033, 3064, 2984, 2941, 1741, 1583, 1497, 1474, 1456, 1441, 1392, 1370, 1318, 1245, 1194, 1154, 1101, 1091, 1034, 1004, 858, 756, 726, 698, 690, 543, 498, 469 cm^{-1}

Ethyl 3-((4-(*tert*-butyl)phenyl)thio)-2-phenyl-3,3-bis((trifluoromethyl)thio)propanoate (10b)

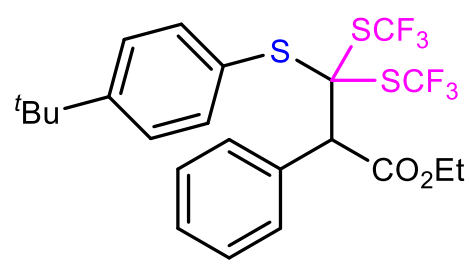

10b

The residue was purified by column chromatography on silica gel with an eluent of petroleum ether/EA (40:1, R_f = 0.42), followed by HPLC purification (Material: 5μm C18, Dimensions: 20×250mm, CH_3CN, flow rate 16.0 mL/min, λ = 220 nm, 25 °C, t_R = 6.698 min) to afford product **10b** (170.7 mg, 63% yield) as a yellow oil.

^{1}H NMR (400 MHz, $CDCl_3$) δ 7.63 (d, J = 7.6 Hz, 2H), 7.46 – 7.37 (m, 5H), 7.37 – 7.32 (m, 2H), 4.65 (s, 1H), 4.20 – 4.11 (m, 1H), 4.10 – 4.00 (m, 1H), 1.32 (s, 9H), 1.19 (t, J = 7.2 Hz, 3H).

^{19}F NMR (376 MHz, $CDCl_3$) δ -35.80 (q, J = 3.0 Hz, 3F), -36.55 (q, J = 3.1 Hz, 3F).

^{13}C NMR (101 MHz, $CDCl_3$) δ 168.8, 154.6, 138.0, 132.5, 131.6, 129.4, 128.61 (q, J = 312.9 Hz), 128.55 (q, J = 314.7 Hz), 128.5, 126.0, 125.6, 72.9, 62.2, 61.9, 35.1, 31.3, 14.0.

HRMS (DART) m/z: $[M+NH_4]^+$ Calcd for $C_{23}H_{28}O_2NF_6S_3$ 560.1181; Found 560.1179.

IR (KBr) 3065, 3032, 2965, 2926, 2871, 1742, 1593, 1489, 1456, 1396, 1368, 1317, 1267, 1195, 1154, 1101, 1089, 1034, 1014, 883, 834, 756, 726, 697, 557 cm^{-1}

Ethyl 3-((4-methoxyphenyl)thio)-2-phenyl-3,3-bis((trifluoromethyl)thio)propanoate (10c)

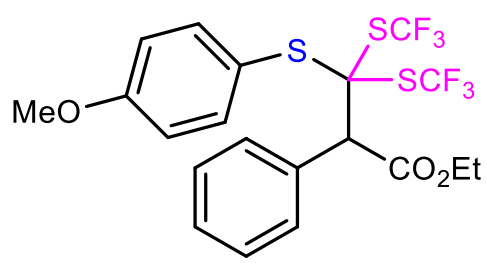

10c

The residue was purified by column chromatography on silica gel with an eluent of petroleum ether/EA (30:1, R_f = 0.31) to afford product **10c** (188.3 mg, 73% yield) as a yellow oil.

^{1}H NMR (400 MHz, $CDCl_3$) δ 7.69 – 7.64 (m, 2H), 7.47 (d, J = 8.7 Hz, 2H), 7.43 – 7.37 (m, 3H), 6.86 (d, J = 8.8 Hz, 2H), 4.66 (s, 1H), 4.27 – 4.15 (m, 1H), 4.15 – 4.05 (m, 1H), 3.81 (s, 3H), 1.22 (t, J = 7.1 Hz, 3H).

^{19}F NMR (376 MHz, $CDCl_3$) δ -35.76 (q, J = 3.1 Hz, 3F), -36.50 (q, J = 3.0 Hz, 3F).

^{13}C NMR (101 MHz, $CDCl_3$) δ 168.6, 162.0, 140.1, 132.3, 131.5, 129.3, 128.5 (q, J = 312.7 Hz), 128.4 (q, J = 314.3 Hz), 128.3, 119.3, 114.3, 73.0, 61.8, 61.7, 55.3, 13.8.

HRMS (FI) m/z: $[M]^+$ Calcd for $C_{20}H_{18}O_3F_6S_3$ 516.0317; Found 516.0311.

IR (KBr) 3066, 3031, 2982, 2941, 2907, 2841, 1738, 1591, 1568, 1494, 1456, 1443, 1408, 1392, 1370, 1291, 1254, 1190, 1162, 1083, 1031, 947, 917, 879, 832, 800, 757, 726, 698, 644, 628, 528, 496, 472 cm^{-1}

Ethyl 3-((4-(methylthio)phenyl)thio)-2-phenyl-3,3-bis((trifluoromethyl)thio)propanoate (10d)

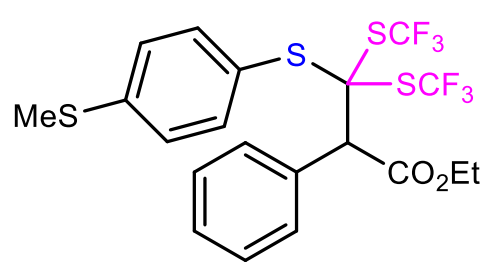

10d

The residue was purified by column chromatography on silica gel with an eluent of

petroleum ether/EA (30:1, R_f = 0.45) to afford product **10d** (119.7 mg, 45% yield) as a yellow oil.

^{1}H NMR (400 MHz, $CDCl_3$) δ 7.67 – 7.61 (m, 2H), 7.44 – 7.36 (m, 5H), 7.15 (d, J = 8.3 Hz, 2H), 4.64 (s, 1H), 4.25 – 4.14 (m, 1H), 4.13 – 4.02 (m, 1H), 2.48 (s, 3H), 1.20 (t, J = 7.1 Hz, 3H).

^{19}F NMR (376 MHz, $CDCl_3$) δ -35.74 (q, J = 2.7 Hz, 3F), -36.53 (q, J = 2.7 Hz, 3F).

^{13}C NMR (101 MHz, $CDCl_3$) δ 168.7, 143.5, 138.5, 132.4, 131.6, 129.5, 128.6 (q, J = 312.9 Hz), 128.50, 128.48 (q, J = 314.6 Hz), 125.6, 124.3, 73.0, 62.0, 61.9, 15.0, 14.0.

HRMS (FI) m/z: $[M]^+$ Calcd for $C_{20}H_{18}O_2F_6S_4$ 532.0088; Found 532.0085.

IR (KBr) 3064, 3033, 2984, 2925, 1740, 1575, 1496, 1476, 1455, 1440, 1392, 1369, 1318, 1240, 1195, 1155, 1101, 1085, 1034, 1013, 968, 914, 879, 858, 814, 756, 726, 698, 501, 472 cm^{-1}

Ethyl 3-((4-chlorophenyl)thio)-2-phenyl-3,3-bis((trifluoromethyl)thio)propanoate (10e)

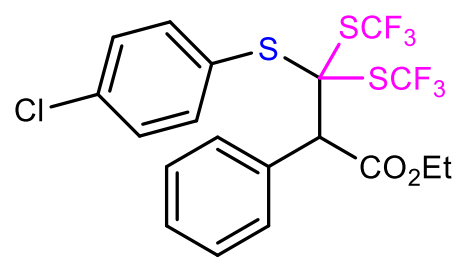

10e

The residue was purified by column chromatography on silica gel with an eluent of petroleum ether/EA (50:1, R_f = 0.45), followed by HPLC purification (Material: 5μm C18, Dimensions: 20×250mm, CH_3CN/H_2O = 90:10, flow rate 16.0 mL/min, λ = 220 nm, 25 °C, t_R = 12.217 min) to afford product **10e** (78.1 mg, 30% yield) as a yellow oil.

^{1}H NMR (400 MHz, $CDCl_3$) δ 7.66 – 7.59 (m, 2H), 7.46 – 7.35 (m, 5H), 7.33 – 7.27 (m, 2H), 4.64 (s, 1H), 4.25 – 4.15 (m, 1H), 4.13 – 4.03 (m, 1H), 1.20 (t, J = 7.2 Hz, 3H).

^{19}F NMR (376 MHz, $CDCl_3$) δ -35.75 (q, J = 2.7 Hz, 3F), -36.65 (q, J = 2.8 Hz, 3F).

^{13}C NMR (101 MHz, $CDCl_3$) δ 168.7, 139.4, 137.7, 132.2, 131.6, 129.6, 129.2, 128.6, 128.4 (q, J = 313.8 Hz), 127.6, 72.8, 62.0, 14.0.

HRMS (DART) m/z: $[M+NH_4]^+$ Calcd for $C_{19}H_{19}O_2NClF_6S_3$ 538.0165; Found 538.0165.

IR (KBr) 3066, 3033, 2984, 2933, 2855, 1742, 1573, 1497, 1475, 1455, 1391, 1370, 1317, 1244, 1197, 1155, 1093, 1034, 1014, 948, 917, 879, 858, 824, 757, 725, 698, 502,

471 cm^{-1}

Ethyl 3-((4-bromophenyl)thio)-2-phenyl-3,3-bis((trifluoromethyl)thio)propanoate (10f)

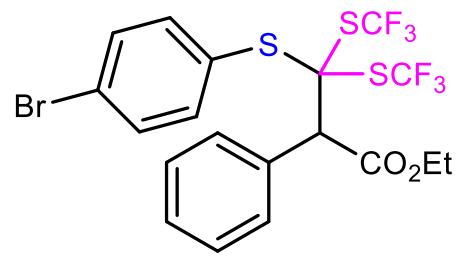

10f

The residue was purified by column chromatography on silica gel with an eluent of petroleum ether/EA (50:1, R_f = 0.49), followed by HPLC purification (Material: 5μm C18, Dimensions: 20×250mm, CH_3CN/H_2O = 95:5, flow rate 16.0 mL/min, λ = 220 nm, 25 °C, t_R = 8.122 min) to afford product **10f** (112.8 mg, 40% yield) as a yellow oil.

^{1}H NMR (400 MHz, $CDCl_3$) δ 7.66 – 7.60 (m, 2H), 7.49 – 7.36 (m, 5H), 7.35 – 7.28 (m, 2H), 4.65 (s, 1H), 4.25 – 4.15 (m, 1H), 4.13 – 4.03 (m, 1H), 1.21 (t, J = 7.1 Hz, 3H).

^{19}F NMR (376 MHz, $CDCl_3$) δ -35.74 (q, J = 2.8 Hz, 3F), -36.64 (q, J = 2.7 Hz, 3F).

^{13}C NMR (101 MHz, $CDCl_3$) δ 168.7, 139.5, 132.2, 131.6, 129.6, 128.6, 128.4 (q, J = 313.9 Hz), 128.2, 126.2, 72.7, 62.0, 14.0.

HRMS (FI) m/z: $[M]^+$ Calcd for $C_{19}H_{15}O_2BrF_6S_3$ 563.9316; Found 563.9314.

IR (KBr) 3065, 3033, 2983, 2937, 1741, 1566, 1496, 1473, 1456, 1387, 1370, 1317, 1243, 1195, 1154, 1104, 1087, 1034, 1010, 947, 879, 857, 819, 757, 729, 698, 514, 493, 483 cm^{-1}

Ethyl 3-((4-cyanophenyl)thio)-2-phenyl-3,3-bis((trifluoromethyl)thio)propanoate (10g)

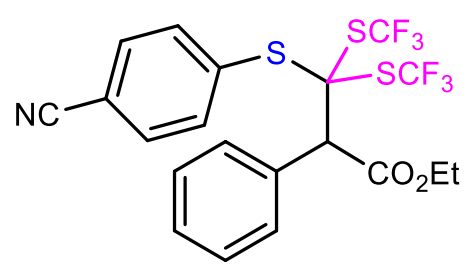

10g

The residue was purified by column chromatography on silica gel with an eluent of petroleum ether/EA (10:1, R_f = 0.51) to afford product **10g** (92.0 mg, 36% yield) as a yellow oil.

^{1}H NMR (400 MHz, $CDCl_3$) δ 7.63 – 7.57 (m, 2H), 7.57 – 7.50 (m, 2H), 7.47 – 7.35 (m, 5H), 4.68 (s, 1H), 4.27 – 4.17 (m, 1H), 4.13 – 4.03 (m, 1H), 1.20 (t, J = 7.1 Hz, 3H).

^{19}F NMR (376 MHz, $CDCl_3$) δ -35.80 (q, J = 2.9 Hz, 3F), -36.91 (q, J = 2.8 Hz, 3F).

^{13}C NMR (101 MHz, $CDCl_3$) δ 168.5, 137.3, 136.0, 132.1, 131.8, 131.5, 129.8, 128.7, 128.3 (q, J = 314.0 Hz), 128.1 (q, J = 313.3 Hz), 118.1, 114.0, 72.6, 62.13, 62.10, 14.0.

HRMS (FI) m/z: $[M]^+$ Calcd for $C_{20}H_{15}O_2NF_6S_3$ 511.0164; Found 511.0168.

IR (KBr) 3066, 3034, 2984, 2939, 2231, 1739, 1592, 1497, 1484, 1456, 1396, 1370, 1318, 1245, 1195, 1155, 1101, 1087, 1033, 1018, 878, 838, 780, 757, 726, 698, 550, 492 cm^{-1}

Ethyl 2-phenyl-3-((4-(trifluoromethyl)phenyl)thio)-3,3-bis((trifluoromethyl)thio)propanoate (10h)

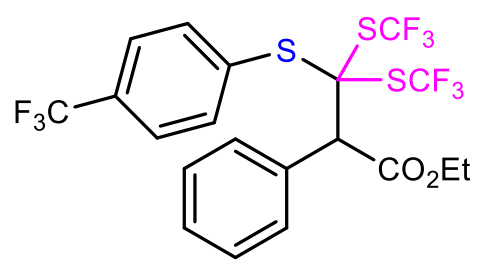

10h

The residue was purified by column chromatography on silica gel with an eluent of petroleum ether/EA (50:1, R_f = 0.40), followed by HPLC purification (Material: 5μm C18, Dimensions: 20×250mm, CH_3CN/H_2O = 90:10, flow rate 16.0 mL/min, λ = 220 nm, 25 °C, t_R = 9.354 min) to afford product **10h** (80.3 mg, 29% yield) as a yellow oil.

^{1}H NMR (400 MHz, $CDCl_3$) δ 7.64 – 7.59 (m, 2H), 7.57 – 7.49 (m, 4H), 7.46 – 7.36 (m, 3H), 4.68 (s, 1H), 4.25 – 4.15 (m, 1H), 4.13 – 4.03 (m, 1H), 1.20 (t, J = 7.1 Hz, 3H).

^{19}F NMR (376 MHz, $CDCl_3$) δ -35.82 (q, J = 2.9 Hz, 3F), -36.80 (q, J = 2.8 Hz, 3F), -63.11 (s, 3F).

^{13}C NMR (101 MHz, $CDCl_3$) δ 168.7, 137.7, 134.1, 132.5 (q, J = 32.8 Hz), 132.0, 131.6, 129.8, 128.7, 128.4 (q, J = 314.5 Hz), 128.3 (q, J = 313.1 Hz), 125.6 (q, J = 3.8 Hz), 123.9 (q, J = 272.5 Hz), 72.6, 62.2, 62.1, 14.0.

HRMS (FI) m/z: $[M]^+$ Calcd for $C_{20}H_{15}O_2F_9S_3$ 554.0085; Found 554.0089.

IR (KBr) 3067, 3034, 2985, 2934, 1742, 1606, 1497, 1456, 1397, 1371, 1324, 1244, 1192, 1170, 1135, 1104, 1063, 1034, 1016, 879, 841, 780, 757, 724, 699, 599 cm^{-1}

Ethyl 3-((4-nitrophenyl)thio)-2-phenyl-3,3-bis((trifluoromethyl)thio)propanoate (10i)

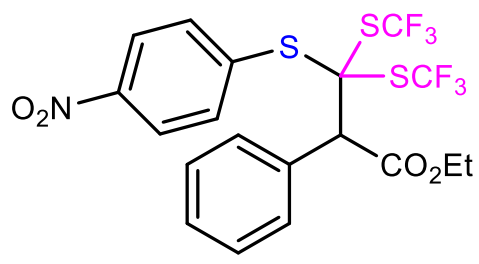

10i

The residue was purified by column chromatography on silica gel with an eluent of petroleum ether/EA (20:1, R_f = 0.58) to afford product **10i** (66.4 mg, 25% yield) as a yellow oil.

^{1}H NMR (400 MHz, $CDCl_3$) δ 8.08 (d, J = 8.8 Hz, 2H), 7.61 (d, J = 7.0 Hz, 2H), 7.50 – 7.35 (m, 5H), 4.70 (s, 1H), 4.30 – 4.19 (m, 1H), 4.15 – 4.04 (m, 1H), 1.21 (t, J = 7.1 Hz, 3H).

^{19}F NMR (376 MHz, $CDCl_3$) δ -35.79 (q, J = 2.3 Hz, 3F), -36.94 (q, J = 2.3 Hz, 3F).

^{13}C NMR (101 MHz, $CDCl_3$) δ 168.6, 148.7, 138.3, 137.2, 131.8, 131.5, 129.9, 128.8, 128.3 (q, J = 314.4 Hz), 128.1 (q, J = 313.3 Hz), 123.5, 72.5, 62.21, 62.19, 14.0.

HRMS (FI) m/z: $[M]^+$ Calcd for $C_{19}H_{15}O_4NF_6S_3$ 531.0062; Found 531.0064.

IR (KBr) 3100, 3067, 3033, 2985, 2939, 2857, 1739, 1599, 1579, 1525, 1497, 1476, 1456, 1394, 1370, 1346, 1317, 1280, 1244, 1197, 1158, 1104, 1085, 1034, 1014, 853, 757, 743, 728, 698, 492, 463 cm^{-1}

Ethyl 2-(4-methoxyphenyl)-3-(phenylthio)-3,3-bis((trifluoromethyl)thio)propanoate (10j)

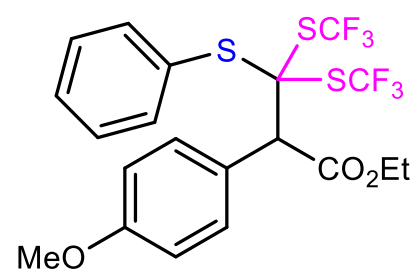

10j

The residue was purified by column chromatography on silica gel with an eluent of petroleum ether/EA (20:1, R_f = 0.26) to afford product **10j** (203.8 mg, 79% yield) as a yellow oil.

^{1}H NMR (400 MHz, $CDCl_3$) δ 7.61 – 7.52 (m, 4H), 7.48 – 7.40 (m, 1H), 7.38 – 7.31 (m, 2H), 6.97 – 6.89 (m, 2H), 4.63 (s, 1H), 4.23 – 4.13 (m, 1H), 4.12 – 4.02 (m, 1H), 3.83 (s, 3H), 1.21 (t, J = 7.1 Hz, 3H).

^{19}F NMR (376 MHz, $CDCl_3$) δ -35.83 (q, J = 3.0 Hz, 3F), -36.63 (q, J = 3.0 Hz, 3F).

^{13}**C NMR** (101 MHz, $CDCl_3$) δ 168.9, 160.6, 138.2, 132.8, 131.0, 129.2, 128.9, 128.54 (q, J = 312.9 Hz), 128.50 (q, J = 314.4 Hz), 124.3, 113.9, 73.3, 61.8, 61.3, 55.4, 14.0.

HRMS (ESI) m/z: $[M+Na]^+$ Calcd for $C_{20}H_{18}O_3F_6NaS_3$ 539.0214; Found 539.0209.

IR (KBr) 3062, 2983, 2938, 2908, 2840, 1738, 1608, 1582, 1513, 1473, 1441, 1427, 1392, 1370, 1307, 1263, 1158, 1100, 1034, 1003, 950, 882, 859, 835, 800, 754, 704, 689, 529, 499, 468, 419 cm^{-1}

Ethyl 2-(4-cyanophenyl)-3-(phenylthio)-3,3-bis((trifluoromethyl)thio)propanoate (10k)

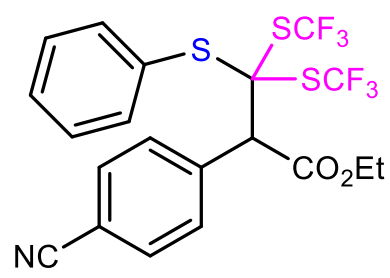

10k

The residue was purified by column chromatography on silica gel with an eluent of petroleum ether/EA (30:1, R_f = 0.23), followed by HPLC purification (Material: 5μm C18, Dimensions: 20×250mm, CH_3CN/H_2O = 90:10, flow rate 16.0 mL/min, λ = 220 nm, 25 °C, t_R = 5.955 min) to afford product **10k** (74.1 mg, 29% yield) as a yellow oil.

^{1}**H NMR** (400 MHz, $CDCl_3$) δ 7.77 (d, J = 8.1 Hz, 2H), 7.68 (d, J = 8.2 Hz, 2H), 7.60 – 7.53 (m, 2H), 7.51 – 7.44 (m, 1H), 7.41 – 7.34 (m, 2H), 4.71 (s, 1H), 4.23 – 4.13 (m, 1H), 4.13 – 4.03 (m, 1H), 1.21 (t, J = 7.1 Hz, 3H).

^{19}**F NMR** (376 MHz, $CDCl_3$) δ -35.59 (q, J = 2.6 Hz, 3F), -36.22 (q, J = 2.6 Hz, 3F).

^{13}**C NMR** (101 MHz, $CDCl_3$) δ 168.0, 138.2, 137.5, 132.3, 132.1, 131.4, 129.2, 128.34 (q, J = 313.3 Hz), 128.31 (q, J = 314.6 Hz), 128.2, 118.4, 113.5, 72.2, 62.5, 62.2, 14.0.

HRMS (DART) m/z: $[M+NH_4]^+$ Calcd for $C_{20}H_{19}O_2N_2F_6S_3$ 529.0507; Found 529.0507.

IR (KBr) 3063, 2985, 2937, 2862, 2231, 1741, 1607, 1503, 1474, 1441, 1417, 1393, 1370, 1315, 1242, 1199, 1158, 1101, 1087, 1024, 1003, 883, 859, 843, 754, 704, 689, 556, 501, 470, 419 cm^{-1}

Ethyl 2-(4-nitrophenyl)-3-(phenylthio)-3,3-bis((trifluoromethyl)thio)propanoate (10l)

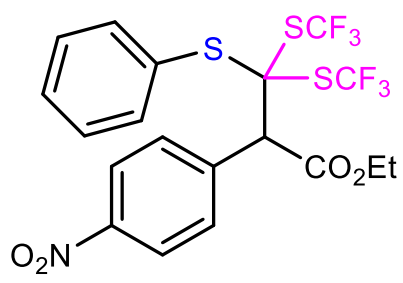

10l

The residue was purified by column chromatography on silica gel with an eluent of petroleum ether/EA (10:1, R_f = 0.41), followed by HPLC purification (Material: 5μm C18, Dimensions: 20×250mm, CH_3CN/H_2O = 90:10, flow rate 16.0 mL/min, λ = 220 nm, 25 °C, t_R = 8.002 min) to afford product **10l** (42.5 mg, 16% yield) as a yellow oil.

^{1}H NMR (400 MHz, $CDCl_3$) δ 8.23 (d, J = 8.4 Hz, 2H), 7.84 (d, J = 8.4 Hz, 2H), 7.58 (d, J = 7.7 Hz, 2H), 7.48 (t, J = 7.5 Hz, 1H), 7.37 (t, J = 7.6 Hz, 2H), 4.78 (s, 1H), 4.24 – 4.14 (m, 1H), 4.14 – 4.04 (m, 1H), 1.21 (t, J = 7.2 Hz, 3H).

^{19}F NMR (376 MHz, $CDCl_3$) δ -35.56 (q, J = 2.1 Hz, 3F), -36.17 (q, J = 2.1 Hz, 3F).

^{13}C NMR (101 MHz, $CDCl_3$) δ 167.9, 148.6, 139.4, 138.2, 132.6, 131.5, 129.2, 128.34 (q, J = 313.2 Hz), 128.31 (q, J = 314.7 Hz), 128.2, 123.5, 72.2, 62.6, 61.9, 14.0.

HRMS (FI) m/z: $[M]^+$ Calcd for $C_{19}H_{15}O_4NF_6S_3$ 531.0062; Found 531.0058.

IR (KBr) 3066, 2993, 2928, 2855, 1740, 1607, 1527, 1494, 1474, 1441, 1392, 1369, 1349, 1313, 1260, 1201, 1156, 1102, 1090, 1026, 913, 857, 754, 705, 689 cm^{-1}

Ethyl 3-((4-methoxyphenyl)thio)-2-(4-nitrophenyl)-3,3-bis((trifluoromethyl)thio)propanoate (10m)

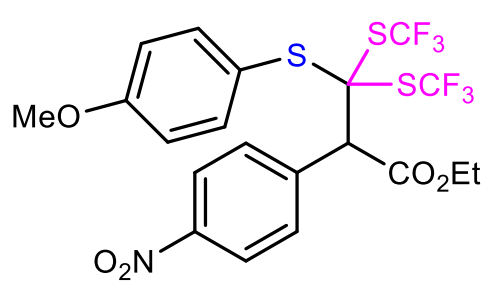

10m

The residue was purified by column chromatography on silica gel with an eluent of petroleum ether/EA (40:1, R_f = 0.30), followed by HPLC purification (Material: 5μm C18, Dimensions: 20×250mm, CH_3CN/H_2O = 90:10, flow rate 16.0 mL/min, λ = 220 nm, 25 °C, t_R = 7.291 min) to afford product **10m** (44.9 mg, 16% yield) as a yellow oil (a little amount of impurity was contained in the product).

^{1}H NMR (400 MHz, $CDCl_3$) δ 8.24 (d, J = 8.8 Hz, 2H), 7.85 (d, J = 8.8 Hz, 2H), 7.49 (d, J = 8.8 Hz, 2H), 6.88 (d, J = 8.8 Hz, 2H), 4.75 (s, 1H), 4.26 – 4.16 (m, 1H), 4.16 – 4.05 (m, 1H), 3.83 (s, 3H), 1.22 (t, J = 7.1 Hz, 3H).

^{19}F NMR (376 MHz, $CDCl_3$) δ -35.56 (q, J = 2.7 Hz, 3F), -36.15 (q, J = 2.8 Hz, 3F).

^{13}C NMR (101 MHz, $CDCl_3$) δ 168.0, 162.4, 148.5, 140.3, 139.5, 132.6, 128.5 (q, J = 312.9 Hz), 128.4 (q, J = 314.6 Hz), 123.4, 118.4, 114.7, 72.6, 62.5, 61.7, 55.6, 14.0.

HRMS (FI) m/z: $[M]^+$ Calcd for $C_{20}H_{17}O_5NF_6S_3$ 561.0168; Found 561.0171.

IR (KBr) 3080, 2939, 2843, 1742, 1591, 1568, 1526, 1494, 1464, 1443, 1422, 1409, 1392, 1369, 1349, 1292, 1254, 1158, 1096, 1029, 957, 884, 857, 832, 800, 756, 731, 697, 643, 528, 498, 473 cm^{-1}

Ethyl 2-phenyl-3-(thiophen-2-ylthio)-3,3-bis((trifluoromethyl)thio)propanoate (10n)

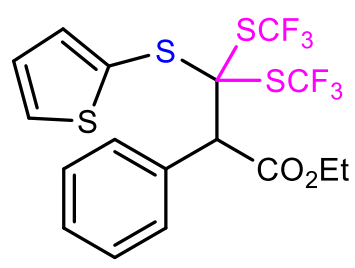

10n

The residue was purified by column chromatography on silica gel with an eluent of petroleum ether/EA (50:1, R_f = 0.53), followed by HPLC purification (Material: 5μm C18, Dimensions: 20×250mm, CH_3CN/H_2O = 90:10, flow rate 16.0 mL/min, λ = 220 nm, 25 °C, t_R = 8.733 min) to afford product **10n** (93.5 mg, 38% yield) as a yellow oil.

^{1}H NMR (400 MHz, $CDCl_3$) δ 7.63 (d, J = 7.9 Hz, 2H), 7.59 (d, J = 5.4 Hz, 1H), 7.46 – 7.37 (m, 3H), 7.33 – 7.27 (m, 1H), 7.08 – 7.03 (m, 1H), 4.62 (s, 1H), 4.28 – 4.18 (m, 1H), 4.15 – 4.05 (m, 1H), 1.22 (t, J = 7.2 Hz, 3H).

^{19}F NMR (376 MHz, $CDCl_3$) δ -35.65 (q, J = 2.7 Hz, 3F), -36.16 (q, J = 2.7 Hz, 3F).

^{13}C NMR (101 MHz, $CDCl_3$) δ 168.7, 141.2, 135.1, 132.2, 131.6, 129.6, 128.7 (q, J = 313.1 Hz), 128.6, 128.5 (q, J = 314.2 Hz),127.7, 126.9, 73.4, 62.0, 61.5, 14.1.

HRMS (ESI) m/z: $[M+Na]^+$ Calcd for $C_{17}H_{14}O_2F_6NaS_4$ 514.9673; Found 514.9677.

IR (KBr) 3066, 3033, 2984, 2936, 1740, 1497, 1456, 1398, 1370, 1318, 1243, 1220, 1195, 1155, 1103, 1087, 1034, 991, 917, 853, 757, 698, 569, 498, 470 cm^{-1}

Ethyl 3-(ethylthio)-2-phenyl-3-((trifluoromethyl)thio)acrylate (10o)

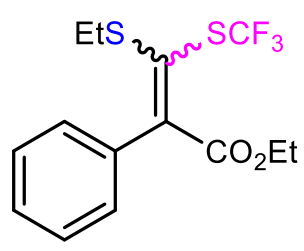

10o

The residue was purified by column chromatography on silica gel with an eluent of

petroleum ether/EA (40:1, R_f = 0.42), followed by HPLC purification (Material: 5μm C18, Dimensions: 20×250mm, CH_3CN/H_2O = 90:10, flow rate 16.0 mL/min, λ = 220 nm, 25 °C, t_R = 5.265 min) to afford product **10o** (104.2 mg, 62% yield, isomers, 2.8 : 1) as a yellow oil.

One isomer:

^{1}H NMR (400 MHz, $CDCl_3$) δ 7.45 – 7.31 (m, 5H), 4.28 (q, J = 7.1 Hz, 2H), 2.97 (q, J = 7.4 Hz, 2H), 1.35 – 1.28 (m, 6H).

^{19}F NMR (376 MHz, $CDCl_3$) δ -39.75 (s, 3F).

^{13}C NMR (101 MHz, $CDCl_3$) δ 166.8, 150.0, 135.2, 129.2, 128.8, 128.7 (q, J = 311.4 Hz), 128.6, 126.7, 62.1, 28.2, 14.5, 14.3.

The other isomer:

^{1}H NMR (400 MHz, $CDCl_3$) δ 7.45 – 7.31 (m, 5H), 4.28 (q, J = 7.0 Hz, 2H), 2.84 (q, J = 7.4 Hz, 2H), 1.35 – 1.28 (m, 3H), 1.17 (t, J = 7.4 Hz, 3H).

^{19}F NMR (376 MHz, $CDCl_3$) δ -40.79 (s, 3F).

^{13}C NMR (101 MHz, $CDCl_3$) δ 166.8, 149.4, 134.8, 129.4, 128.81, 128.79 (q, J = 311.8 Hz), 128.6, 126.1, 62.2, 28.3, 14.3, 14.1.

HRMS (FI) m/z: $[M]^+$ Calcd for $C_{14}H_{15}O_2F_3S_2$ 336.0460; Found 336.0456.

IR (KBr) 3060, 2981, 2930, 2873, 1731, 1492, 1444, 1423, 1367, 1288, 1267, 1201, 1164, 1139, 1105, 1038, 1025, 1002, 900, 860, 756, 739, 697, 578, 489, 420 cm^{-1}

Ethyl 3-(benzylthio)-2-phenyl-3,3-bis((trifluoromethyl)thio)propanoate (10p)

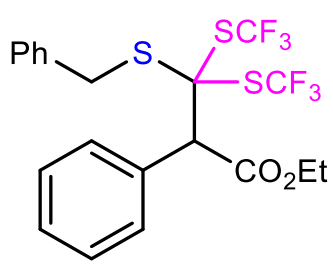

10p

The residue was purified by column chromatography on silica gel with an eluent of petroleum ether/EA (100:1, R_f = 0.30) to afford product **10p** (177.5 mg, 71% yield) as a yellow oil.

^{1}H NMR (400 MHz, $CDCl_3$) δ 7.67 – 7.60 (m, 2H), 7.42 – 7.33 (m, 3H), 7.28 – 7.20 (m, 5H), 4.70 (s, 1H), 4.31 – 4.21 (m, 1H), 4.13 – 4.03 (m, 1H), 4.00 (d, J = 10.6 Hz, 1H), 3.79 (d, J = 10.6 Hz, 1H), 1.20 (t, J = 7.1 Hz, 3H).

^{19}F NMR (376 MHz, $CDCl_3$) δ -36.19 (s, 3F), -36.34 (s, 3F).

^{13}C NMR (101 MHz, $CDCl_3$) δ 169.0, 134.0, 132.2, 131.4, 129.9, 129.6, 128.9, 128.8 (q, J = 313.5 Hz), 128.7 (q, J = 313.5 Hz), 128.6, 128.1, 72.4, 63.2, 62.0, 38.6, 14.1.

HRMS (FI) m/z: $[M]^+$ Calcd for $C_{20}H_{18}O_2F_6S_3$ 500.0368; Found 500.0365.

IR (KBr) 3065, 3032, 2984, 2940, 1738, 1602, 1585, 1496, 1455, 1391, 1370, 1318, 1262, 1240, 1196, 1154, 1103, 1089, 1031, 1004, 950, 917, 879, 858, 772, 756, 697, 568, 542, 492, 480 cm^{-1}

(2,2-Diphenyl-1-((trifluoromethyl)thio)vinyl)(phenyl)sulfane (10q)

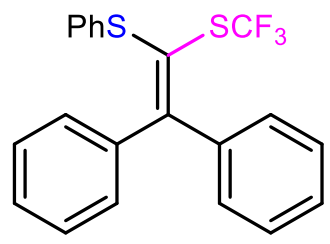

10q

The residue was purified by column chromatography on silica gel with an eluent of petroleum ether (R_f = 0.47), followed by HPLC purification (Material: 5μm C18, Dimensions: 20×250mm, CH_3CN/H_2O = 90:10, flow rate 16.0 mL/min, λ = 220 nm, 25 °C, t_R = 7.776 min) to afford product **10q** (102.8 mg, 53% yield) as a yellow oil.

^{1}H NMR (400 MHz, $CDCl_3$) δ 7.37 – 7.27 (m, 10H), 7.27 – 7.22 (m, 3H), 7.22 – 7.18 (m, 2H).

^{19}F NMR (376 MHz, $CDCl_3$) δ -40.96 (s, 3F).

^{13}C NMR (101 MHz, $CDCl_3$) δ 161.3 (q, J = 1.8 Hz), 141.6, 141.3, 134.9, 131.2, 129.7, 129.4, 129.3 (q, J = 311.2 Hz), 129.2, 128.7, 128.5, 128.3, 127.6, 121.2 (q, J = 1.4 Hz).

HRMS (FI) m/z: $[M]^+$ Calcd for $C_{21}H_{15}F_3S_2$ 388.0562; Found 388.0566.

IR (KBr) 3081, 3059, 3027, 2926, 1580, 1477, 1442, 1155, 1132, 1104, 1032, 1025, 1000, 968, 846, 755, 743, 698, 635, 616, 610, 567, 518, 480, 463, 419 cm^{-1}

Scrambling reaction of 1,2-Migration (Stevens rearrangement) of sulfur ylide from *sec*-benzyl sulfides with :$C(SCF_3)_2$

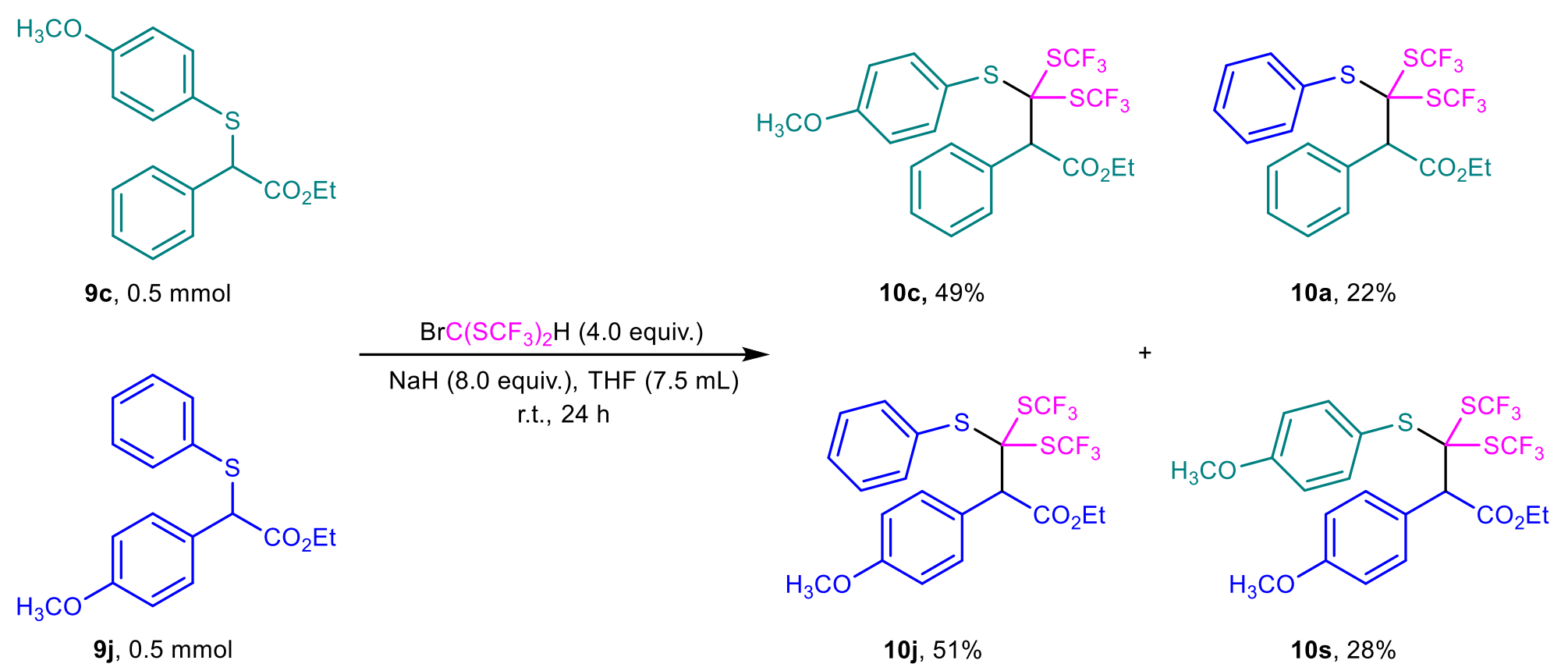

A 25 mL bottom flask was charged with *sec*-benzyl sulfide **9c** (151.0 mg, 0.5 mmol, 1.0 equiv.), **9j** (151.0 mg, 0.5 mmol, 1.0 equiv.), NaH (96.0 mg, 4.0 mmol, 8.0 equiv.) and dry THF (7.5 mL) under a N_2 atmosphere. $BrCH(SCF_3)_2$ (590.0 mg, 2.0 mmol, 4.0 equiv.) was then added. The resulting mixture was stirred at room temperature for 24 h under a N_2 atmosphere. The resulting mixture was then diluted with ethyl acetate (20 mL) and saturated NH_4Cl solution (30 mL). The organic phase was separated and the aqueous phase was extracted 3 times. All organic solutions were combined and then dried over anhydrous Na_2SO_4. The solvent was removed under vacuum and the residue was subjected to column chromatography on silica gel with an eluent of petroleum ether/EA (100:1 to 30:1) to give final products **10a** (53.5 mg, 22% yield, R_f = 0.53 (petroleum ether/EA = 50:1)), mixture of **10c** and **10j** (243.1 mg, 49% yield for **10c**, 51% yield for **10j**, R_f = 0.29 (petroleum ether/EA = 50:1)), and **10s** (76.4 mg, 28% yield, R_f = 0.11 (petroleum ether/EA = 50:1)) as yellow oils.

Ethyl 2-(4-methoxyphenyl)-3-((4-methoxyphenyl)thio)-3,3-bis((trifluoromethyl)thio)propanoate (10s)

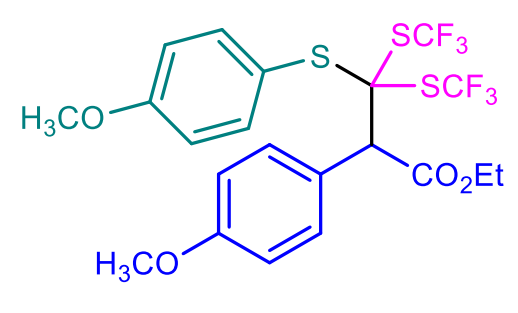

10s

^{1}H NMR (400 MHz, $CDCl_3$) δ 7.56 (d, *J* = 8.7 Hz, 2H), 7.47 (d, *J* = 8.5 Hz, 2H), 6.91 (d, *J* = 8.6 Hz, 2H), 6.85 (d, *J* = 8.5 Hz, 2H), 4.58 (s, 1H), 4.23 – 4.14 (m, 1H), 4.12 – 4.03 (m, 1H), 3.83 (s, 3H), 3.82 (s, 3H), 1.21 (t, *J* = 7.1 Hz, 3H).

^{19}F NMR (376 MHz, $CDCl_3$) δ -35.78 (q, *J* = 3.1 Hz, 3F), -36.52 (q, *J* = 3.2 Hz, 3F).

^{13}C NMR (101 MHz, $CDCl_3$) δ 169.0, 162.1, 160.5, 140.3, 132.9, 128.7 (q, J = 312.7 Hz), 128.6 (q, J = 314.5 Hz), 124.4, 119.6, 114.4, 113.8, 73.6, 61.8, 61.1, 55.5, 55.4, 14.0.

HRMS (ESI) m/z: $[M+Na]^+$ Calcd for $C_{21}H_{20}O_4F_6NaS_3$ 569.0320; Found 569.0316.

IR (KBr) 2939, 2840, 1738, 1609, 1591, 1569, 1512, 1494, 1464, 1443, 1408, 1392, 1369, 1291, 1254, 1176, 1104, 1032, 832, 799, 756, 699, 643, 529, 499, 471 cm^{-1}

Radical inhibition experiment of 1,2-Migration (Stevens rearrangement) of sulfur ylide from *sec*-benzyl sulfides with :$C(SCF_3)_2$

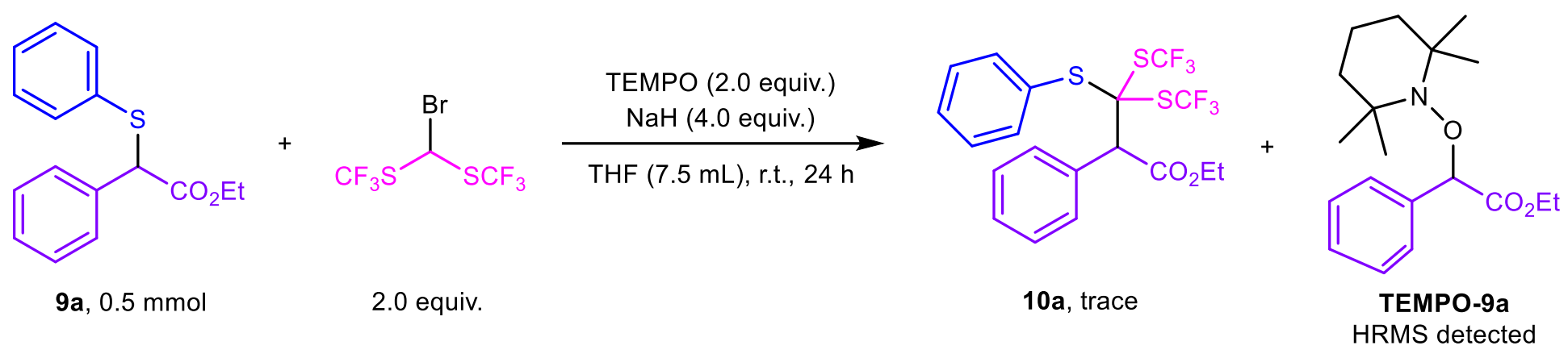

A 25 mL bottom flask was charged with *sec*-benzyl sulfide **9a** (151.0 mg, 0.5 mmol, 1.0 equiv.), TEMPO (156.0 mg, 1.0 mmol, 2.0 equiv.), NaH (48.0 mg, 2.0 mmol, 4.0 equiv.) and dry THF (7.5 mL) under a N_2 atmosphere. $BrCH(SCF_3)_2$ (295.0 mg, 1.0 mmol, 2.0 equiv.) was then added. The resulting mixture was stirred at room temperature for 24 h under a N_2 atmosphere. The resulting mixture was then diluted with ethyl acetate (20 mL) and saturated NH_4Cl solution (30 mL). The organic phase was separated and the aqueous phase was extracted 3 times. All organic solutions were combined and then dried over anhydrous Na_2SO_4. The solvent was removed under vacuum and the residue was subjected to crude ^{19}F NMR spectrum (0.5 mmol $PhOCF_3$ was added as as an internal standard) and HRMS detection. The results show the trace yield of the desired product **10a** and the formation of radical inhibition product **TEMPO-9a** (**HRMS (ESI)** m/z: $[M+H]^+$ Calcd for $C_{19}H_{30}O_3N$ 320.2220; Found 320.2216.).

The procedure for [4+1] cyclization of 2-vinyl pyridines with :$C(SCF_3)_2$ to obtain products 12a-12b, 12d-12r, 12t

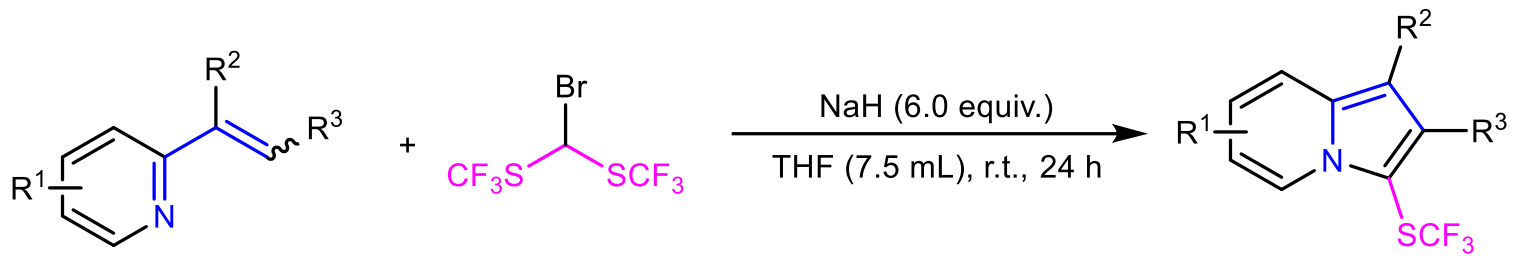

A 25 mL bottom flask was charged with 2-vinyl pyridines **11** (0.5 mmol, 1.0 equiv.), NaH (72.0 mg, 3.0 mmol, 6.0 equiv.) and dry THF (7.5 mL) under a N_2 atmosphere. $BrCH(SCF_3)_2$ (442.5 mg, 1.5 mmol, 3.0 equiv.) was then added. The resulting mixture was stirred at room temperature for 24 h under a N_2 atmosphere. The resulting mixture was then diluted with ethyl acetate (20 mL) and saturated NH_4Cl solution (30 mL). The organic phase was separated and the aqueous phase was extracted 3 times. All organic solutions were combined and then dried over anhydrous Na_2SO_4. The solvent was removed under vacuum and the residue was subjected to column chromatography to give final products **12**.

3-((Trifluoromethyl)thio)indolizine (12a)

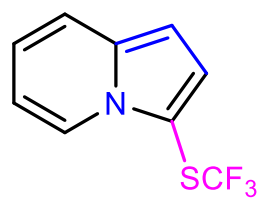

12a

The residue was purified by column chromatography on silica gel with an eluent of petroleum ether (R_f = 0.80) to afford product **12a** (57.5 mg, 53% yield) as a yellow solid, m.p. 50-52 ºC.

^{1}H NMR (400 MHz, $CDCl_3$) δ 8.46 (d, J = 7.1 Hz, 1H), 7.45 (d, J = 8.9 Hz, 1H), 7.23 (d, J = 4.2 Hz, 1H), 6.92 (dd, J = 9.0, 6.6 Hz, 1H), 6.78 – 6.69 (m, 1H), 6.57 (d, J = 4.2 Hz, 1H).

^{19}F NMR (376 MHz, $CDCl_3$) δ -45.08 (s, 3F).

^{13}C NMR (101 MHz, $CDCl_3$) δ 138.1, 128.8 (q, J = 313.9 Hz), 126.7, 124.0, 120.5, 119.4, 112.0, 101.4, 100.0.

HRMS (FI) m/z: $[M]^+$ Calcd for $C_9H_6NF_3S$ 217.0168; Found 217.0170.

IR (KBr) 2967, 2925, 2854, 1498, 1466, 1445, 1381, 1348, 1304, 1273, 1246, 1155, 1133, 1104, 1048, 1010, 828, 764, 750, 733, 481, 464, 422 cm^{-1}

6-Methyl-3-((trifluoromethyl)thio)indolizine (12b)

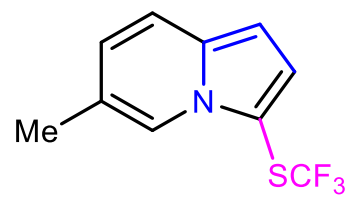

12b

The residue was purified by column chromatography on silica gel with an eluent of petroleum ether (R_f = 0.82) to afford product **12b** (40.4 mg, 35% yield) as a yellow oil.

^{1}H NMR (400 MHz, $CDCl_3$) δ 8.25 (s, 1H), 7.36 (d, *J* = 9.0 Hz, 1H), 7.15 (d, *J* = 4.2 Hz, 1H), 6.78 (dd, *J* = 9.1, 1.5 Hz, 1H), 6.50 (d, *J* = 4.3 Hz, 1H), 2.33 (s, 3H).

^{19}F NMR (376 MHz, $CDCl_3$) δ -45.17 (s, 3F).

^{13}C NMR (101 MHz, $CDCl_3$) δ 136.9, 128.8 (q, *J* = 314.1 Hz), 126.1, 123.8, 121.6, 121.6, 118.8, 101.1, 99.4 (q, *J* = 2.1 Hz), 18.7.

HRMS (ESI) m/z: $[M+H]^+$ Calcd for $C_{10}H_9NF_3S$ 232.0402; Found 232.0400.

IR (KBr) 2926, 2858, 1536, 1520, 1457, 1418, 1379, 1331, 1304, 1286, 1247, 1160, 1134, 1108, 1046, 1023, 926, 797, 752, 712, 626, 614, 575, 482, 464, 419 cm^{-1}

2-Methyl-6-phenyl-3-((trifluoromethyl)thio)indolizine (12d)

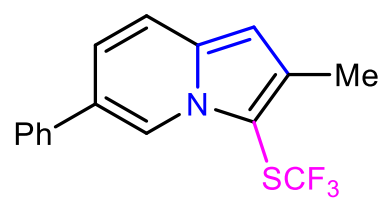

12d

The residue was purified by column chromatography on silica gel with an eluent of petroleum ether (R_f = 0.68) to afford product **12d** (93.6 mg, 61% yield, from **(*E*)-11d**; 95.3 mg, 62% yield, from **(*Z*)-11d**) as a yellow solid, m.p. 74-76 °C.

^{1}H NMR (400 MHz, $CDCl_3$) δ 8.66 (s, 1H), 7.66 – 7.61 (m, 2H), 7.55 – 7.48 (m, 2H), 7.46 – 7.39 (m, 2H), 7.20 (dd, *J* = 9.1, 1.6 Hz, 1H), 6.48 (s, 1H), 2.52 (s, 3H).

^{19}F NMR (376 MHz, $CDCl_3$) δ -44.57 (s, 3F).

^{13}C NMR (101 MHz, $CDCl_3$) δ 138.3, 136.9, 136.5, 129.2, 129.1 (q, *J* = 315.1 Hz), 127.7, 127.0, 125.6, 121.6, 121.5, 118.4, 102.0, 99.5 (q, *J* = 2.2 Hz), 12.7.

HRMS (ESI) m/z: $[M+H]^+$ Calcd for $C_{16}H_{13}NF_3S$ 308.0715; Found 308.0719.

IR (KBr) 3035, 2924, 1600, 1542, 1496, 1486, 1449, 1410, 1390, 1374, 1341, 1322, 1312, 1251, 1214, 1132, 1112, 1079, 1013, 942, 851, 809, 767, 747, 718, 698, 639, 630, 606, 562, 537, 524, 502, 486, 464, 430 cm^{-1}

1-Methyl-3-((trifluoromethyl)thio)indolizine (12e)

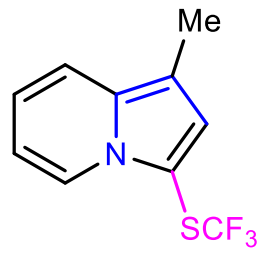

12e

The residue was purified by column chromatography on silica gel with an eluent of petroleum ether (R_f = 0.88) to afford product **12e** (54.3 mg, 47% yield) as a yellow oil.

^{1}H NMR (400 MHz, $CDCl_3$) δ 8.40 (d, J = 7.1 Hz, 1H), 7.39 (d, J = 9.0 Hz, 1H), 7.04 (s, 1H), 6.87 (dd, J = 9.0, 6.5 Hz, 1H), 6.68 (t, J = 6.7 Hz, 1H), 2.36 (s, 3H).

^{19}F NMR (376 MHz, $CDCl_3$) δ -45.26 (s, 3F).

^{13}C NMR (101 MHz, $CDCl_3$) δ 135.9, 128.8 (q, J = 314.3 Hz), 126.8, 123.8, 119.2, 117.5, 111.6, 110.5, 98.2 (q, J = 2.5 Hz), 10.6.

HRMS (FI) m/z: $[M]^+$ Calcd for $C_{10}H_8NF_3S$ 231.0324; Found 231.0328.

IR (KBr) 2925, 2868, 1629, 1544, 1455, 1400, 1357, 1322, 1292, 1247, 1157, 1132, 1110, 1083, 1010, 984, 842, 740, 724, 618, 570, 545, 459, 419 cm^{-1}

1-Phenyl-3-((trifluoromethyl)thio)indolizine (12f)

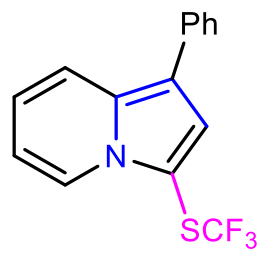

12f

The residue was purified by column chromatography on silica gel with an eluent of petroleum ether (R_f = 0.60) to afford product **12f** (79.1 mg, 54% yield) as a brown solid, m.p. 93-95 °C.

^{1}H NMR (400 MHz, $CDCl_3$) δ 8.53 (d, J = 7.0 Hz, 1H), 7.82 (d, J = 9.0 Hz, 1H), 7.62 (d, J = 7.6 Hz, 2H), 7.50 (t, J = 7.7 Hz, 2H), 7.43 (s, 1H), 7.34 (t, J = 7.3 Hz, 1H), 7.04 – 6.96 (m, 1H), 6.79 (t, J = 6.9 Hz, 1H).

^{19}F NMR (376 MHz, $CDCl_3$) δ -44.71 (s, 3F).

^{13}C NMR (101 MHz, $CDCl_3$) δ 135.2, 134.7, 129.1, 128.8 (q, J = 314.2 Hz), 127.9, 126.4, 125.8, 124.3, 121.4, 118.3, 116.9, 112.4, 100.6 (q, J = 2.1 Hz).

HRMS (FI) m/z: $[M]^+$ Calcd for $C_{15}H_{10}NF_3S$ 293.0481; Found 293.0485.

IR (KBr) 3108, 3052, 1704, 1627, 1599, 1574, 1538, 1519, 1457, 1440, 1399, 1353, 1324, 1309, 1294, 1246, 1191, 1164, 1137, 1104, 1032, 1014, 933, 914, 857, 848, 822, 768, 760, 740, 728, 698, 643, 630, 618, 581, 557, 497, 472, 461, 421 cm^{-1}

7-Methoxy-3-((trifluoromethyl)thio)indolizine (12g)

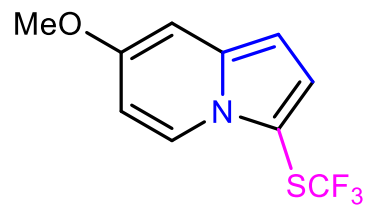

12g

The residue was purified by column chromatography on silica gel with an eluent of petroleum ether (R_f = 0.50) to afford product **12g** (48.2 mg, 39% yield) as a yellow oil.

^{1}H NMR (400 MHz, $CDCl_3$) δ 8.29 (d, J = 7.6 Hz, 1H), 7.13 (d, J = 4.0 Hz, 1H), 6.68 (d, J = 2.5 Hz, 1H), 6.47 (dd, J = 7.6, 2.5 Hz, 1H), 6.34 (d, J = 4.2 Hz, 1H), 3.83 (s, 3H).

^{19}F NMR (376 MHz, $CDCl_3$) δ -45.79 (s, 3F).

^{13}C NMR (101 MHz, $CDCl_3$) δ 154.8, 139.1, 128.7 (q, J = 314.3 Hz), 127.5, 125.2, 107.2, 99.4, 98.0 (q, J = 2.2 Hz), 96.1, 55.5.

HRMS (FI) m/z: $[M]^+$ Calcd for $C_{10}H_8ONF_3S$ 247.0273; Found 247.0268.

IR (KBr) 3099, 3009, 2958, 2936, 2837, 1650, 1539, 1480, 1447, 1368, 1341, 1291, 1248, 1222, 1192, 1161, 1130, 1105, 1048, 1025, 988, 943, 822, 785, 750, 710, 630, 611, 565, 548, 464, 420 cm^{-1}

6-Methoxy-3-((trifluoromethyl)thio)indolizine (12h)

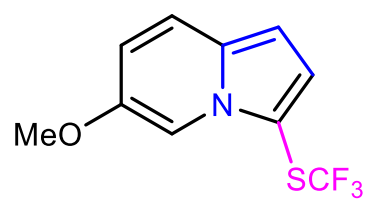

12h

The residue was purified by column chromatography on silica gel with an eluent of petroleum ether (R_f = 0.59) to afford product **12h** (53.1 mg, 43% yield) as a yellow oil.

^{1}H NMR (400 MHz, $CDCl_3$) δ 8.00 (d, J = 2.2 Hz, 1H), 7.33 (d, J = 9.5 Hz, 1H), 7.12 (d, J = 4.2 Hz, 1H), 6.75 (dd, J = 9.5, 2.1 Hz, 1H), 6.51 (d, J = 4.3 Hz, 1H), 3.86 (s, 3H).

^{19}F NMR (376 MHz, $CDCl_3$) δ -44.92 (s, 3F).

^{13}C NMR (101 MHz, $CDCl_3$) δ 149.5, 134.9, 128.8 (q, J = 314.2 Hz), 125.7, 119.7, 116.3, 105.9, 101.7, 100.6 (q, J = 2.3 Hz), 56.1.

HRMS (EI) m/z: $[M]^+$ Calcd for $C_{10}H_8ONF_3S$ 247.0273; Found 247.0274.

IR (KBr) 3008, 2958, 2937, 2839, 1644, 1525, 1456, 1431, 1381, 1332, 1303, 1243,

1218, 1162, 1133, 1106, 1047, 1028, 930, 833, 801, 751, 706, 624, 597, 463, 417 cm^{-1}

2-Methoxy-3-((trifluoromethyl)thio)indolizine (12i)

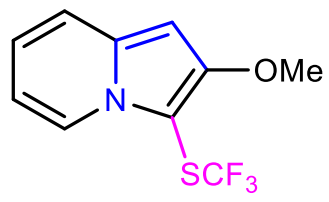

12i

The residue was purified by column chromatography on silica gel with an eluent of petroleum ether (R_f = 0.46) to afford product **12i** (38.3 mg, 31% yield) as a red oil.

^{1}H NMR (400 MHz, $CDCl_3$) δ 8.37 (d, J = 7.0 Hz, 1H), 7.30 (dt, J = 8.8, 1.2 Hz, 1H), 6.94 (ddd, J = 8.9, 6.7, 1.1 Hz, 1H), 6.69 (td, J = 6.8, 1.3 Hz, 1H), 6.14 (s, 1H), 3.96 (s, 3H).

^{19}F NMR (376 MHz, $CDCl_3$) δ -45.35 (s, 3F).

^{13}C NMR (101 MHz, $CDCl_3$) δ 158.8, 136.2, 128.8 (q, J = 316.1 Hz), 123.7, 121.6, 117.9, 111.2, 86.1, 85.1, 58.2.

HRMS (FI) m/z: $[M]^+$ Calcd for $C_{10}H_8ONF_3S$ 247.0273; Found 247.0272.

IR (KBr) 3010, 2944, 2892, 1634, 1541, 1522, 1501, 1463, 1454, 1408, 1346, 1329, 1275, 1245, 1201, 1155, 1135, 1113, 1099, 1027, 919, 828, 754, 729, 713, 695, 662, 568, 496, 474, 462, 437 cm^{-1}

8-Bromo-3-((trifluoromethyl)thio)indolizine (12j)

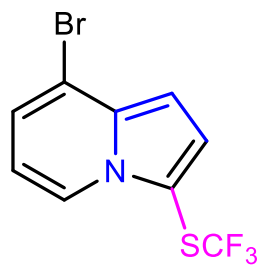

12j

The residue was purified by column chromatography on silica gel with an eluent of petroleum ether (R_f = 0.79) to afford product **12j** (87.0 mg, 59% yield) as a yellow oil.

^{1}H NMR (400 MHz, $CDCl_3$) δ 8.43 (d, J = 7.0 Hz, 1H), 7.28 (d, J = 4.3 Hz, 1H), 7.17 (d, J = 7.1 Hz, 1H), 6.74 (d, J = 4.4 Hz, 1H), 6.62 (t, J = 7.1 Hz, 1H).

^{19}F NMR (376 MHz, $CDCl_3$) δ -44.65 (s, 3F).

^{13}C NMR (101 MHz, $CDCl_3$) δ 136.9, 128.7 (q, J = 313.8 Hz), 127.1, 123.2, 123.1, 113.6, 111.9, 103.3, 102.7 (q, J = 2.4 Hz).

HRMS (ESI) m/z: $[M+H]^+$ Calcd for $C_9H_6NBrF_3S$ 295.9351; Found 295.9350.

IR (KBr) 2954, 2925, 2855, 1625, 1518, 1502, 1446, 1428, 1376, 1340, 1287, 1184, 1166, 1136, 1107, 1058, 1041, 995, 887, 872, 826, 768, 751, 704, 648, 623, 562, 514, 499, 461 cm^{-1}

7-Bromo-3-((trifluoromethyl)thio)indolizine (12k)

3,7-Bis((trifluoromethyl)thio)indolizine (12k')

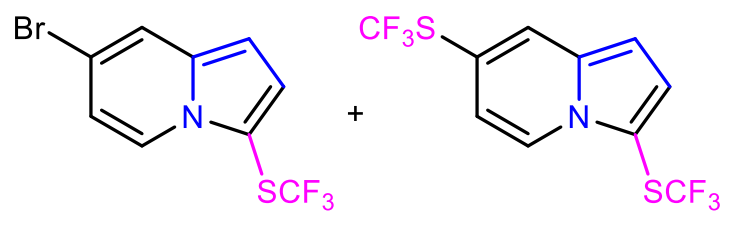

12k **12k'**

The residue was purified by column chromatography on silica gel with an eluent of petroleum ether (R_f = 0.79), followed by HPLC purification (Material: 5μm C18, Dimensions: 20×250mm, CH_3CN/H_2O = 80:20, flow rate 16.0 mL/min, λ = 220 nm, 25 ºC) to afford product **12k** (70.8 mg, 48% yield, t_R = 3.711 min) as a brown oil and **12k'** (15.8 mg, 10% yield, t_R = 4.122 min) as a yellow oil.

For the product **12k**:

^{1}H NMR (400 MHz, $CDCl_3$) δ 8.29 (d, J = 7.4 Hz, 1H), 7.62 (dd, J = 2.0, 0.8 Hz, 1H), 7.19 (d, J = 4.3 Hz, 1H), 6.80 (dd, J = 7.4, 2.0 Hz, 1H), 6.50 (dd, J = 4.3, 0.9 Hz, 1H).

^{19}F NMR (376 MHz, $CDCl_3$) δ -44.86 (s, 3F).

^{13}C NMR (101 MHz, $CDCl_3$) δ 138.2, 128.5 (q, J = 313.9 Hz), 127.5, 124.7, 121.5, 115.8, 114.4, 101.4, 101.2 (q, J = 2.1 Hz).

HRMS (FI) m/z: $[M]^+$ Calcd for $C_9H_5NBrF_3S$ 294.9273; Found 294.9271.

IR (KBr) 1621, 1511, 1433, 1364, 1331, 1278, 1261, 1243, 1188, 1166, 1144, 1133, 1106, 1056, 1046, 983, 902, 858, 774, 755, 706, 698, 619, 606, 489, 462, 413 cm^{-1}

For the product **12k'**:

^{1}H NMR (400 MHz, $CDCl_3$) δ 8.44 (d, J = 7.3 Hz, 1H), 7.82 (t, J = 1.3 Hz, 1H), 7.28 (d, J = 4.3 Hz, 1H), 6.90 (dd, J = 7.3, 1.9 Hz, 1H), 6.71 (dd, J = 4.3, 0.9 Hz, 1H).

^{19}F NMR (376 MHz, $CDCl_3$) δ -42.50 (s, 3F), -44.32 (s, 3F).

^{13}C NMR (101 MHz, $CDCl_3$) δ 137.0, 129.6 (q, J = 309.1 Hz), 128.7, 128.6 (q, J = 313.8 Hz), 127.6, 124.3, 117.2, 116.2 (q, J = 2.3 Hz), 104.1, 103.2 (q, J = 3.3 Hz).

HRMS (FI) m/z: $[M]^+$ Calcd for $C_{10}H_5NF_6S_2$ 316.9762; Found 316.9767.

IR (KBr) 2954, 2927, 2856, 1513, 1453, 1431, 1334, 1281, 1265, 1246, 1162, 1133,

1107, 1066, 1047, 982, 874, 782, 762, 711, 625, 496, 472, 418 cm^{-1}

7-Nitro-3-((trifluoromethyl)thio)indolizine (12l)

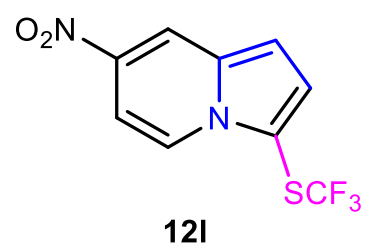

12l

The residue was purified by column chromatography on silica gel with an eluent of petroleum ether/EA (30:1, R_f = 0.27) to afford product **12l** (58.9 mg, 45% yield) as a yellow solid, m.p. 69-71 ºC.

^{1}H NMR (400 MHz, $CDCl_3$) δ 8.56 – 8.41 (m, 2H), 7.53 (dd, *J* = 7.7, 2.4 Hz, 1H), 7.37 (d, *J* = 4.4 Hz, 1H), 7.03 (d, *J* = 4.3 Hz, 1H).

^{19}F NMR (376 MHz, $CDCl_3$) δ -43.61 (s, 3F).

^{13}C NMR (101 MHz, $CDCl_3$) δ 140.8, 134.8, 128.8, 128.4 (q, *J* = 313.7 Hz), 124.3, 117.2, 109.2, 106.5 (q, *J* = 2.5 Hz), 105.9.

HRMS (ESI) m/z: $[M+H]^+$ Calcd for $C_9H_6O_2N_2F_3S$ 263.0097; Found 263.0095.

IR (KBr) 3150, 3119, 3102, 3048, 2958, 1623, 1564, 1520, 1504, 1454, 1430, 1383, 1340, 1279, 1269, 1242, 1191, 1167, 1141, 1104, 1073, 1050, 986, 932, 900, 836, 768, 753, 739, 701, 626, 610, 544, 464, 420 cm^{-1}

3-((Trifluoromethyl)thio)indolizine-7-carbonitrile (12m)

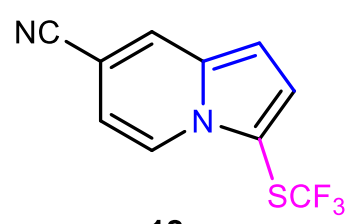

12m

The residue was purified by column chromatography on silica gel with an eluent of petroleum ether/EA (20:1, R_f = 0.35) to afford product **12m** (81.1 mg, 67% yield) as a brown solid, m.p. 63-65 ºC.

^{1}H NMR (400 MHz, $CDCl_3$) δ 8.46 (d, *J* = 7.3 Hz, 1H), 7.86 (s, 1H), 7.32 (d, *J* = 4.4 Hz, 1H), 6.89 – 6.77 (m, 2H).

^{19}F NMR (376 MHz, $CDCl_3$) δ -43.91 (s, 3F).

^{13}C NMR (101 MHz, $CDCl_3$) δ 135.4, 128.4 (q, *J* = 313.7 Hz), 128.0, 126.2, 124.5, 118.2, 111.6, 106.0, 105.2 (q, *J* = 2.4 Hz), 103.4.

HRMS (ESI) m/z: $[M+H]^+$ Calcd for $C_{10}H_6N_2F_3S$ 243.0198; Found 243.0201.

IR (KBr) 3133, 3079, 3051, 2225, 1772, 1627, 1557, 1519, 1464, 1428, 1377, 1337, 1282, 1250, 1162, 1126, 1105, 1041, 983, 925, 897, 883, 780, 769, 748, 710, 627, 562, 480, 460, 415 cm^{-1}

Methyl 3-((trifluoromethyl)thio)indolizine-7-carboxylate (12n)

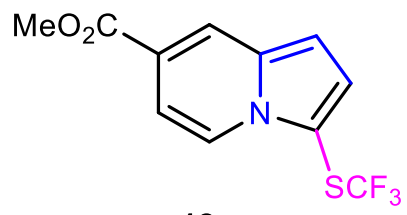

12n

The residue was purified by column chromatography on silica gel with an eluent of petroleum ether/EA (40:1, R_f = 0.32) to afford product **12n** (55.0 mg, 40% yield) as a brown solid, m.p. 70-72 ºC.

^{1}H NMR (400 MHz, $CDCl_3$) δ 8.41 (d, J = 7.4 Hz, 1H), 8.24 – 8.18 (m, 1H), 7.32 – 7.22 (m, 2H), 6.79 (dd, J = 4.4, 0.9 Hz, 1H), 3.93 (s, 3H).

^{19}F NMR (376 MHz, $CDCl_3$) δ -44.30 (s, 3F).

^{13}C NMR (101 MHz, $CDCl_3$) δ 166.0, 136.5, 128.6 (q, J = 313.5 Hz), 127.4, 123.5, 122.8, 122.1, 111.0, 105.8, 103.6 (q, J = 2.4 Hz), 52.5.

HRMS (ESI) m/z: $[M+H]^+$ Calcd for $C_{11}H_9O_2NF_3S$ 276.0301; Found 276.0304.

IR (KBr) 2954, 1722, 1632, 1523, 1511, 1471, 1435, 1339, 1309, 1282, 1253, 1178, 1136, 1108, 1048, 994, 967, 905, 816, 753, 710, 694, 624, 521, 465, 422 cm^{-1}

Ethyl 3-((trifluoromethyl)thio)indolizine-2-carboxylate (12o)

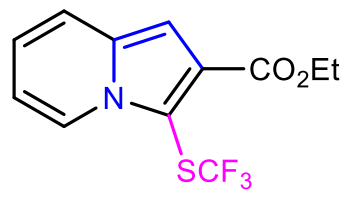

12o

The residue was purified by column chromatography on silica gel with an eluent of petroleum ether/EA (40:1, R_f = 0.22) to afford product **12o** (104.1 mg, 72% yield) as a brown oil.

^{1}H NMR (400 MHz, $CDCl_3$) δ 8.50 (d, J = 7.2 Hz, 1H), 7.44 (dt, J = 8.9, 1.2 Hz, 1H), 7.06 (s, 1H), 6.94 (ddd, J = 9.0, 6.6, 1.0 Hz, 1H), 6.83 – 6.75 (m, 1H), 4.40 (q, J = 7.1 Hz, 2H), 1.40 (t, J = 7.1 Hz, 3H).

^{19}F NMR (376 MHz, $CDCl_3$) δ -43.65 (s, 3F).

^{13}C NMR (101 MHz, $CDCl_3$) δ 163.7, 136.4, 128.8 (q, J = 314.3 Hz), 127.8, 124.1, 121.6, 120.3, 113.9, 104.7, 102.0 (q, J = 2.3 Hz), 60.9, 14.3.

HRMS (ESI) m/z: $[M+H]^+$ Calcd for $C_{12}H_{11}O_2NF_3S$ 290.0457; Found 290.0455.

IR (KBr) 2984, 2941, 2906, 1724, 1635, 1538, 1508, 1486, 1445, 1386, 1365, 1348, 1332, 1280, 1247, 1211, 1156, 1135, 1104, 1054, 1015, 855, 833, 808, 767, 751, 737, 724, 565, 473, 463, 430 cm^{-1}

Ethyl 3-((trifluoromethyl)thio)indolizine-1-carboxylate (12p)

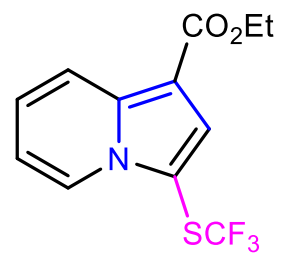

12p

The residue was purified by column chromatography on silica gel with an eluent of petroleum ether/EA (40:1, R_f = 0.31) to afford product **12p** (118.5 mg, 82% yield) as a brown oil.

^{1}H NMR (400 MHz, $CDCl_3$) δ 8.48 (d, J = 7.0 Hz, 1H), 8.26 (dt, J = 9.0, 1.2 Hz, 1H), 7.69 (s, 1H), 7.22 (ddd, J = 9.0, 6.8, 1.2 Hz, 1H), 6.90 (td, J = 6.9, 1.3 Hz, 1H), 4.36 (q, J = 7.1 Hz, 2H), 1.39 (t, J = 7.1 Hz, 3H).

^{19}F NMR (376 MHz, $CDCl_3$) δ -44.54 (s, 3F).

^{13}C NMR (101 MHz, $CDCl_3$) δ 164.0, 139.5, 129.3, 128.5 (q, J = 313.6 Hz), 125.0, 124.6, 120.1, 113.9, 106.1, 102.5 (q, J = 2.5 Hz), 60.1, 14.7.

HRMS (FI) m/z: $[M]^+$ Calcd for $C_{12}H_{10}O_2NF_3S$ 289.0379; Found 289.0380.

IR (KBr) 3112, 2982, 2936, 1703, 1635, 1531, 1518, 1470, 1448, 1413, 1387, 1365, 1283, 1222, 1166, 1137, 1104, 1055, 1017, 970, 868, 843, 778, 745, 733, 621, 605, 570, 554, 479, 465, 423 cm^{-1}

1-((Trifluoromethyl)thio)benzo[*d*]pyrrolo[2,1-*b*]thiazole (12q)

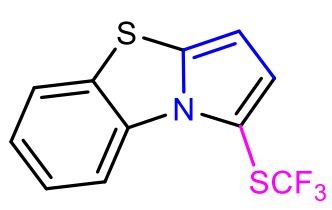

12q

The residue was purified by column chromatography on silica gel with an eluent of petroleum ether (R_f = 0.71) to afford product **12q** (76.4 mg, 56% yield) as a yellow solid, m.p. 65-67 °C.

^{1}H NMR (400 MHz, $CDCl_3$) δ 8.60 (d, *J* = 8.3 Hz, 1H), 7.63 (dd, *J* = 7.9, 1.3 Hz, 1H), 7.42 (ddd, *J* = 8.4, 7.3, 1.2 Hz, 1H), 7.30 (td, *J* = 7.7, 1.1 Hz, 1H), 7.03 (d, *J* = 4.0 Hz, 1H), 6.39 (d, *J* = 4.0 Hz, 1H).

^{19}F NMR (376 MHz, $CDCl_3$) δ -45.91 (s, 3F).

^{13}C NMR (101 MHz, $CDCl_3$) δ 135.64, 135.56, 131.5, 130.2, 128.3 (q, *J* = 313.3 Hz), 125.8, 124.4, 123.9, 114.6, 102.6 (q, *J* = 2.7 Hz), 101.4.

HRMS (ESI) m/z: $[M+H]^+$ Calcd for $C_{11}H_7NF_3S_2$ 273.9967; Found 273.9965.

IR (KBr) 3121, 3108, 2924, 1942, 1900, 1860, 1776, 1710, 1579, 1512, 1504, 1469, 1458, 1407, 1373, 1342, 1310, 1291, 1247, 1172, 1159, 1142, 1132, 1107, 1077, 1031, 973, 944, 931, 861, 852, 758, 744, 716, 684, 649, 627, 605, 557, 476, 459, 423 cm^{-1}

2-Phenyl-6-((trifluoromethyl)thio)pyrrolo[1,2-*a*]pyrimidine (12r)

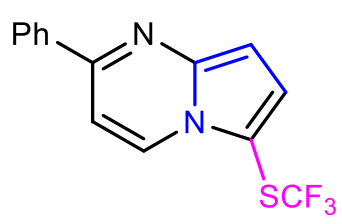

12r

The residue was purified by column chromatography on silica gel with an eluent of petroleum ether/EA (40:1, R_f = 0.35), followed by HPLC purification (Material: 5μm C18, Dimensions: 20×250mm, CH_3CN/H_2O = 80:20, flow rate 16.0 mL/min, λ = 220 nm, 25 °C, t_R = 10.855 min) to afford product **12r** (73.5 mg, 50% yield) as a yellow solid, m.p. 124-126 °C.

^{1}H NMR (400 MHz, $CDCl_3$) δ 8.65 (d, *J* = 7.4 Hz, 1H), 8.12 – 8.04 (m, 2H), 7.55 – 7.44 (m, 3H), 7.41 (d, *J* = 4.3 Hz, 1H), 7.23 (d, *J* = 7.5 Hz, 1H), 6.79 (dd, *J* = 4.3, 0.9 Hz, 1H).

^{19}F NMR (376 MHz, $CDCl_3$) δ -44.71 (s, 3F).

^{13}C NMR (101 MHz, $CDCl_3$) δ 153.1, 144.9, 137.5, 130.9, 130.3, 129.5, 129.1, 128.6 (q, *J* = 313.8 Hz), 127.2, 105.7, 101.8, 98.1 (q, *J* = 2.1 Hz).

HRMS (FI) m/z: $[M]^+$ Calcd for $C_{14}H_9N_2F_3S$ 294.0433; Found 294.0438.

IR (KBr) 3090, 1614, 1532, 1507, 1491, 1456, 1410, 1361, 1315, 1280, 1259, 1240, 1188, 1163, 1146, 1129, 1110, 1057, 1035, 925, 810, 795, 764, 689, 656, 615, 590, 532, 462 cm^{-1}

(6-Methoxy-3-((trifluoromethyl)thio)indolizin-1-yl)(3,4,5-trimethoxyphenyl)methanone (12t)

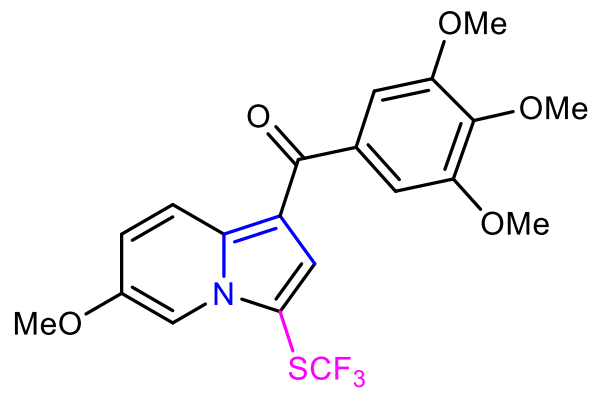

12t

The residue was purified by column chromatography on silica gel with an eluent of petroleum ether/EA (3:1, $R_f = 0.20$) to afford product **12t** (154.4 mg, 70% yield) as a yellow solid, m.p. 140-142 °C.

^{1}H NMR (400 MHz, $CDCl_3$) δ 8.36 (d, $J = 9.6$ Hz, 1H), 8.03 (d, $J = 2.3$ Hz, 1H), 7.46 (s, 1H), 7.08 (dd, $J = 9.7, 2.2$ Hz, 1H), 7.04 (s, 2H), 3.88 (s, 3H), 3.87 – 3.80 (m, 9H).

^{19}F NMR (376 MHz, $CDCl_3$) δ -44.58 (s, 3F).

^{13}C NMR (101 MHz, $CDCl_3$) δ 188.6, 153.0, 151.2, 141.0, 136.6, 135.3, 130.5, 128.4 (q, $J = 313.9$ Hz), 121.01, 120.96, 113.7, 106.7, 106.5, 102.8 (q, $J = 2.5$ Hz), 60.9, 56.2, 56.1.

HRMS (ESI) m/z: $[M+H]^+$ Calcd for $C_{20}H_{19}O_5NF_3S$ 442.0931; Found 442.0931.

IR (KBr) 3105, 3002, 2940, 2838, 2637, 2250, 1697, 1621, 1582, 1505, 1470, 1414, 1328, 1293, 1223, 1127, 1027, 998, 962, 912, 884, 861, 819, 802, 769, 755, 730, 688, 651, 632, 608, 564, 540, 468, 424 cm^{-1}

7 Gram-scale experiments

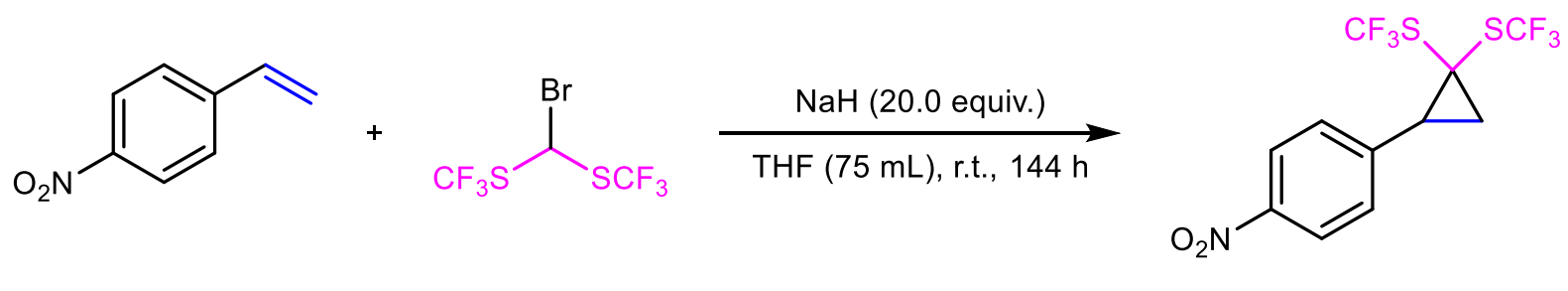

A 250 mL bottom flask was charged with substrates **1j** (5.0 mmol, 745.8 mg), NaH (2.4 g, 100 mmol, 20.0 equiv.) and dry THF (75 mL) under a N_2 atmosphere. $BrCH(SCF_3)_2$ (3.69 g, 12.5 mmol, 2.5 equiv.) was added every 36 h. The resulting mixture was stirred at room temperature for total 144 h under a N_2 atmosphere. The resulting mixture was then diluted with ethyl acetate (200 mL) and saturated NH_4Cl solution (300 mL). The

organic phase was separated and the aqueous phase was extracted 3 times. All organic solutions were combined and then dried over anhydrous Na_2SO_4. The solvent was removed under vacuum and the residue was purified by column chromatography on silica gel with an eluent of petroleum ether to petroleum ether/EA (50:1) to afford product **2j** (1.05 g, 58% yield) as a yellow oil.

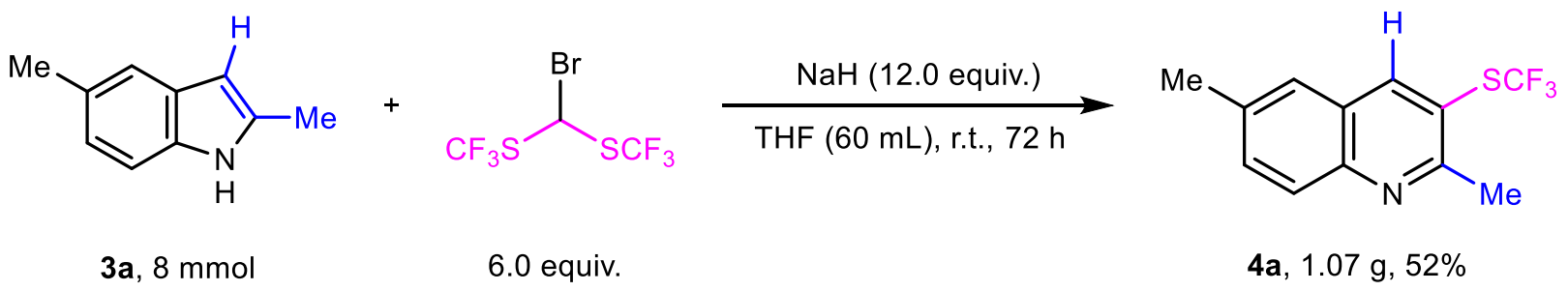

A 250 mL bottom flask was charged with substrates **3a** (8.0 mmol, 1.16 g), NaH (2.3 g, 96 mmol, 12.0 equiv.) and dry THF (60 mL) under a N_2 atmosphere. $BrCH(SCF_3)_2$ (7.08 g, 24 mmol, 3.0 equiv.) was added every 36 h. The resulting mixture was stirred at room temperature for total 72 h under a N_2 atmosphere. The resulting mixture was then diluted with ethyl acetate (200 mL) and saturated NH_4Cl solution (300 mL). The organic phase was separated and the aqueous phase was extracted 3 times. All organic solutions were combined and then dried over anhydrous Na_2SO_4. The solvent was removed under vacuum and the residue was purified by column chromatography on silica gel with an eluent of petroleum ether/EA (30:1 to 10:1) to afford product **4a** (1.07 g, 52% yield) as a yellow solid.

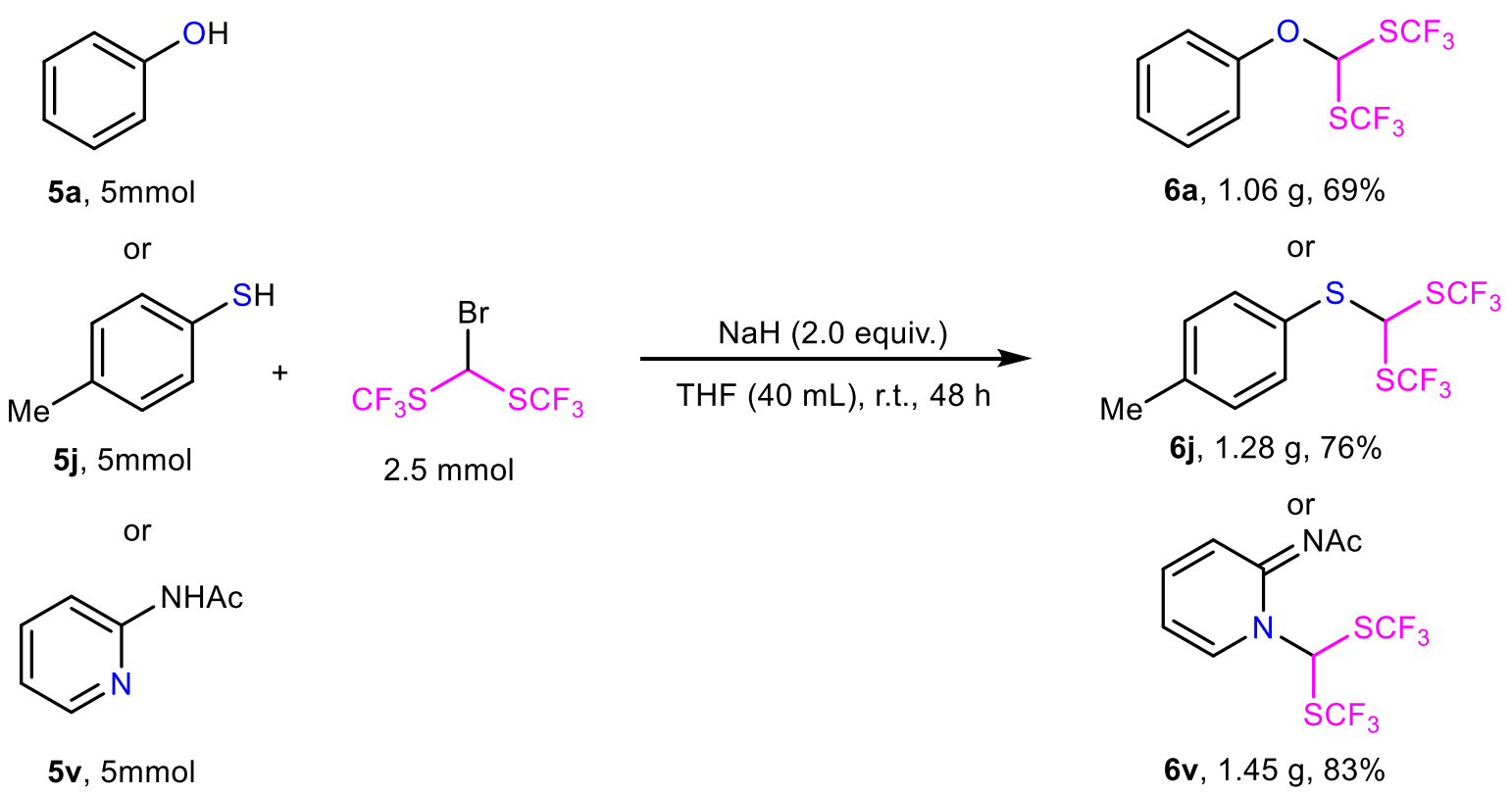

A 100 mL bottom flask was charged with substrates **5a**, **5j** or **5v** (5.0 mmol, 470 mg for **5a**, 620 mg for **5j**, 680 mg for **5v**), NaH (240 mg, 10 mmol, 2.0 equiv.) and dry THF (60 mL) under a N_2 atmosphere. $BrCH(SCF_3)_2$ (3.69 g, 12.5 mmol, 2.5 equiv.) was then added. The resulting mixture was stirred at room temperature for 48 h under a N_2

atmosphere. The resulting mixture was then diluted with ethyl acetate (100 mL) and saturated NH_4Cl solution (150 mL). The organic phase was separated and the aqueous phase was extracted 3 times. All organic solutions were combined and then dried over anhydrous Na_2SO_4. The solvent was removed under vacuum and the residue was purified by column chromatography on silica gel with an eluent of petroleum ether to afford product **6a** (1.06 g, 69% yield) as a yellow oil, **6j** (1.28 g, 76% yield) as a yellow oil or with an eluent of petroleum ether/EA (10:1 to 3:1) to afford product **6v** (1.45 g, 83% yield) as a white solid.

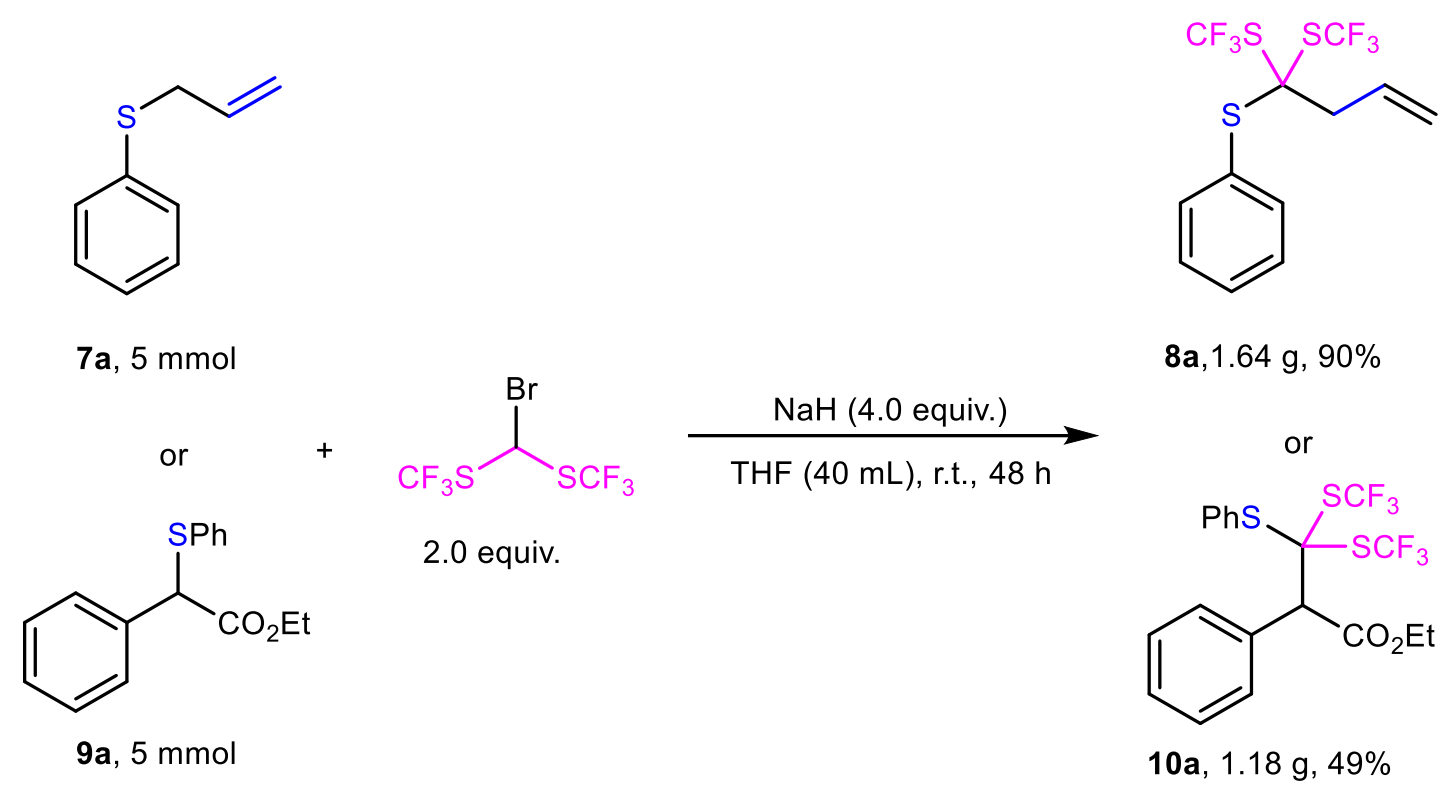

A 100 mL bottom flask was charged with substrates **7a** or **9a** (5.0 mmol, 750 mg for **7a**, 1.36 g for **9a**), NaH (480 mg, 20 mmol, 4.0 equiv.) and dry THF (40 mL) under a N_2 atmosphere. $BrCH(SCF_3)_2$ (2.95 g, 10.0 mmol, 2.0 equiv.) was then added. The resulting mixture was stirred at room temperature for 48 h under a N_2 atmosphere. The resulting mixture was then diluted with ethyl acetate (100 mL) and saturated NH_4Cl solution (150 mL). The organic phase was separated and the aqueous phase was extracted 3 times. All organic solutions were combined and then dried over anhydrous Na_2SO_4. The solvent was removed under vacuum and the residue was purified by column chromatography on silica gel with an eluent of petroleum ether to afford product **8a** (1.64 g, 90% yield) as a yellow oil or with an eluent of petroleum ether/EA (40:1) to afford product **10a** (1.18 g, 49% yield) as a yellow oil.

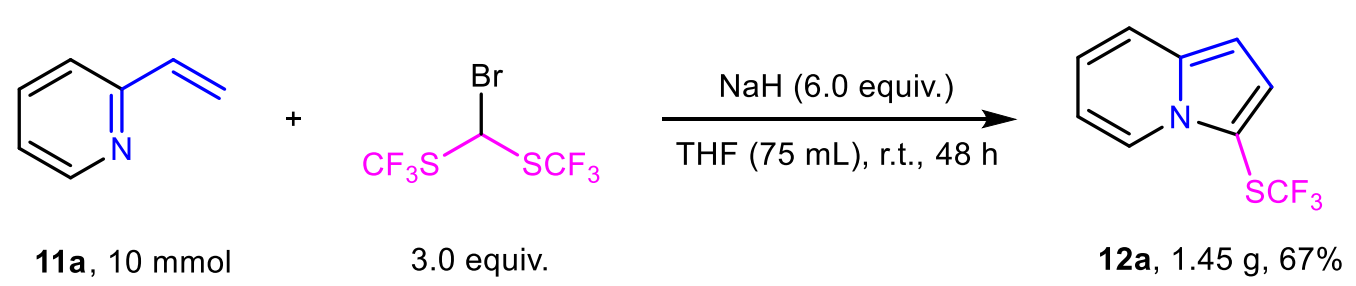

A 250 mL bottom flask was charged with substrates **11a** (10.0 mmol, 1.05 g), NaH (1.44

g, 60 mmol, 6.0 equiv.) and dry THF (75 mL) under a N_2 atmosphere. $BrCH(SCF_3)_2$ (8.85 g, 30.0 mmol, 3.0 equiv.) was then added. The resulting mixture was stirred at room temperature for 48 h under a N_2 atmosphere. The resulting mixture was then diluted with ethyl acetate (200 mL) and saturated NH_4Cl solution (300 mL). The organic phase was separated and the aqueous phase was extracted 3 times. All organic solutions were combined and then dried over anhydrous Na_2SO_4. The solvent was removed under vacuum and the residue was purified by column chromatography on silica gel with an eluent of petroleum ether to afford product **12a** (1.45 g, 67% yield) as a yellow solid.

8 Crystal data and structure refinement

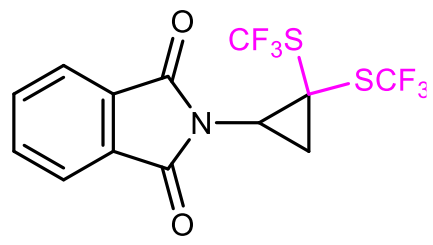

2p

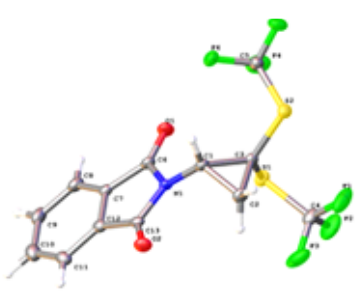

X-ray structure of **2p**
CCDC 2516953

Experimental. The crystal structure of **2p** has been deposited at the Cambridge Crystallographic Data Centre (CCDC 2516953). The compound **2p** was crystallized from DCM solution as the solvent slowly evaporated, affording colorless block crystals. A suitable crystal with dimensions 0.17 × 0.17 × 0.05 mm^3 was selected and mounted on a Bruker D8 Venture diffractometer using Ga K_α radiation (λ = 1.34139 Å). The crystal was kept at a steady T = 170.00 K during data collection. The structure was solved with SHELXT 2018/2 (Sheldrick, 2018) solution program using intrinsic phasing and refined with SHELXL 2018/3 (Sheldrick, 2015) by full-matrix least-squares minimization on $\boldsymbol{F^2}$. All non-hydrogen atoms were refined anisotropically. Hydrogen atoms were placed in geometrically calculated positions and refined using a riding model.

Crystal Data. $C_{13}H_7F_6NO_2S_2$, M_r = 387.32, orthorhombic, $P2_12_12_1$ (No. 19), a = 6.9706(2) Å, b = 14.5642(4) Å, c = 15.1331(4) Å, $\alpha = \beta = \gamma = 90°$, V = 1536.33(7) $Å^3$, T = 170.00 K, Z = 4, Z' = 1, μ(Ga K_α) = 2.536 mm^{-1}, 11551 reflections measured, 2903 unique (R_{int} = 0.0541) which were used in all calculations. Multi-scan absorption correction using SADABS-2016/2 was applied (T_{min} = 0.5253, T_{max} = 0.7508). The final wR_2 was 0.0801 (all data) and R_1 was 0.0351 [I ≥ 2σ(I)]. The absolute structure parameter (Flack x) was −0.027(14). The largest diff. peak and hole were 0.291 and −0.227 e·$Å^{-3}$.

Identification code	mj25026_0m
Empirical formula	C13 H7 F6 N O2 S2
Formula weight	387.32
Temperature	170.00 K

Wavelength	1.34139 Å	
Crystal system	Orthorhombic	
Space group	$P2_12_12_1$	
Unit cell dimensions	a = 6.9706(2) Å	a= 90°.
	b = 14.5642(4) Å	b= 90°.
	c = 15.1331(4) Å	g = 90°.
Volume	1536.33(7) $Å^3$	
Z	4	
Density (calculated)	1.675 Mg/m^3	
Absorption coefficient	2.536 mm^{-1}	
F(000)	776	
Crystal size	0.17 x 0.17 x 0.05 mm^3	
Theta range for data collection	3.664 to 54.868°.	
Index ranges	-8<=h<=8, -17<=k<=13, -14<=l<=18	
Reflections collected	11551	
Independent reflections	2903 [R(int) = 0.0541]	
Completeness to theta = 53.594°	99.8 %	
Absorption correction	Semi-empirical from equivalents	
Max. and min. transmission	0.7508 and 0.5253	
Refinement method	Full-matrix least-squares on F^2	
Data / restraints / parameters	2903 / 0 / 217	
Goodness-of-fit on F^2	1.022	
Final R indices [I>2sigma(I)]	R1 = 0.0351, wR2 = 0.0746	
R indices (all data)	R1 = 0.0464, wR2 = 0.0801	
Absolute structure parameter	-0.027(14)	
Extinction coefficient	n/a	
Largest diff. peak and hole	0.291 and -0.227 e.$Å^{-3}$	

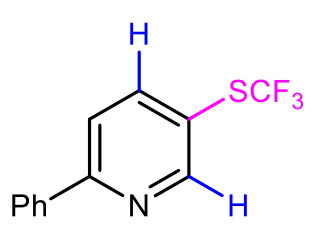

4t

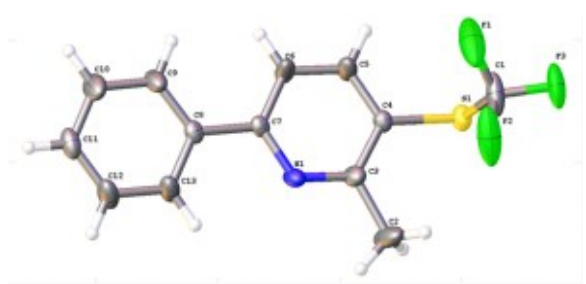

X-ray structure of **4t**
CCDC 2516954

Experimental. The crystal structure of **4t** has been deposited at the Cambridge Crystallographic Data Centre (CCDC 2516954). The compound **4t** was crystallized from DCM solution as the solvent slowly evaporated, affording colorless block crystals. A suitable crystal with dimensions 0.17 × 0.17 × 0.05 mm^3 was selected and mounted on a Bruker D8 Venture diffractometer using Ga K_α radiation (λ = 1.34139 Å). The crystal was kept at a steady T = 170.00 K during data collection. The structure was solved with SHELXT 2018/2 (Sheldrick, 2018) solution program using intrinsic phasing and refined with SHELXL 2018/3 (Sheldrick, 2015) by full-matrix least-squares minimization on $\boldsymbol{F^2}$.

All non-hydrogen atoms were refined anisotropically. Hydrogen atoms were placed in geometrically calculated positions and refined using a riding model.

Crystal Data. $C_{13}H_{10}F_3NS$, M_r = 269.28, monoclinic, P2$_1$/c (No. 14), a = 11.7781(6) Å, b = 10.6093(5) Å, c = 10.0368(5) Å, β = 101.493(2)°, V = 1229.02(11) Å^3, T = 170.00 K, Z = 4, Z' = 1, μ(Ga K$_\alpha$) = 1.698 mm^{-1}, 15469 reflections measured, 2333 unique (R_{int} = 0.0440) which were used in all calculations. Multi-scan absorption correction using SADABS-2016/2 was applied (T_{min} = 0.5834, T_{max} = 0.7508). The final wR_2 was 0.1475 (all data) and R_1 was 0.0649 [I ≥ 2σ(I)]. The largest diff. peak and hole were 0.630 and −0.703 e·Å^{-3}.

Identification code	mj25392	
Empirical formula	C13 H10 F3 N S	
Formula weight	269.28	
Temperature	170.00 K	
Wavelength	1.34139 Å	
Crystal system	Monoclinic	
Space group	P 1 21/c 1	
Unit cell dimensions	a = 11.7781(6) Å	α= 90°.
	b = 10.6093(5) Å	β= 101.493(2)°.
	c = 10.0368(5) Å	γ = 90°.
Volume	1229.02(11) Å^3	
Z	4	
Density (calculated)	1.455 Mg/m^3	
Absorption coefficient	1.698 mm^{-1}	
F(000)	552	
Crystal size	0.17 x 0.17 x 0.05 mm^3	
Theta range for data collection	3.331 to 54.939°.	
Index ranges	-13<=h<=14, -12<=k<=12, -11<=l<=12	
Reflections collected	15469	
Independent reflections	2333 [R(int) = 0.0440]	
Completeness to theta = 53.594°	99.7 %	
Absorption correction	Semi-empirical from equivalents	
Max. and min. transmission	0.7508 and 0.5834	
Refinement method	Full-matrix least-squares on F^2	
Data / restraints / parameters	2333 / 24 / 164	
Goodness-of-fit on F^2	1.081	
Final R indices [I>2sigma(I)]	R1 = 0.0649, wR2 = 0.1386	
R indices (all data)	R1 = 0.0839, wR2 = 0.1475	
Extinction coefficient	n/a	
Largest diff. peak and hole	0.630 and -0.703 e.Å^{-3}	

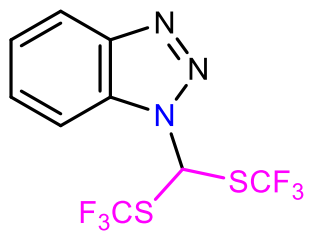

6v

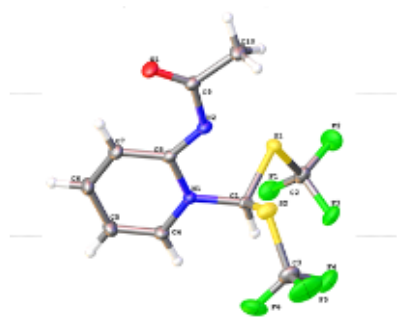
X-ray of structure of **6v**
CCDC 2516955

Experimental. The crystal structure of **6v** has been deposited at the Cambridge Crystallographic Data Centre (CCDC 2516955). The compound **6v** was crystallized from DCM solution as the solvent slowly evaporated, affording colorless block crystals. A suitable crystal with dimensions 0.17 × 0.17 × 0.05 mm^3 was selected and mounted on a Bruker D8 Venture diffractometer using Ga K_α radiation (λ = 1.34139 Å). The crystal was kept at a steady T = 170.00 K during data collection. The structure was solved with SHELXT 2018/2 (Sheldrick, 2018) solution program using intrinsic phasing and refined with SHELXL 2018/3 (Sheldrick, 2015) by full-matrix least-squares minimization on $\boldsymbol{F^2}$. All non-hydrogen atoms were refined anisotropically. Hydrogen atoms were placed in geometrically calculated positions and refined using a riding model.

Crystal Data. $C_{10}H_8F_6N_2OS_2$, M_r = 350.30, monoclinic, C2/c (No. 15), a = 15.1440(5) Å, b = 15.5457(5) Å, c = 12.9955(4) Å, β = 113.0080(10)°, V = 2816.07(16) Å^3, T = 170.00 K, Z = 8, Z' = 1, μ(Ga K_α) = 2.694 mm^{-1}, 17941 reflections measured, 2674 unique (R_{int} = 0.0502) which were used in all calculations. Multi-scan absorption correction using SADABS-2016/2 was applied (T_{min} = 0.5652, T_{max} = 0.7508). The final wR_2 was 0.0844 (all data) and R_1 was 0.0340 [I ≥ 2σ(I)]. The largest diff. peak and hole were 0.276 and −0.273 e·Å^{-3}.

Identification code	mj25053_0m	
Empirical formula	C10 H8 F6 N2 O S2	
Formula weight	350.30	
Temperature	170.00 K	
Wavelength	1.34139 Å	
Crystal system	Monoclinic	
Space group	C 1 2/c 1	
Unit cell dimensions	a = 15.1440(5) Å	α= 90°.
	b = 15.5457(5) Å	β= 113.0080(10)°.
	c = 12.9955(4) Å	γ = 90°.
Volume	2816.07(16) Å^3	
Z	8	
Density (calculated)	1.652 Mg/m^3	
Absorption coefficient	2.694 mm^{-1}	
F(000)	1408	
Crystal size	0.17 x 0.17 x 0.05 mm^3	
Theta range for data collection	3.705 to 54.953°.	

Index ranges	-18<=h<=18, -18<=k<=18, -15<=l<=15
Reflections collected	17941
Independent reflections	2674 [R(int) = 0.0502]
Completeness to theta = 53.594°	99.6 %
Absorption correction	Semi-empirical from equivalents
Max. and min. transmission	0.7508 and 0.5652
Refinement method	Full-matrix least-squares on F^2
Data / restraints / parameters	2674 / 0 / 191
Goodness-of-fit on F^2	1.075
Final R indices [I>2sigma(I)]	R1 = 0.0340, wR2 = 0.0810
R indices (all data)	R1 = 0.0405, wR2 = 0.0844
Extinction coefficient	n/a
Largest diff. peak and hole	0.276 and -0.273 e.Å^{-3}

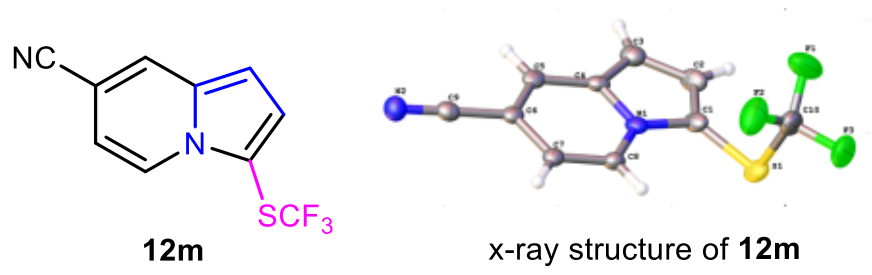

Experimental. The crystal structure of **12m** has been deposited at the Cambridge Crystallographic Data Centre (CCDC 2516956). The compound **12m** was crystallized from DCM solution as the solvent slowly evaporated, affording colorless block crystals. A suitable crystal with dimensions 0.17 × 0.17 × 0.05 mm^3 was selected and mounted on a Bruker D8 Venture diffractometer using Ga K_α radiation (λ = 1.34139 Å). The crystal was kept at a steady T = 170.00 K during data collection. The structure was solved with SHELXT 2018/2 (Sheldrick, 2018) solution program using intrinsic phasing and refined with SHELXL 2018/3 (Sheldrick, 2015) by full-matrix least-squares minimization on $\boldsymbol{F^2}$. All non-hydrogen atoms were refined anisotropically. Hydrogen atoms were placed in geometrically calculated positions and refined using a riding model.

Crystal Data. $C_{10}H_5F_3N_2S$, M_r = 242.22, triclinic, P-1 (No. 2), a = 7.1507(7) Å, b = 15.7188(15) Å, c = 18.6996(18) Å, α = 93.583(4)°, β = 90.162(4)°, γ = 96.438(4)°, V = 2084.4(3) Å^3, T = 170.00 K, Z = 8, Z' = 4, μ(Ga K_α) = 1.935 mm^{-1}, 32443 reflections measured, 7875 unique (R_{int} = 0.0715) which were used in all calculations. Multi-scan absorption correction using SADABS-2016/2 was applied (T_{min} = 0.4933, T_{max} = 0.7508). The final wR_2 was 0.3057 (all data) and R_1 was 0.1143 [I ≥ 2σ(I)]. The largest diff. peak and hole were 2.353 and −0.615 e·Å^{-3}.

Identification code	mj25063_0m
Empirical formula	C10 H5 F3 N2 S
Formula weight	242.22
Temperature	170.00 K
Wavelength	1.34139 Å
Crystal system	Triclinic

Space group	P-1	
Unit cell dimensions	a = 7.1507(7) Å	α= 93.583(4)°.
	b = 15.7188(15) Å	β= 90.162(4)°.
	c = 18.6996(18) Å	γ = 96.438(4)°.
Volume	2084.4(3) $Å^3$	
Z	8	
Density (calculated)	1.544 Mg/m^3	
Absorption coefficient	1.935 mm^{-1}	
F(000)	976	
Crystal size	0.17 x 0.17 x 0.05 mm^3	
Theta range for data collection	3.312 to 55.036°.	
Index ranges	-8<=h<=8, -18<=k<=19, -22<=l<=22	
Reflections collected	32443	
Independent reflections	7875 [R(int) = 0.0715]	
Completeness to theta = 53.594°	99.4 %	
Absorption correction	Semi-empirical from equivalents	
Max. and min. transmission	0.7508 and 0.4933	
Refinement method	Full-matrix least-squares on F^2	
Data / restraints / parameters	7875 / 0 / 577	
Goodness-of-fit on F^2	1.075	
Final R indices [I>2sigma(I)]	R1 = 0.1143, wR2 = 0.2875	
R indices (all data)	R1 = 0.1393, wR2 = 0.3057	
Extinction coefficient	n/a	
Largest diff. peak and hole	2.353 and -0.615 e.$Å^{-3}$	

9 Computational Details

Geometries and thermodynamic data of :$C(SCF_3)_2$ and $H_2C(SCF_3)_2$

The structures of bis(trifluoromethylthio)carbene (including S_0 and T_1 states) and bis(trifluoromethylthio)methane were optimized with (U)M06-2X functional[39] and def2-QZVP basis sets[40] for all atoms with an ultrafine integration grid at gas phase. Frequency calculations of each conformer were performed at the same level of theory. The thermal Gibbs free energy corrections at 298.15 K and 1.0 atm were obtained by treating frequencies below 100 cm^{-1} with Minenkov's quasi-rigid-rotor-harmonic-oscillator (quasi-RRHO) approximation[41] and a zero-point energy (ZPE) scale factor of 0.970[42] using Shermo 2.6 code[43]. Single point energy was further calculated at a

more accurate DLPNO-(U)CCSD(T)[44]/def2-QZVPP[40] level with tightPNO and RIJCOSX options using ORCA 6.1.0 package[45].

A typical keyword line of ORCA input files is presented below:

```
! DLPNO-CCSD(T) tightPNO RIJCOSX def2QZVPP def2/J def2-QZVPP/C tightSCF noautostart miniprint
```

Mayer bond orders were provided by Multiwfn 3.8 (dev) (update date: 2025-Aug-2)[46] based on ultrafine-grid (U)M06-2X/def2-SVP[47] wavefunctions using (U)M06-2X/def2-QZVP geometries.

The relative Gibbs free energy (G_{rel}), central S–C–S angles ($\theta_{S–C–S}$), average central C–S distances ($d_{C–S}$) and Mayer bond orders (MBO) are summarized as follows:

Supplementary Table 3. Theoretical data for bis(trifluoromethylthio)carbene and bis(trifluoromethylthio)methane

species[a]	conformer[b]	G_{rel} (kcal·mol^{-1})	$\theta_{S–C–S}$ (°)	$d_{C–S}$ (Å)	MBO[c]
$^1C(SCF_3)_2$	1 (*trans*-*cis*)	**0.0**	126.8	1.63	1.46
	2 (*trans*-*trans*)	0.6	118.0	1.65	1.43
	3 (*cis*-*cis*)	5.7	154.6	1.59	1.55
$^3C(SCF_3)_2$	1	28.8	133.5	1.68	1.11
	2	29.0	134.8	1.68	1.11
	3	30.1	139.1	1.67	1.12
$H_2C(SCF_3)_2$	1	**0.0**	111.5	1.81	0.98
	2	0.7	117.1	1.81	0.98
	3	1.4	114.7	1.81	0.98

[a]The superscripts 1 and 3 indicate the singlet and triplet spin states, respectively; [b]Conformers are numbered according to their relative Gibbs free energies; [c]MBO, Mayer bond order.

Computed reactivity indices of bis(trifluoromethylthio)carbene

The reactivity indices of bis(trifluoromethylthio)carbene (based on the *trans*-*cis* conformer), including stabilization energy (ΔE_{stab}), global electrophilicity (ω), and absolute hardness (η), were obtained according to the reported methods[48]. The ΔE_{stab} of singlet carbene ^1CXY was defined as the $-\Delta E$ of the reaction $^1CH_2 + CH_3X + CH_3Y \rightarrow {}^1CXY + 2\ CH_4$ at B3LYP[49]/6-311++G(2d,p)[50] level of theory with the fine

integration grid, while ω and η values were computed at HF/6-31G(d,p)[50c, 51] //MP2/6-31G(d,p) level of theory with the following equations:

$$\omega = (E_{LU} + E_{HO})^2/[8(E_{LU} - E_{HO})] \quad \text{(Eq. 1)}$$

$$\eta = E_{LU} - E_{HO} \quad \text{(Eq. 2)}$$

where E_{LU} and E_{HO} are the theoretically obtained lowest unoccupied (LU) and highest occupied (HO) molecular orbital energy of carbenes, respectively.

The results are shown below:

Supplementary Table 4. Computed properties of bis(trifluoromethylthio)carbene compared with dihalocarbenes.

carbene[a]	ΔE_{stab} (kcal·mol^{-1})	E_{LU} (eV)	E_{HO} (eV)	ω (eV)	η (eV)
$^1CF_2$[48]	70.9	2.74	−12.85	0.82	15.60
$^1C(SCF_3)_2$	65.3	1.37	−10.66	0.90	12.03
^1CFCl[48]	56.1	1.74	−11.71	0.92	13.45
$^1CCl_2$[48]	45.5	1.00	−10.91	1.03	11.91

[a]The superscript 1 indicates the singlet spin state.

The Cartesian coordinates and raw computational data for the optimized structures are available in Source Data.

10 Copies of ^{1}H NMR, ^{19}F NMR and ^{13}C NMR Spectra

^{1}H NMR Spectrum of **$HC(SCF_3)_2(SCN)$** (400 MHz, $CDCl_3$)

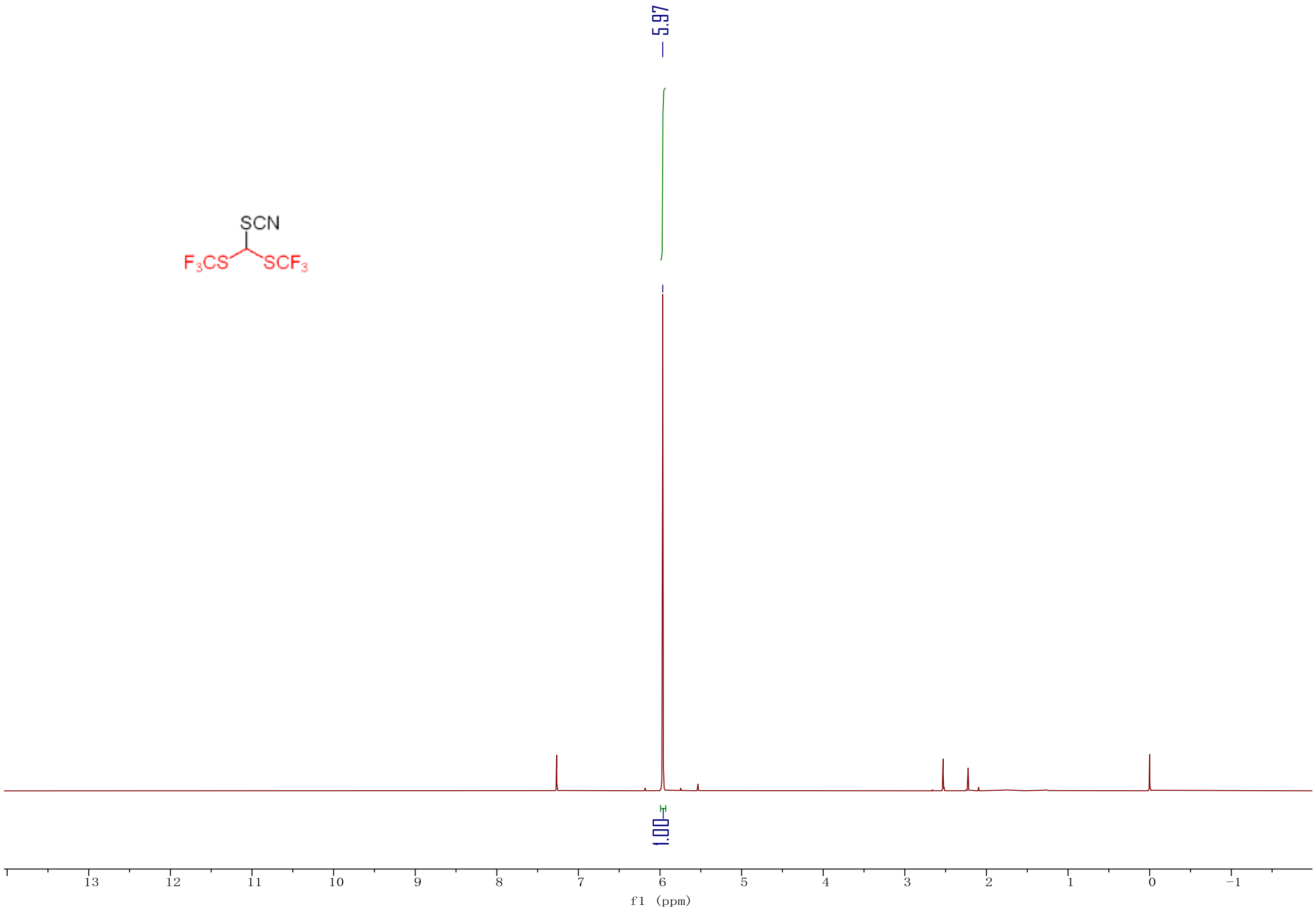

^{19}F NMR Spectrum of **$HC(SCF_3)_2(SCN)$** (376 MHz, $CDCl_3$)

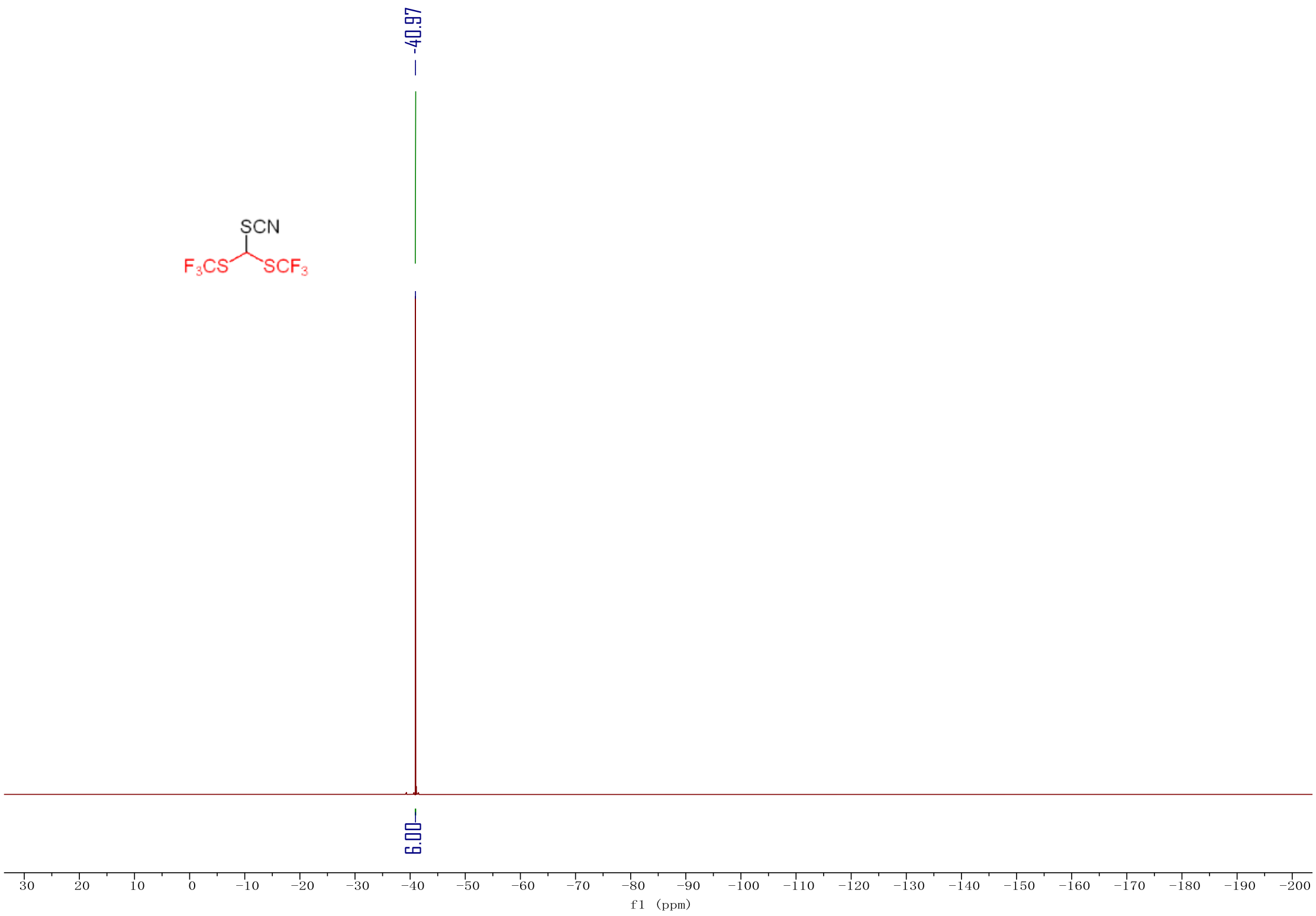

^{13}C **NMR** Spectrum of **HC(SCF$_3$)$_2$(SCN)** (101 MHz, CDCl$_3$)

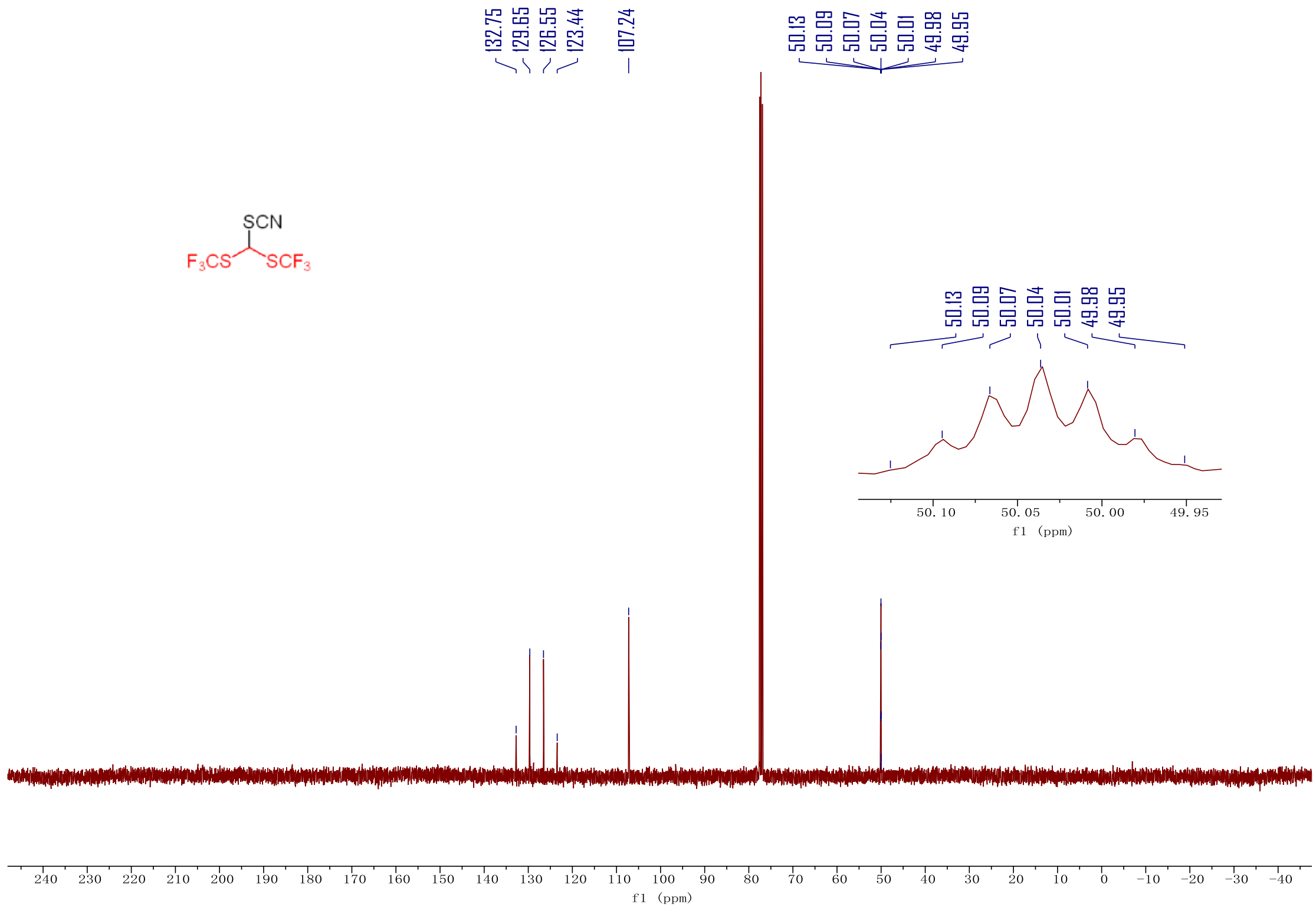

^{19}F **NMR** Spectrum of **DC(SCF$_3$)$_2$(SCN) (61% D)** (376 MHz, CDCl$_3$)

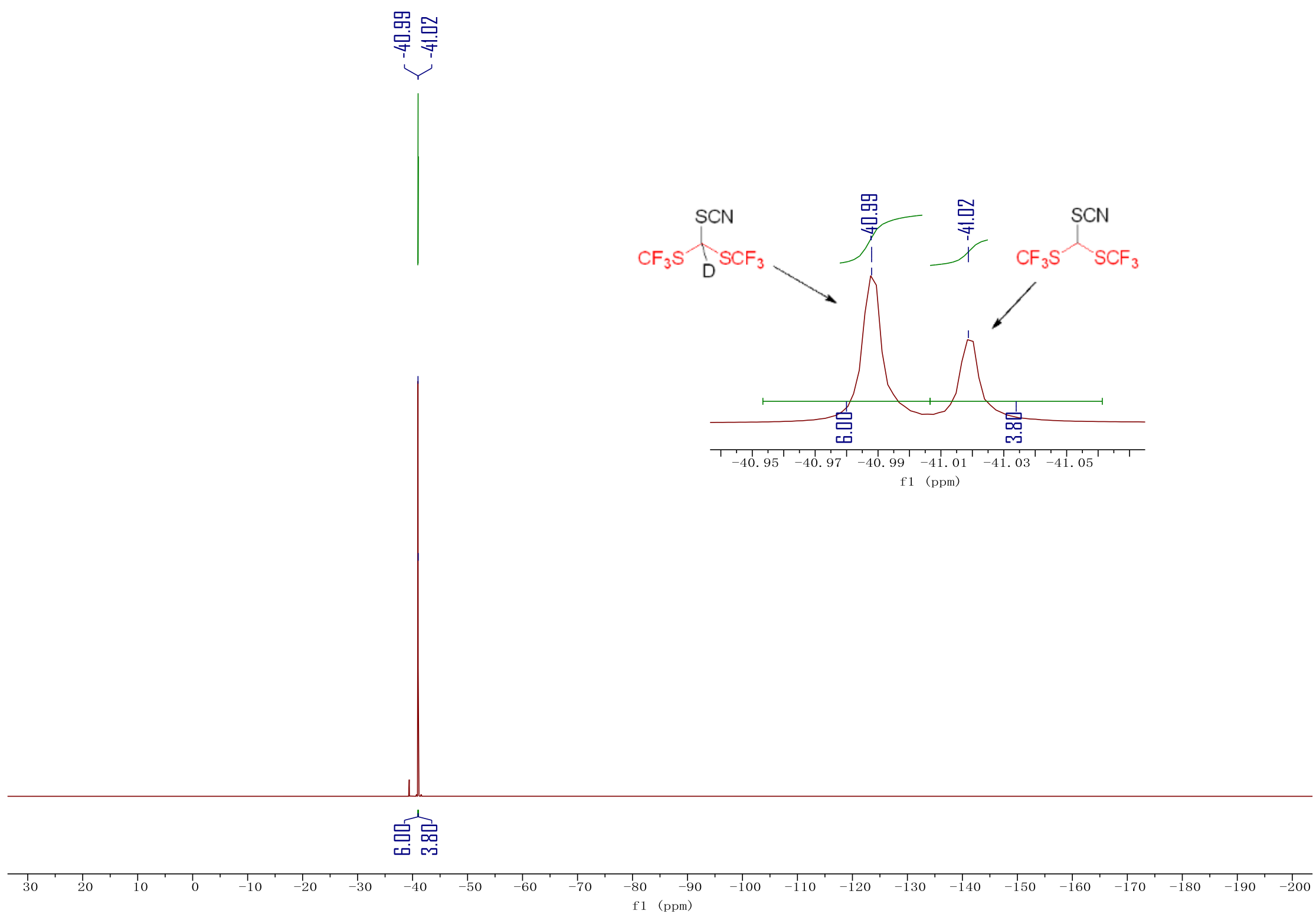

^{13}C NMR Spectrum of **$DC(SCF_3)_2(SCN)$ (61% D)**
(101 MHz, $CDCl_3$)

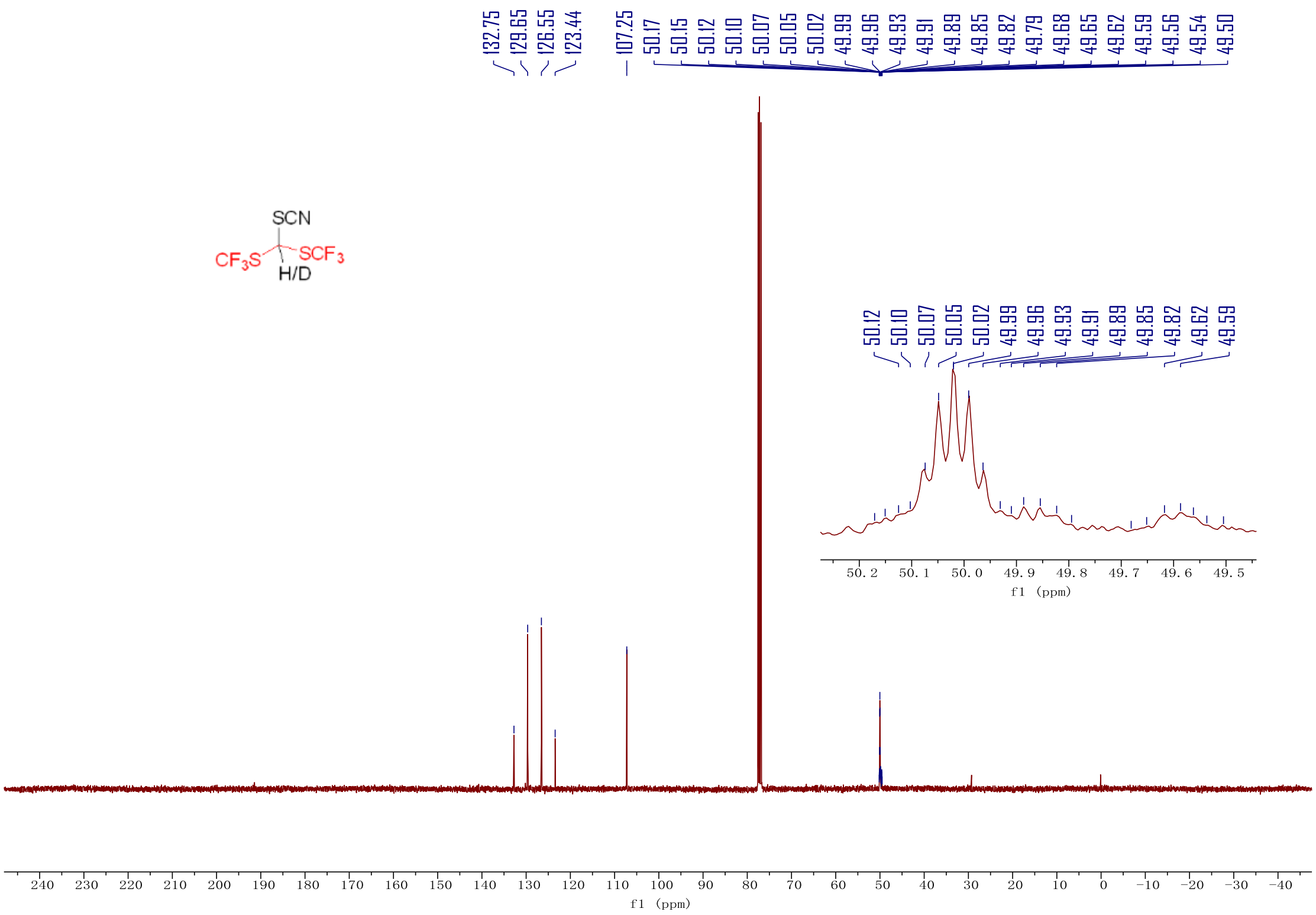

^{1}H NMR Spectrum of **$BrCH(SCF_3)_2$** (400 MHz, $CDCl_3$)

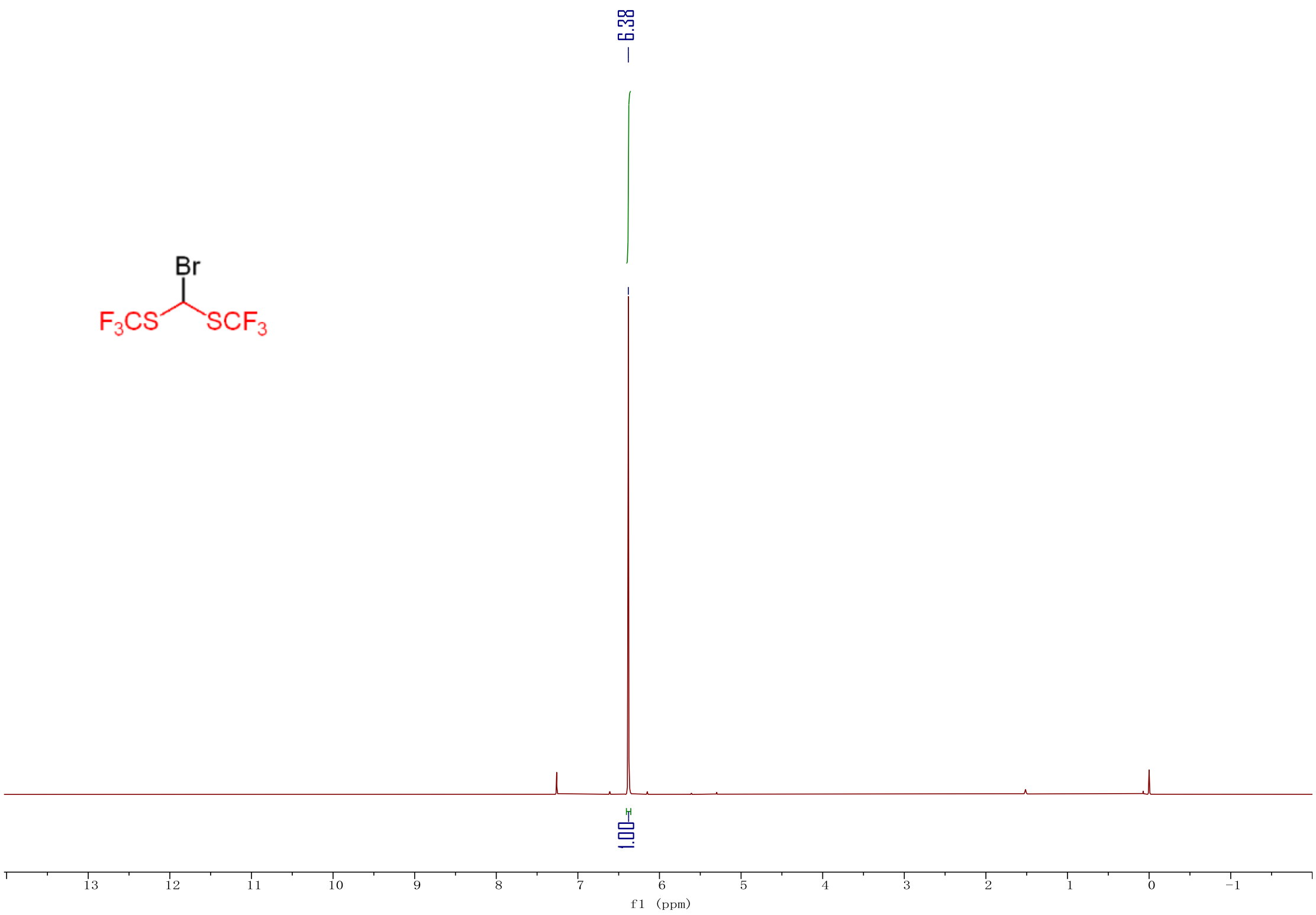

^{19}F NMR Spectrum of **$BrCH(SCF_3)_2$** (376 MHz, $CDCl_3$)

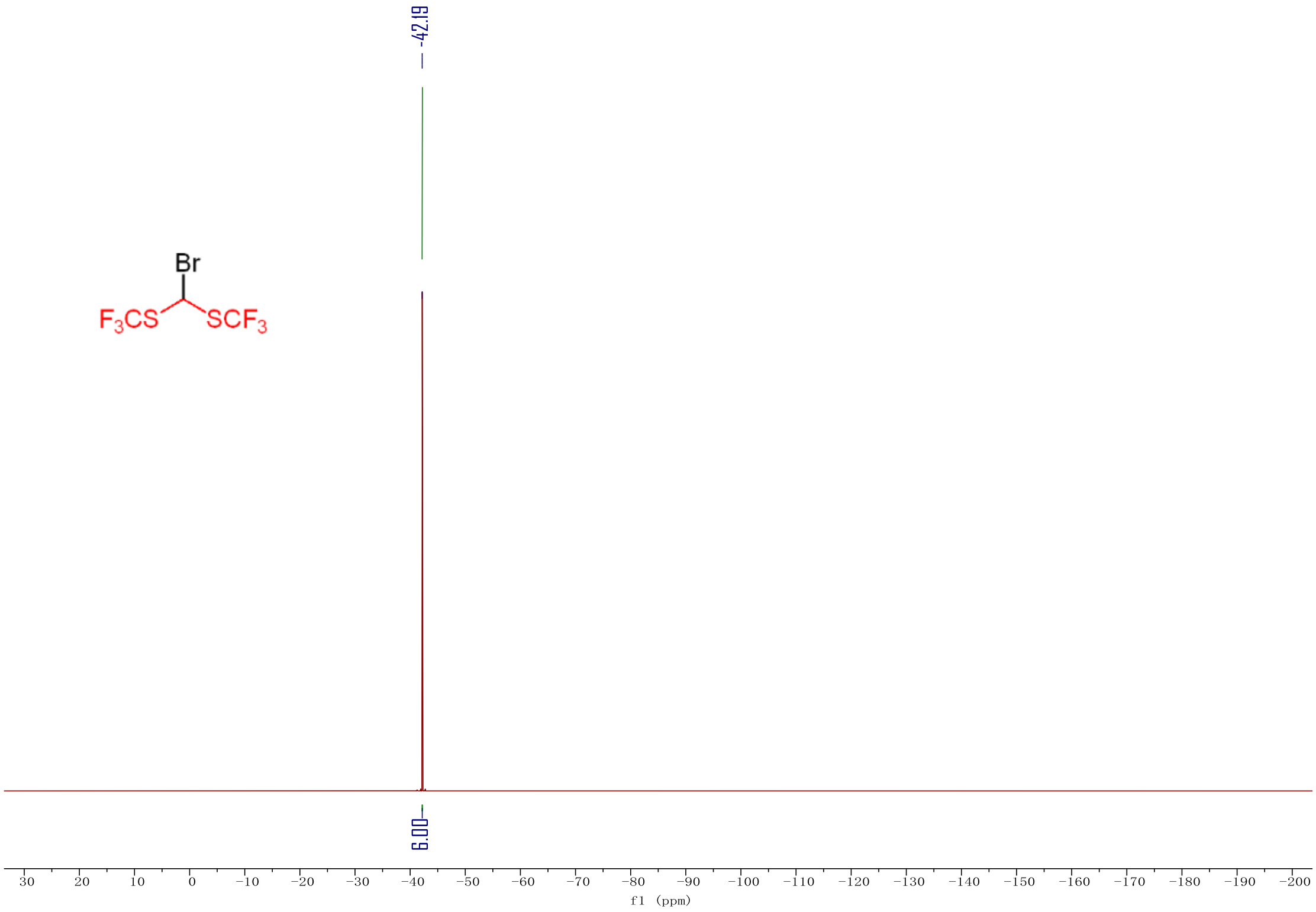

^{13}C NMR Spectrum of **$BrCH(SCF_3)_2$** (101 MHz, $CDCl_3$)

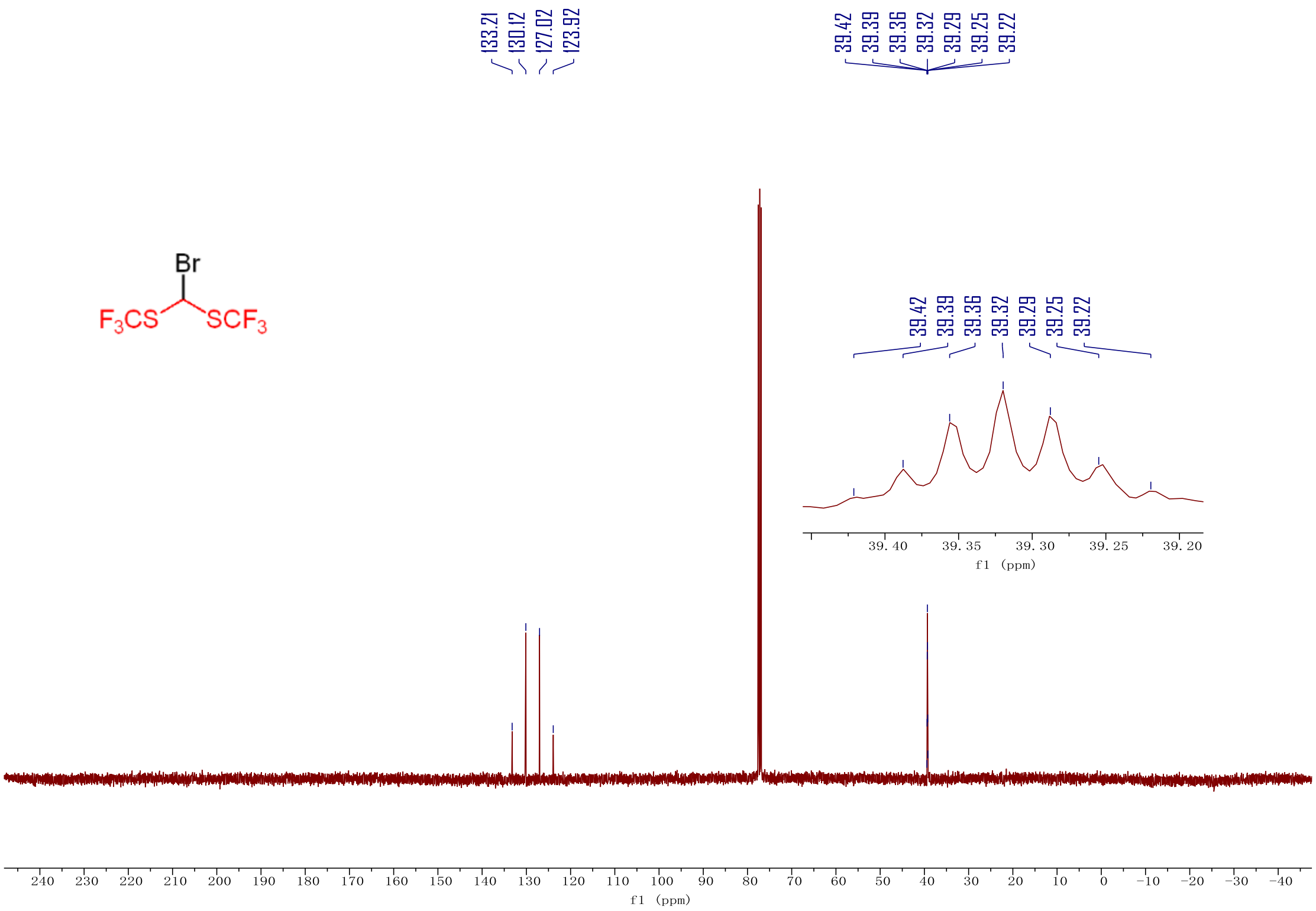

^{19}F NMR Spectrum of **$BrCH(SCF_3)_2$ (61% D)** (376 MHz, $CDCl_3$)

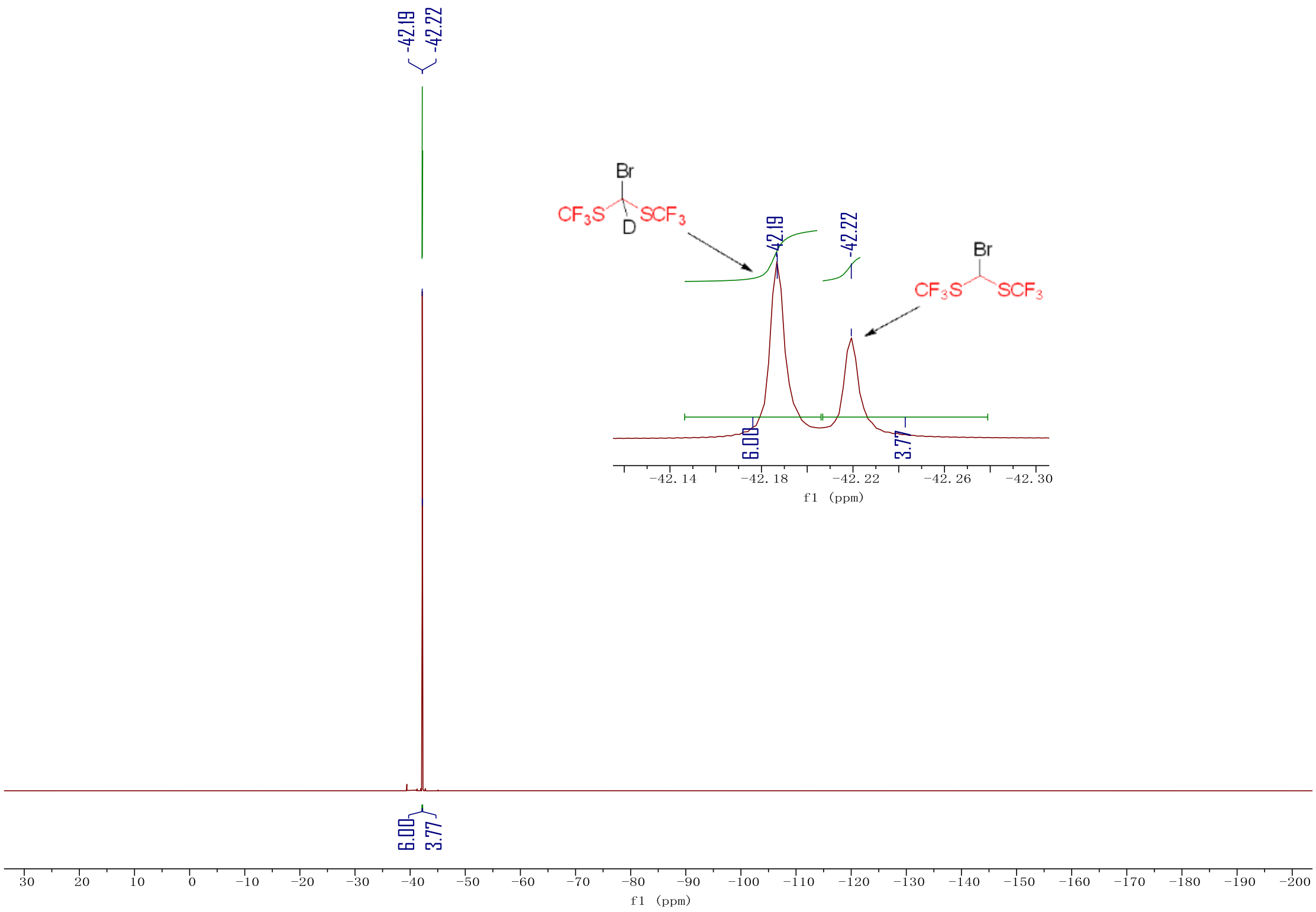

^{13}C NMR Spectrum of **$BrCH(SCF_3)_2$ (61% D)** (101 MHz, $CDCl_3$)

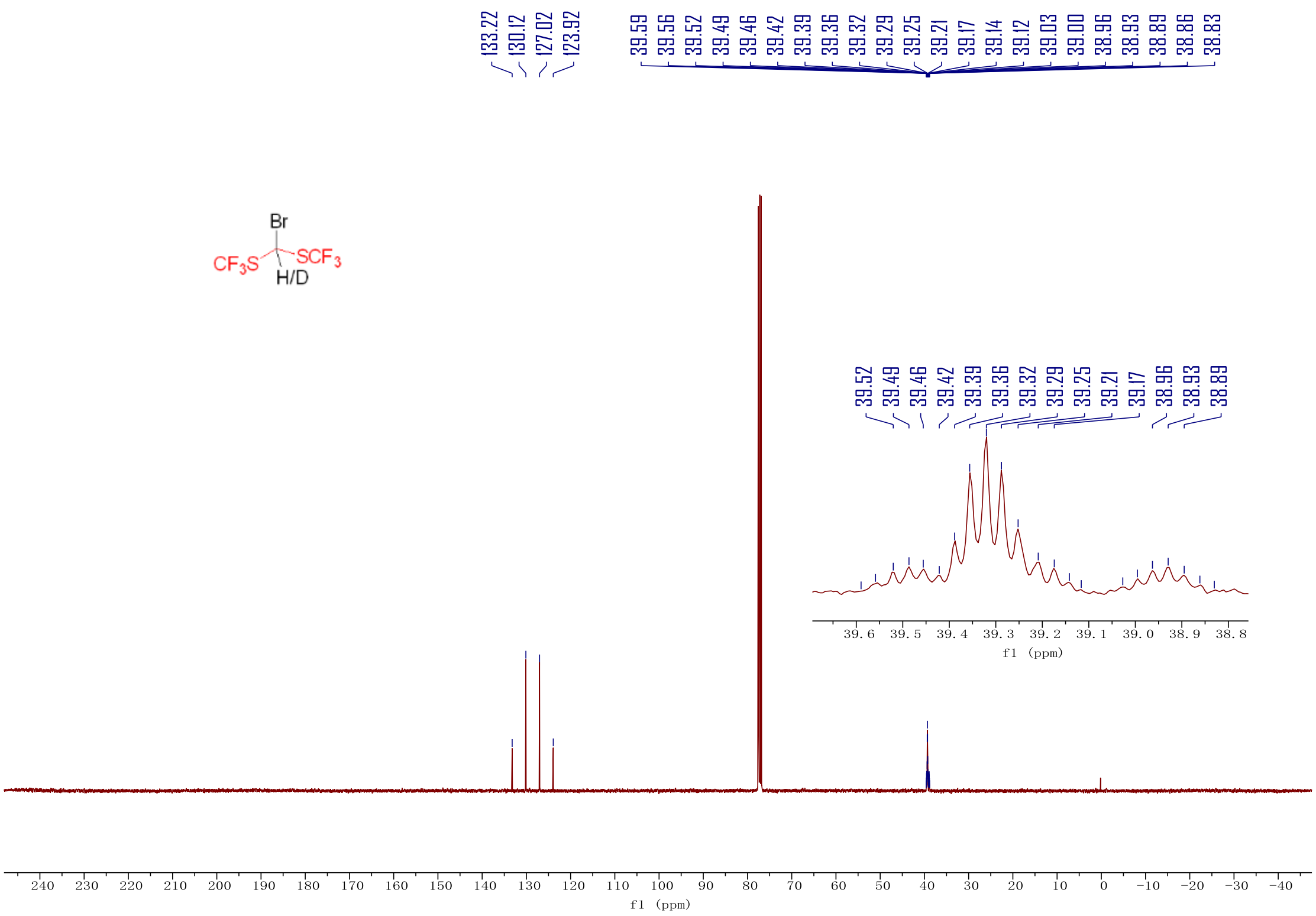

^{19}F NMR Spectrum of **$(F_3CS)_2C{=}C(SCF_3)_2$** (376 MHz, $CDCl_3$)

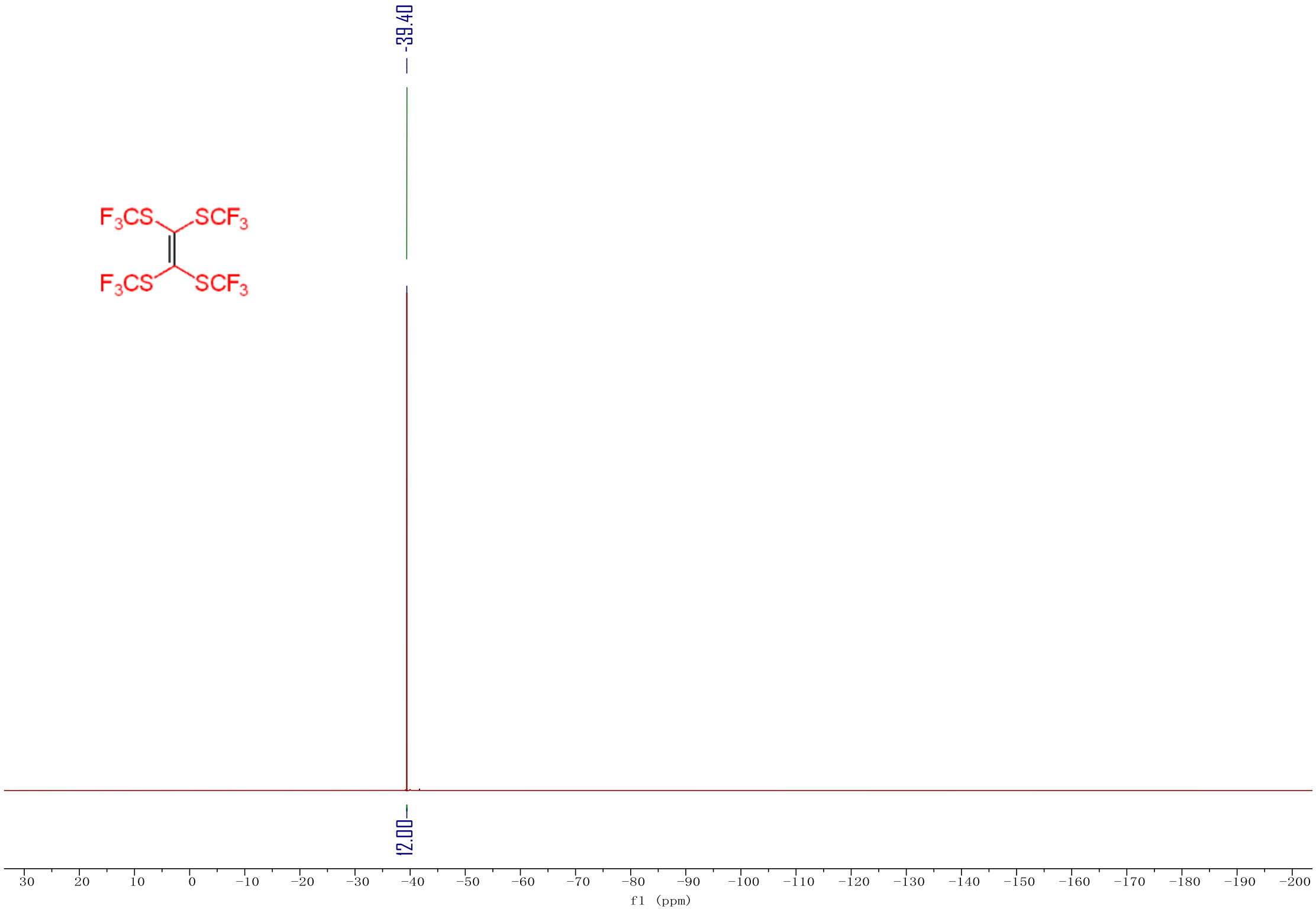

^{13}C NMR Spectrum of **$(F_3CS)_2C{=}C(SCF_3)_2$** (101 MHz, $CDCl_3$)

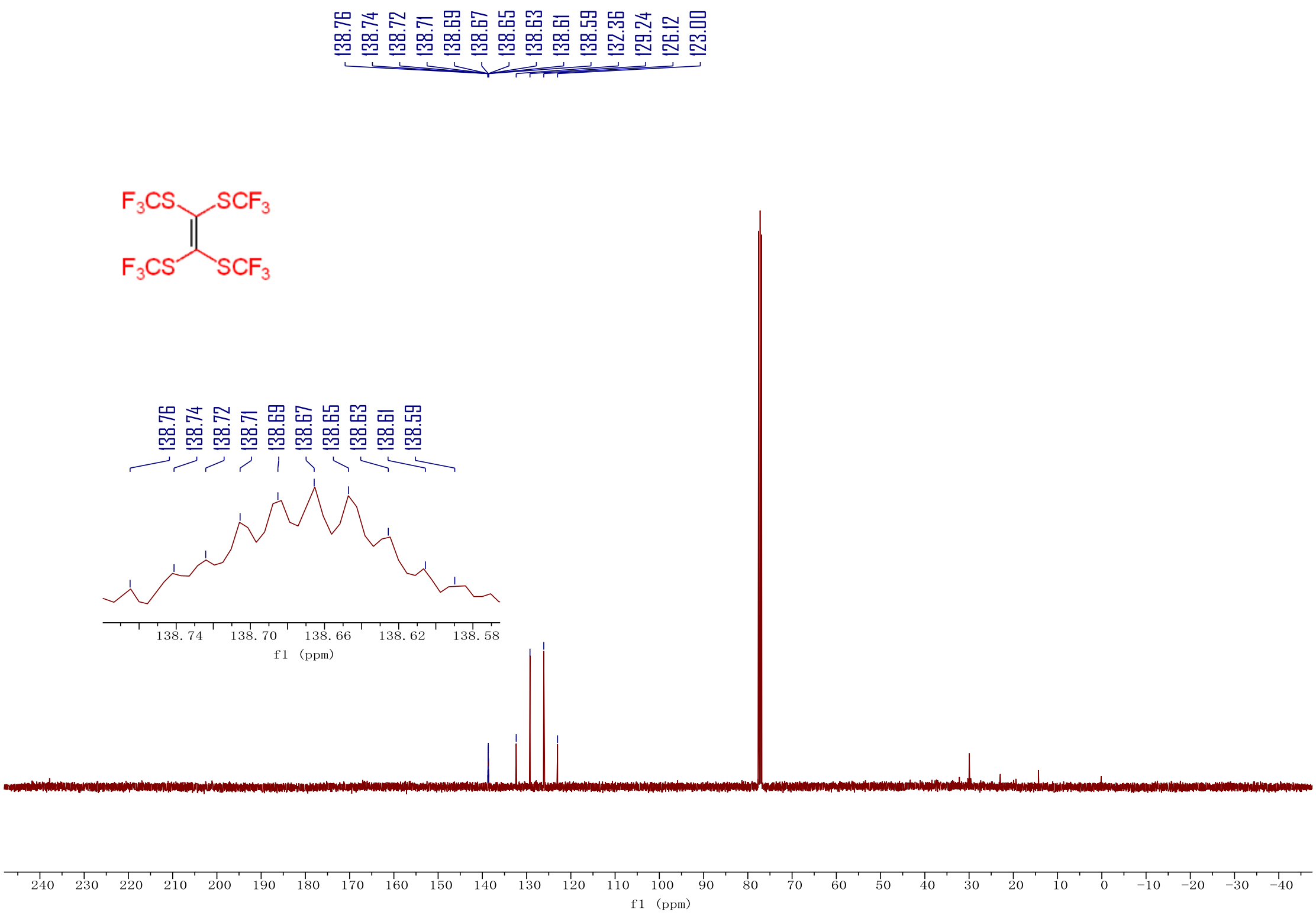

^{1}H NMR Spectrum of **1n** (400 MHz, $CDCl_3$)

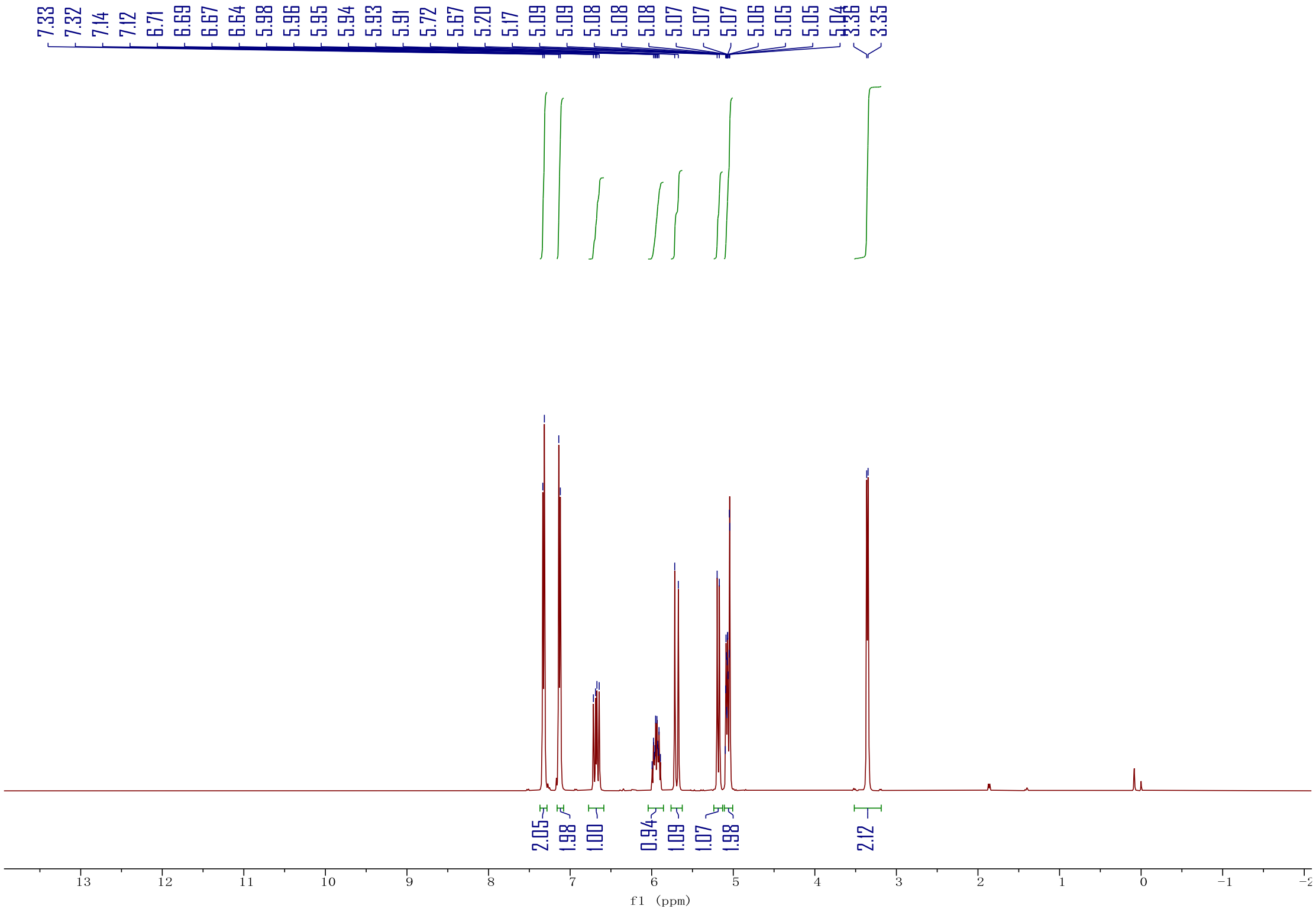

^{13}C NMR Spectrum of **1n** (101 MHz, $CDCl_3$)

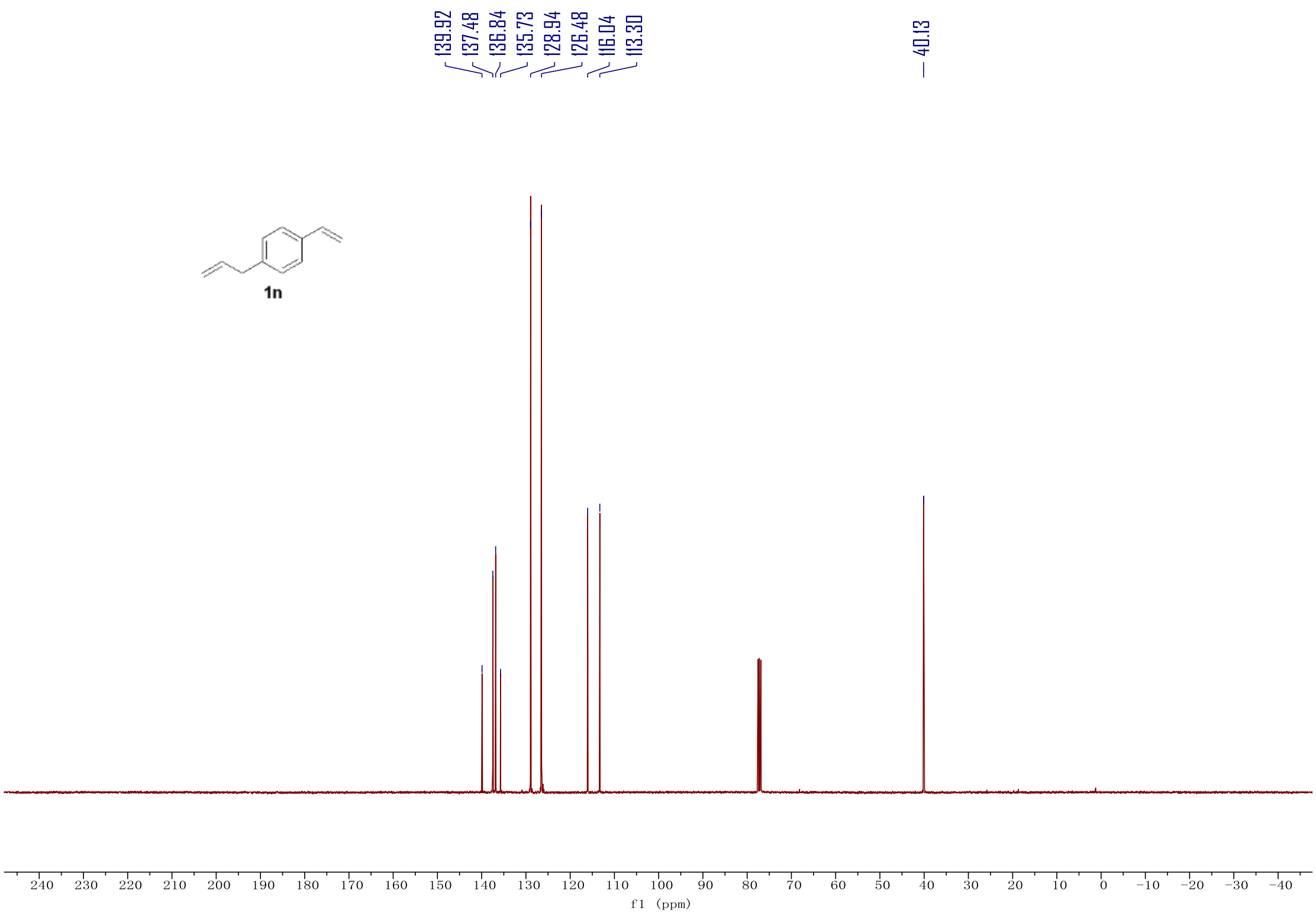

^{1}H NMR Spectrum of **1s** (400 MHz, $CDCl_3$)

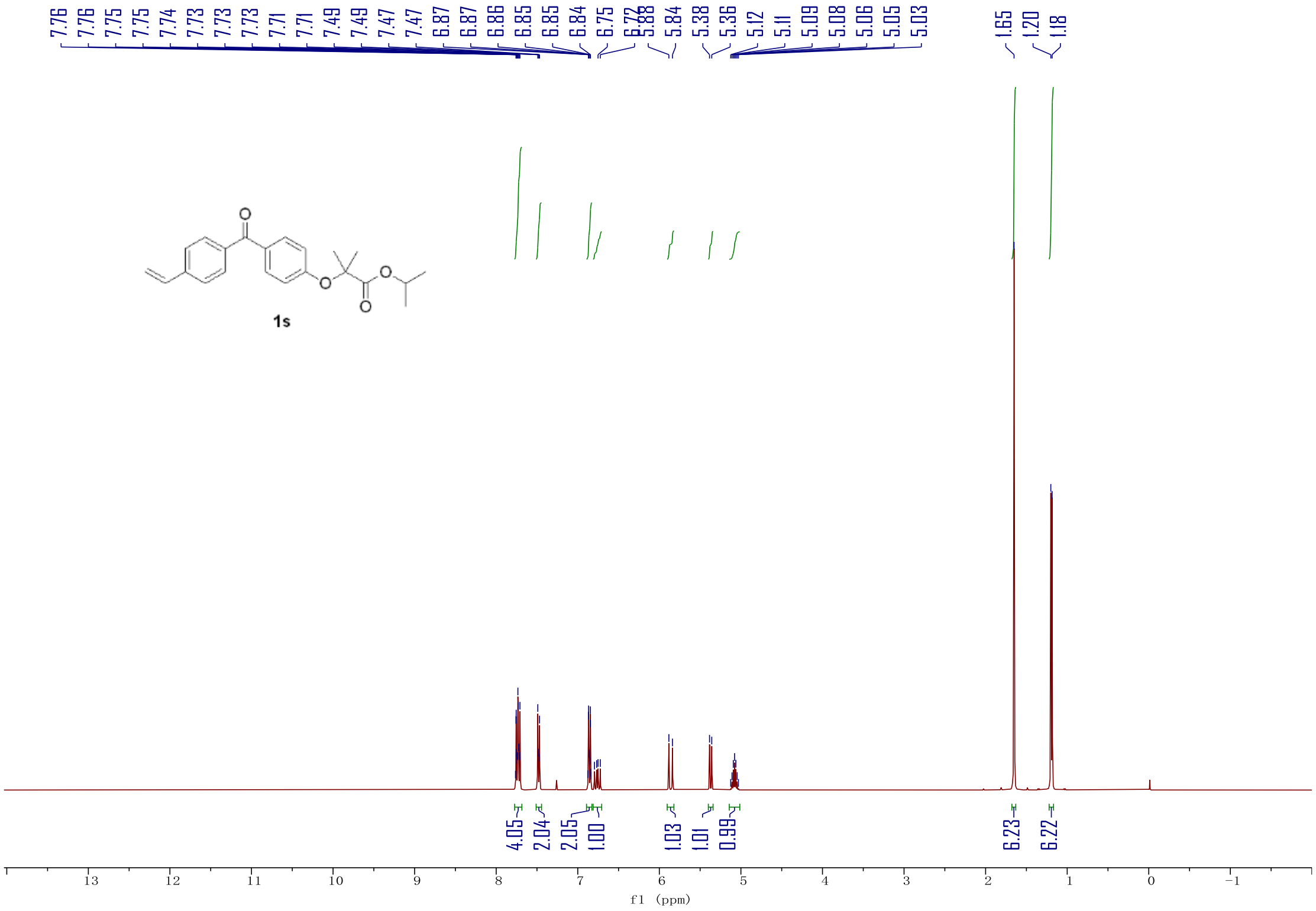

^{13}C NMR Spectrum of **1s** (101 MHz, $CDCl_3$)

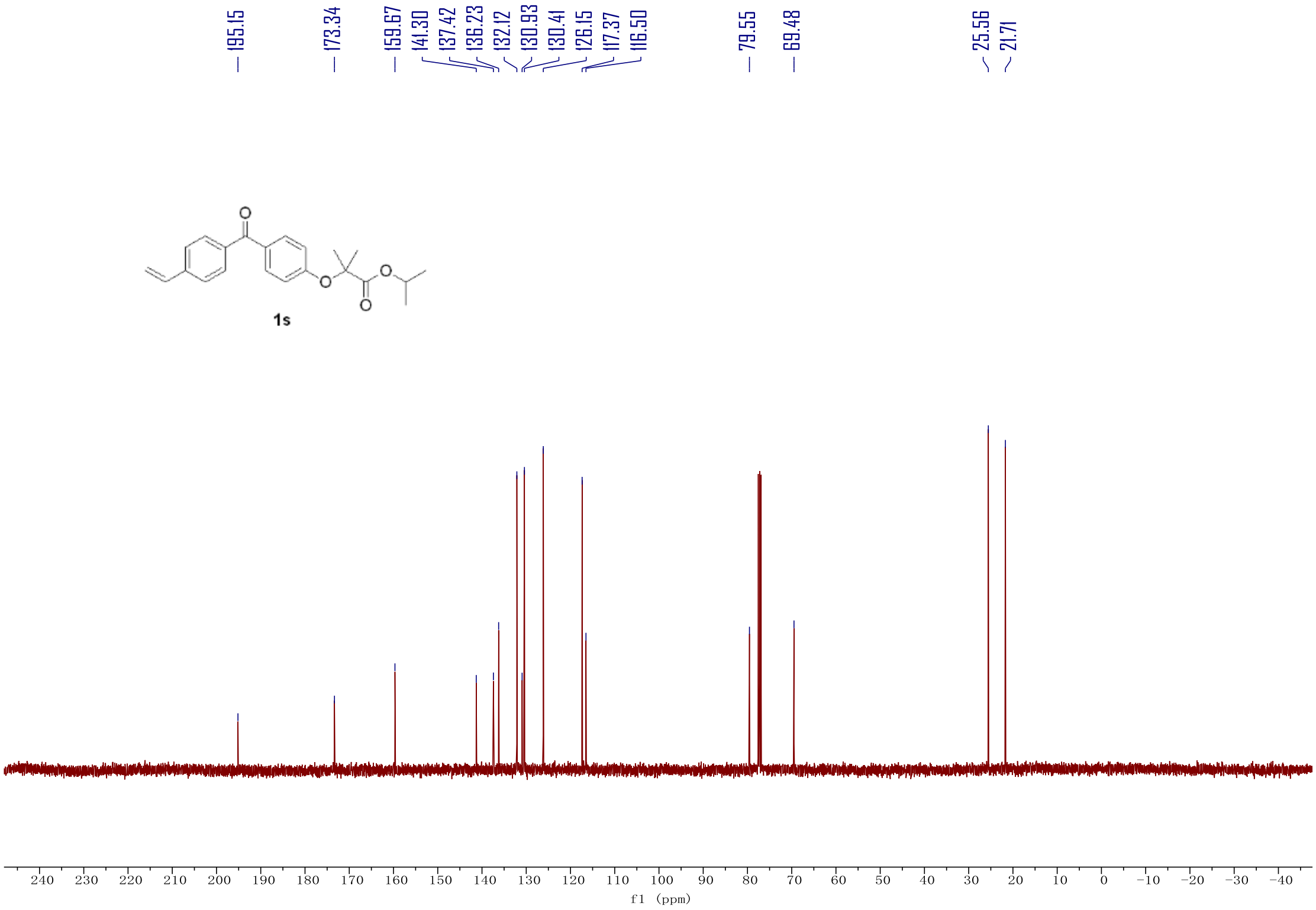

^{1}H NMR Spectrum of **1u** (400 MHz, $CDCl_3$)

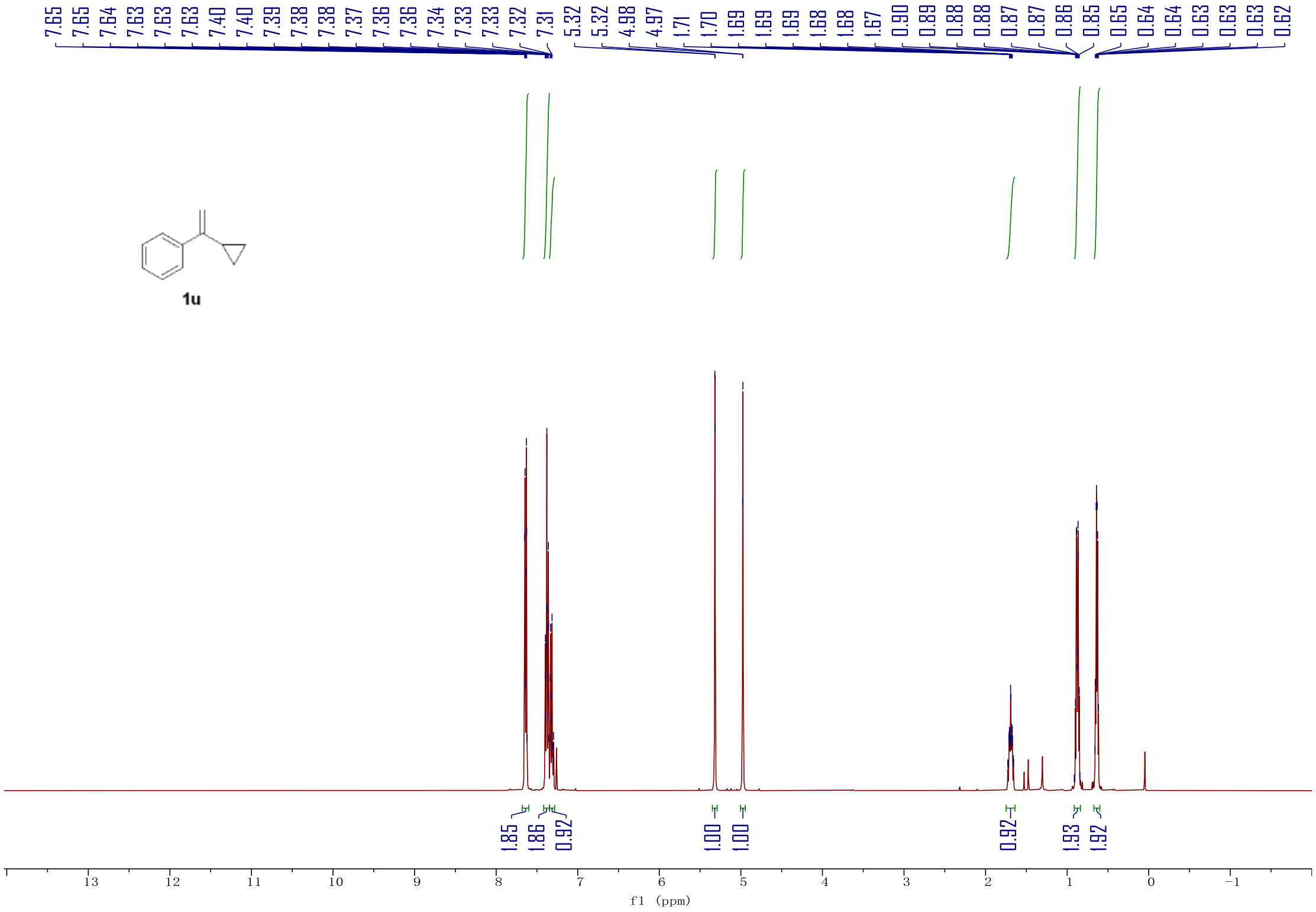

^{13}C NMR Spectrum of **1u** (101 MHz, $CDCl_3$)

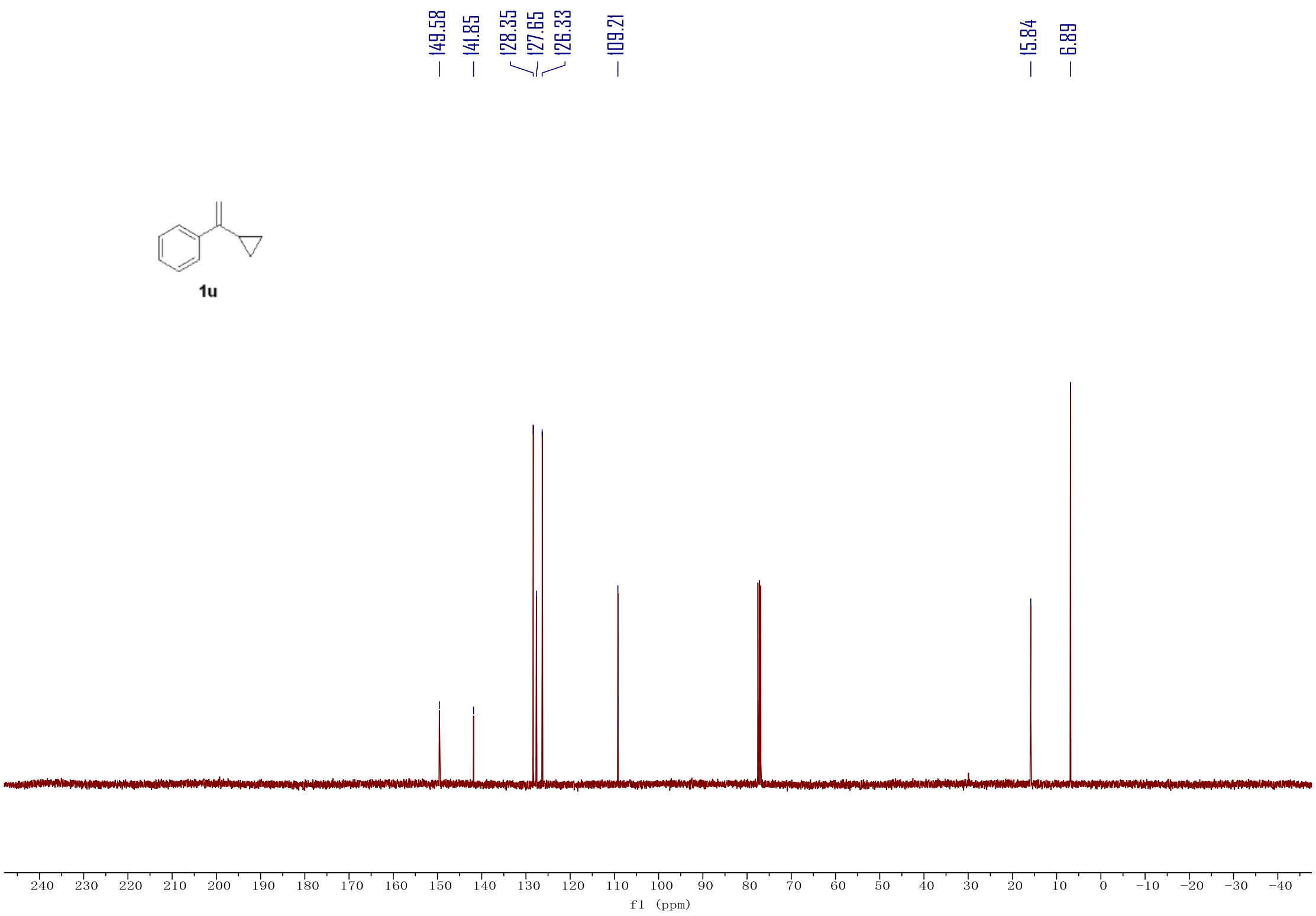

^{1}H NMR Spectrum of 3g (400 MHz, $CDCl_3$)

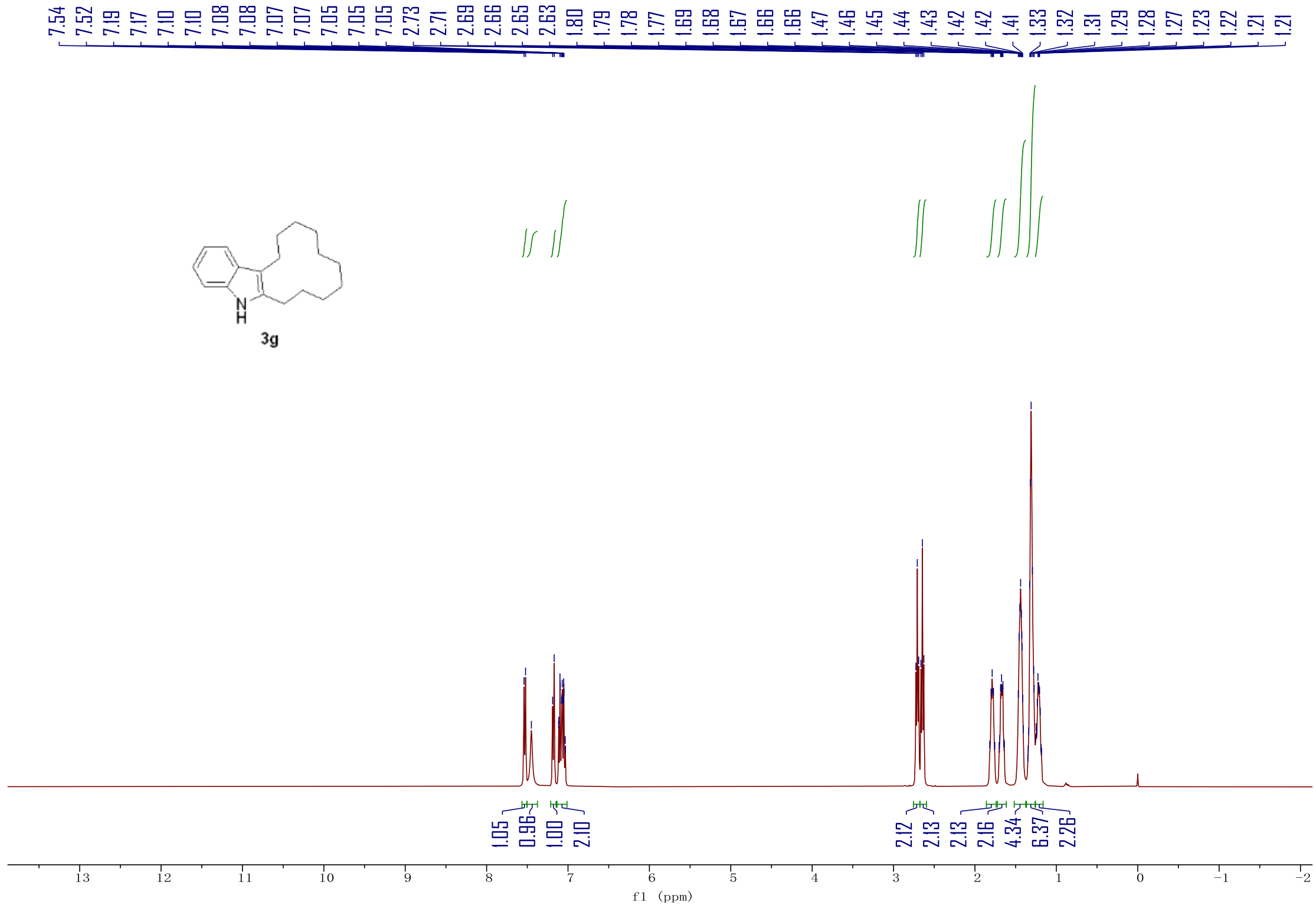

^{13}C NMR Spectrum of 3g (101 MHz, $CDCl_3$)

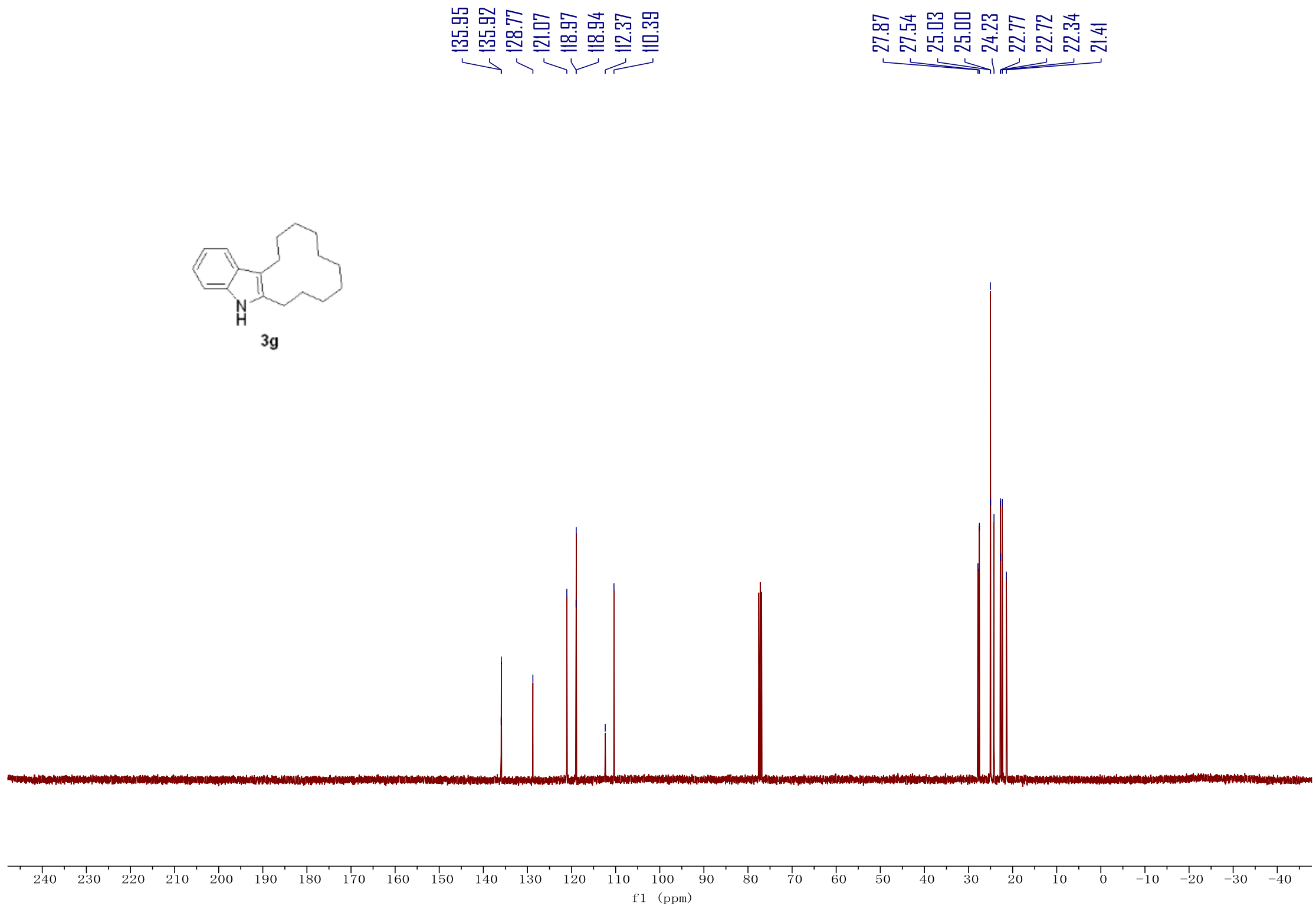

^{1}H NMR Spectrum of **3o** (400 MHz, $CDCl_3$)

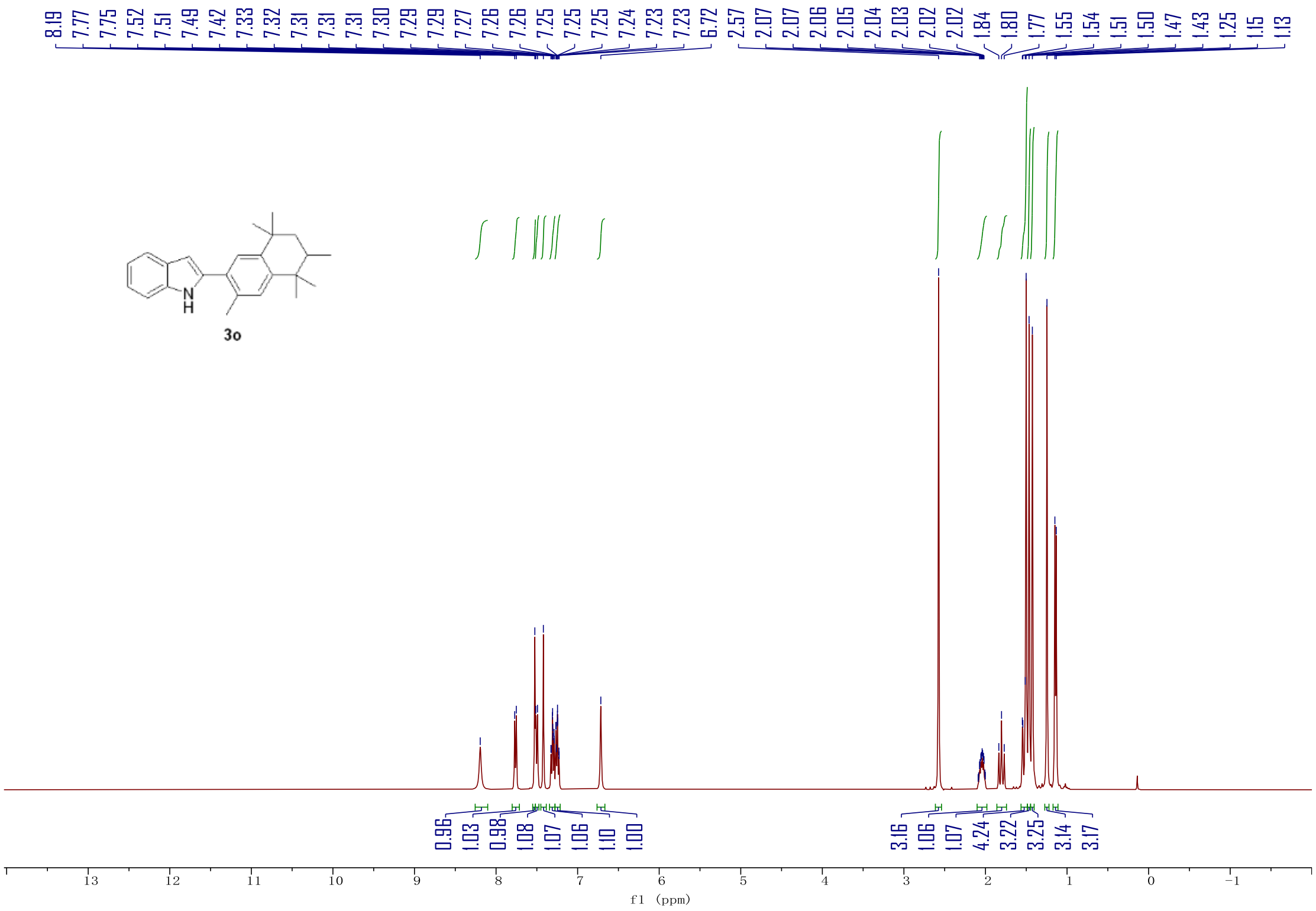

^{13}C NMR Spectrum of **3o** (101 MHz, $CDCl_3$)

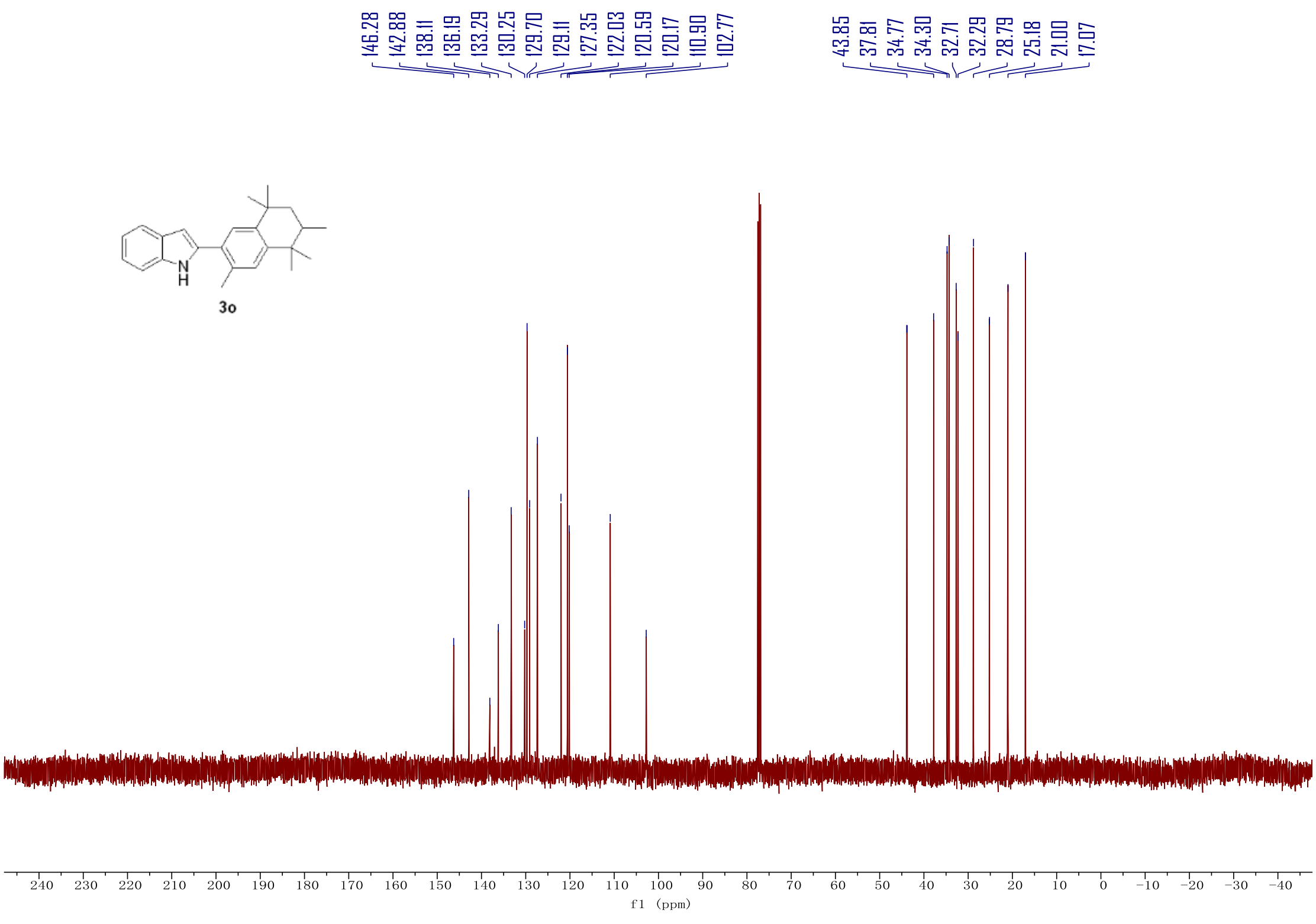

^{1}H NMR Spectrum of **3q** (400 MHz, $CDCl_3$)

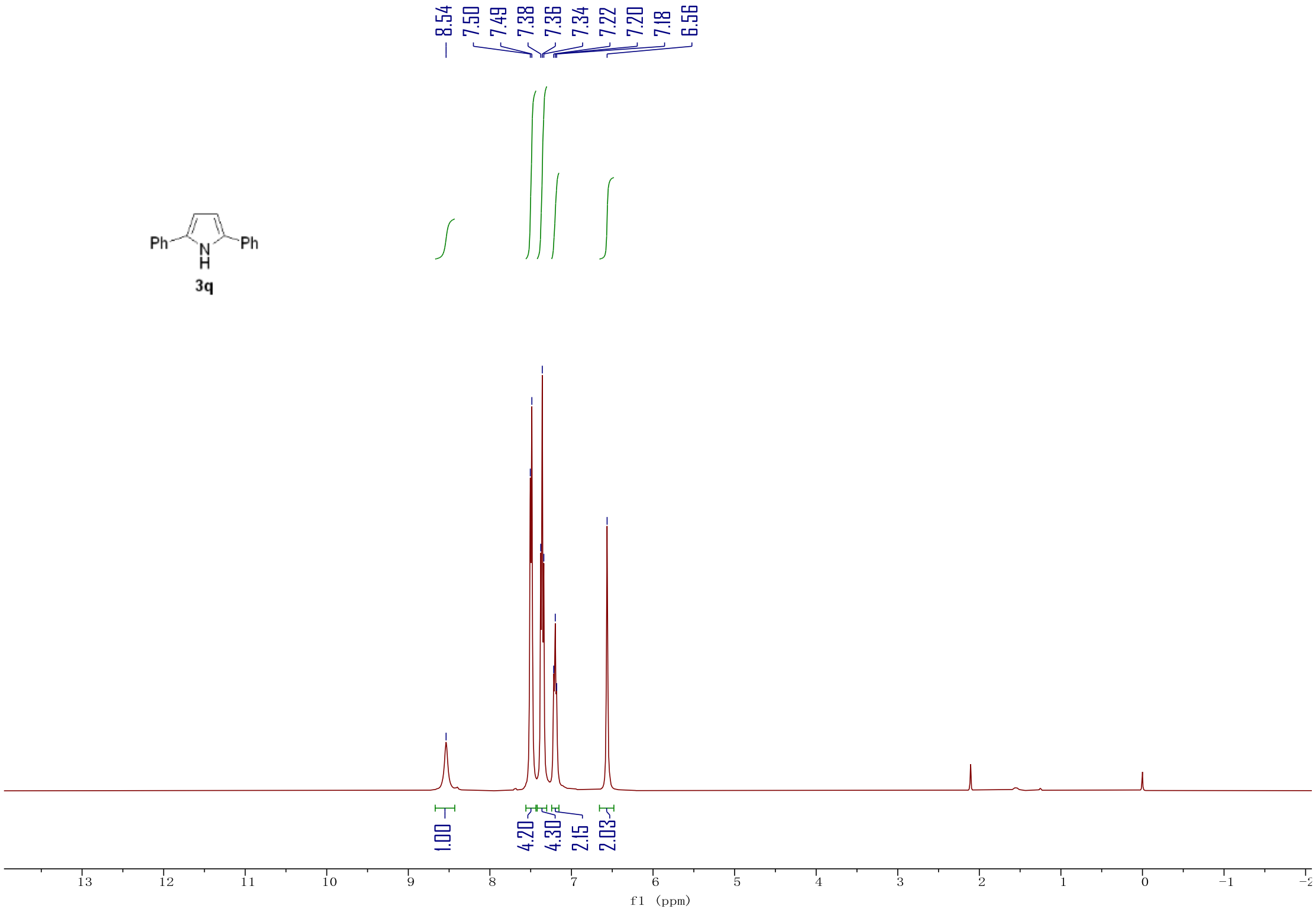

^{13}C NMR Spectrum of **3q** (101 MHz, $CDCl_3$)

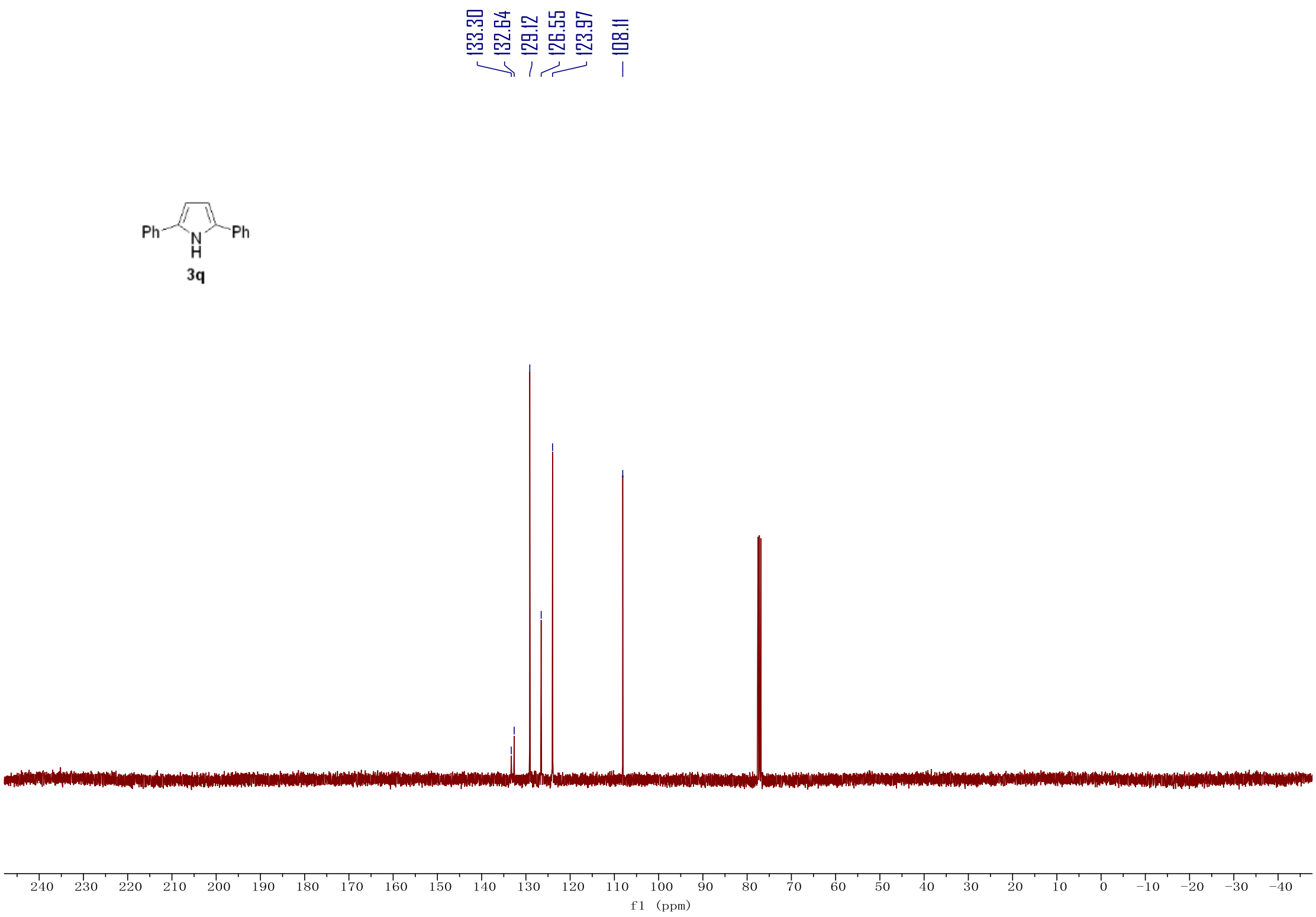

^{1}H NMR Spectrum of 3t (400 MHz, $CDCl_3$)

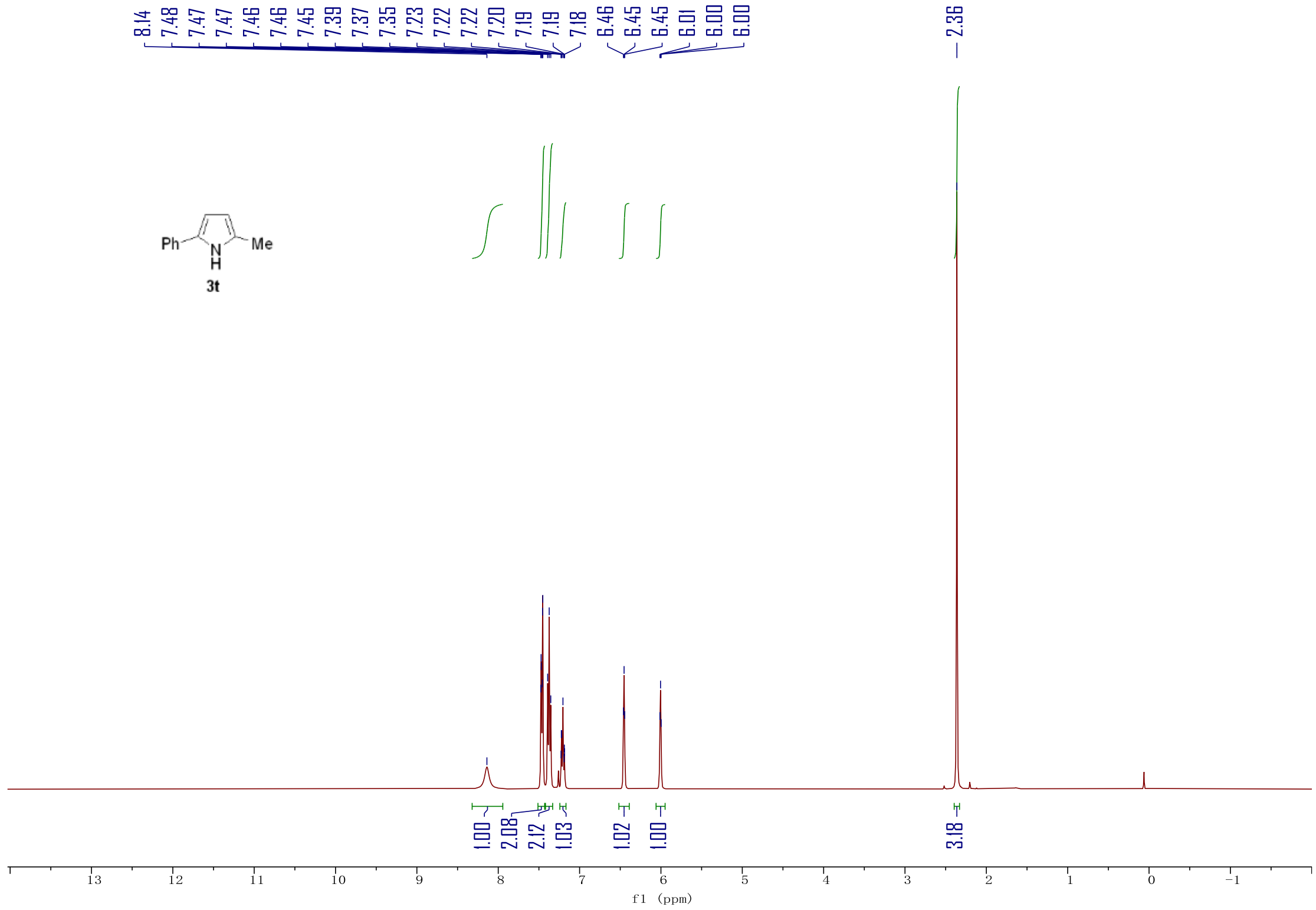

^{13}C NMR Spectrum of 3t (101 MHz, $CDCl_3$)

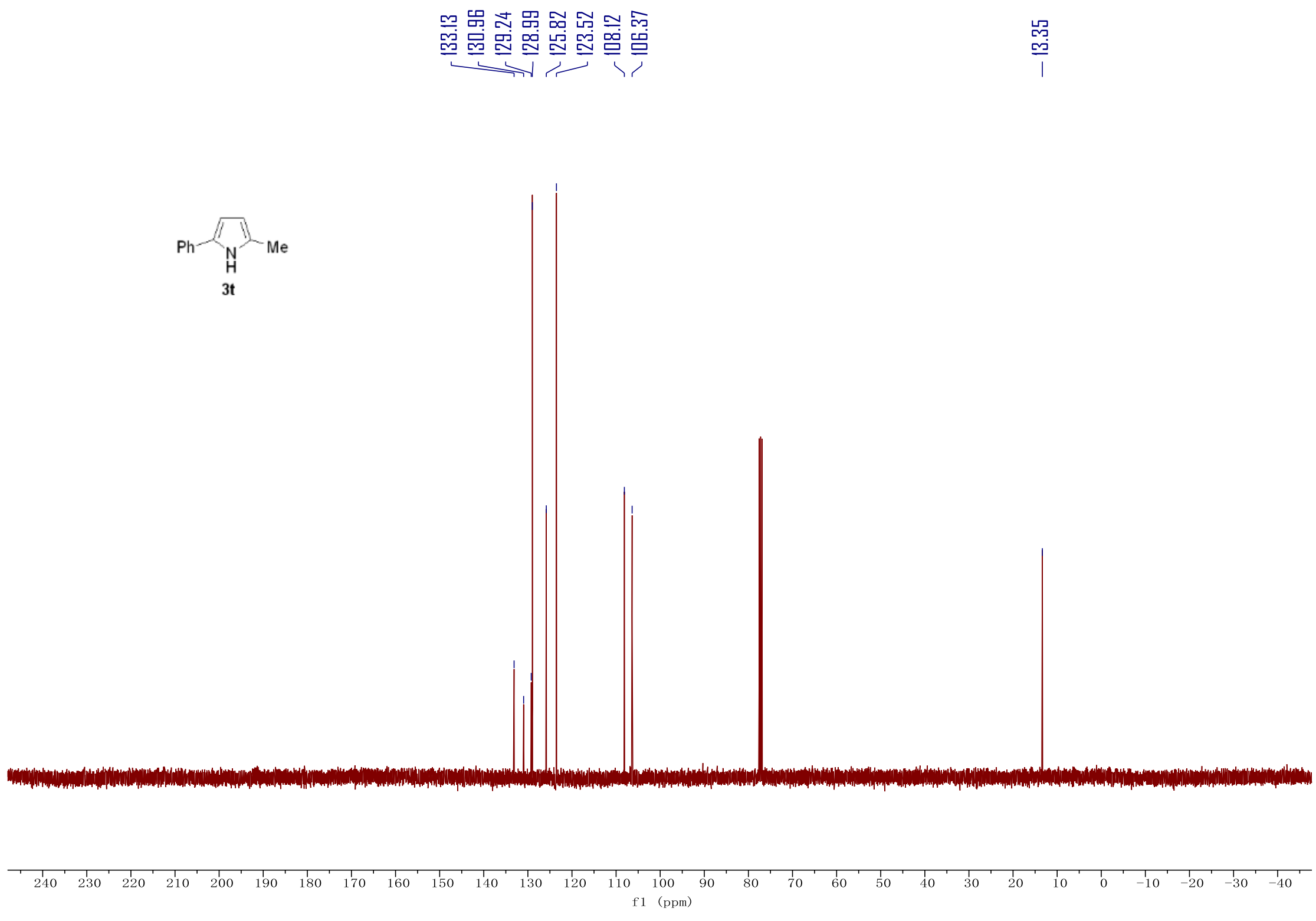

^{1}H NMR Spectrum of **3u** (400 MHz, $CDCl_3$)

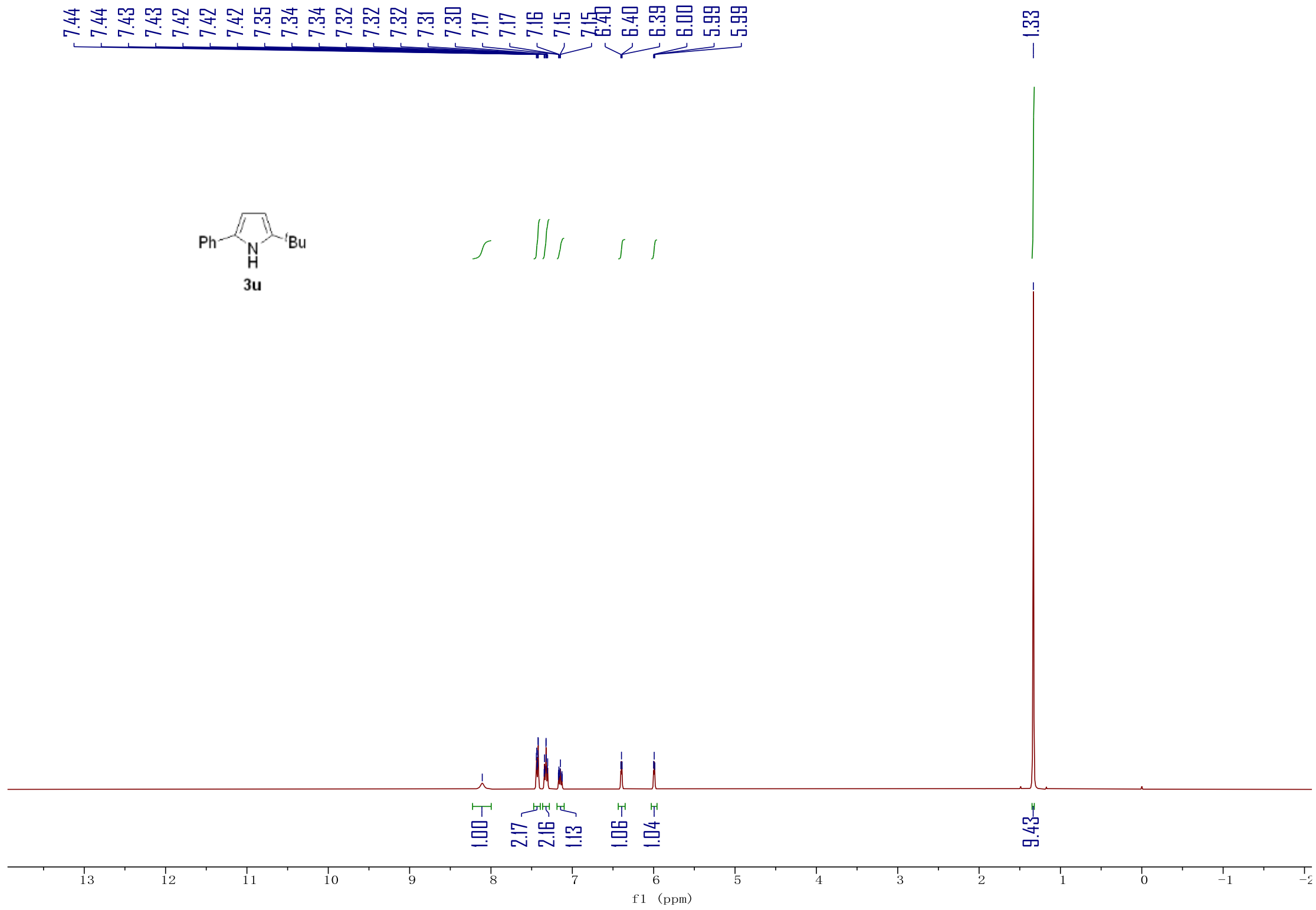

^{13}C NMR Spectrum of **3u** (101 MHz, $CDCl_3$)

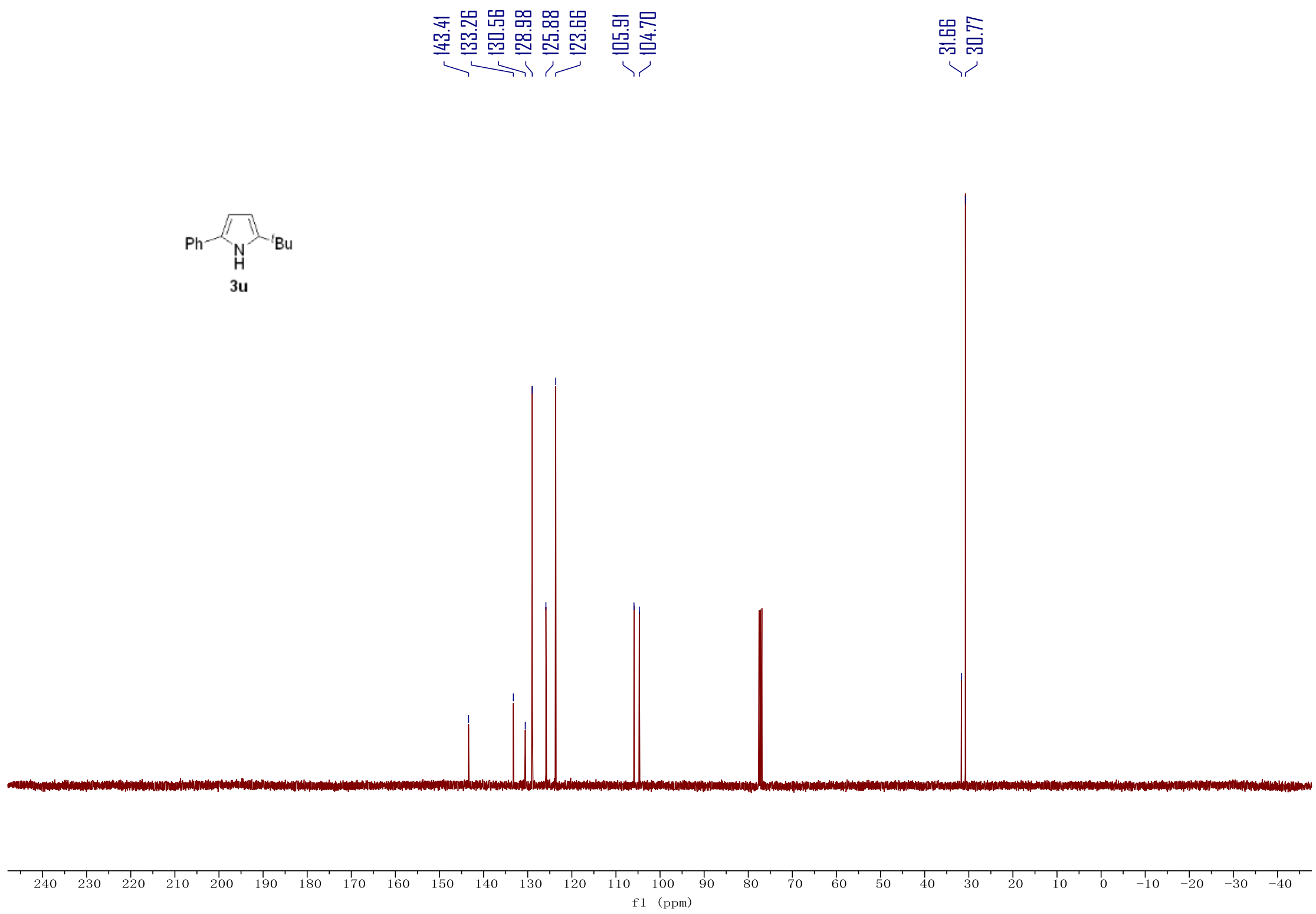

^{1}H NMR Spectrum of **7b** (400 MHz, $CDCl_3$)

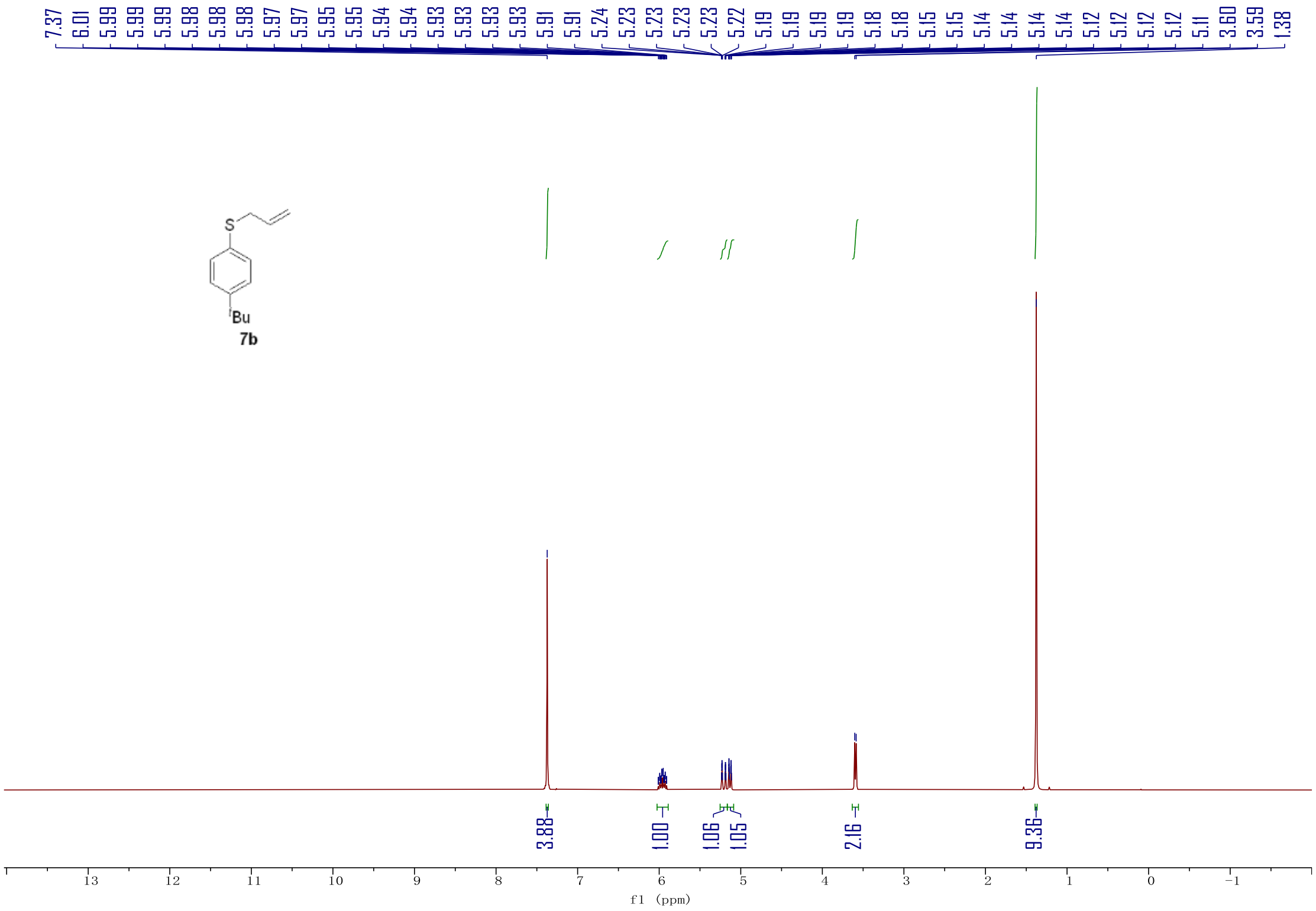

^{13}C NMR Spectrum of **1s** (101 MHz, $CDCl_3$)

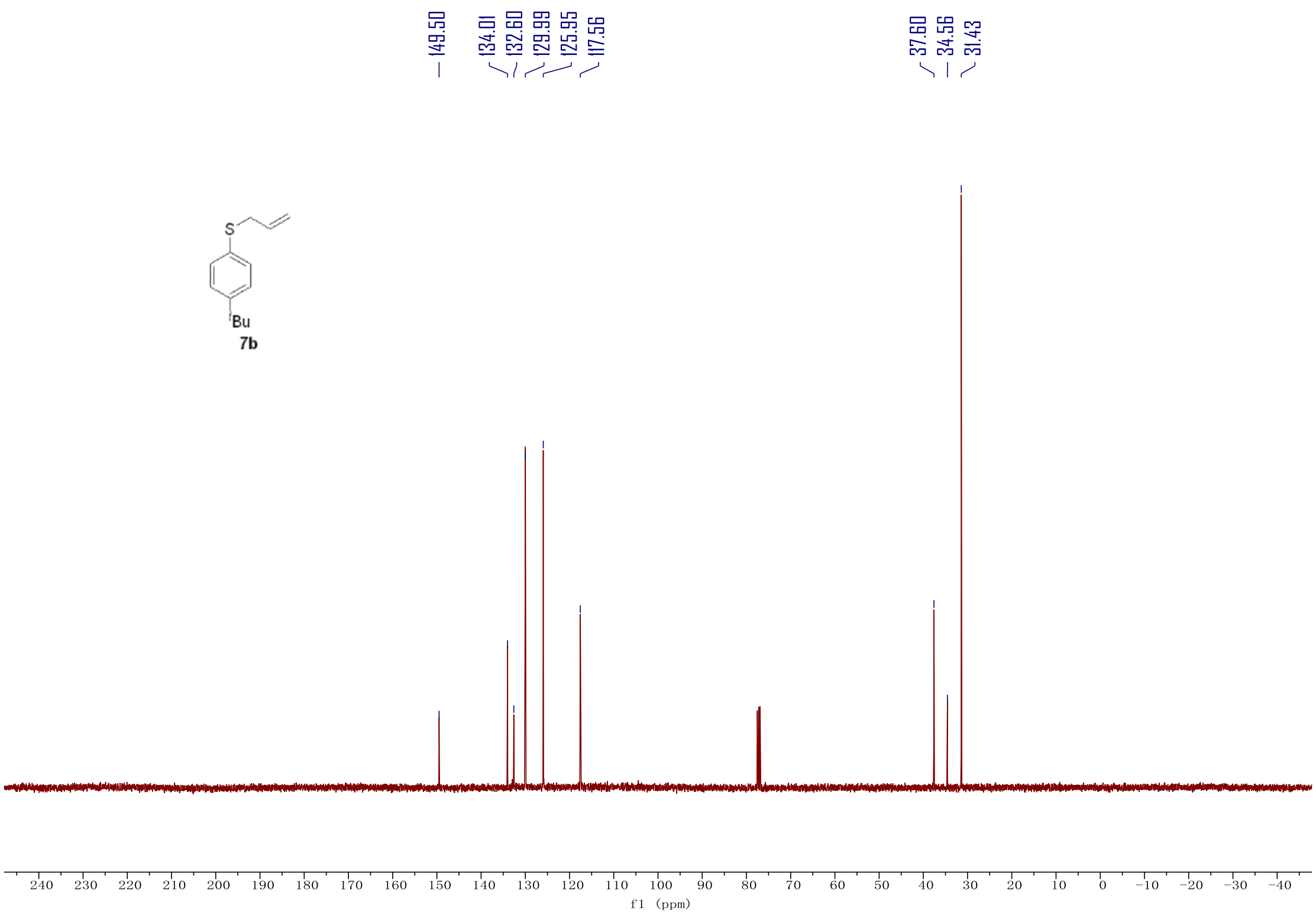

^{1}H NMR Spectrum of **7c** (400 MHz, $CDCl_3$)

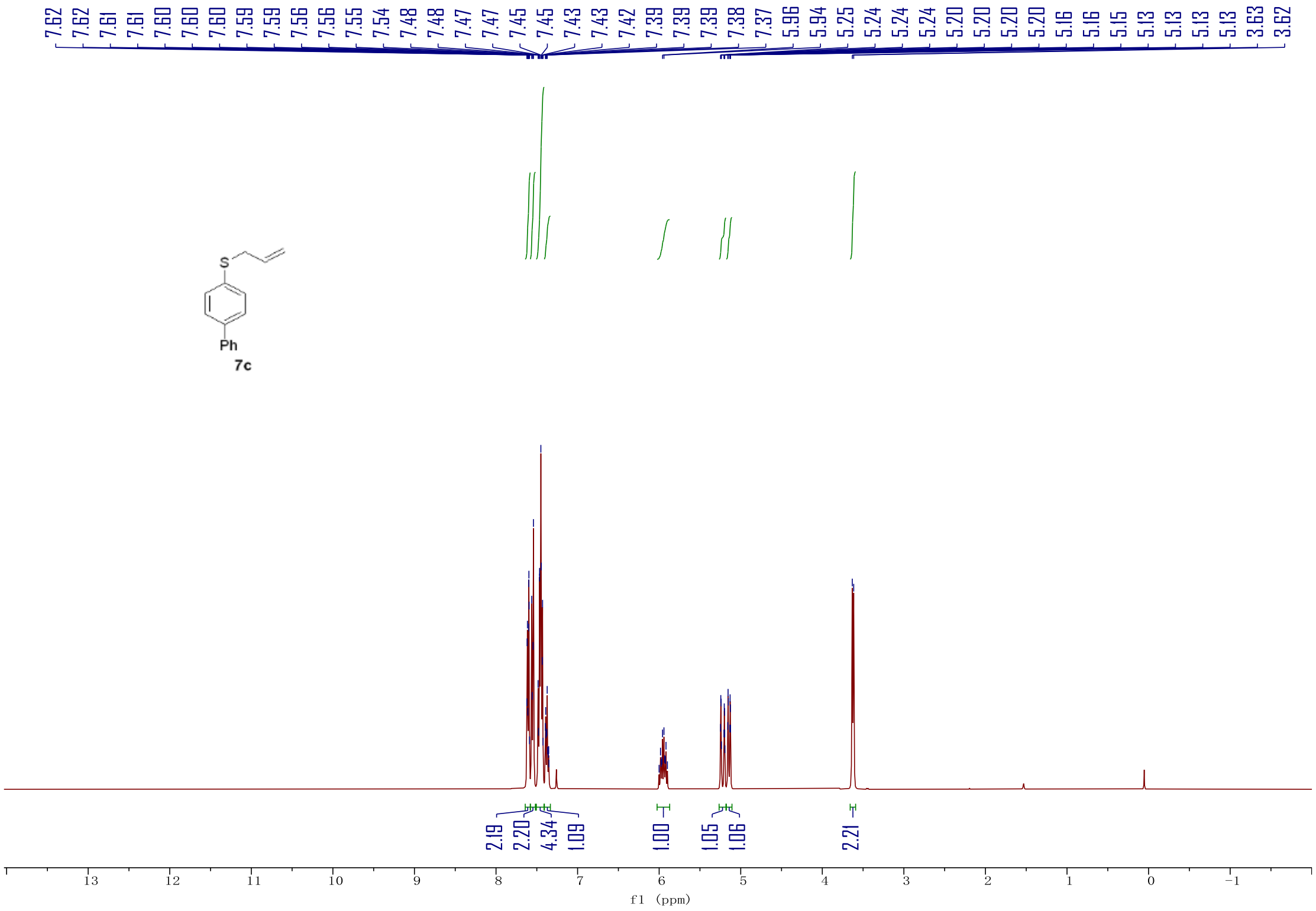

^{13}C NMR Spectrum of **7c** (101 MHz, $CDCl_3$)

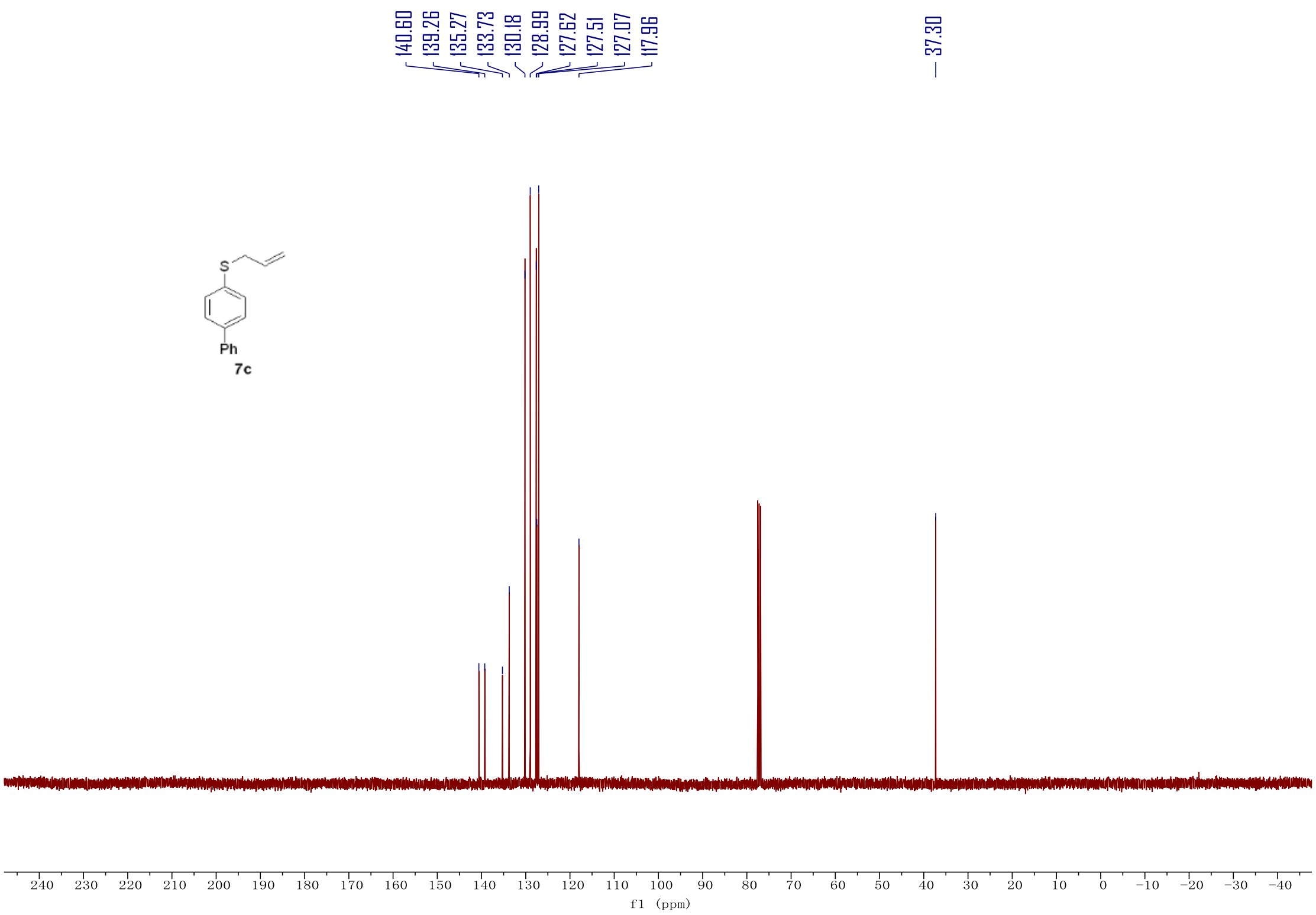

^{1}H NMR Spectrum of **7d** (400 MHz, $CDCl_3$)

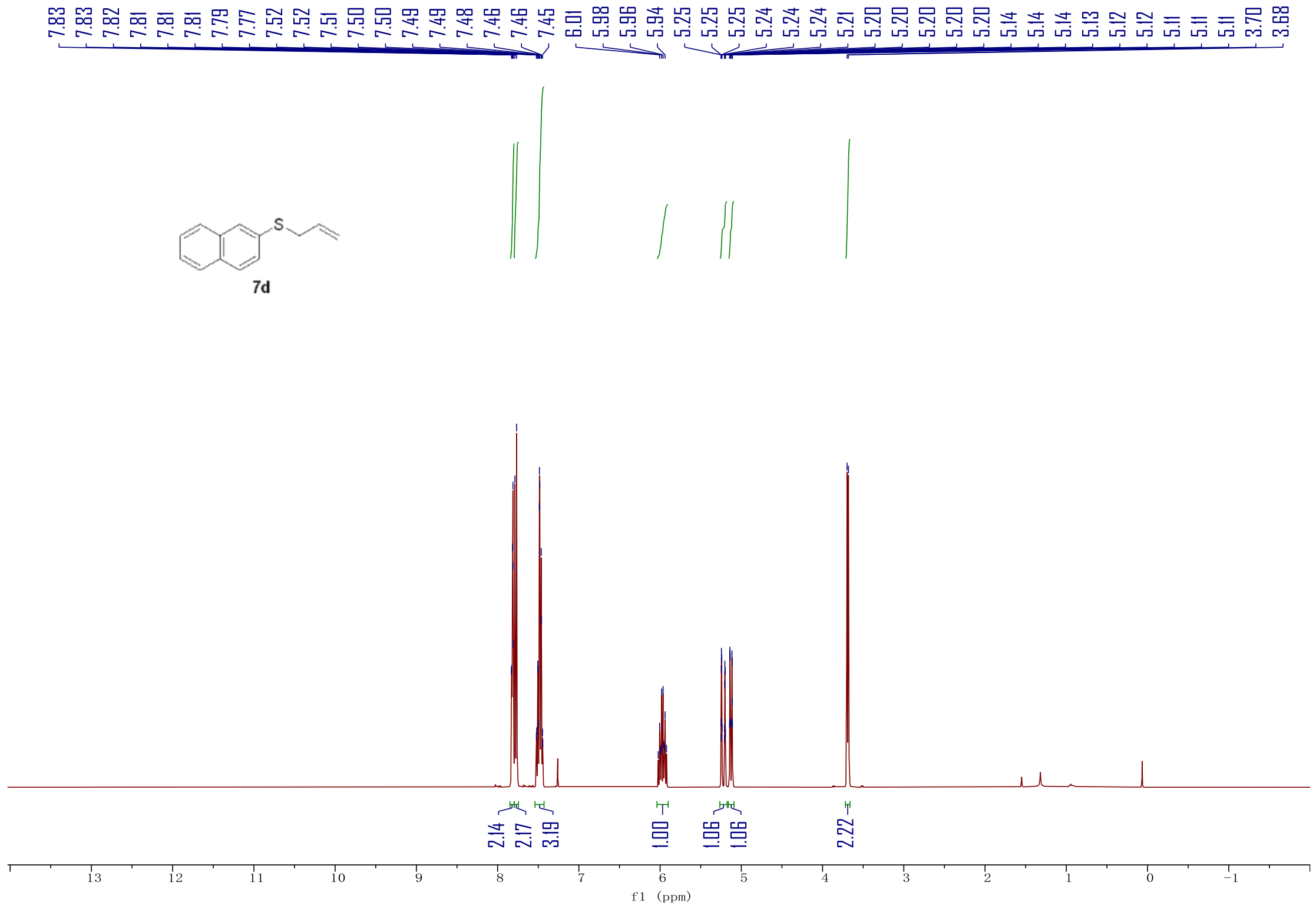

^{13}C NMR Spectrum of **7d** (101 MHz, $CDCl_3$)

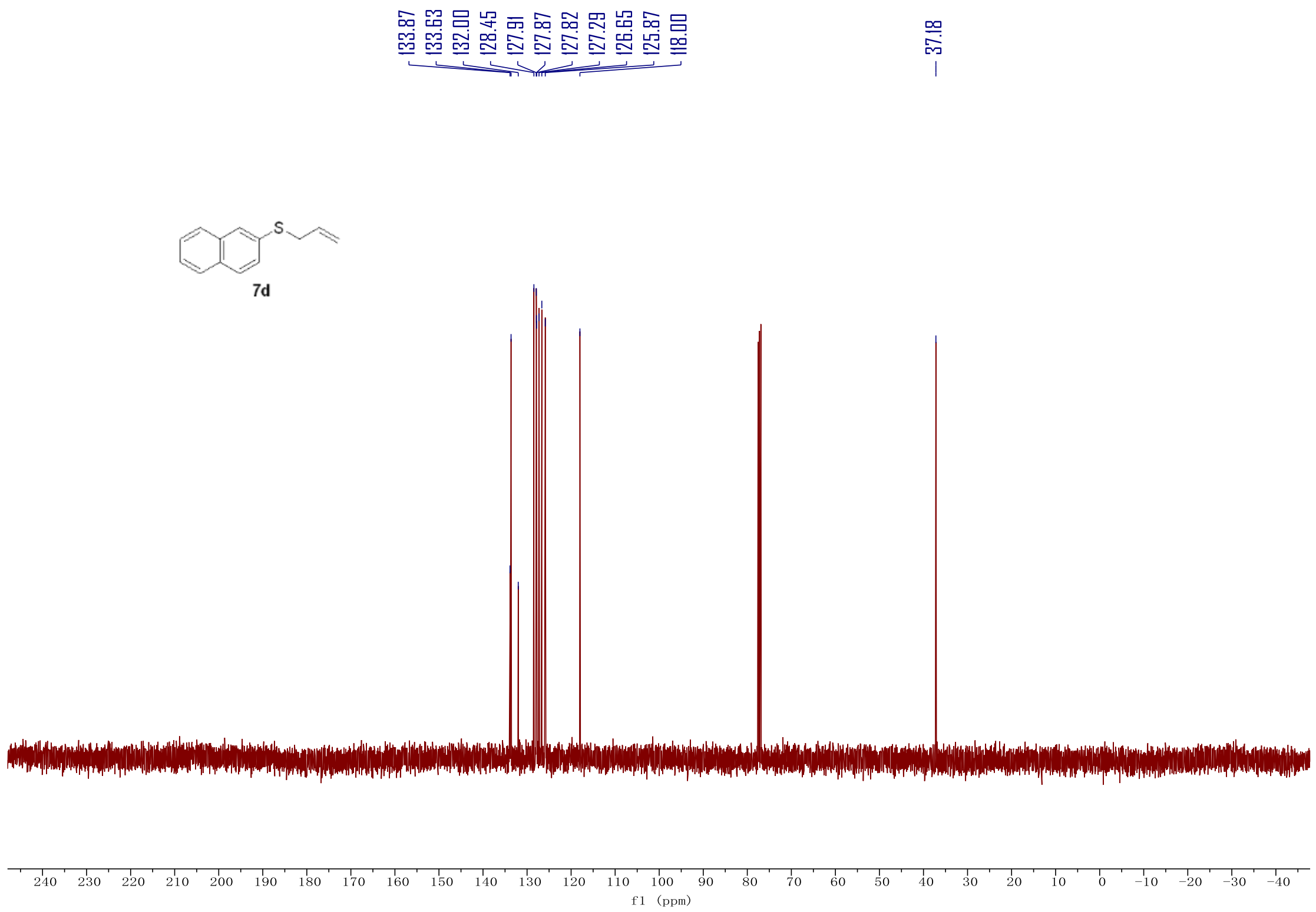

^{1}H NMR Spectrum of **7e** (400 MHz, $CDCl_3$)

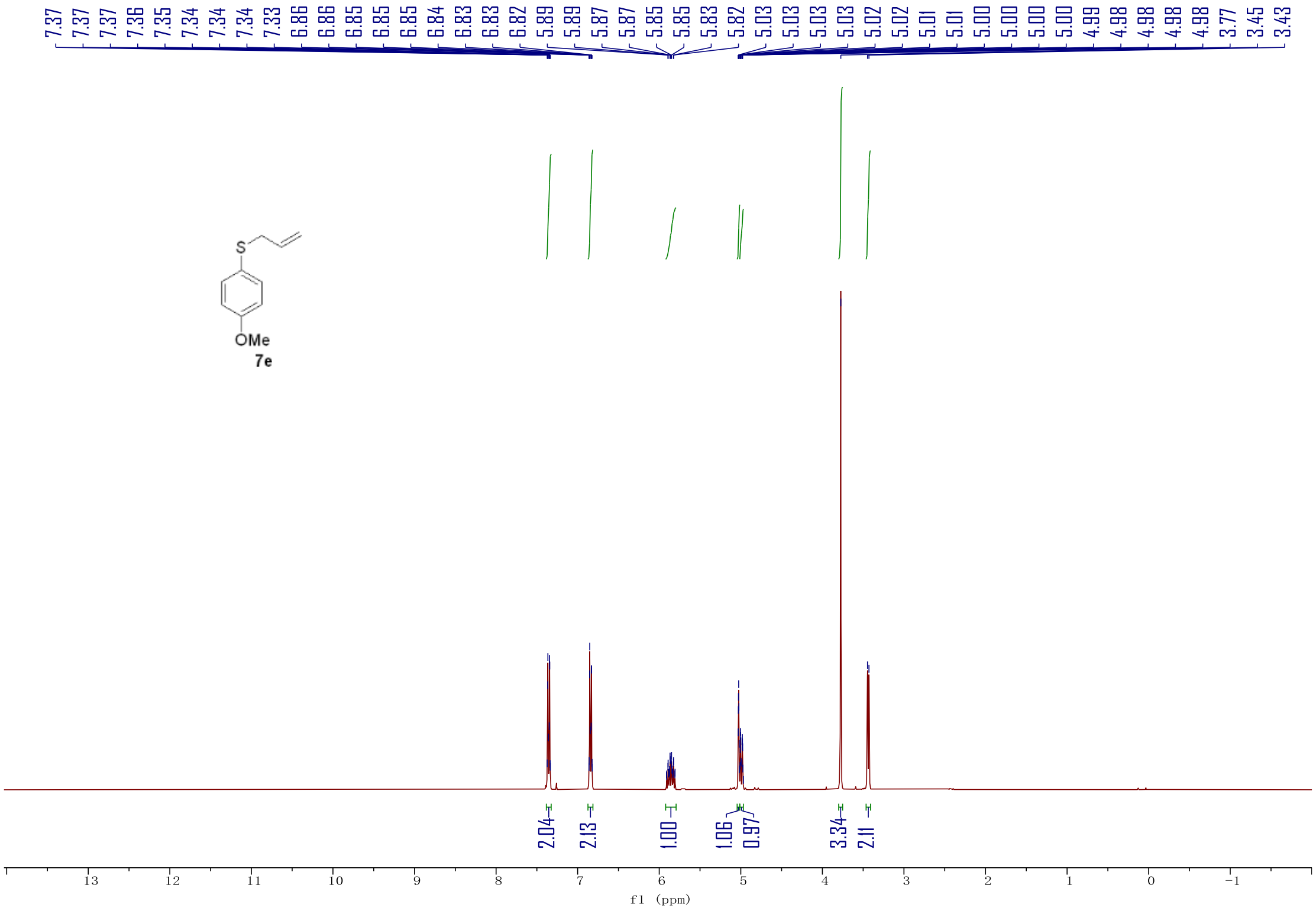

^{13}C NMR Spectrum of **7e** (101 MHz, $CDCl_3$)

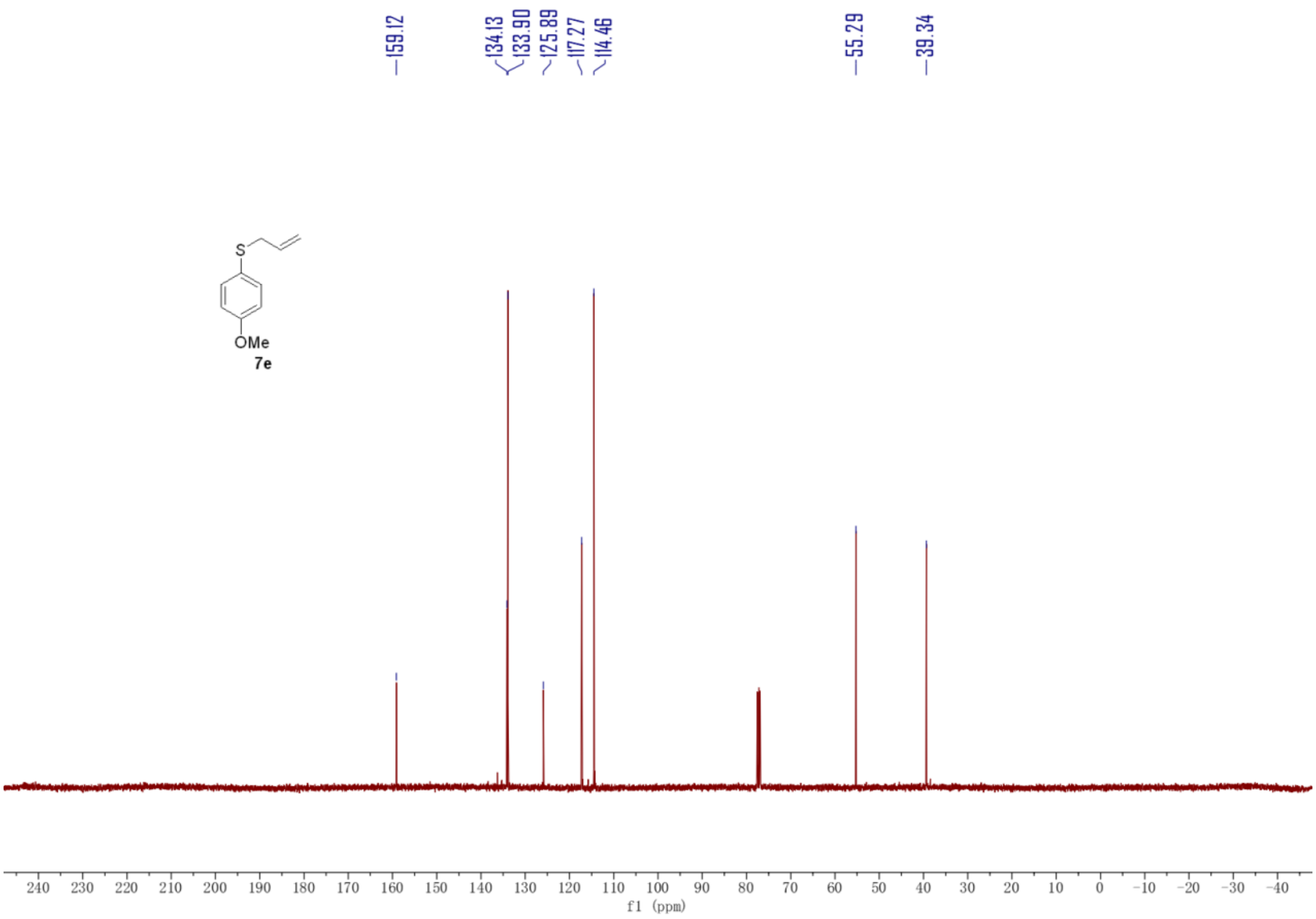

^{1}H NMR Spectrum of **7f** (400 MHz, $CDCl_3$)

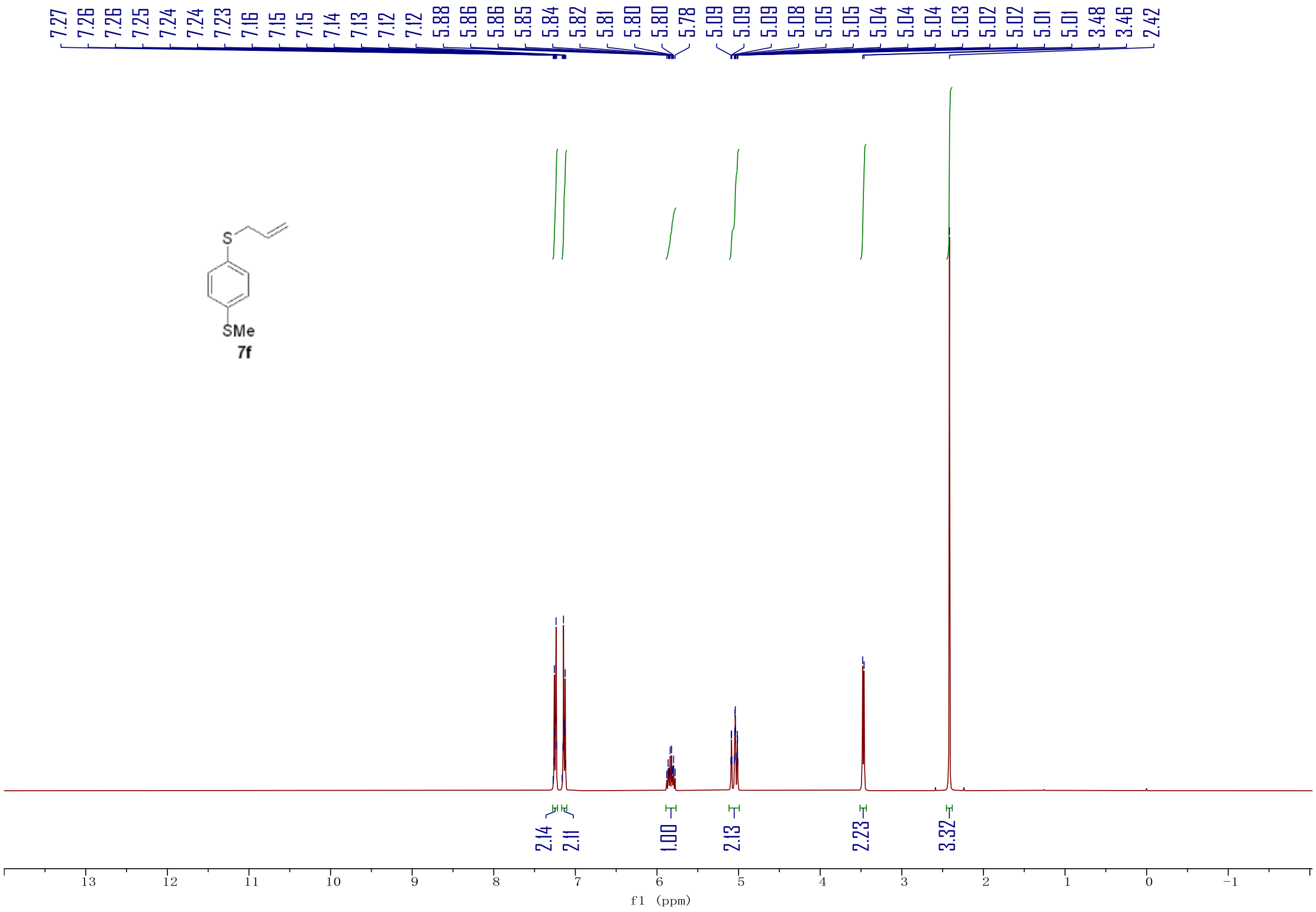

^{13}C NMR Spectrum of **7f** (101 MHz, $CDCl_3$)

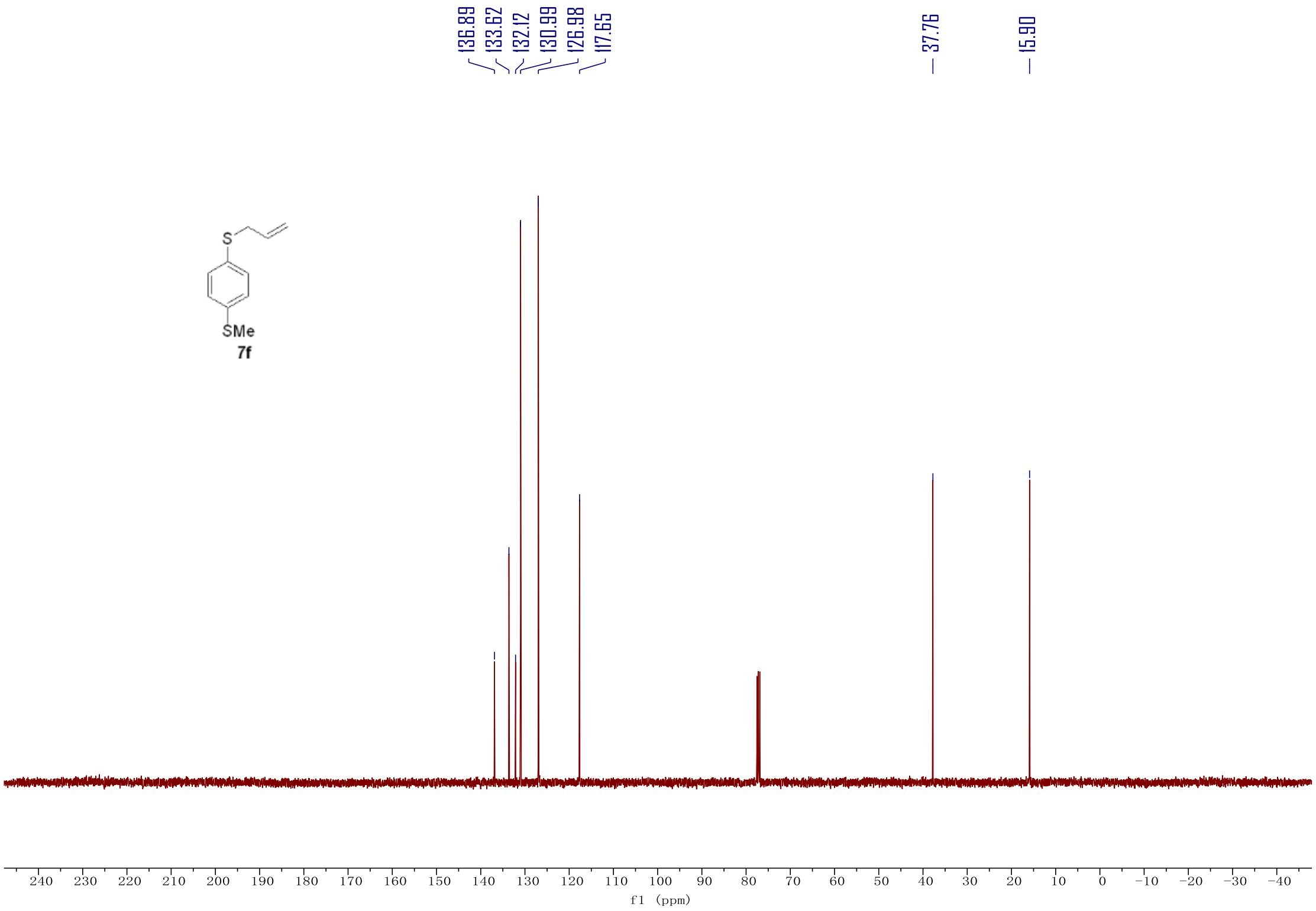

^{1}H NMR Spectrum of **7g** (400 MHz, $CDCl_3$)

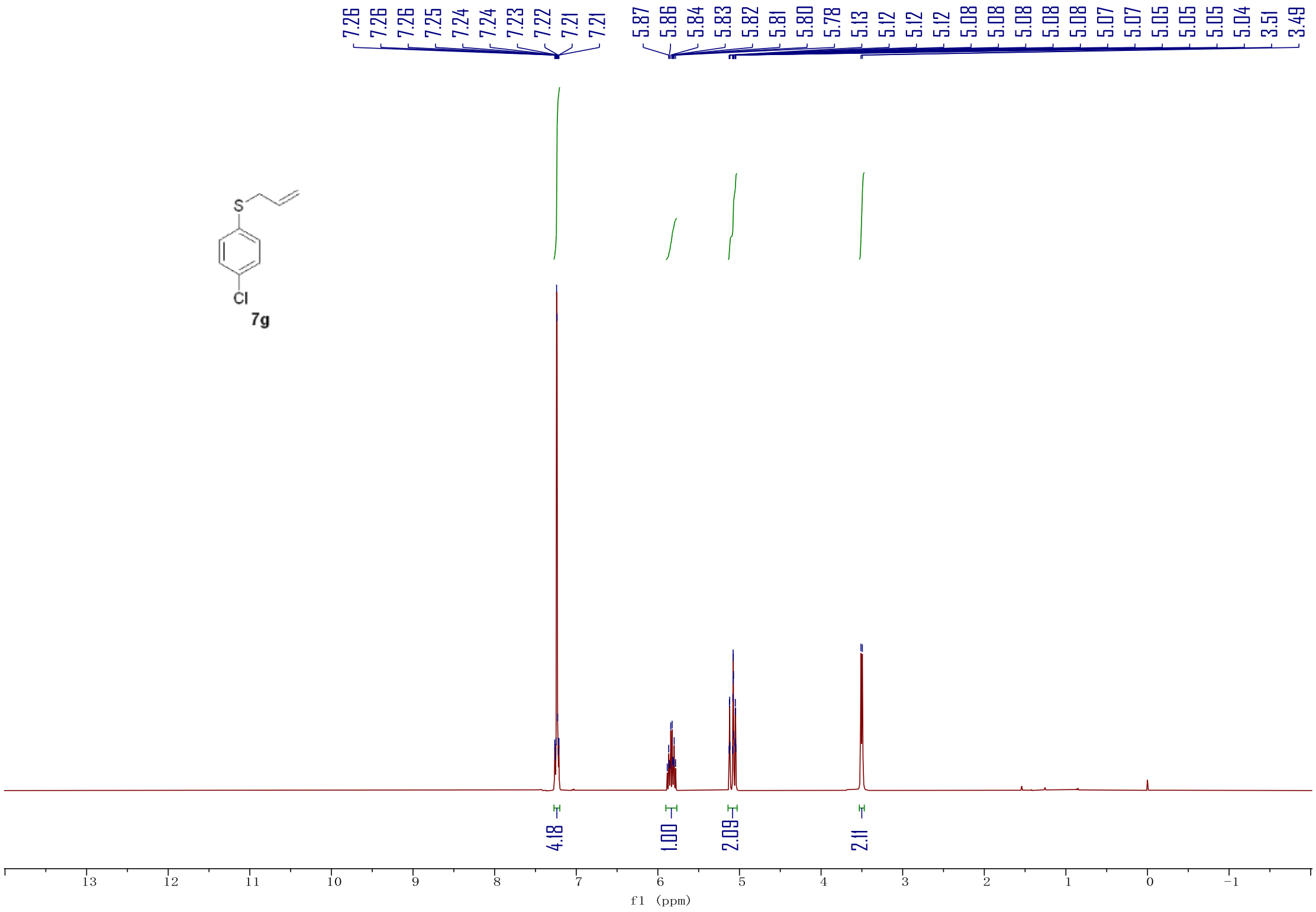

^{13}C NMR Spectrum of **7g** (101 MHz, $CDCl_3$)

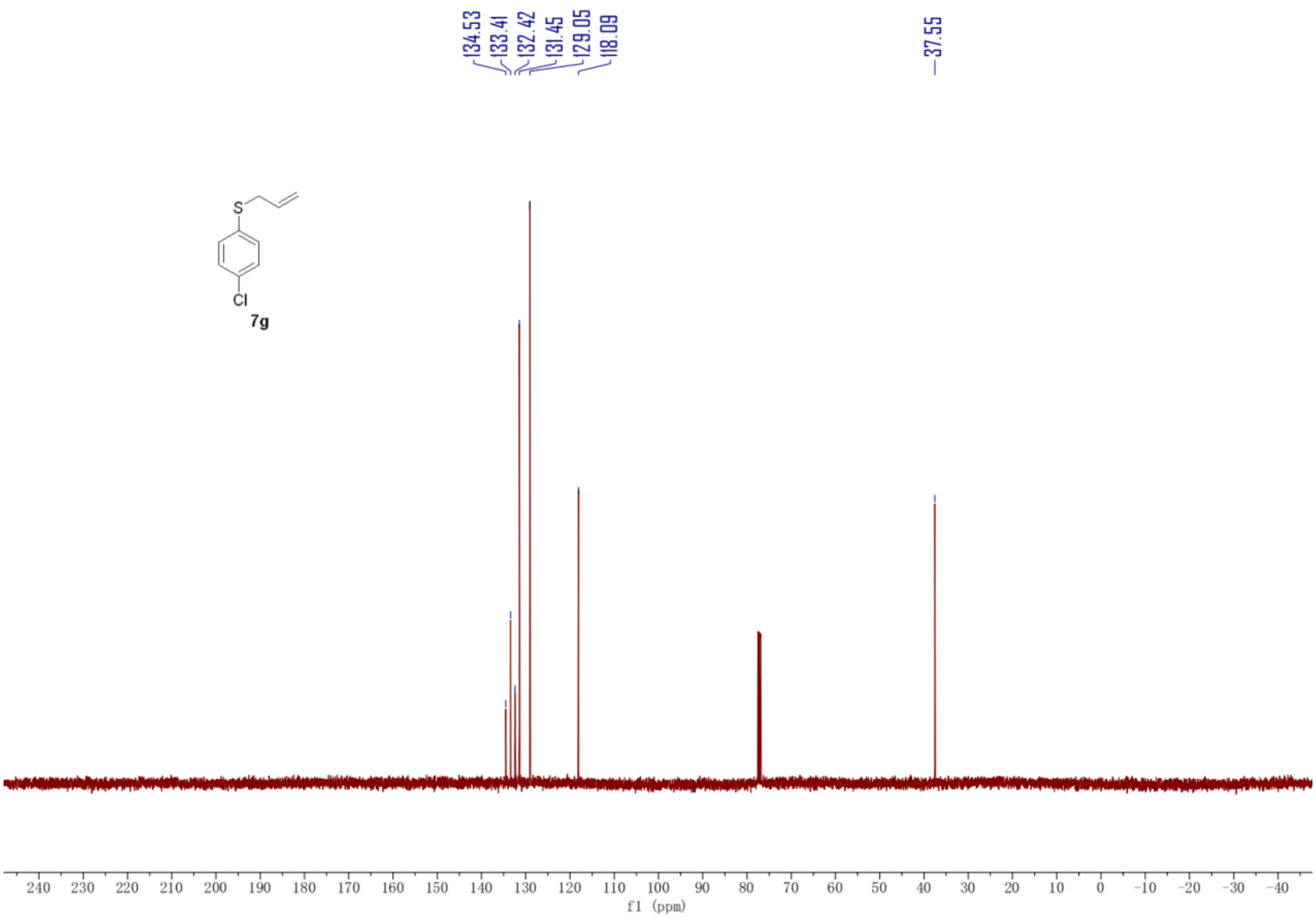

^{1}H **NMR** Spectrum of **7h** (400 MHz, $CDCl_3$)

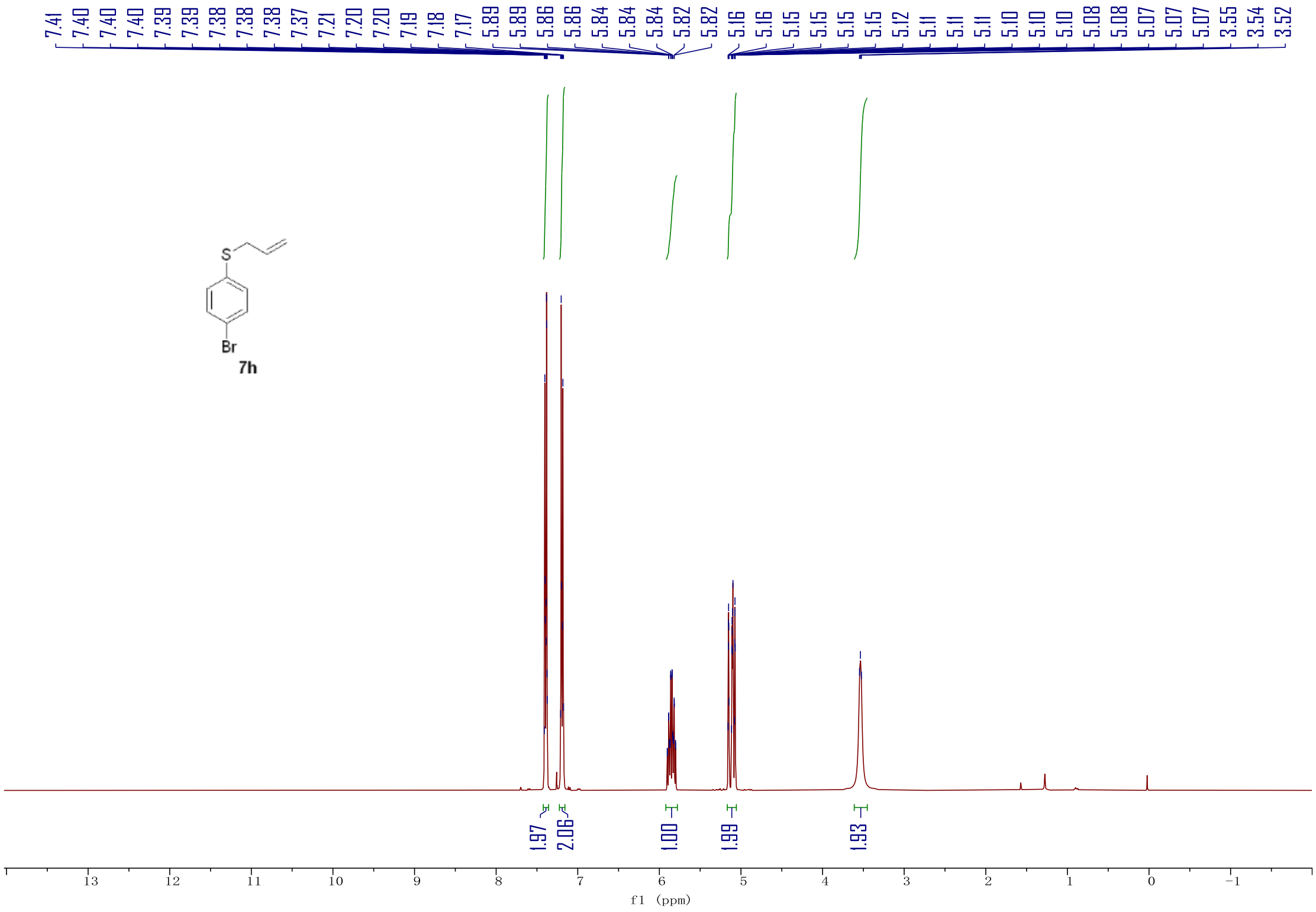

^{13}C **NMR** Spectrum of **7h** (101 MHz, $CDCl_3$)

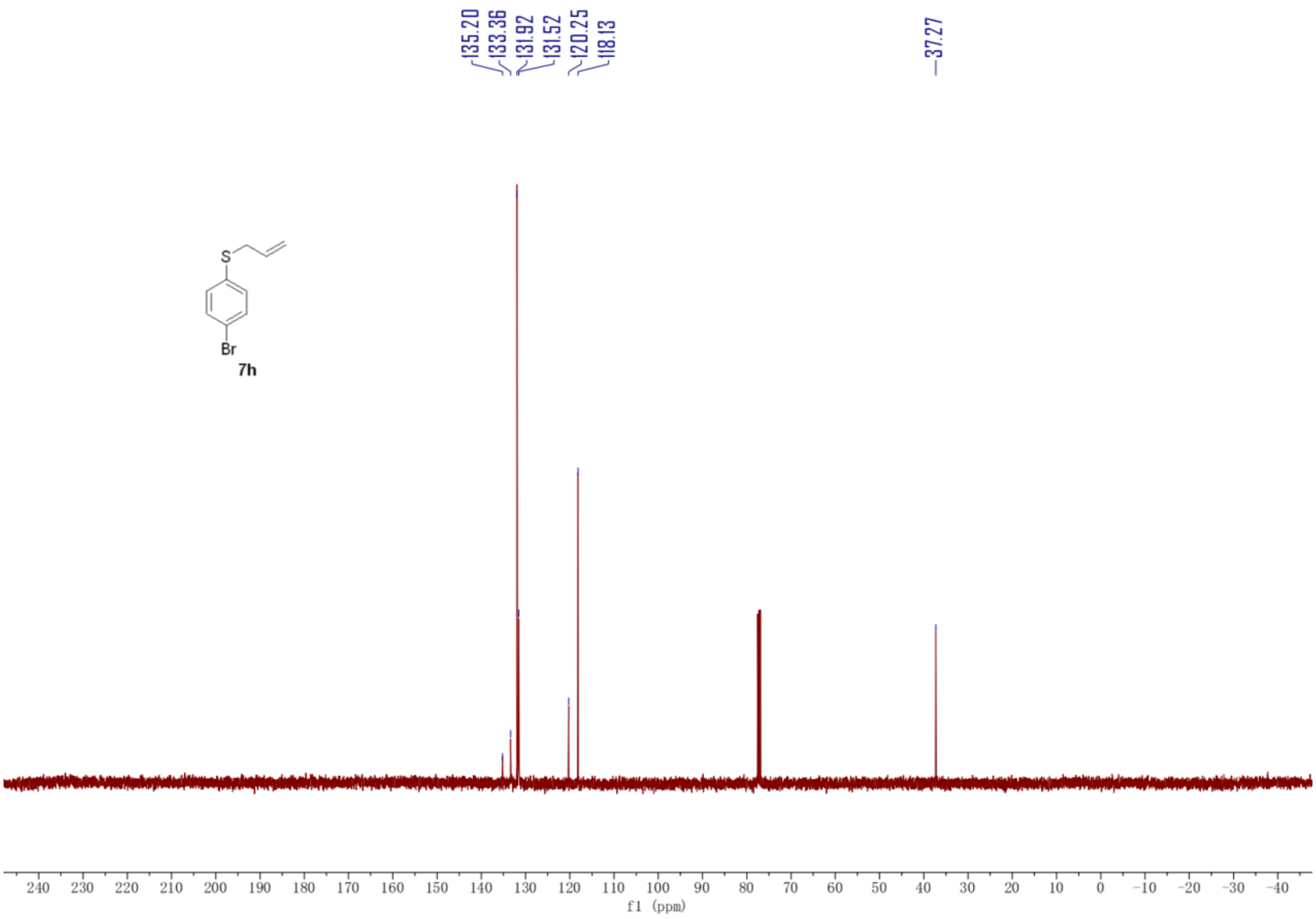

^{1}H NMR Spectrum of **7i** (400 MHz, $CDCl_3$)

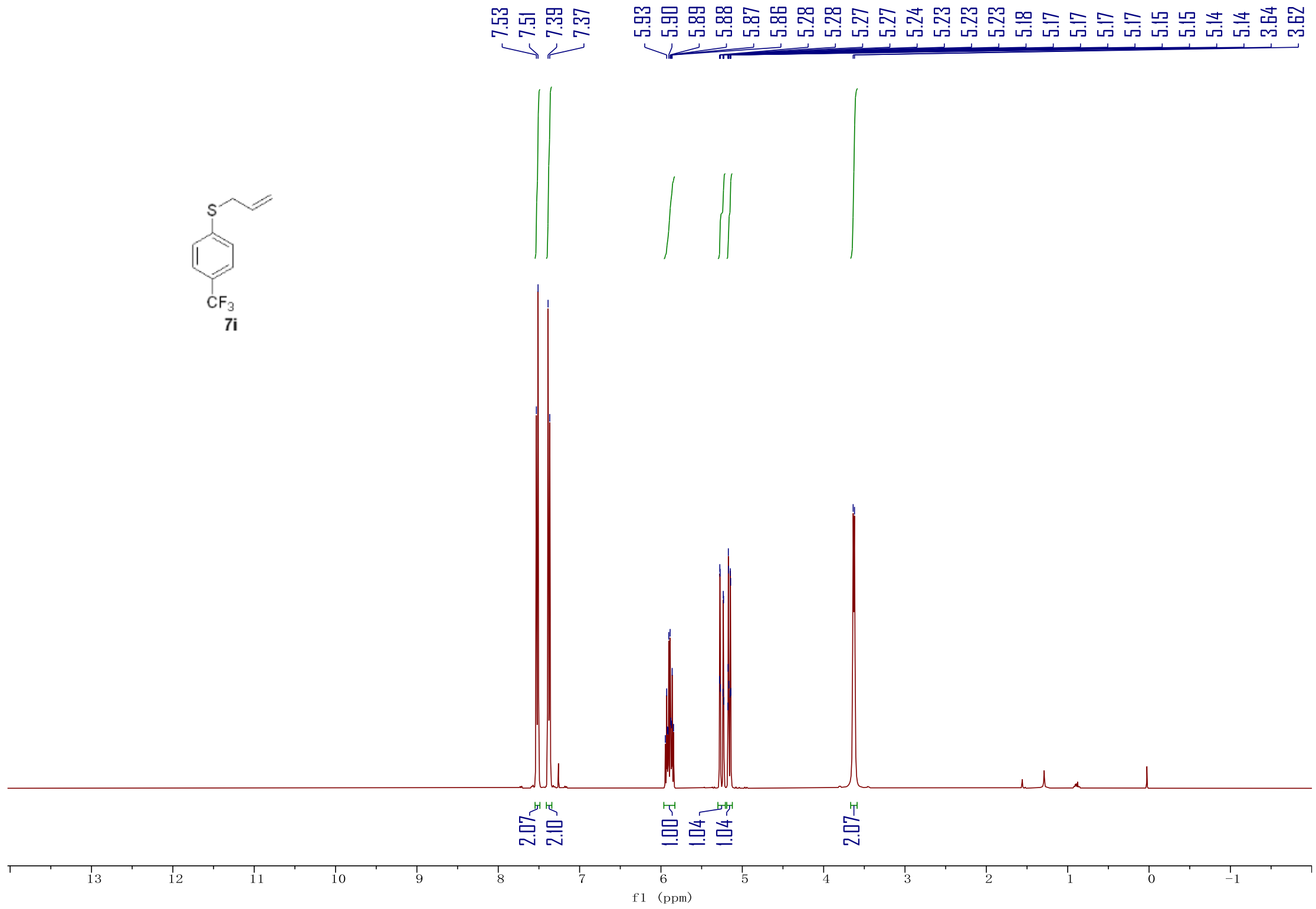

^{19}F NMR Spectrum of **7i** (376 MHz, $CDCl_3$)

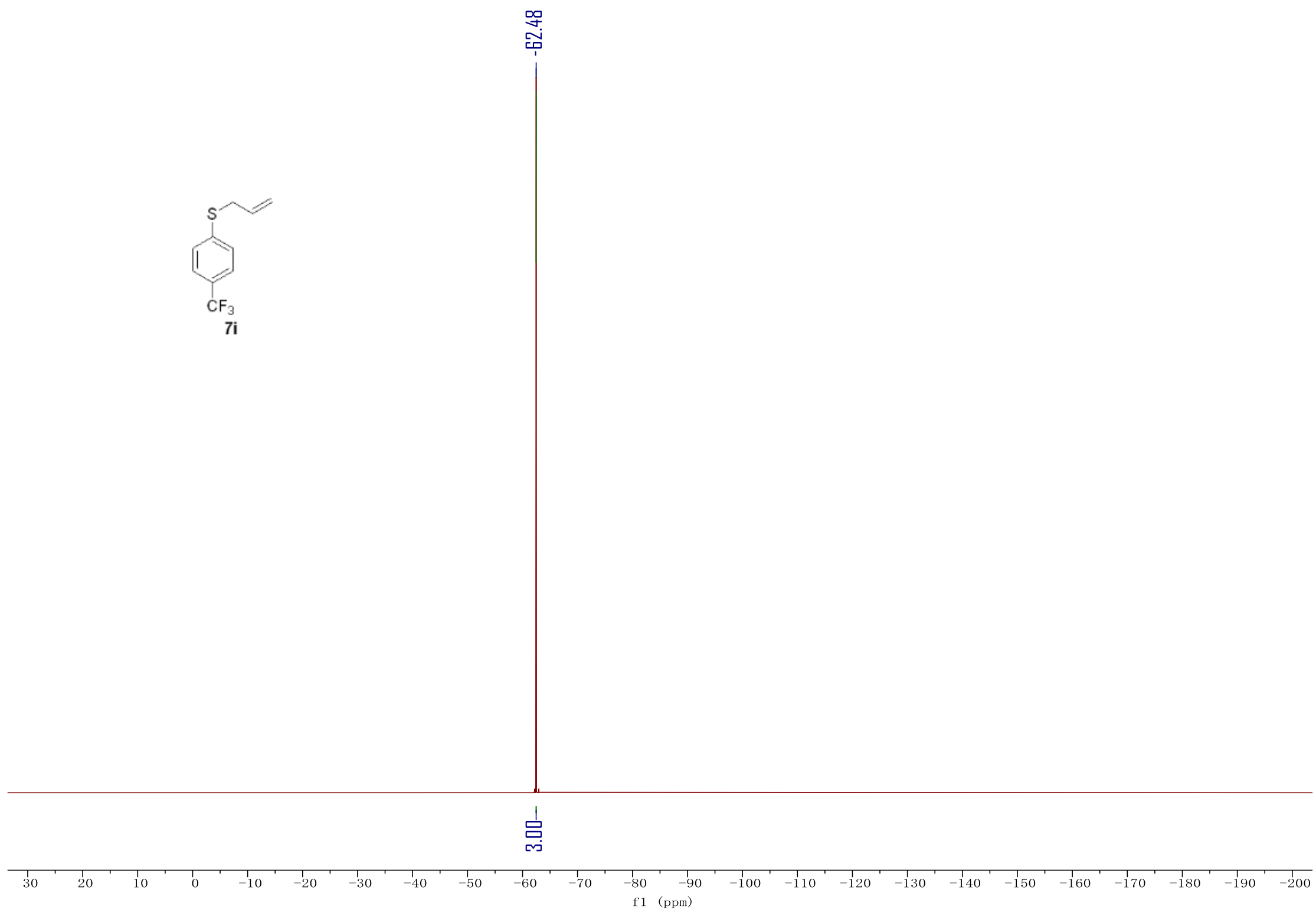

^{13}C NMR Spectrum of **7i** (101 MHz, $CDCl_3$)

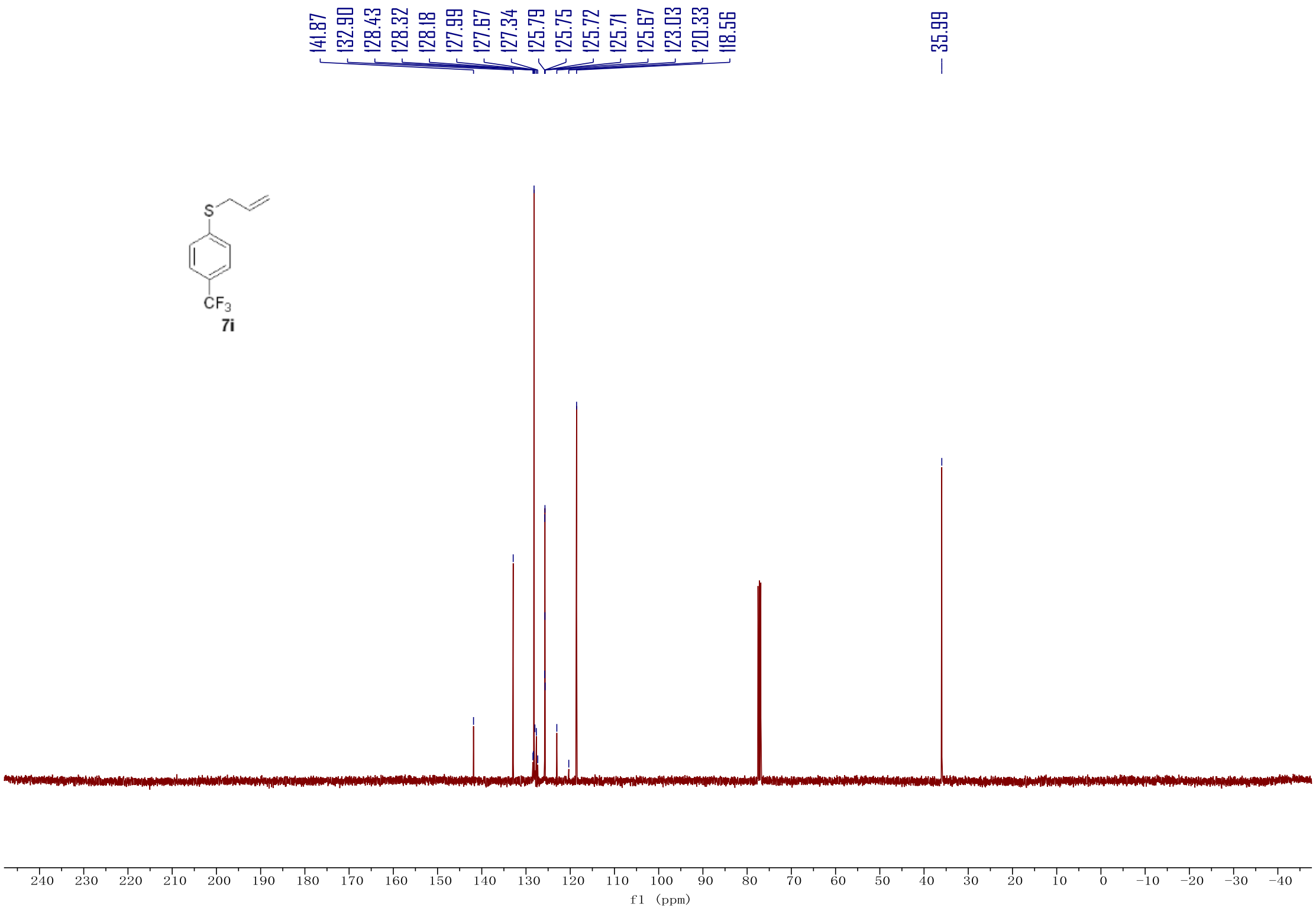

^{1}H NMR Spectrum of **7j** (400 MHz, $CDCl_3$)

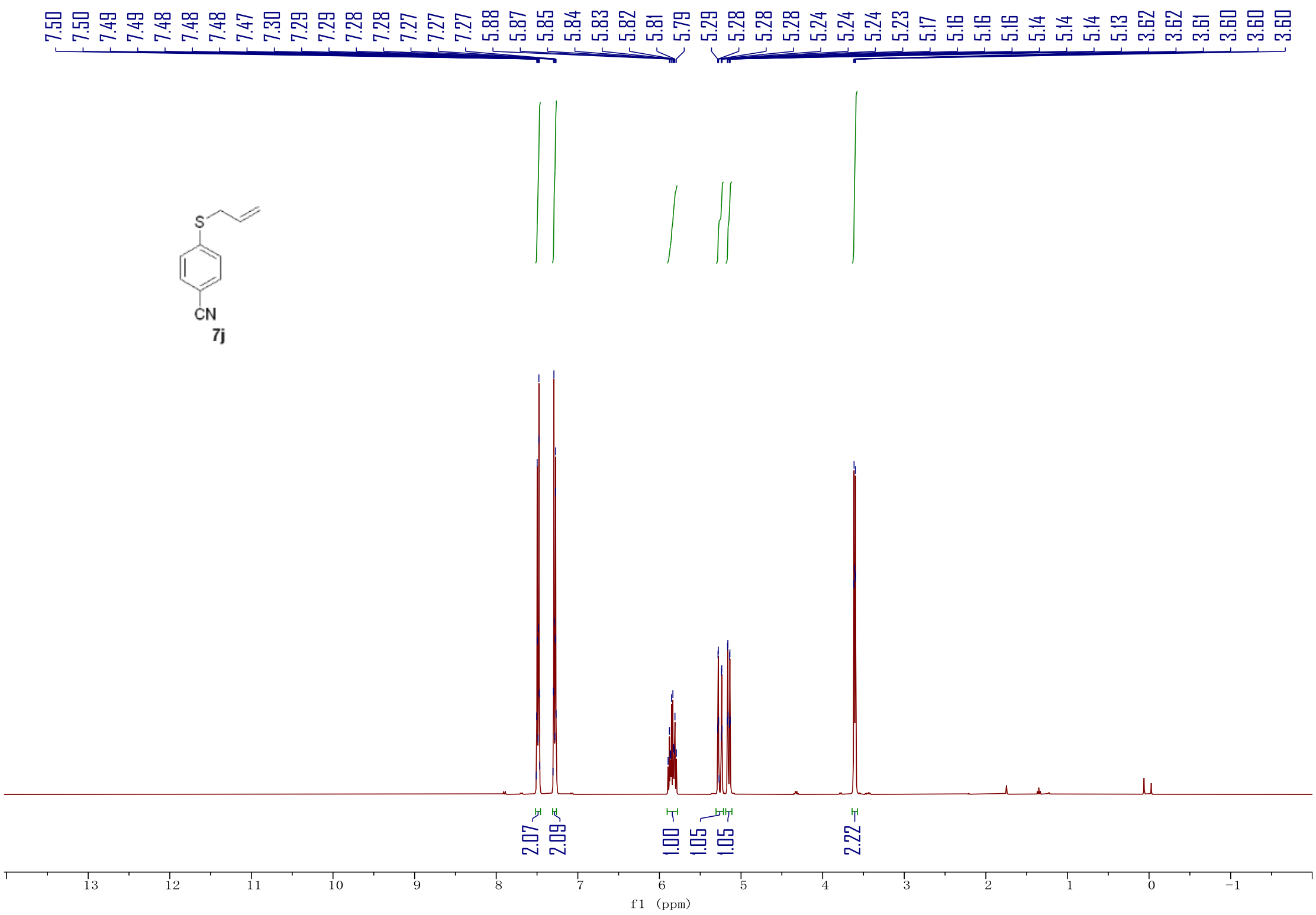

^{13}C NMR Spectrum of **7j** (101 MHz, $CDCl_3$)

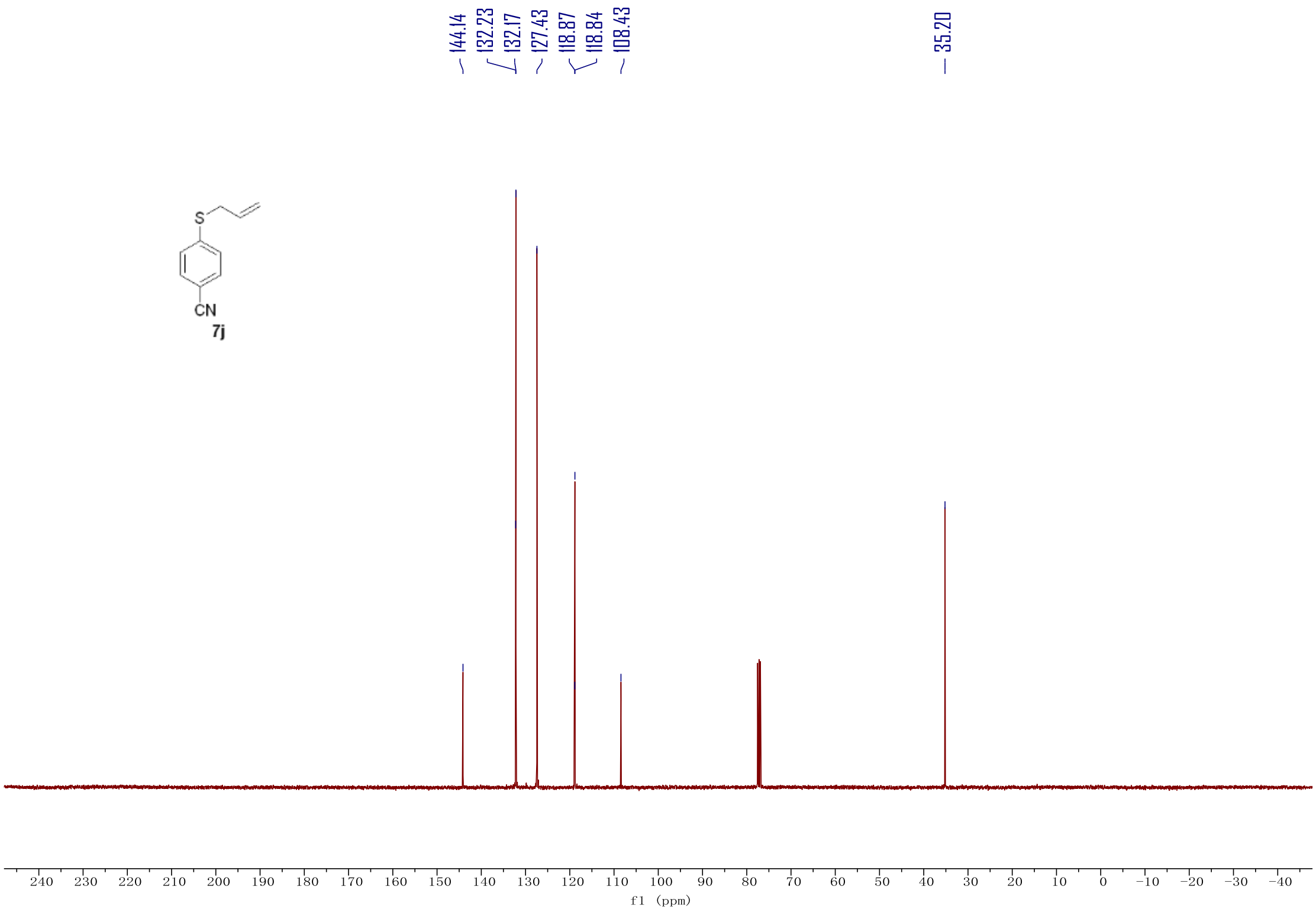

^{1}H NMR Spectrum of **7k** (400 MHz, $CDCl_3$)

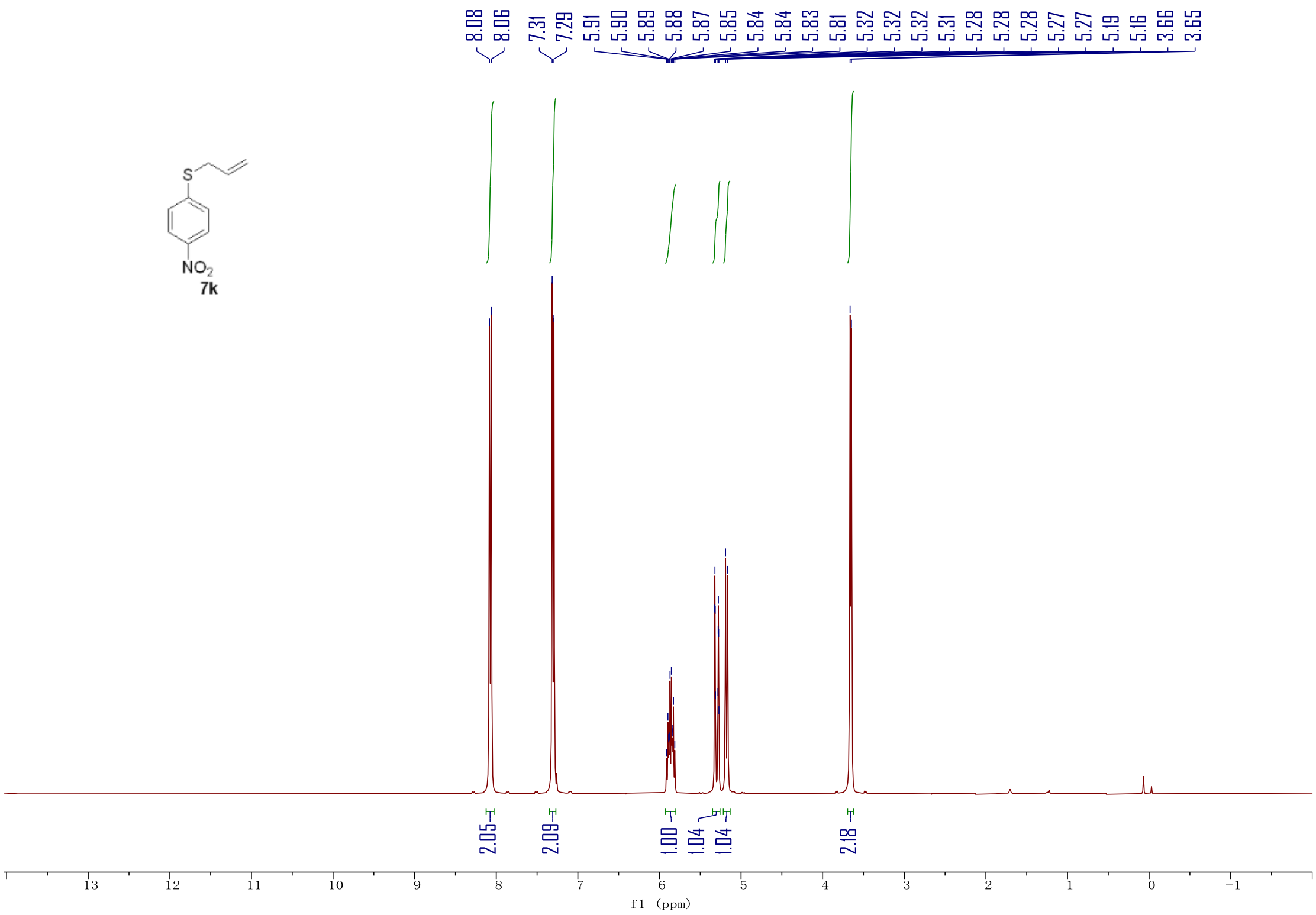

^{13}C NMR Spectrum of **7k** (101 MHz, $CDCl_3$)

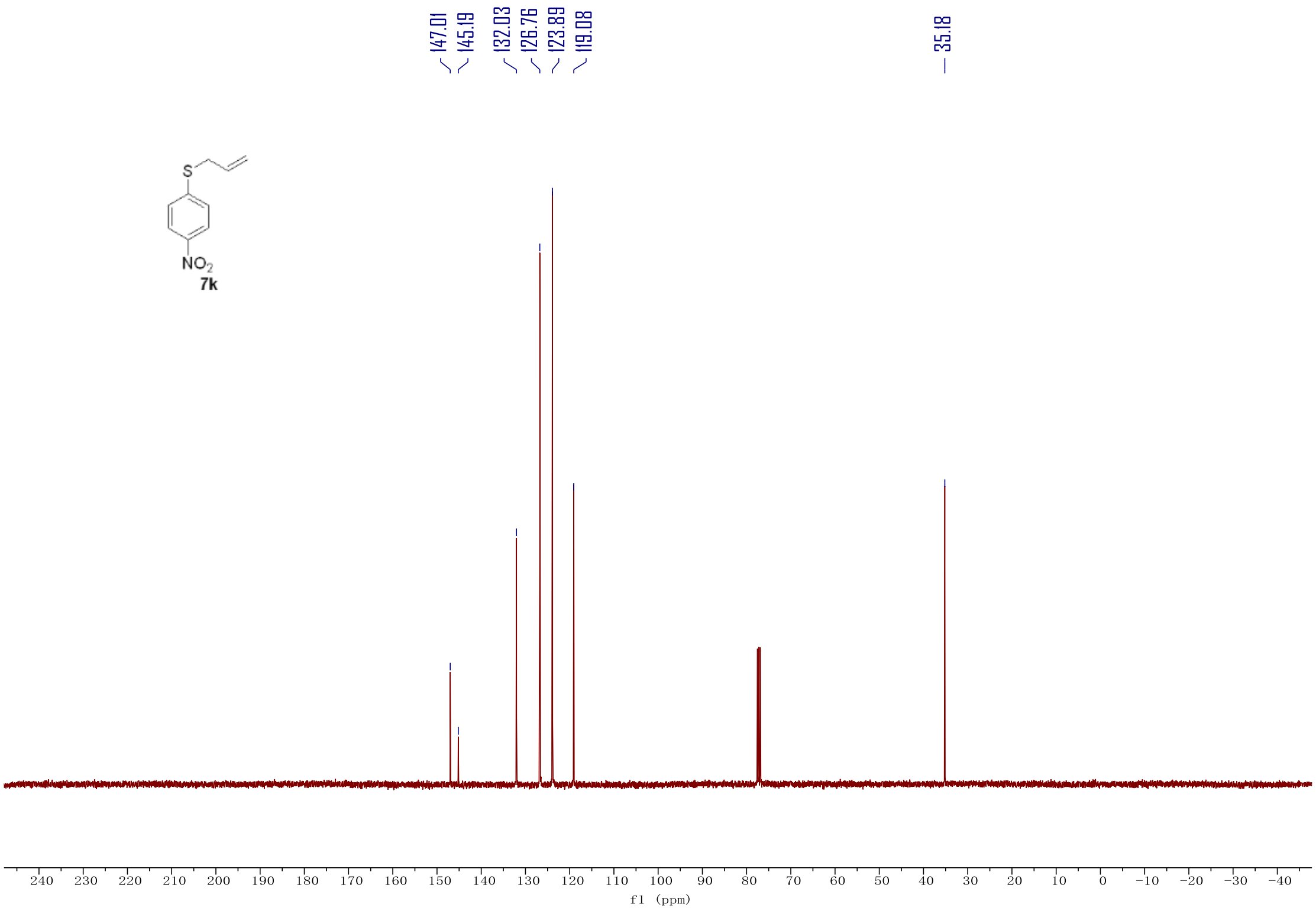

^{1}H NMR Spectrum of **7l** (400 MHz, $CDCl_3$)

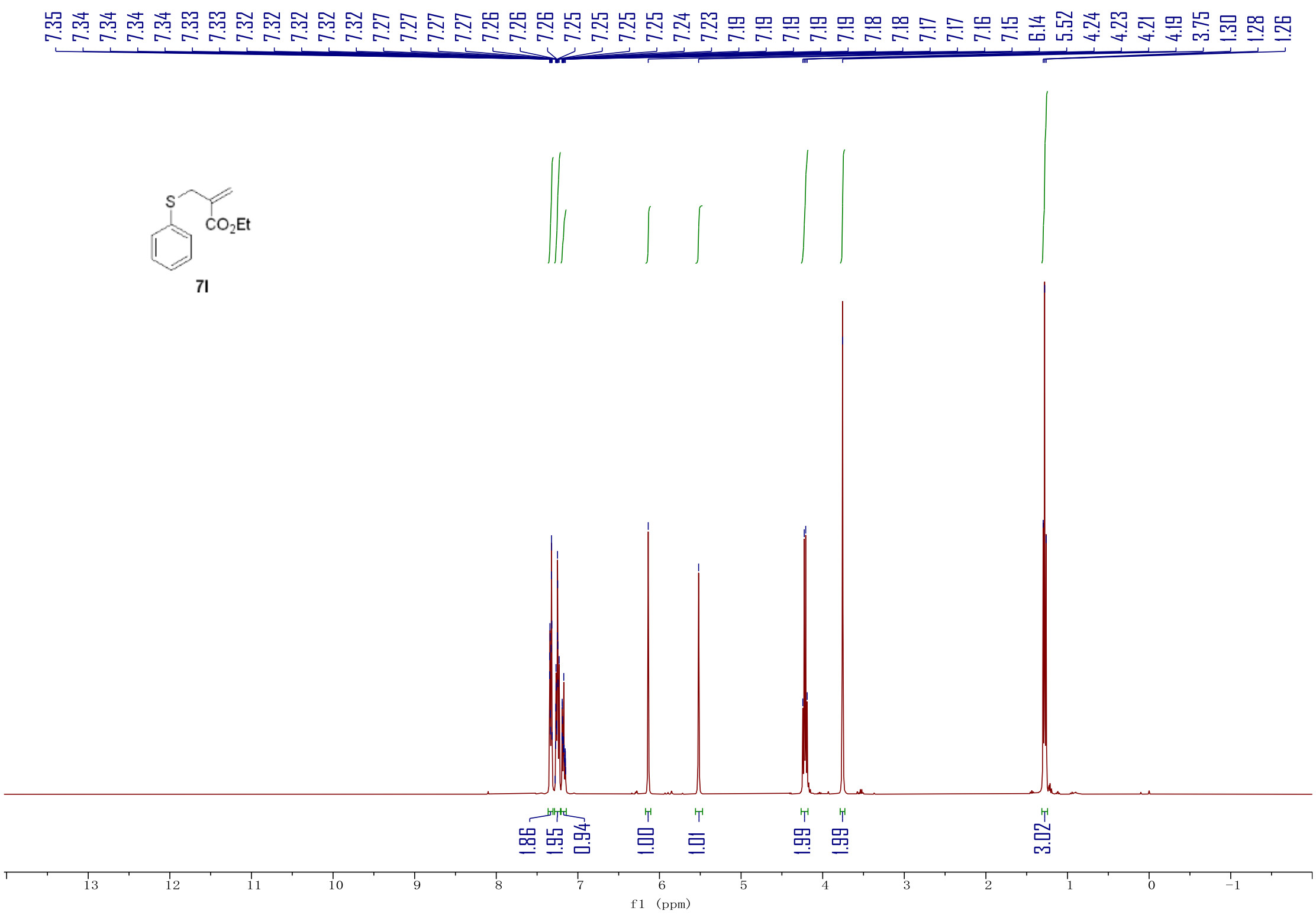

13C NMR Spectrum of **7l** (101 MHz, CDCl3)

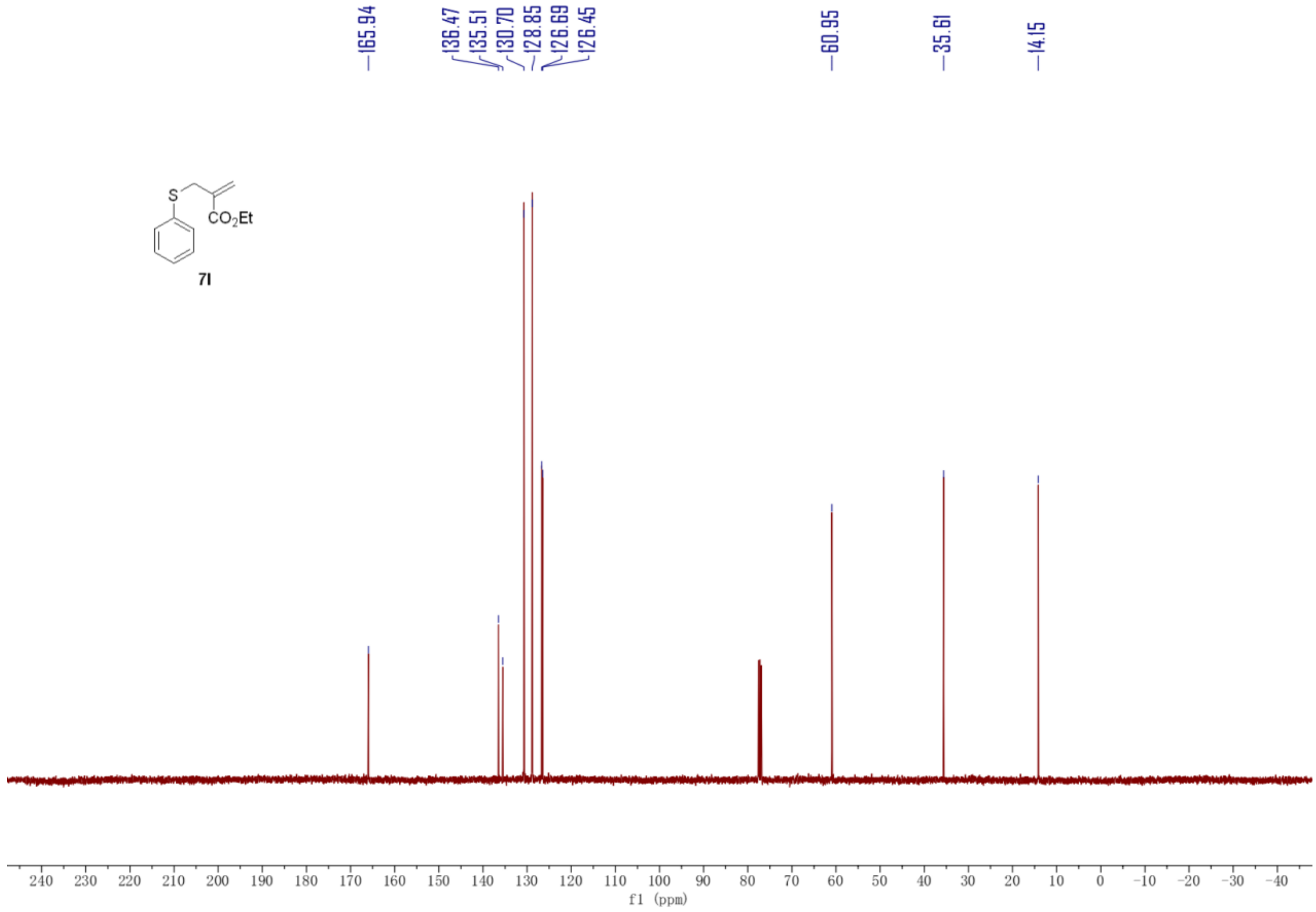

1H NMR Spectrum of **7m** (400 MHz, CDCl3)

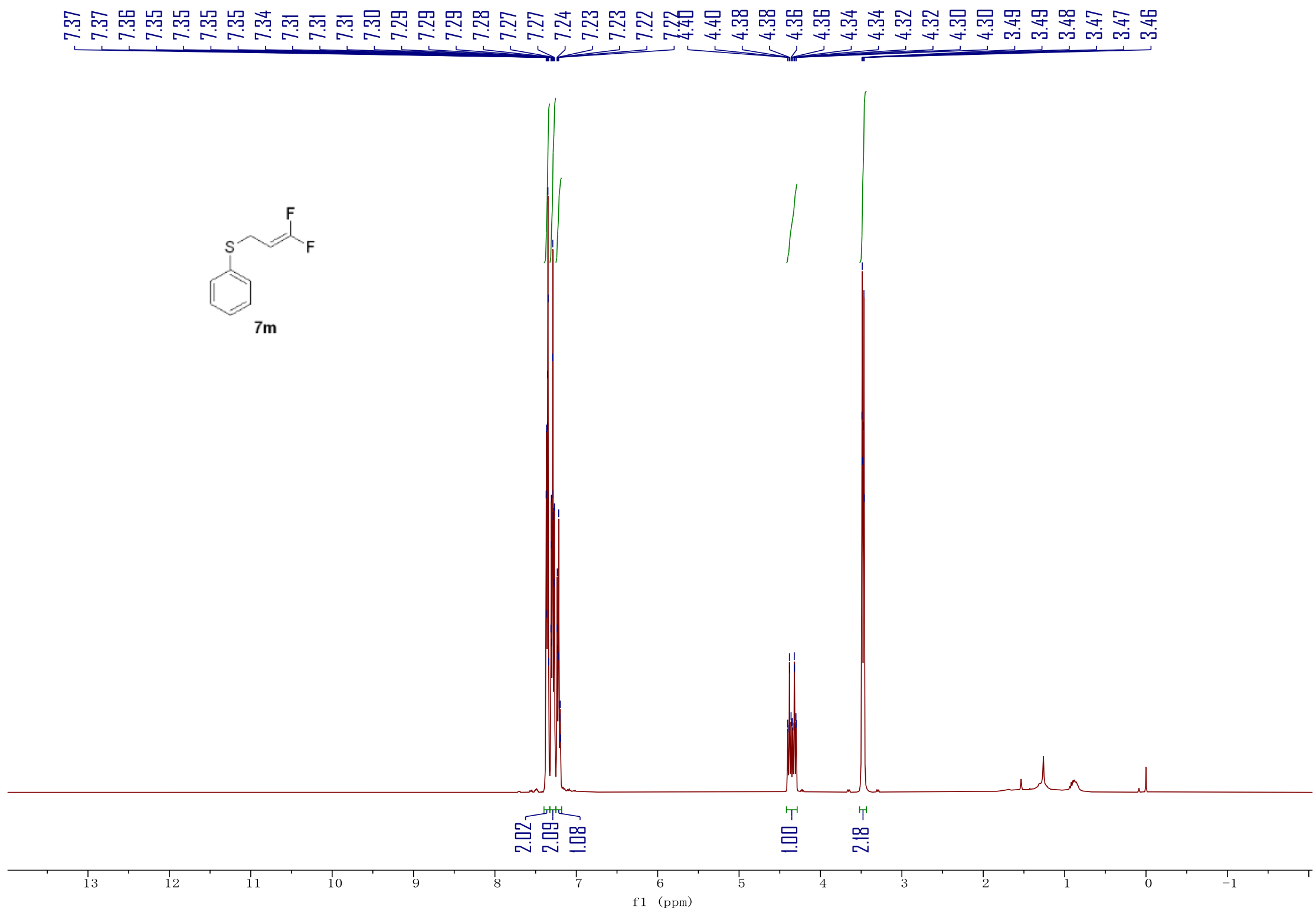

^{19}F NMR Spectrum of **7m** (376 MHz, $CDCl_3$)

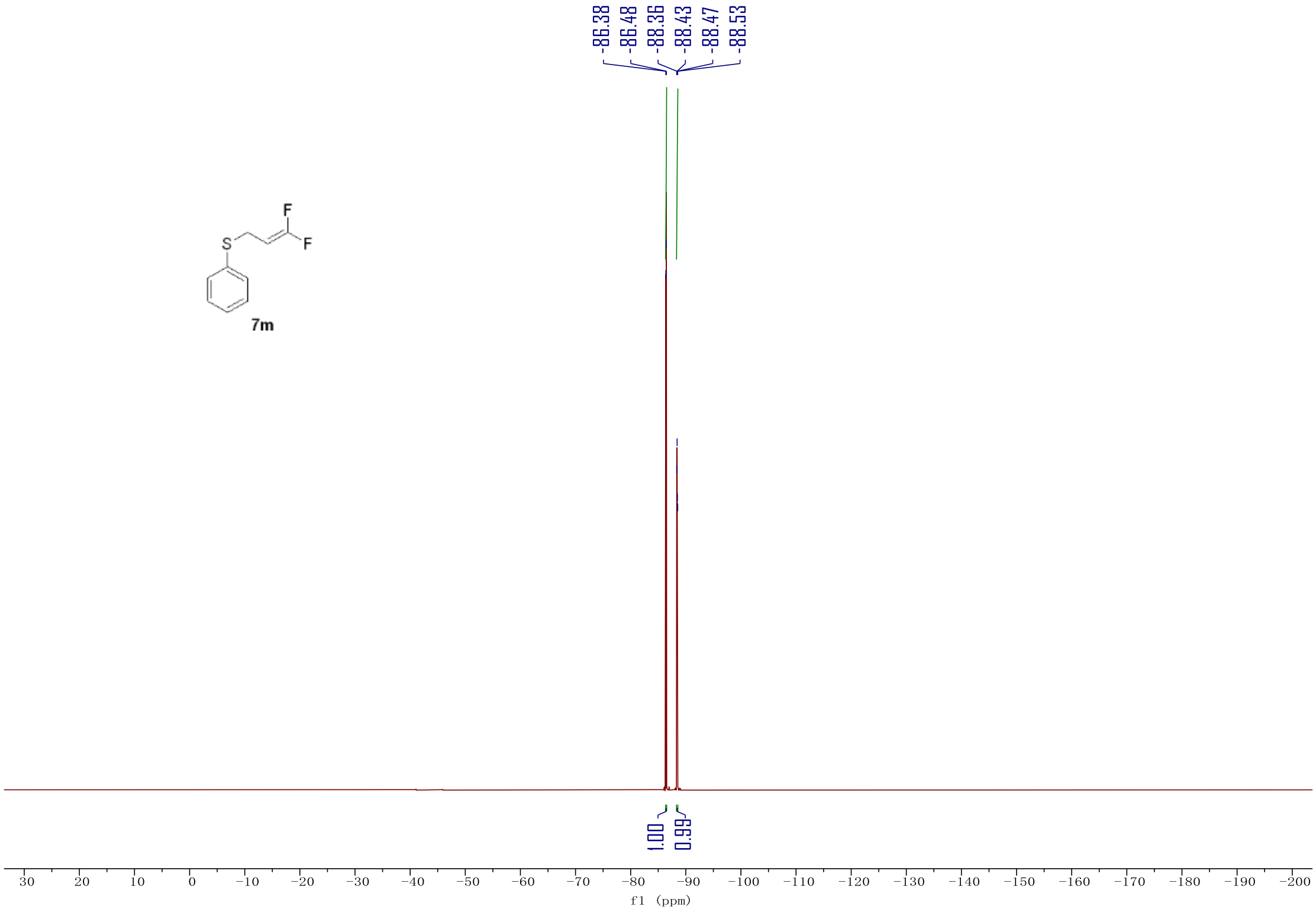

^{13}C NMR Spectrum of **7m** (101 MHz, $CDCl_3$)

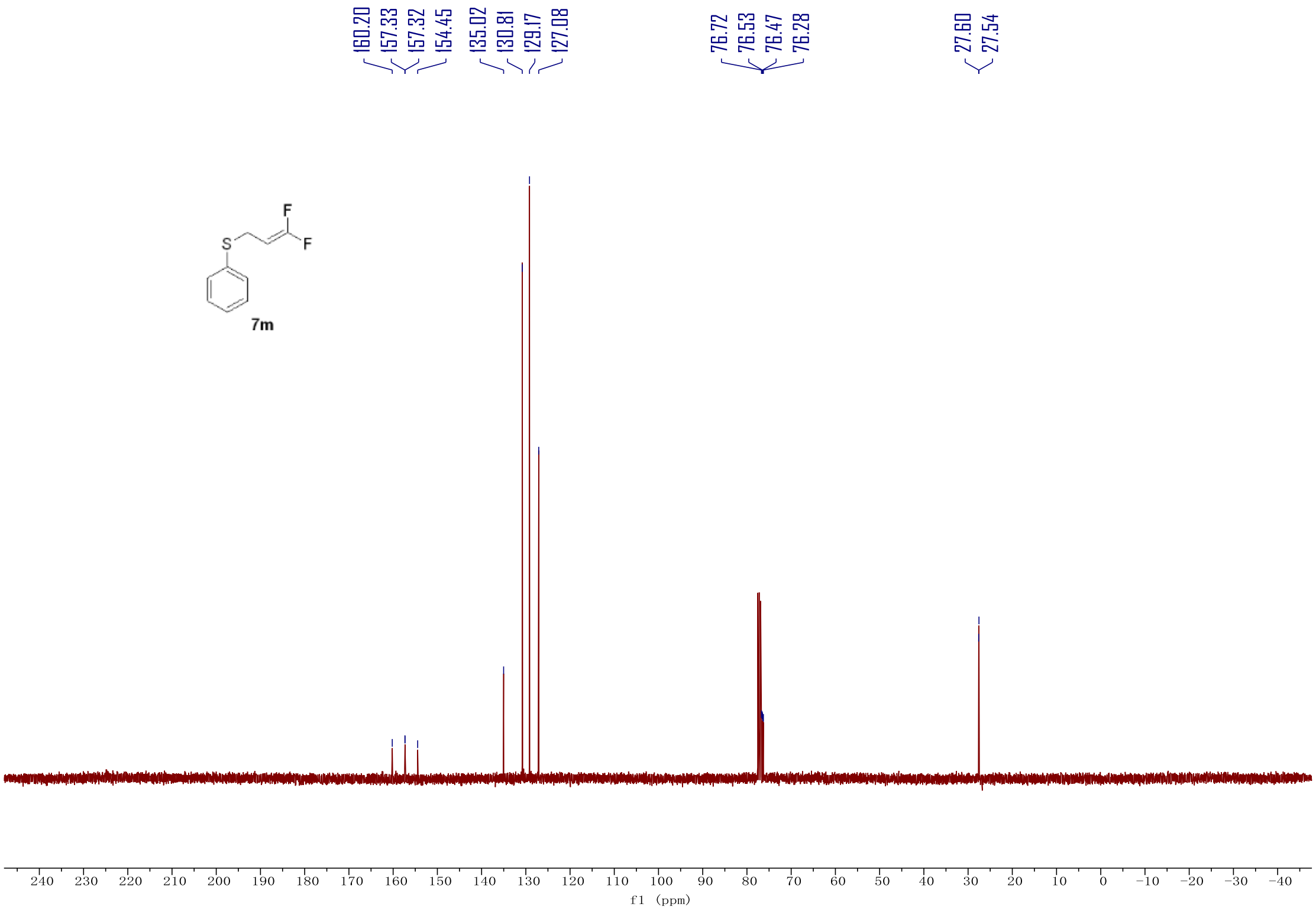

^{1}H NMR Spectrum of **7n** (400 MHz, $CDCl_3$)

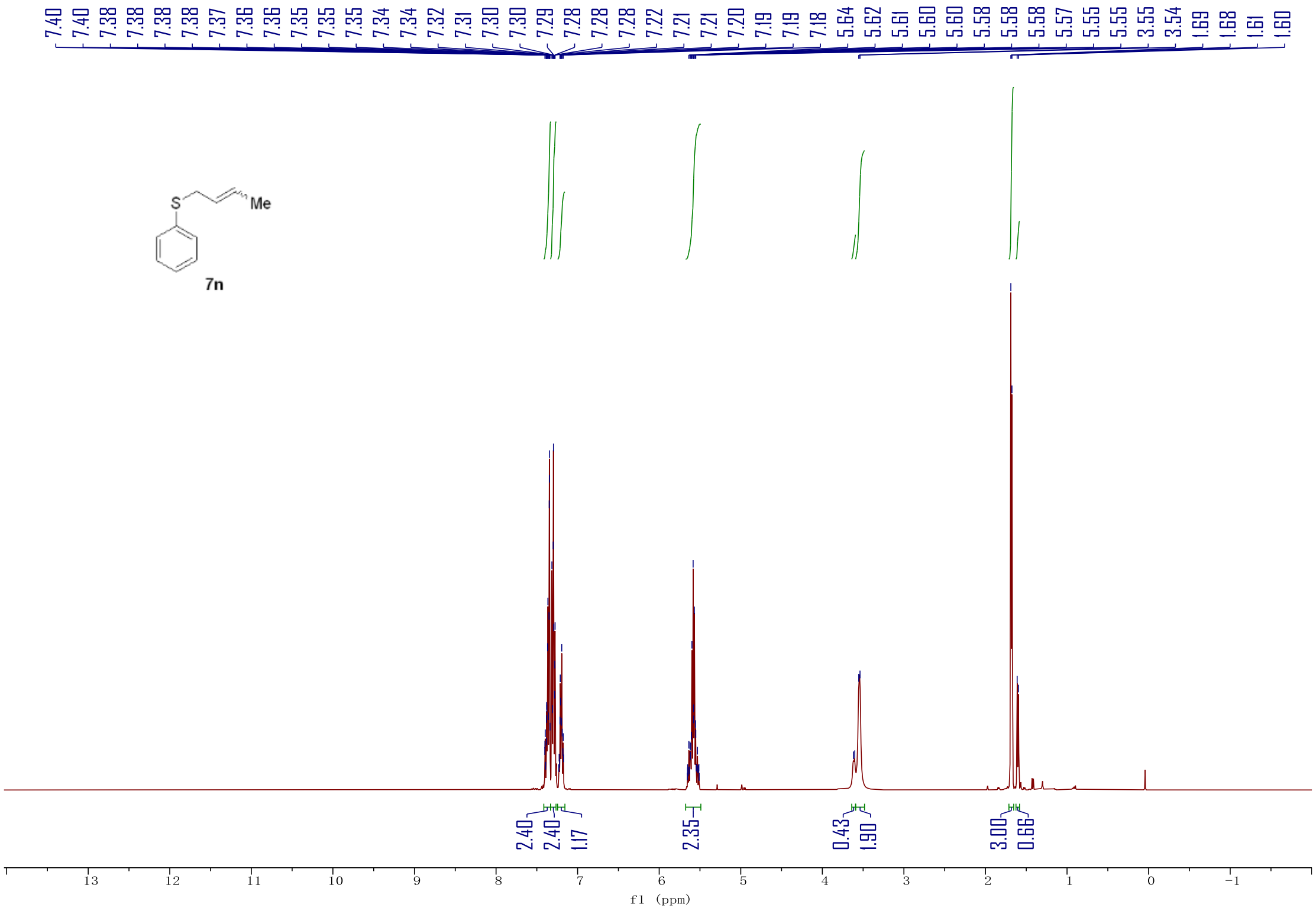

^{13}C NMR Spectrum of **7n** (101 MHz, $CDCl_3$)

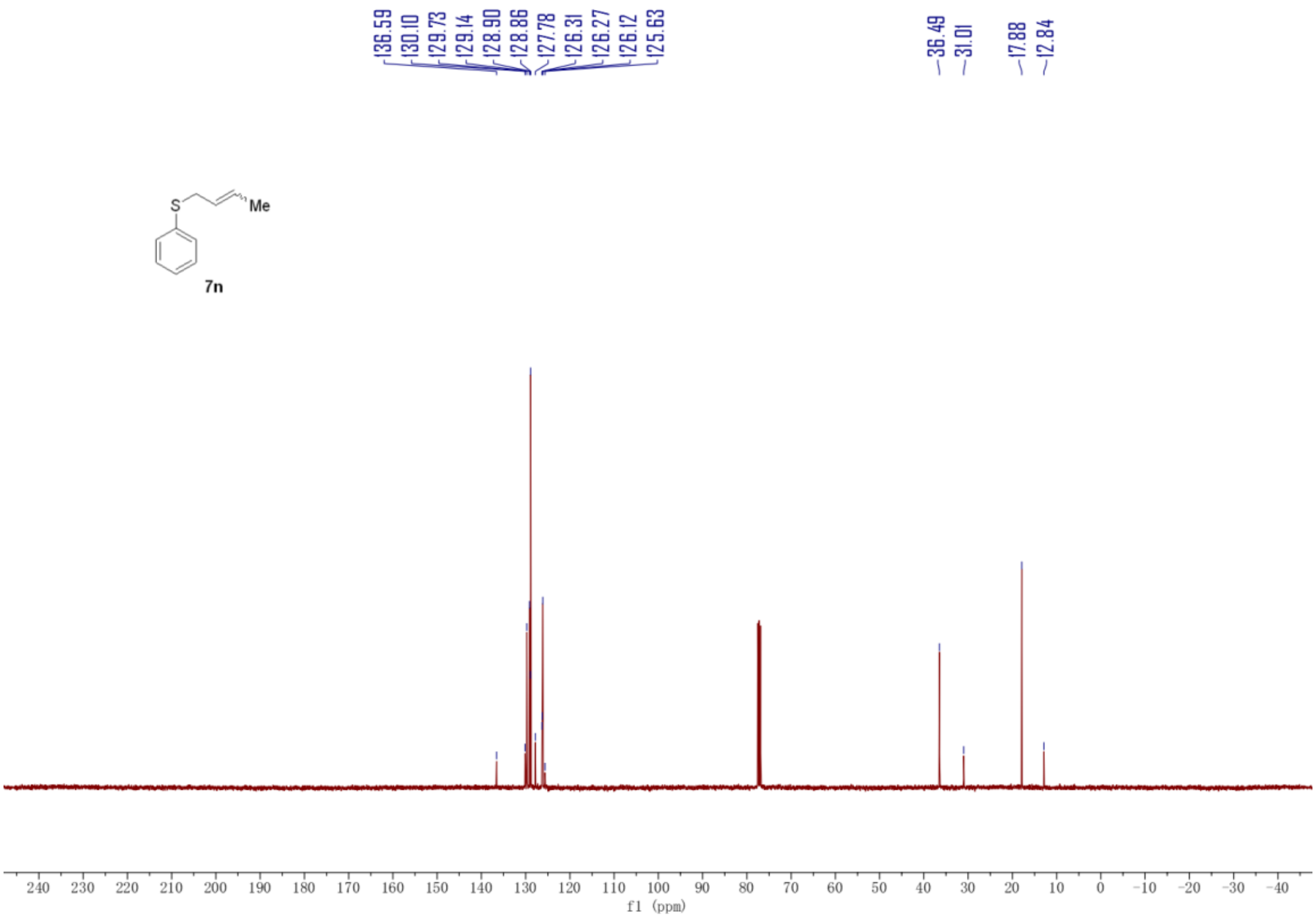

^{1}H NMR Spectrum of **7o** (400 MHz, $CDCl_3$)

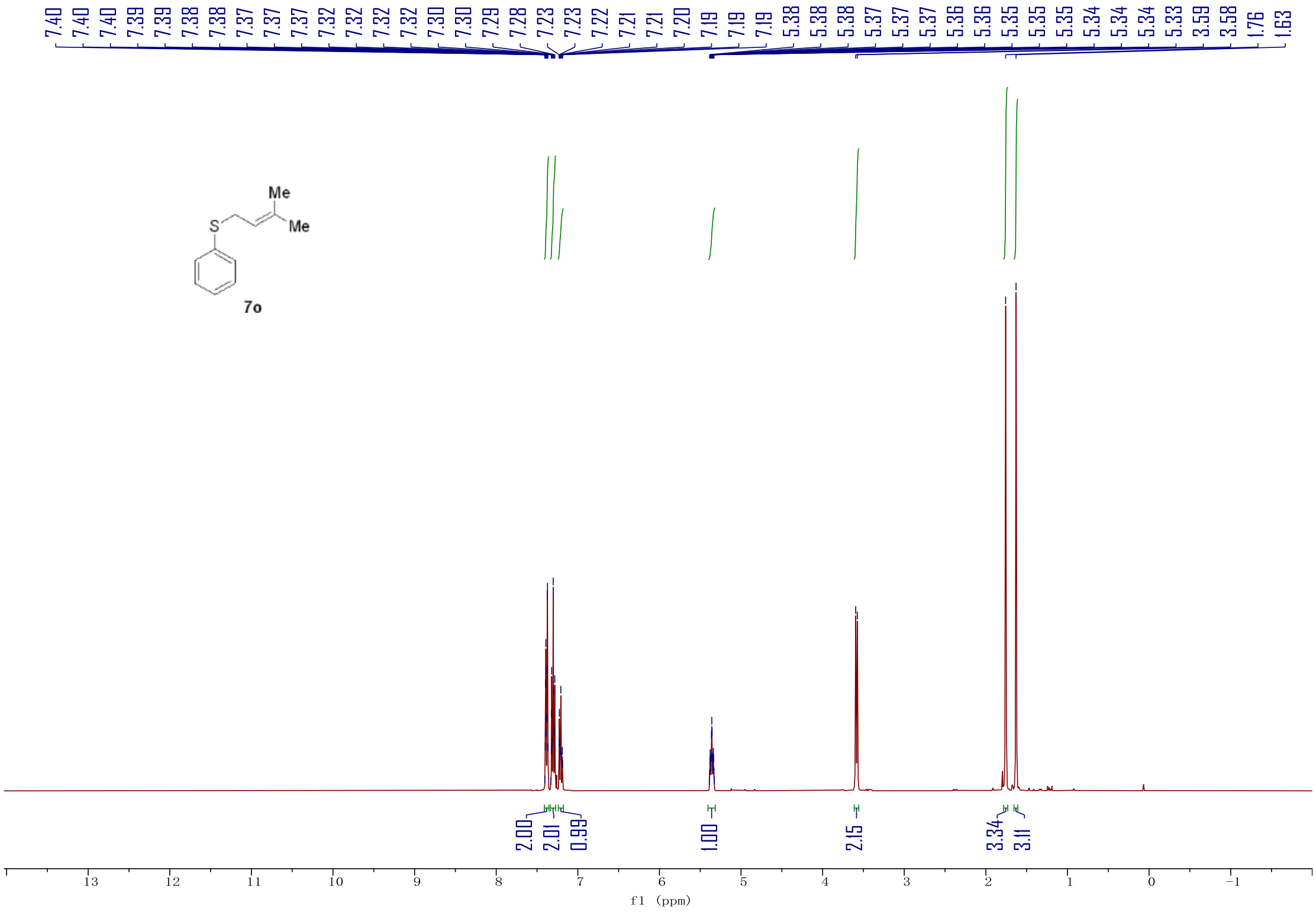

^{13}C NMR Spectrum of **7o** (101 MHz, $CDCl_3$)

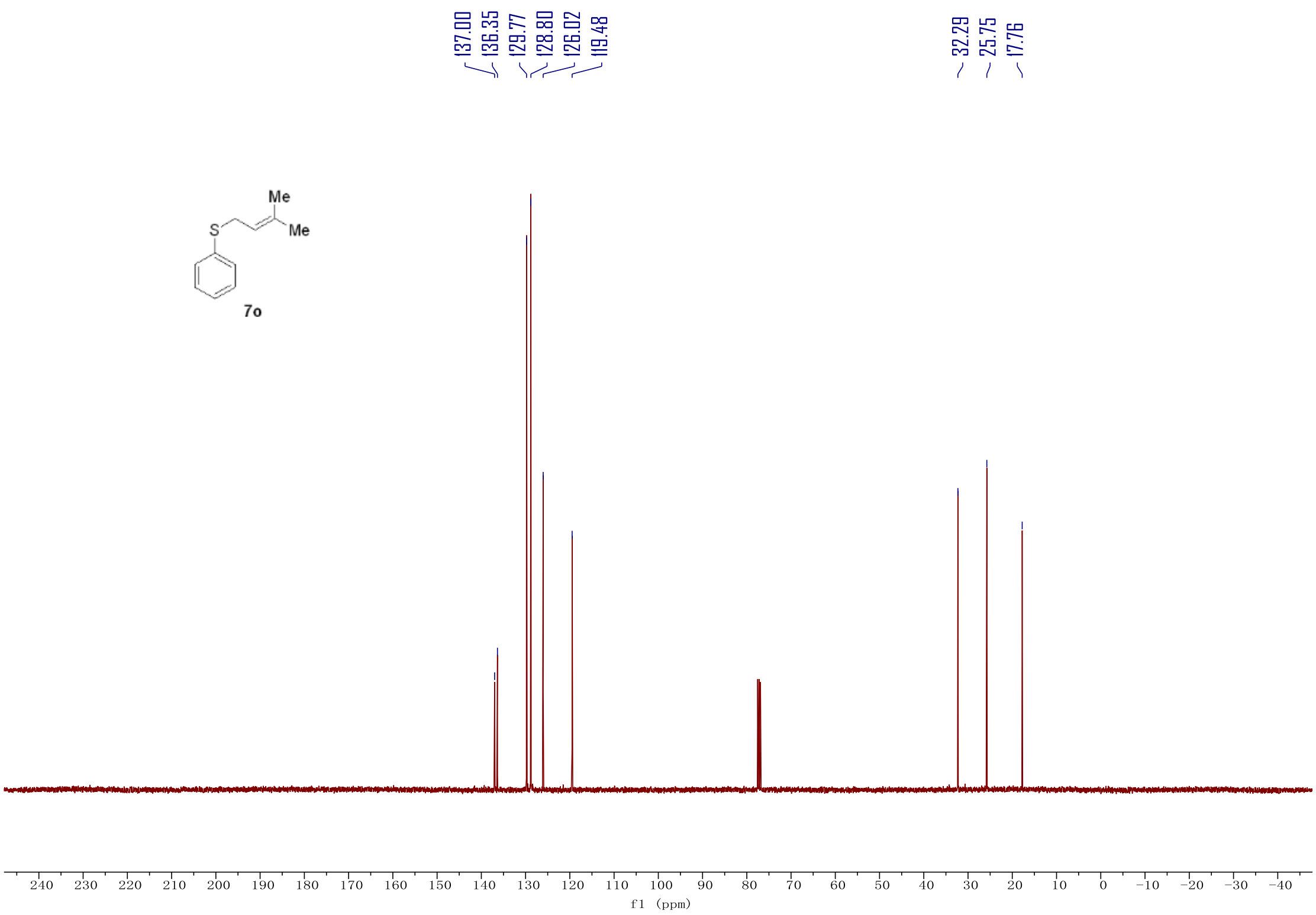

^{1}H NMR Spectrum of **7p** (400 MHz, $CDCl_3$)

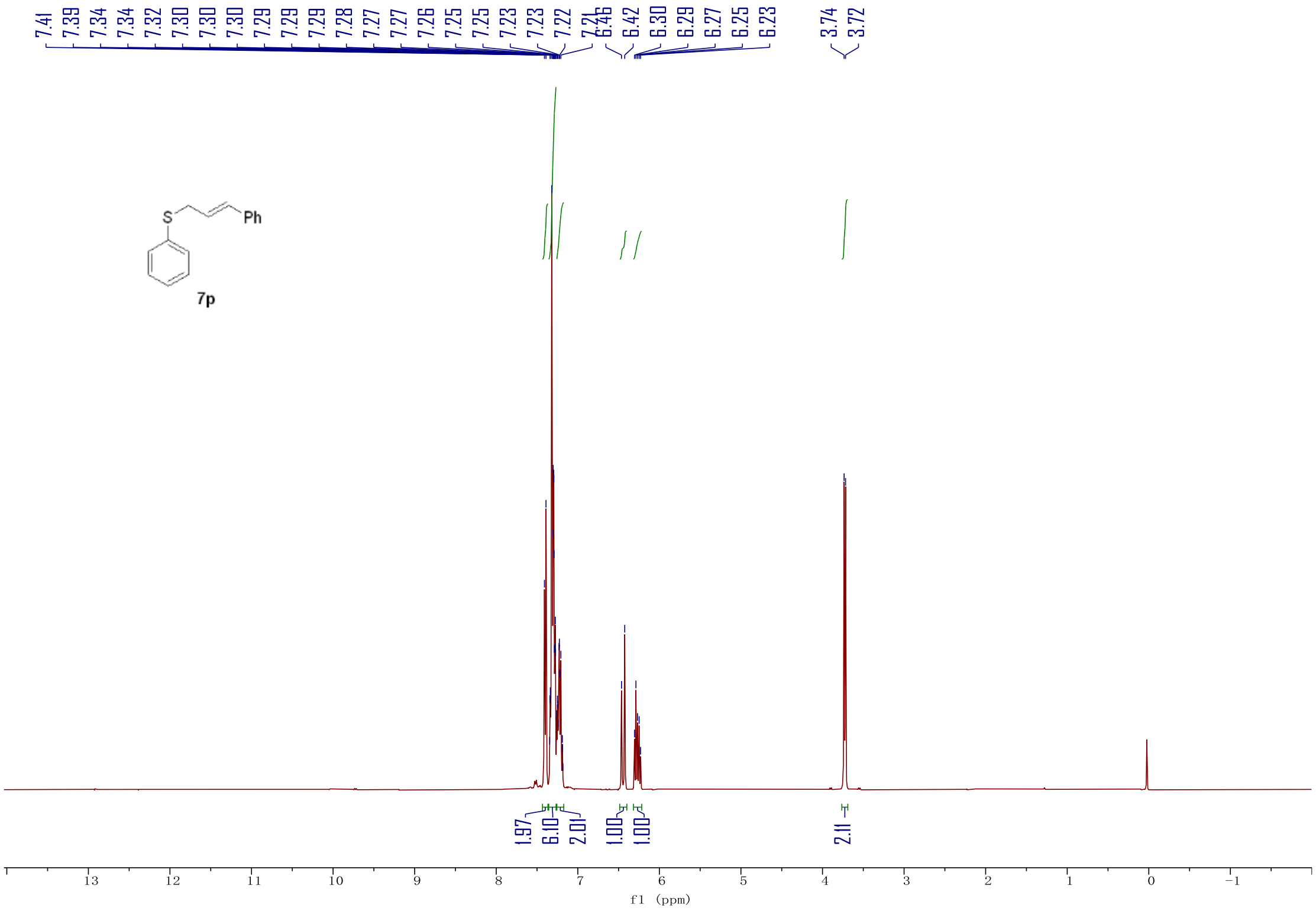

^{13}C NMR Spectrum of **7p** (101 MHz, $CDCl_3$)

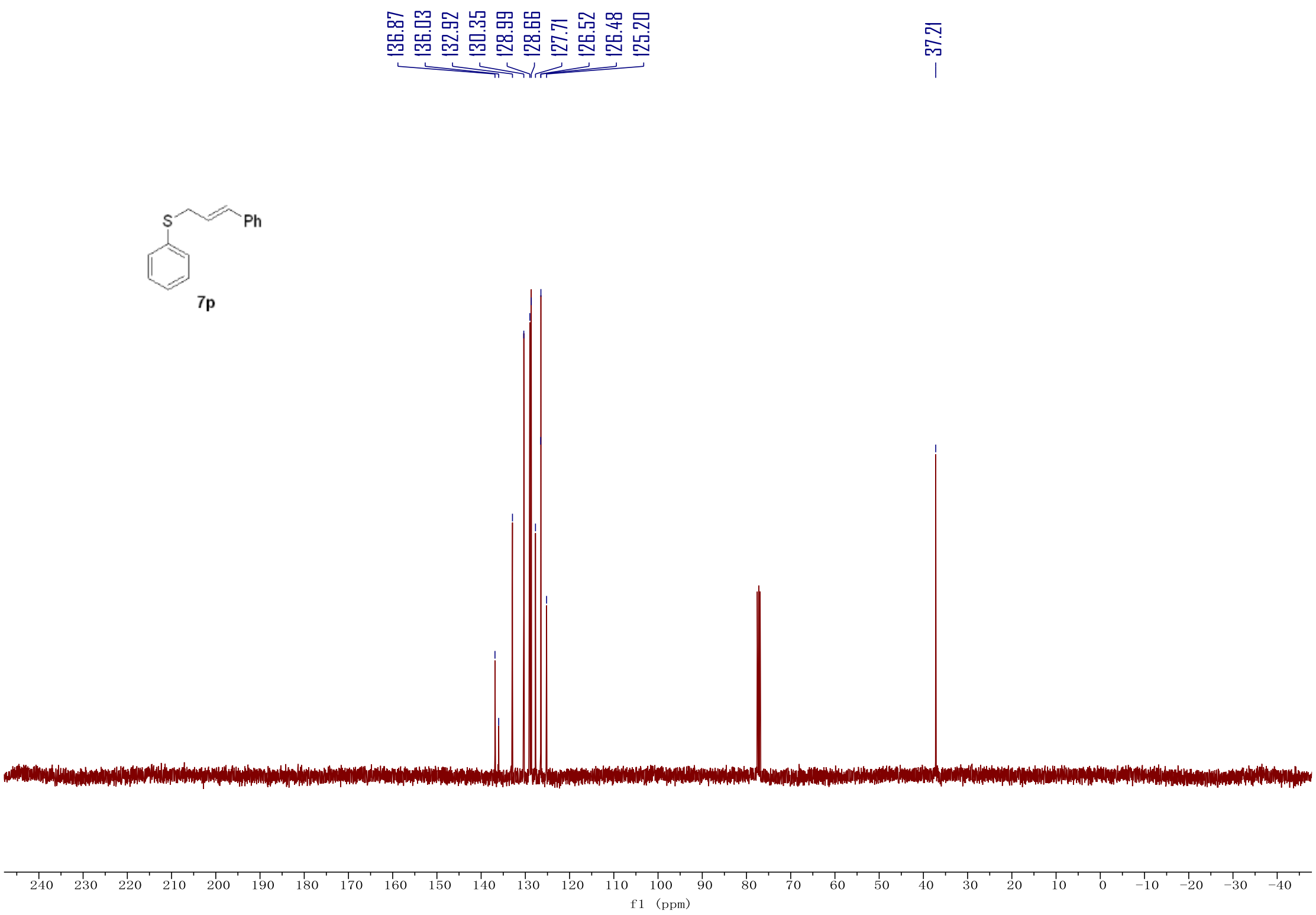

^{1}H NMR Spectrum of **7q** (400 MHz, CDCl3)

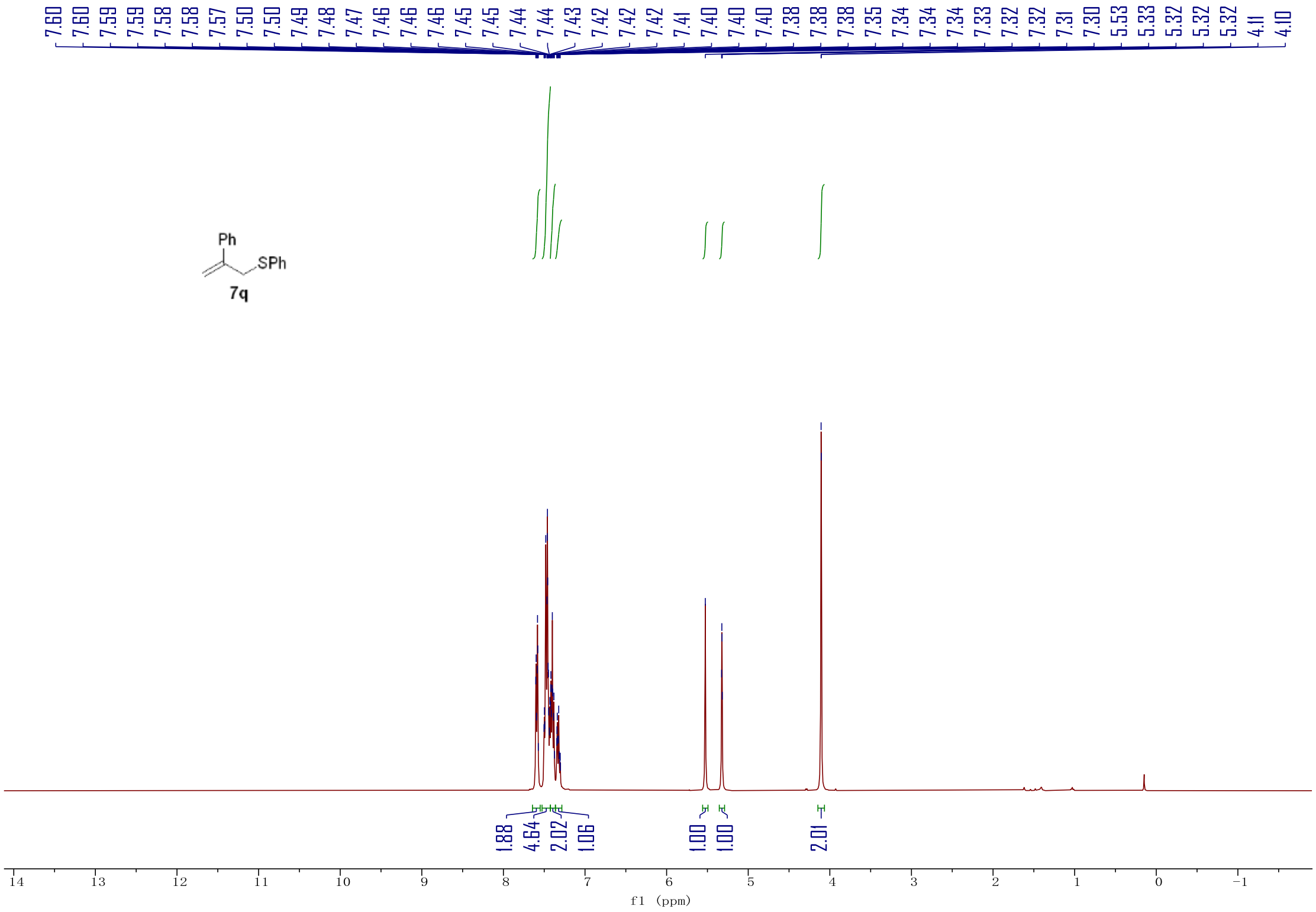

^{13}C NMR Spectrum of **7q** (101 MHz, CDCl3)

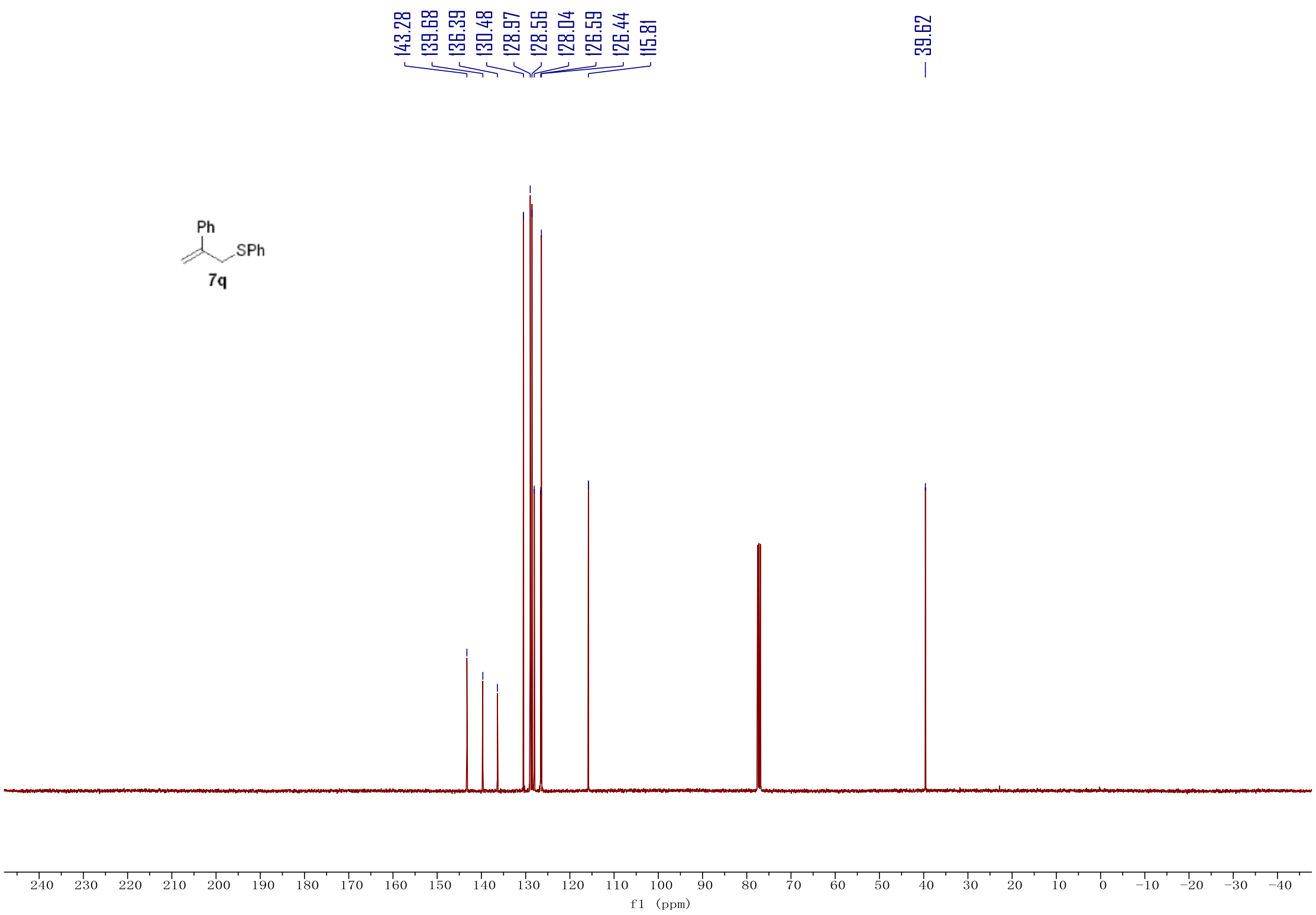

^{1}H NMR Spectrum of **7r** (400 MHz, $CDCl_3$)

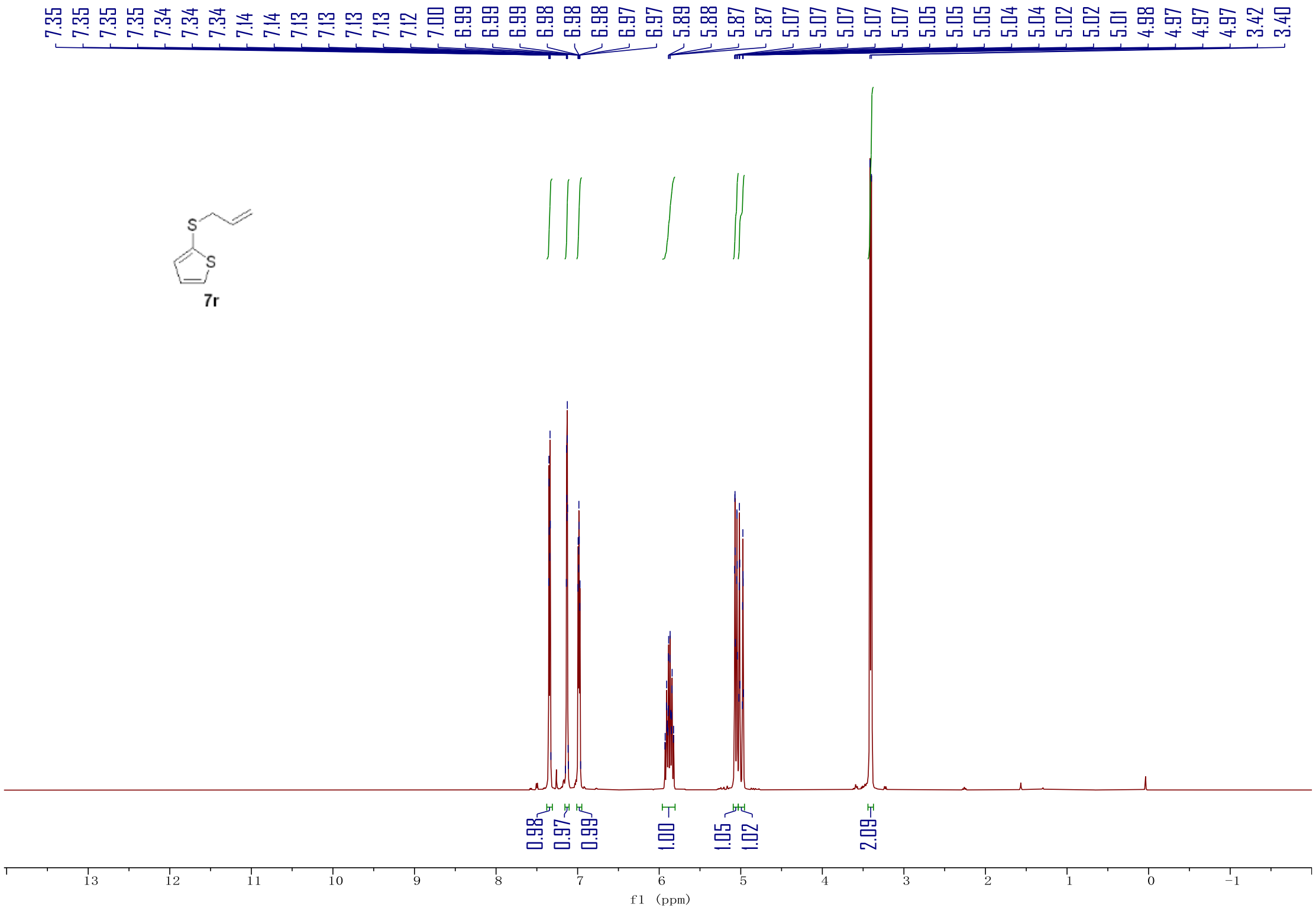

^{13}C NMR Spectrum of **7r** (101 MHz, $CDCl_3$)

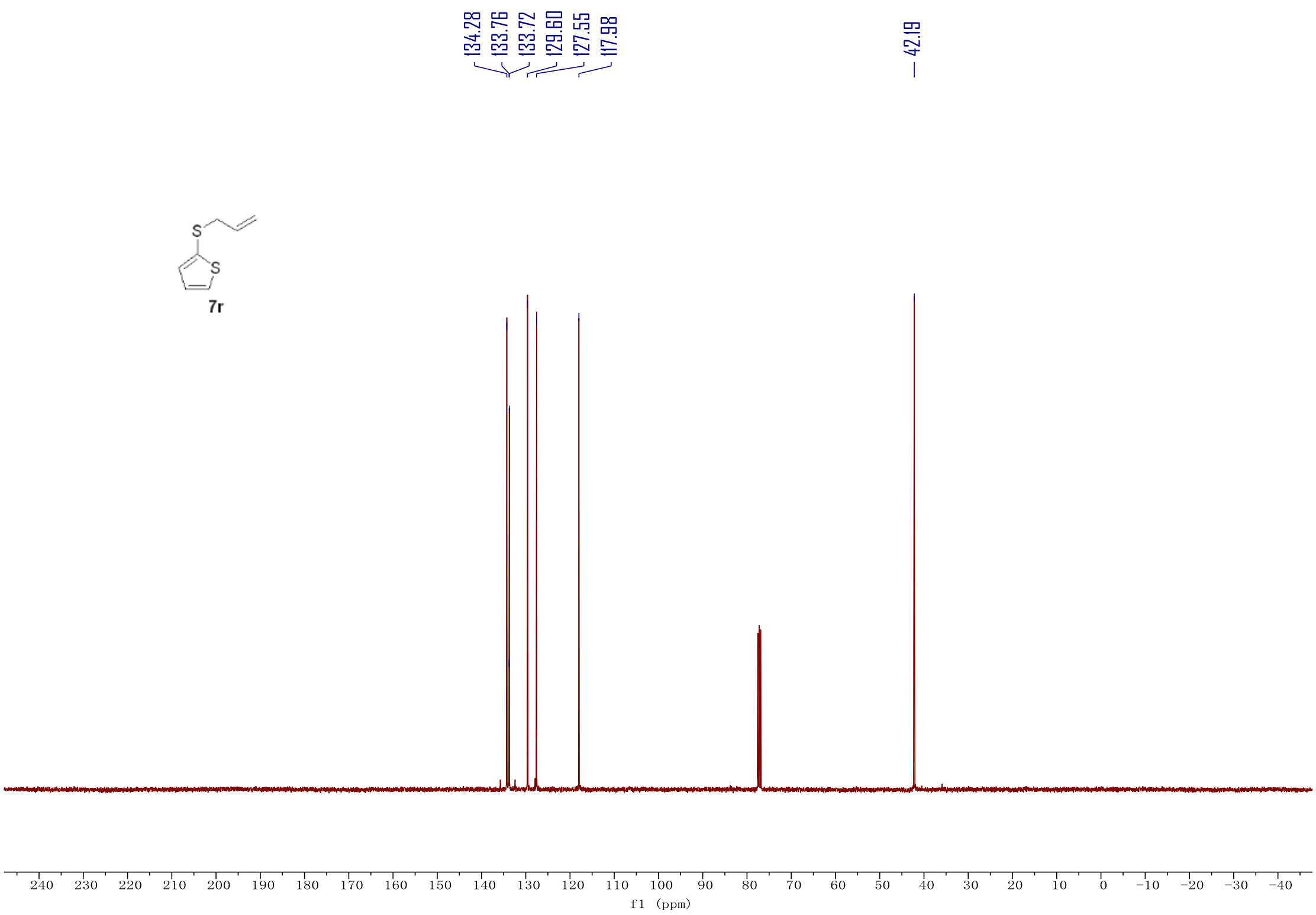

^{1}H NMR Spectrum of **7s** (400 MHz, $CDCl_3$)

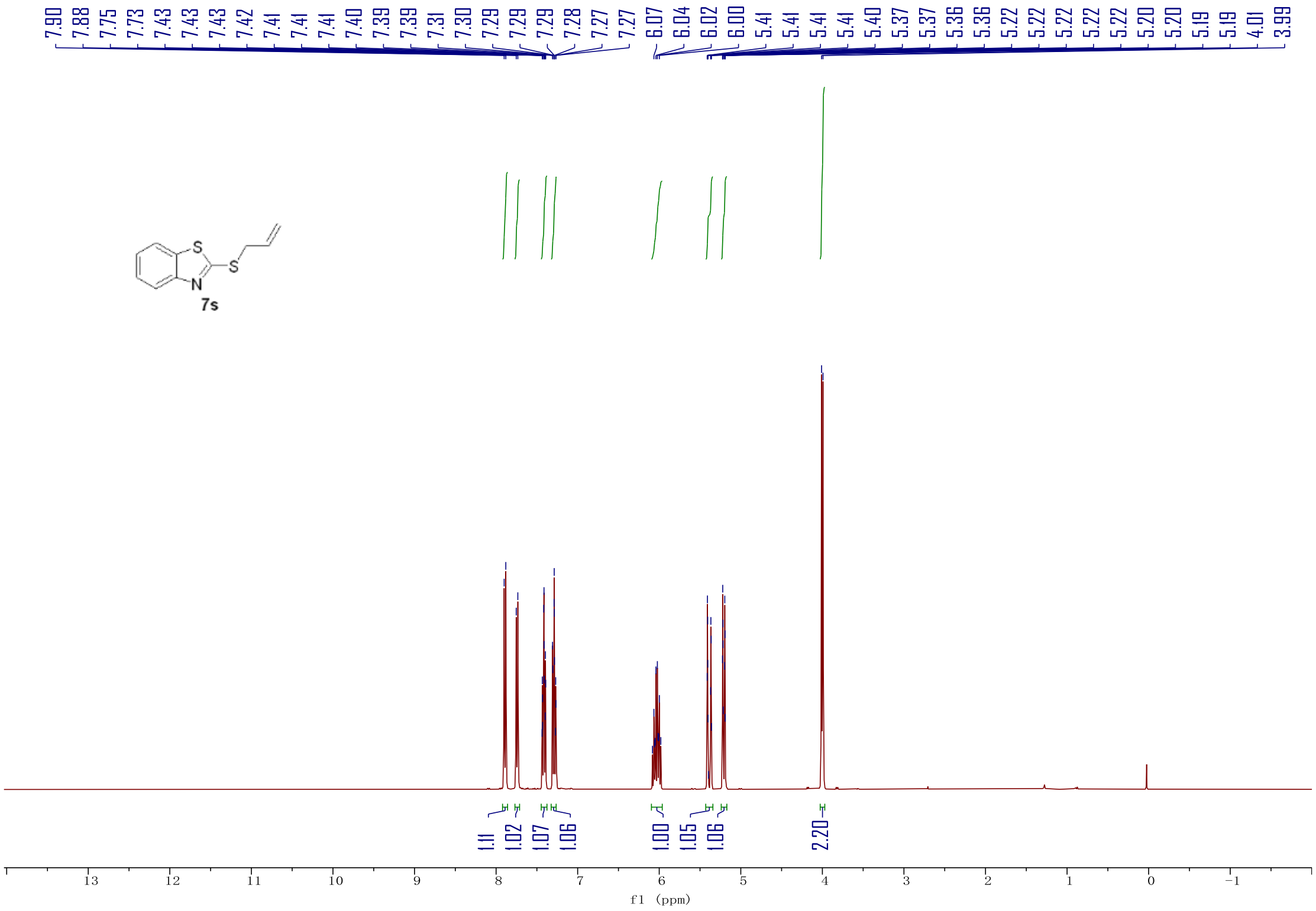

^{13}C NMR Spectrum of **7s** (101 MHz, $CDCl_3$)

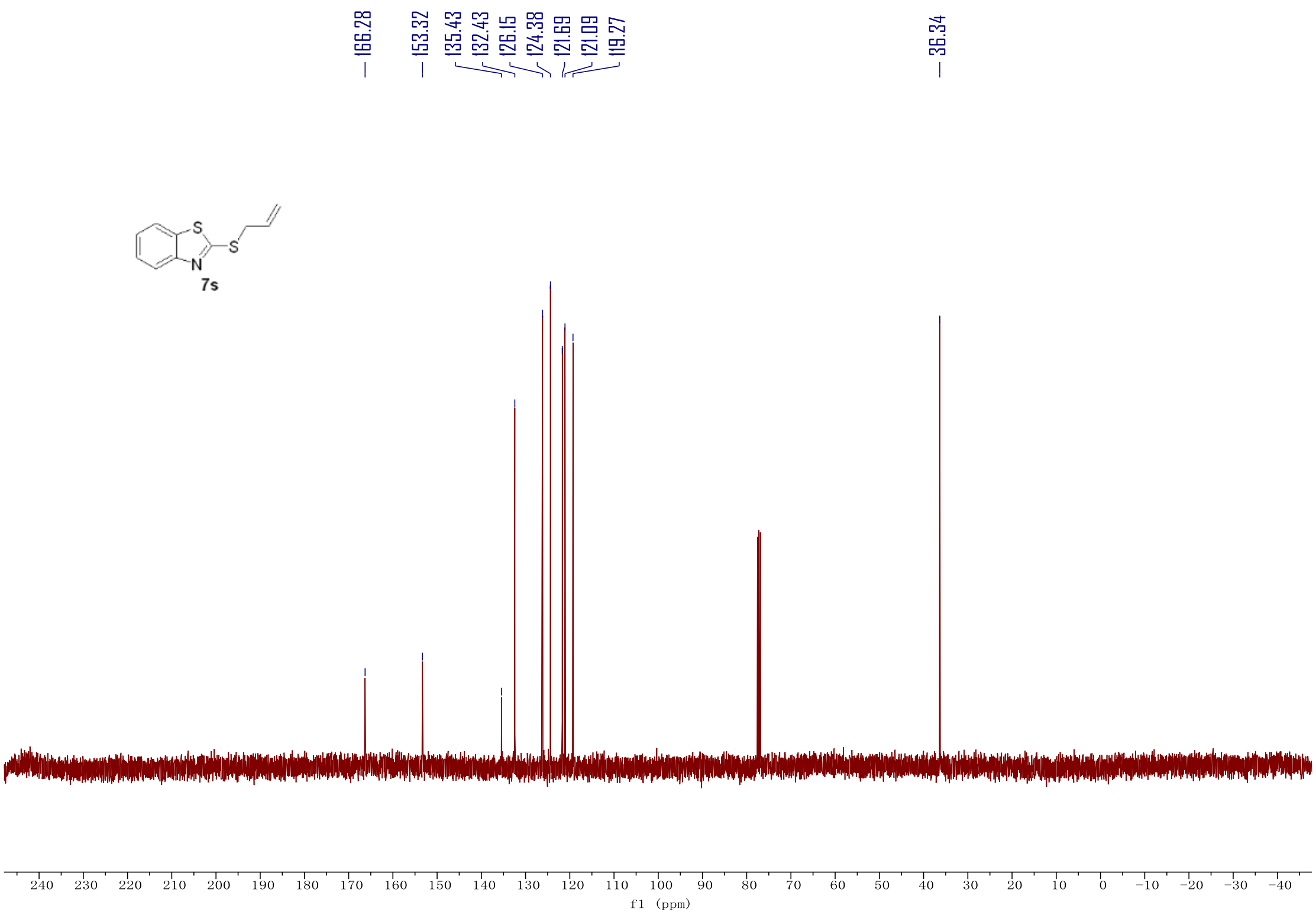

^{1}H NMR Spectrum of **7t** (400 MHz, $CDCl_3$)

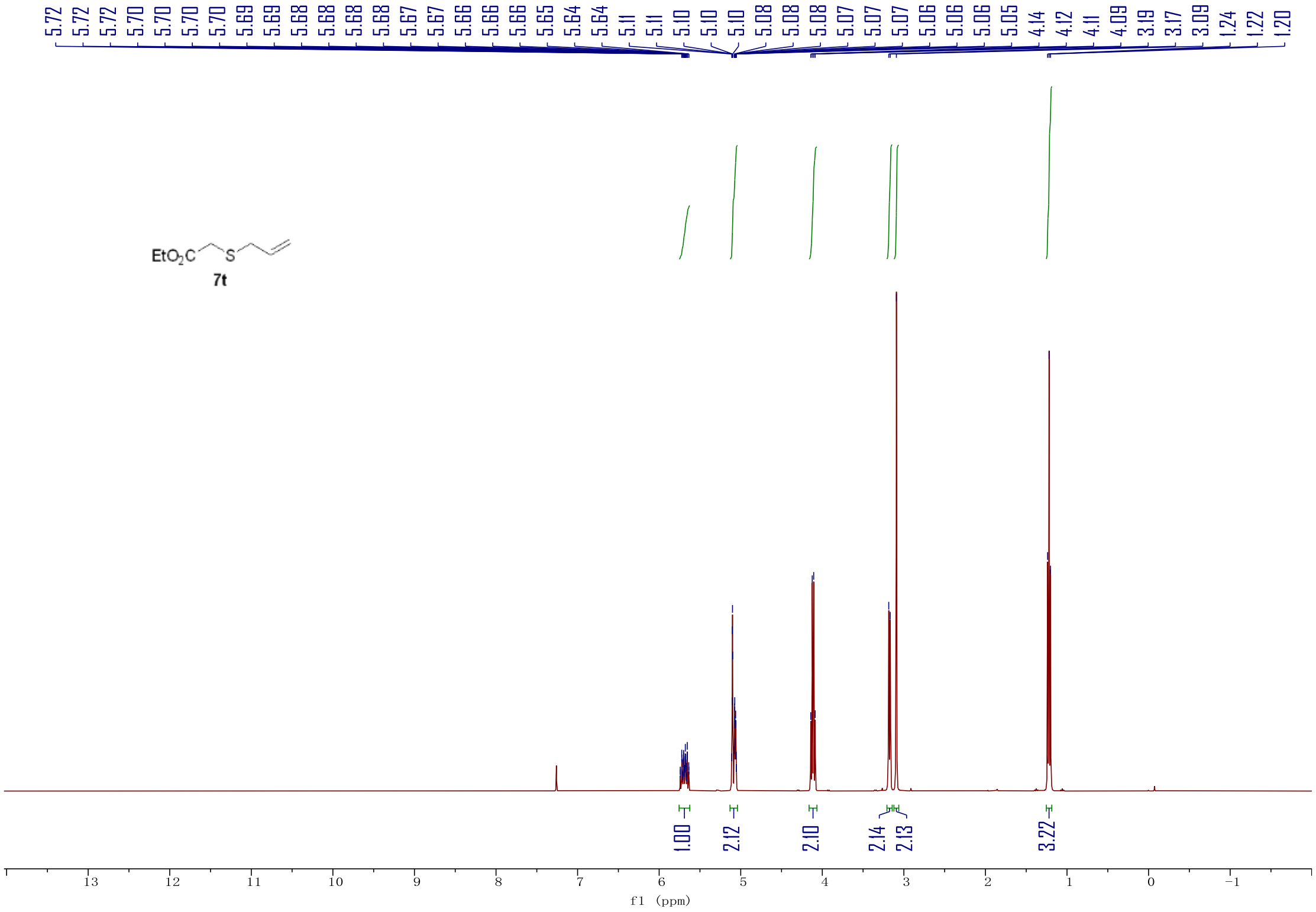

^{13}C NMR Spectrum of **7t** (101 MHz, $CDCl_3$)

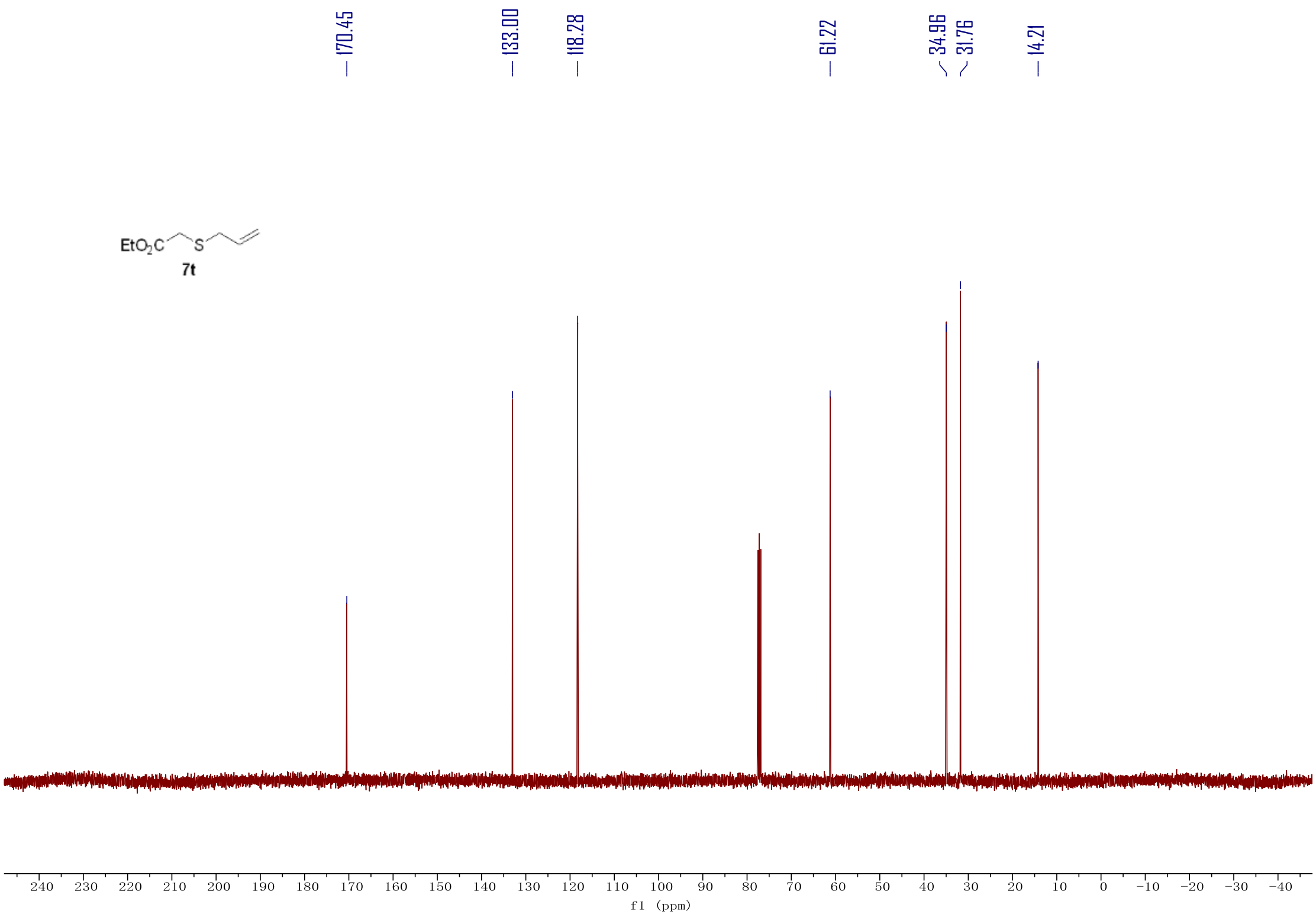

^{1}H **NMR** Spectrum of **7u** (400 MHz, $CDCl_3$)

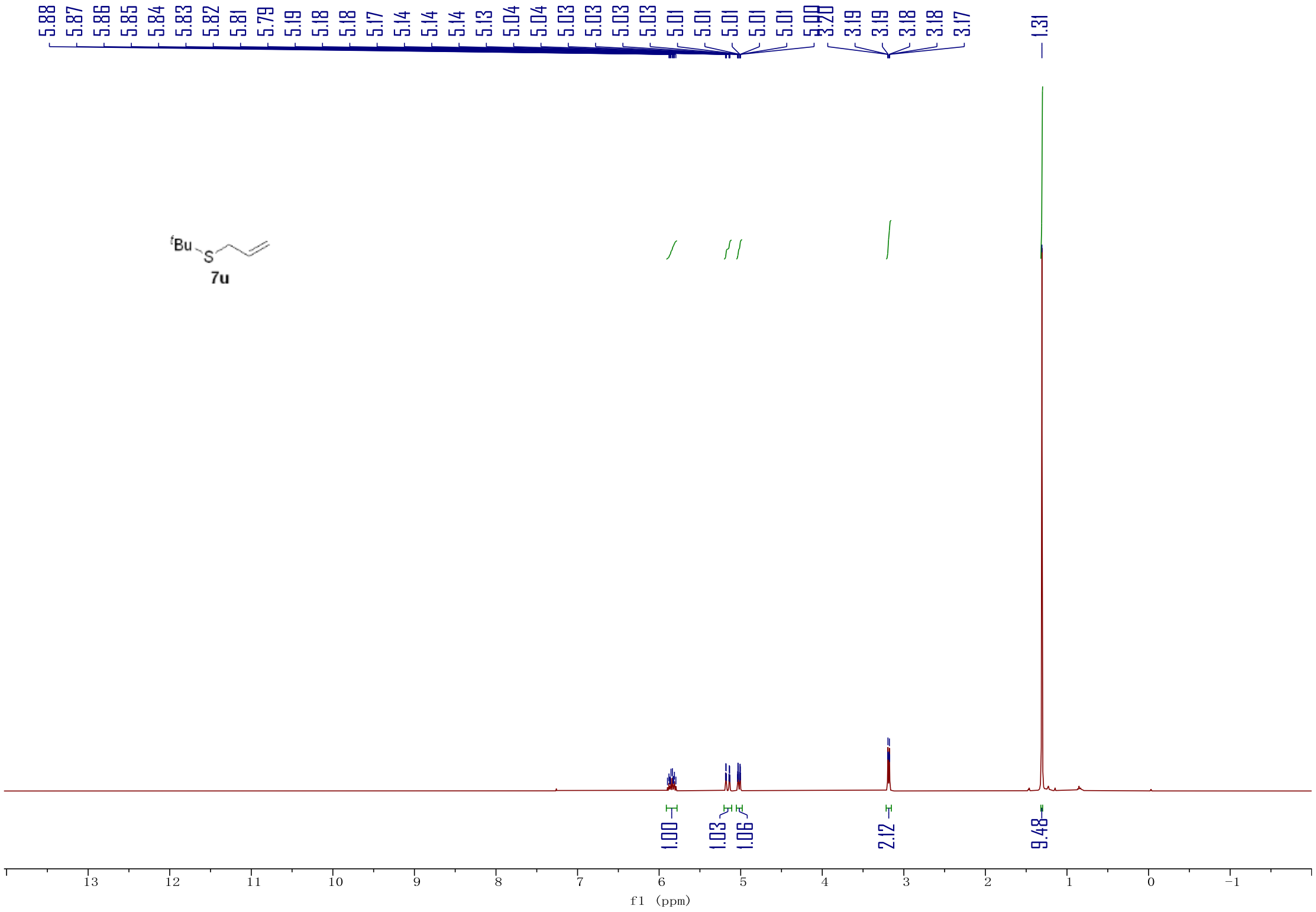

^{13}C **NMR** Spectrum of **7u** (101 MHz, $CDCl_3$)

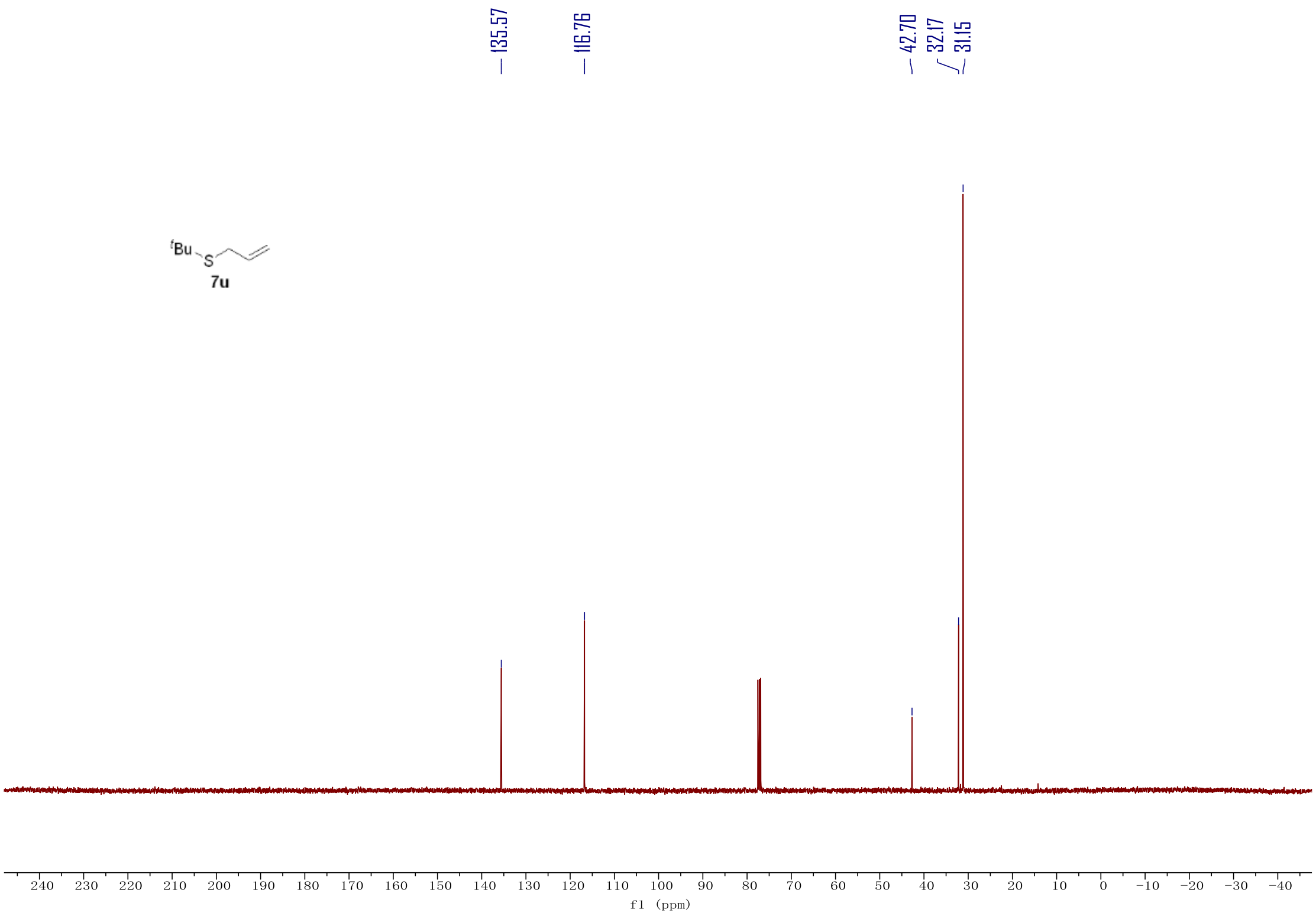

^{1}H NMR Spectrum of **9a** (400 MHz, $CDCl_3$)

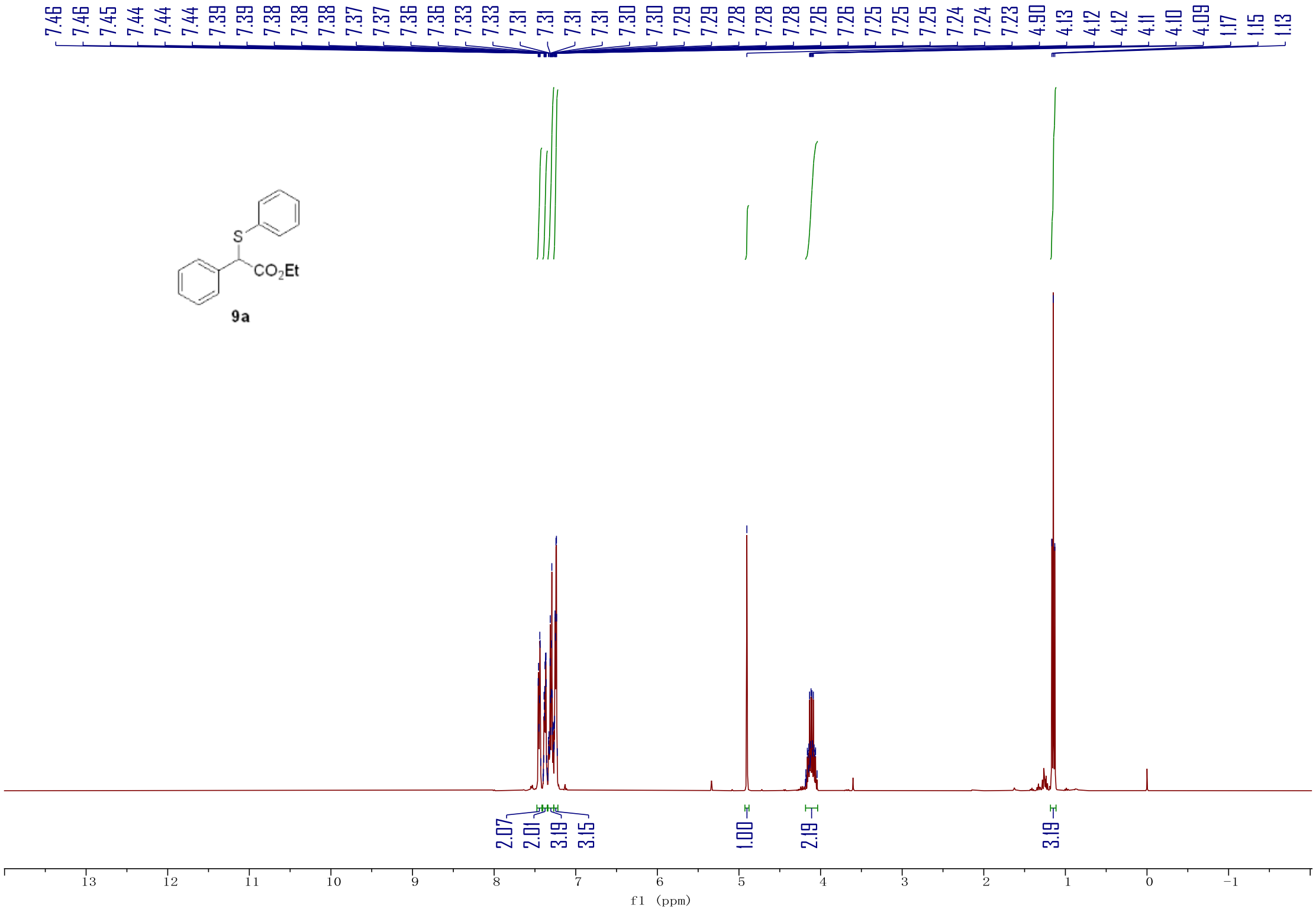

^{13}C NMR Spectrum of **9a** (101 MHz, $CDCl_3$)

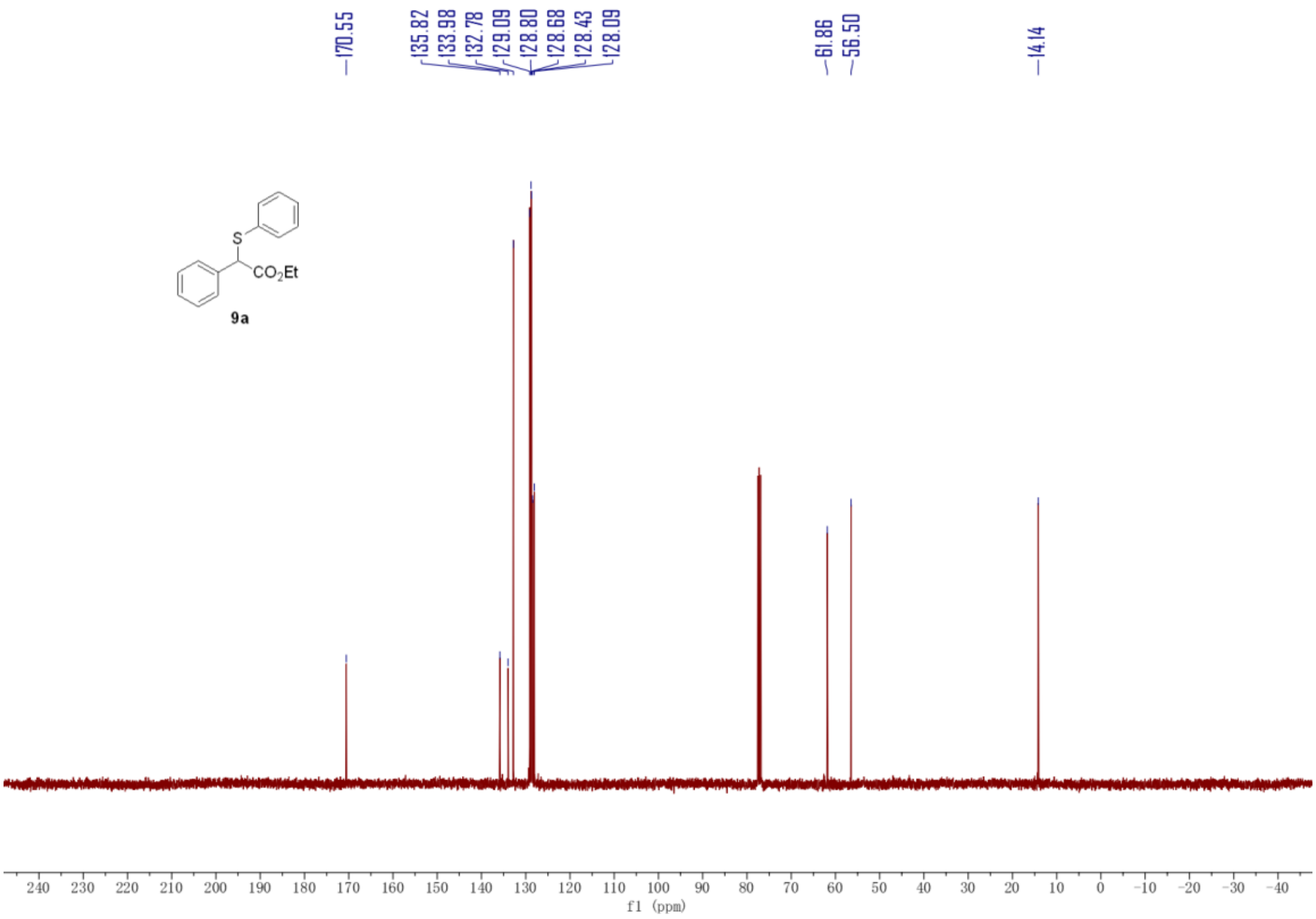

^{1}H NMR Spectrum of 9b (400 MHz, $CDCl_3$)

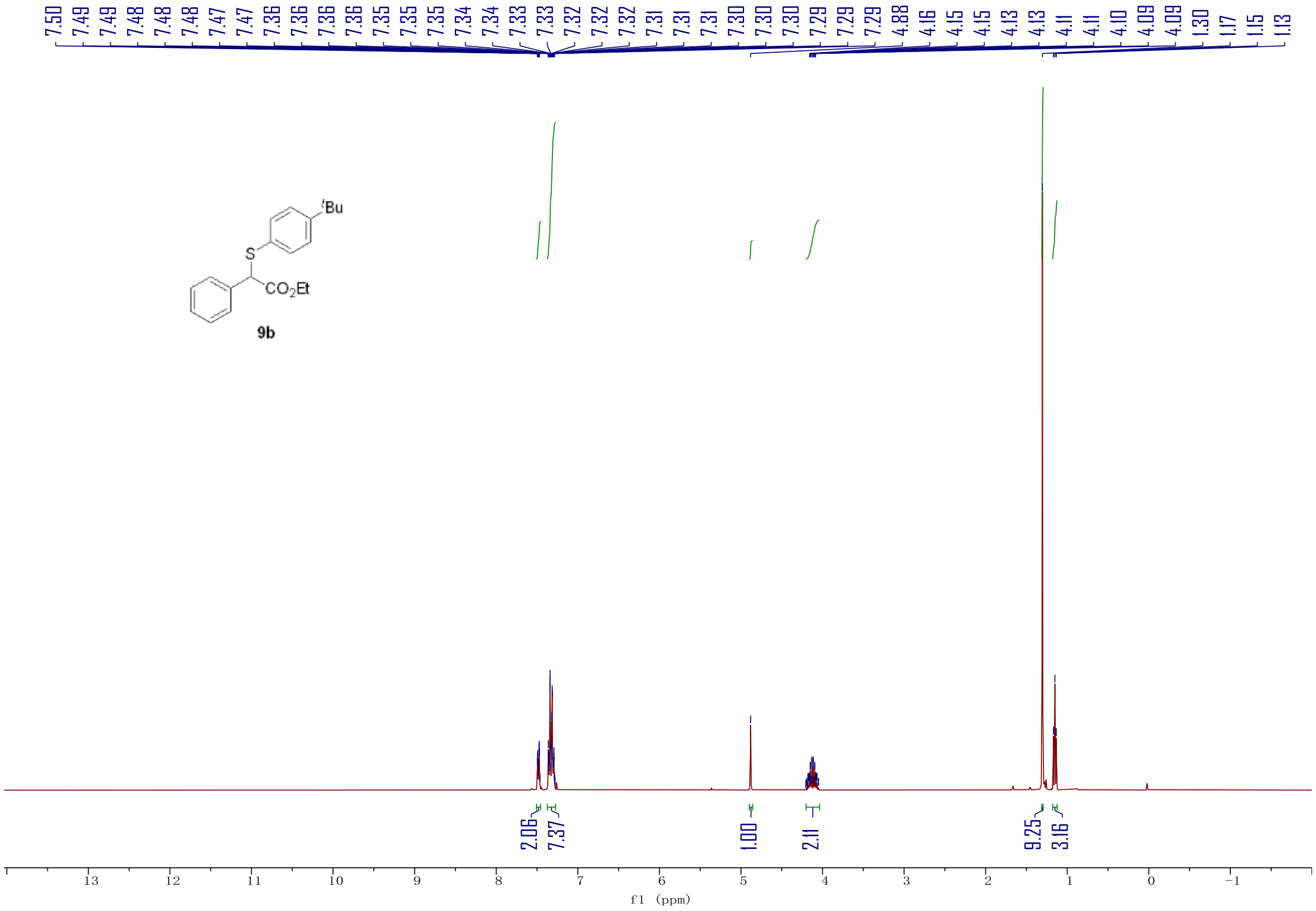

^{13}C NMR Spectrum of 9b (101 MHz, $CDCl_3$)

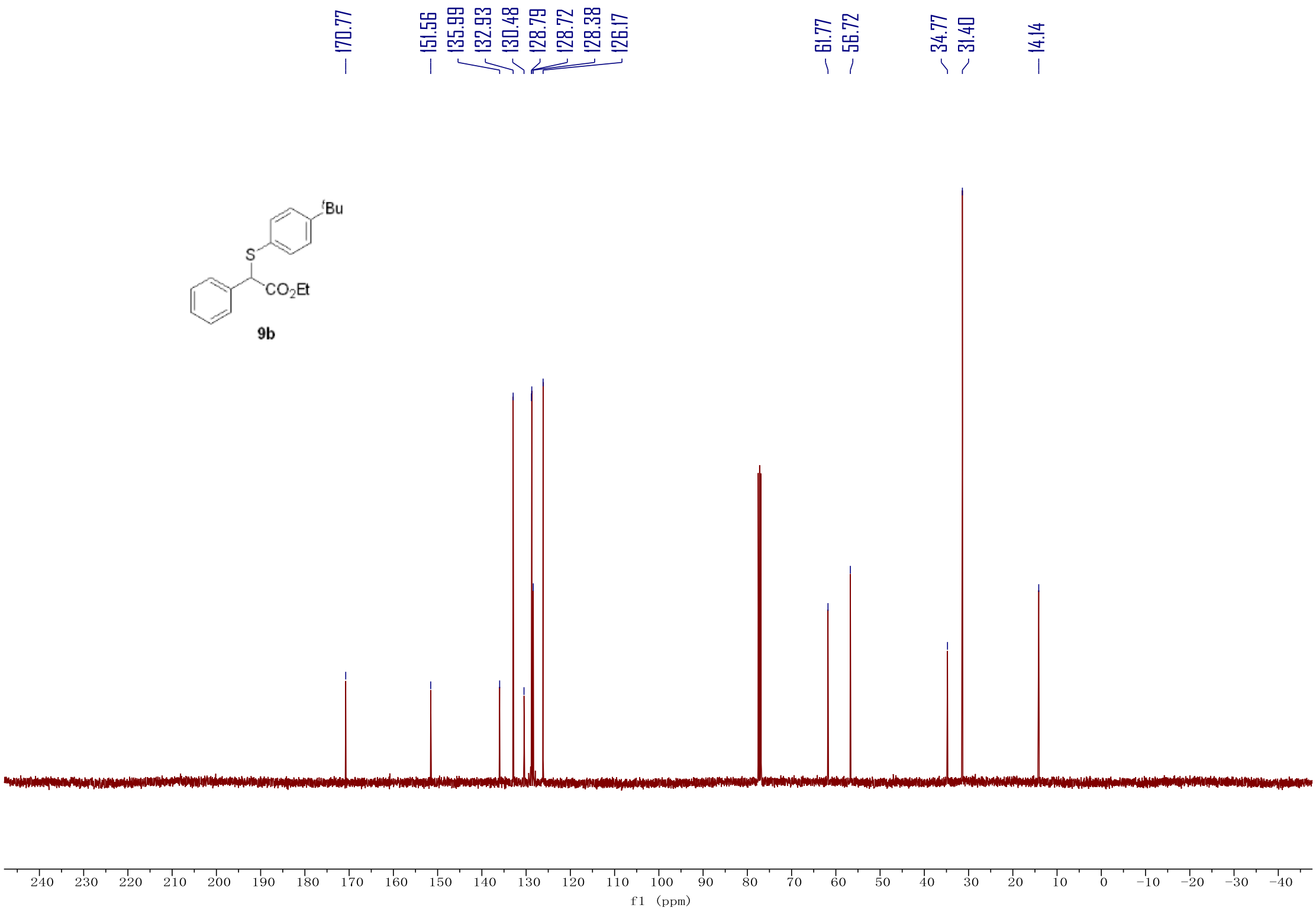

^{1}H **NMR** Spectrum of **9c** (400 MHz, $CDCl_3$)

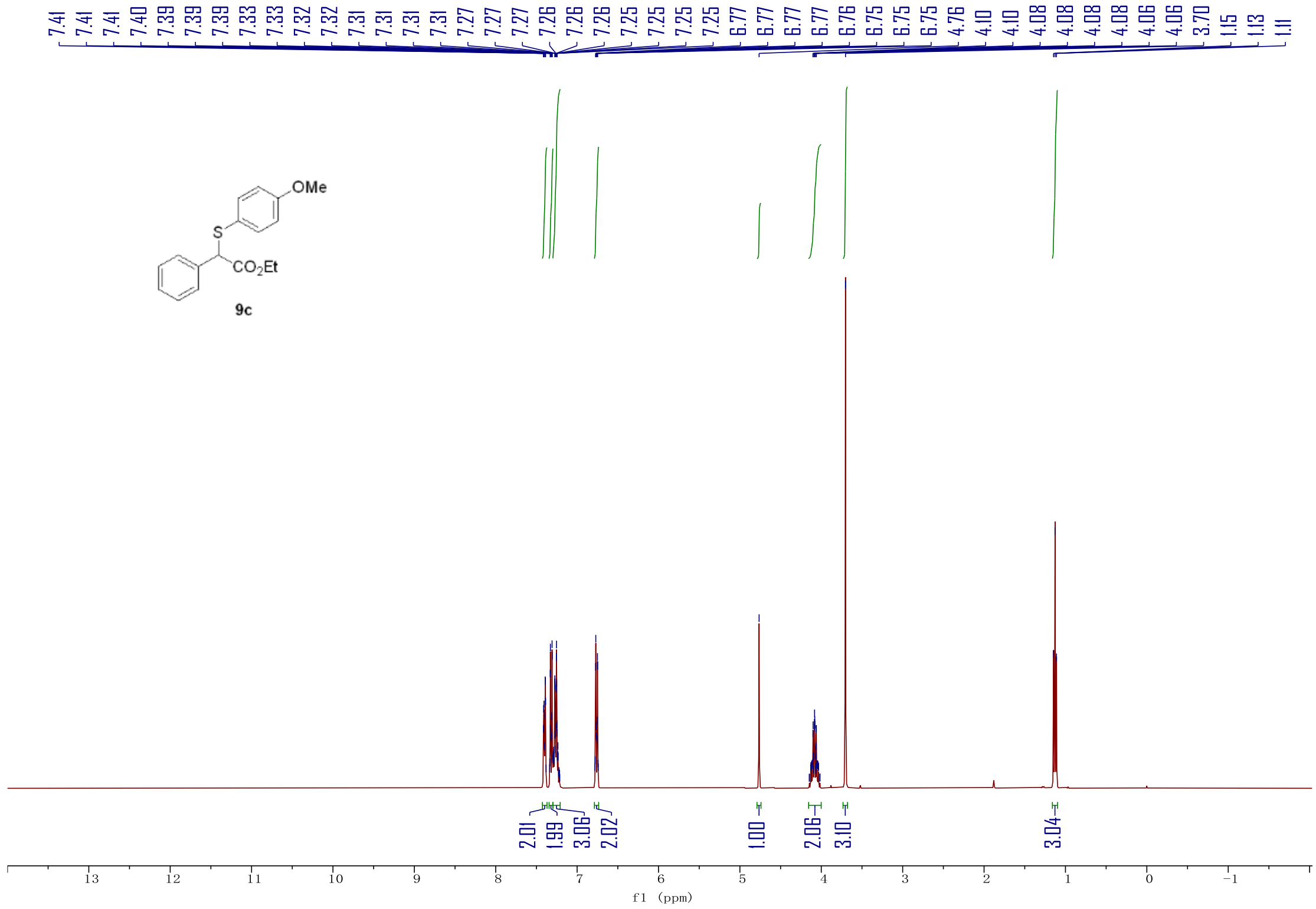

^{13}C **NMR** Spectrum of **9c** (101 MHz, $CDCl_3$)

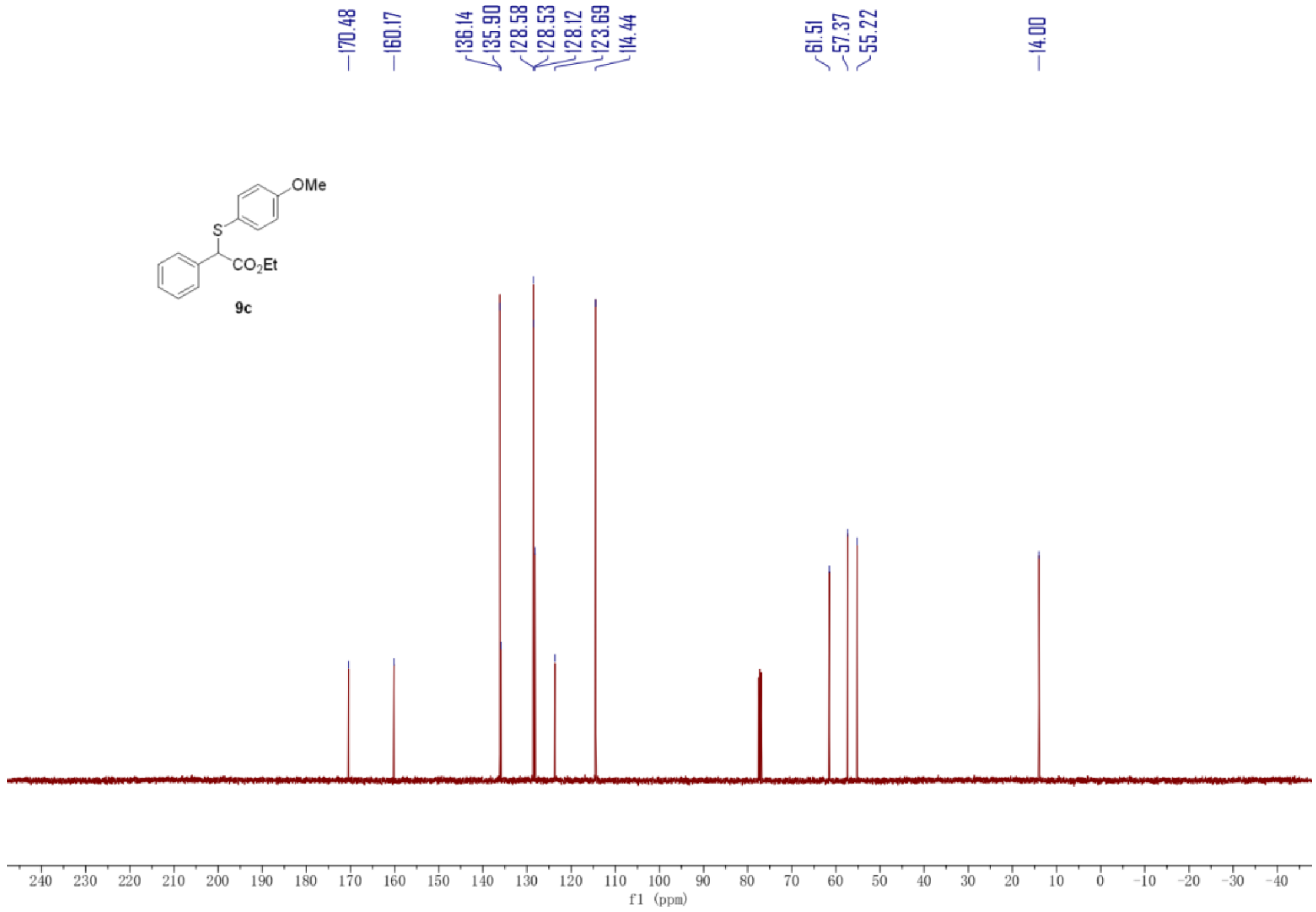

^{1}**H NMR** Spectrum of **9d** (400 MHz, $CDCl_3$)

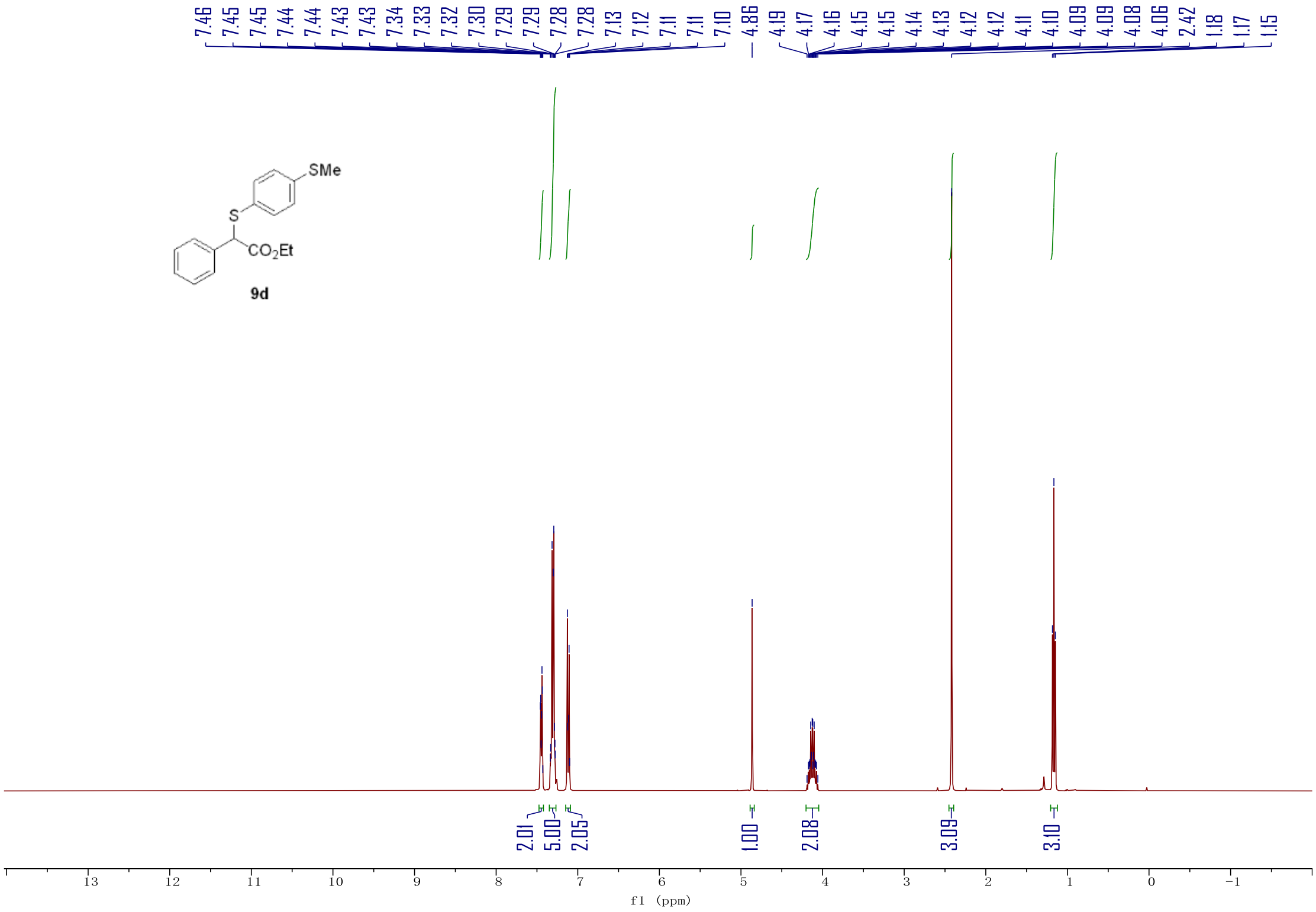

^{13}**C NMR** Spectrum of **9d** (101 MHz, $CDCl_3$)

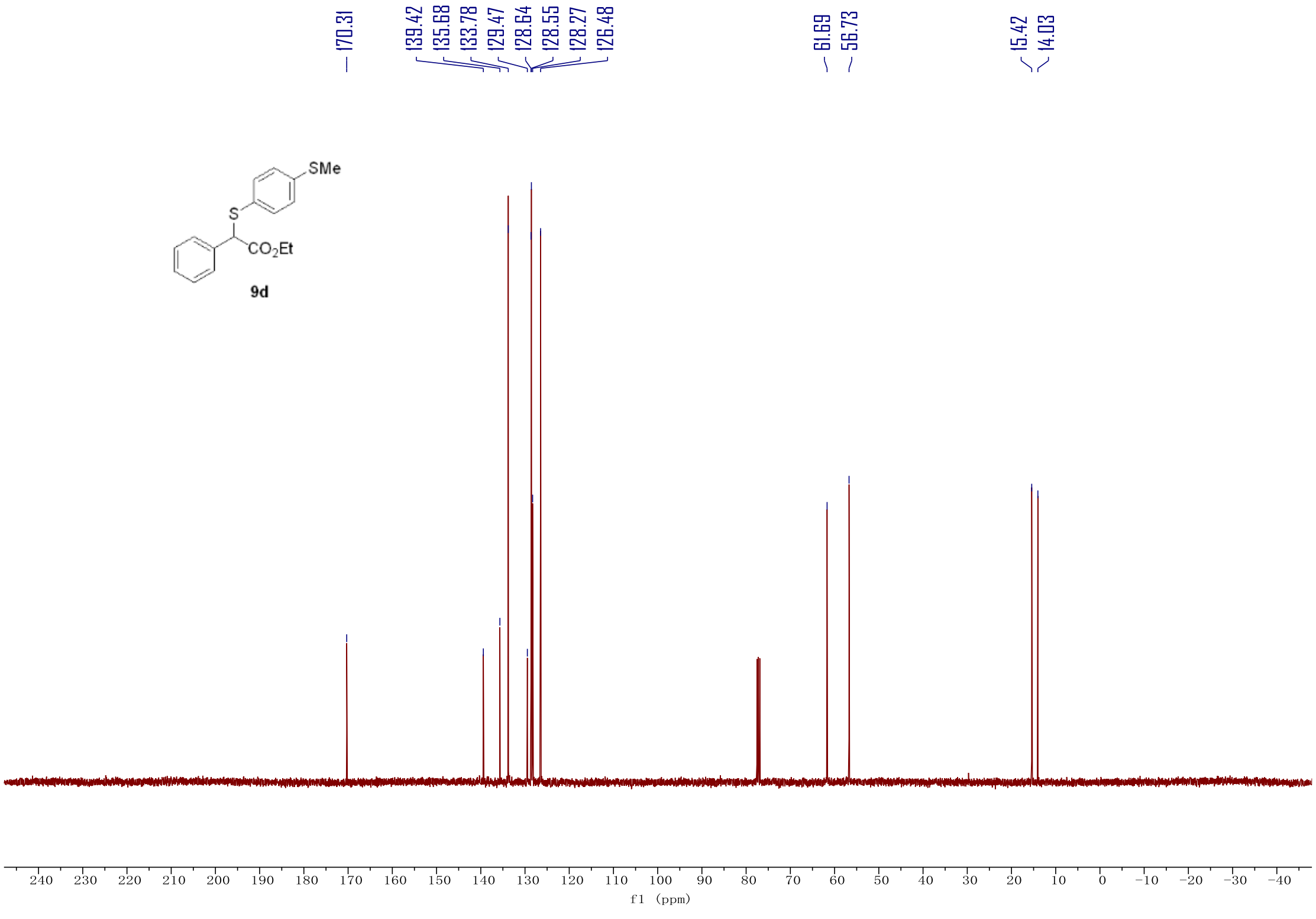

^{1}H NMR Spectrum of **9e** (400 MHz, $CDCl_3$)

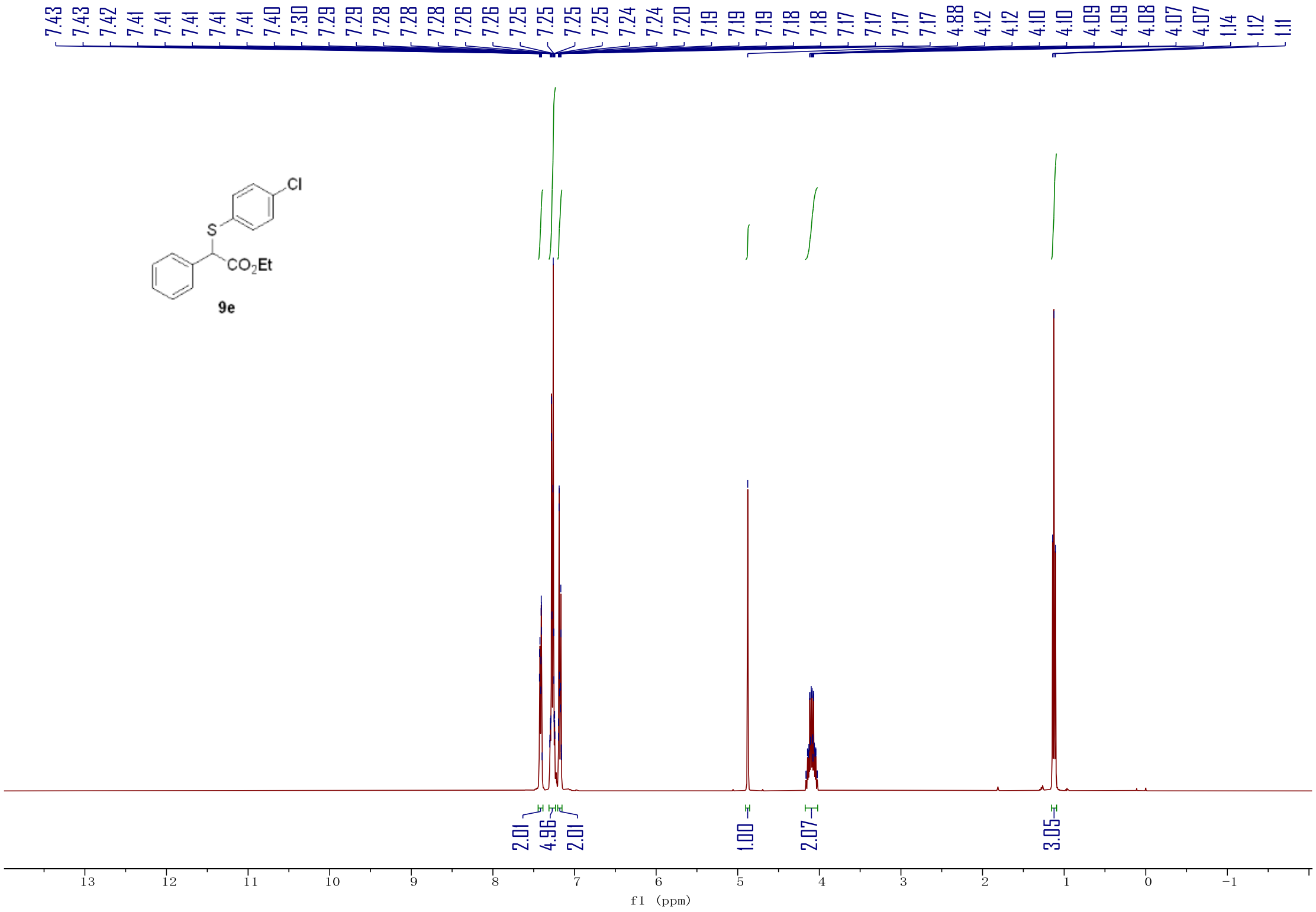

^{13}C NMR Spectrum of **9e** (101 MHz, $CDCl_3$)

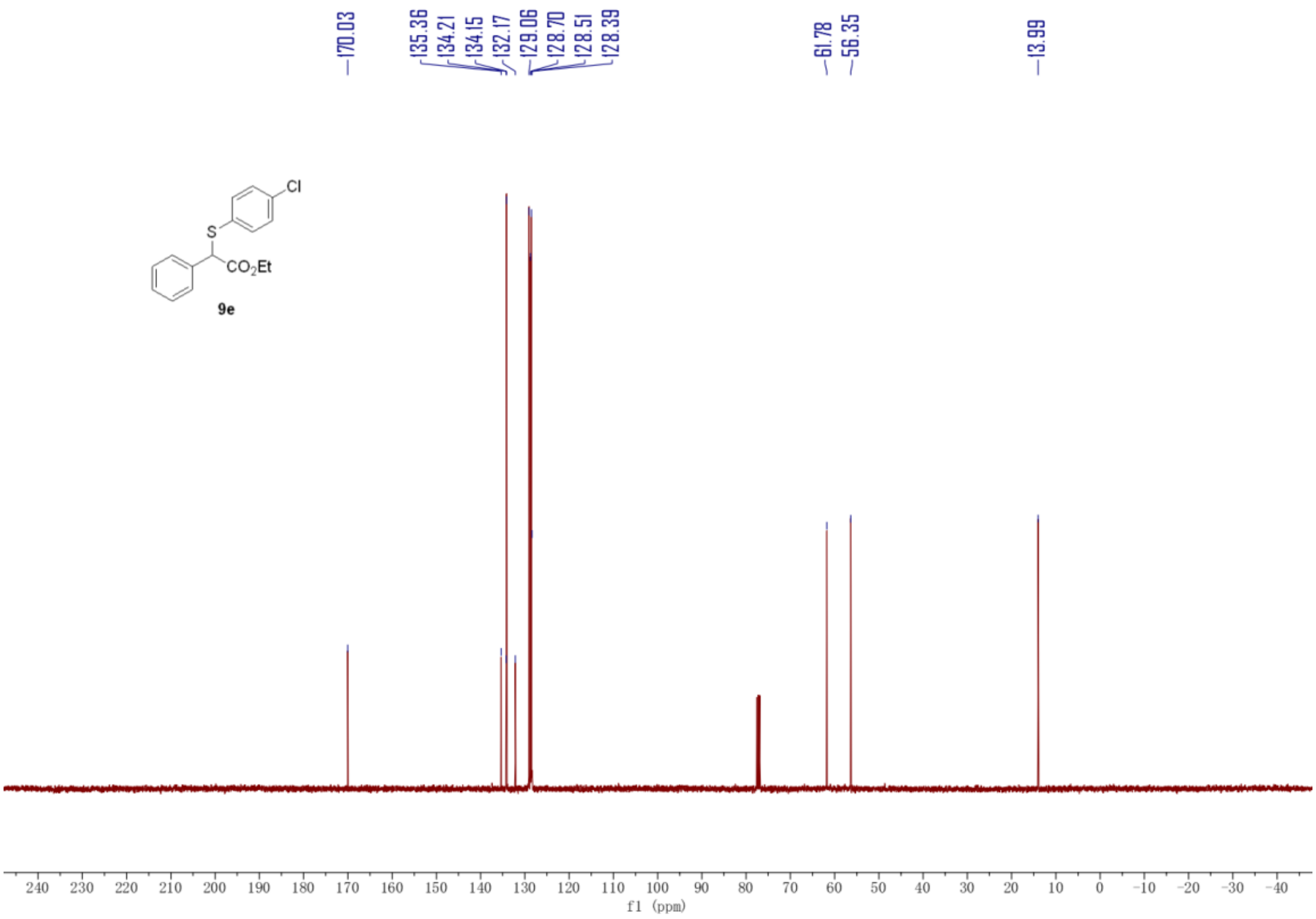

^{1}H NMR Spectrum of **9f** (400 MHz, $CDCl_3$)

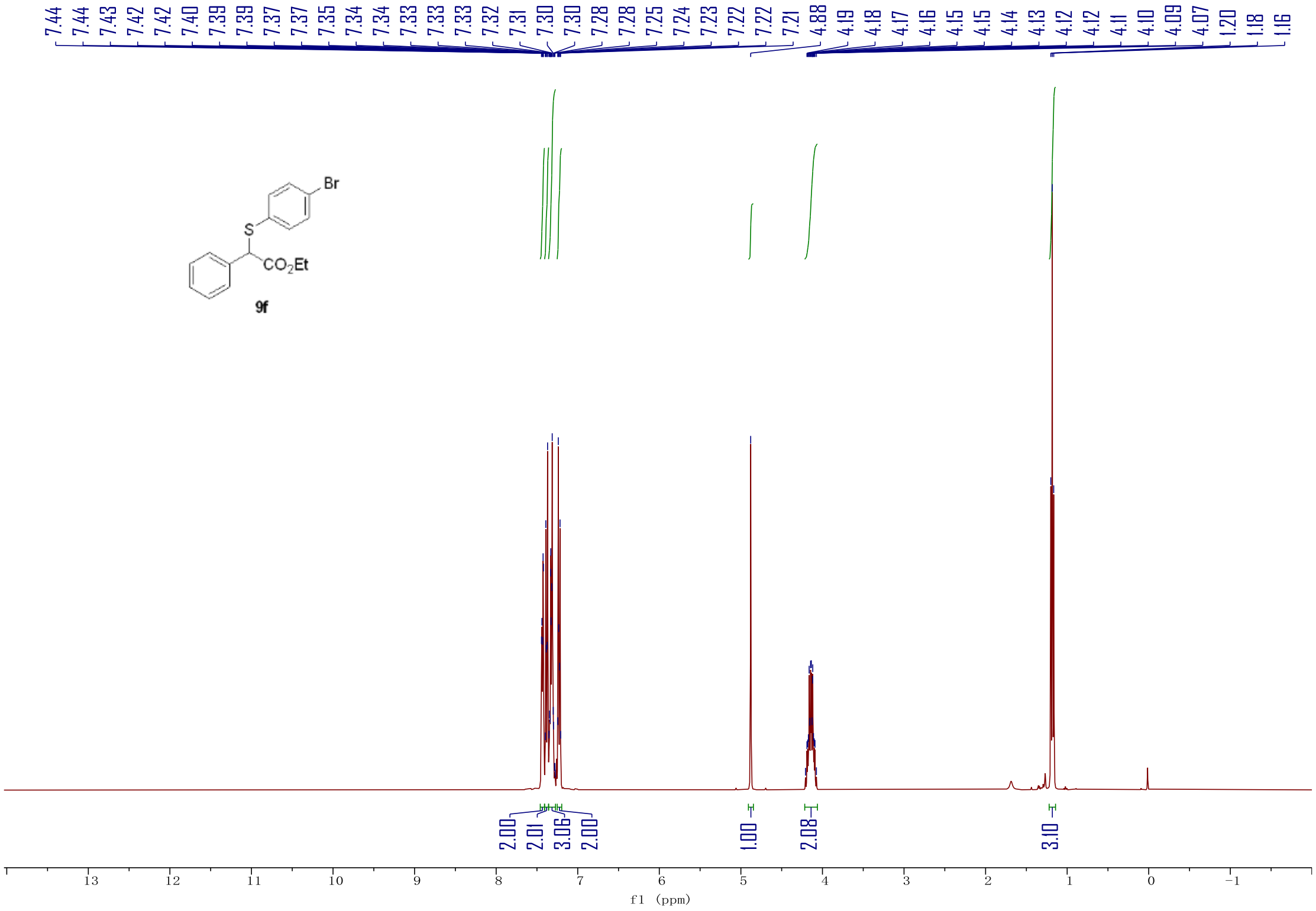

^{13}C NMR Spectrum of **9f** (101 MHz, $CDCl_3$)

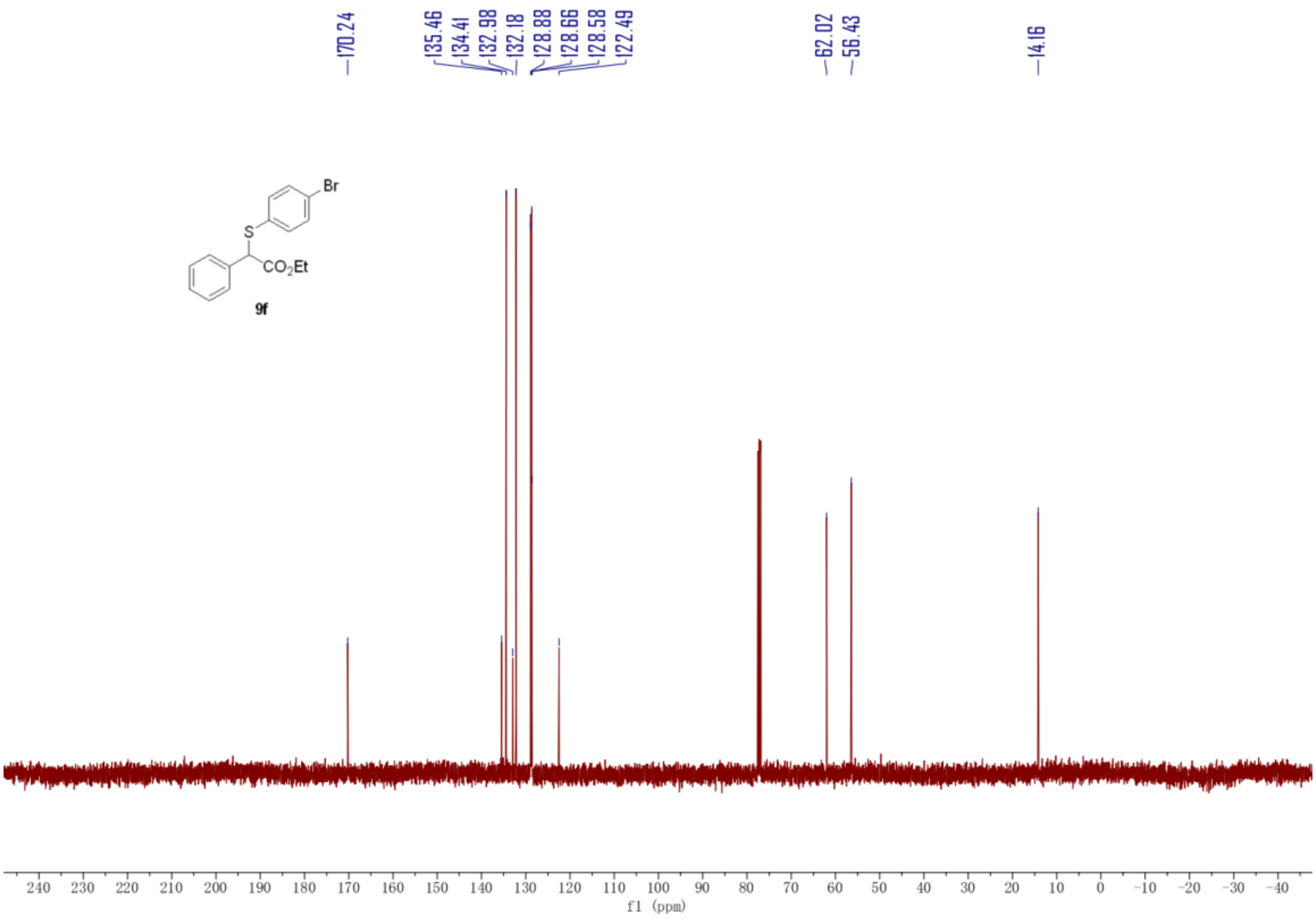

^{1}H NMR Spectrum of **9g** (400 MHz, $CDCl_3$)

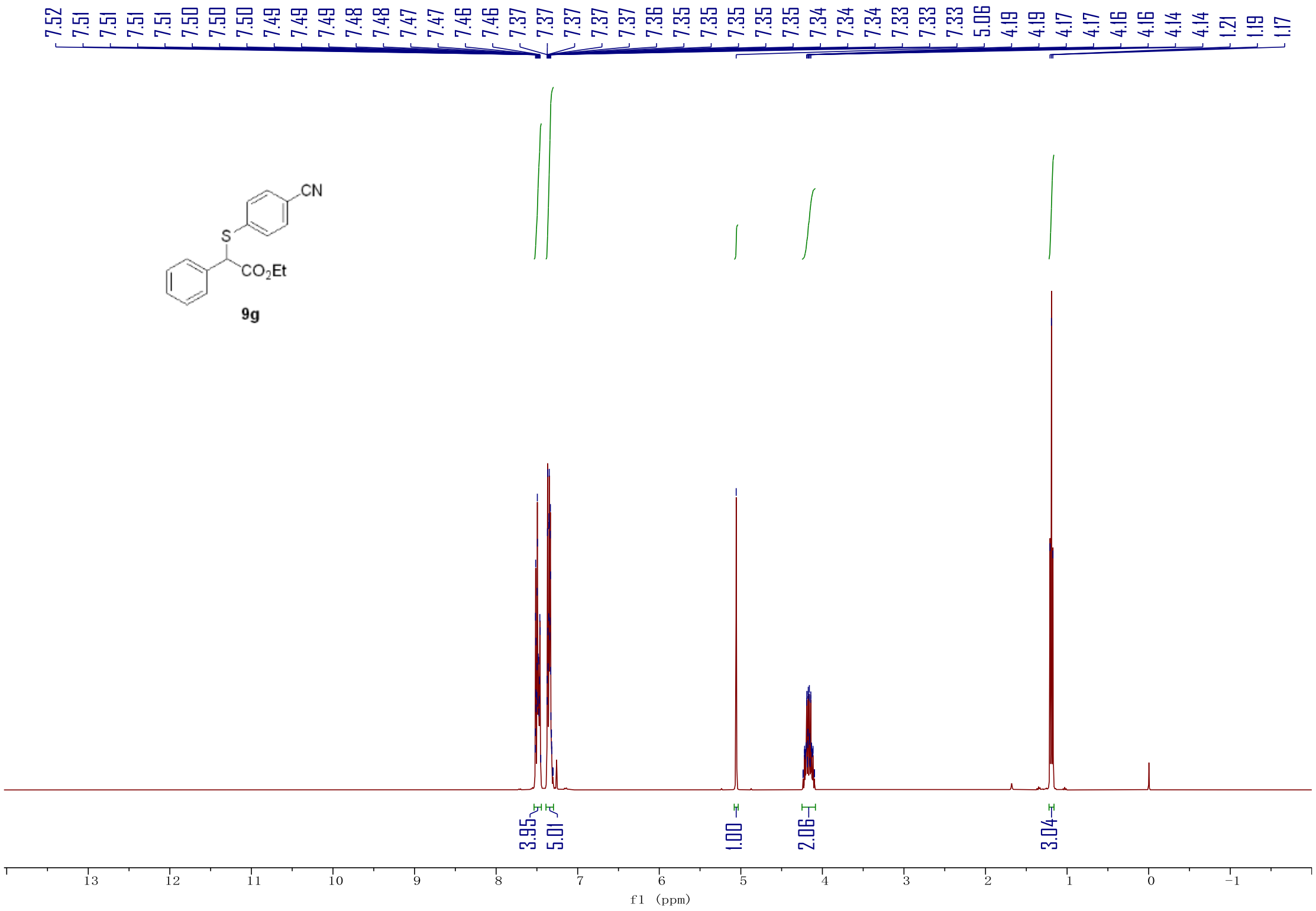

^{13}C NMR Spectrum of **9g** (101 MHz, $CDCl_3$)

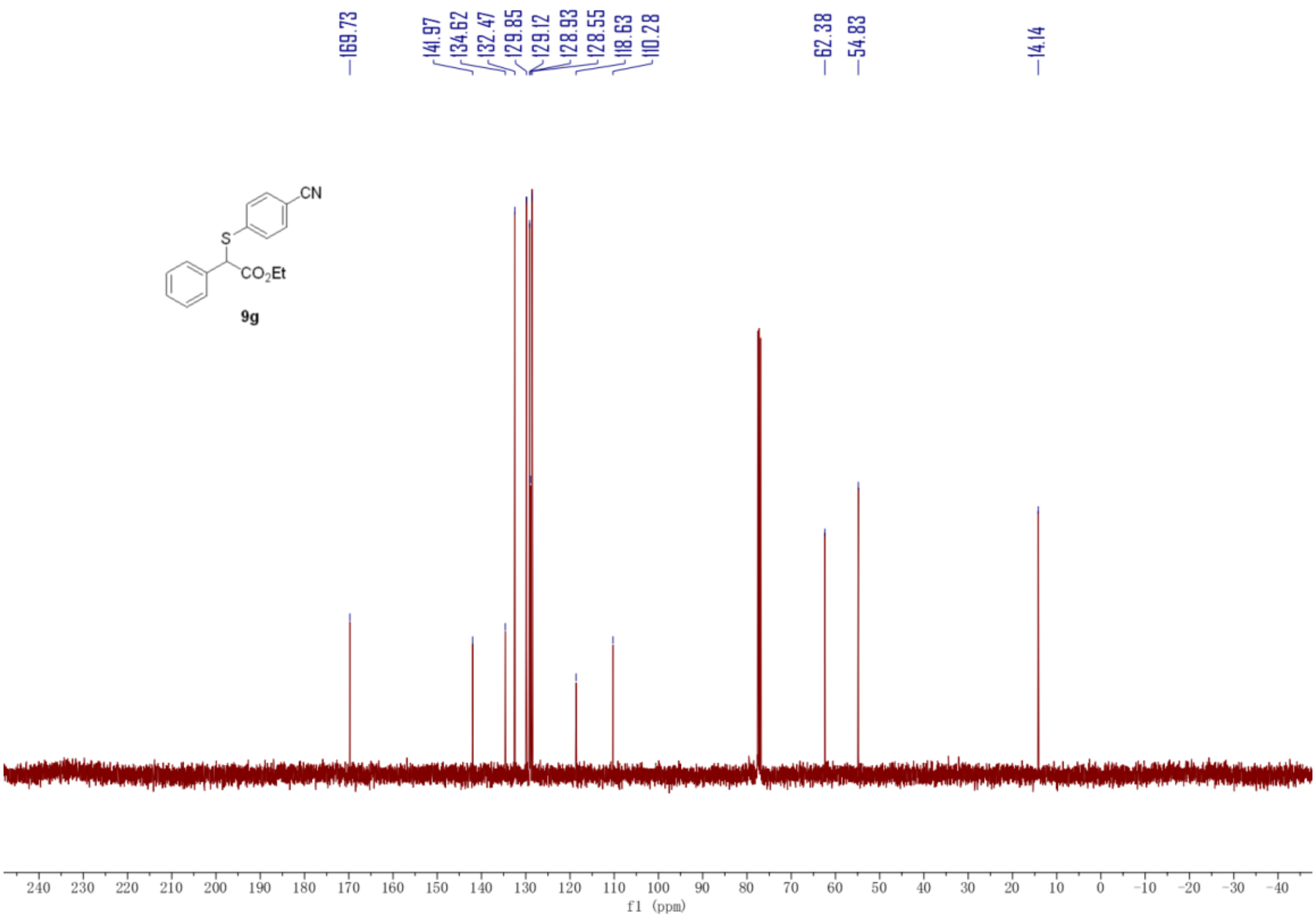

^{1}H NMR Spectrum of **9h** (400 MHz, $CDCl_3$)

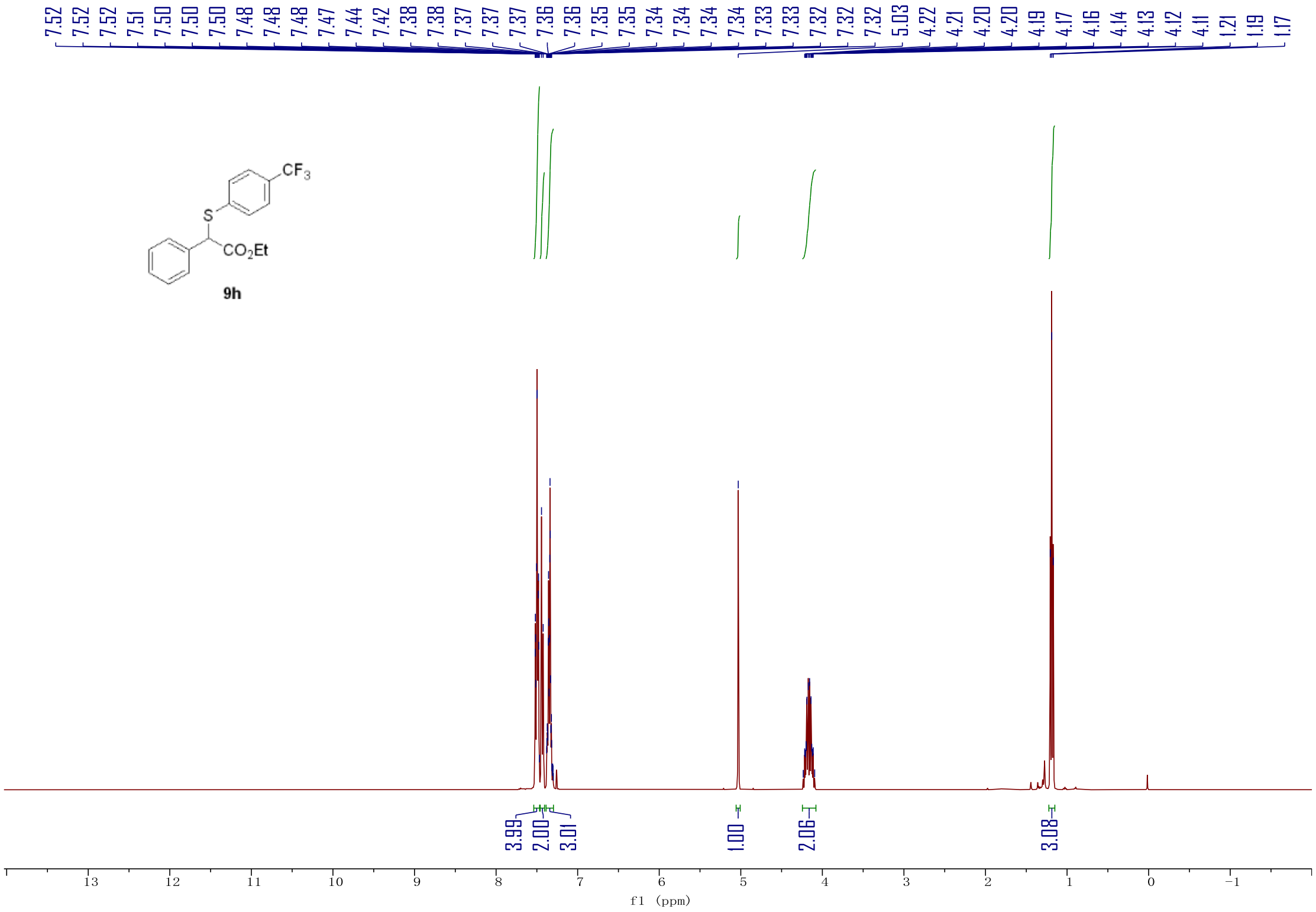

^{19}F NMR Spectrum of **9h** (376 MHz, $CDCl_3$)

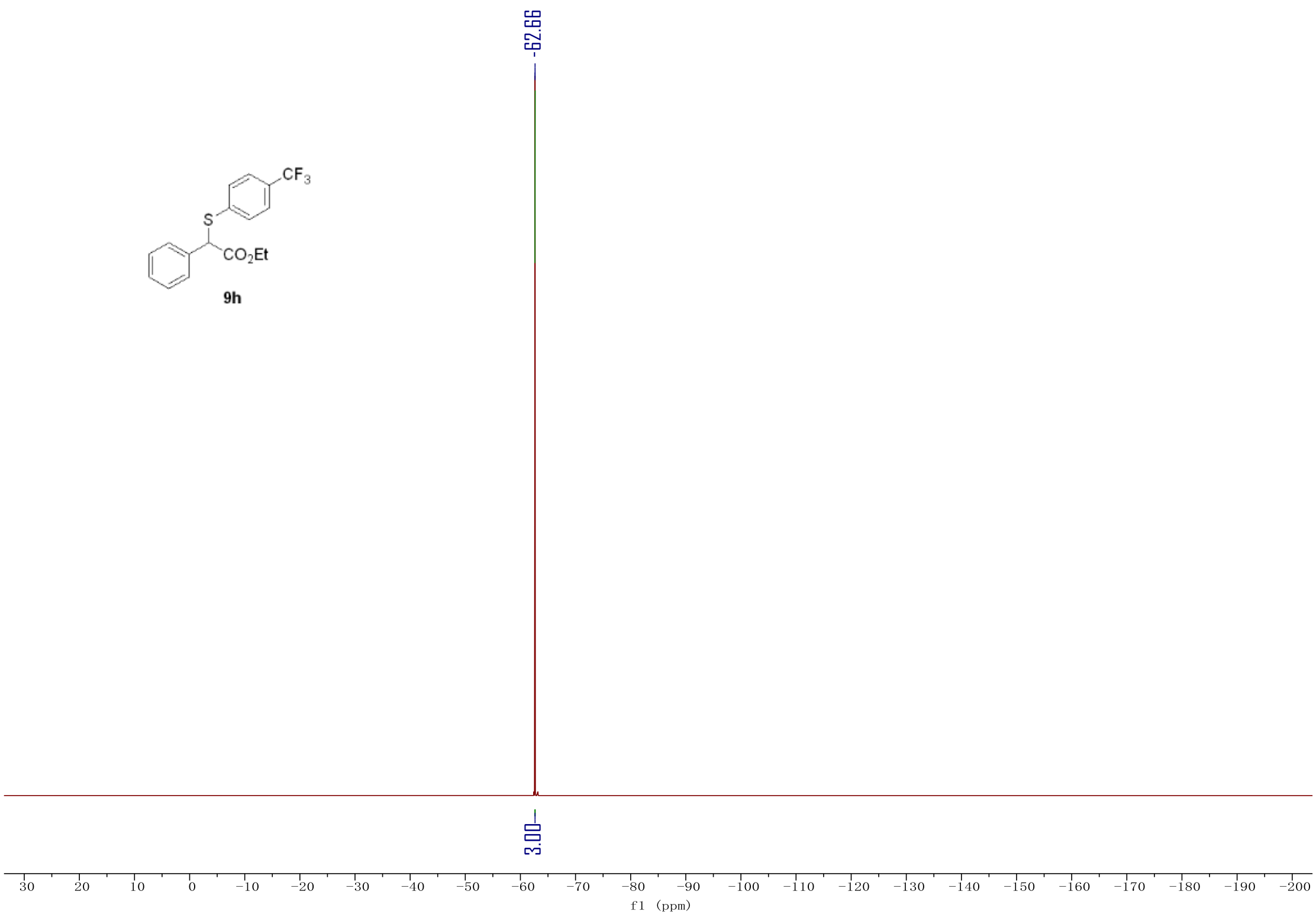

^{13}**C NMR** Spectrum of **9h** (101 MHz, $CDCl_3$)

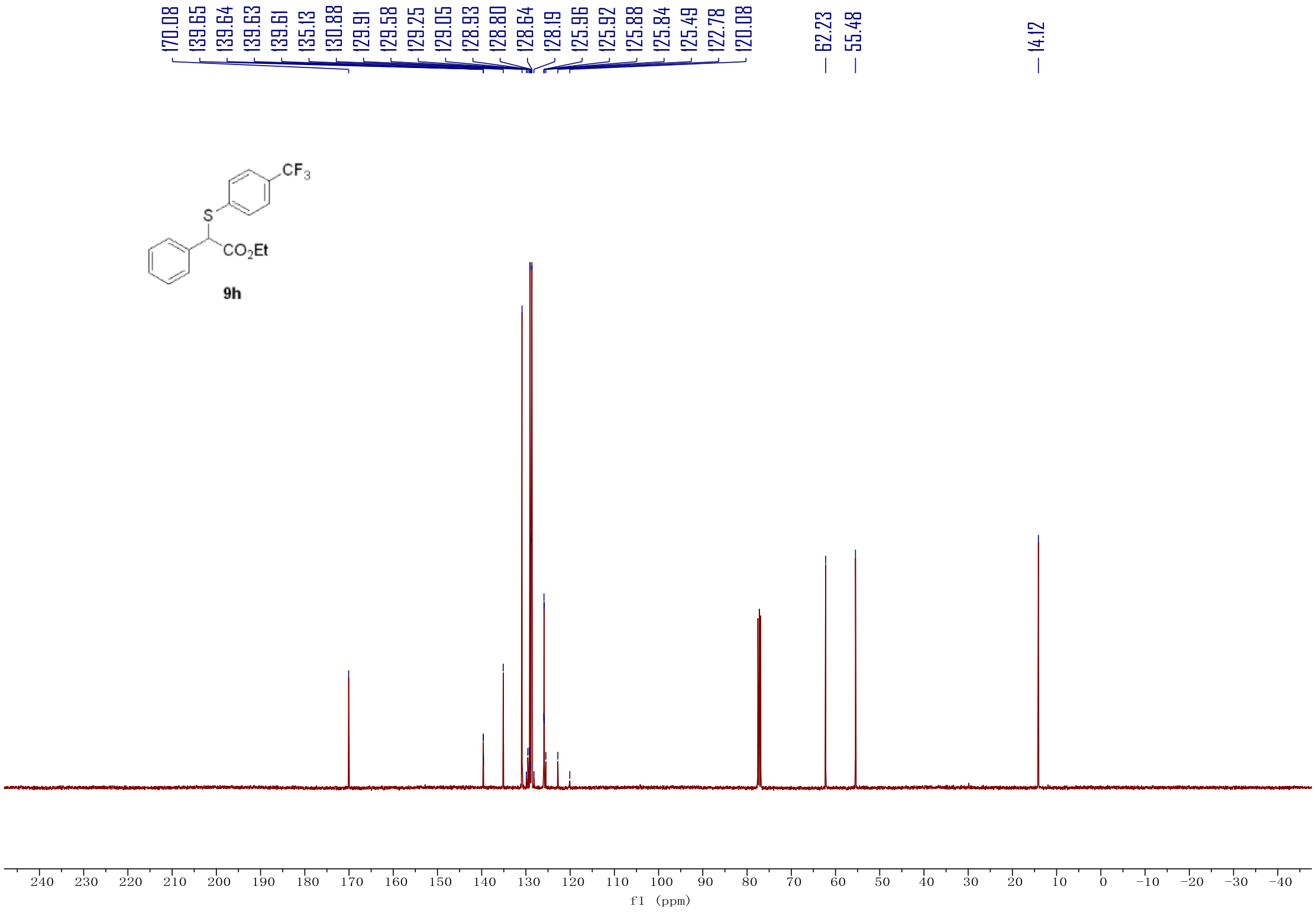

^{1}**H NMR** Spectrum of **9i** (400 MHz, $CDCl_3$)

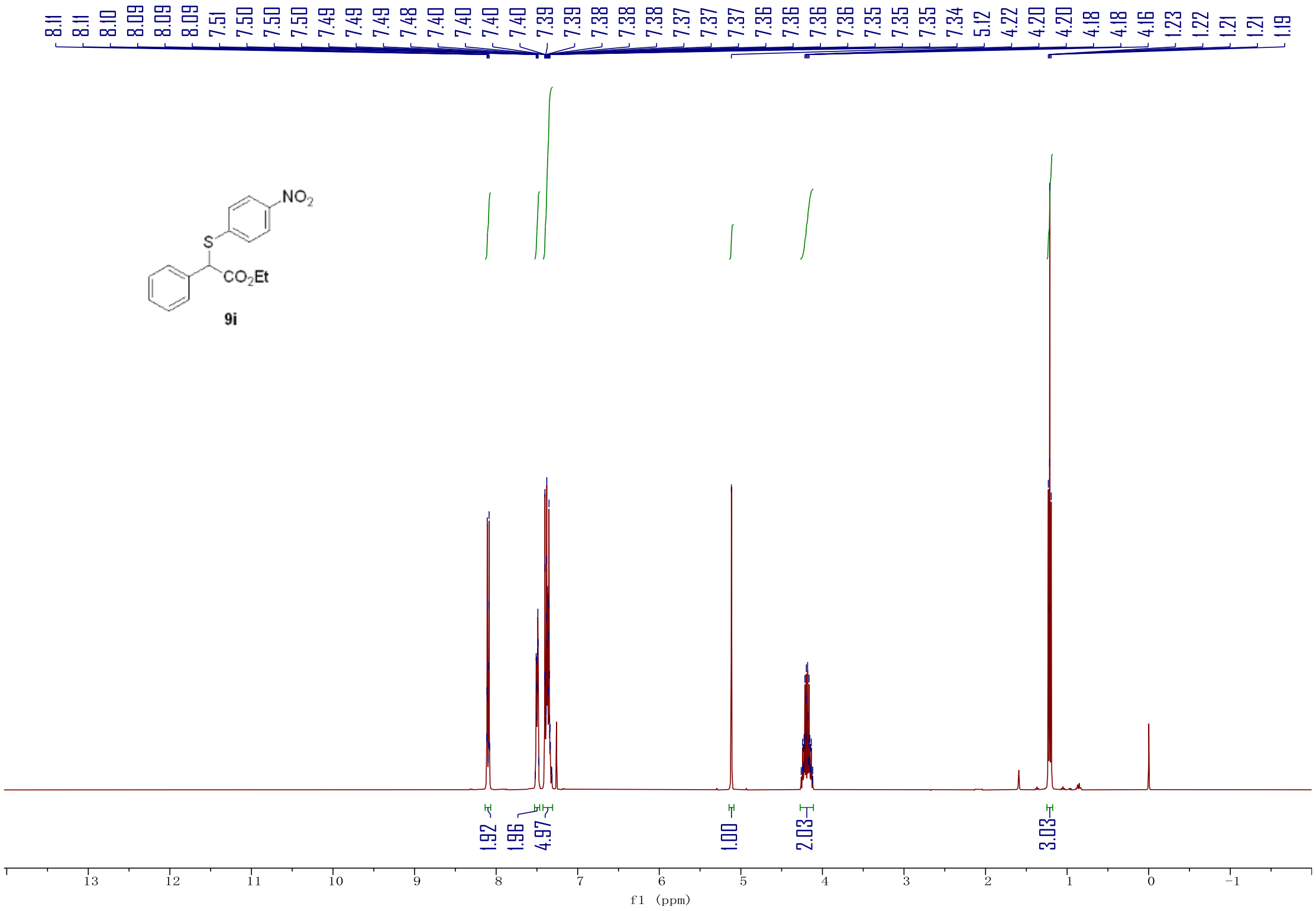

^{13}C NMR Spectrum of 9i (101 MHz, $CDCl_3$)

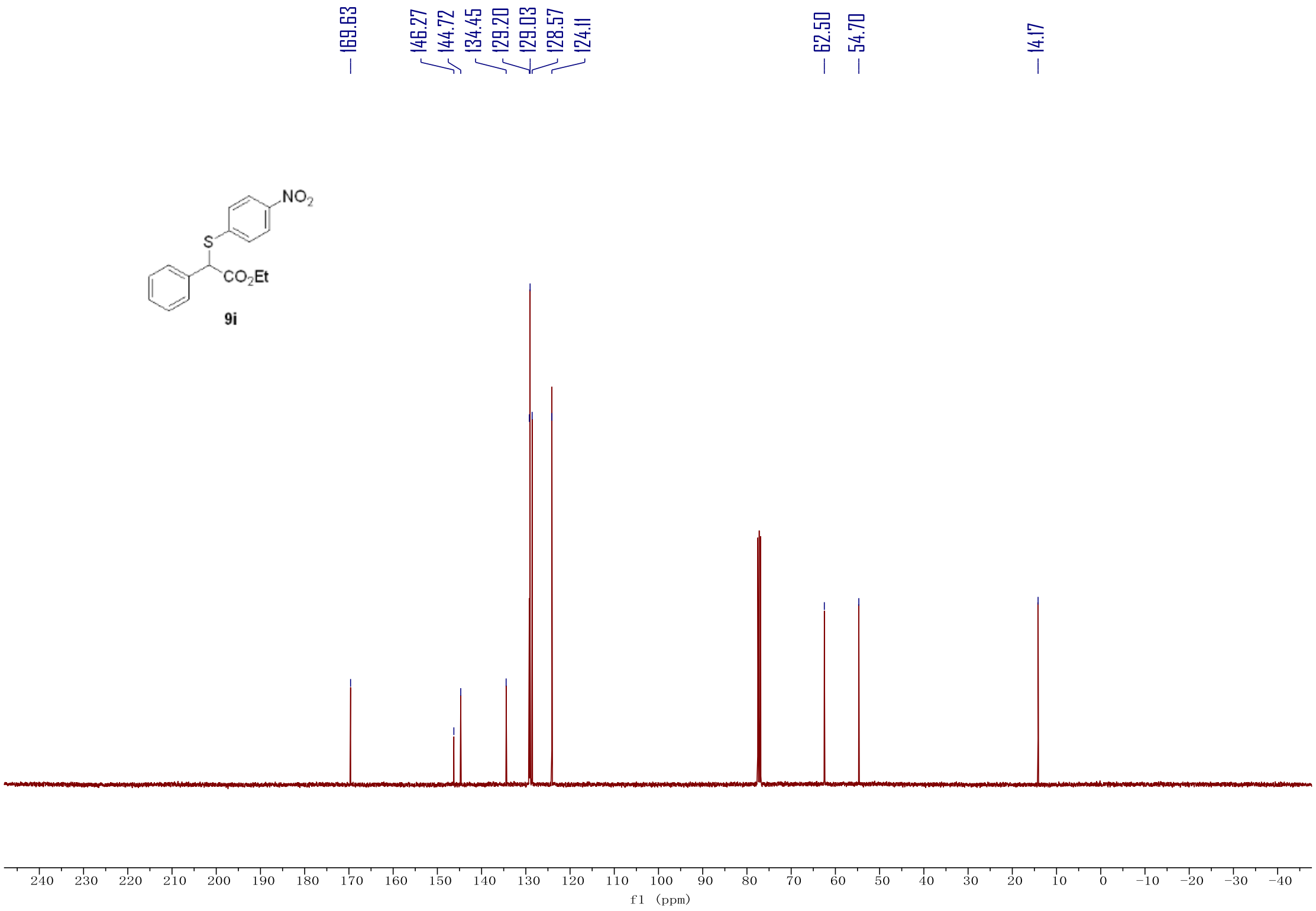

^{1}H NMR Spectrum of 9j (400 MHz, $CDCl_3$)

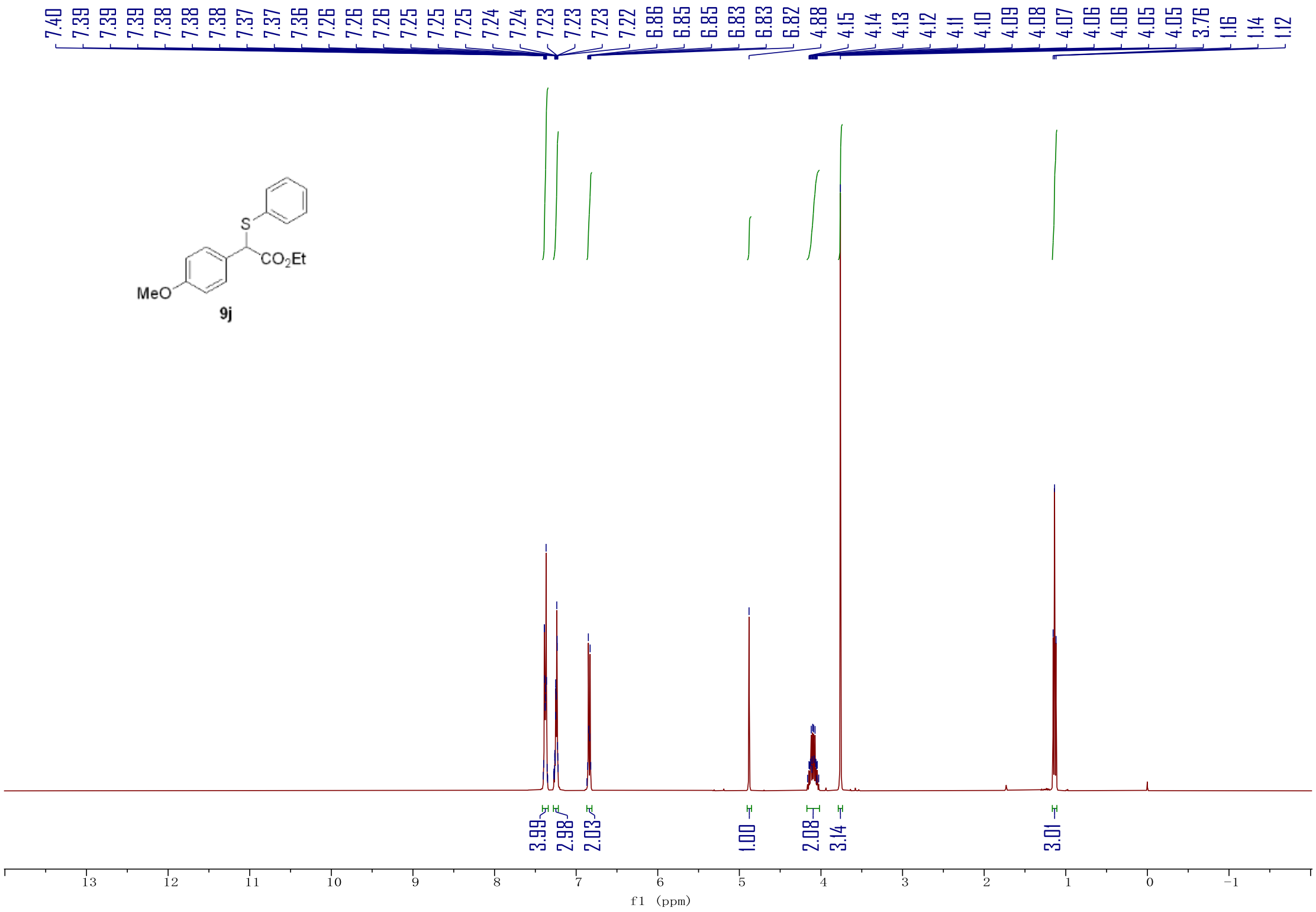

^{13}C NMR Spectrum of **9j** (101 MHz, $CDCl_3$)

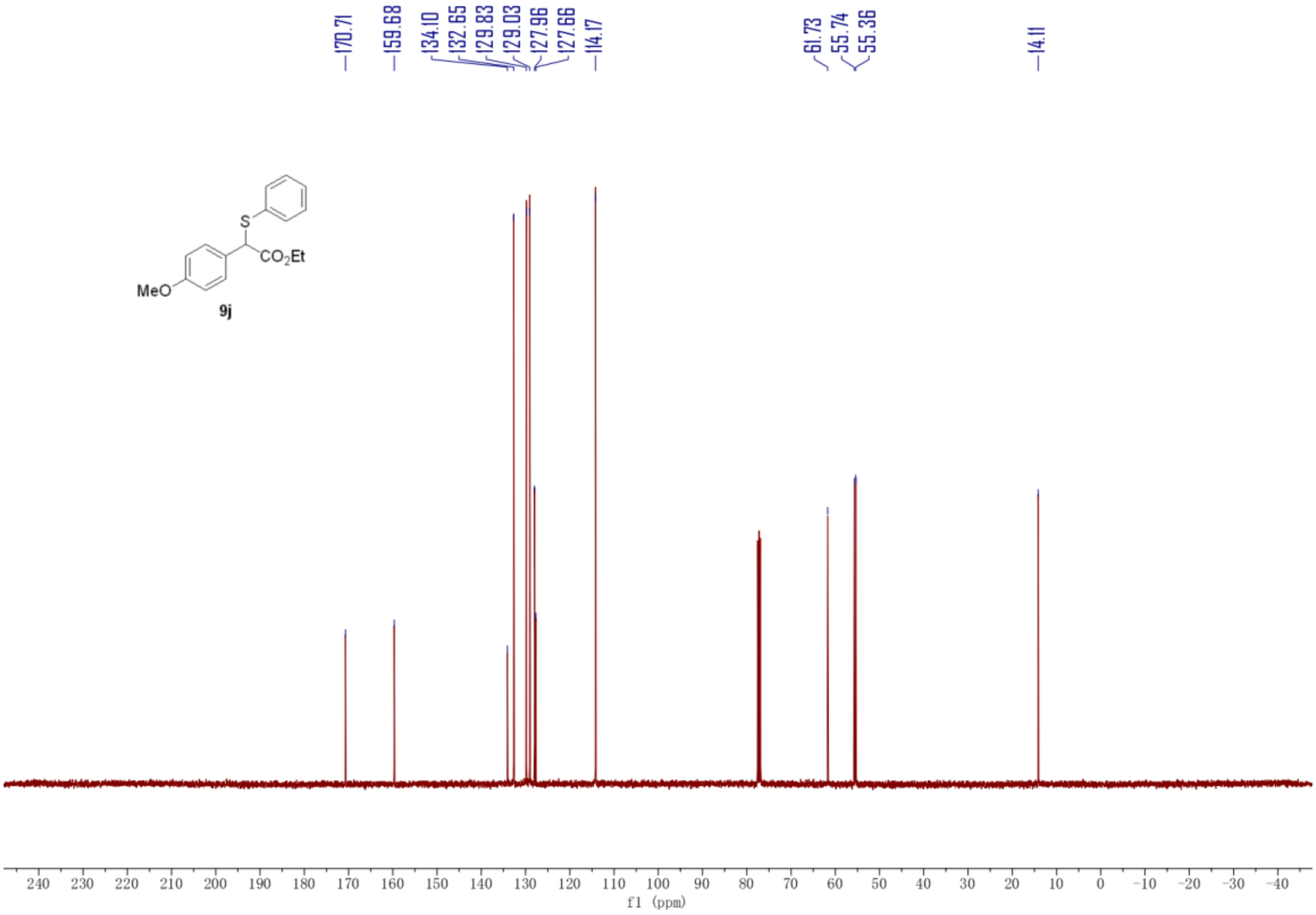

^{1}H NMR Spectrum of **9k** (400 MHz, $CDCl_3$)

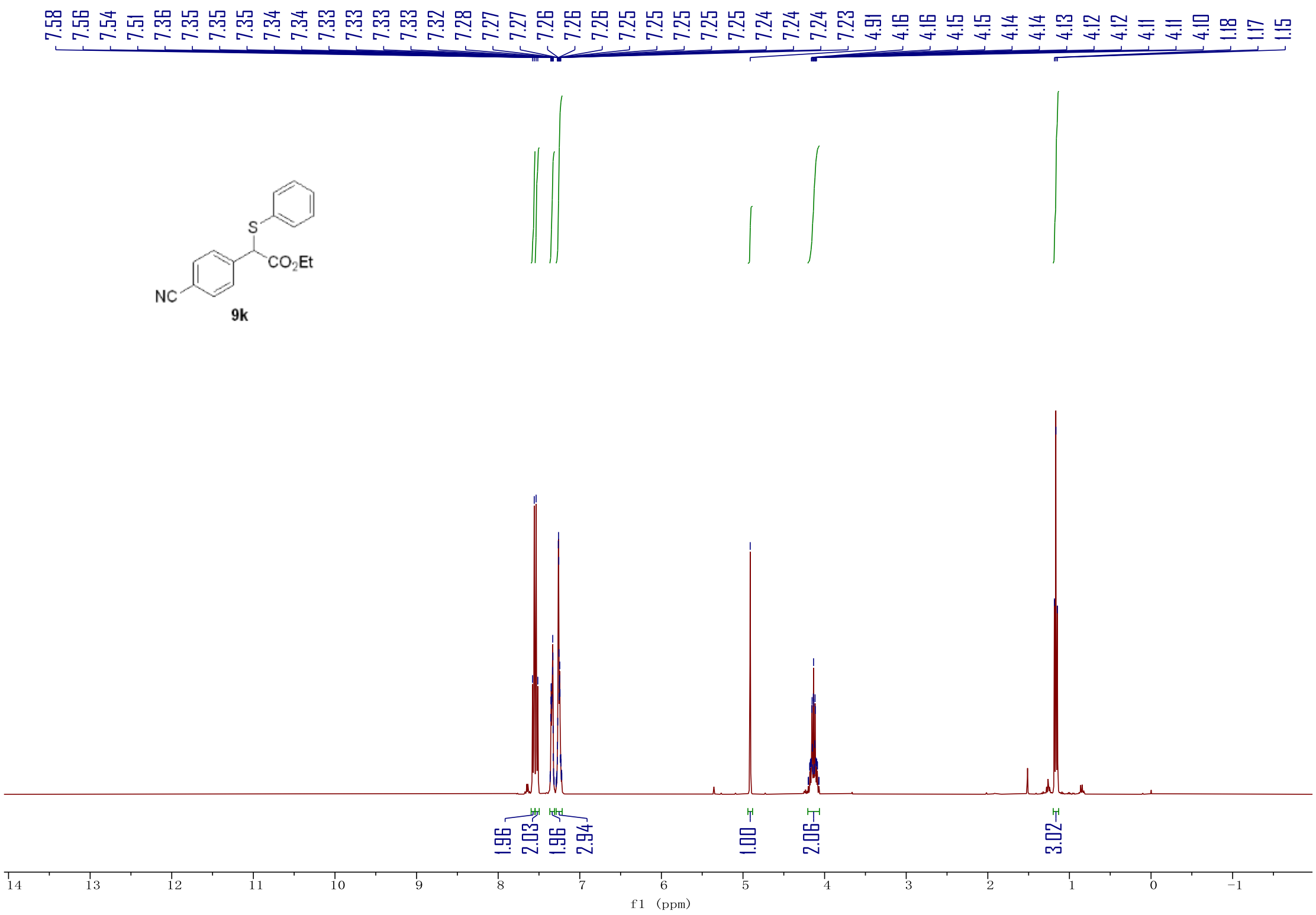

^{13}C NMR Spectrum of **9k** (101 MHz, $CDCl_3$)

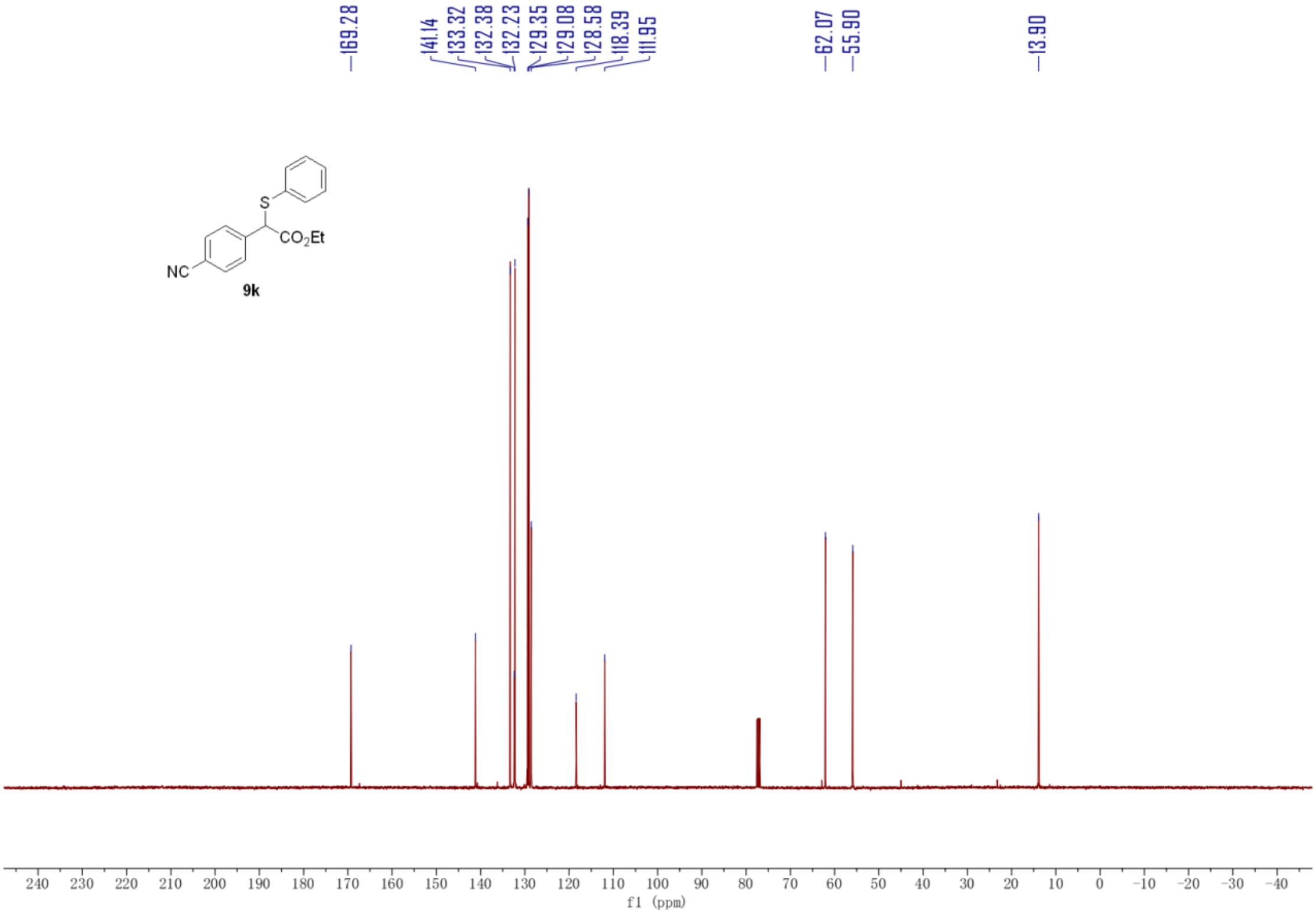

^{1}H NMR Spectrum of **9l** (400 MHz, $CDCl_3$)

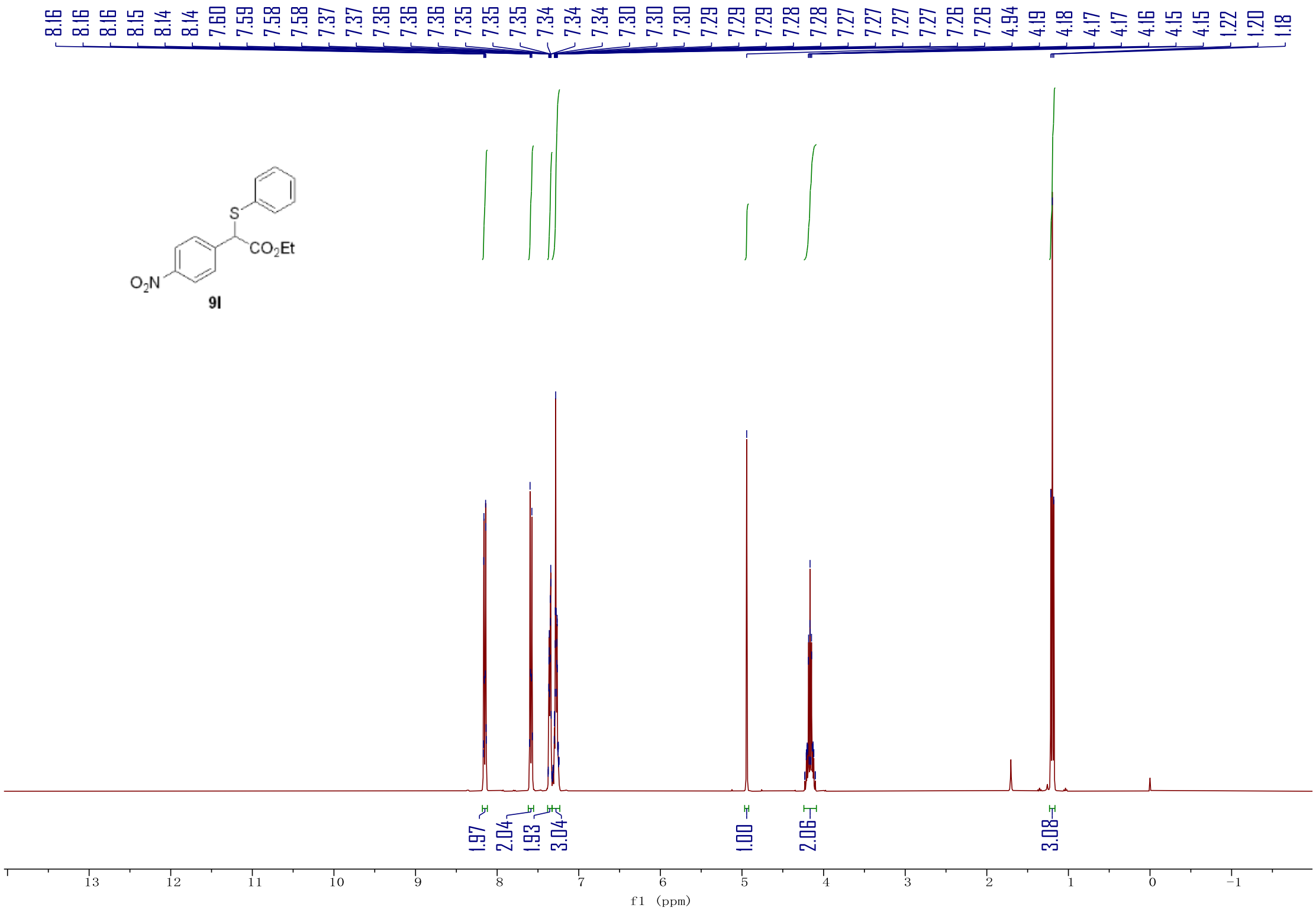

^{13}C NMR Spectrum of 9l (101 MHz, $CDCl_3$)

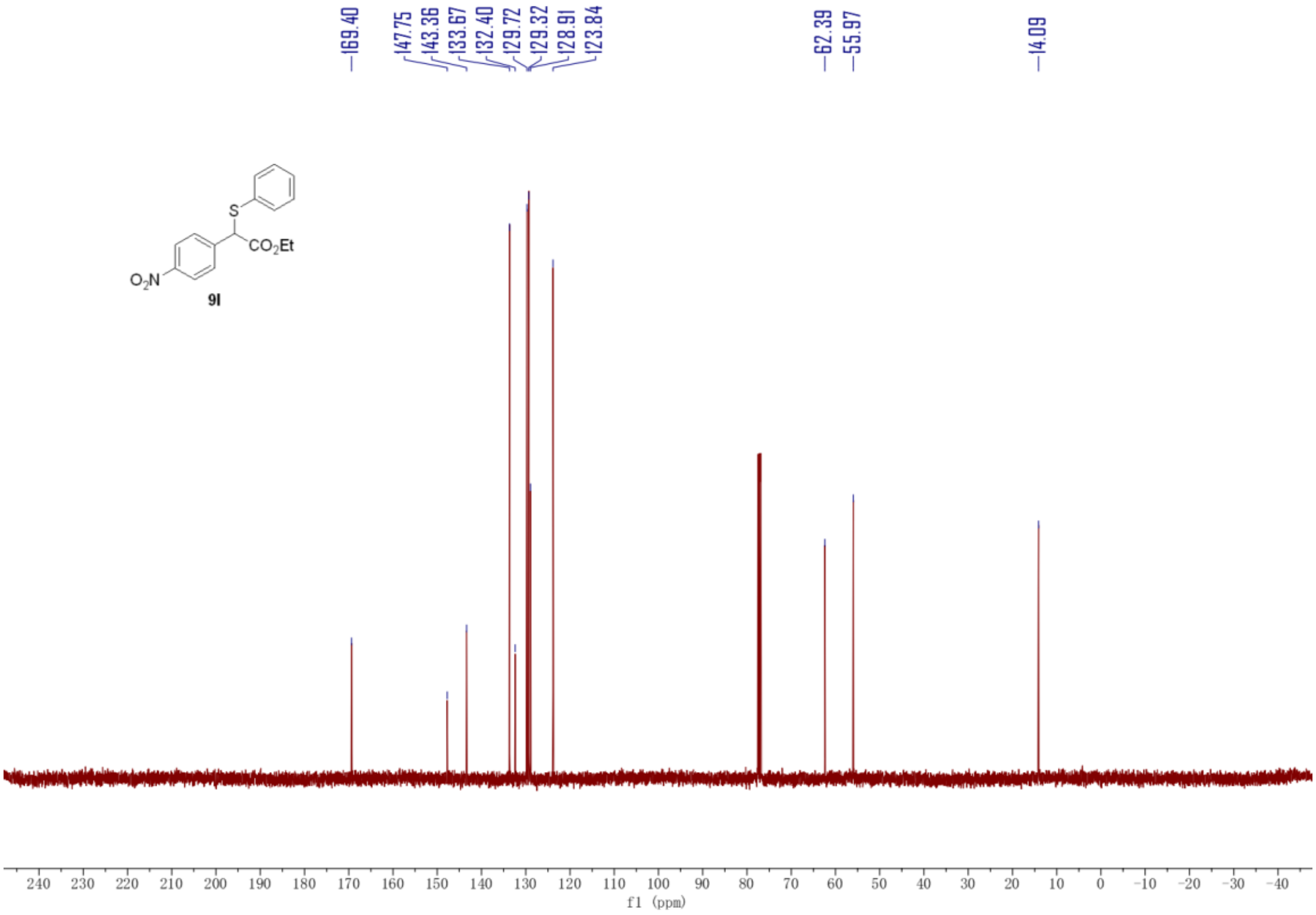

^{1}H NMR Spectrum of 9m (400 MHz, $CDCl_3$)

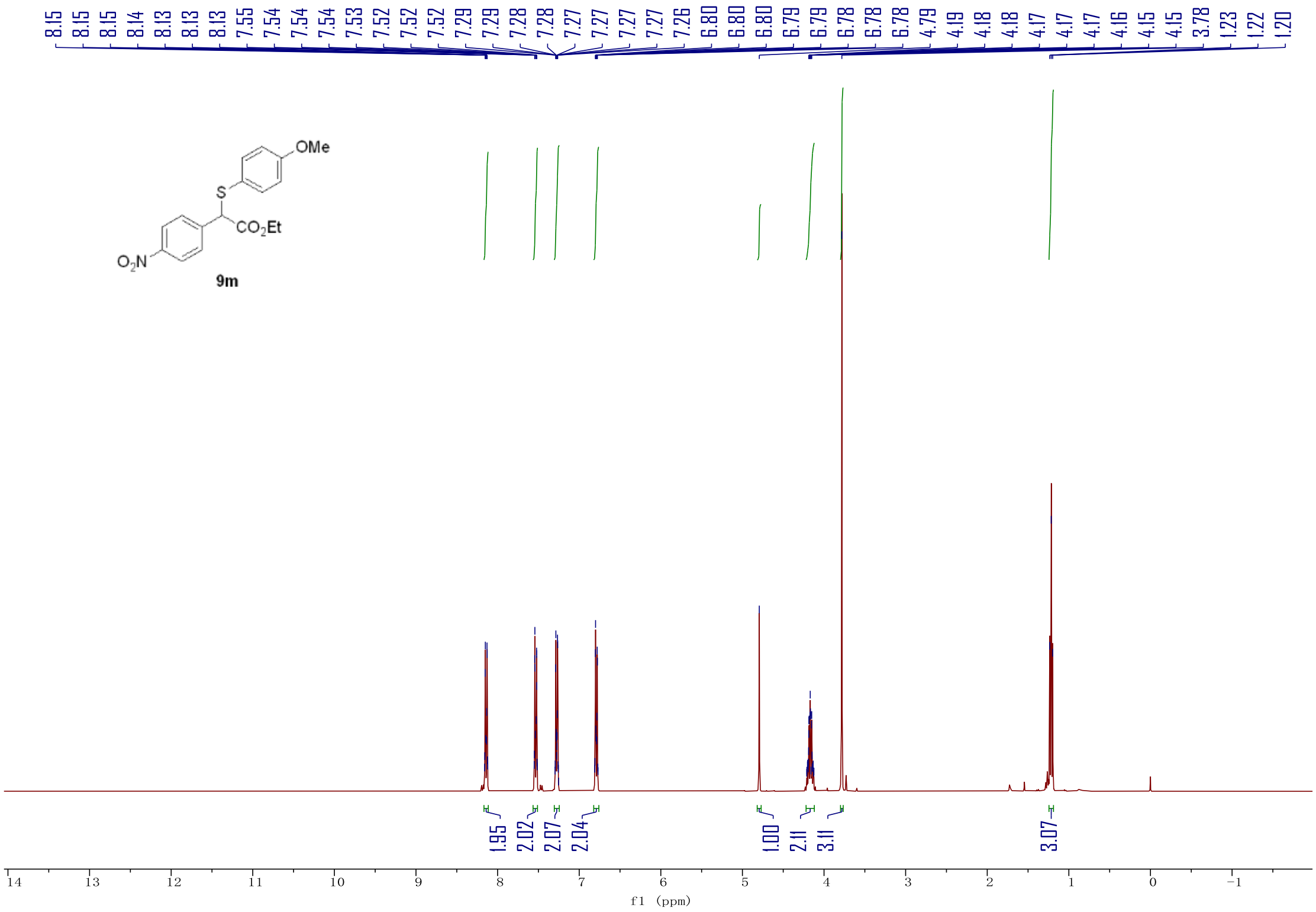

^{13}C NMR Spectrum of 9m (101 MHz, $CDCl_3$)

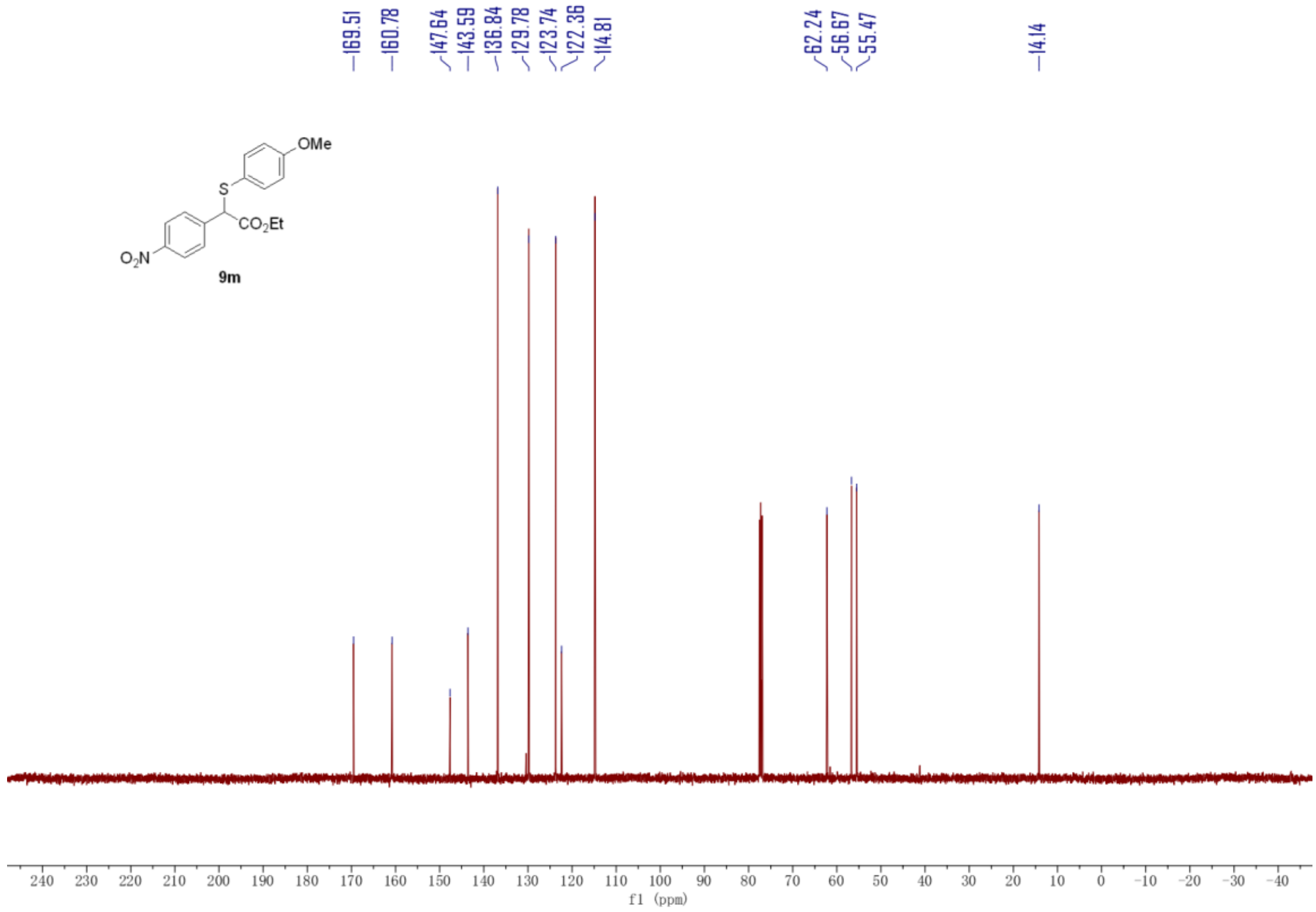

9m

^{1}H NMR Spectrum of 9n (400 MHz, $CDCl_3$)

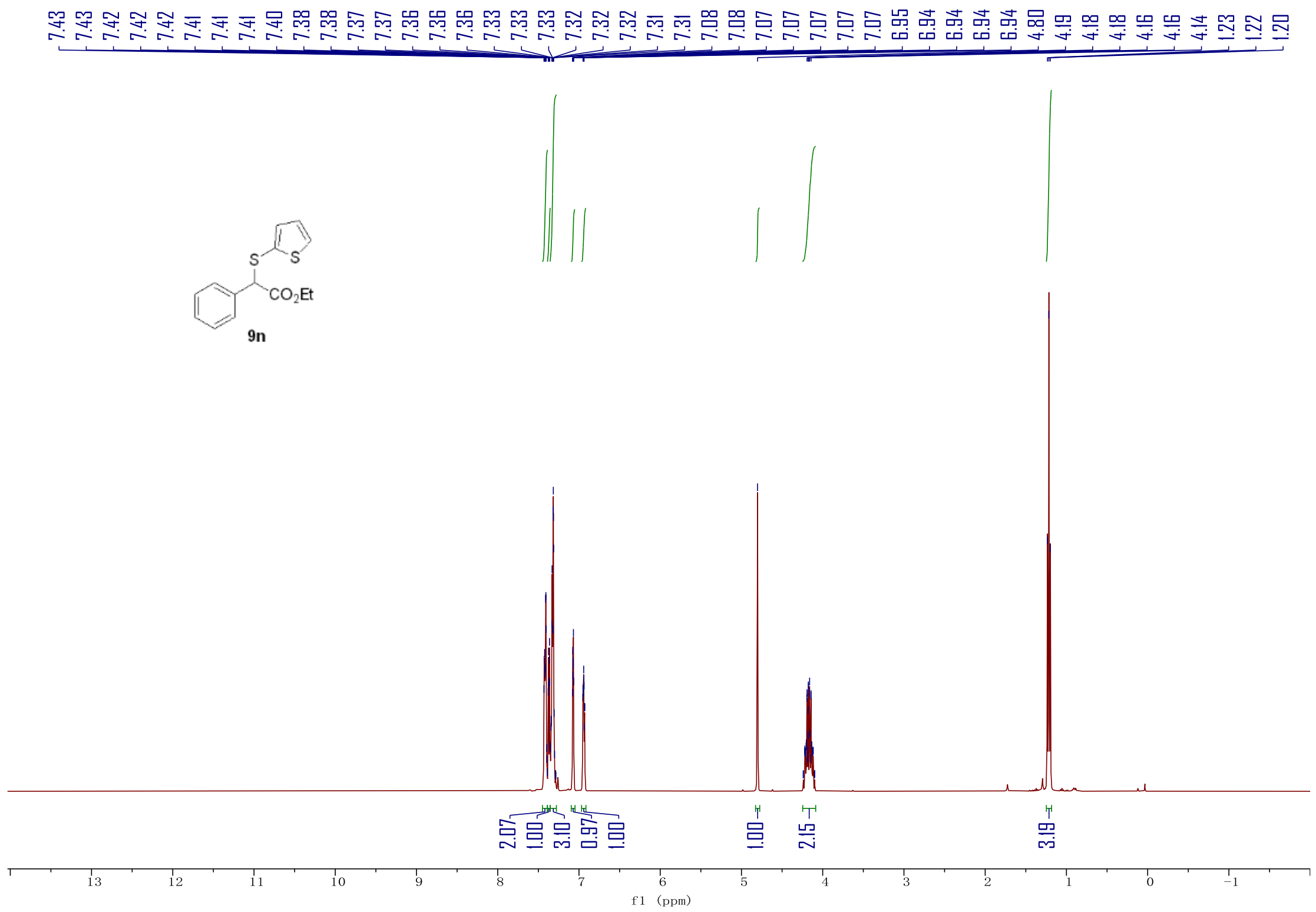

9n

^{13}C NMR Spectrum of **9n** (101 MHz, $CDCl_3$)

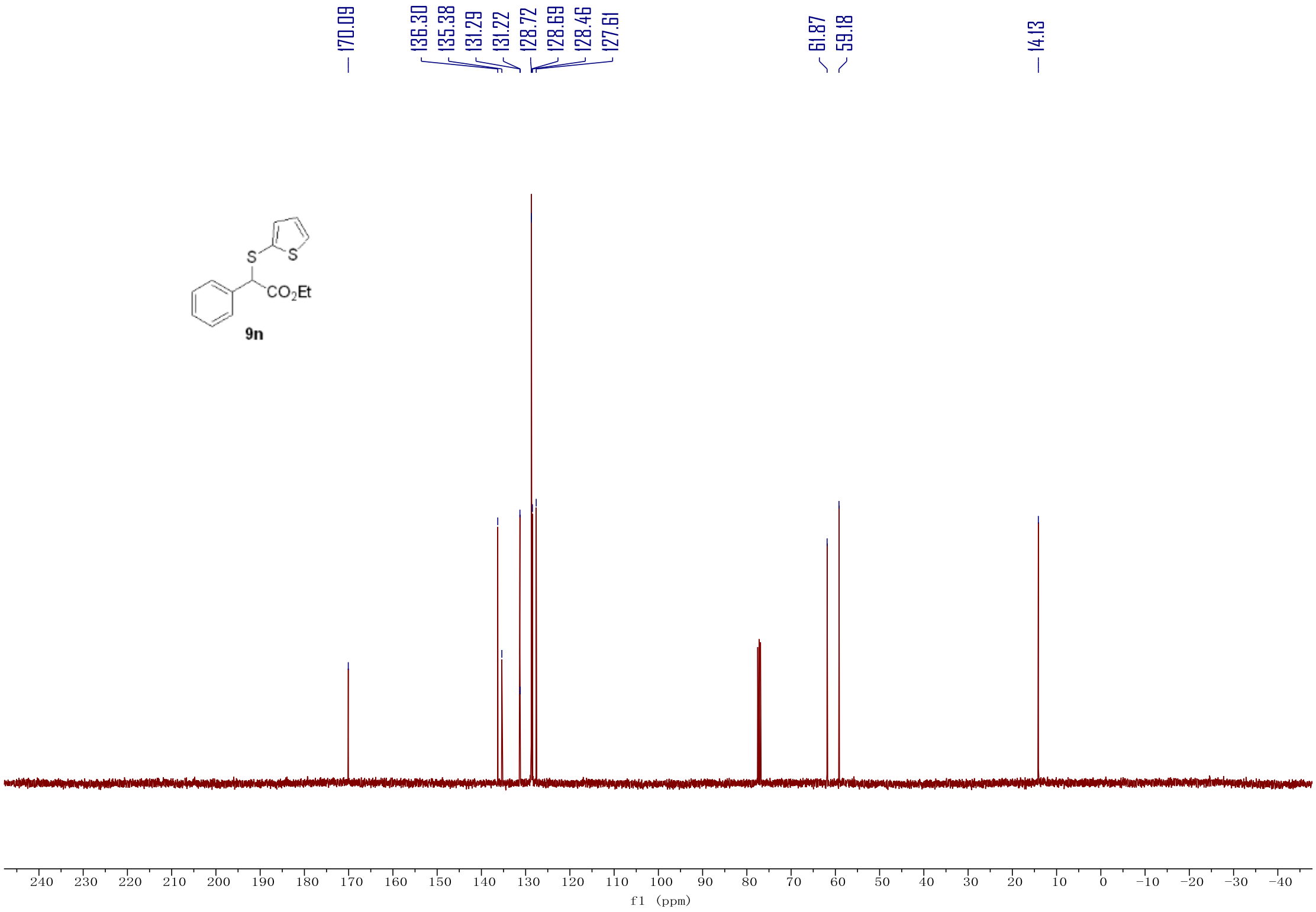

^{1}H NMR Spectrum of **9o** (400 MHz, $CDCl_3$)

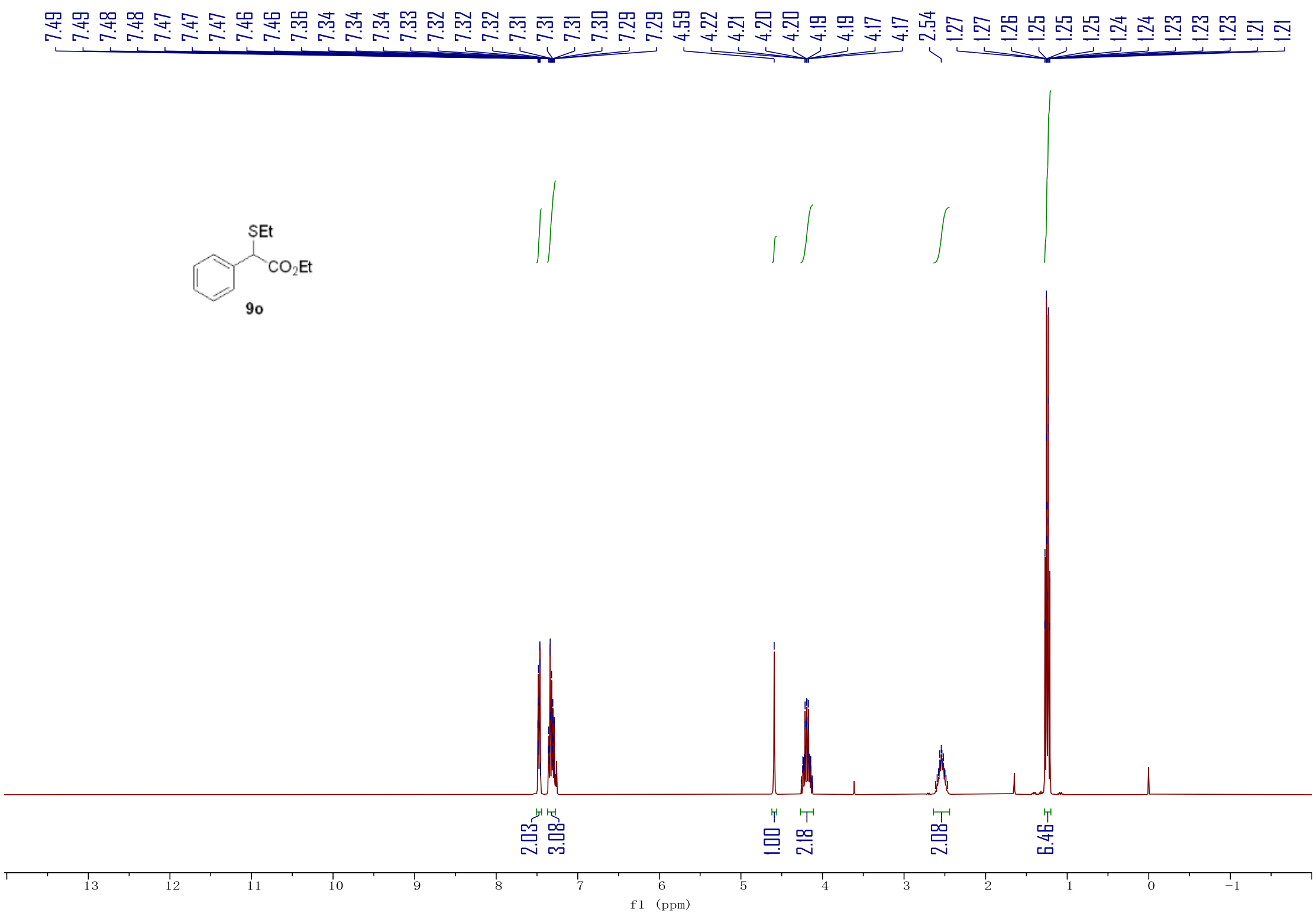

^{13}C NMR Spectrum of **9o** (101 MHz, $CDCl_3$)

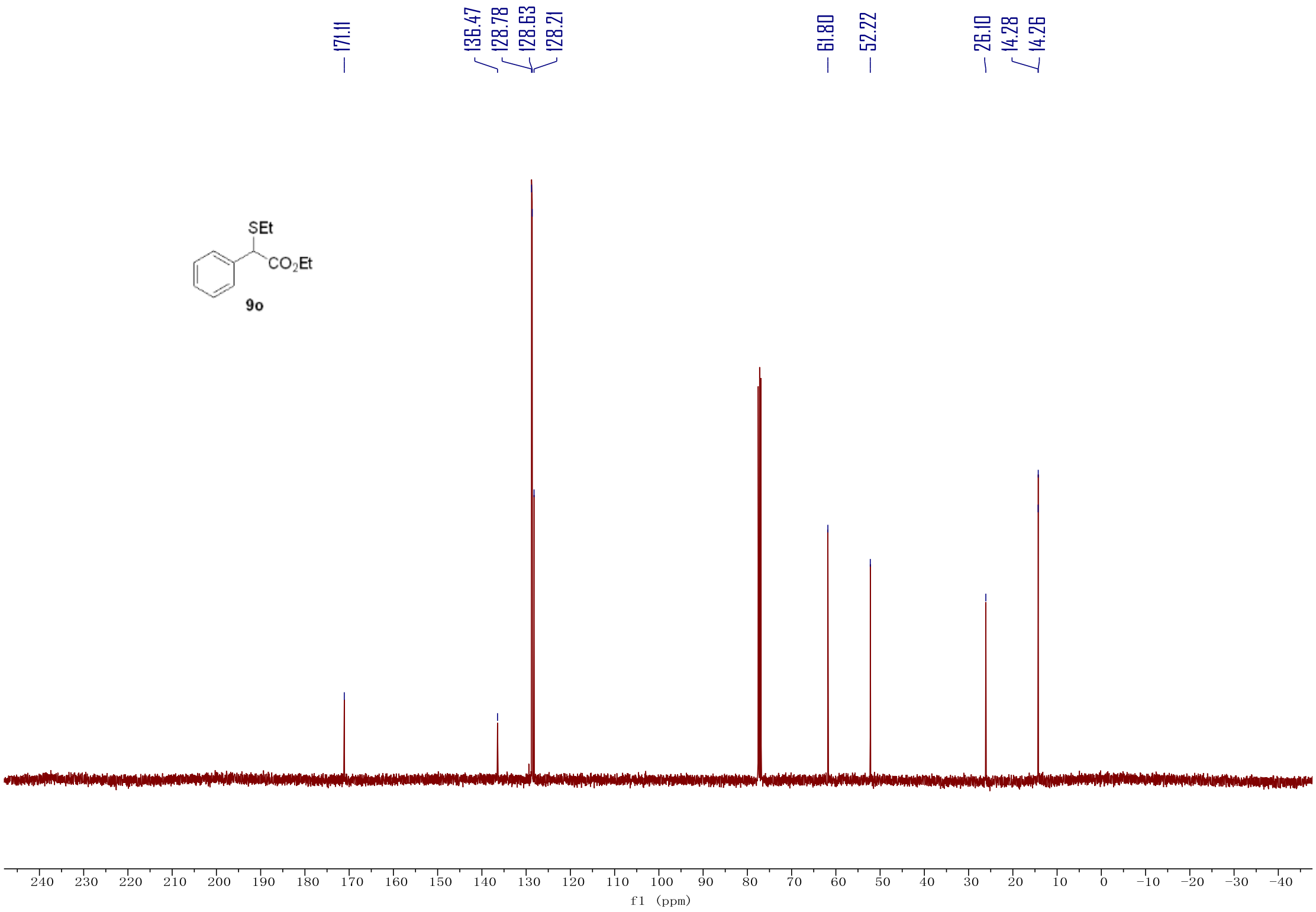

^{1}H NMR Spectrum of **9p** (400 MHz, $CDCl_3$)

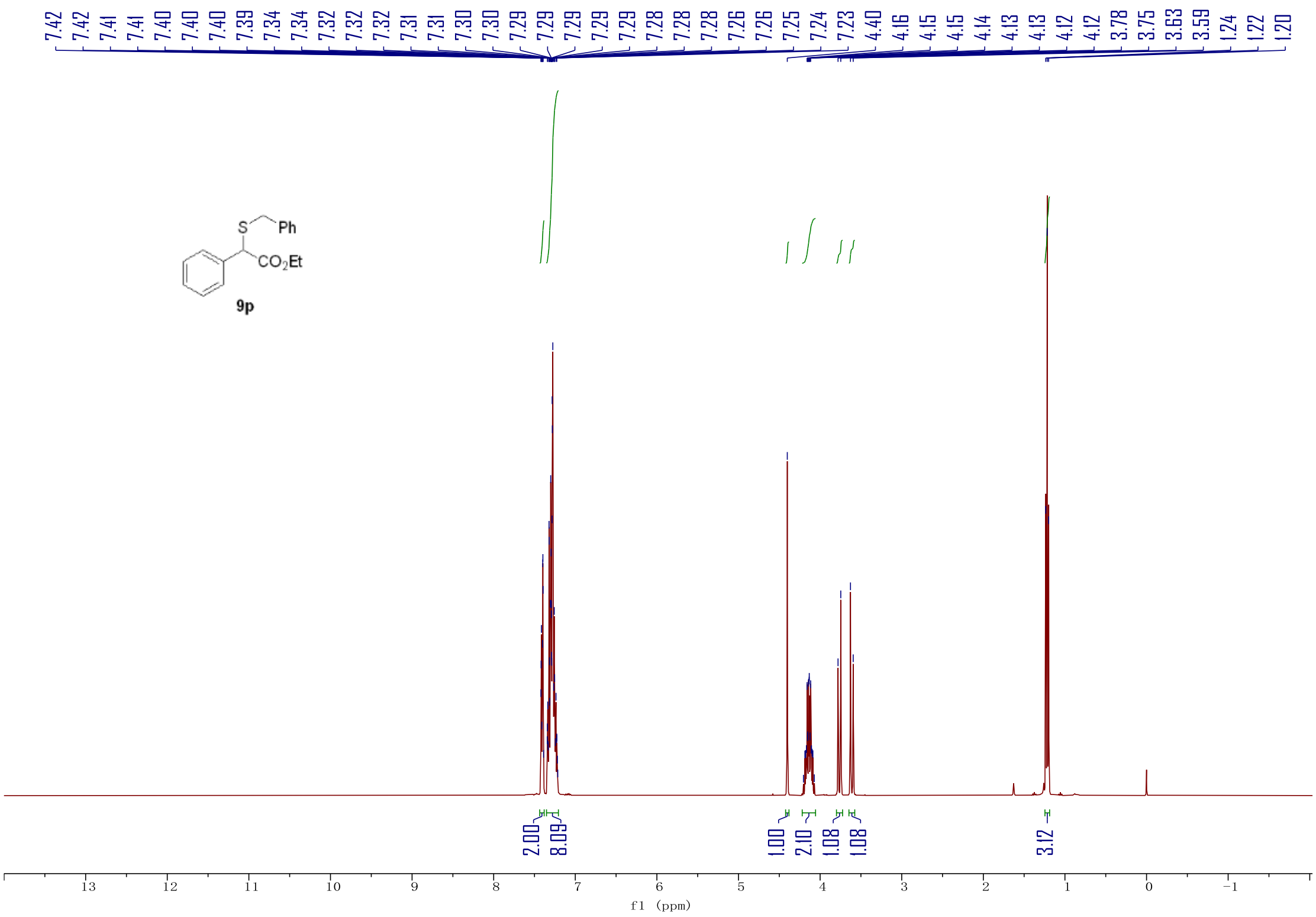

^{13}**C NMR** Spectrum of **9p** (101 MHz, $CDCl_3$)

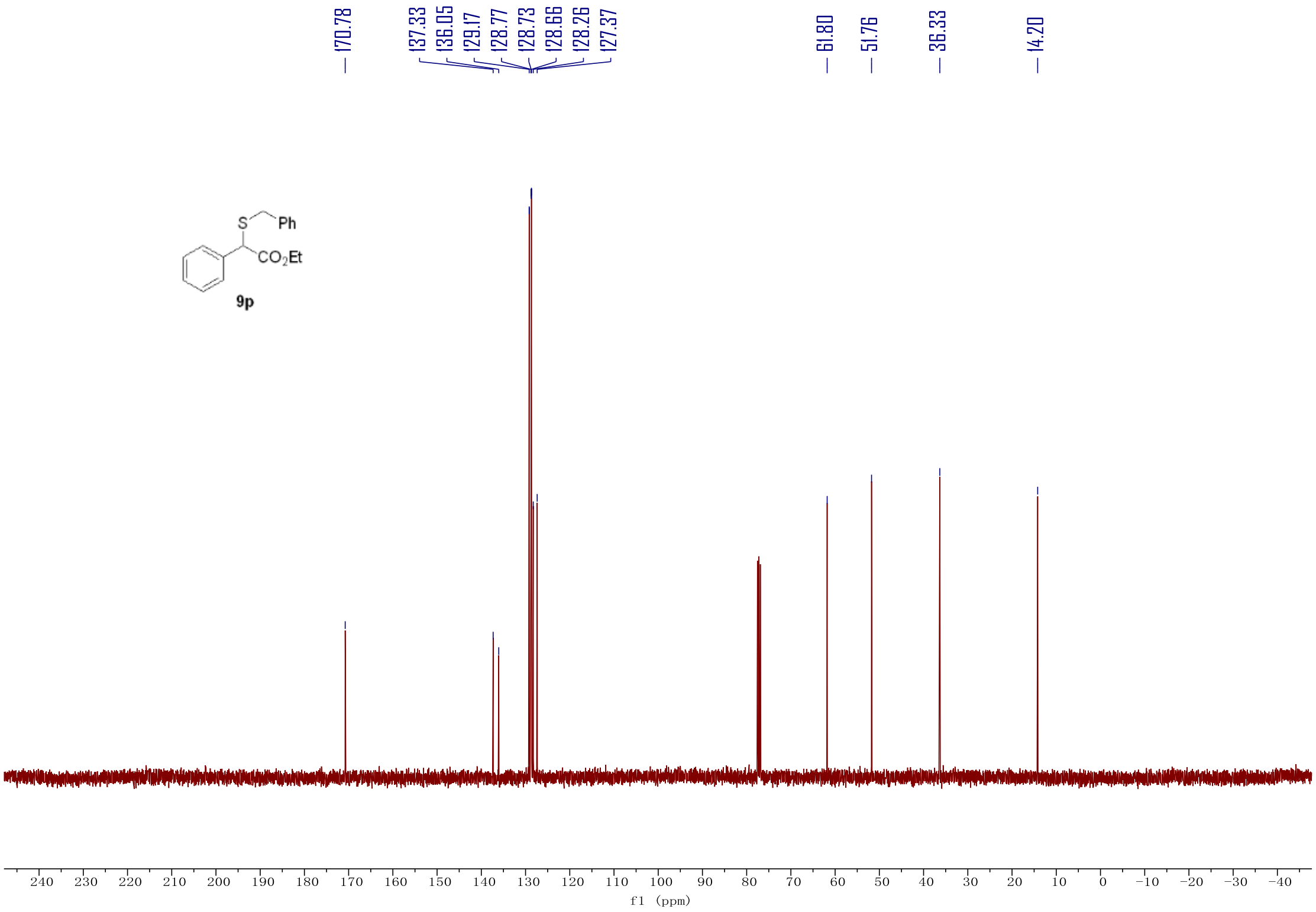

^{1}**H NMR** Spectrum of **9q** (400 MHz, $CDCl_3$)

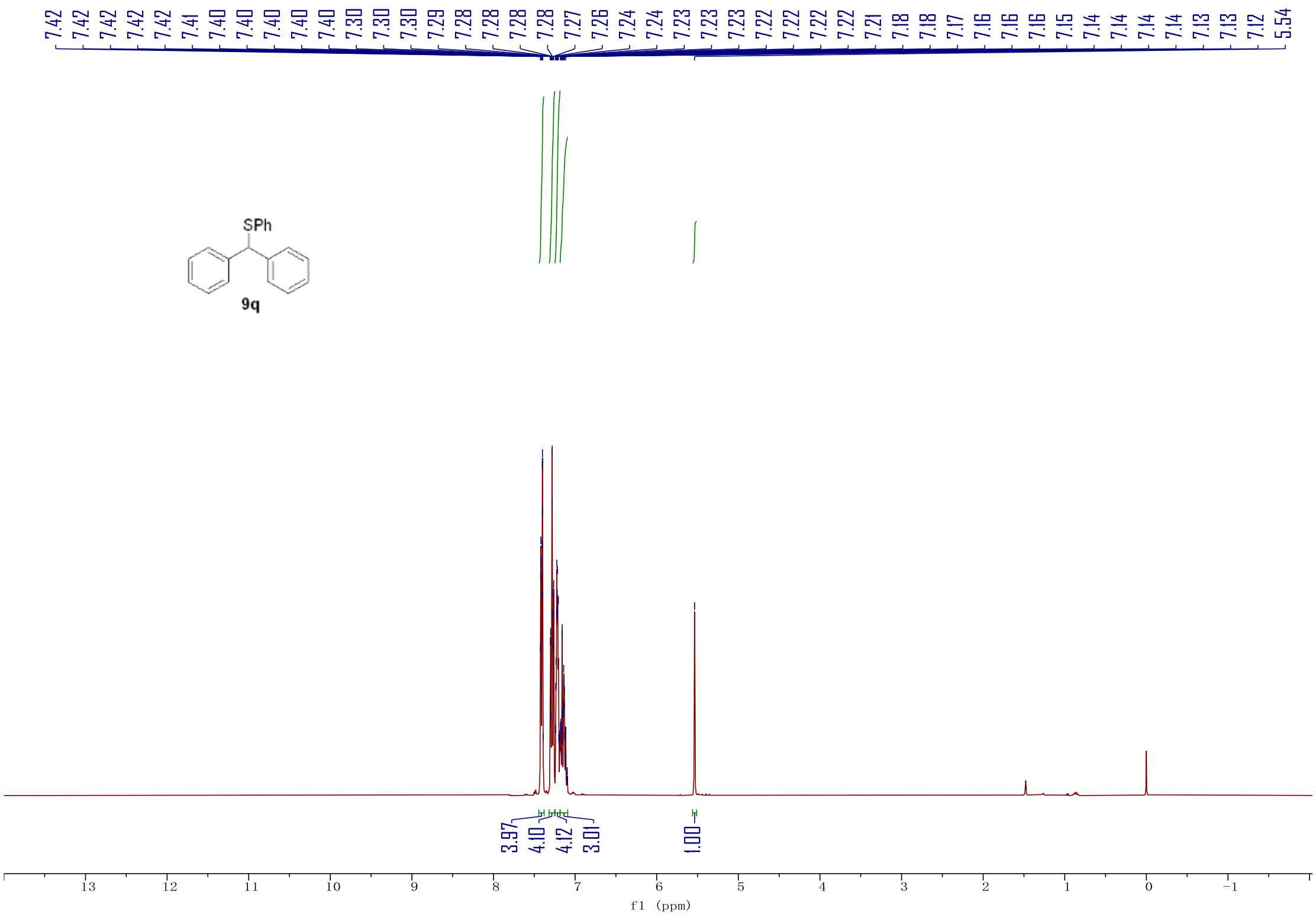

^{13}C NMR Spectrum of **9q** (101 MHz, $CDCl_3$)

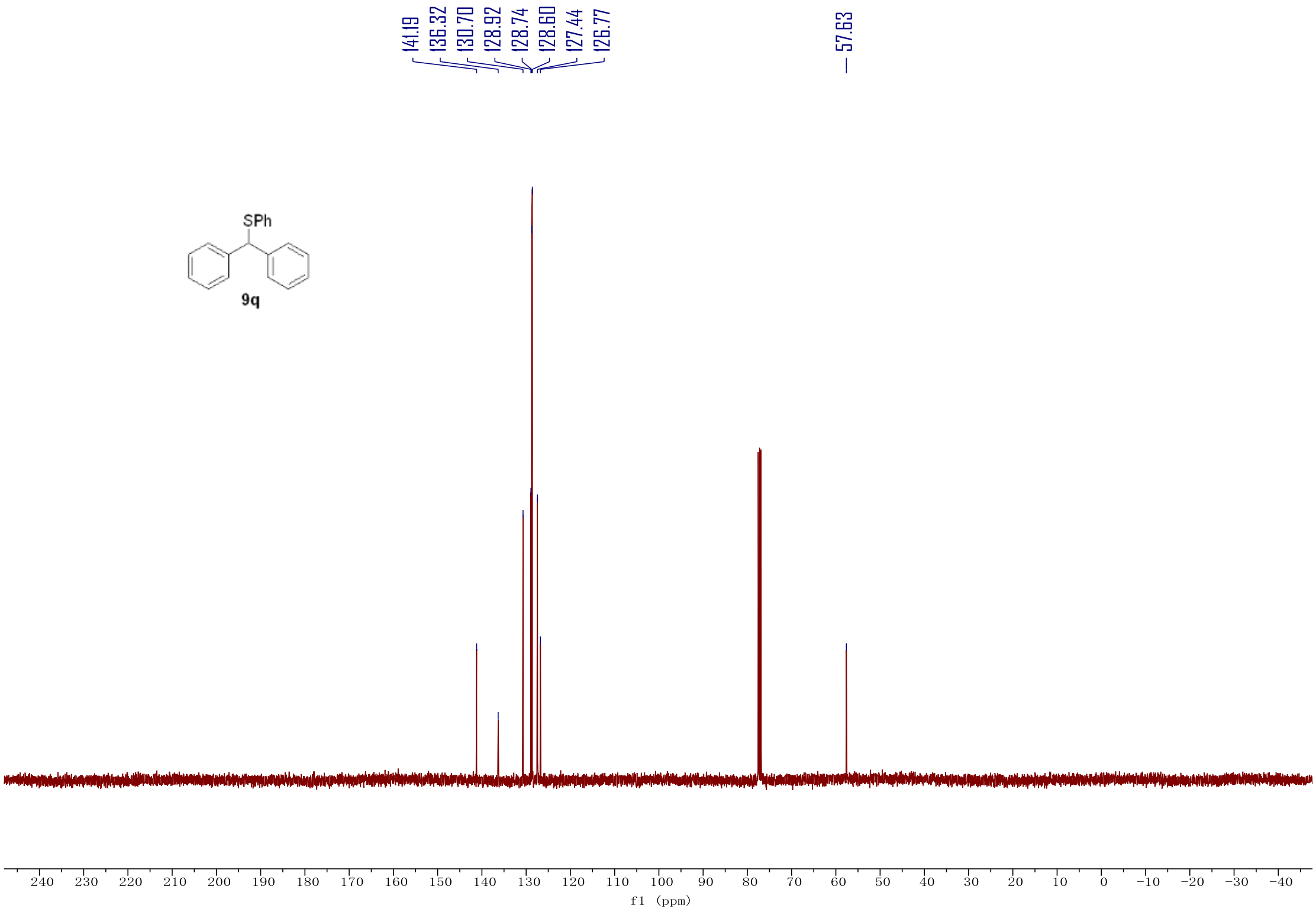

^{1}H NMR Spectrum of **(*Z*)-11d** (400 MHz, $CDCl_3$)

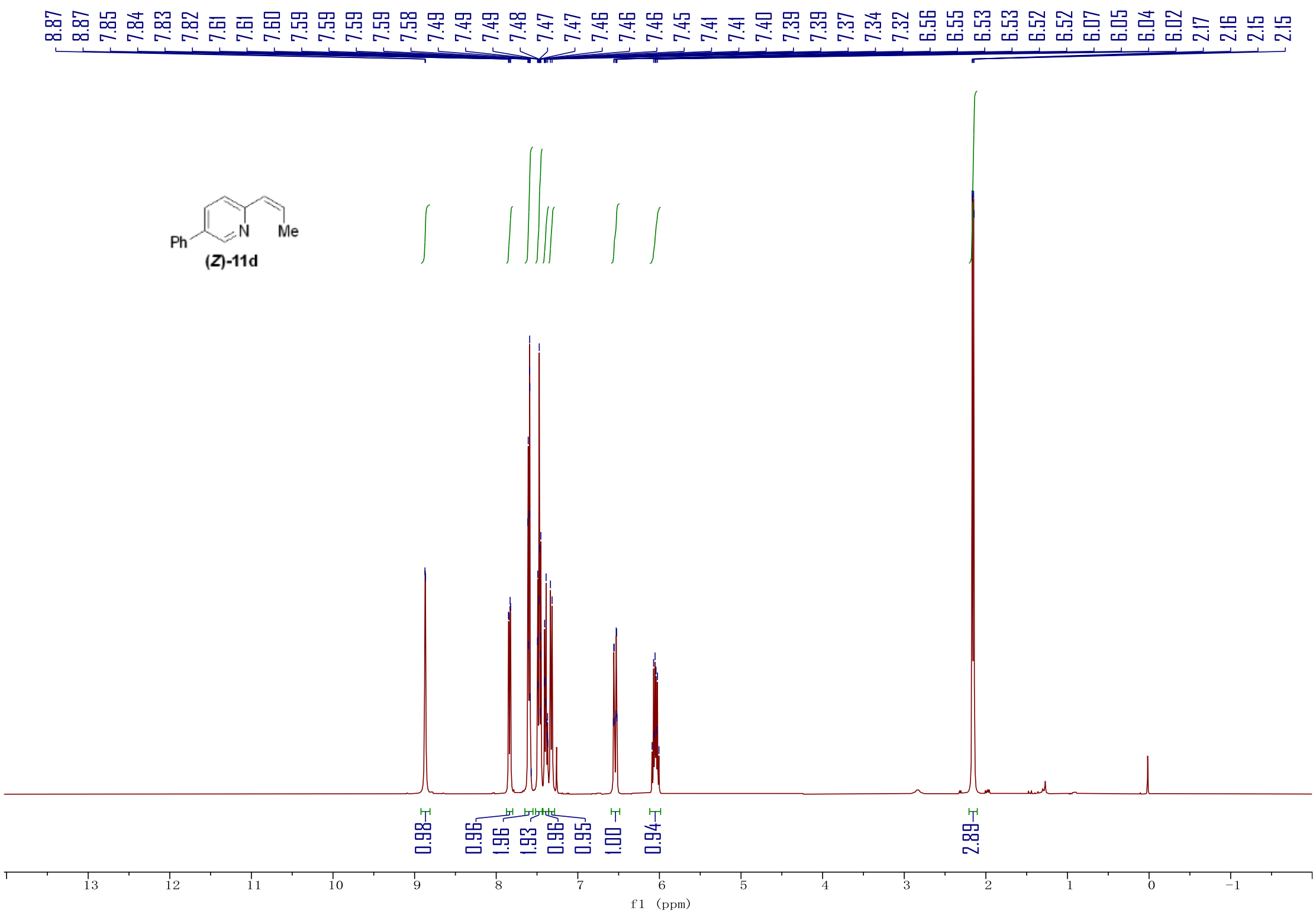

^{13}C NMR Spectrum of ***(Z)*-11d** (101 MHz, $CDCl_3$)

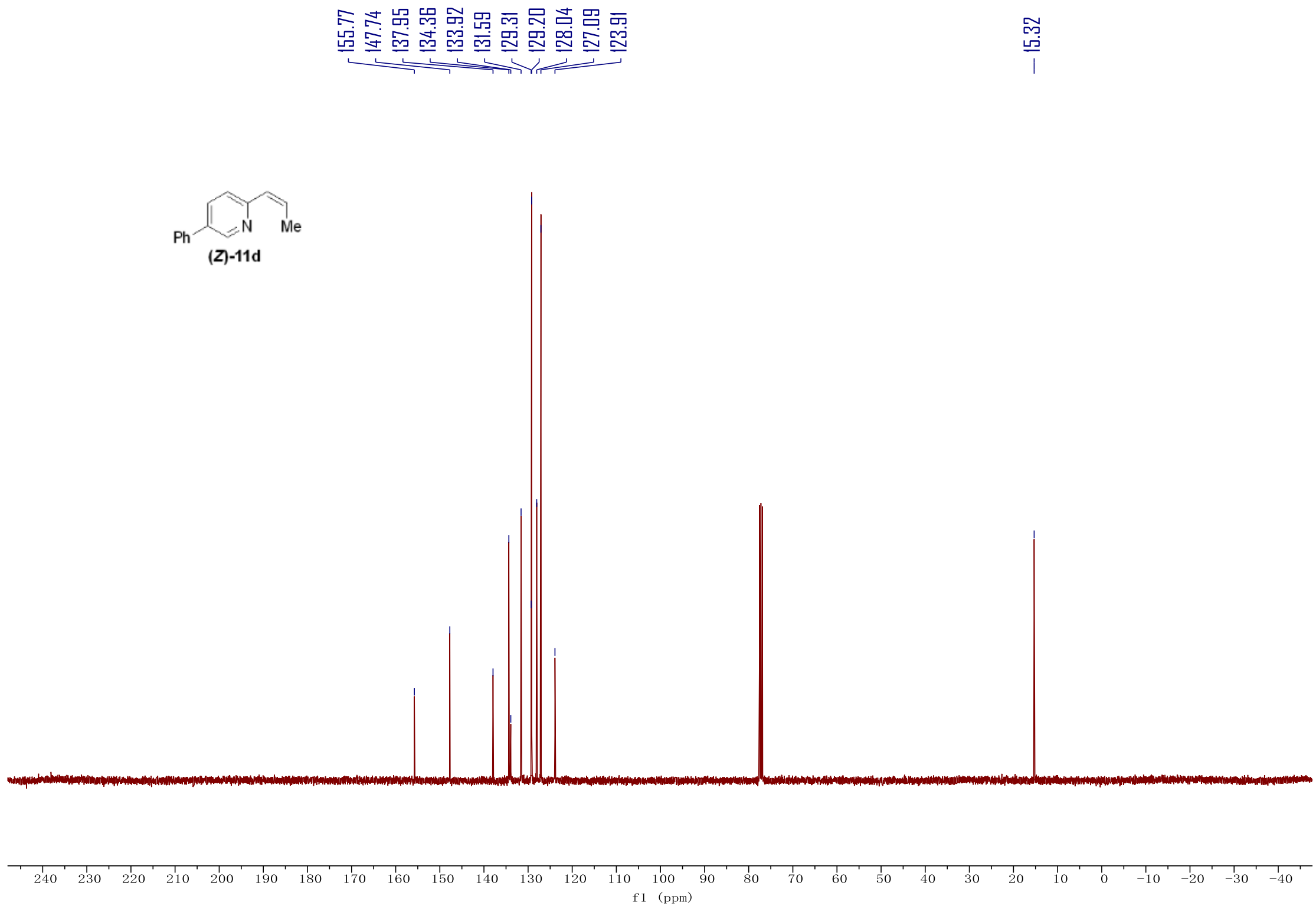

^{1}H NMR Spectrum of ***(E)*-11d** (400 MHz, $CDCl_3$)

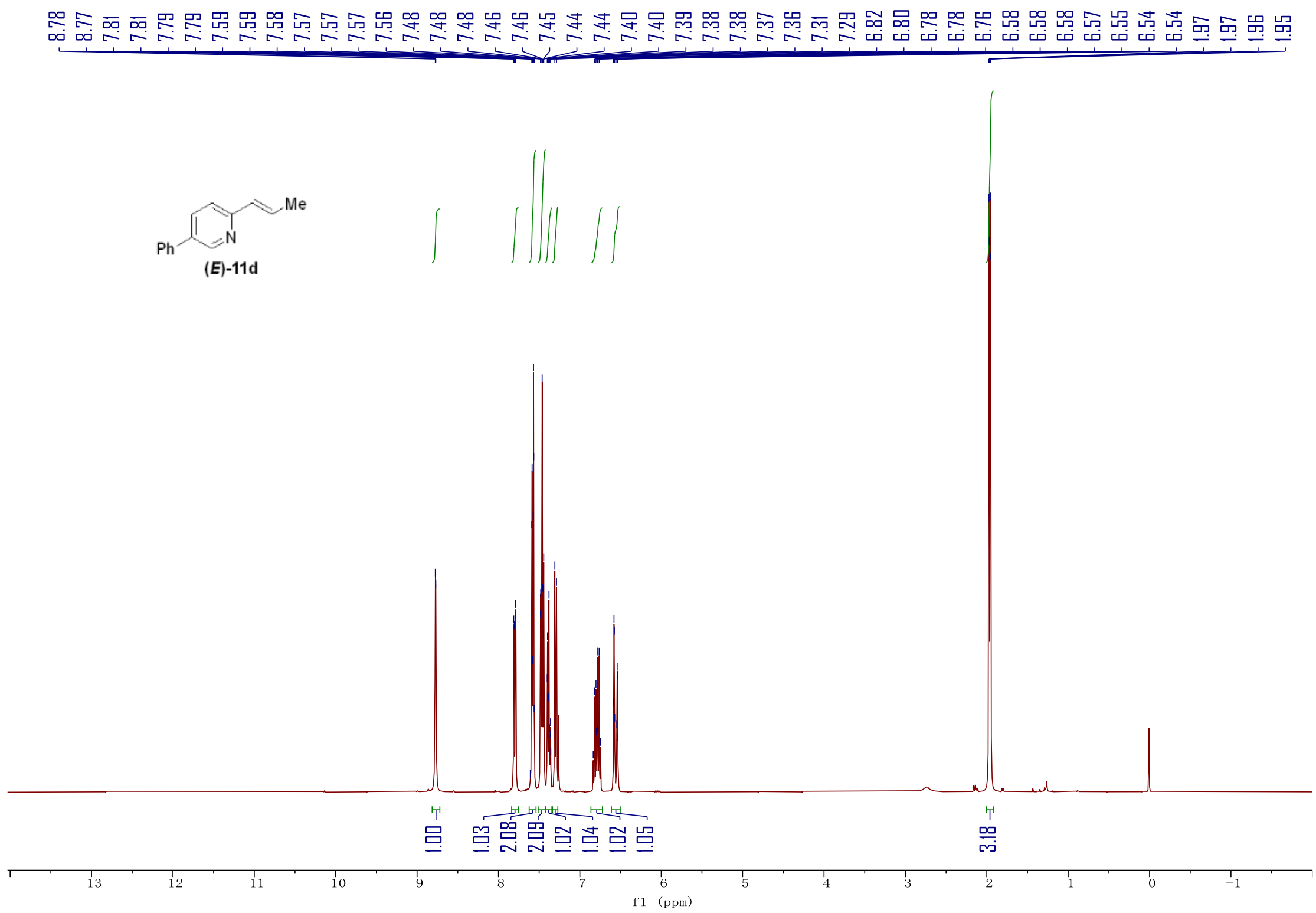

^{13}C NMR Spectrum of ***(E)*-11d** (101 MHz, $CDCl_3$)

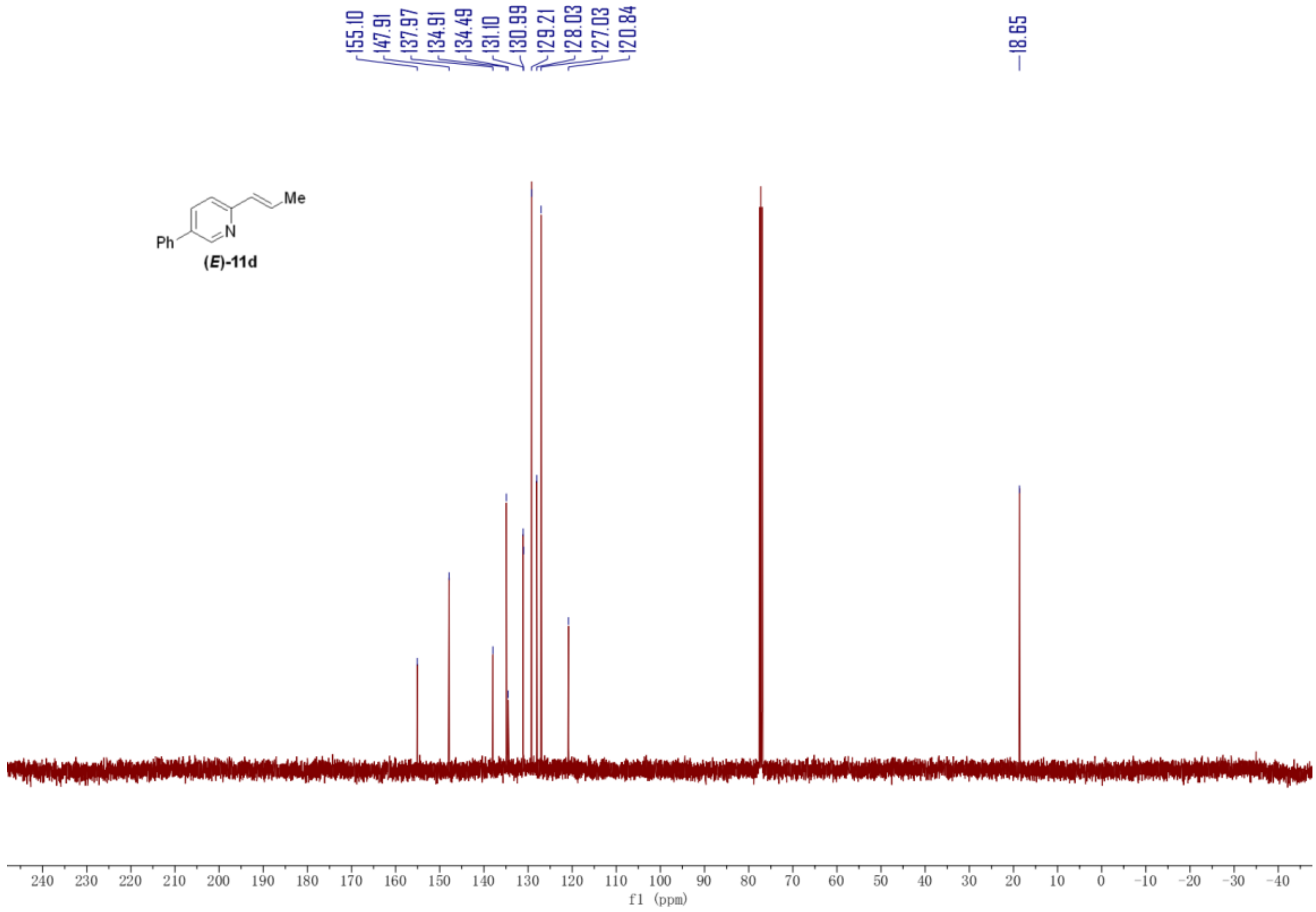

^{1}H NMR Spectrum of **11f** (400 MHz, $CDCl_3$)

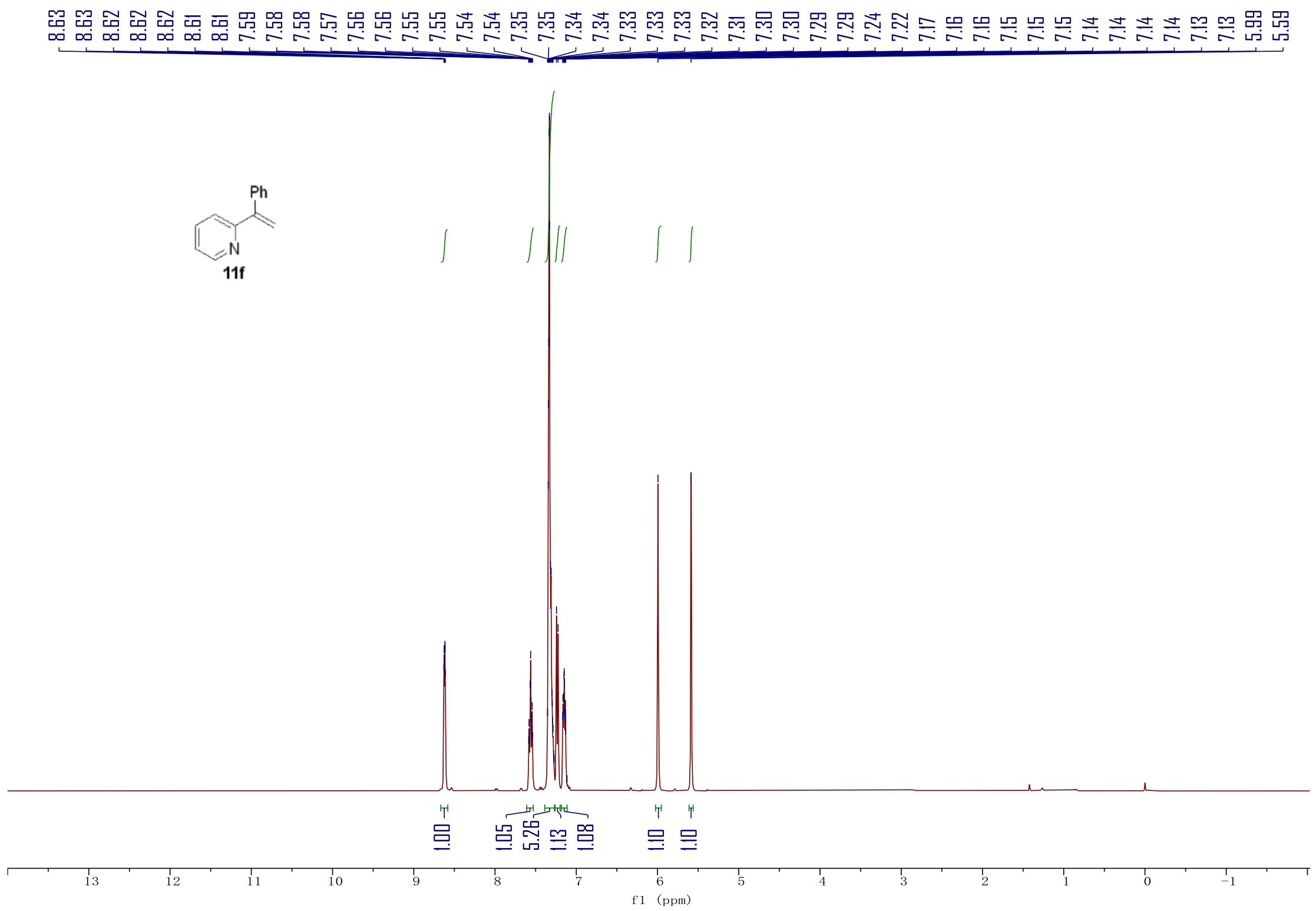

^{13}C NMR Spectrum of **11f** (101 MHz, $CDCl_3$)

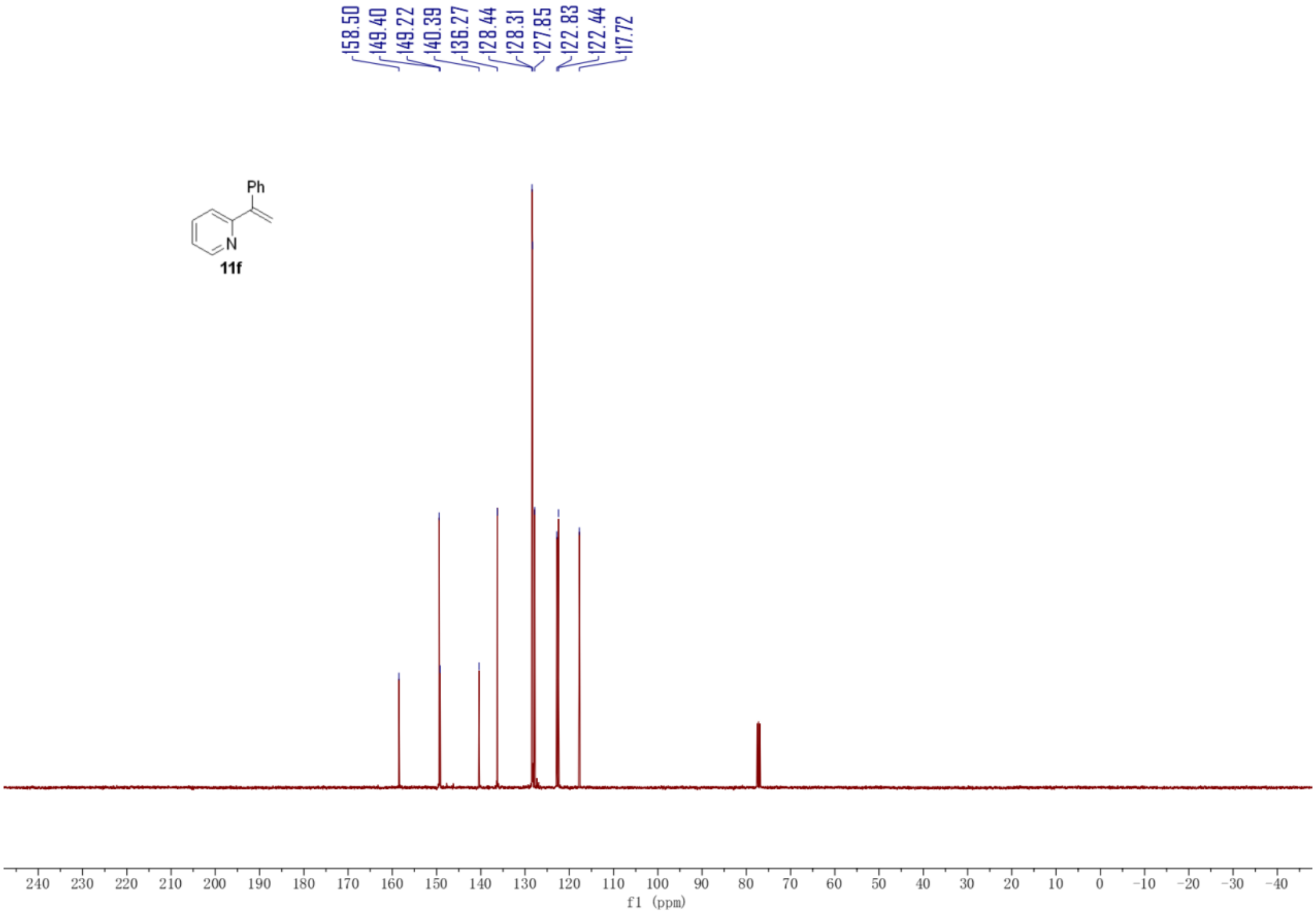

^{1}H NMR Spectrum of **11g** (400 MHz, $CDCl_3$)

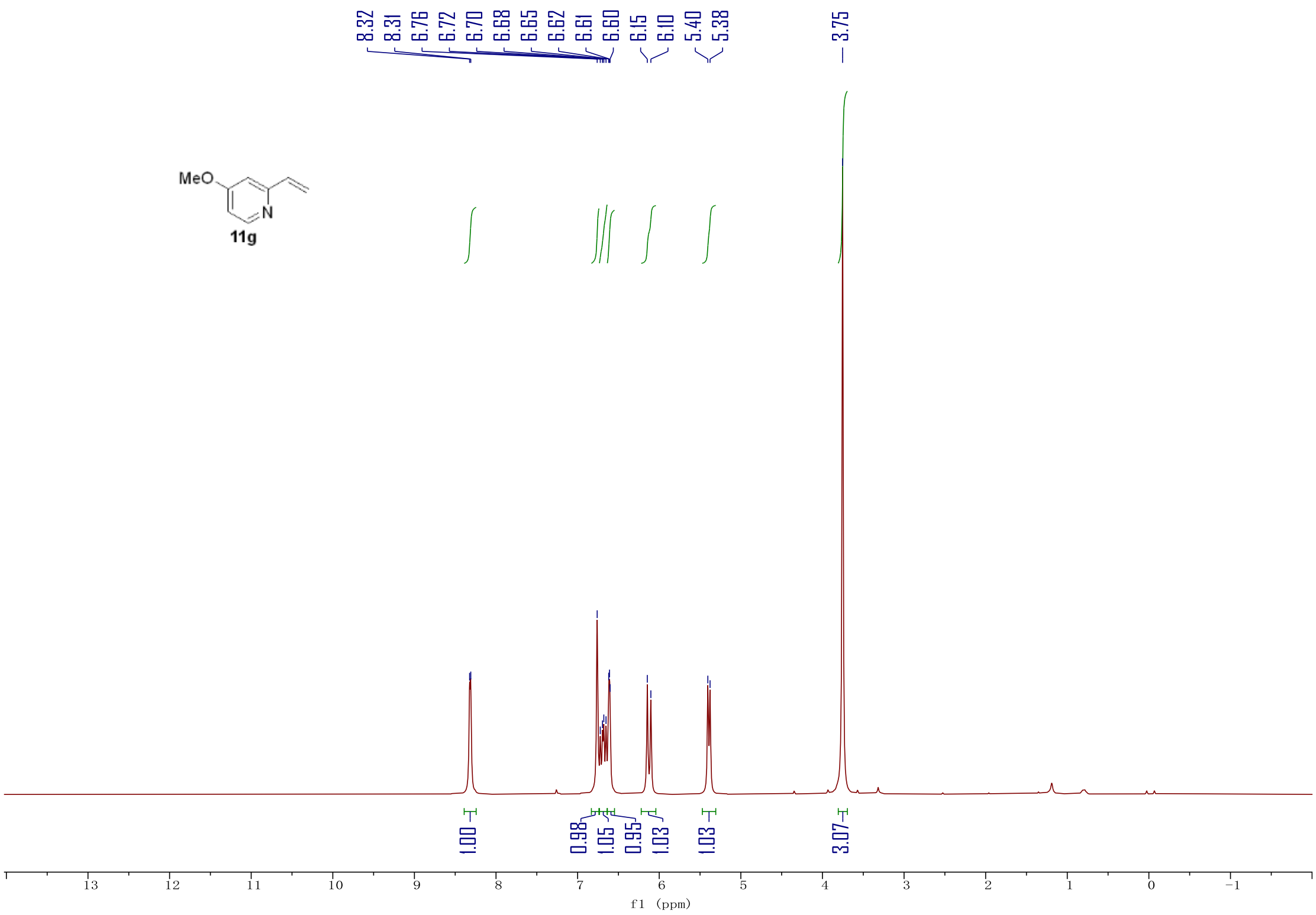

^{13}C NMR Spectrum of **11g** (101 MHz, $CDCl_3$)

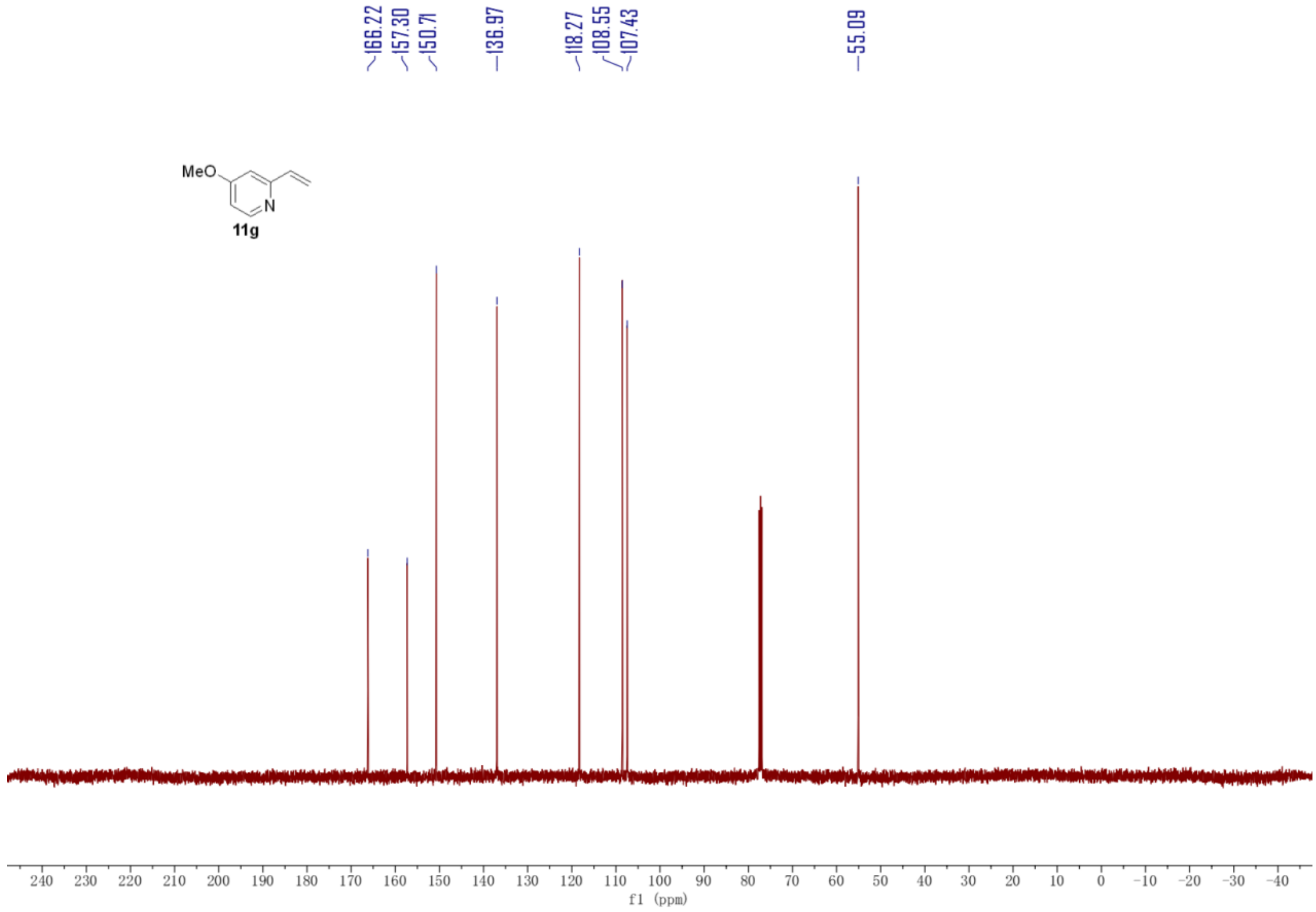

^{1}H NMR Spectrum of **11h** (400 MHz, $CDCl_3$)

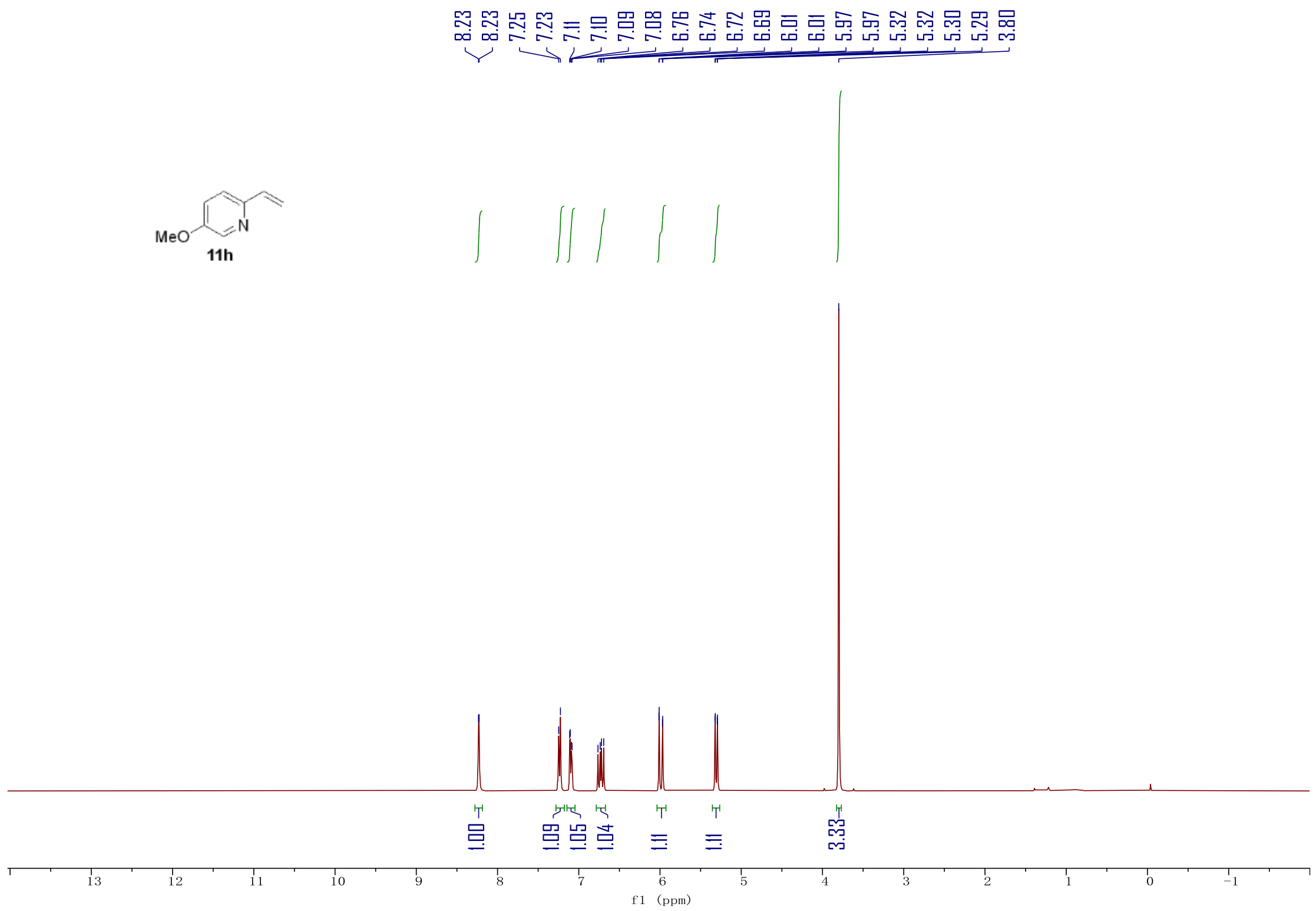

^{13}C NMR Spectrum of **11h** (101 MHz, $CDCl_3$)

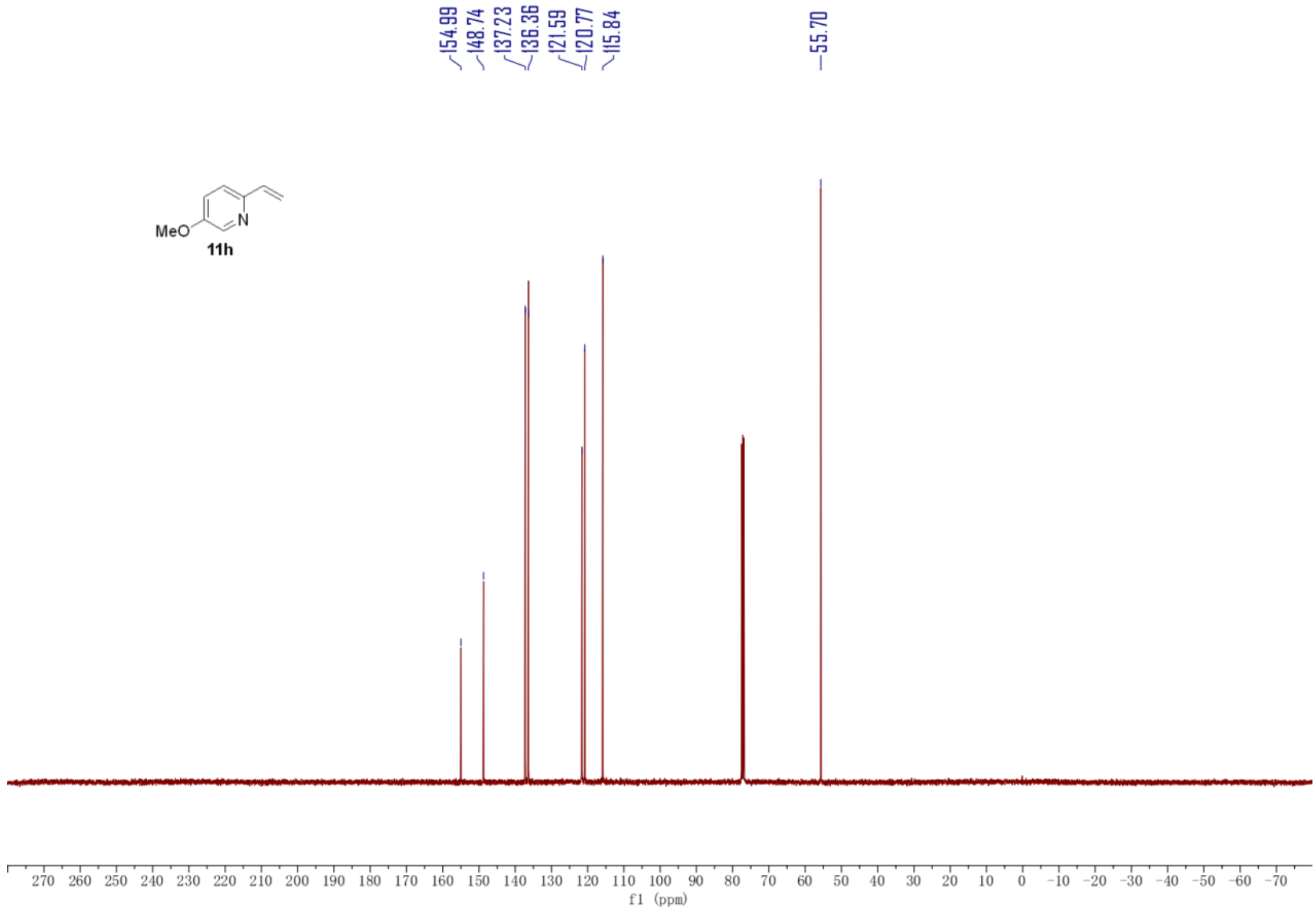

^{1}H NMR Spectrum of **11i** (400 MHz, $CDCl_3$)

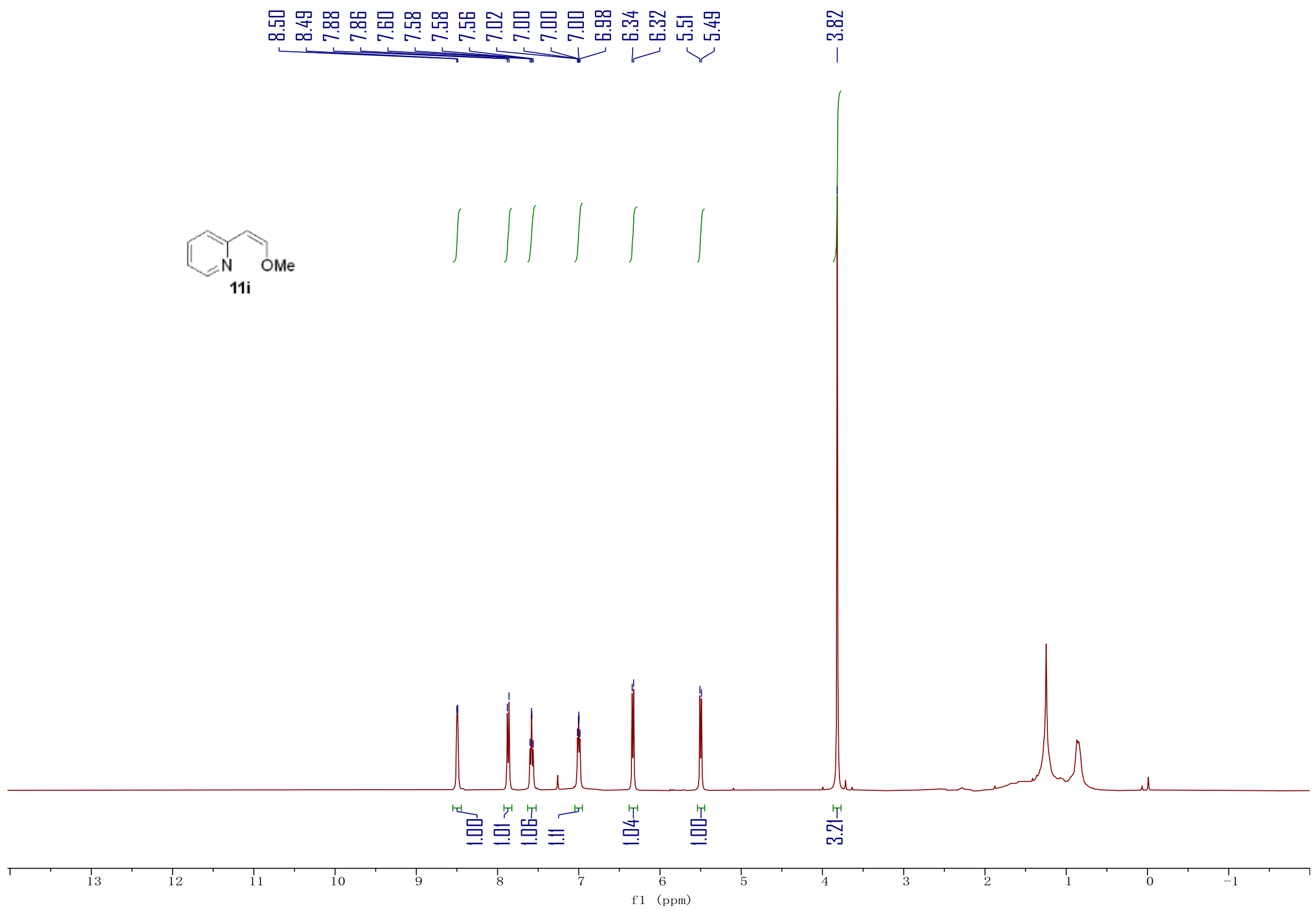

^{13}C NMR Spectrum of **11i** (101 MHz, $CDCl_3$)

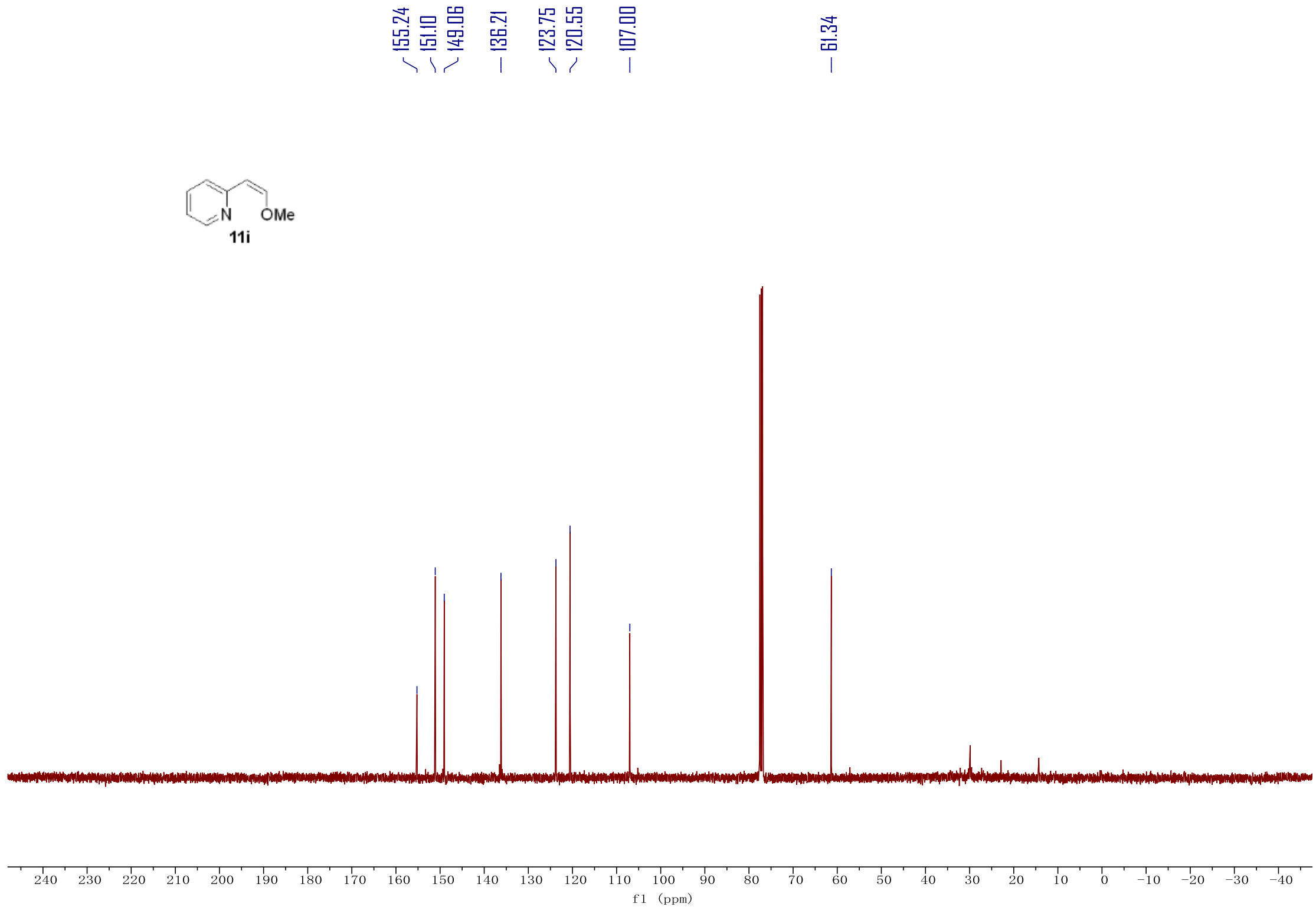

^{1}H NMR Spectrum of **11l** (400 MHz, $CDCl_3$)

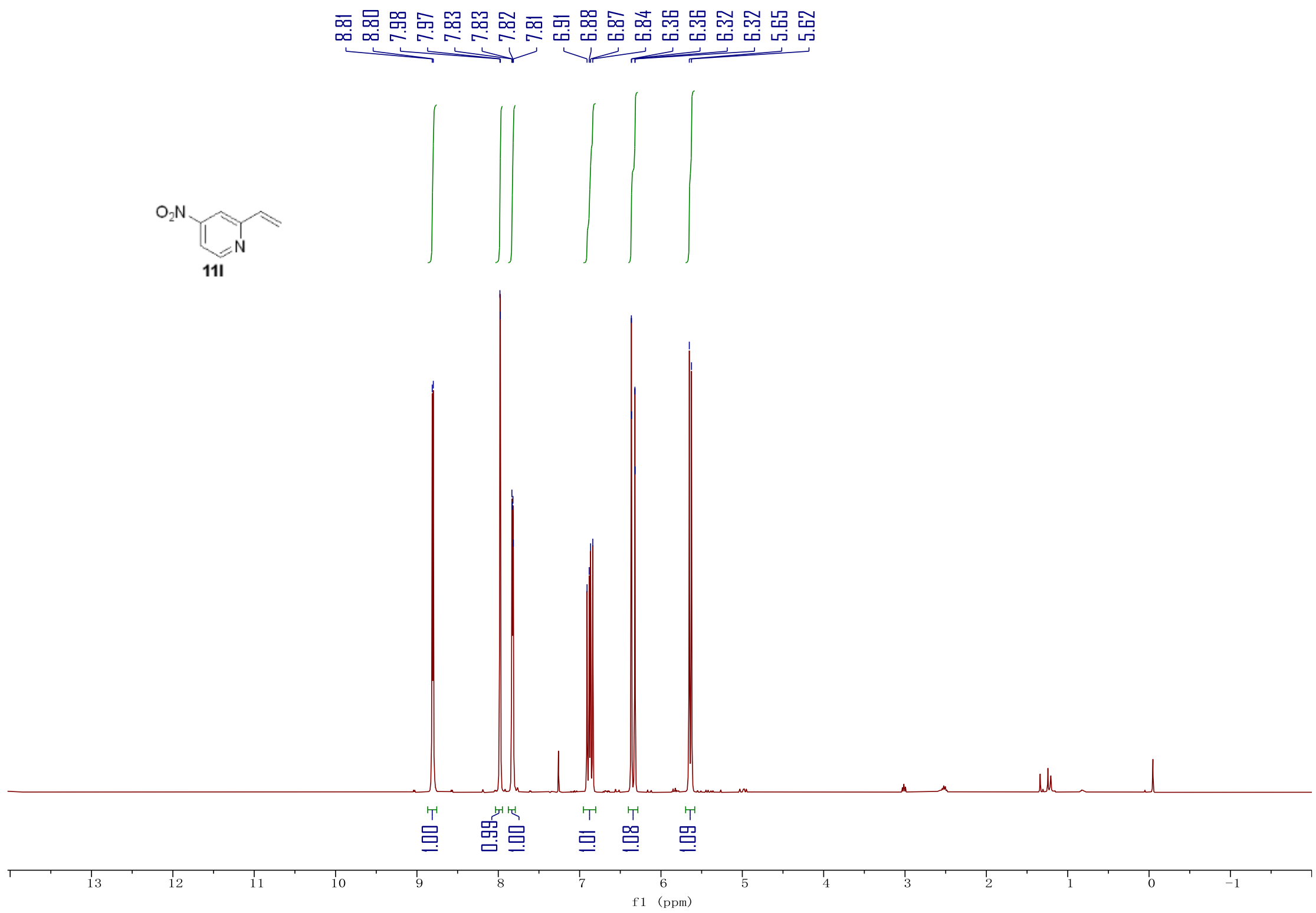

^{13}C NMR Spectrum of **11l** (101 MHz, $CDCl_3$)

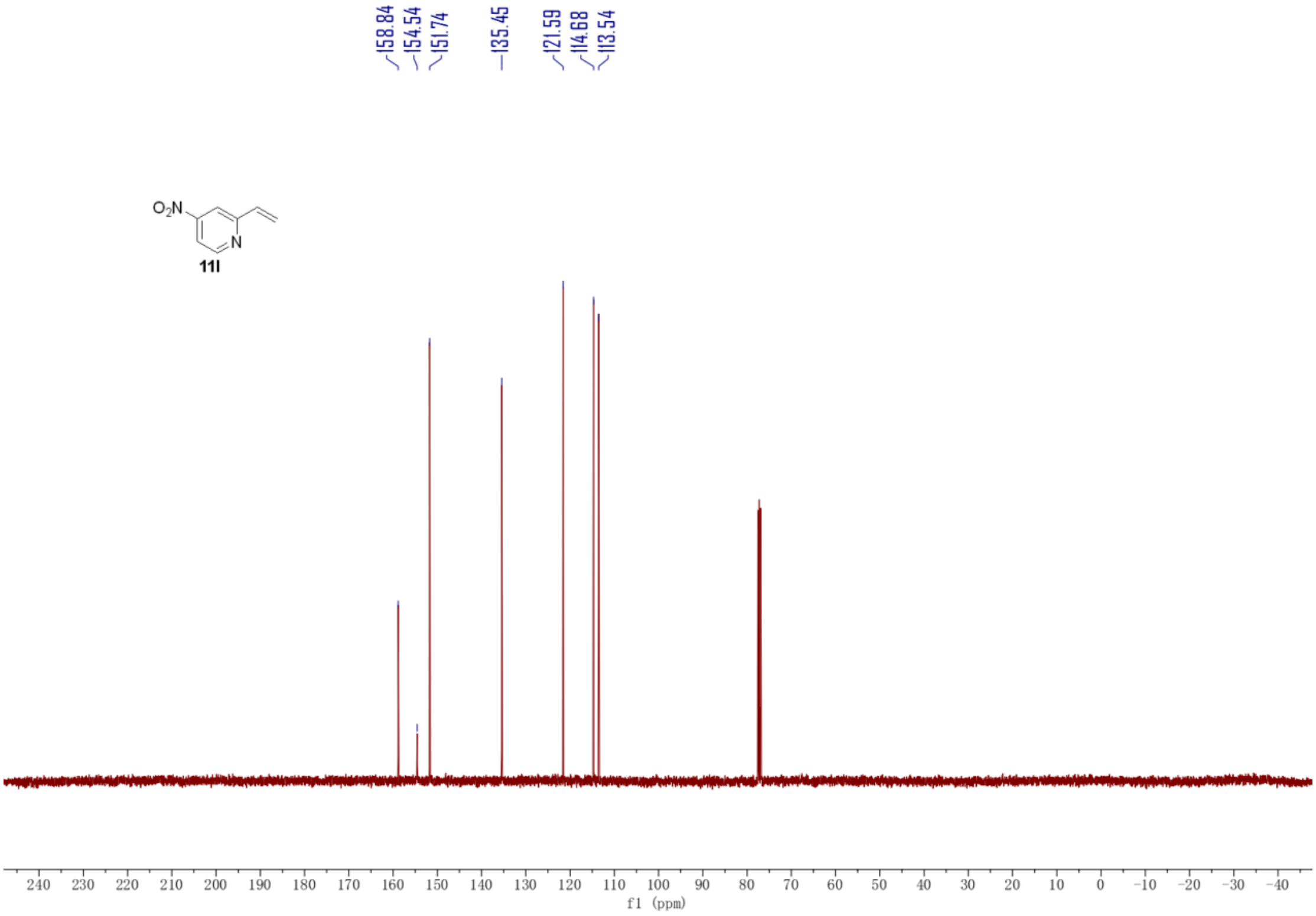

^{1}H NMR Spectrum of **11m** (400 MHz, $CDCl_3$)

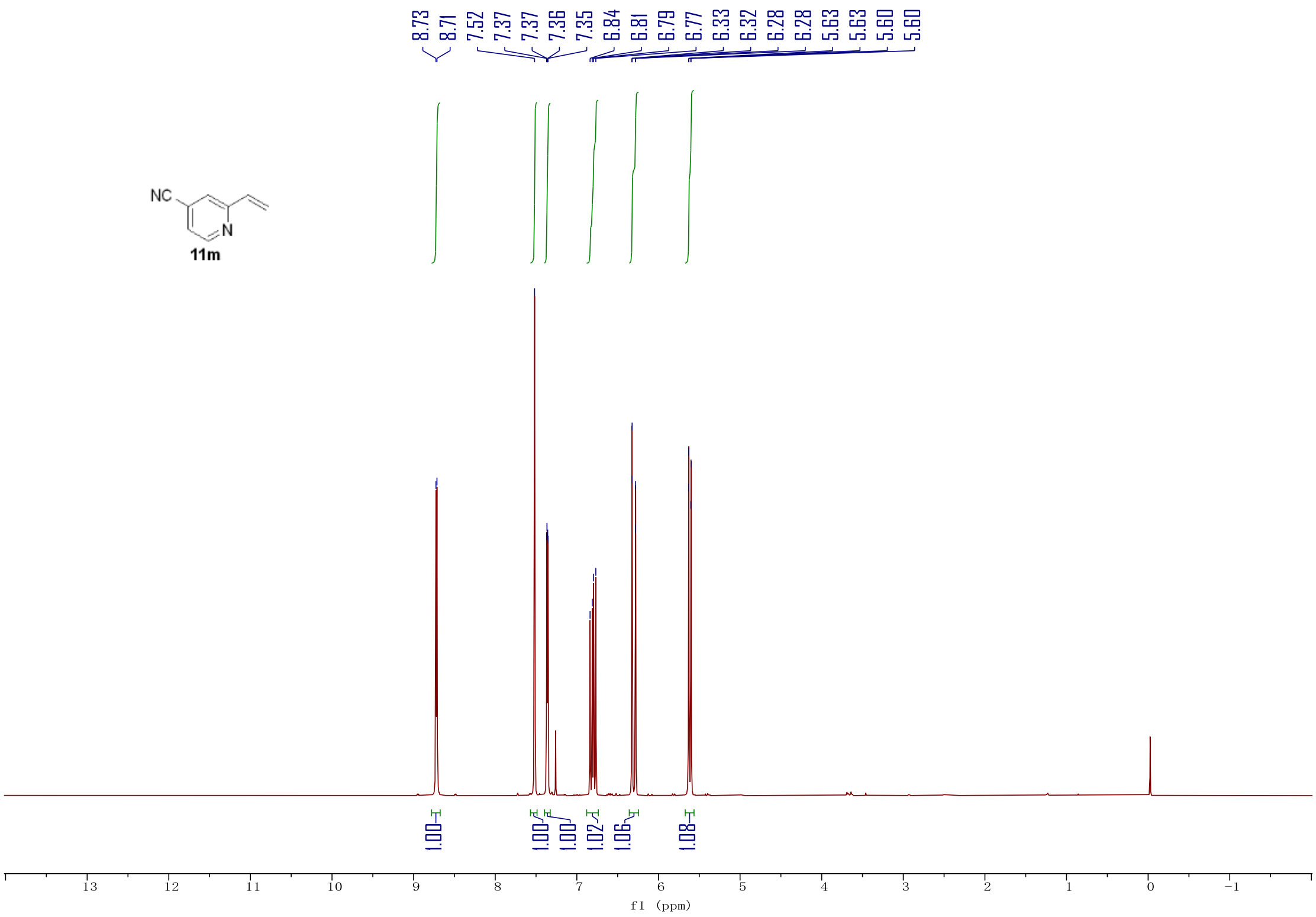

^{13}**C NMR** Spectrum of **11m** (101 MHz, $CDCl_3$)

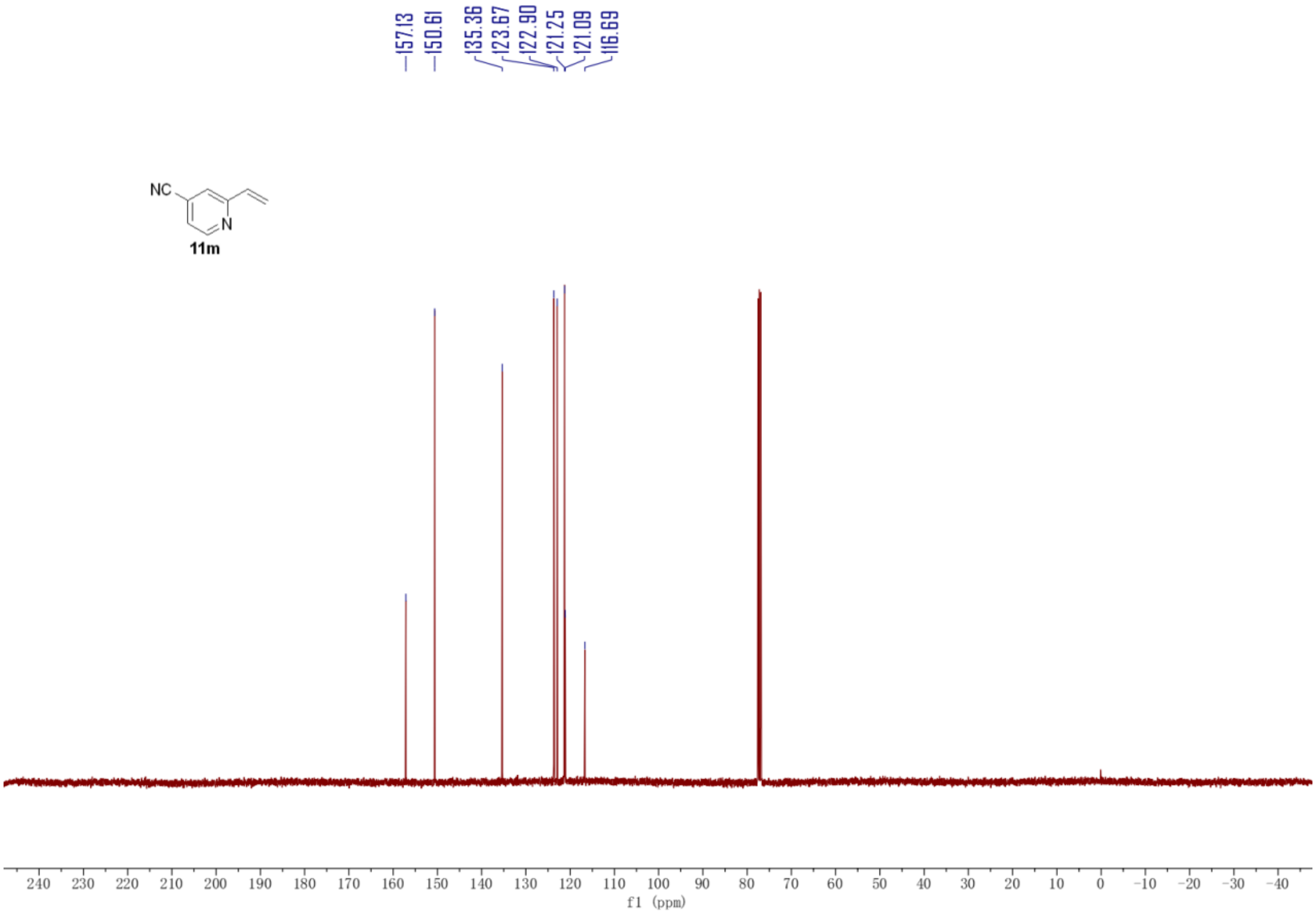

^{1}**H NMR** Spectrum of **11n** (400 MHz, $CDCl_3$)

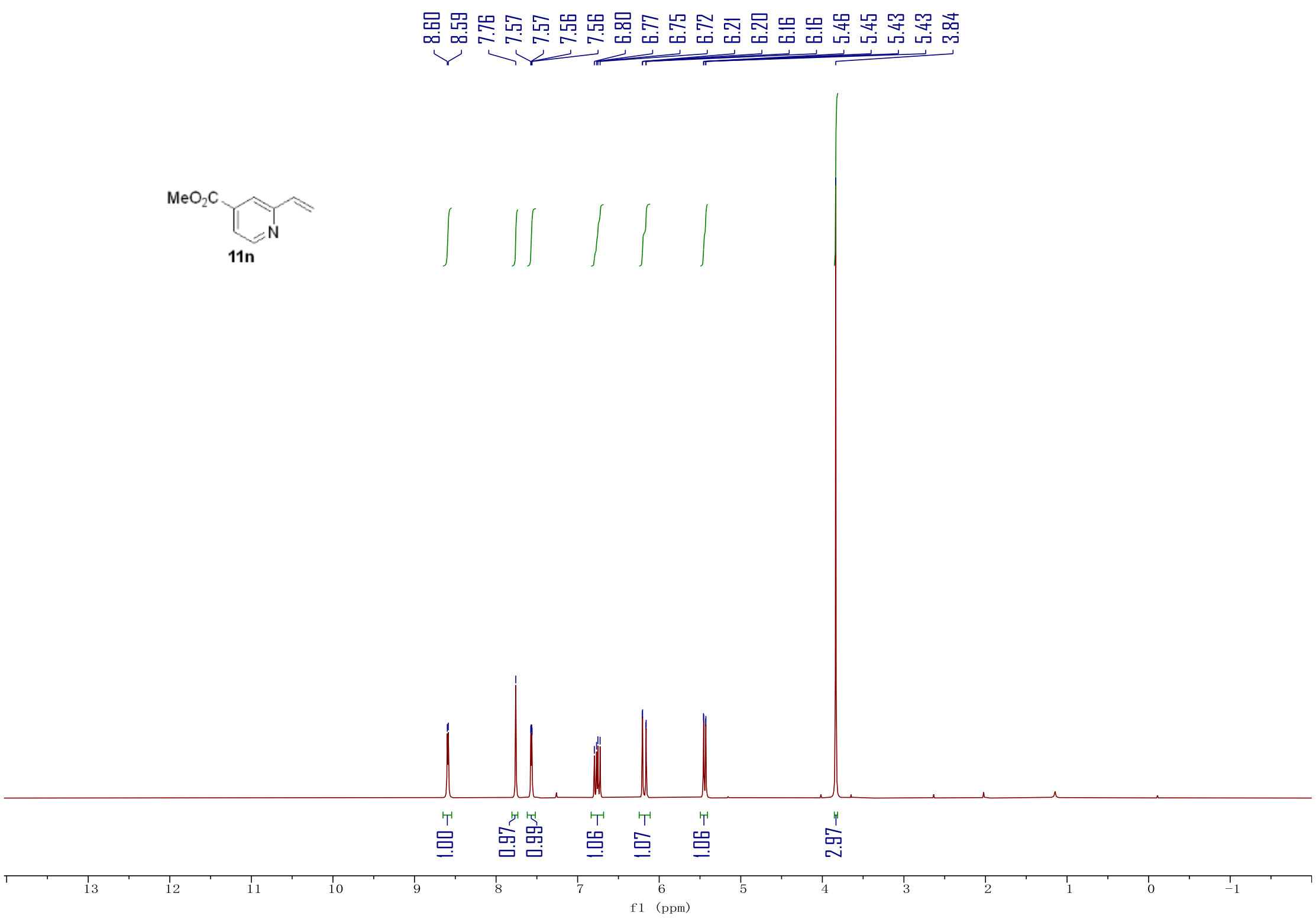

^{13}C NMR Spectrum of **11n** (101 MHz, $CDCl_3$)

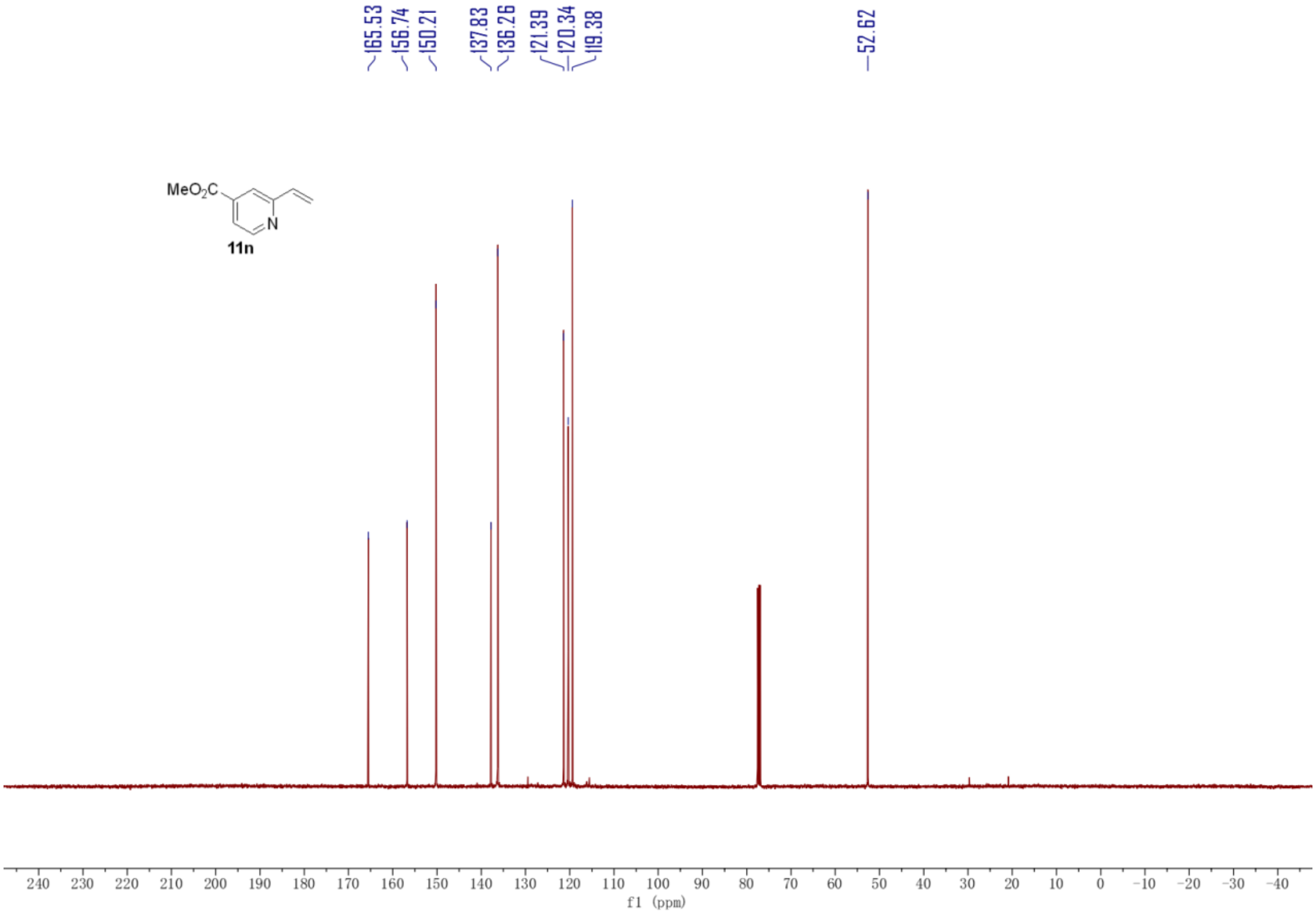

^{1}H NMR Spectrum of **11p** (400 MHz, $CDCl_3$)

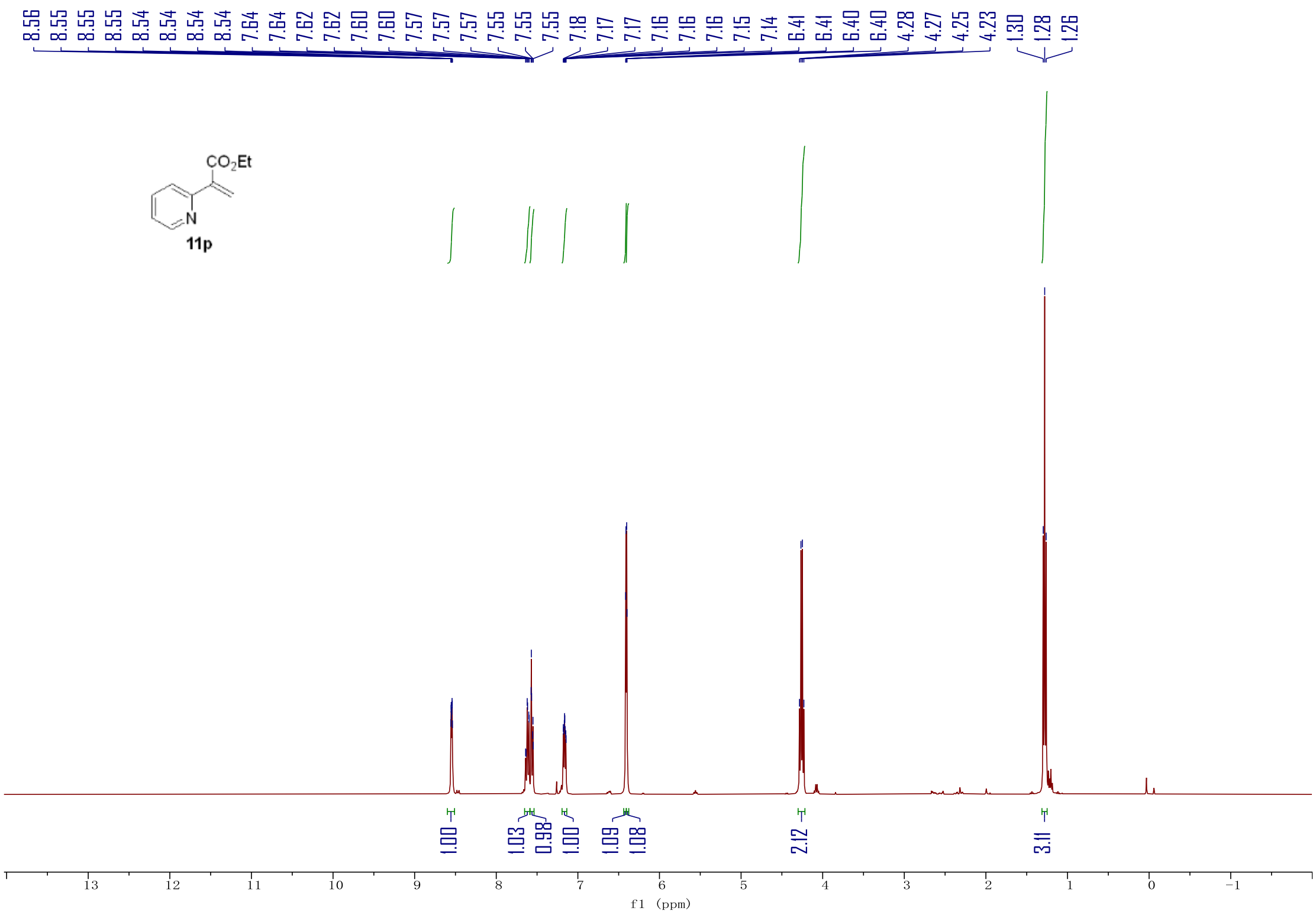

^{13}C NMR Spectrum of **11p** (101 MHz, $CDCl_3$)

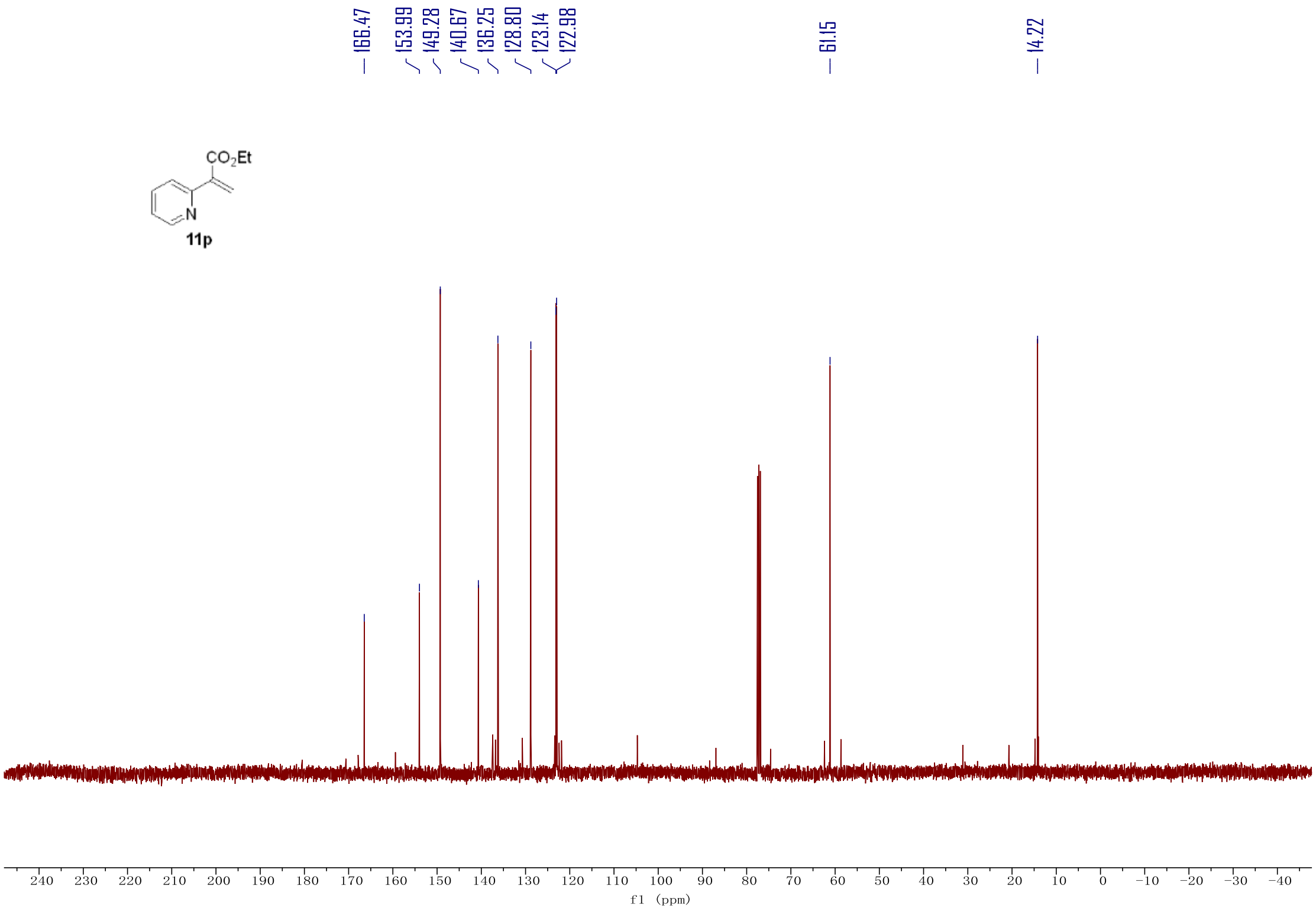

^{1}H NMR Spectrum of **11q** (400 MHz, $CDCl_3$)

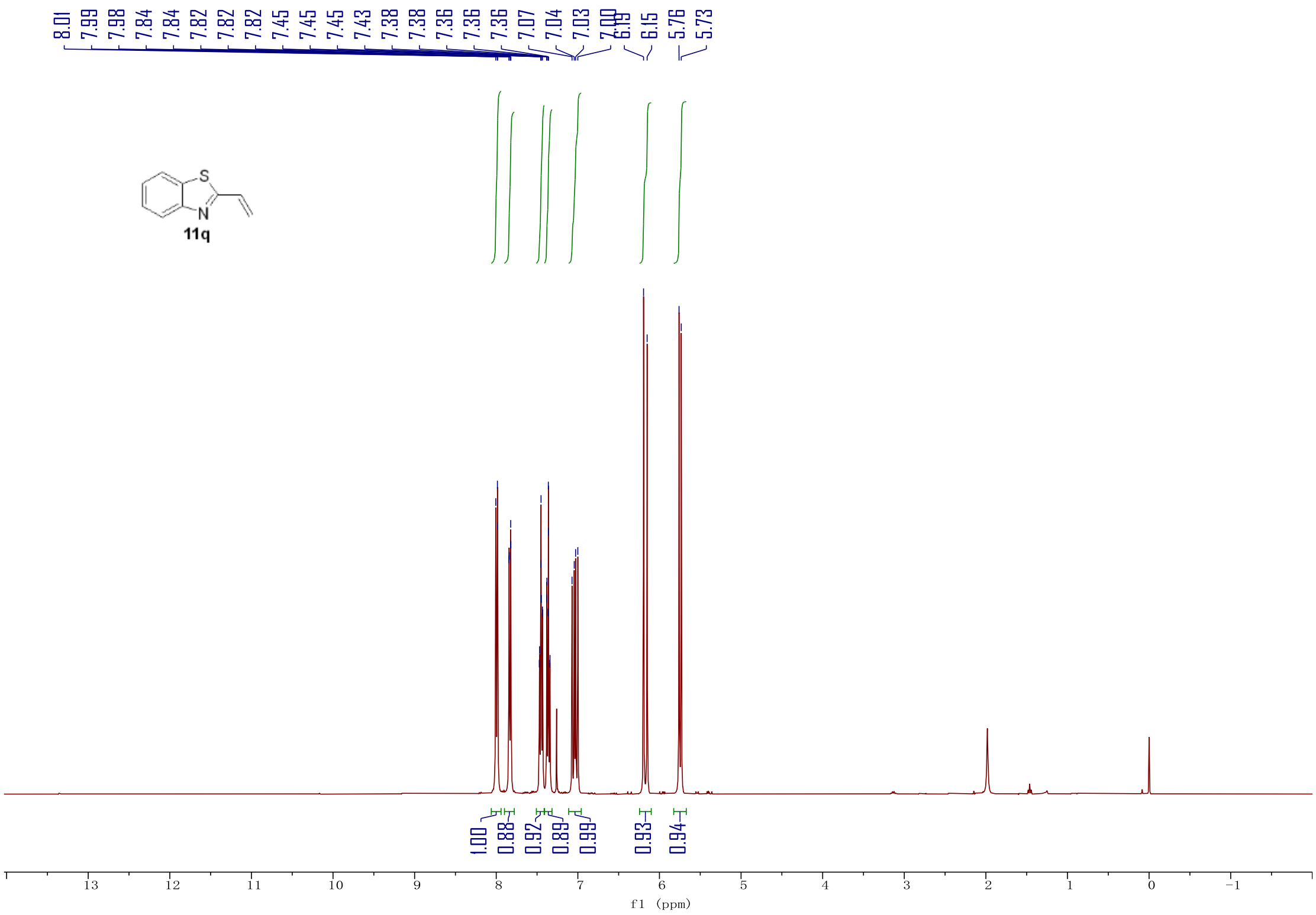

^{13}C NMR Spectrum of 11q (101 MHz, $CDCl_3$)

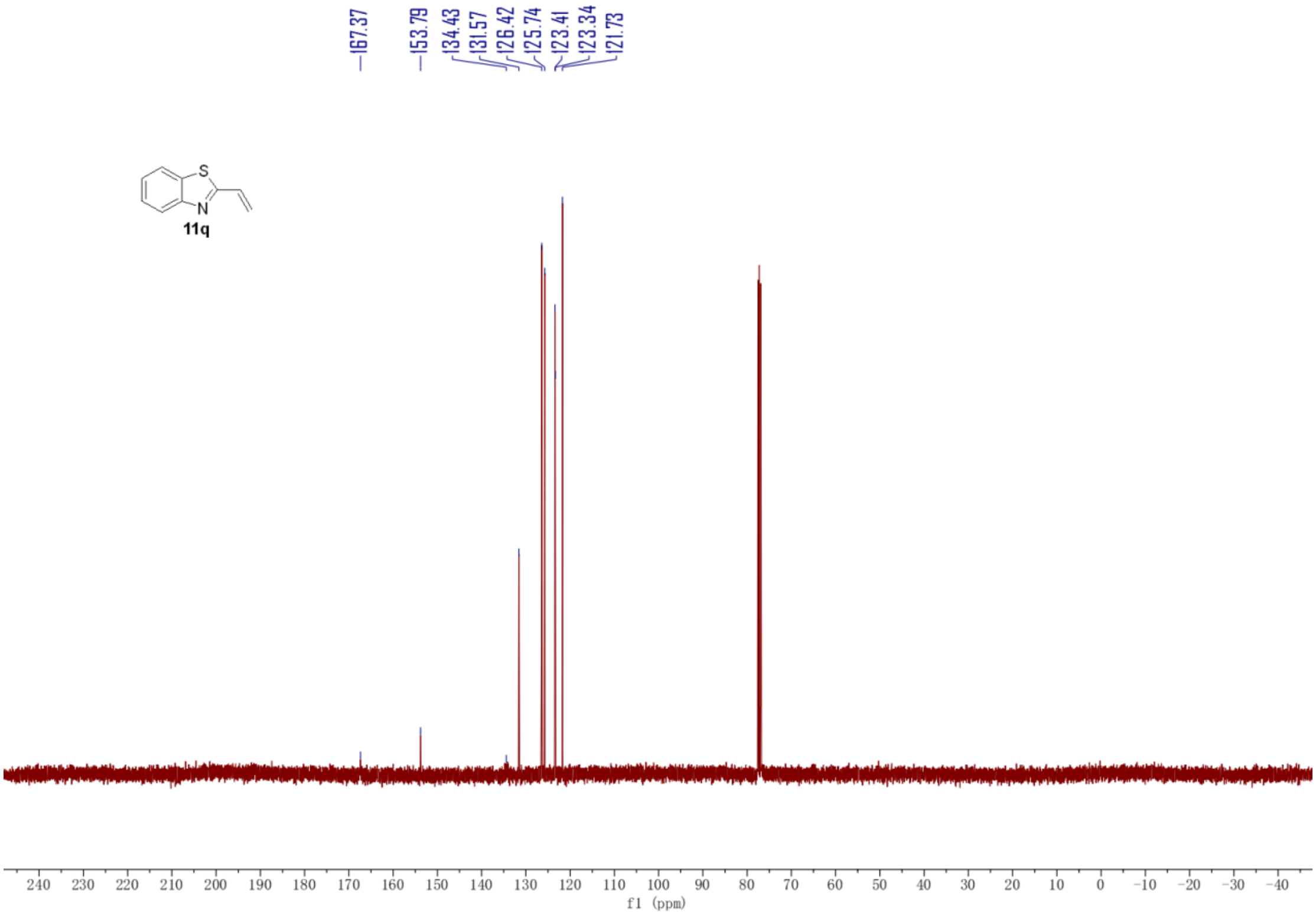

^{1}H NMR Spectrum of 11r (400 MHz, $CDCl_3$)

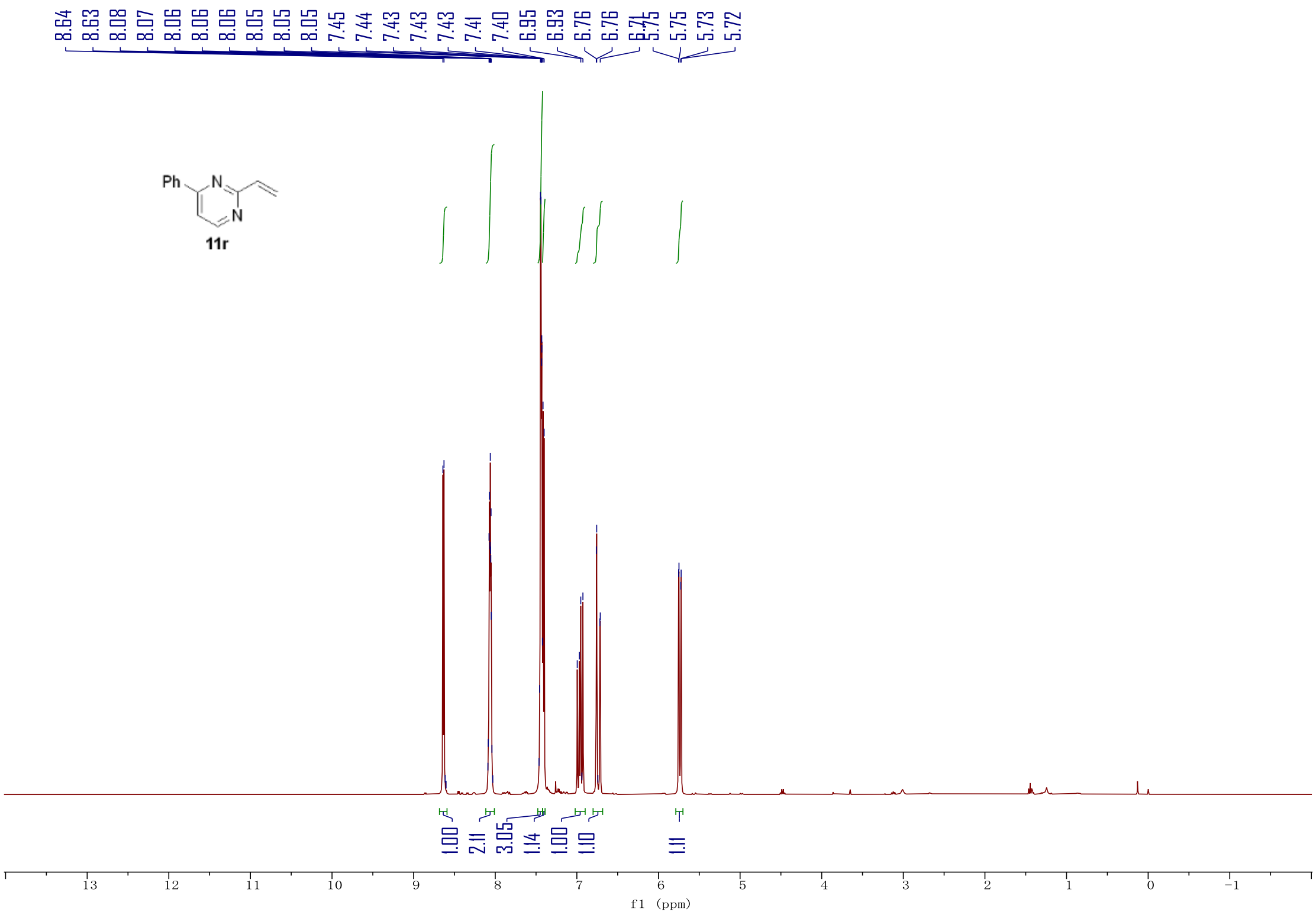

^{13}C NMR Spectrum of **11r** (101 MHz, $CDCl_3$)

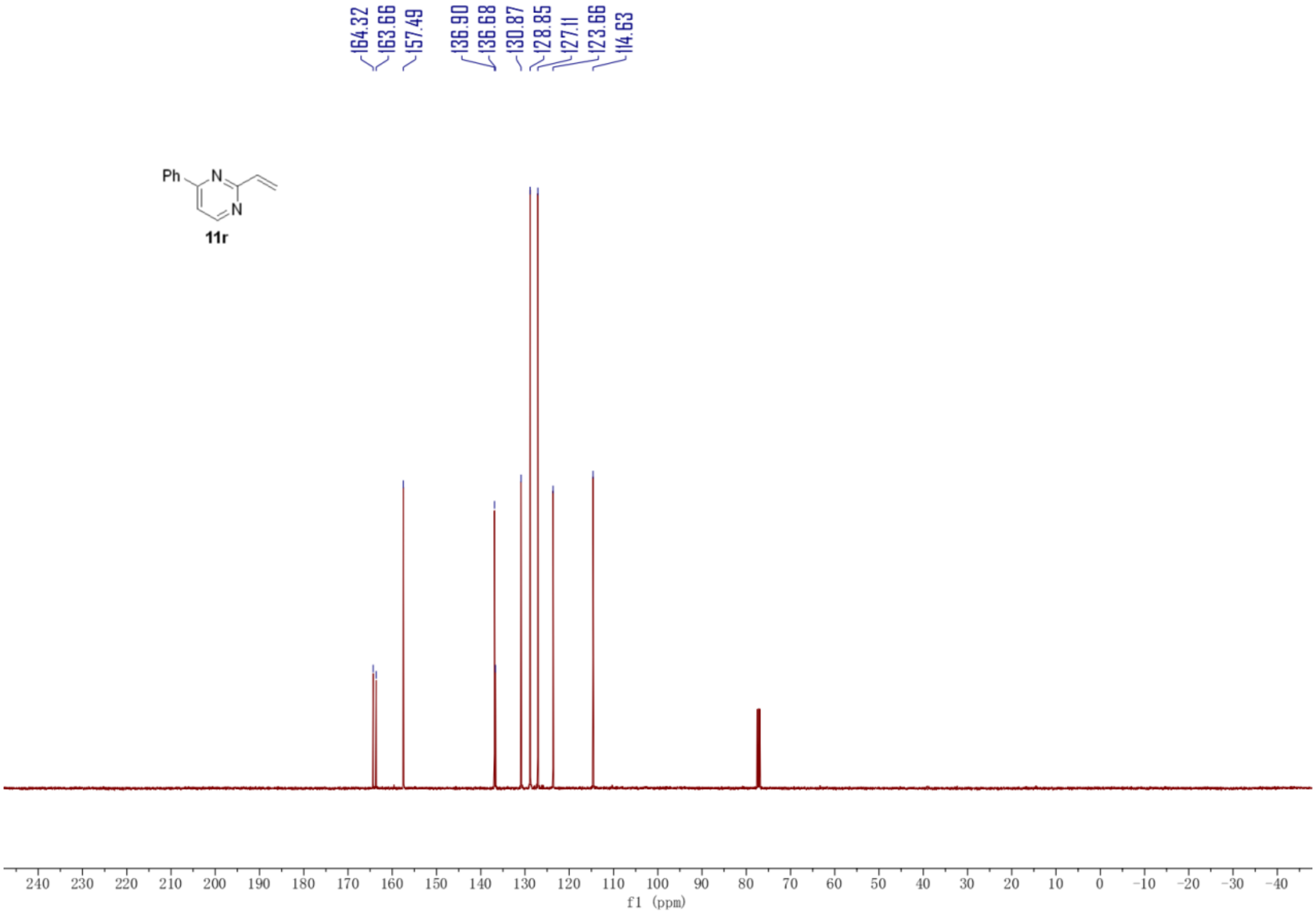

^{1}H NMR Spectrum of **11t** (400 MHz, $CDCl_3$)

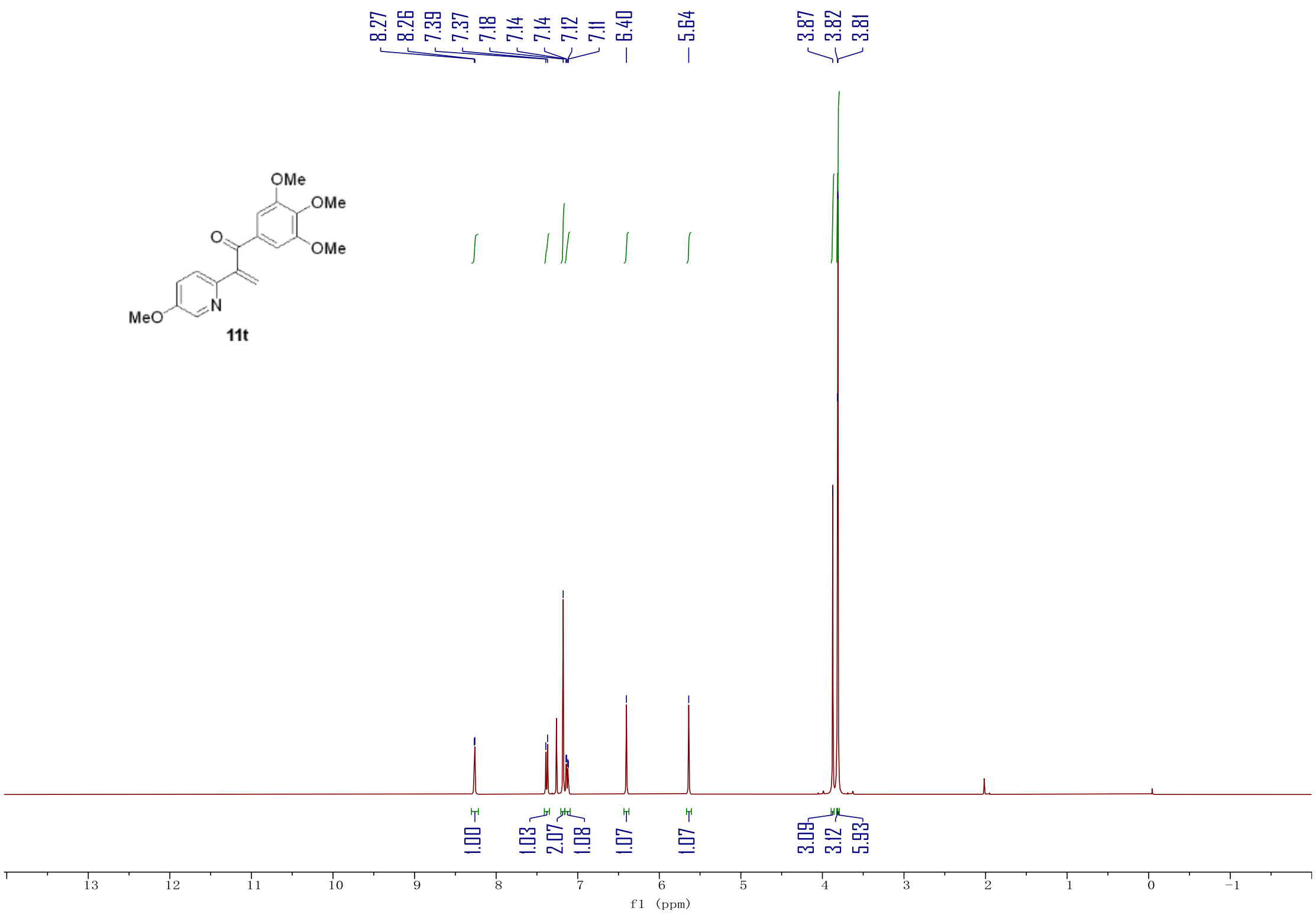

^{13}C NMR Spectrum of **11t** (101 MHz, $CDCl_3$)

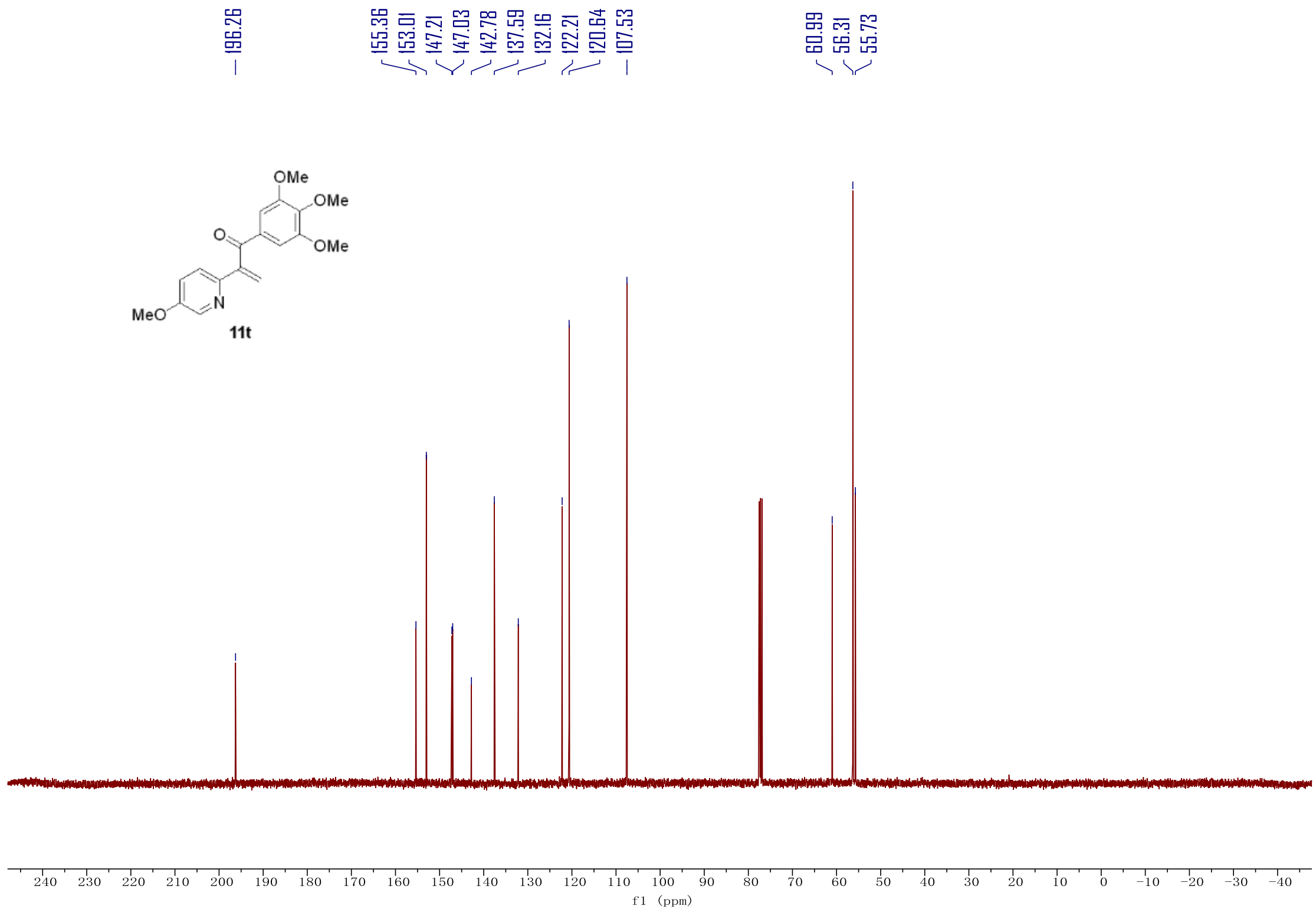

^{1}H NMR Spectrum of **2a** (400 MHz, $CDCl_3$)

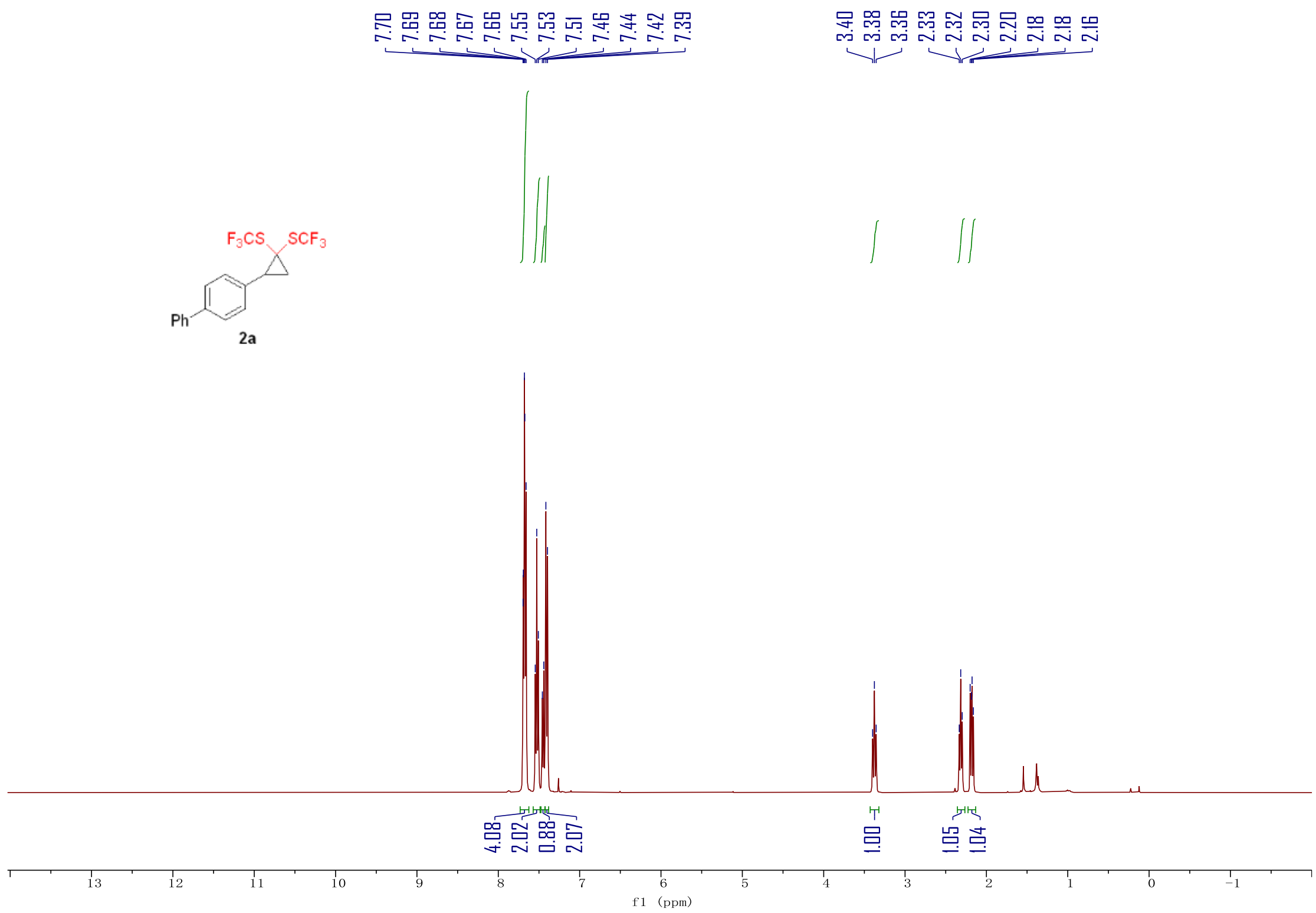

^{19}**F NMR** Spectrum of **2a** (376 MHz, $CDCl_3$)

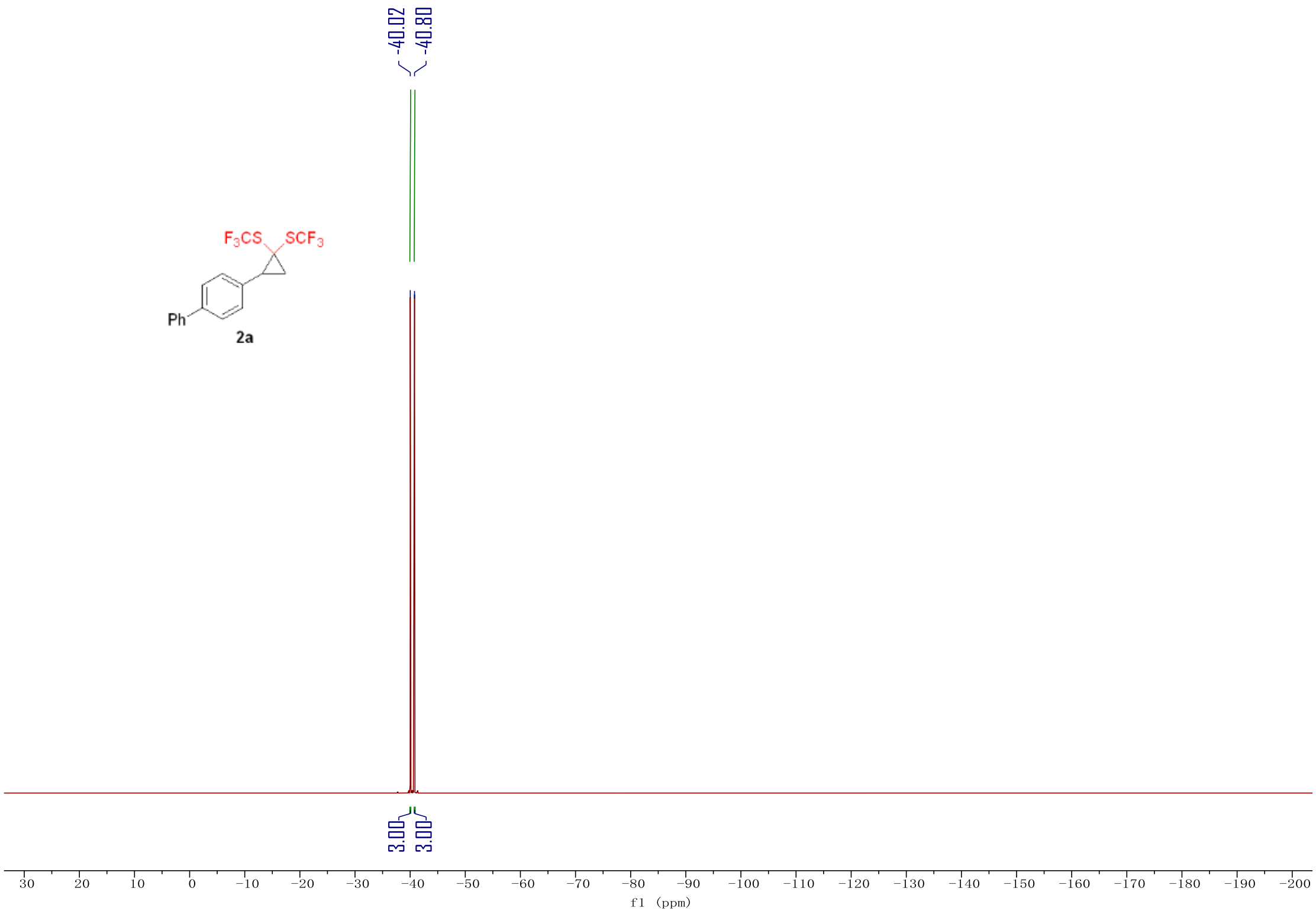

^{13}**C NMR** Spectrum of **2a** (101 MHz, $CDCl_3$)

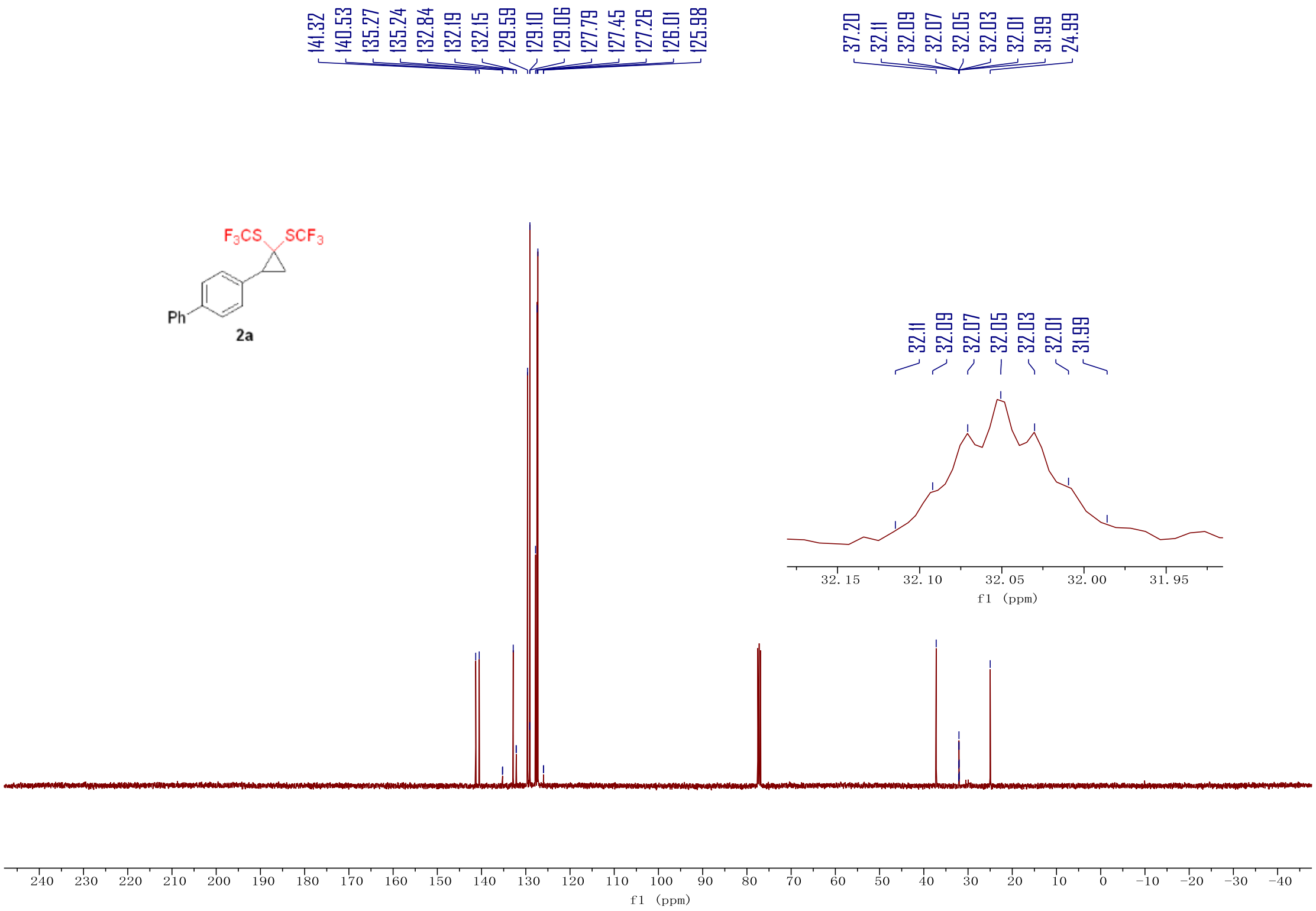

^{1}**H NMR** Spectrum of **2b** (400 MHz, $CDCl_3$)

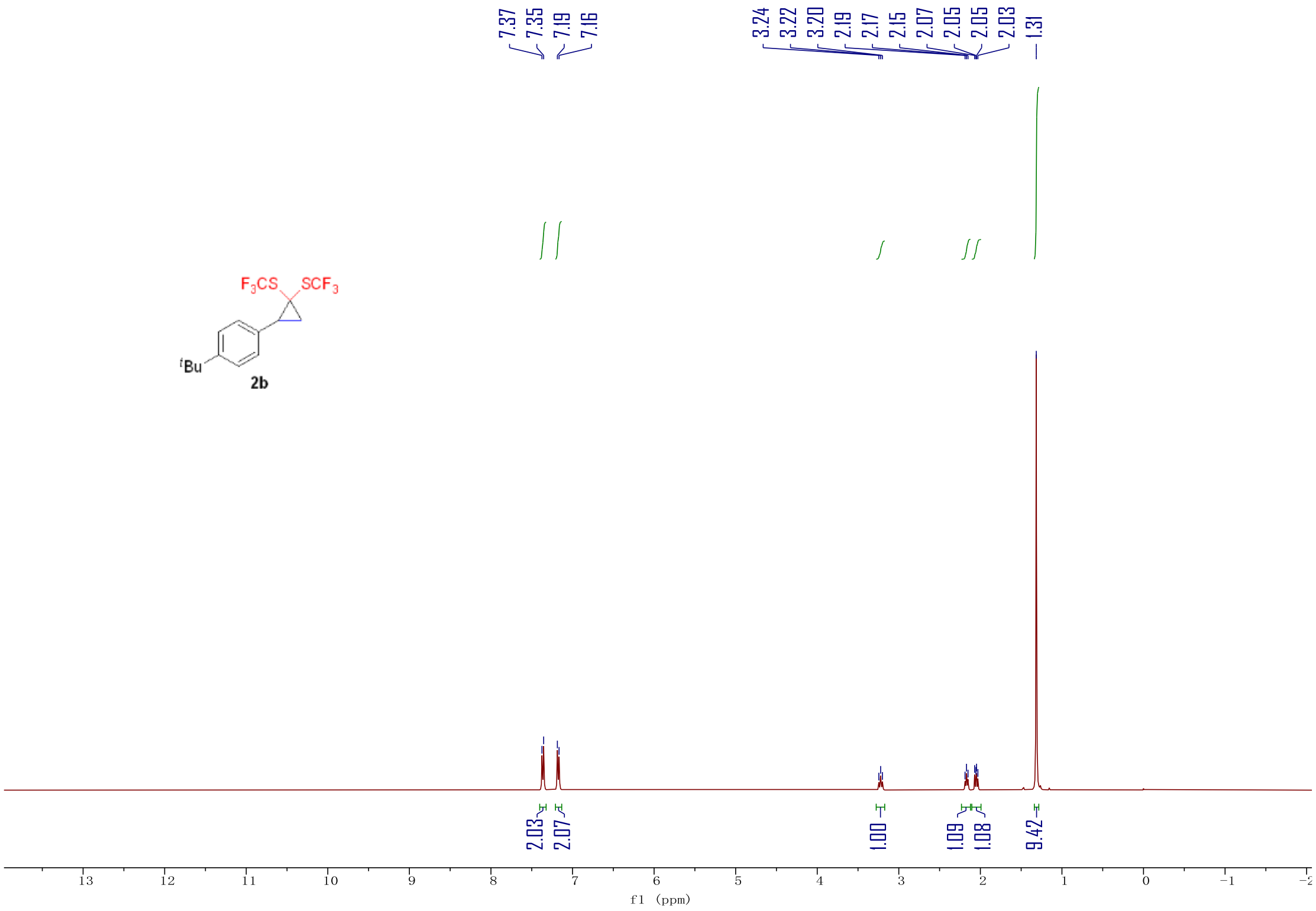

^{19}**F NMR** Spectrum of **2b** (376 MHz, $CDCl_3$)

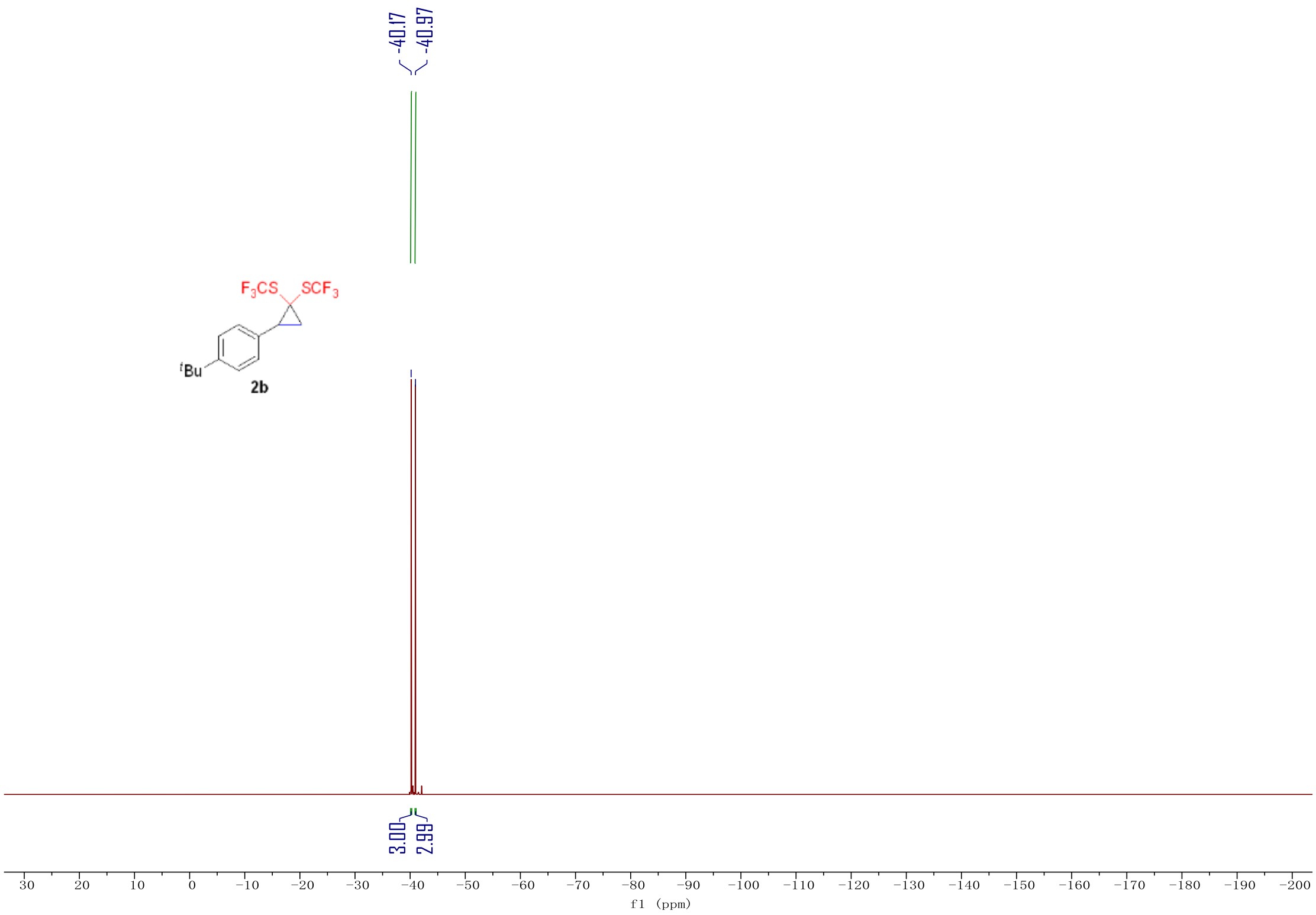

^{13}C **NMR** Spectrum of **2b** (101 MHz, $CDCl_3$)

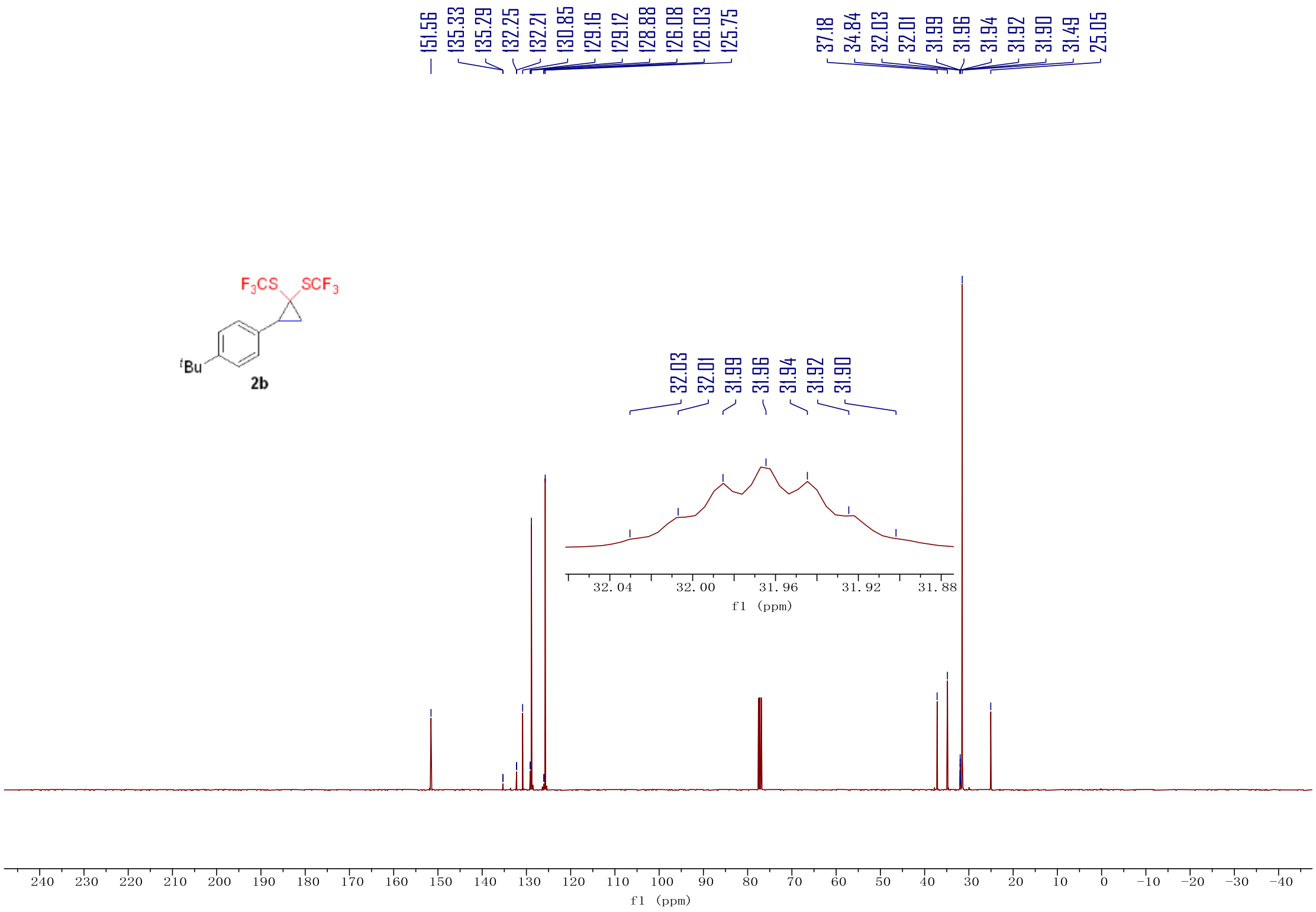

^{1}H **NMR** Spectrum of **2c** (400 MHz, $CDCl_3$)

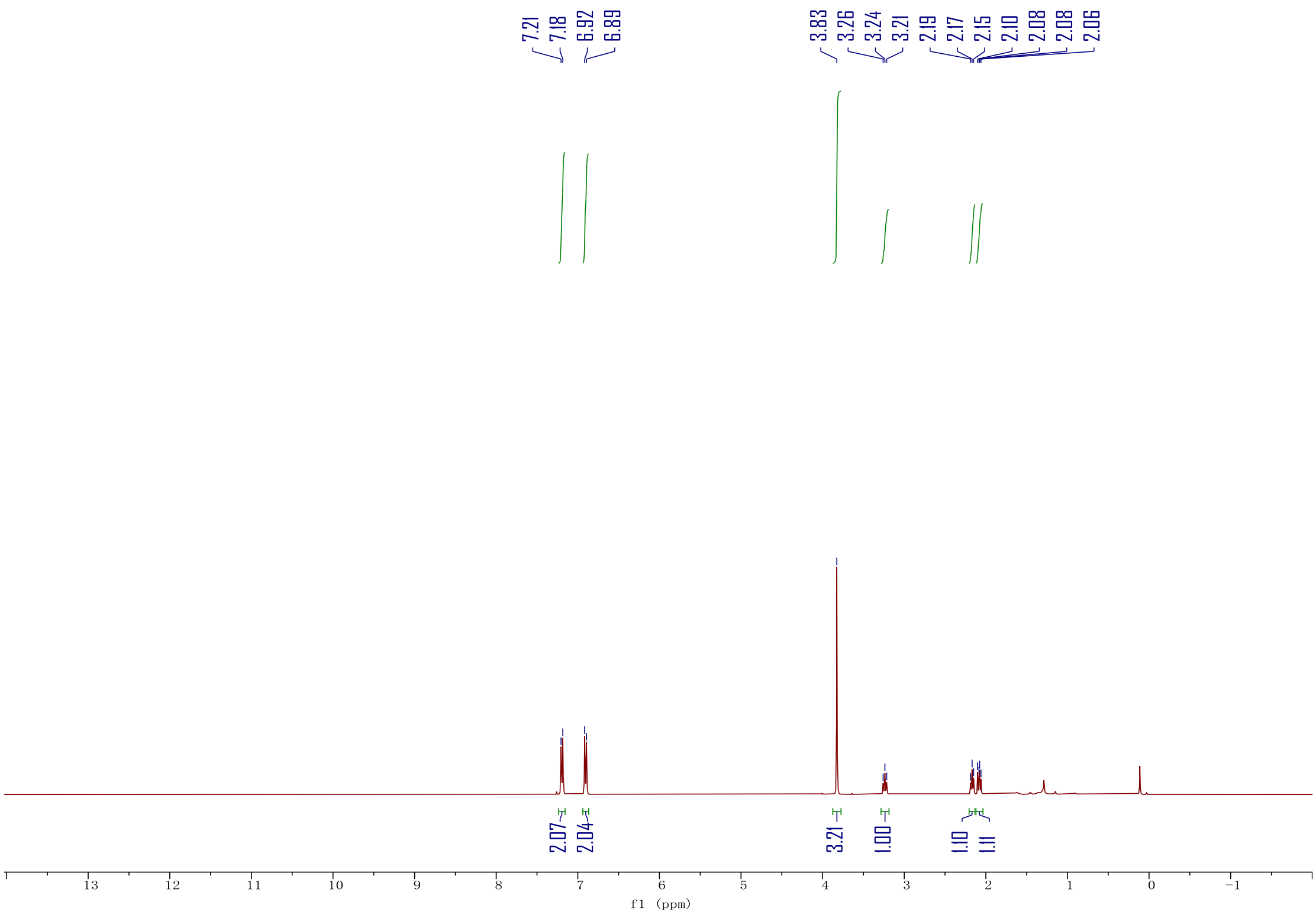

^{19}F NMR Spectrum of **2c** (376 MHz, $CDCl_3$)

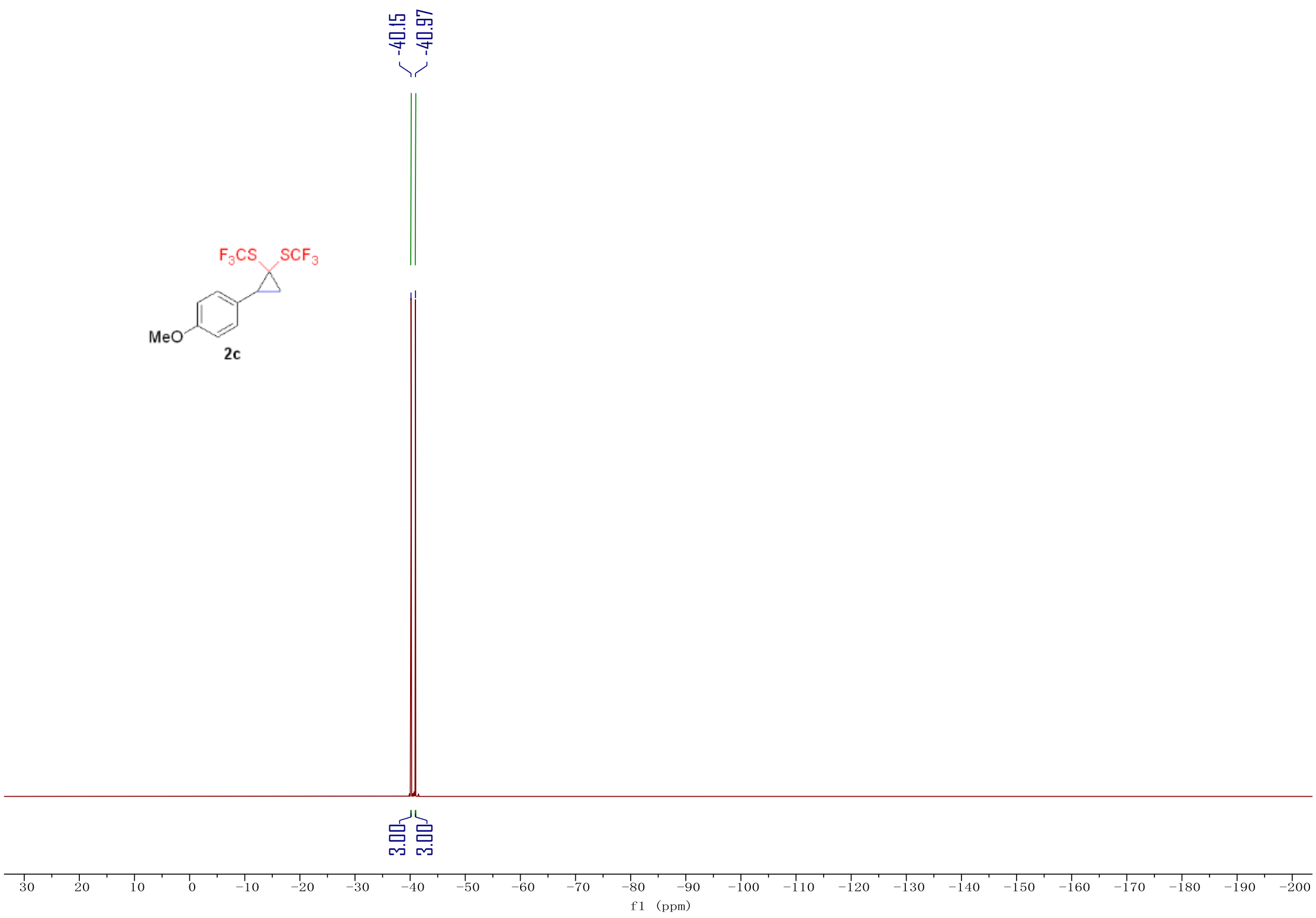

^{13}C NMR Spectrum of **2c** (101 MHz, $CDCl_3$)

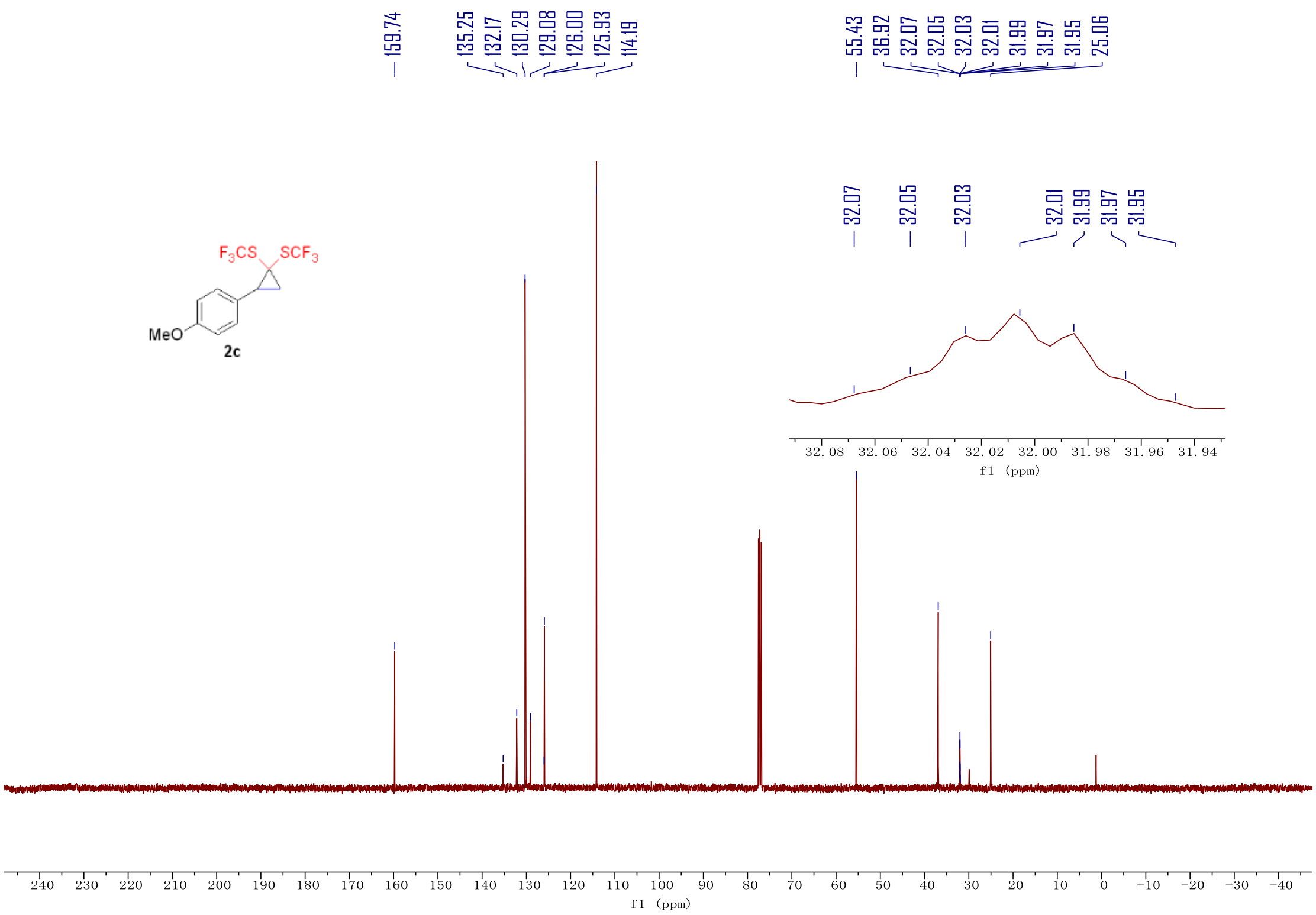

^{1}H NMR Spectrum of **2d** (400 MHz, $CDCl_3$)

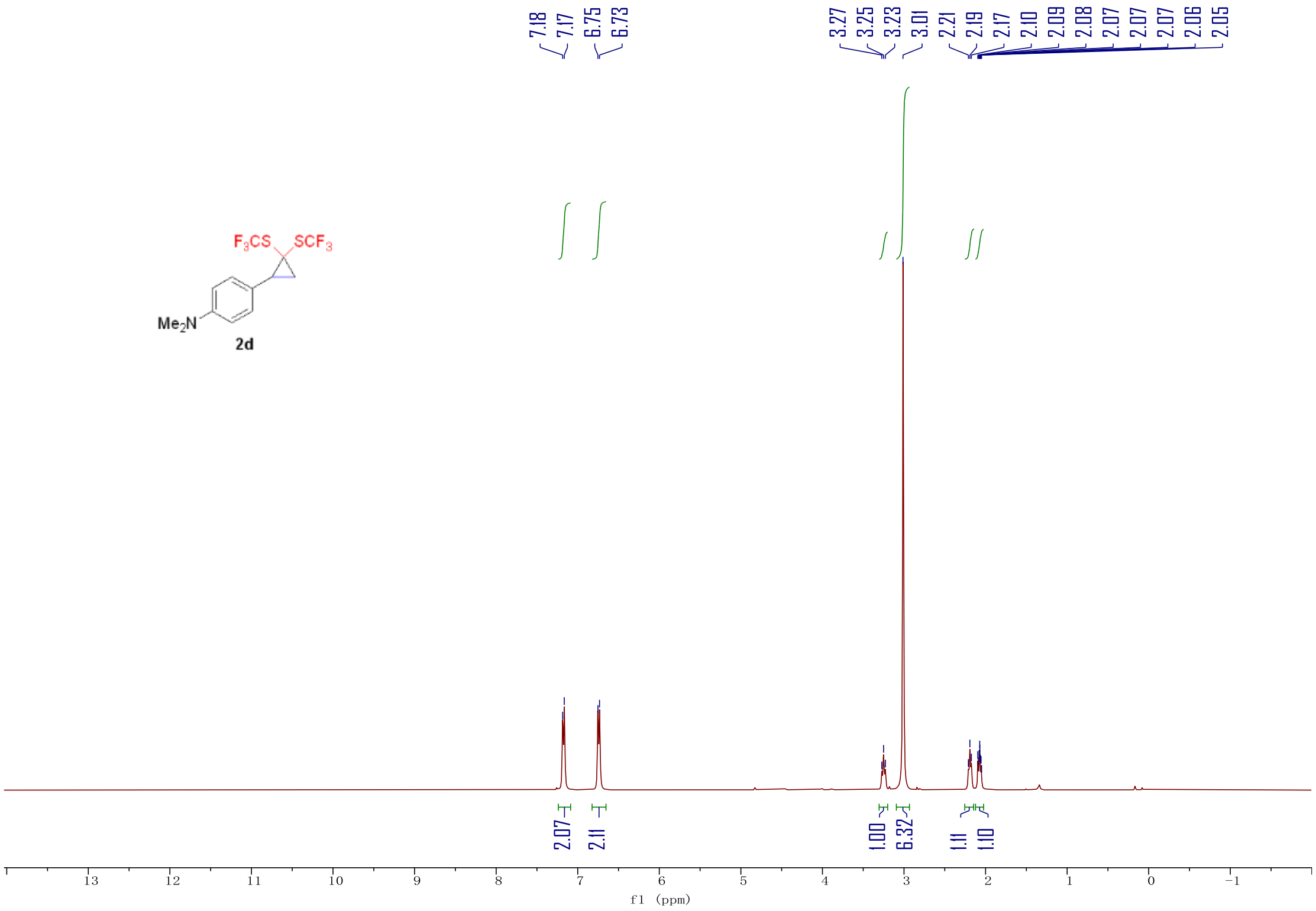

^{19}F NMR Spectrum of **2d** (376 MHz, $CDCl_3$)

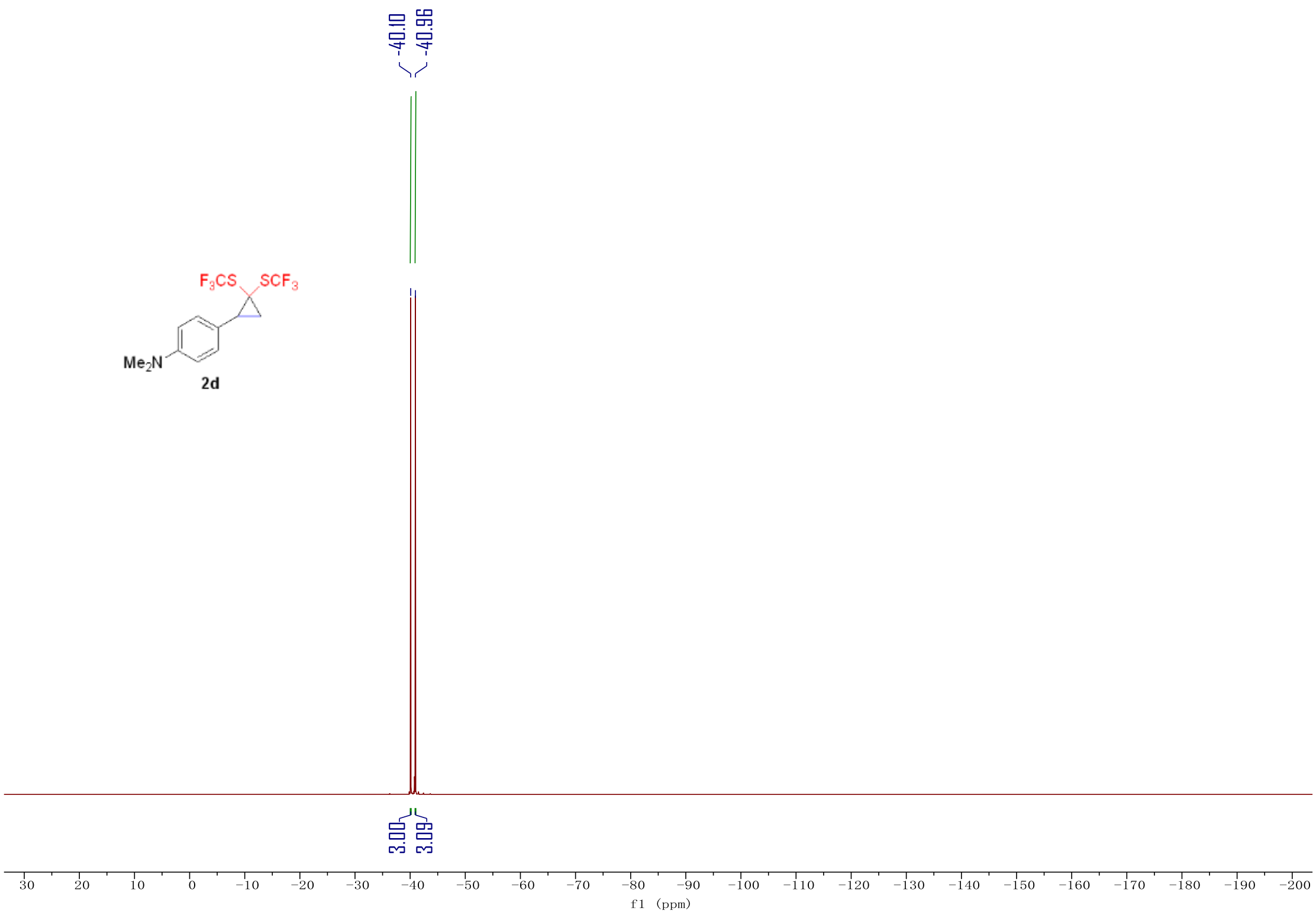

^{13}C NMR Spectrum of **2d** (101 MHz, $CDCl_3$)

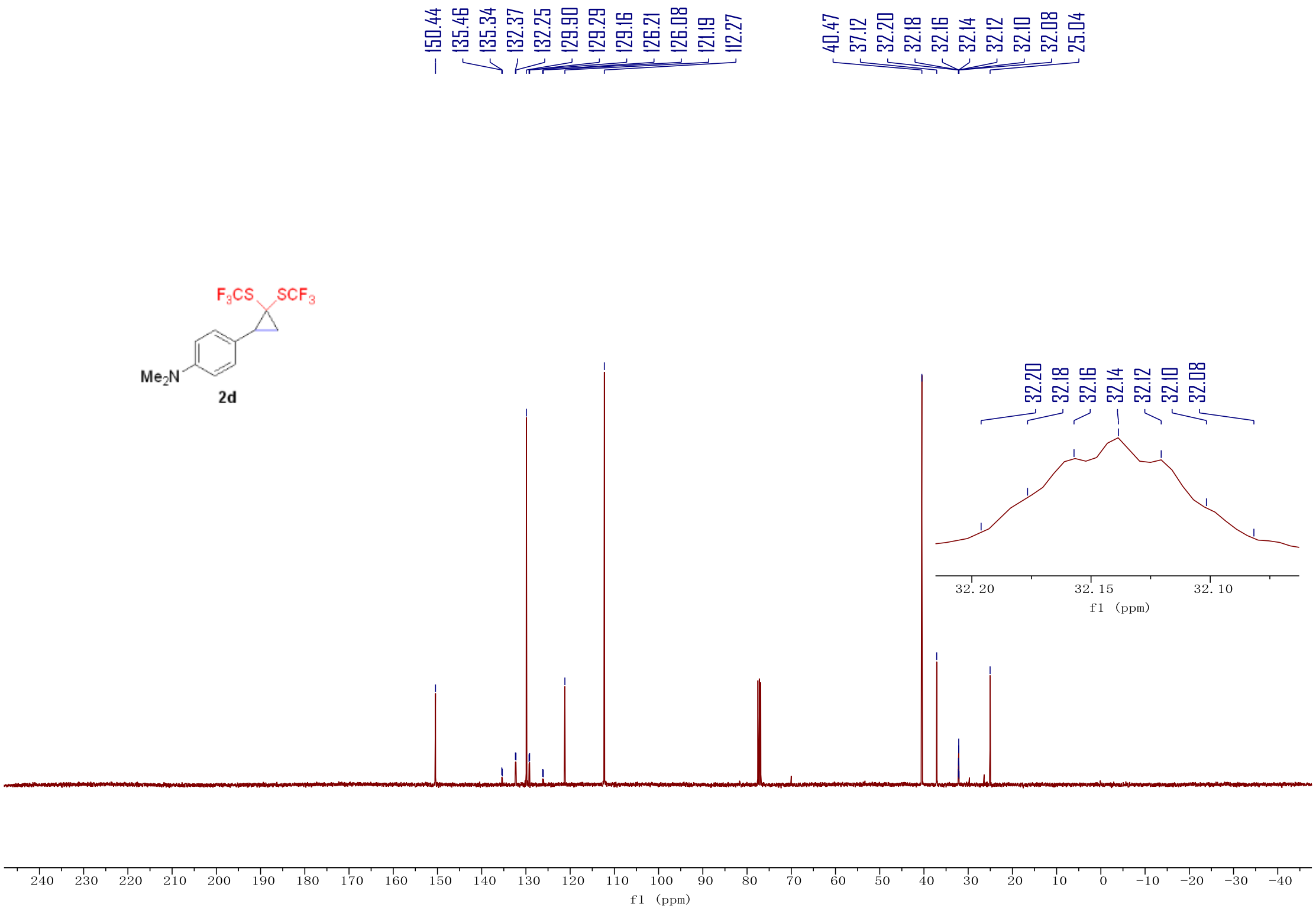

^{1}H NMR Spectrum of **2e** (400 MHz, $CDCl_3$)

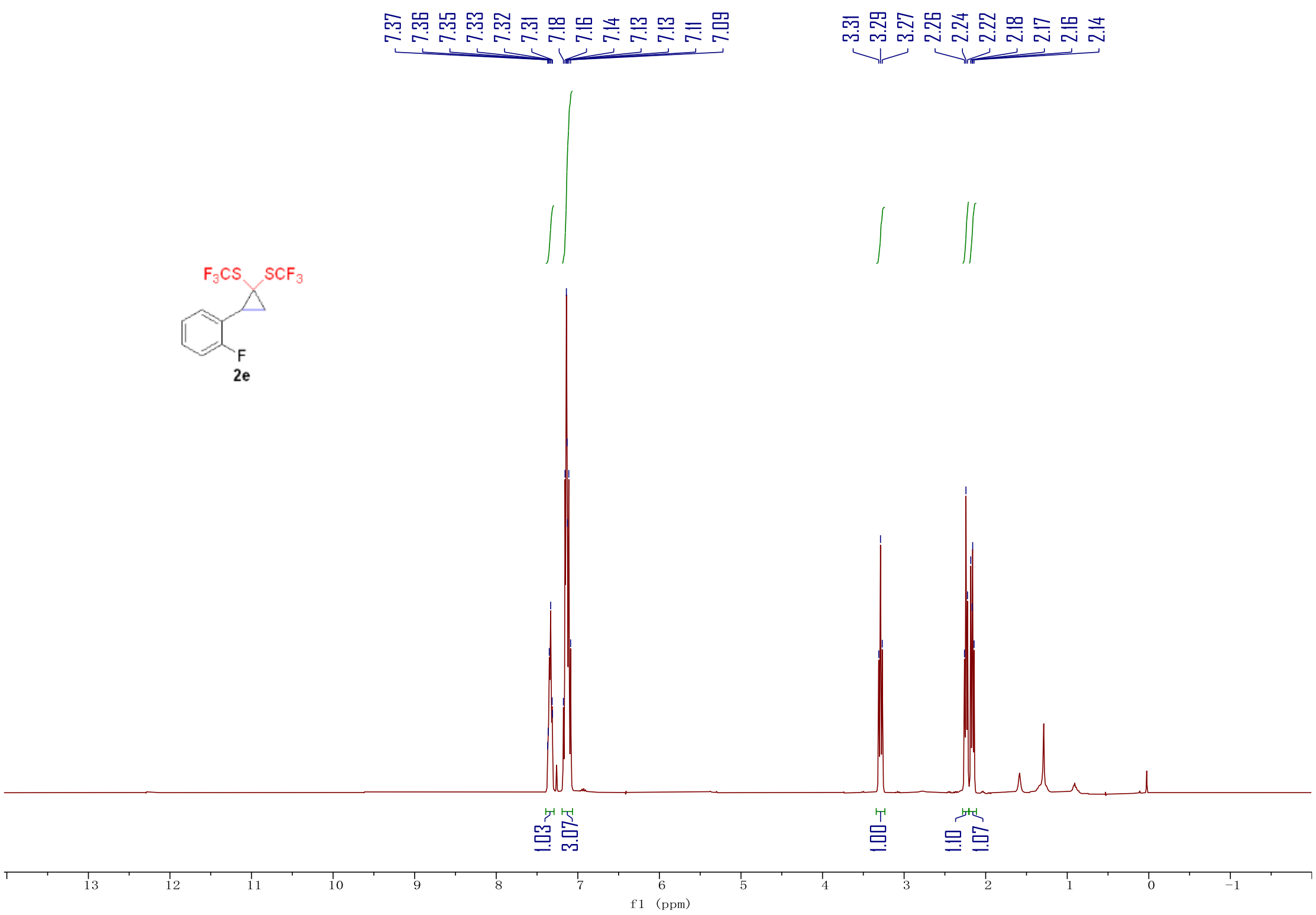

^{19}**F NMR** Spectrum of **2e** (376 MHz, $CDCl_3$)

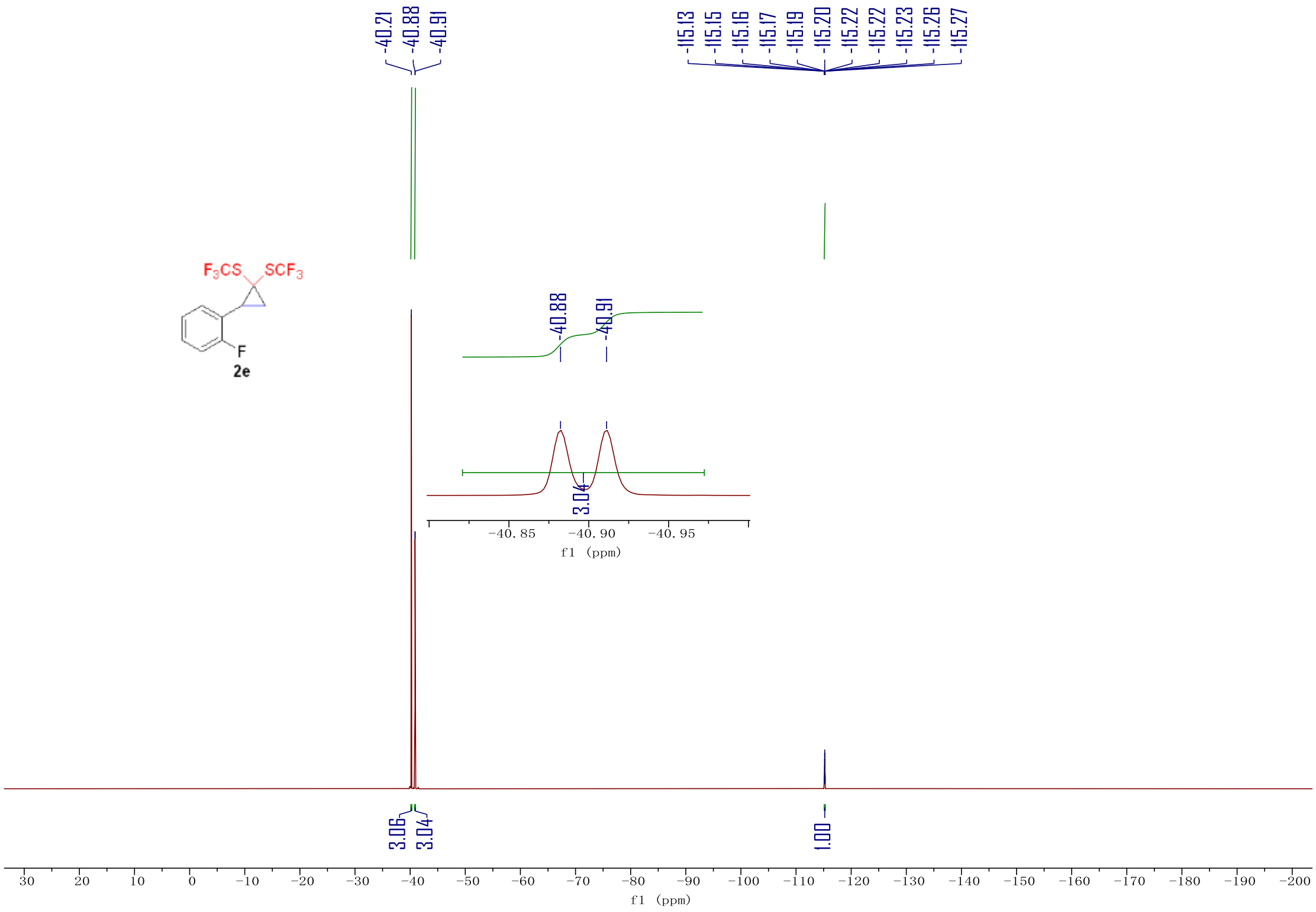

^{13}**C NMR** Spectrum of **2e** (101 MHz, $CDCl_3$)

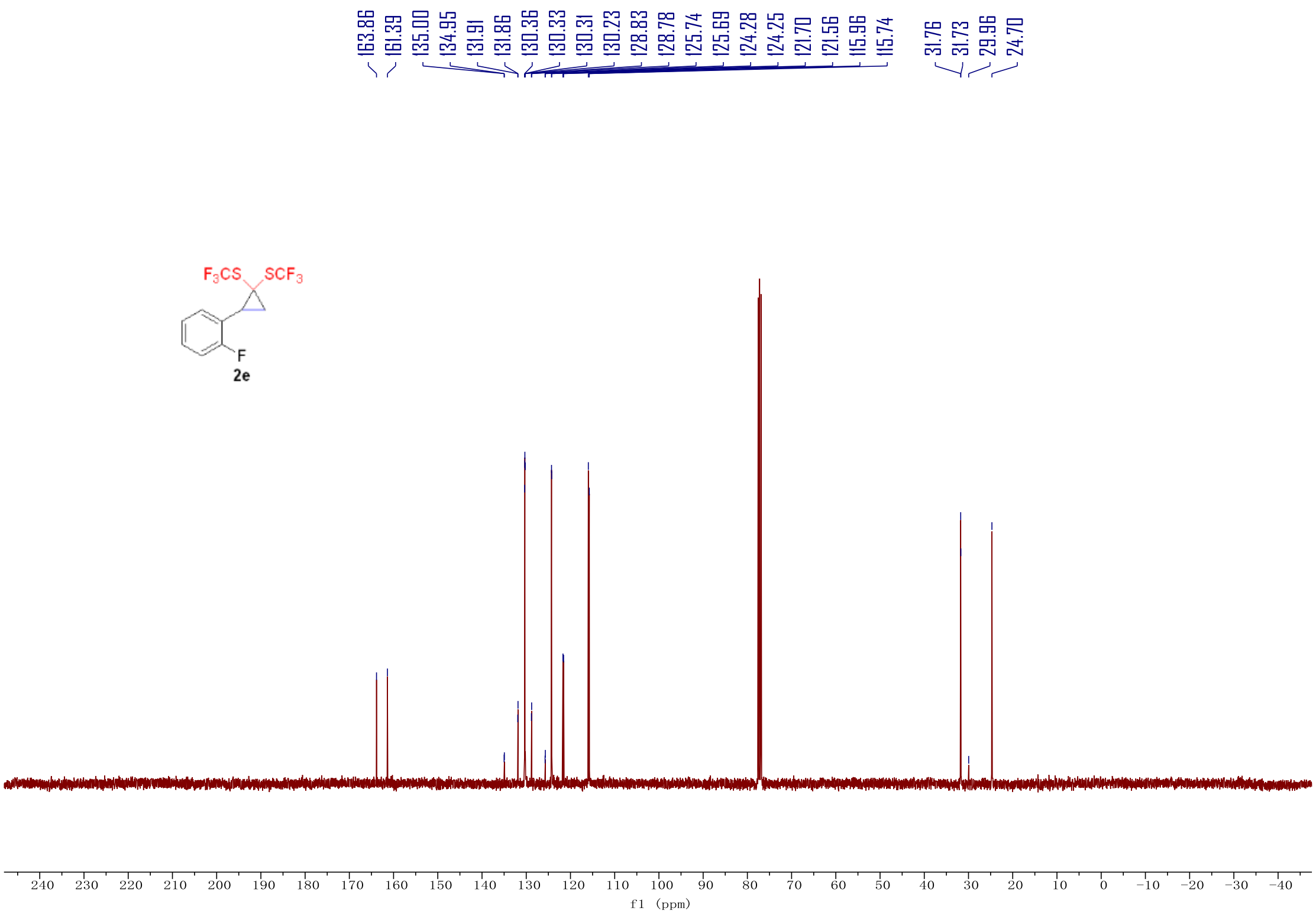

^{1}H NMR Spectrum of **2f** (400 MHz, $CDCl_3$)

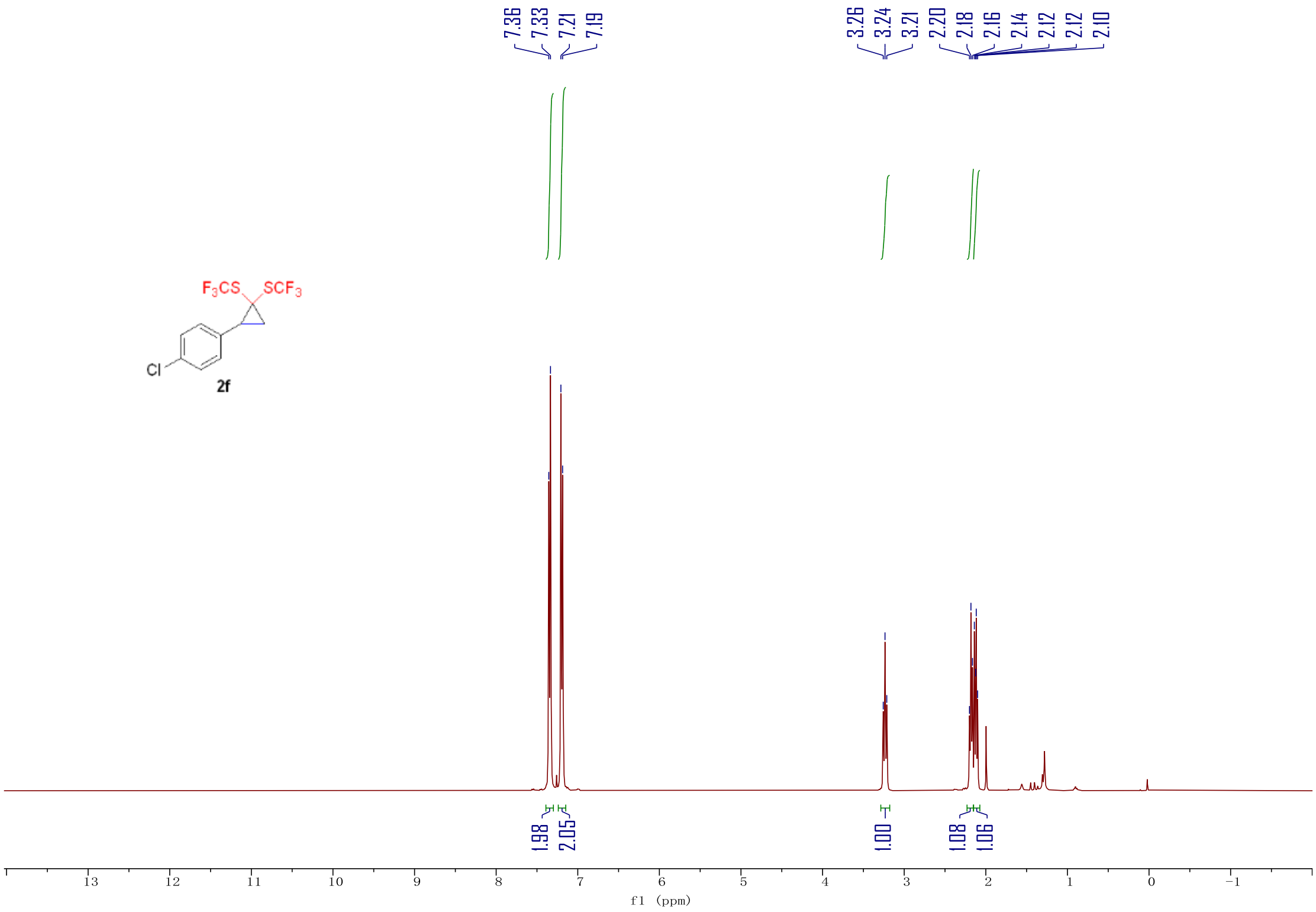

^{19}F NMR Spectrum of **2f** (376 MHz, $CDCl_3$)

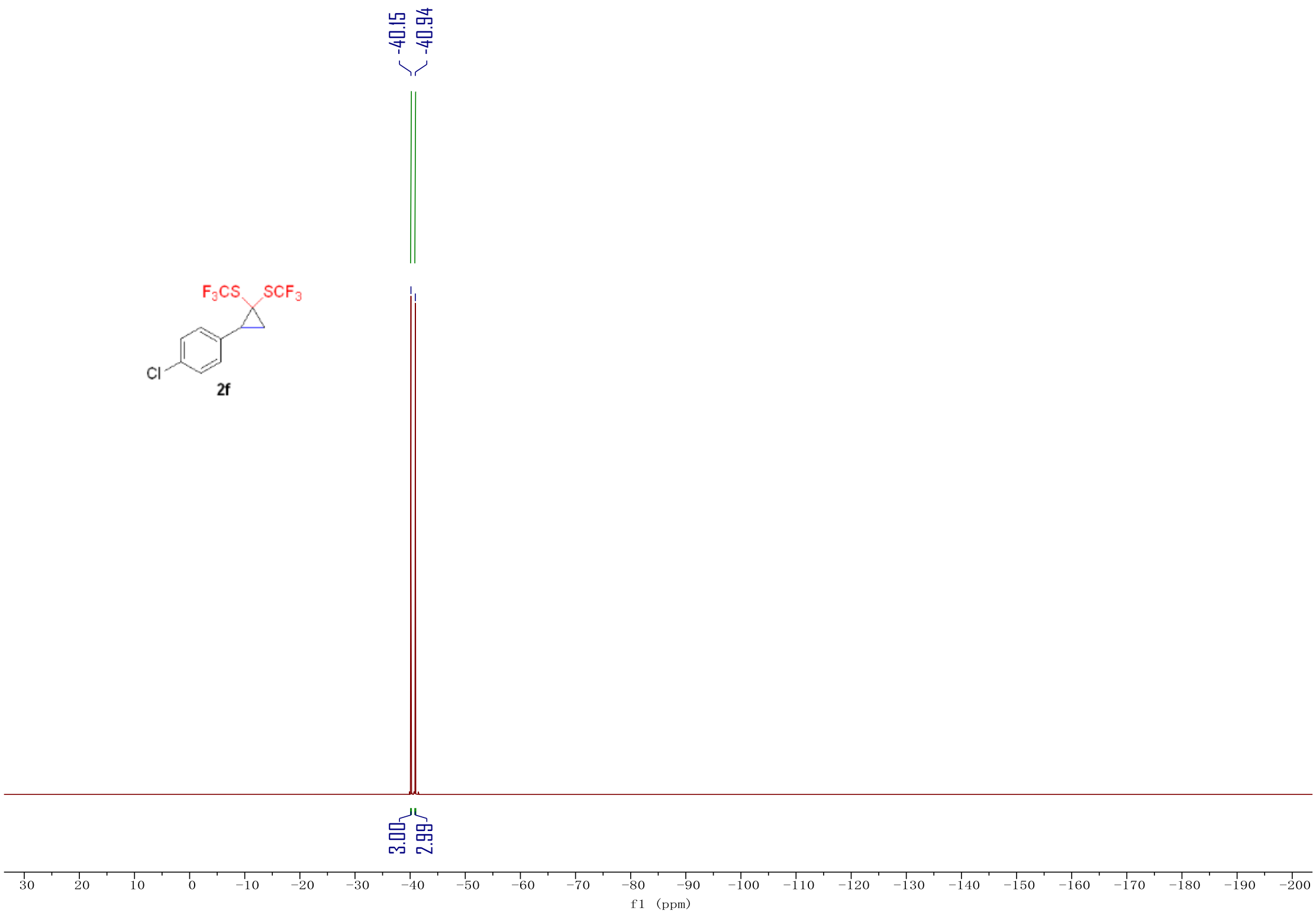

^{13}C NMR Spectrum of **2f** (101 MHz, $CDCl_3$)

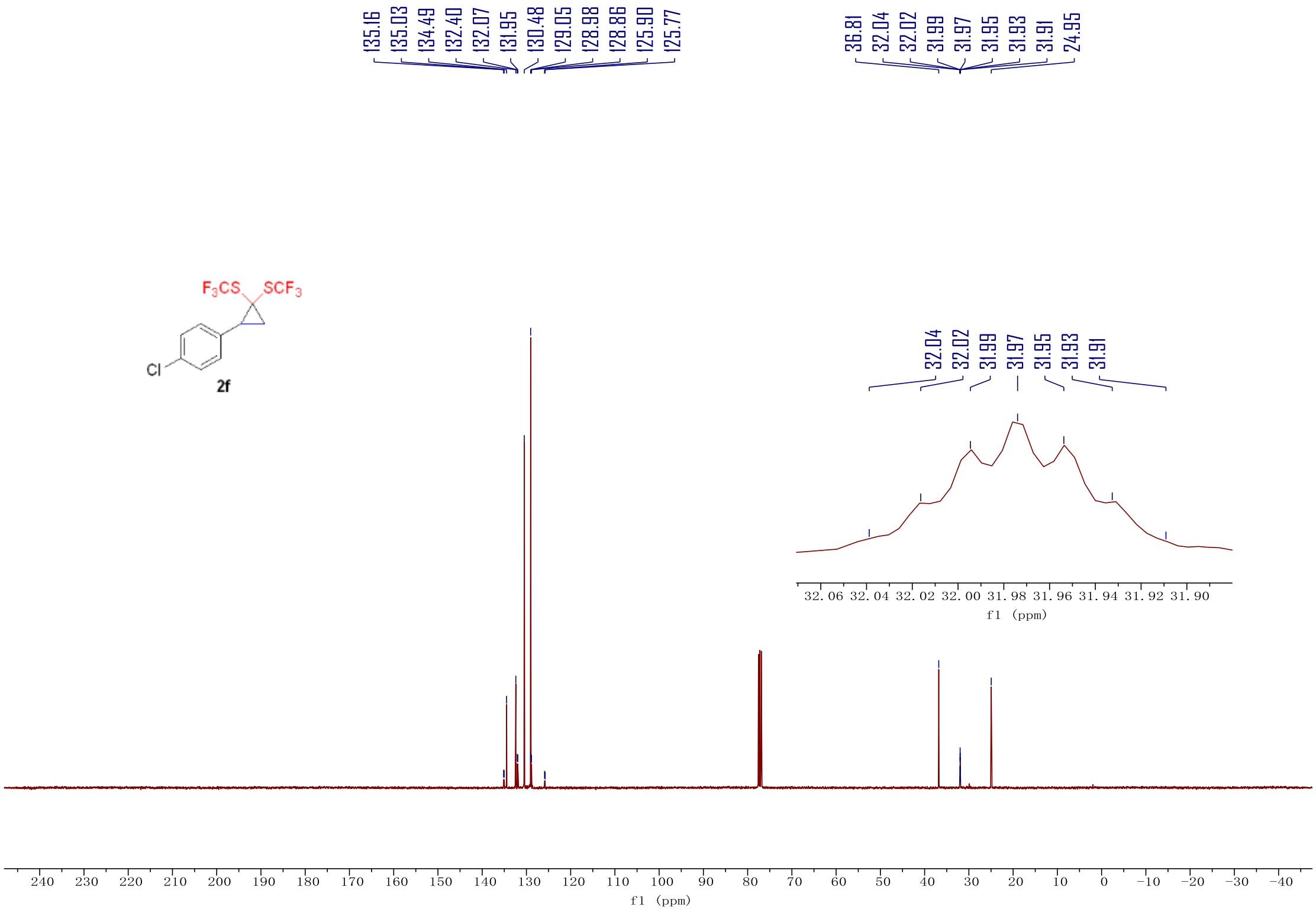

^{1}H NMR Spectrum of **2g** (400 MHz, $CDCl_3$)

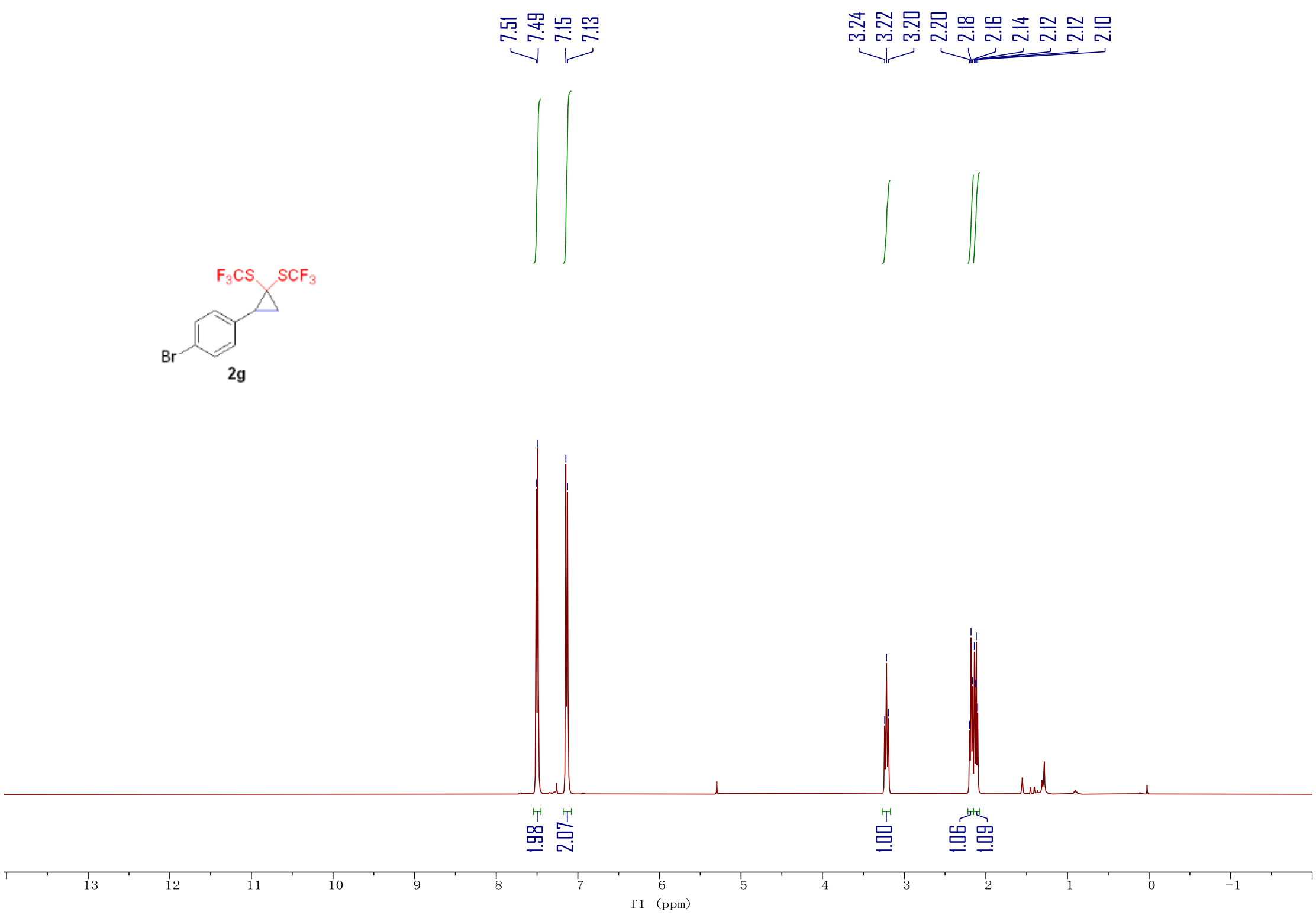

^{19}F NMR Spectrum of **2g** (376 MHz, $CDCl_3$)

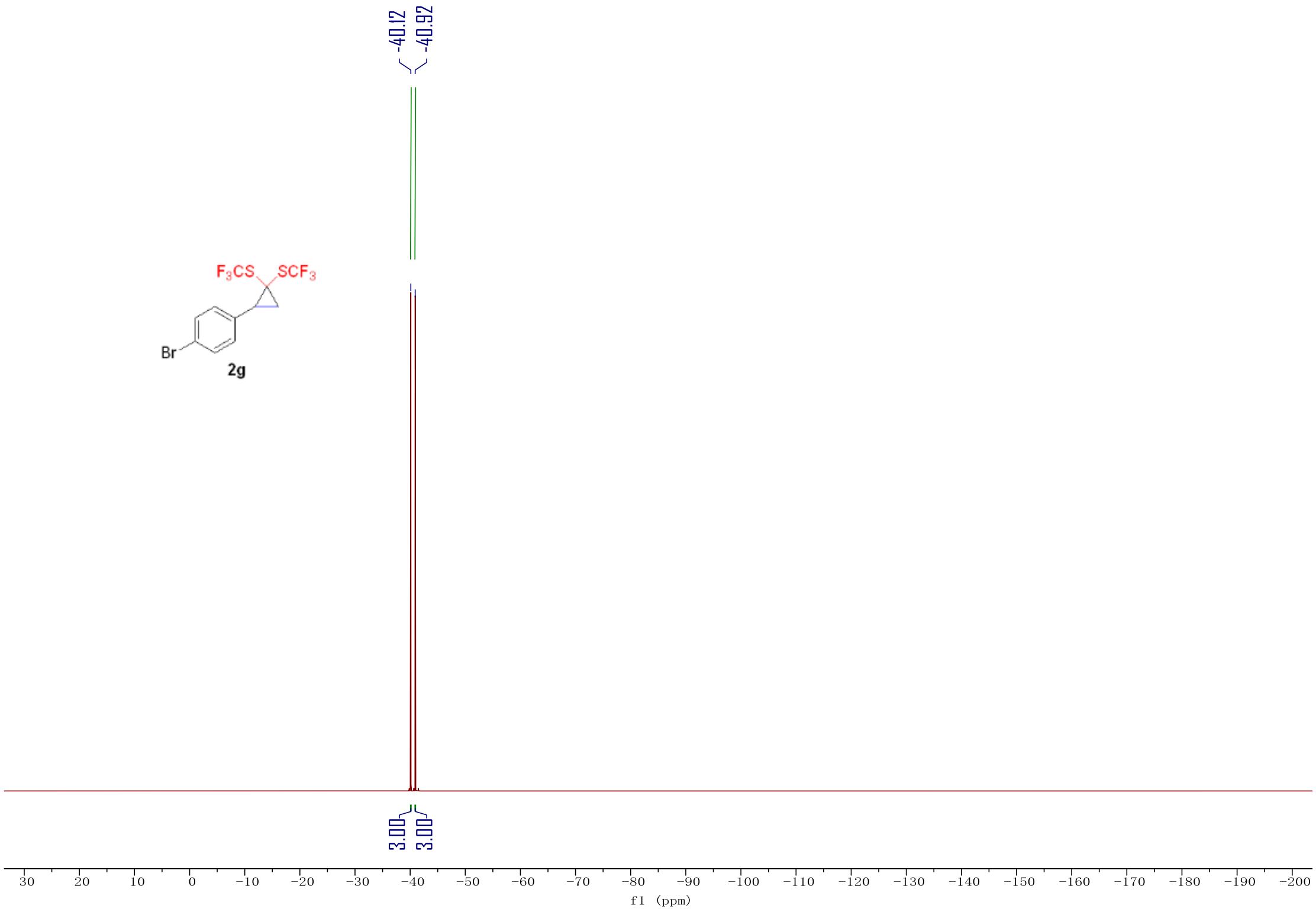

^{13}C NMR Spectrum of **2g** (101 MHz, $CDCl_3$)

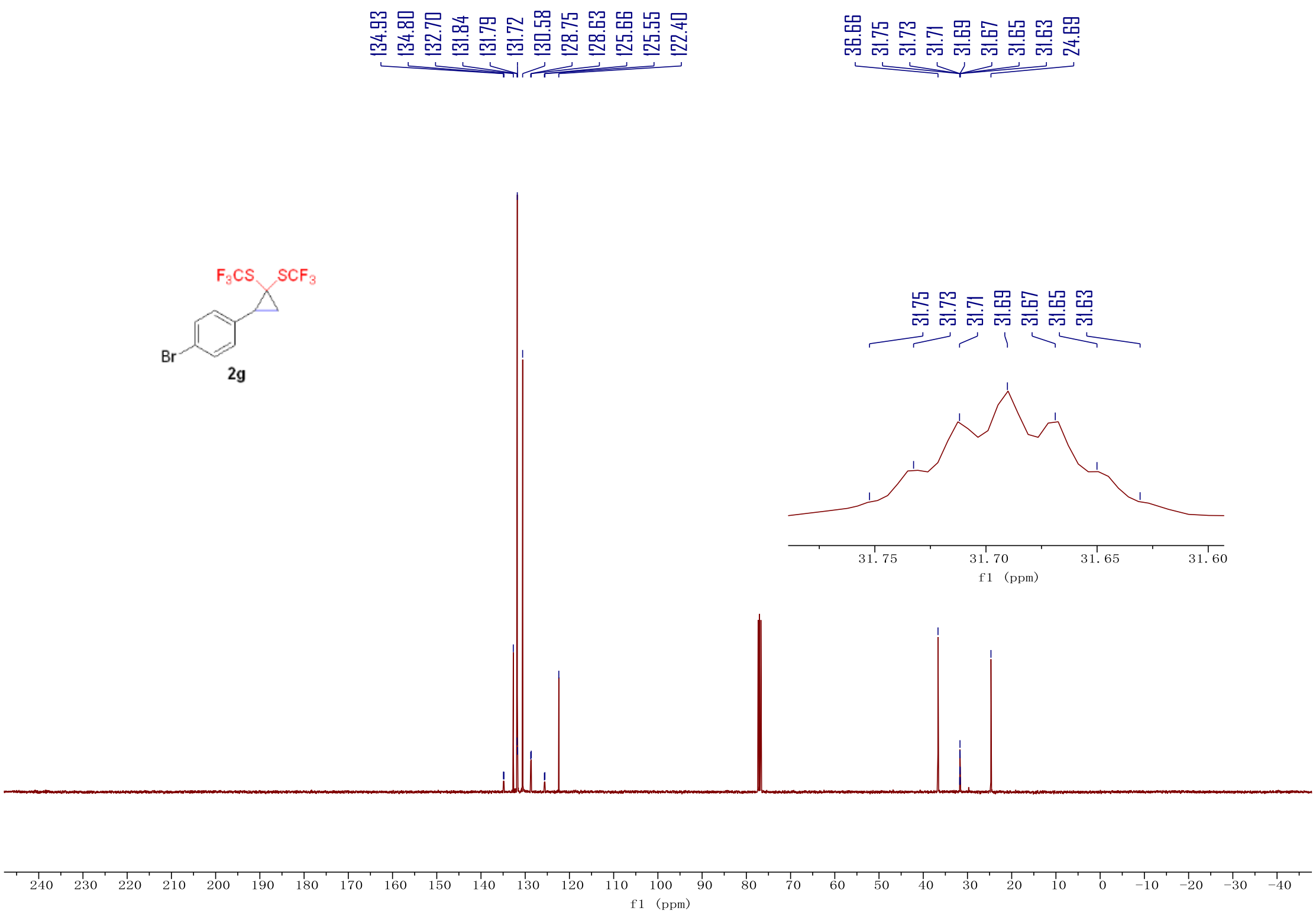

^{1}H NMR Spectrum of **2h** (400 MHz, $CDCl_3$)

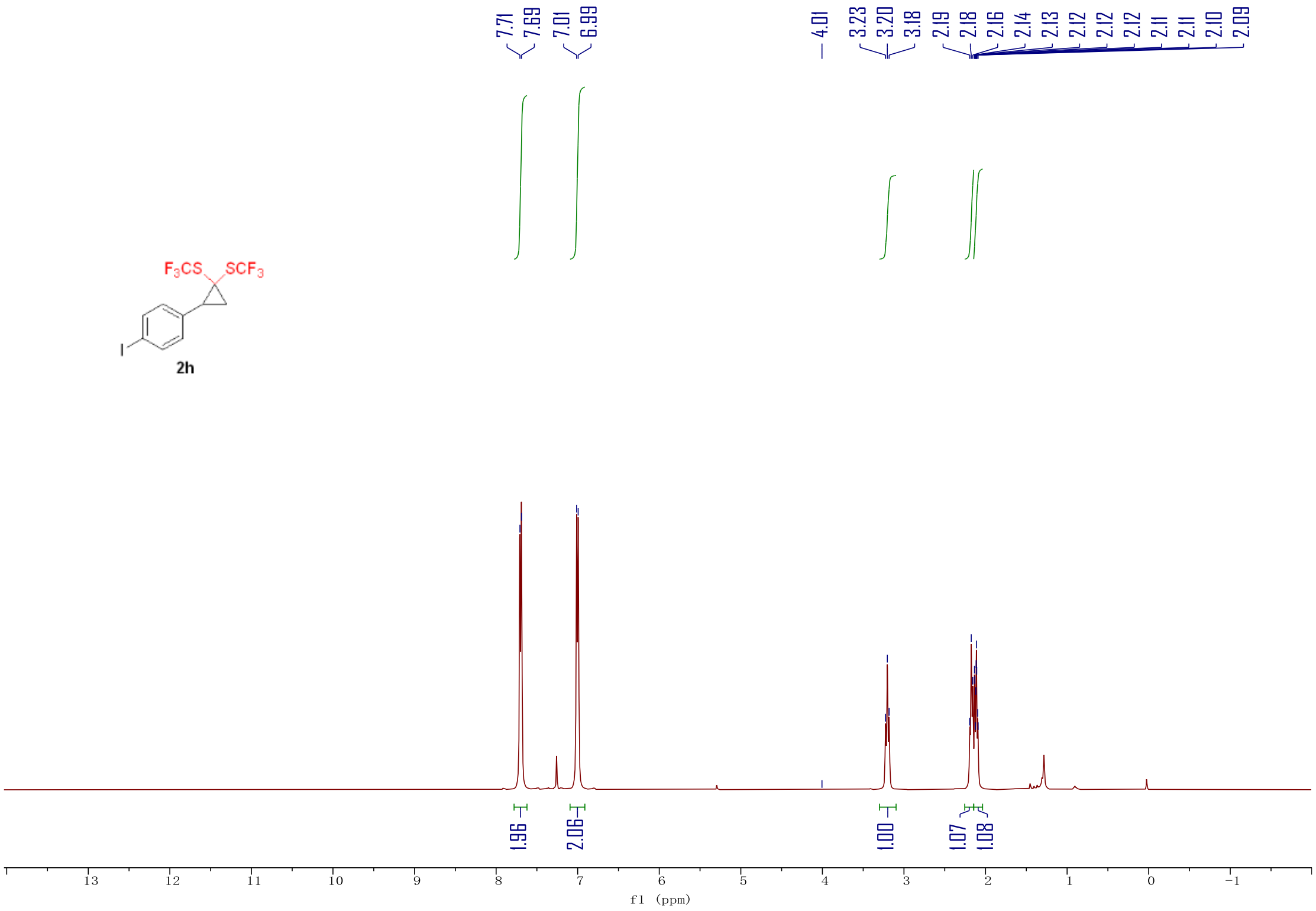

^{19}F NMR Spectrum of **2h** (376 MHz, $CDCl_3$)

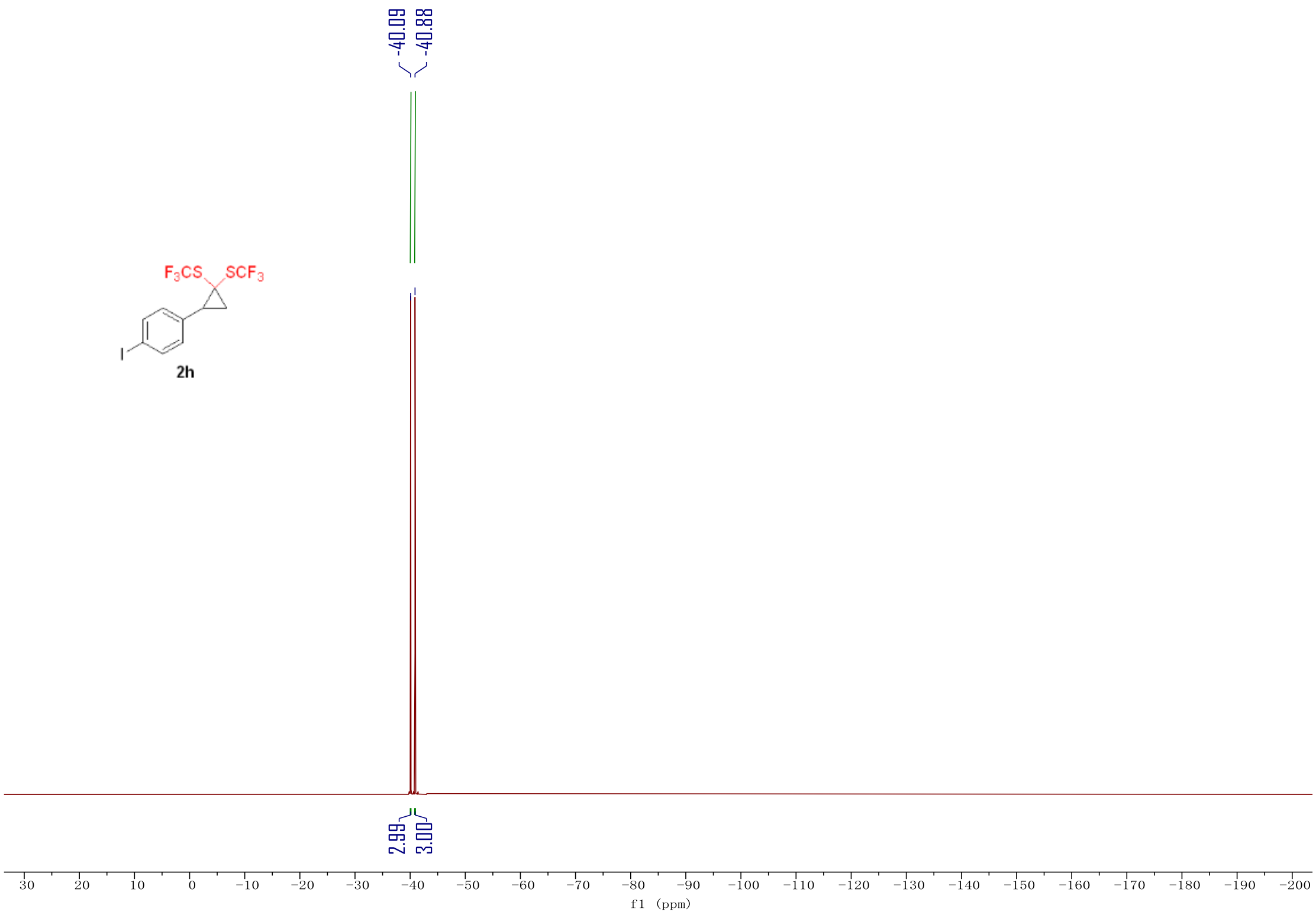

^{13}C NMR Spectrum of **2h** (101 MHz, $CDCl_3$)

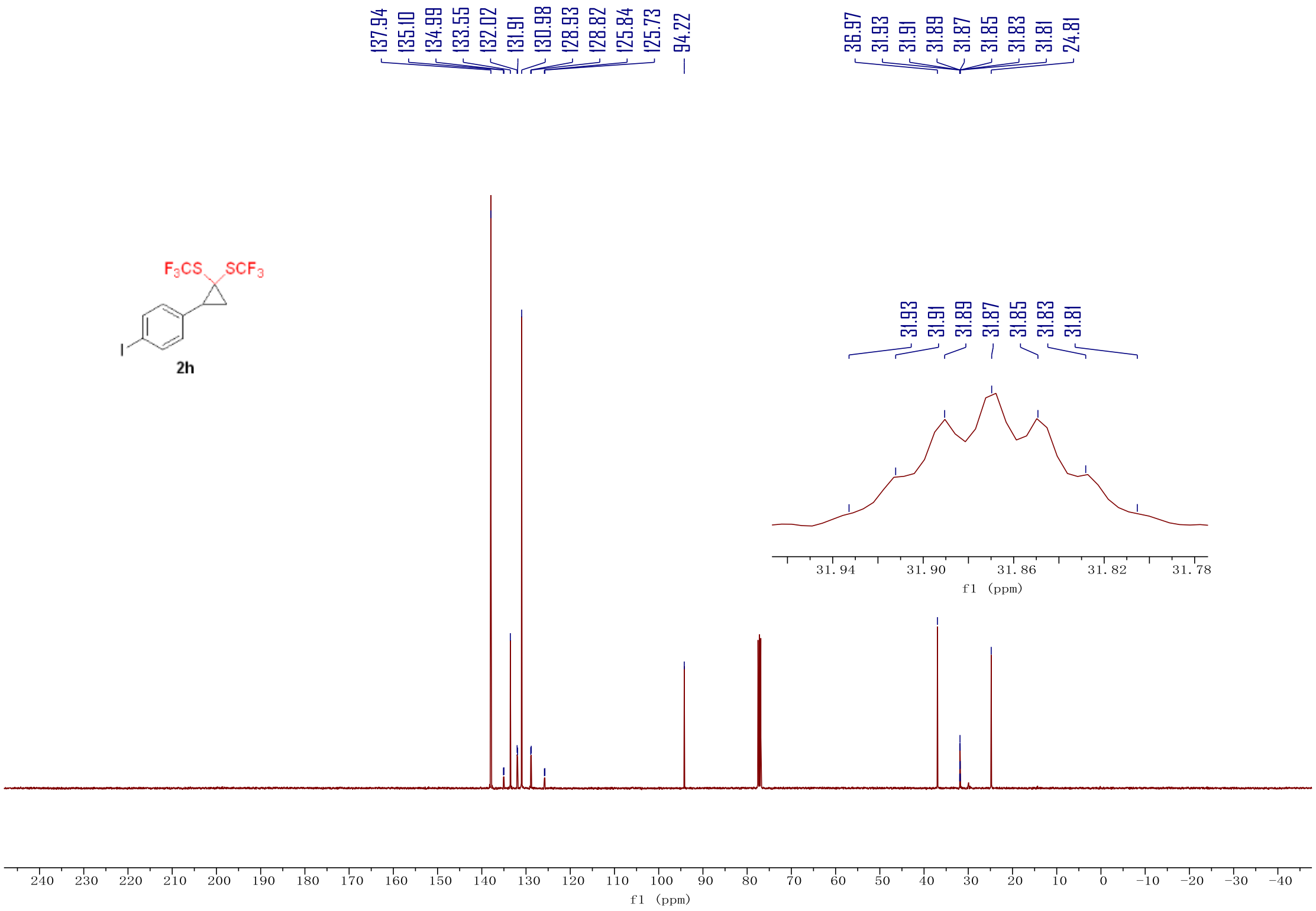

^{1}H NMR Spectrum of **2i** (400 MHz, $CDCl_3$)

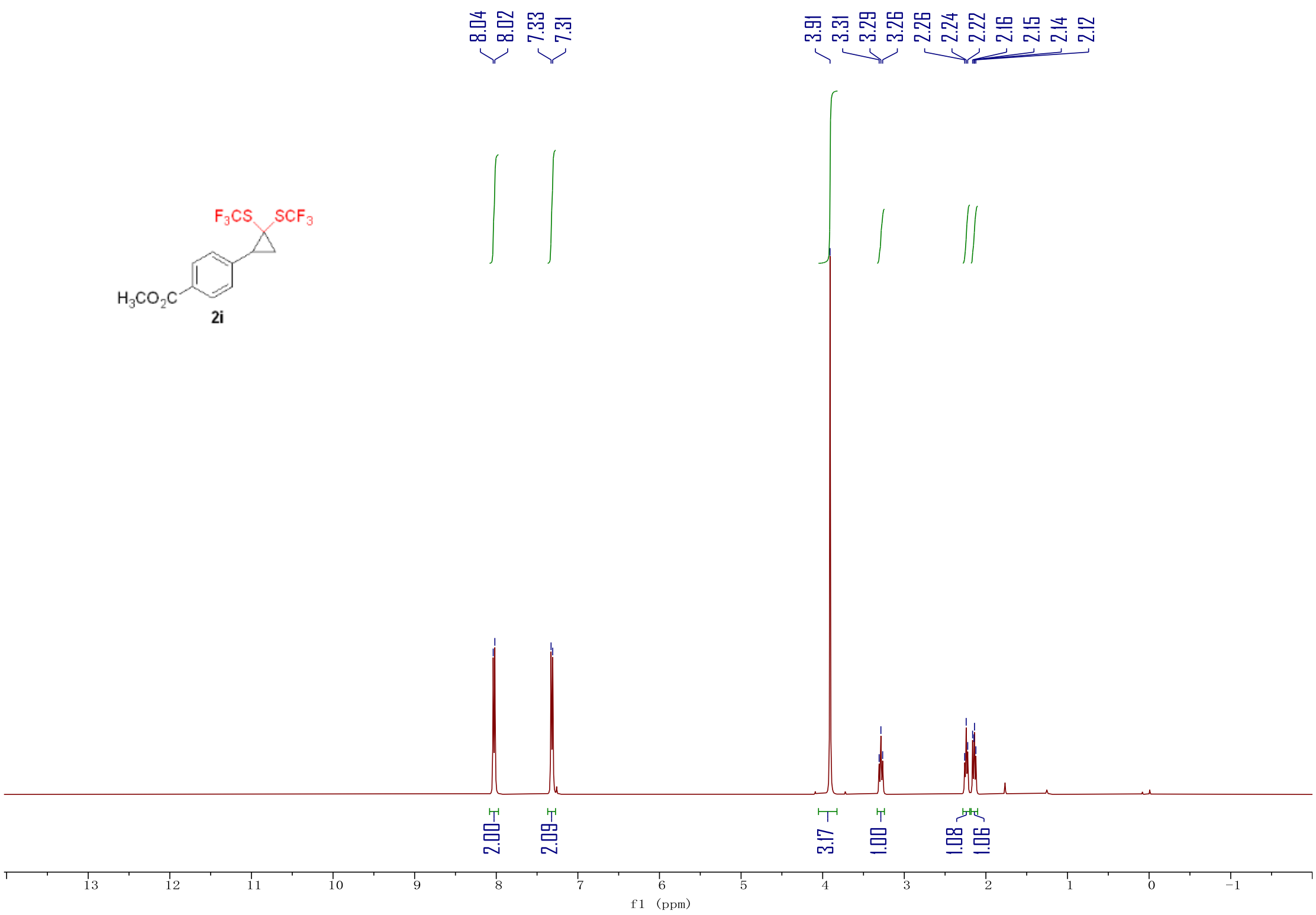

^{19}**F NMR** Spectrum of **2i** (376 MHz, $CDCl_3$)

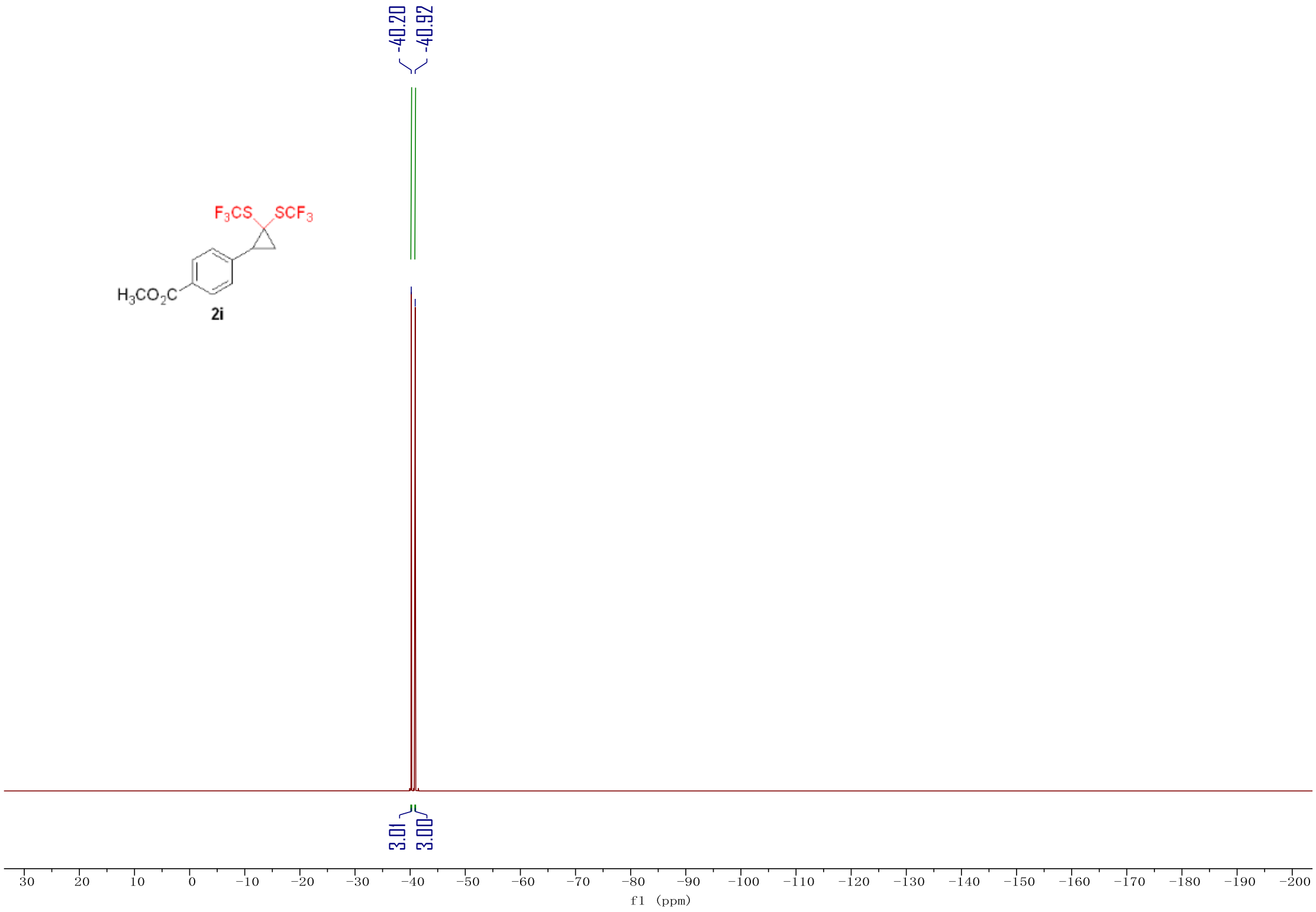

^{13}**C NMR** Spectrum of **2i** (101 MHz, $CDCl_3$)

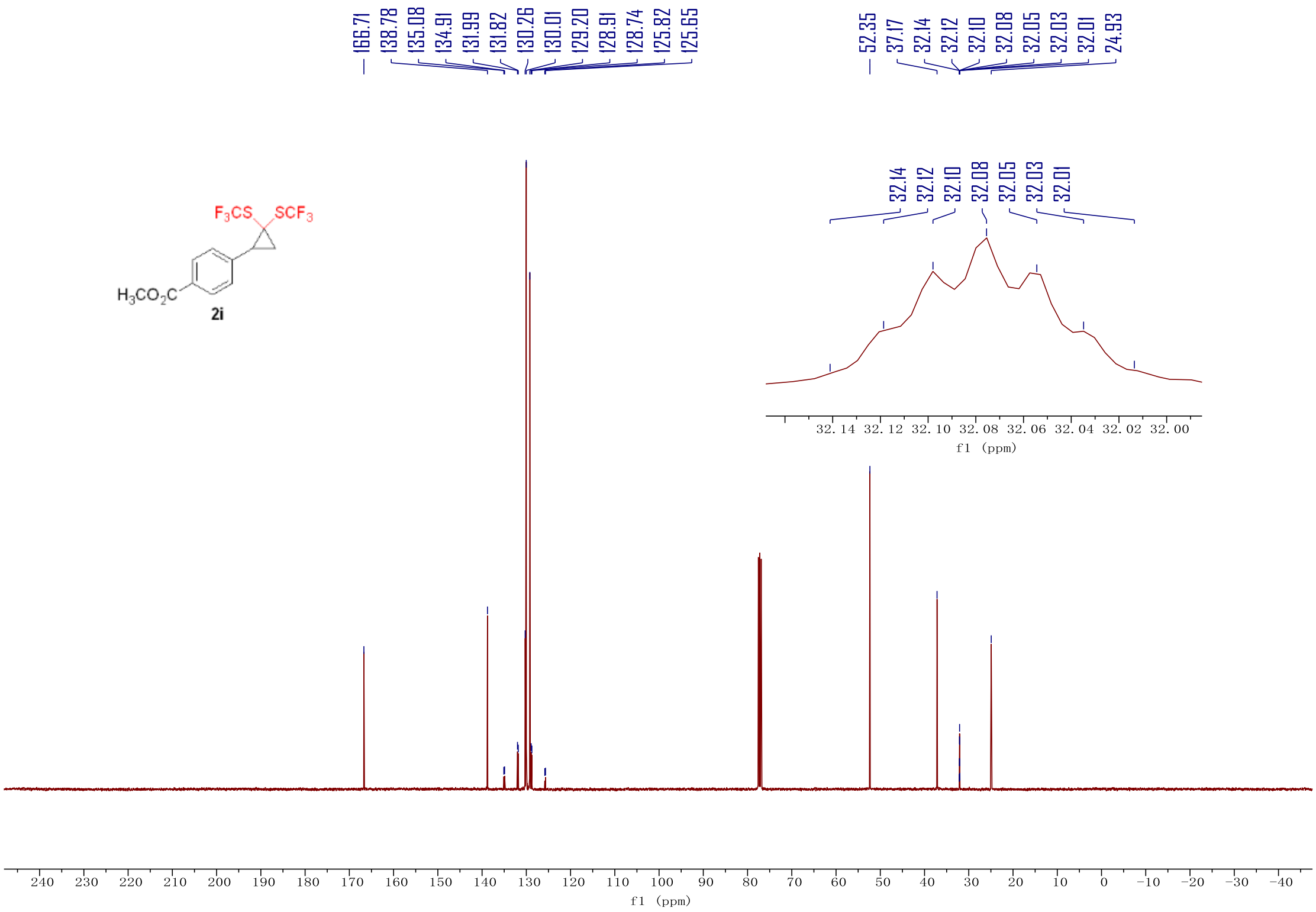

^{1}H NMR Spectrum of **2j** (400 MHz, $CDCl_3$)

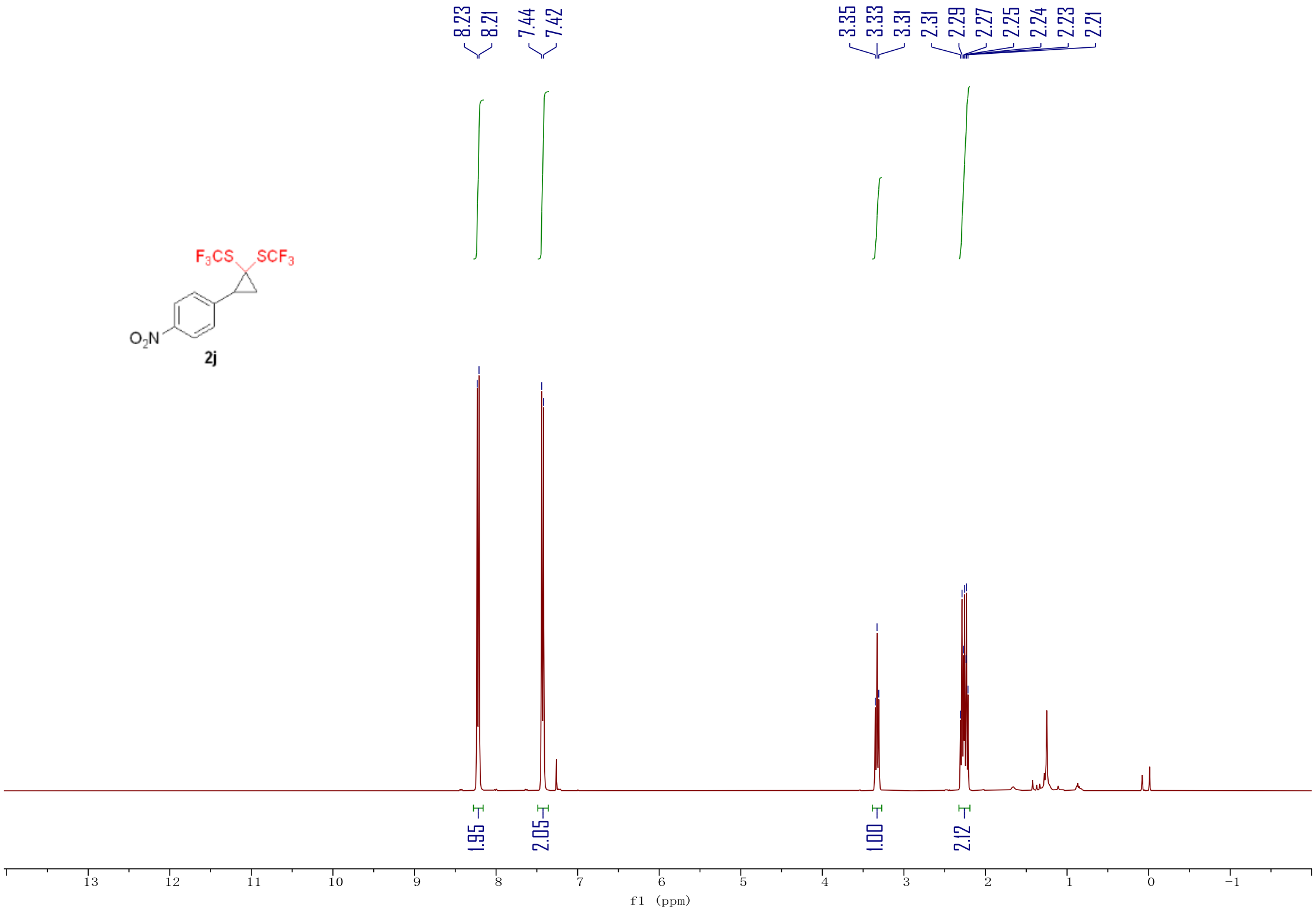

^{19}F NMR Spectrum of **2j** (376 MHz, $CDCl_3$)

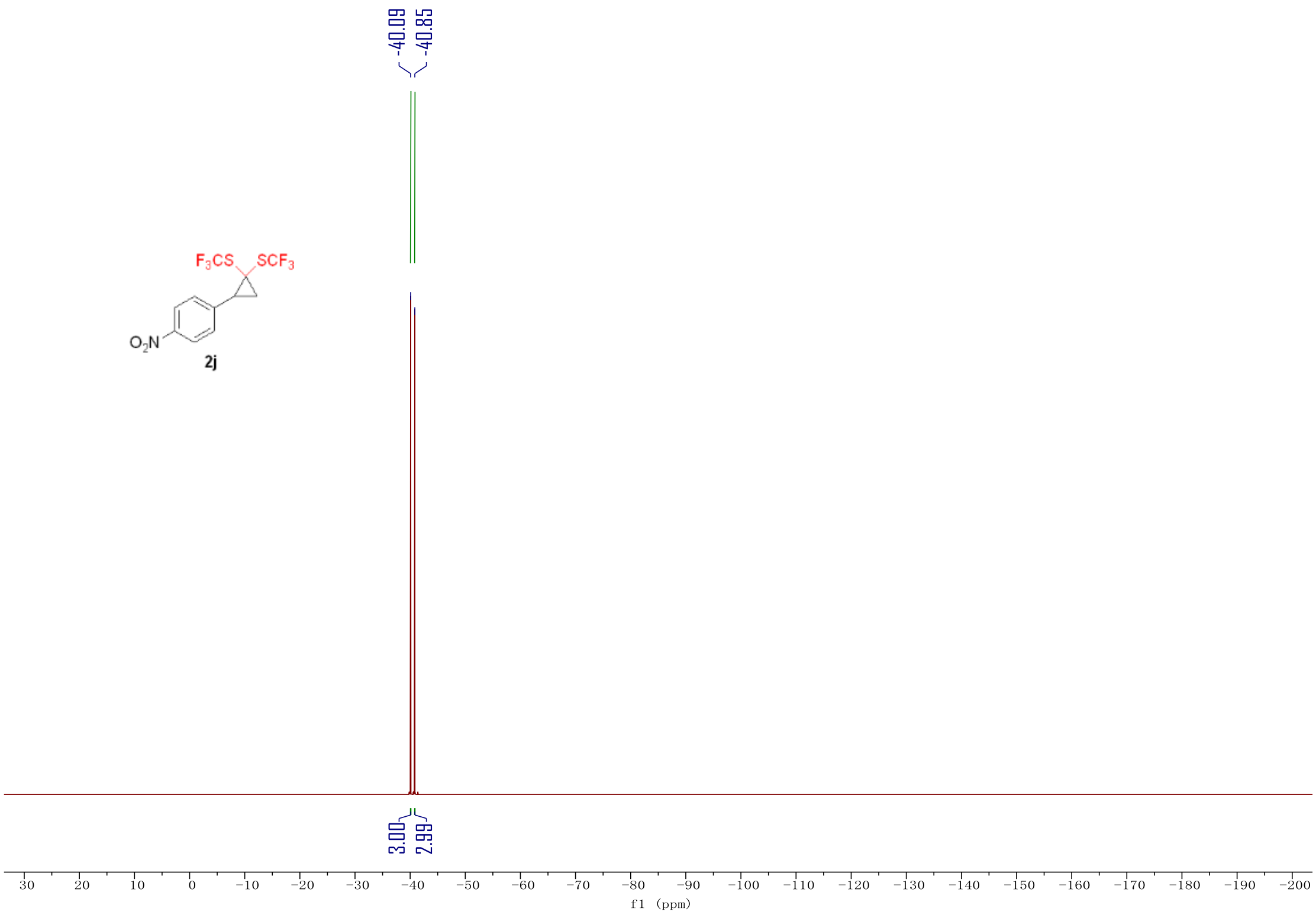

^{13}C NMR Spectrum of **2j** (101 MHz, $CDCl_3$)

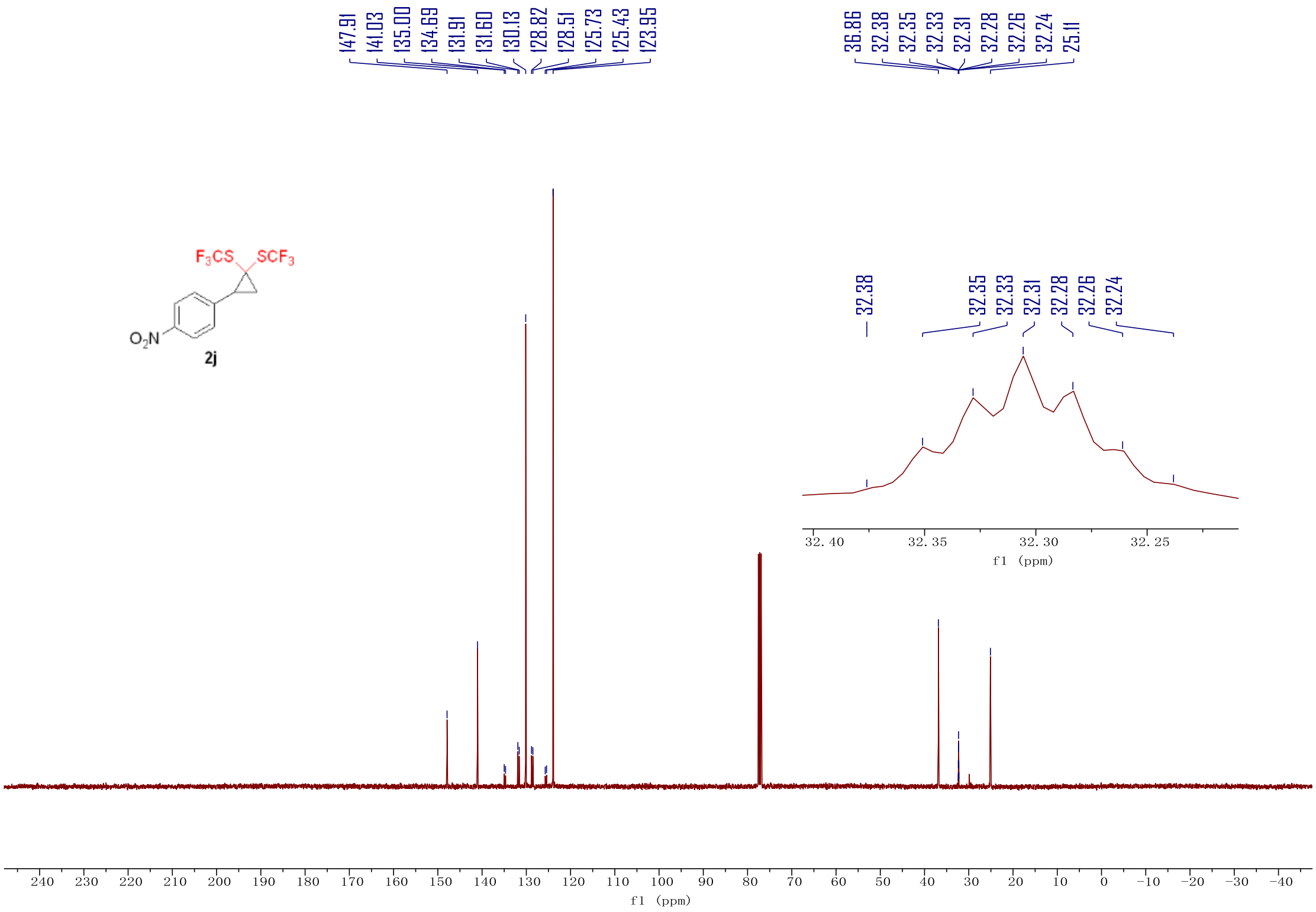

^{1}H NMR Spectrum of **2k** (400 MHz, $CDCl_3$)

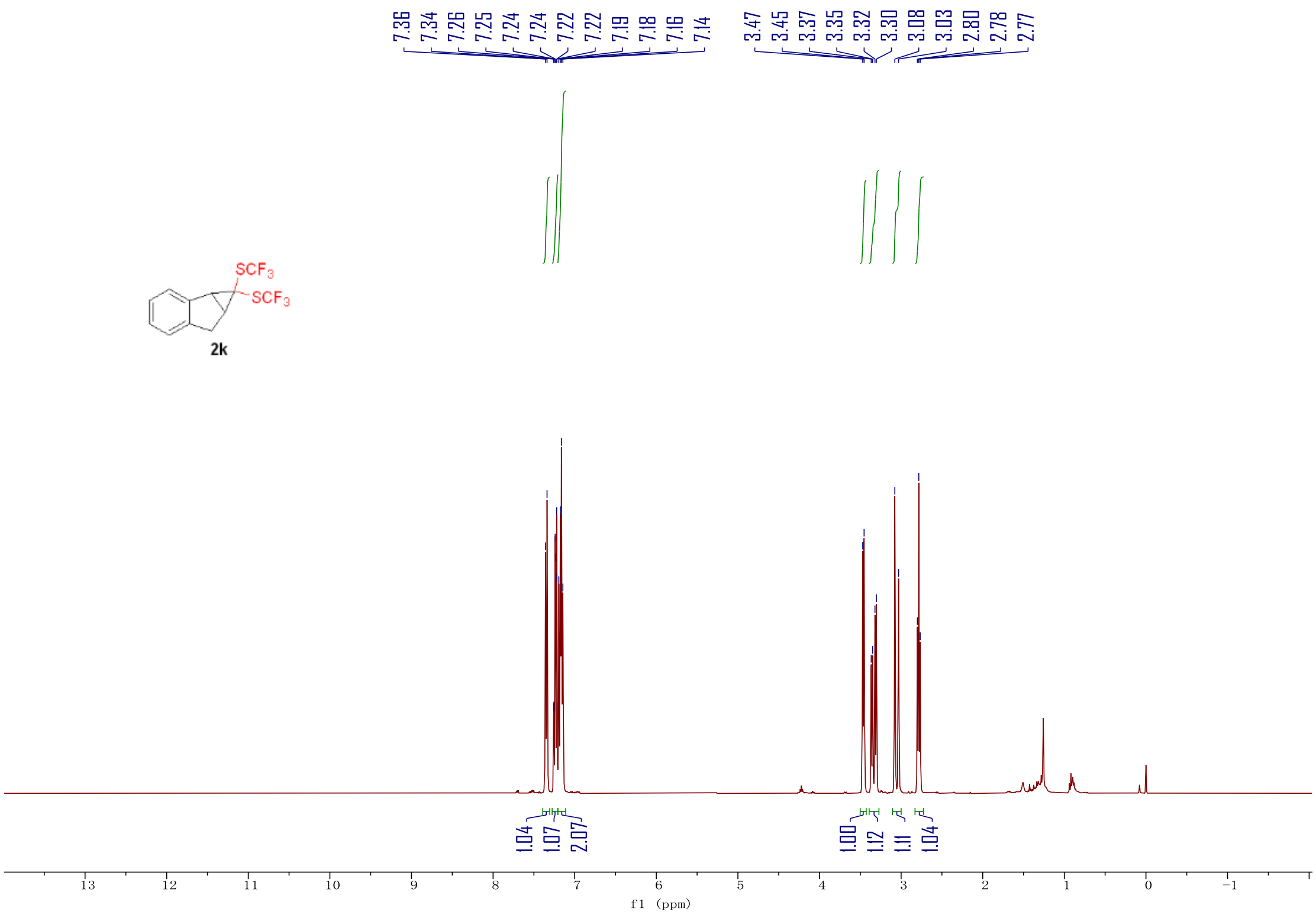

^{19}F NMR Spectrum of **2k** (376 MHz, $CDCl_3$)

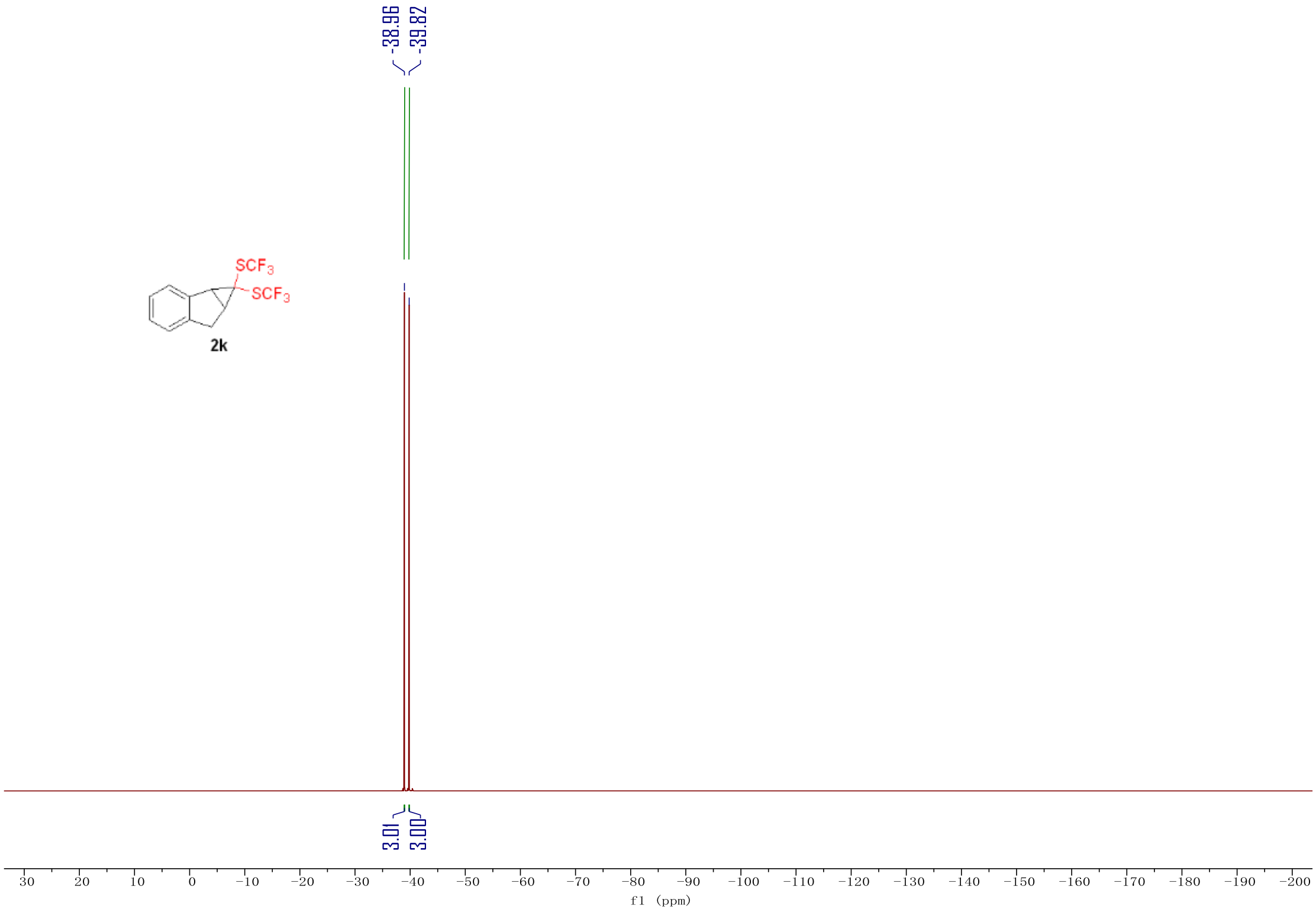

^{13}C NMR Spectrum of **2k** (101 MHz, $CDCl_3$)

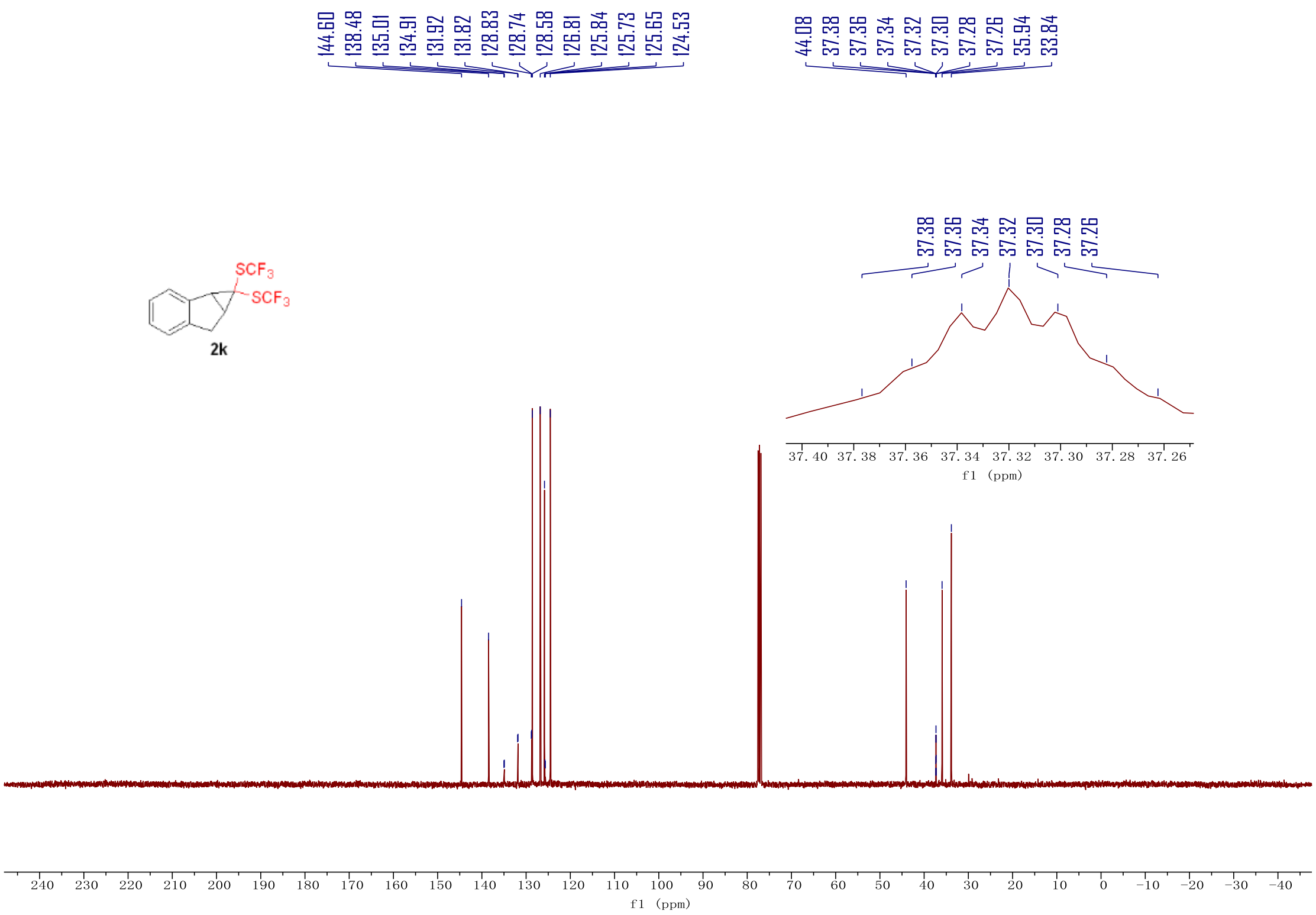

^{1}H NMR Spectrum of **2l** (400 MHz, $CDCl_3$)

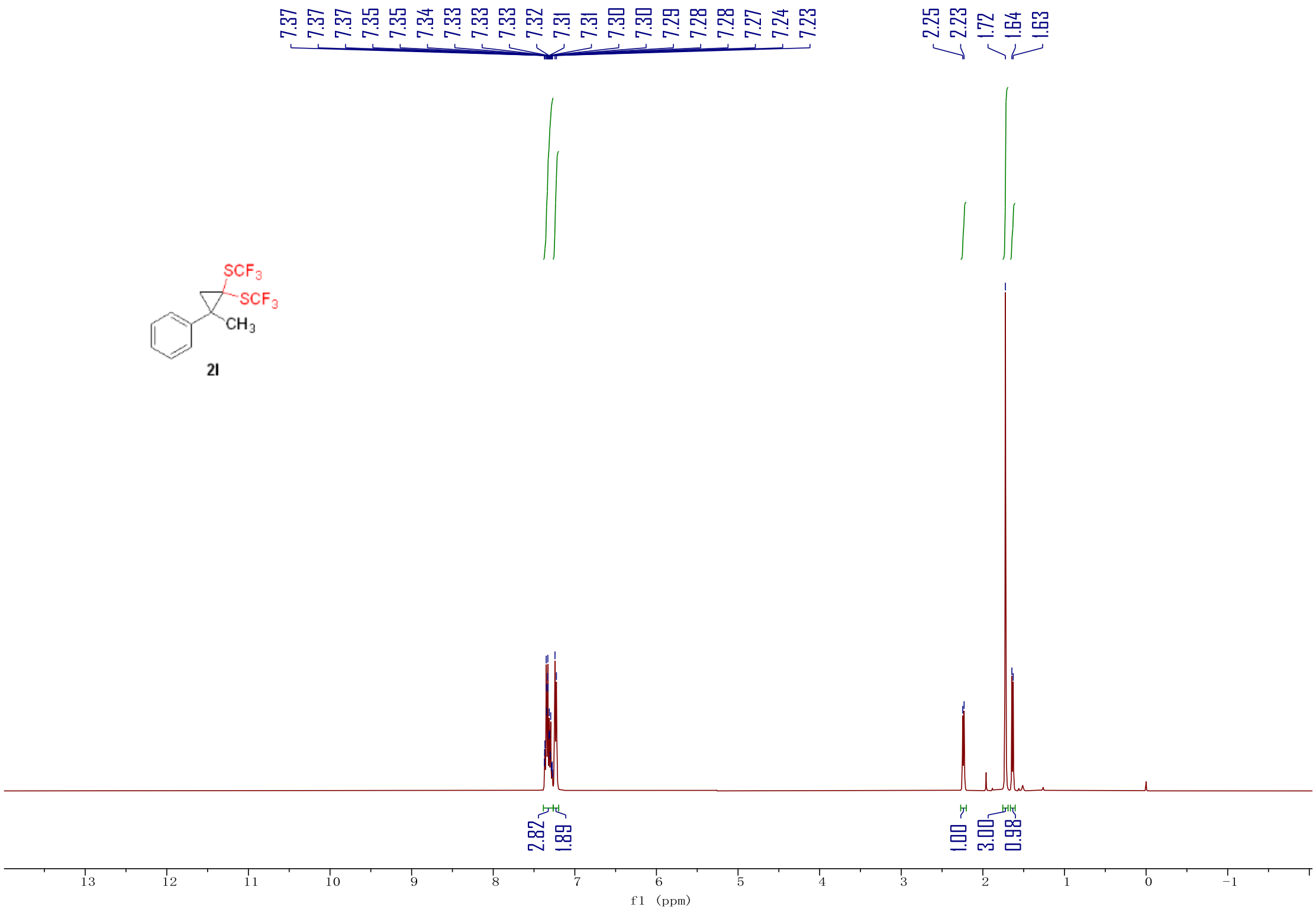

^{19}F NMR Spectrum of **2l** (376 MHz, $CDCl_3$)

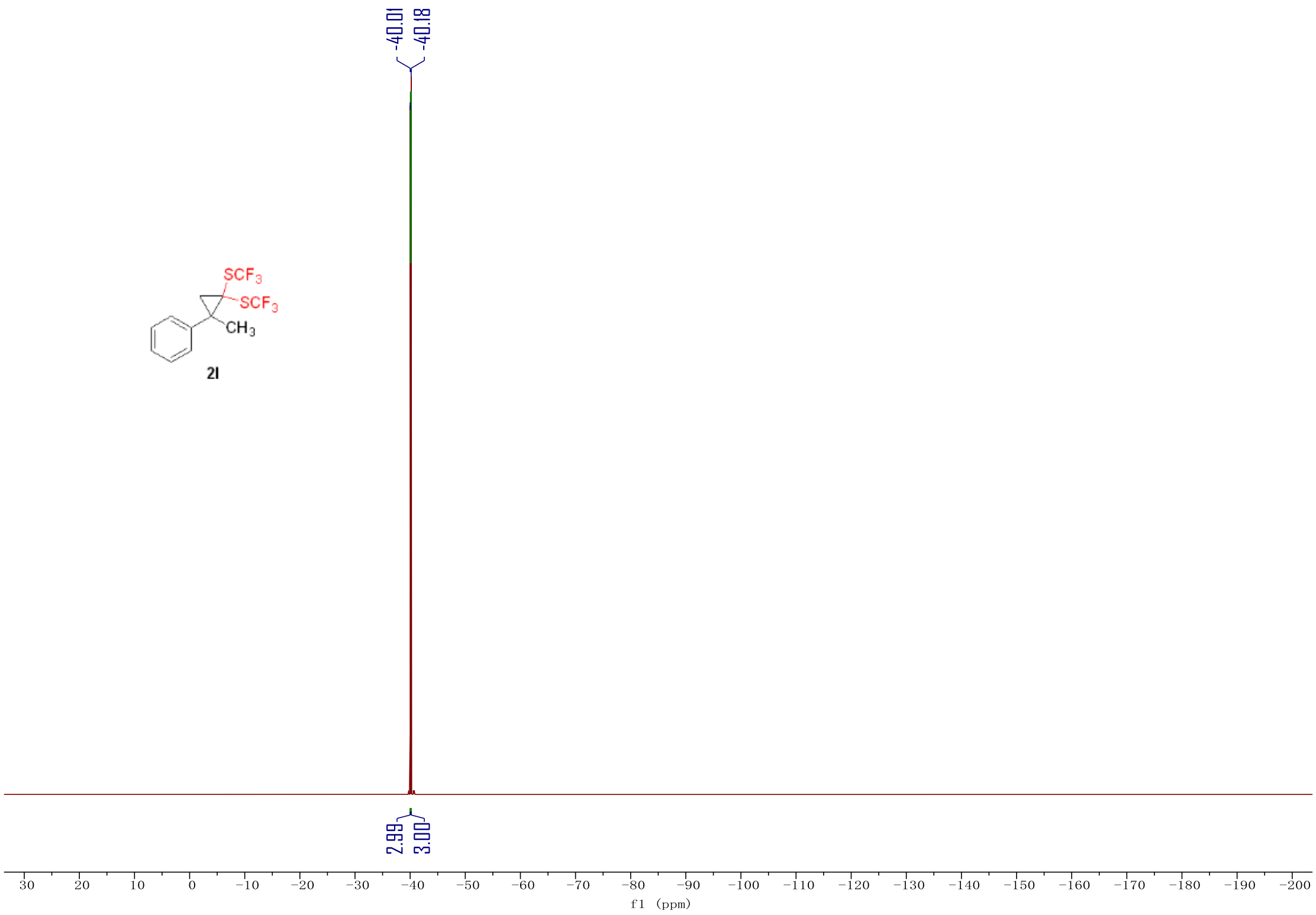

^{13}C NMR Spectrum of **2l** (101 MHz, $CDCl_3$)

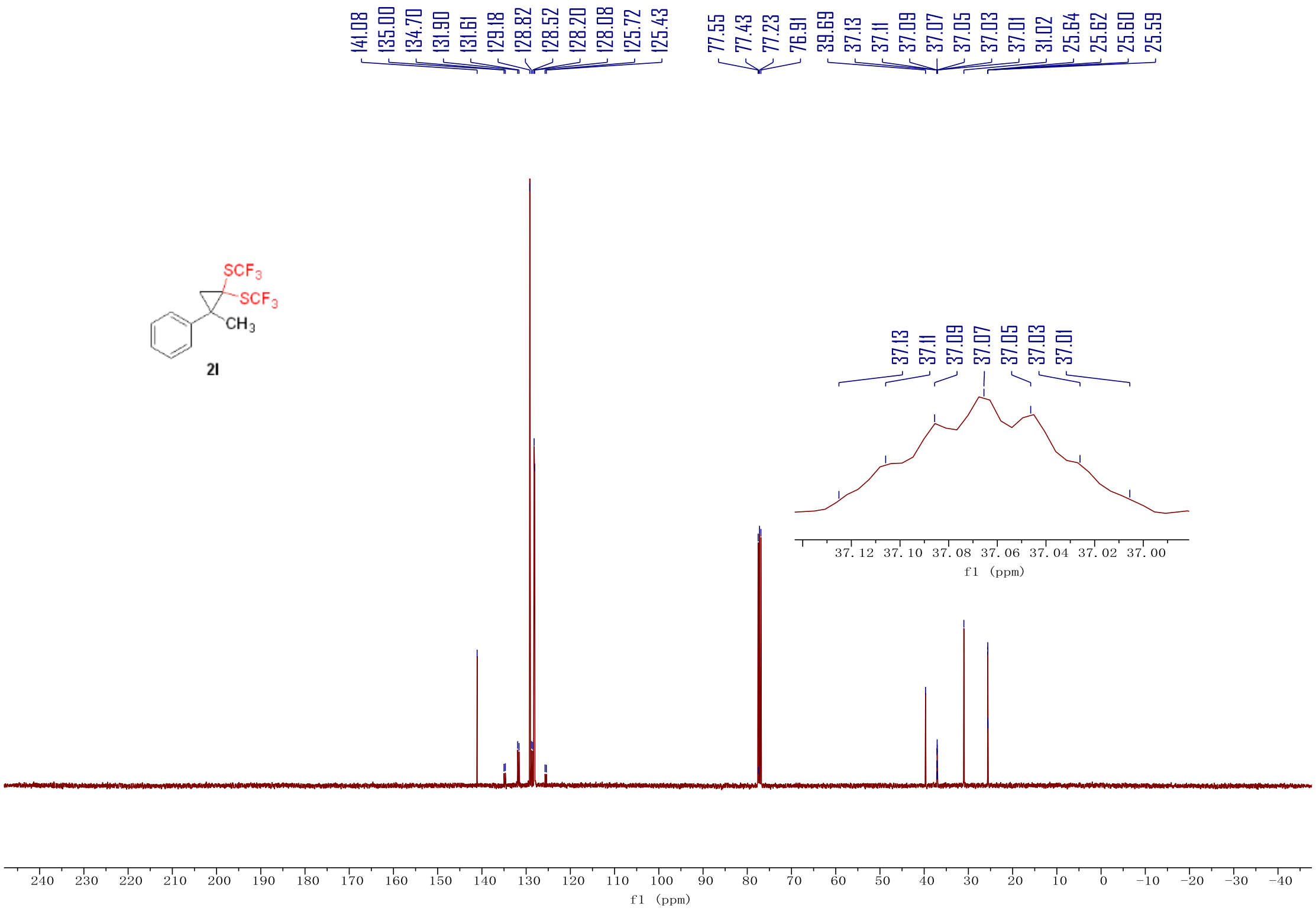

^{1}H NMR Spectrum of **2m** (400 MHz, $CDCl_3$)

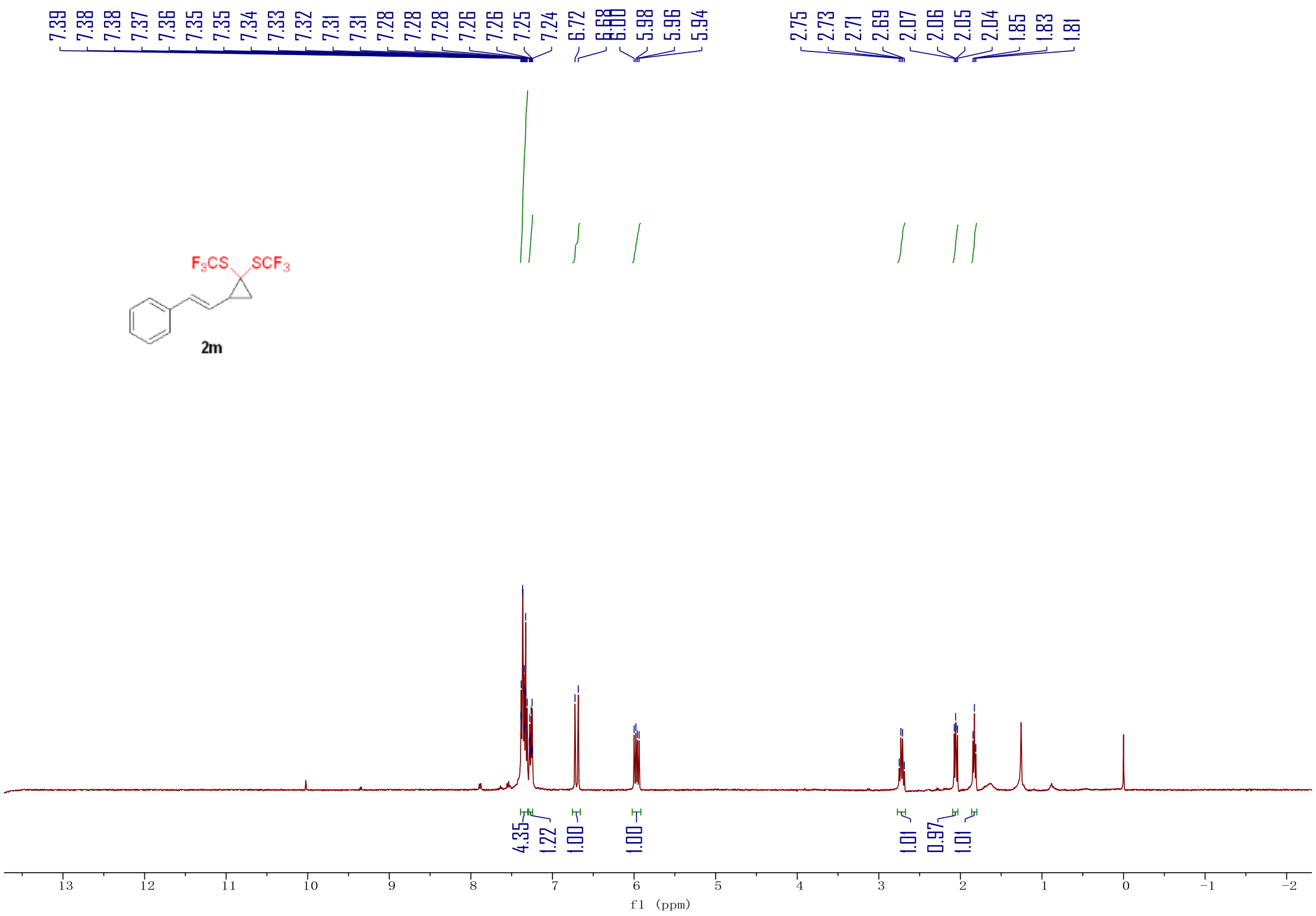

^{19}F NMR Spectrum of **2m** (376 MHz, $CDCl_3$)

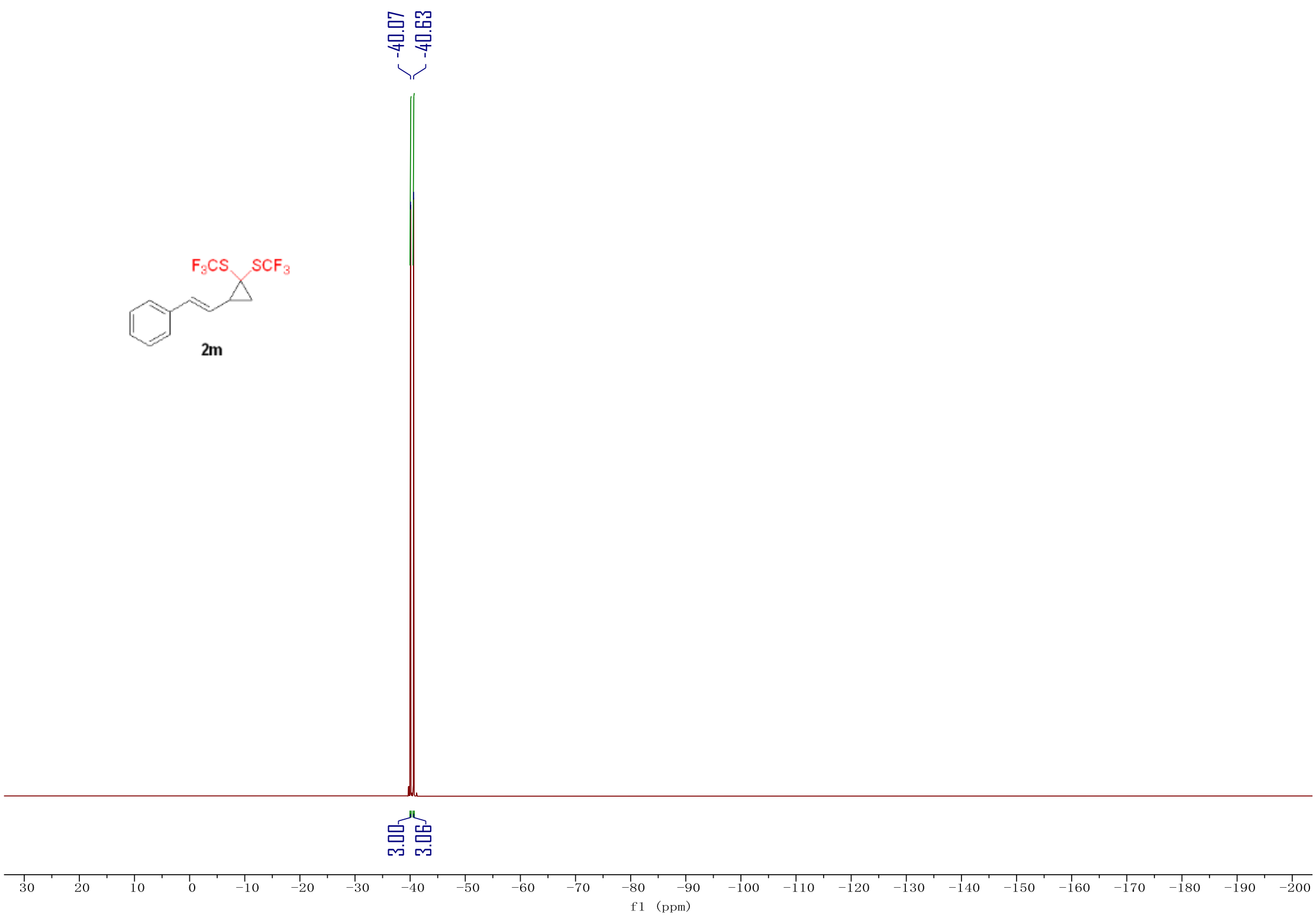

^{13}C NMR Spectrum of **2m** (101 MHz, $CDCl_3$)

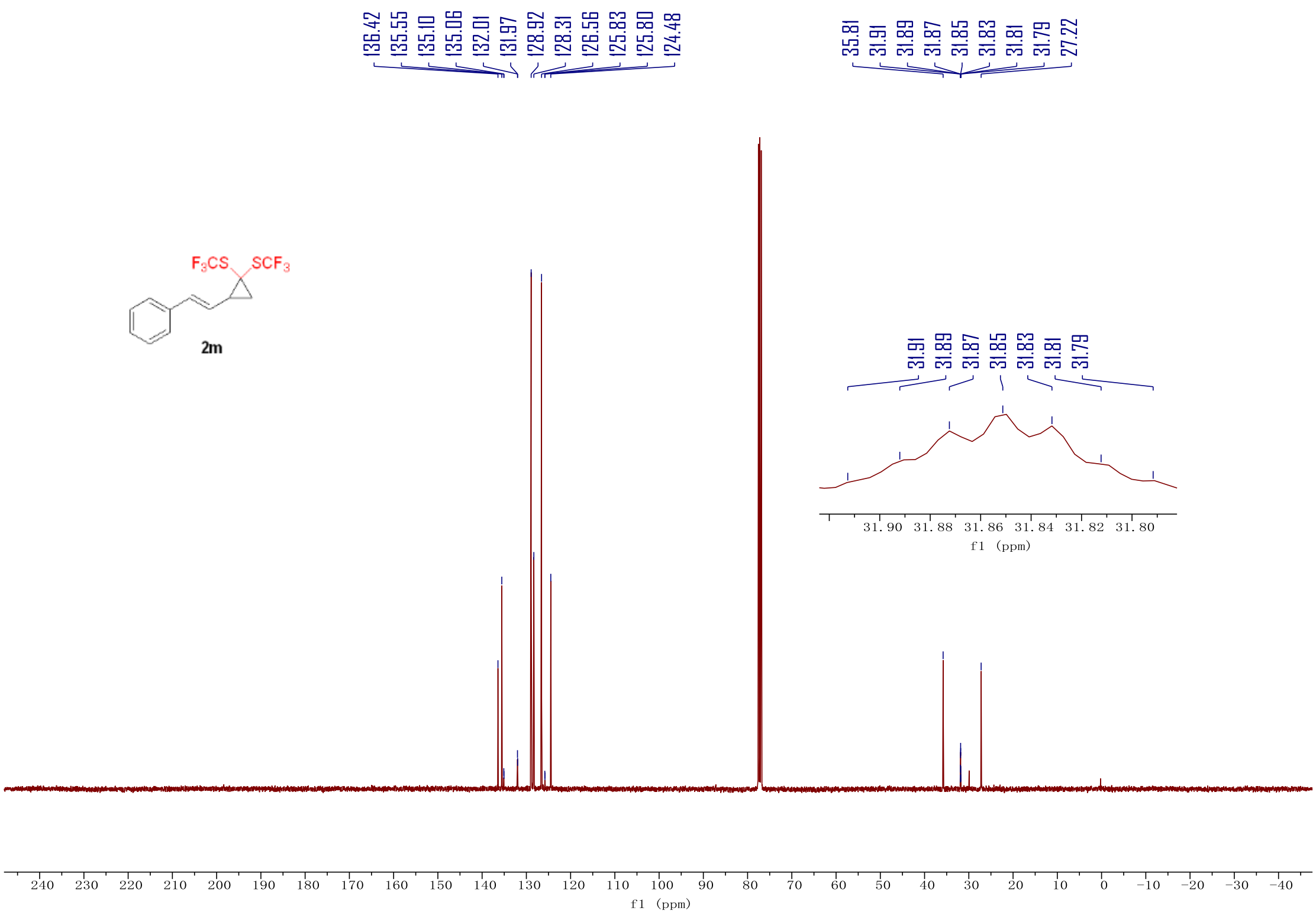

^{1}H NMR Spectrum of **2n** (400 MHz, $CDCl_3$)

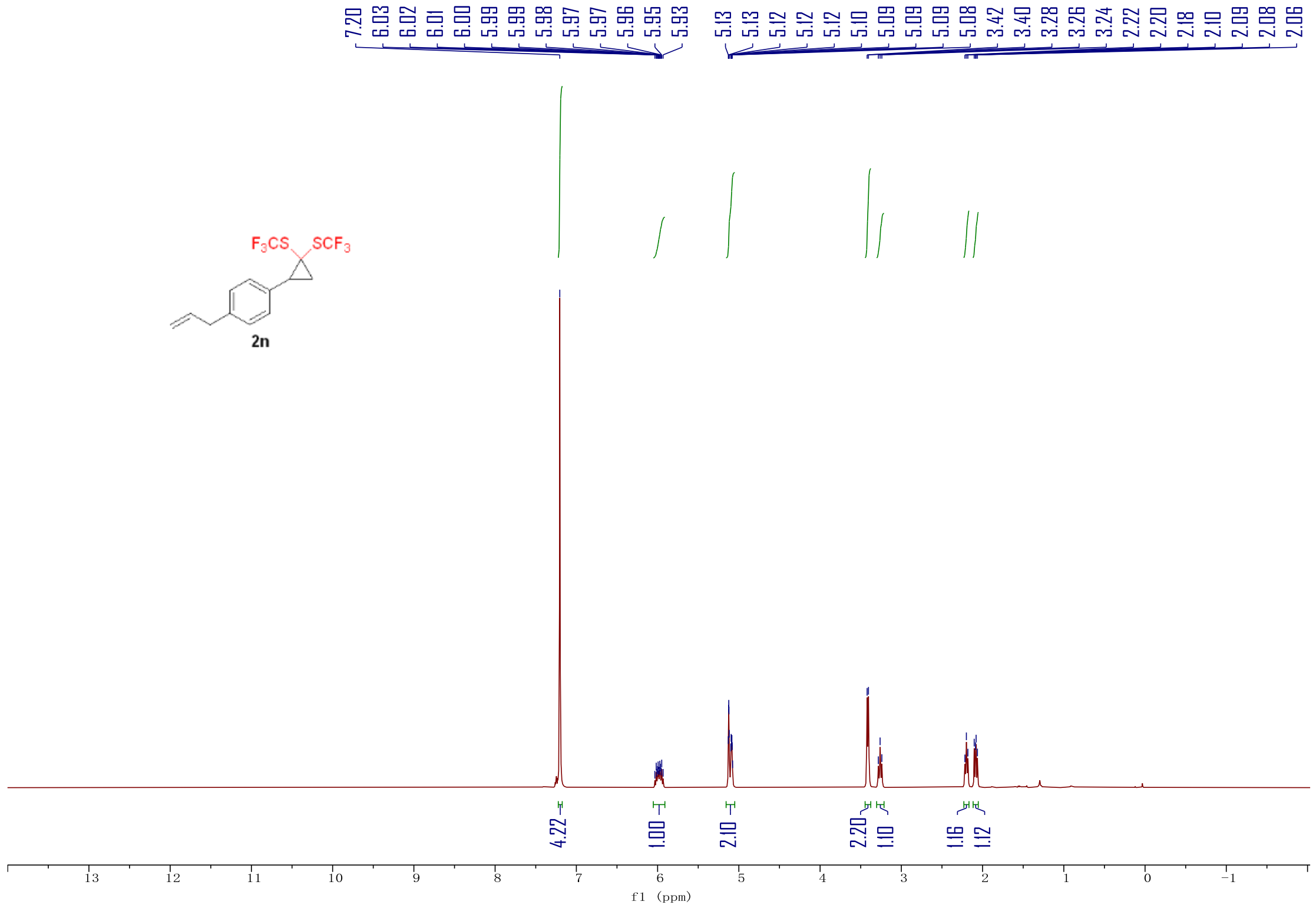

^{19}F NMR Spectrum of **2n** (376 MHz, $CDCl_3$)

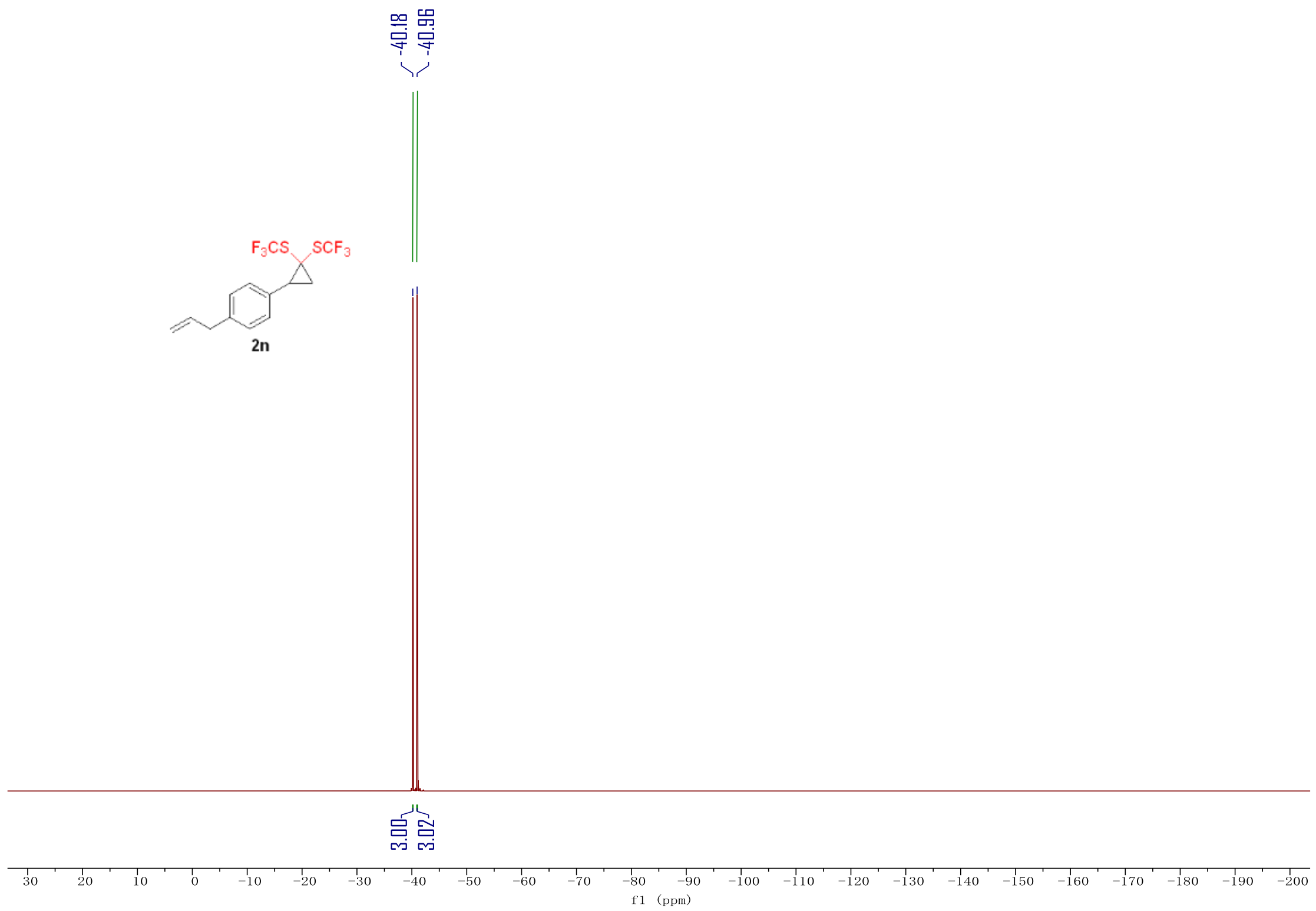

^{13}C NMR Spectrum of **2n** (101 MHz, $CDCl_3$)

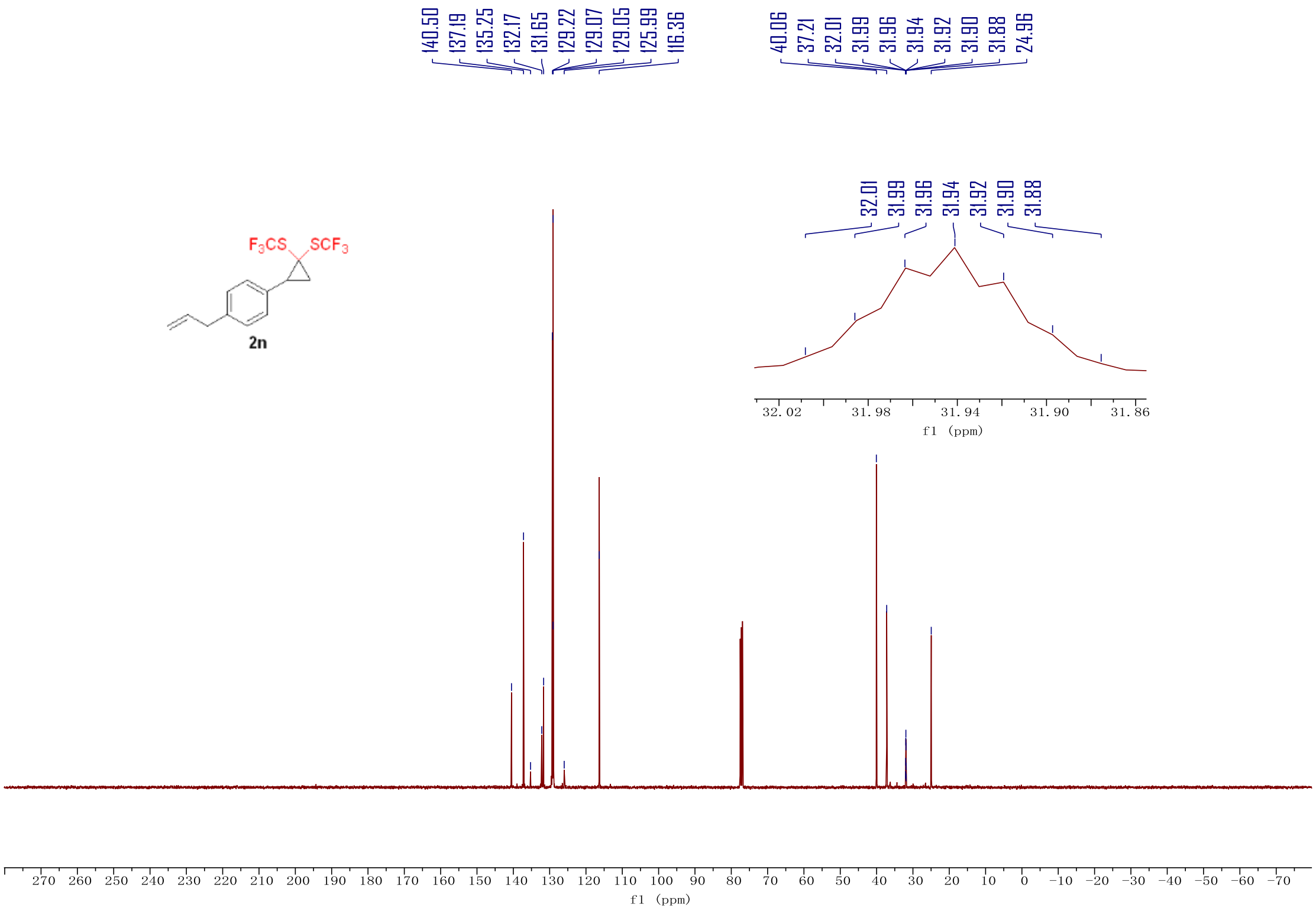

^{1}H NMR Spectrum of **2o** (400 MHz, $CDCl_3$)

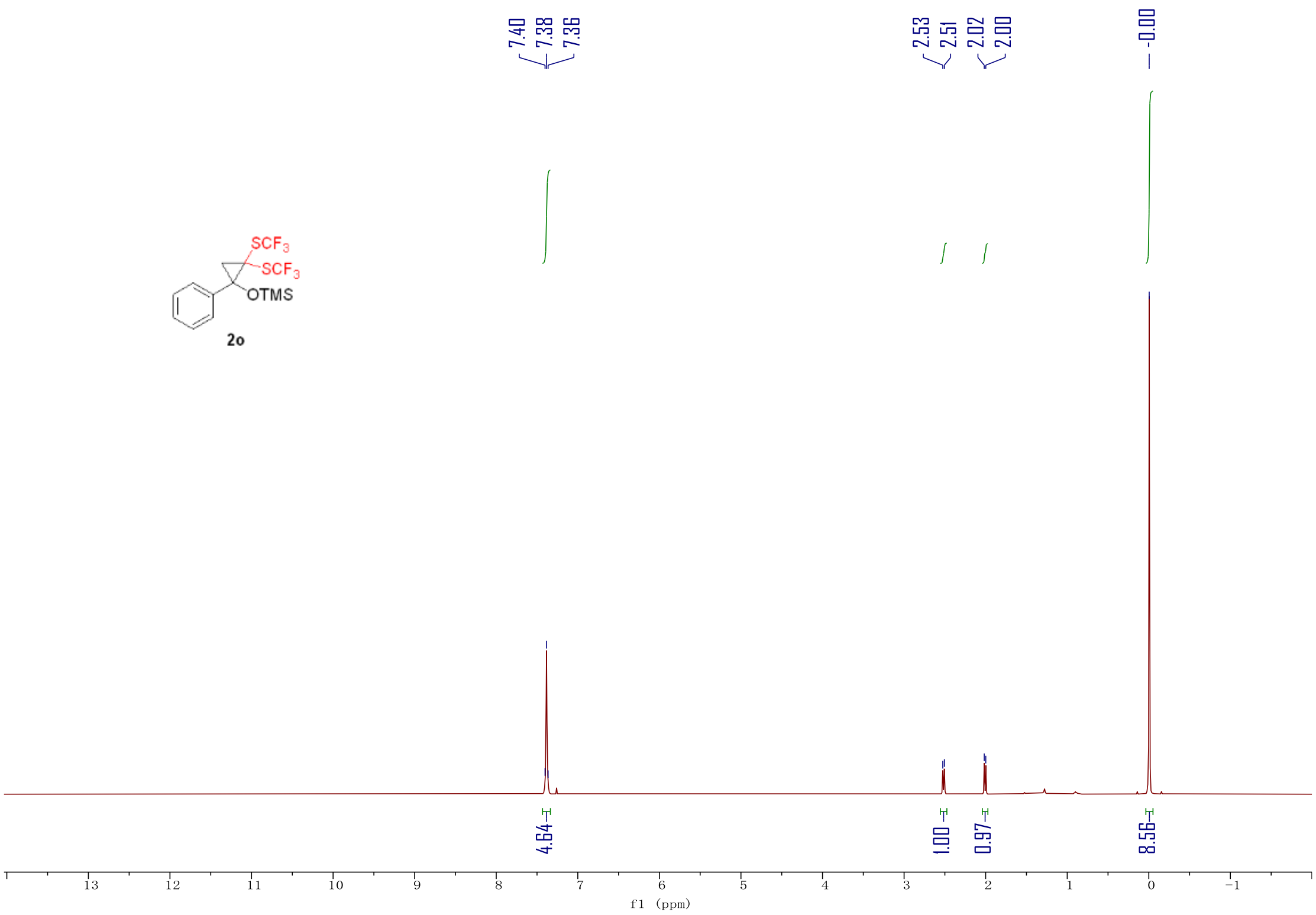

^{19}F NMR Spectrum of **2o** (376 MHz, $CDCl_3$)

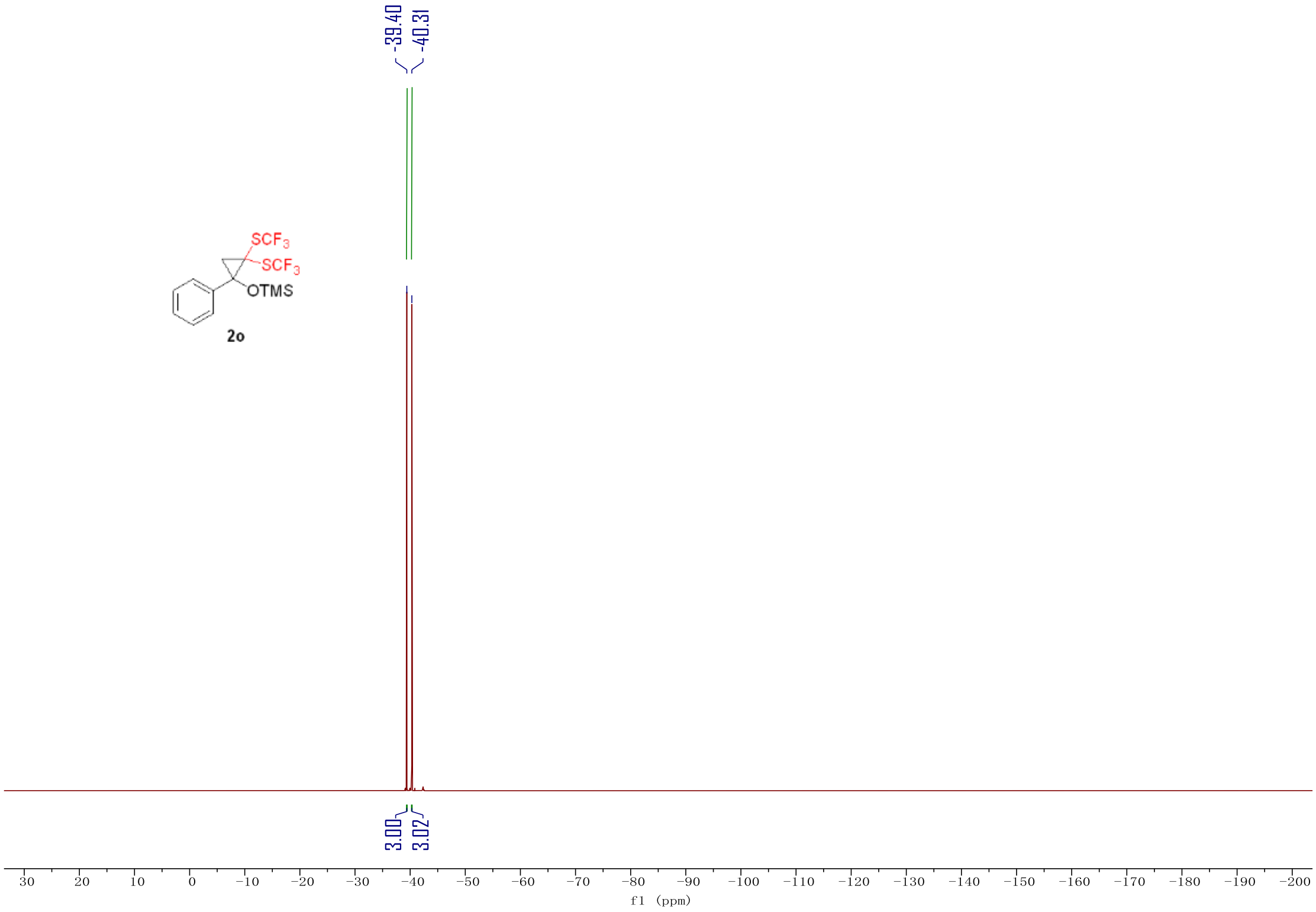

^{13}C NMR Spectrum of **2o** (101 MHz, $CDCl_3$)

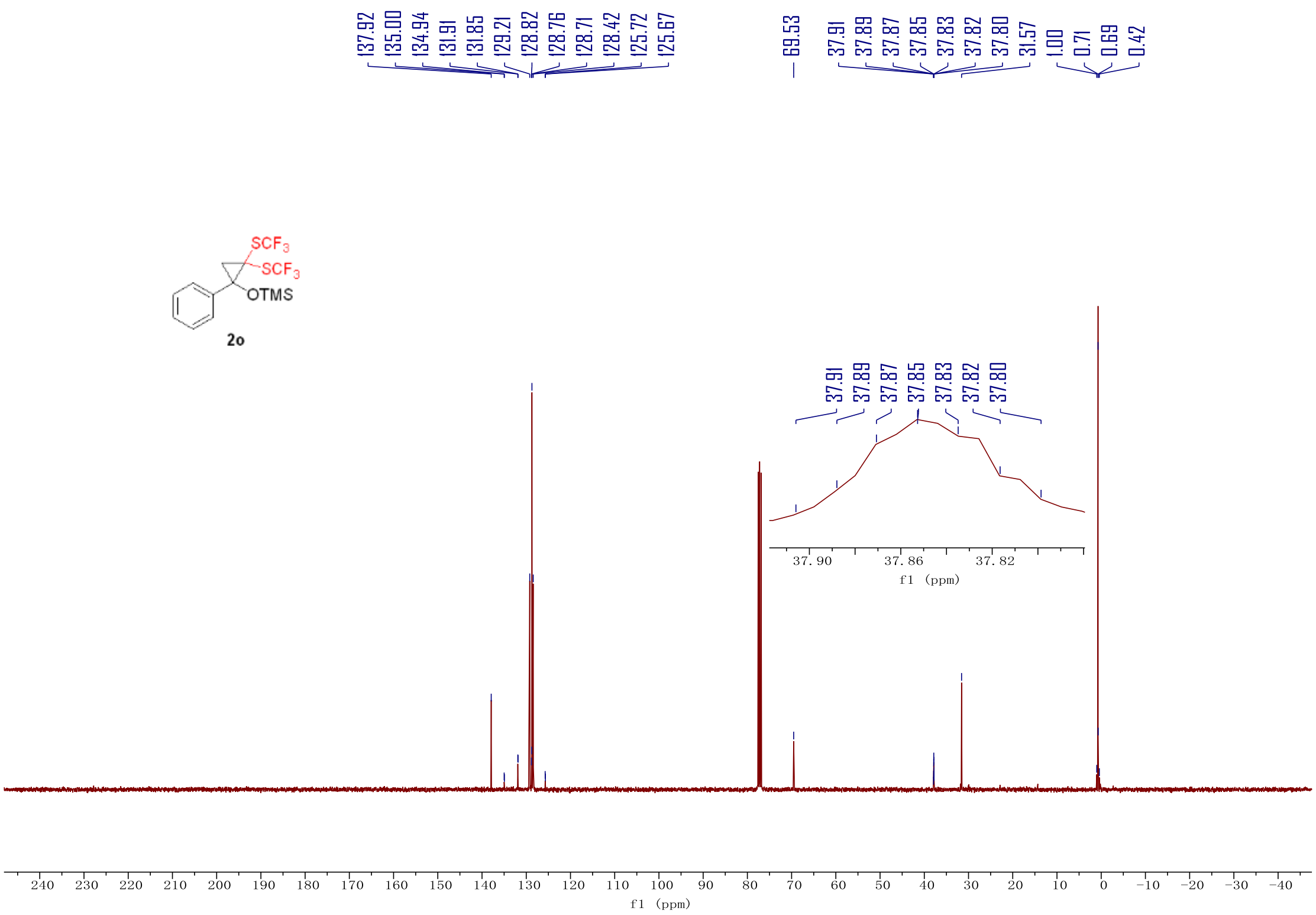

^{1}**H NMR** Spectrum of **2p** (400 MHz, $CDCl_3$)

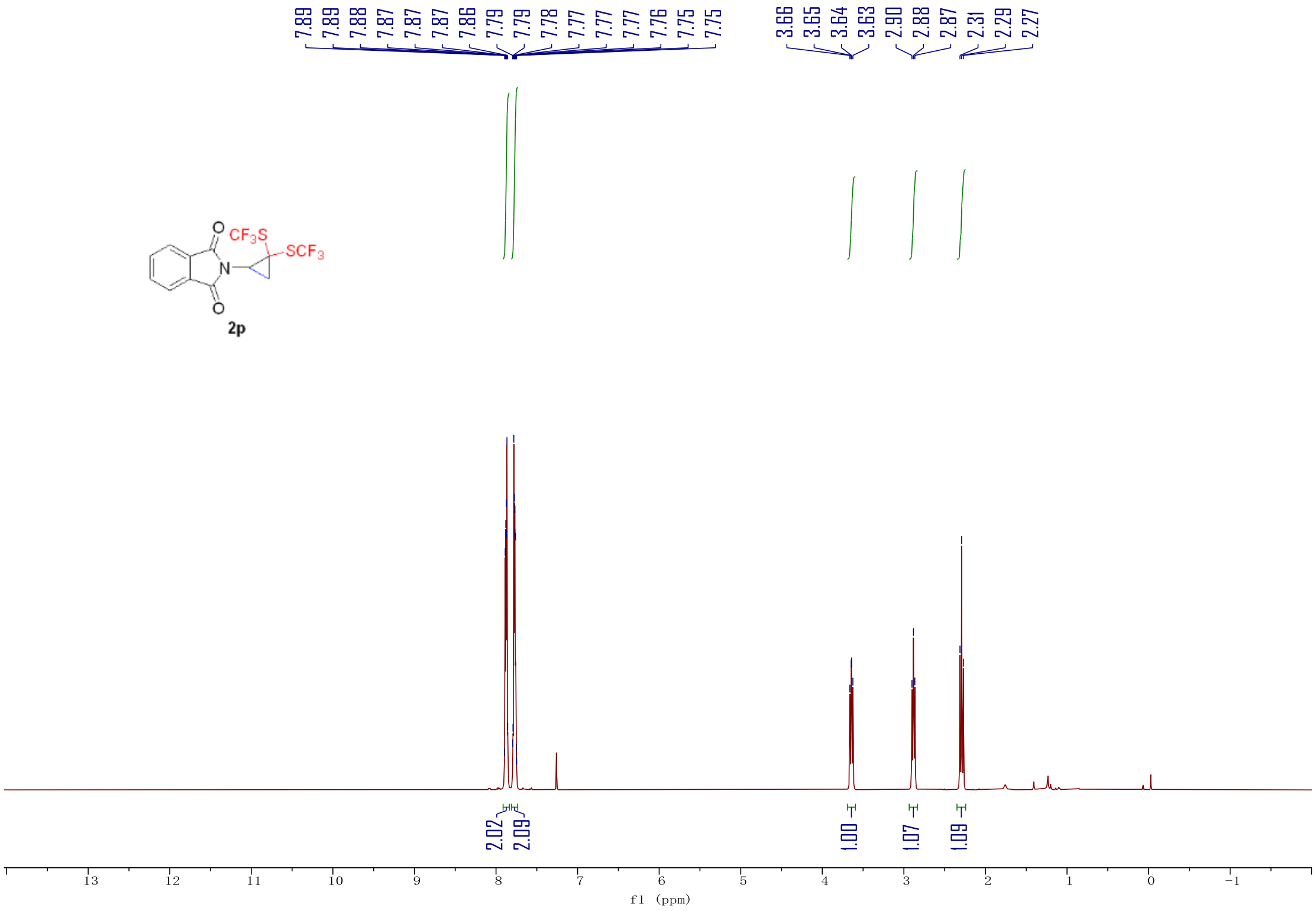

^{19}**F NMR** Spectrum of **2p** (376 MHz, $CDCl_3$)

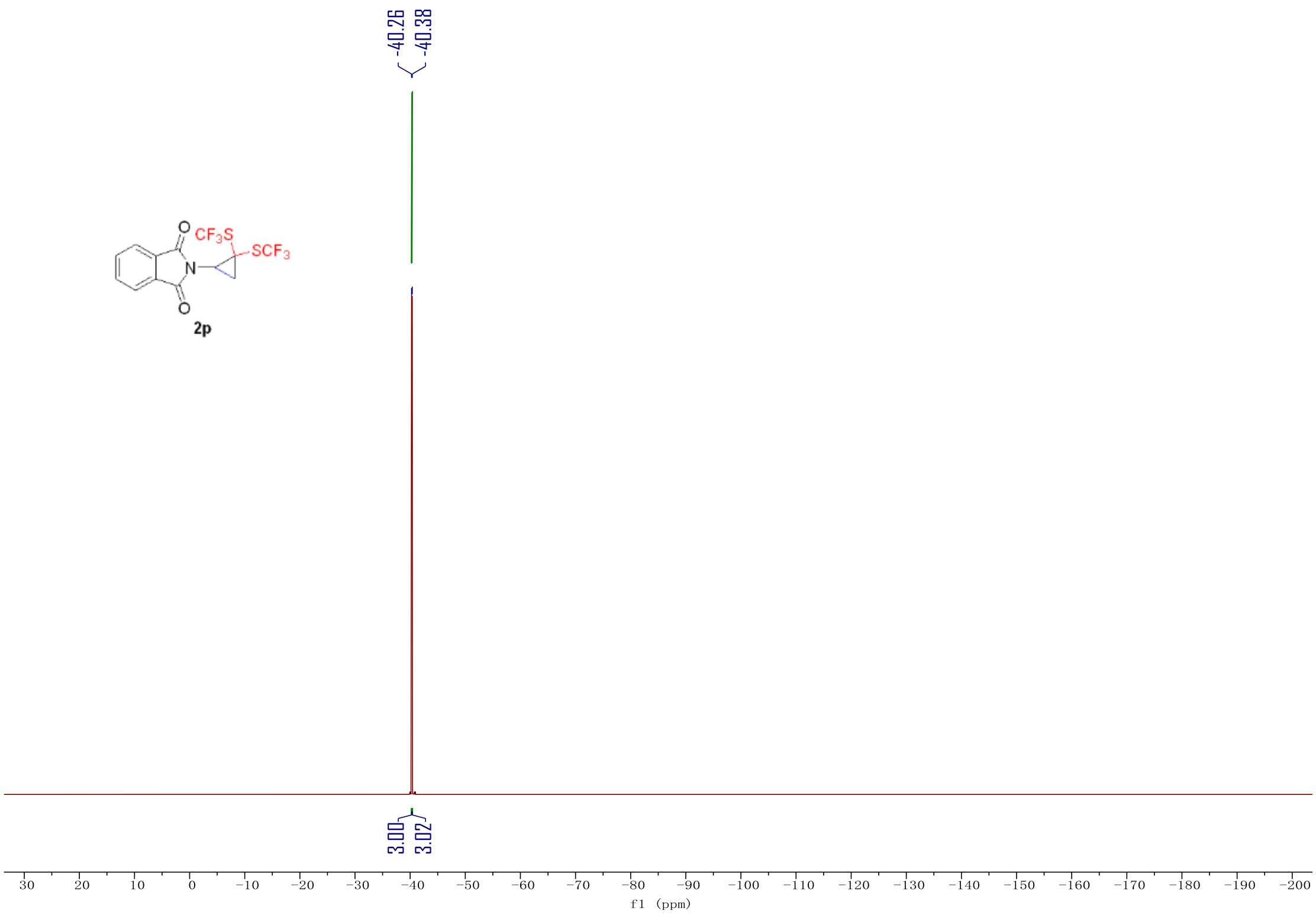

^{13}C NMR Spectrum of **2p** (101 MHz, $CDCl_3$)

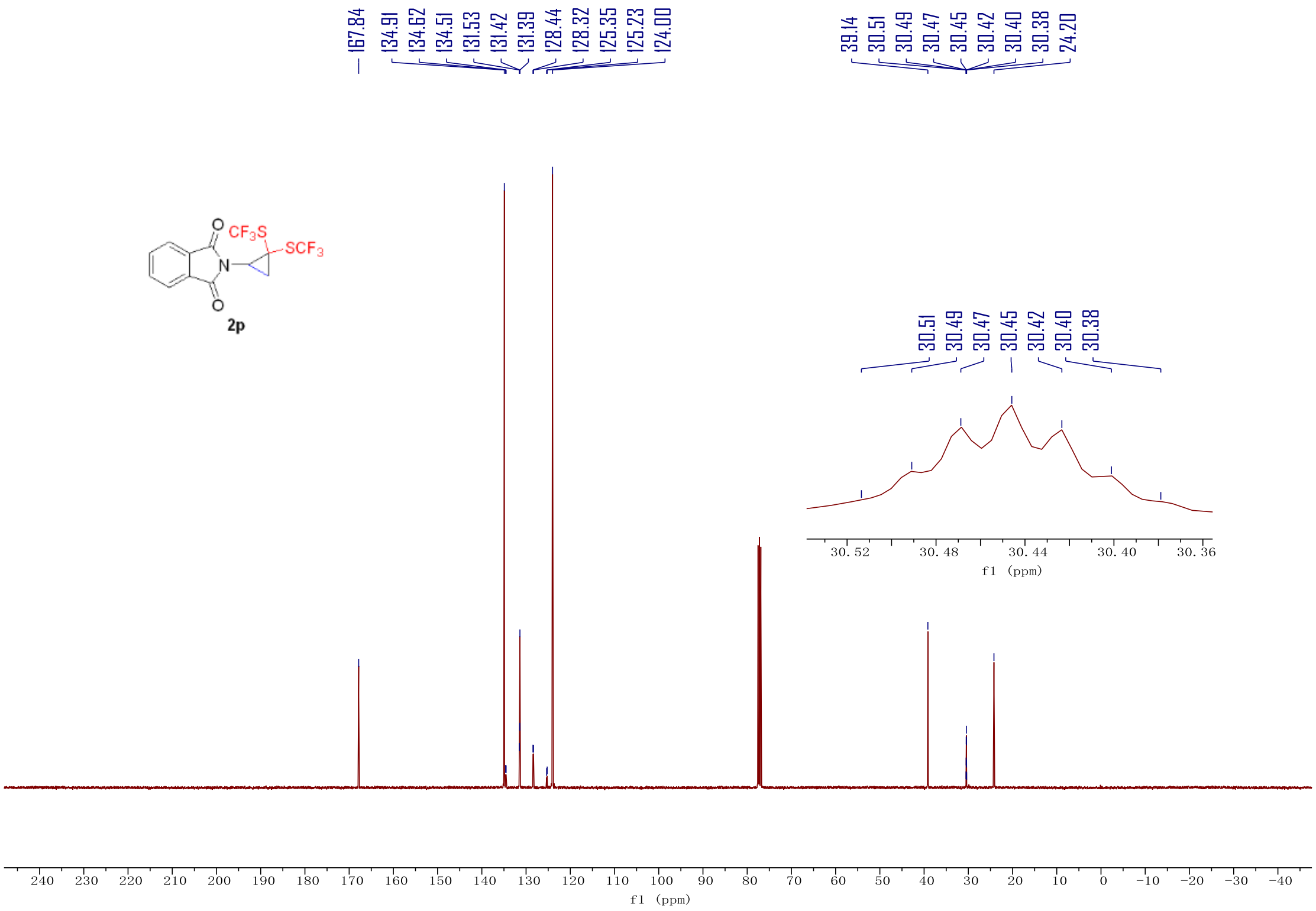

^{1}H NMR Spectrum of **2q** (400 MHz, $CDCl_3$)

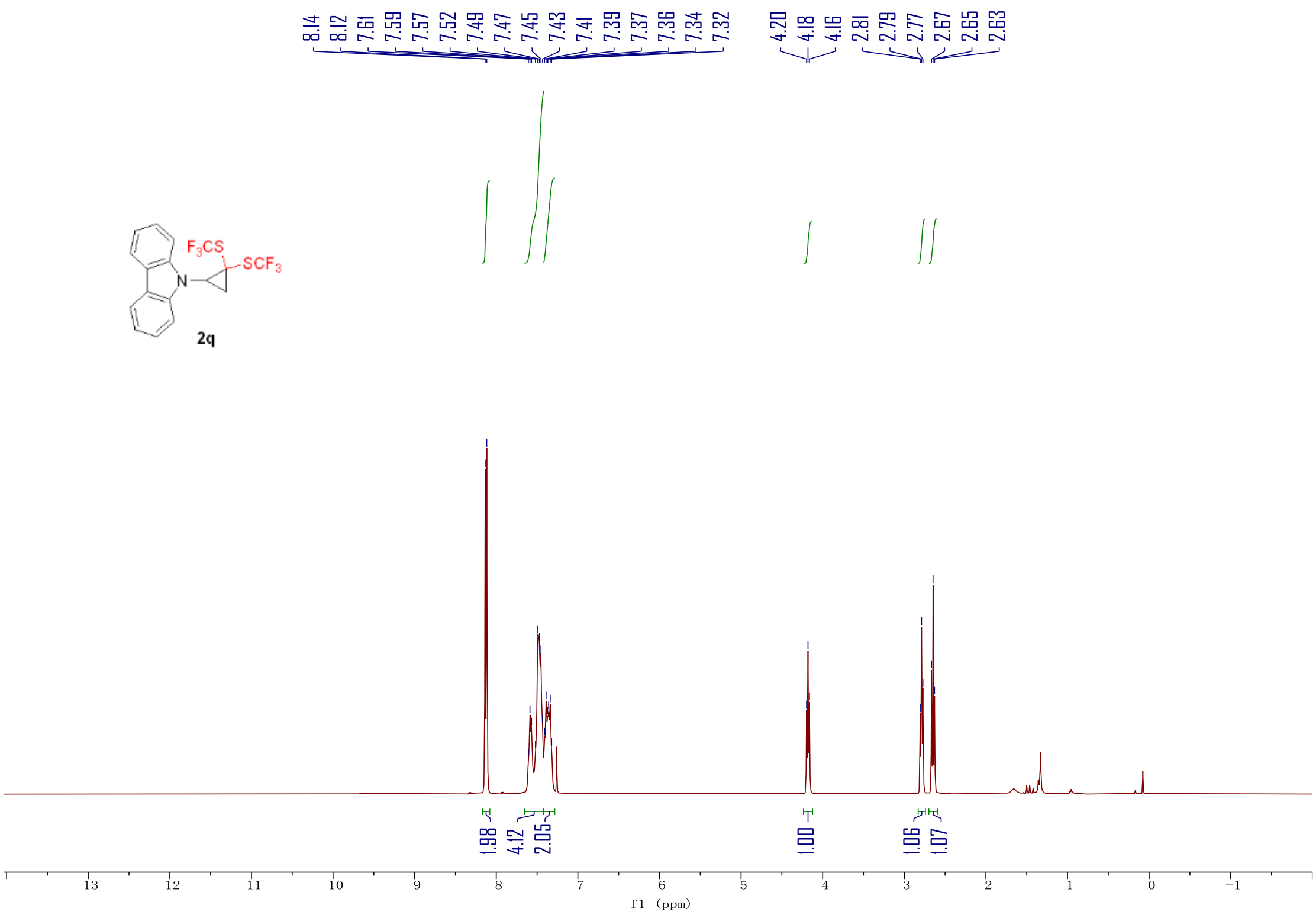

^{19}F NMR Spectrum of **2q** (376 MHz, $CDCl_3$)

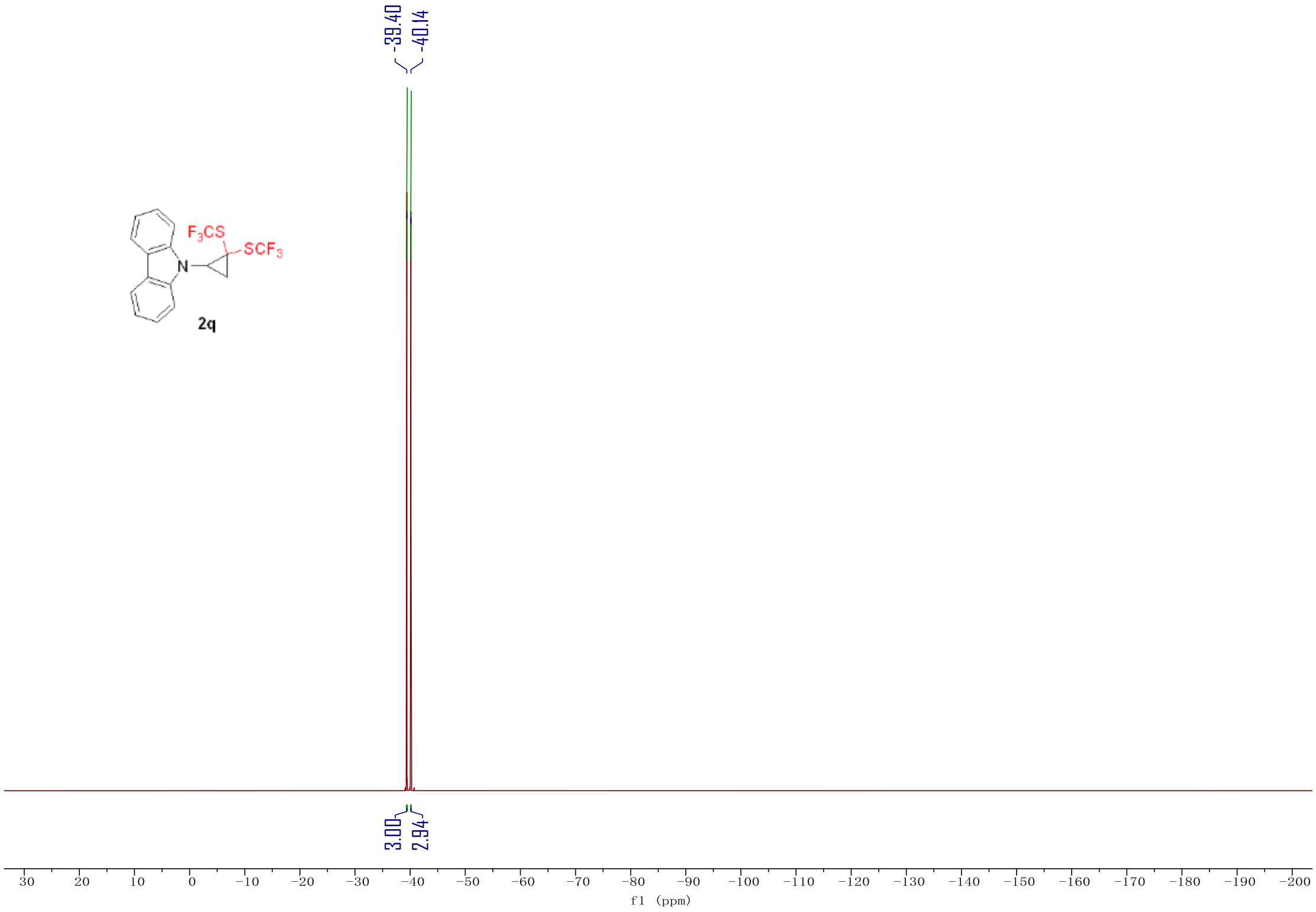

^{13}C NMR Spectrum of **2q** (101 MHz, $CDCl_3$)

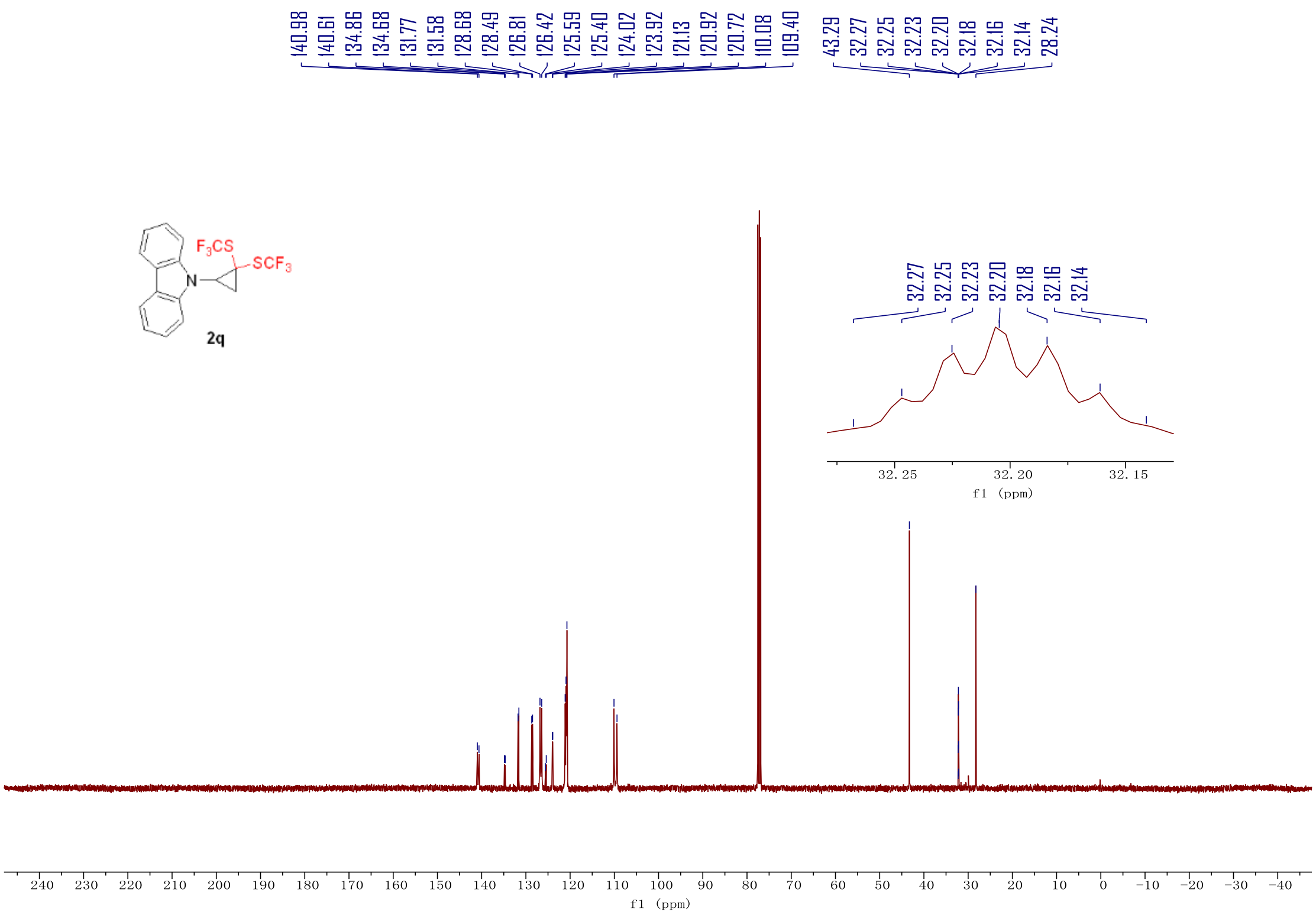

^{1}H NMR Spectrum of **2r** (400 MHz, $CDCl_3$)

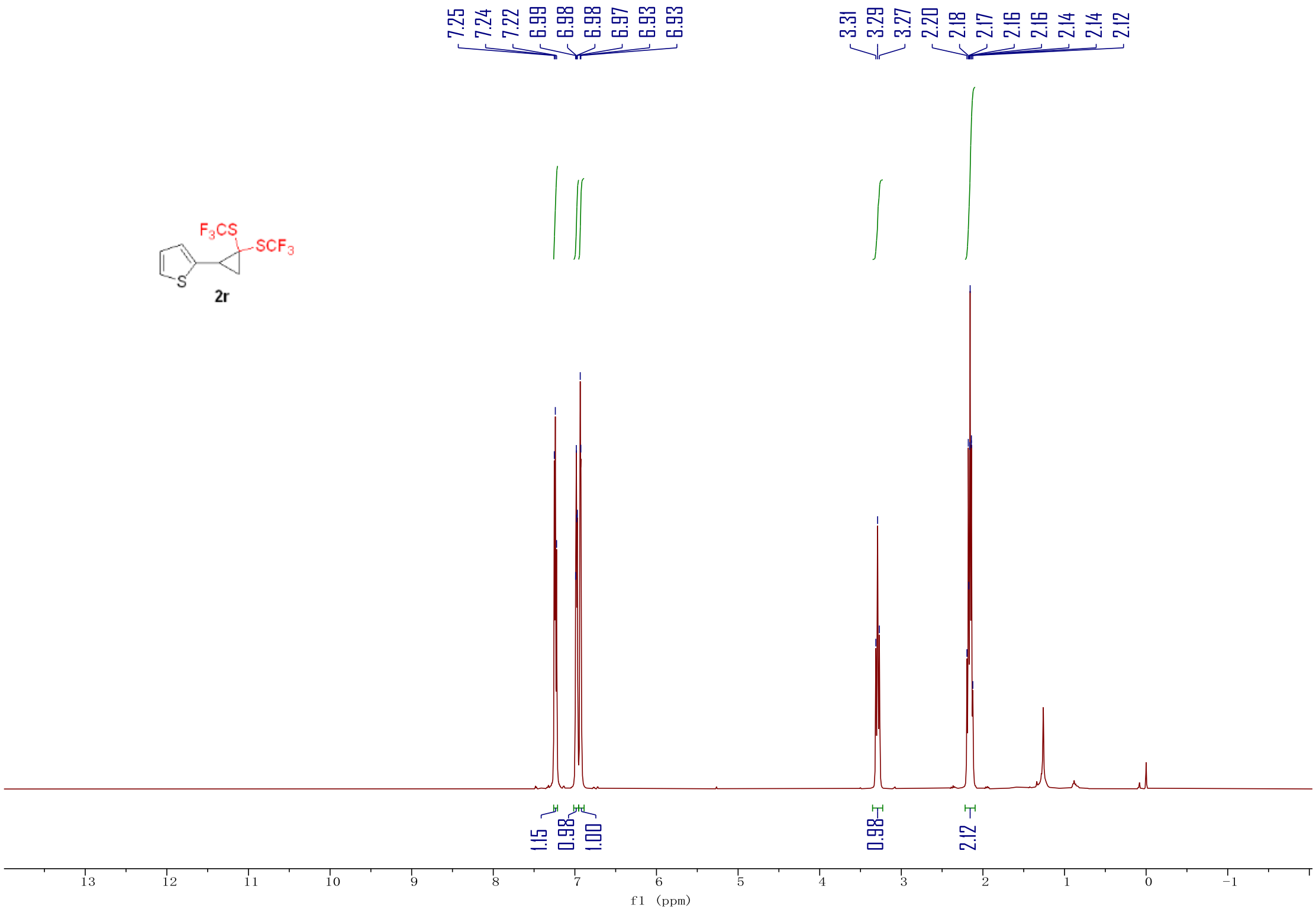

^{19}F NMR Spectrum of **2r** (376 MHz, $CDCl_3$)

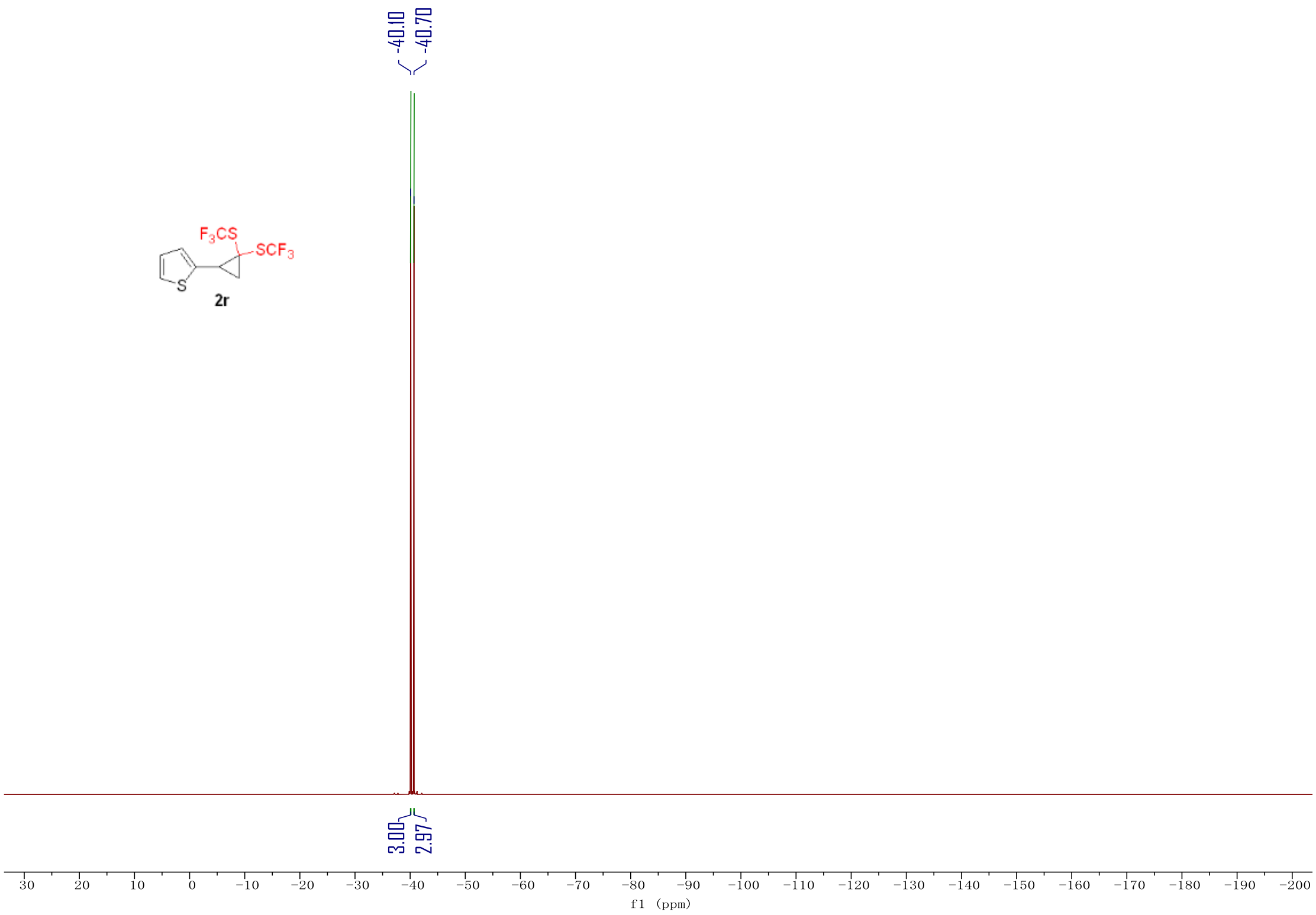

^{13}C NMR Spectrum of **2r** (101 MHz, $CDCl_3$)

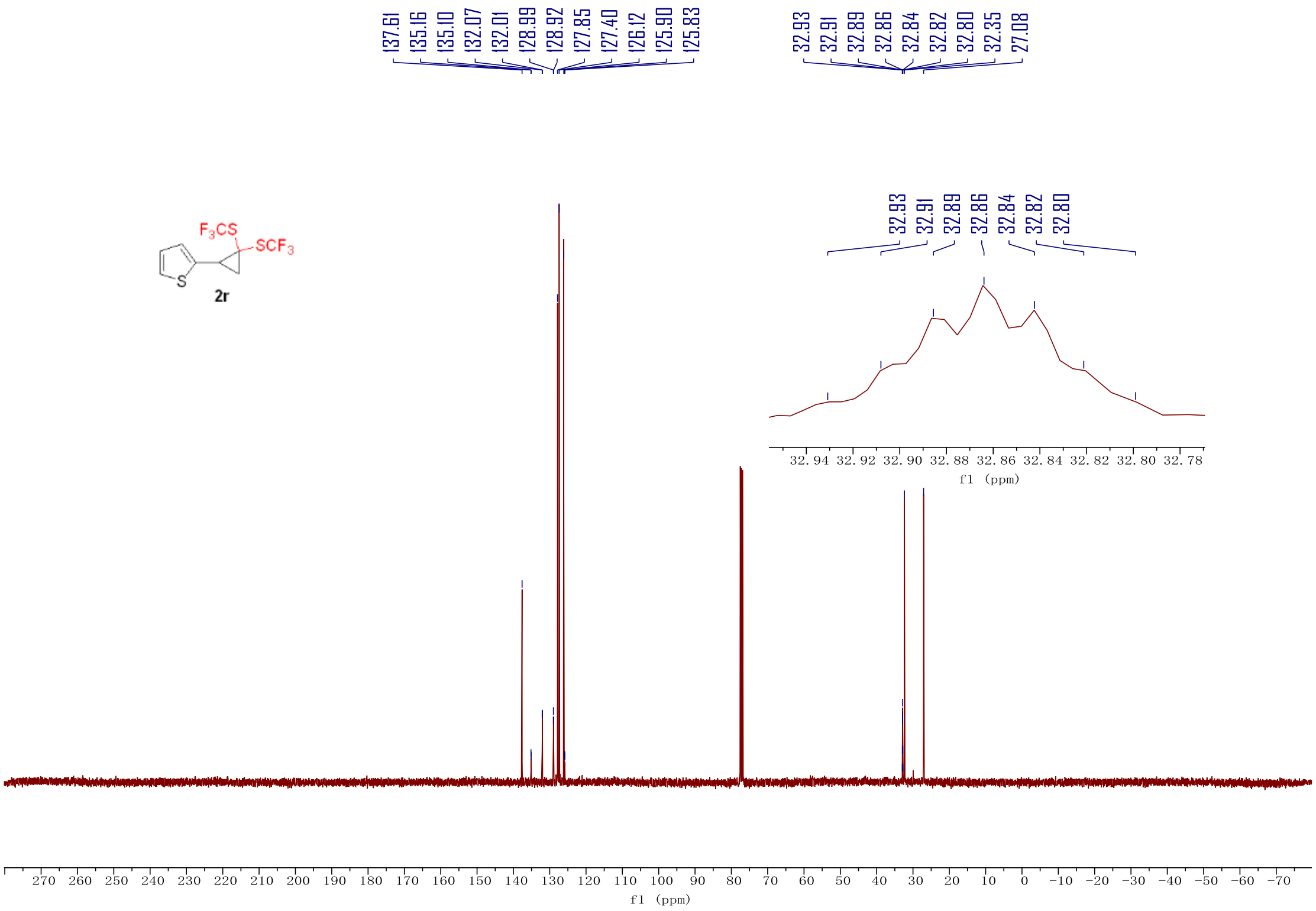

^{1}H NMR Spectrum of **2s** (400 MHz, $CDCl_3$)

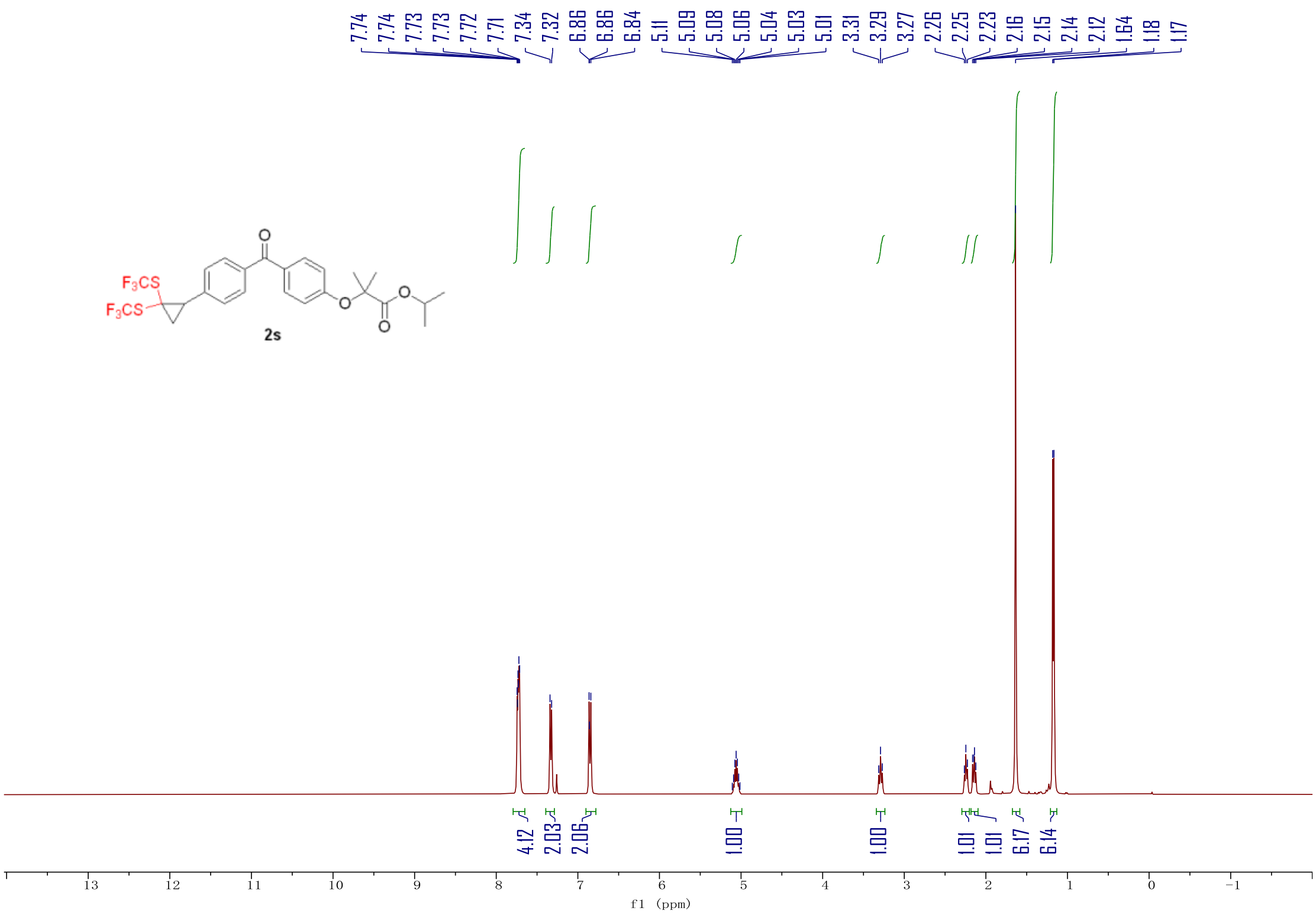

^{19}**F NMR** Spectrum of **2s** (376 MHz, $CDCl_3$)

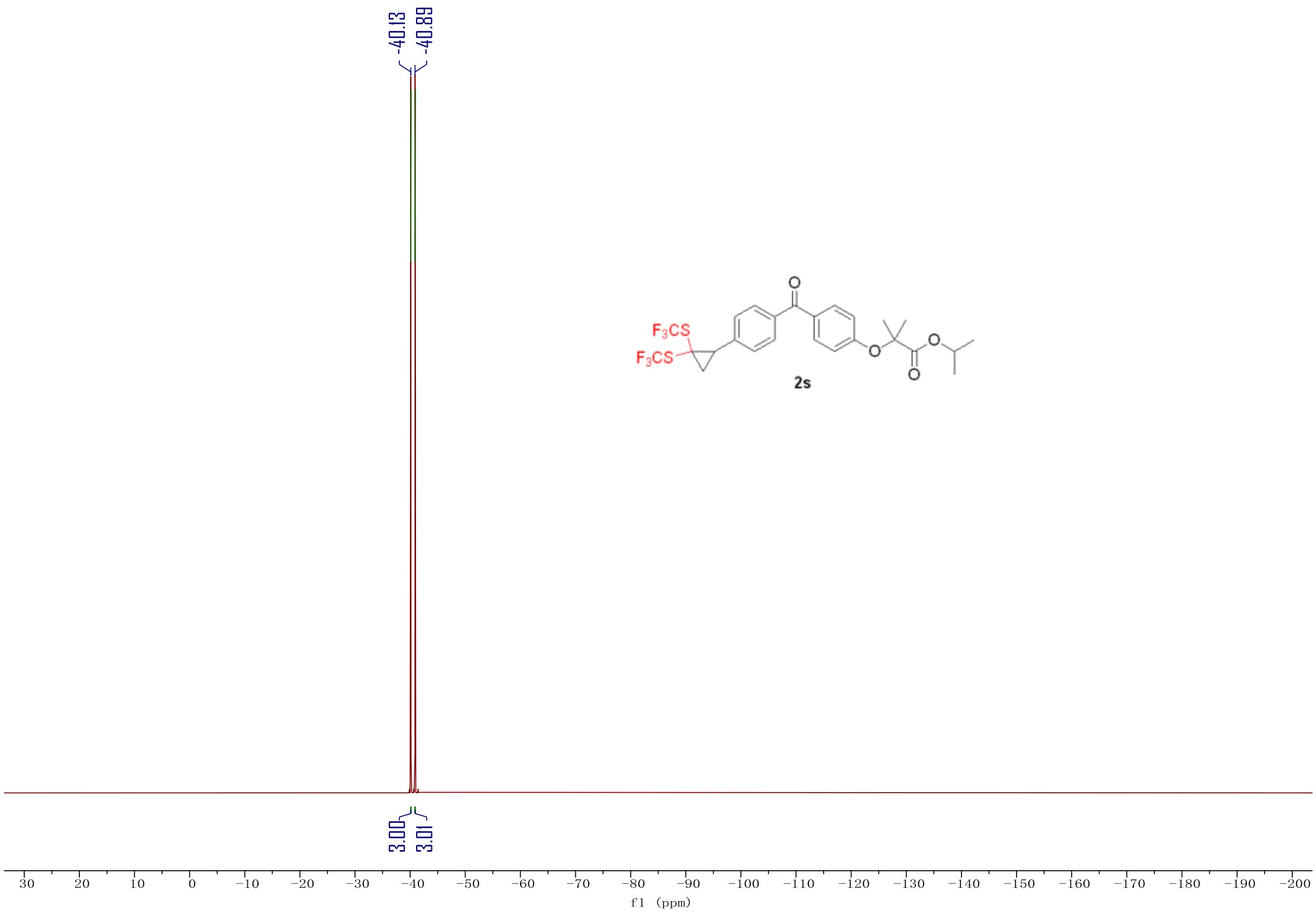

^{13}**C NMR** Spectrum of **2s** (101 MHz, $CDCl_3$)

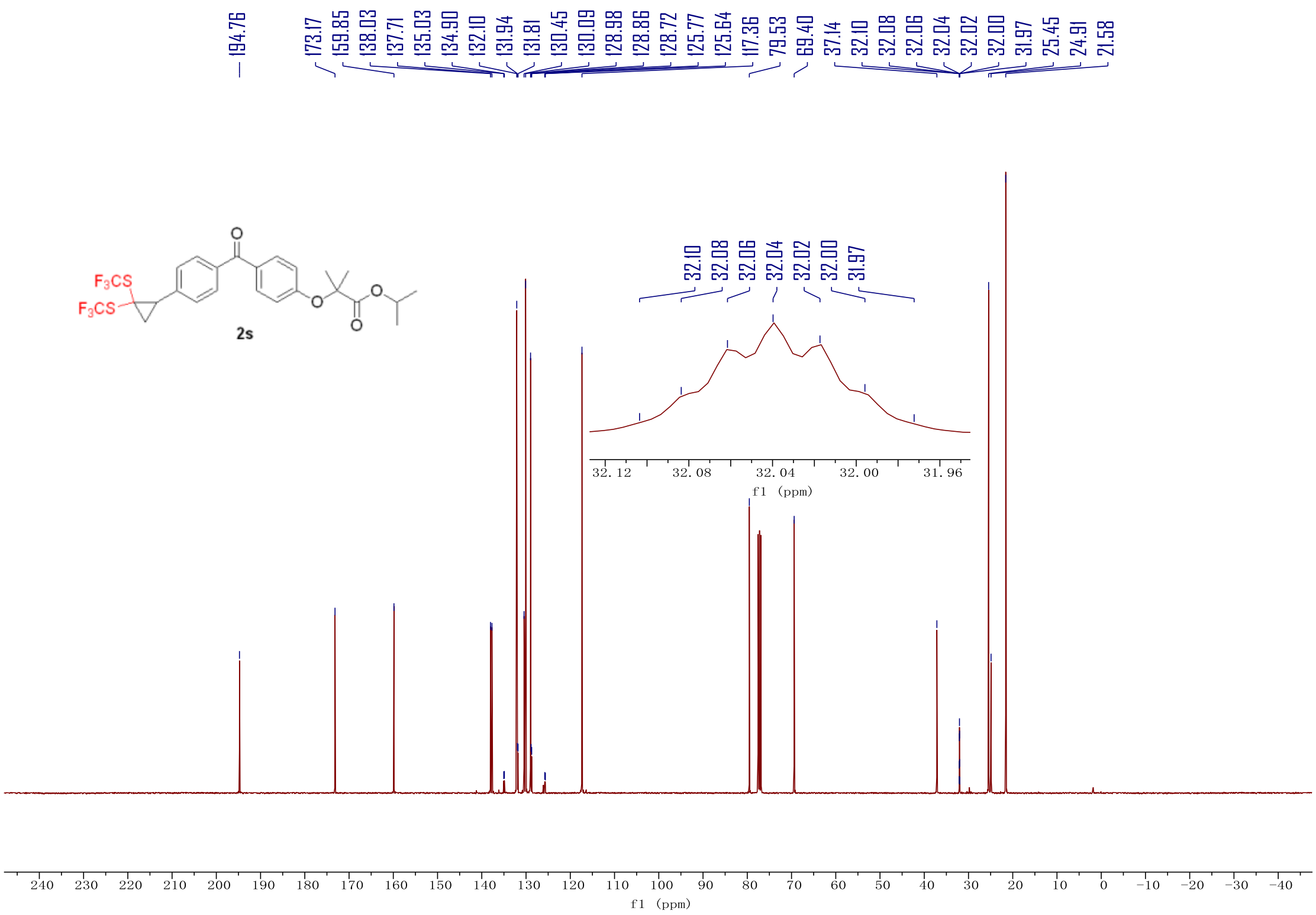

^{1}H NMR Spectrum of **2u** (400 MHz, $CDCl_3$)

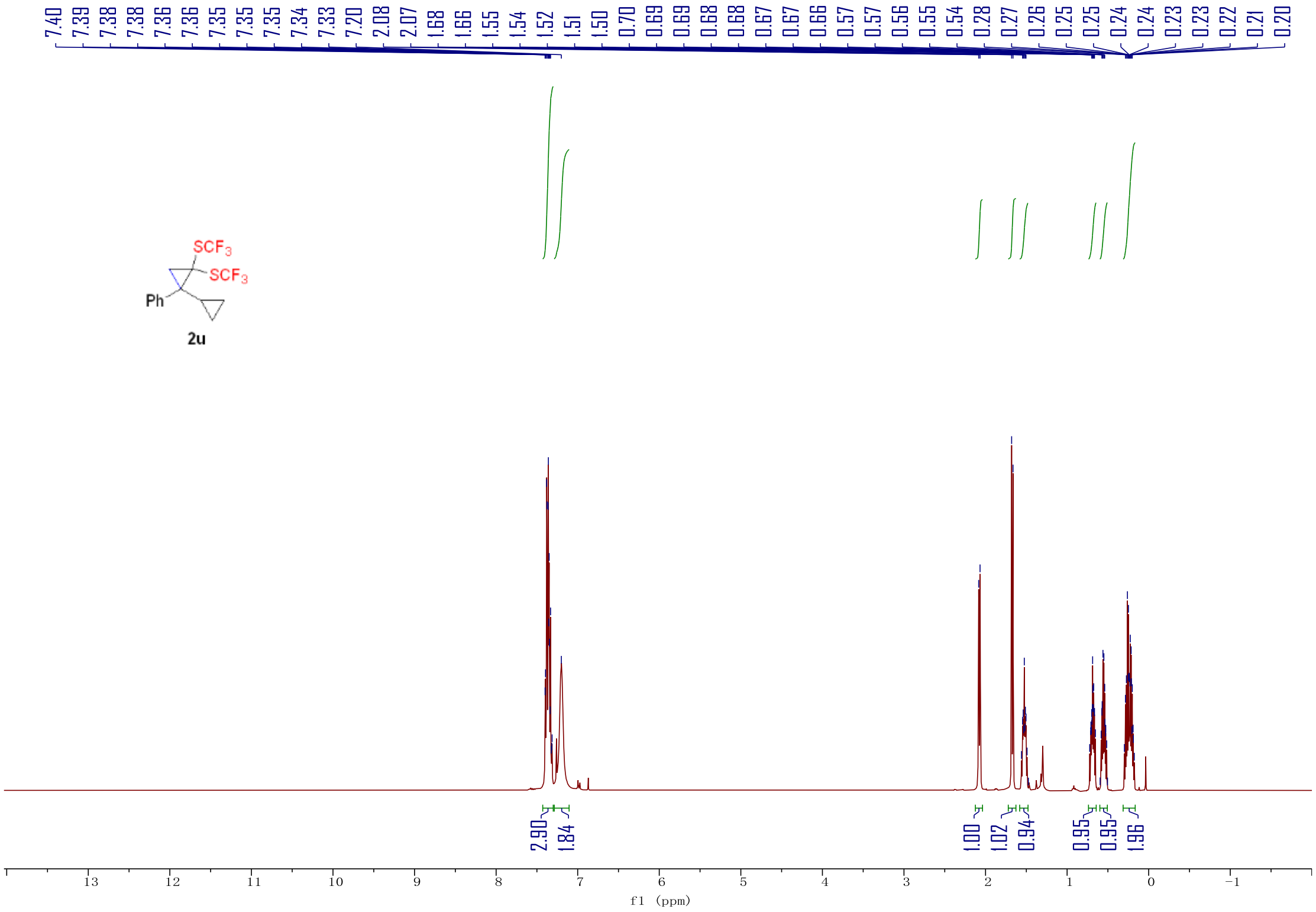

^{19}F NMR Spectrum of **2u** (376 MHz, $CDCl_3$)

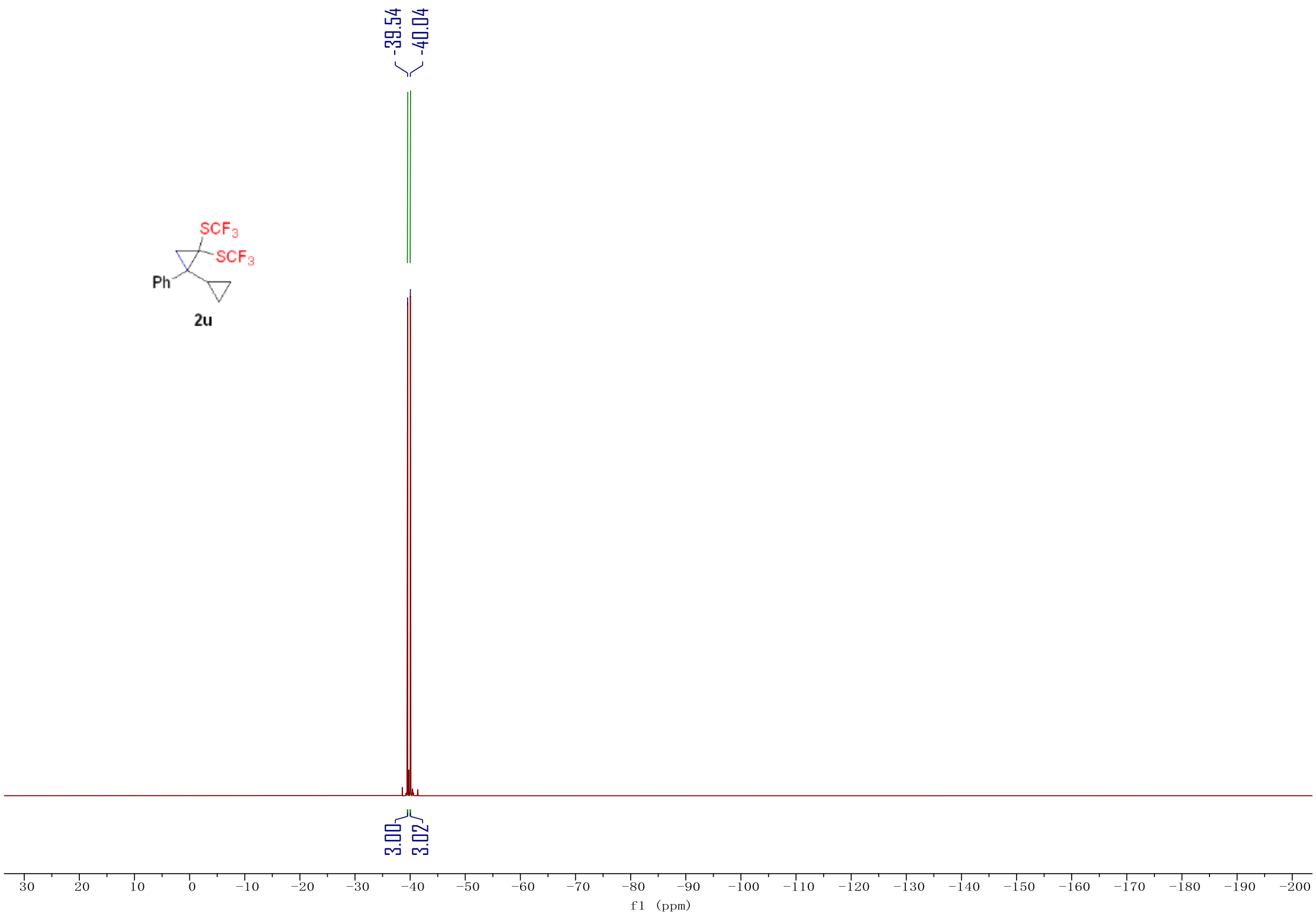

^{13}**C NMR** Spectrum of **2u** (101 MHz, $CDCl_3$)

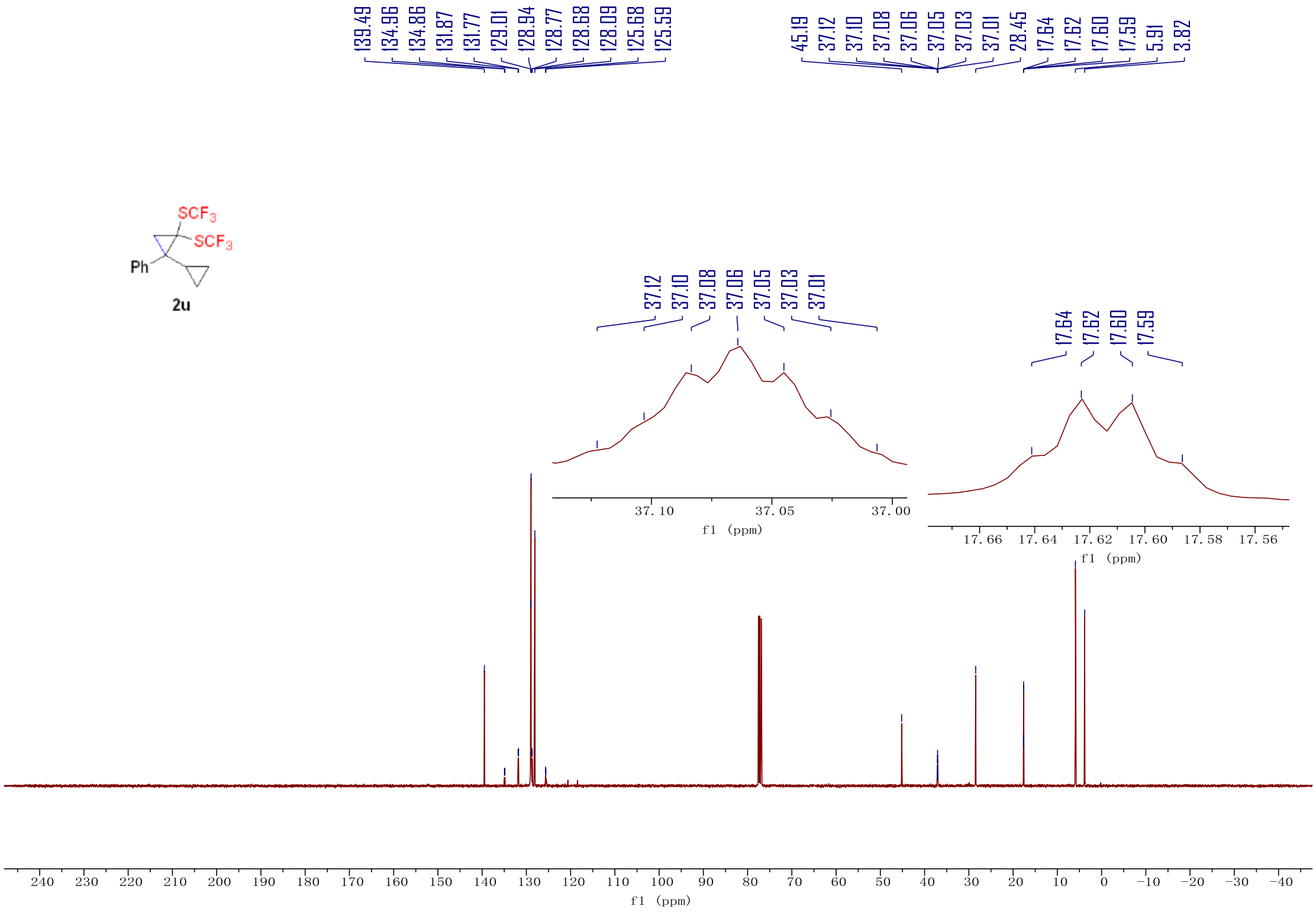

^{1}**H NMR** Spectrum of **2v** (400 MHz, $CDCl_3$)

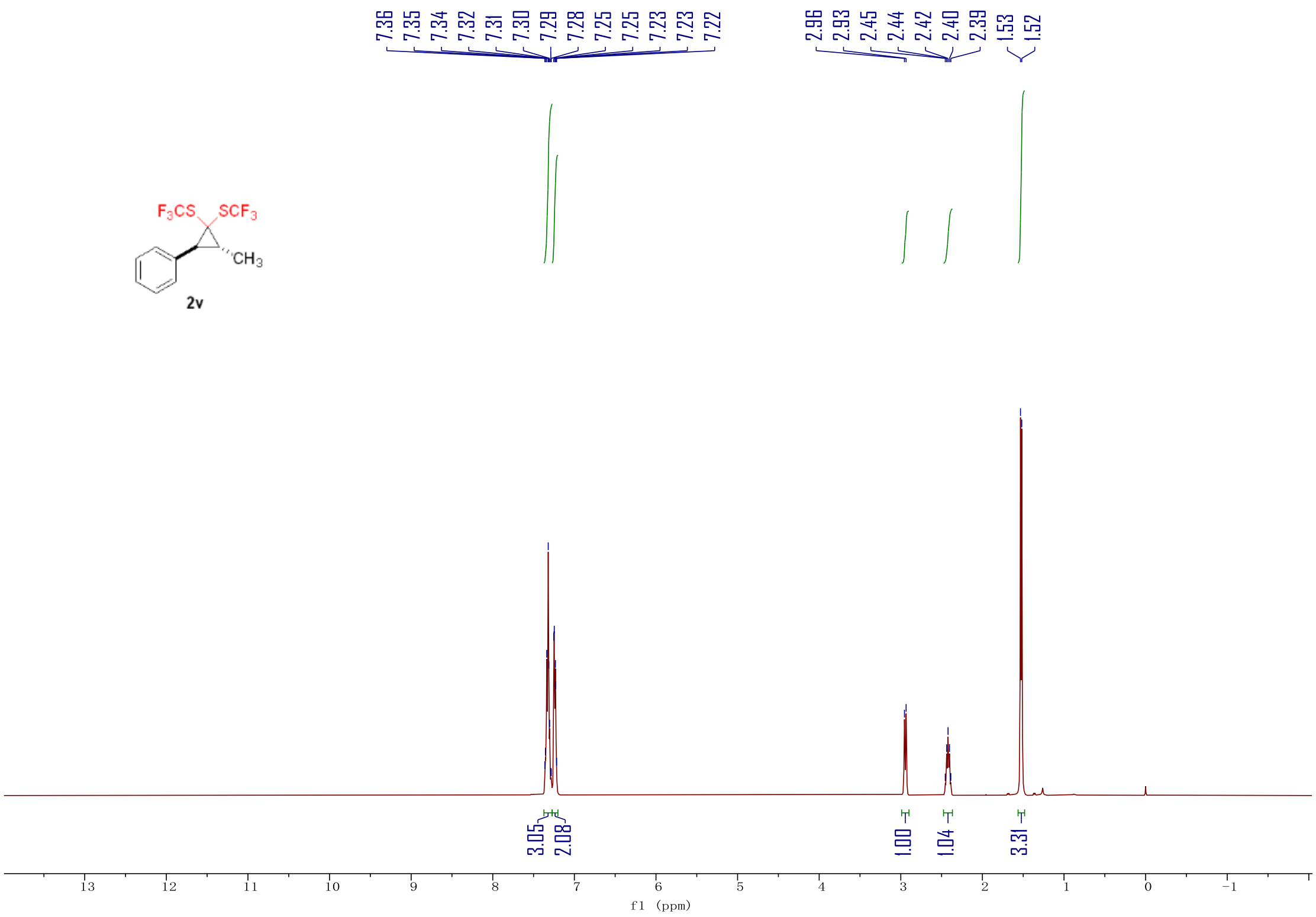

^{19}**F NMR** Spectrum of **2v** (376 MHz, $CDCl_3$)

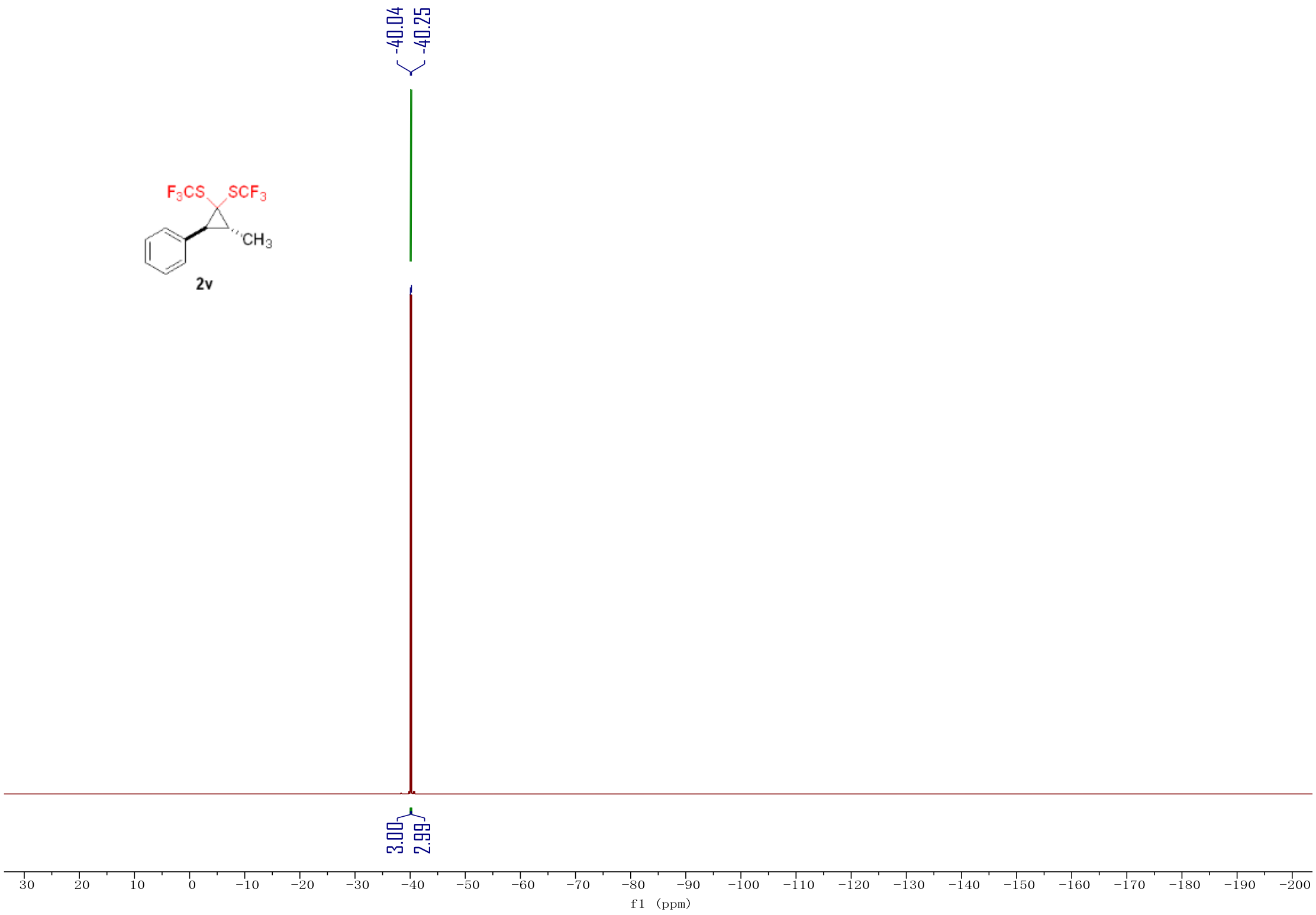

^{13}**C NMR** Spectrum of **2v** (101 MHz, $CDCl_3$)

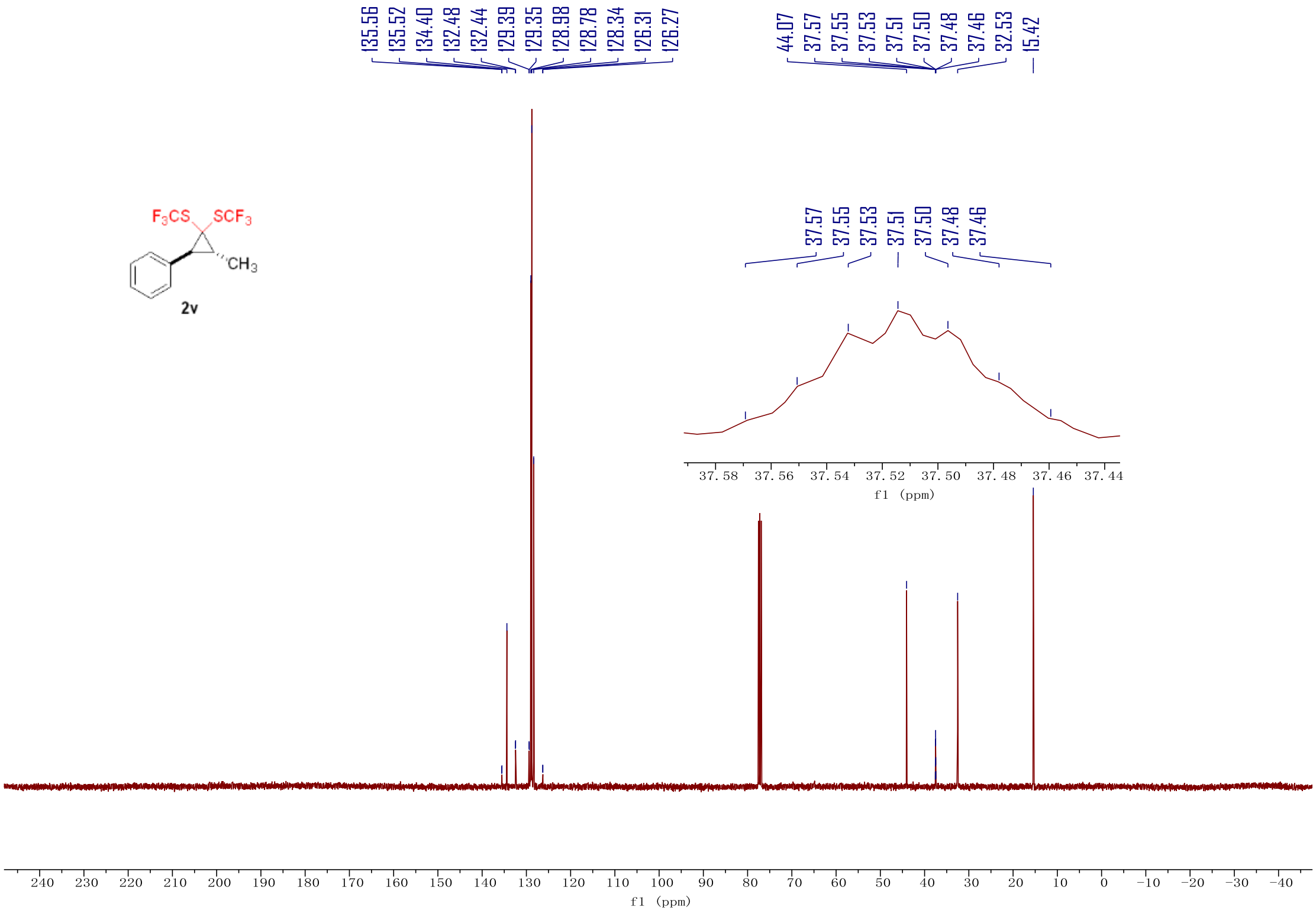

^{1}H NMR Spectrum of **2v'** (400 MHz, $CDCl_3$)

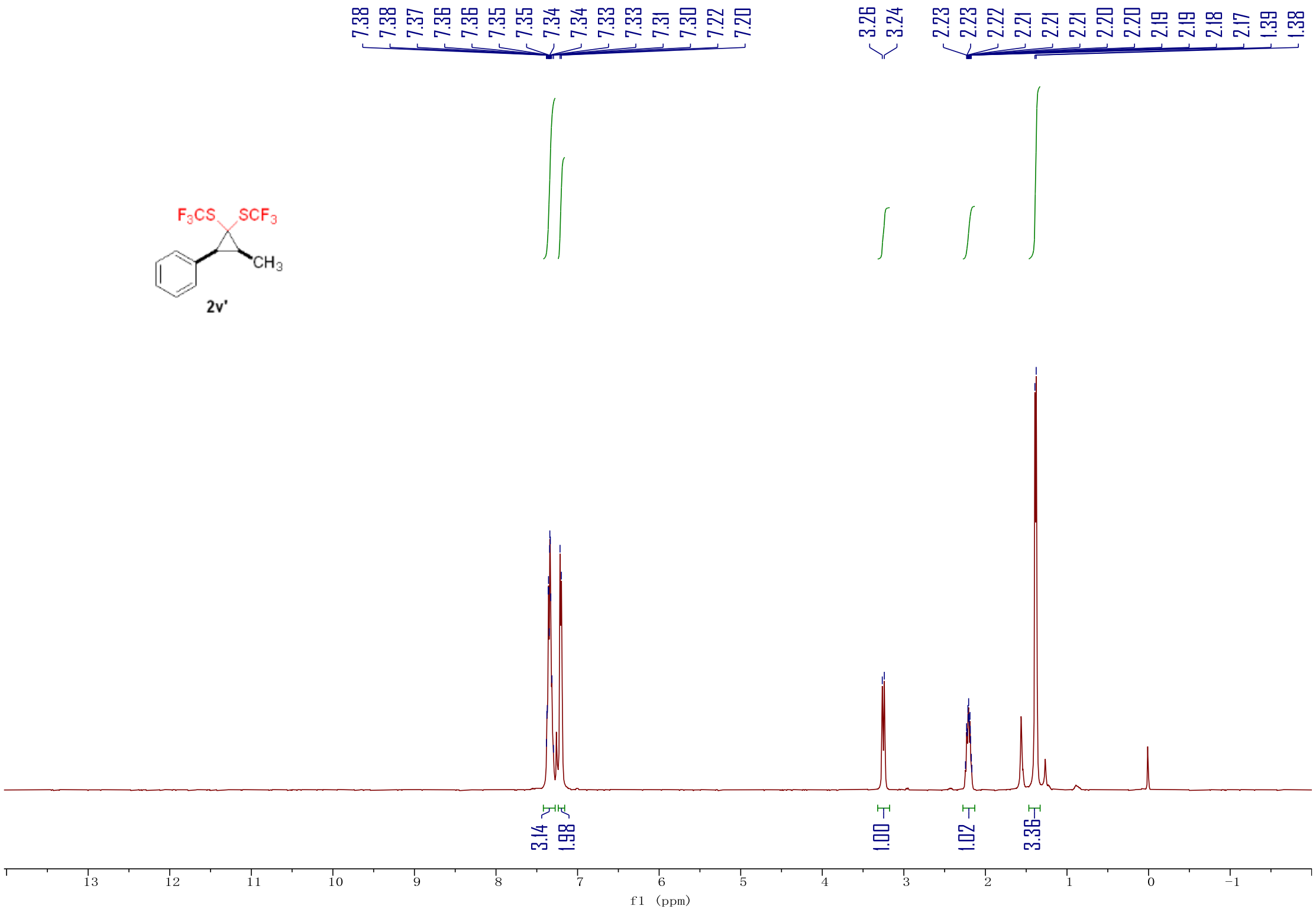

^{19}F NMR Spectrum of **2v'** (376 MHz, $CDCl_3$)

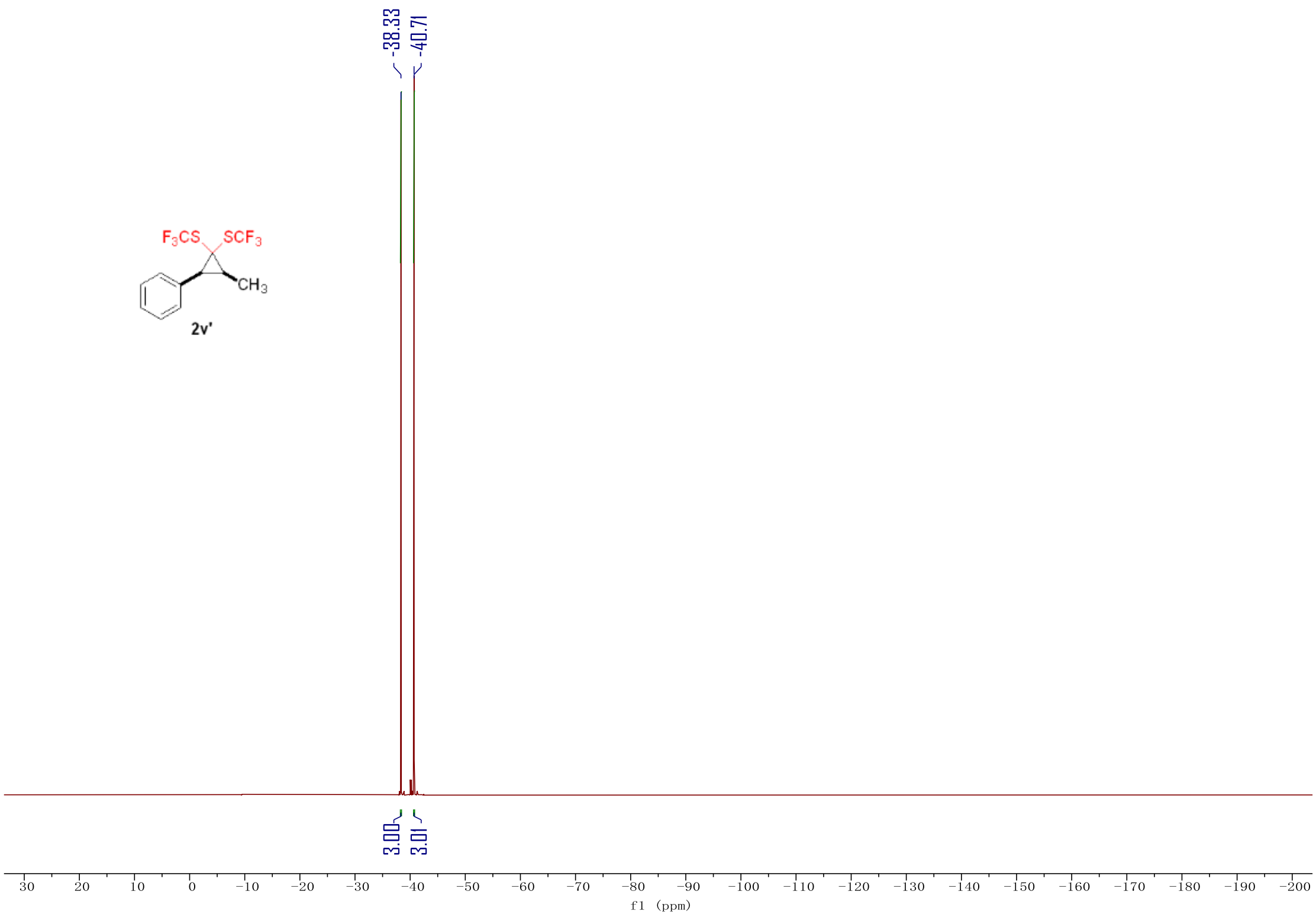

^{13}C NMR Spectrum of **2v'** (101 MHz, $CDCl_3$)

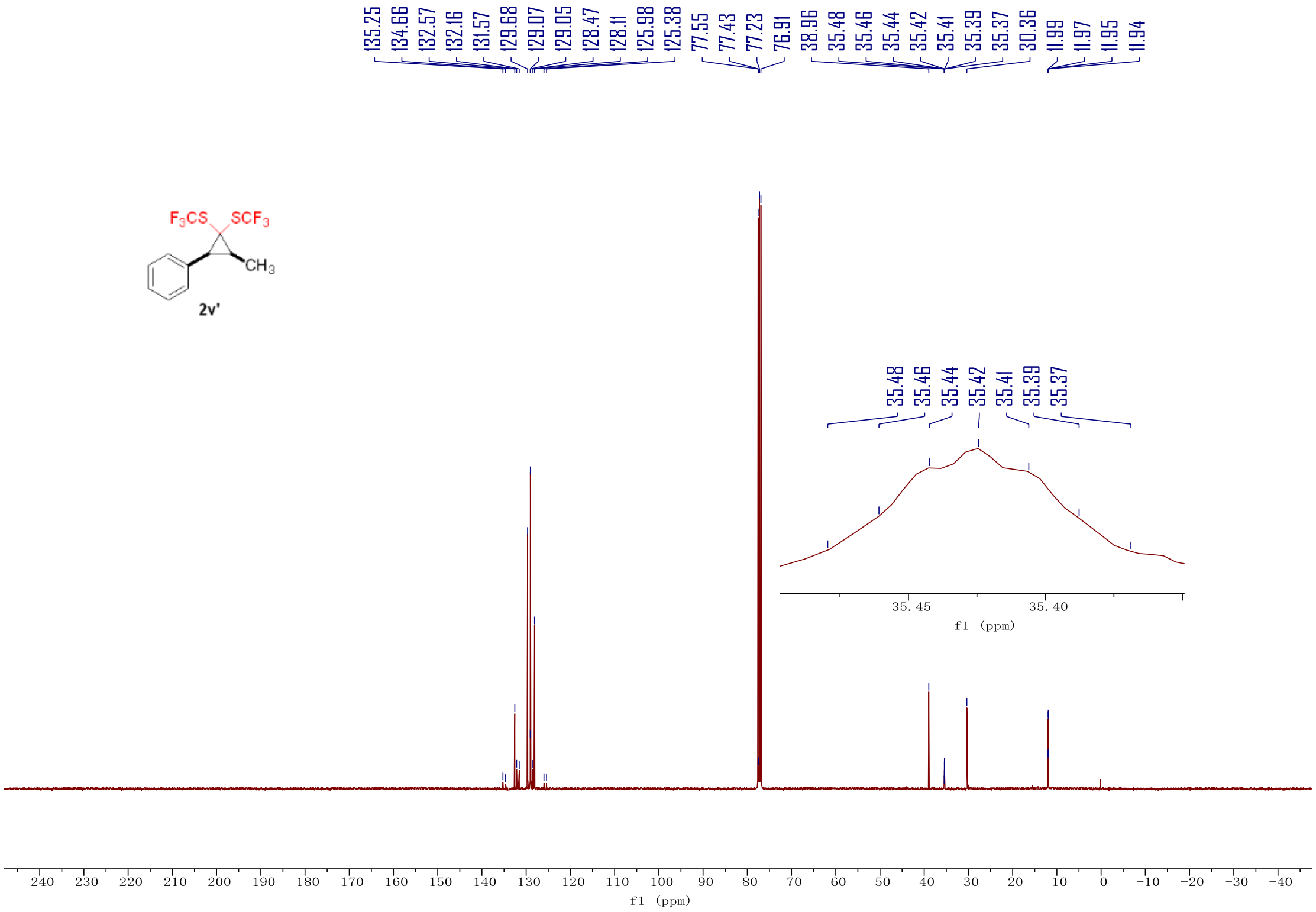

^{1}H NMR Spectrum of **12c'** (400 MHz, $CDCl_3$)

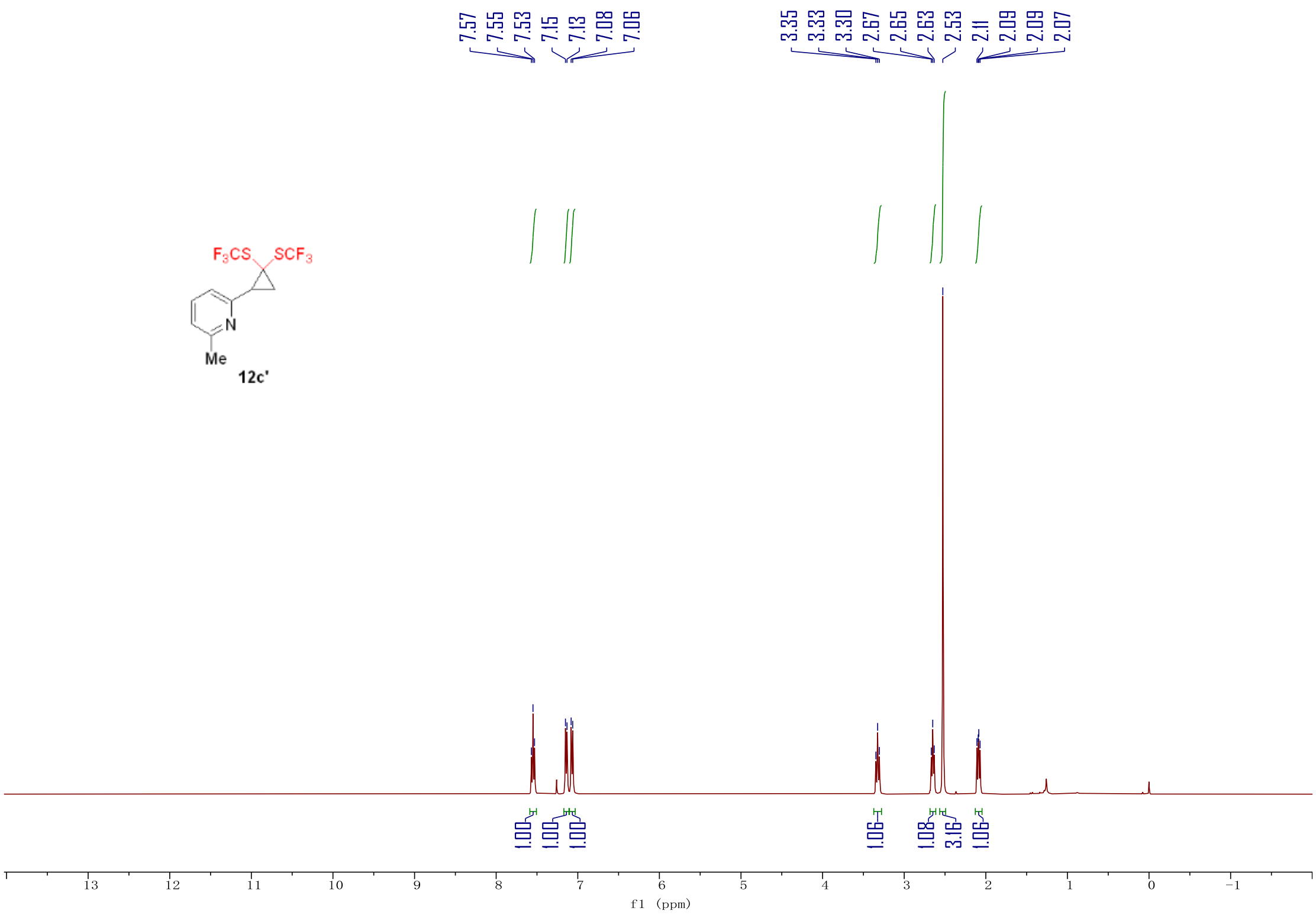

^{19}F NMR Spectrum of **12c'** (376 MHz, $CDCl_3$)

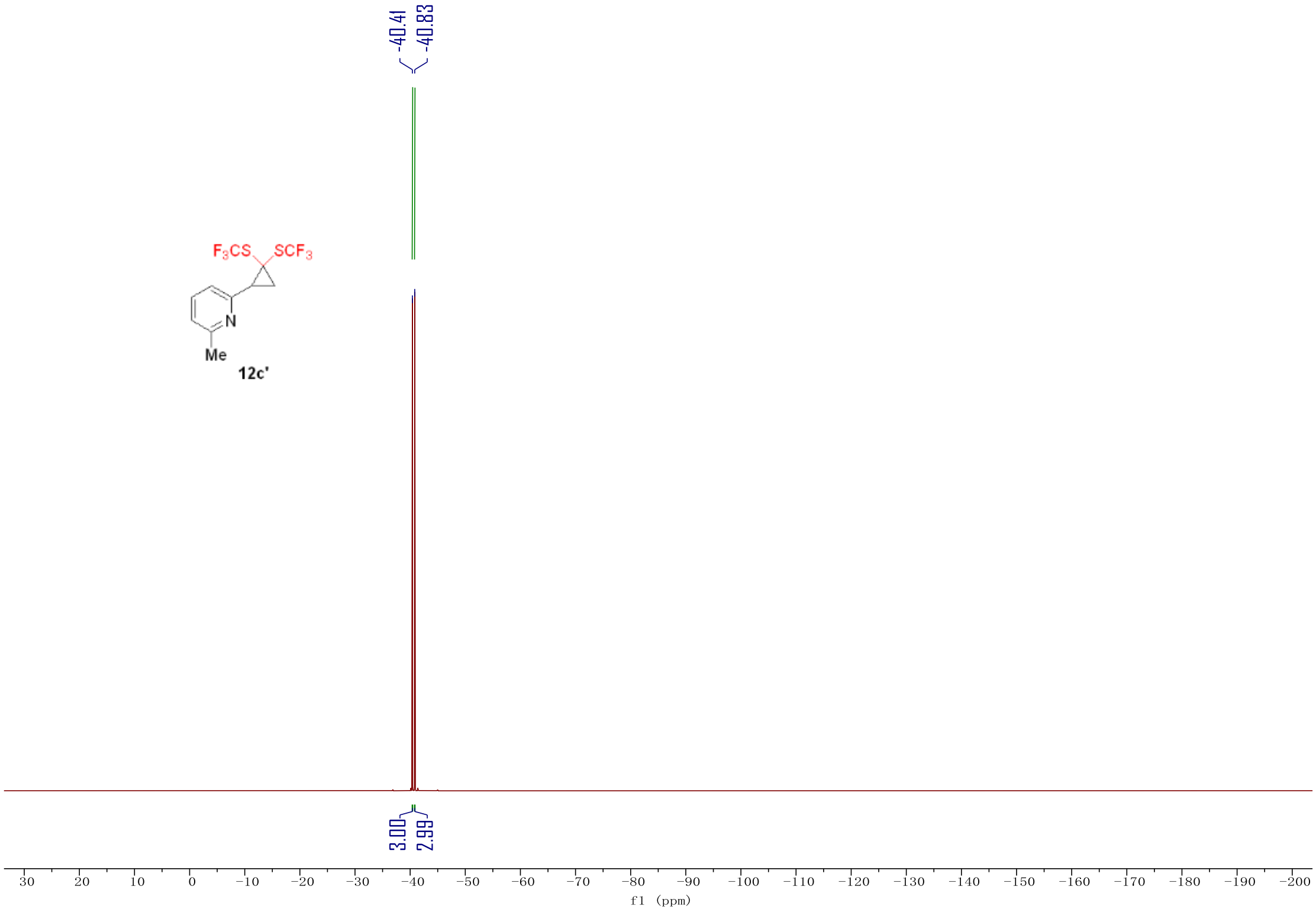

^{13}C NMR Spectrum of **12c'** (101 MHz, $CDCl_3$)

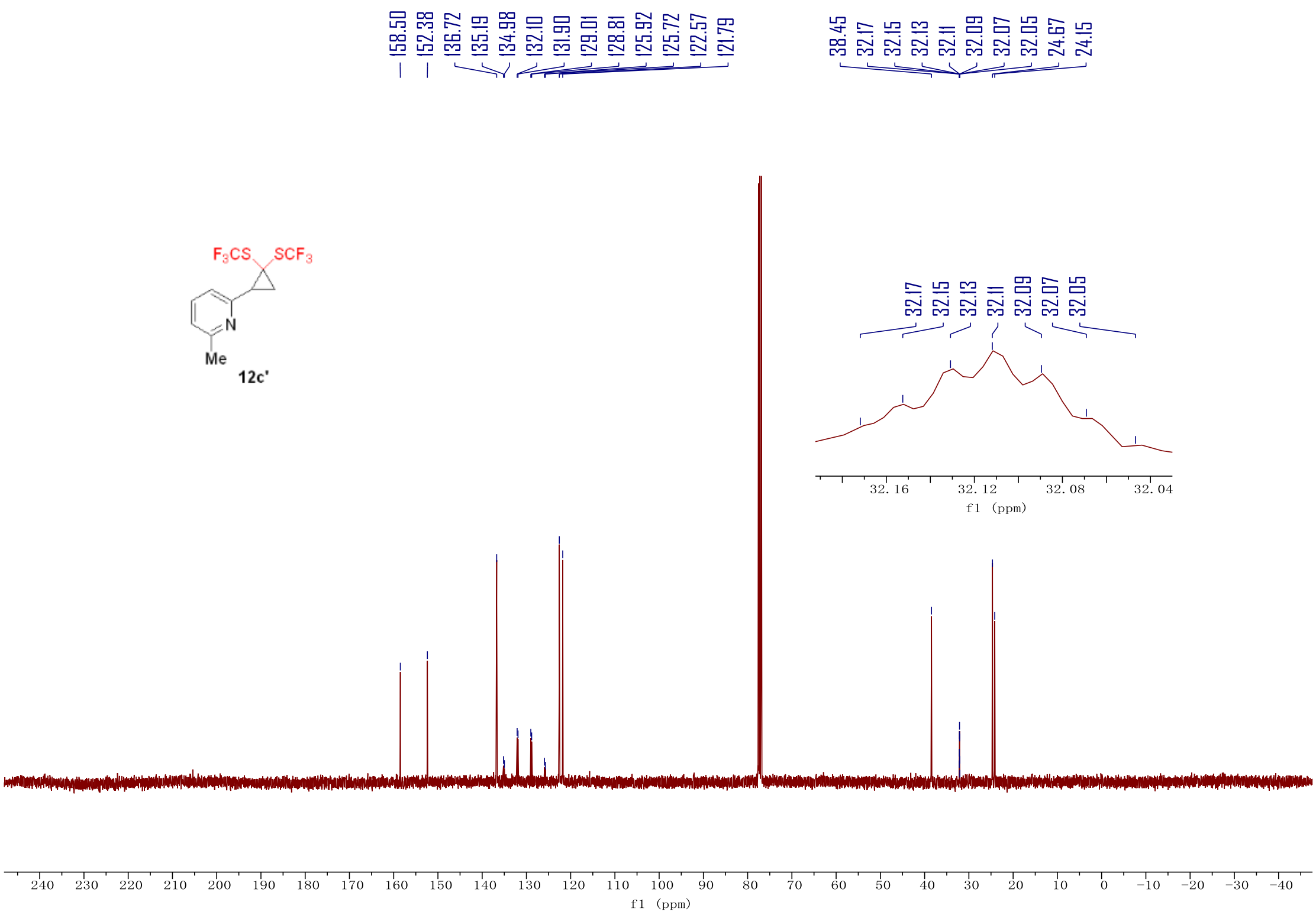

^{1}H NMR Spectrum of **4a** (400 MHz, $CDCl_3$)

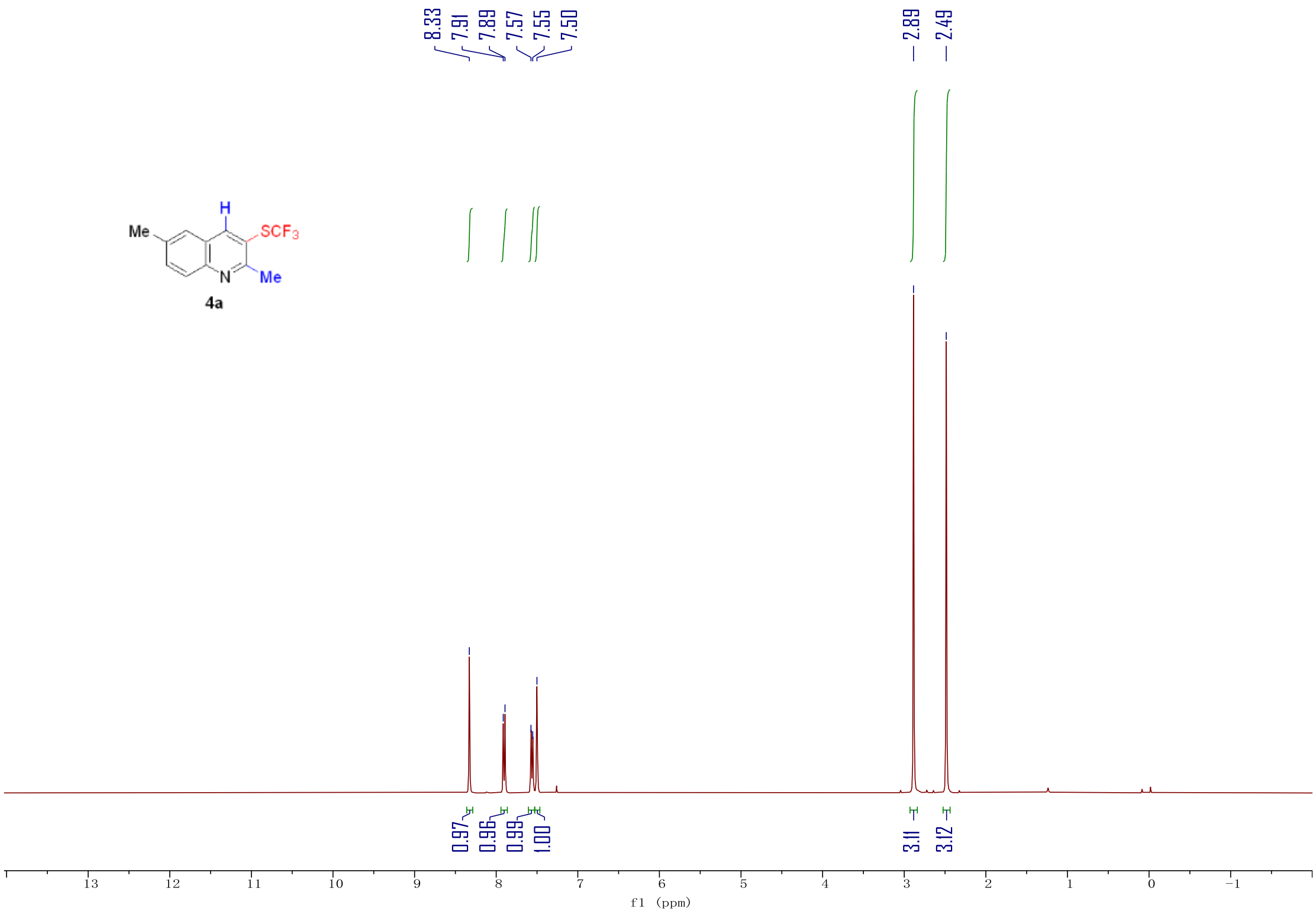

^{19}F NMR Spectrum of **4a** (376 MHz, $CDCl_3$)

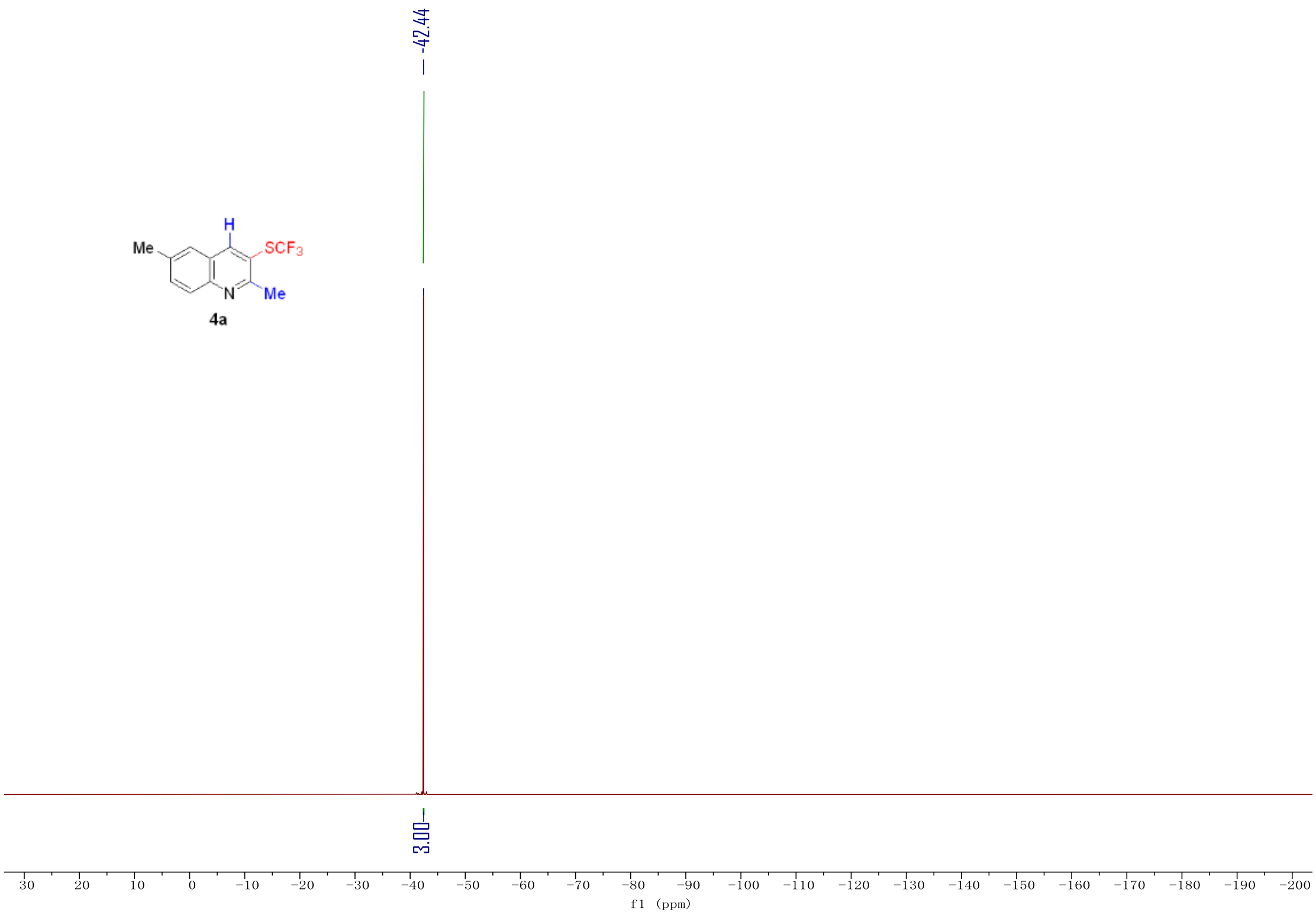

^{13}C NMR Spectrum of **4a** (101 MHz, $CDCl_3$)

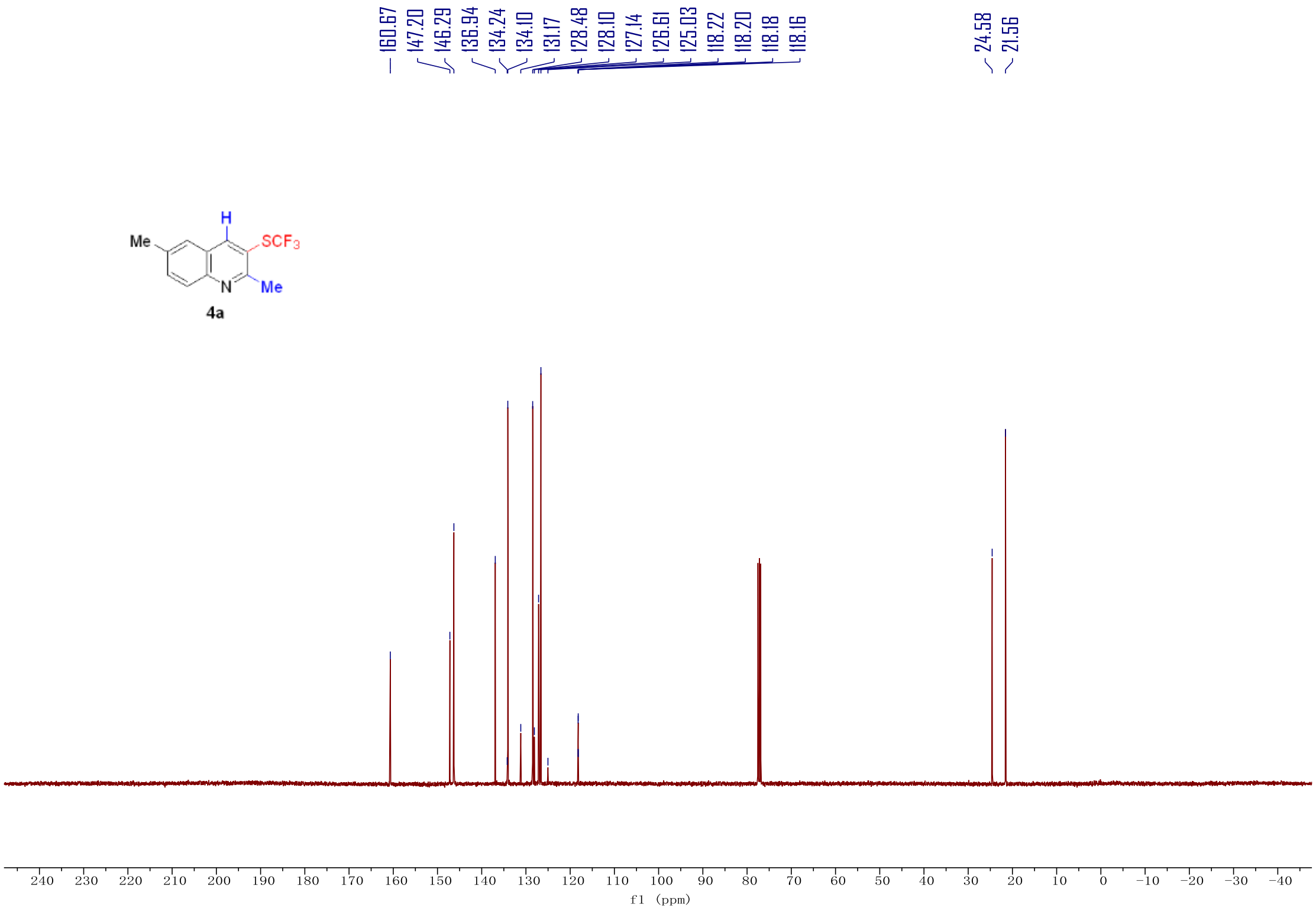

^{1}H NMR Spectrum of **4b** (400 MHz, $CDCl_3$)

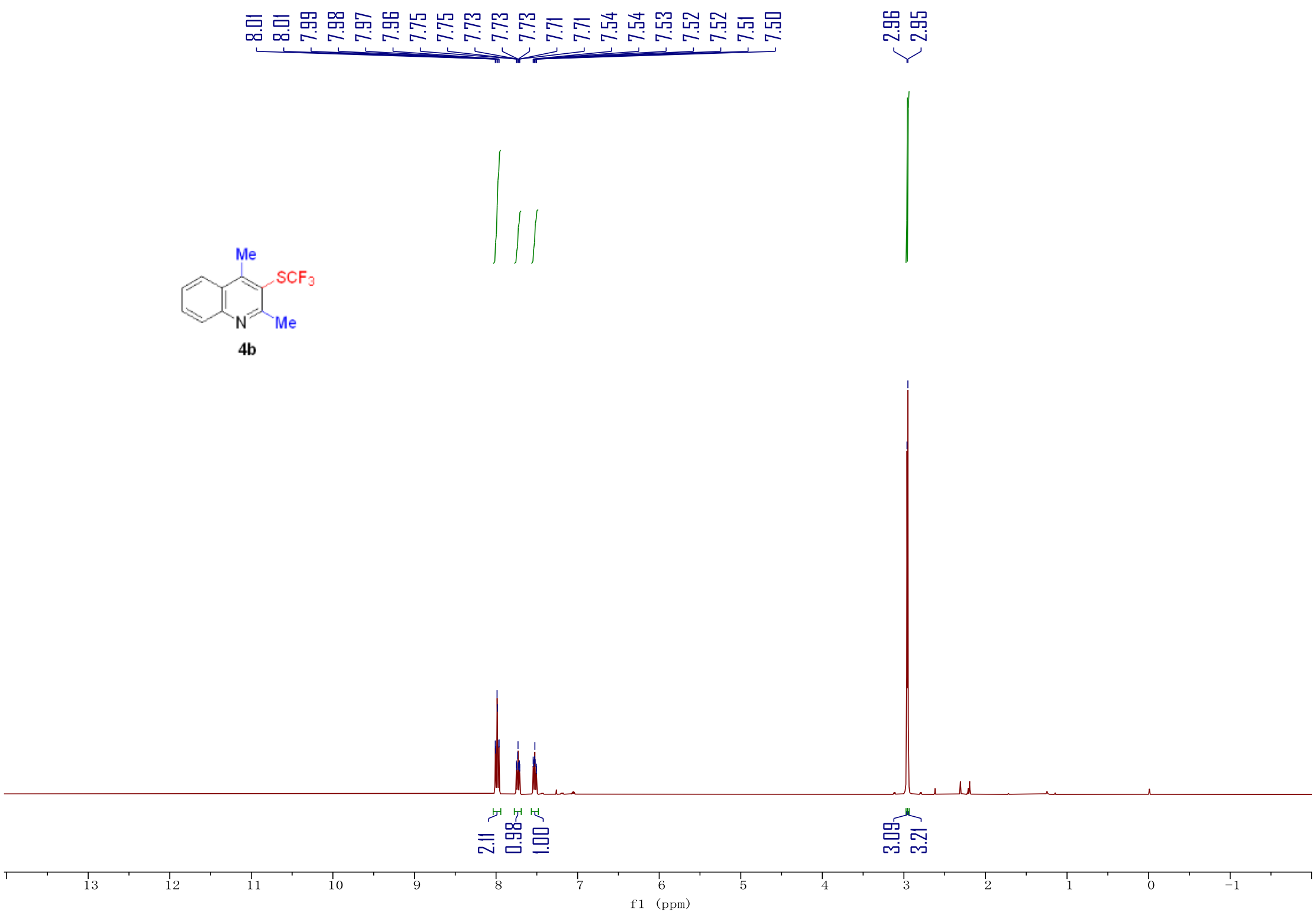

^{19}F NMR Spectrum of **4b** (376 MHz, $CDCl_3$)

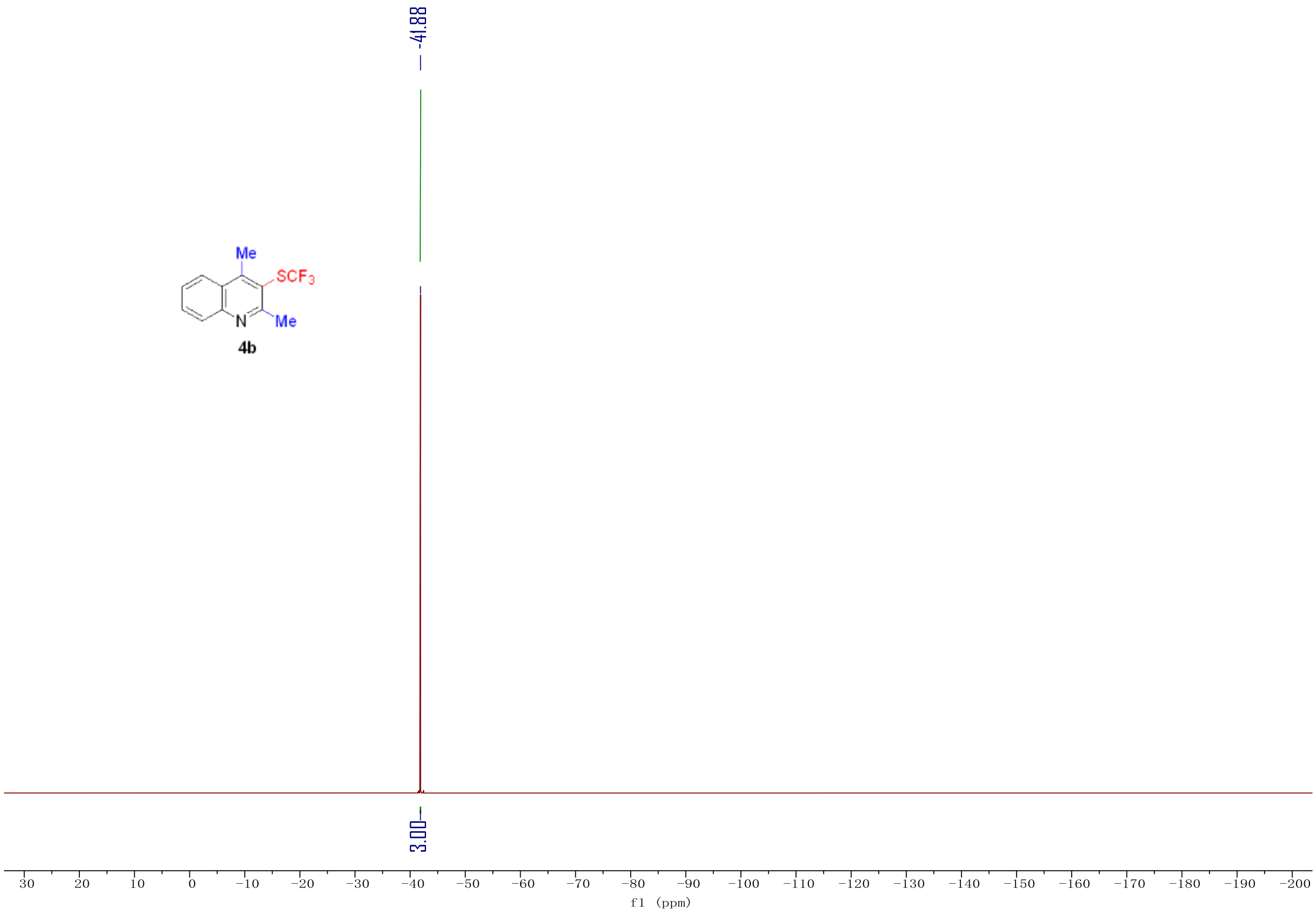

^{13}C NMR Spectrum of **4b** (101 MHz, $CDCl_3$)

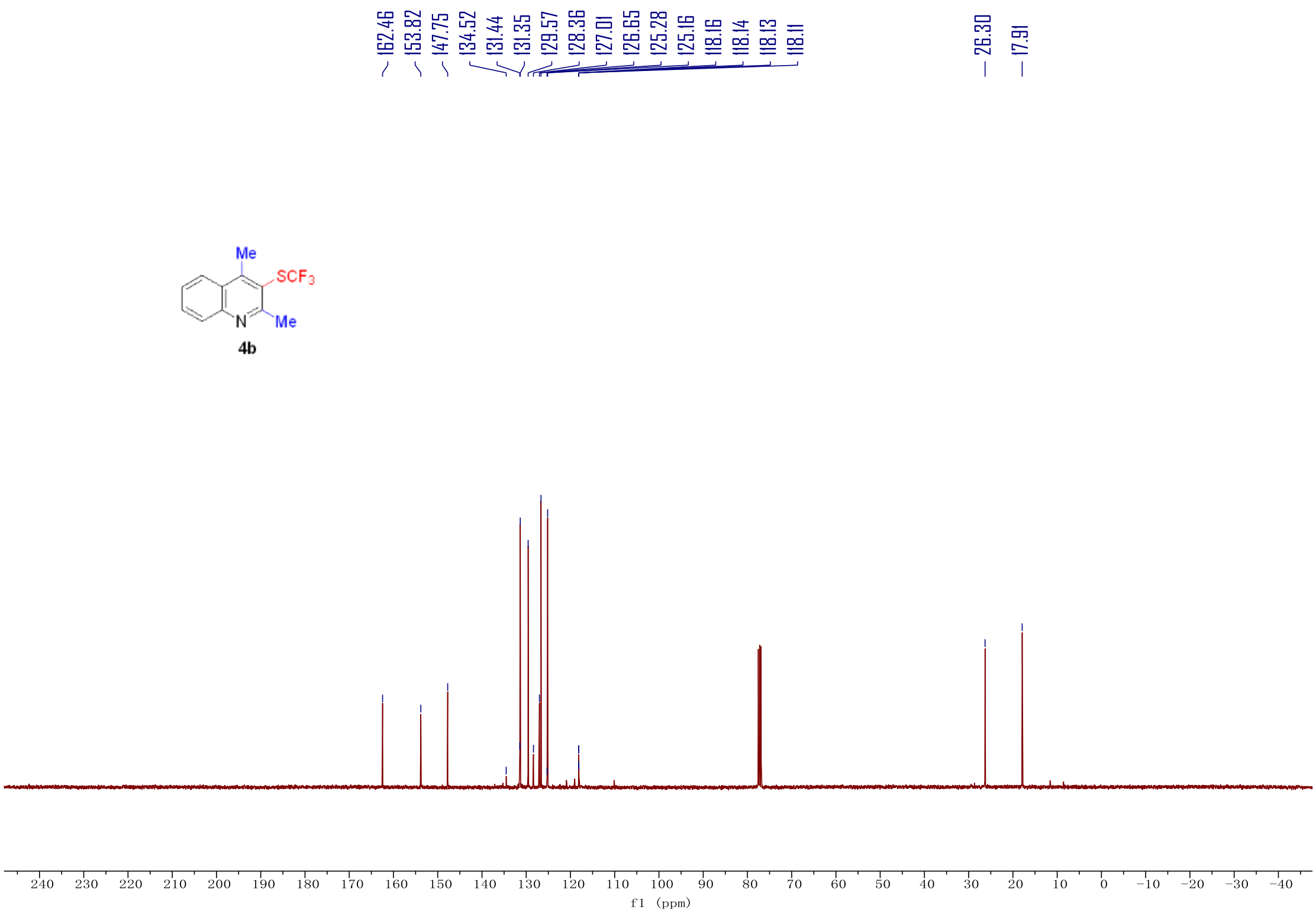

^{1}**H NMR** Spectrum of **4c** (400 MHz, $CDCl_3$)

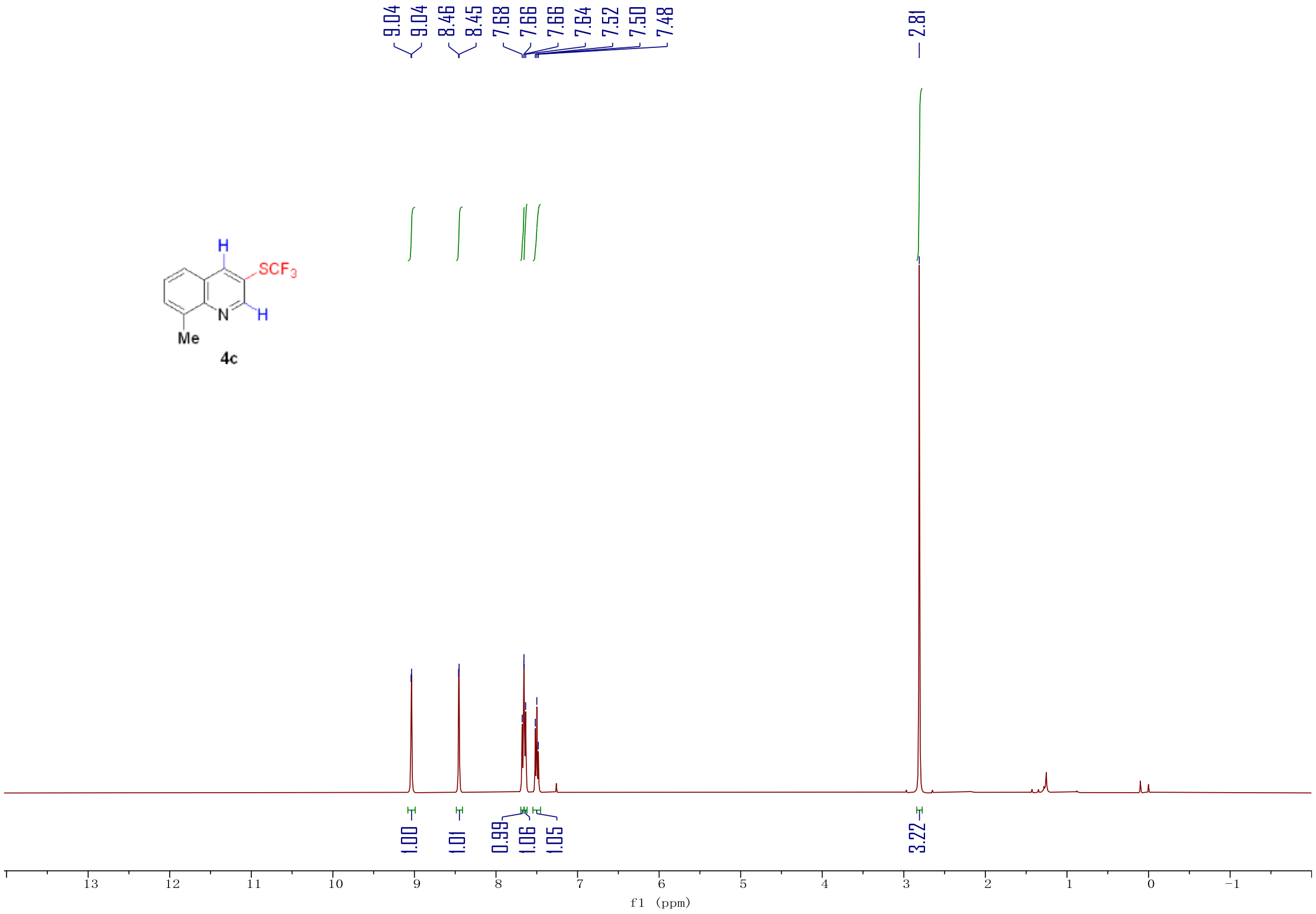

^{19}**F NMR** Spectrum of **4c** (376 MHz, $CDCl_3$)

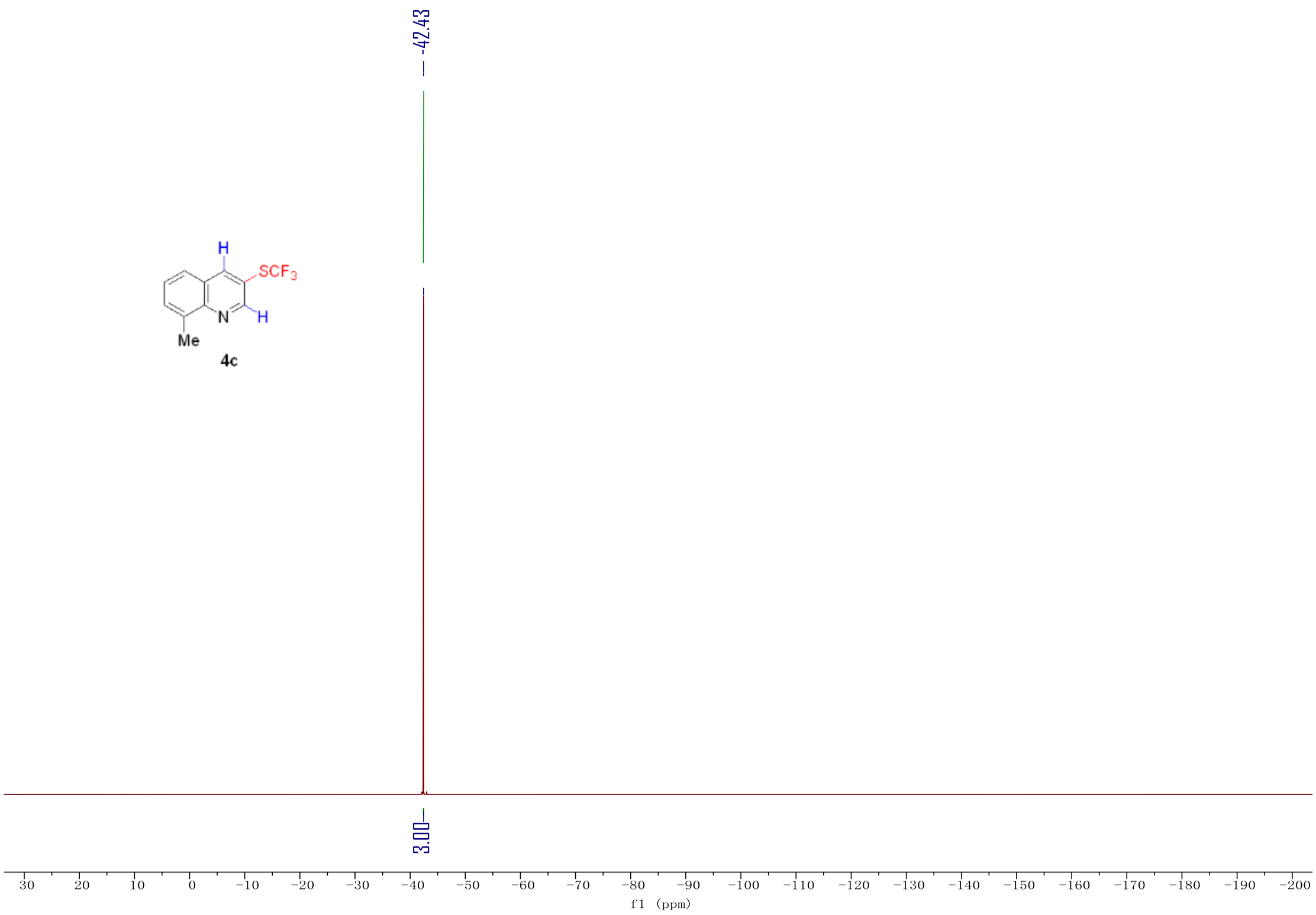

^{13}C NMR Spectrum of **4c** (101 MHz, $CDCl_3$)

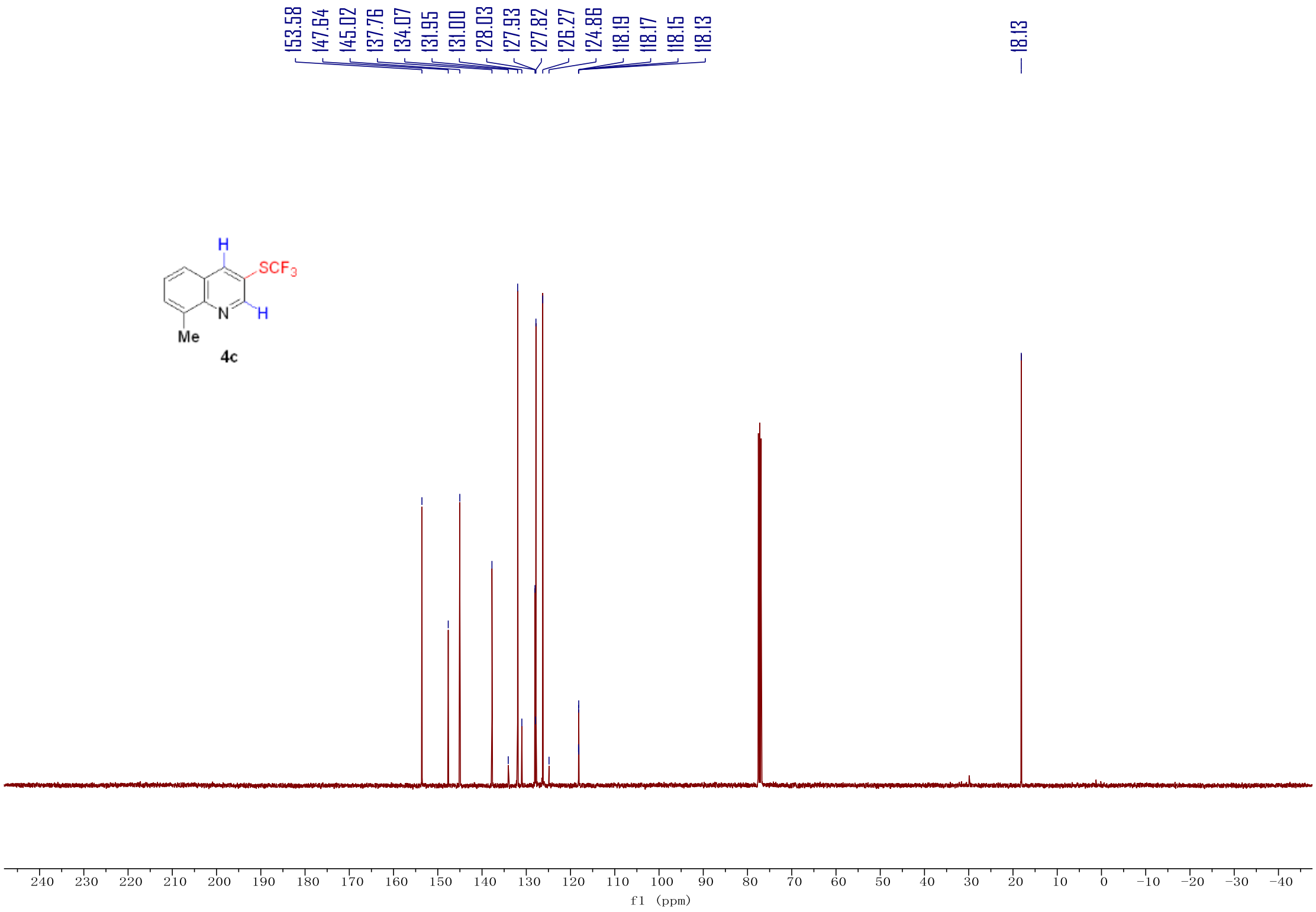

^{1}H NMR Spectrum of **4d** (400 MHz, $CDCl_3$)

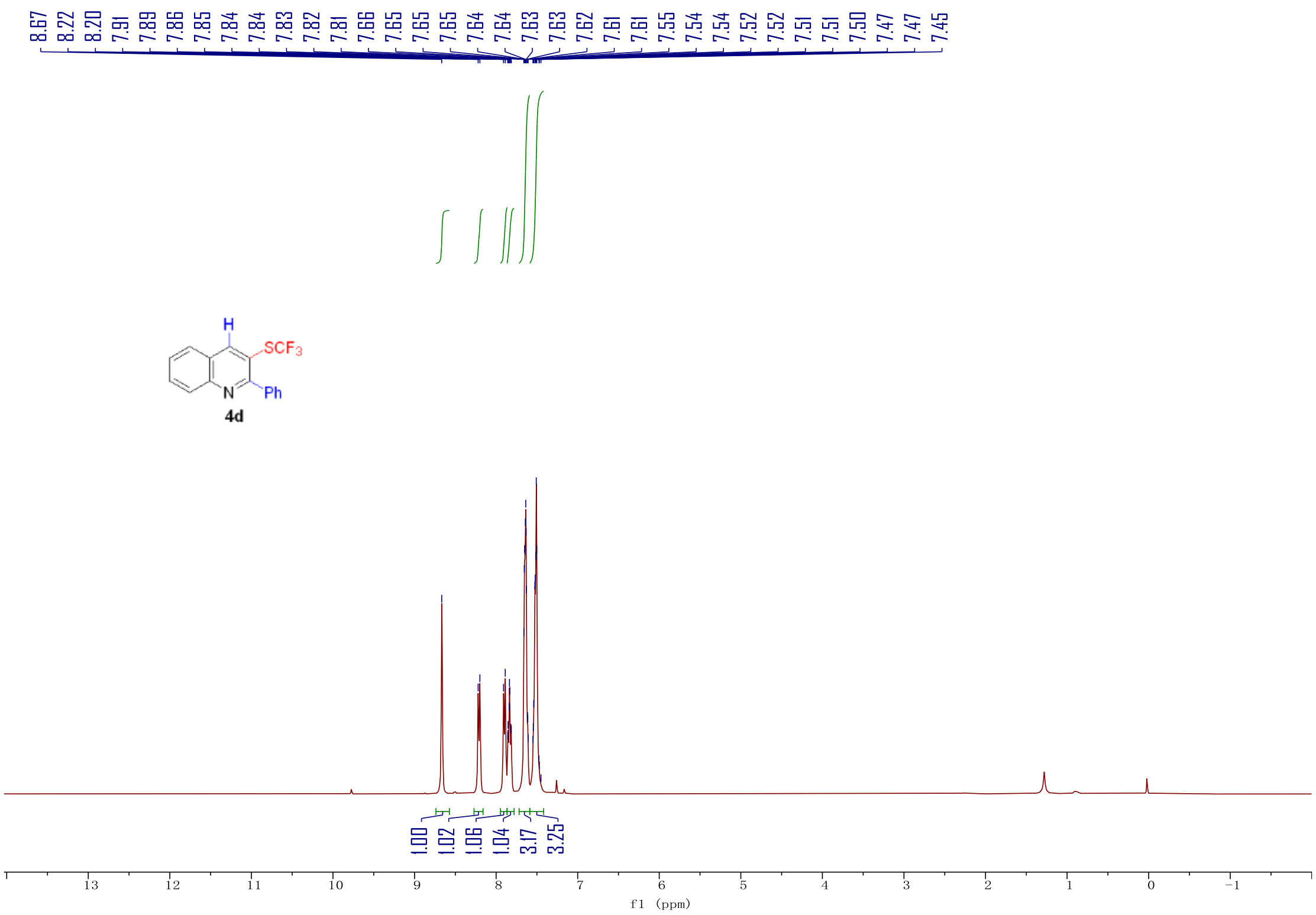

^{19}**F NMR** Spectrum of **4d** (376 MHz, $CDCl_3$)

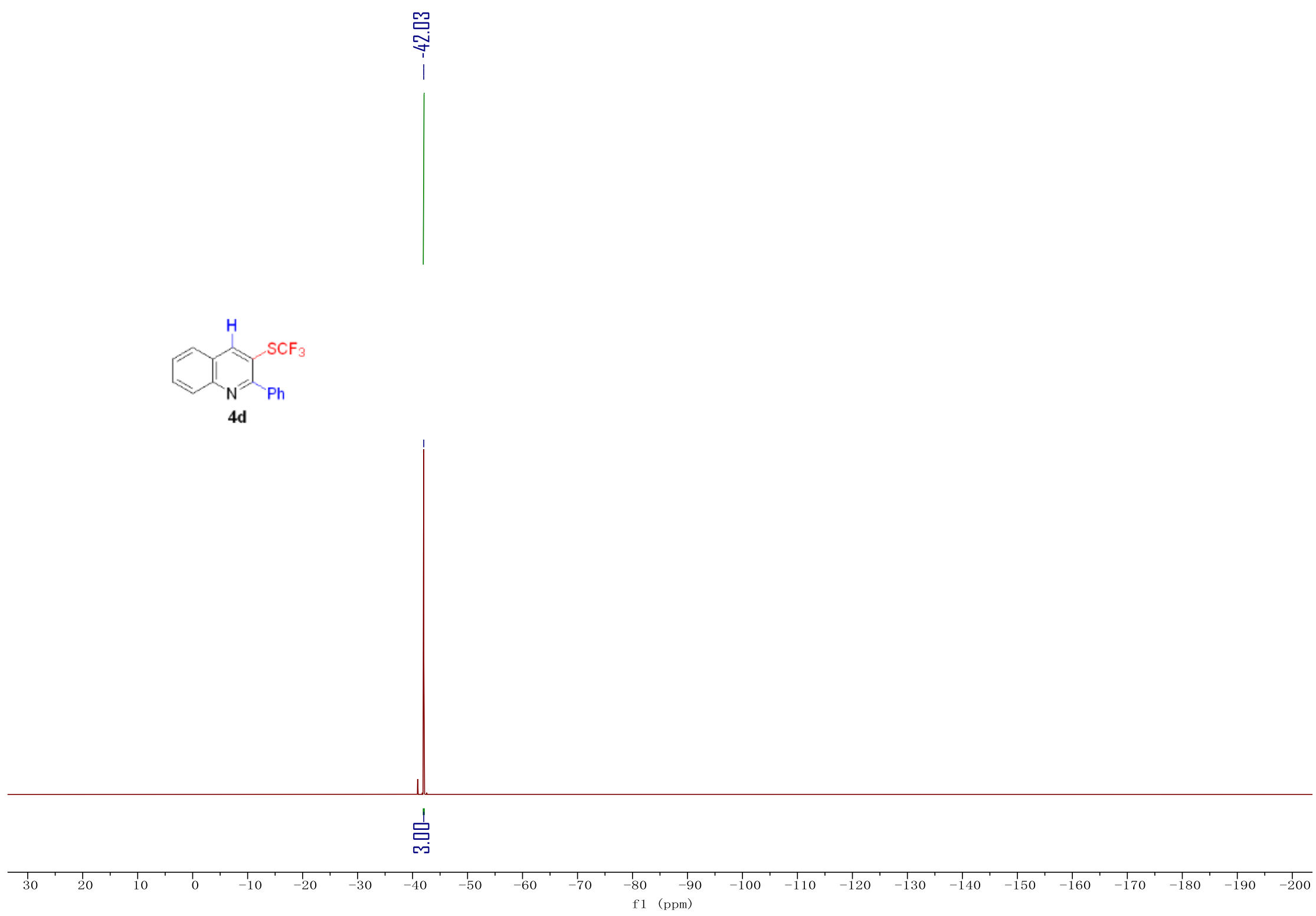

^{13}**C NMR** Spectrum of **4d** (101 MHz, $CDCl_3$)

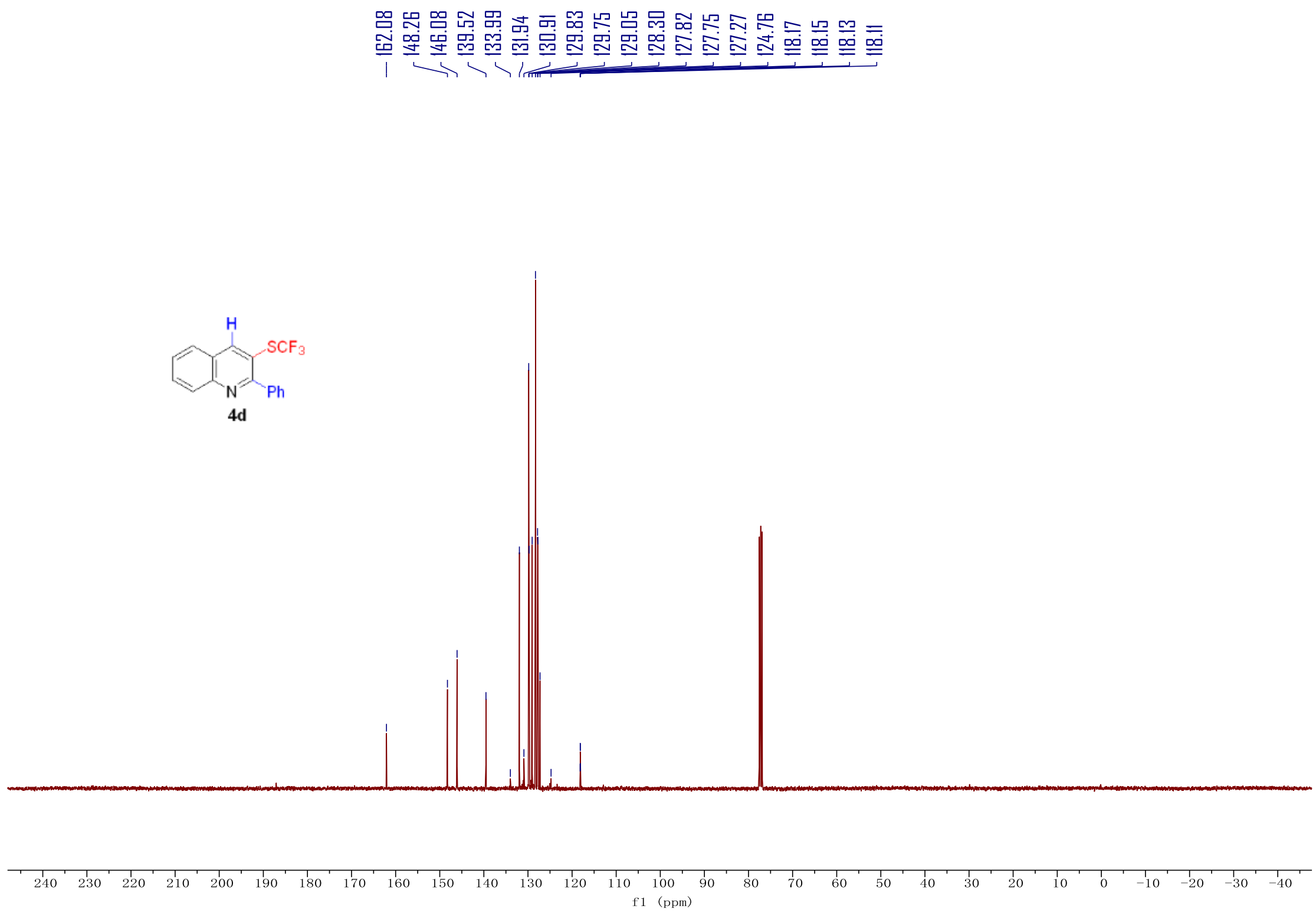

^{1}**H NMR** Spectrum of **4e** (400 MHz, $CDCl_3$)

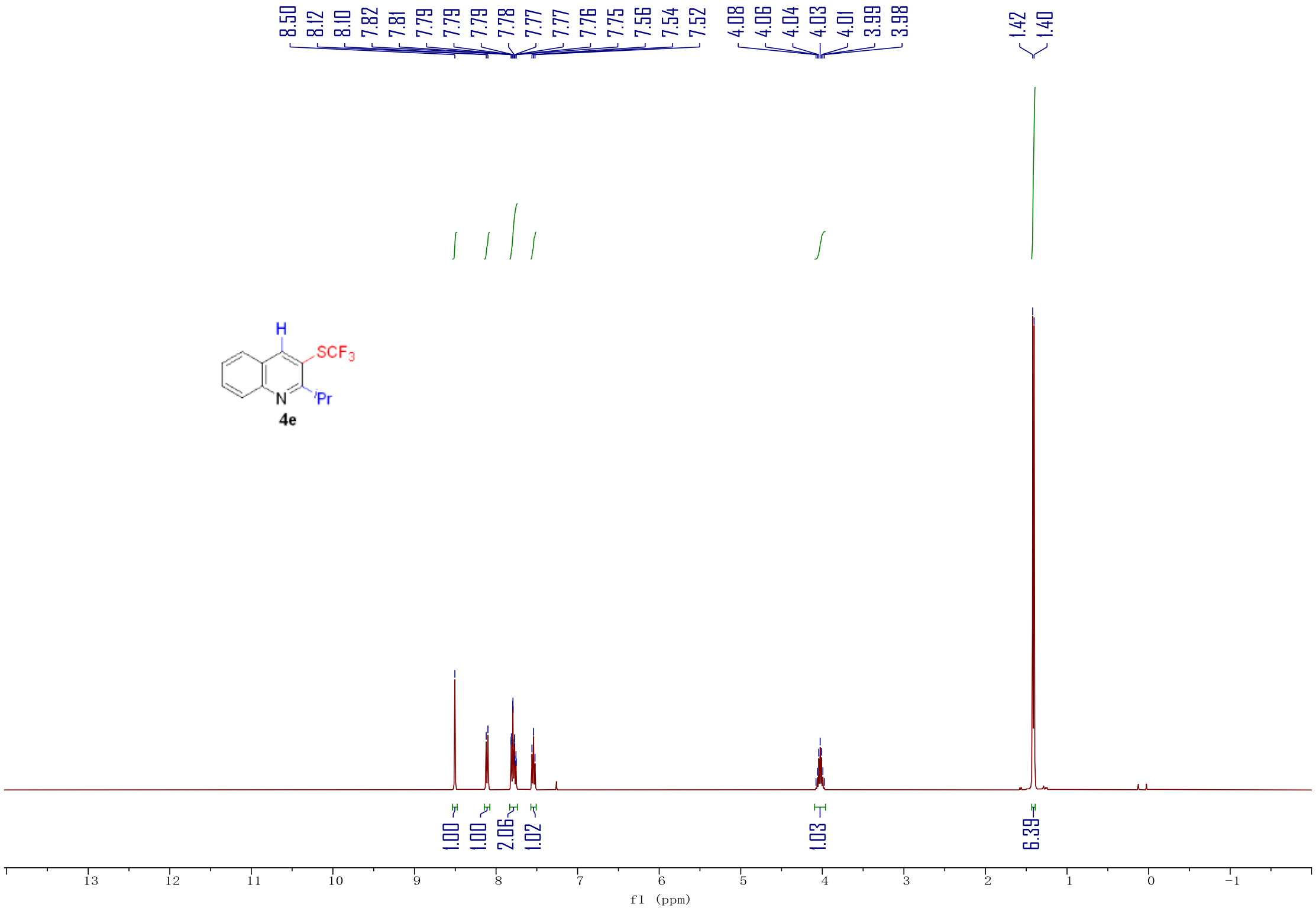

^{19}**F NMR** Spectrum of **4e** (376 MHz, $CDCl_3$)

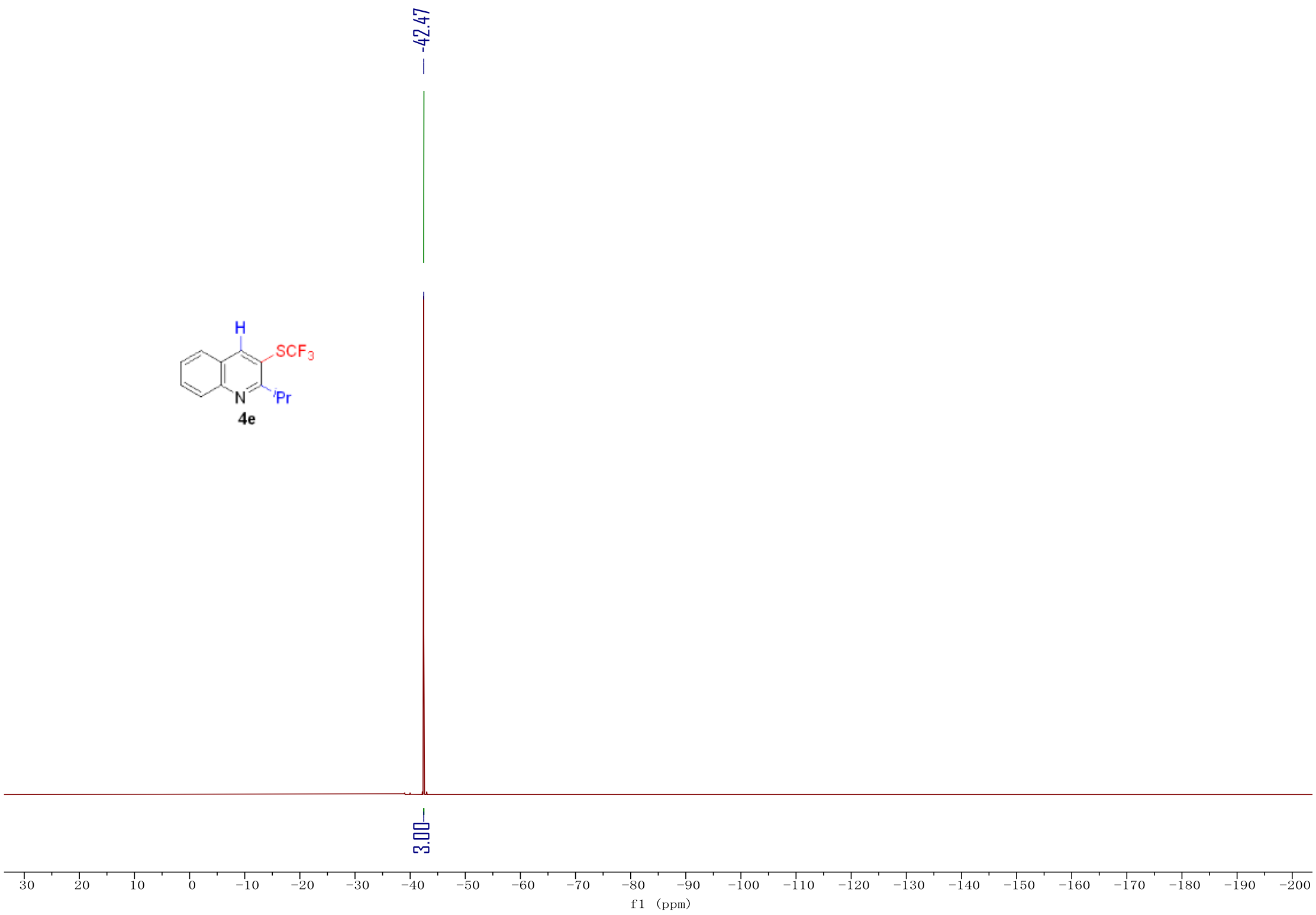

^{13}C NMR Spectrum of **4e** (101 MHz, $CDCl_3$)

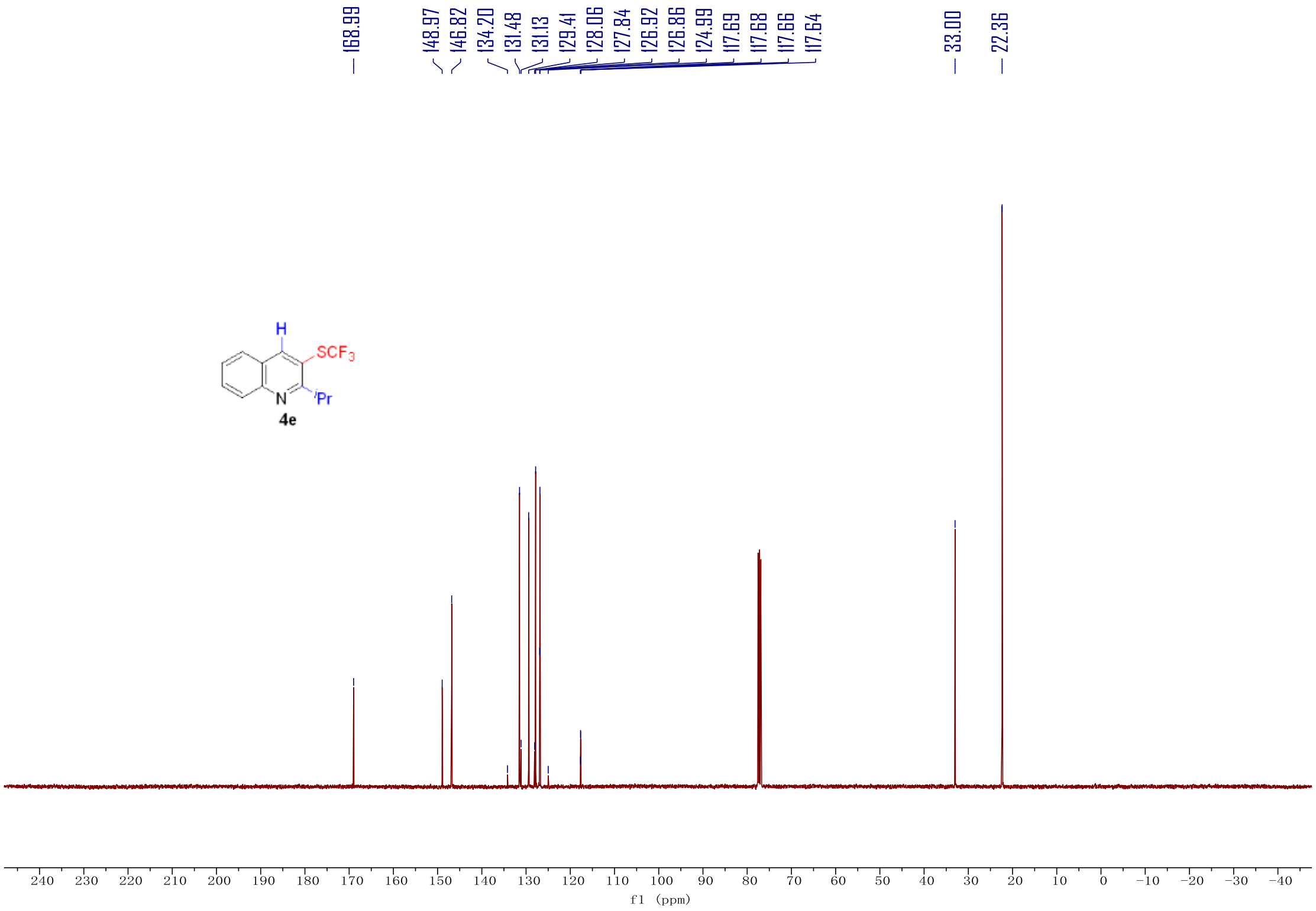

^{1}H NMR Spectrum of **4f** (400 MHz, $CDCl_3$)

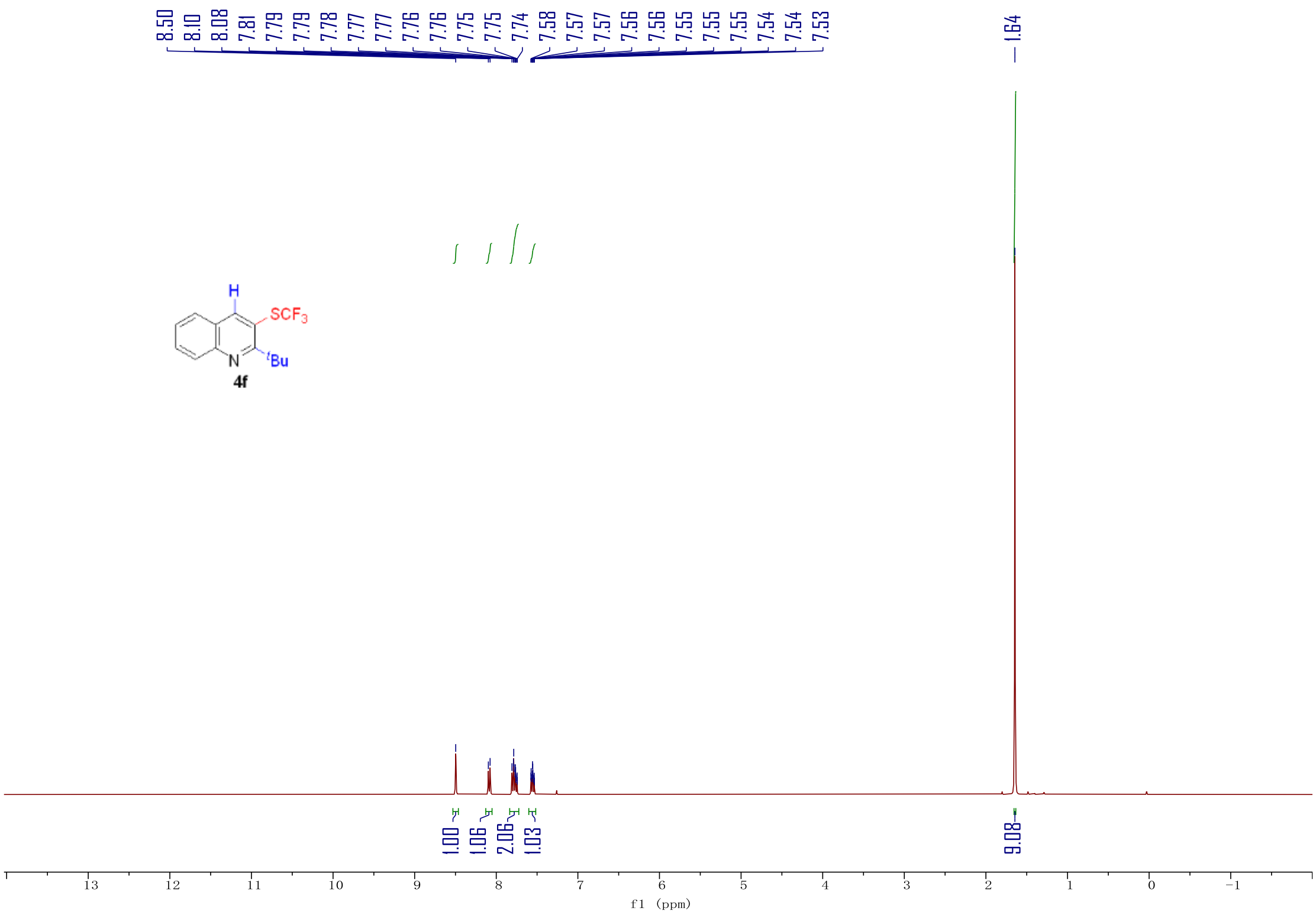

^{19}F NMR Spectrum of **4f** (376 MHz, $CDCl_3$)

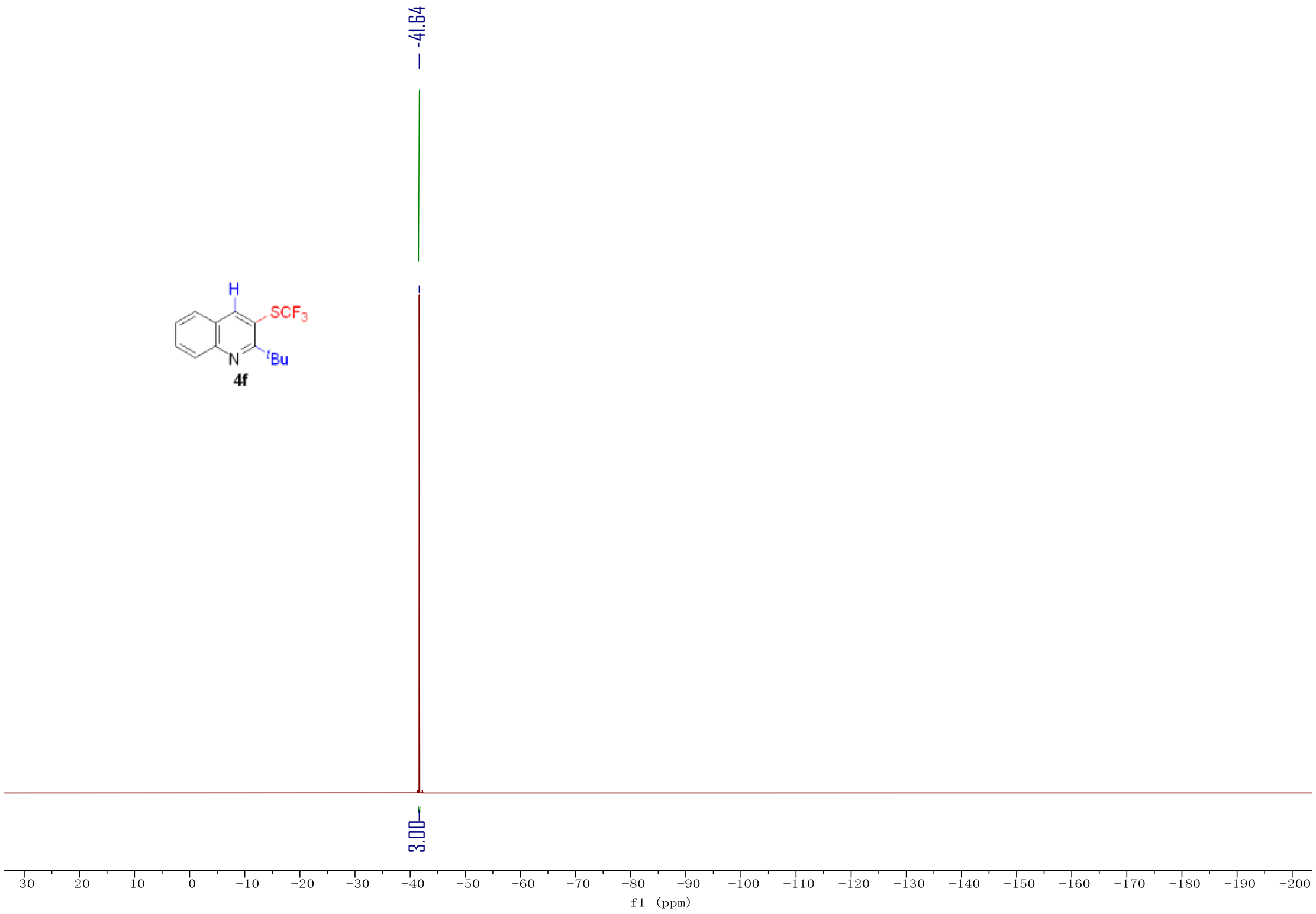

^{13}C NMR Spectrum of **4f** (101 MHz, $CDCl_3$)

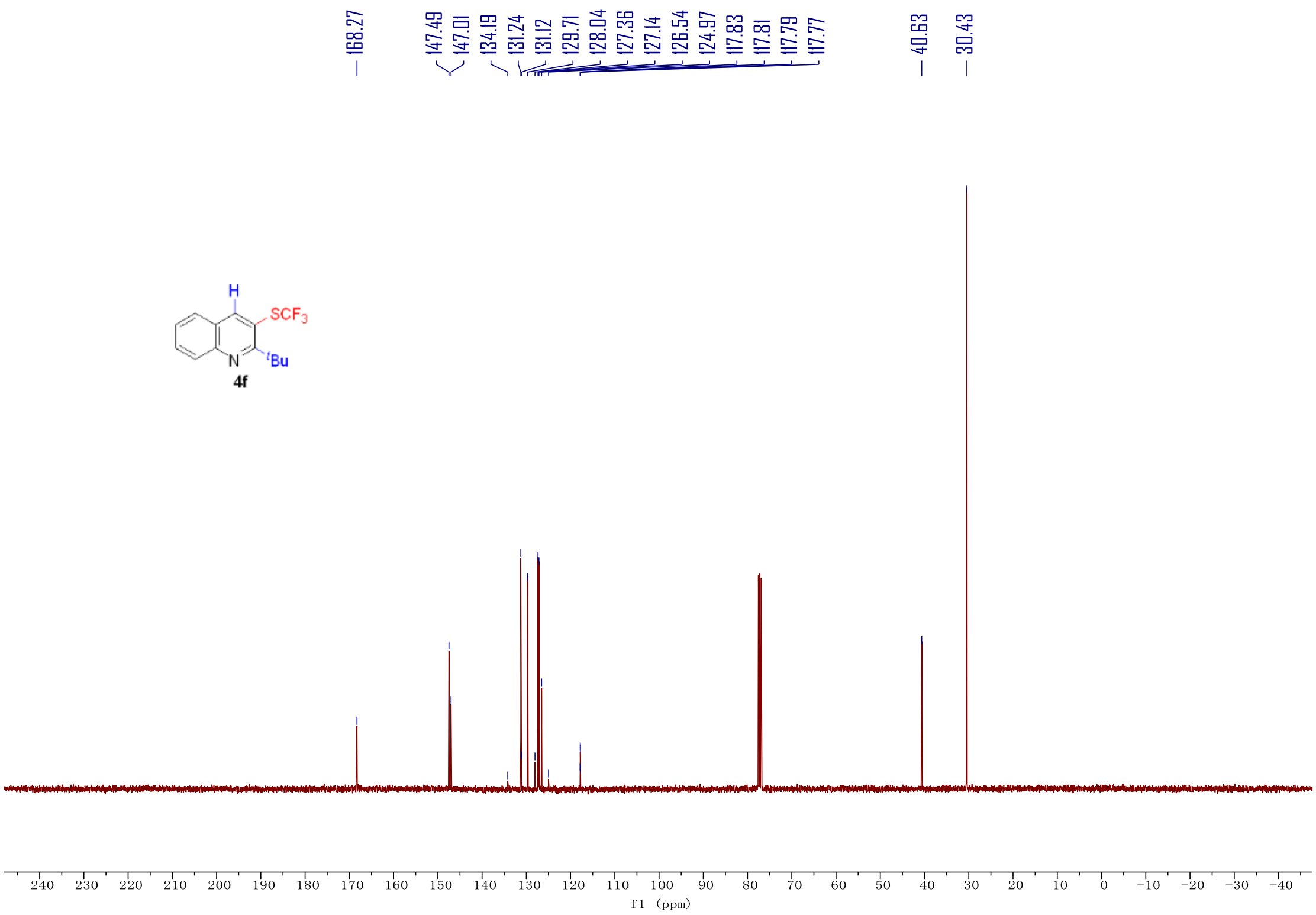

^{1}H NMR Spectrum of **4g** (400 MHz, $CDCl_3$)

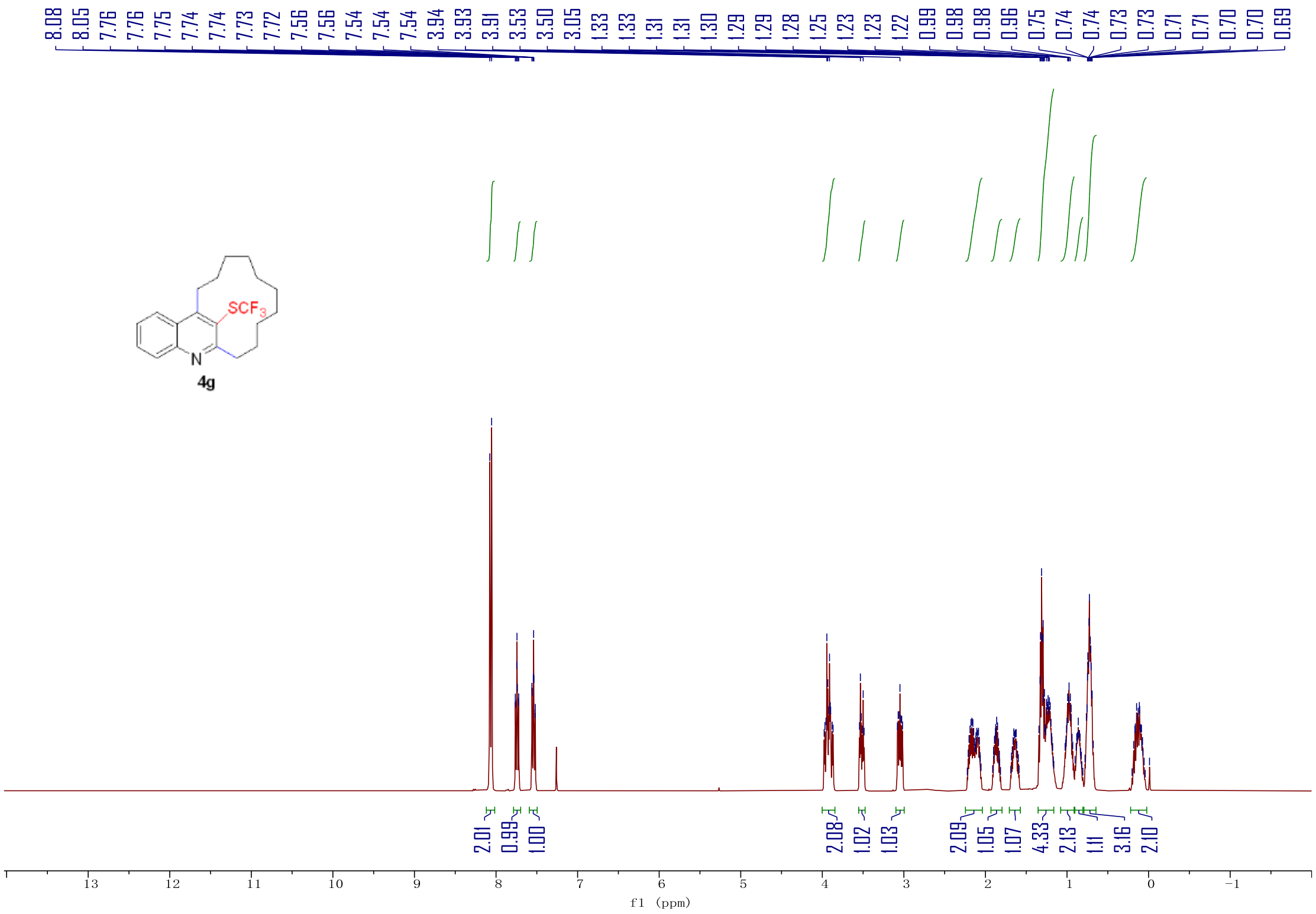

^{19}F NMR Spectrum of **4g** (376 MHz, $CDCl_3$)

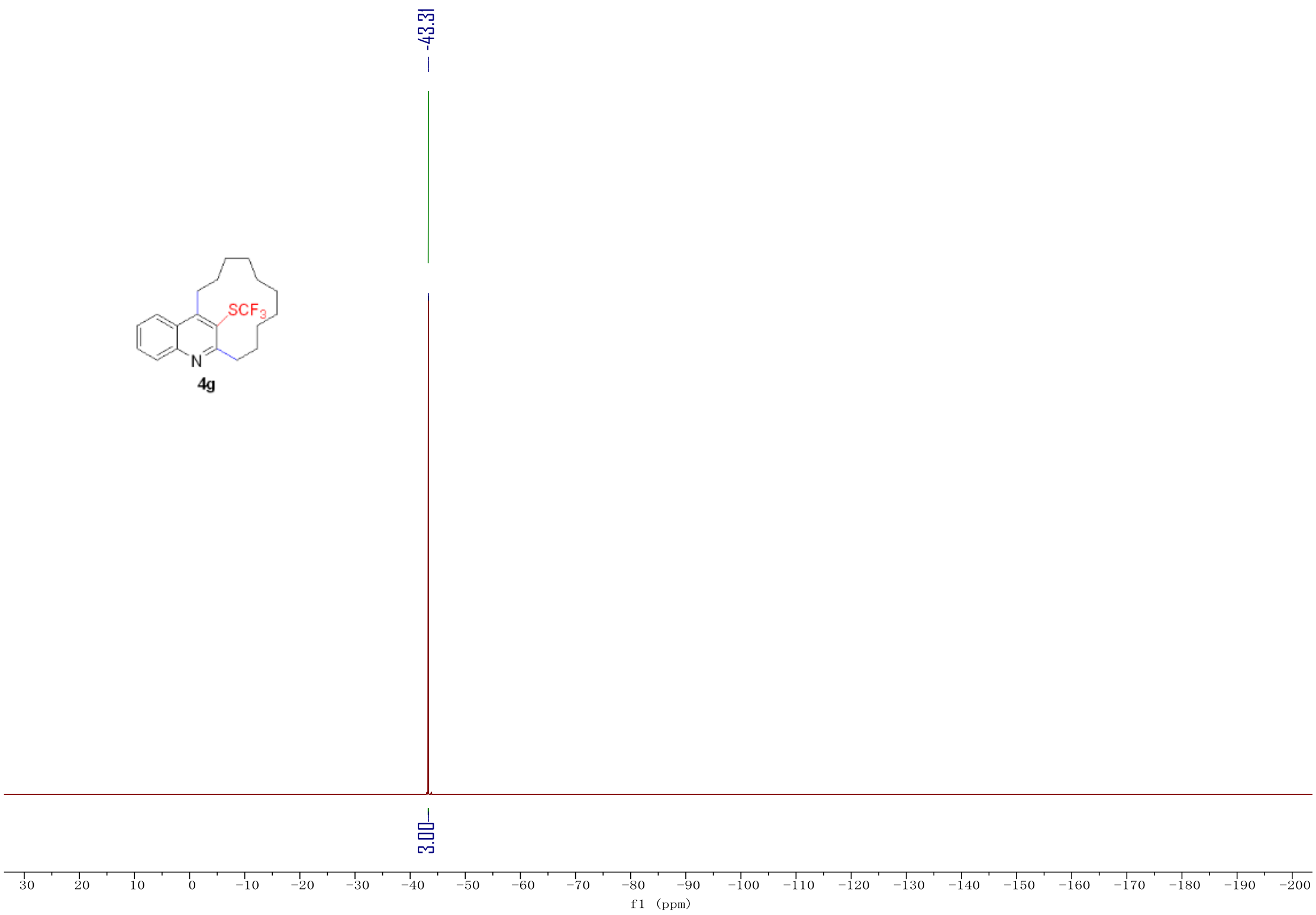

^{13}C NMR Spectrum of **4g** (101 MHz, $CDCl_3$)

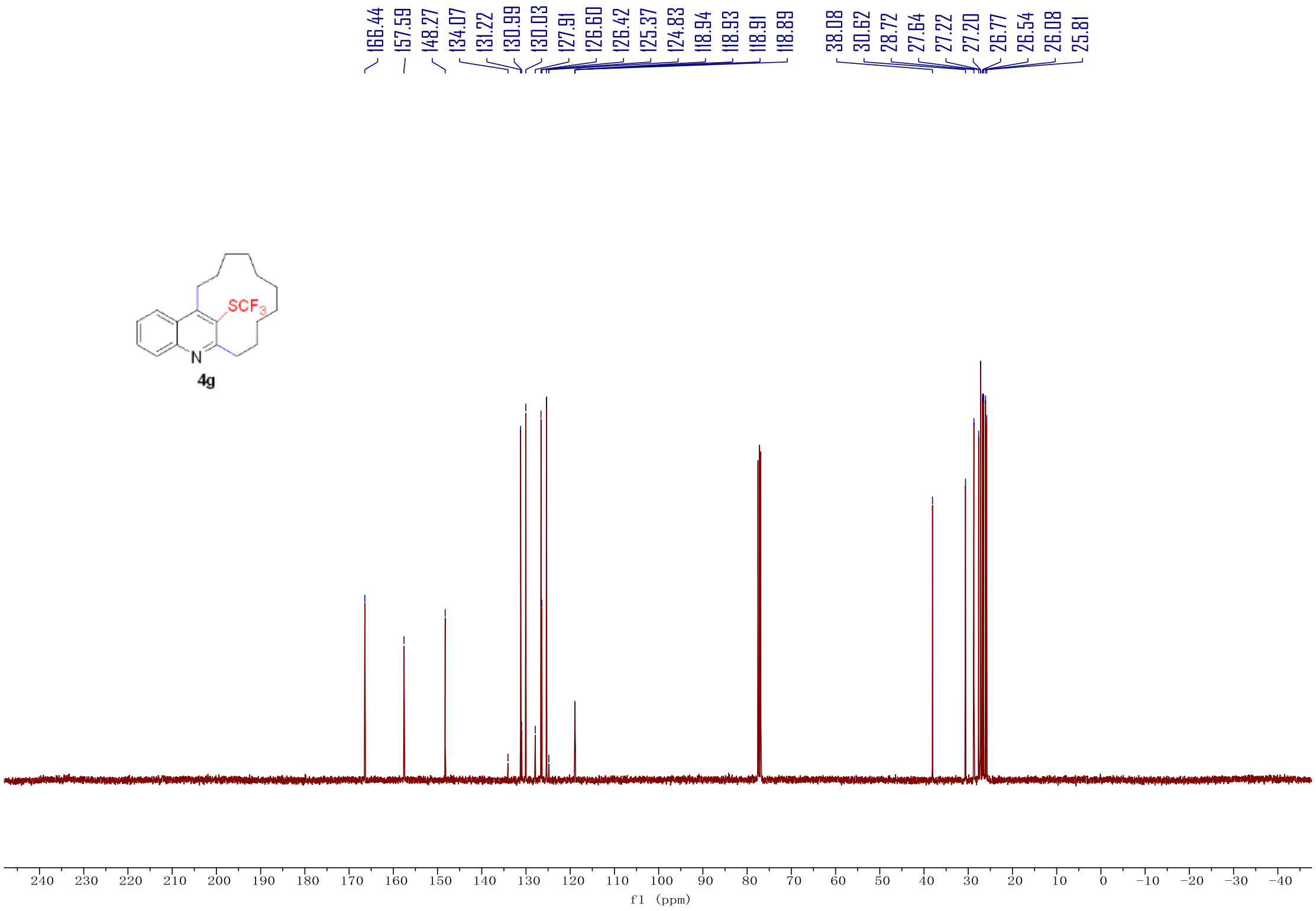

^{1}H NMR Spectrum of **4h** (400 MHz, $CDCl_3$)

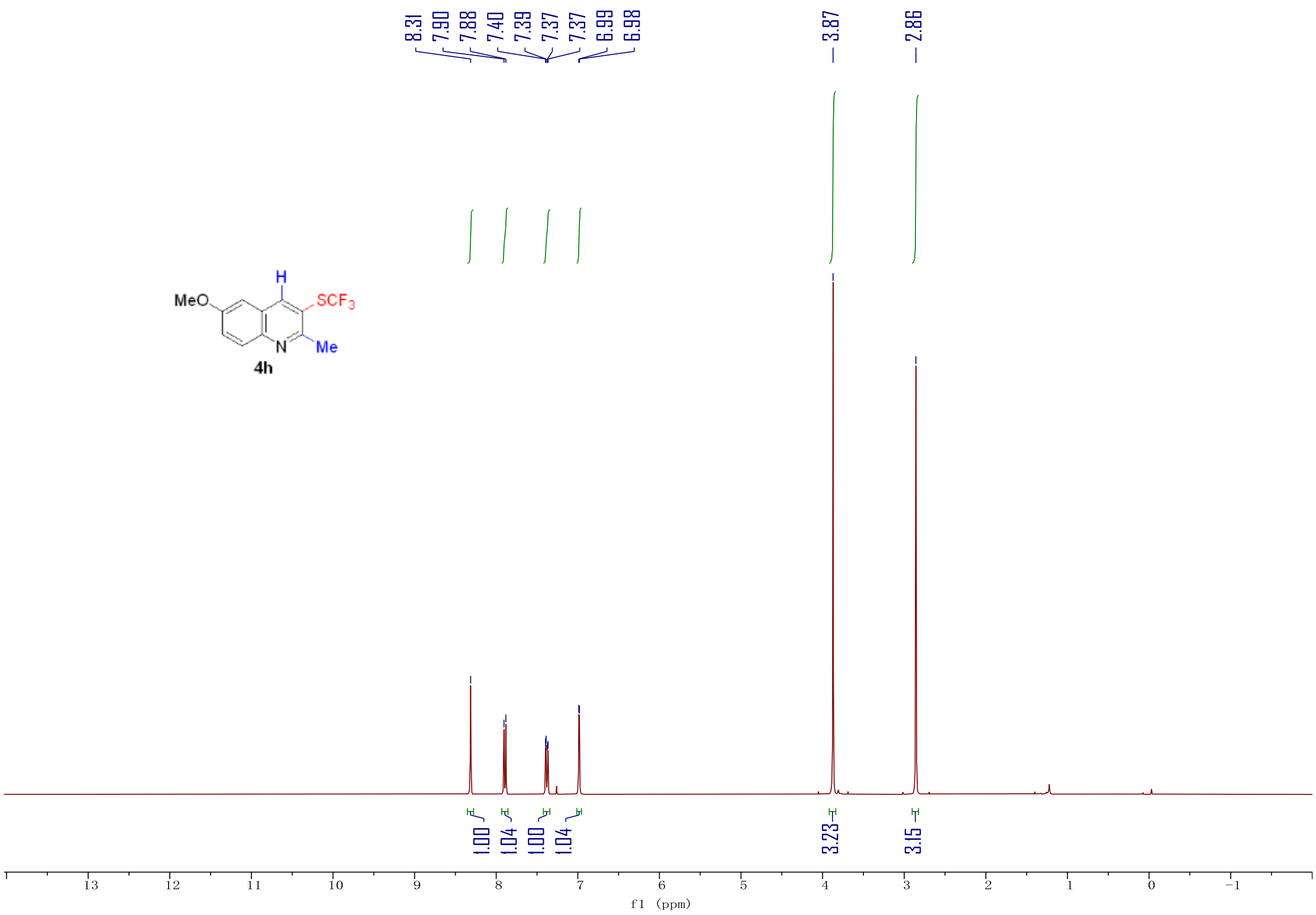

^{19}F NMR Spectrum of **4h** (376 MHz, $CDCl_3$)

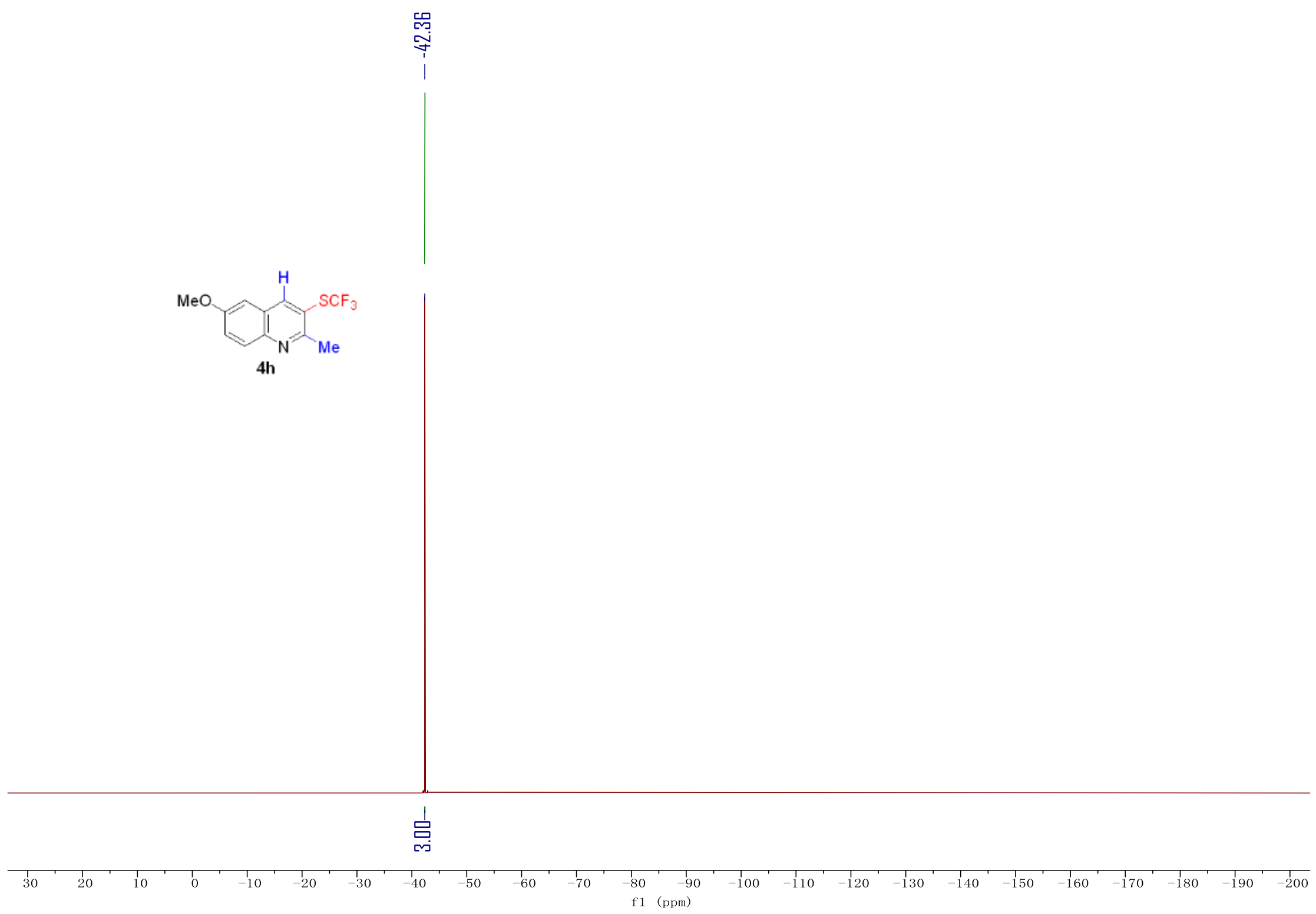

^{13}C NMR Spectrum of **4h** (101 MHz, $CDCl_3$)

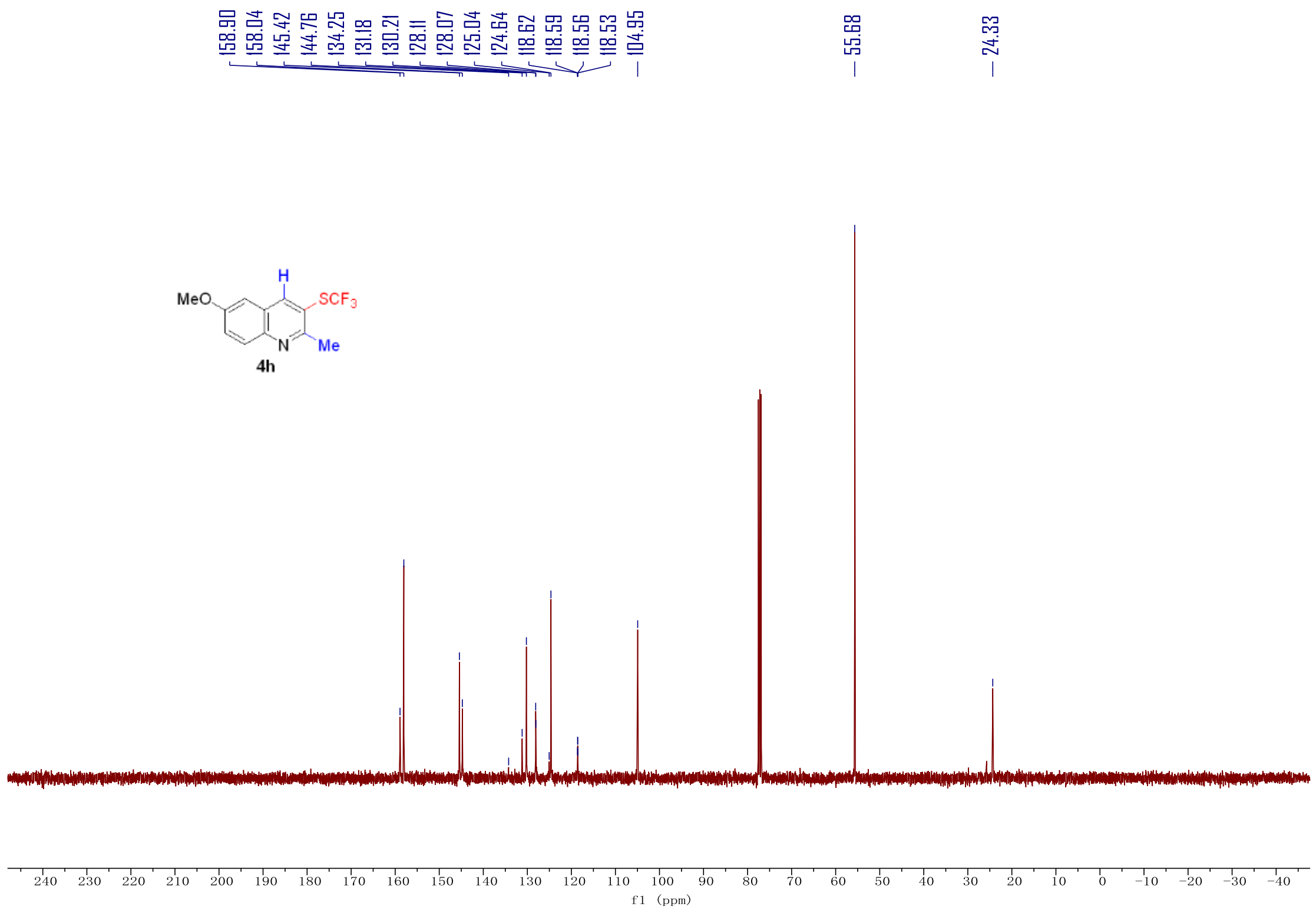

^{1}H NMR Spectrum of **4i** (400 MHz, $CDCl_3$)

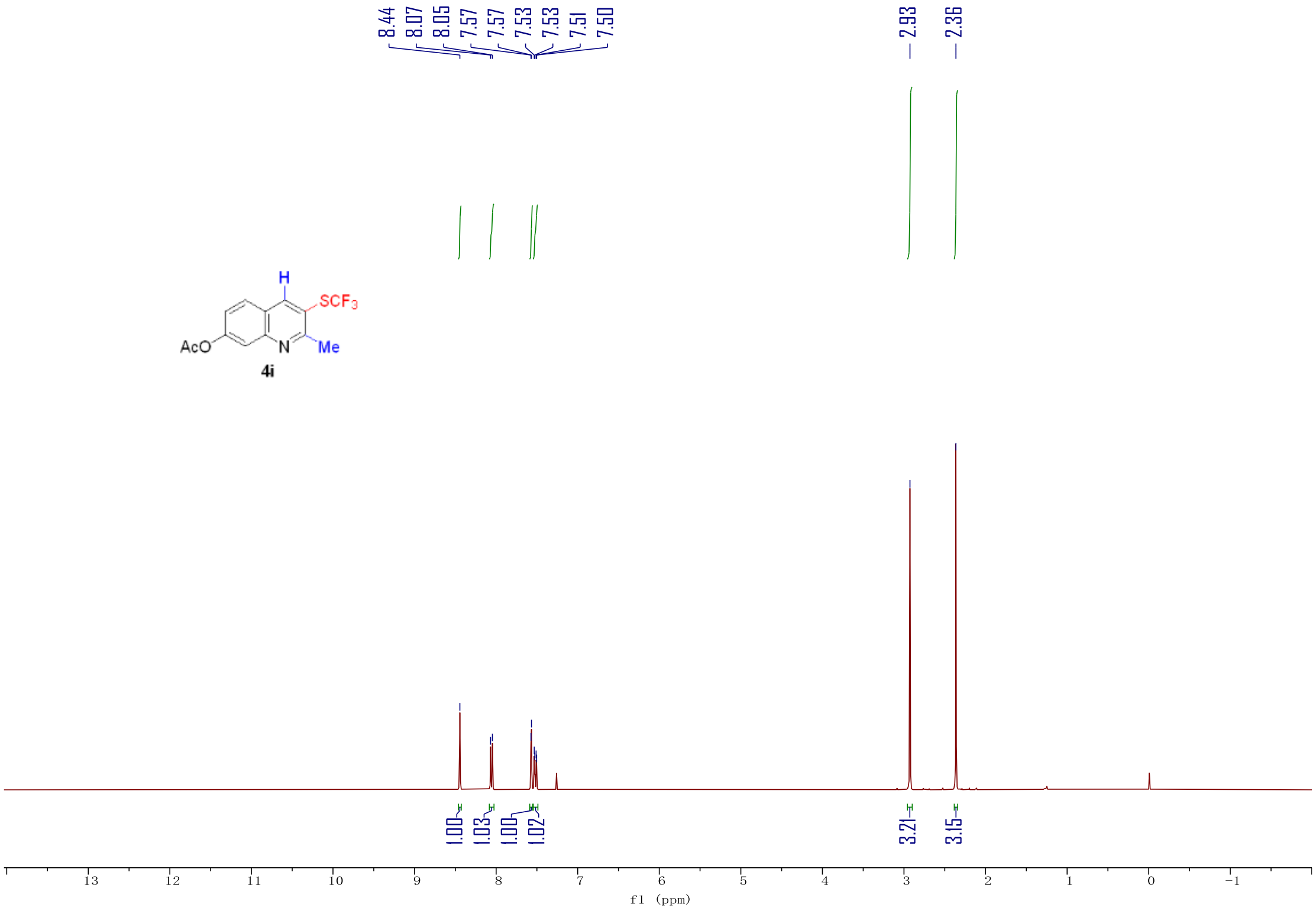

^{19}F NMR Spectrum of **4i** (376 MHz, $CDCl_3$)

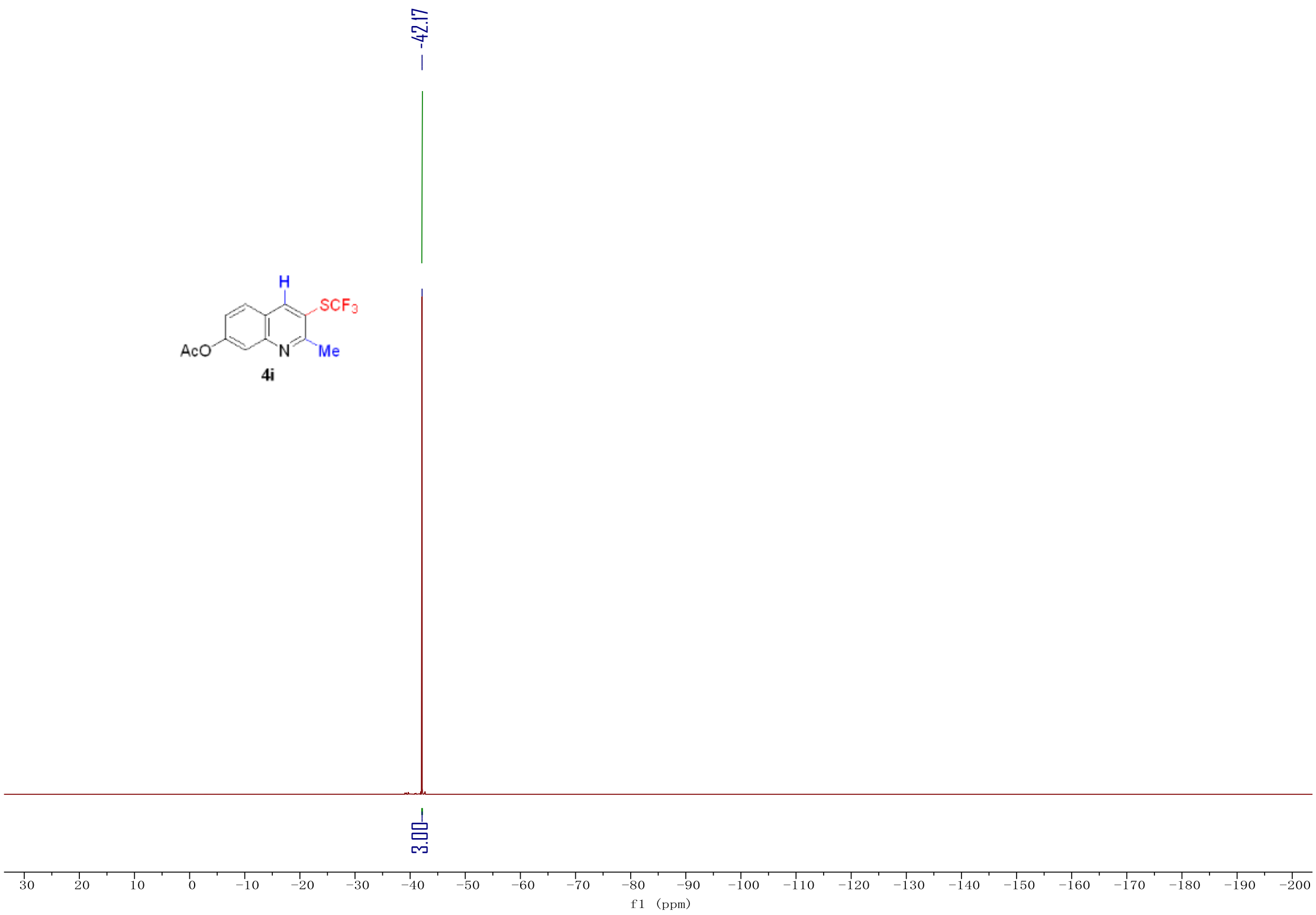

^{13}C NMR Spectrum of **4i** (101 MHz, $CDCl_3$)

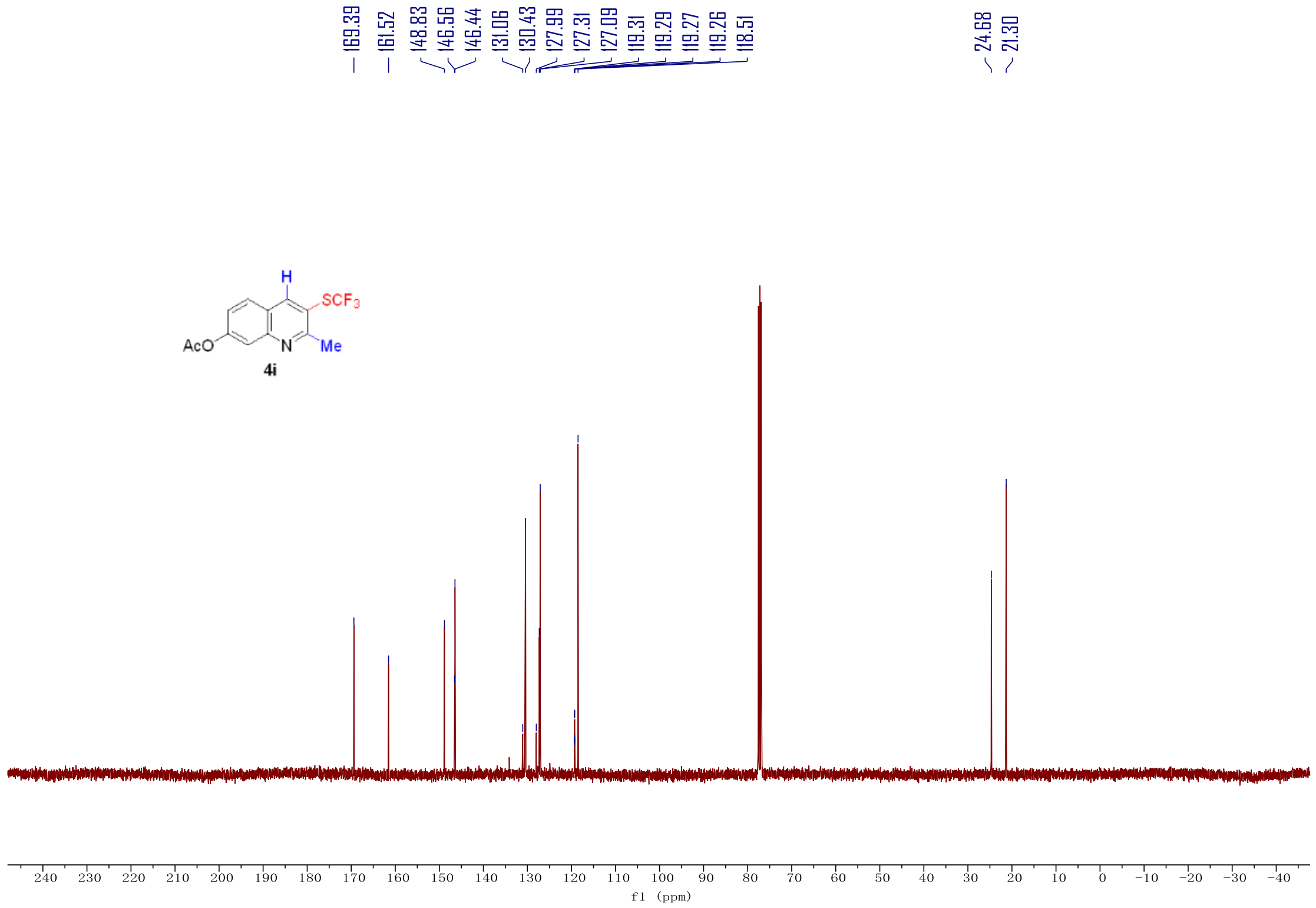

^{1}H NMR Spectrum of **4j** (400 MHz, $CDCl_3$)

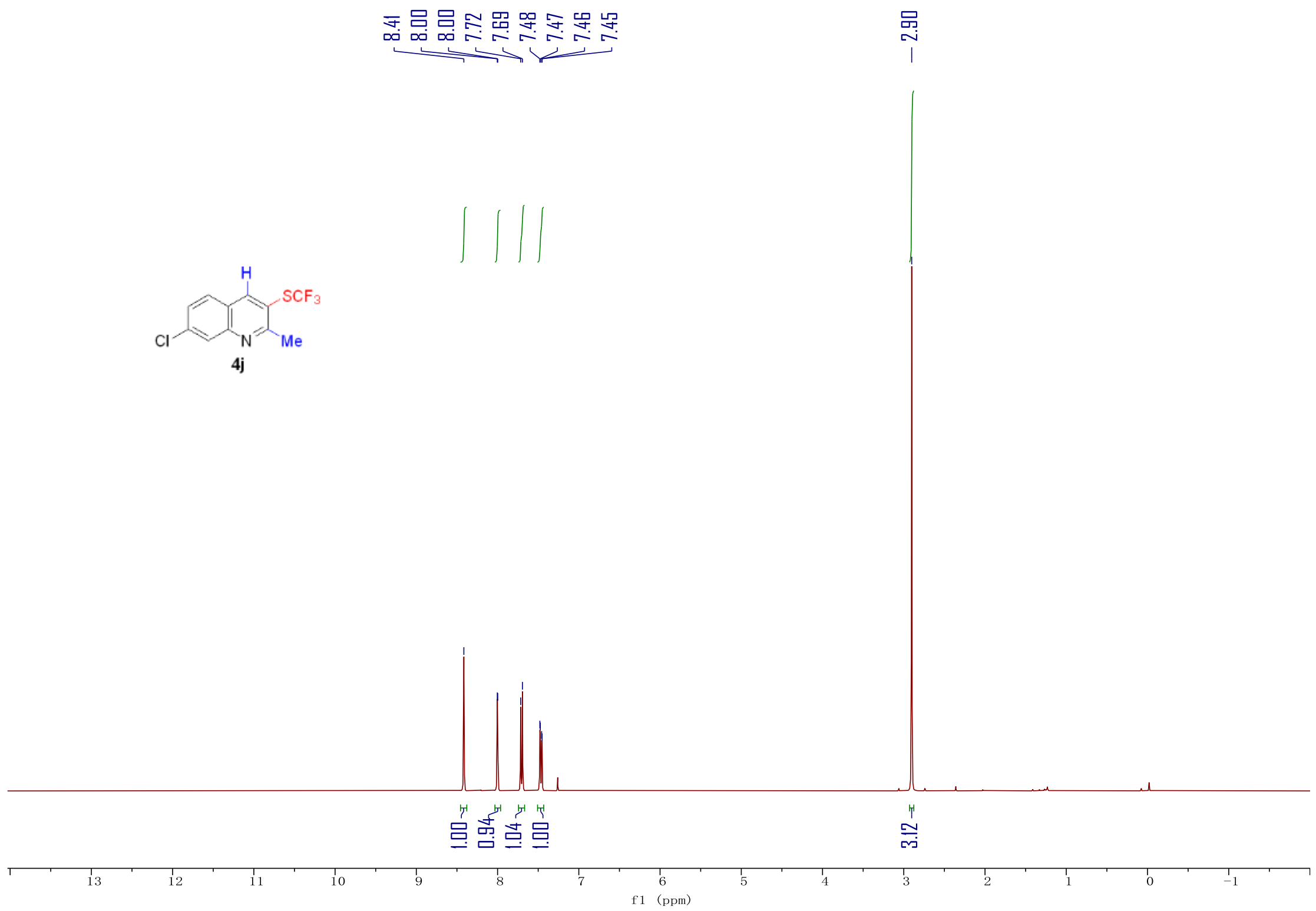

^{19}F NMR Spectrum of **4j** (376 MHz, $CDCl_3$)

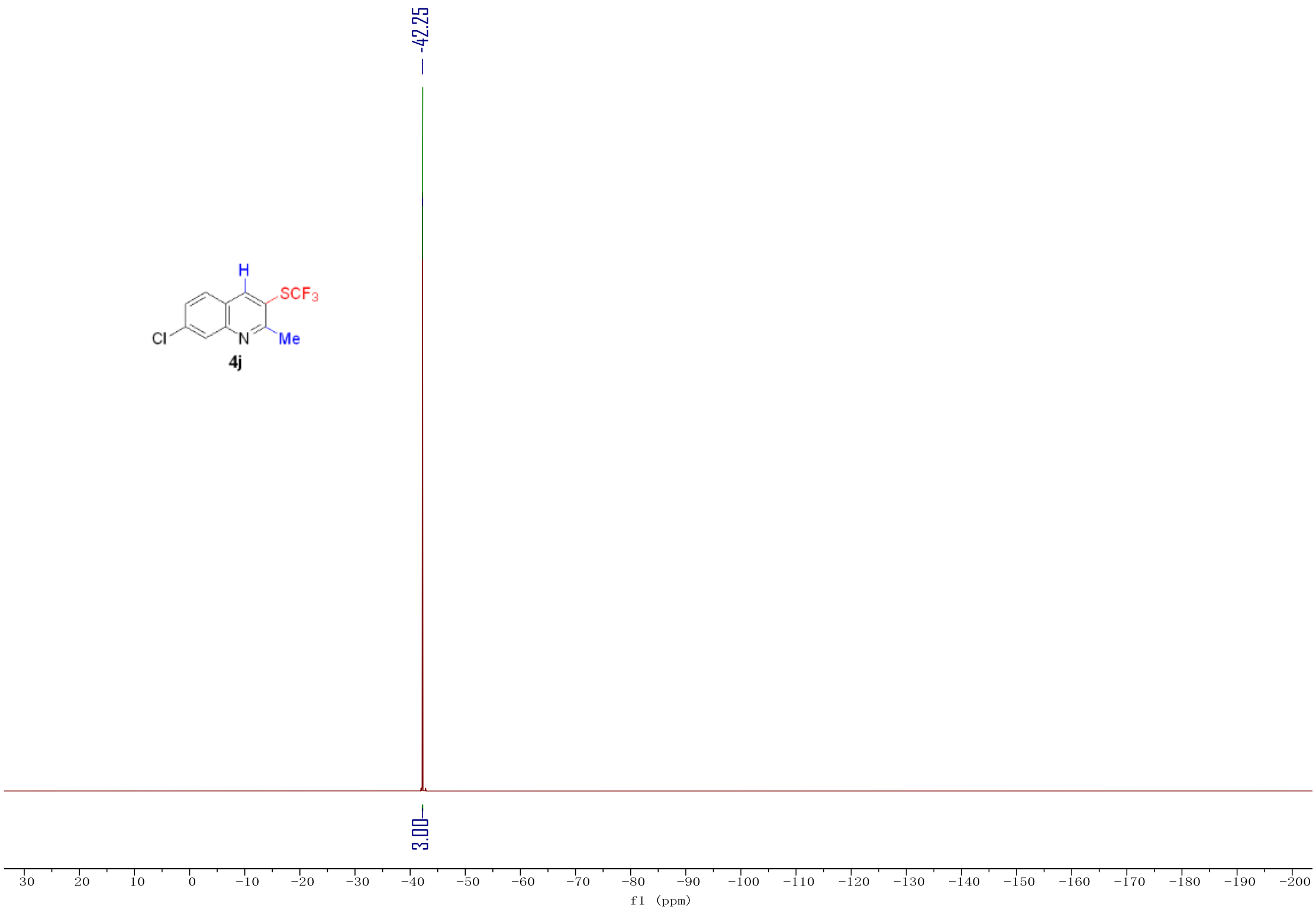

^{13}C NMR Spectrum of **4j** (101 MHz, $CDCl_3$)

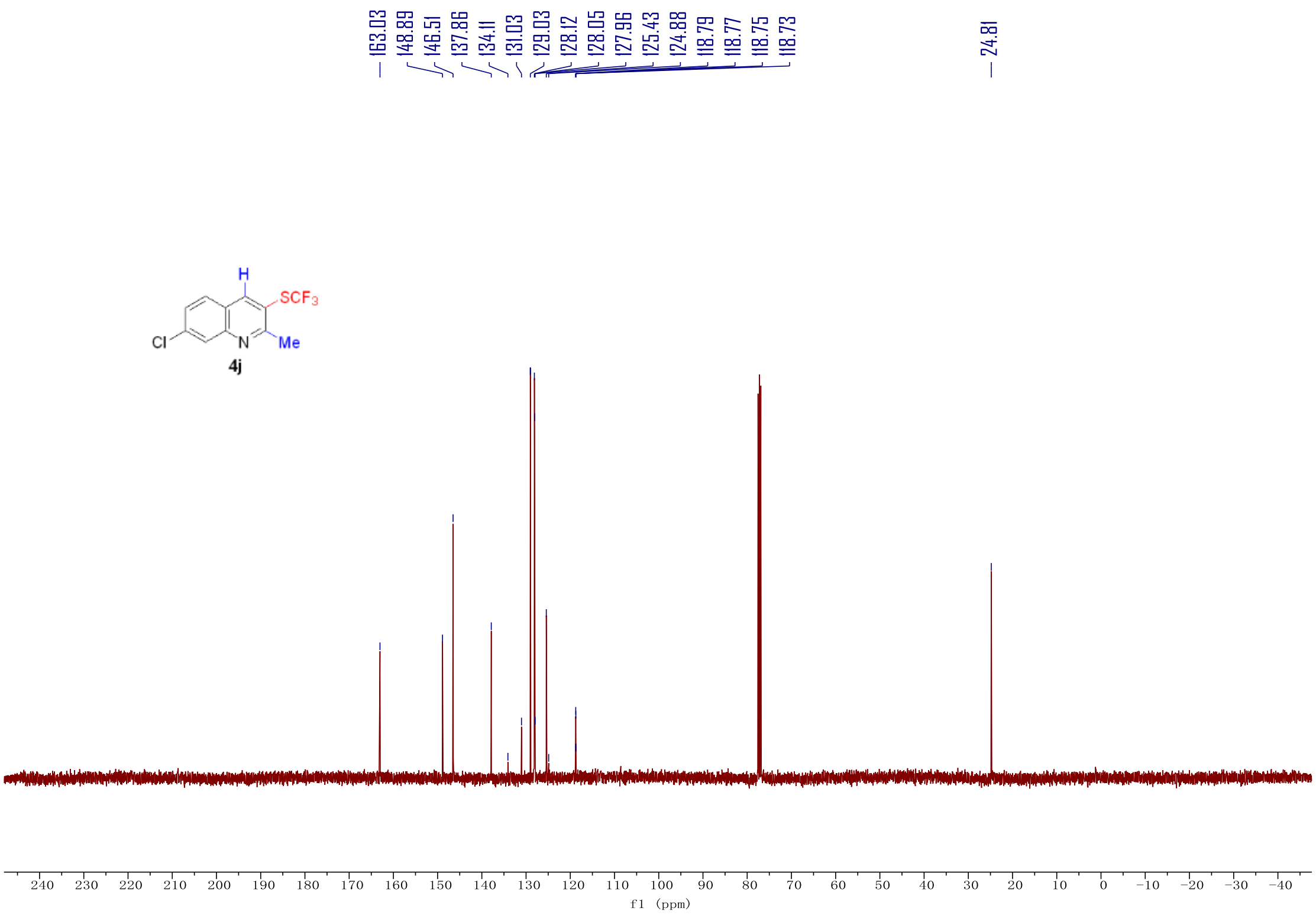

^{1}**H NMR** Spectrum of **4k** (400 MHz, $CDCl_3$)

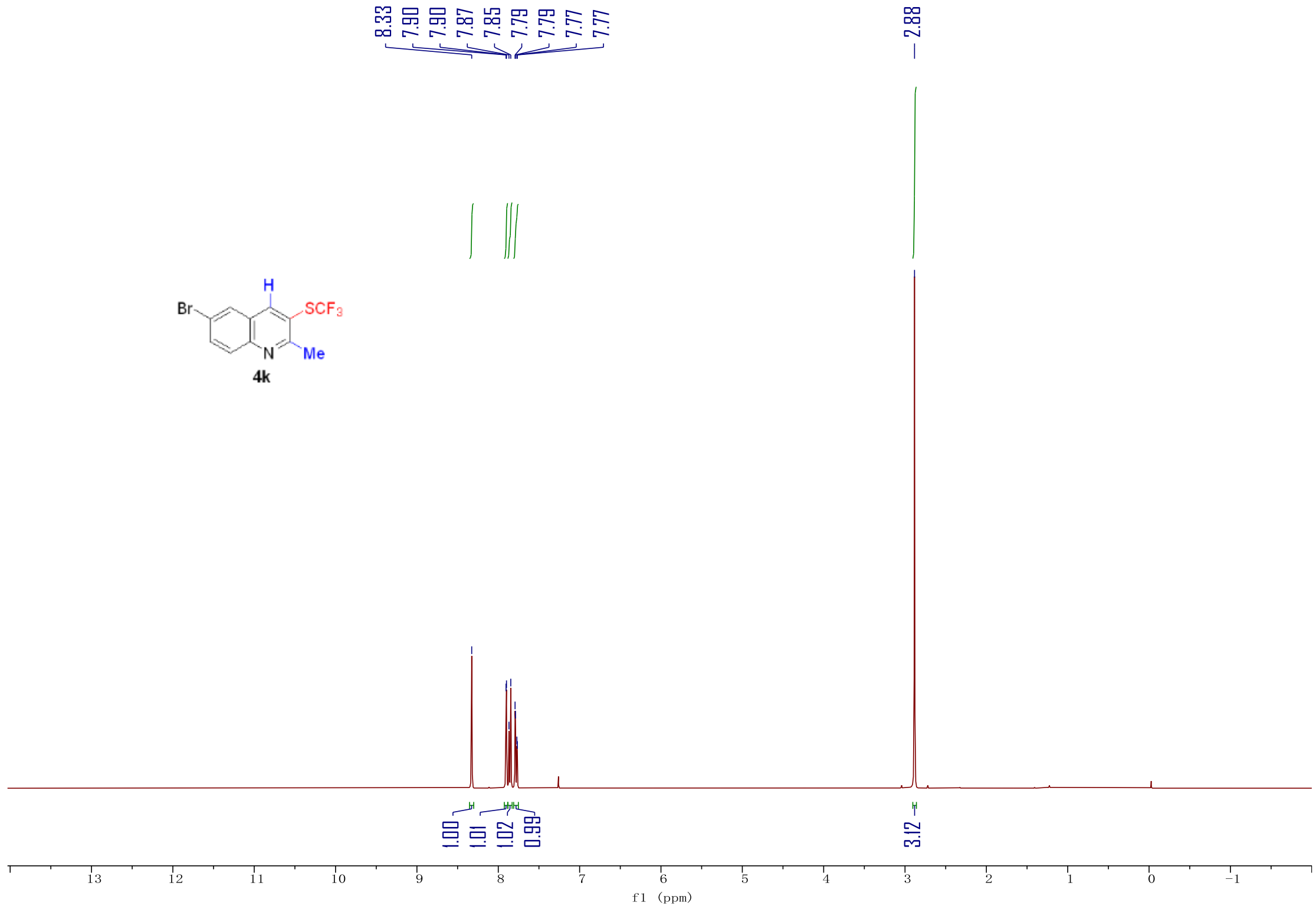

^{19}**F NMR** Spectrum of **4k** (376 MHz, $CDCl_3$)

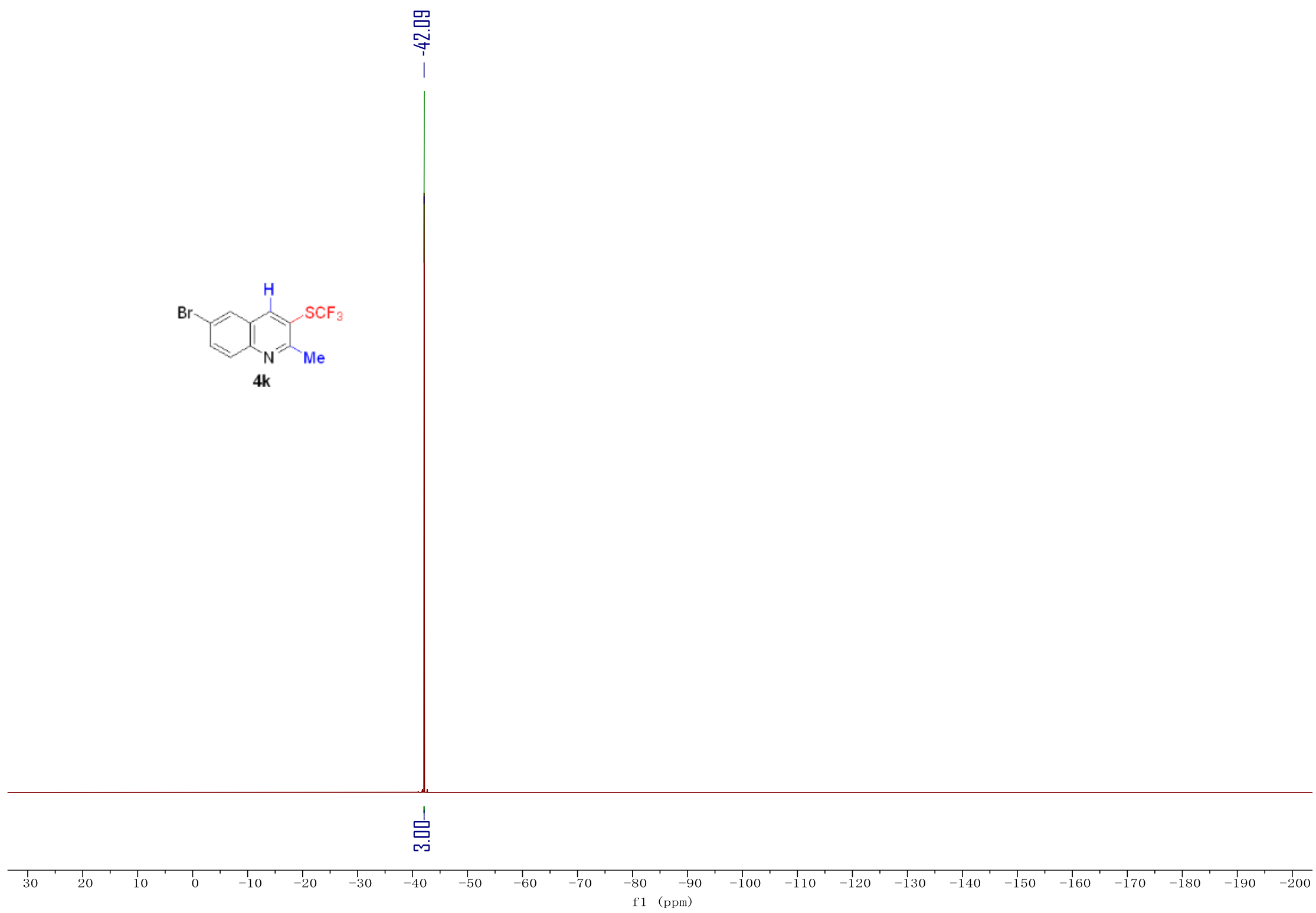

^{13}C NMR Spectrum of **4k** (101 MHz, $CDCl_3$)

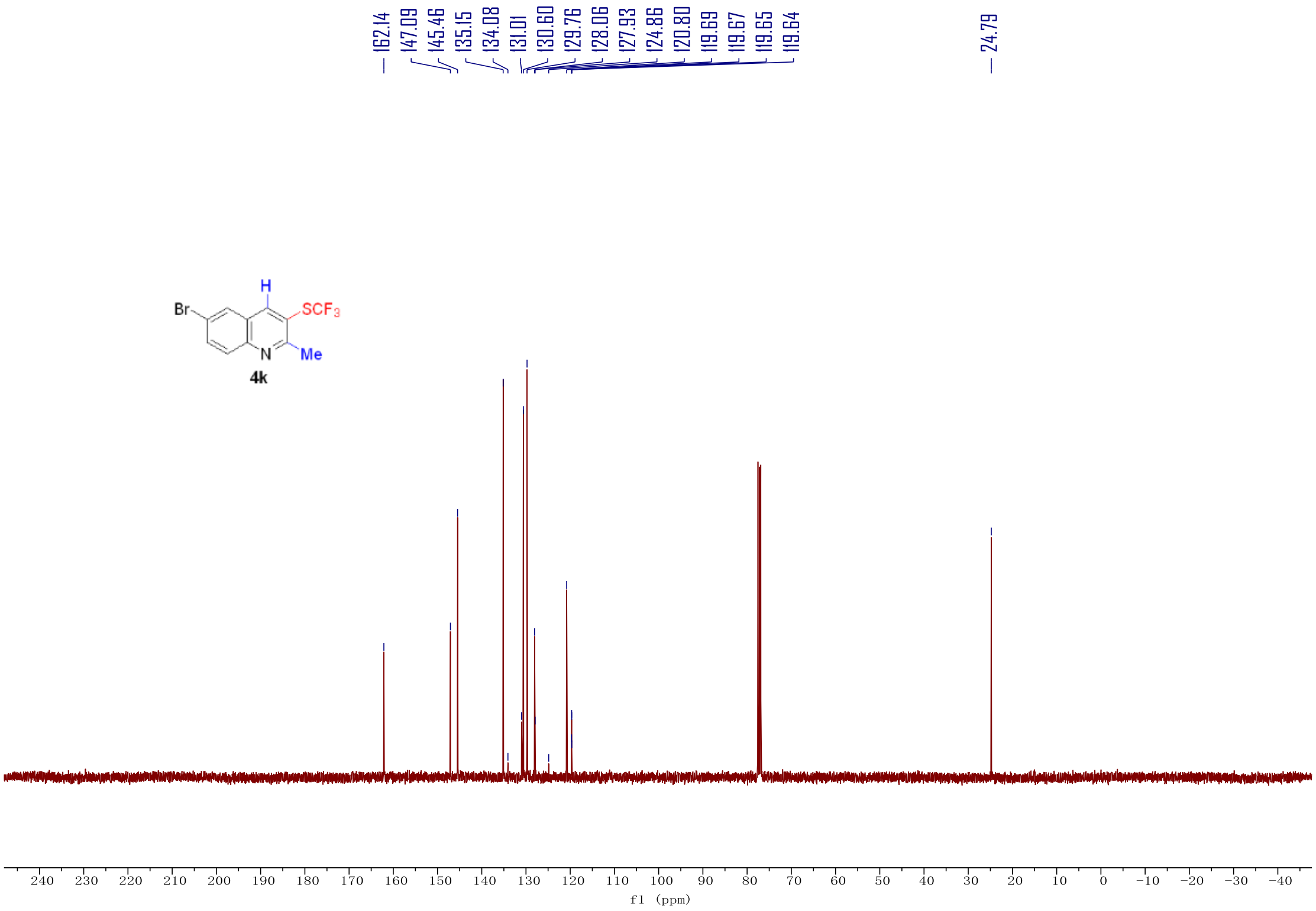

^{1}H NMR Spectrum of **4l** (400 MHz, $CDCl_3$)

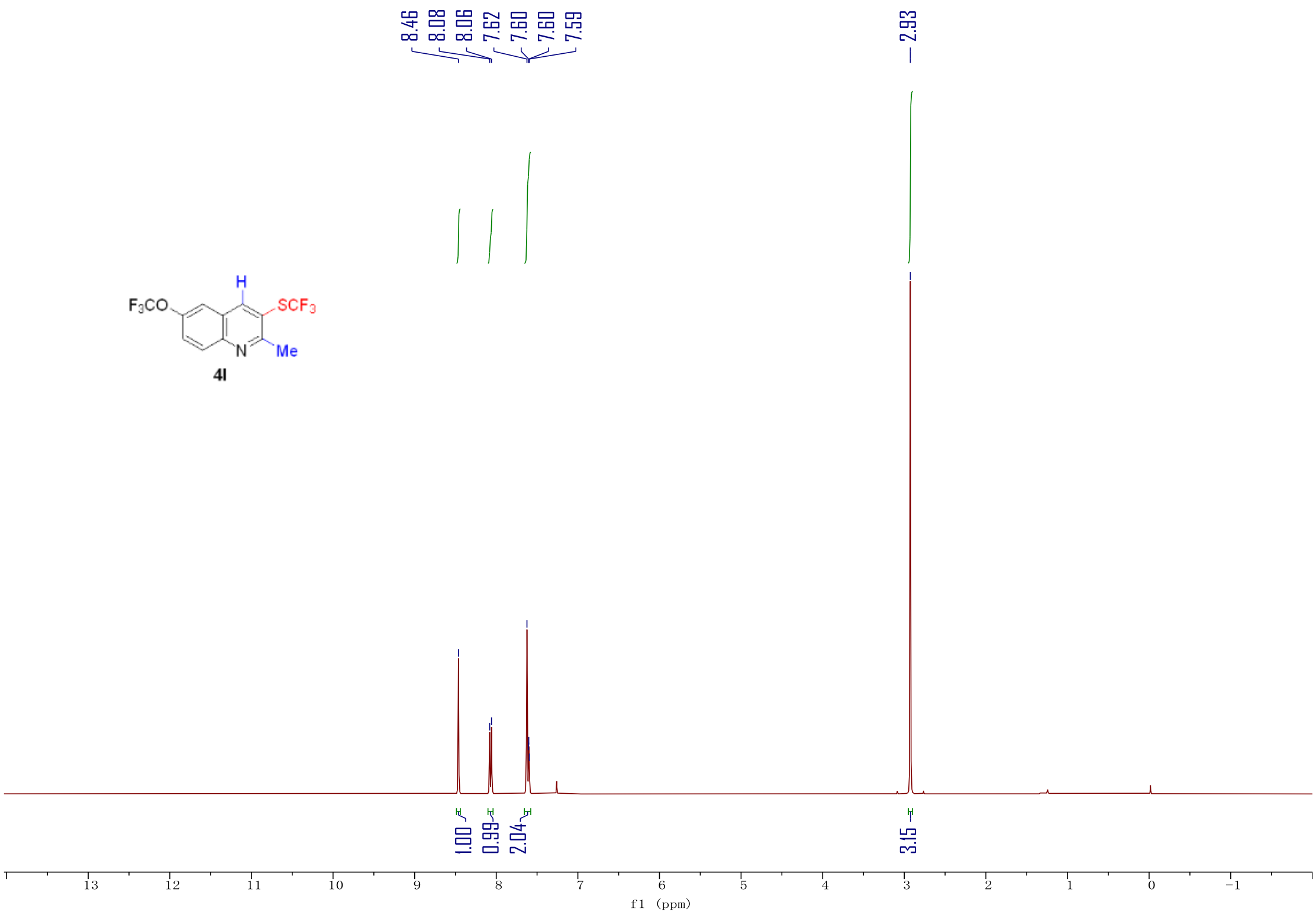

^{19}F NMR Spectrum of **4l** (376 MHz, $CDCl_3$)

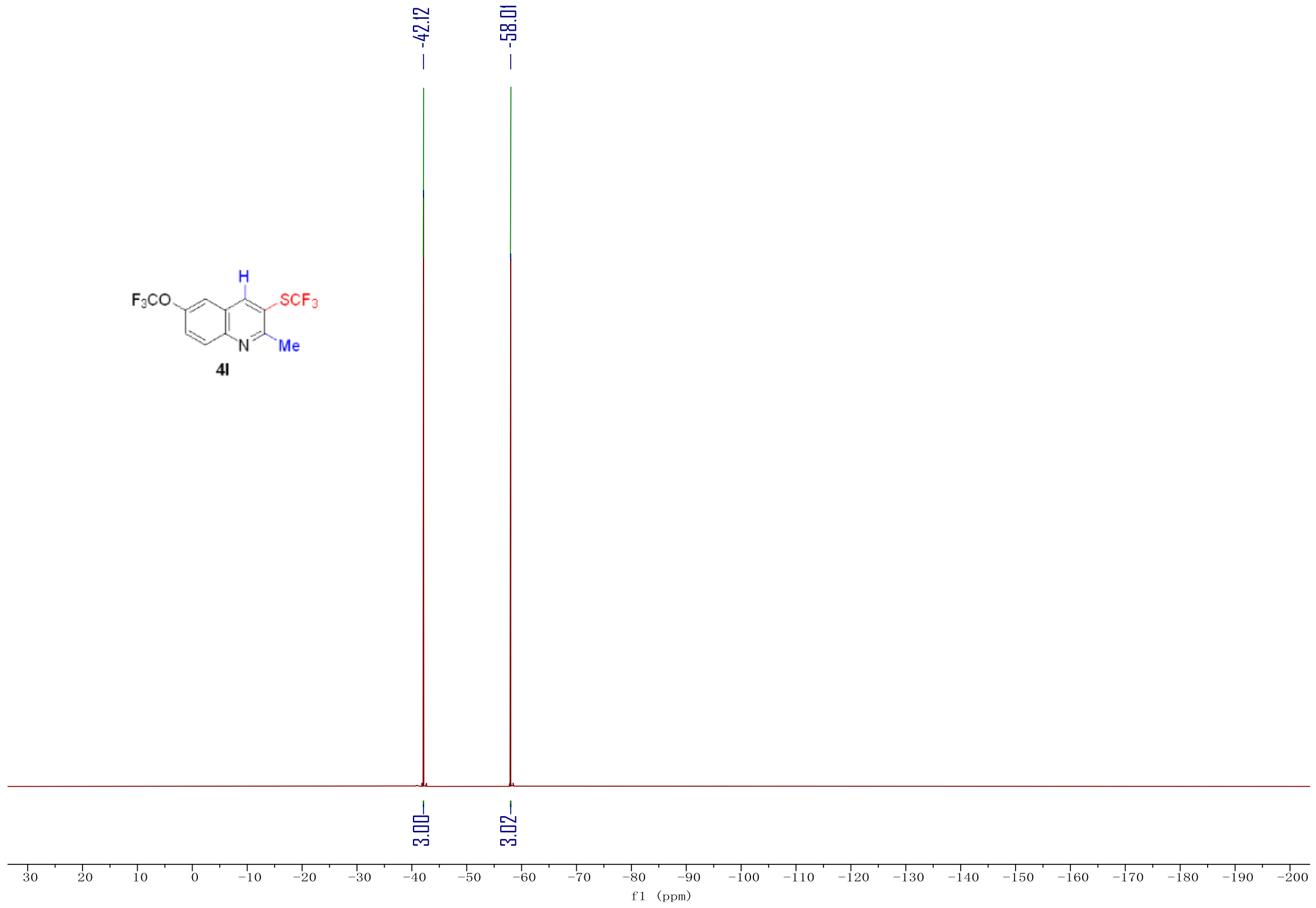

^{13}C NMR Spectrum of **4l** (101 MHz, $CDCl_3$)

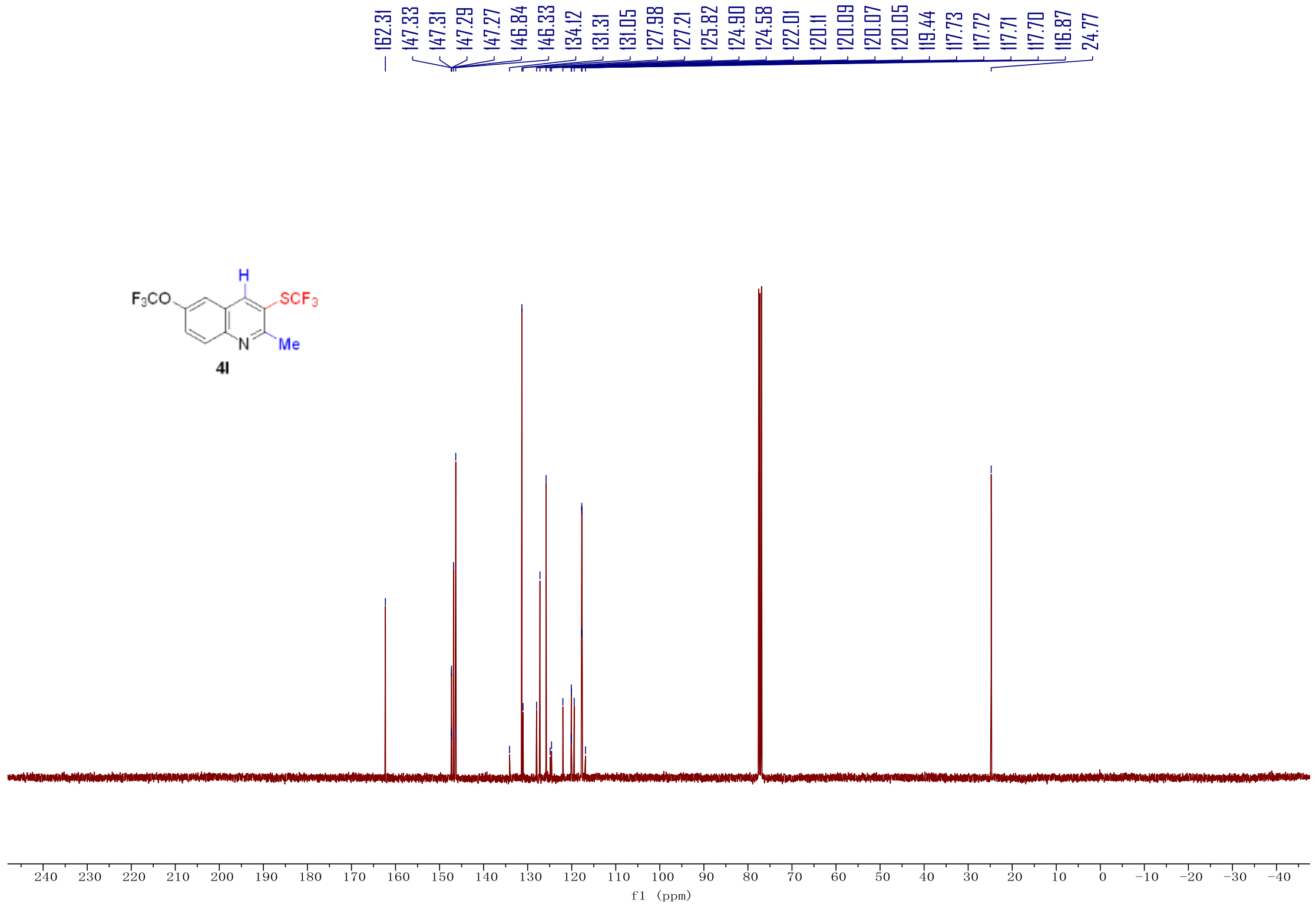

^{1}H NMR Spectrum of **4m** (400 MHz, $CDCl_3$)

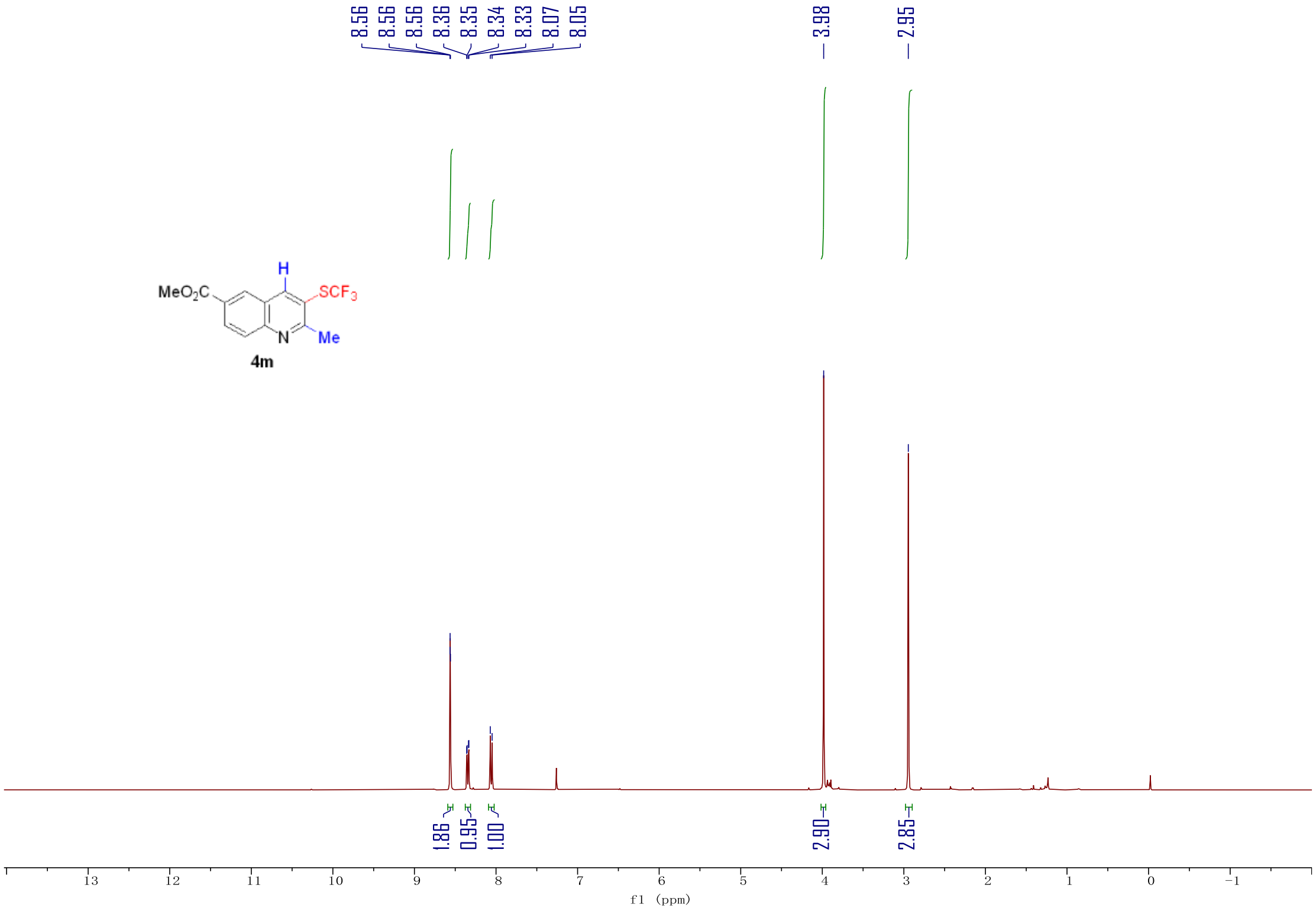

^{19}F NMR Spectrum of **4m** (376 MHz, $CDCl_3$)

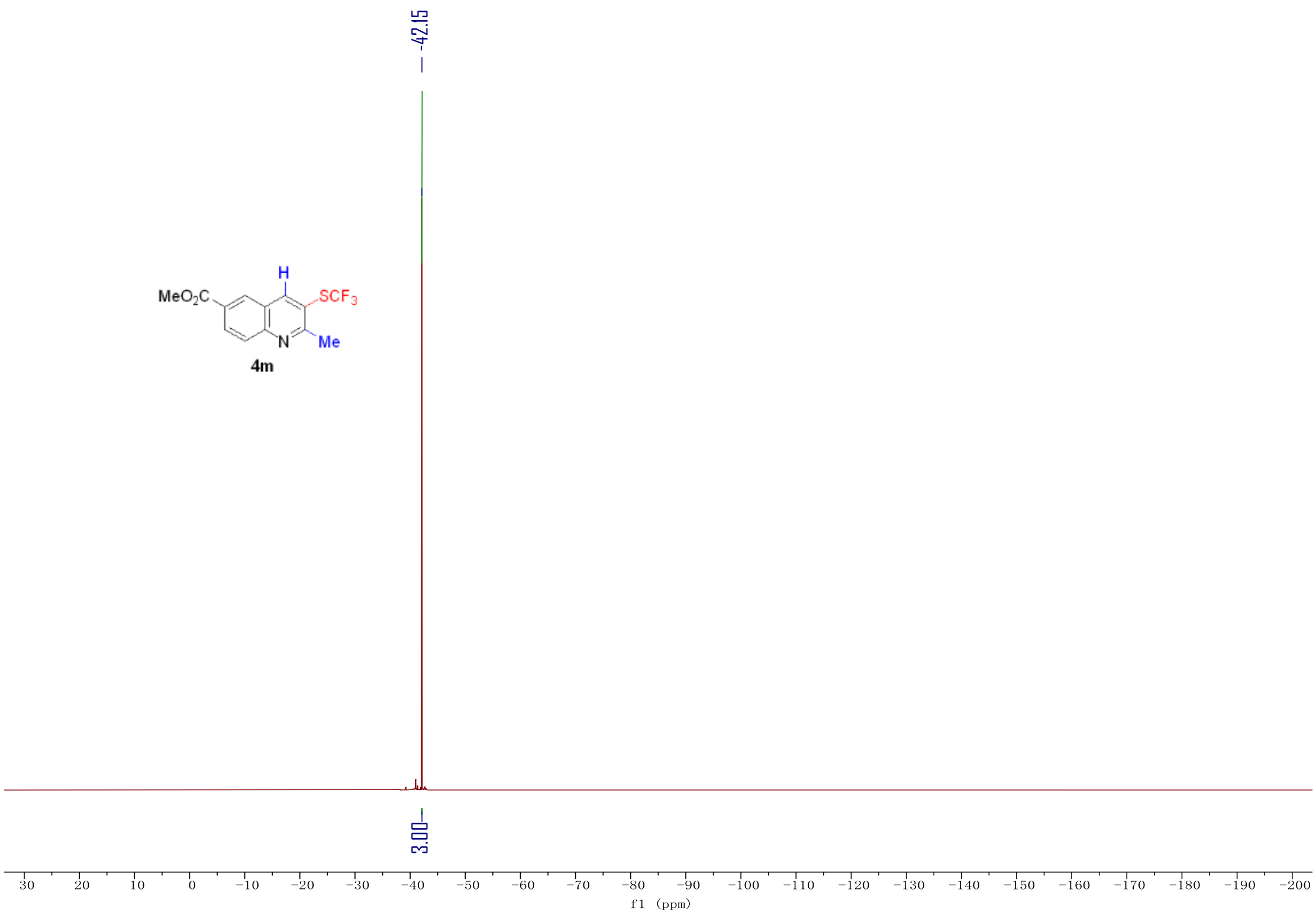

^{13}**C NMR** Spectrum of **4m** (101 MHz, $CDCl_3$)

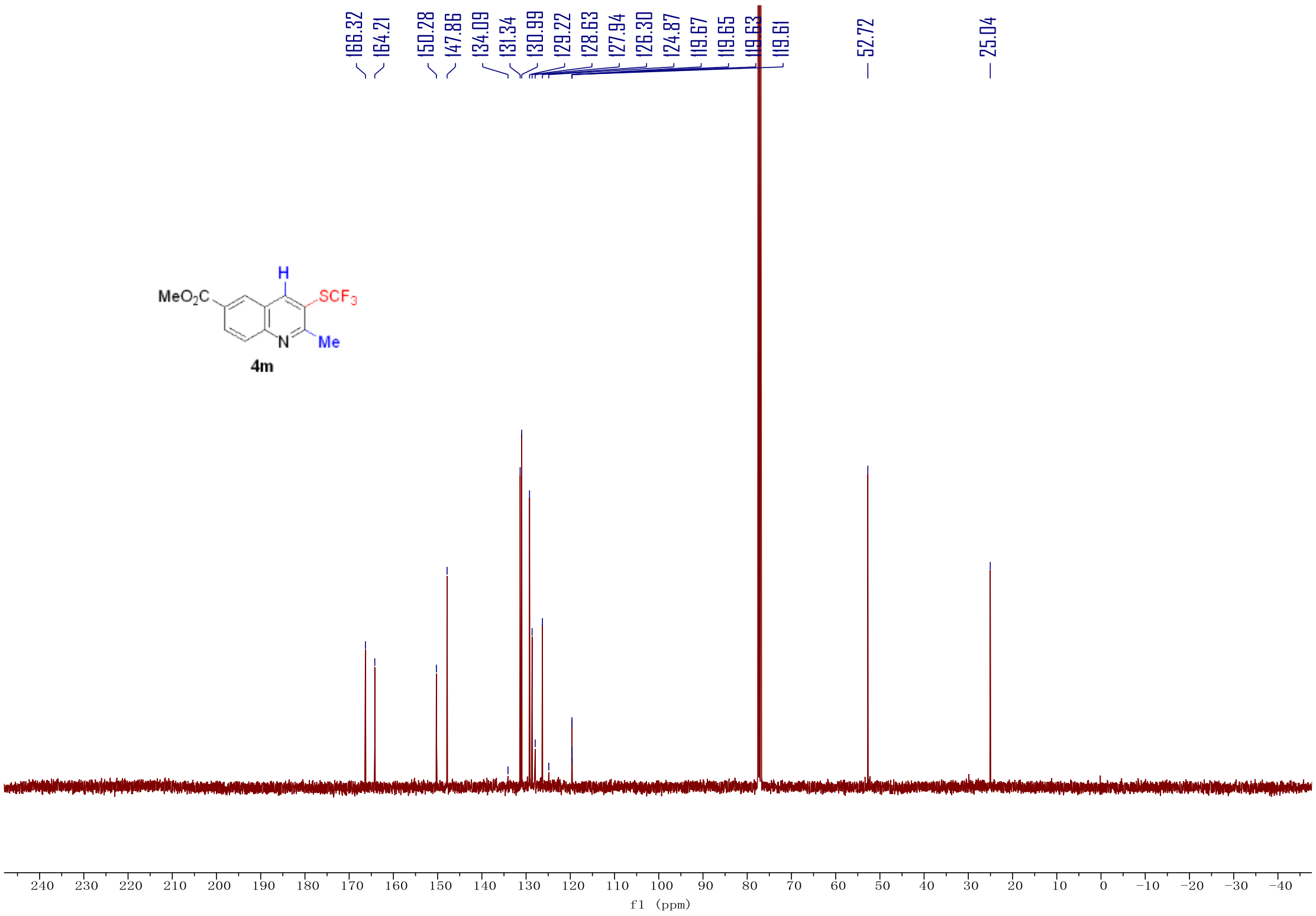

^{1}**H NMR** Spectrum of **4o** (400 MHz, $CDCl_3$)

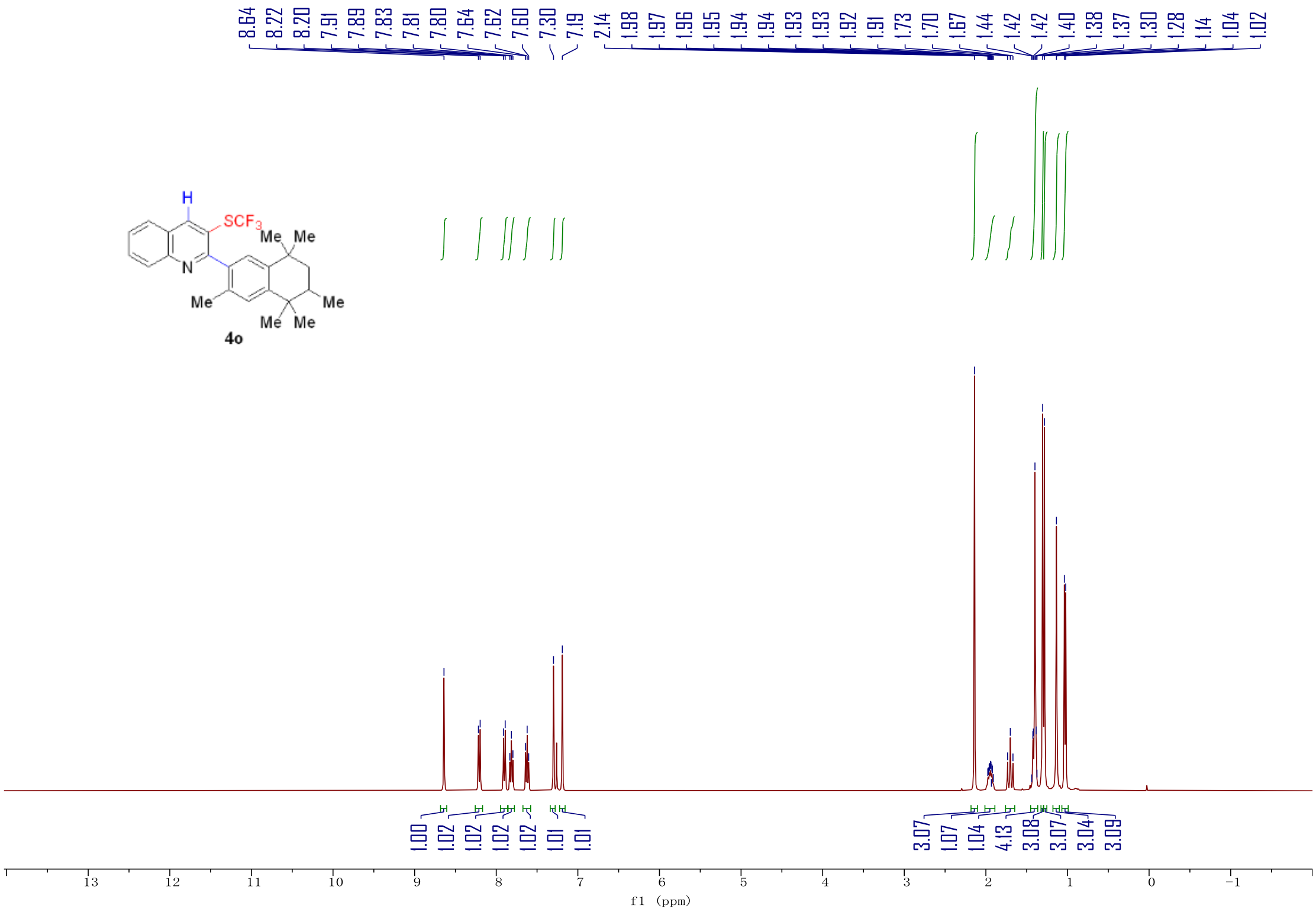

^{19}**F NMR** Spectrum of **4o** (376 MHz, $CDCl_3$)

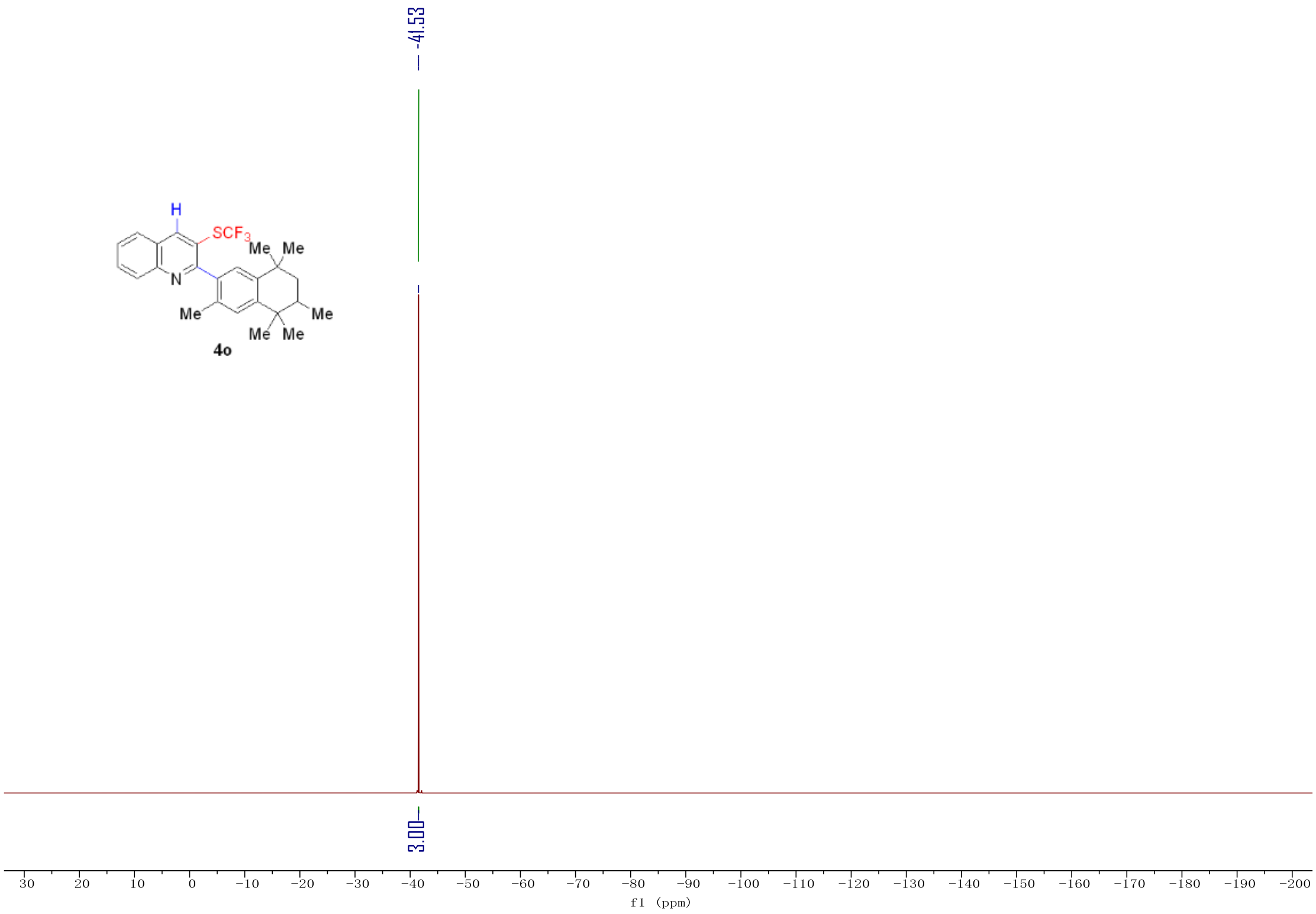

^{13}**C NMR** Spectrum of **4o** (101 MHz, $CDCl_3$)

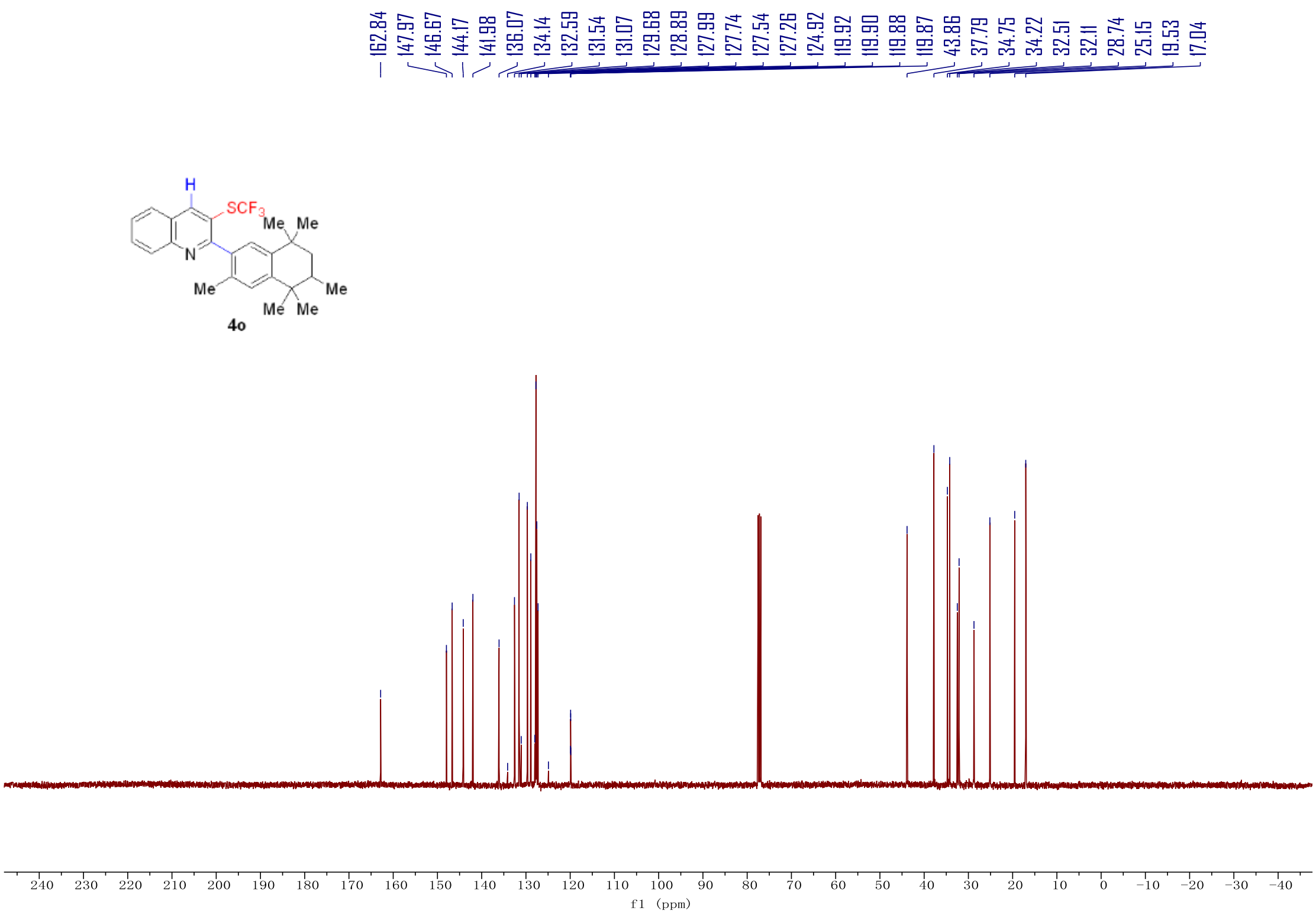

^{1}**H NMR** Spectrum of **4p** (400 MHz, $CDCl_3$)

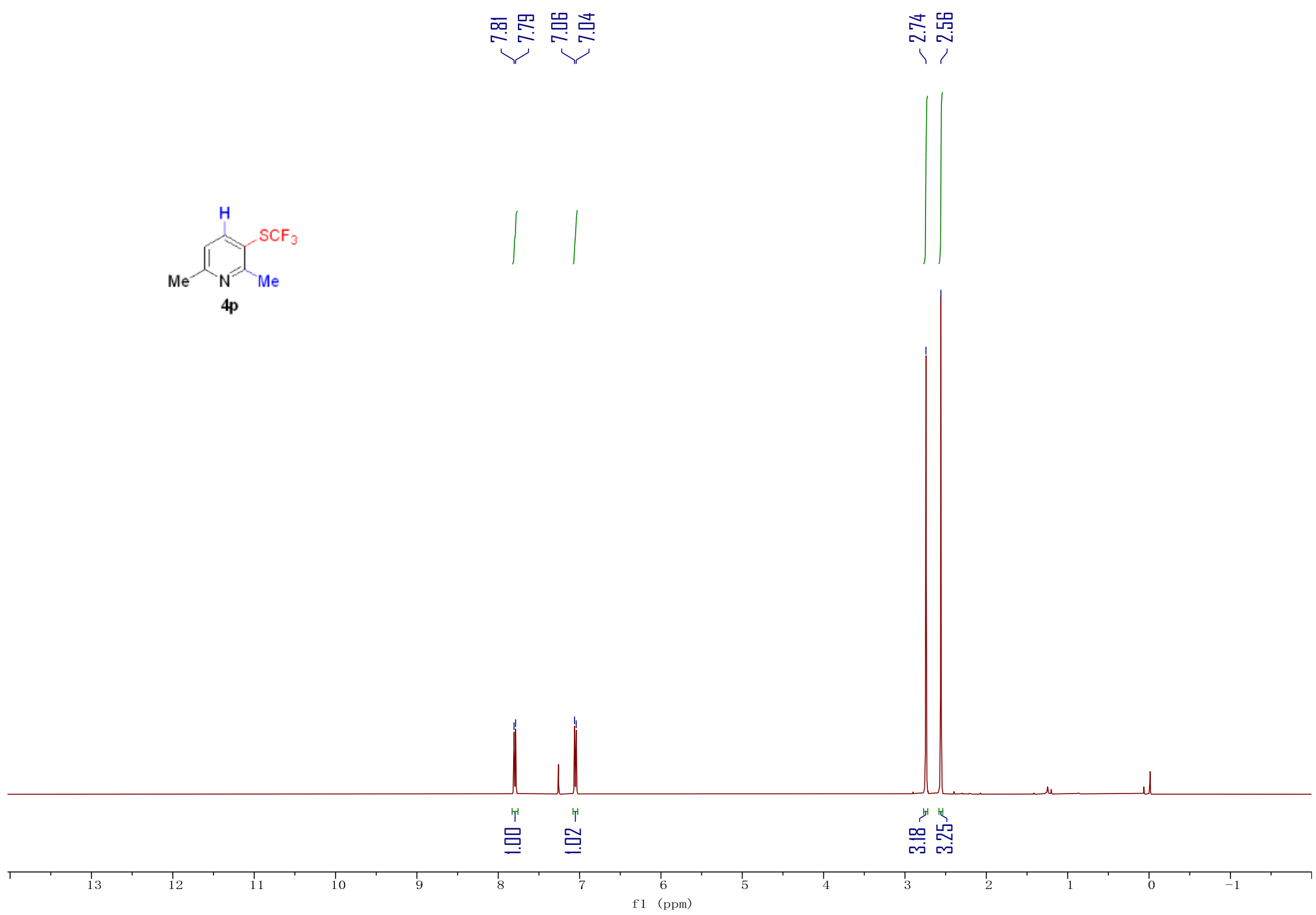

^{19}**F NMR** Spectrum of **4p** (376 MHz, $CDCl_3$)

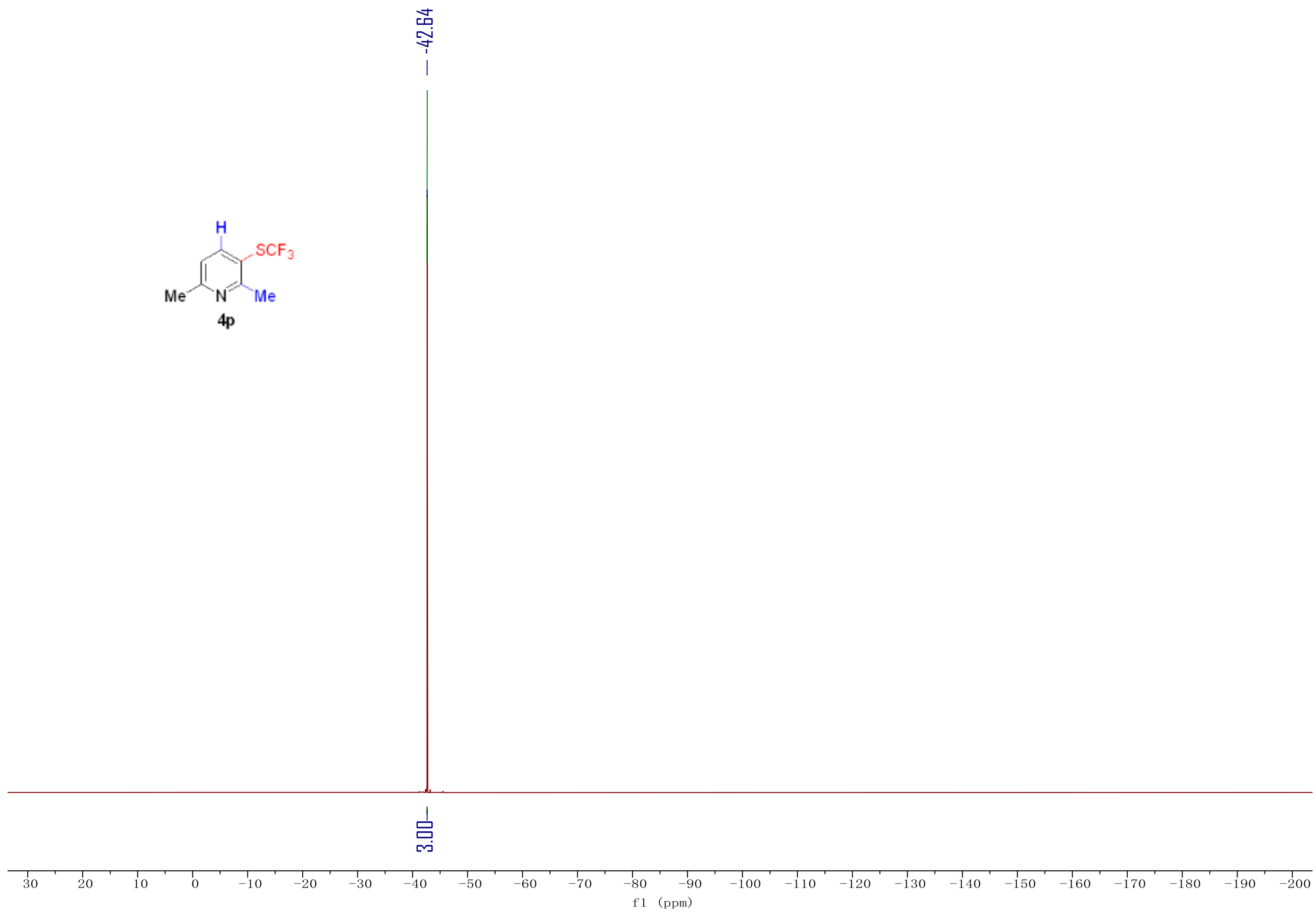

^{13}C NMR Spectrum of **4p** (101 MHz, $CDCl_3$)

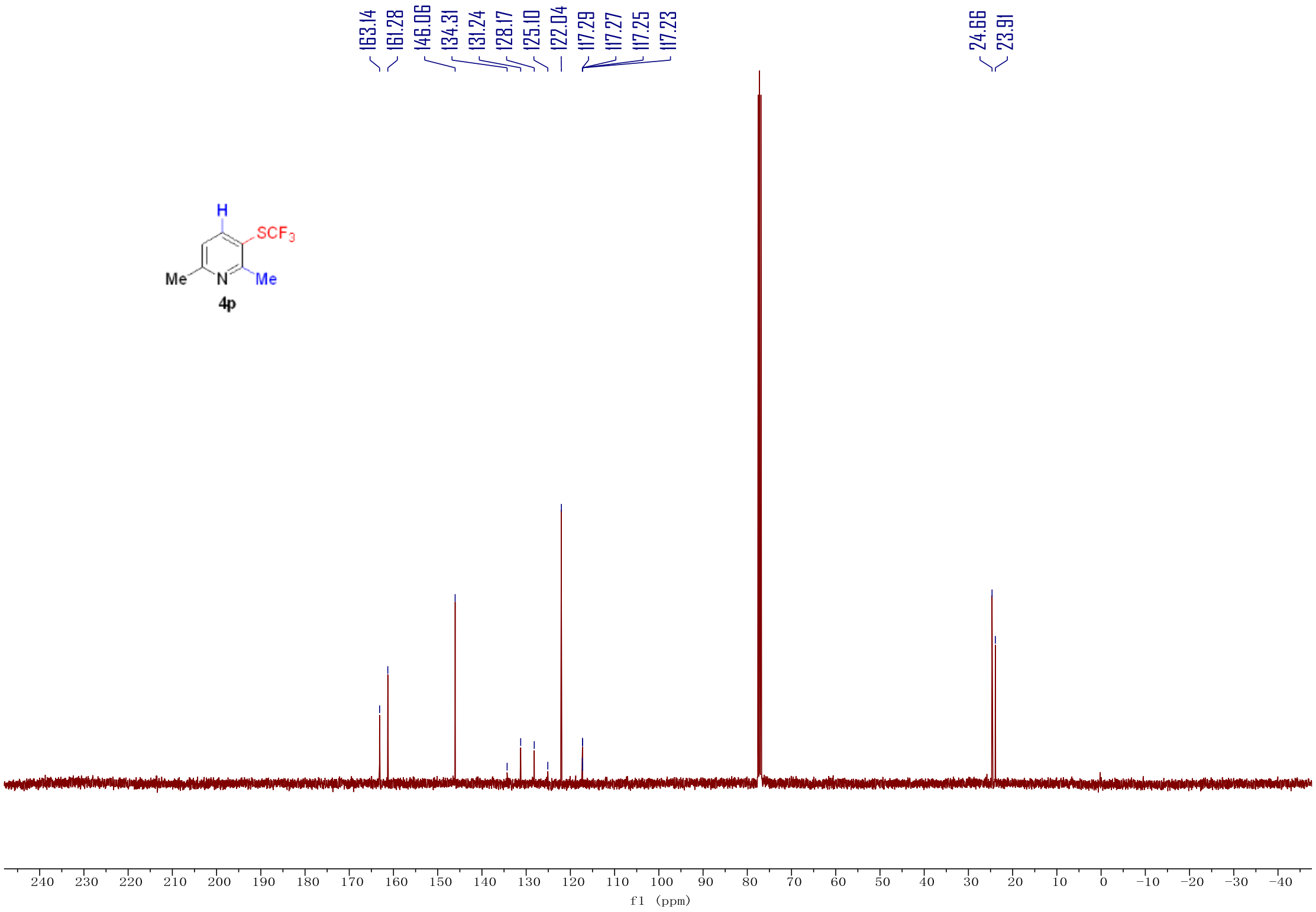

^{1}H NMR Spectrum of **4q** (400 MHz, $CDCl_3$)

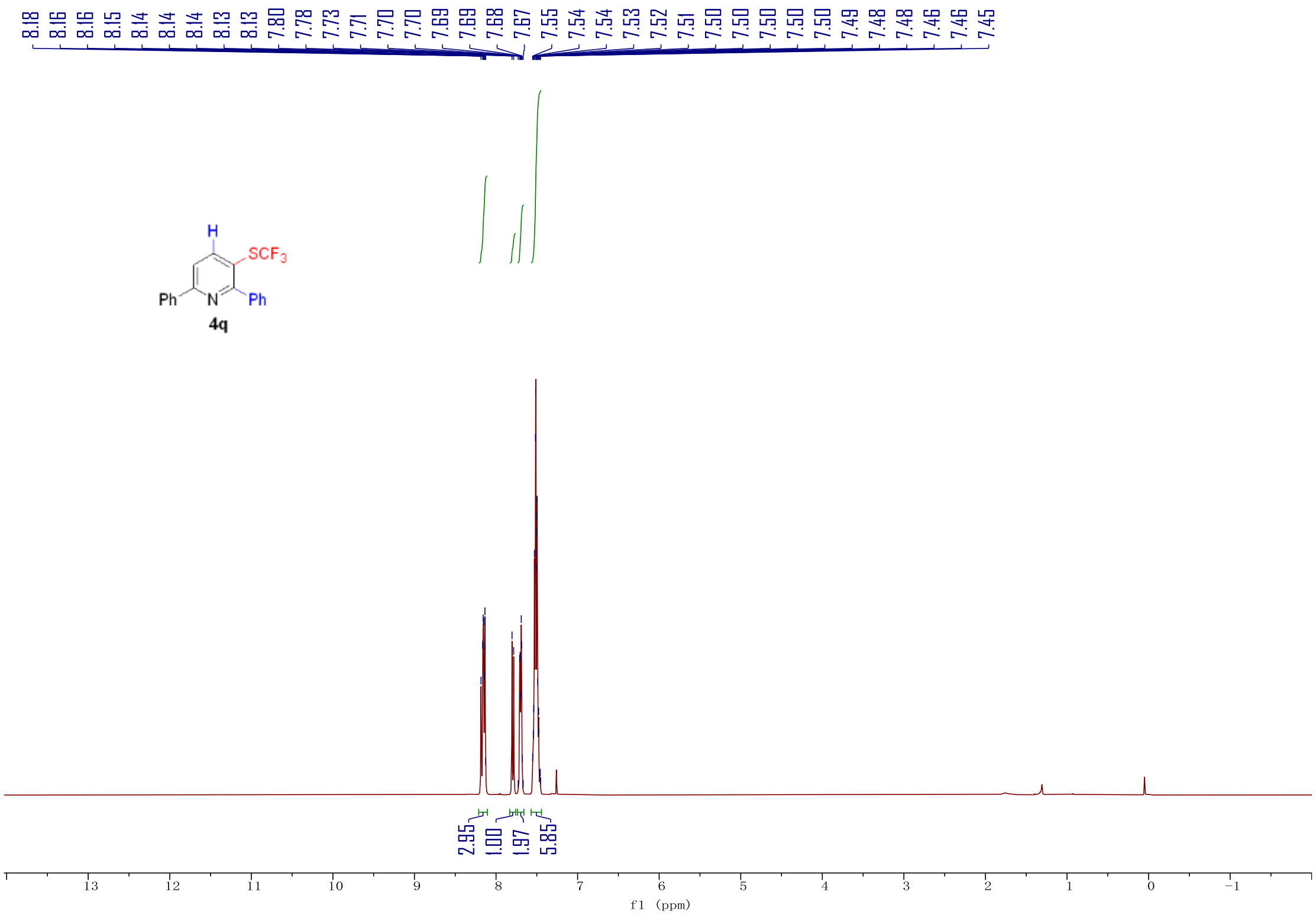

^{19}**F NMR** Spectrum of **4q** (376 MHz, $CDCl_3$)

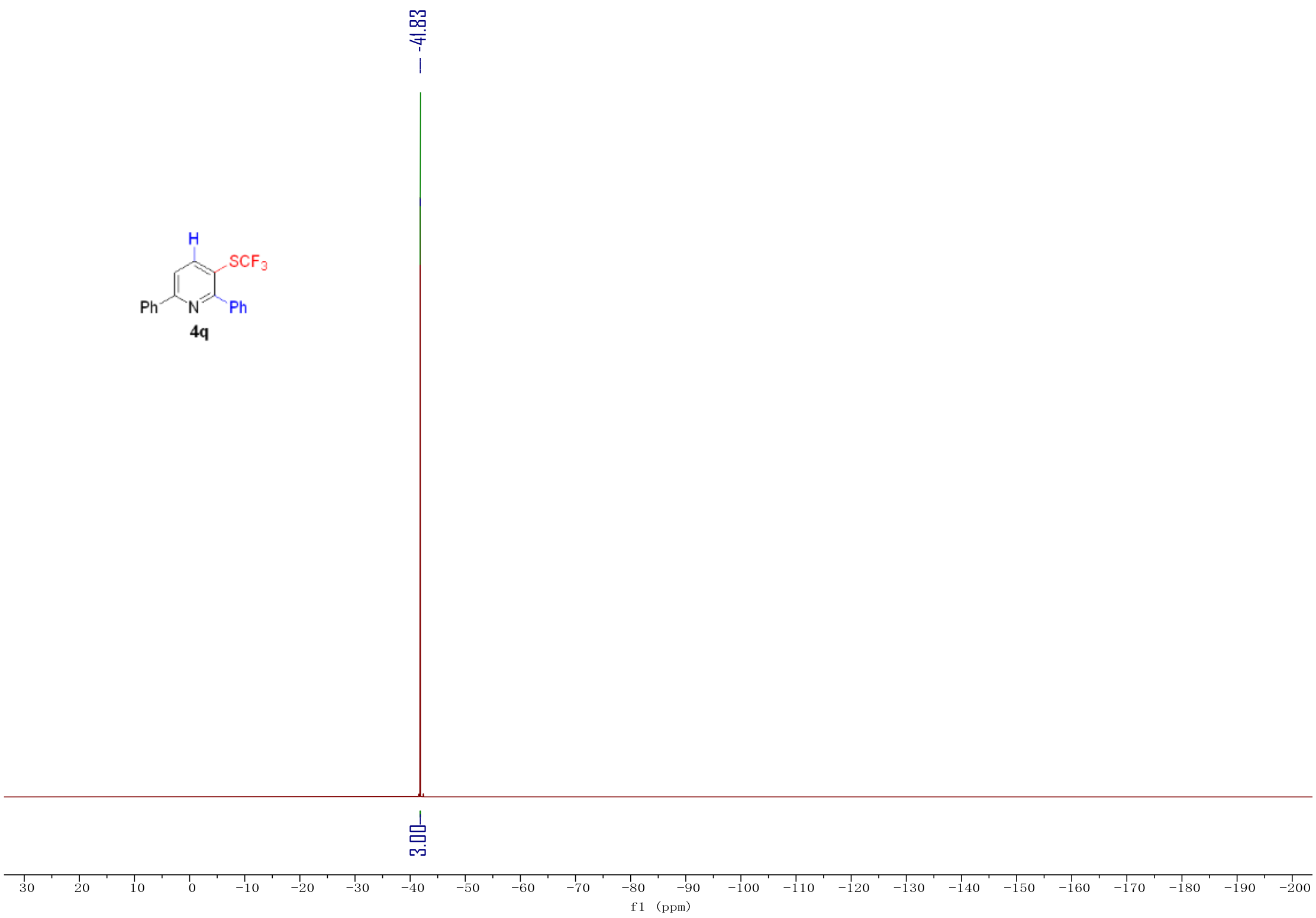

^{13}**C NMR** Spectrum of **4q** (101 MHz, $CDCl_3$)

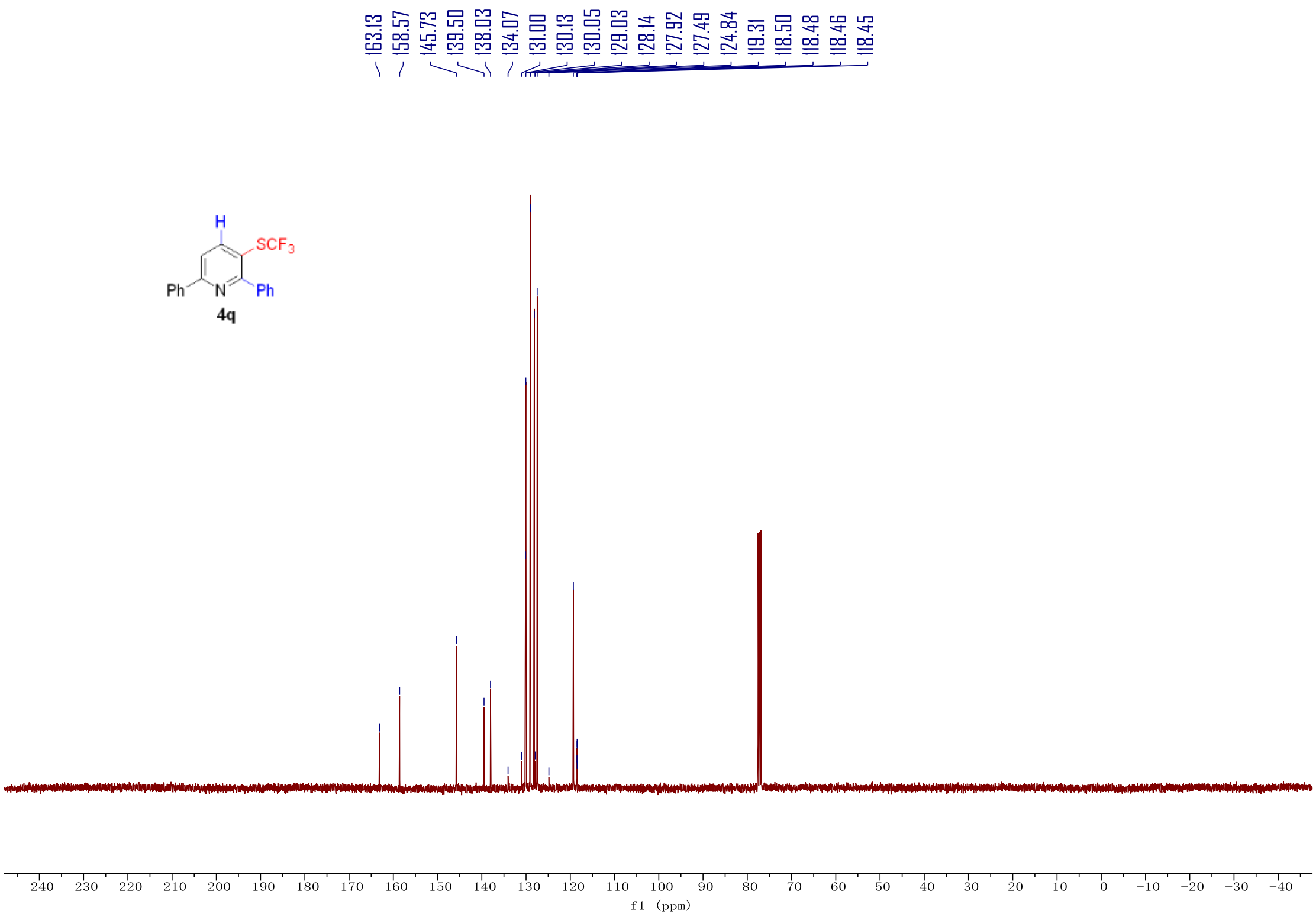

^{1}**H NMR** Spectrum of **4r** (400 MHz, $CDCl_3$)

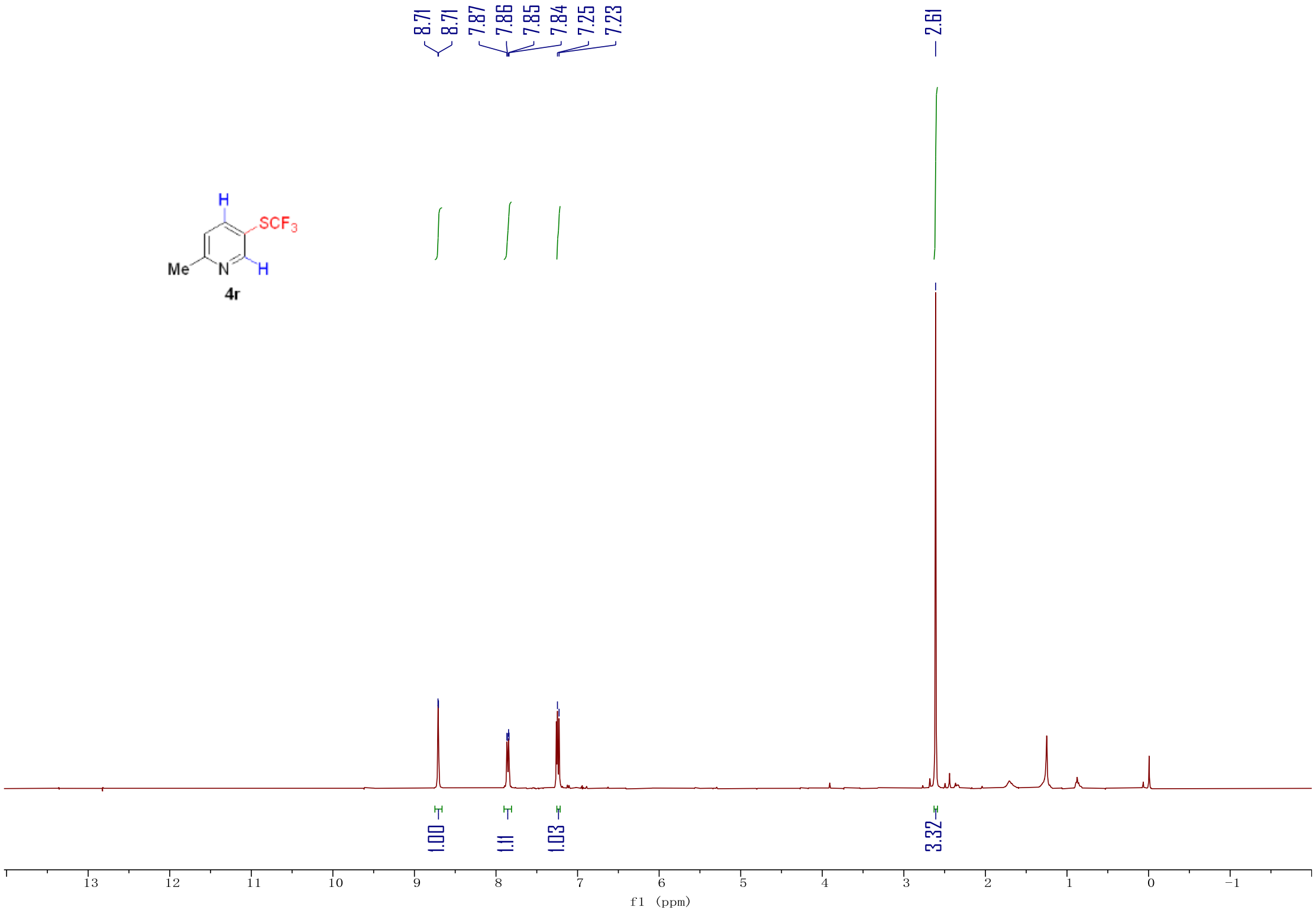

^{19}**F NMR** Spectrum of **4r** (376 MHz, $CDCl_3$)

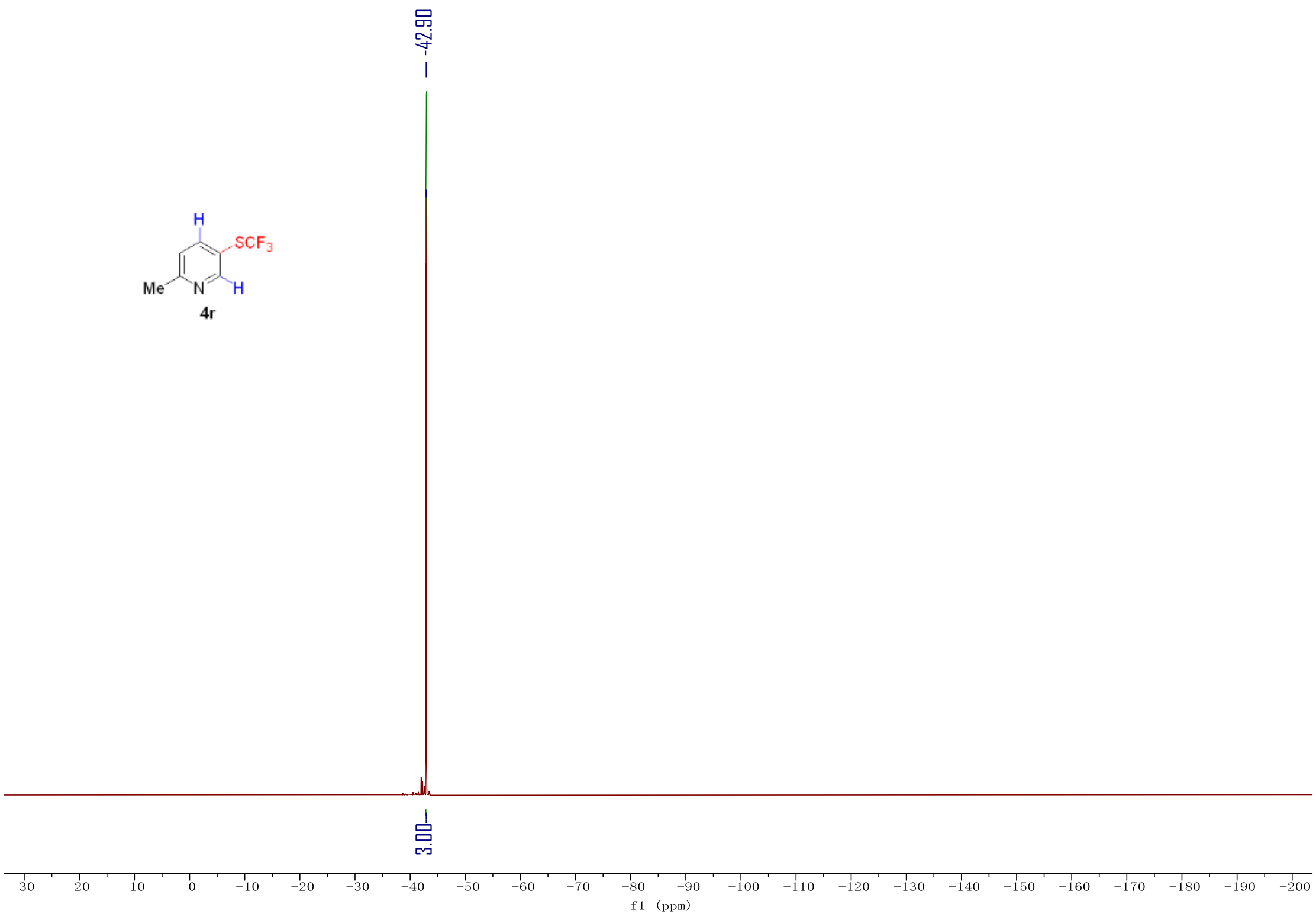

^{13}C NMR Spectrum of **4r** (101 MHz, $CDCl_3$)

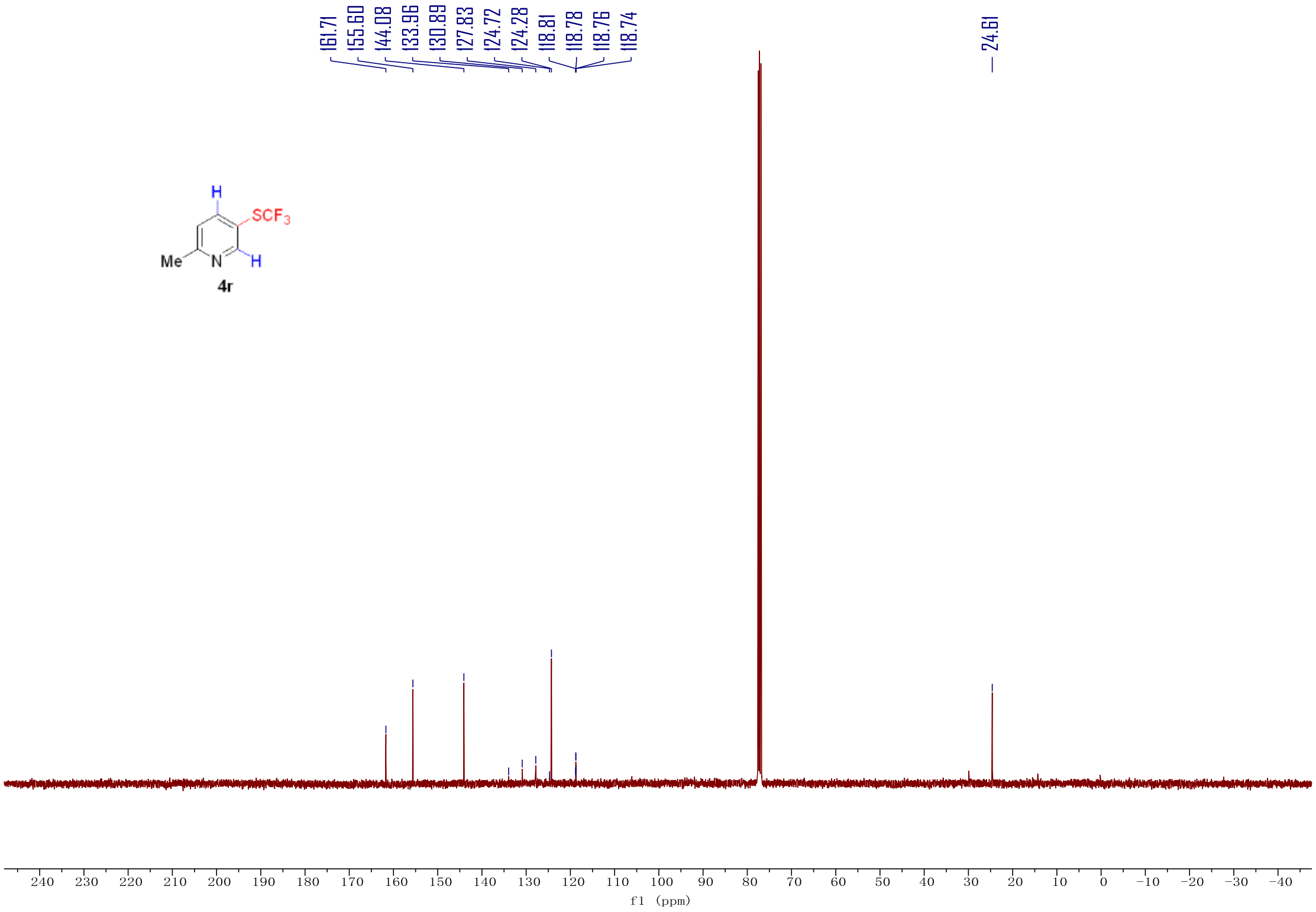

^{1}H NMR Spectrum of **4s** (400 MHz, $CDCl_3$)

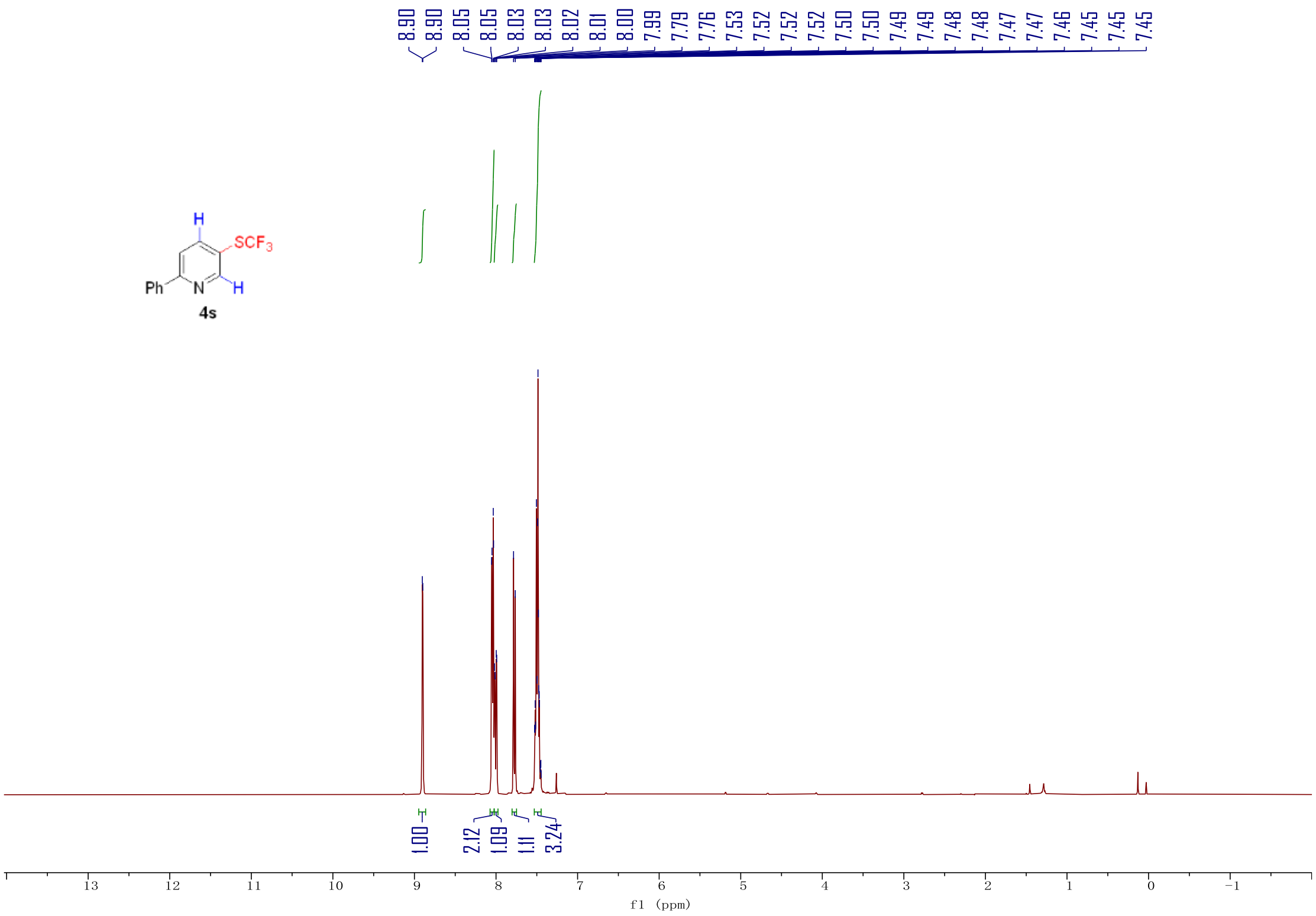

^{19}F NMR Spectrum of **4s** (376 MHz, $CDCl_3$)

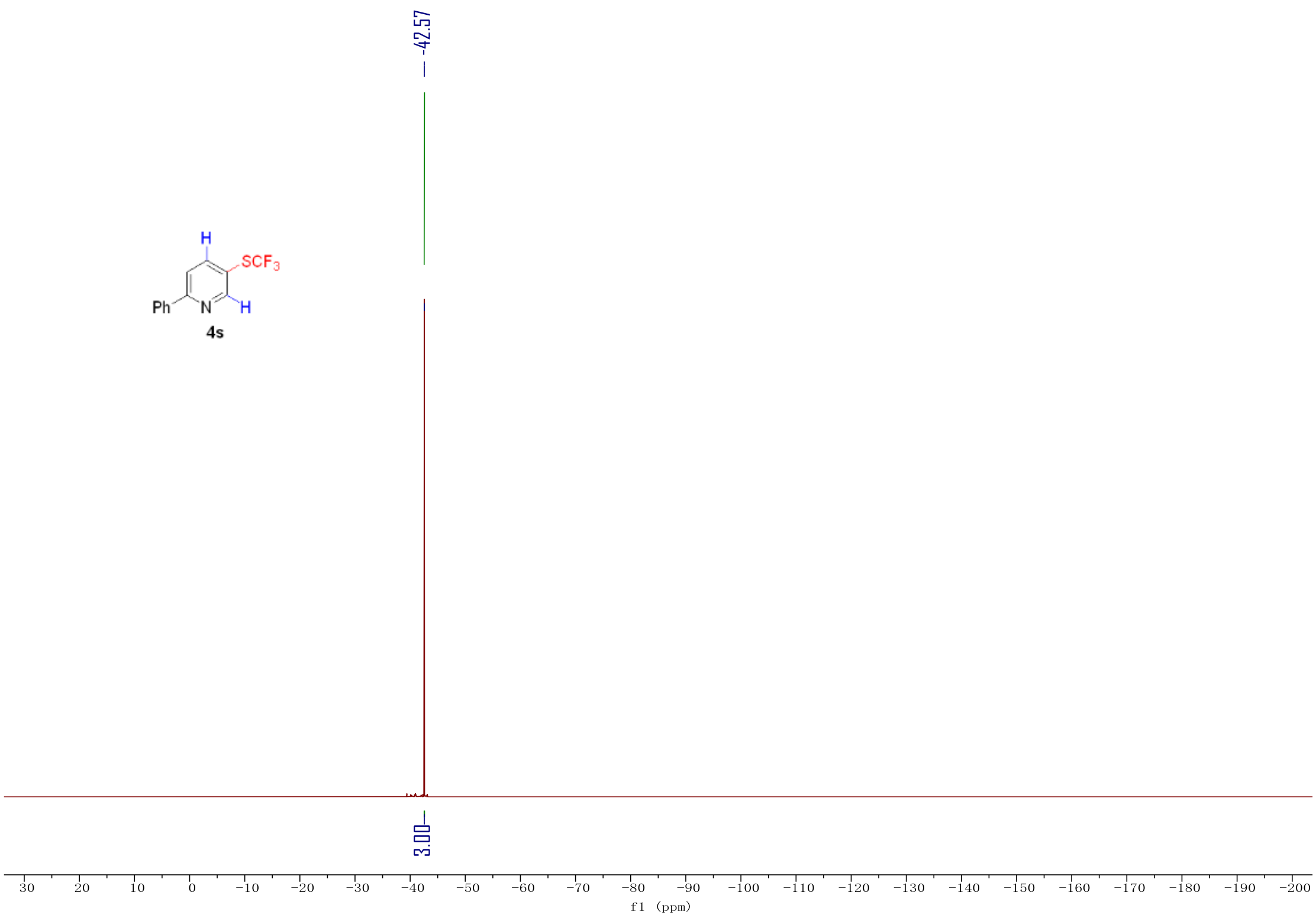

^{13}C NMR Spectrum of **4s** (101 MHz, $CDCl_3$)

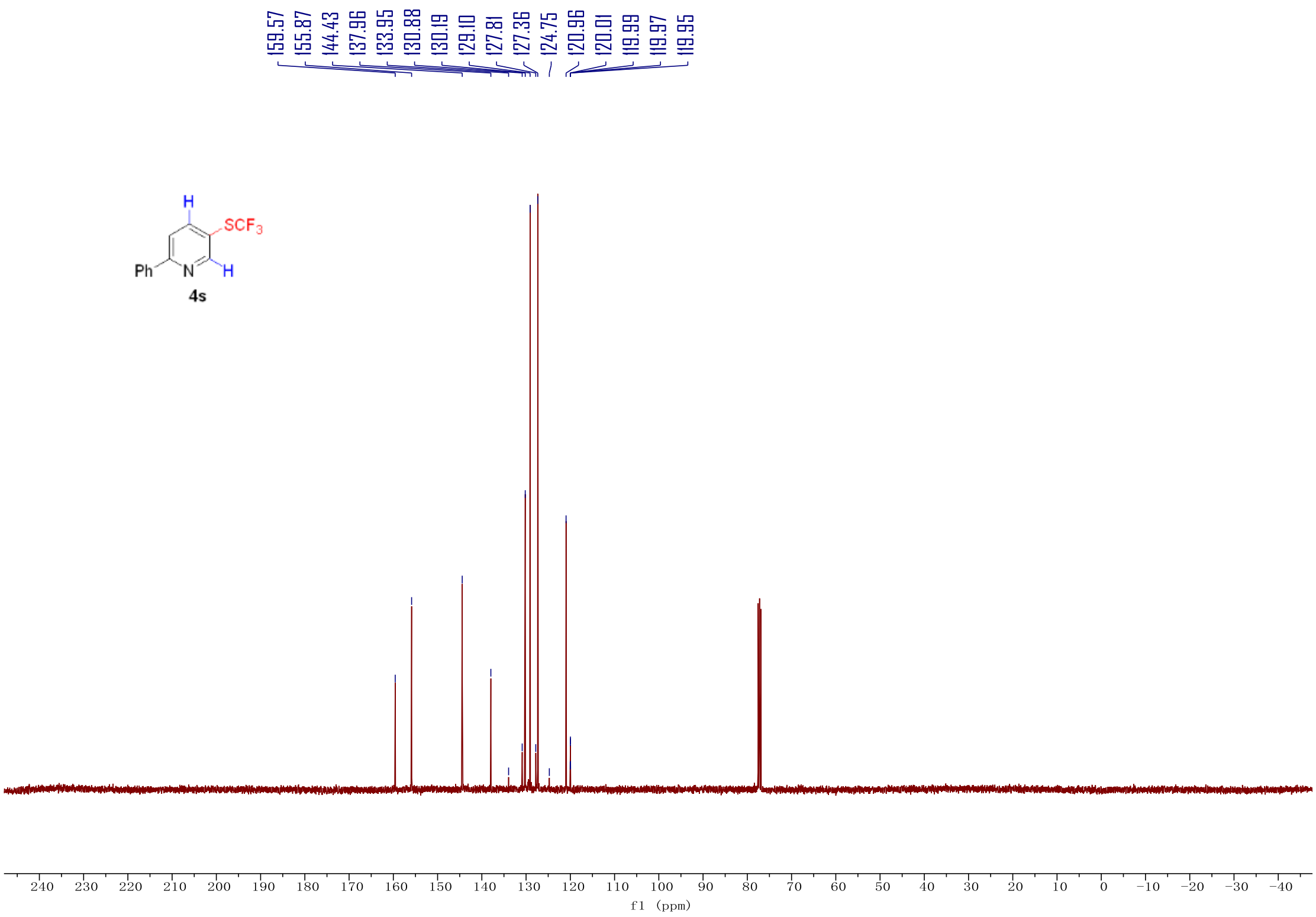

¹H NMR Spectrum of **4t** (400 MHz, $CDCl_3$)

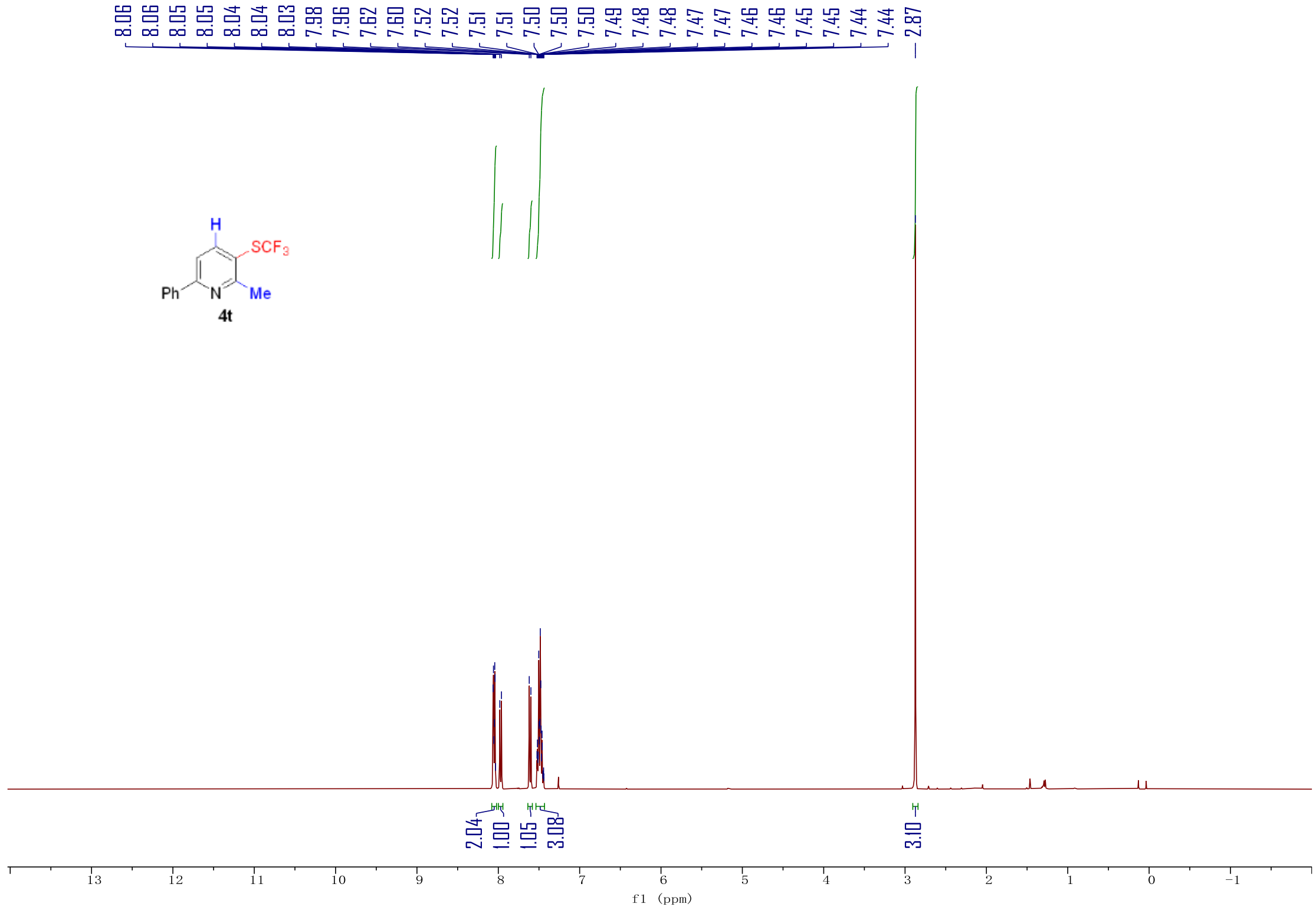

¹⁹F NMR Spectrum of **4t** (376 MHz, $CDCl_3$)

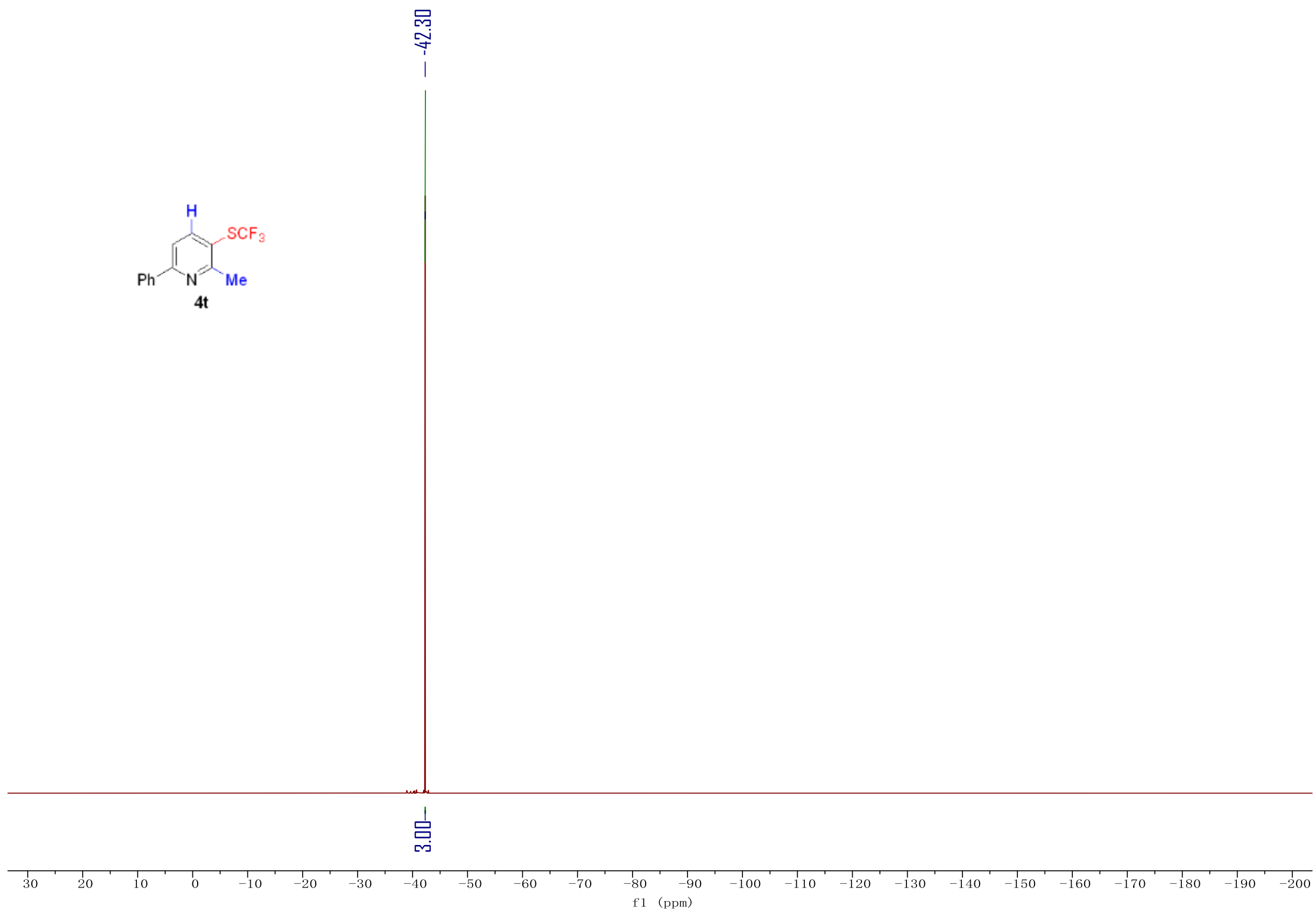

^{13}C NMR Spectrum of **4t** (101 MHz, $CDCl_3$)

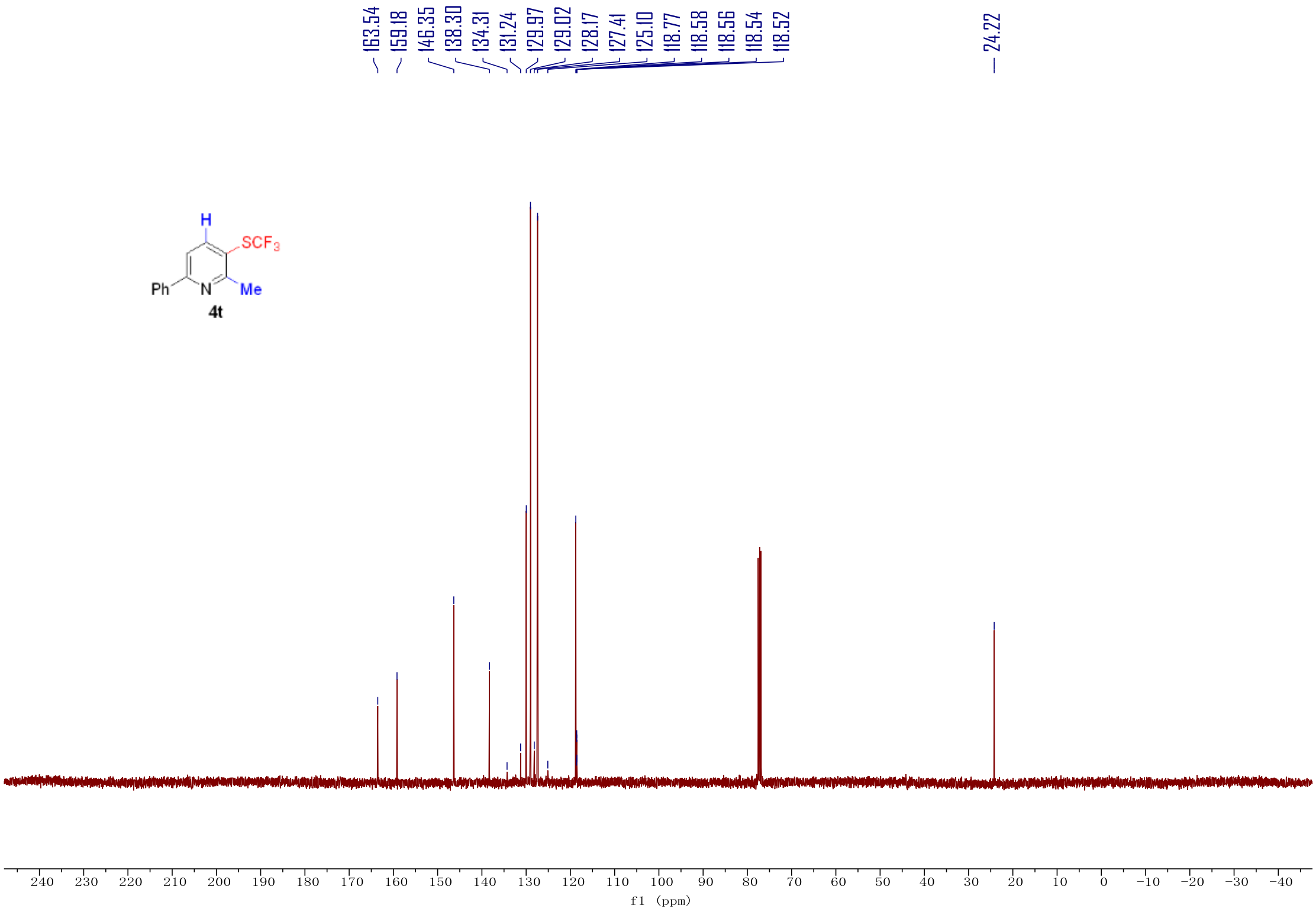

^{1}H NMR Spectrum of **4u** (400 MHz, $CDCl_3$)

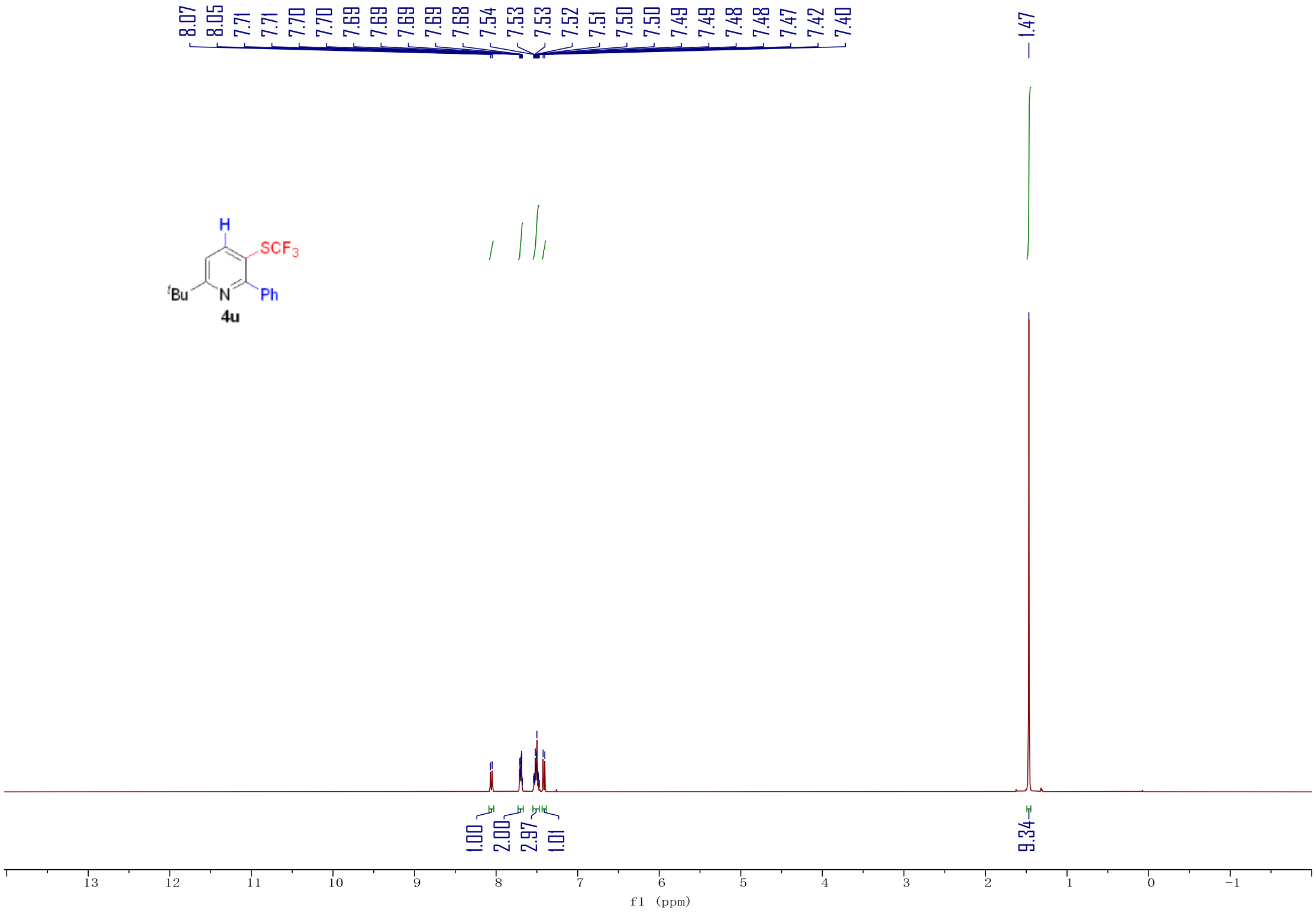

^{19}F NMR Spectrum of **4u** (376 MHz, $CDCl_3$)

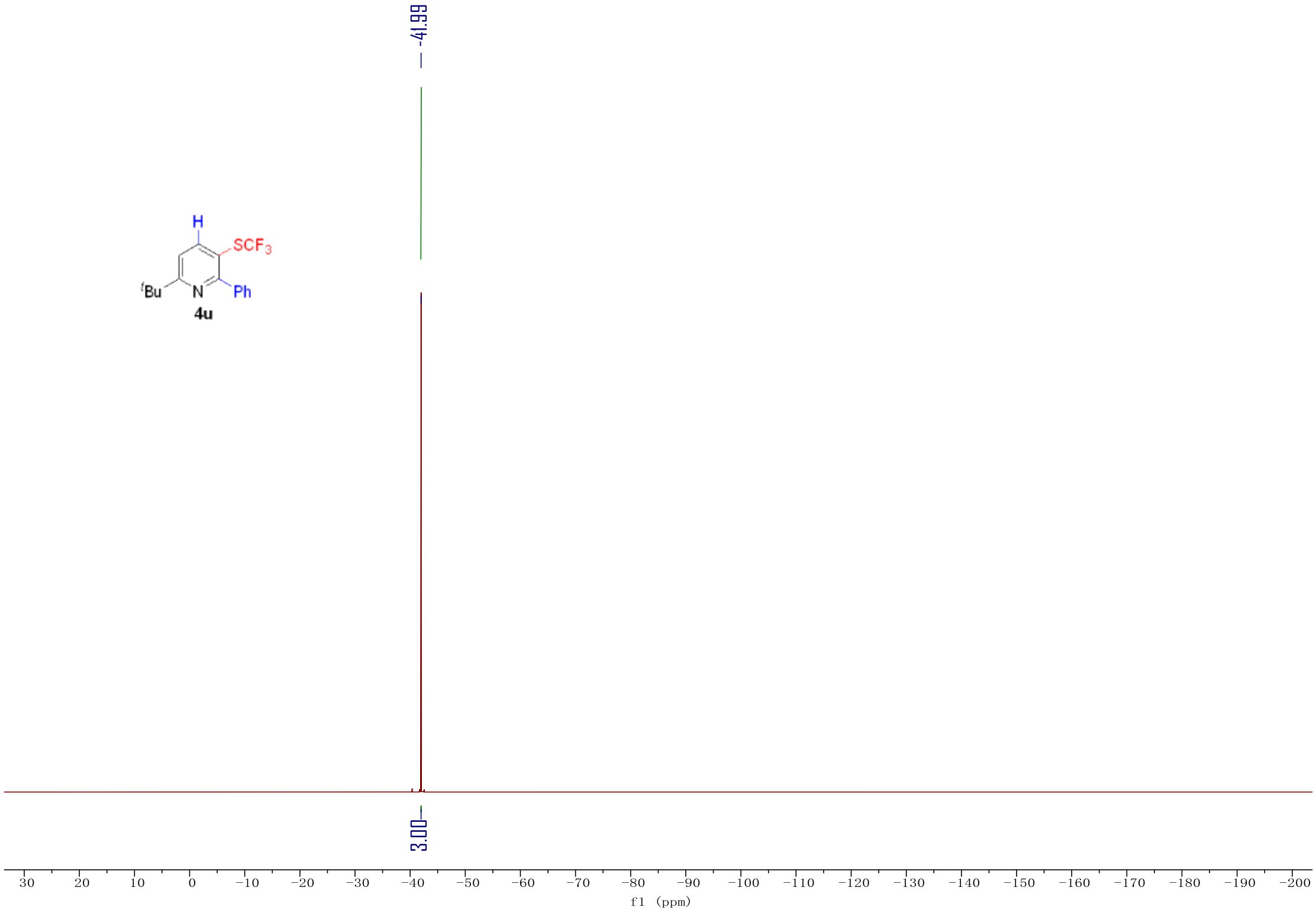

^{13}C NMR Spectrum of **4u** (101 MHz, $CDCl_3$)

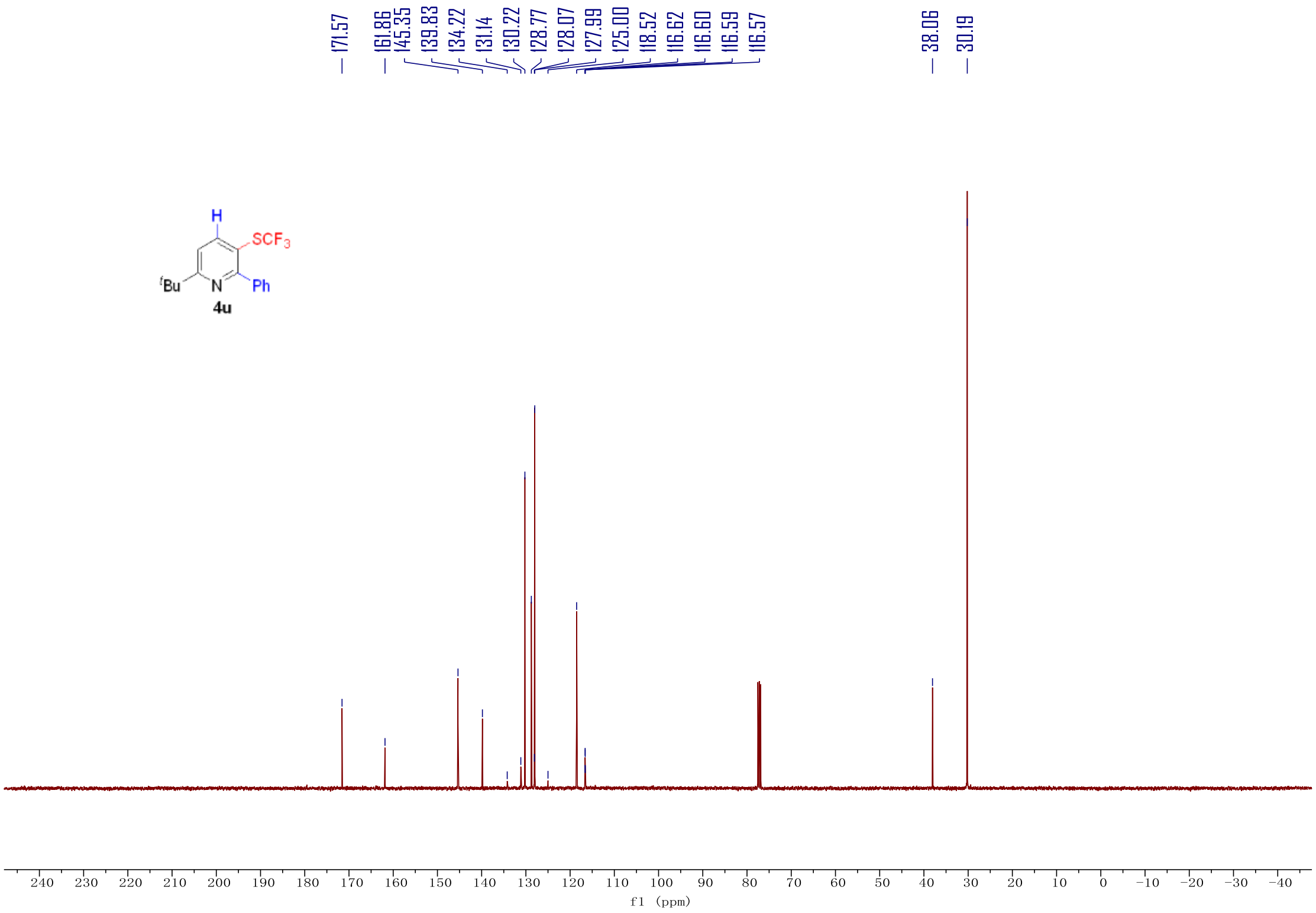

^{1}**H NMR** Spectrum of **6a** (400 MHz, $CDCl_3$)

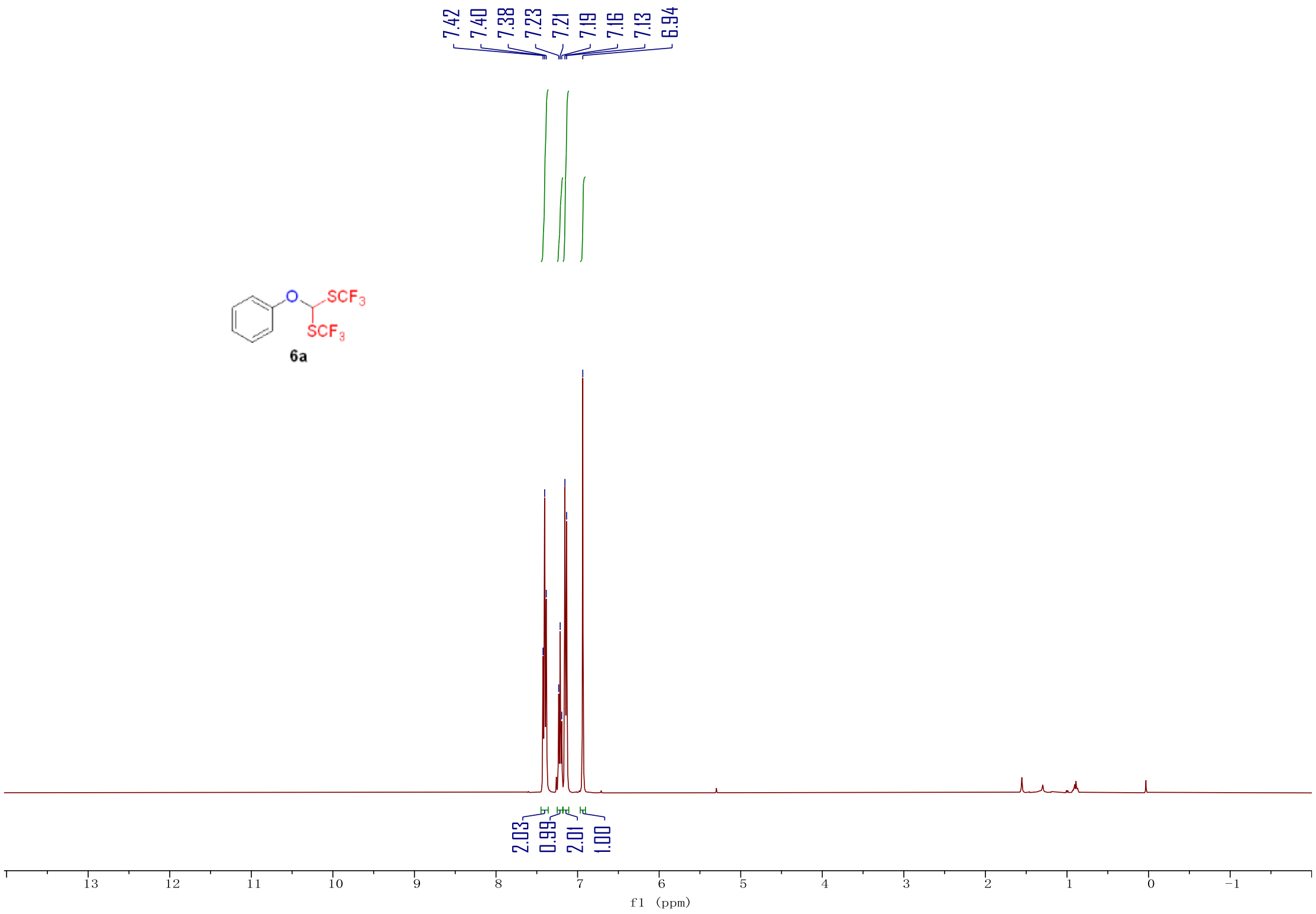

^{19}**F NMR** Spectrum of **6a** (376 MHz, $CDCl_3$)

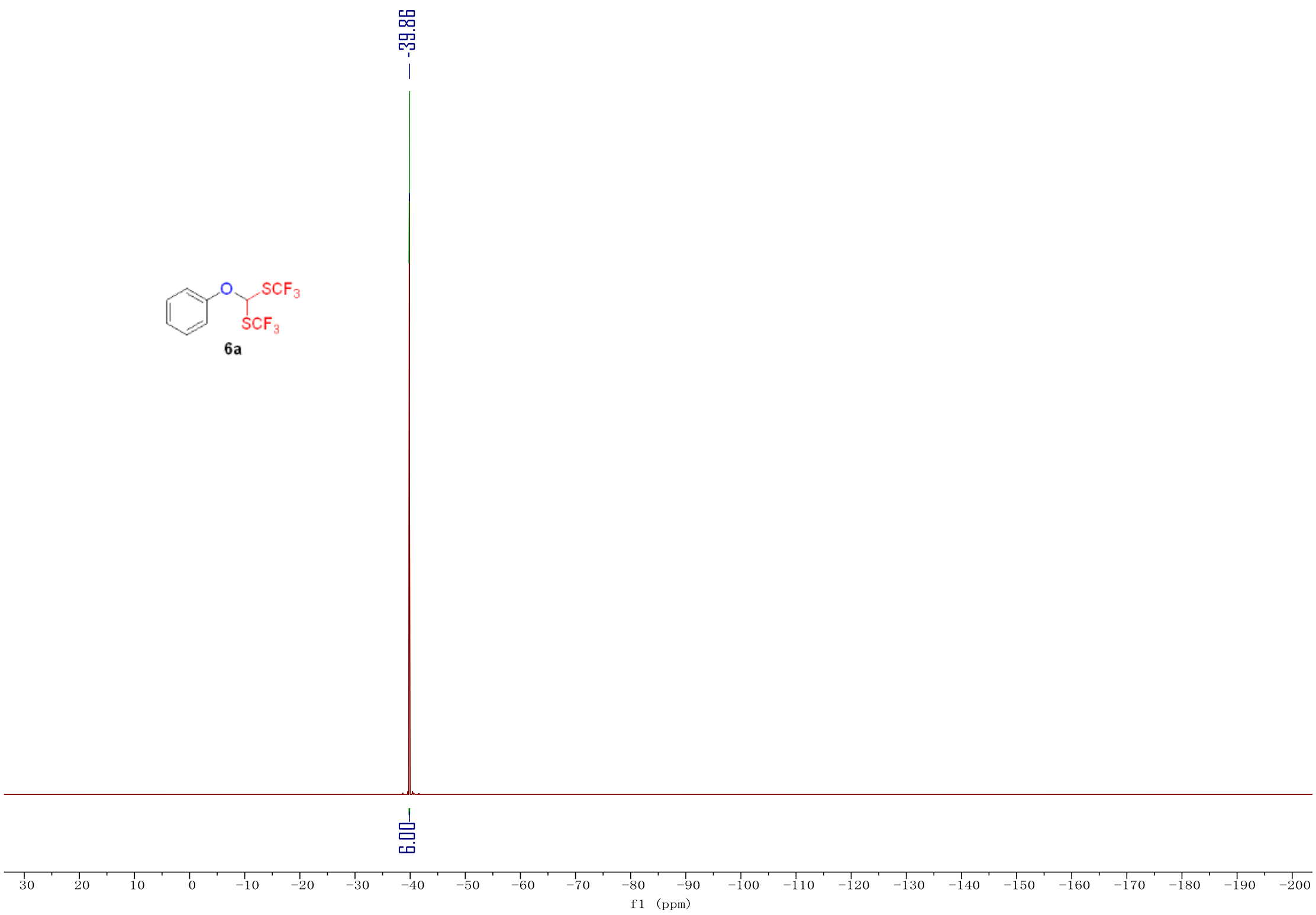

^{13}C NMR Spectrum of **6a** (101 MHz, $CDCl_3$)

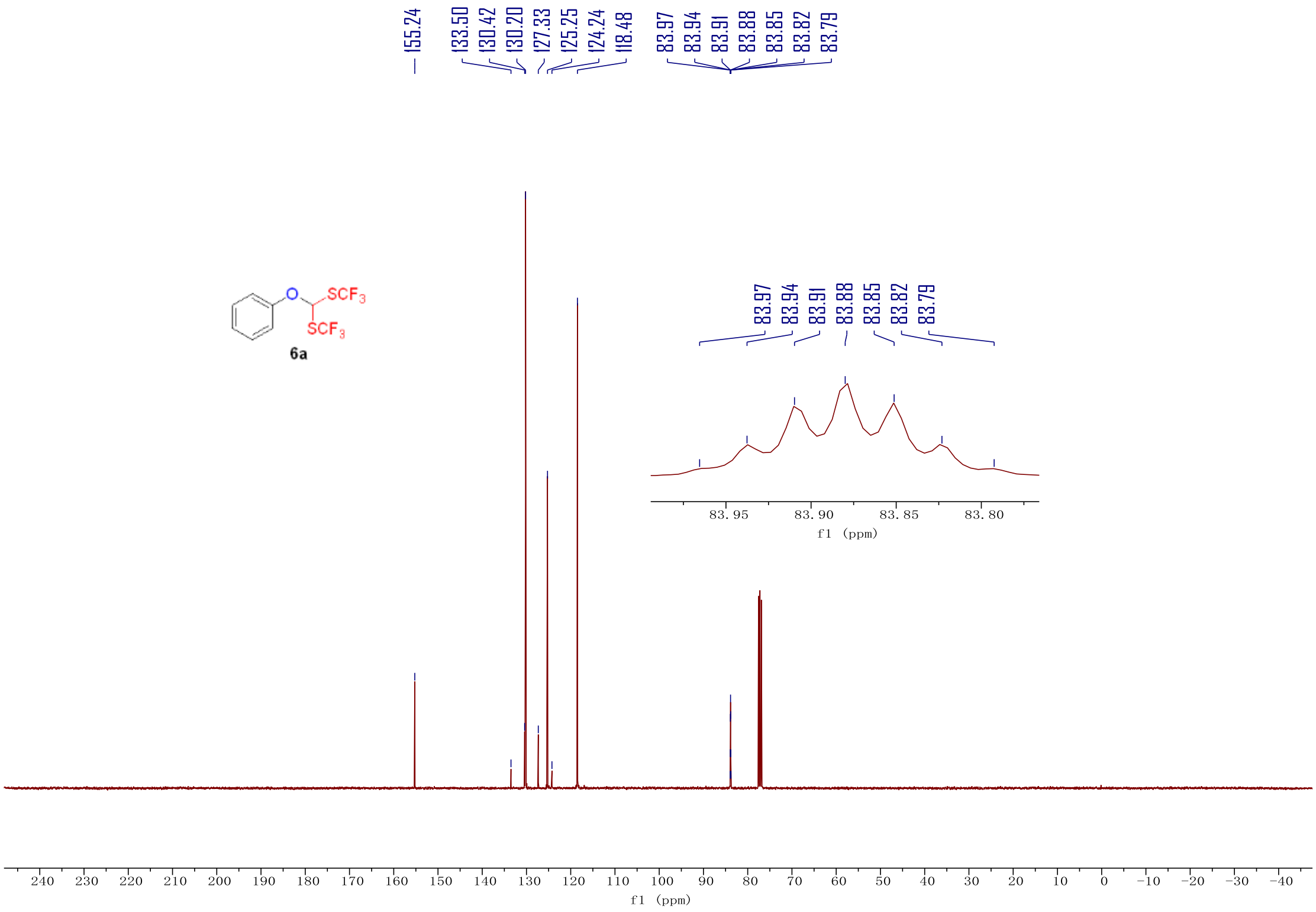

^{1}H NMR Spectrum of **6b** (400 MHz, $CDCl_3$)

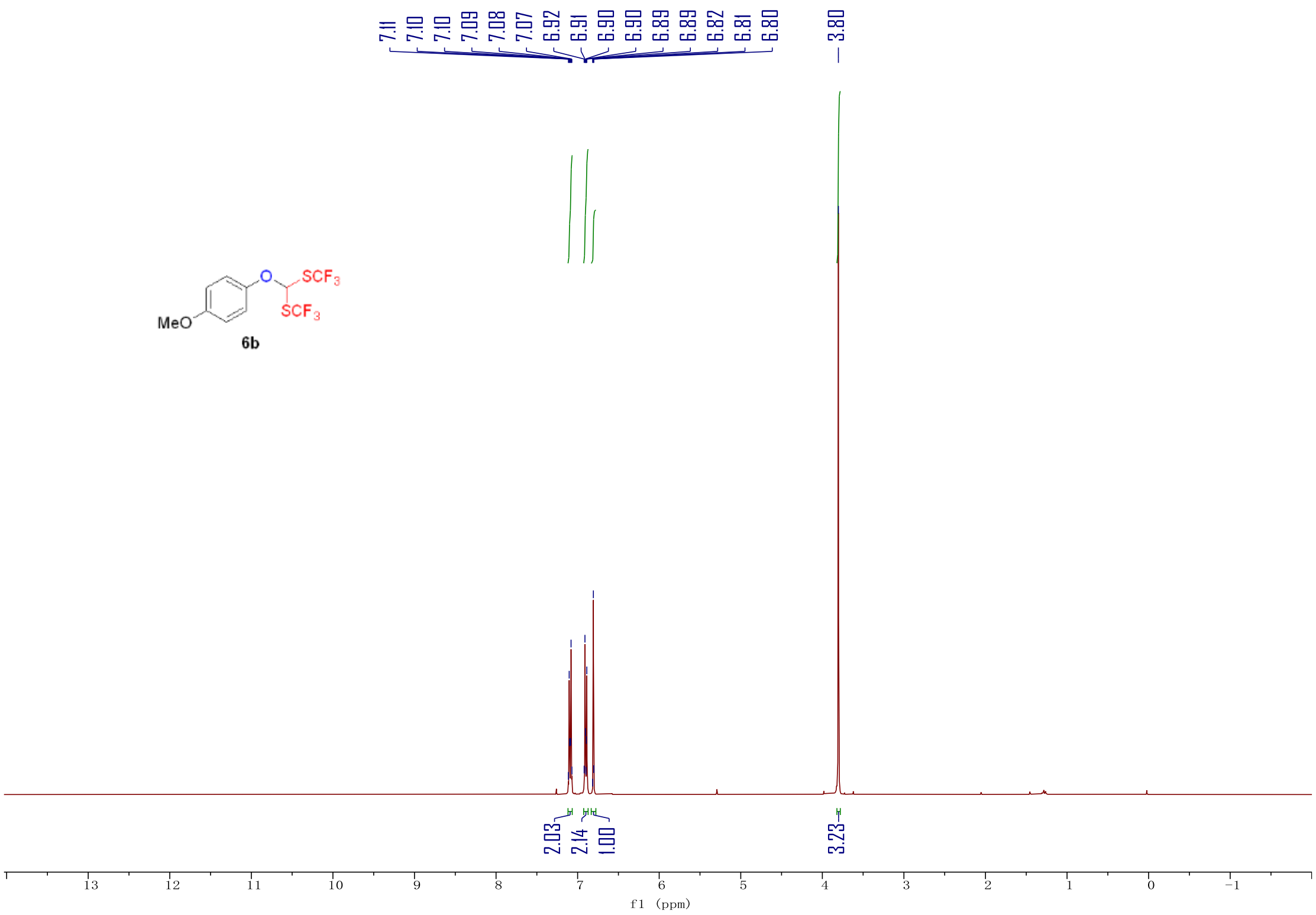

^{19}F **NMR** Spectrum of **6b** (376 MHz, $CDCl_3$)

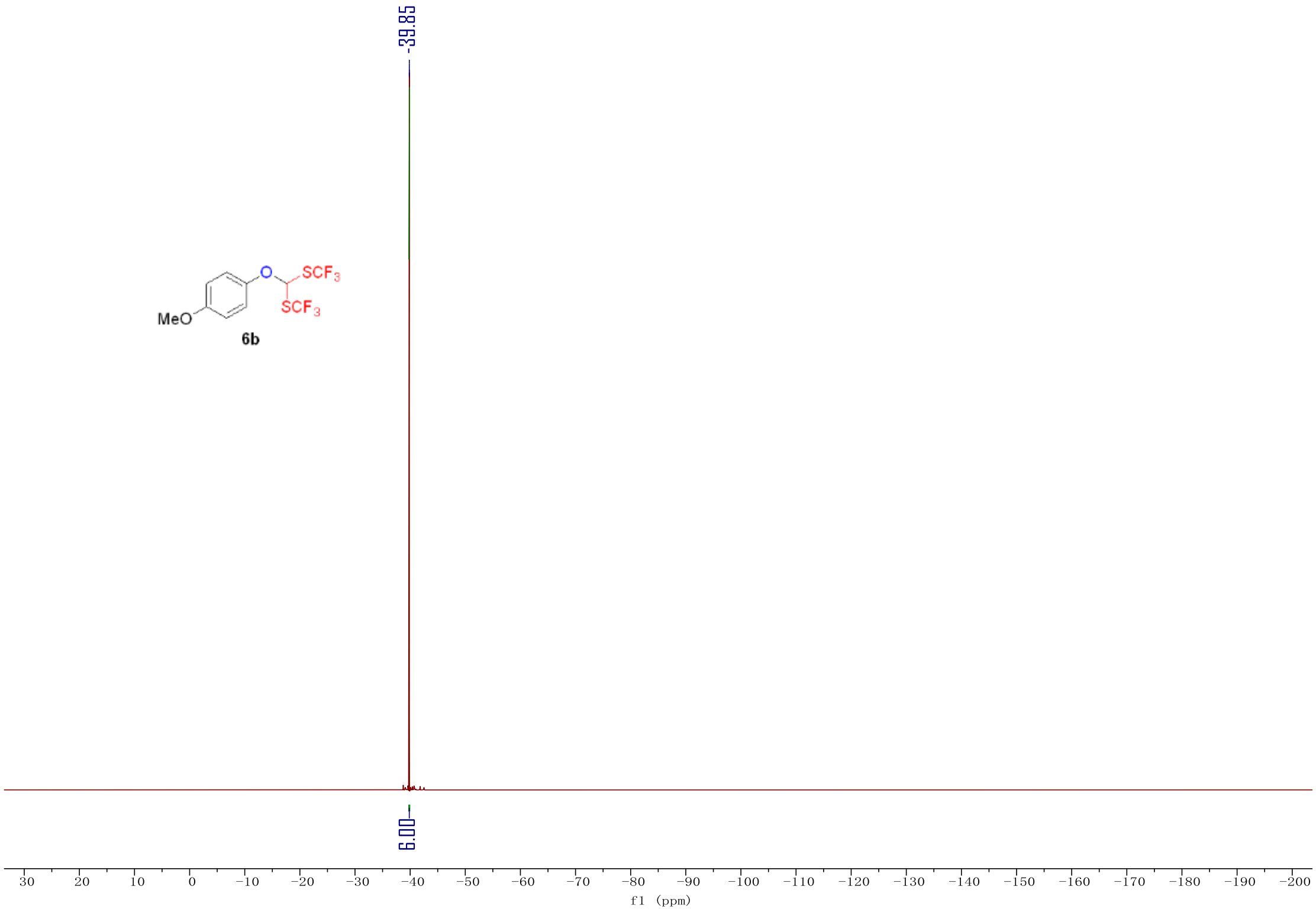

^{13}C **NMR** Spectrum of **6b** (101 MHz, $CDCl_3$)

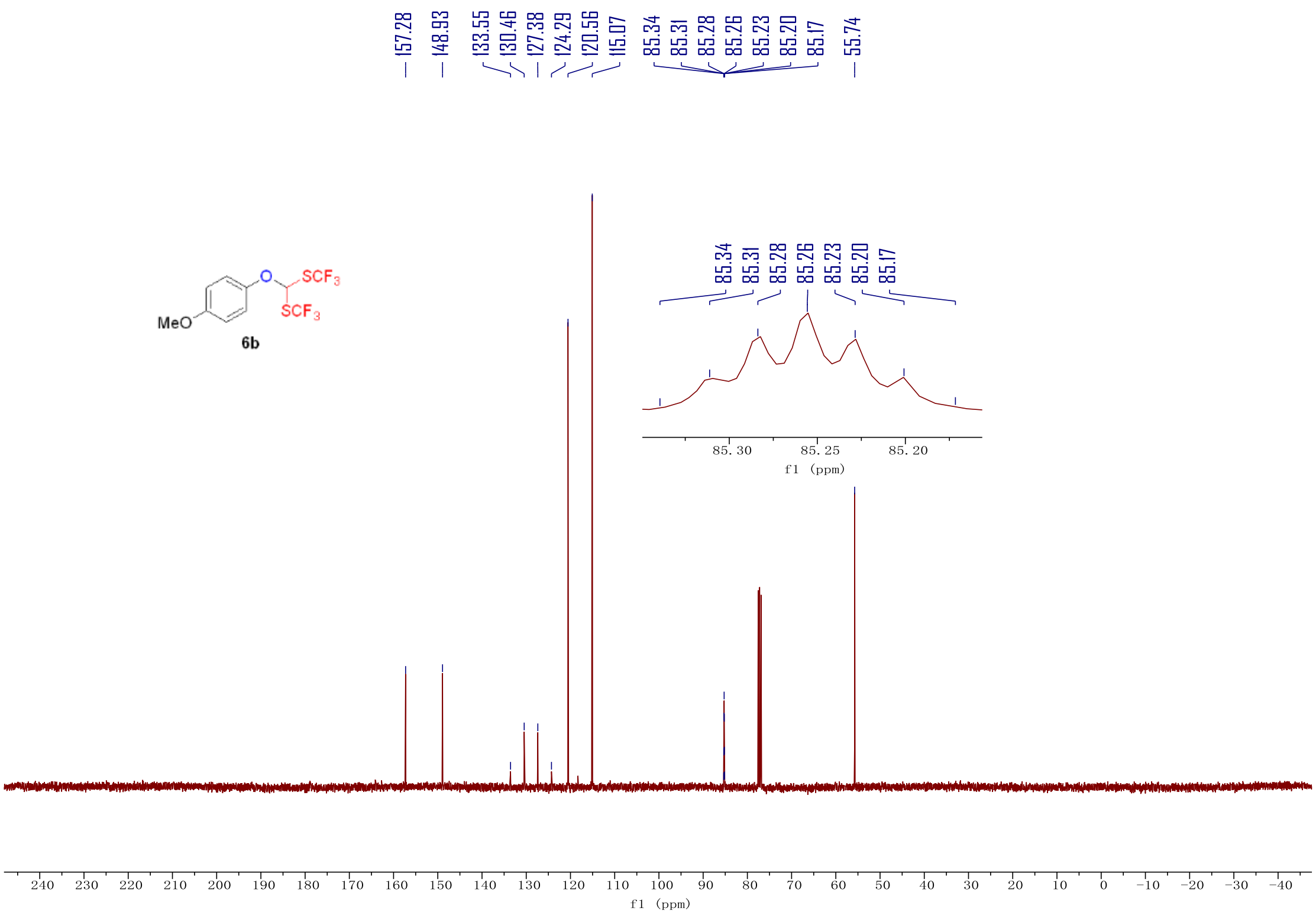

^{1}H NMR Spectrum of **6c** (400 MHz, $CDCl_3$)

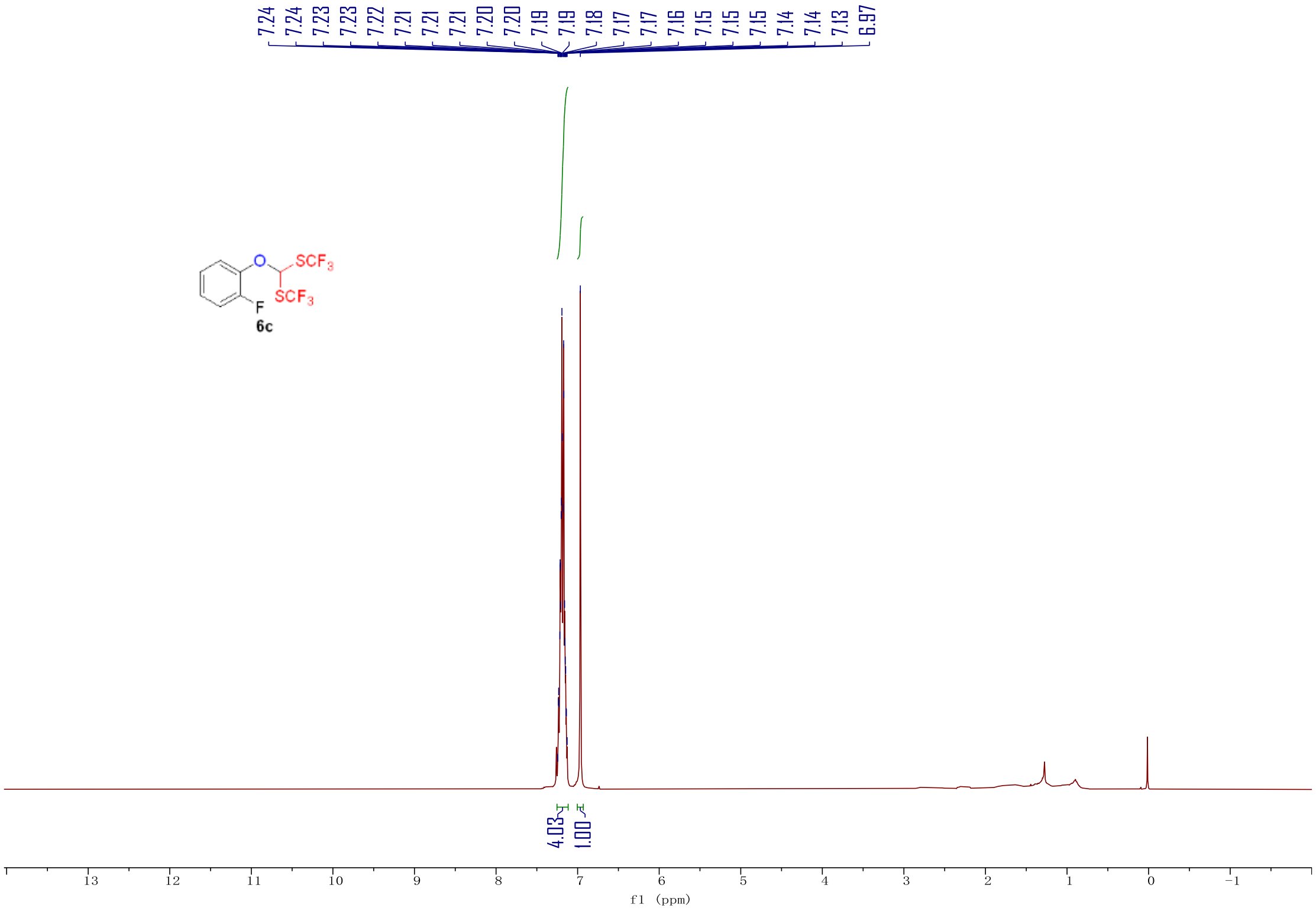

^{19}F NMR Spectrum of **6c** (376 MHz, $CDCl_3$)

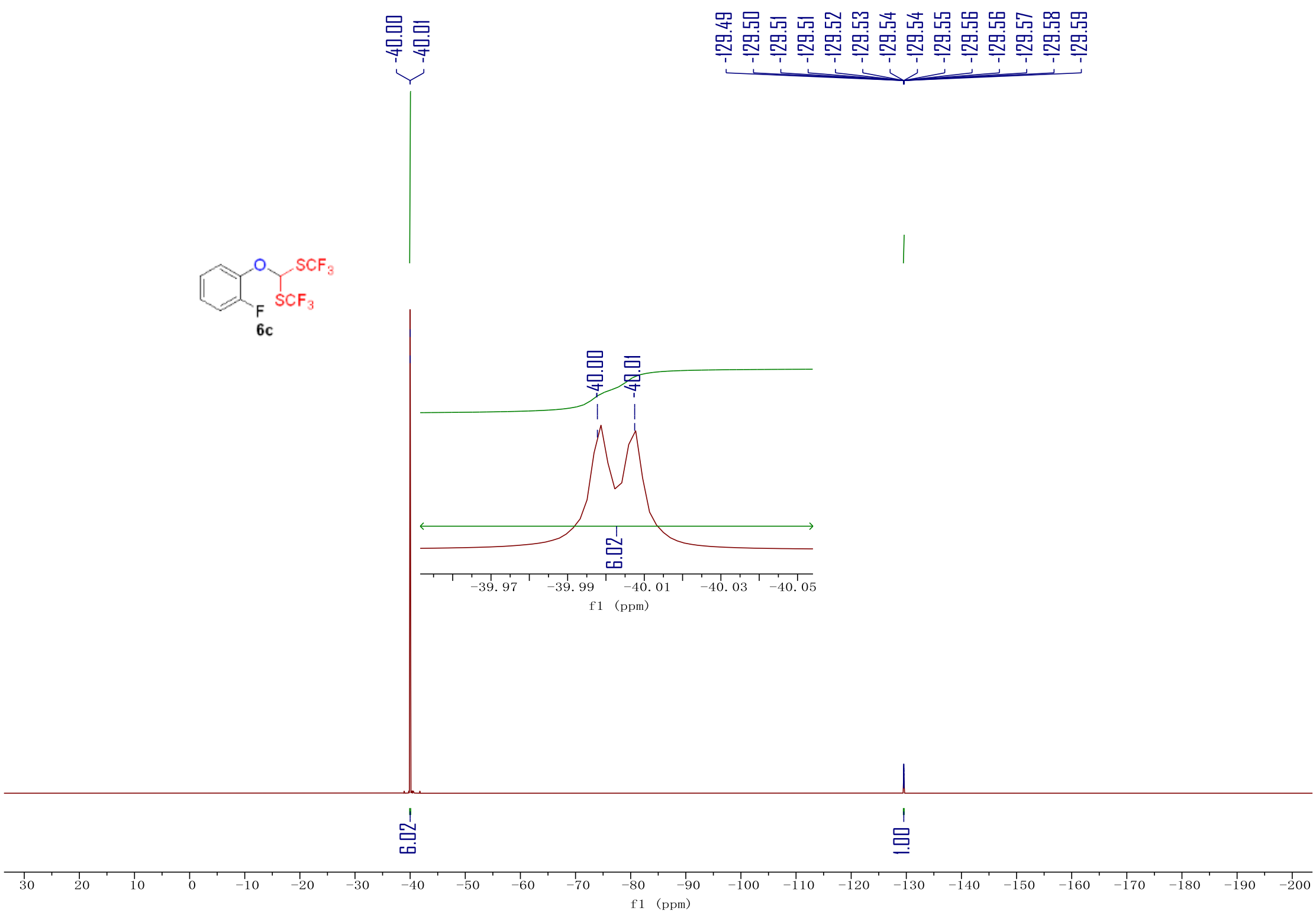

^{13}**C NMR** Spectrum of **6c** (101 MHz, $CDCl_3$)

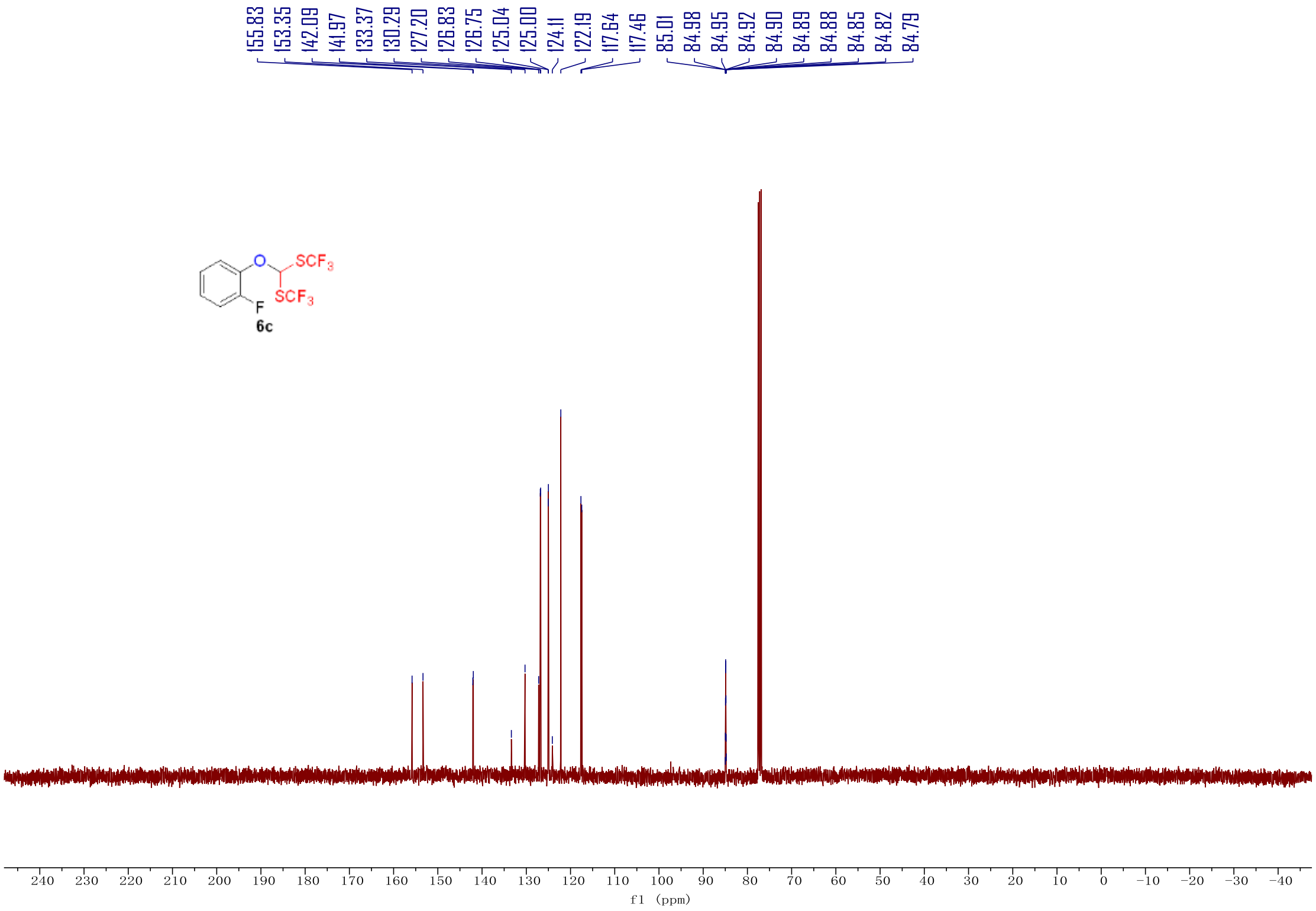

^{1}**H NMR** Spectrum of **6d** (400 MHz, $CDCl_3$)

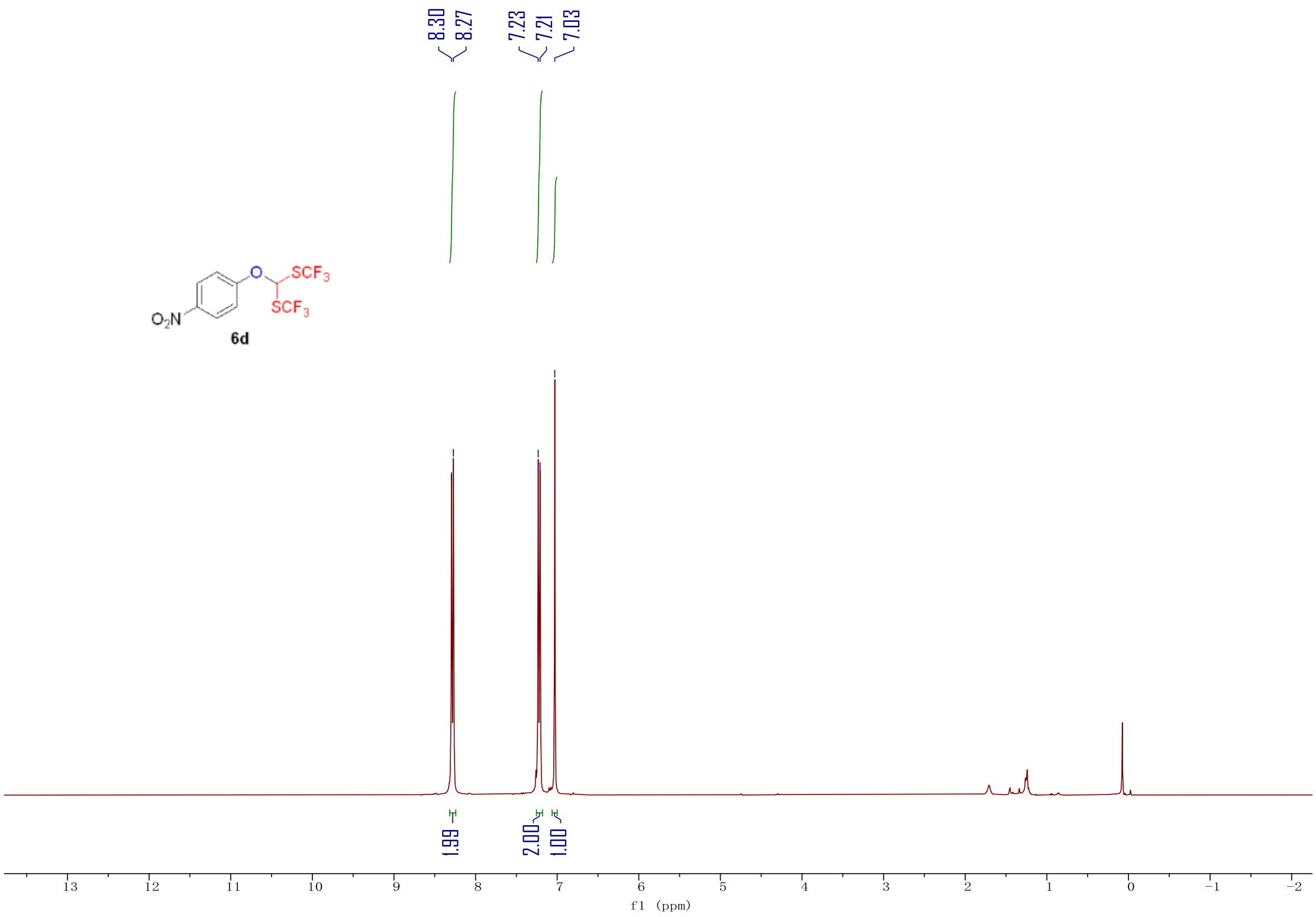

^{19}**F NMR** Spectrum of **6d** (376 MHz, $CDCl_3$)

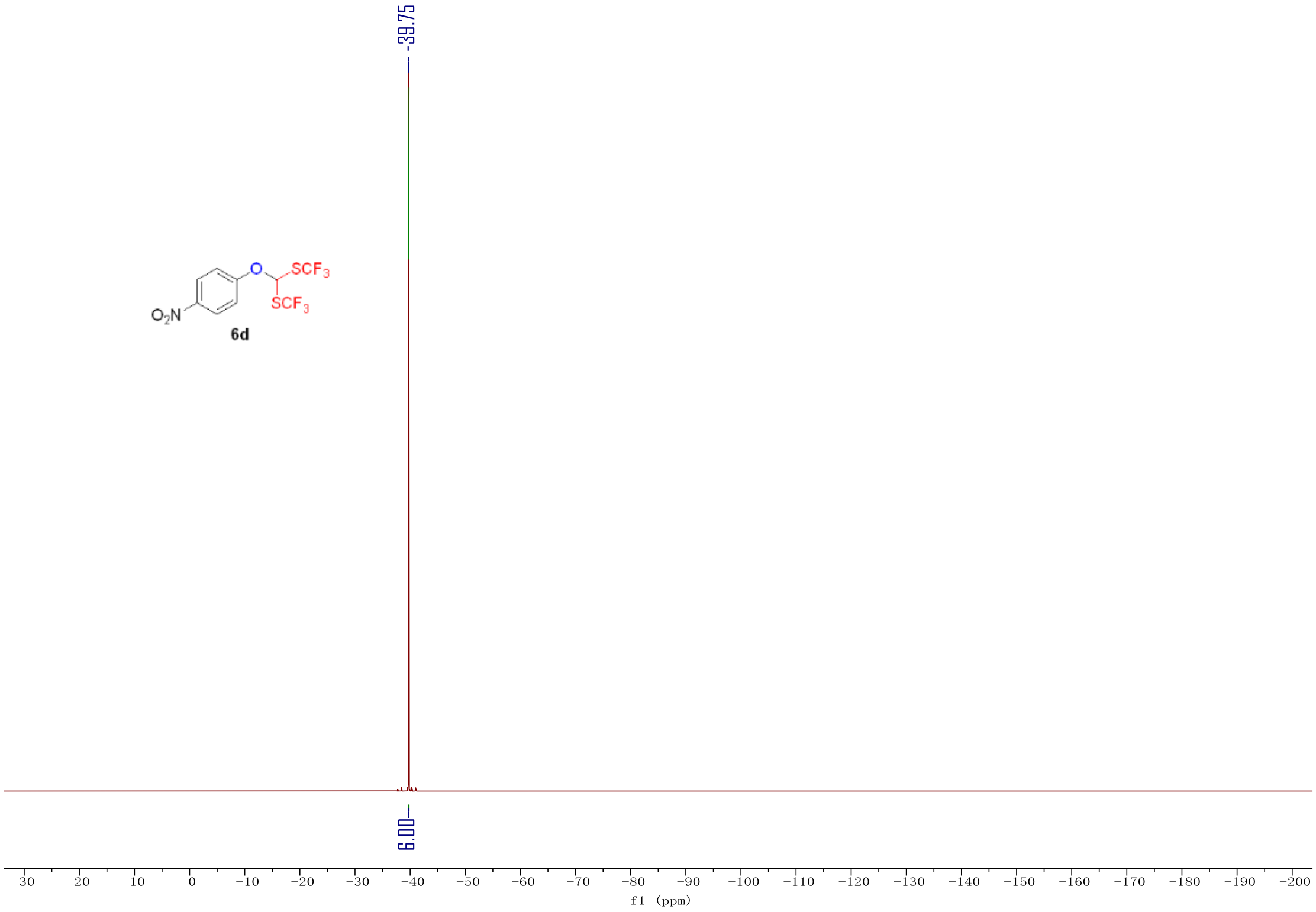

^{13}**C NMR** Spectrum of **6d** (101 MHz, $CDCl_3$)

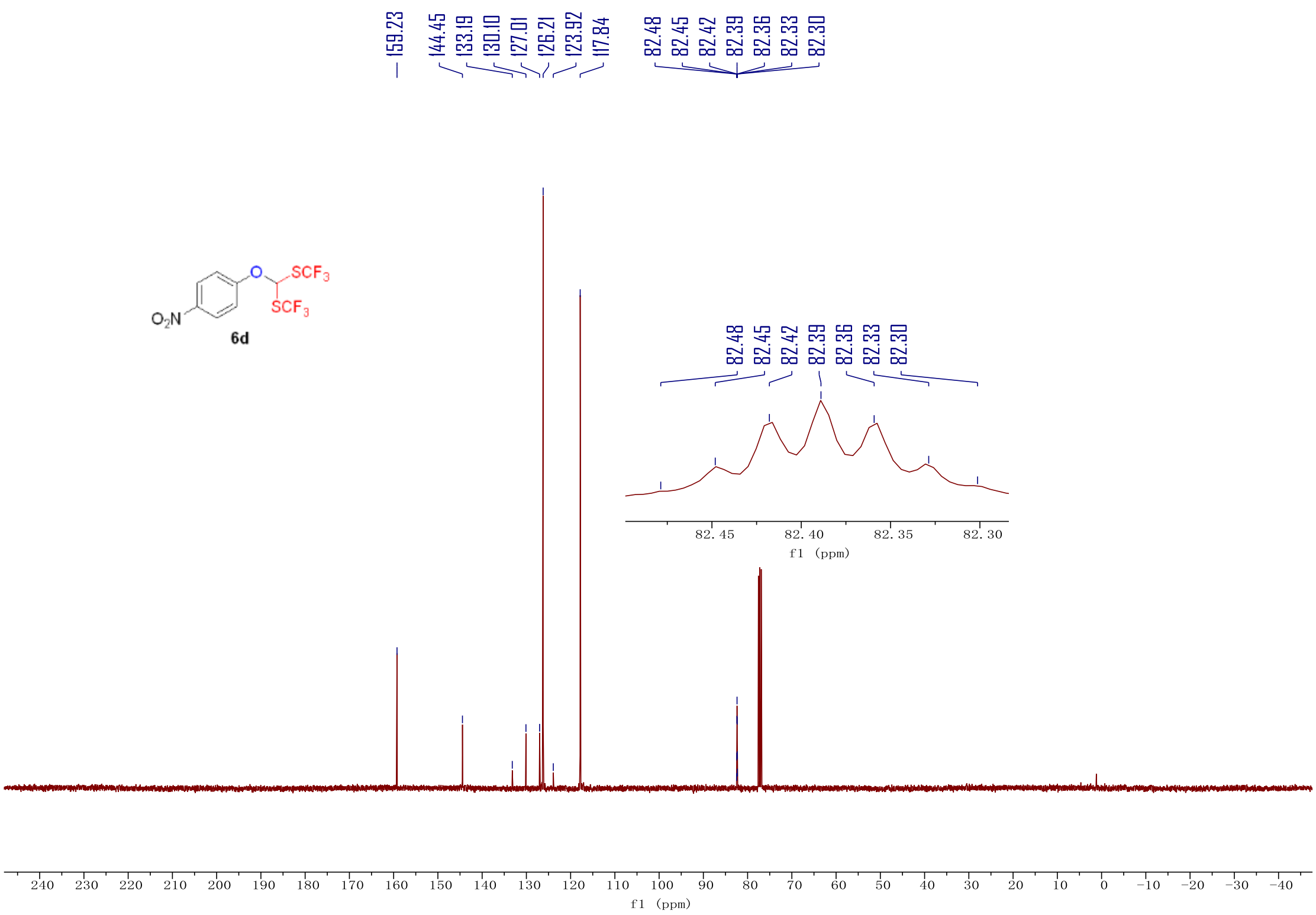

^{1}H NMR Spectrum of **6e** (400 MHz, $CDCl_3$)

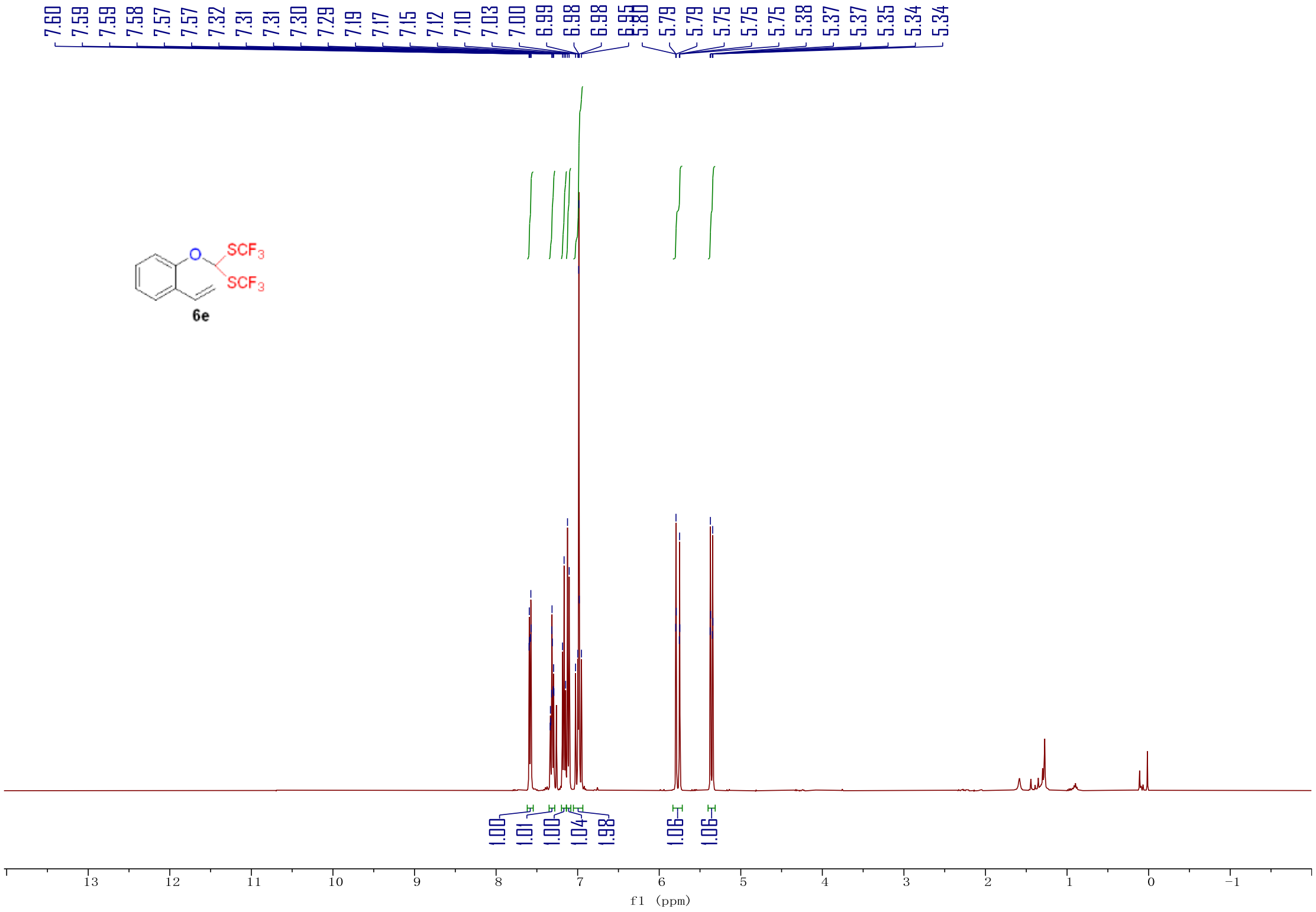

^{19}F NMR Spectrum of **6e** (376 MHz, $CDCl_3$)

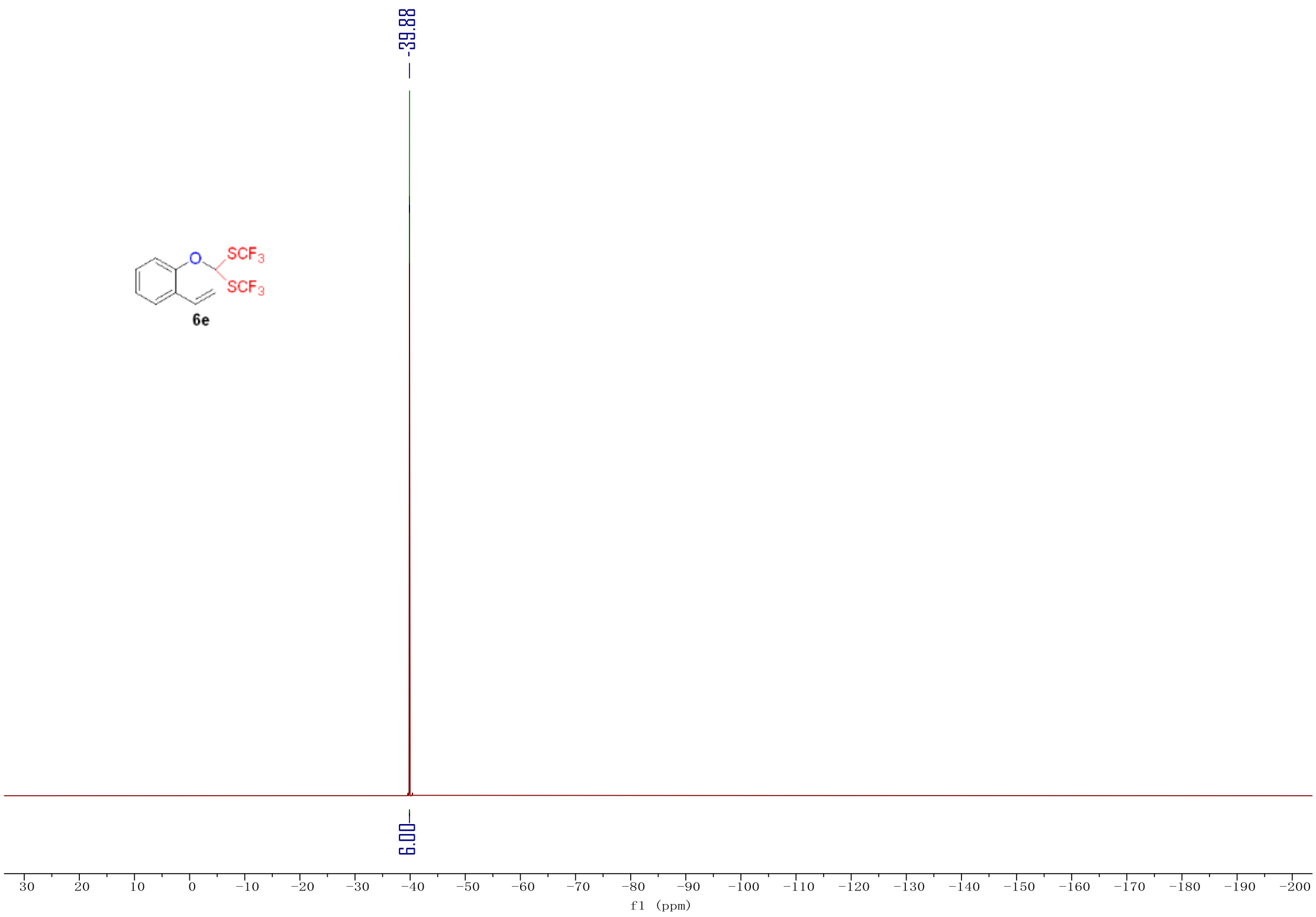

^{13}C NMR Spectrum of **6e** (101 MHz, $CDCl_3$)

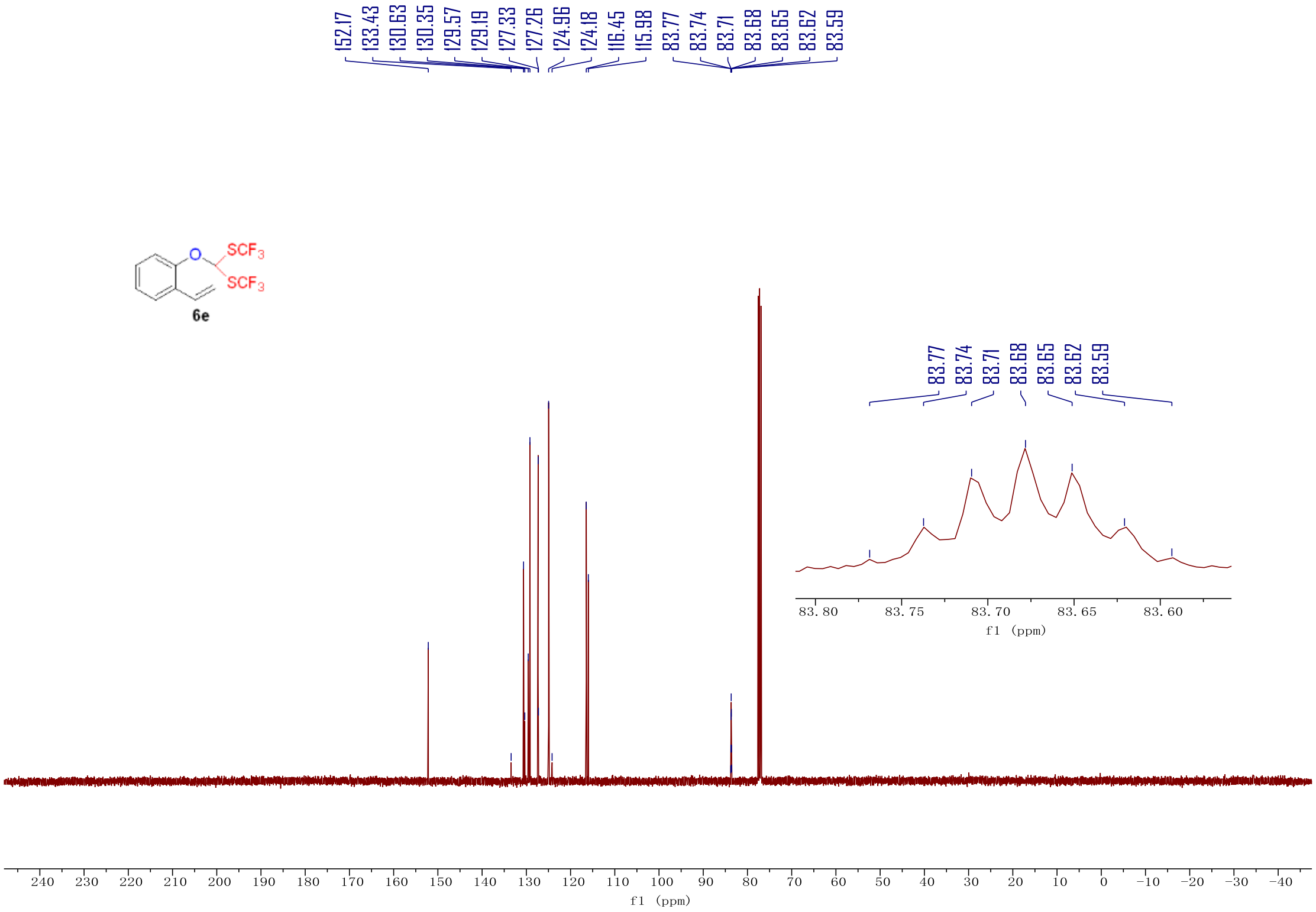

^{1}H NMR Spectrum of **6f** (400 MHz, $CDCl_3$)

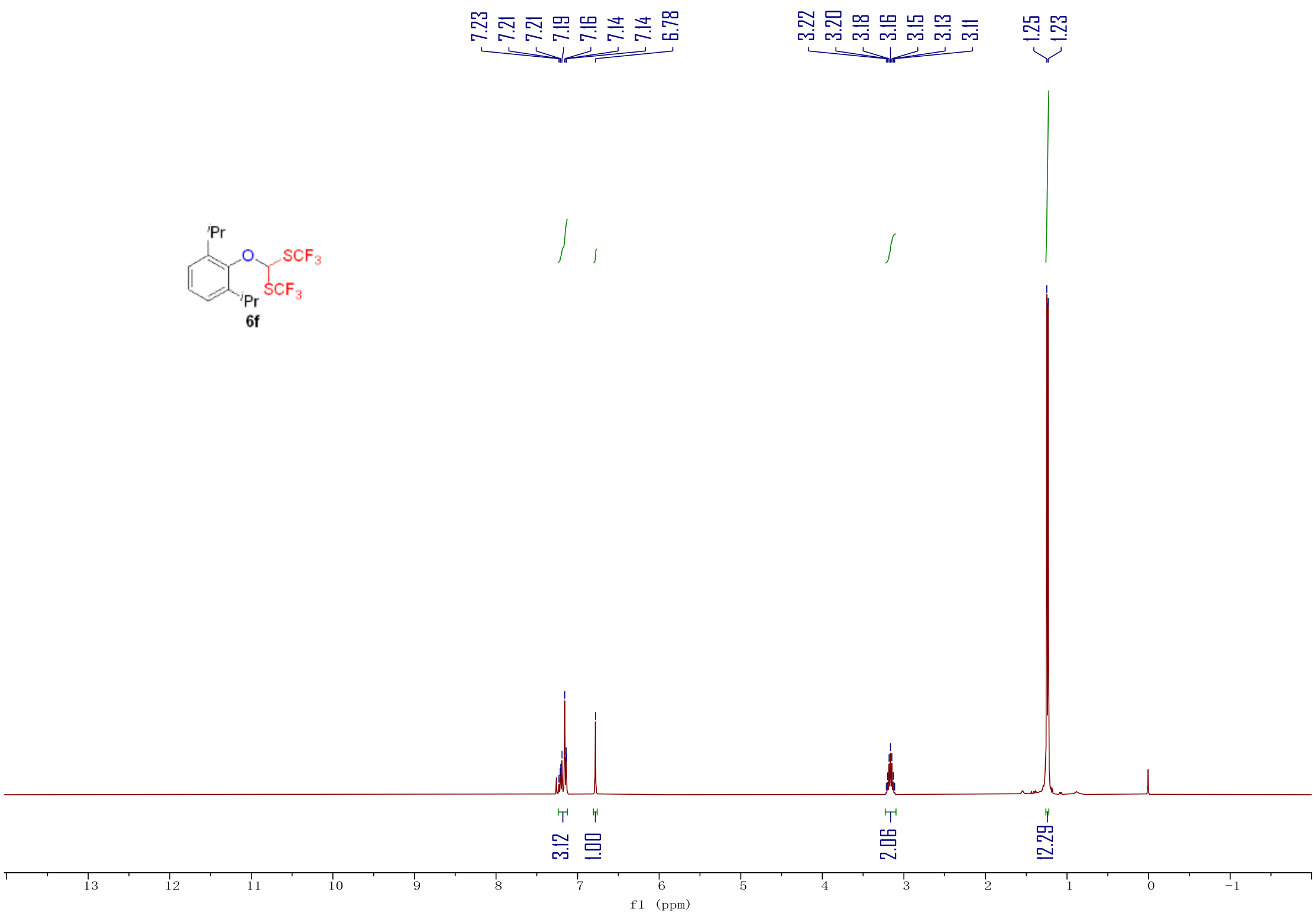

^{19}F NMR Spectrum of **6f** (376 MHz, $CDCl_3$)

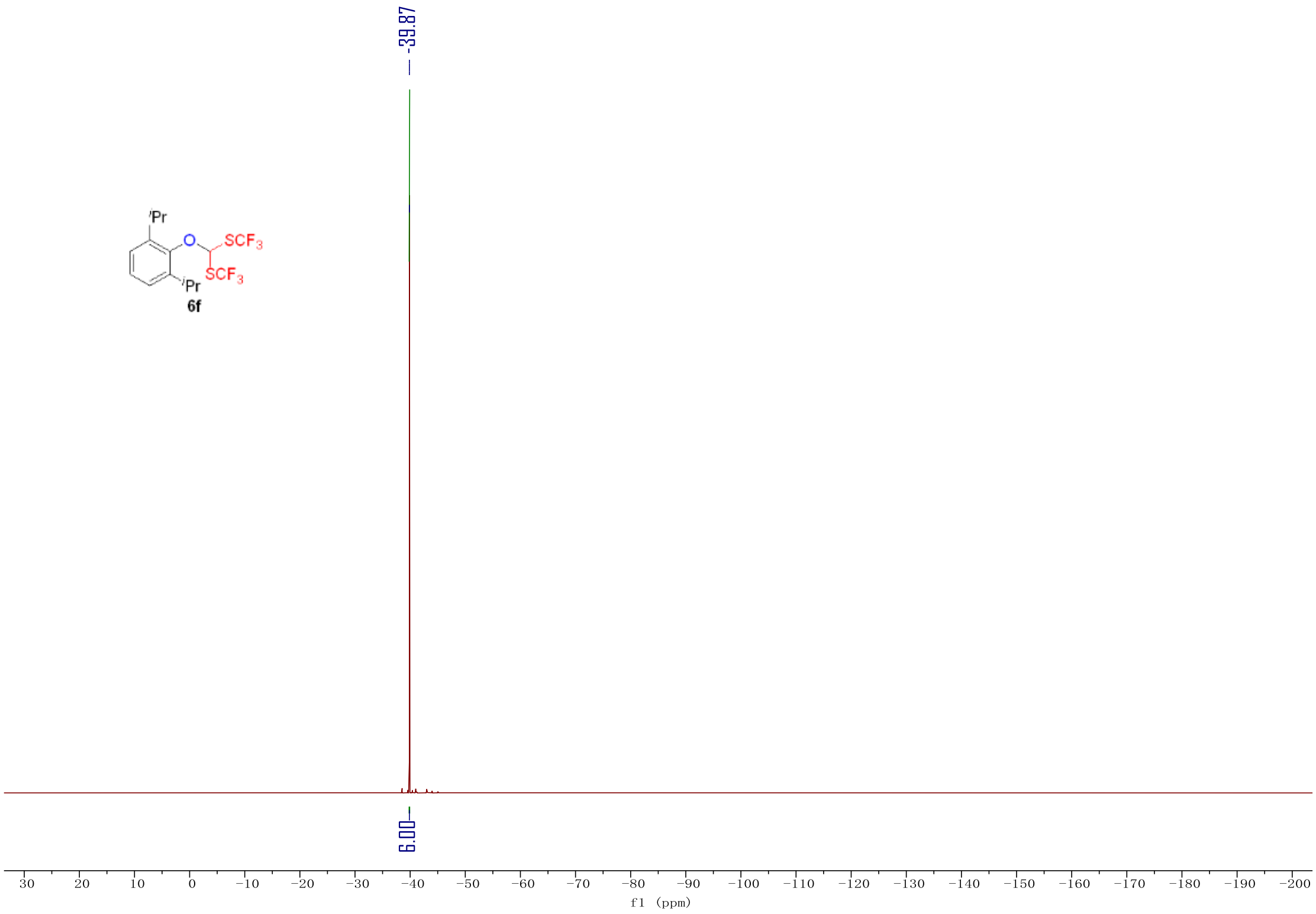

^{13}C NMR Spectrum of **6f** (101 MHz, $CDCl_3$)

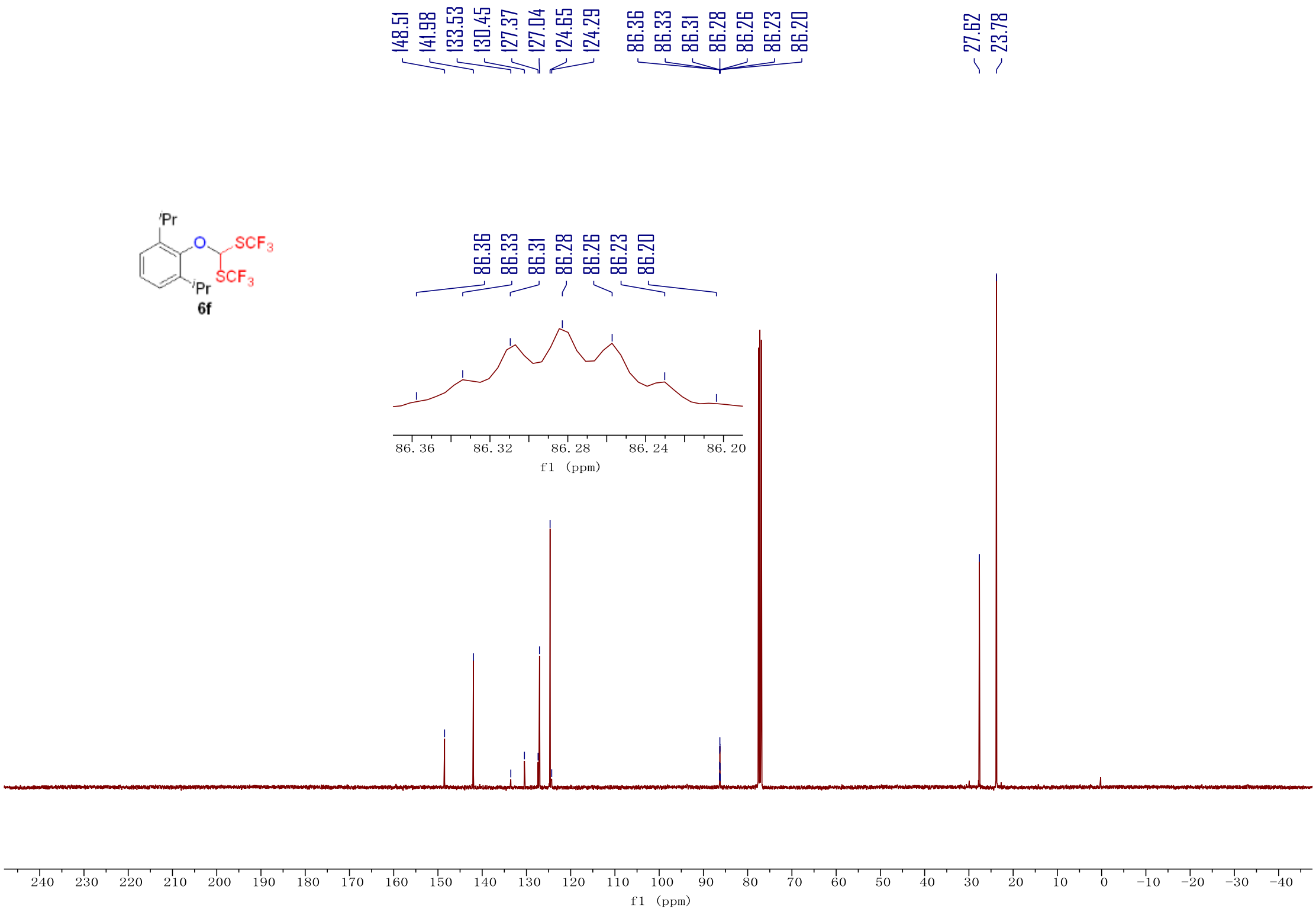

^{1}**H NMR** Spectrum of **6g** (400 MHz, $CDCl_3$)

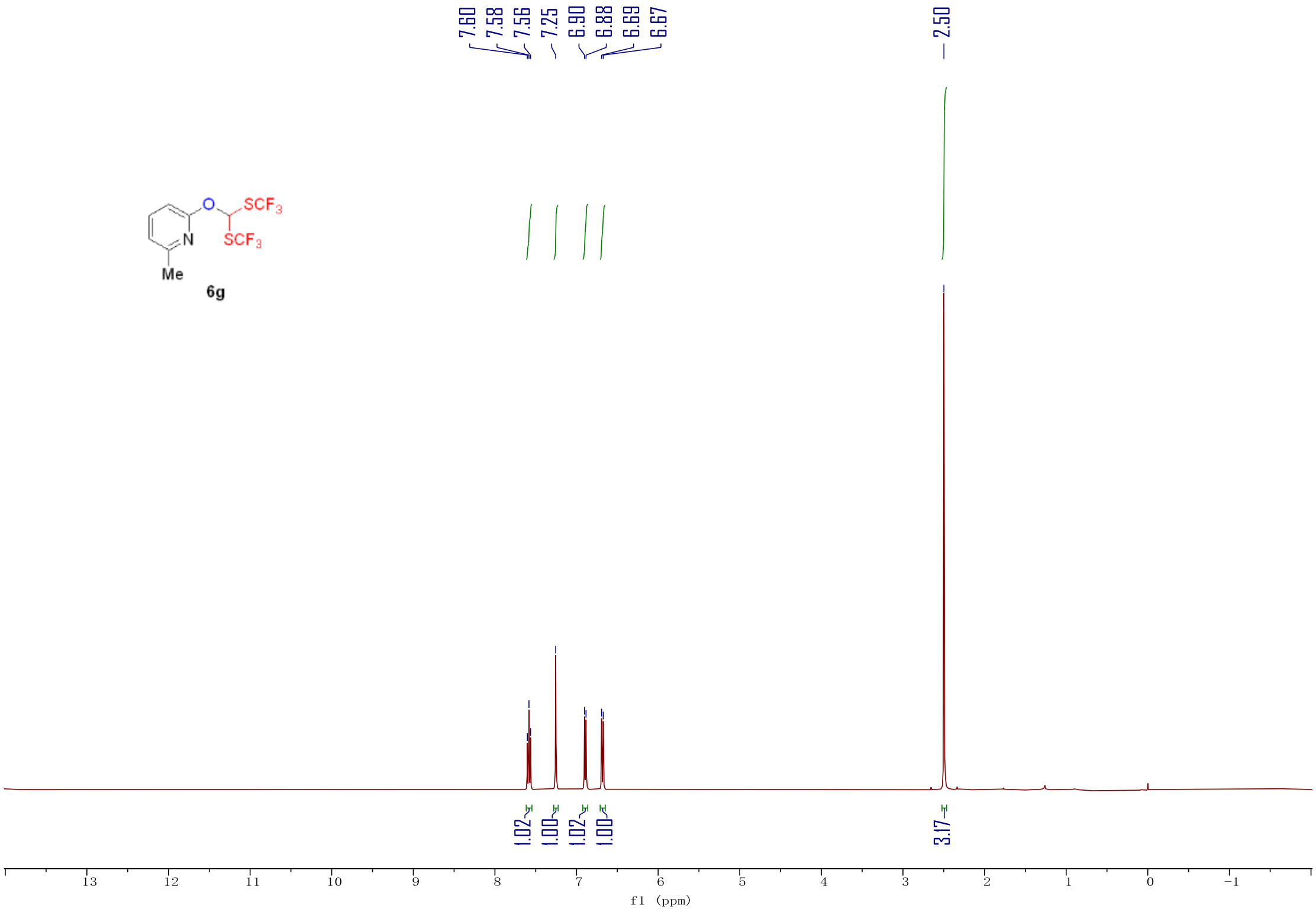

^{19}**F NMR** Spectrum of **6g** (376 MHz, $CDCl_3$)

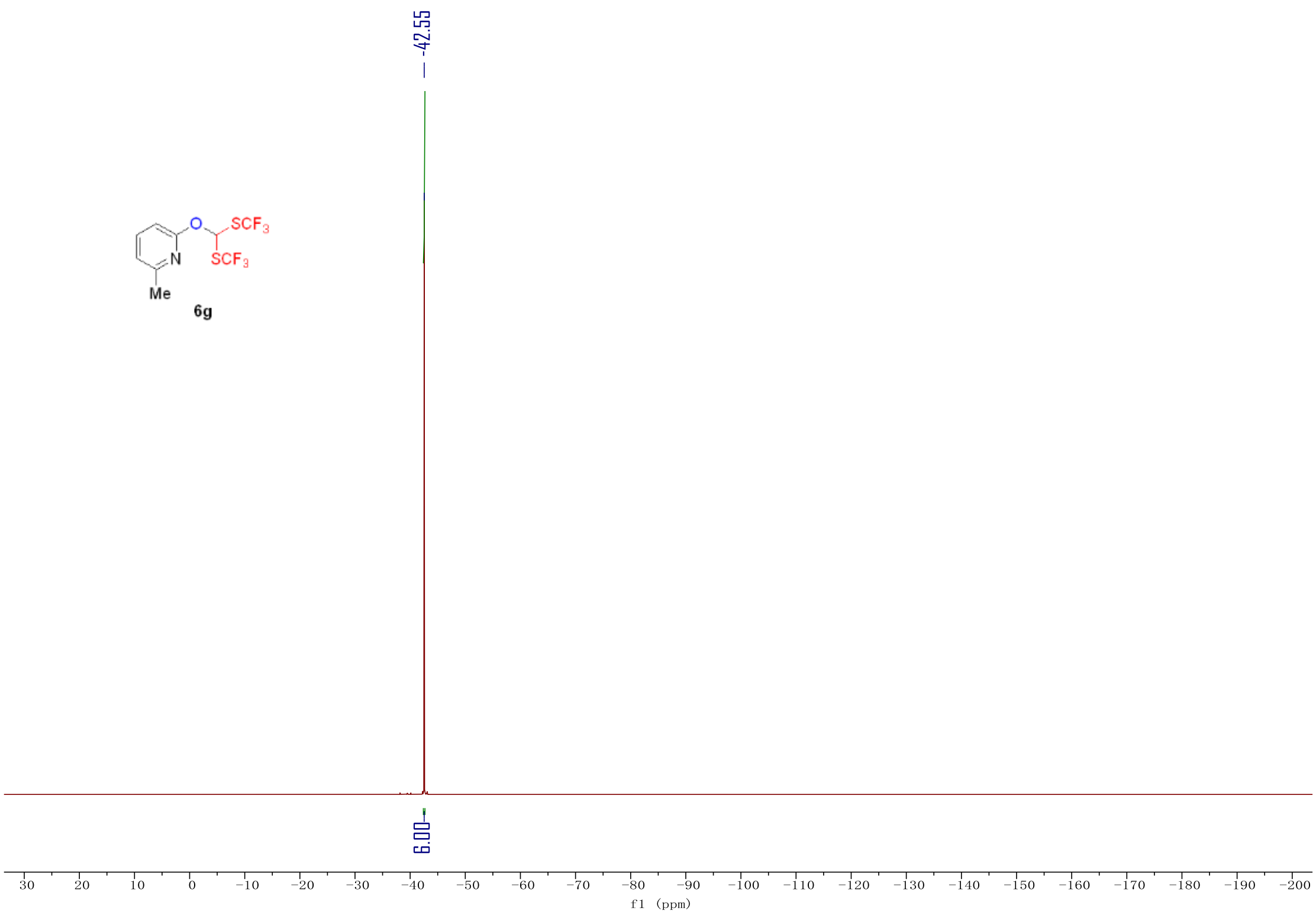

^{13}C NMR Spectrum of **6g** (101 MHz, $CDCl_3$)

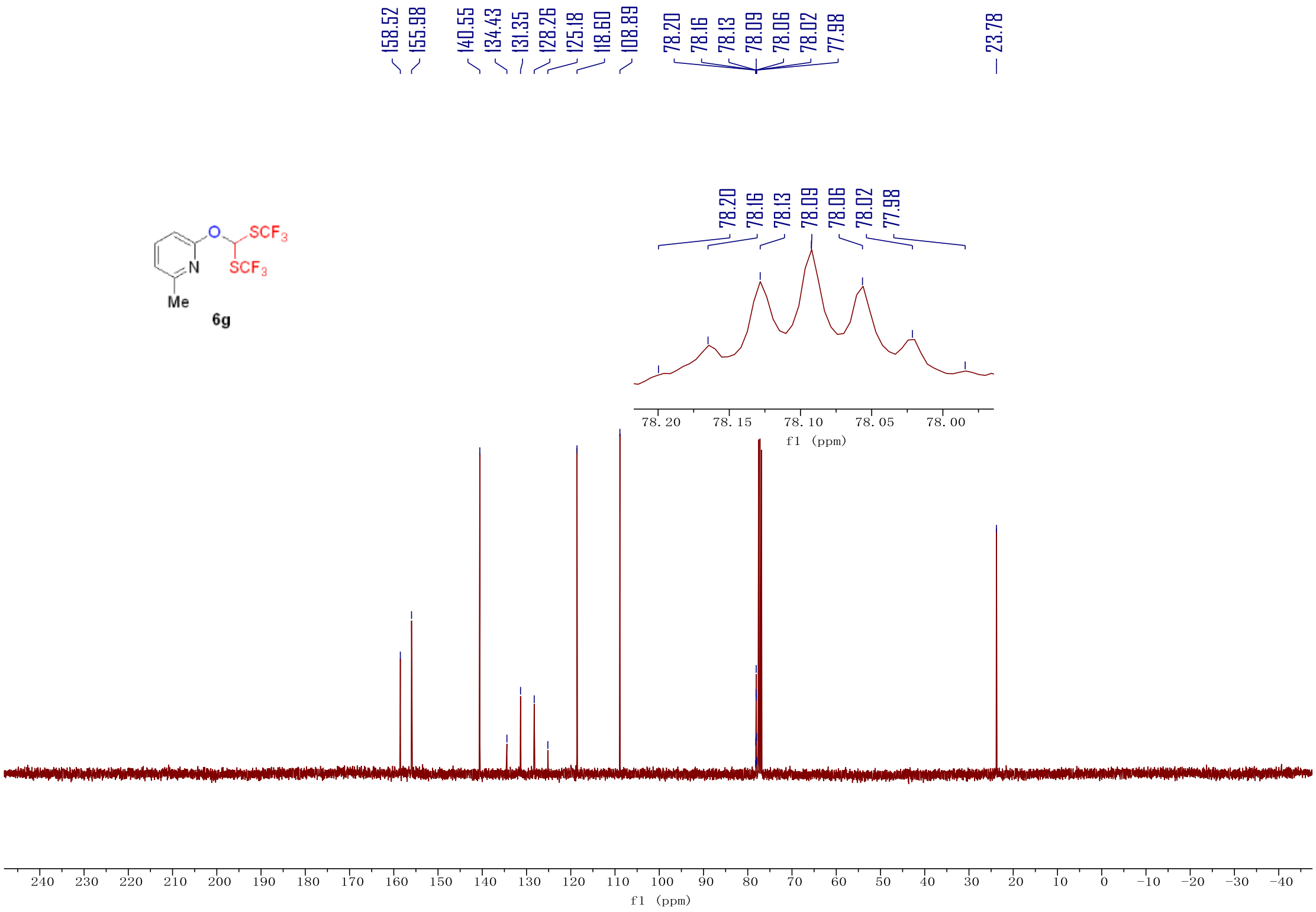

^{1}H NMR Spectrum of **6h** (400 MHz, $CDCl_3$)

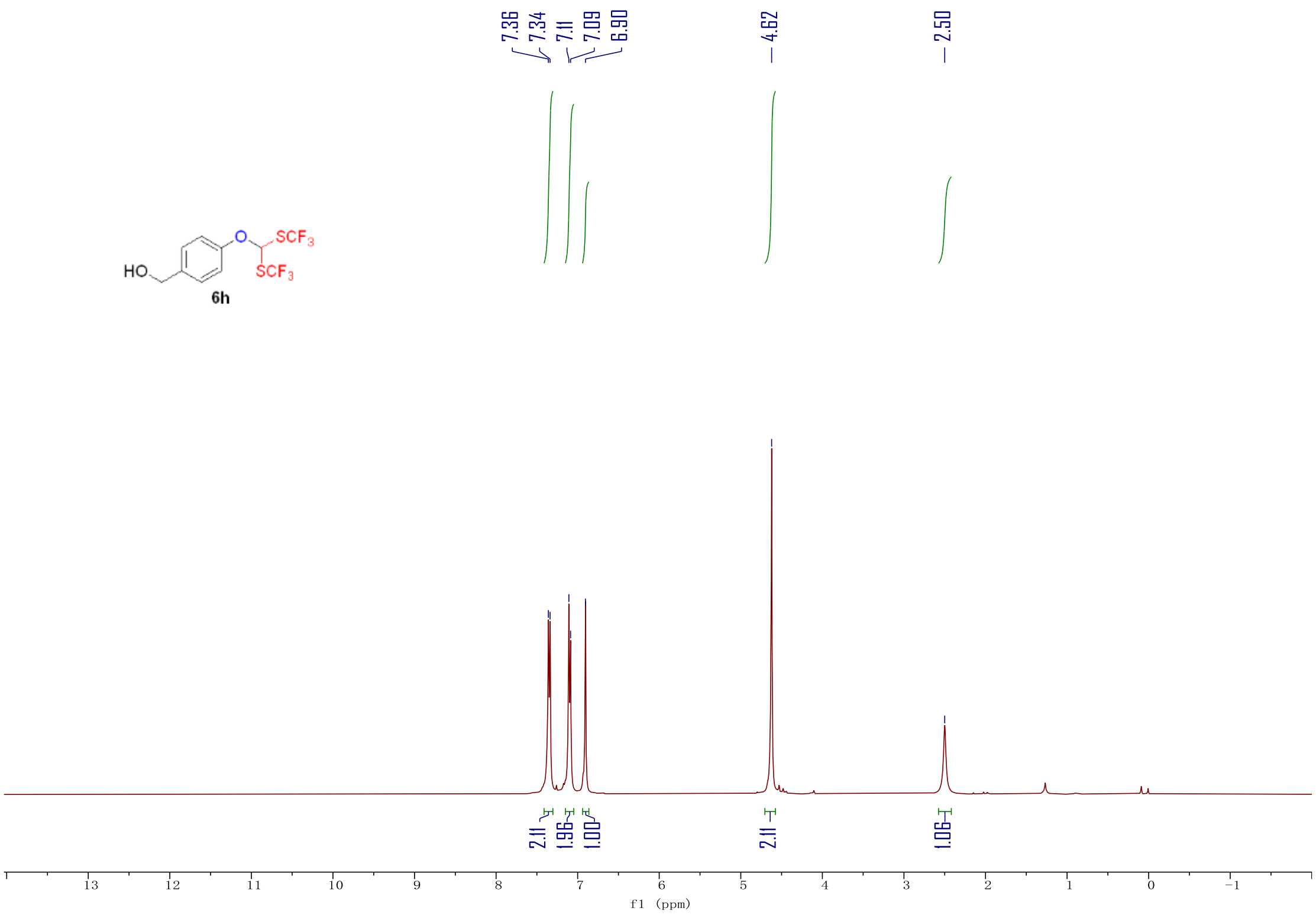

^{19}F **NMR** Spectrum of **6h** (376 MHz, $CDCl_3$)

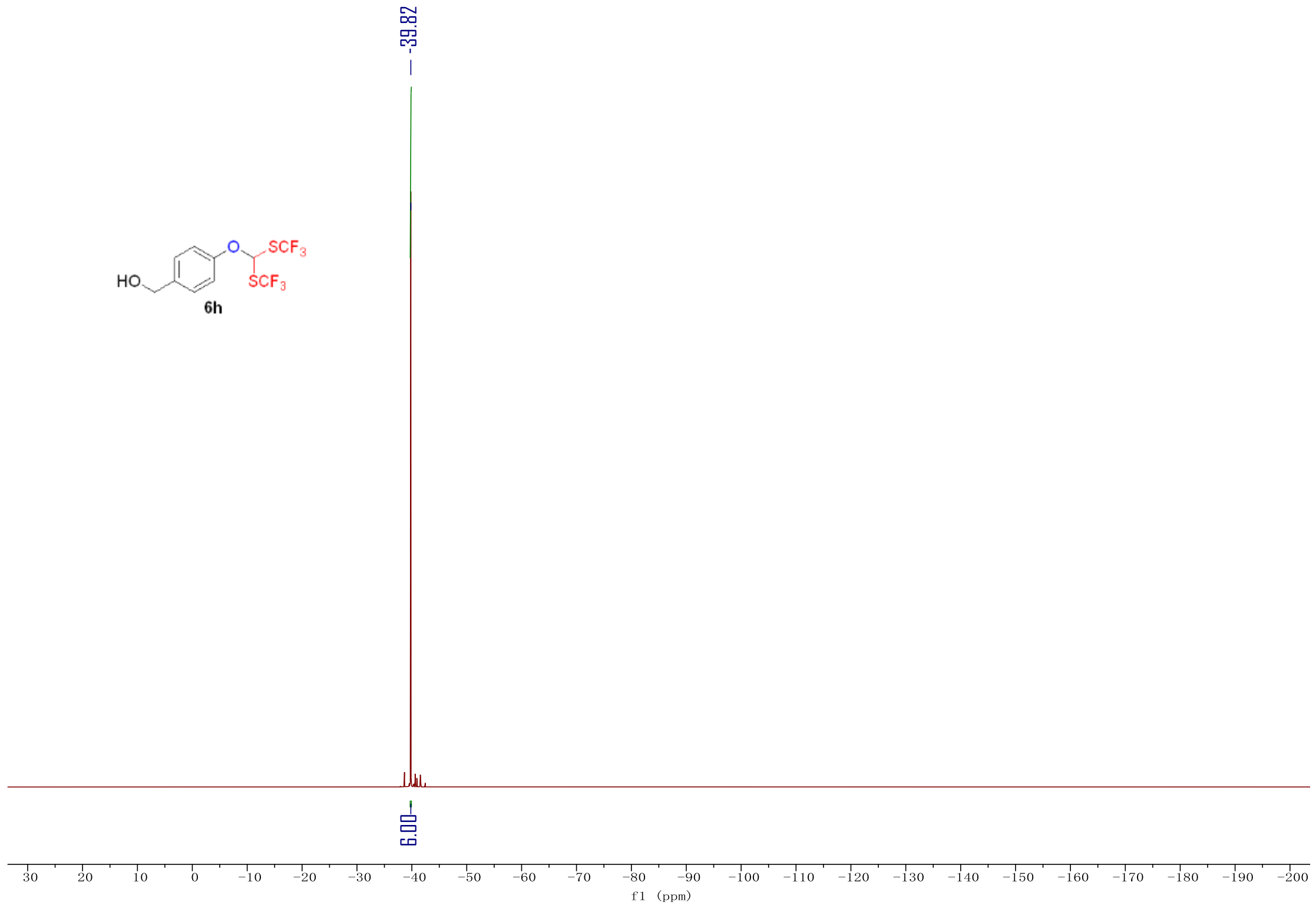

^{13}C **NMR** Spectrum of **6h** (101 MHz, $CDCl_3$)

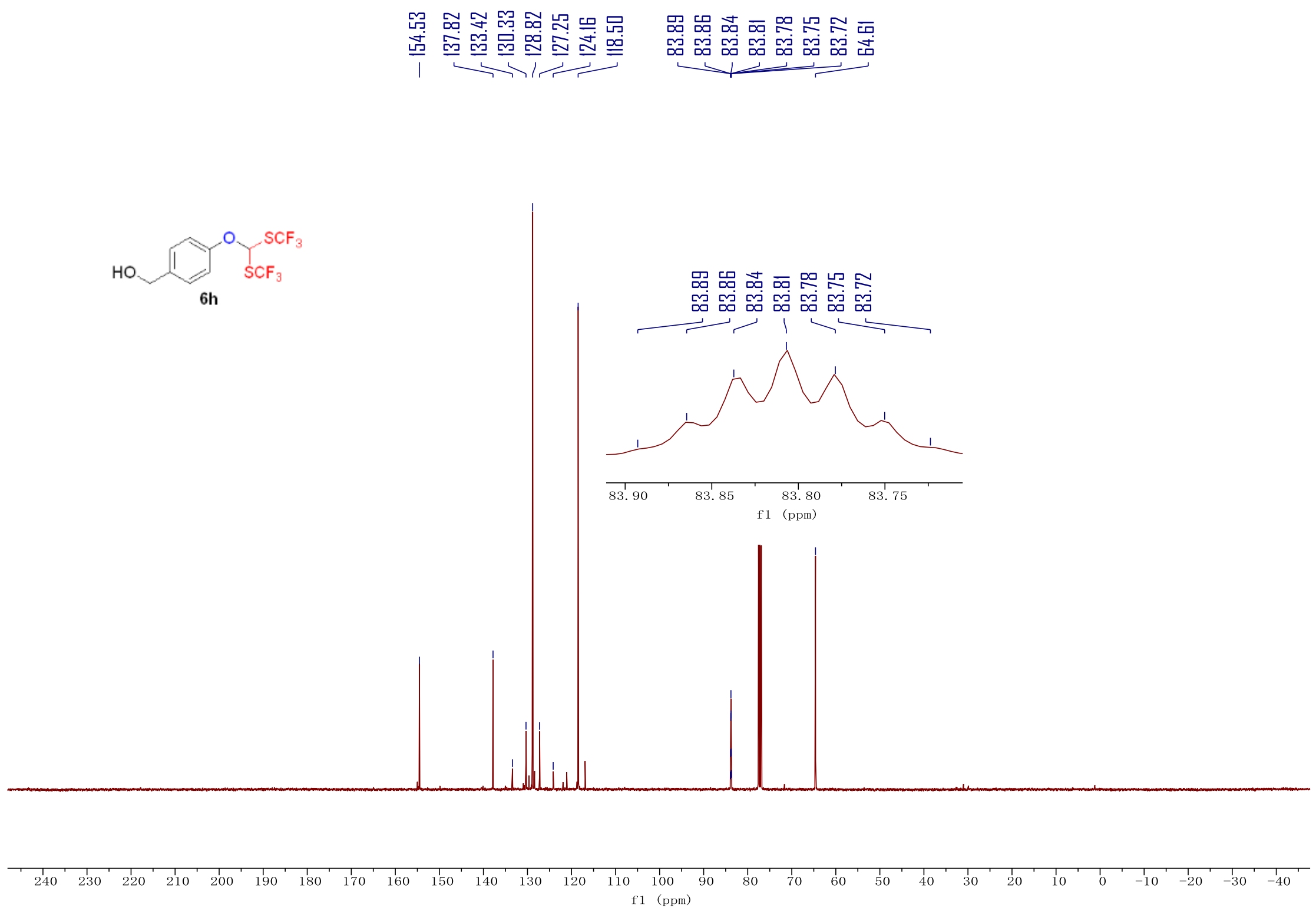

^{1}H NMR Spectrum of **6i** (400 MHz, $CDCl_3$)

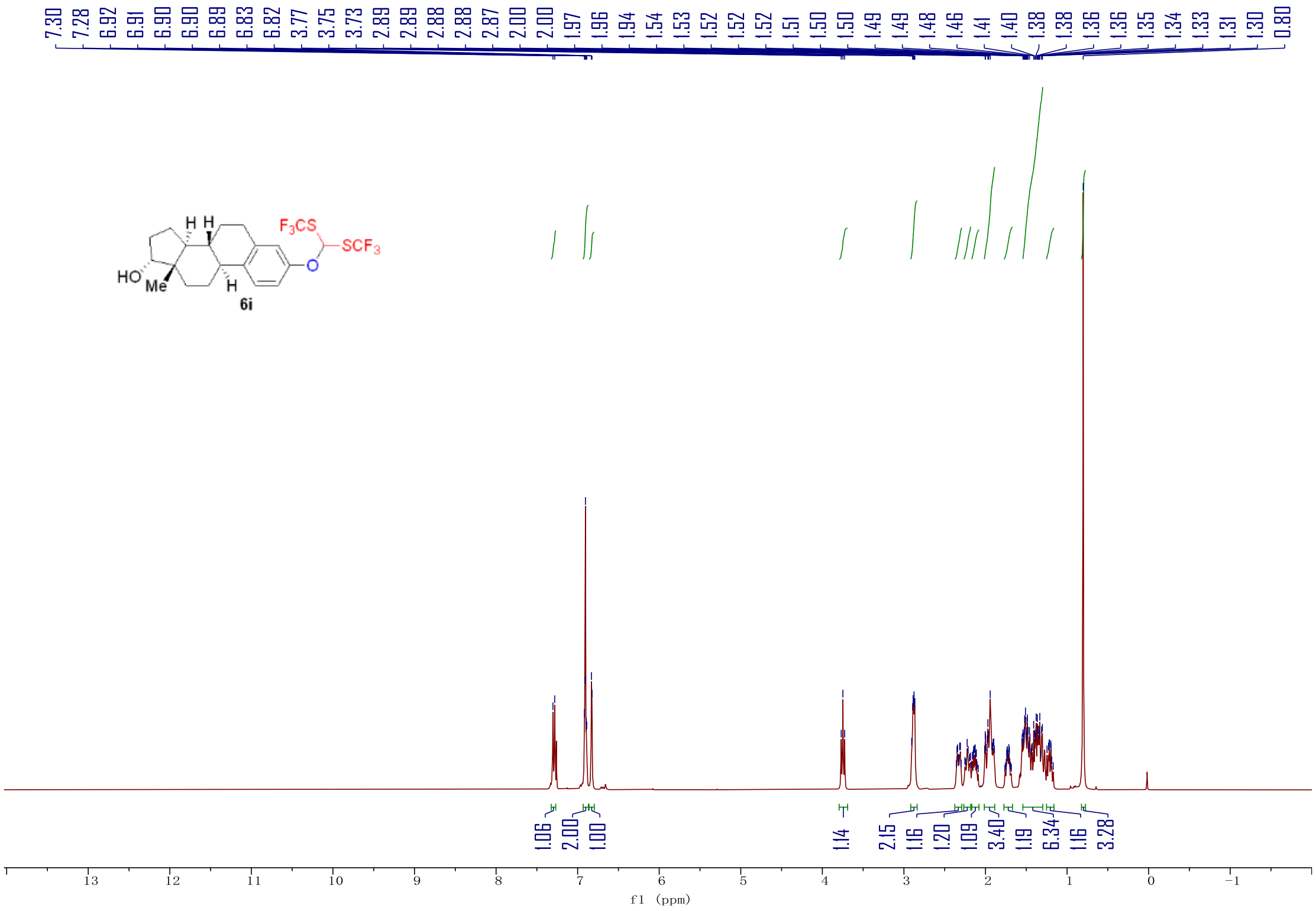

^{19}F NMR Spectrum of **6i** (376 MHz, $CDCl_3$)

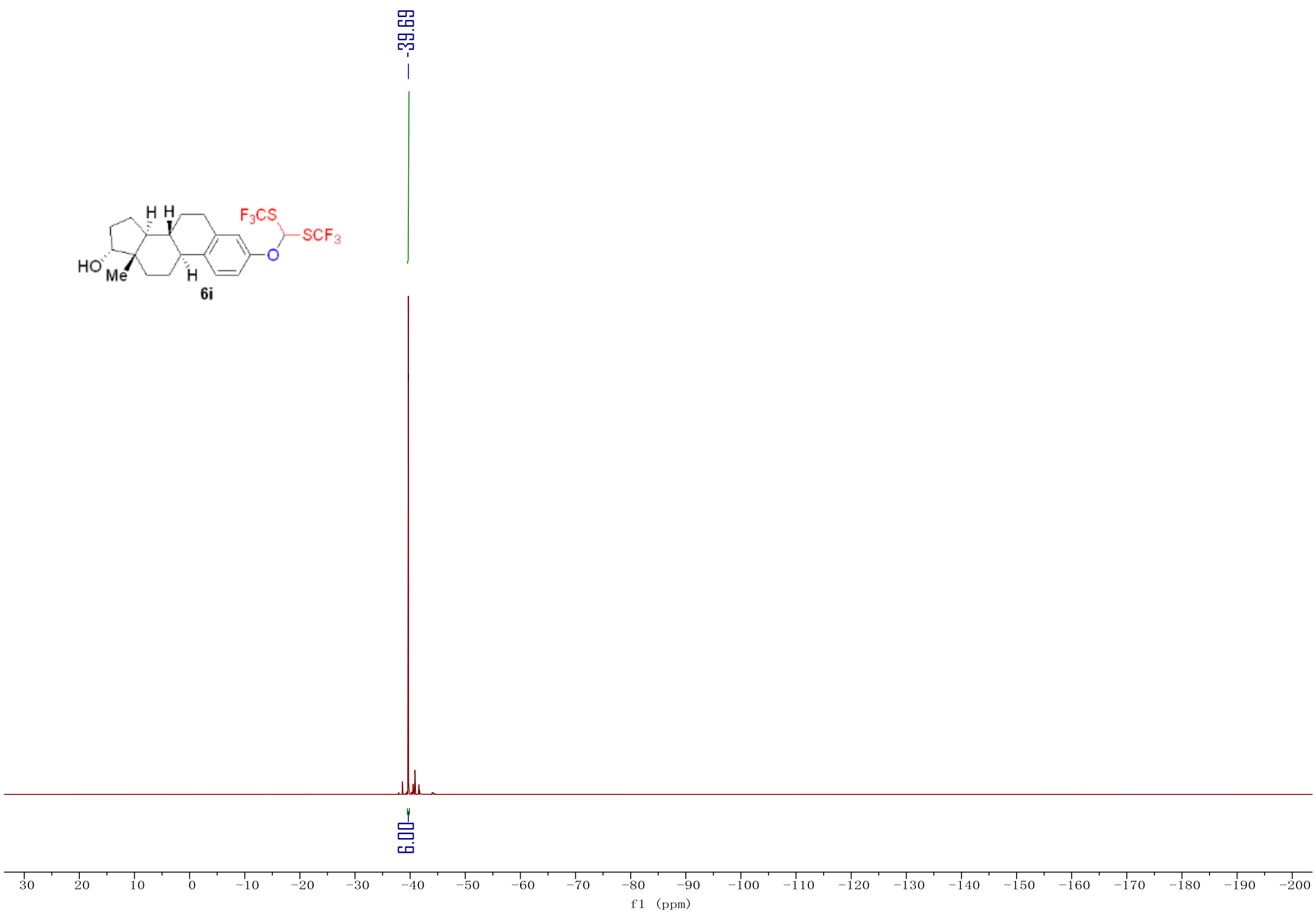

^{13}C NMR Spectrum of **6i** (101 MHz, $CDCl_3$)

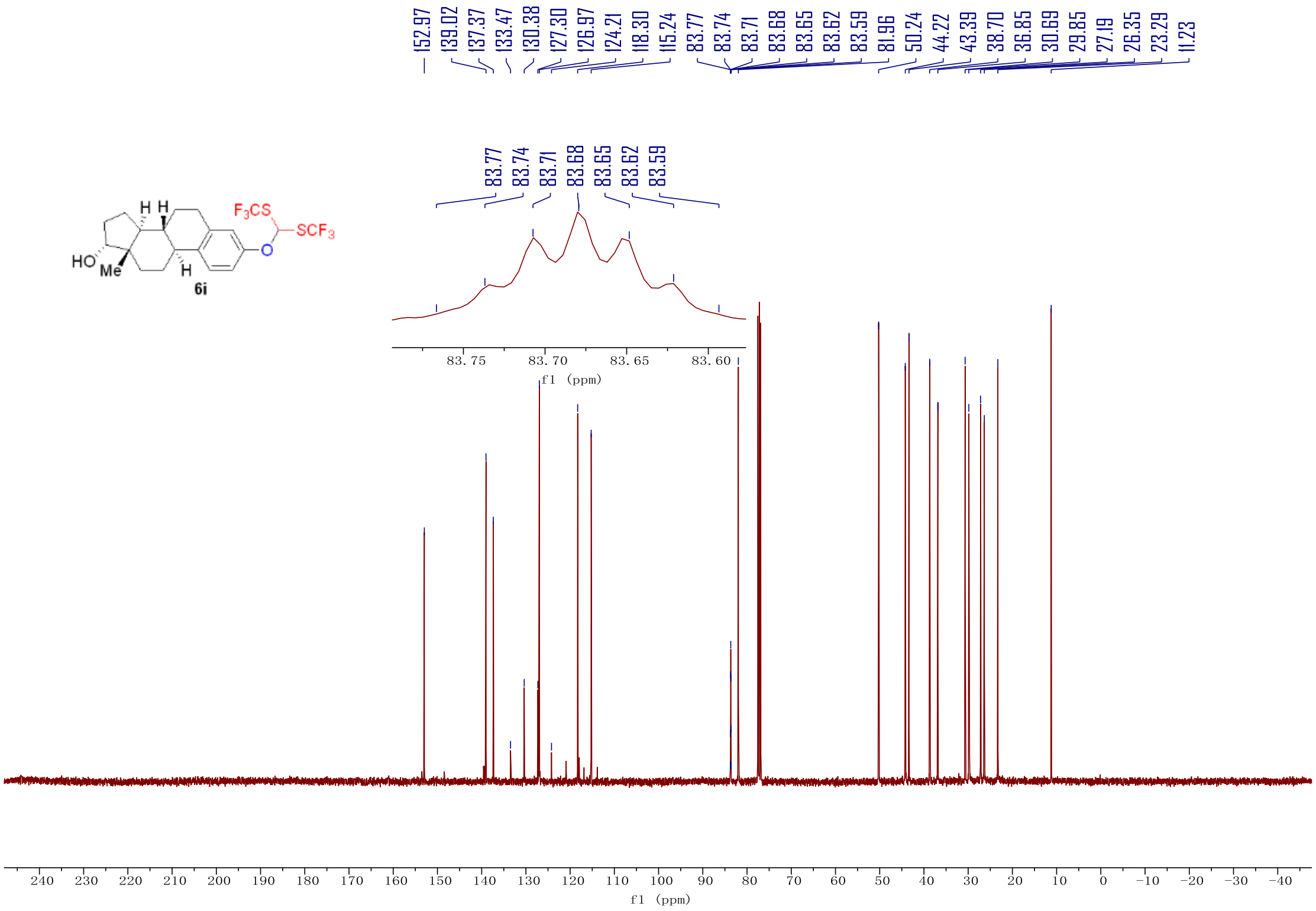

^{1}H NMR Spectrum of **6j** (400 MHz, $CDCl_3$)

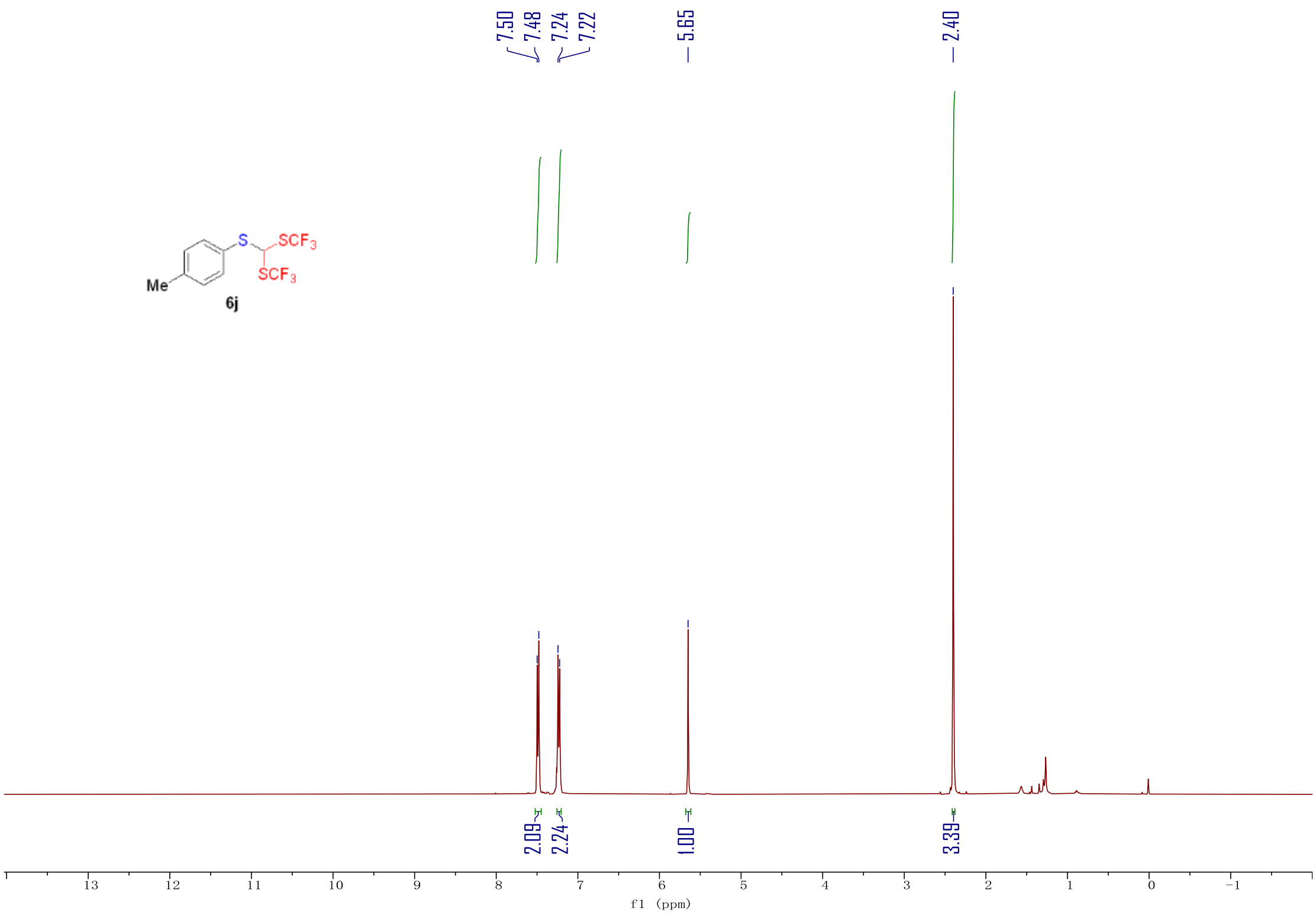

^{19}F NMR Spectrum of **6j** (376 MHz, $CDCl_3$)

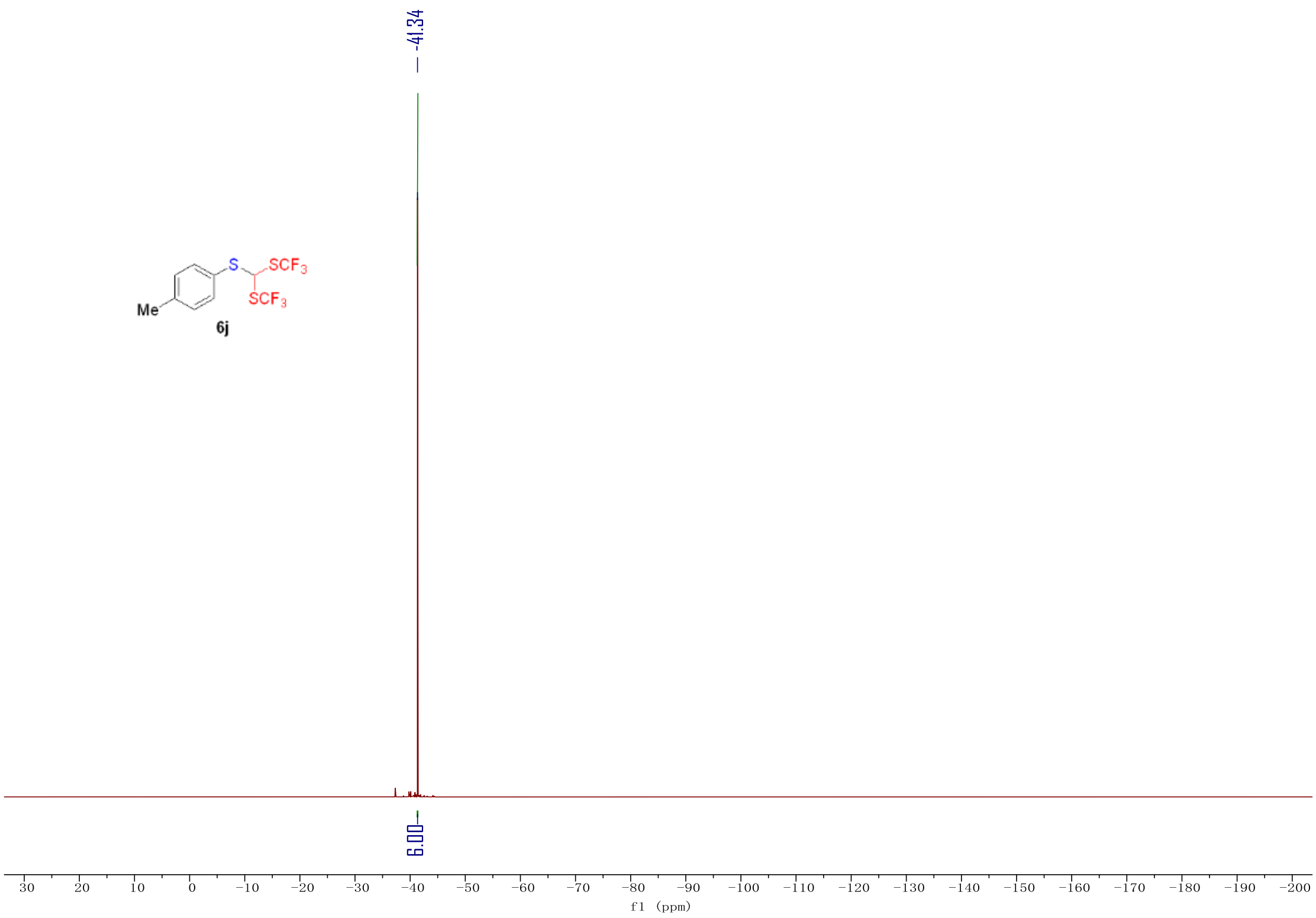

^{13}C NMR Spectrum of **6j** (101 MHz, $CDCl_3$)

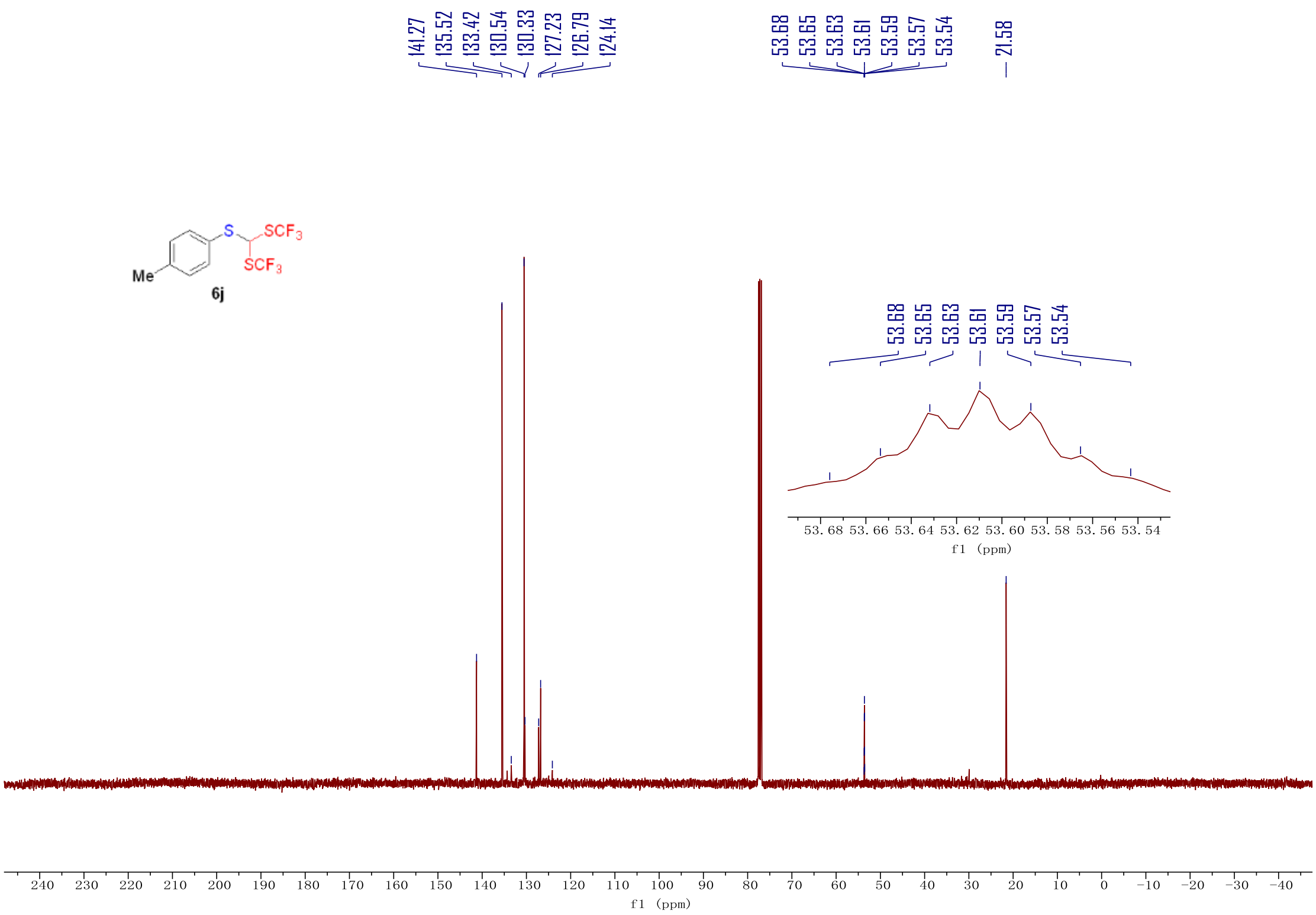

[1]**H NMR** Spectrum of **6k** (400 MHz, $CDCl_3$)

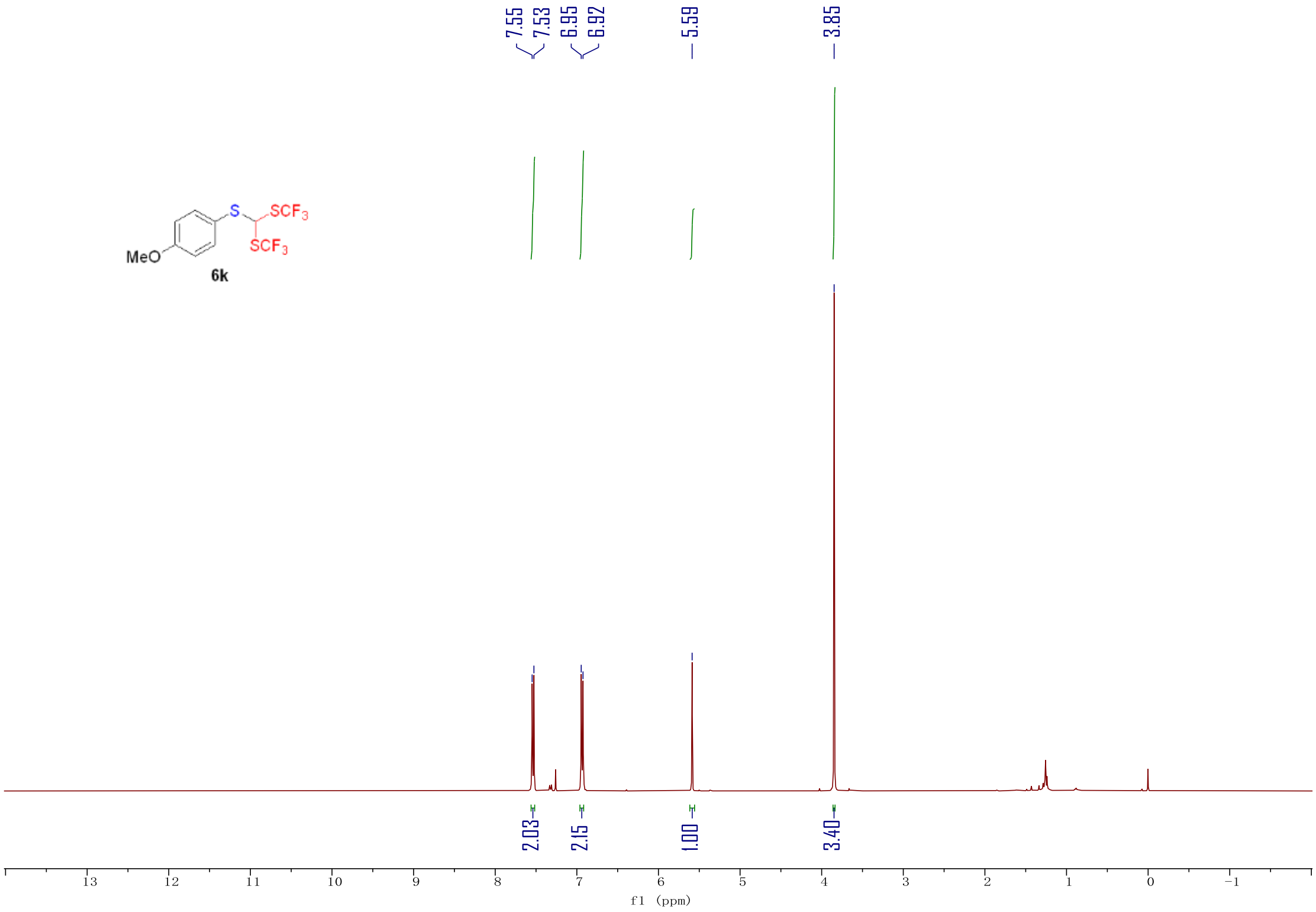

[19]**F NMR** Spectrum of **6k** (376 MHz, $CDCl_3$)

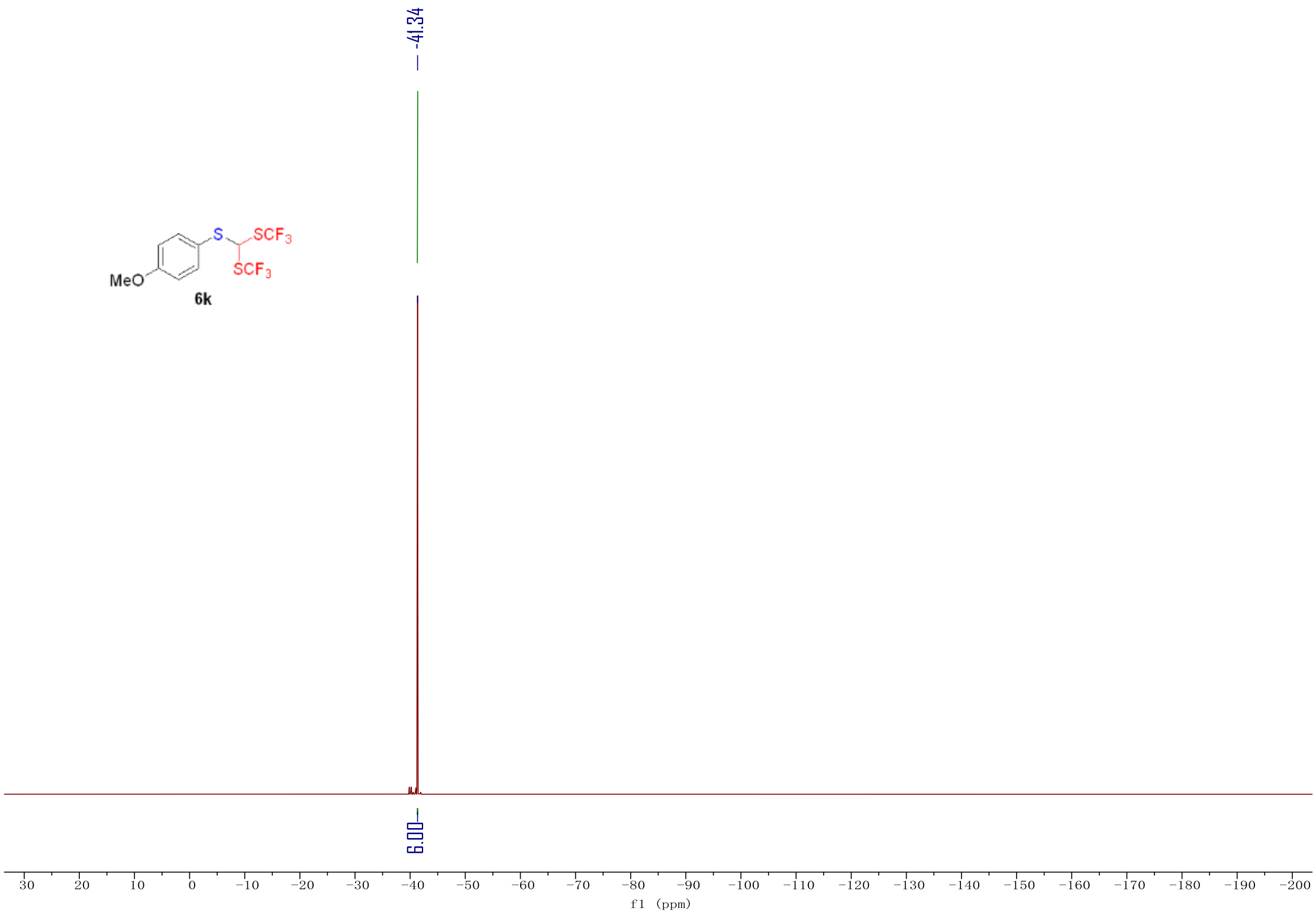

^{13}C NMR Spectrum of **6k** (101 MHz, $CDCl_3$)

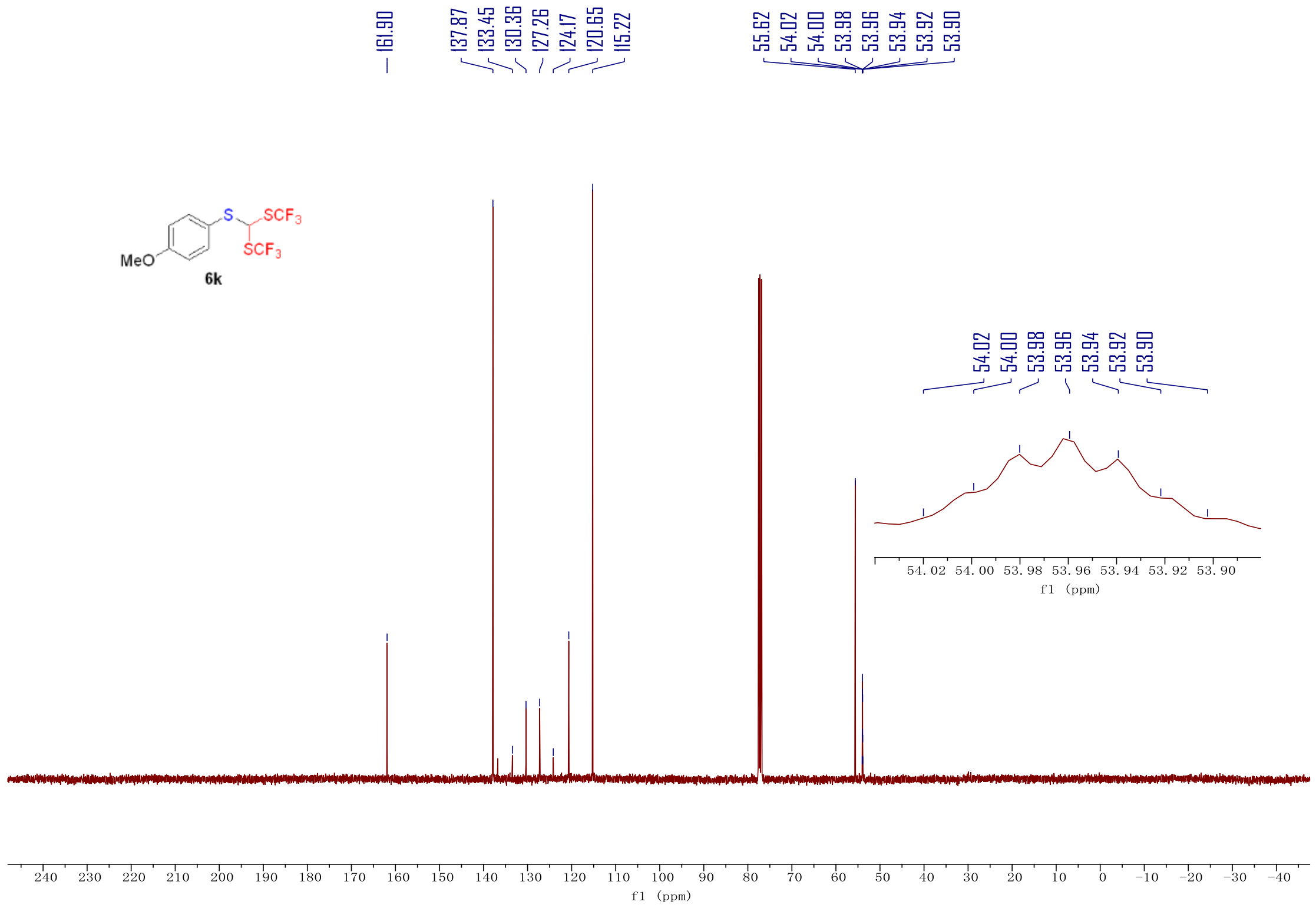

^{1}H NMR Spectrum of **6l** (400 MHz, $CDCl_3$)

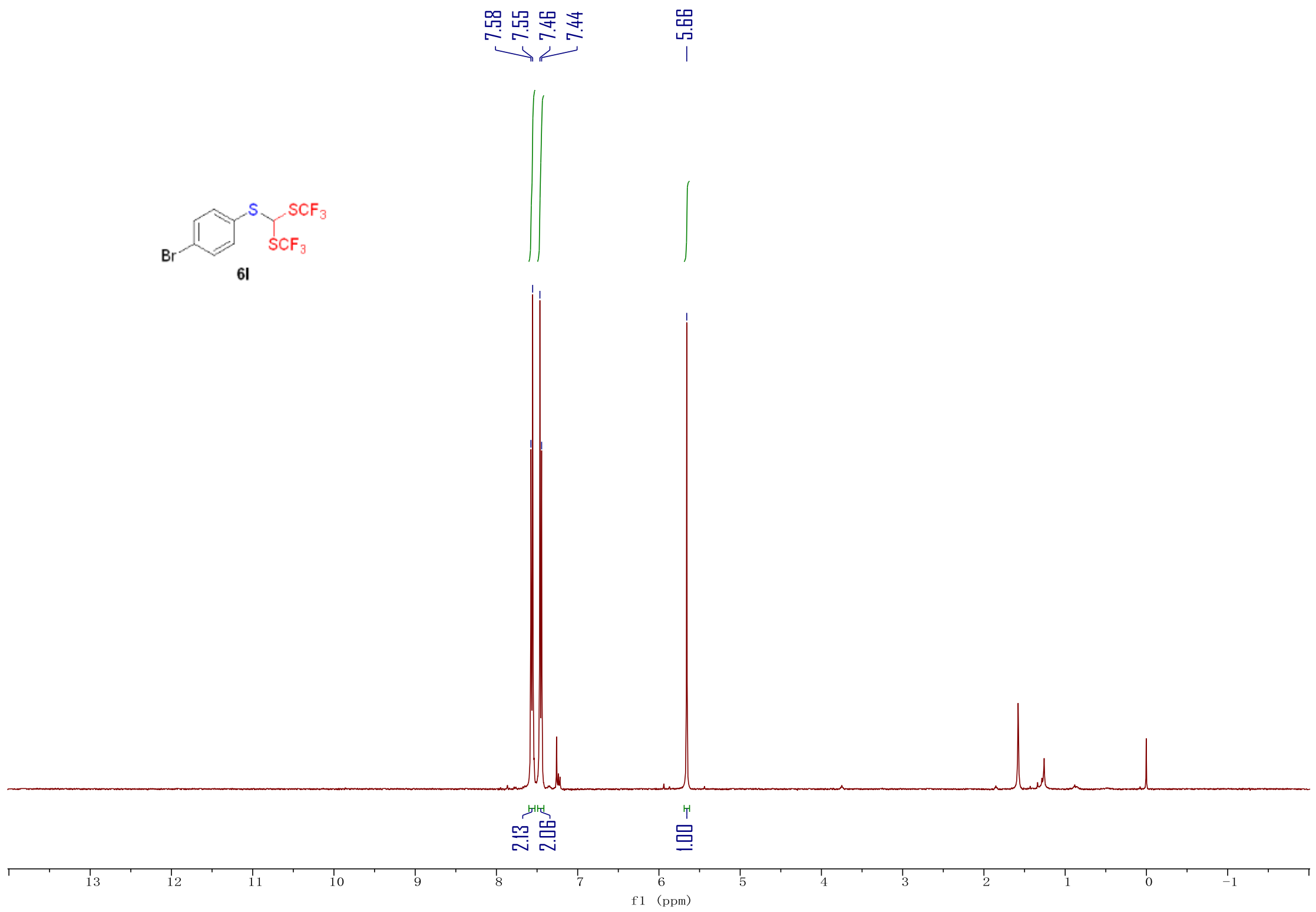

^{19}F NMR Spectrum of **6l** (376 MHz, $CDCl_3$)

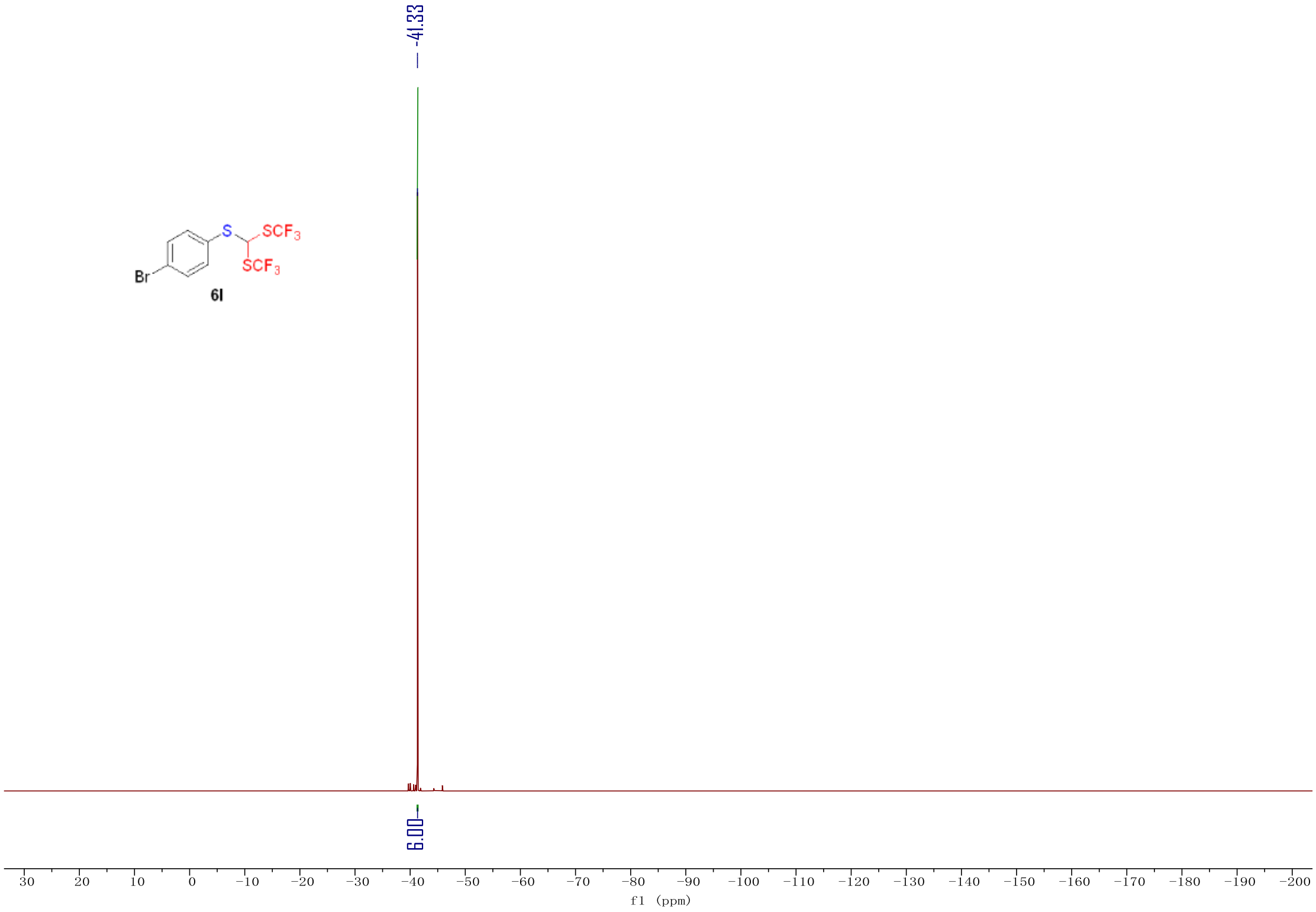

^{13}C NMR Spectrum of **6l** (101 MHz, $CDCl_3$)

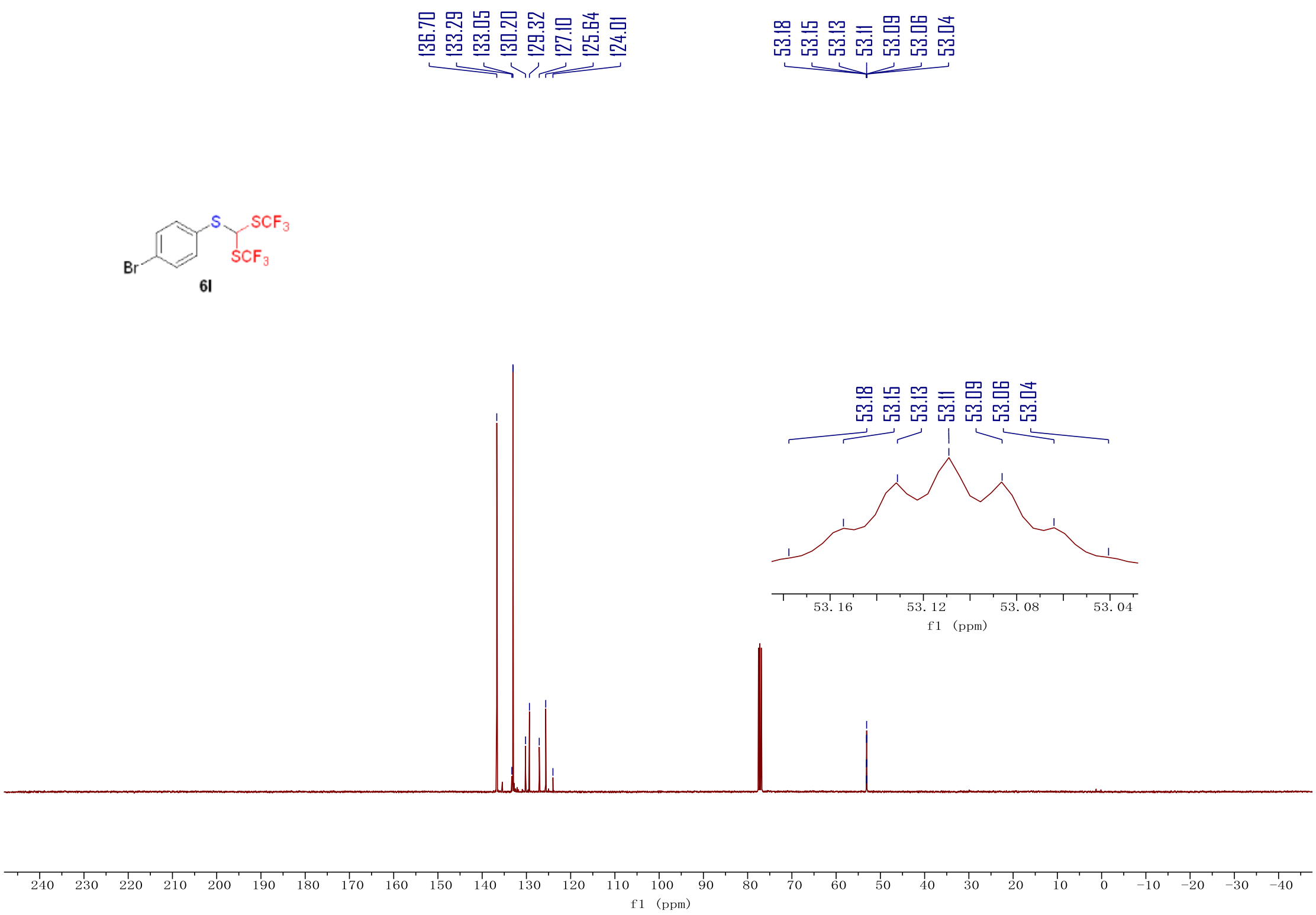

^{1}H NMR Spectrum of **6m** (400 MHz, $CDCl_3$)

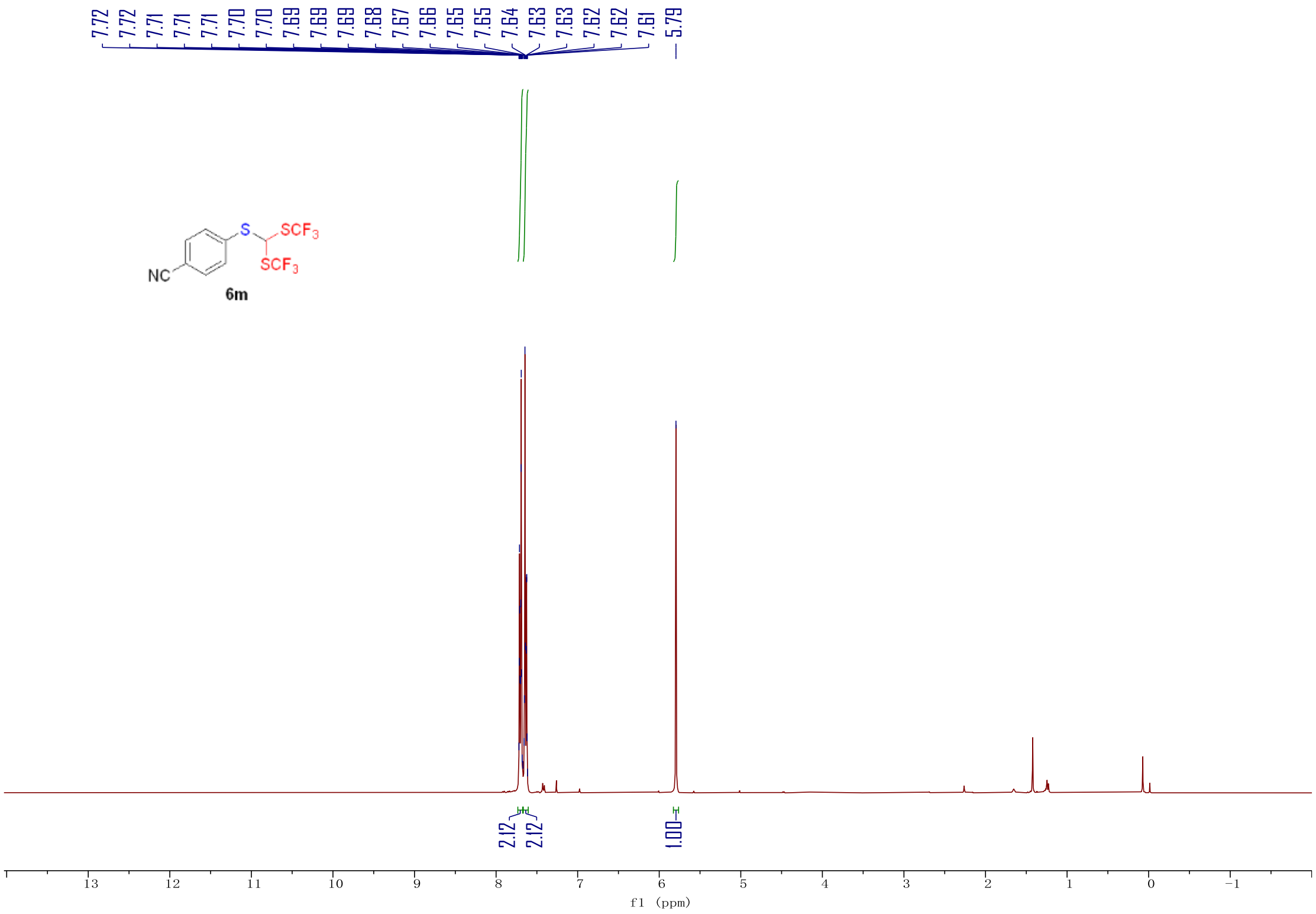

^{19}F NMR Spectrum of **6m** (376 MHz, $CDCl_3$)

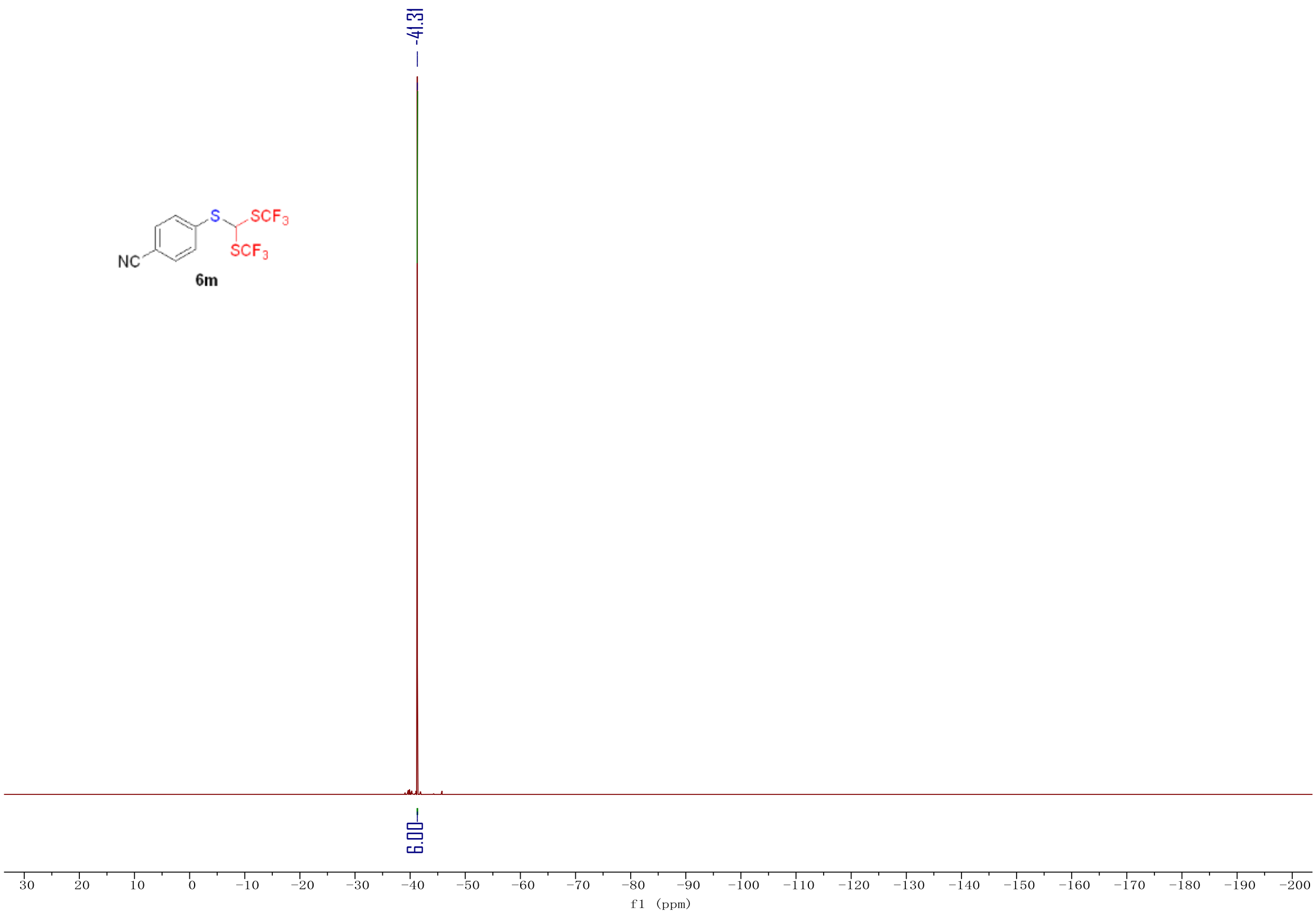

^{13}C NMR Spectrum of **6m** (101 MHz, $CDCl_3$)

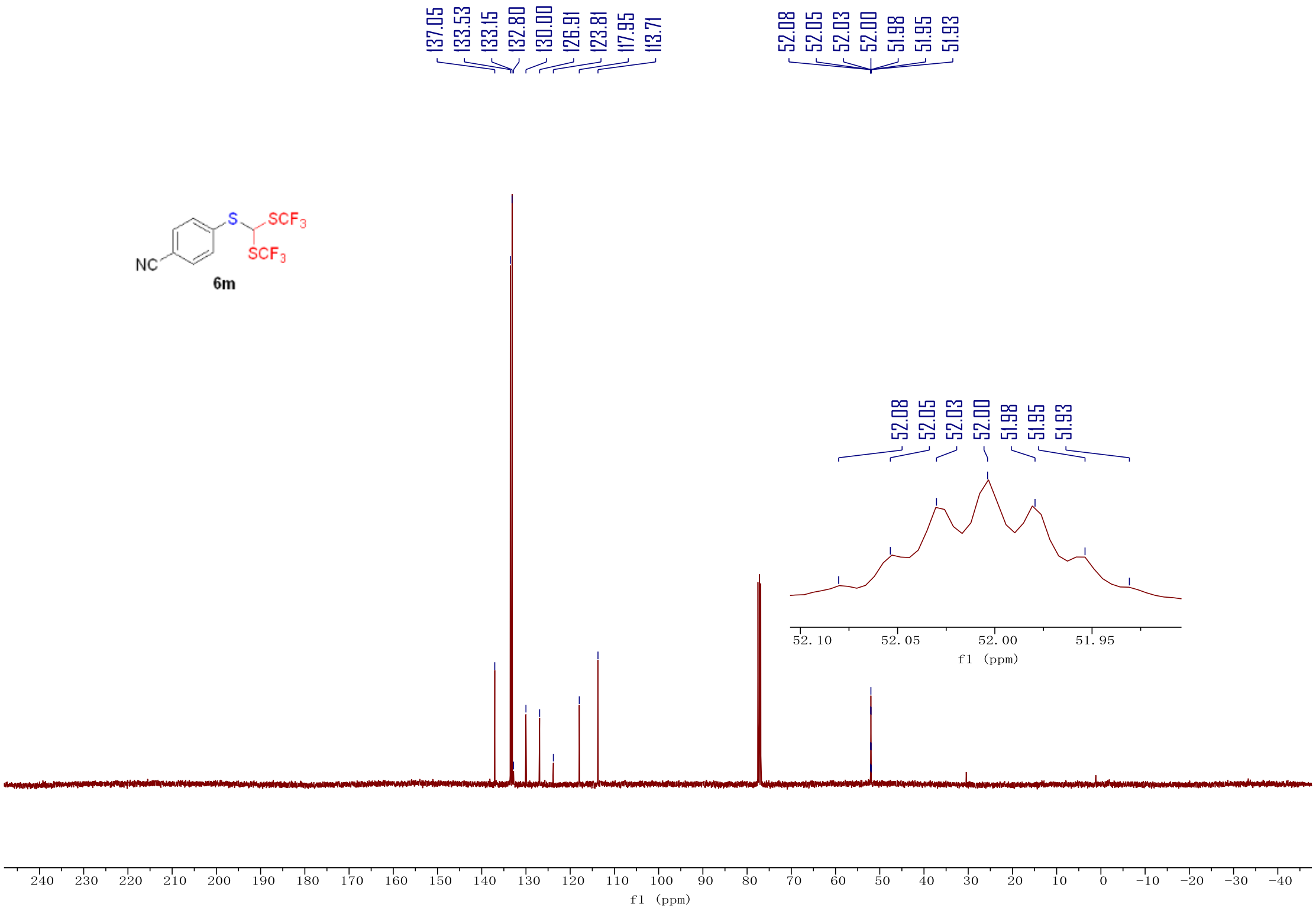

^{1}H NMR Spectrum of **6n** (400 MHz, $CDCl_3$)

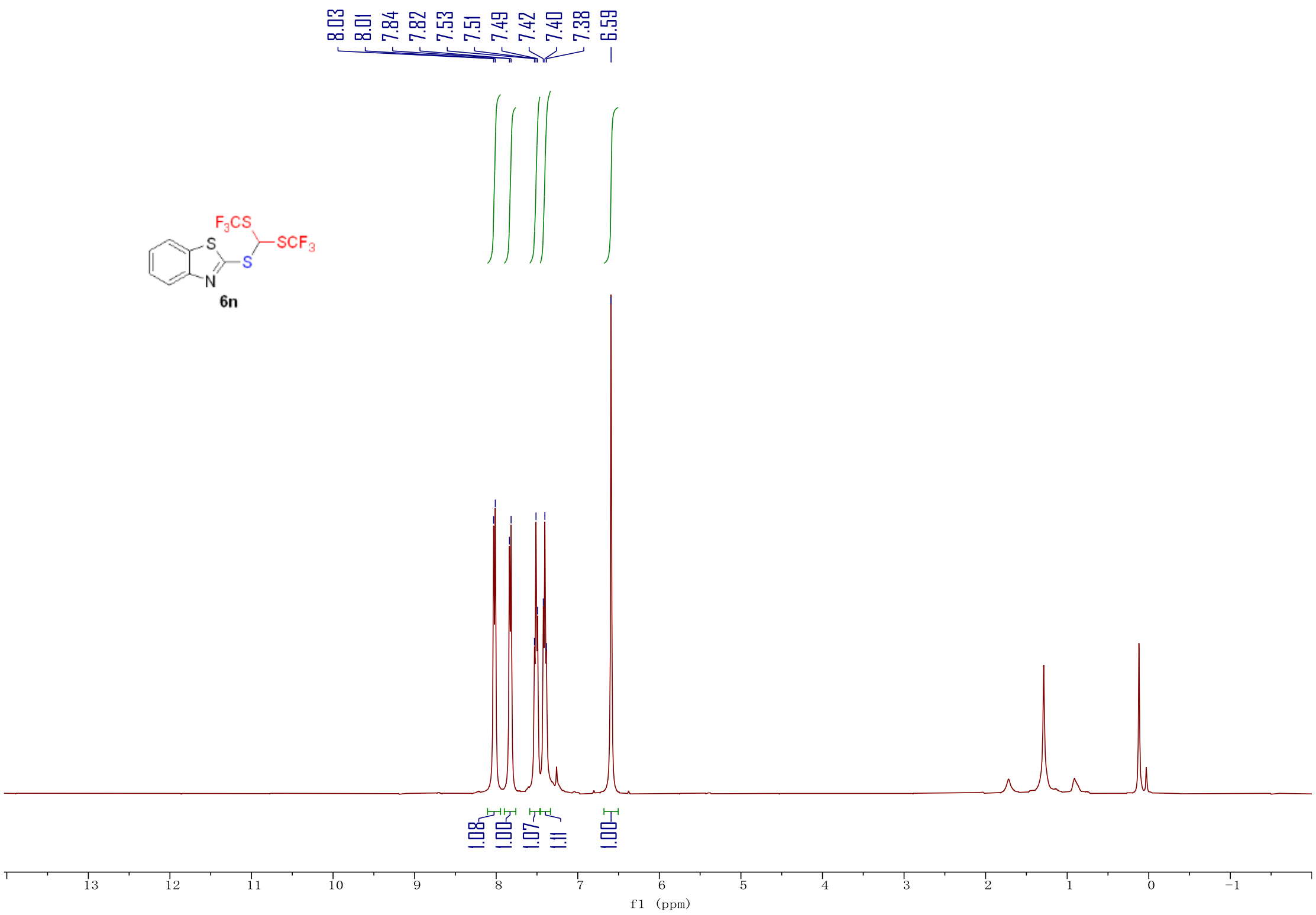

^{19}F NMR Spectrum of **6n** (376 MHz, $CDCl_3$)

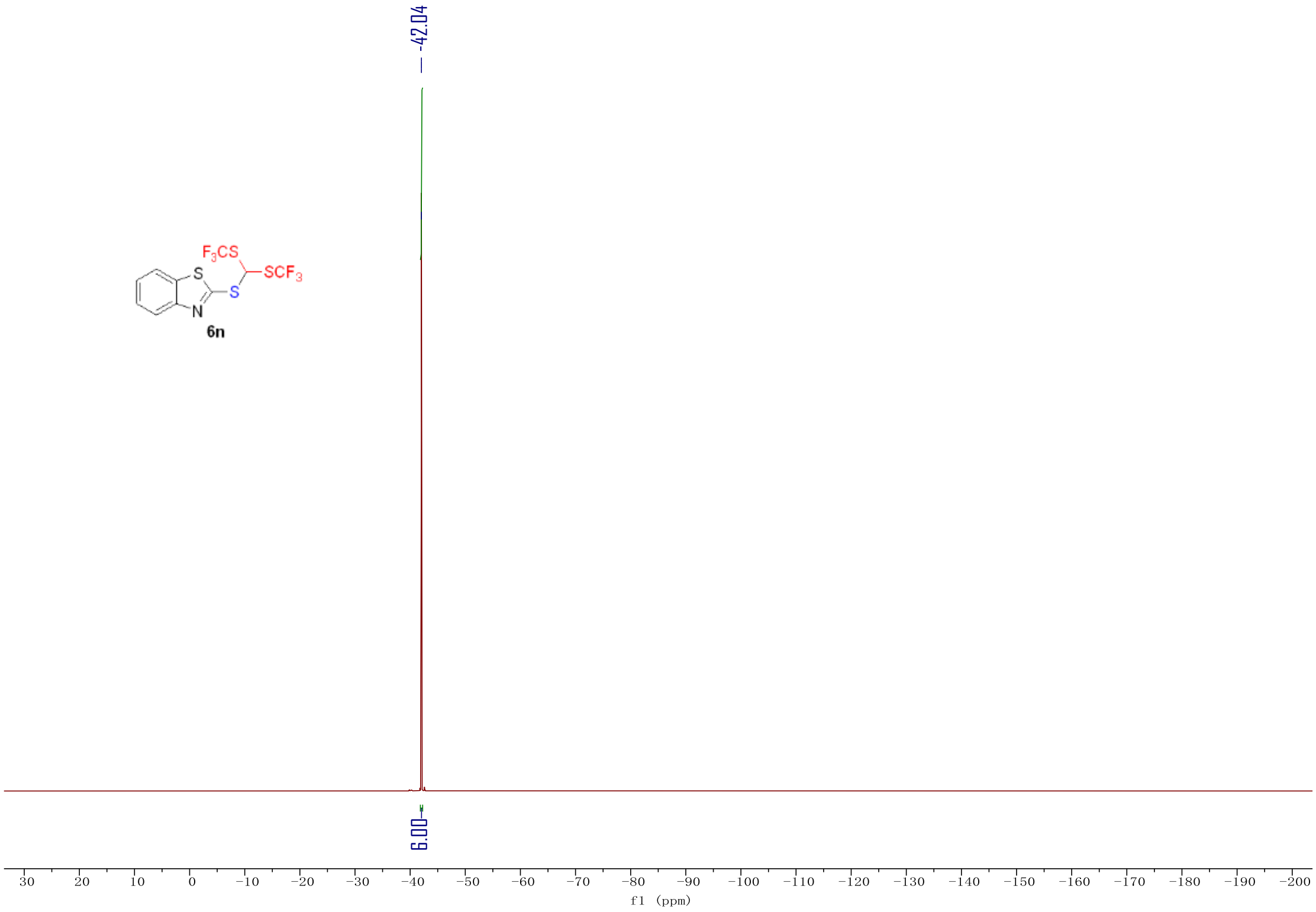

^{13}C NMR Spectrum of **6n** (101 MHz, $CDCl_3$)

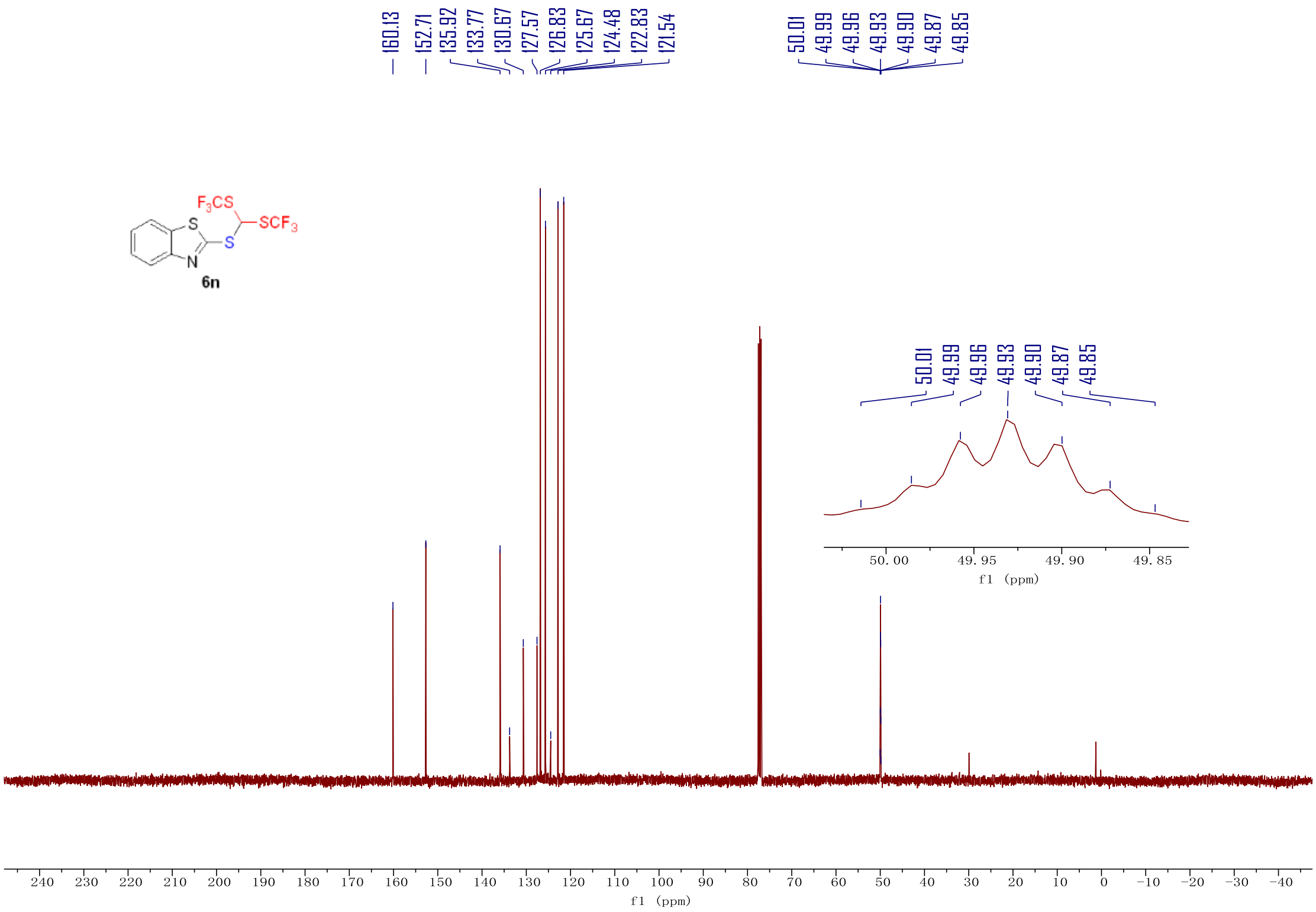

^{1}H NMR Spectrum of **6o** (400 MHz, $CDCl_3$)

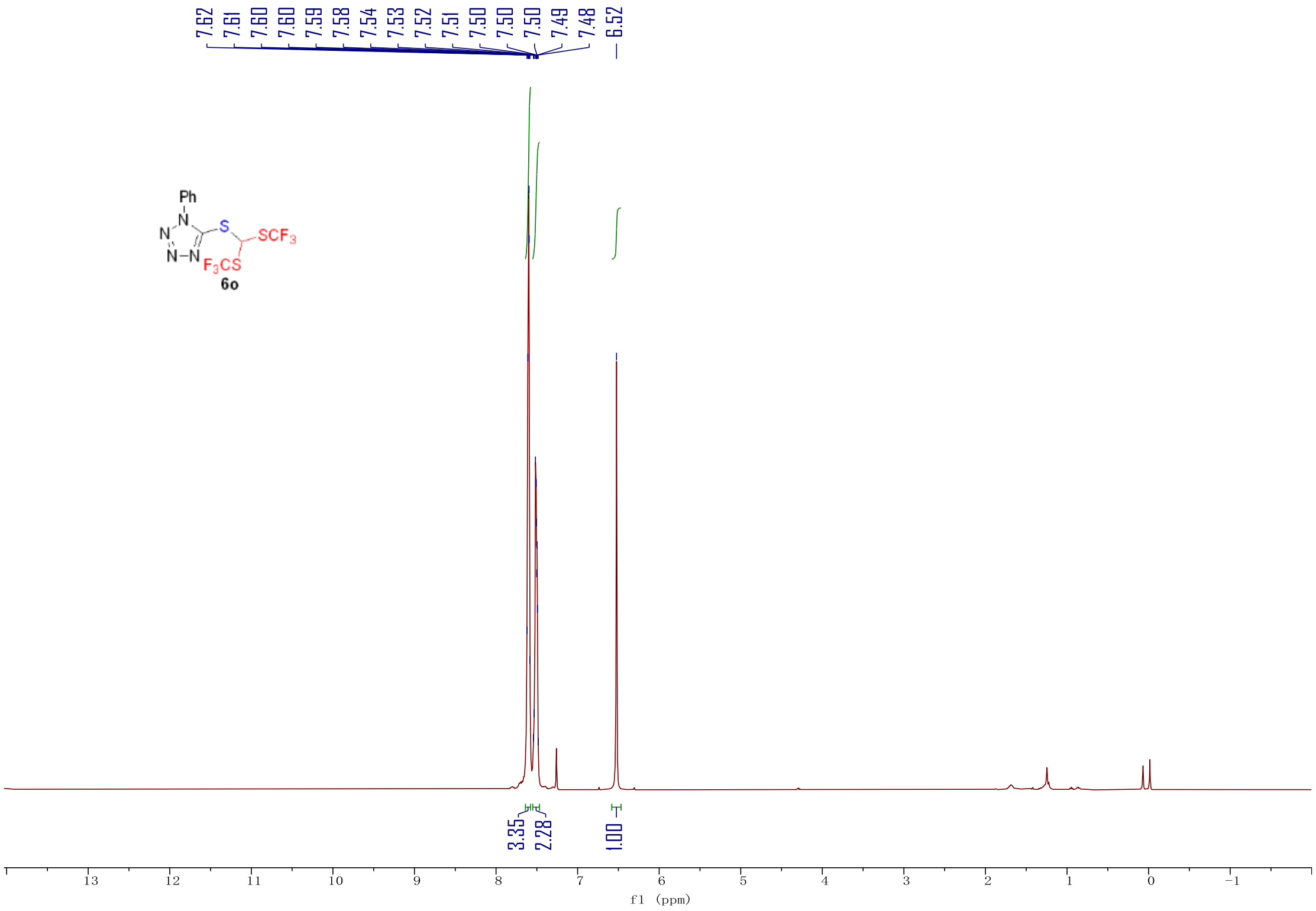

^{19}F NMR Spectrum of **6o** (376 MHz, $CDCl_3$)

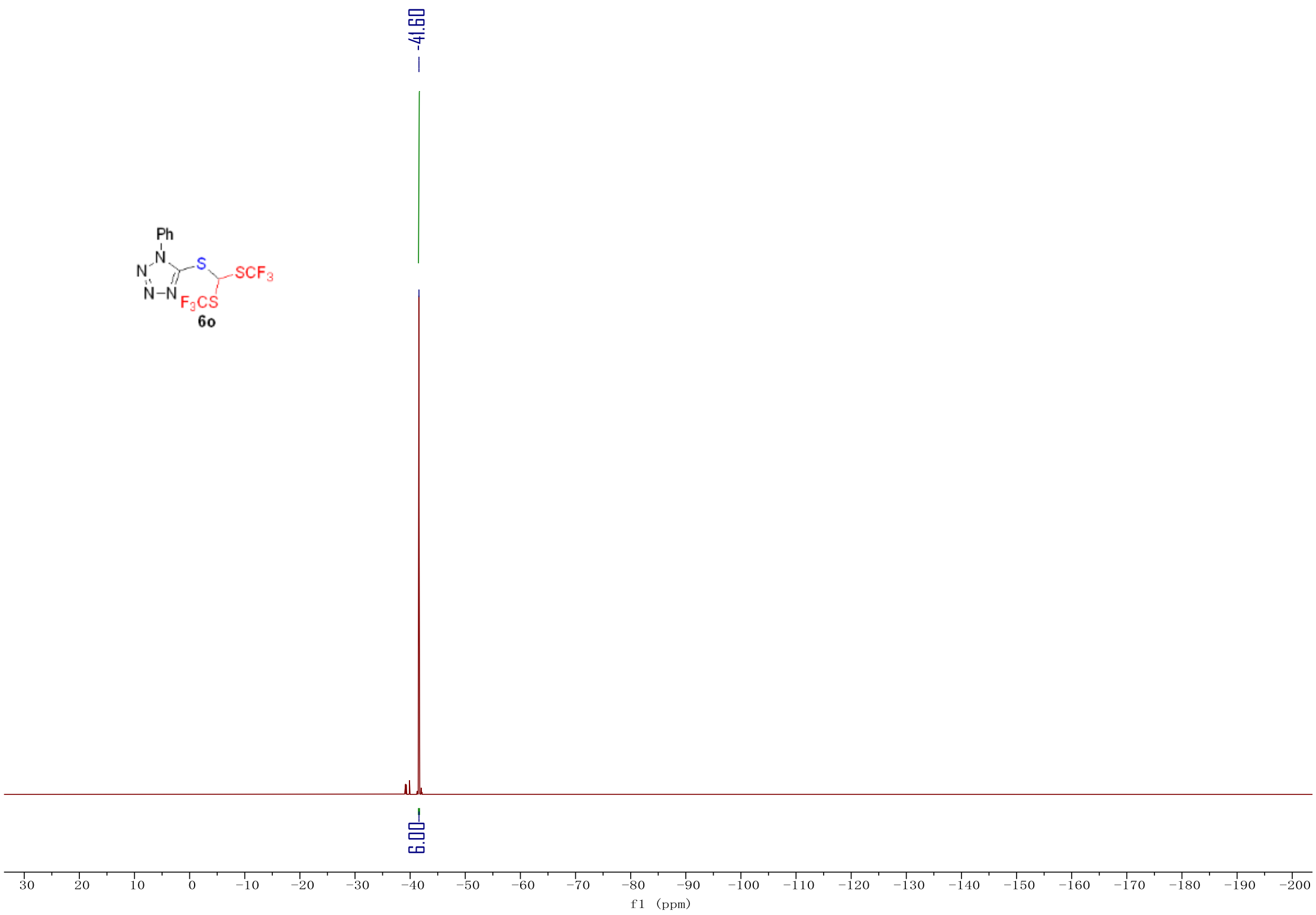

¹³C NMR Spectrum of **6o** (101 MHz, $CDCl_3$)

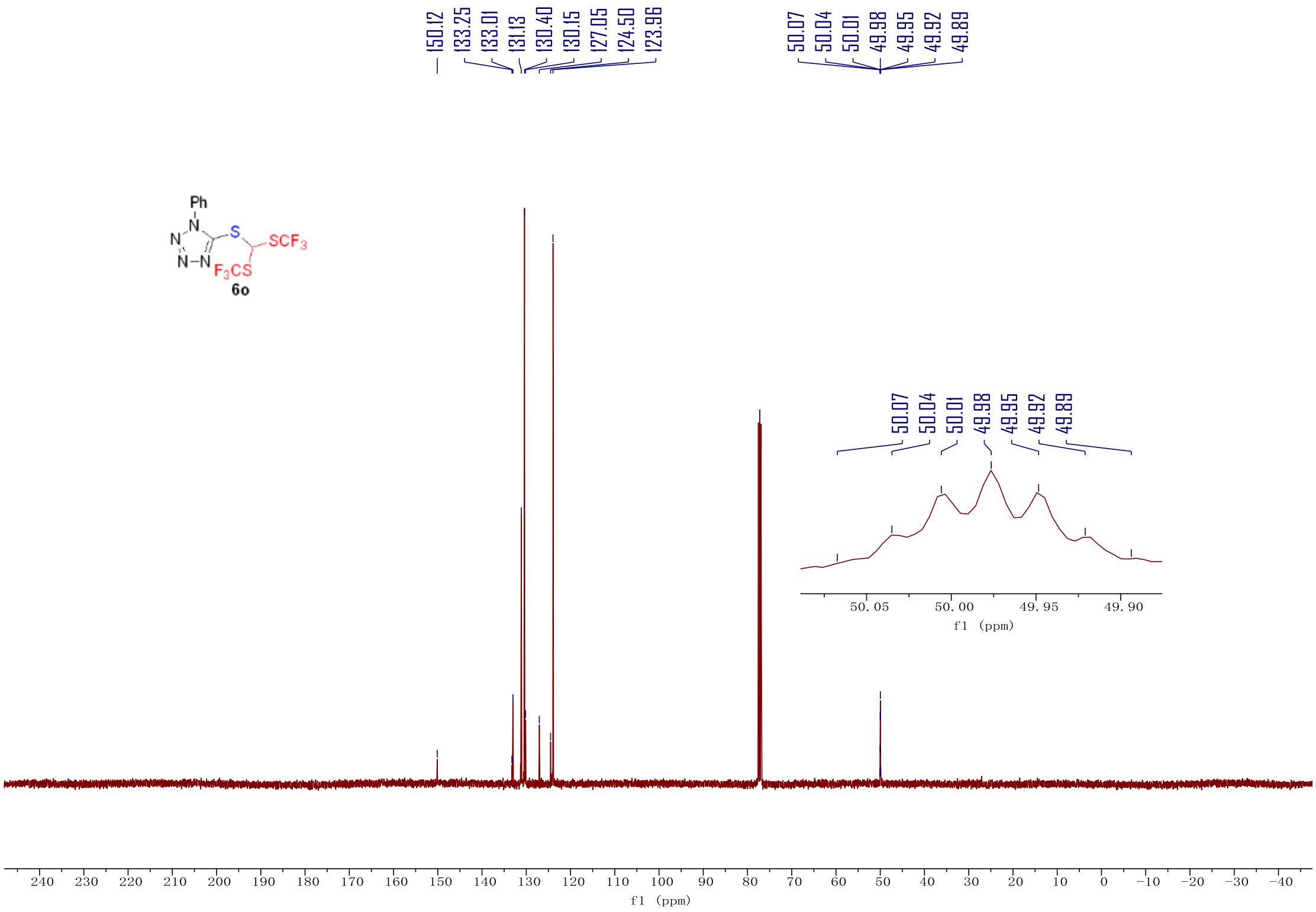

¹H NMR Spectrum of **6p** (400 MHz, $CDCl_3$)

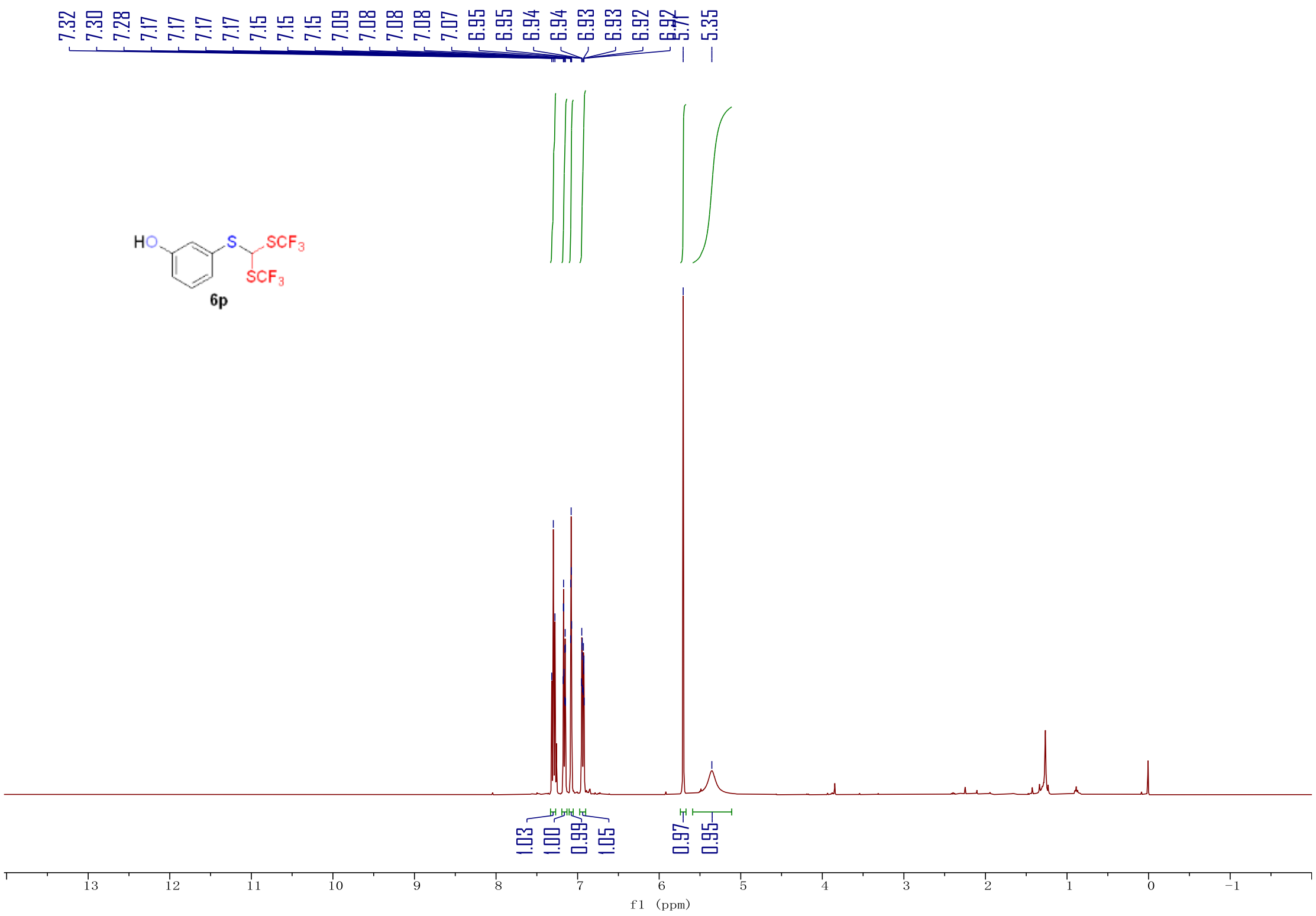

^{19}F NMR Spectrum of **6p** (376 MHz, $CDCl_3$)

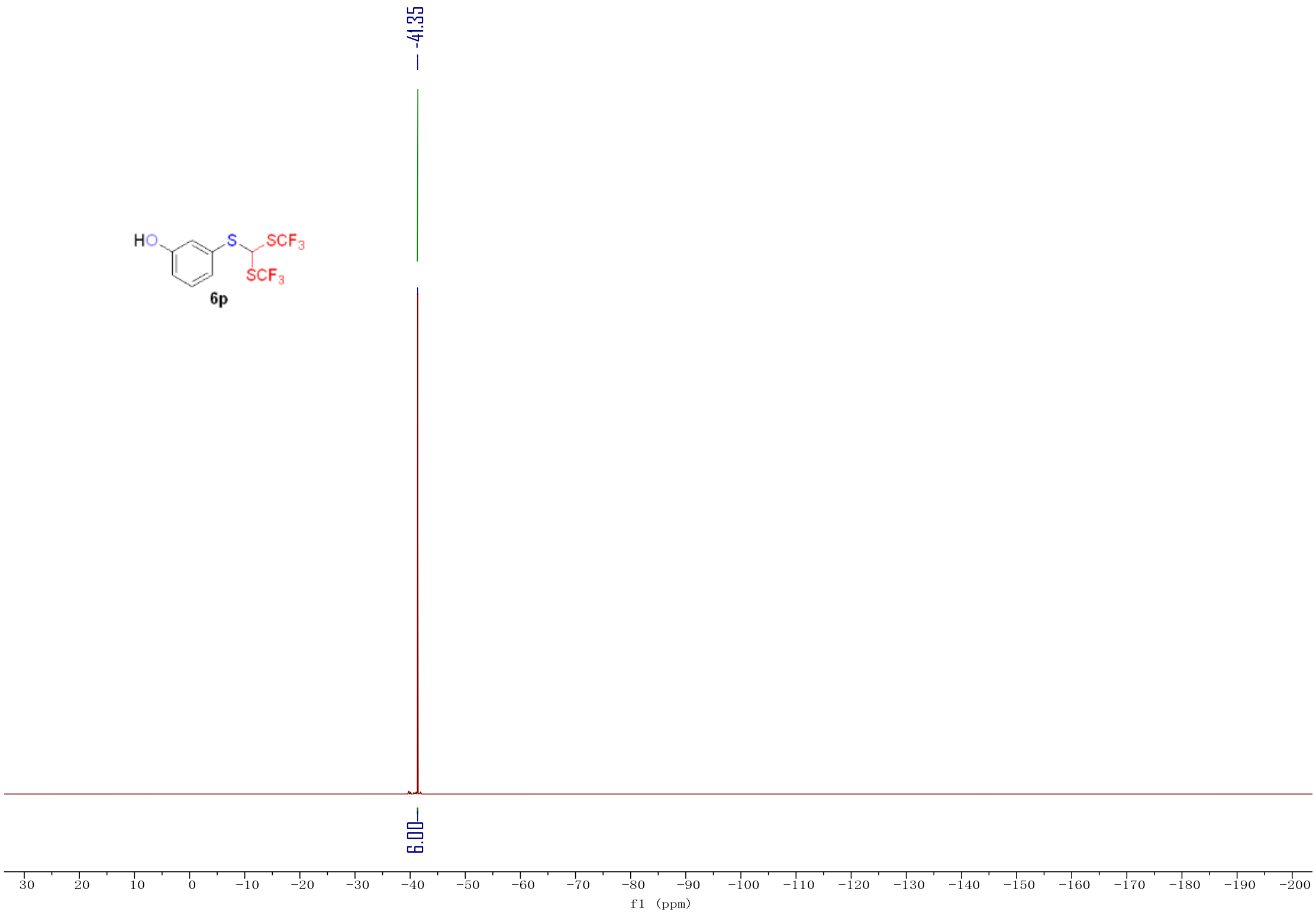

^{13}C NMR Spectrum of **6p** (101 MHz, $CDCl_3$)

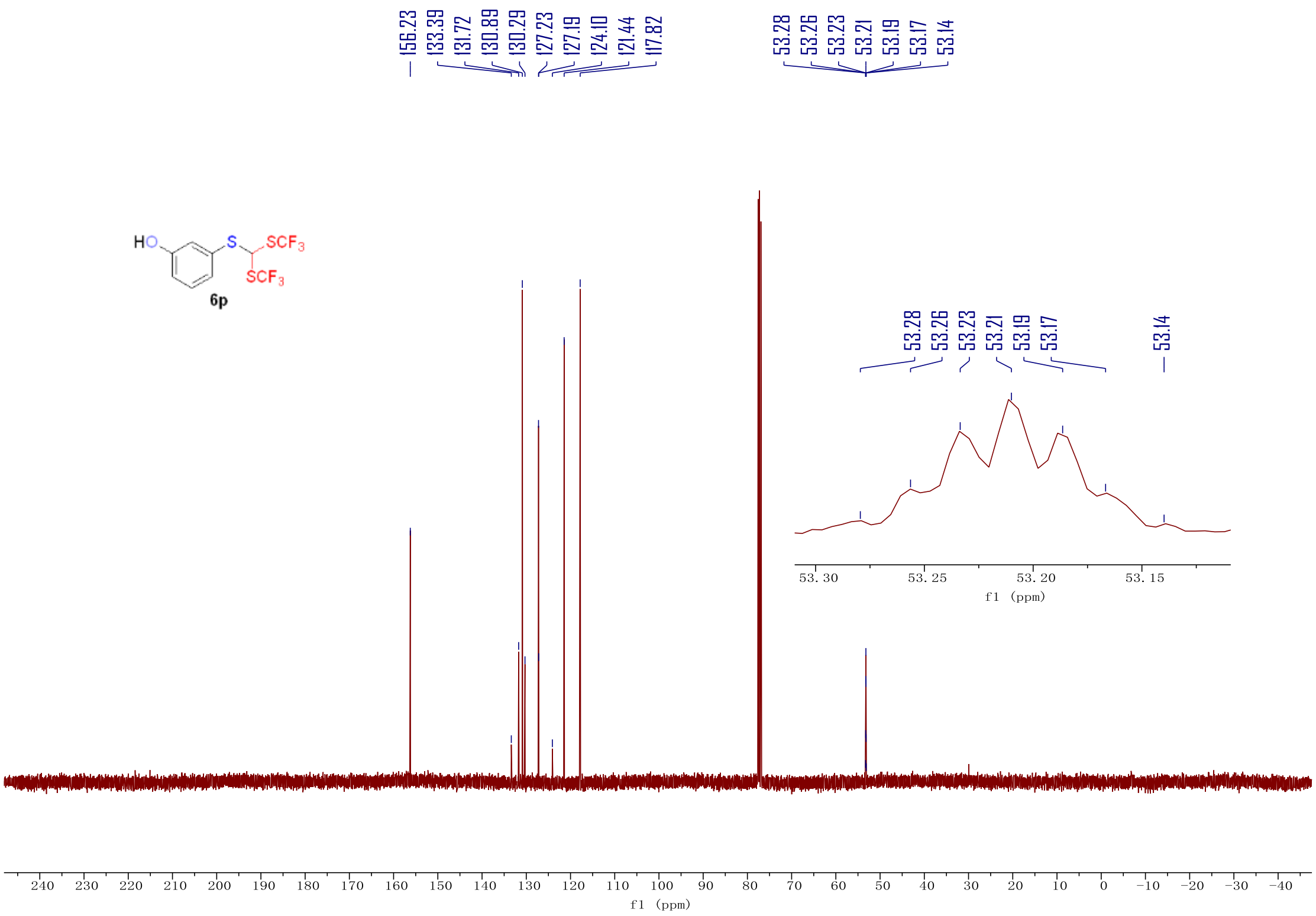

^{1}H NMR Spectrum of **6p′** (400 MHz, $CDCl_3$)

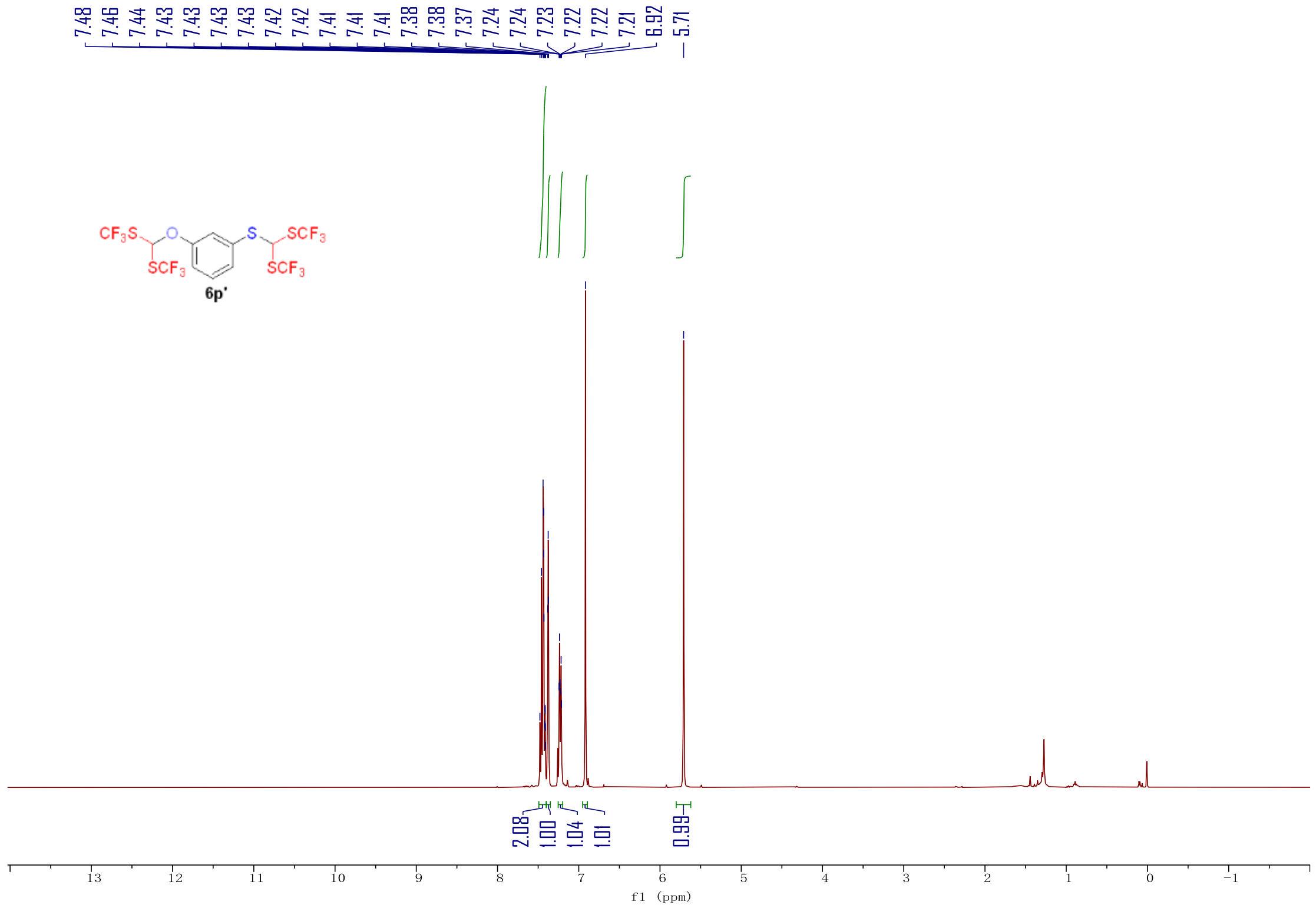

^{19}F NMR Spectrum of **6p′** (376 MHz, $CDCl_3$)

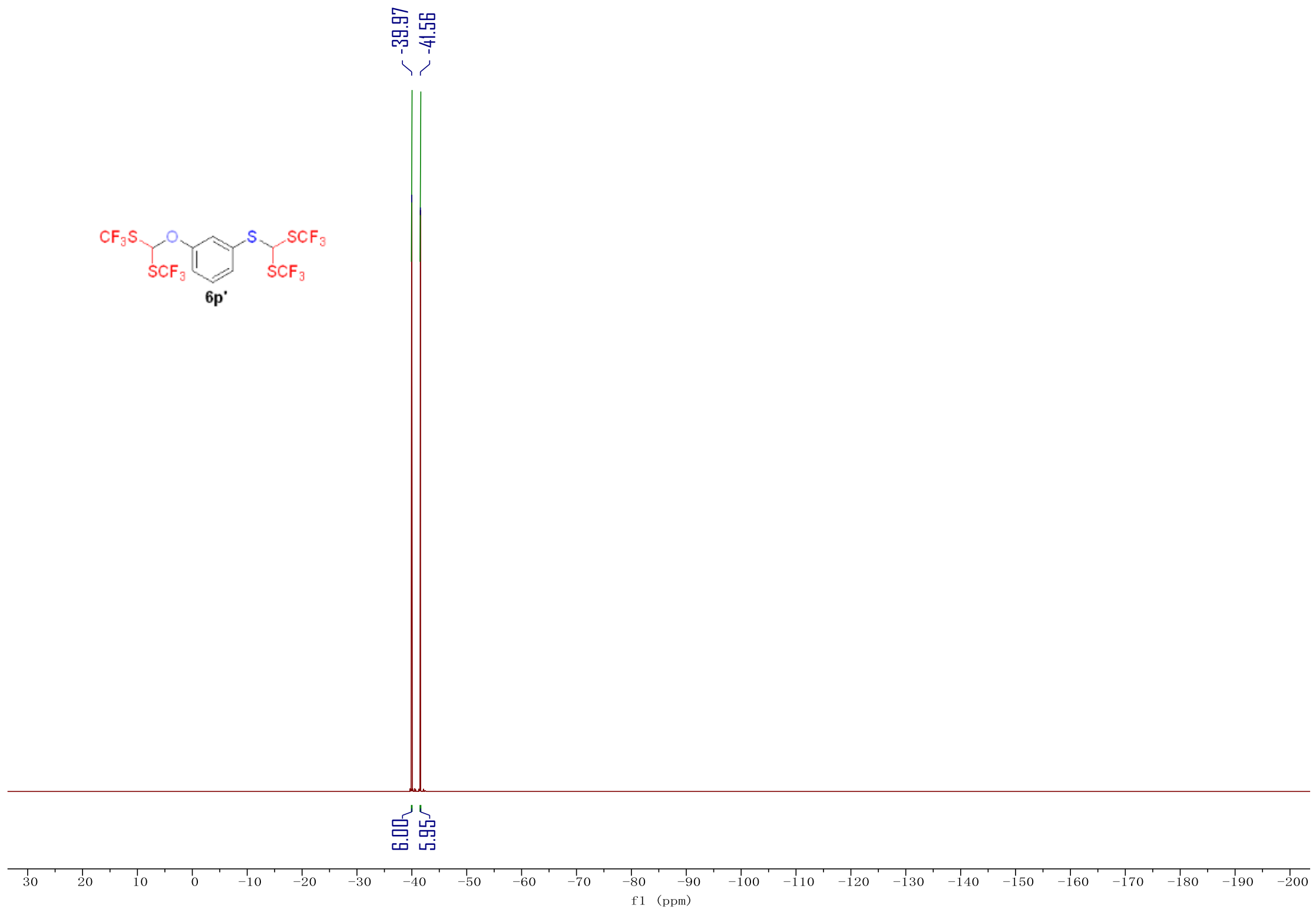

^{13}C NMR Spectrum of **6p′** (101 MHz, $CDCl_3$)

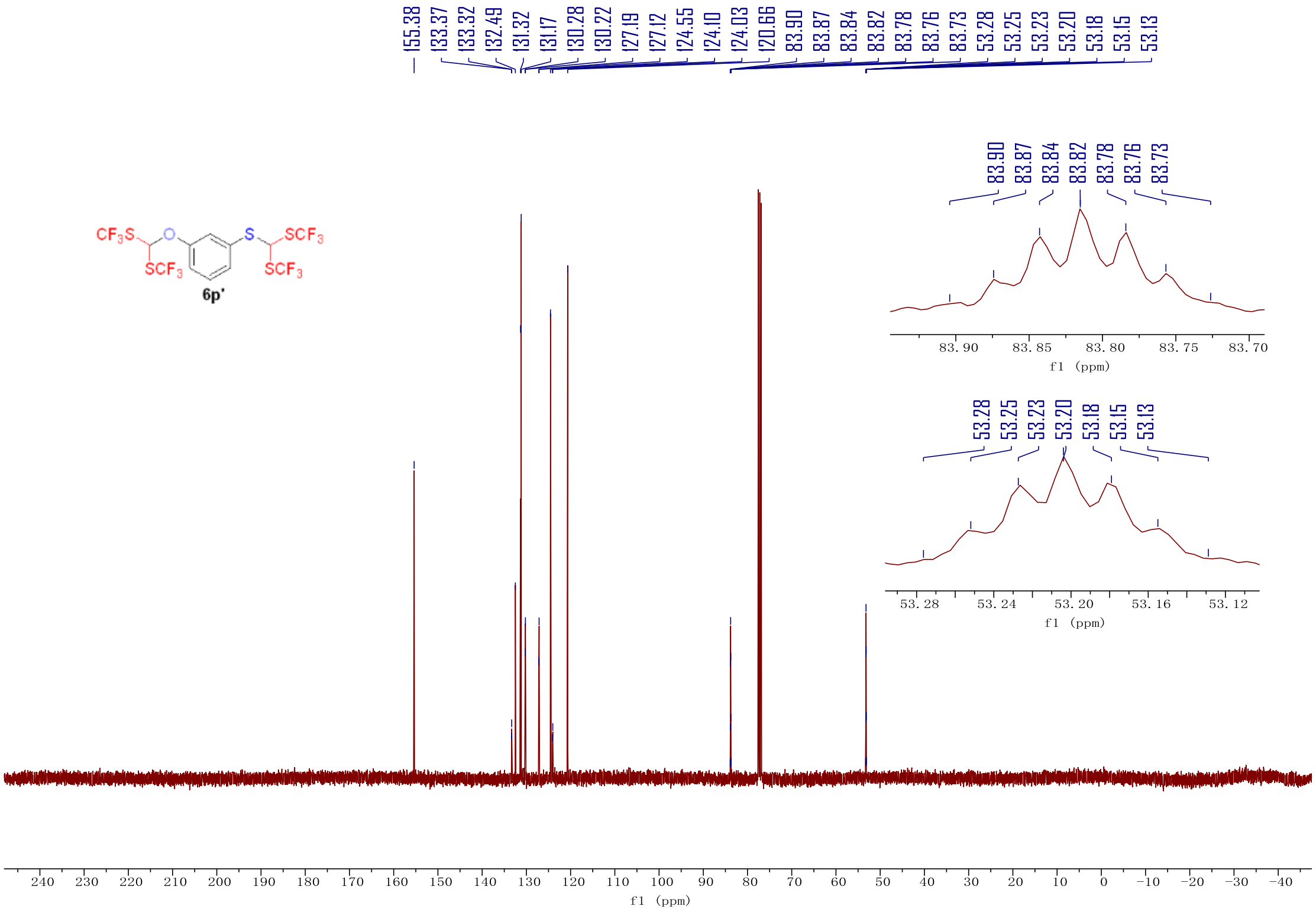

^{1}H NMR Spectrum of **6q** (400 MHz, $CDCl_3$)

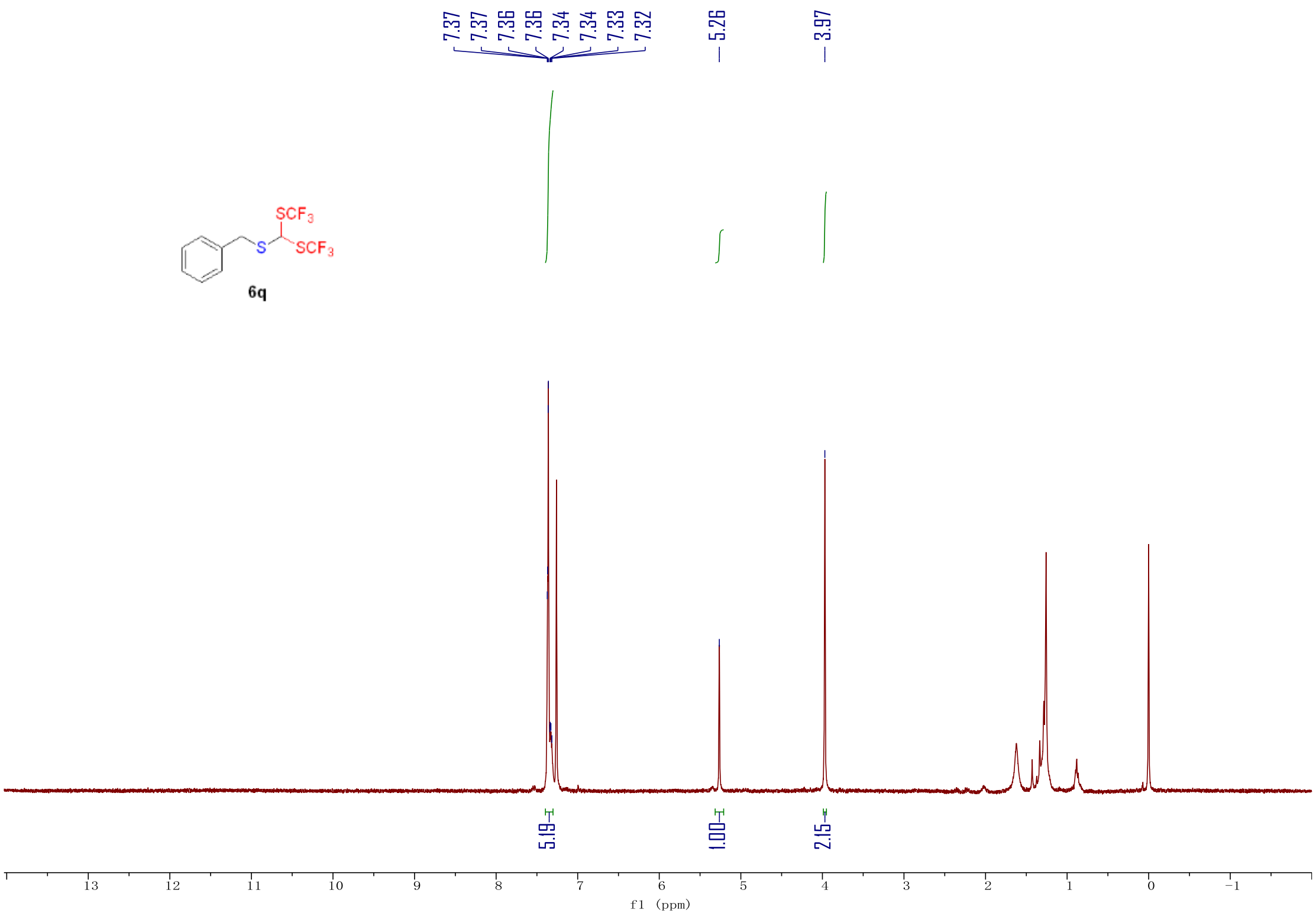

^{19}F **NMR** Spectrum of **6q** (376 MHz, $CDCl_3$)

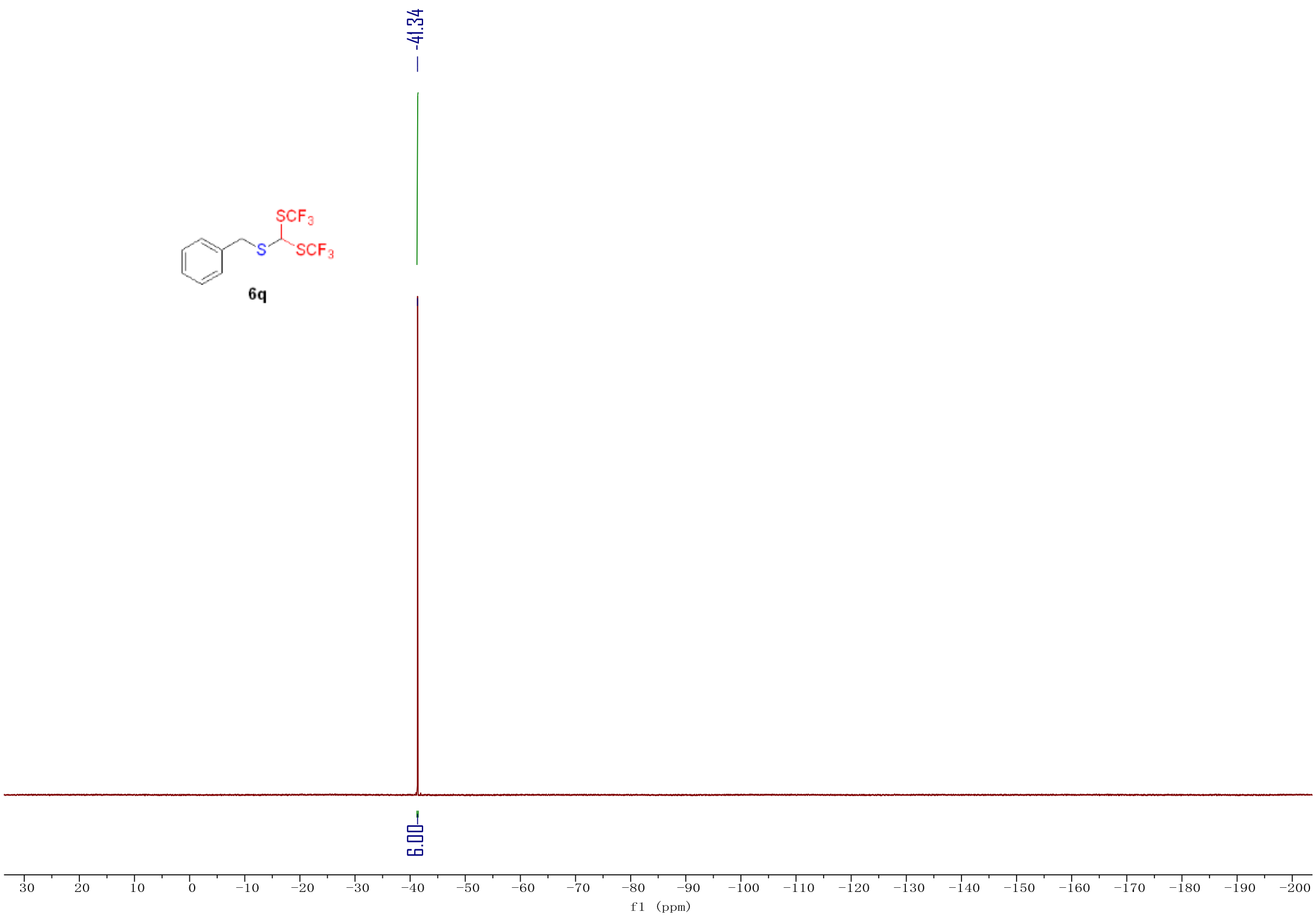

^{13}C **NMR** Spectrum of **6q** (101 MHz, $CDCl_3$)

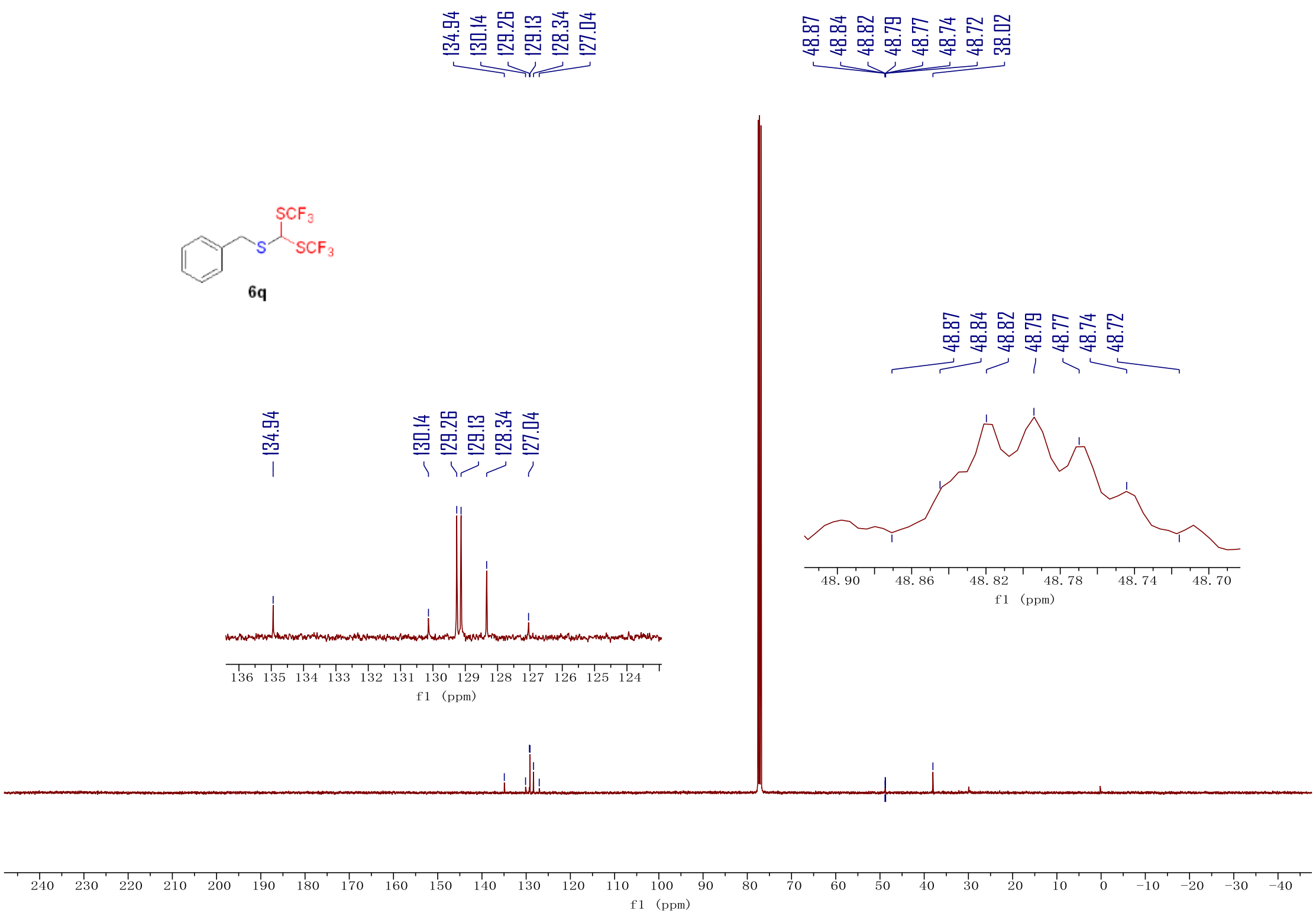

^{1}H NMR Spectrum of **6r** (400 MHz, $CDCl_3$)

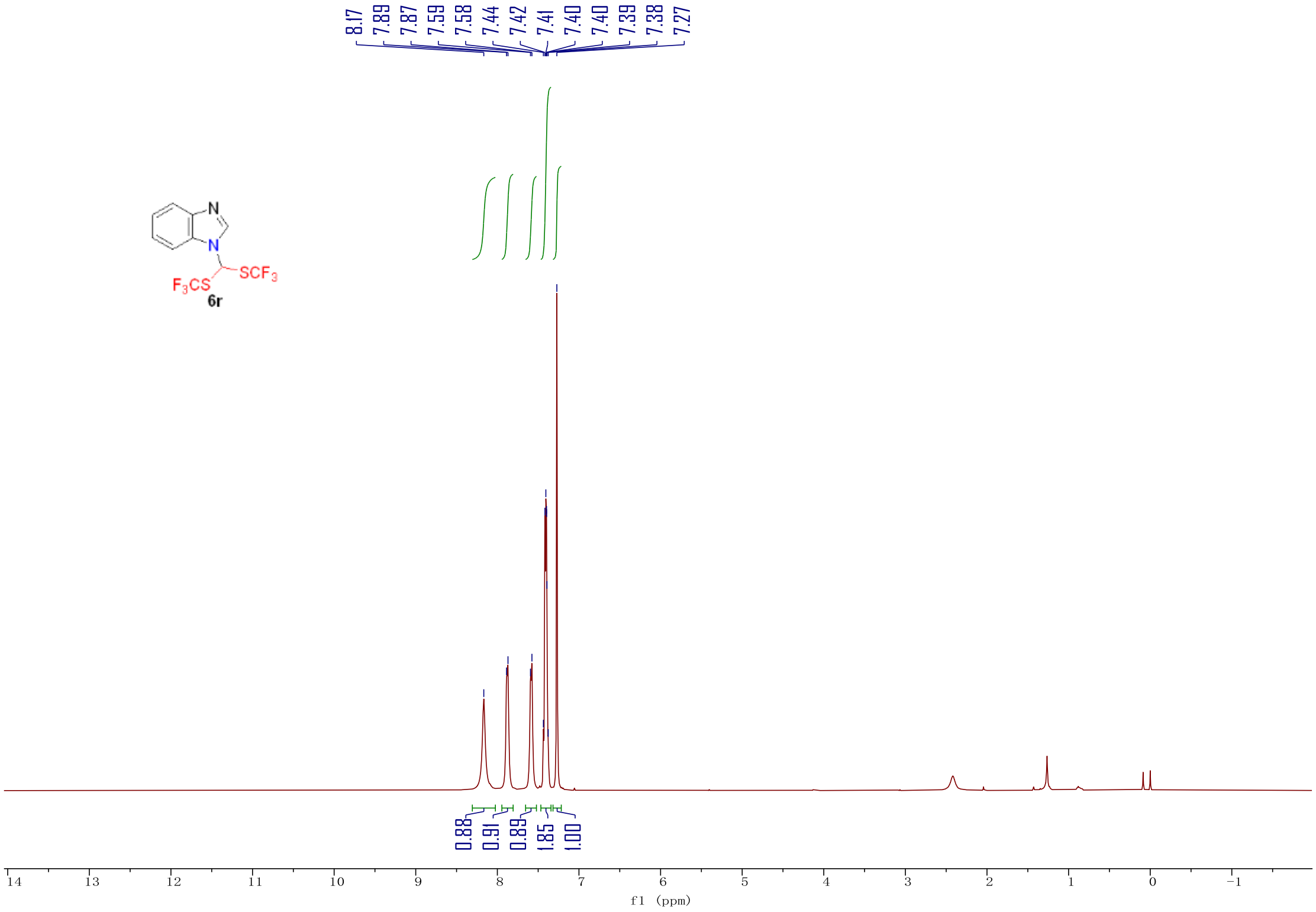

^{19}F NMR Spectrum of **6r** (376 MHz, $CDCl_3$)

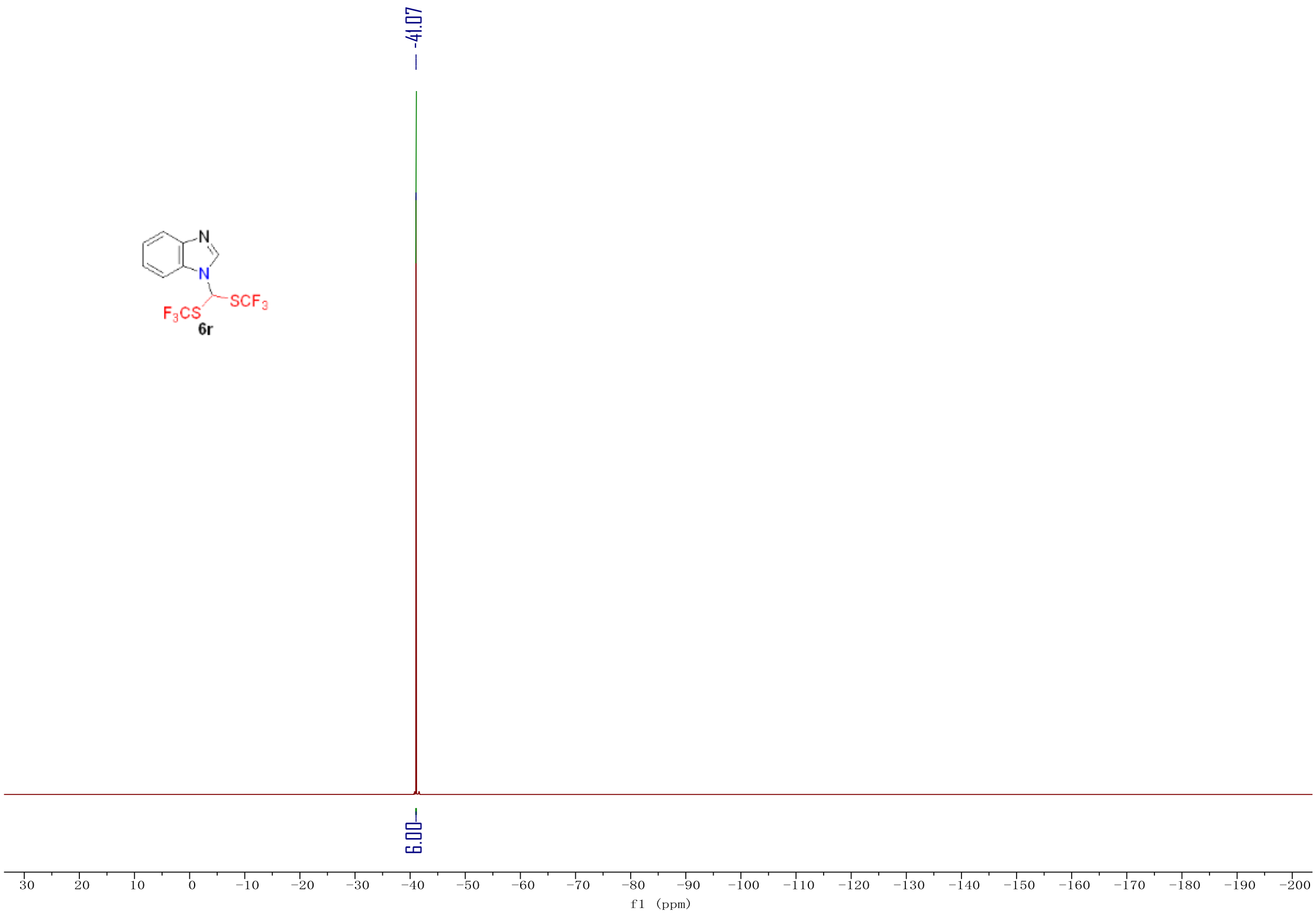

^{13}C NMR Spectrum of **6r** (101 MHz, $CDCl_3$)

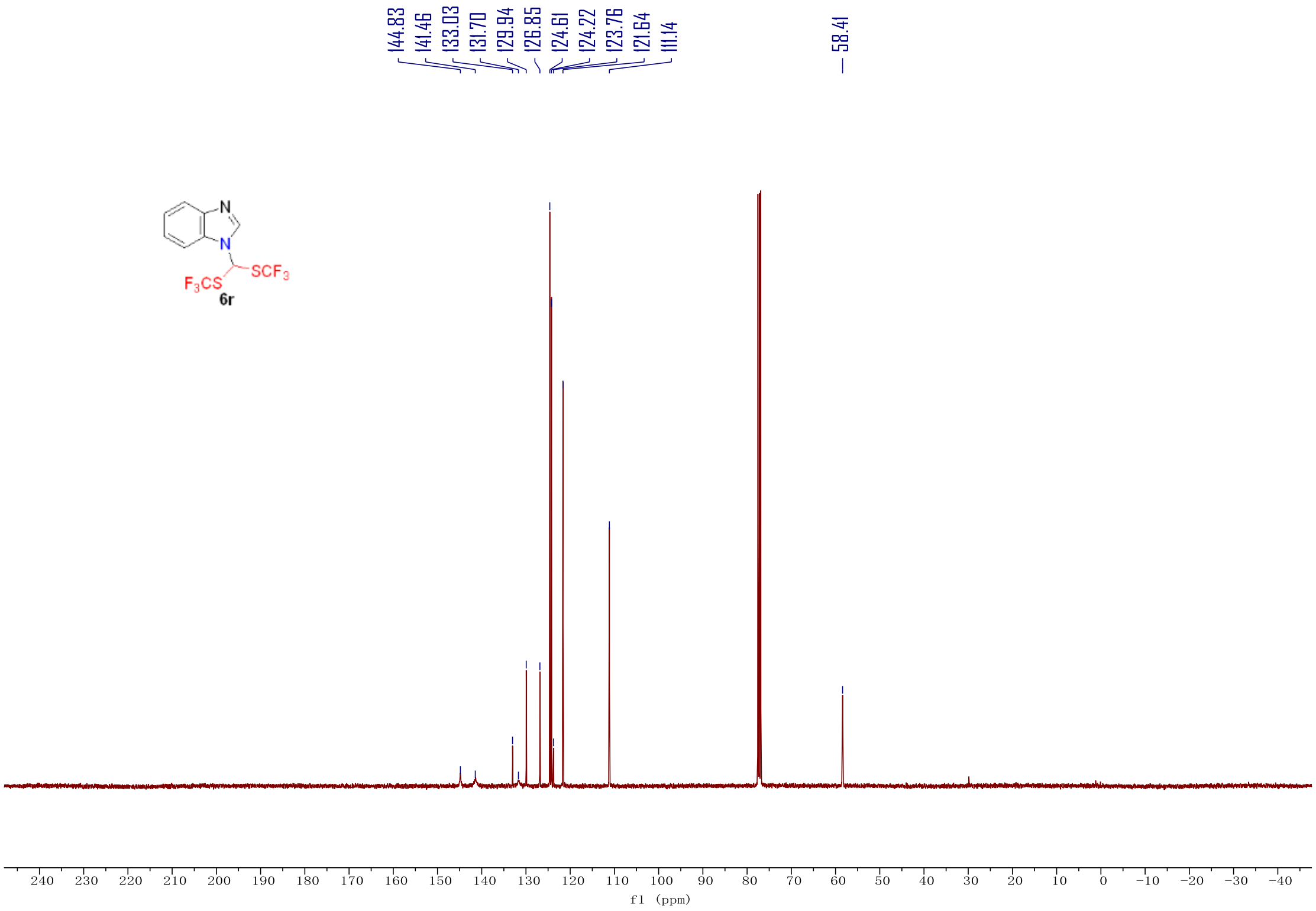

^{1}H NMR Spectrum of **6s** (400 MHz, $CDCl_3$)

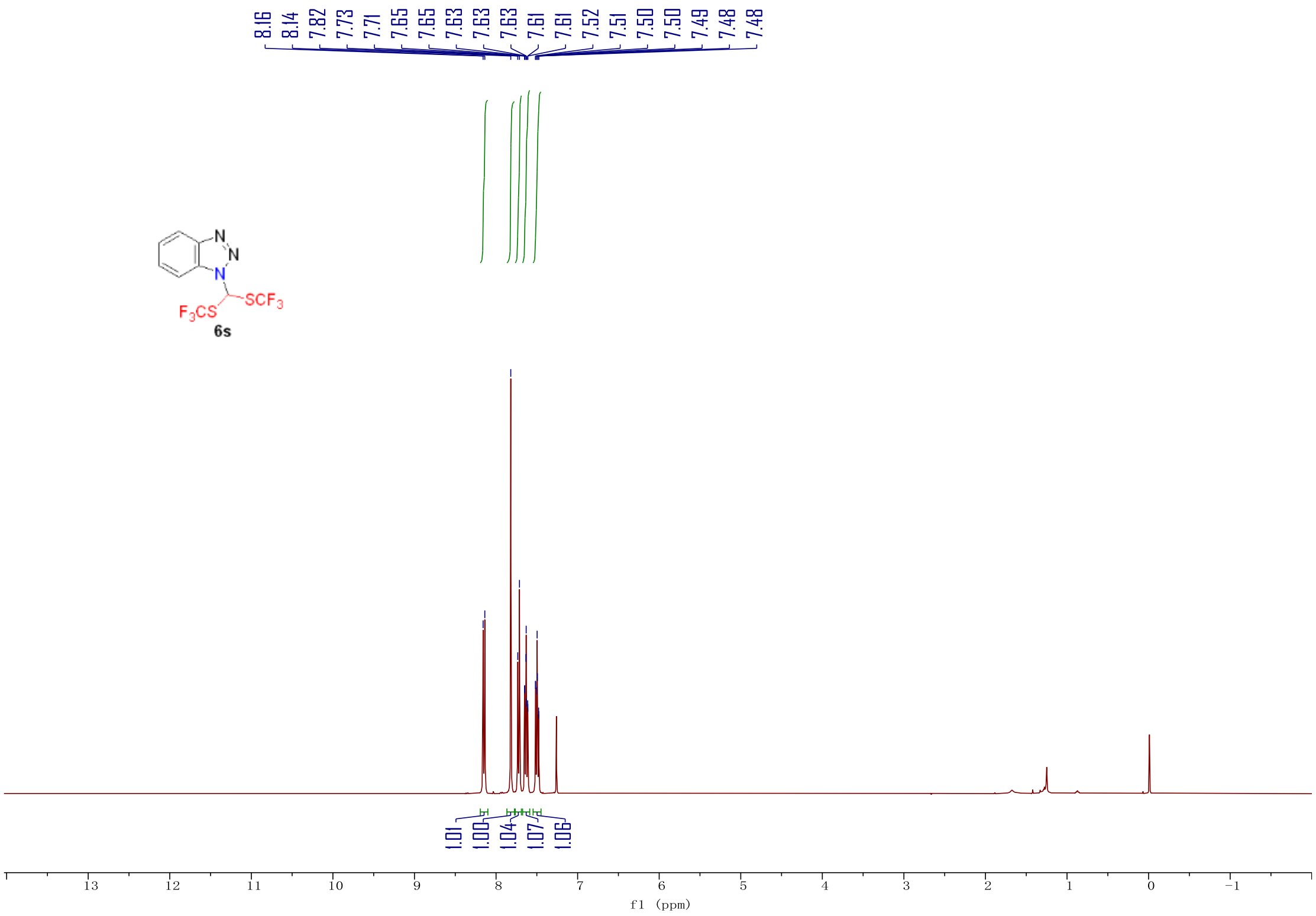

^{19}F NMR Spectrum of **6s** (376 MHz, $CDCl_3$)

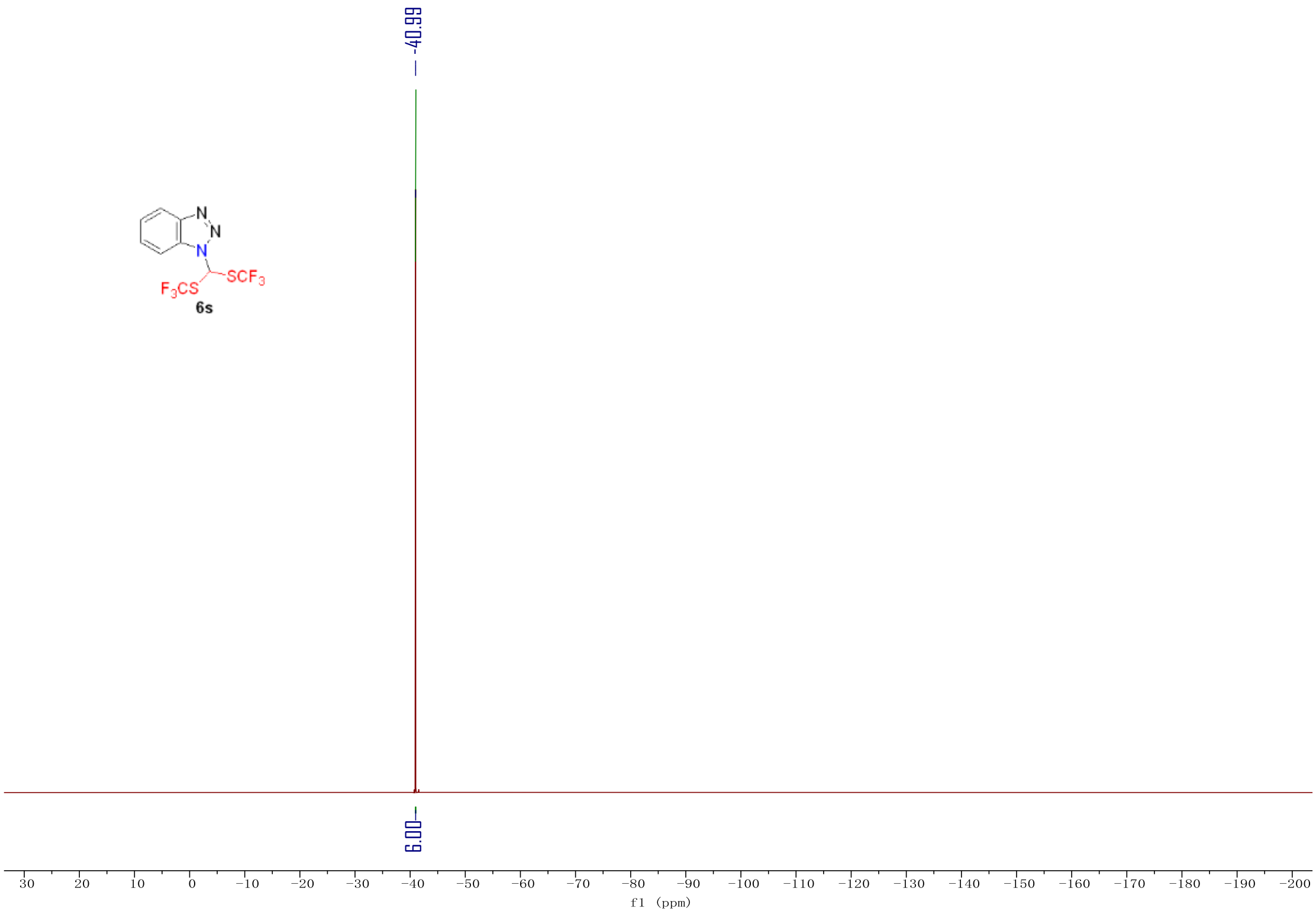

^{13}C NMR Spectrum of **6s** (101 MHz, $CDCl_3$)

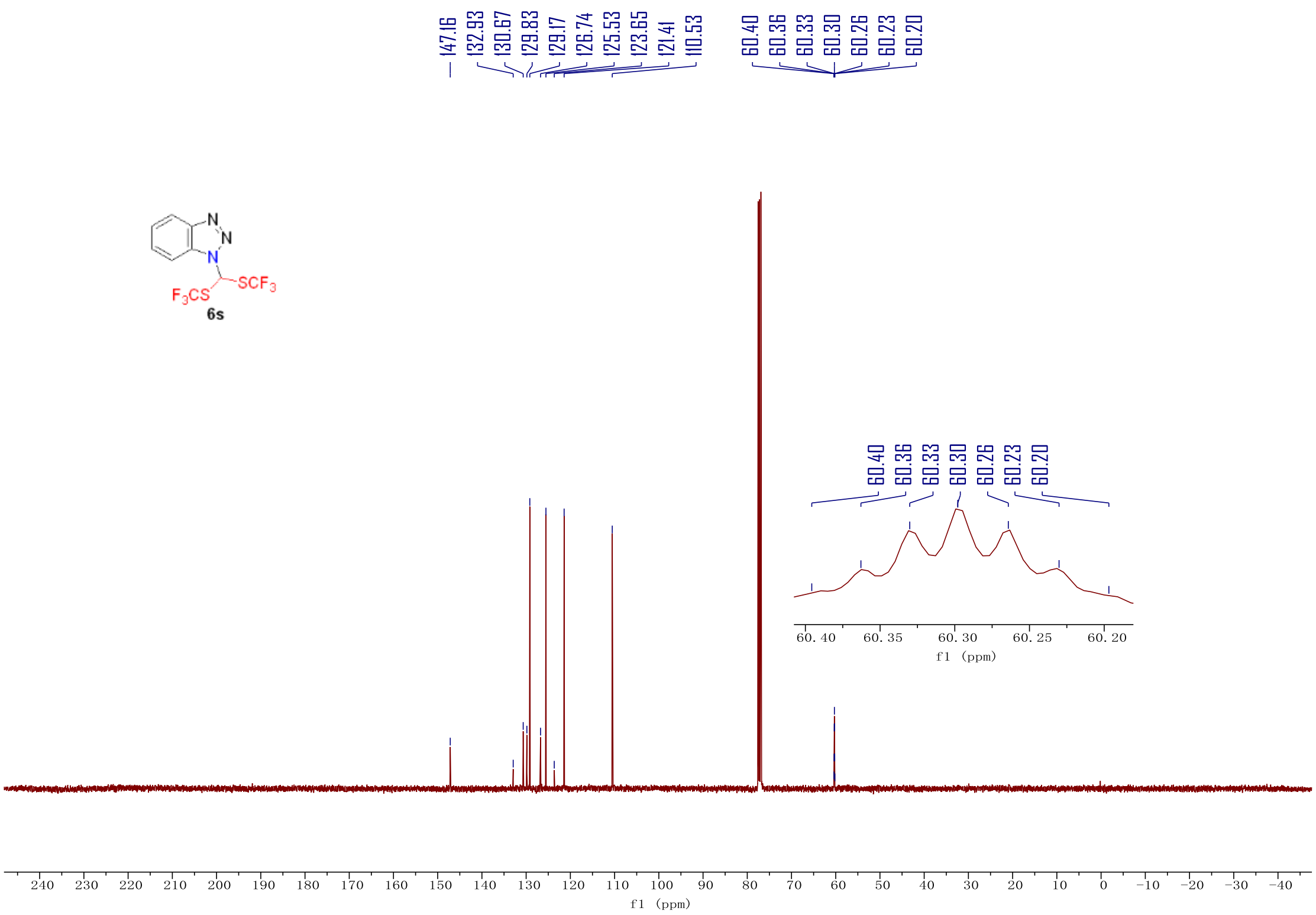

^{1}H NMR Spectrum of **6t** (400 MHz, $CDCl_3$)

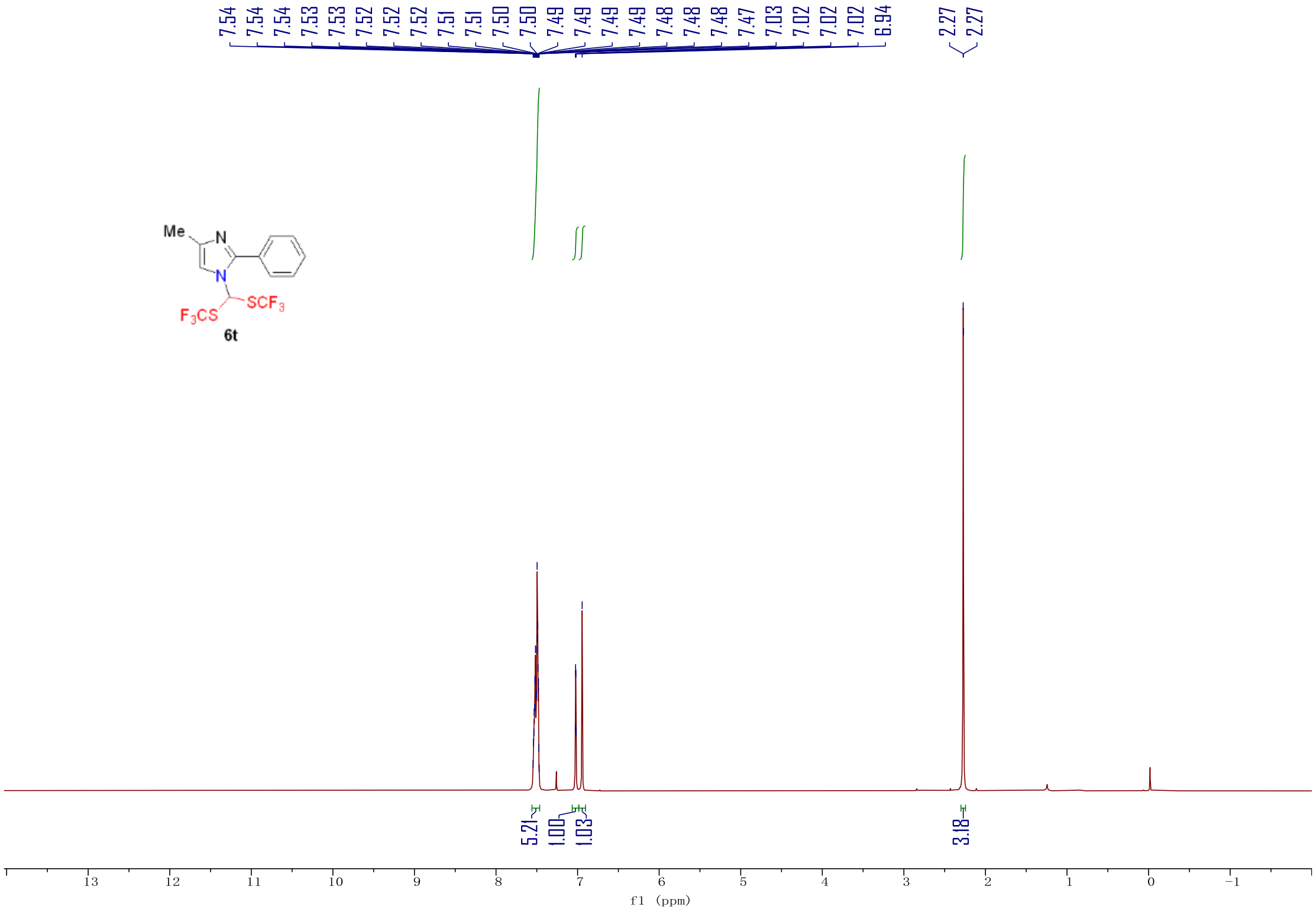

^{19}F NMR Spectrum of **6t** (376 MHz, $CDCl_3$)

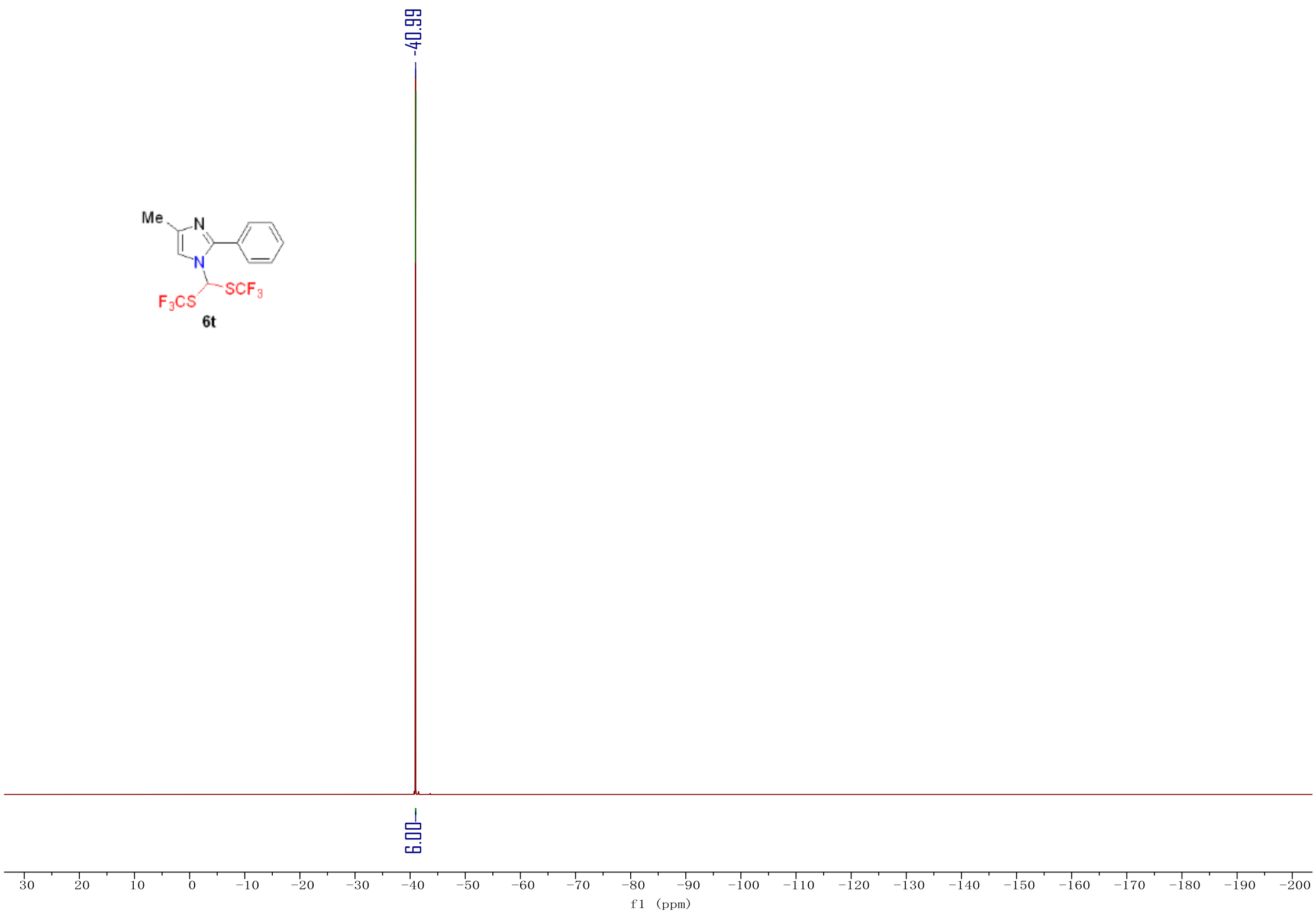

^{13}C NMR Spectrum of **6t** (101 MHz, $CDCl_3$)

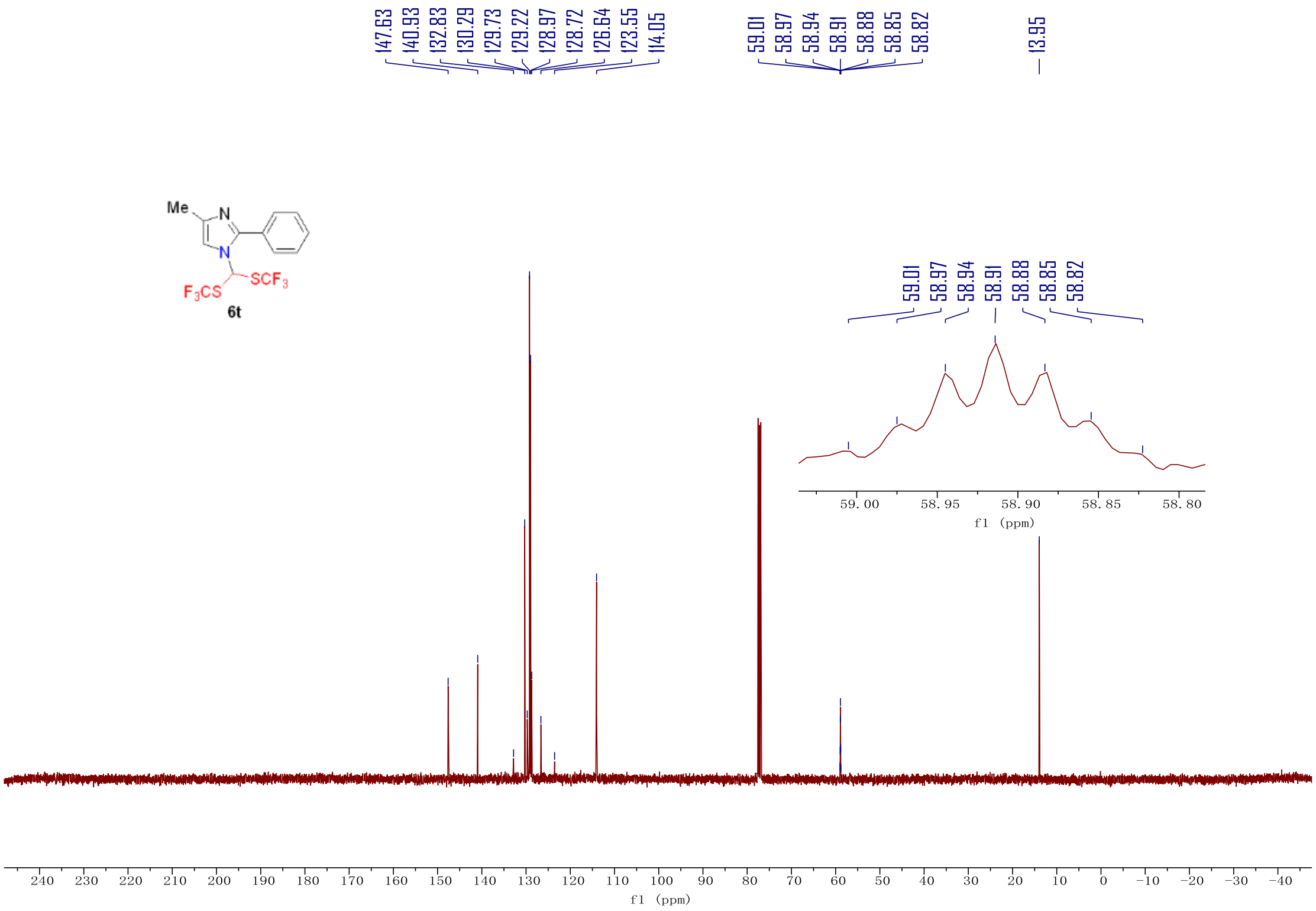

^{1}H NMR Spectrum of **6u** (400 MHz, $CDCl_3$)

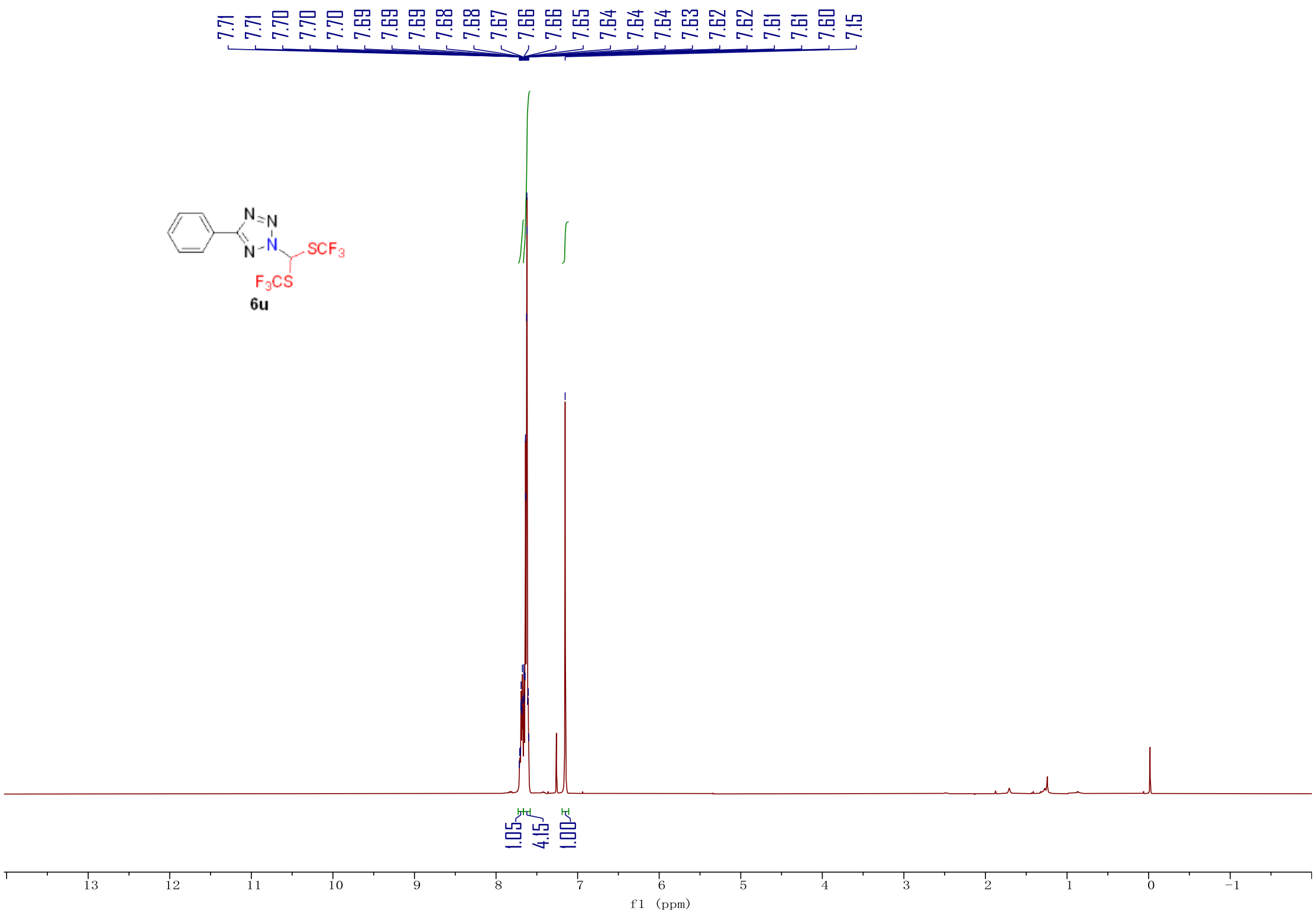

^{19}F NMR Spectrum of **6u** (376 MHz, $CDCl_3$)

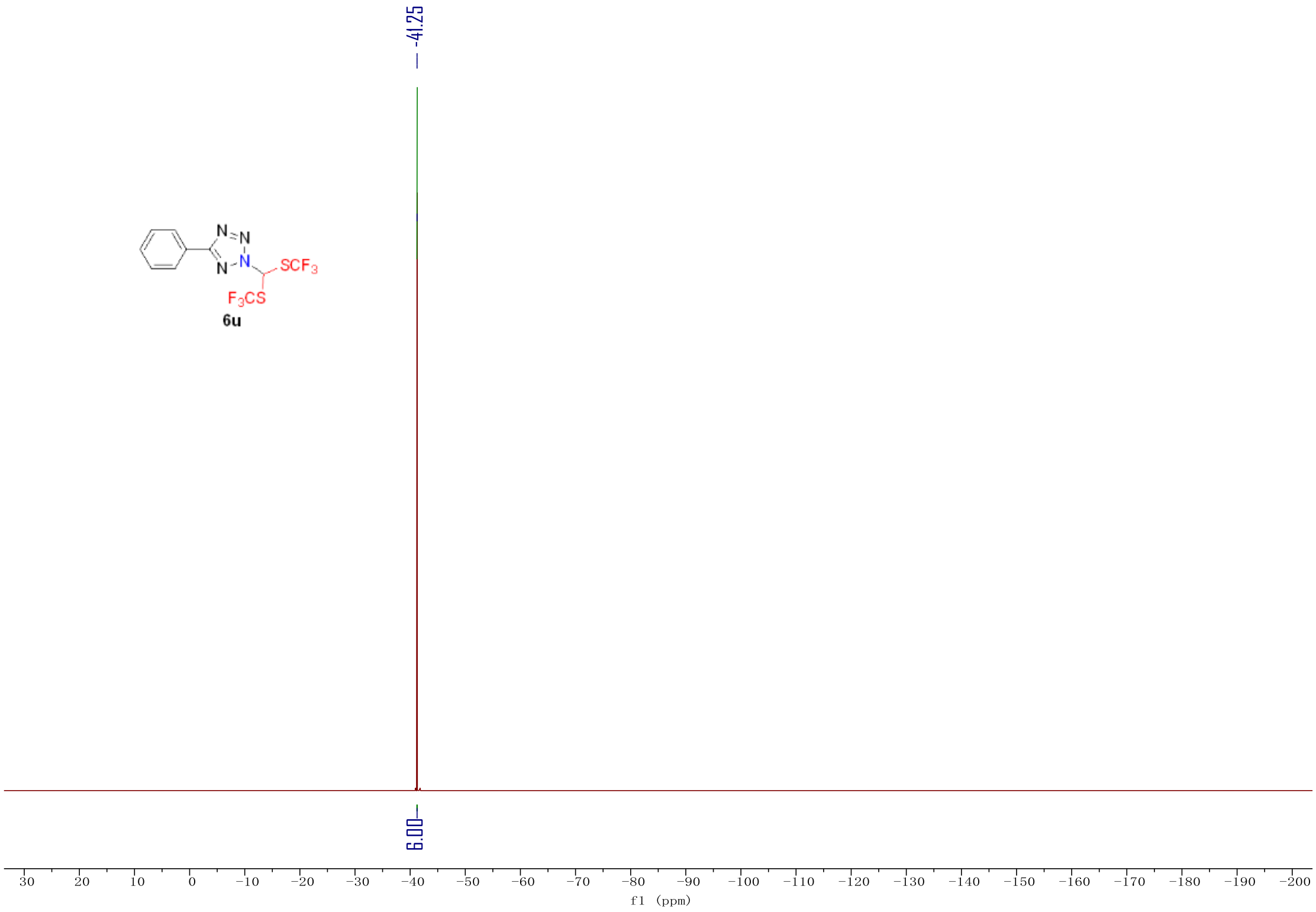

^{13}C NMR Spectrum of **6u** (101 MHz, $CDCl_3$)

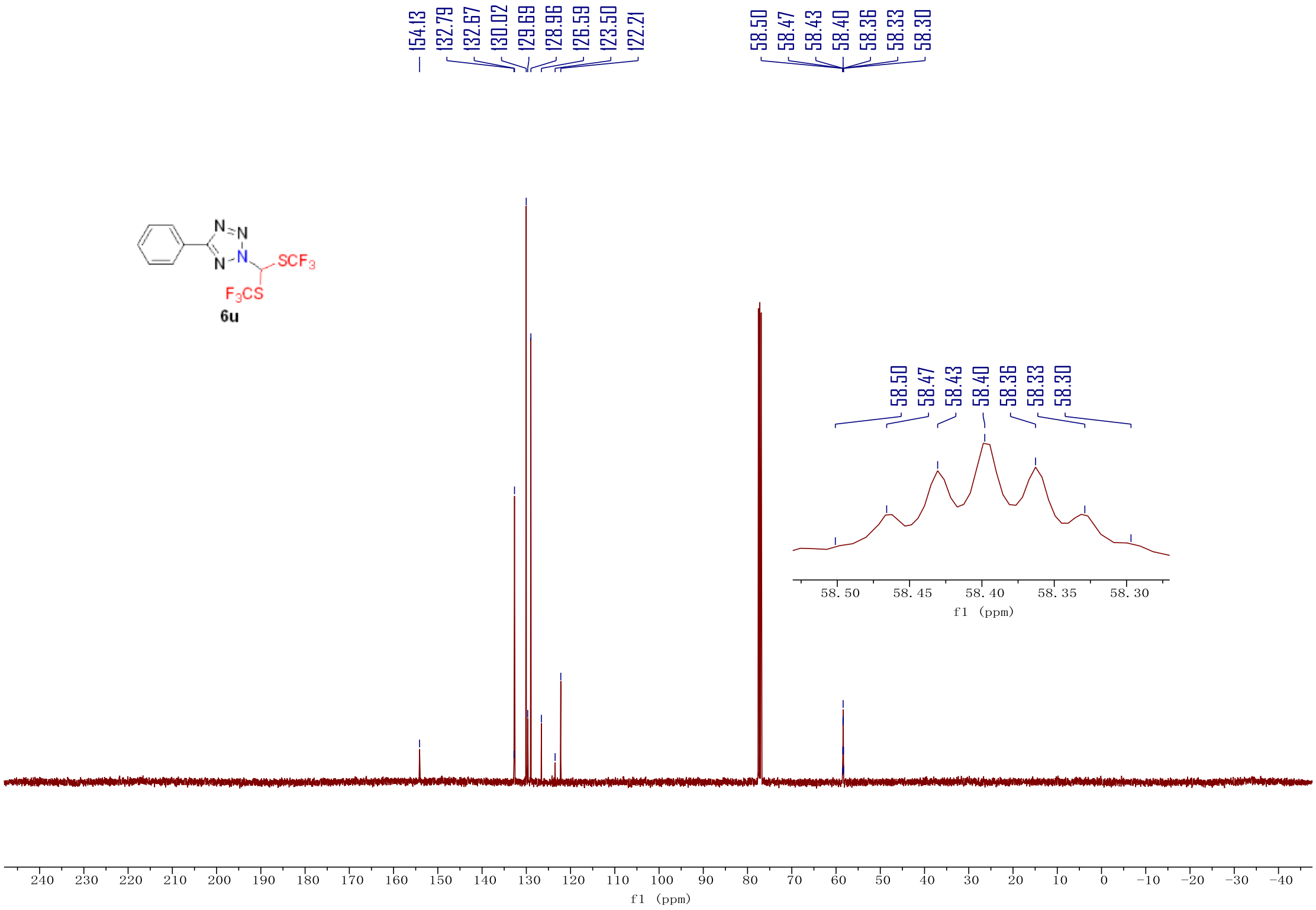

^{1}**H NMR** Spectrum of **6v** (400 MHz, $CDCl_3$)

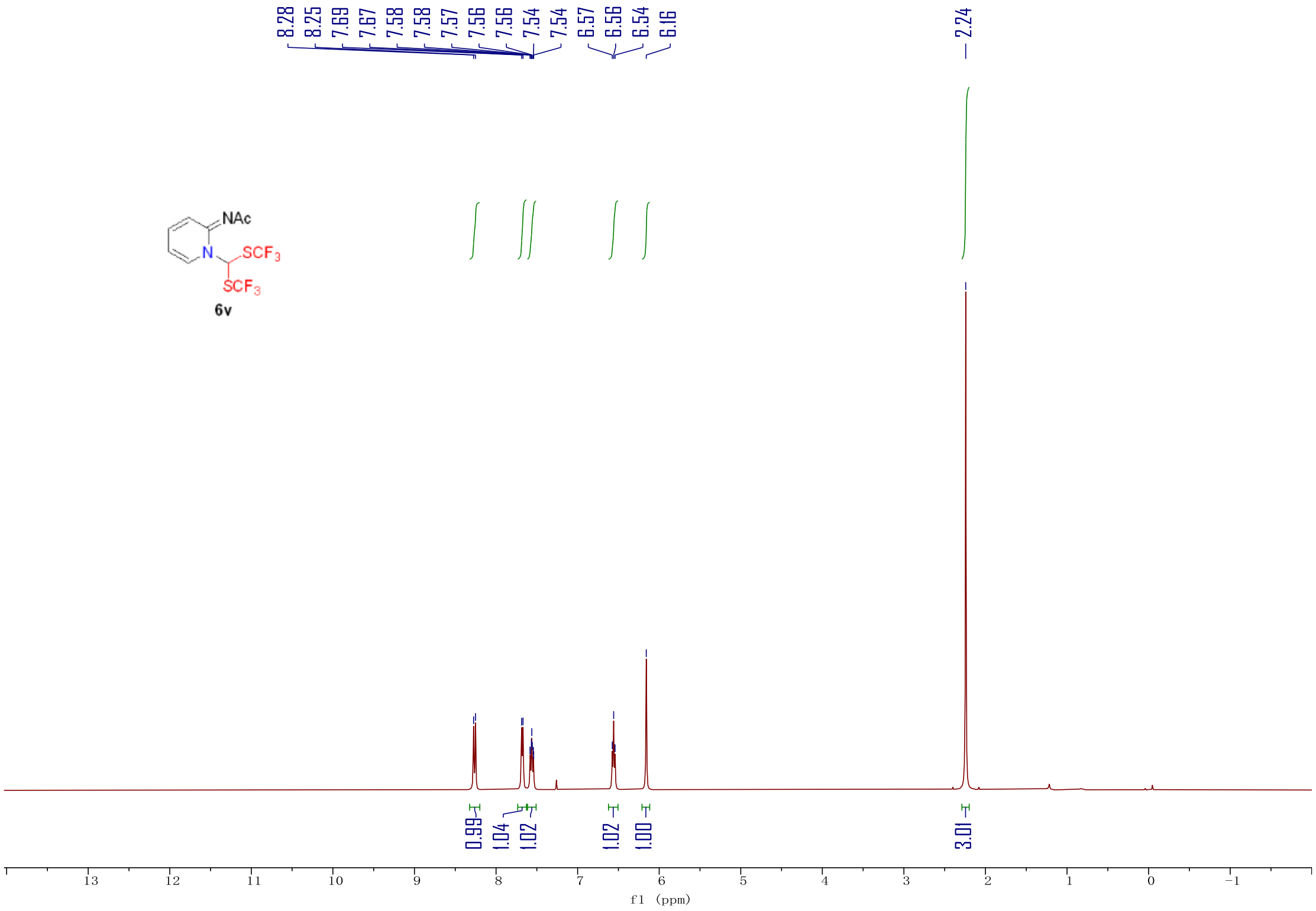

^{19}**F NMR** Spectrum of **6v** (376 MHz, $CDCl_3$)

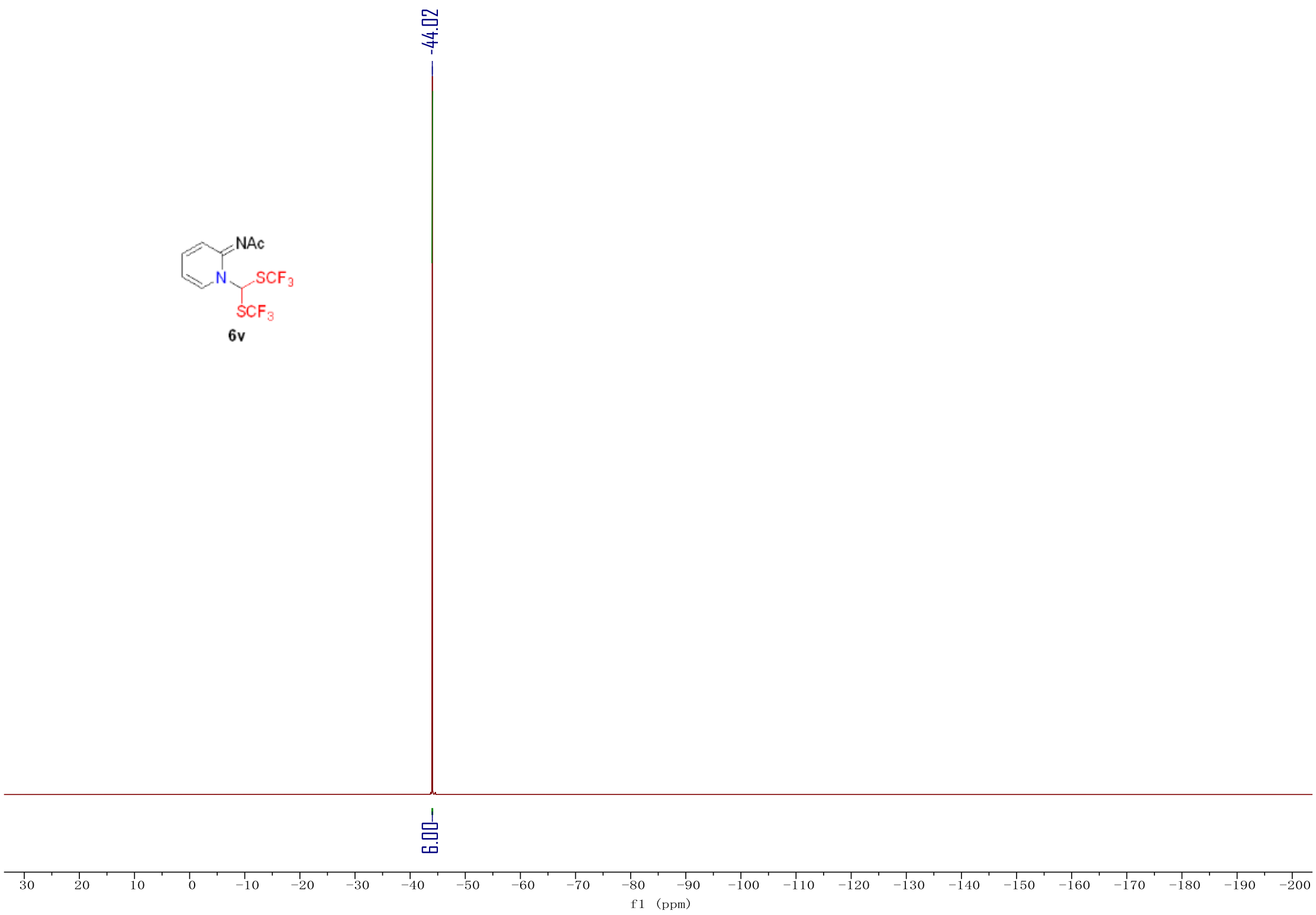

^{13}C NMR Spectrum of **6v** (101 MHz, $CDCl_3$)

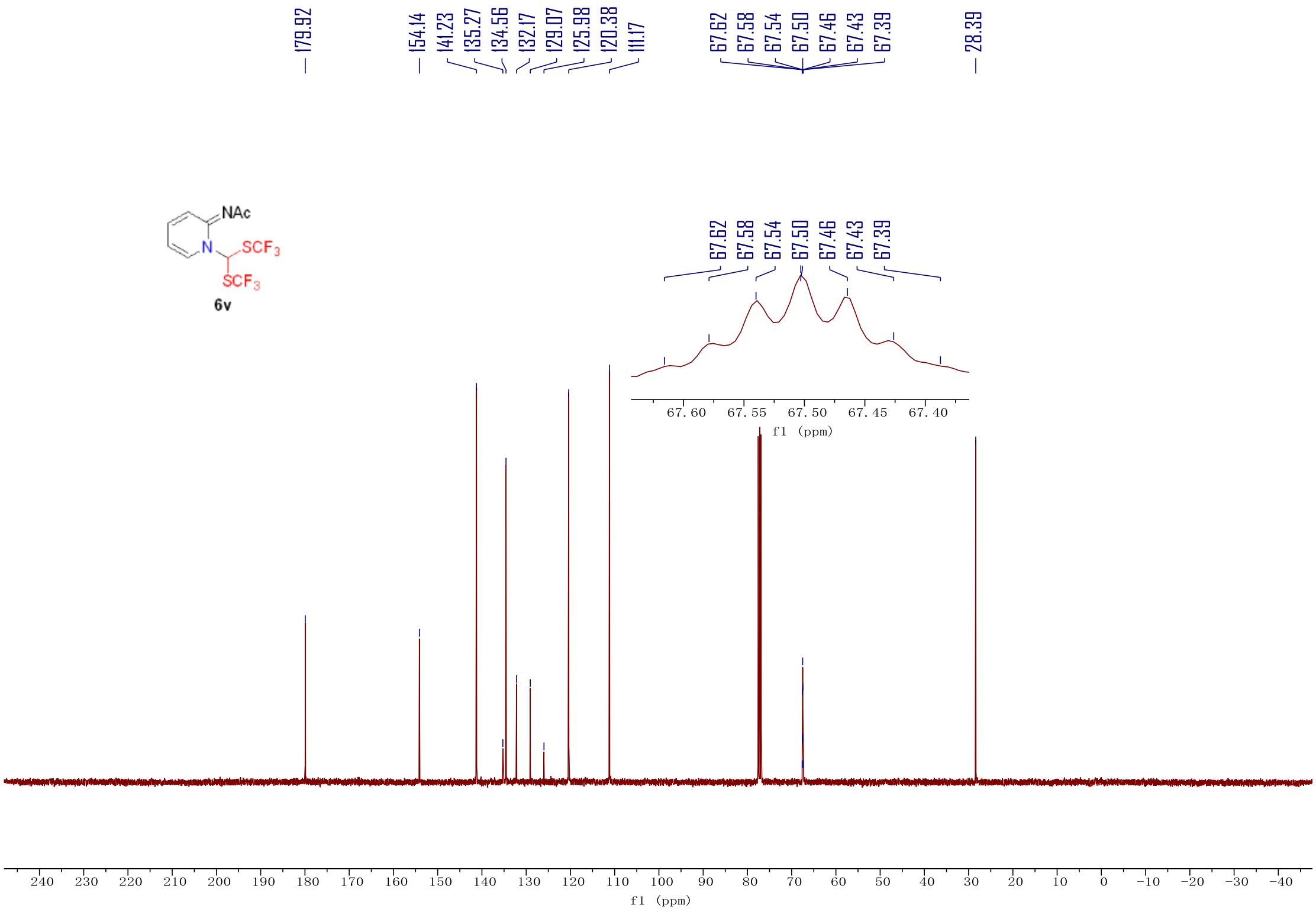

^{1}H NMR Spectrum of **8a** (400 MHz, $CDCl_3$)

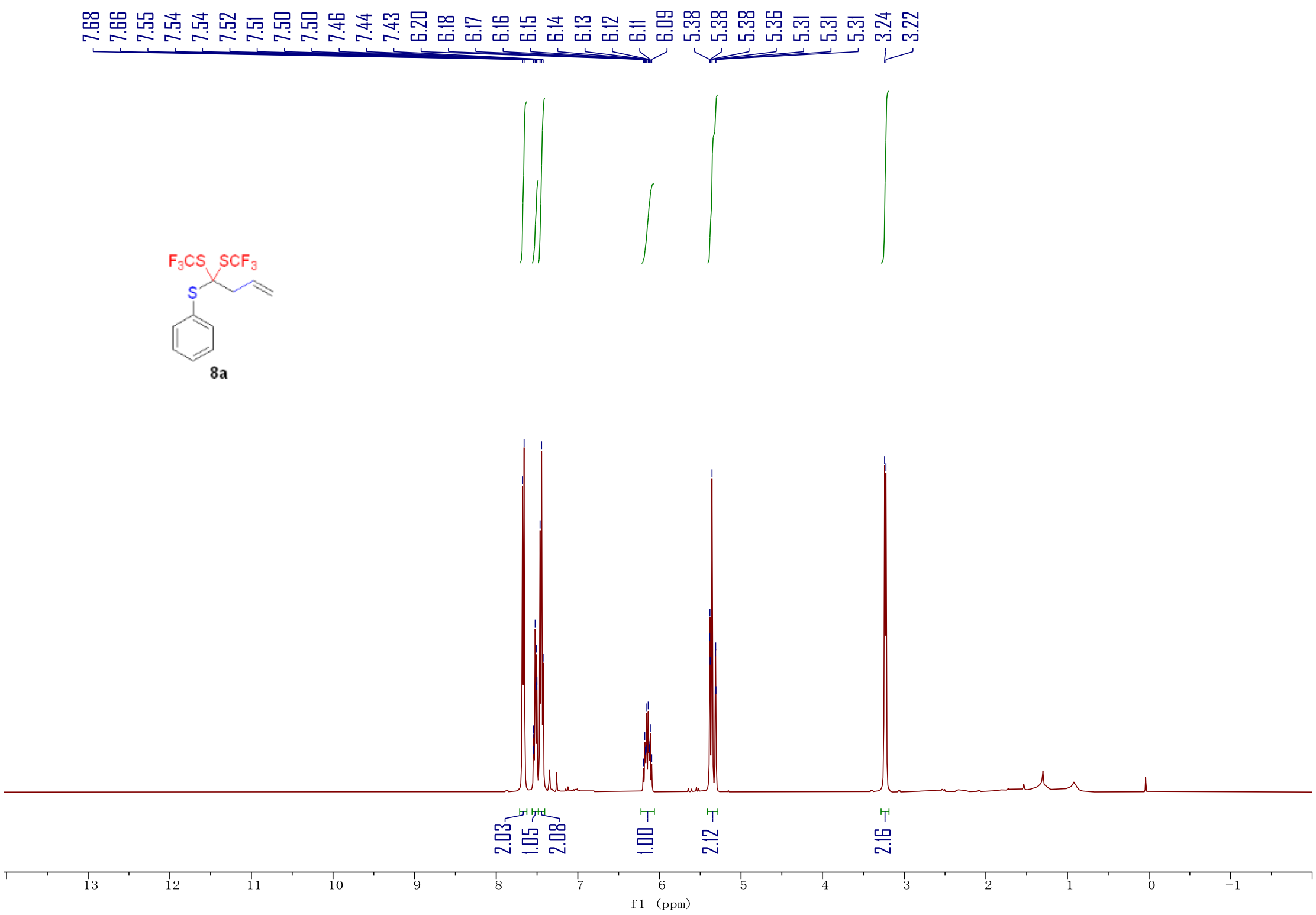

^{19}F NMR Spectrum of **8a** (376 MHz, $CDCl_3$)

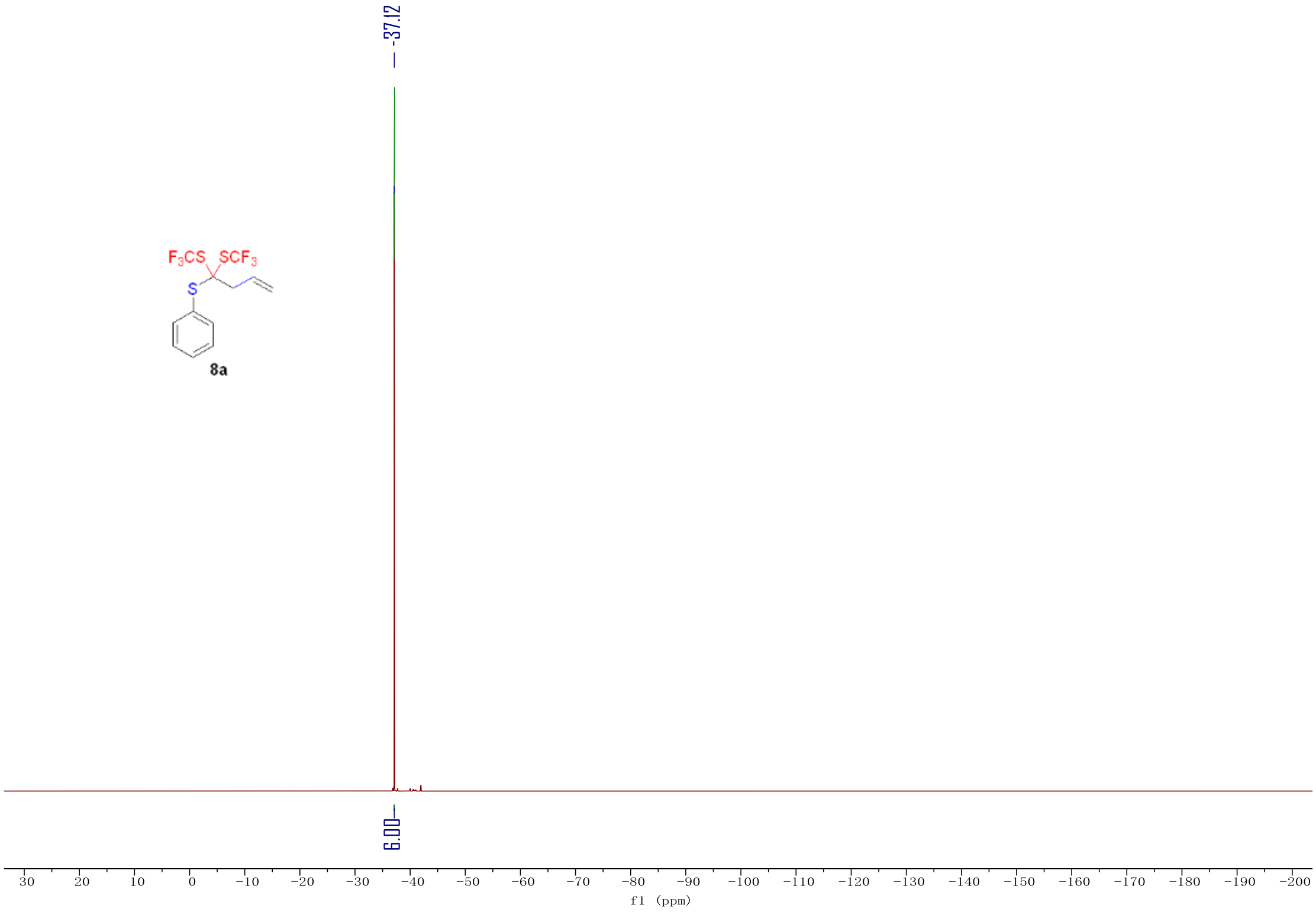

^{13}C NMR Spectrum of **8a** (101 MHz, $CDCl_3$)

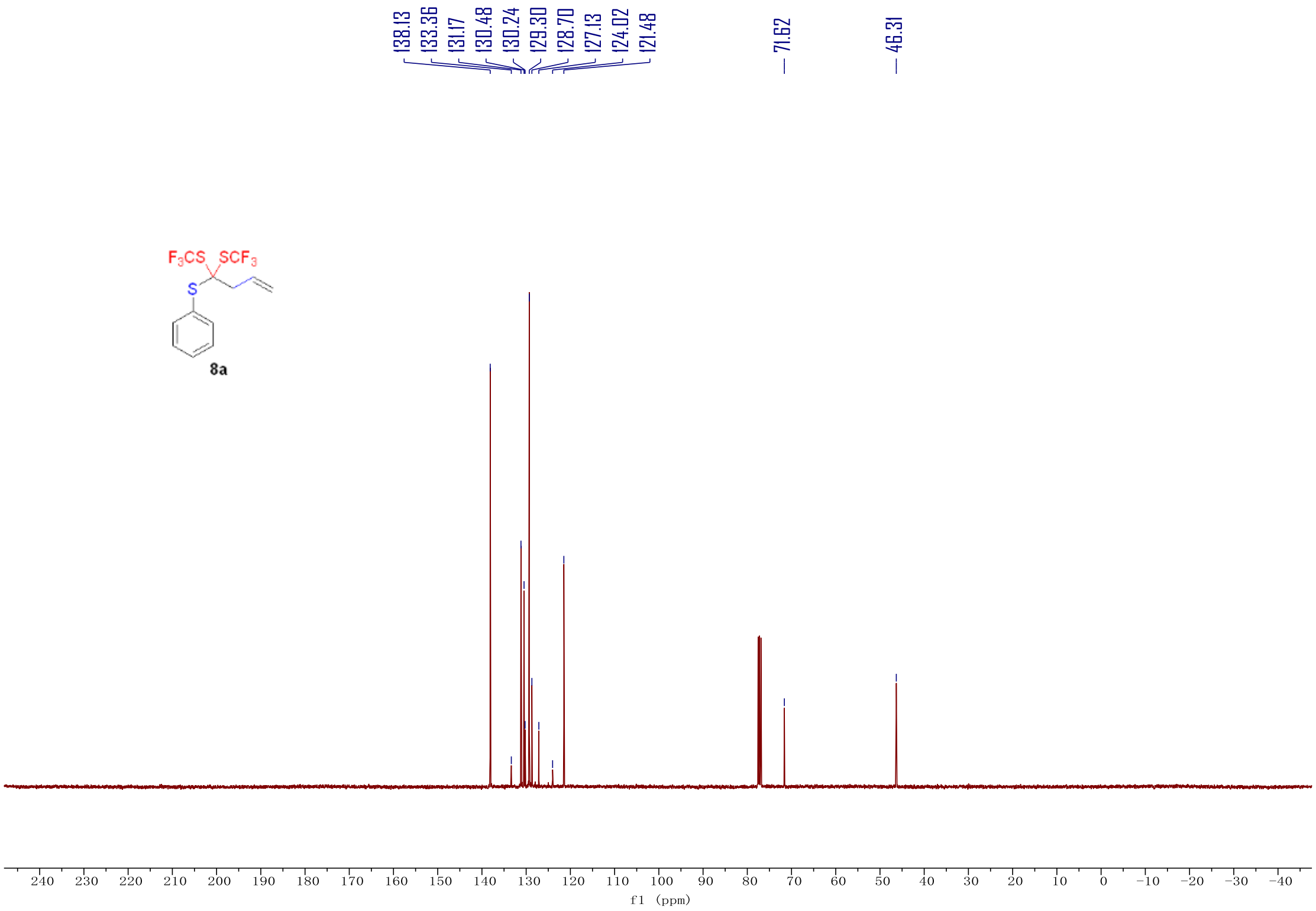

^{1}H NMR Spectrum of **8b** (400 MHz, $CDCl_3$)

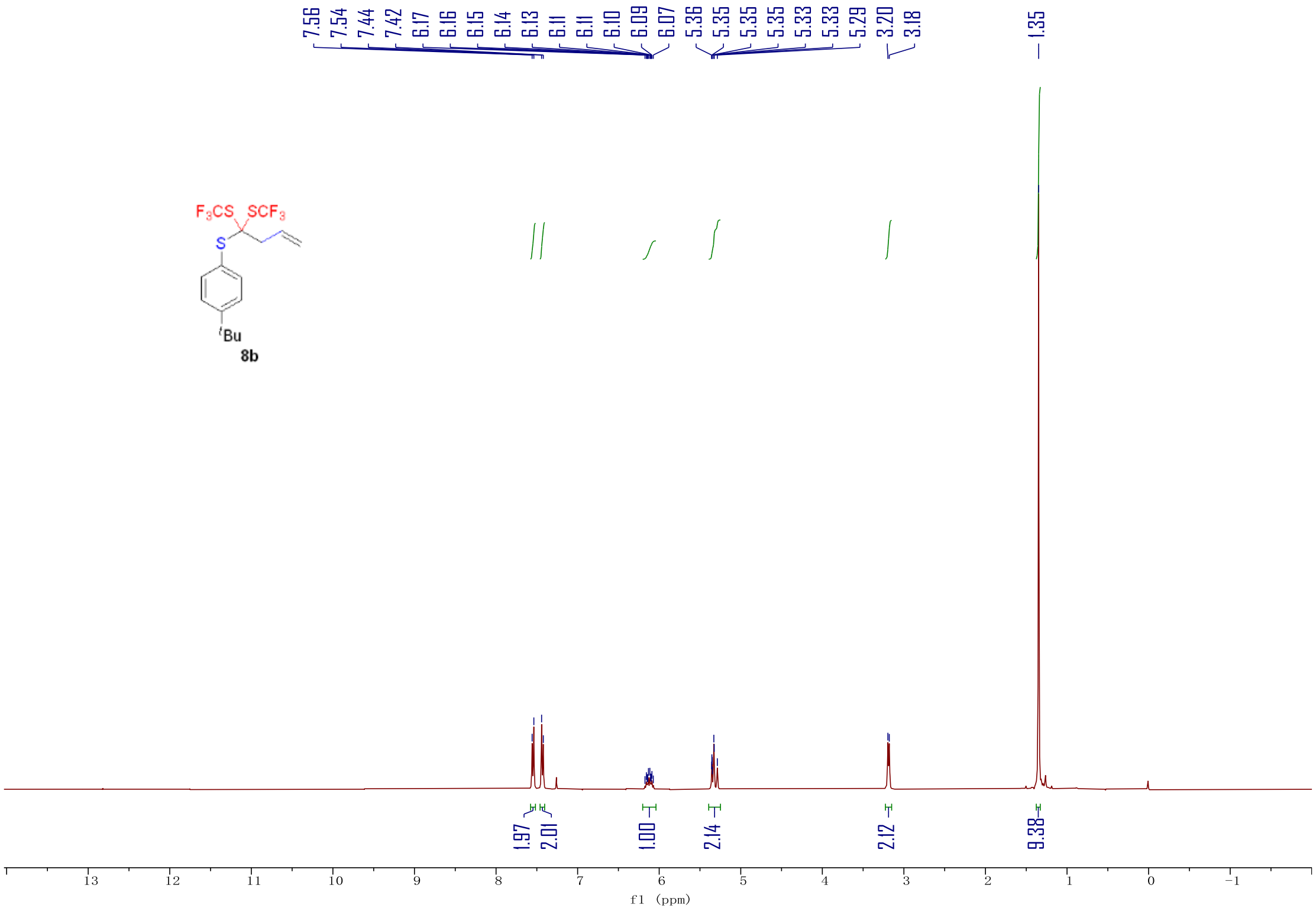

^{19}F NMR Spectrum of **8b** (376 MHz, $CDCl_3$)

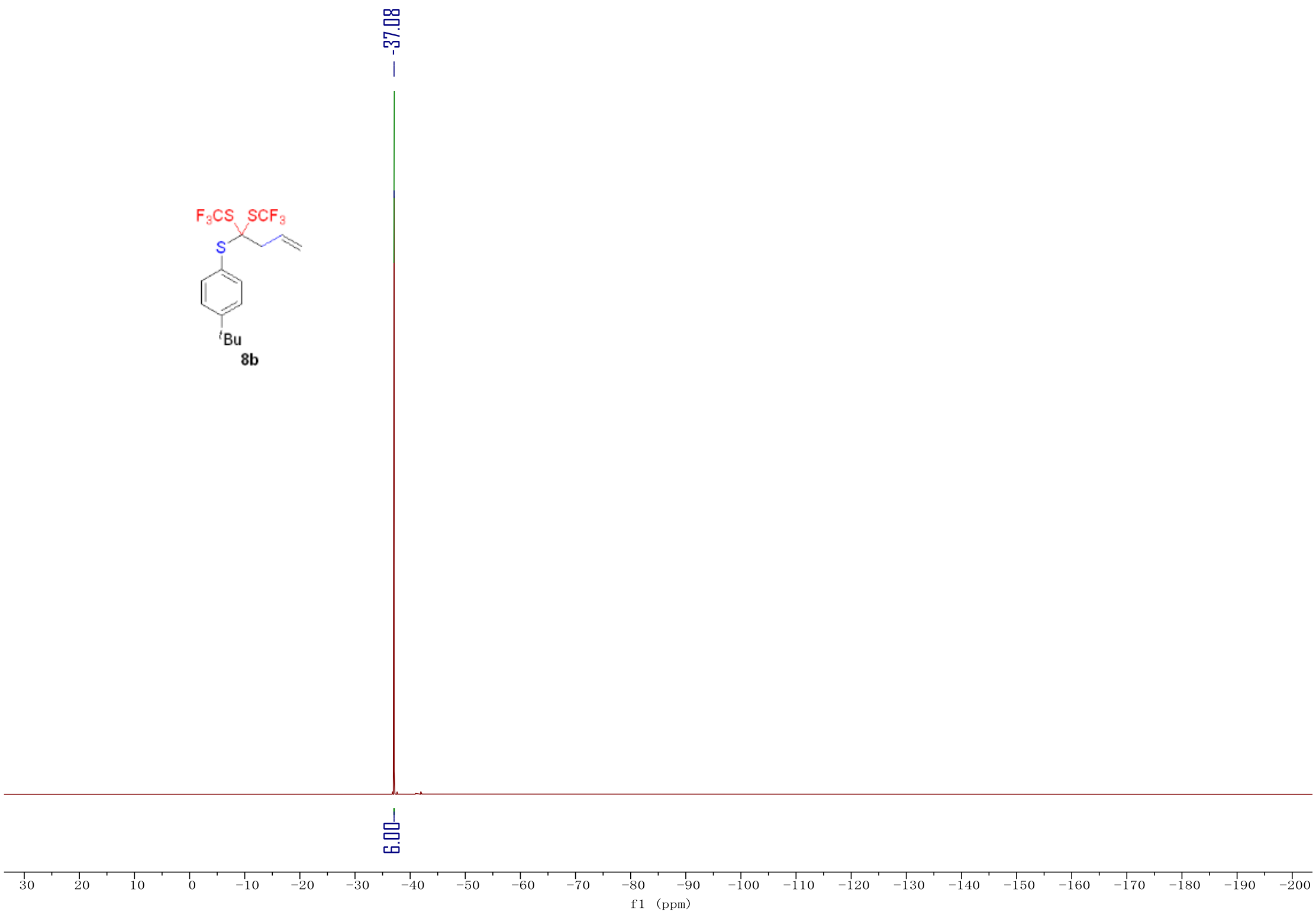

^{13}C **NMR** Spectrum of **8b** (101 MHz, CDCl$_3$)

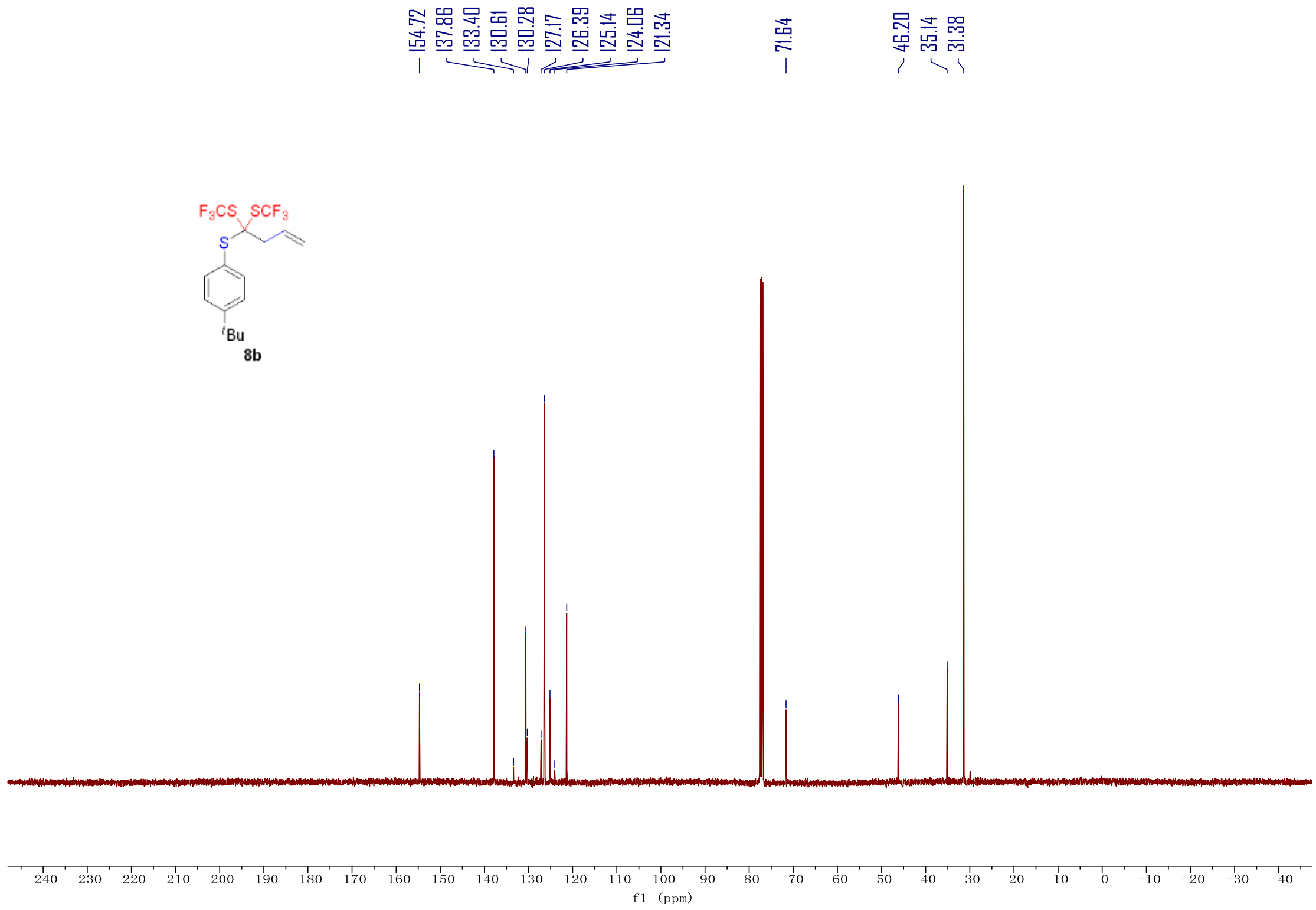

^{1}H **NMR** Spectrum of **8c** (400 MHz, CDCl$_3$)

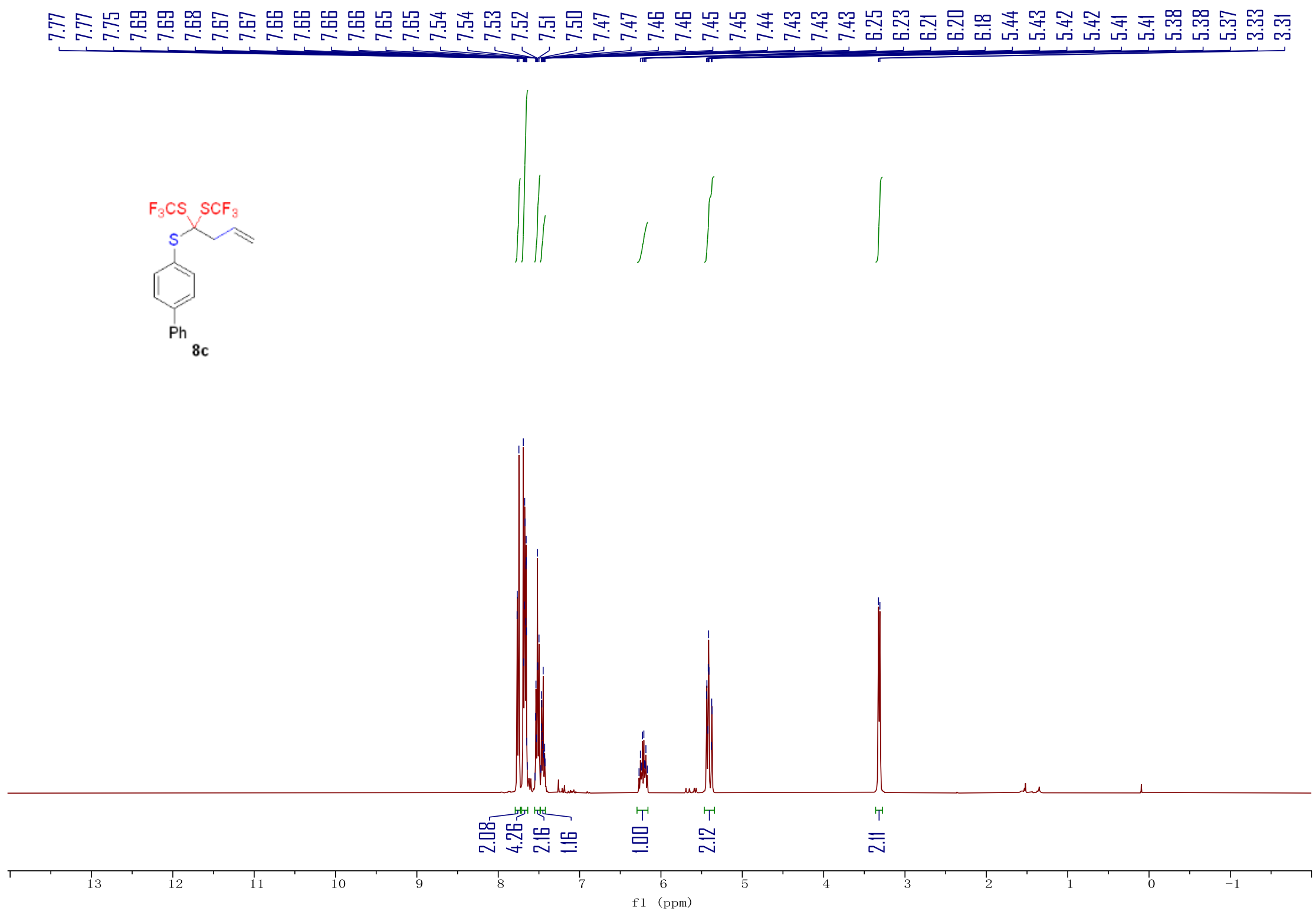

^{19}F NMR Spectrum of **8c** (376 MHz, $CDCl_3$)

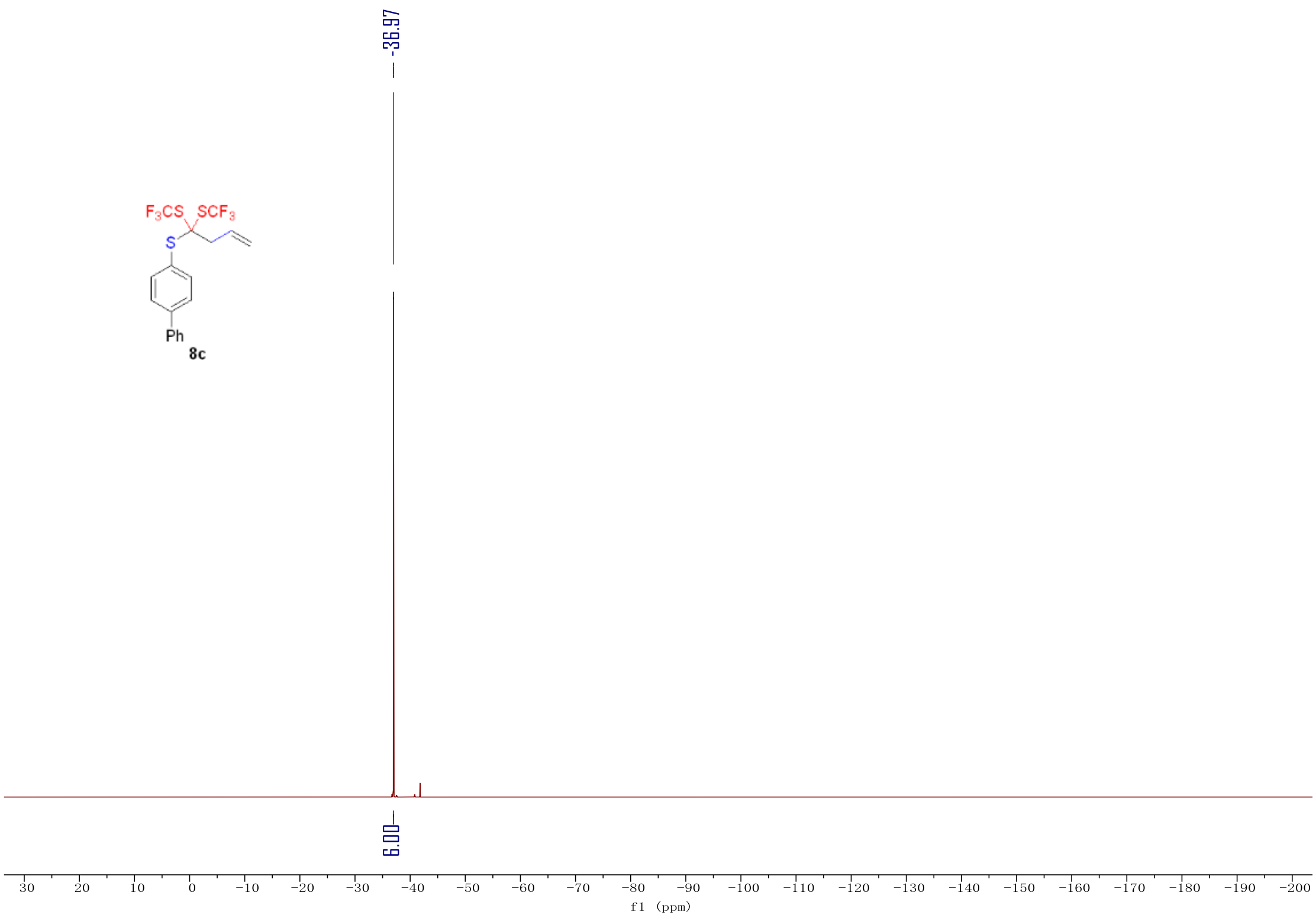

^{13}C NMR Spectrum of **8c** (101 MHz, $CDCl_3$)

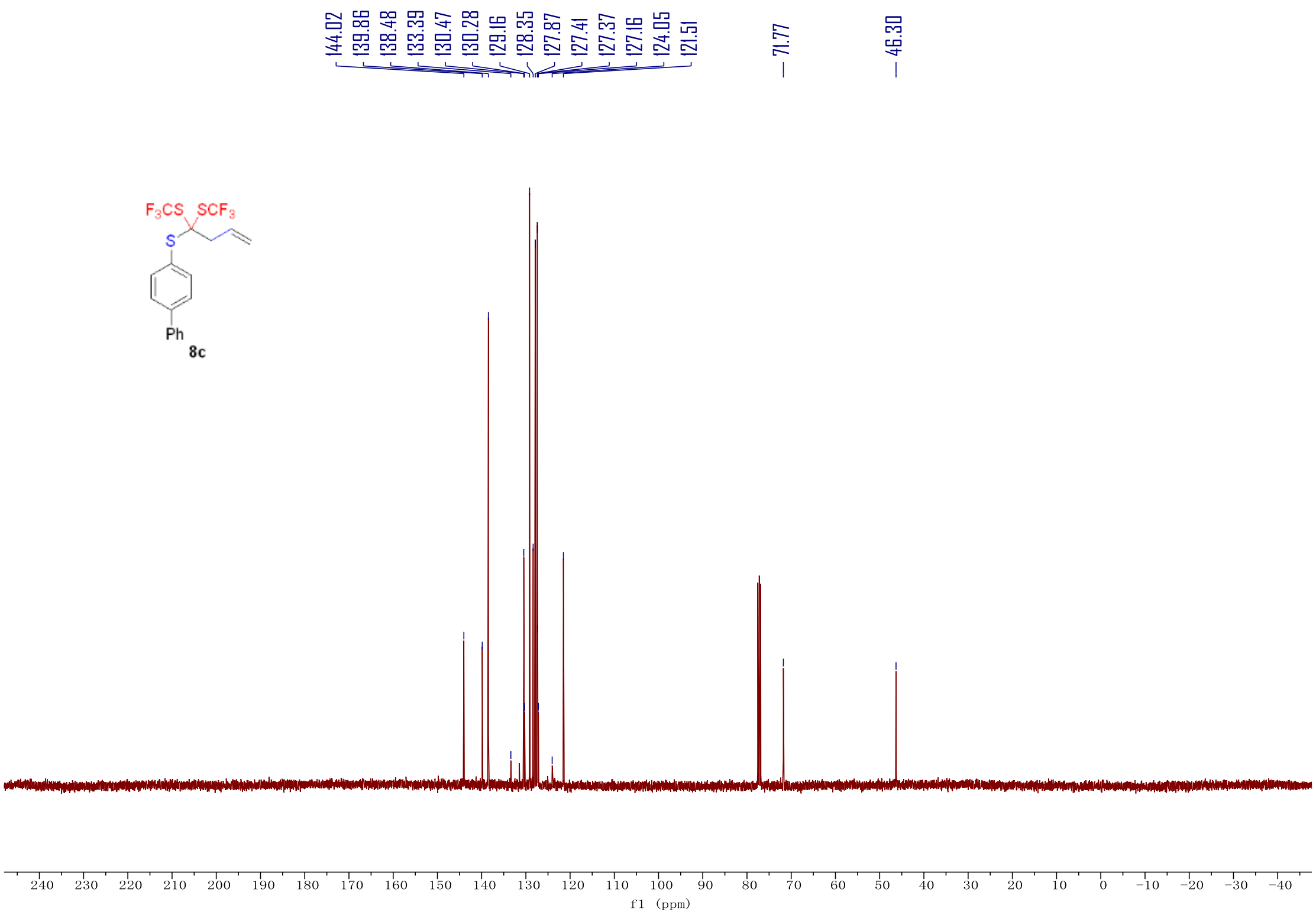

^{1}H NMR Spectrum of **8d** (400 MHz, $CDCl_3$)

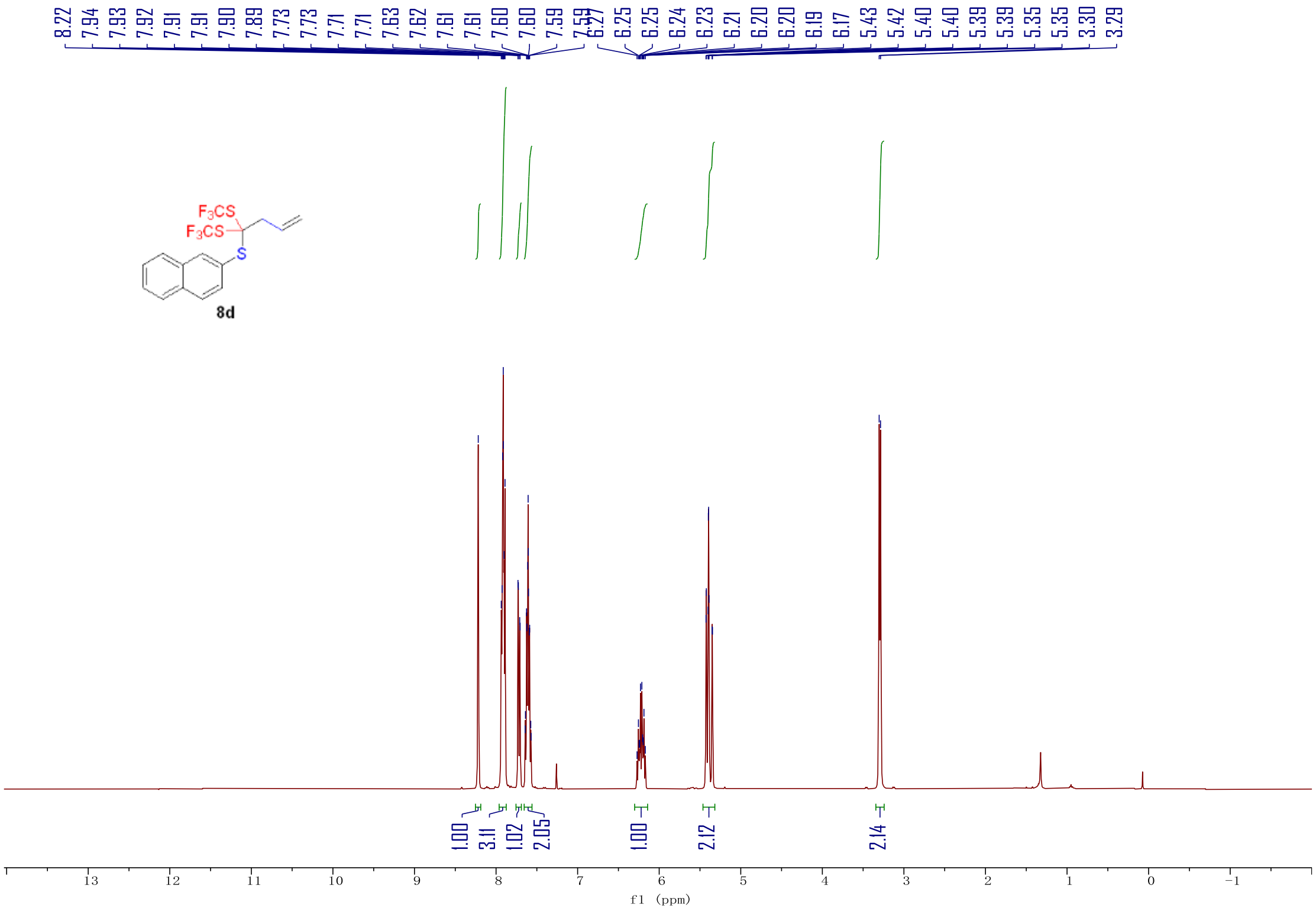

^{19}F NMR Spectrum of **8d** (376 MHz, $CDCl_3$)

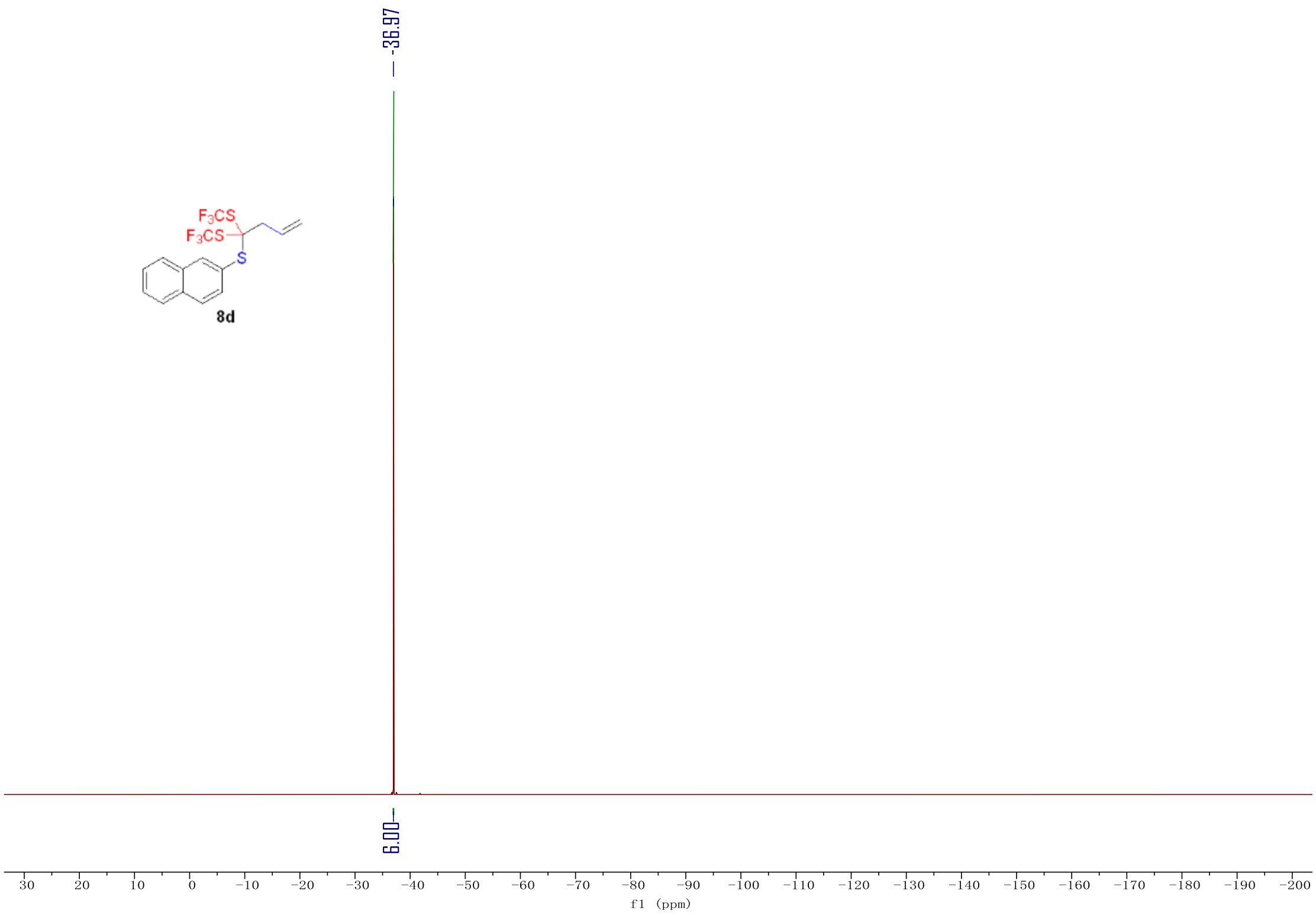

^{13}C NMR Spectrum of **8d** (101 MHz, $CDCl_3$)

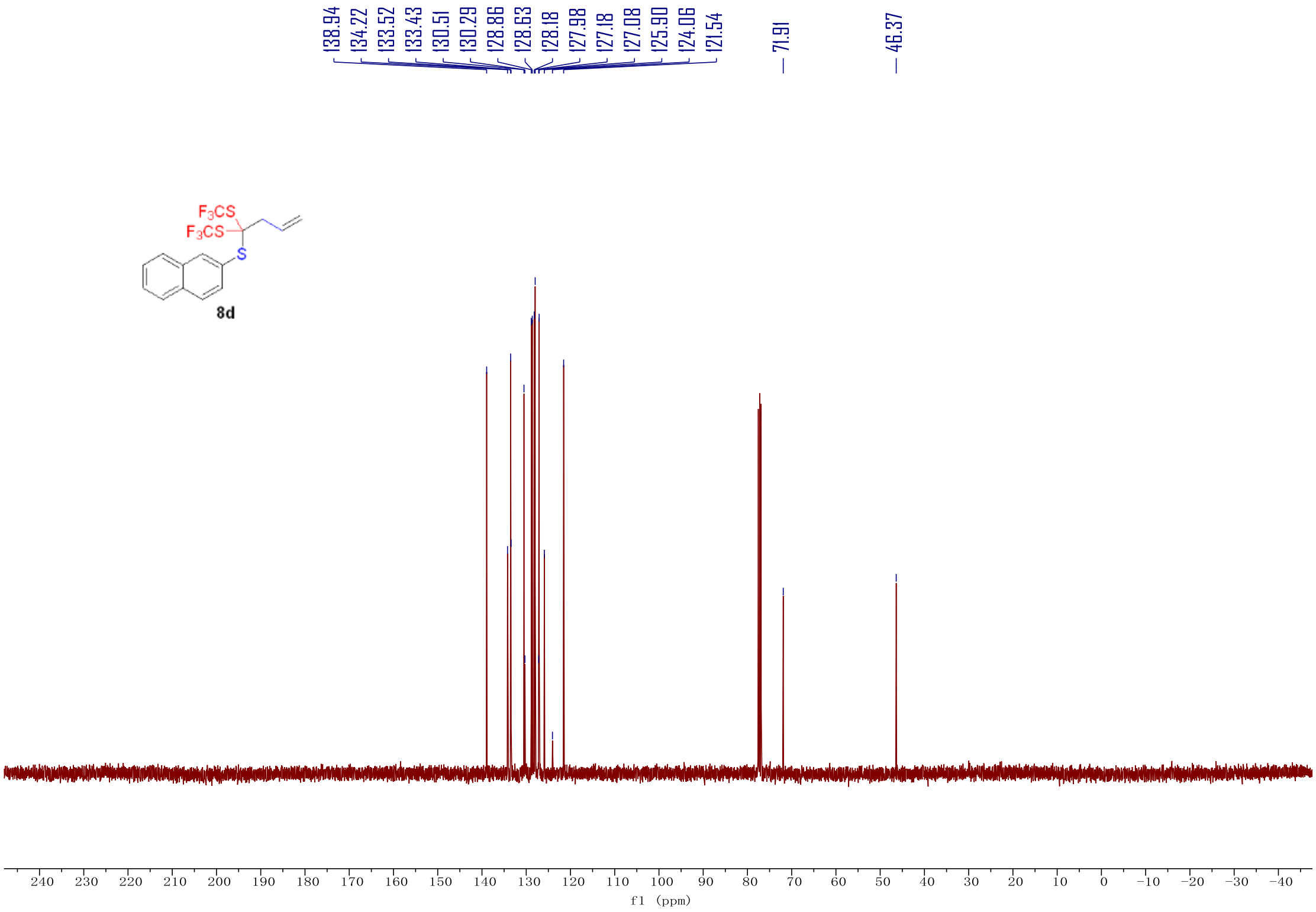

^{1}H NMR Spectrum of **8e** (400 MHz, $CDCl_3$)

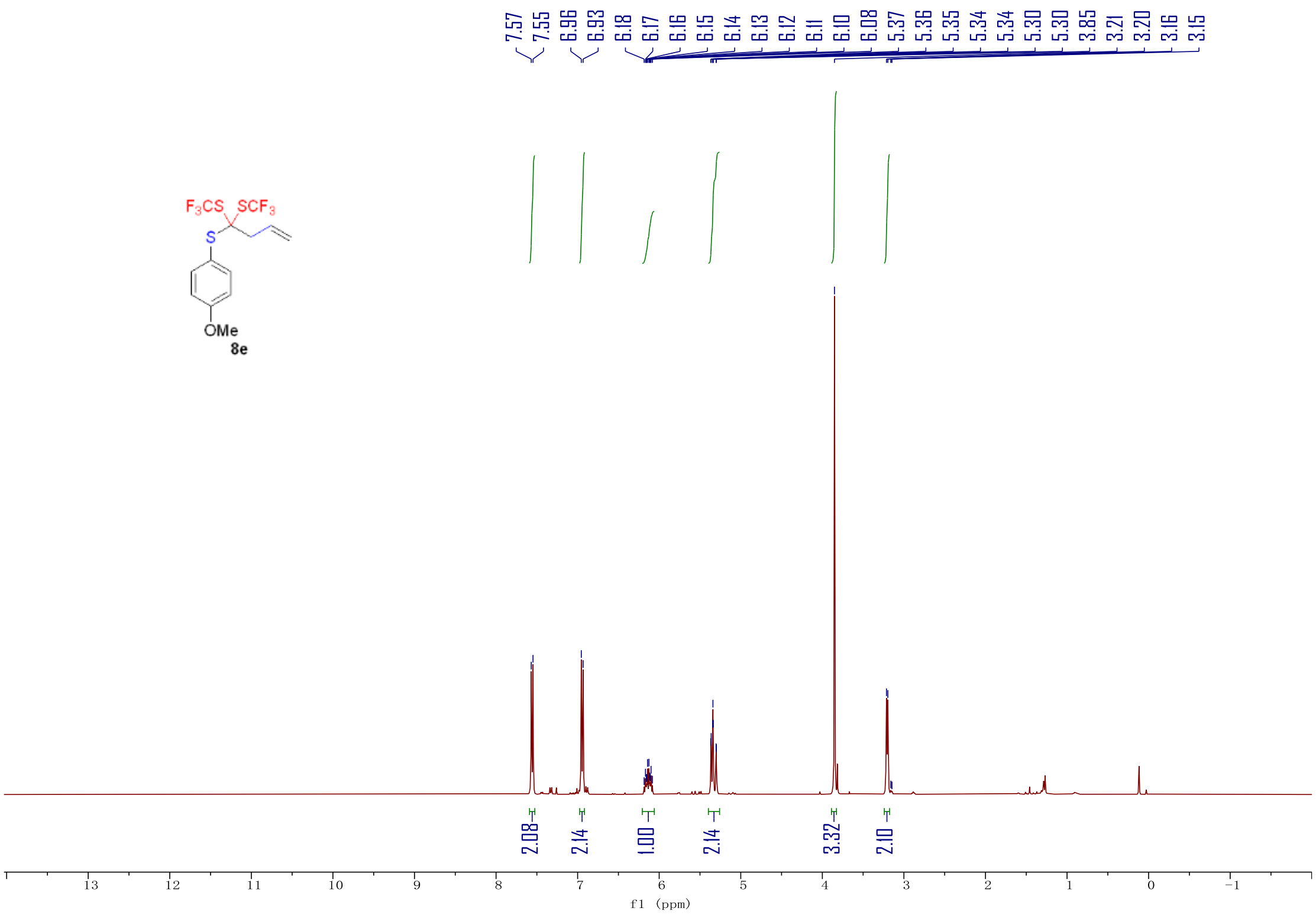

^{19}**F NMR** Spectrum of **8e** (376 MHz, $CDCl_3$)

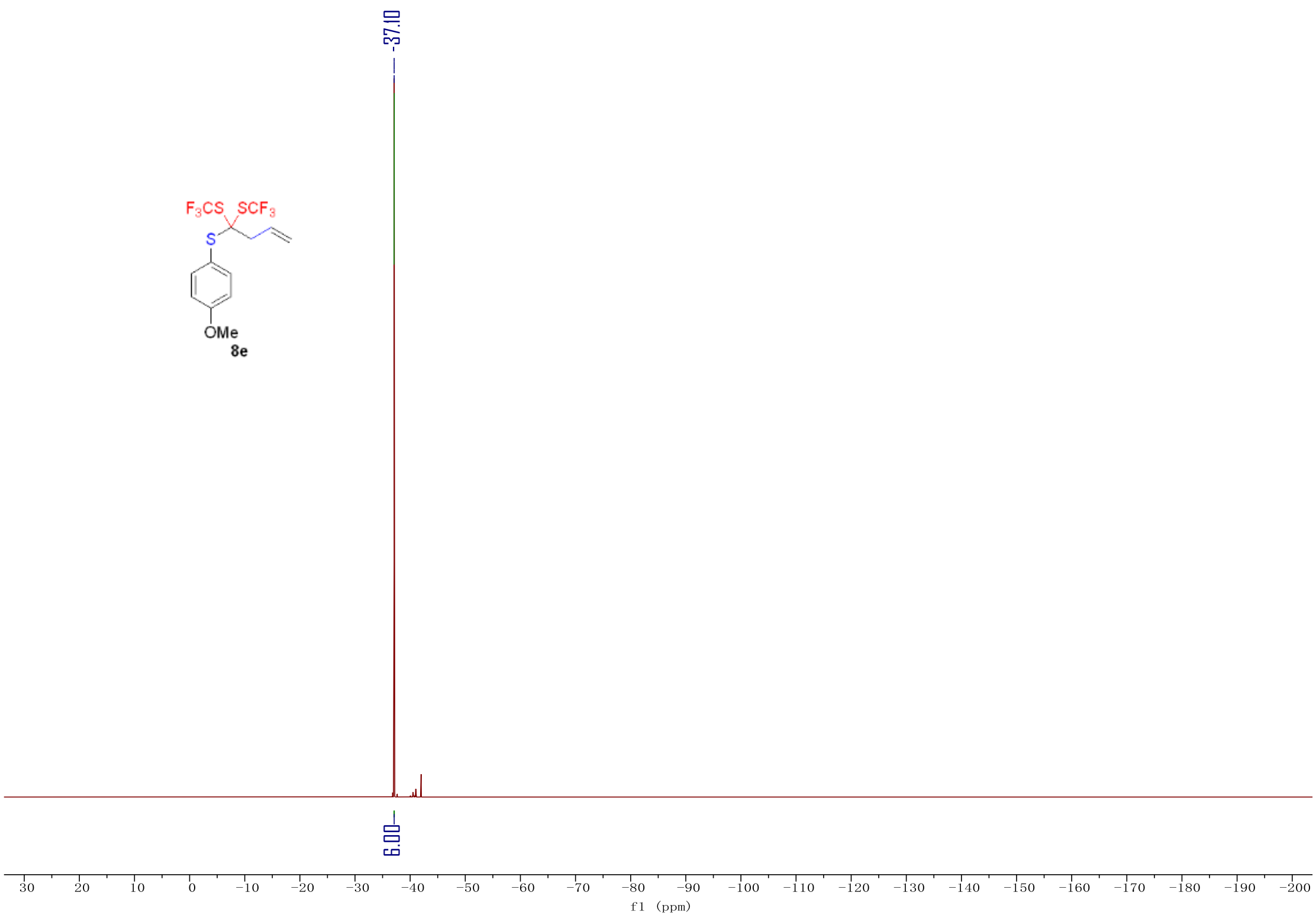

^{13}**C NMR** Spectrum of **8e** (101 MHz, $CDCl_3$)

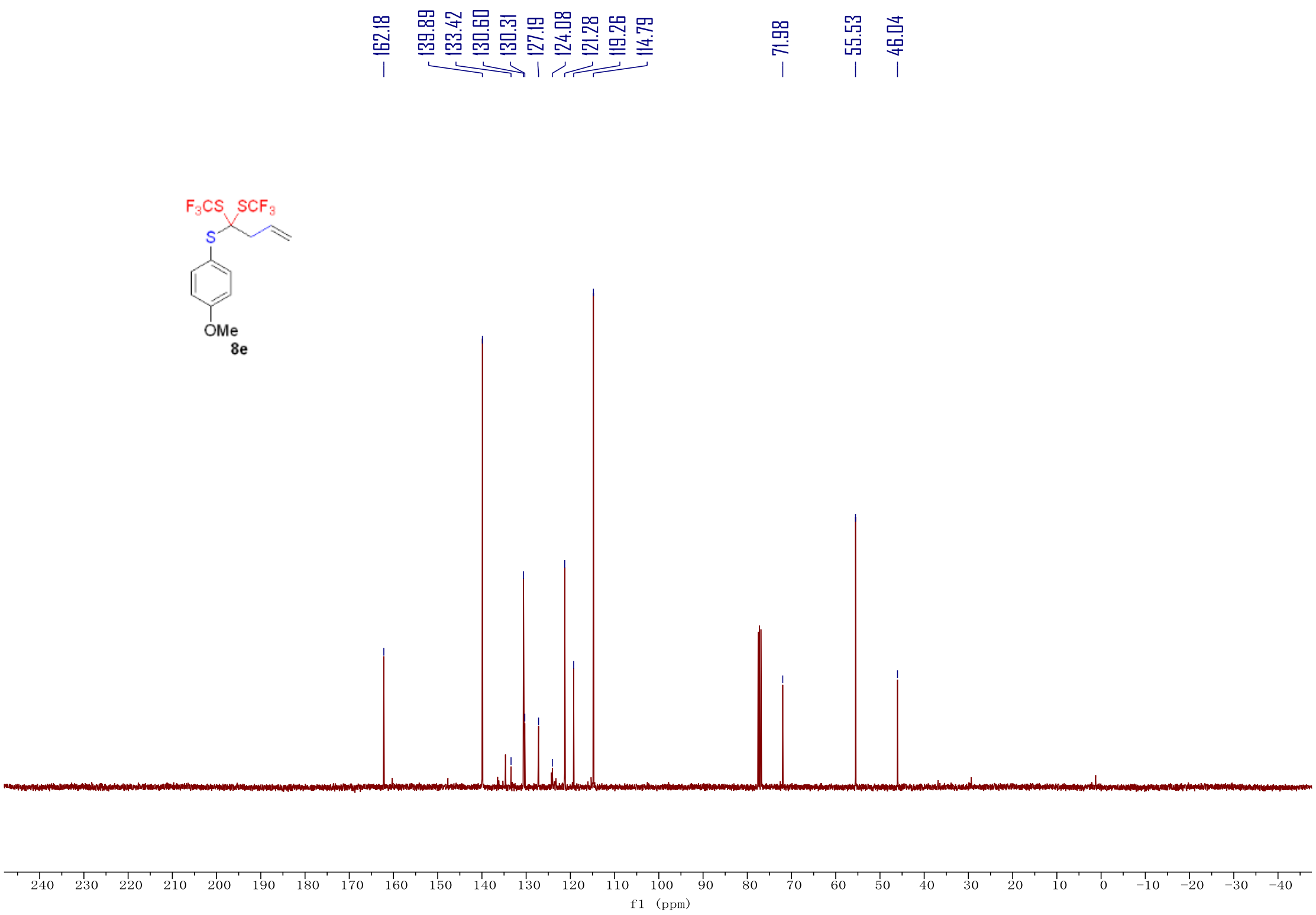

^{1}H NMR Spectrum of **8f** (400 MHz, $CDCl_3$)

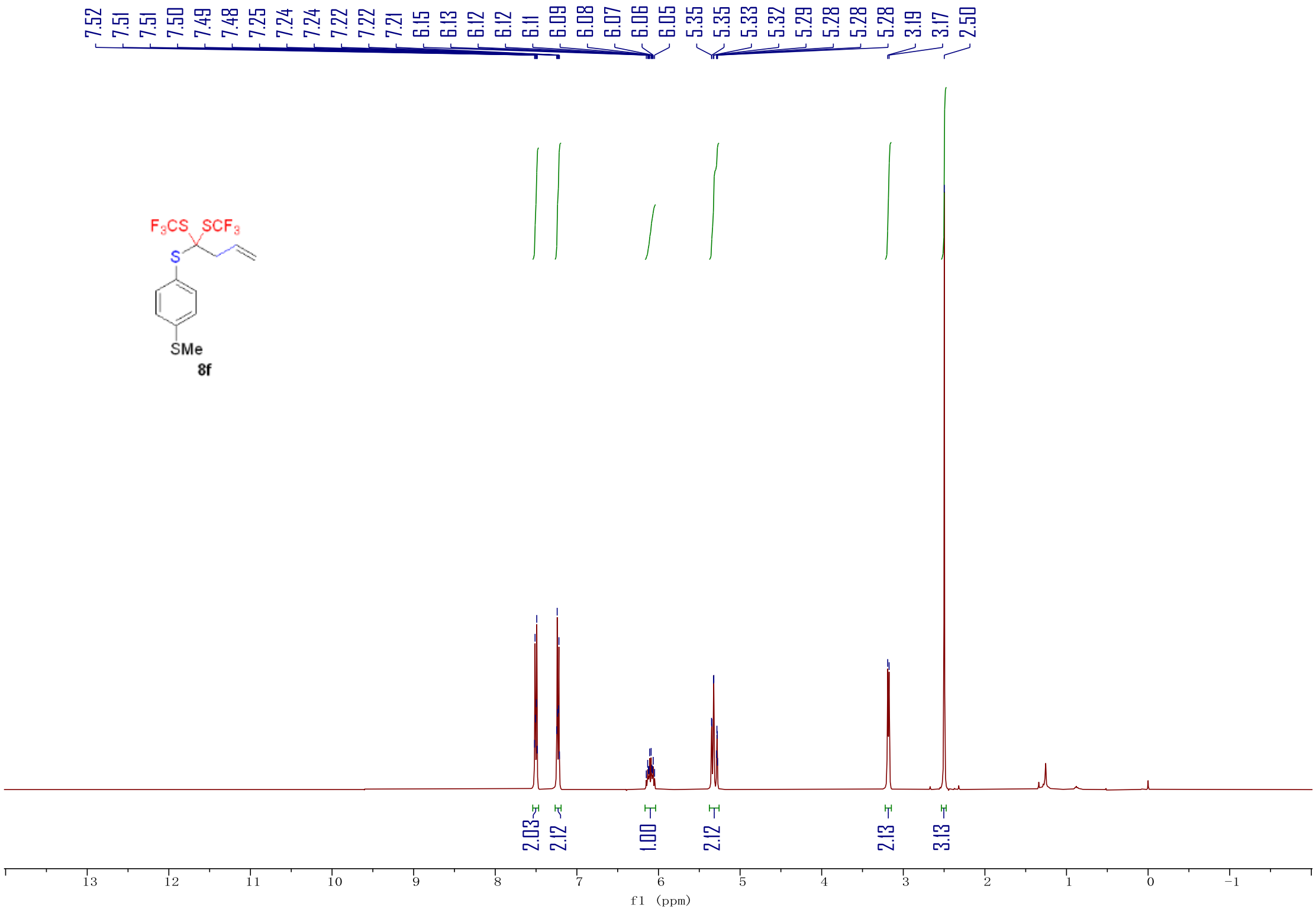

^{19}F NMR Spectrum of **8f** (376 MHz, $CDCl_3$)

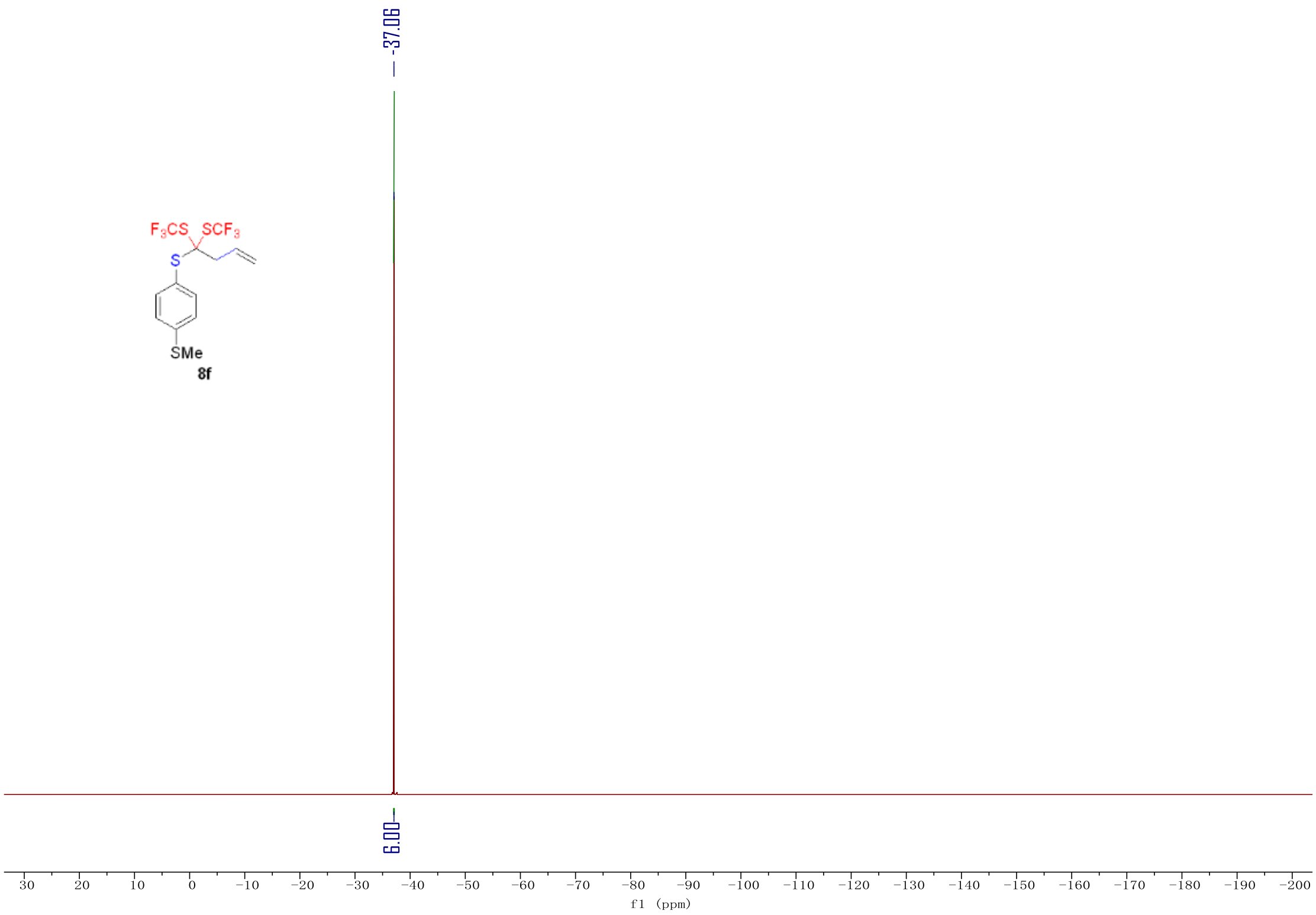

^{13}C NMR Spectrum of **8f** (101 MHz, CDCl$_3$)

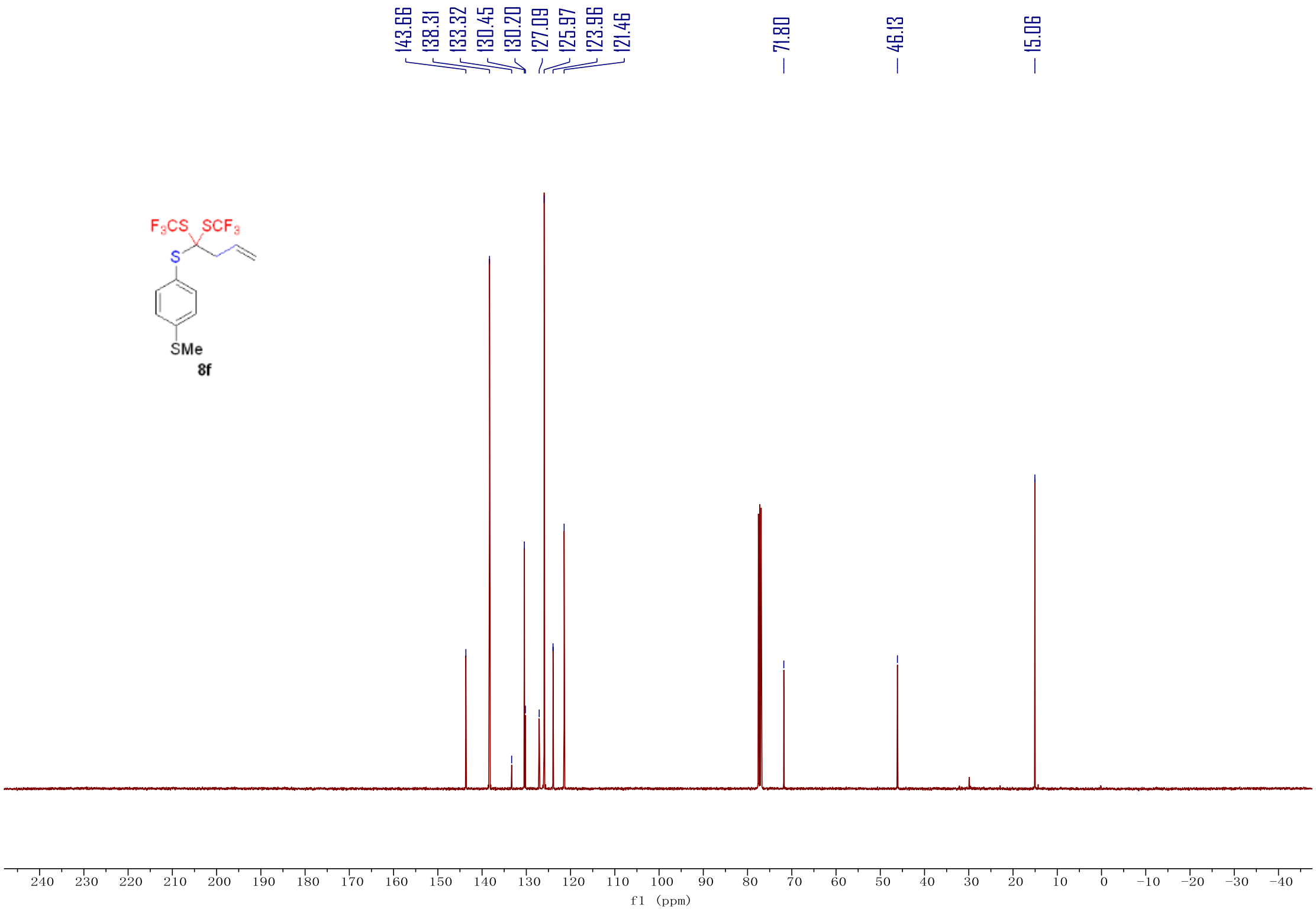

^{1}H NMR Spectrum of **8g** (400 MHz, CDCl$_3$)

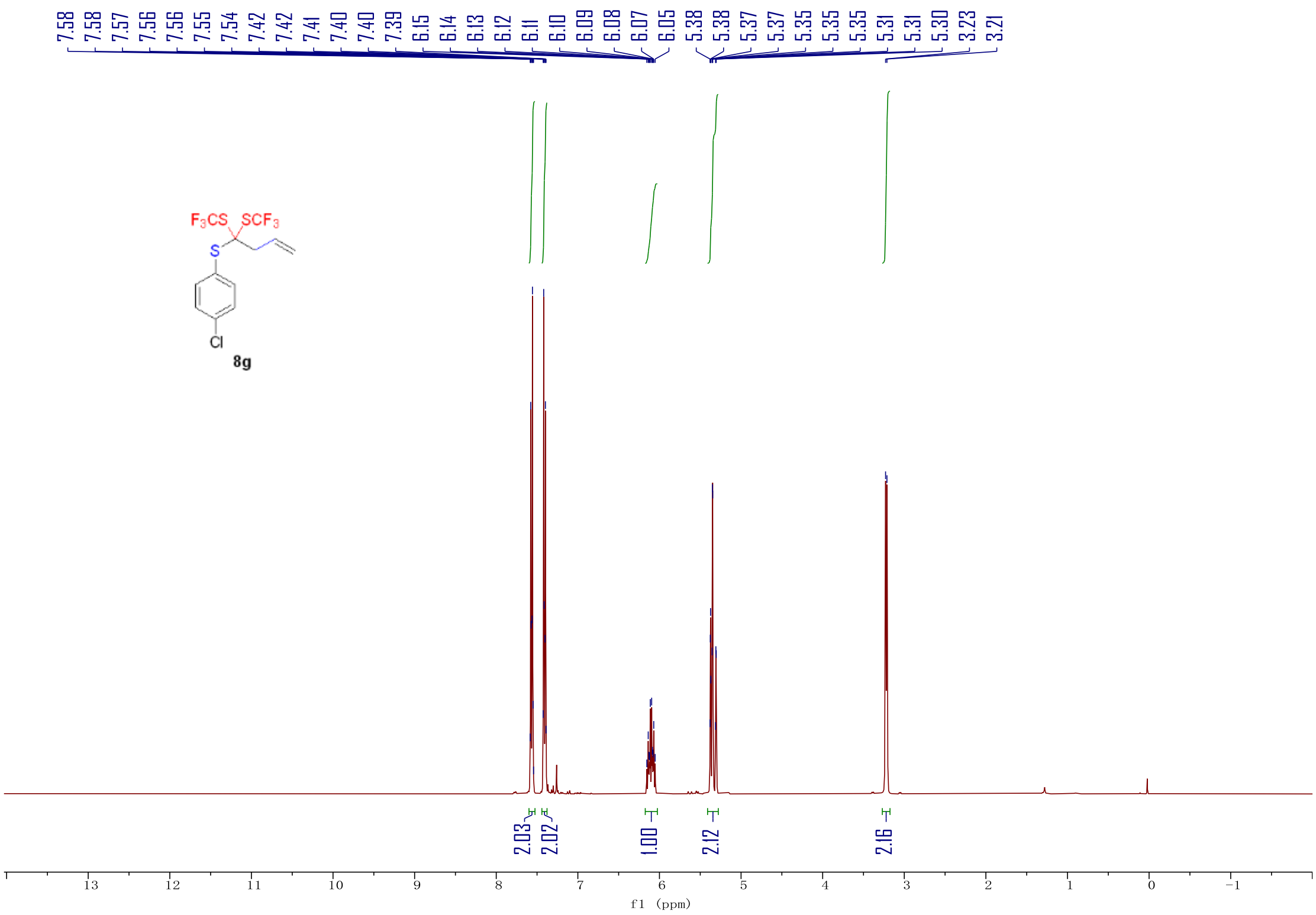

^{19}F NMR Spectrum of **8g** (376 MHz, $CDCl_3$)

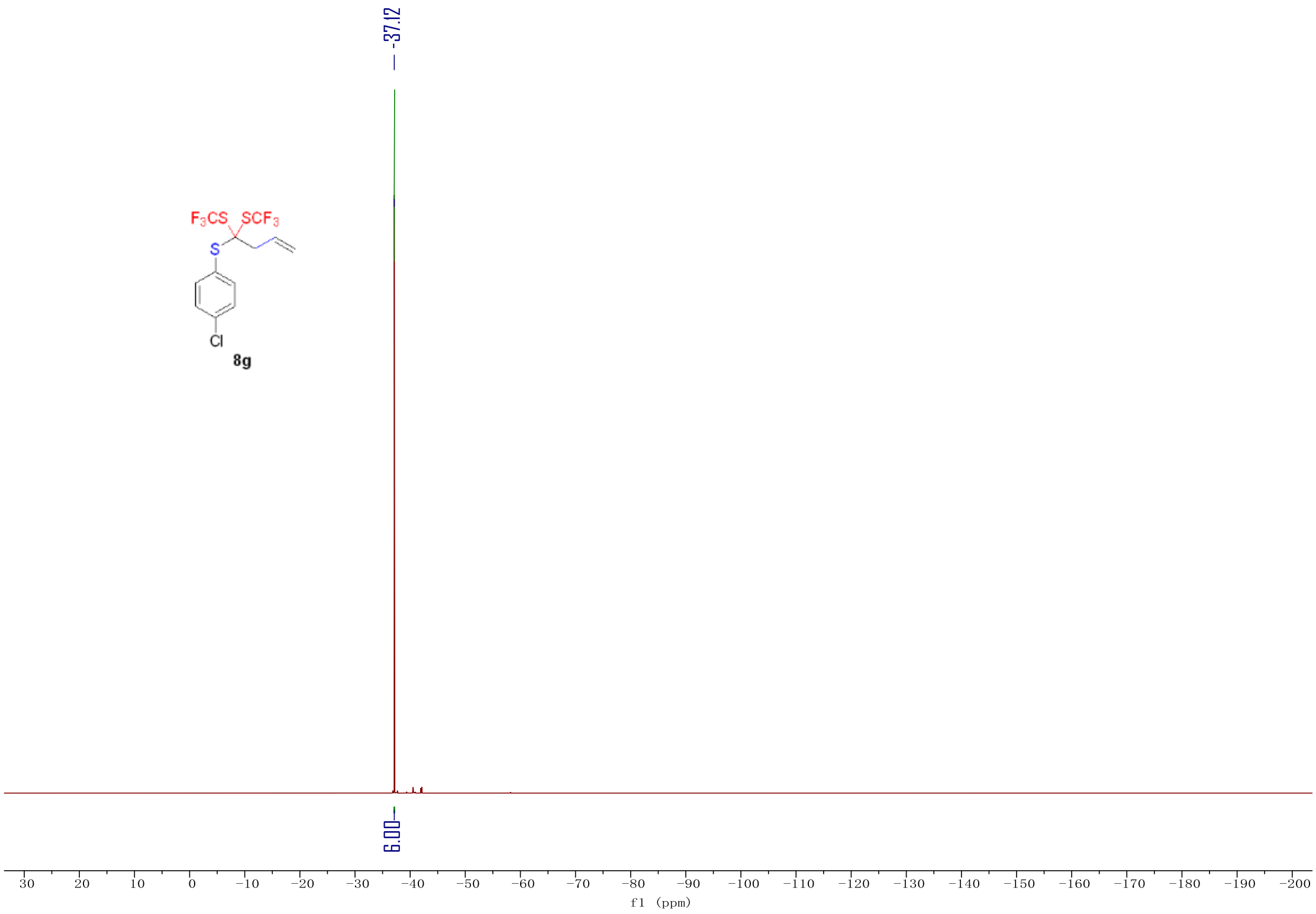

^{13}C NMR Spectrum of **8g** (101 MHz, $CDCl_3$)

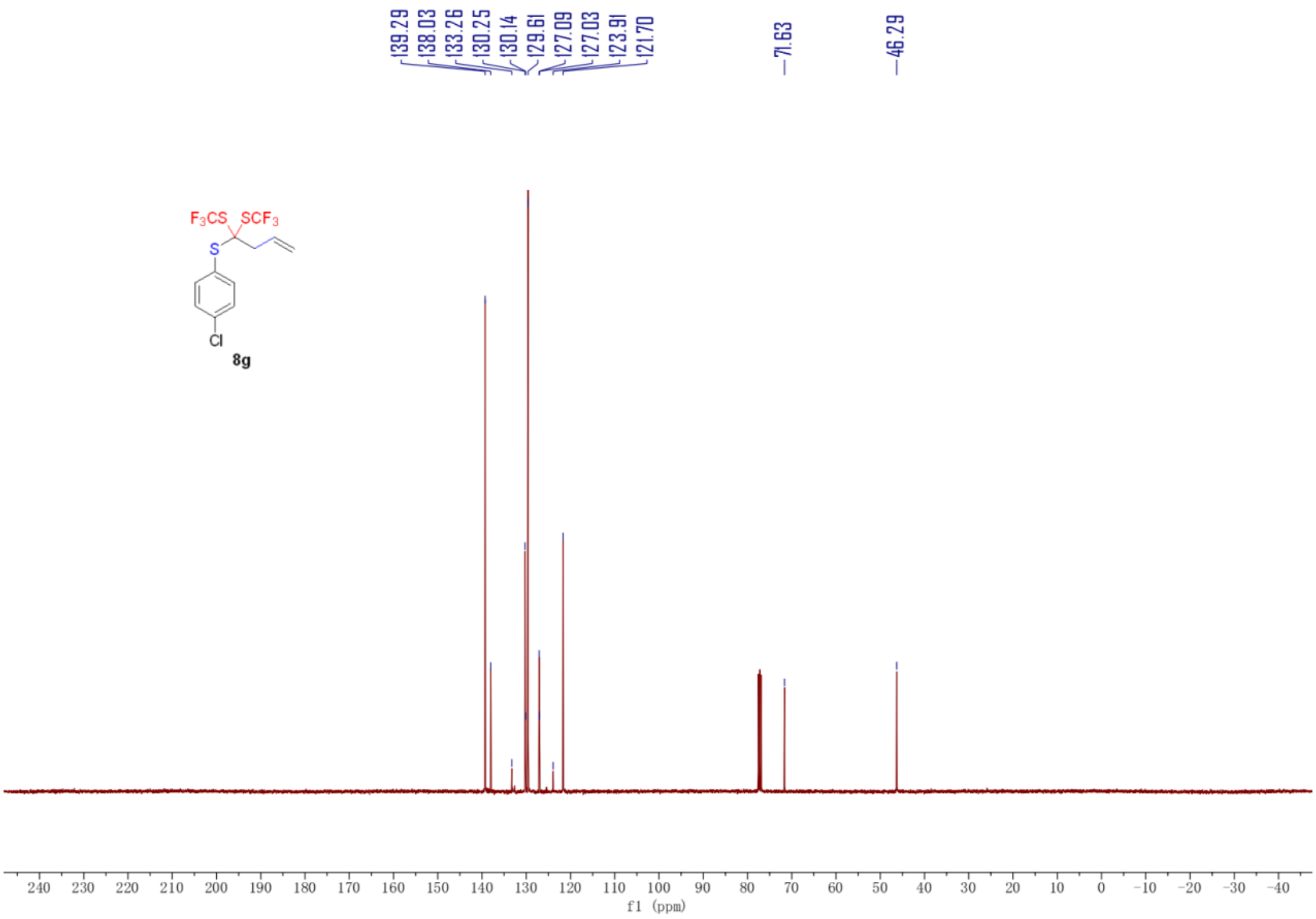

^{1}**H NMR** Spectrum of **8h** (400 MHz, $CDCl_3$)

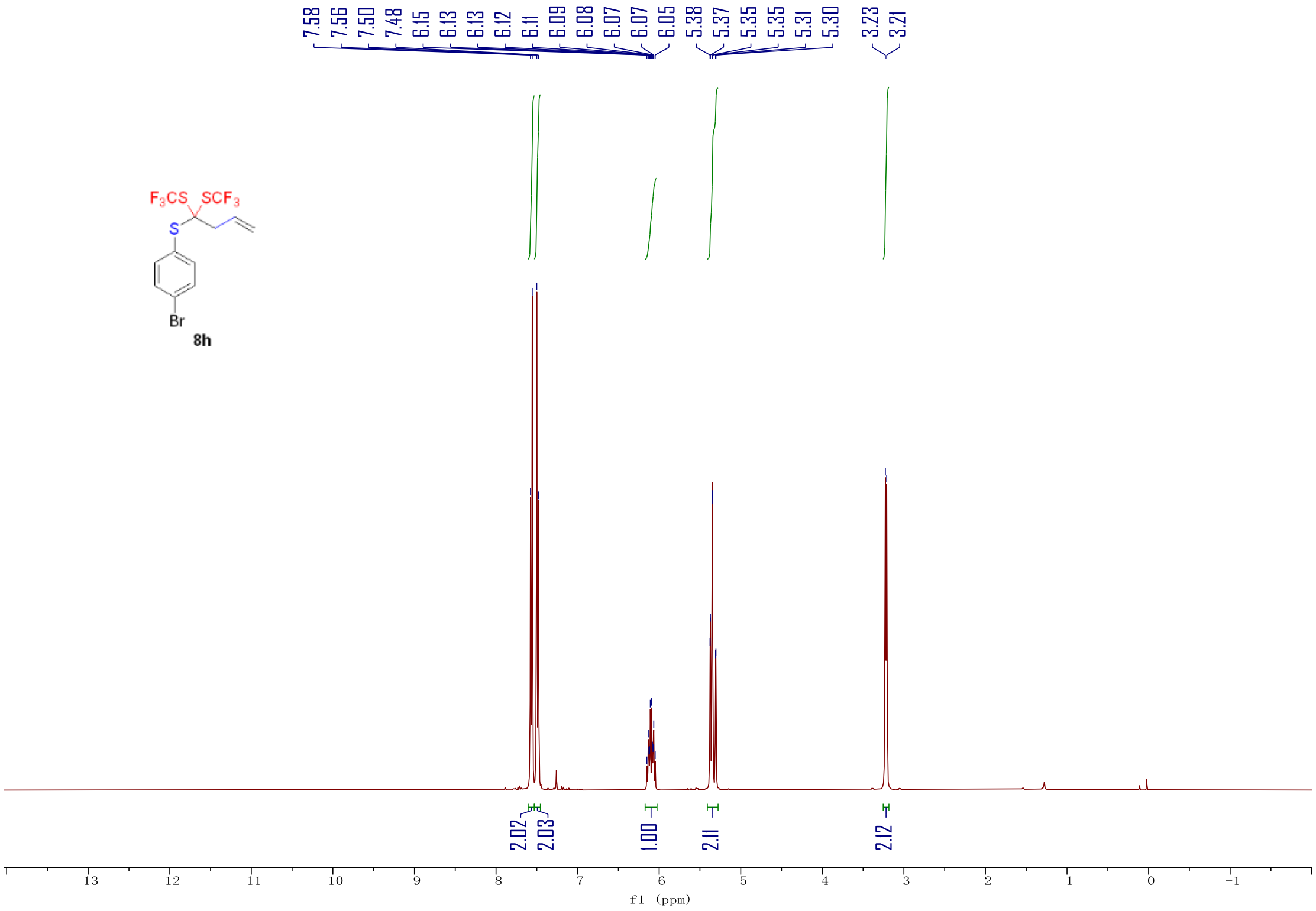

^{19}**F NMR** Spectrum of **8h** (376 MHz, $CDCl_3$)

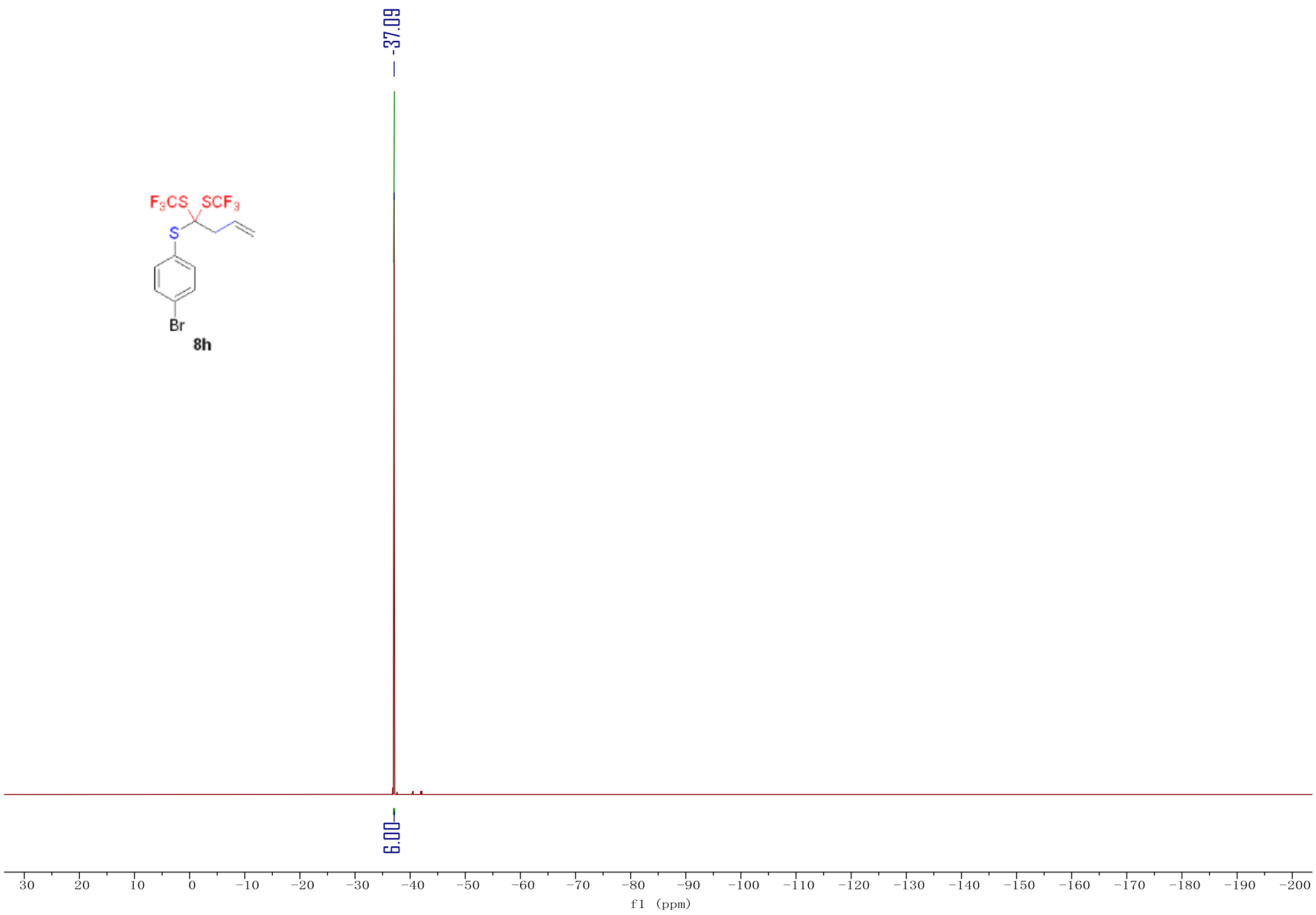

^{13}C NMR Spectrum of **8h** (101 MHz, $CDCl_3$)

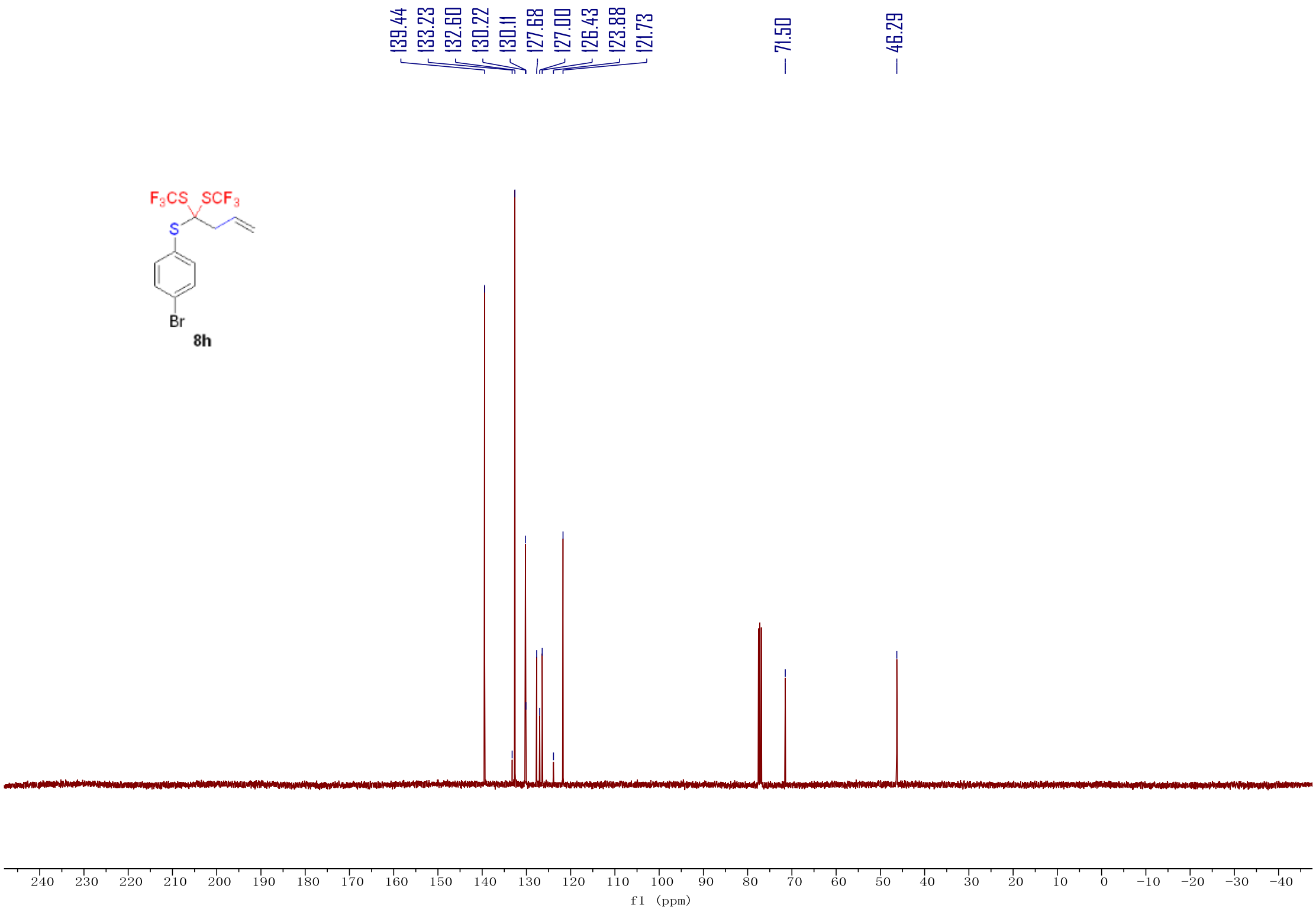

^{1}H NMR Spectrum of **8i** (400 MHz, $CDCl_3$)

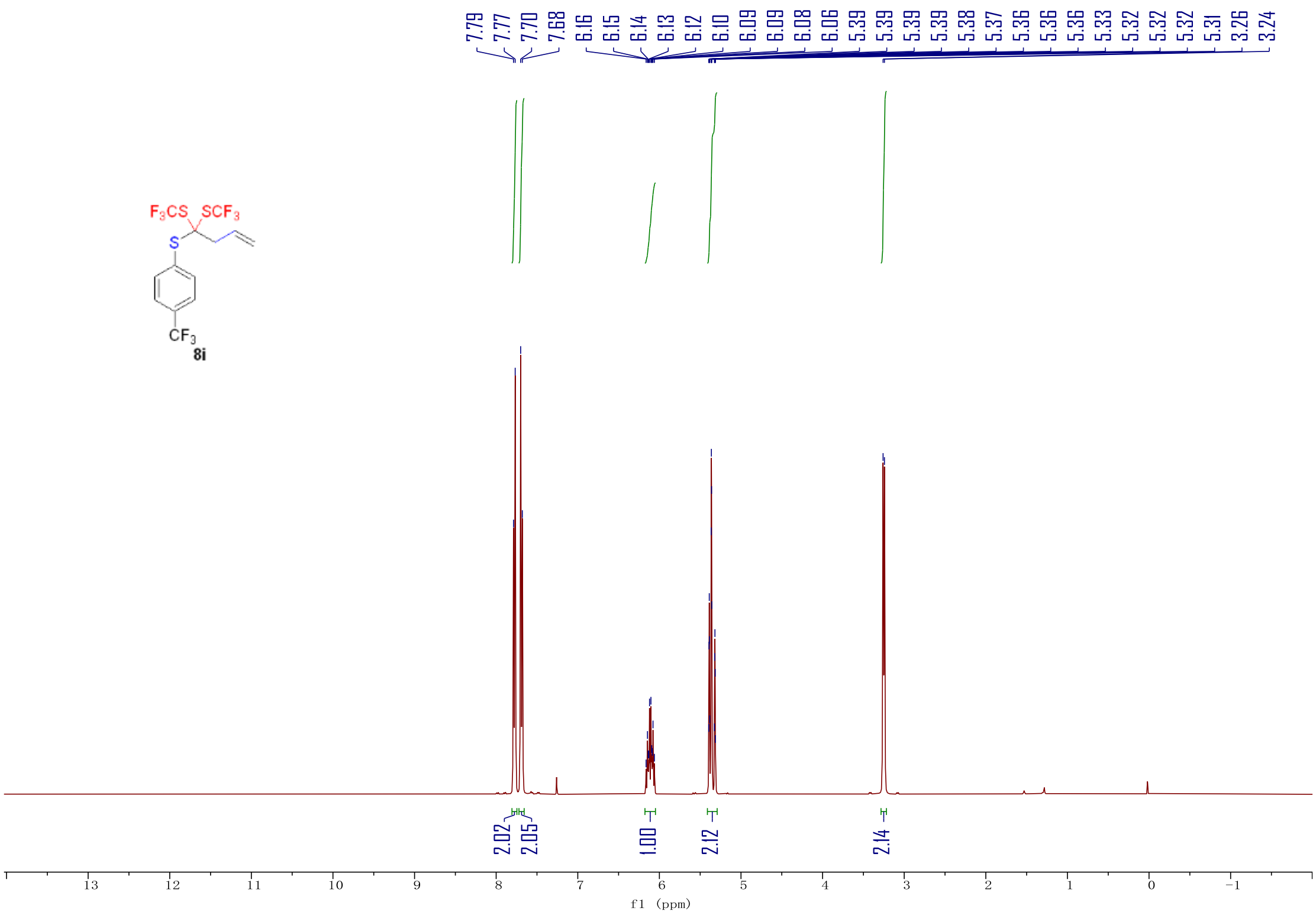

^{19}F **NMR** Spectrum of **8i** (376 MHz, $CDCl_3$)

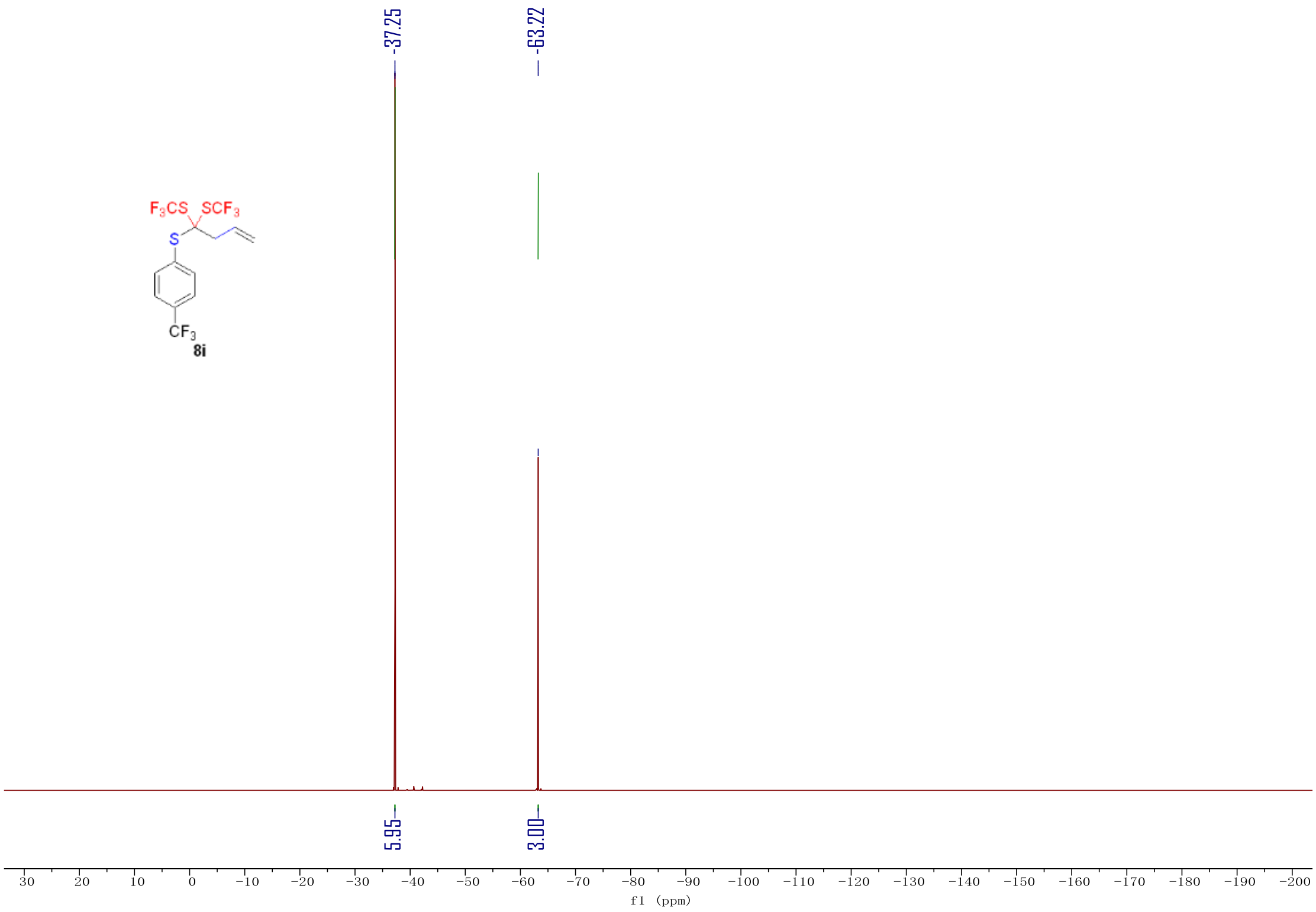

^{13}C **NMR** Spectrum of **8i** (101 MHz, $CDCl_3$)

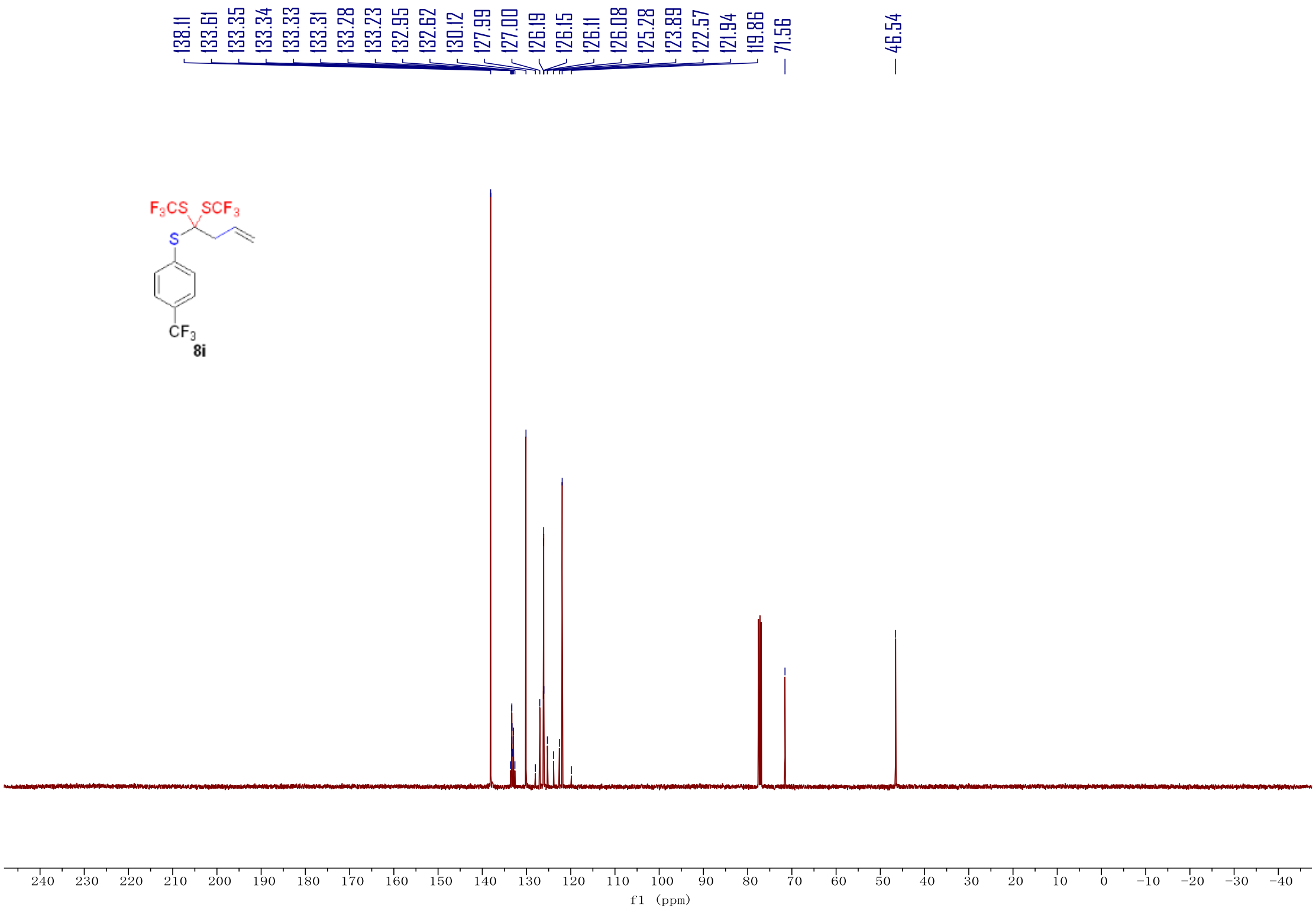

^{1}H NMR Spectrum of **8j** (400 MHz, $CDCl_3$)

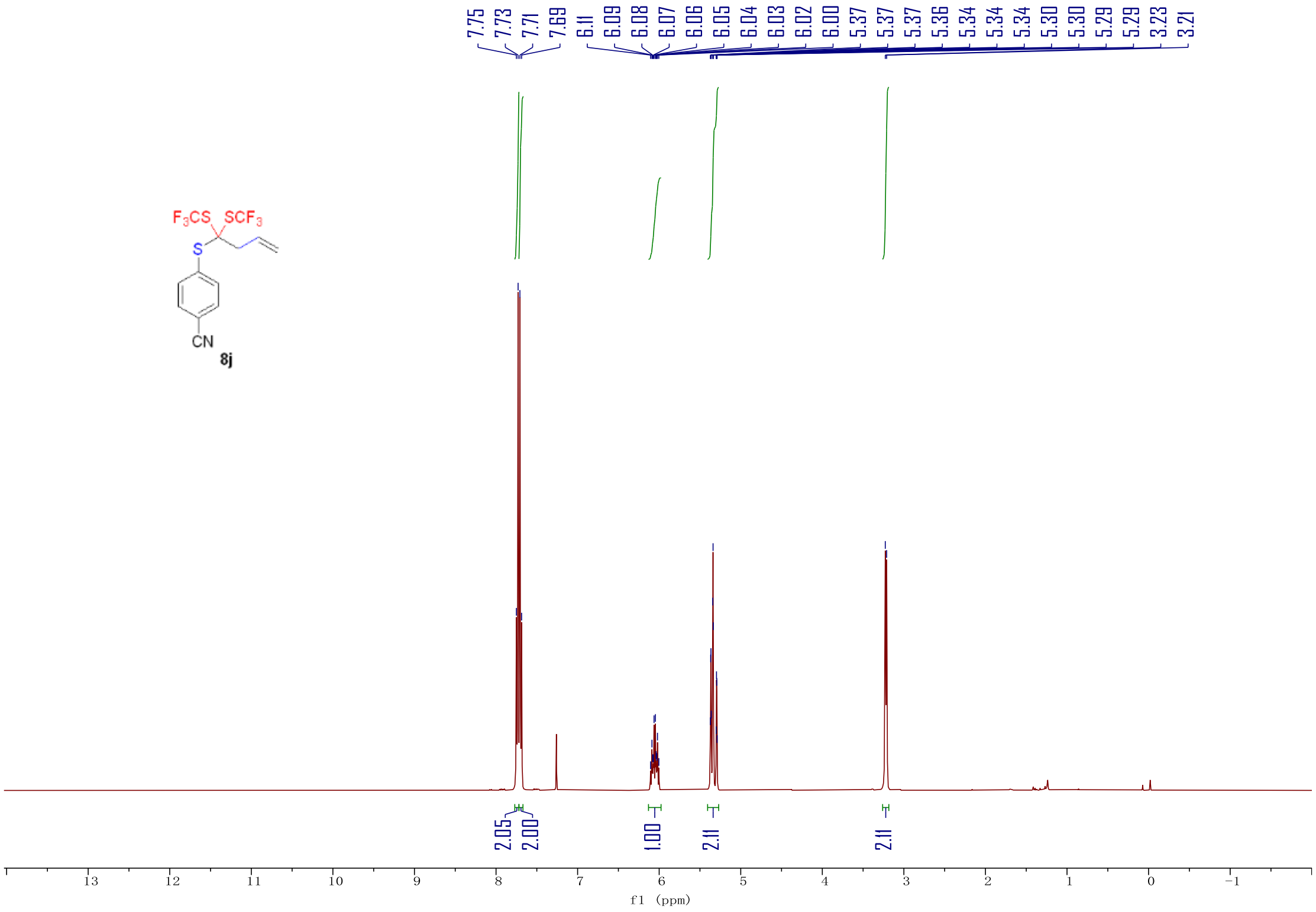

^{19}F NMR Spectrum of **8j** (376 MHz, $CDCl_3$)

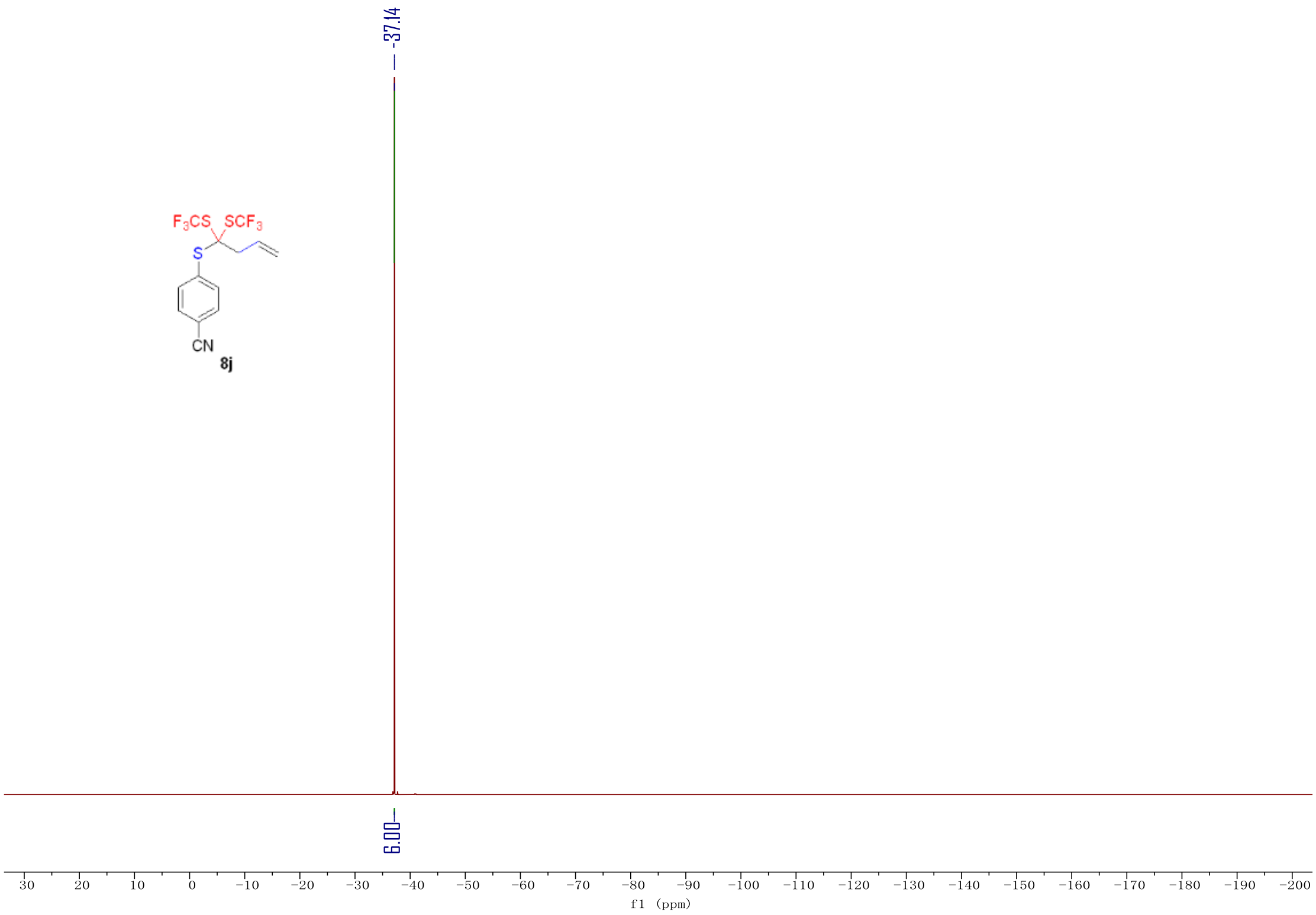

^{13}C NMR Spectrum of **8j** (101 MHz, $CDCl_3$)

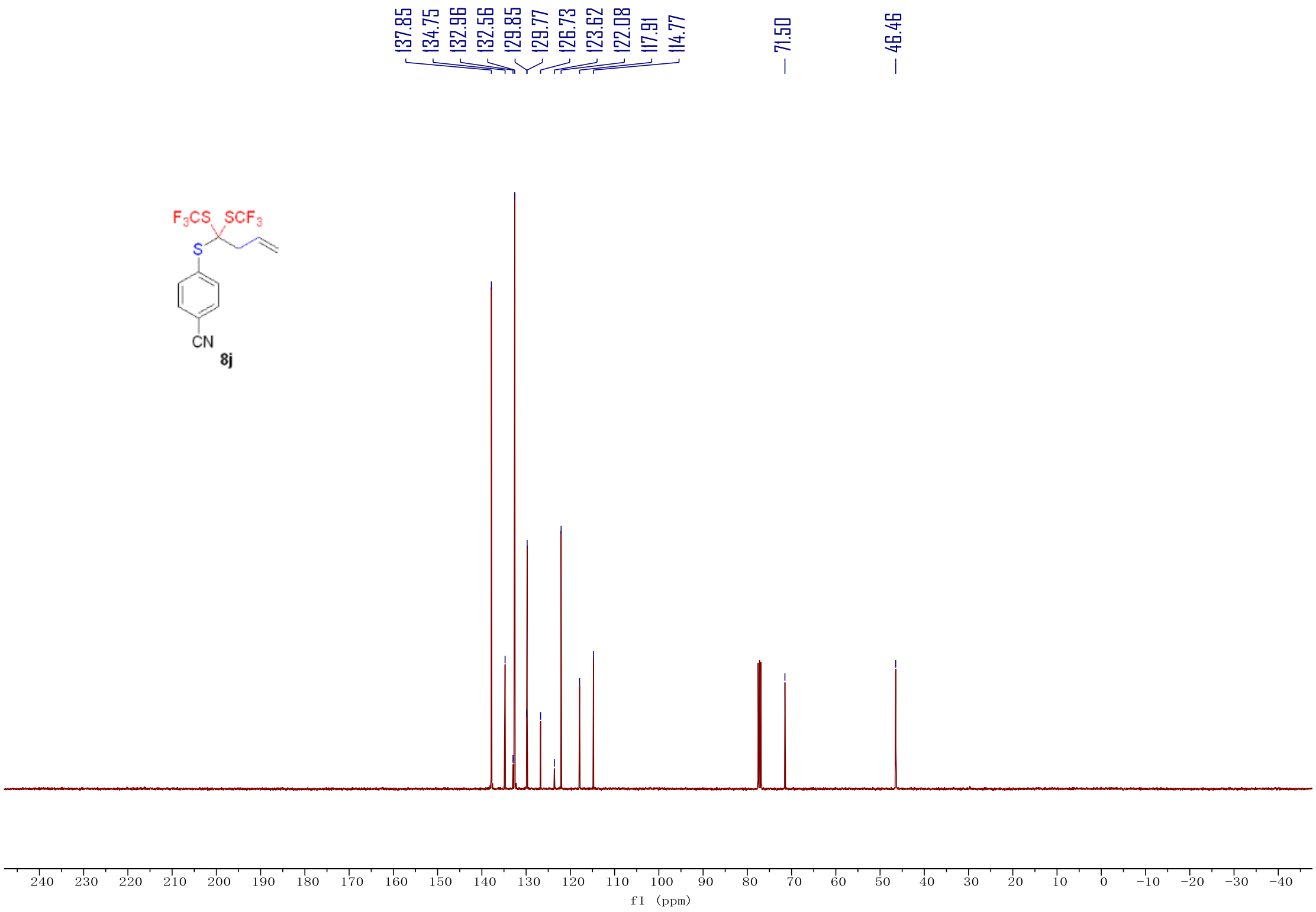

^{1}H NMR Spectrum of **8k** (400 MHz, $CDCl_3$)

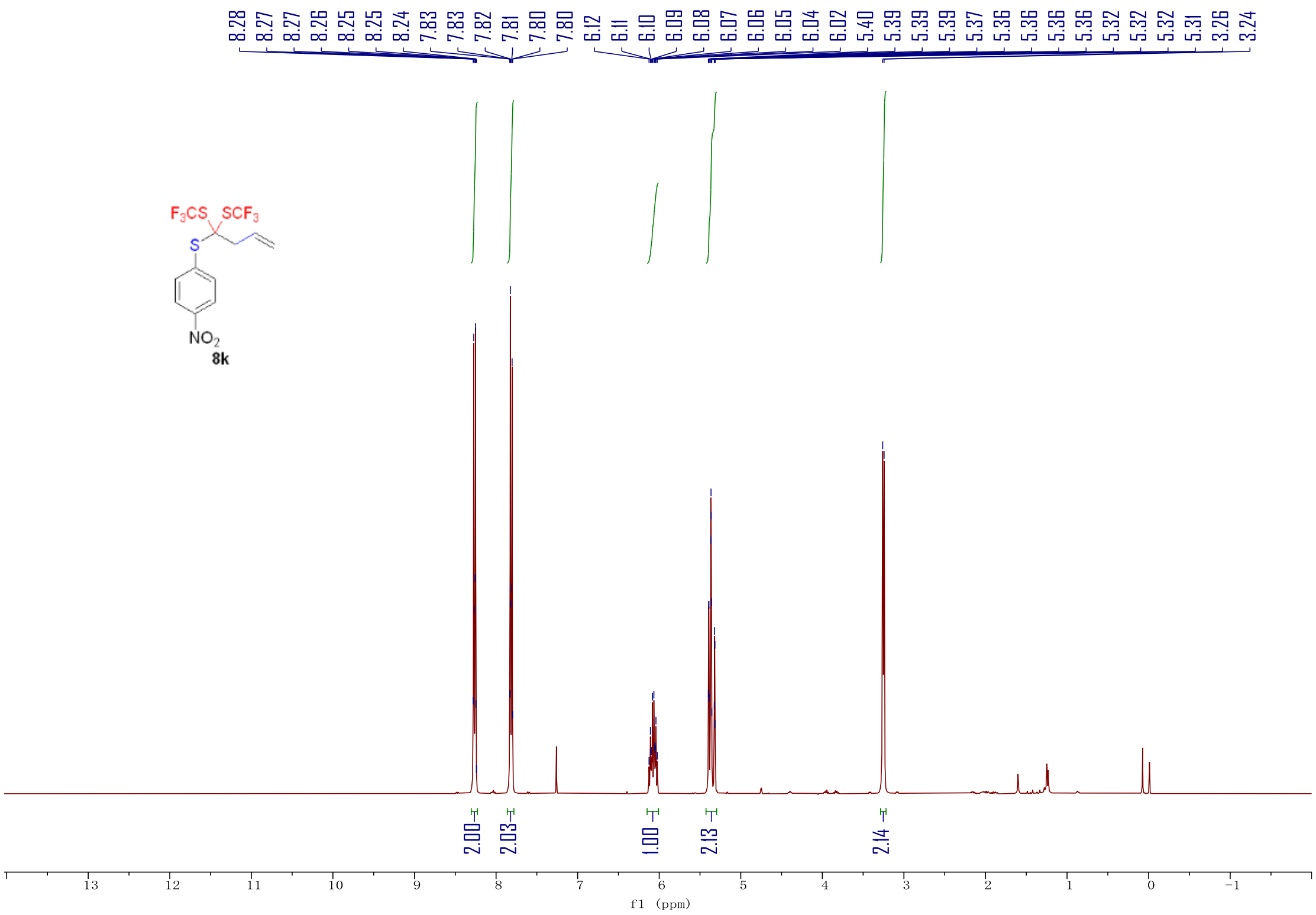

^{19}F NMR Spectrum of **8k** (376 MHz, $CDCl_3$)

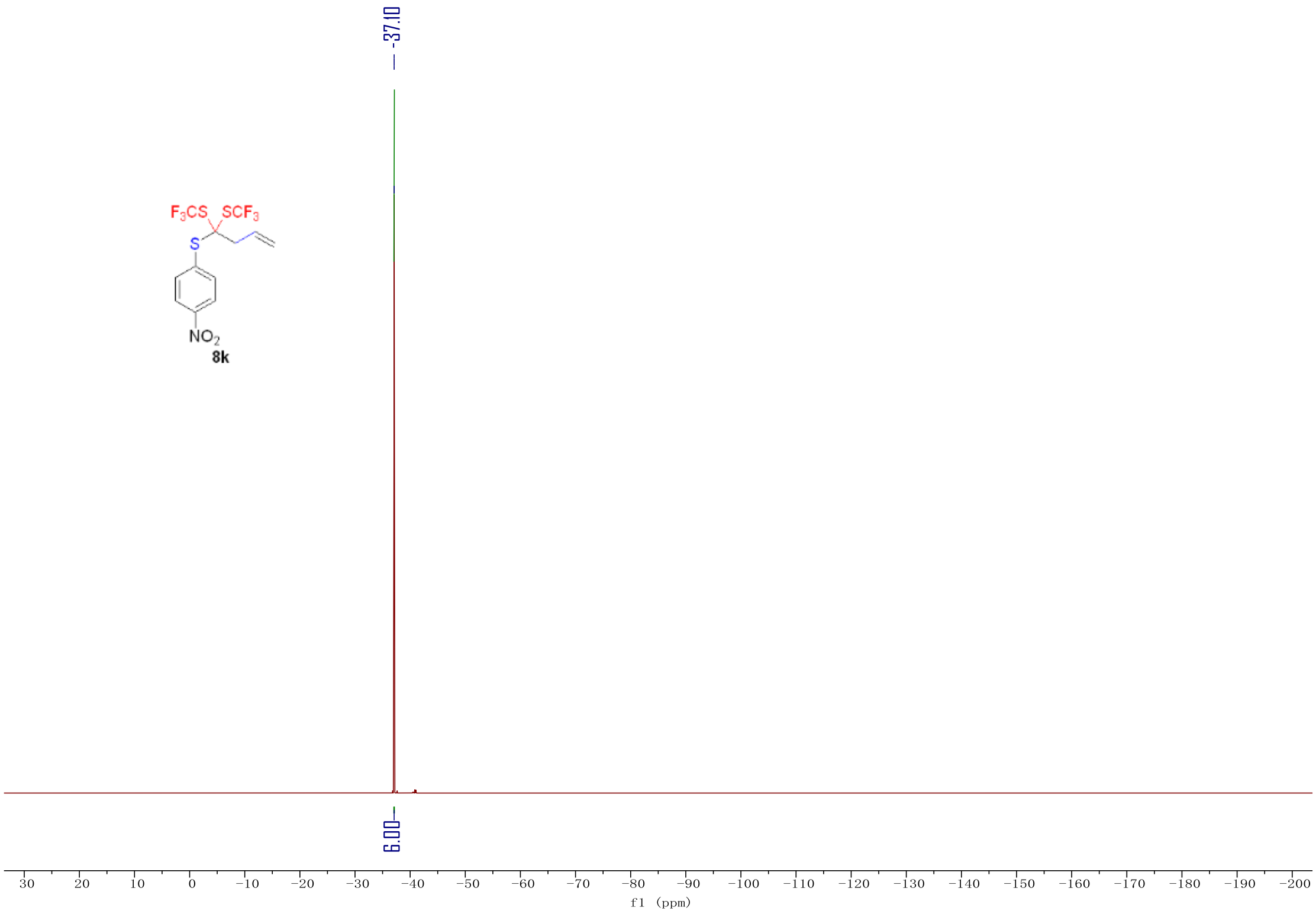

^{13}C NMR Spectrum of **8k** (101 MHz, $CDCl_3$)

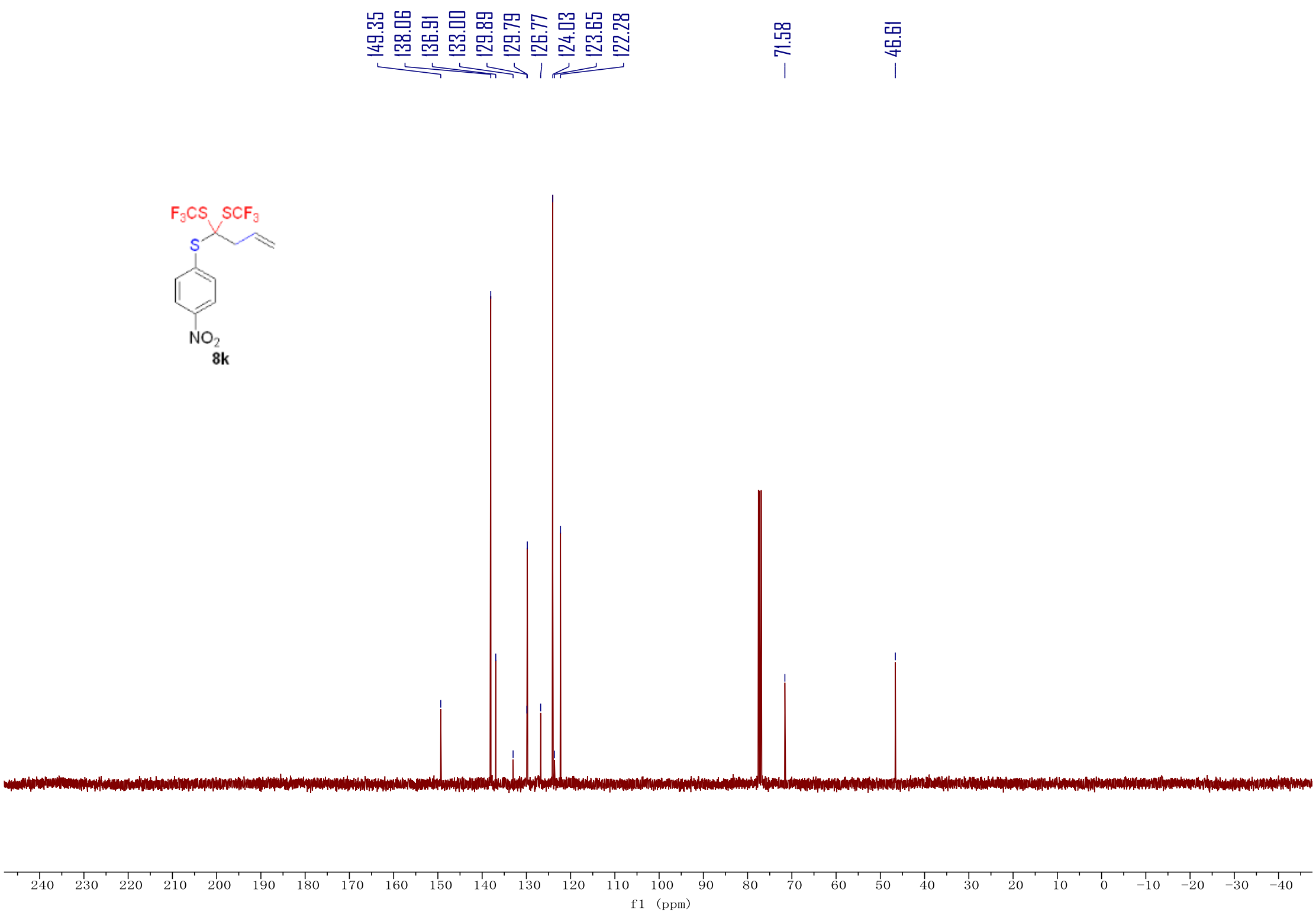

^{1}H NMR Spectrum of **8l** (400 MHz, $CDCl_3$)

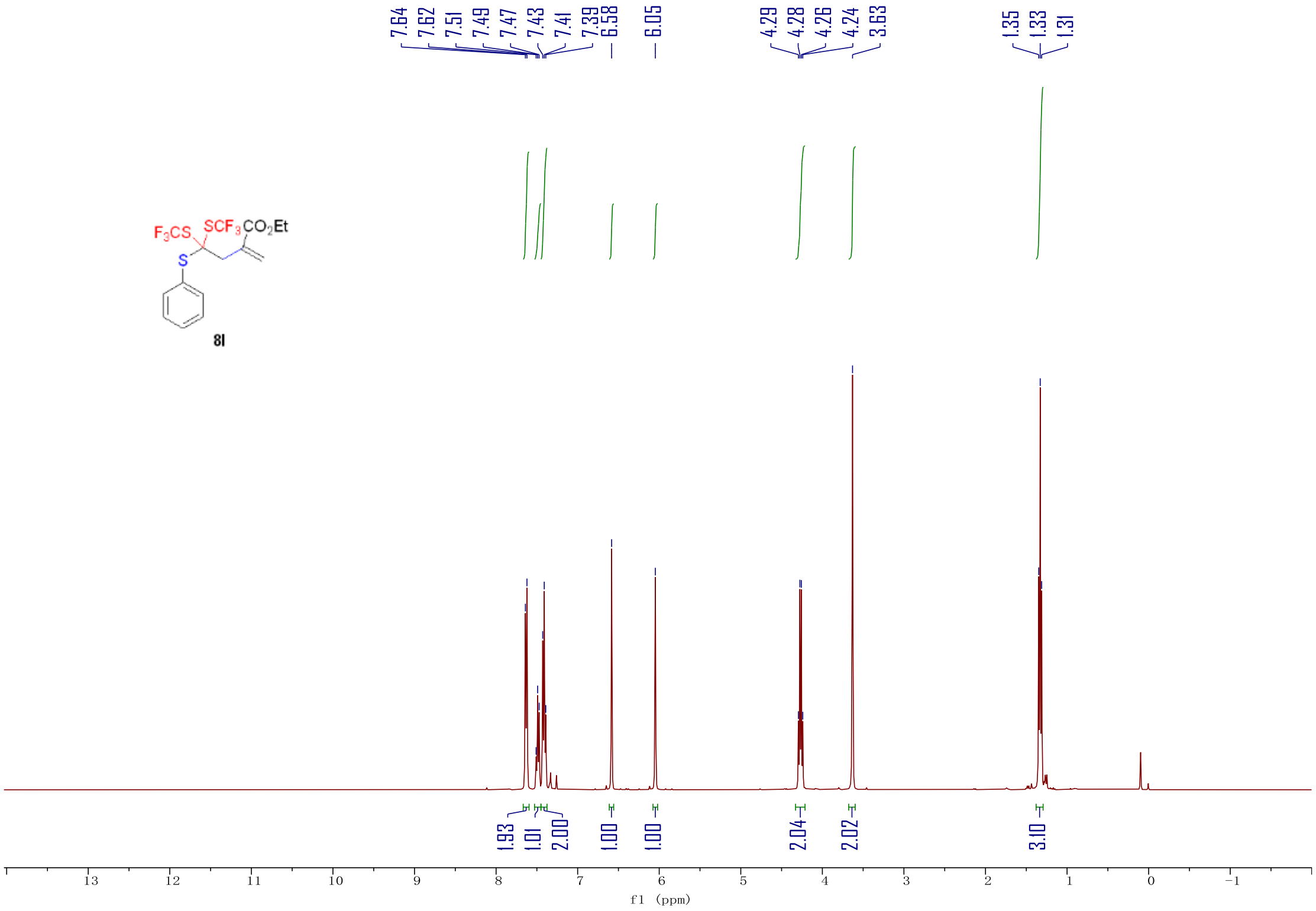

^{19}F NMR Spectrum of **8l** (376 MHz, $CDCl_3$)

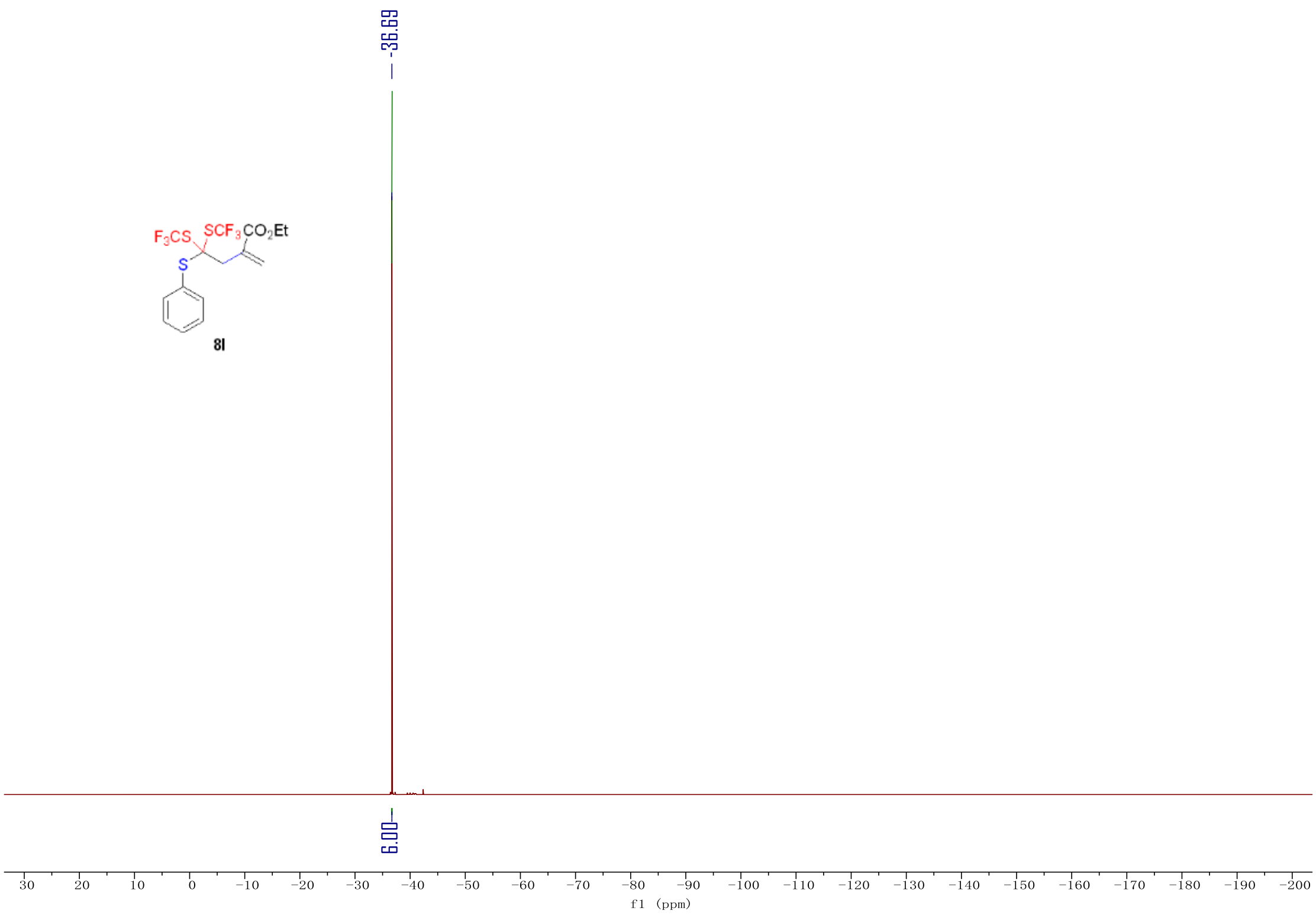

^{13}C NMR Spectrum of **8l** (101 MHz, $CDCl_3$)

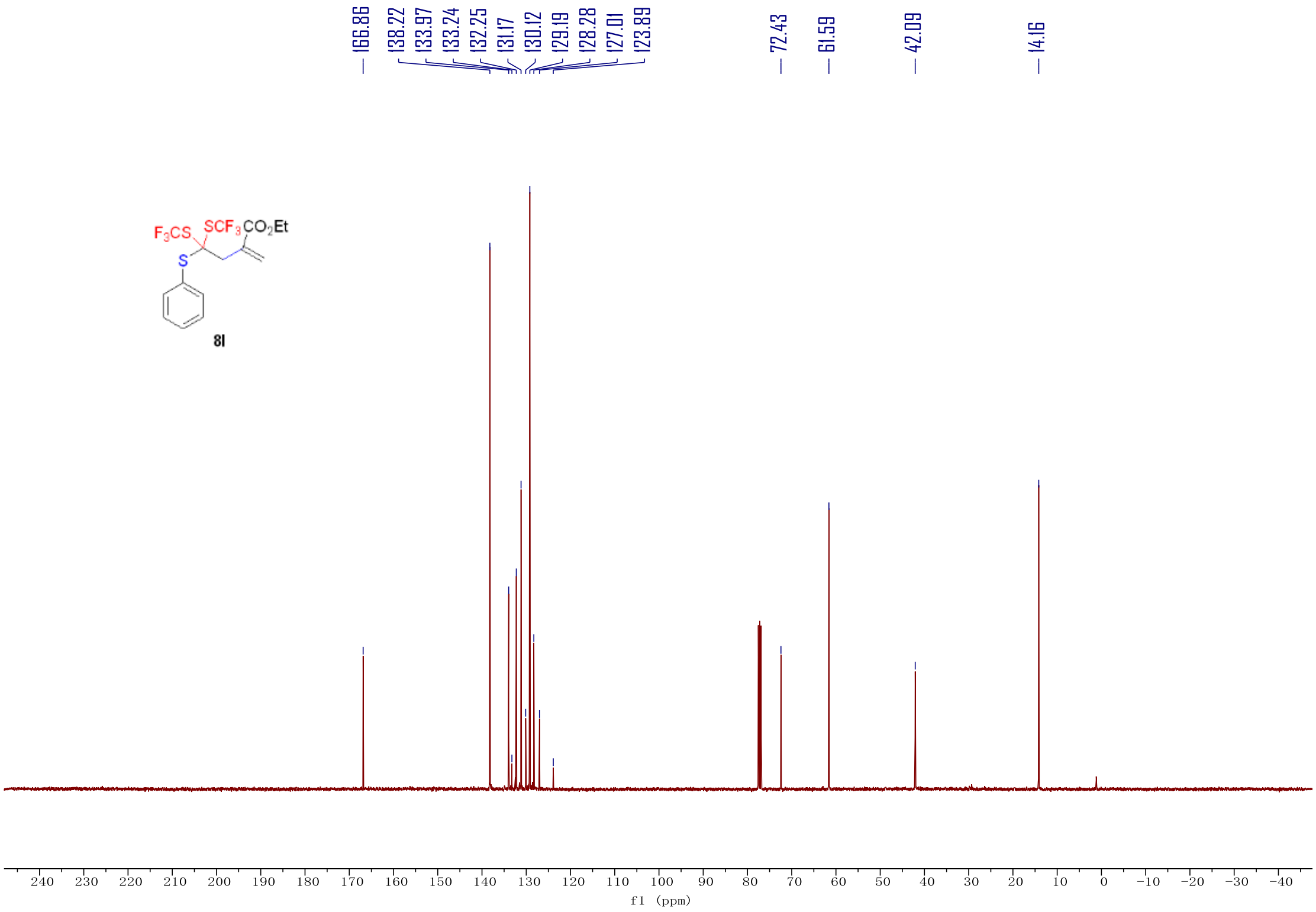

^{1}H NMR Spectrum of **8m** (400 MHz, $CDCl_3$)

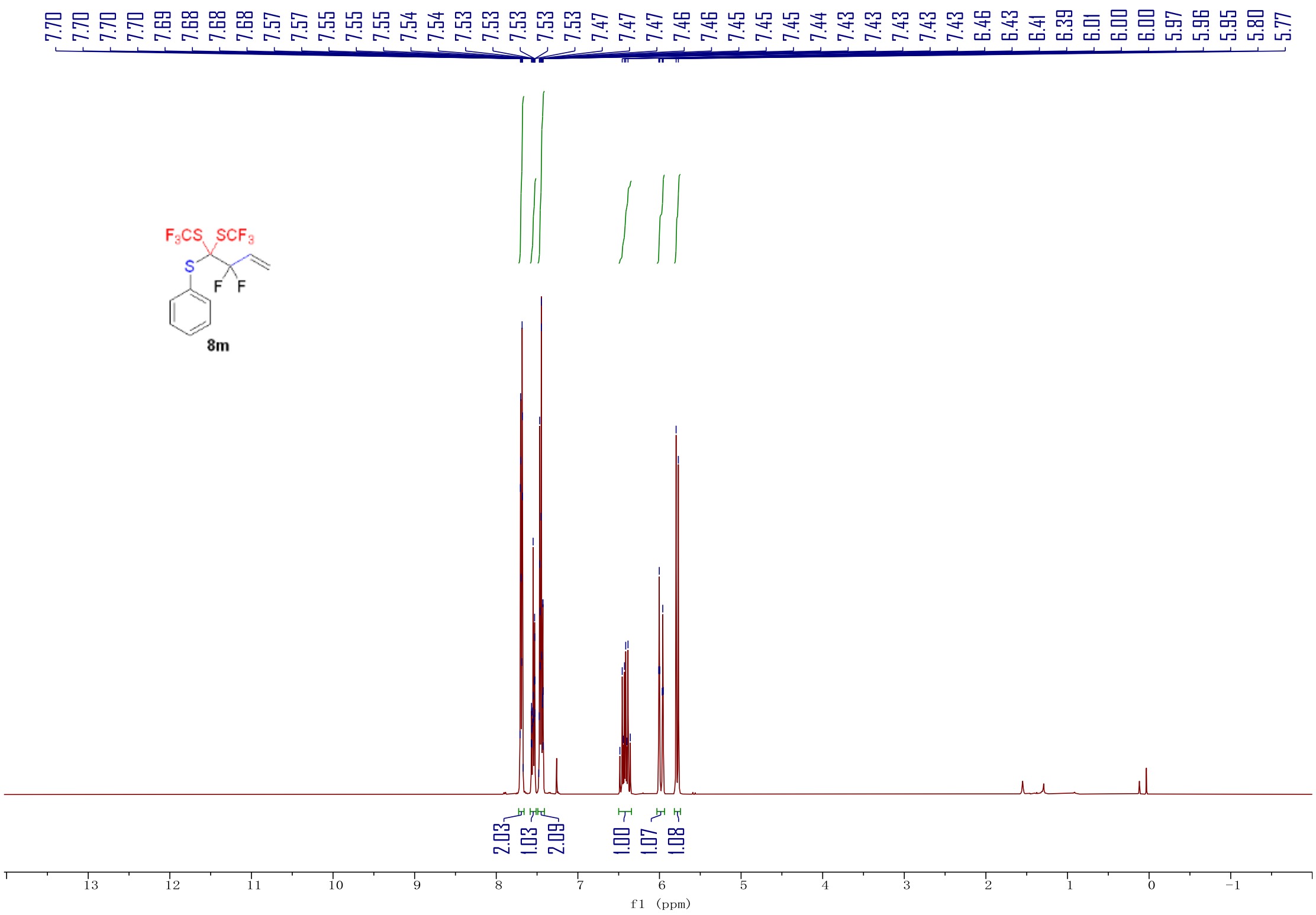

^{19}F NMR Spectrum of **8m** (376 MHz, $CDCl_3$)

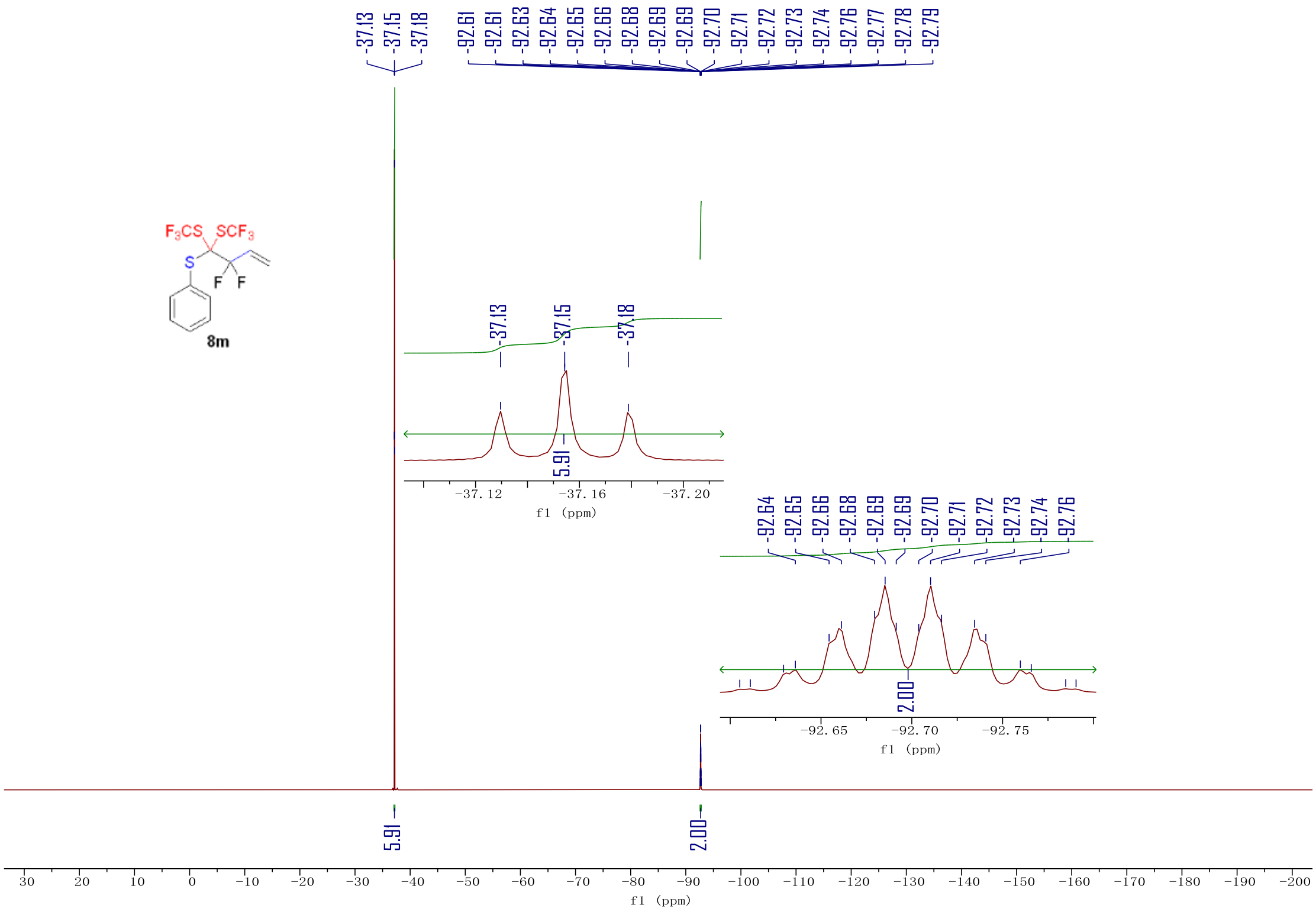

^{13}C NMR Spectrum of **8m** (101 MHz, $CDCl_3$)

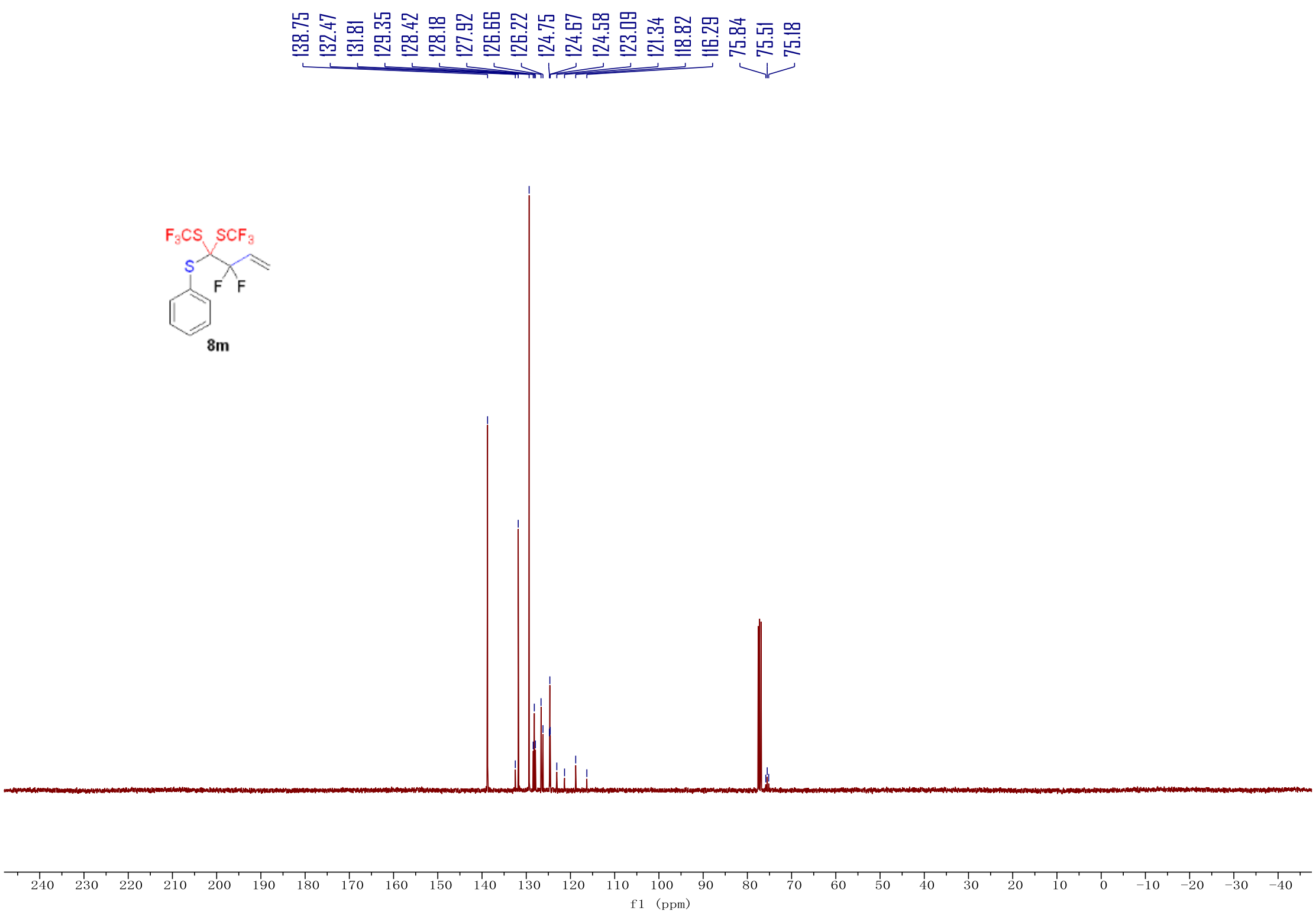

^{1}H NMR Spectrum of **8n** (400 MHz, $CDCl_3$)

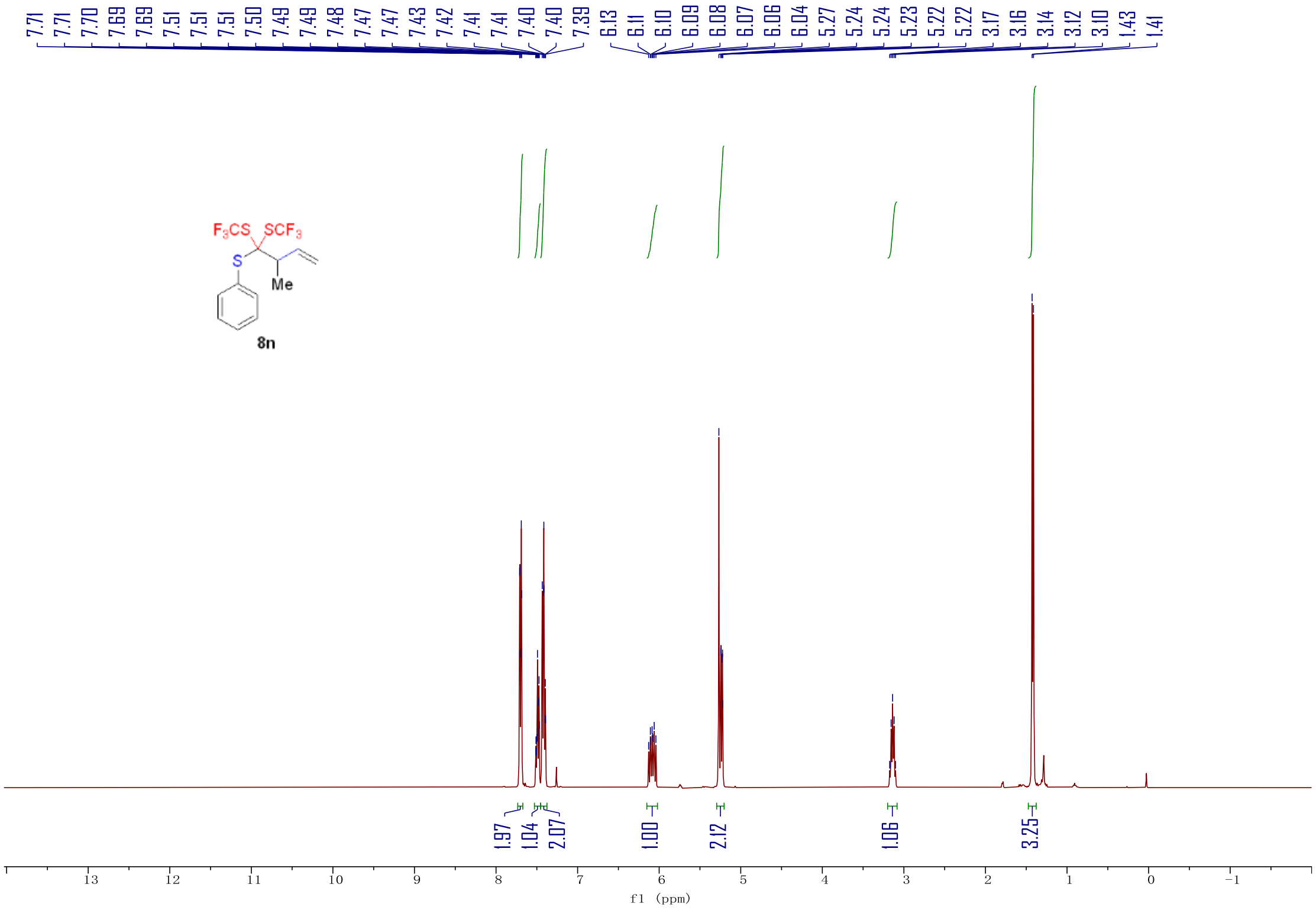

^{19}F NMR Spectrum of **8n** (376 MHz, $CDCl_3$)

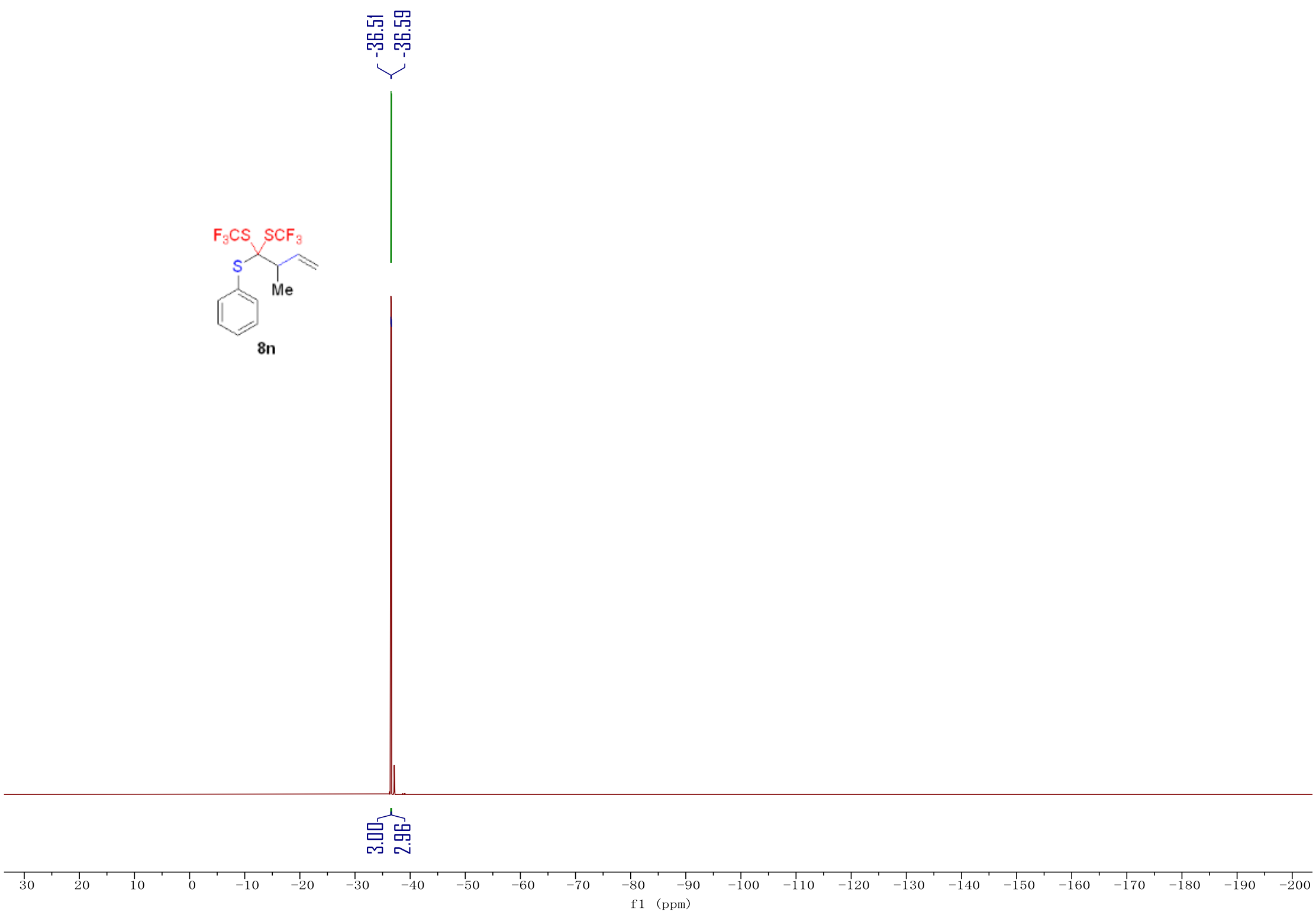

^{13}C NMR Spectrum of **8n** (101 MHz, $CDCl_3$)

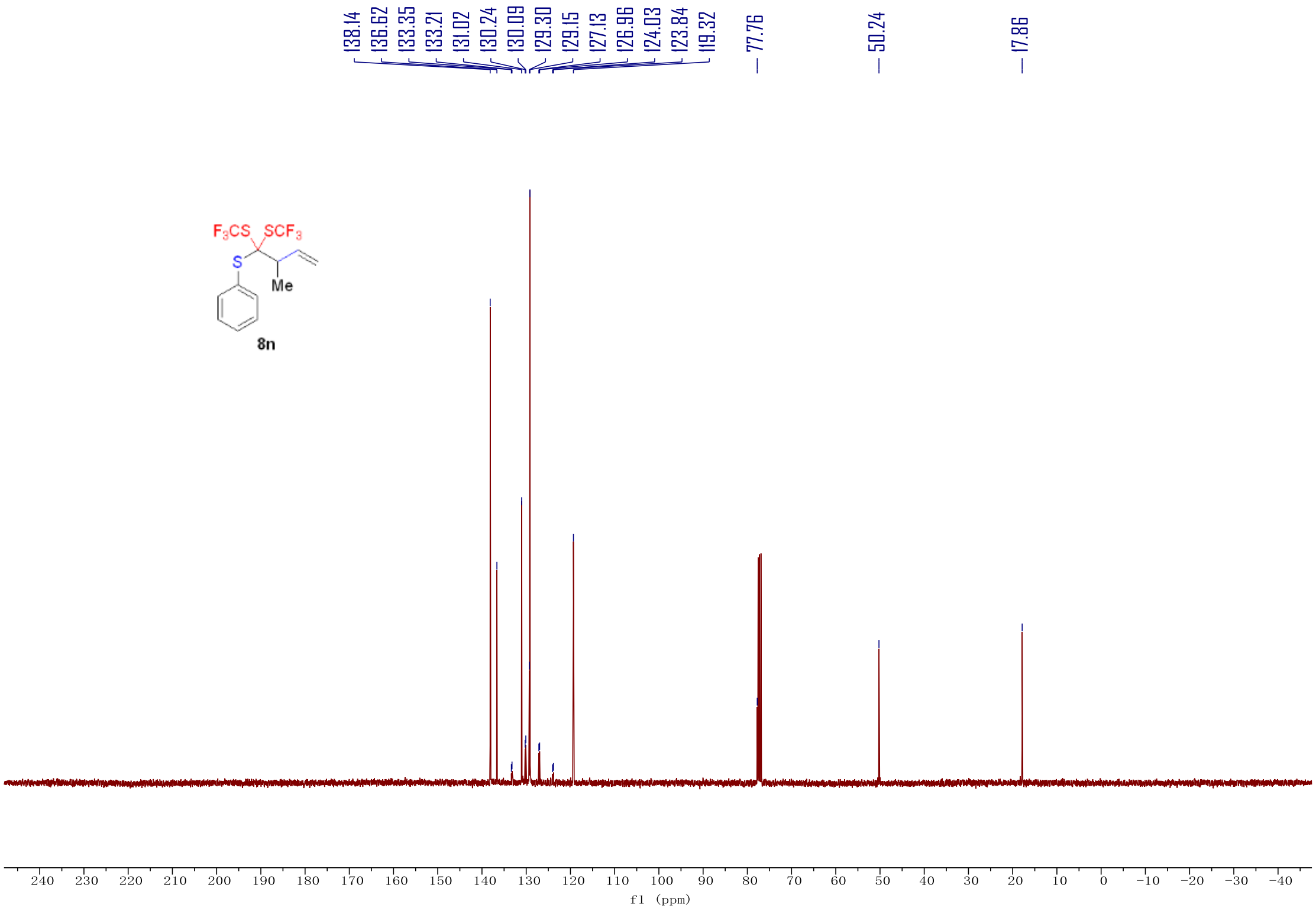

^{1}H NMR Spectrum of **8o** (400 MHz, $CDCl_3$)

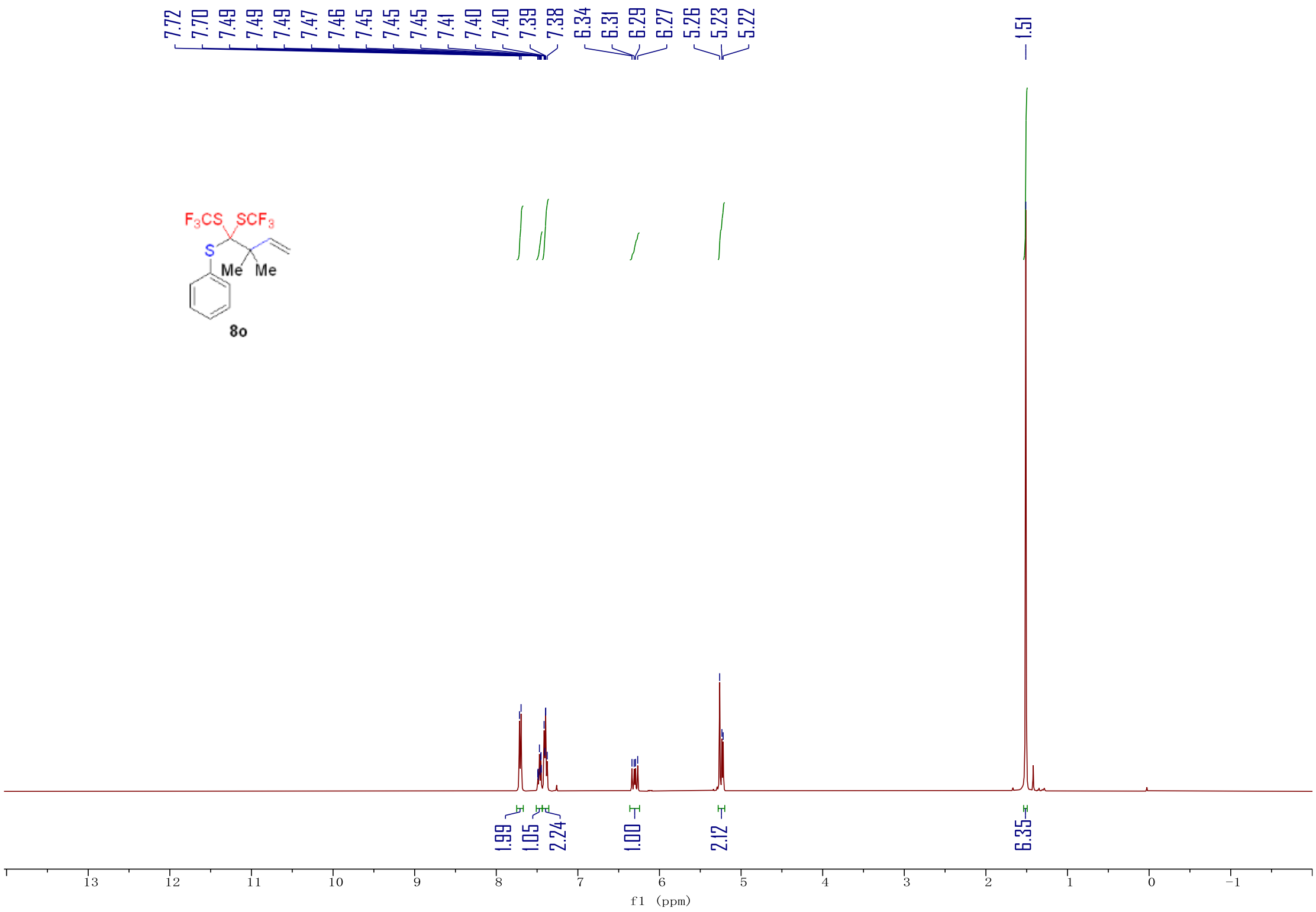

^{19}**F NMR** Spectrum of **8o** (376 MHz, $CDCl_3$)

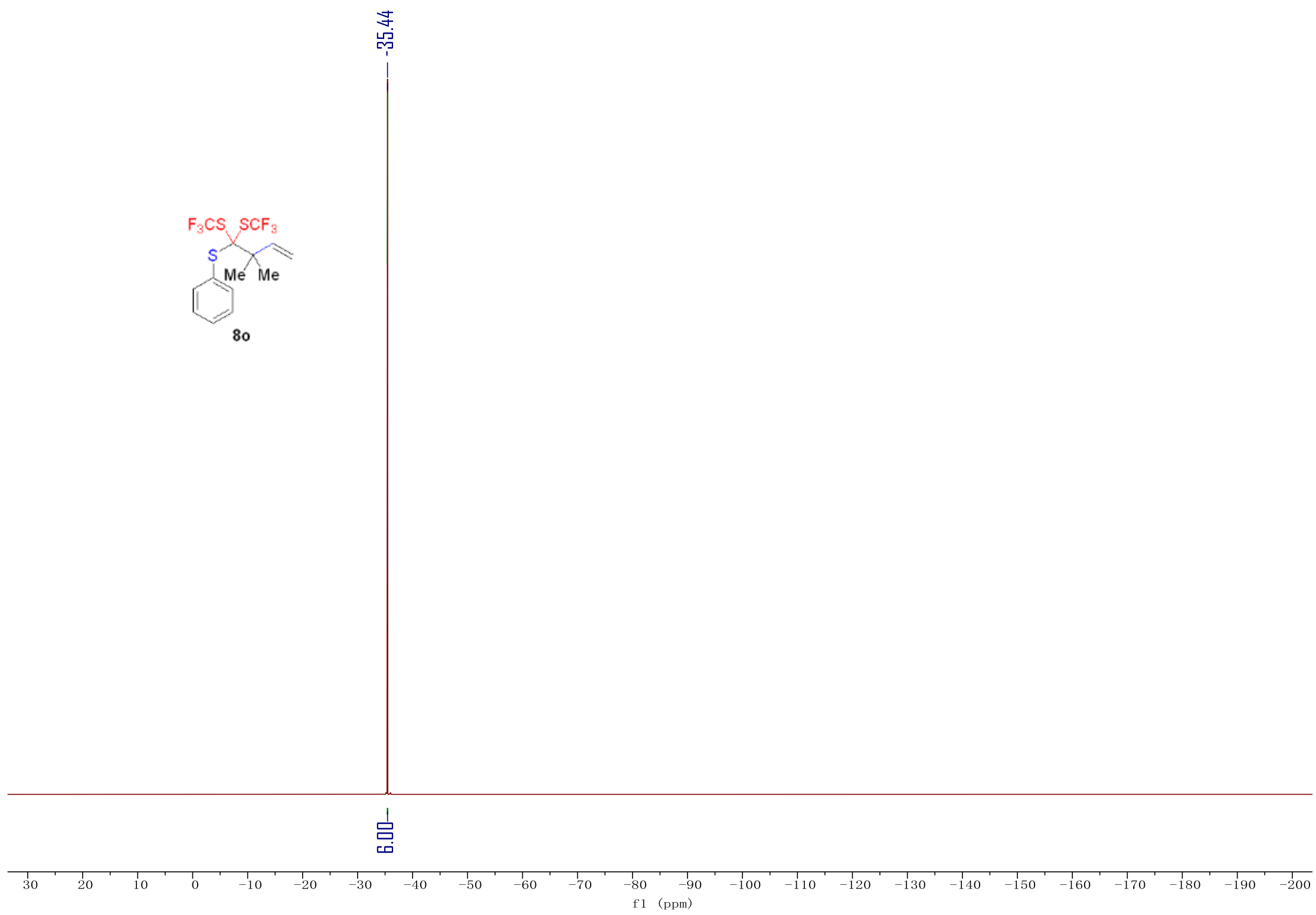

^{13}**C NMR** Spectrum of **8o** (101 MHz, $CDCl_3$)

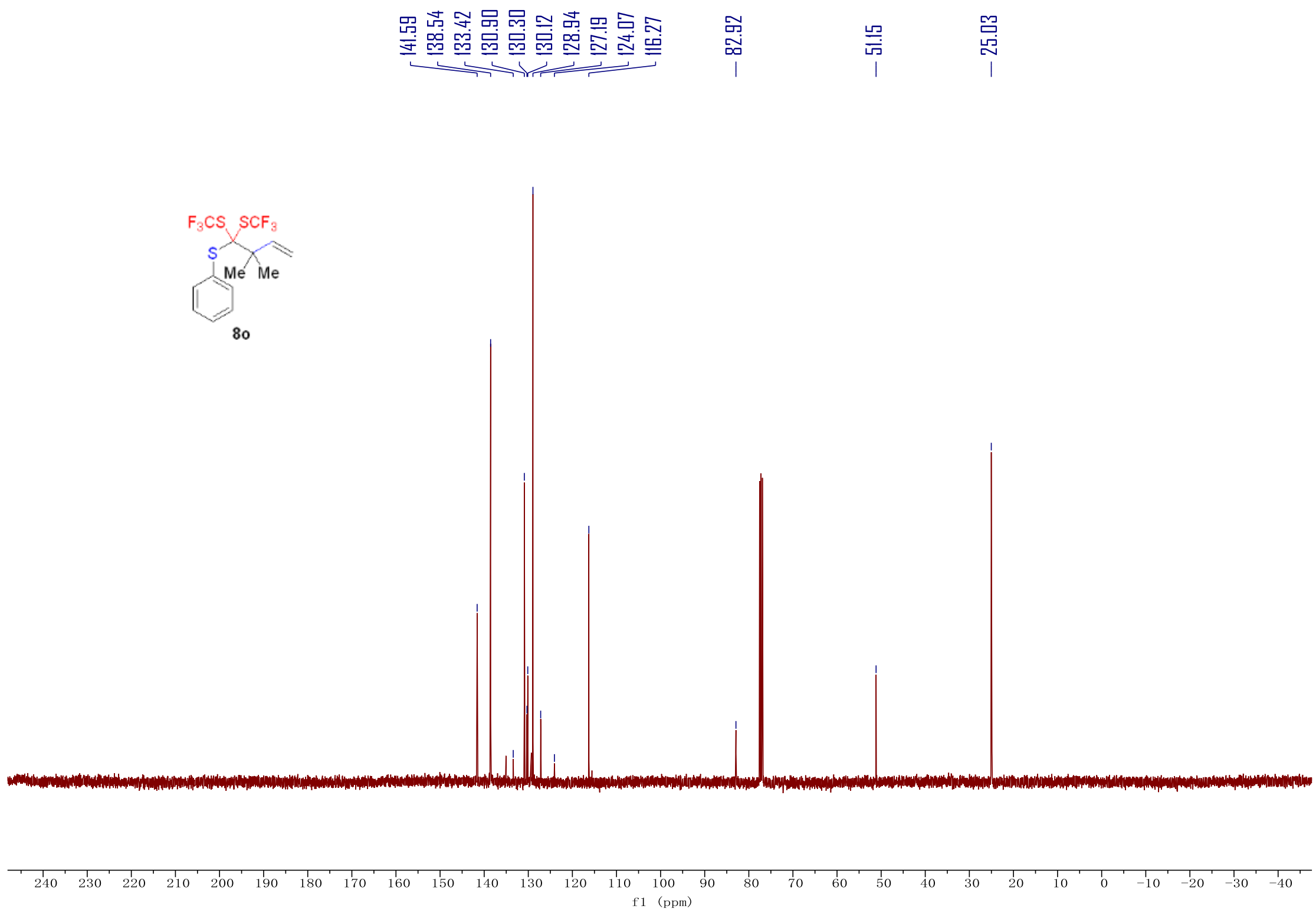

^{1}H NMR Spectrum of **8p** (400 MHz, $CDCl_3$)

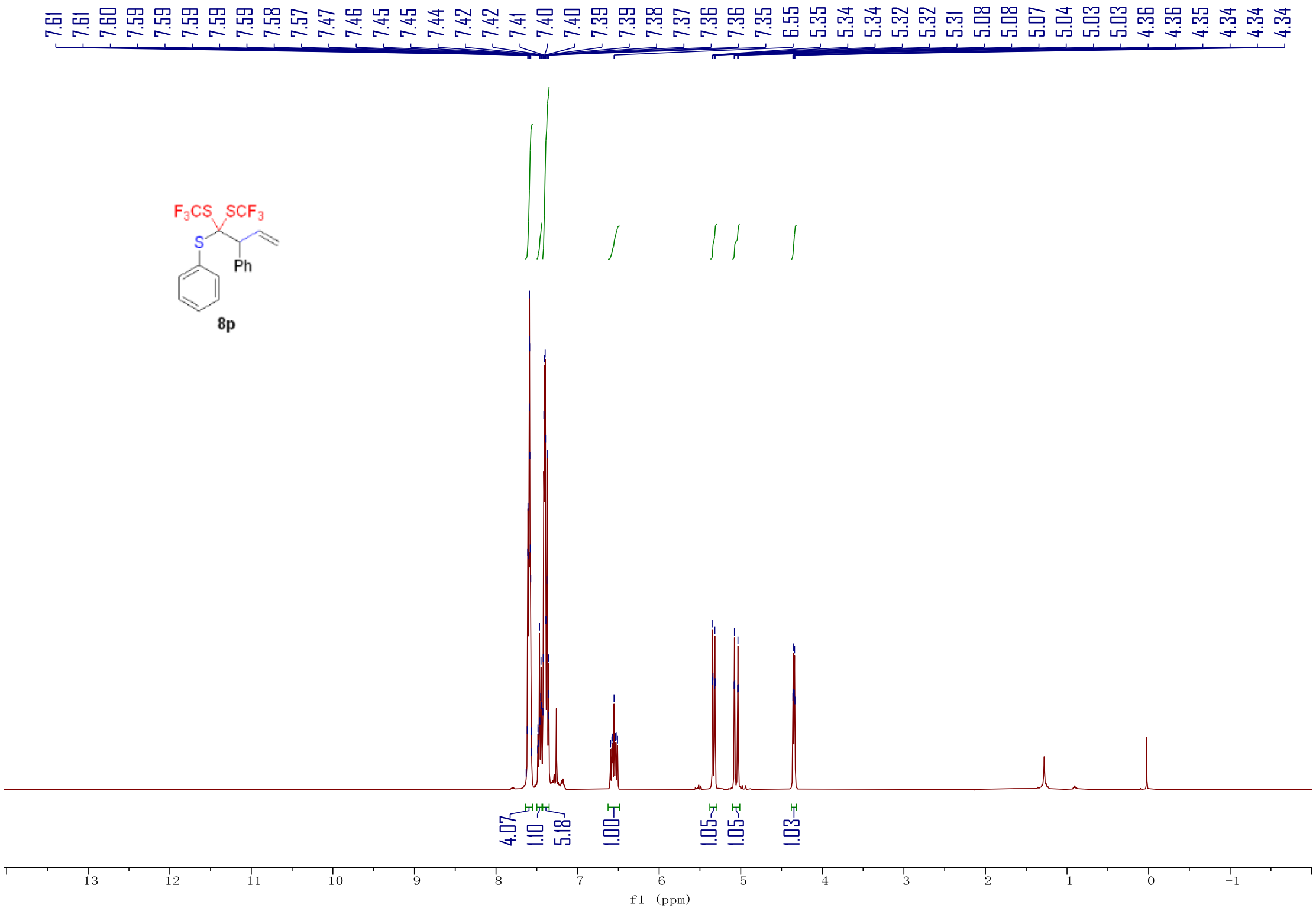

^{19}F NMR Spectrum of **8p** (376 MHz, $CDCl_3$)

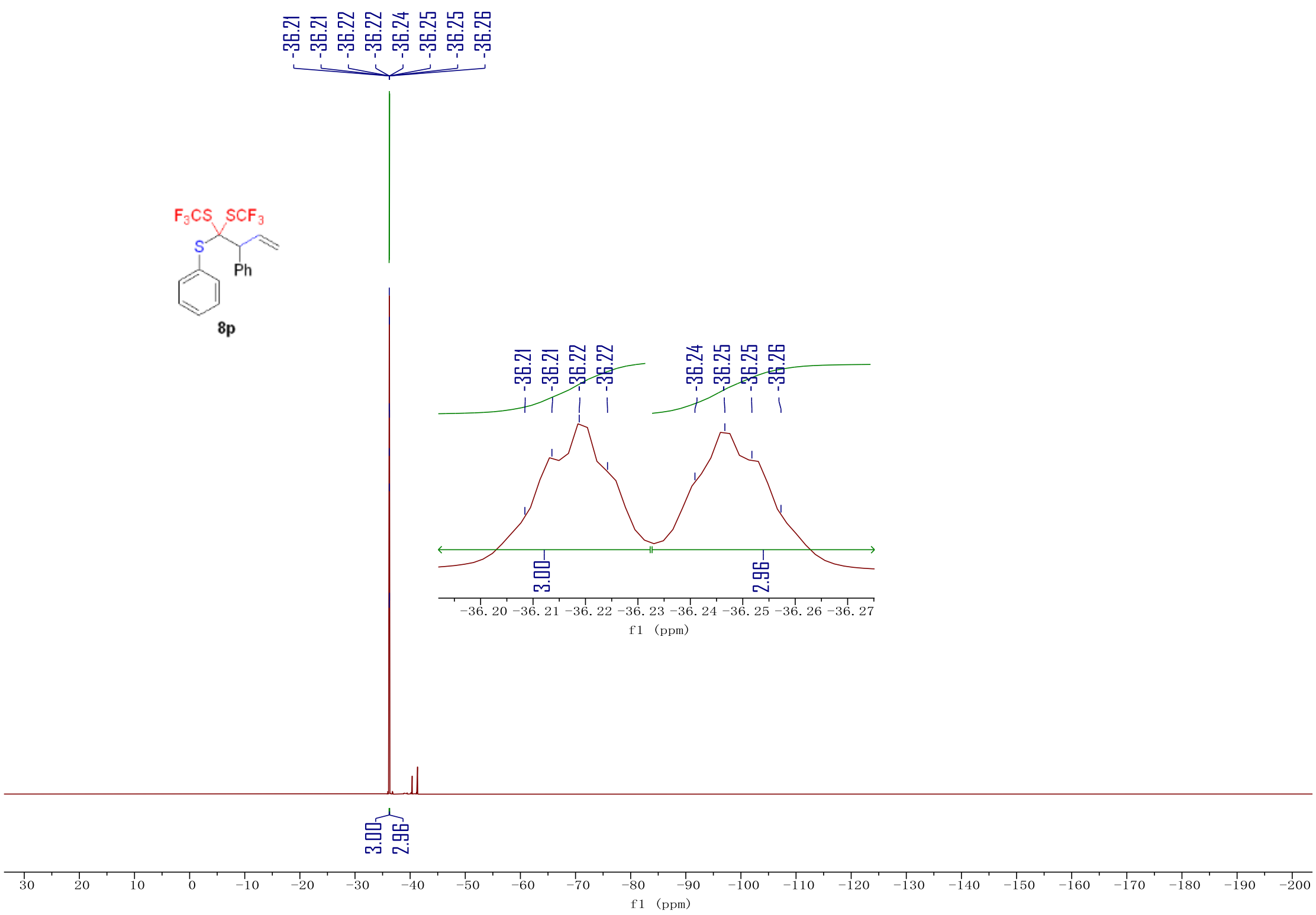

¹³C NMR Spectrum of **8p** (101 MHz, $CDCl_3$)

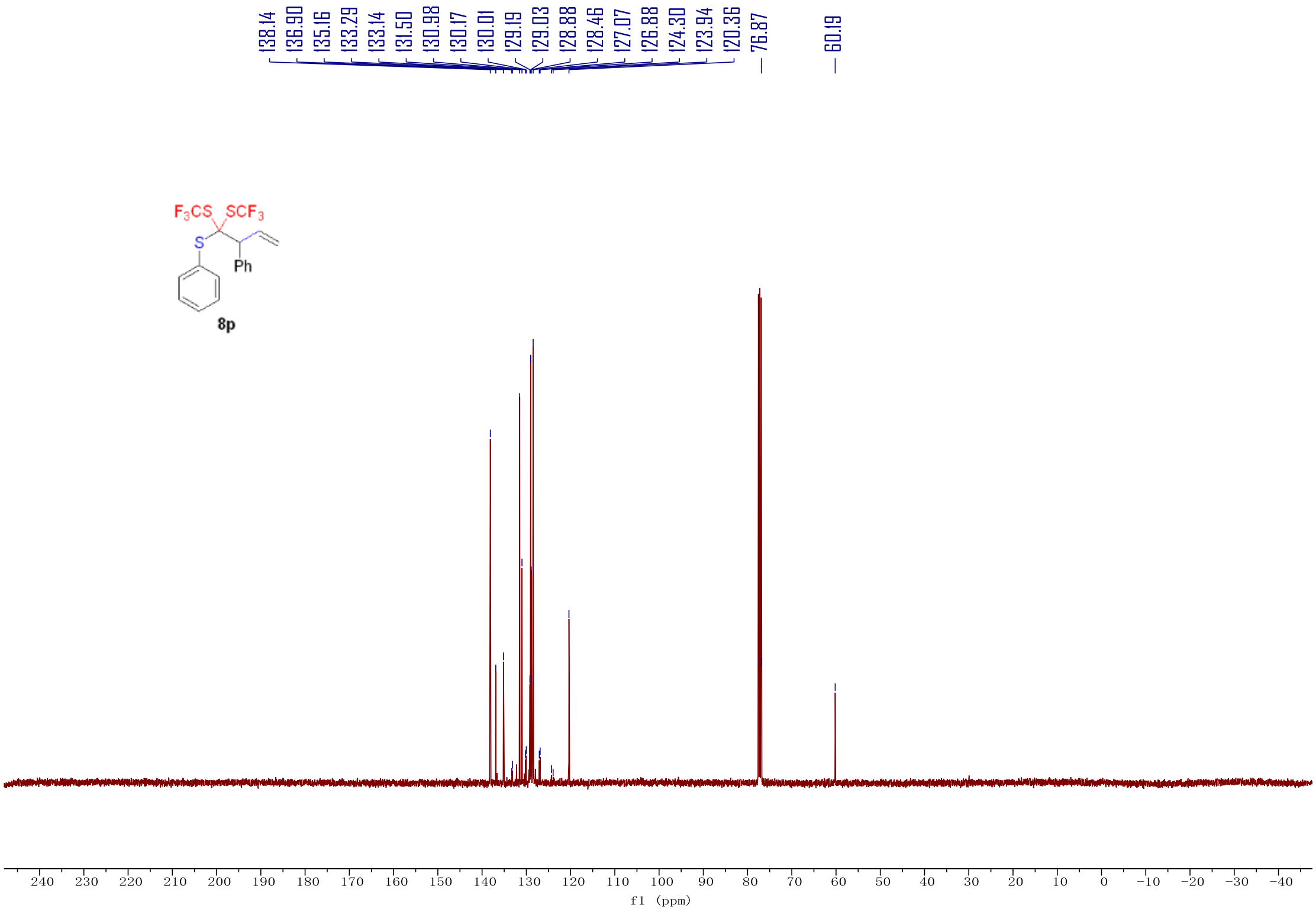

¹H NMR Spectrum of **8q** (400 MHz, $CDCl_3$)

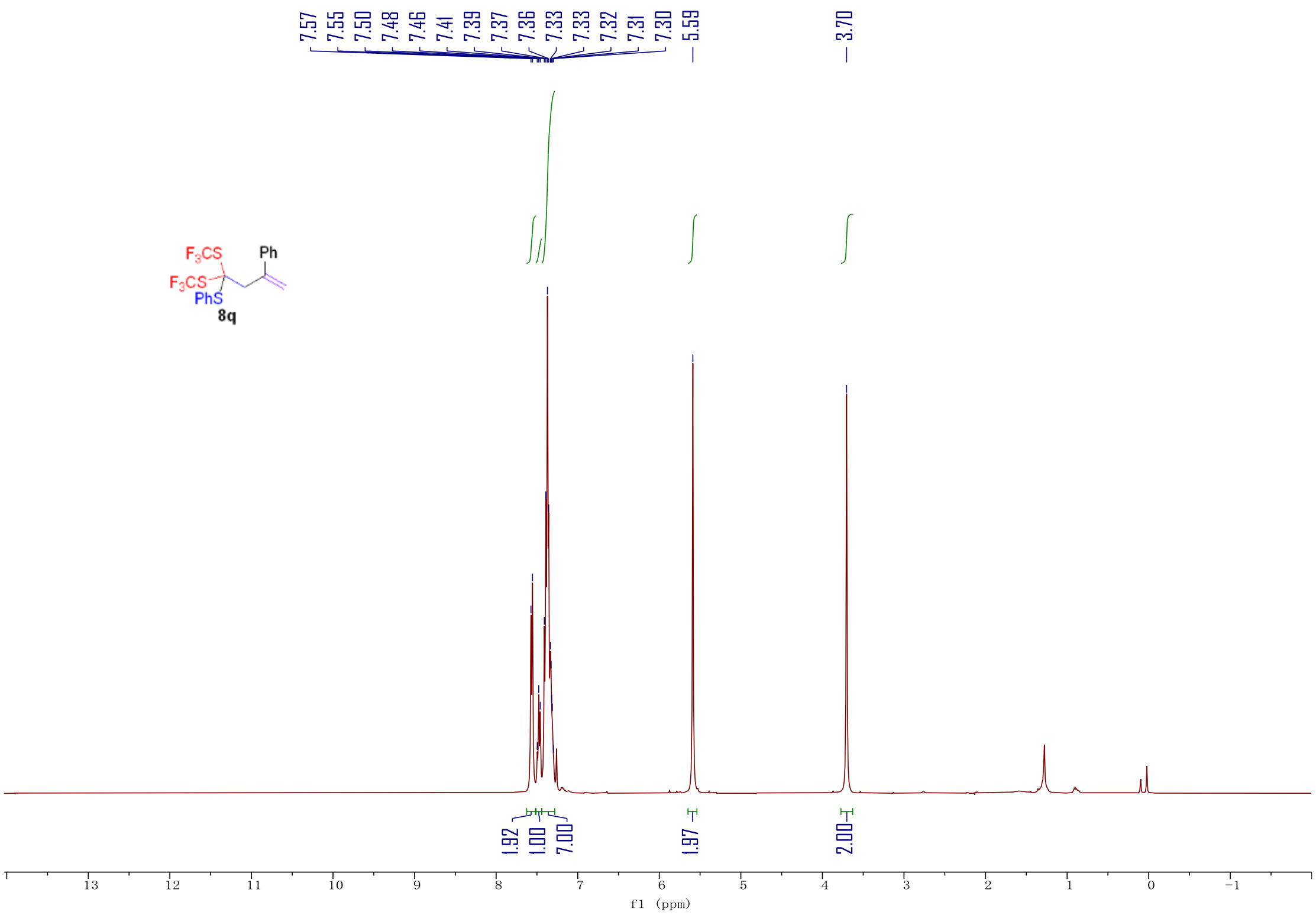

^{19}F NMR Spectrum of **8q** (376 MHz, $CDCl_3$)

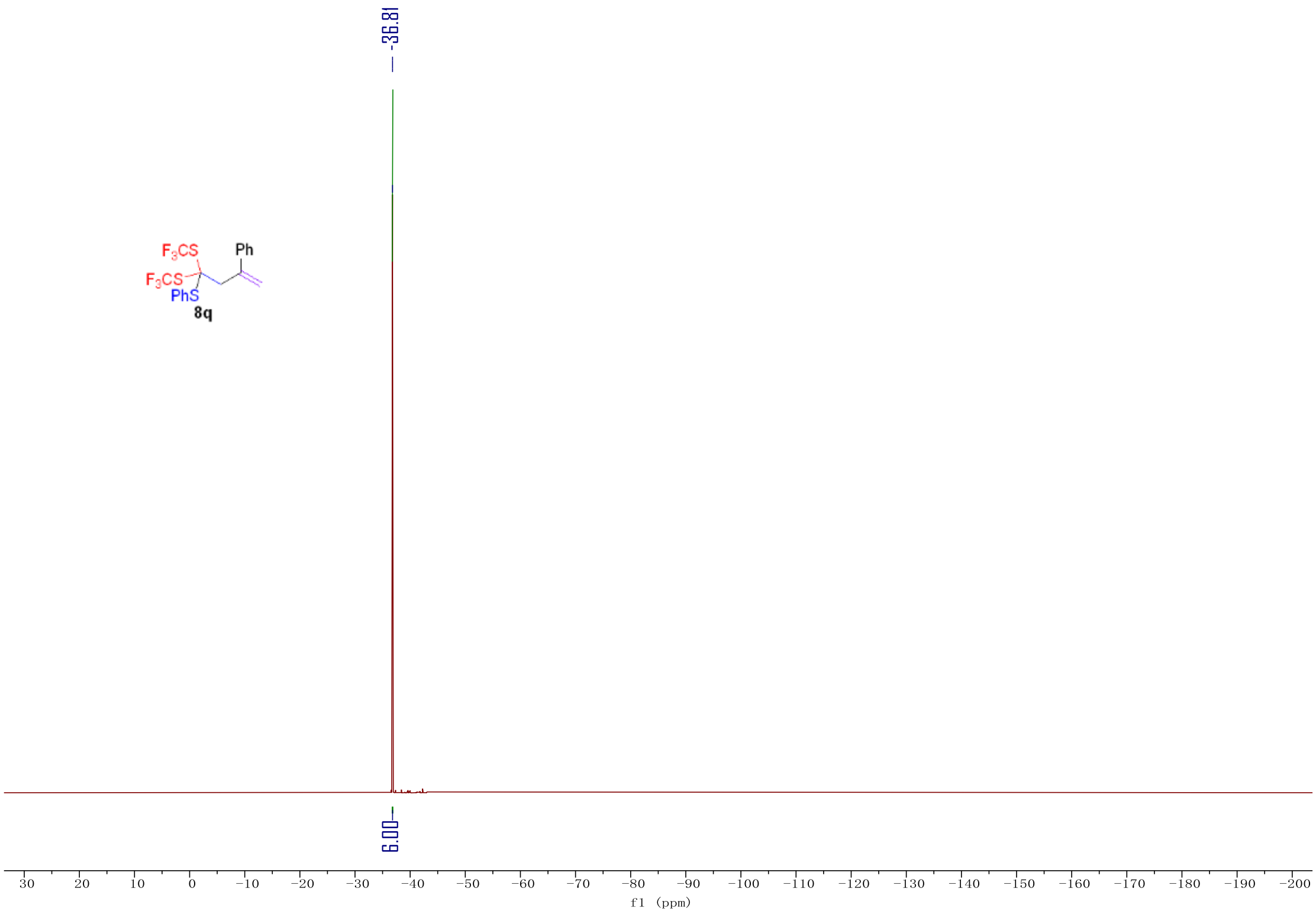

^{13}C NMR Spectrum of **8q** (101 MHz, $CDCl_3$)

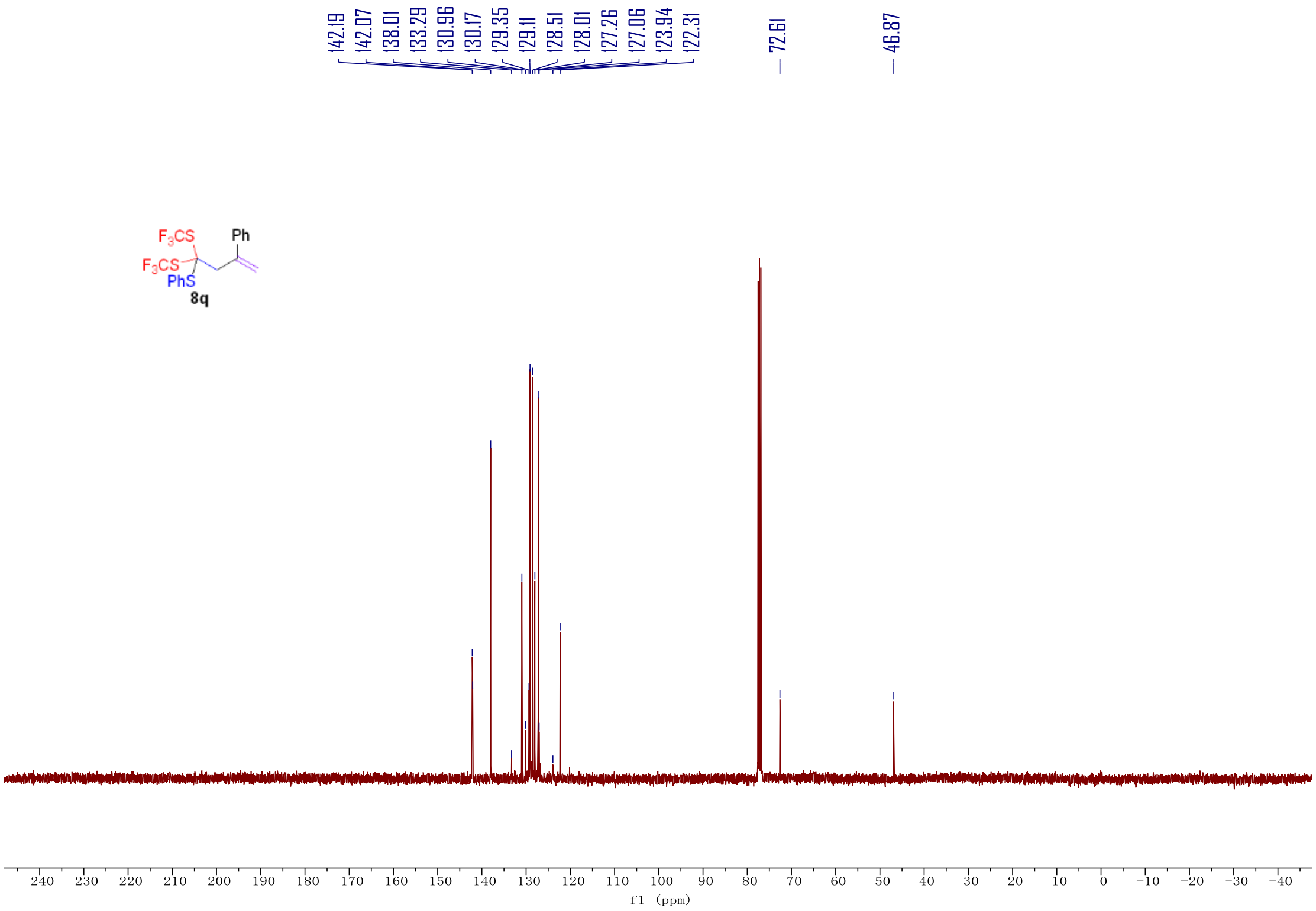

^{1}H **NMR** Spectrum of **8q'** (400 MHz, $CDCl_3$)

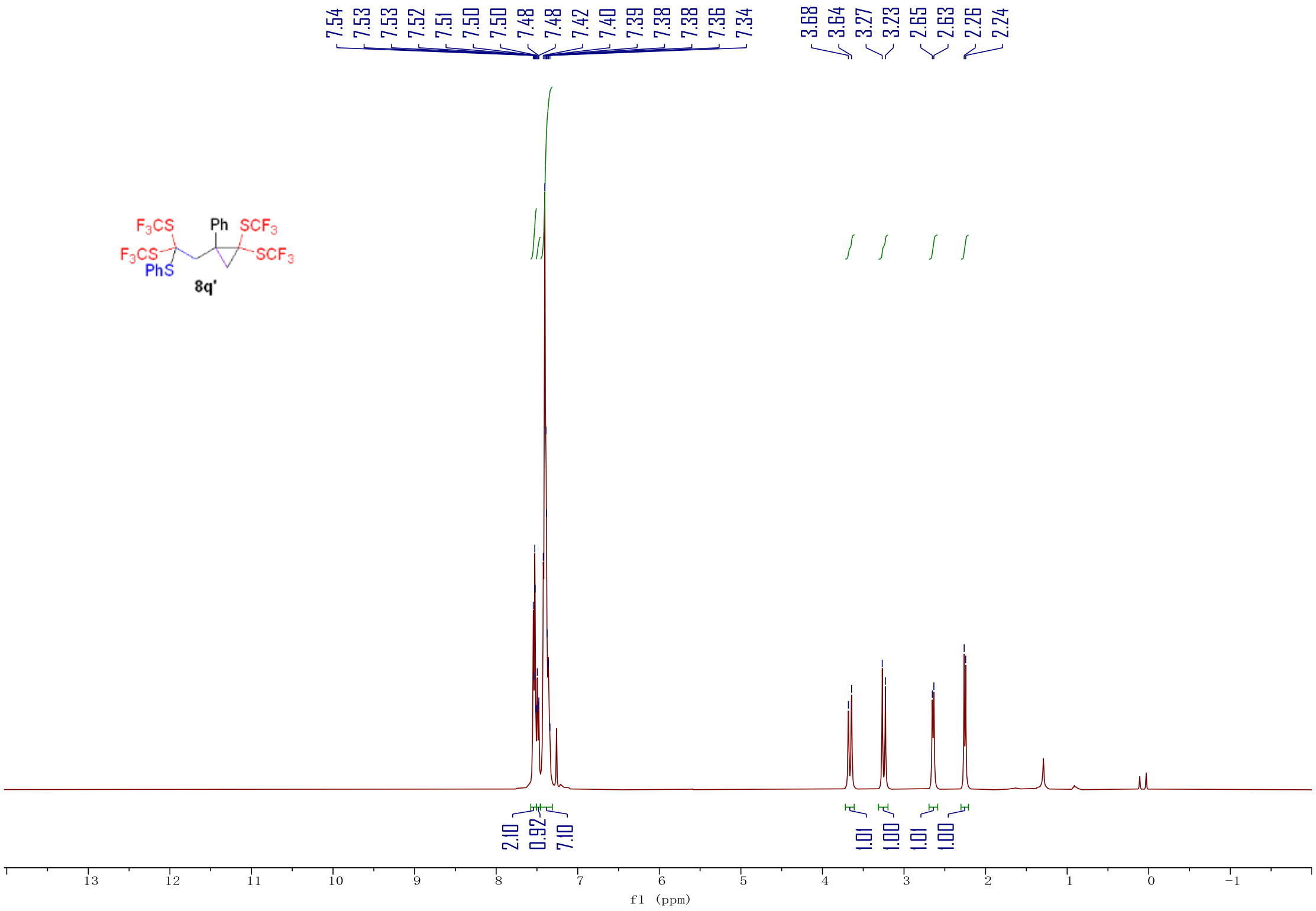

^{19}F **NMR** Spectrum of **8q'** (376 MHz, $CDCl_3$)

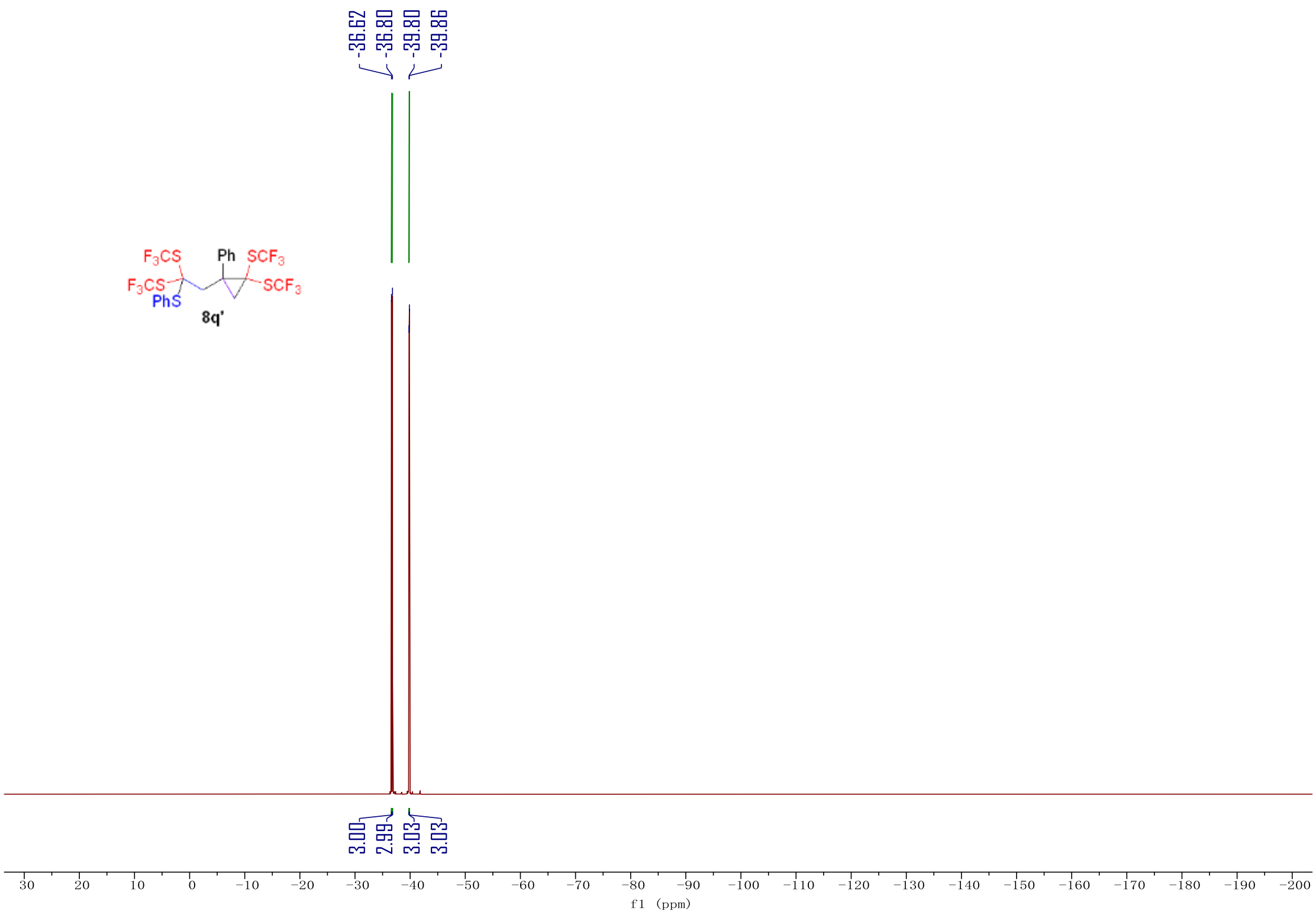

^{13}C NMR Spectrum of **8q'** (101 MHz, $CDCl_3$)

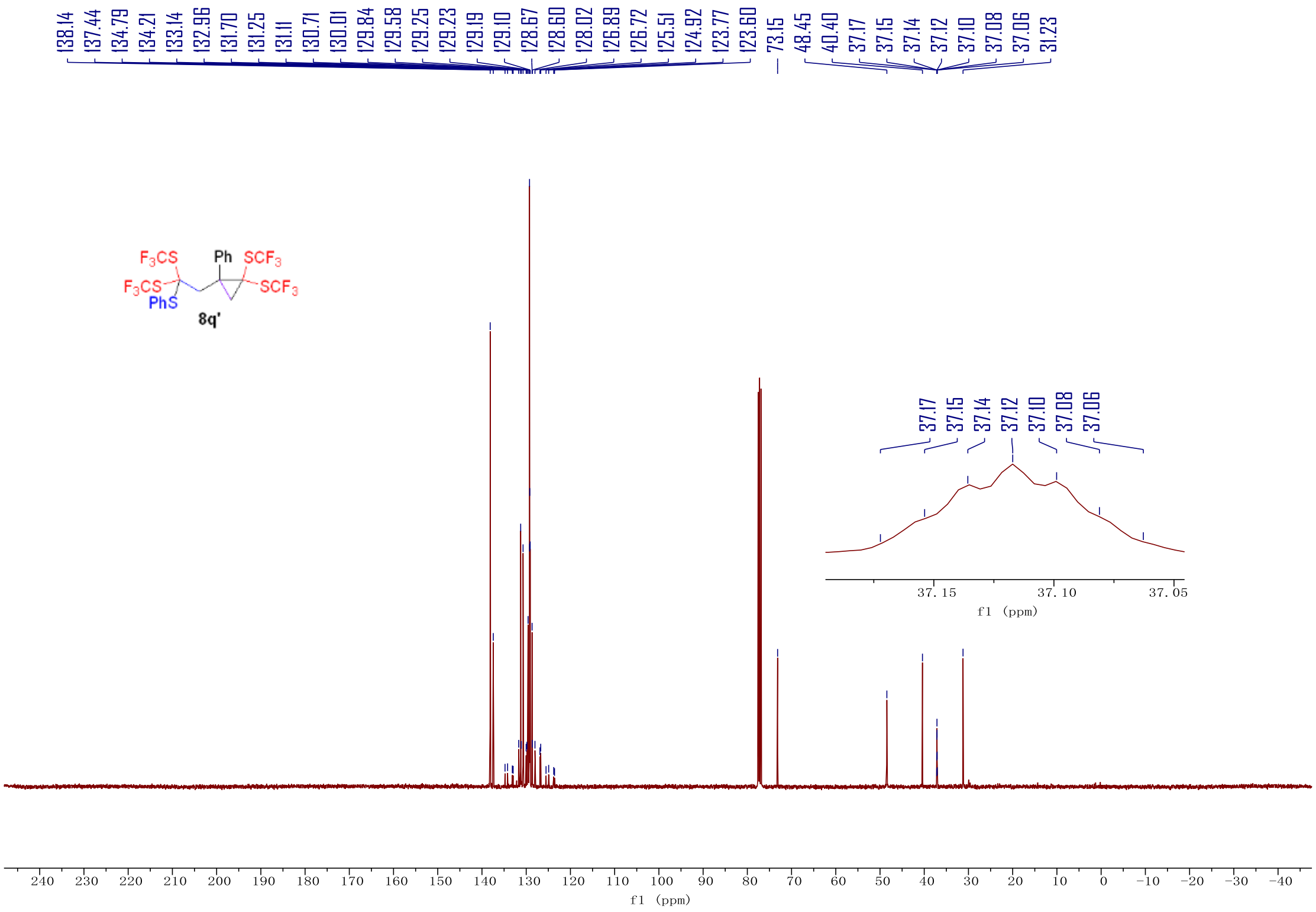

^{1}H NMR Spectrum of **8r** (400 MHz, $CDCl_3$)

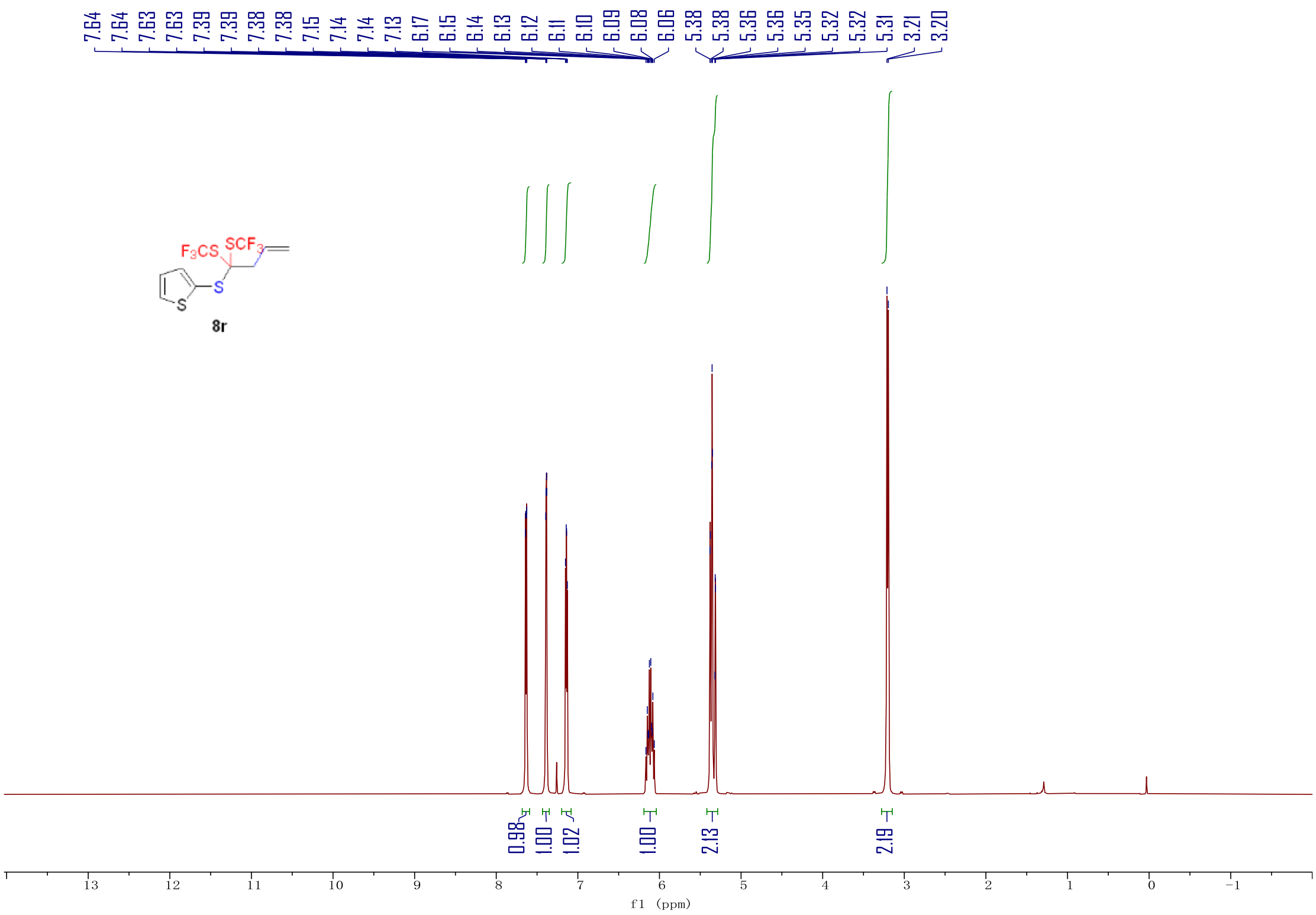

^{19}F **NMR** Spectrum of **8r** (376 MHz, $CDCl_3$)

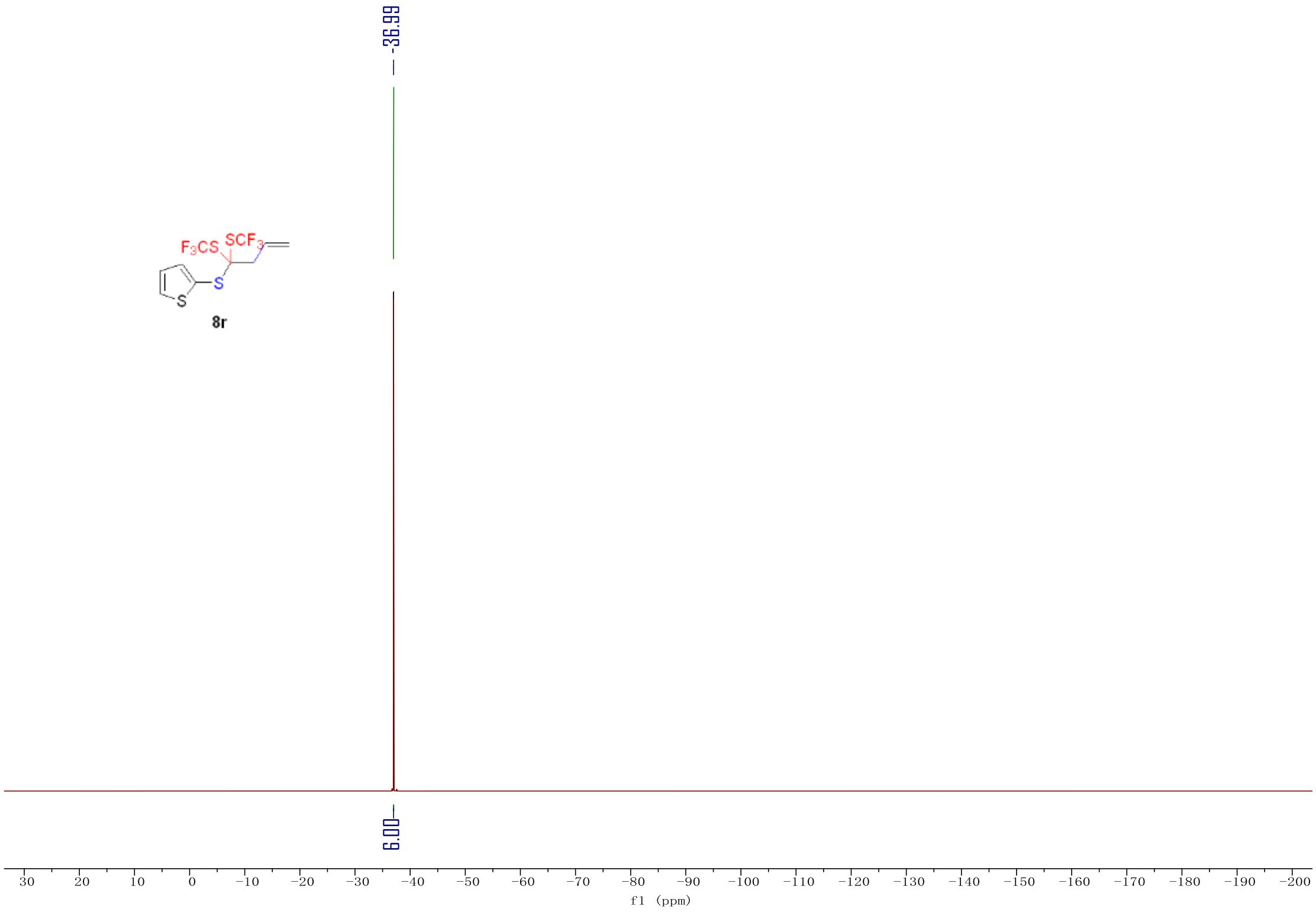

^{13}C **NMR** Spectrum of **8r** (101 MHz, $CDCl_3$)

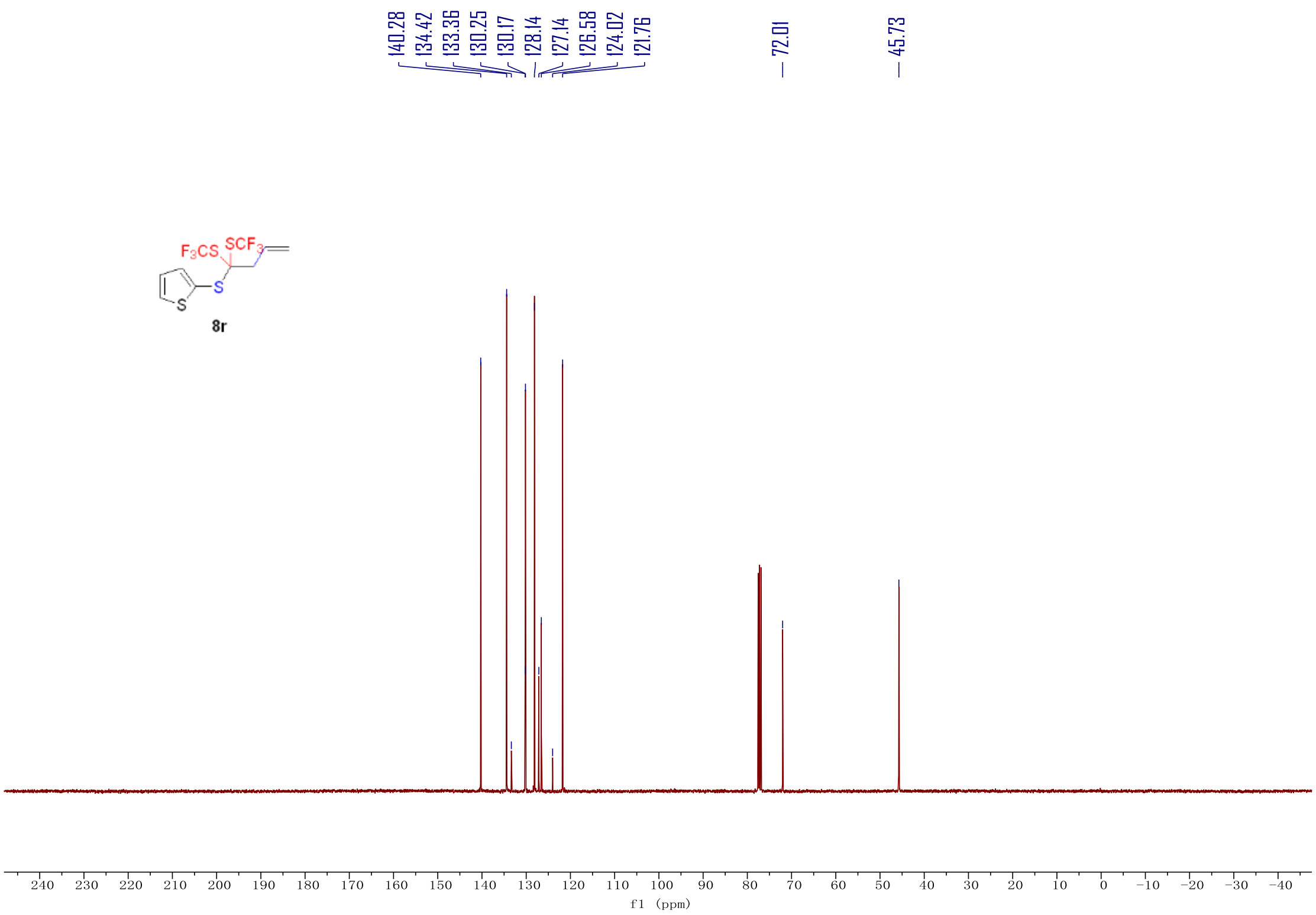

^{1}H NMR Spectrum of **8s** (400 MHz, $CDCl_3$)

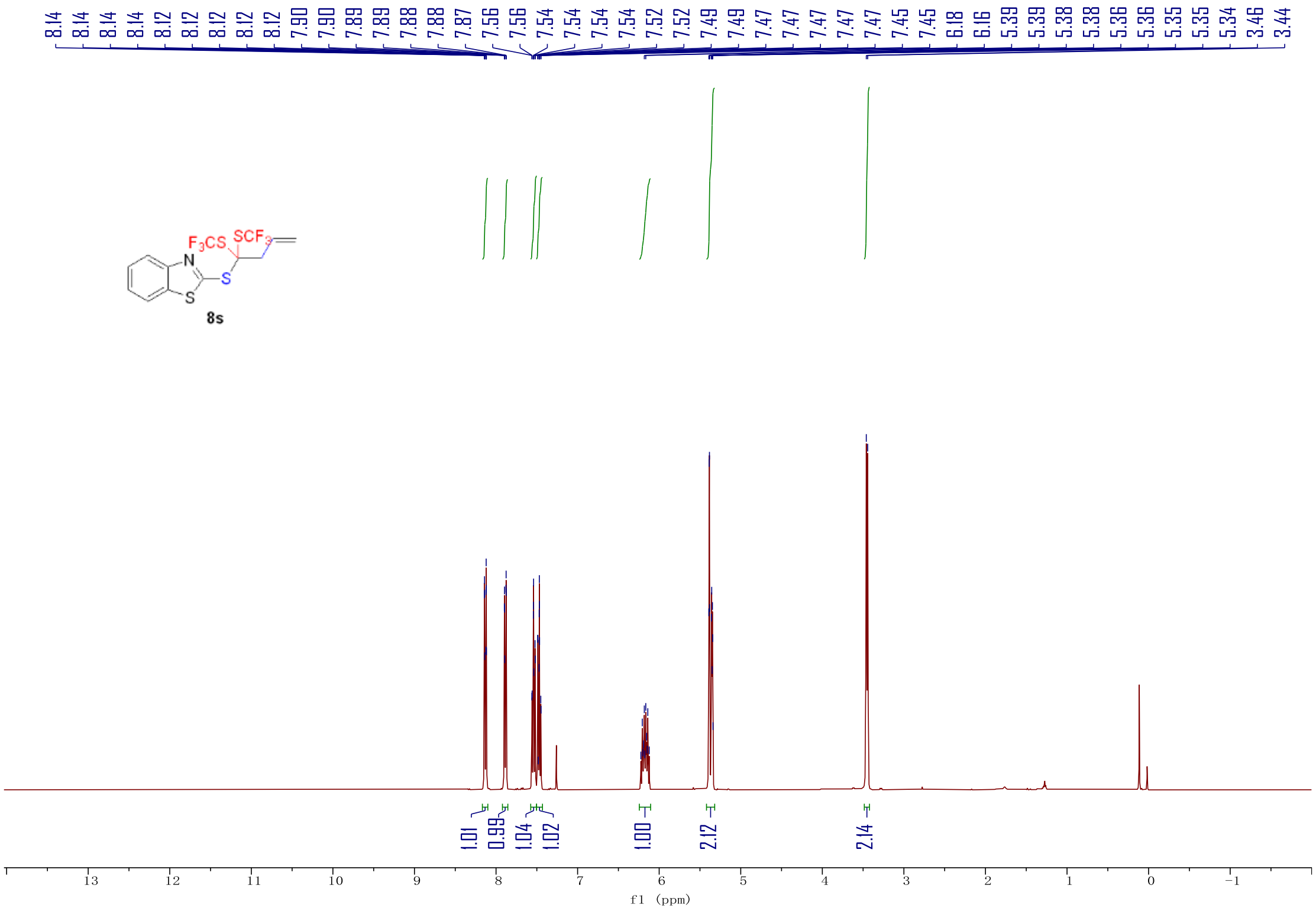

^{19}F NMR Spectrum of **8s** (376 MHz, $CDCl_3$)

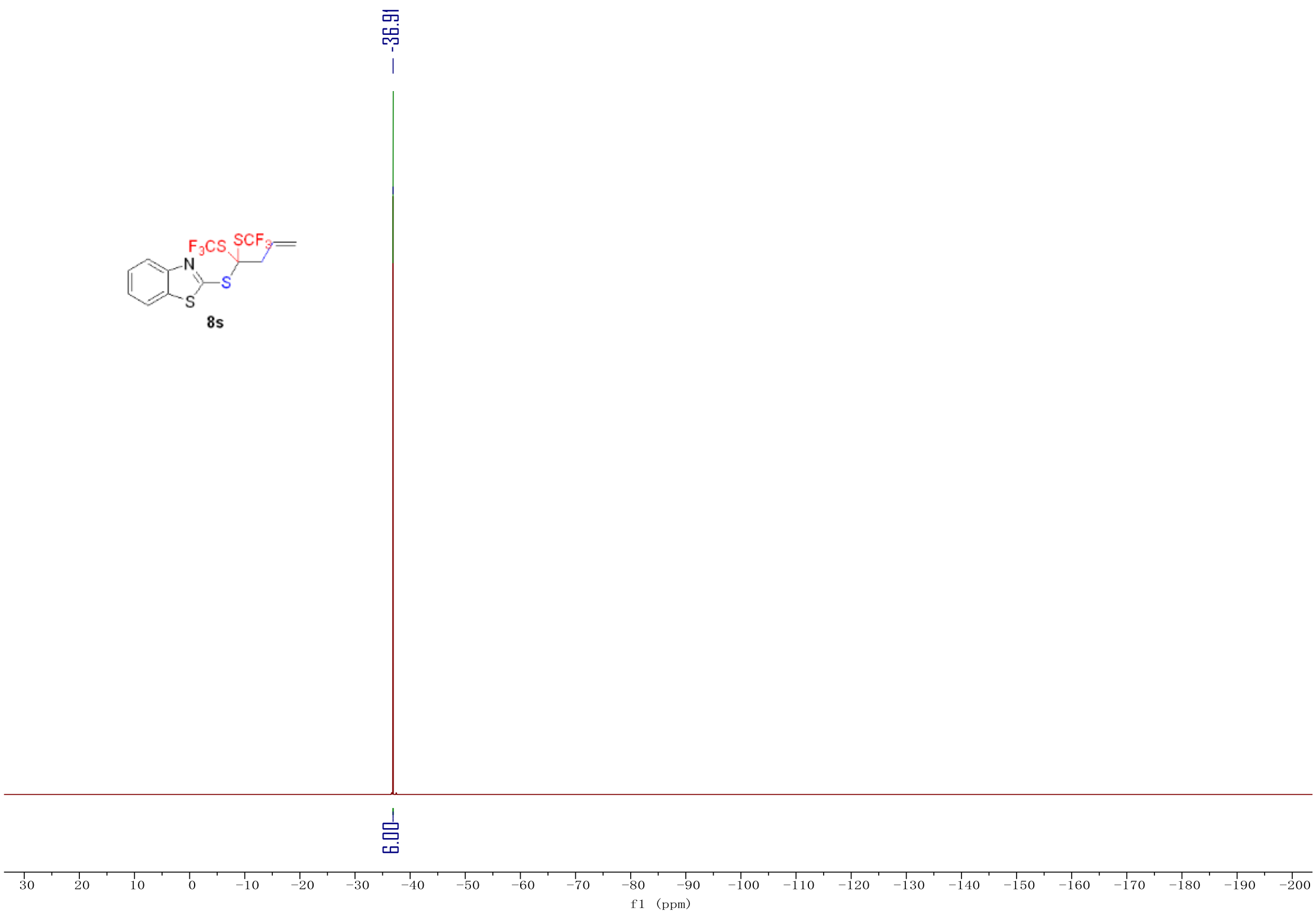

^{13}C NMR Spectrum of **8s** (101 MHz, $CDCl_3$)

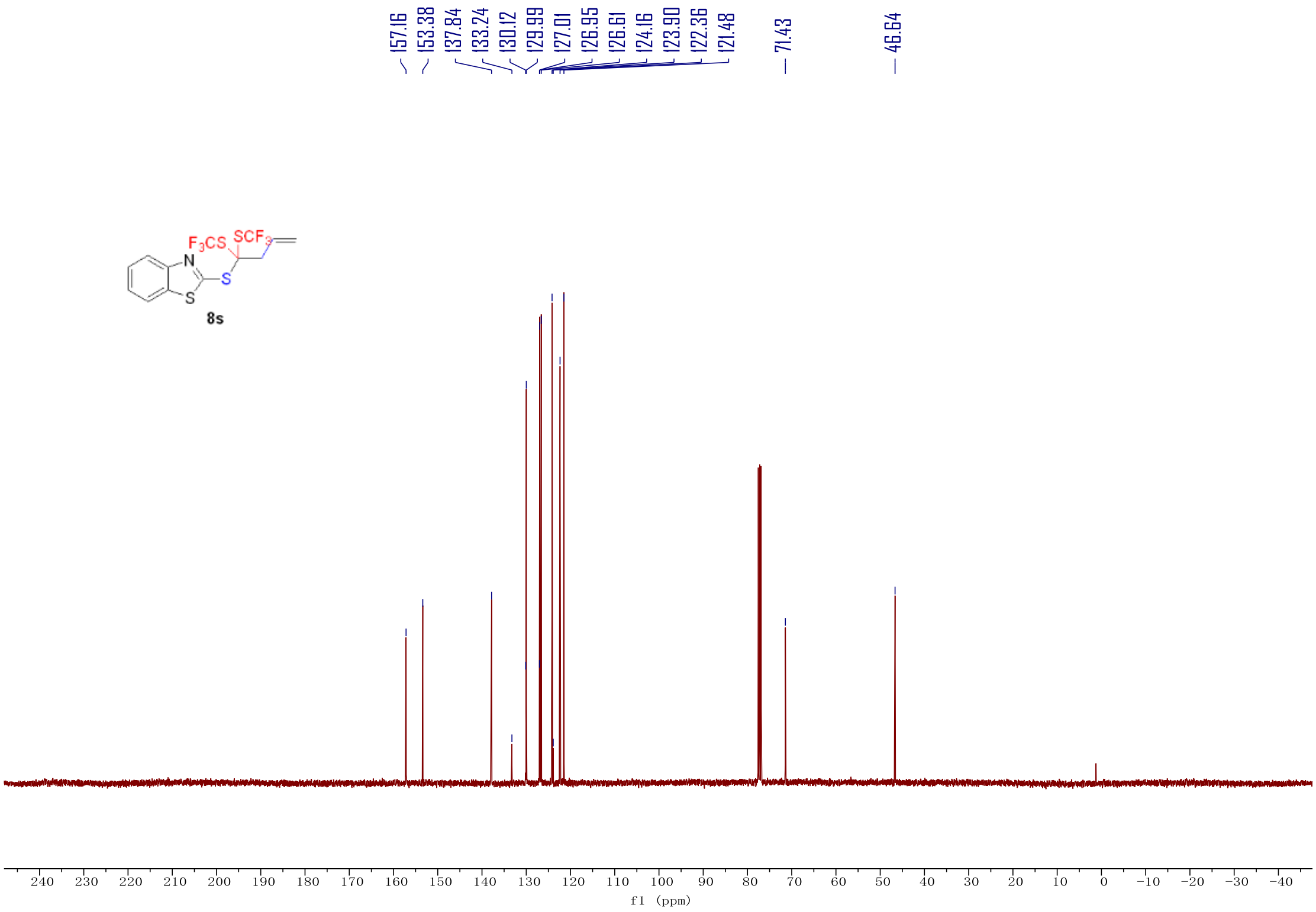

^{1}H NMR Spectrum of **8t** (400 MHz, $CDCl_3$)

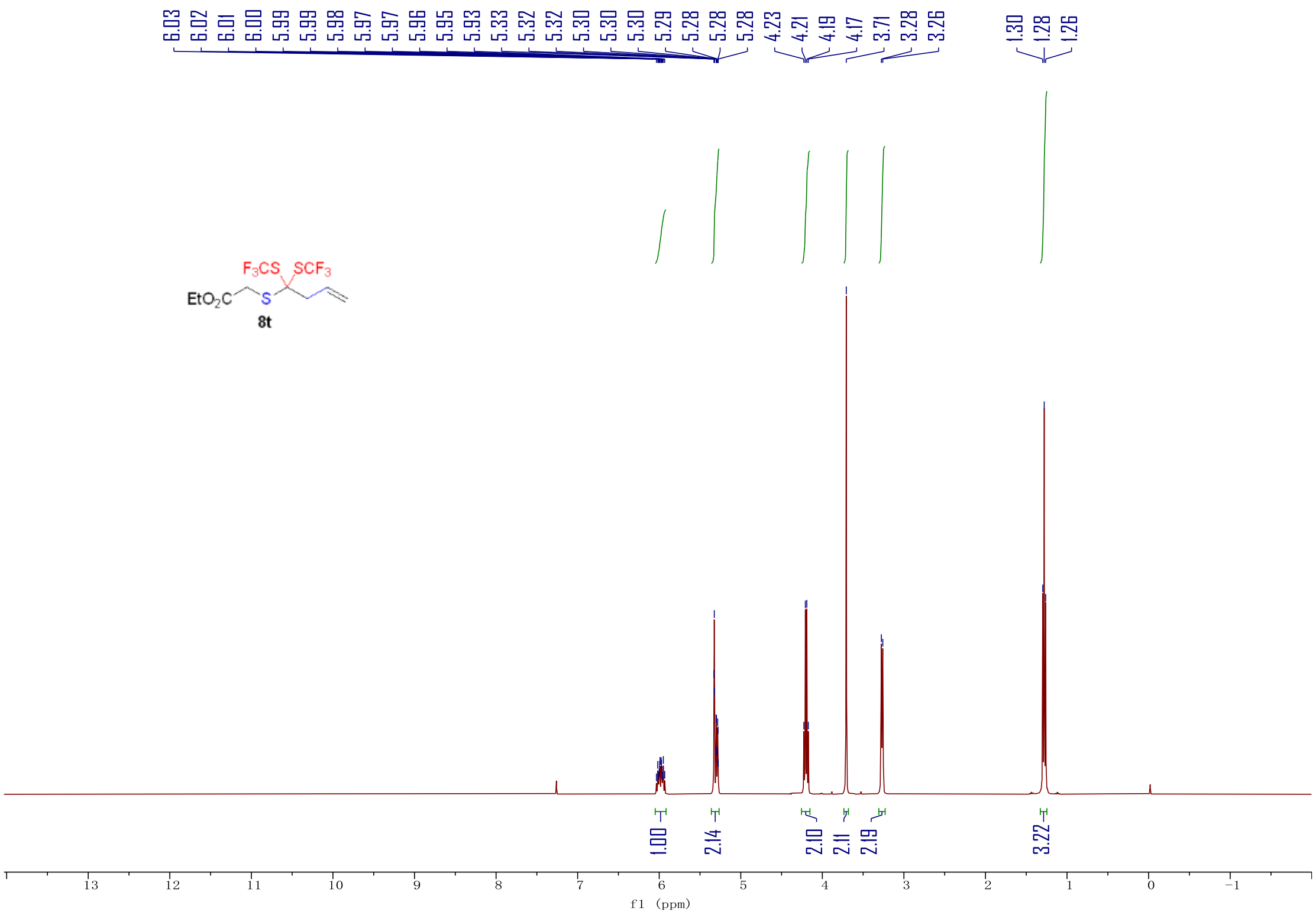

^{19}**F NMR** Spectrum of **8t** (376 MHz, $CDCl_3$)

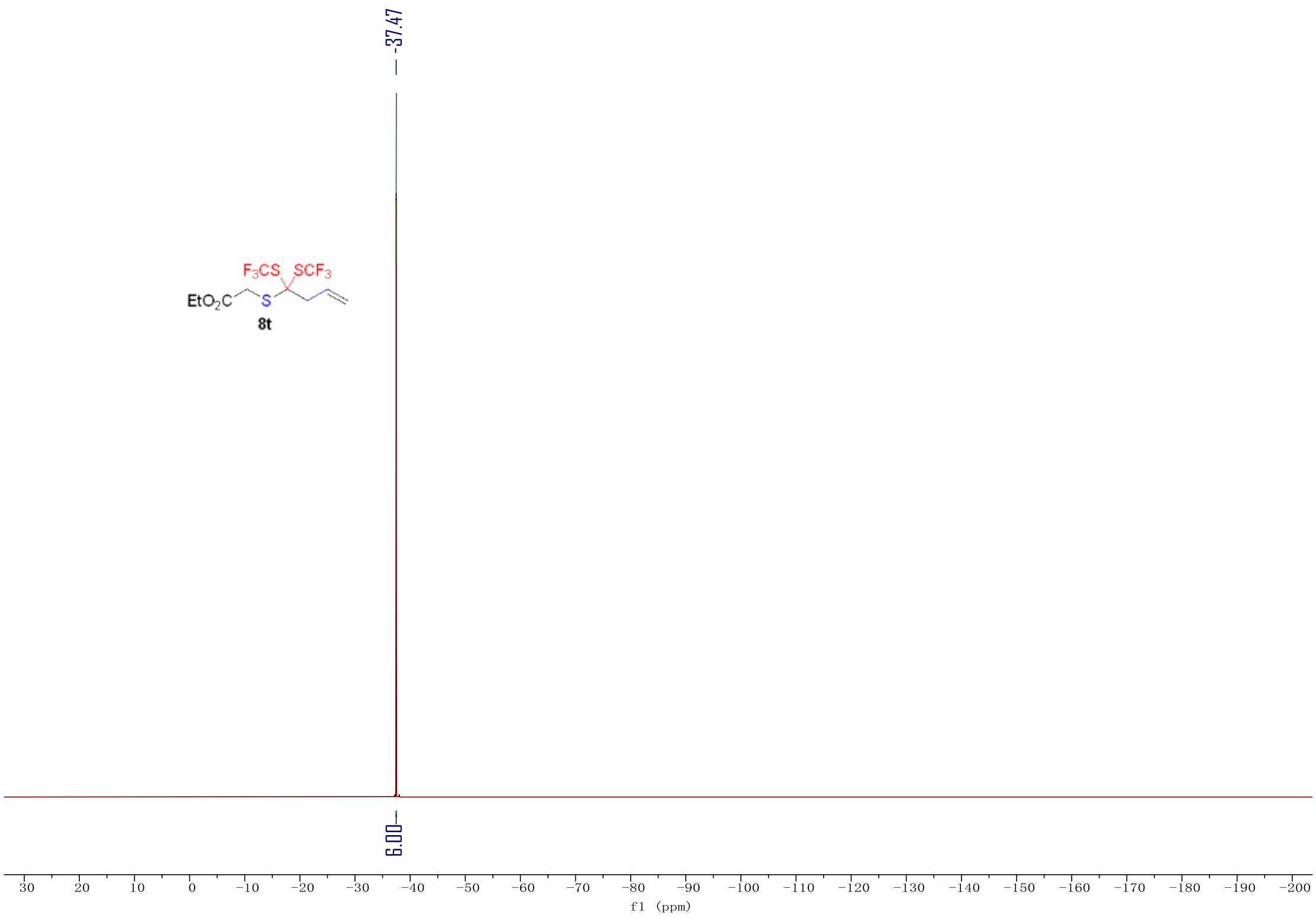

^{13}**C NMR** Spectrum of **8t** (101 MHz, $CDCl_3$)

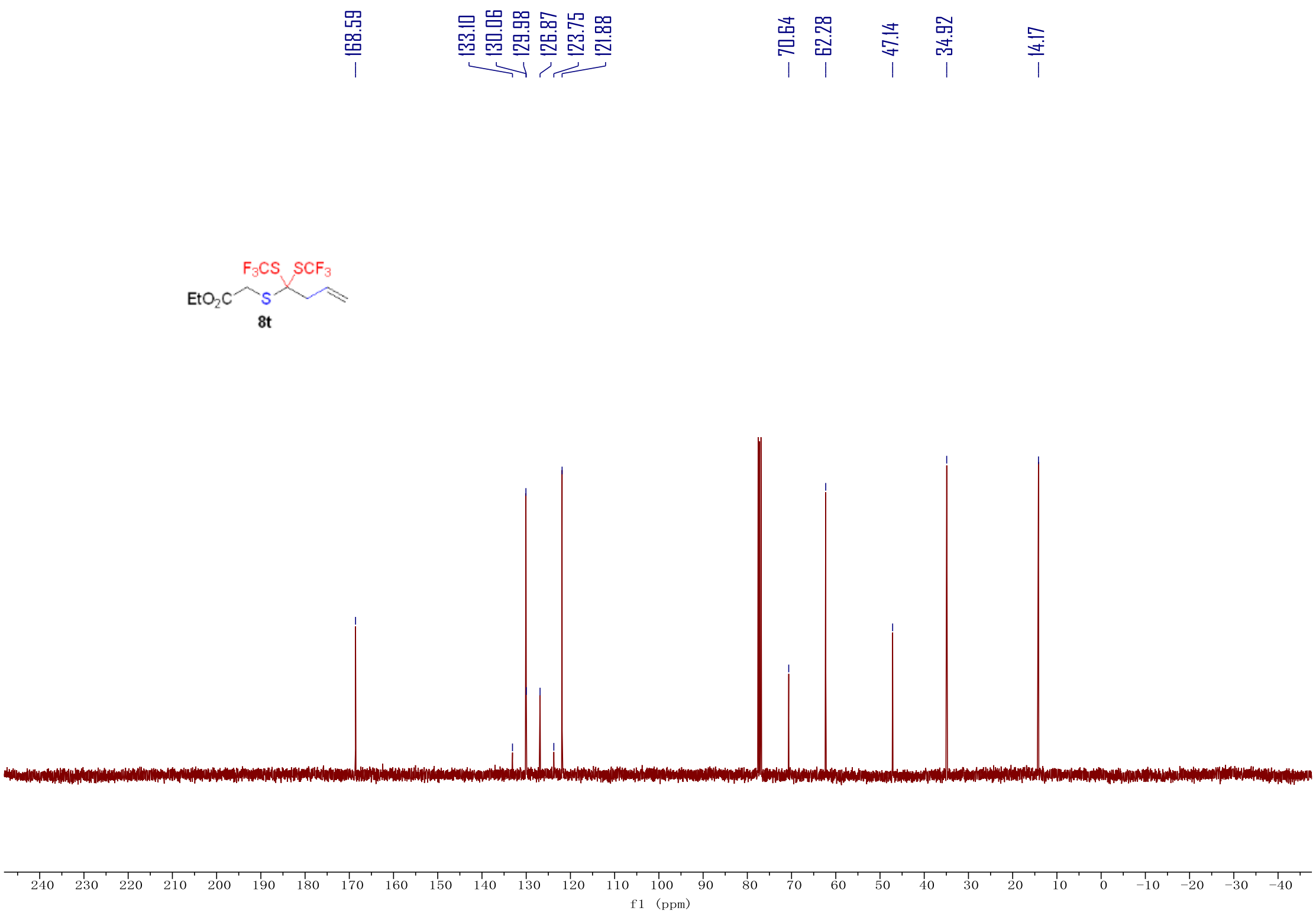

^{1}H NMR Spectrum of **8u** (400 MHz, $CDCl_3$)

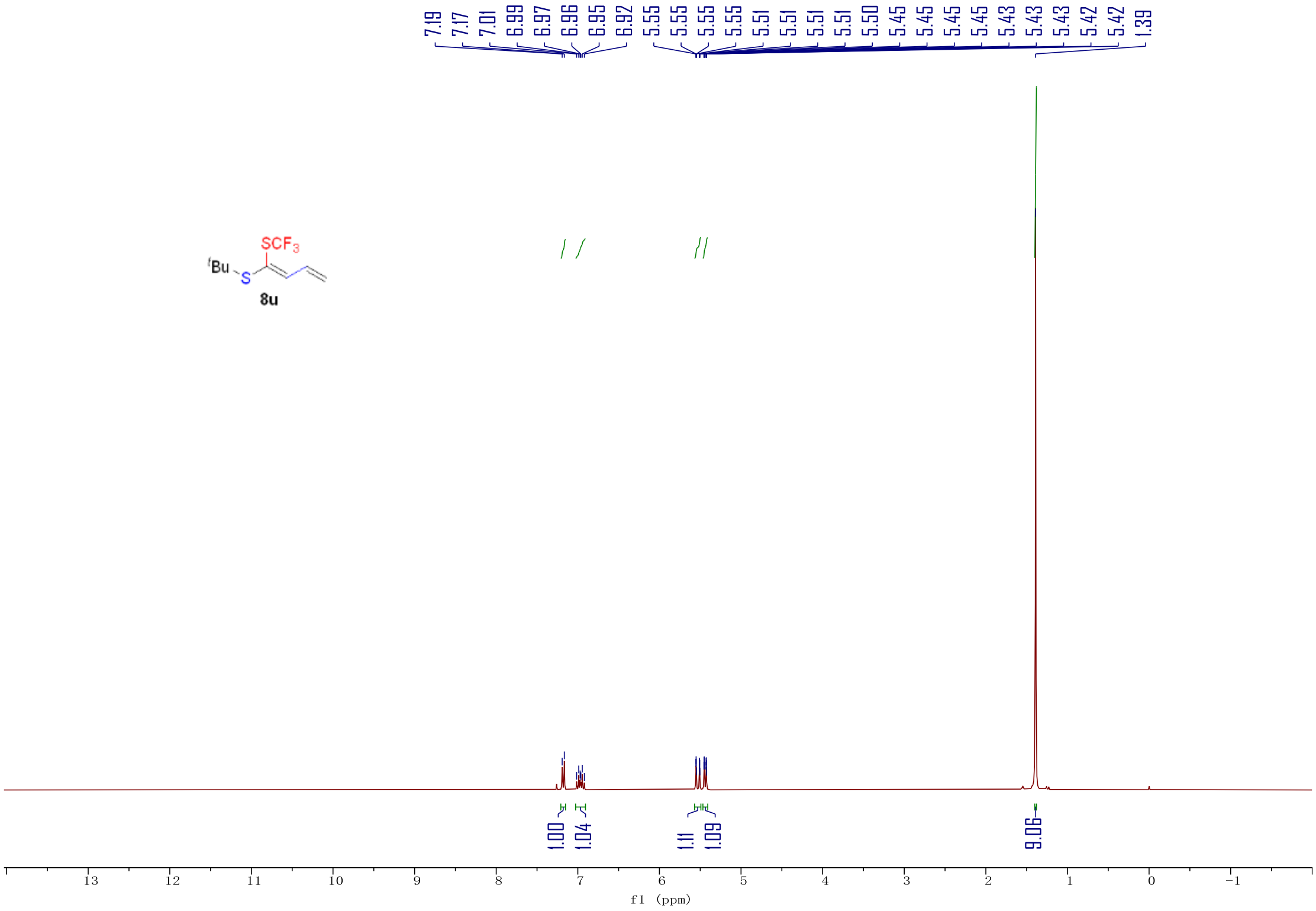

^{19}F NMR Spectrum of **8u** (376 MHz, $CDCl_3$)

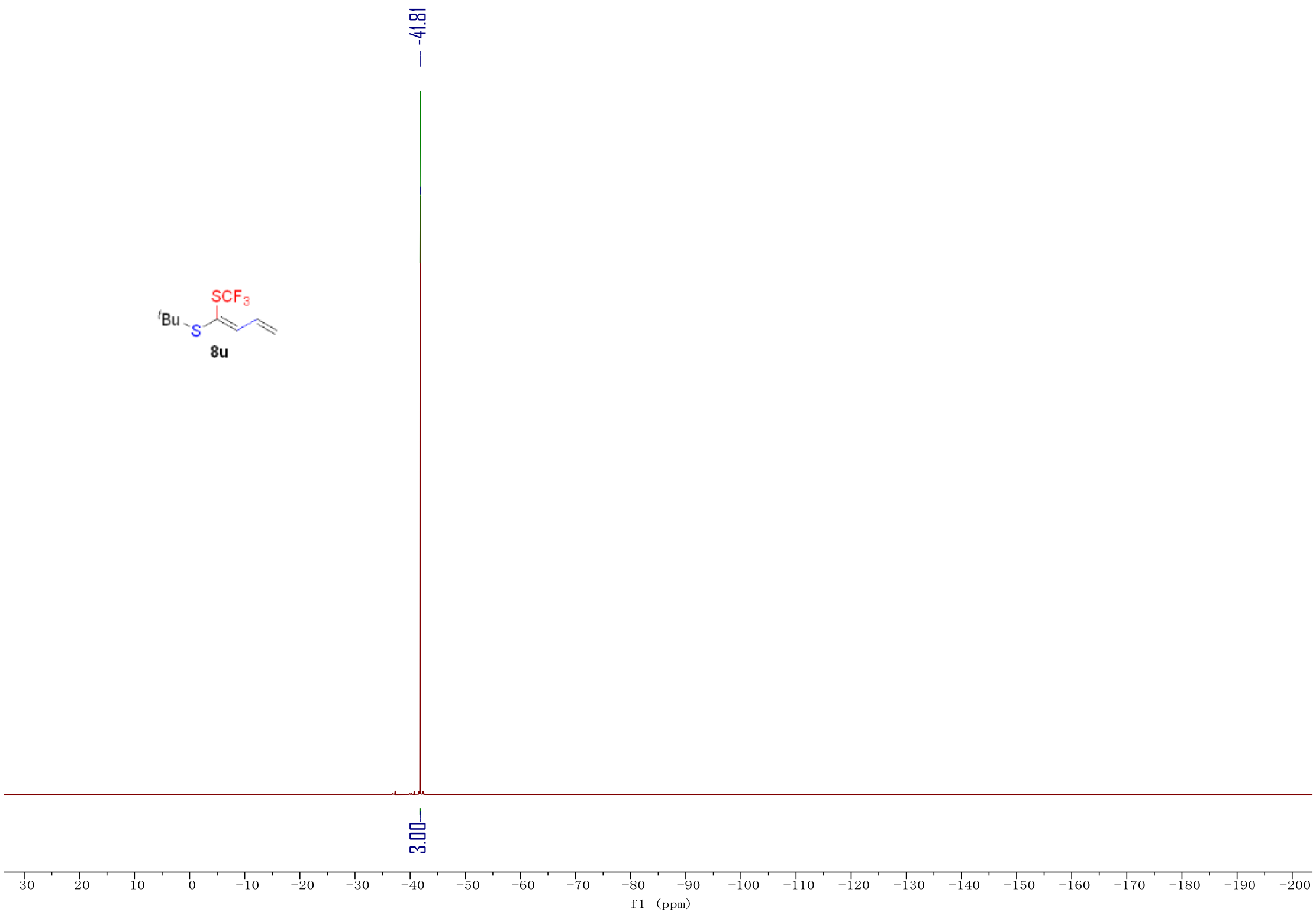

^{13}C **NMR** Spectrum of **8u** (101 MHz, $CDCl_3$)

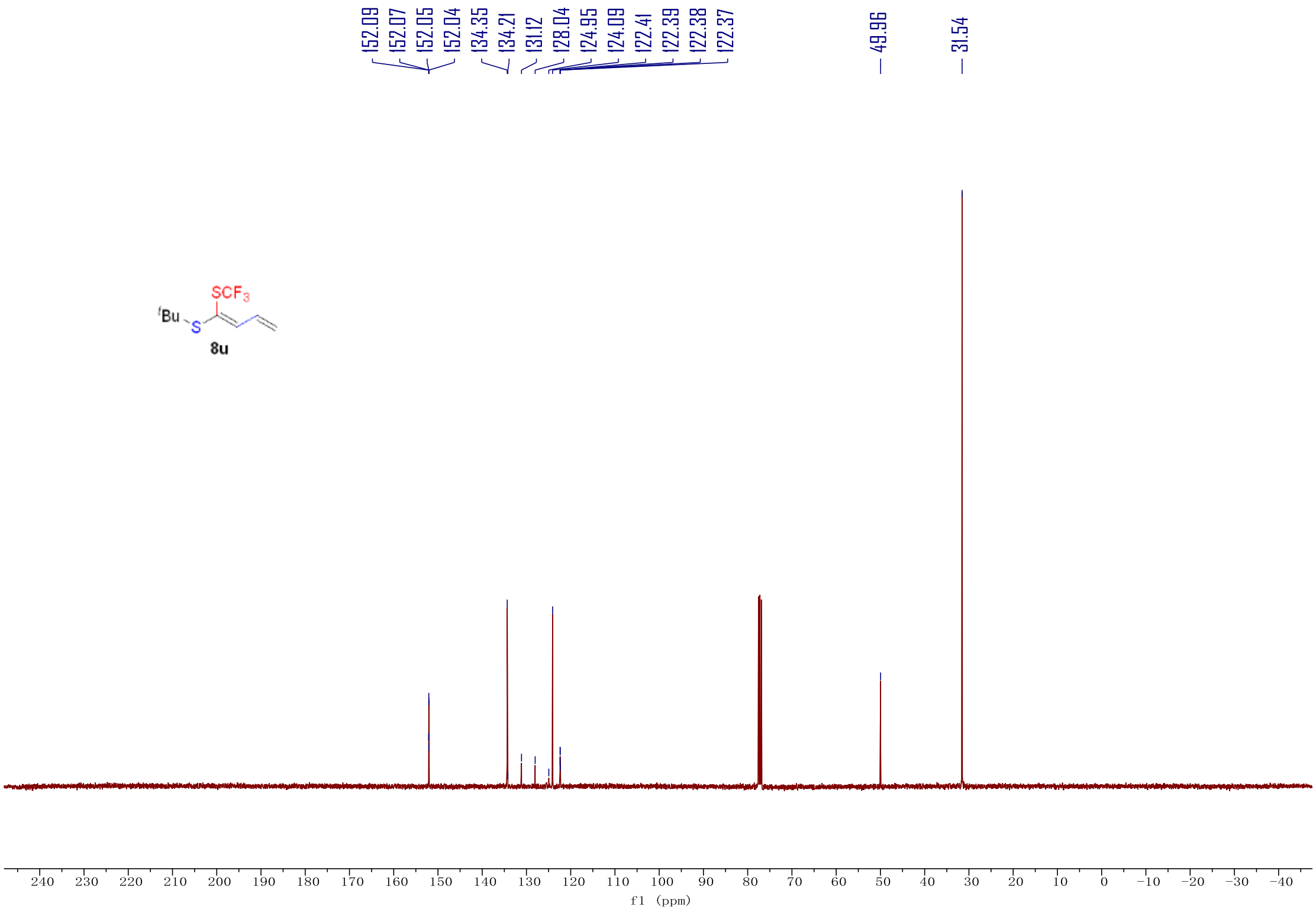

^{1}H **NMR** Spectrum of **8v** (400 MHz, $CDCl_3$)

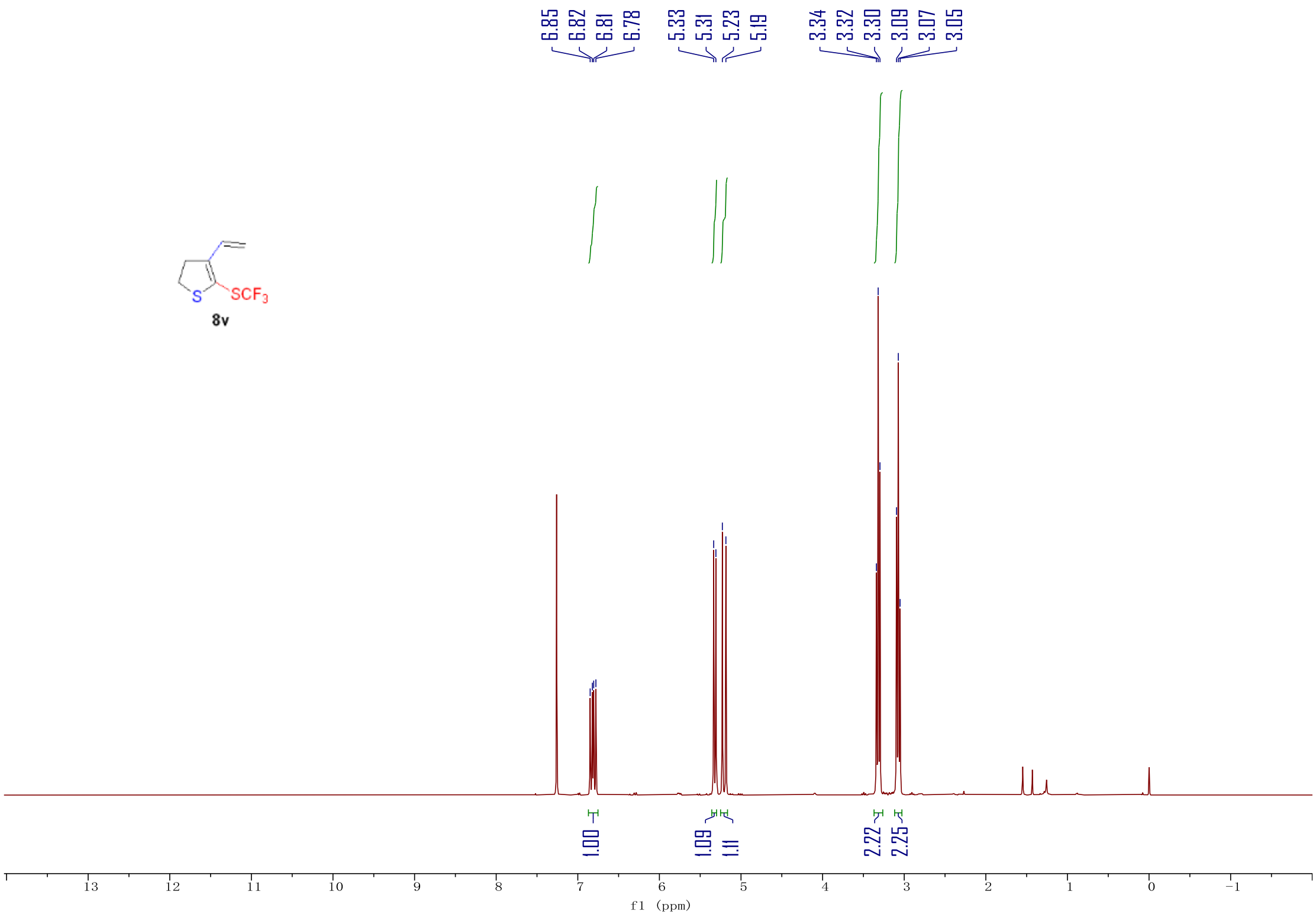

^{19}F NMR Spectrum of **8v** (376 MHz, $CDCl_3$)

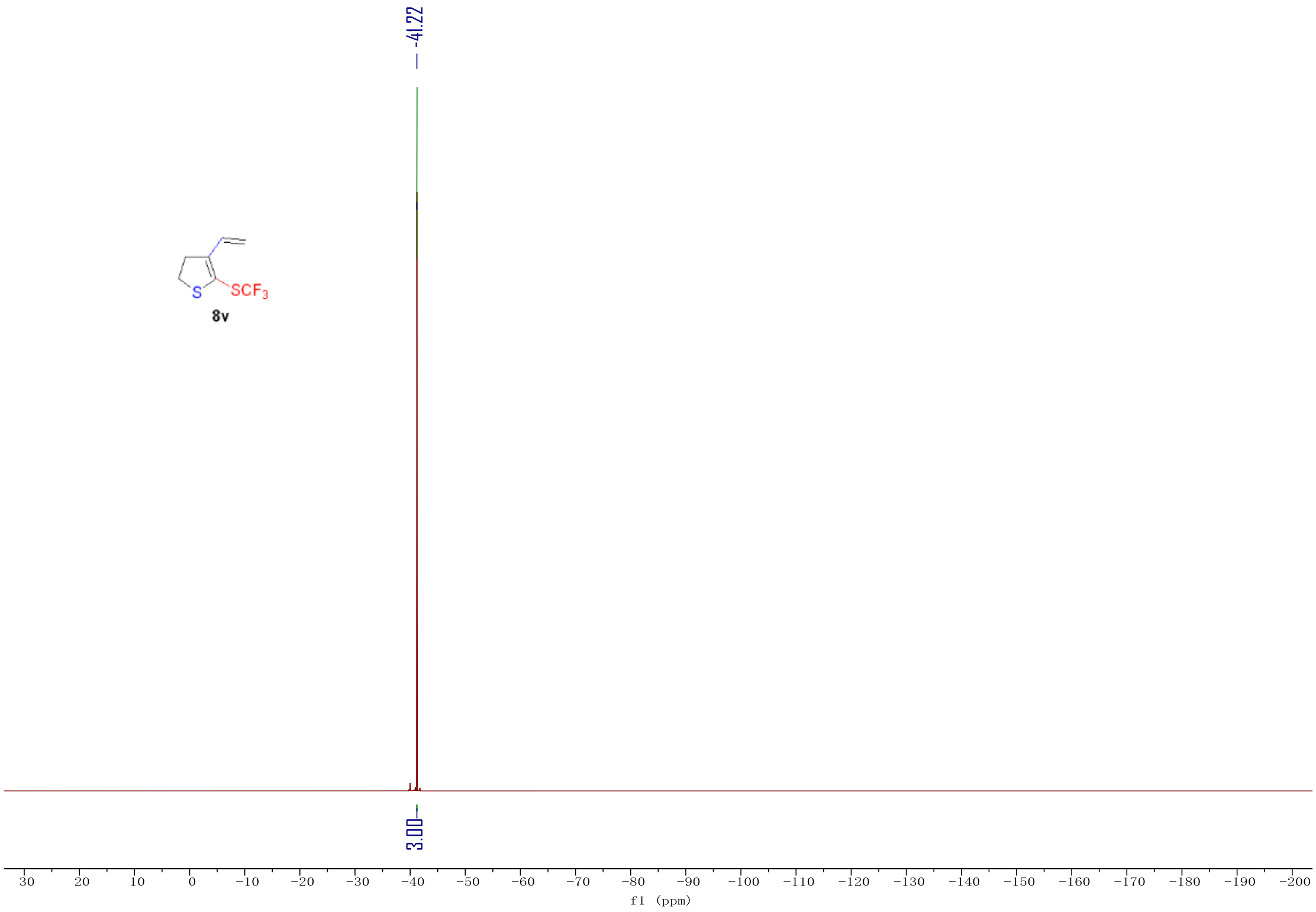

^{13}C NMR Spectrum of **8v** (101 MHz, $CDCl_3$)

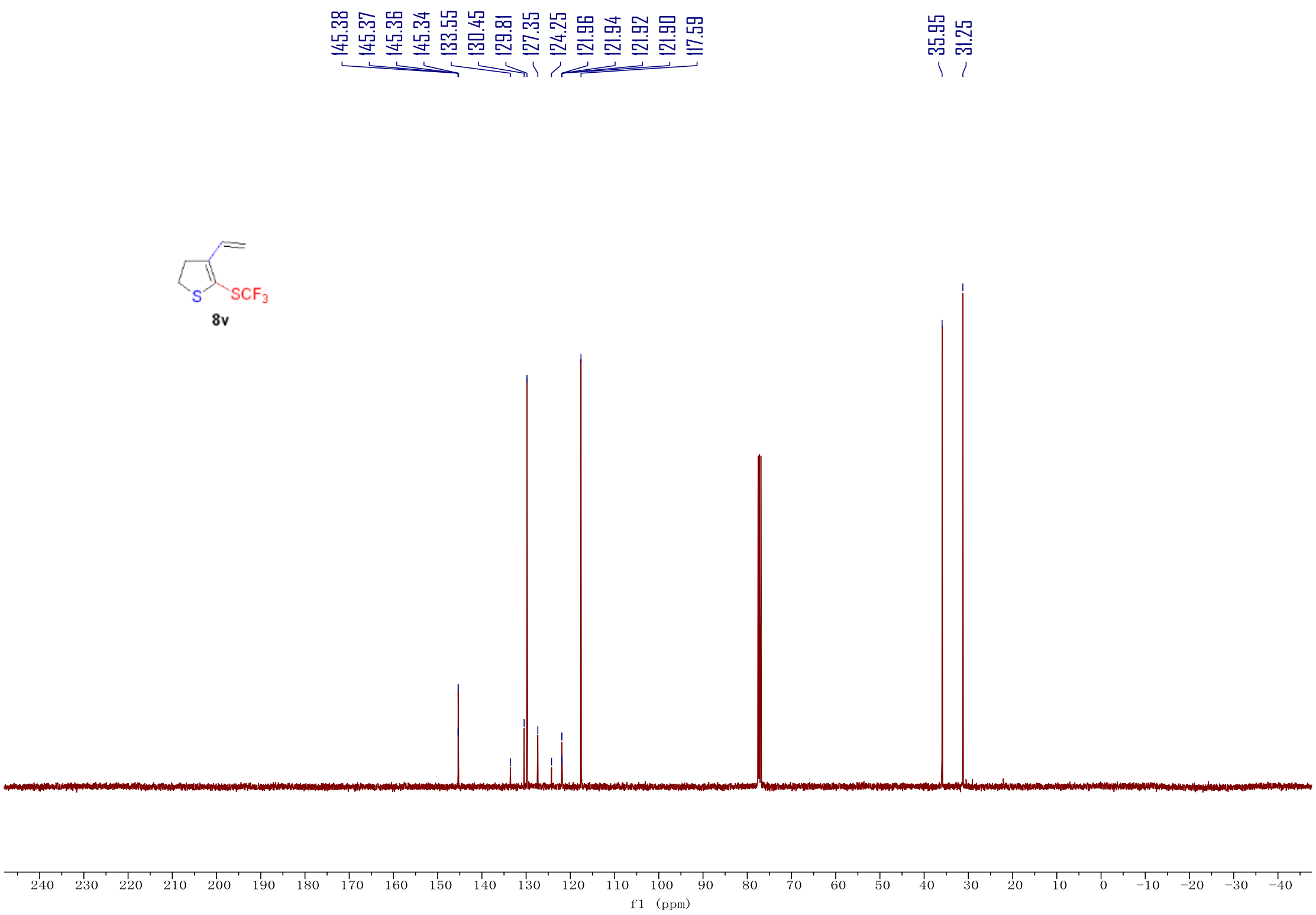

^{1}**H NMR** Spectrum of **8w** (400 MHz, $CDCl_3$)

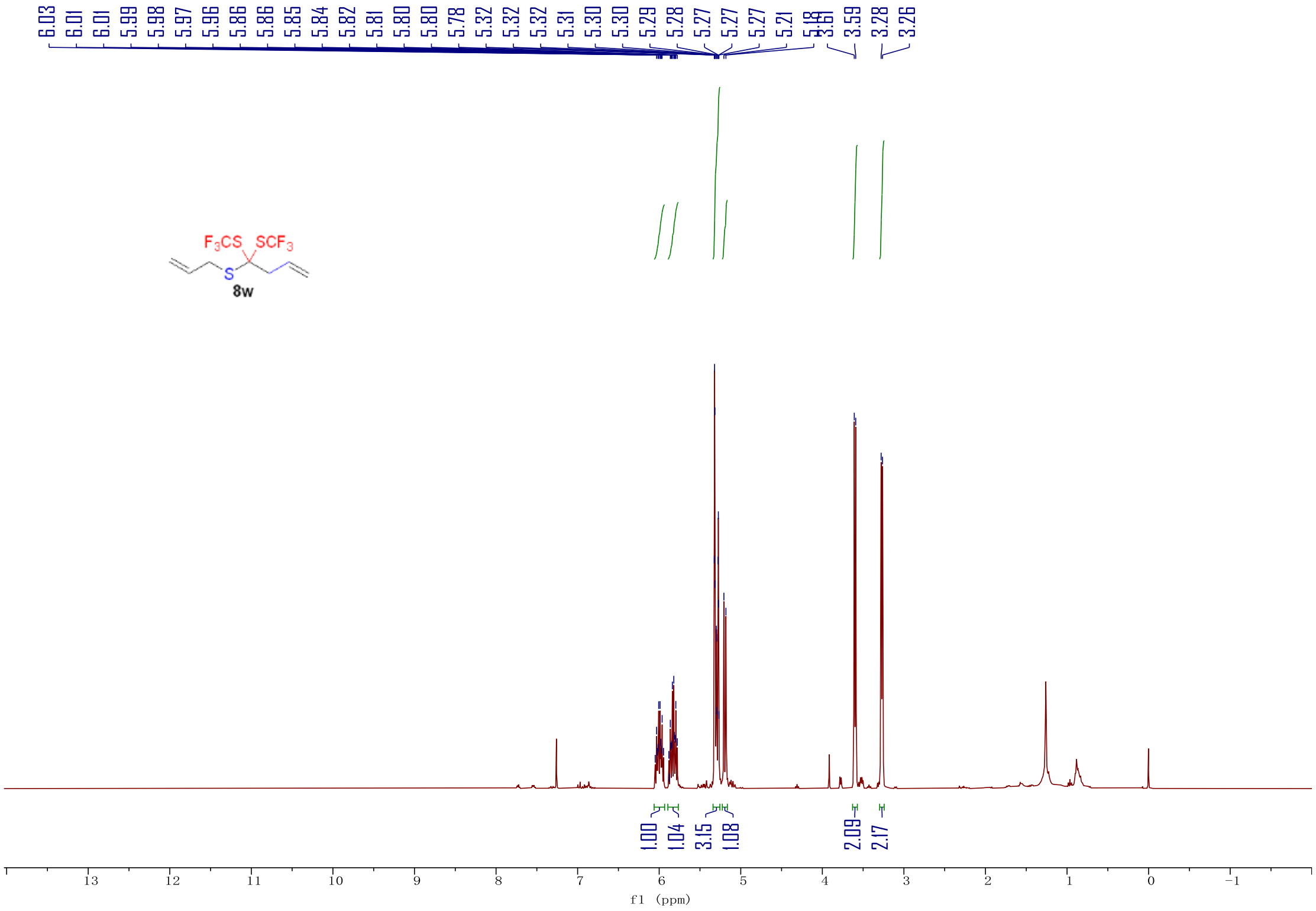

^{19}**F NMR** Spectrum of **8w** (376 MHz, $CDCl_3$)

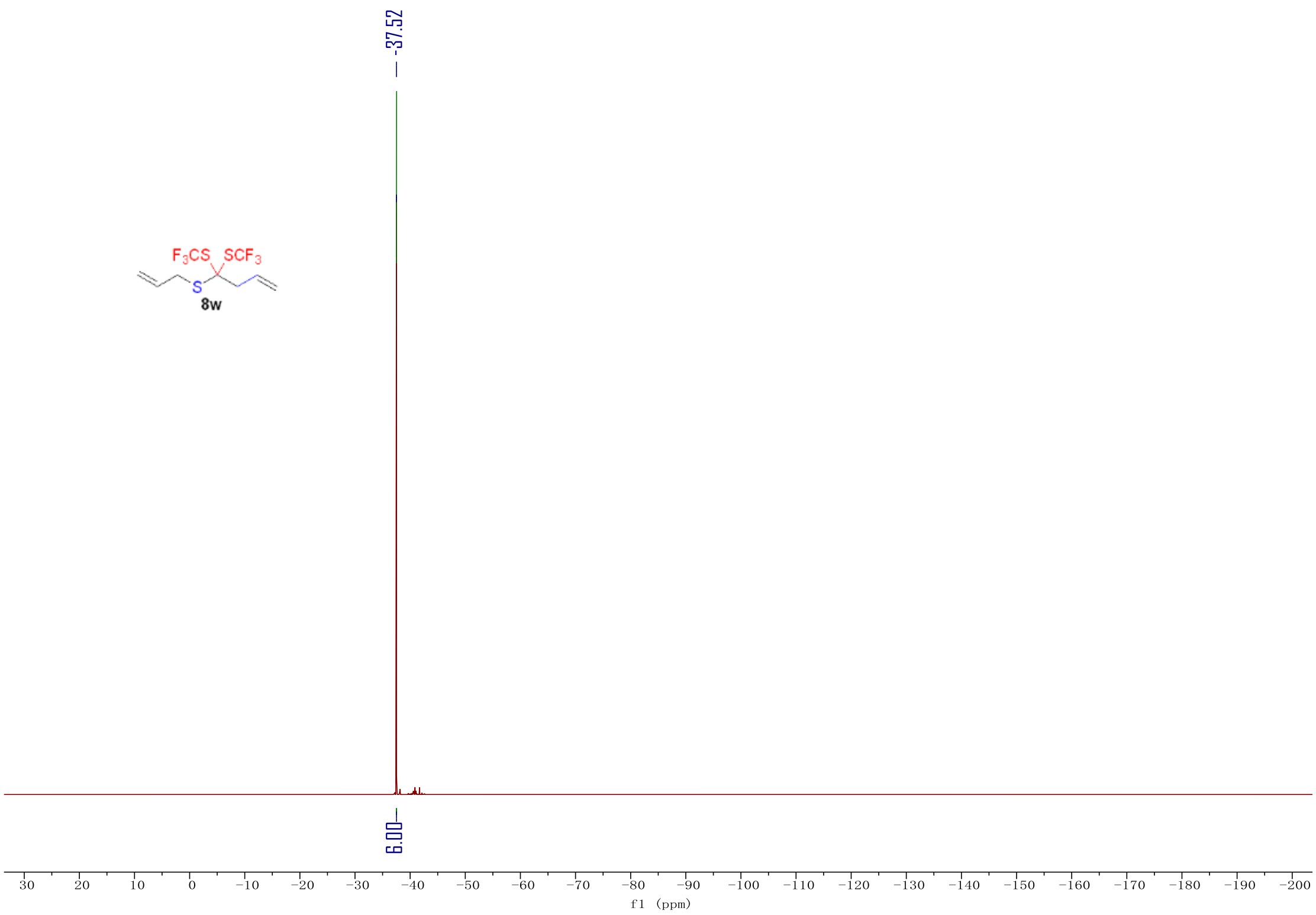

^{13}C NMR Spectrum of **8w** (101 MHz, $CDCl_3$)

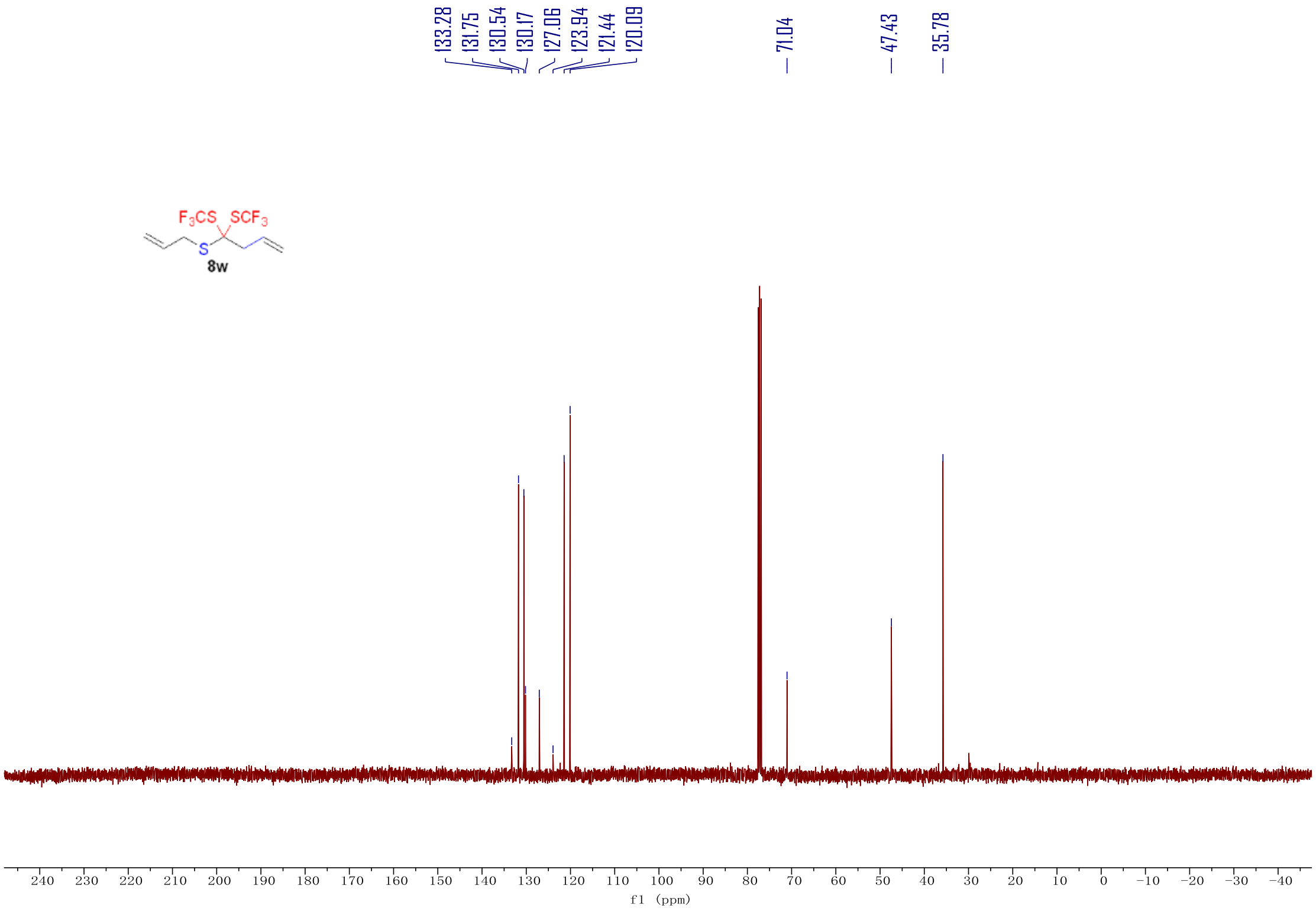

^{1}H NMR Spectrum of **10a** (400 MHz, $CDCl_3$)

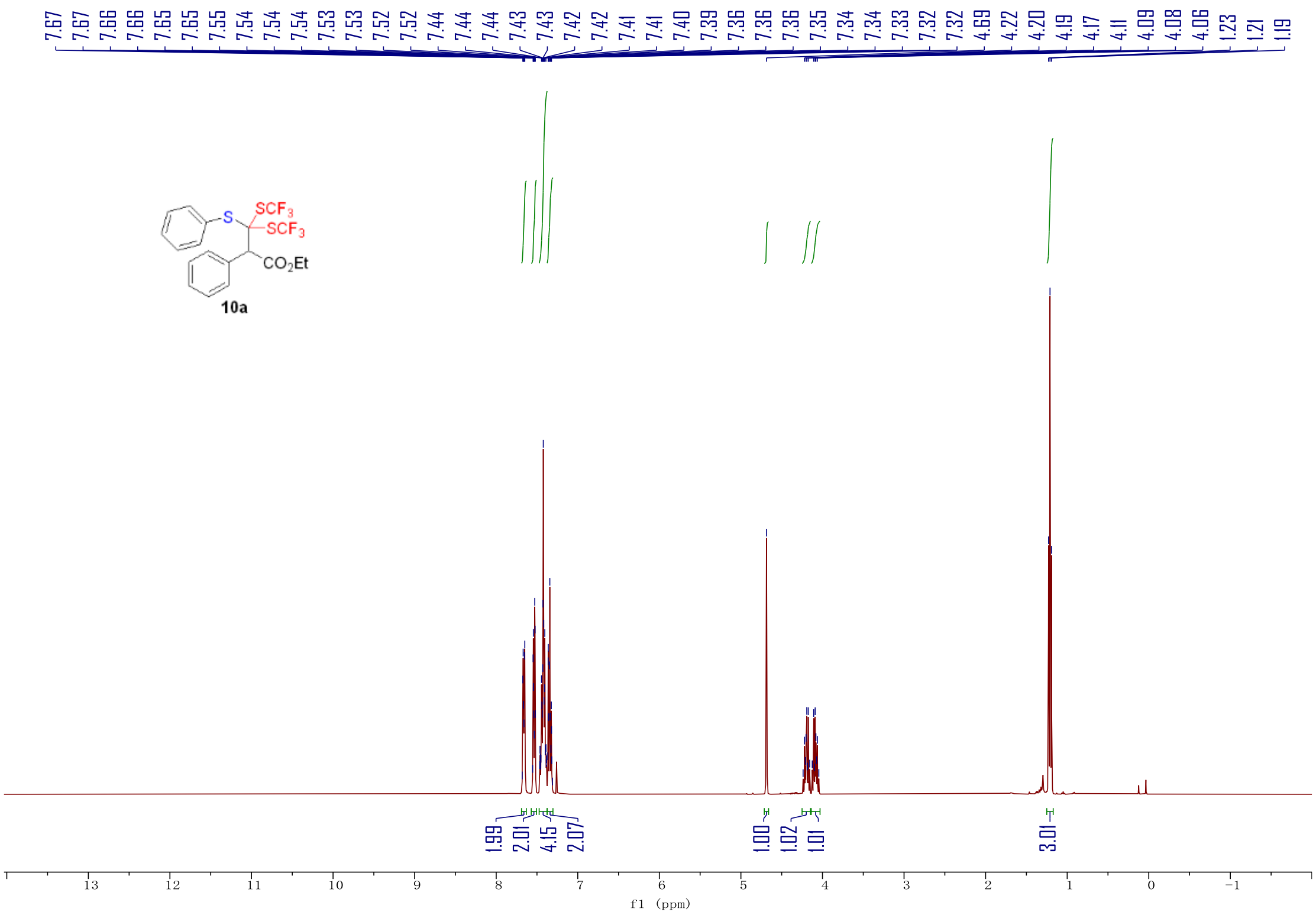

^{19}**F NMR** Spectrum of **10a** (376 MHz, $CDCl_3$)

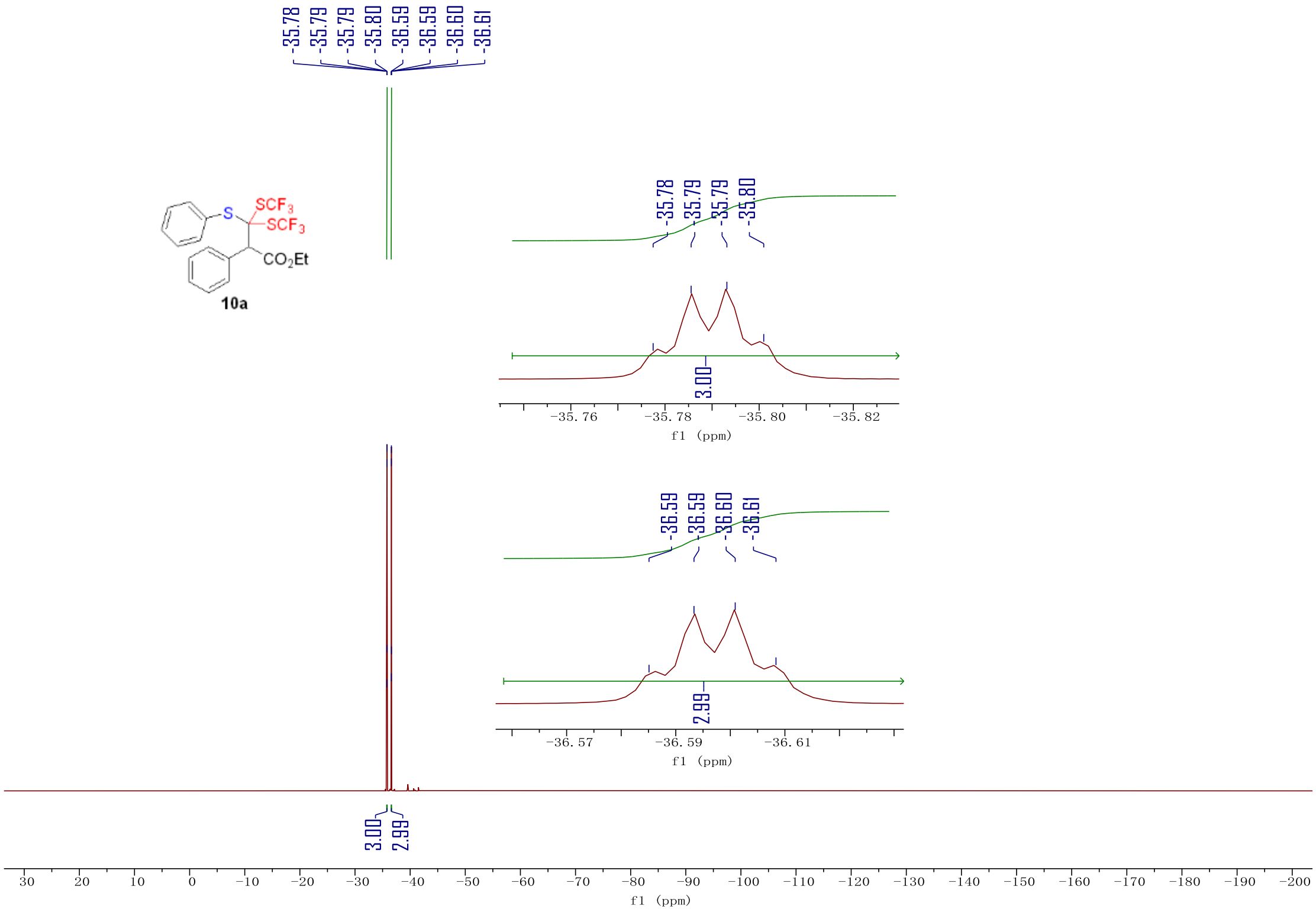

^{13}**C NMR** Spectrum of **10a** (101 MHz, $CDCl_3$)

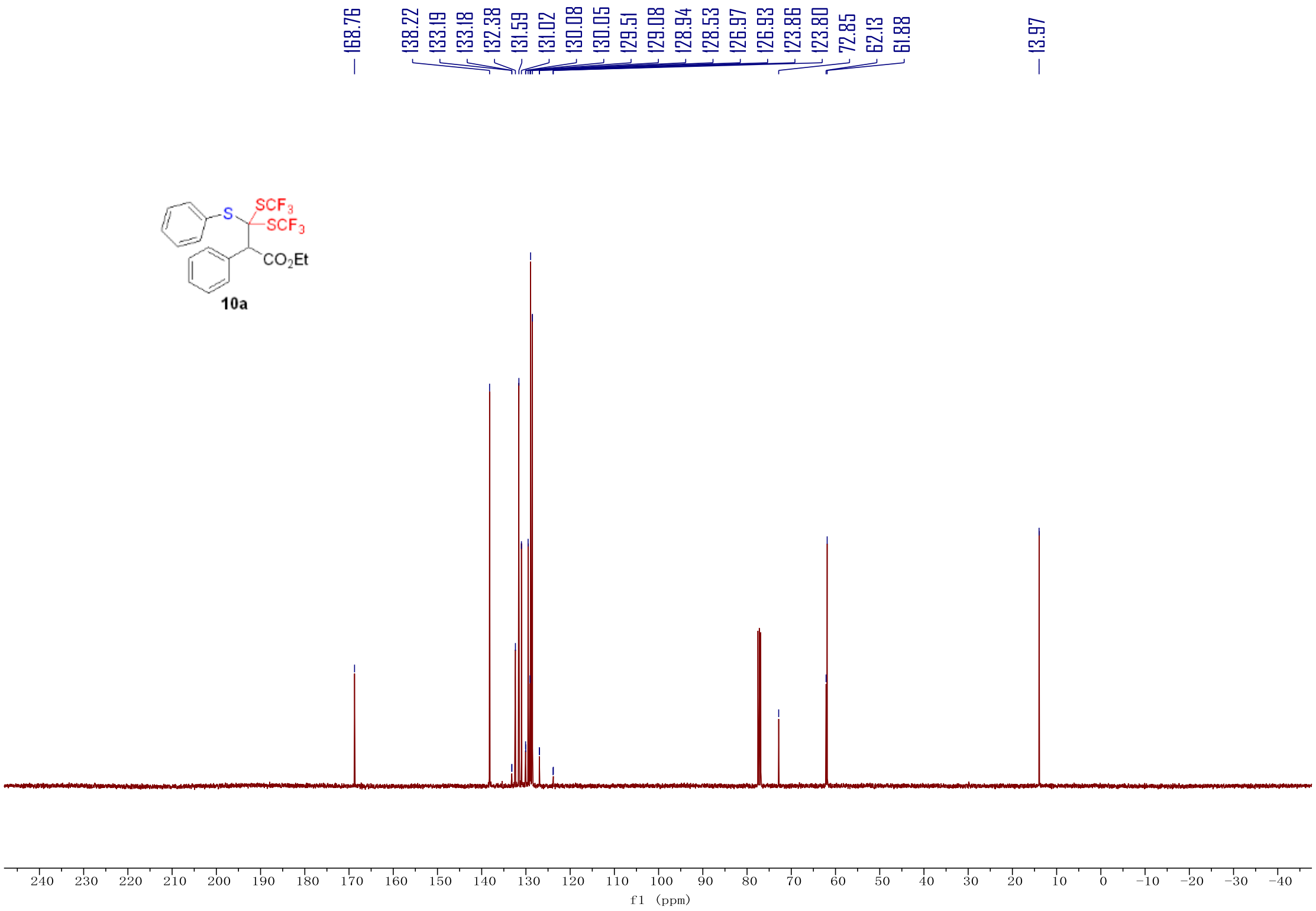

^{1}H NMR Spectrum of **10b** (400 MHz, $CDCl_3$)

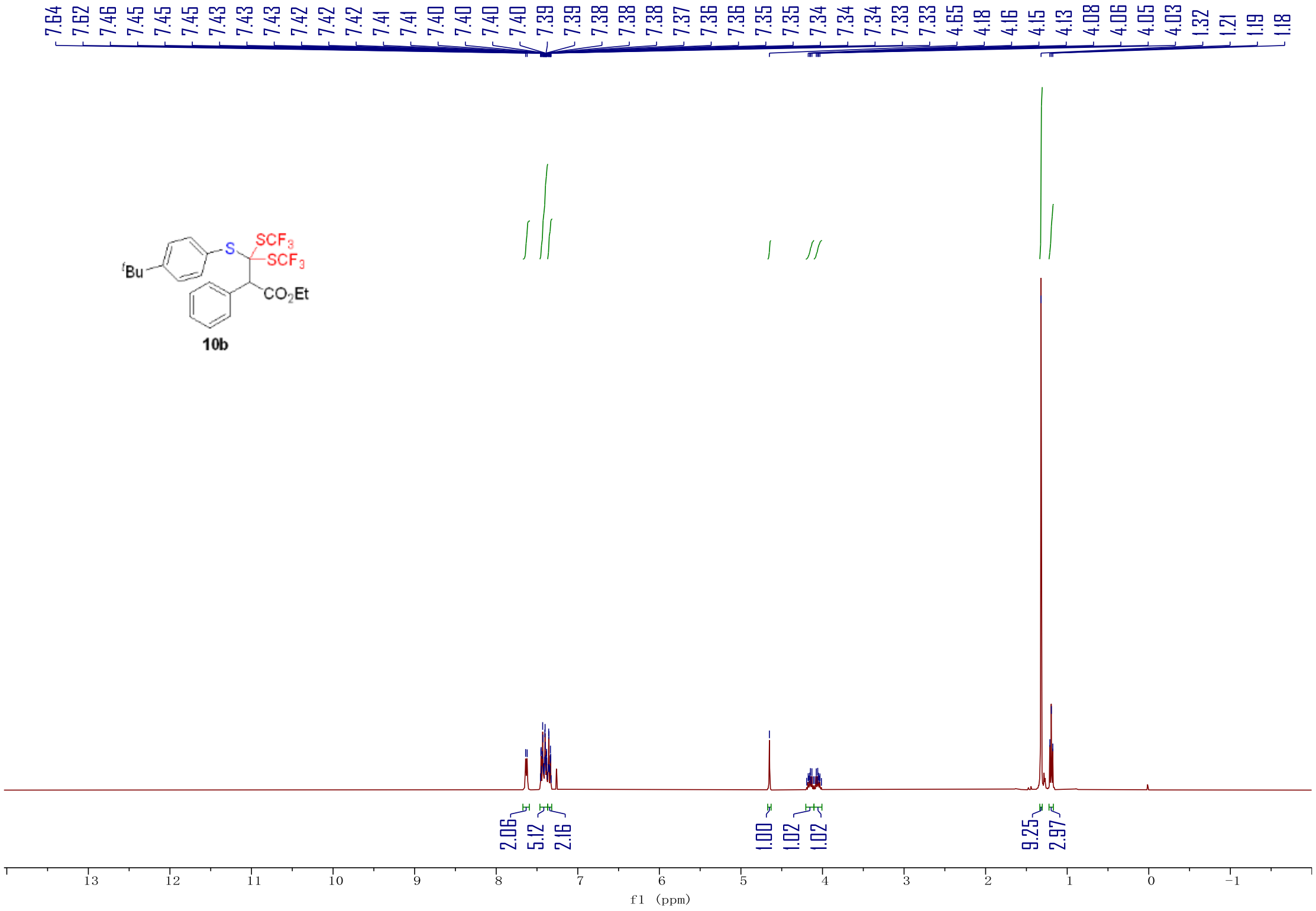

^{19}F NMR Spectrum of **10b** (376 MHz, $CDCl_3$)

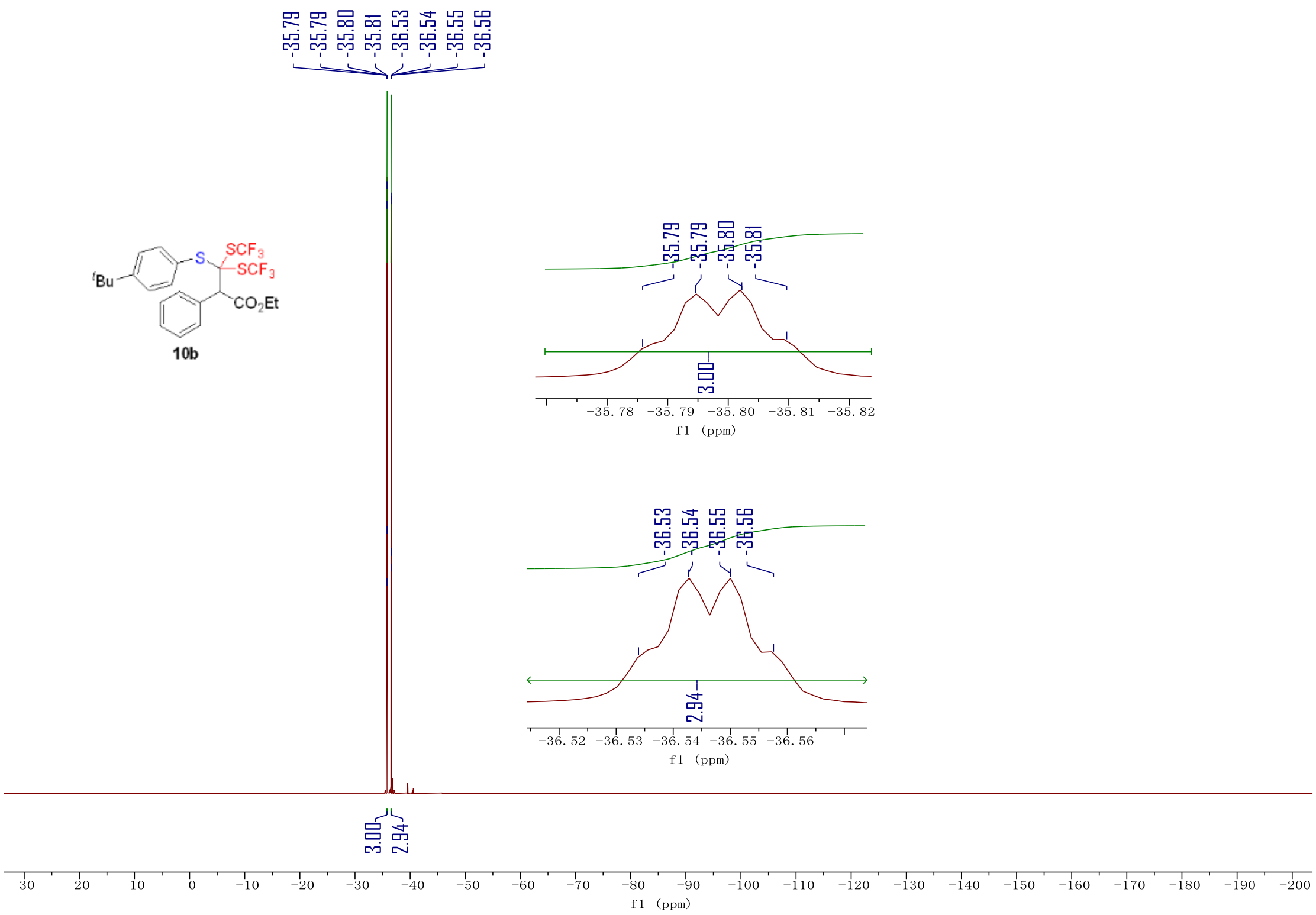

^{13}**C NMR** Spectrum of **10b** (101 MHz, $CDCl_3$)

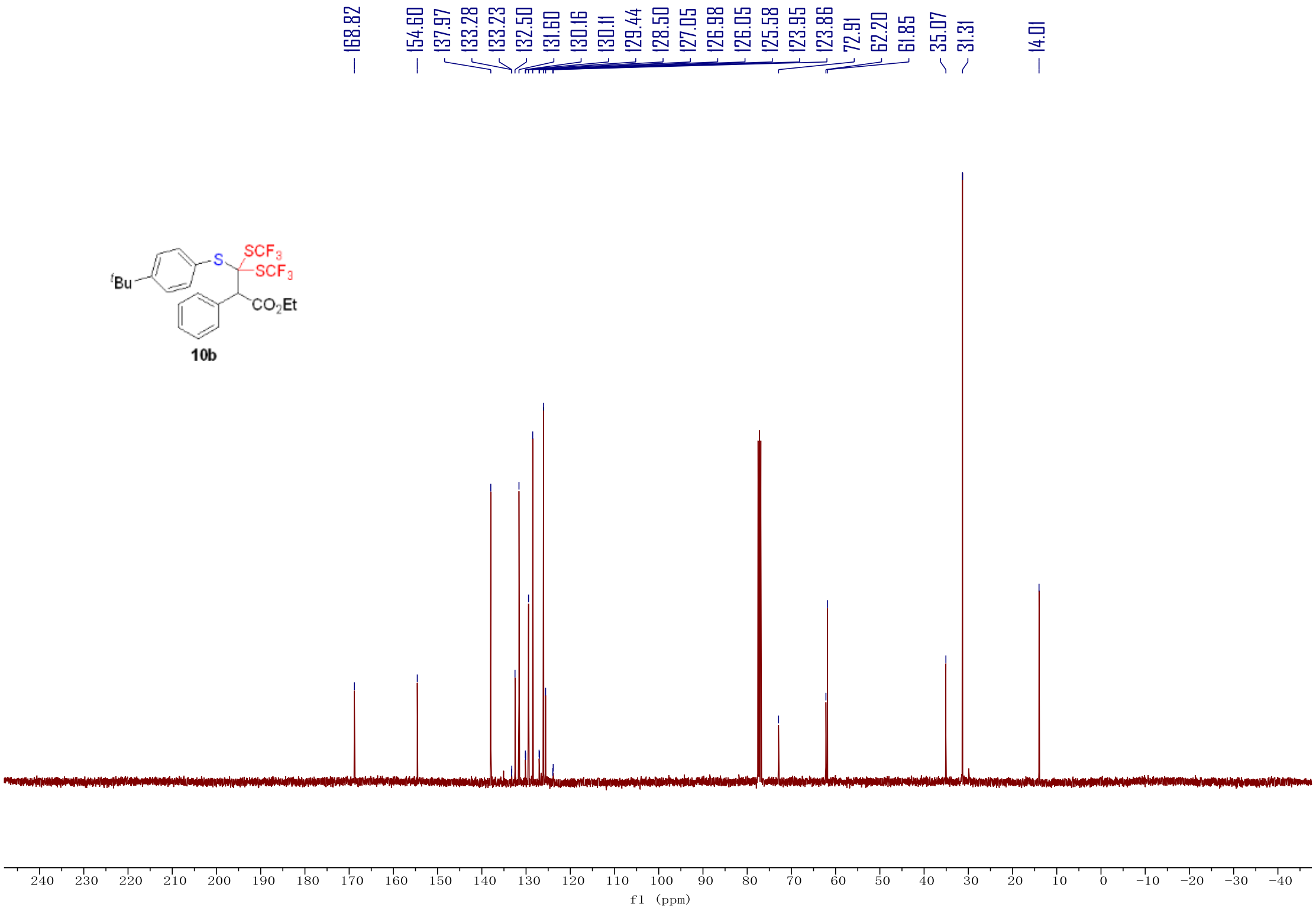

^{1}**H NMR** Spectrum of **10c** (400 MHz, $CDCl_3$)

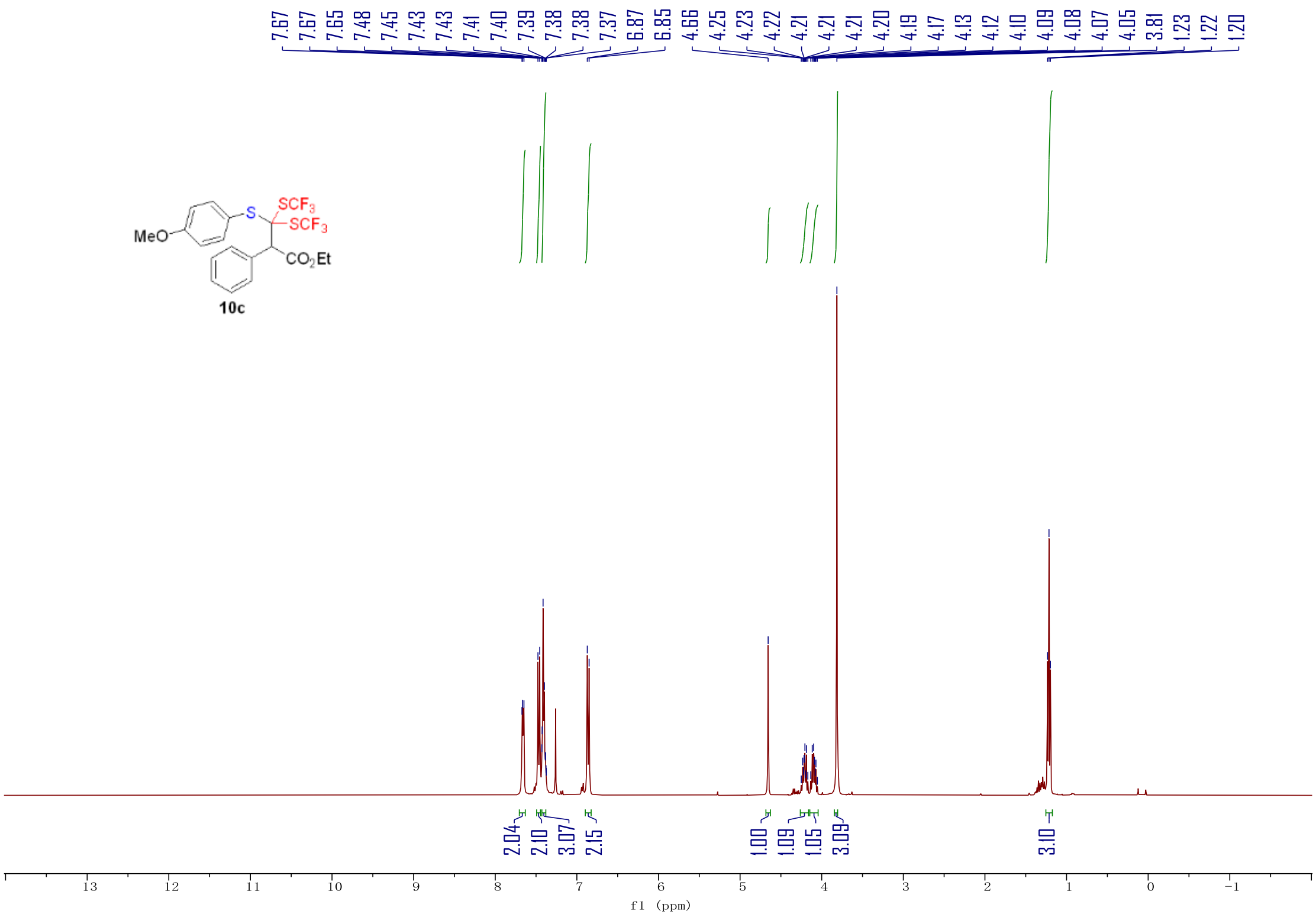

^{19}F NMR Spectrum of **10c** (376 MHz, $CDCl_3$)

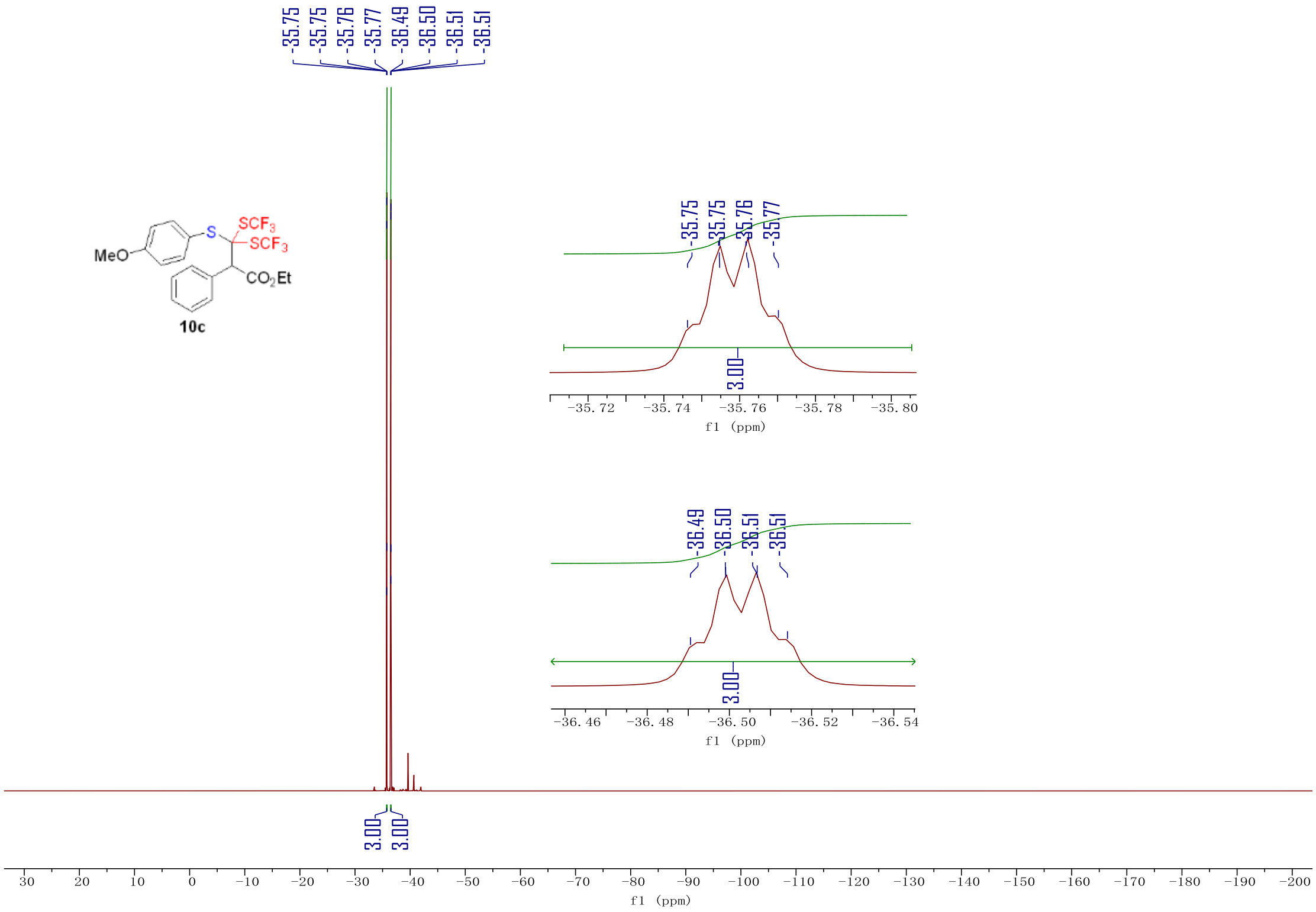

^{13}C NMR Spectrum of **10c** (101 MHz, $CDCl_3$)

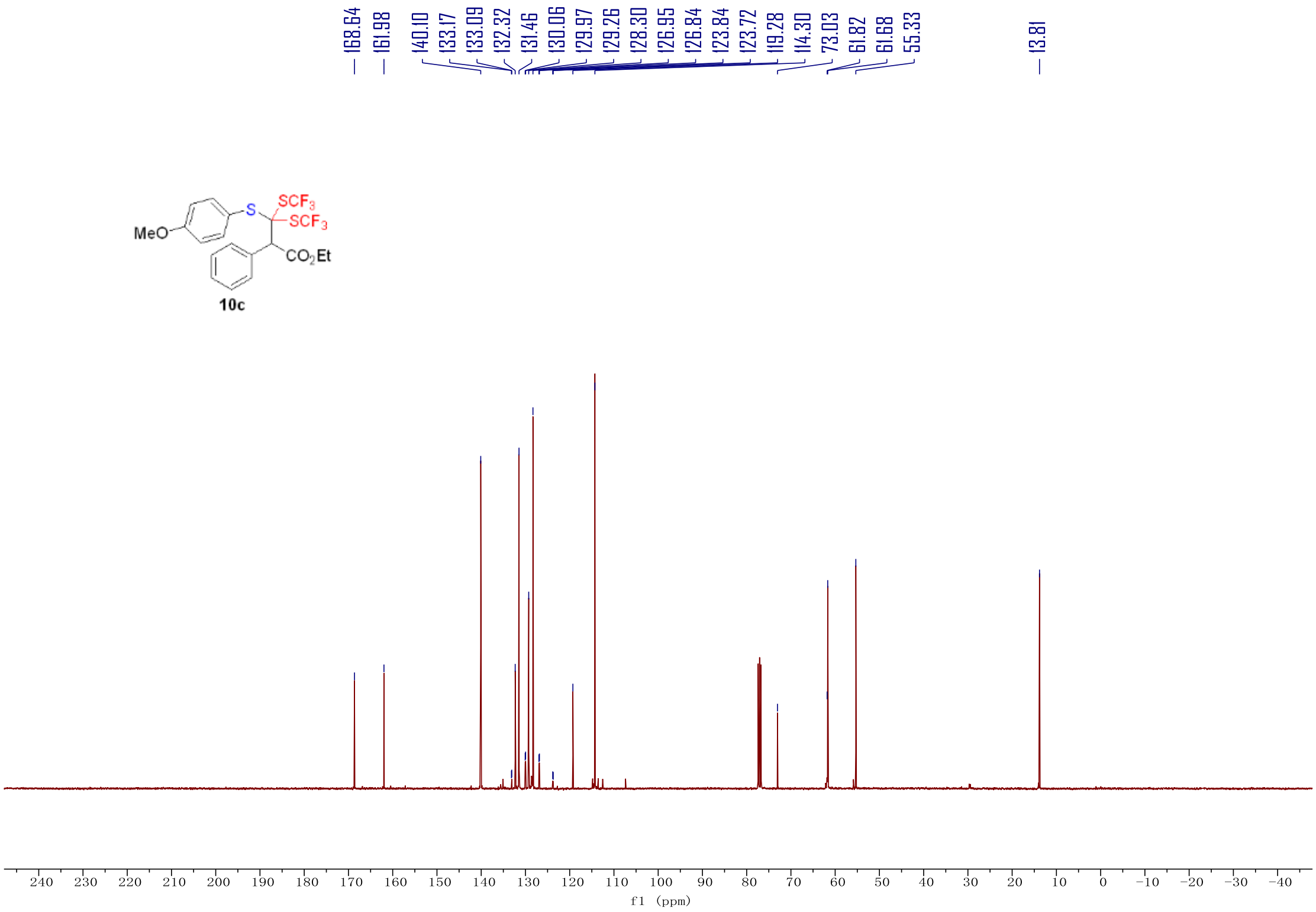

^{1}H NMR Spectrum of **10d** (400 MHz, $CDCl_3$)

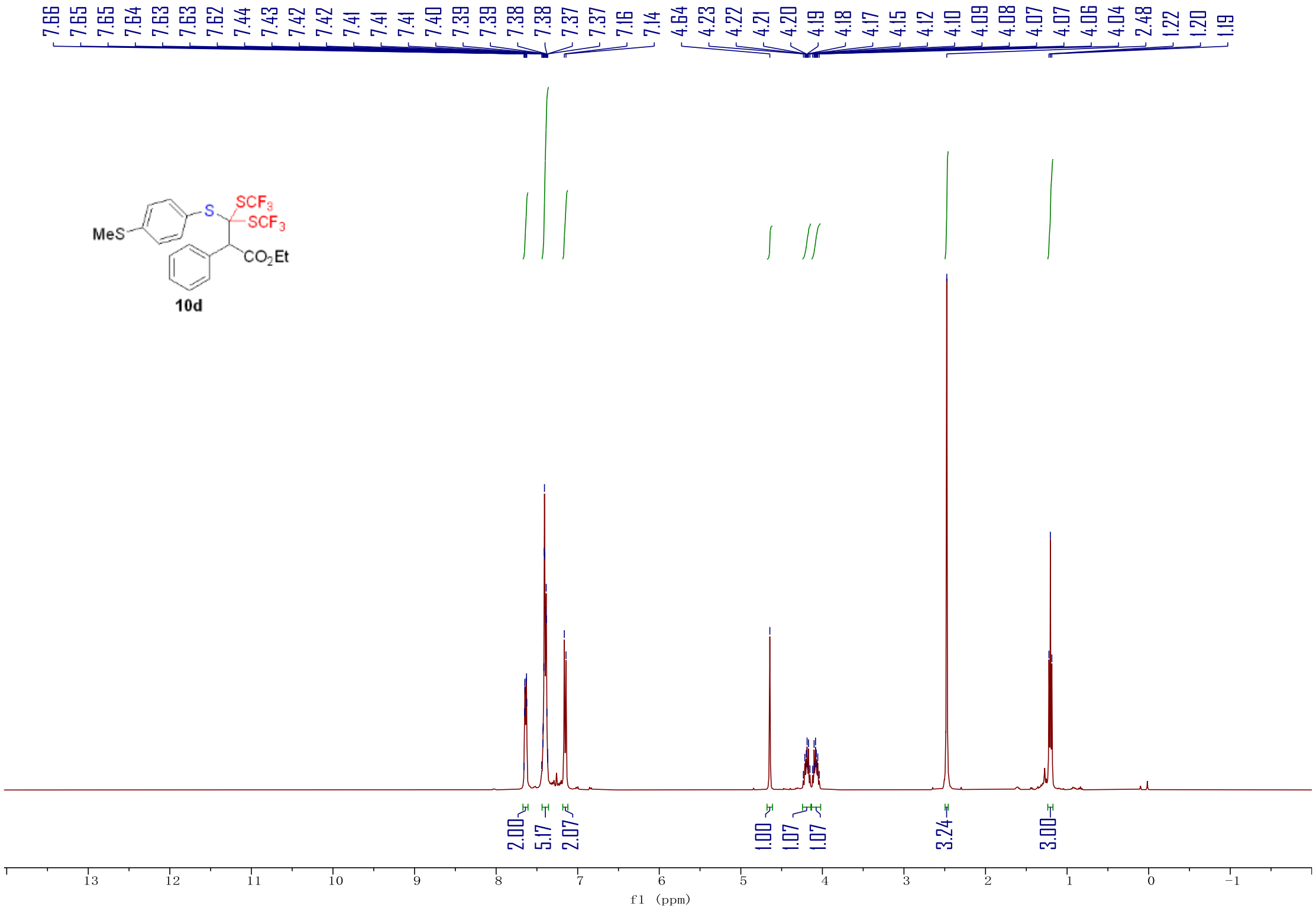

^{19}F NMR Spectrum of **10d** (376 MHz, $CDCl_3$)

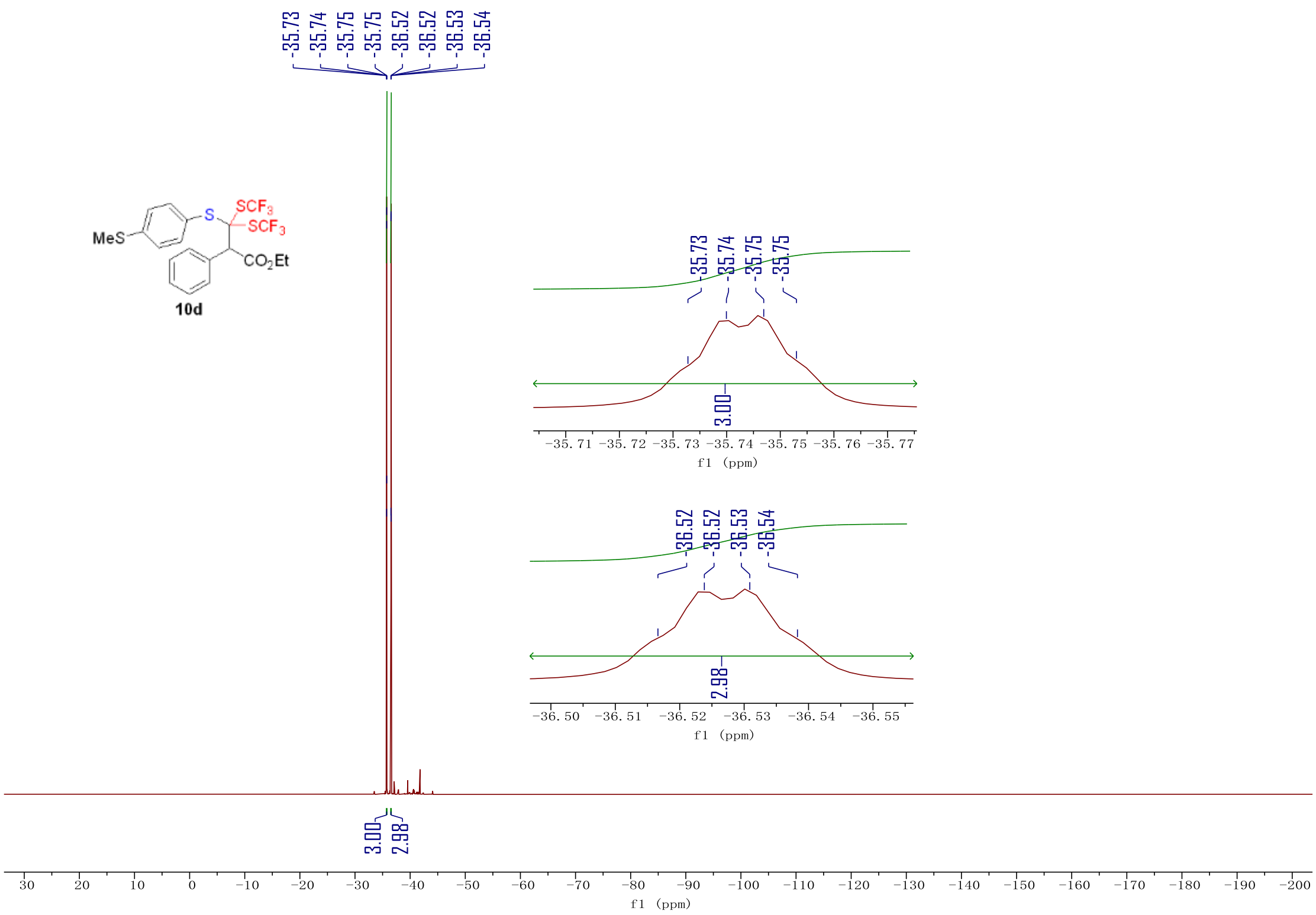

^{13}C NMR Spectrum of **10d** (101 MHz, $CDCl_3$)

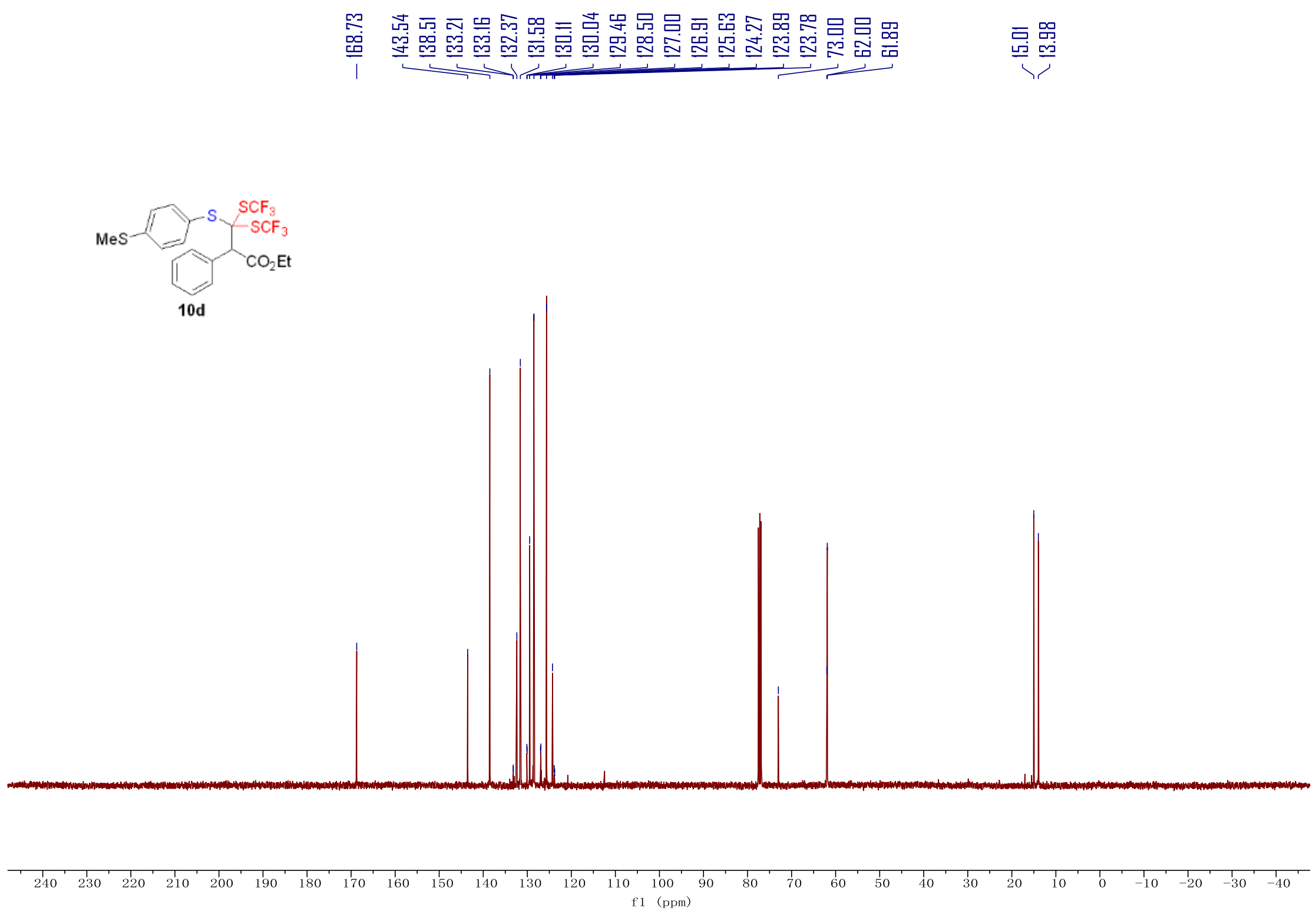

^{1}H NMR Spectrum of **10e** (400 MHz, $CDCl_3$)

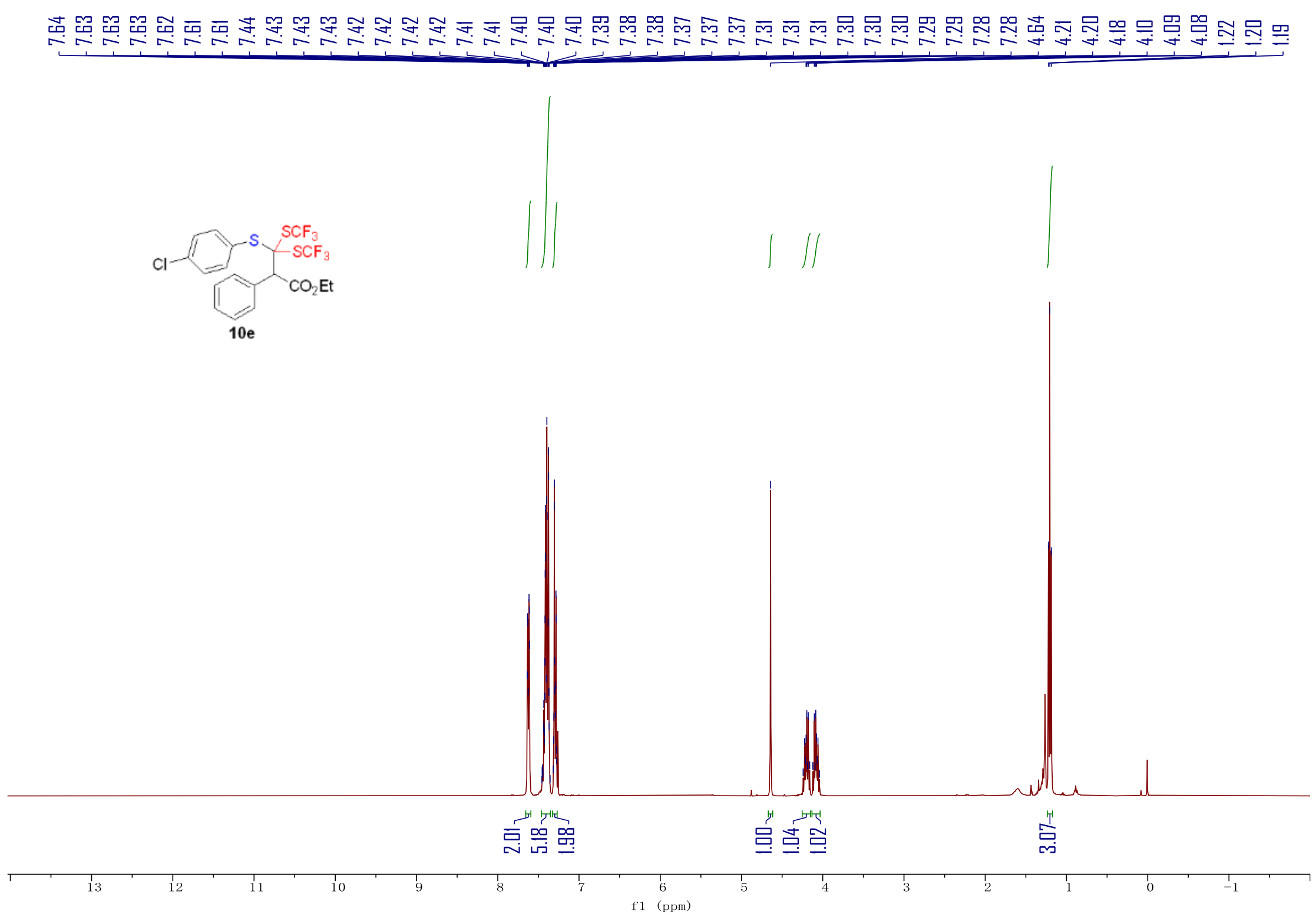

^{19}F NMR Spectrum of **10e** (376 MHz, $CDCl_3$)

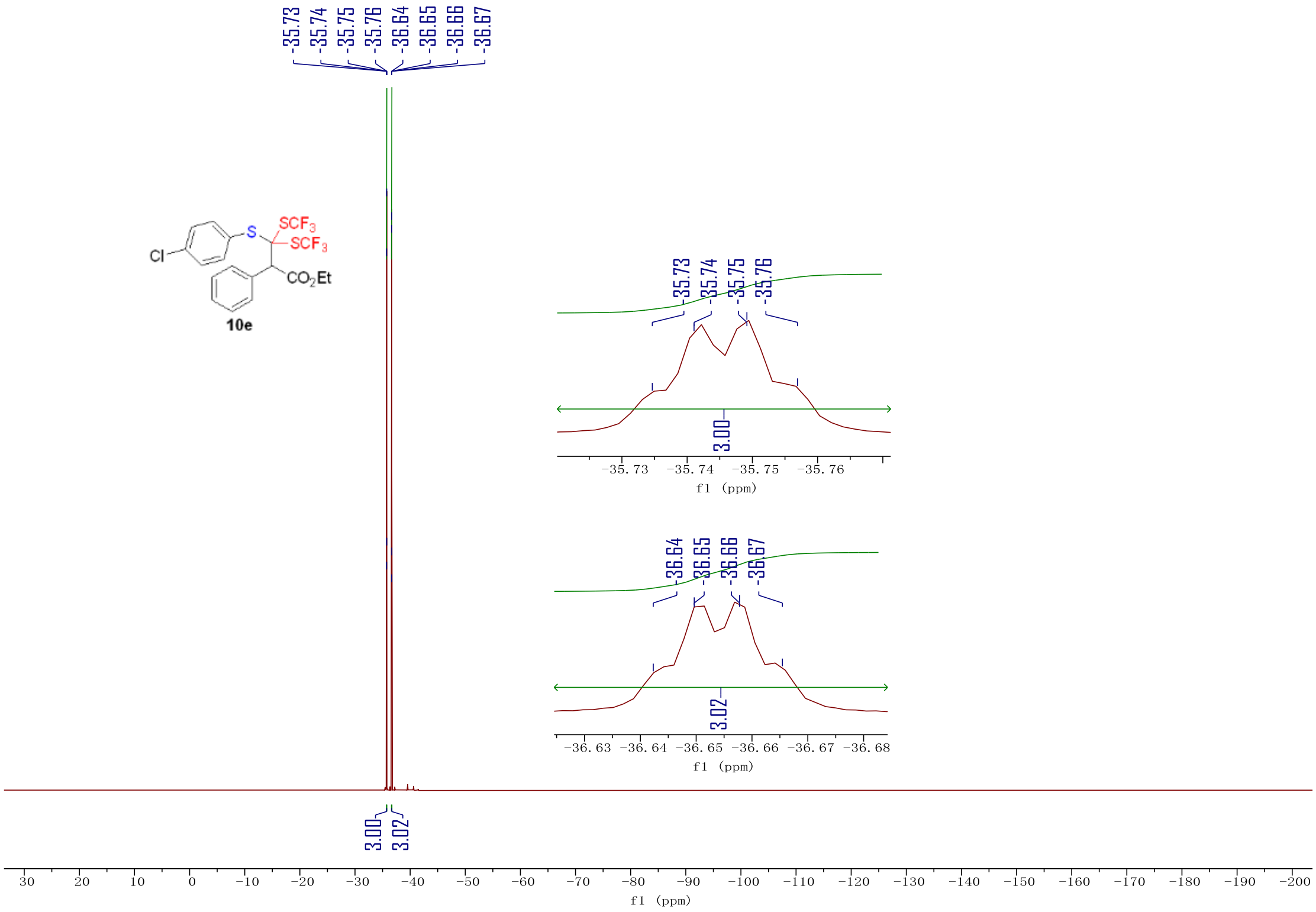

^{13}C NMR Spectrum of **10e** (101 MHz, $CDCl_3$)

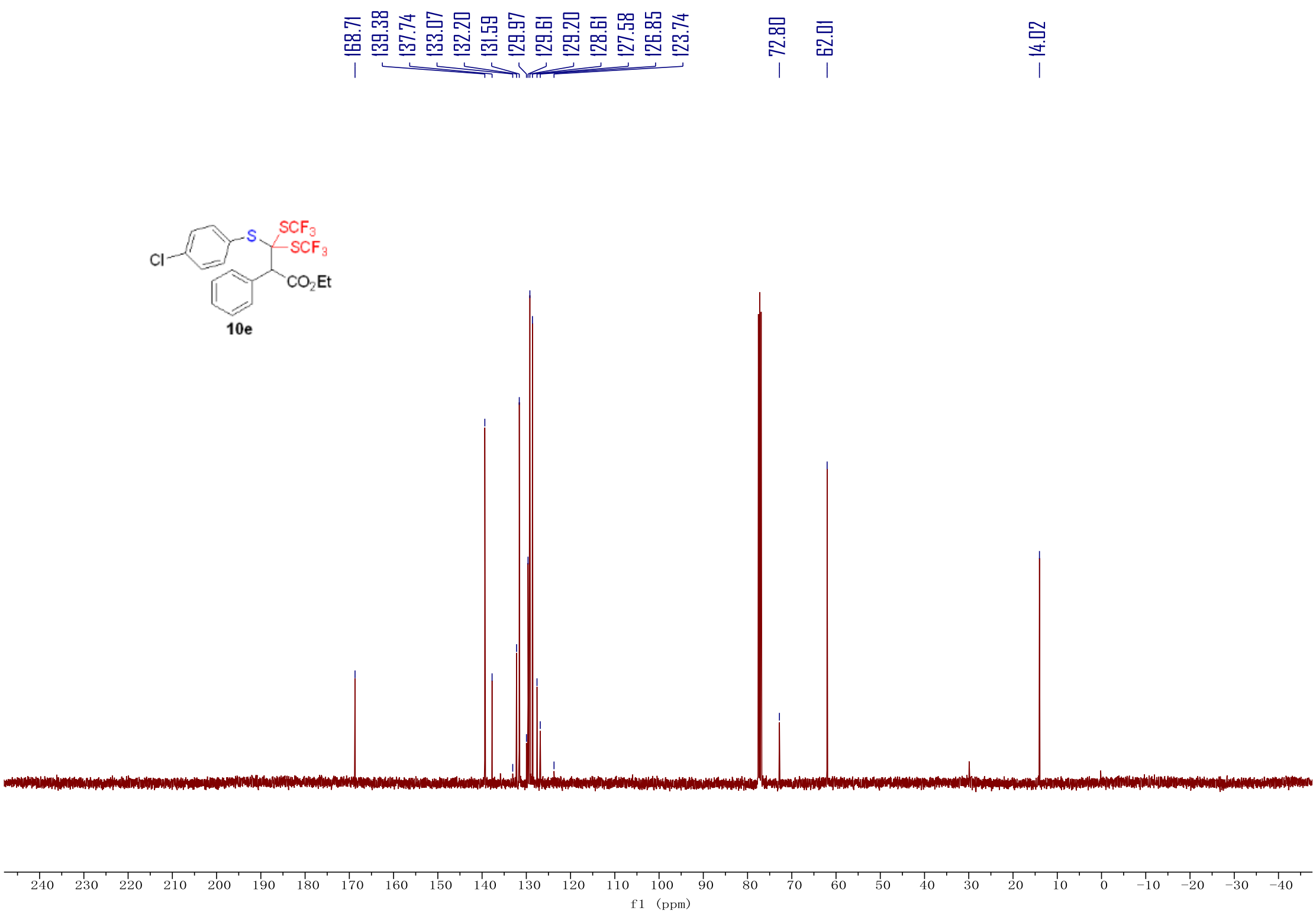

^{1}H NMR Spectrum of **10f** (400 MHz, $CDCl_3$)

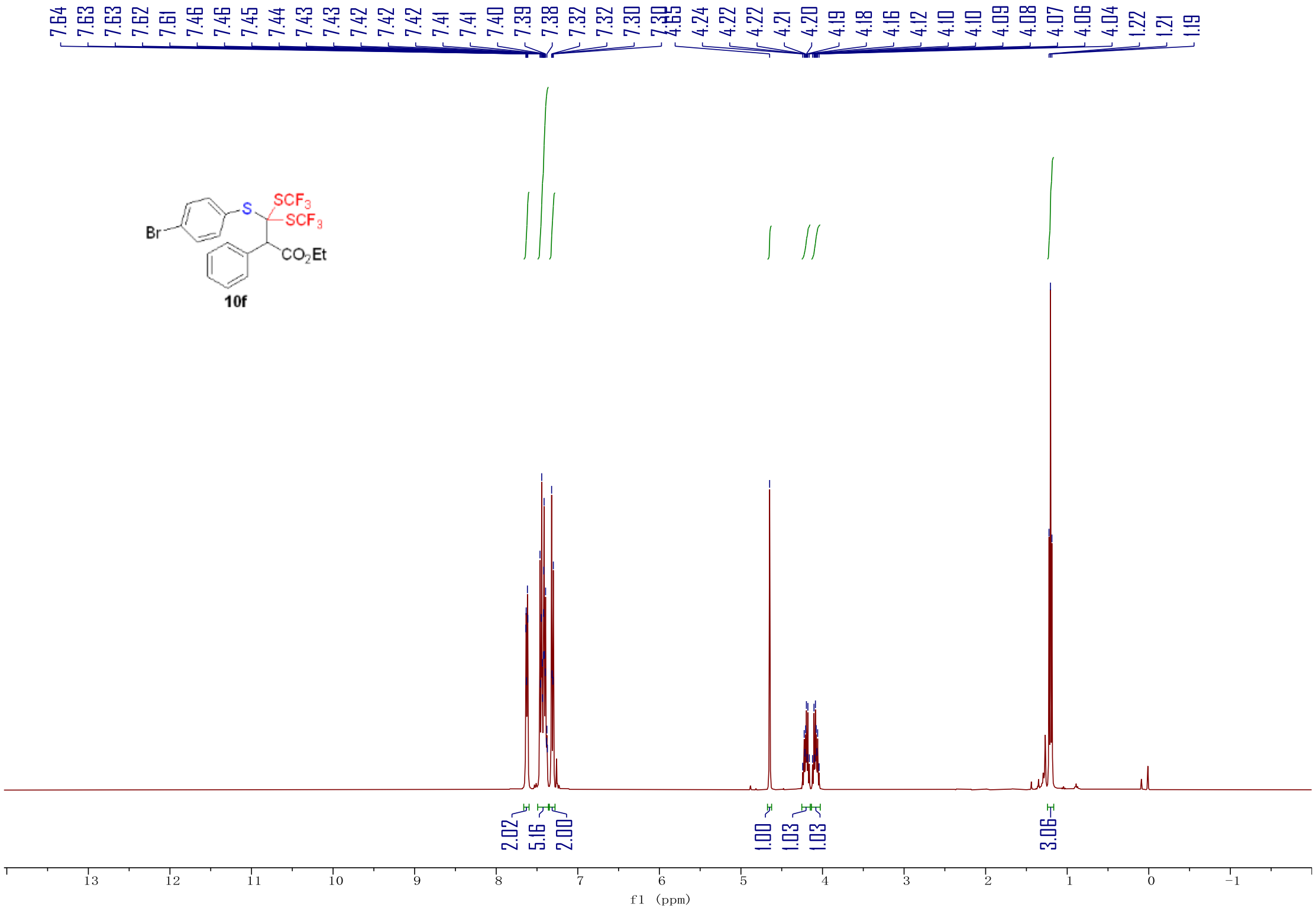

^{19}F NMR Spectrum of **10f** (376 MHz, $CDCl_3$)

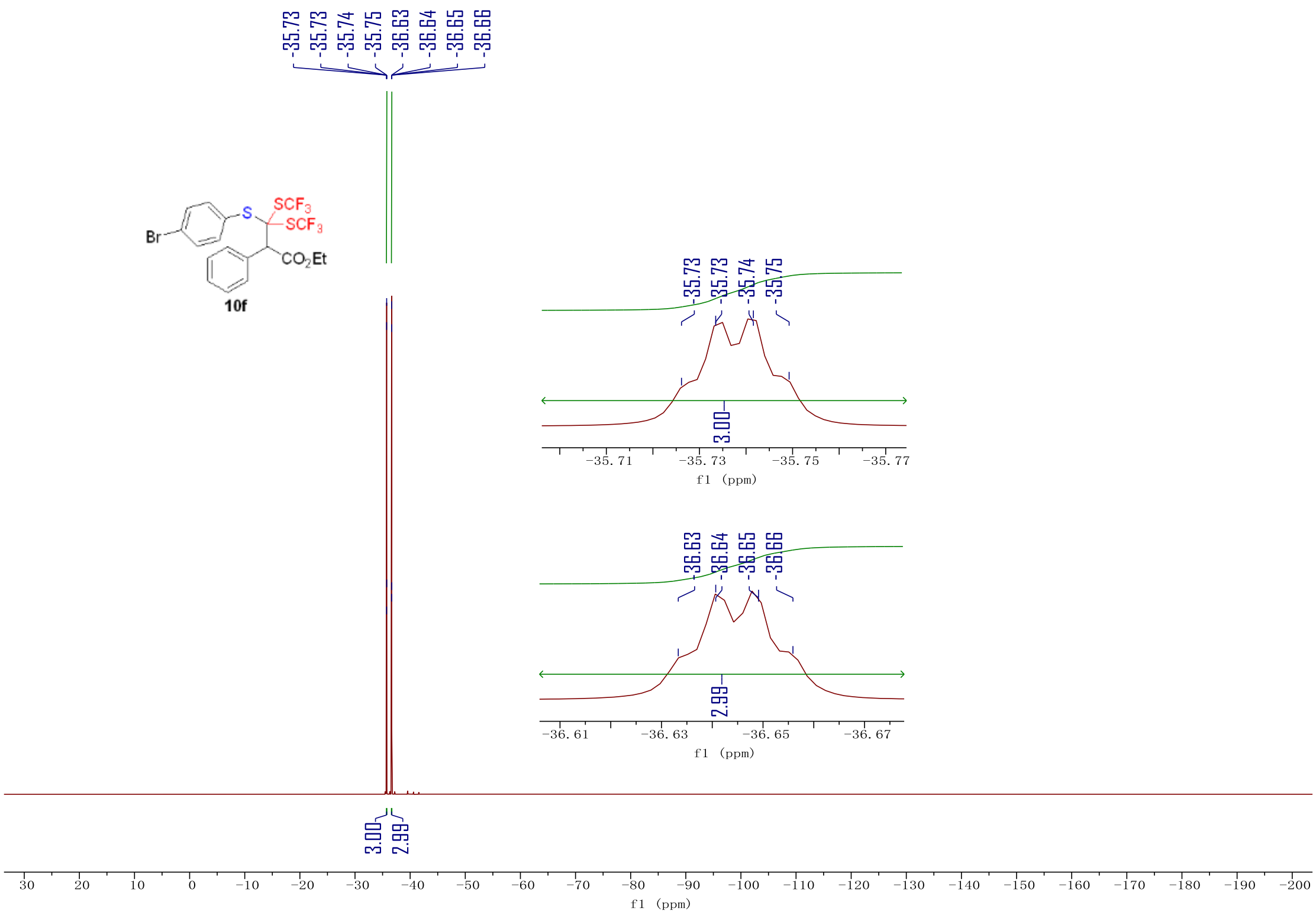

^{13}C NMR Spectrum of **10f** (101 MHz, $CDCl_3$)

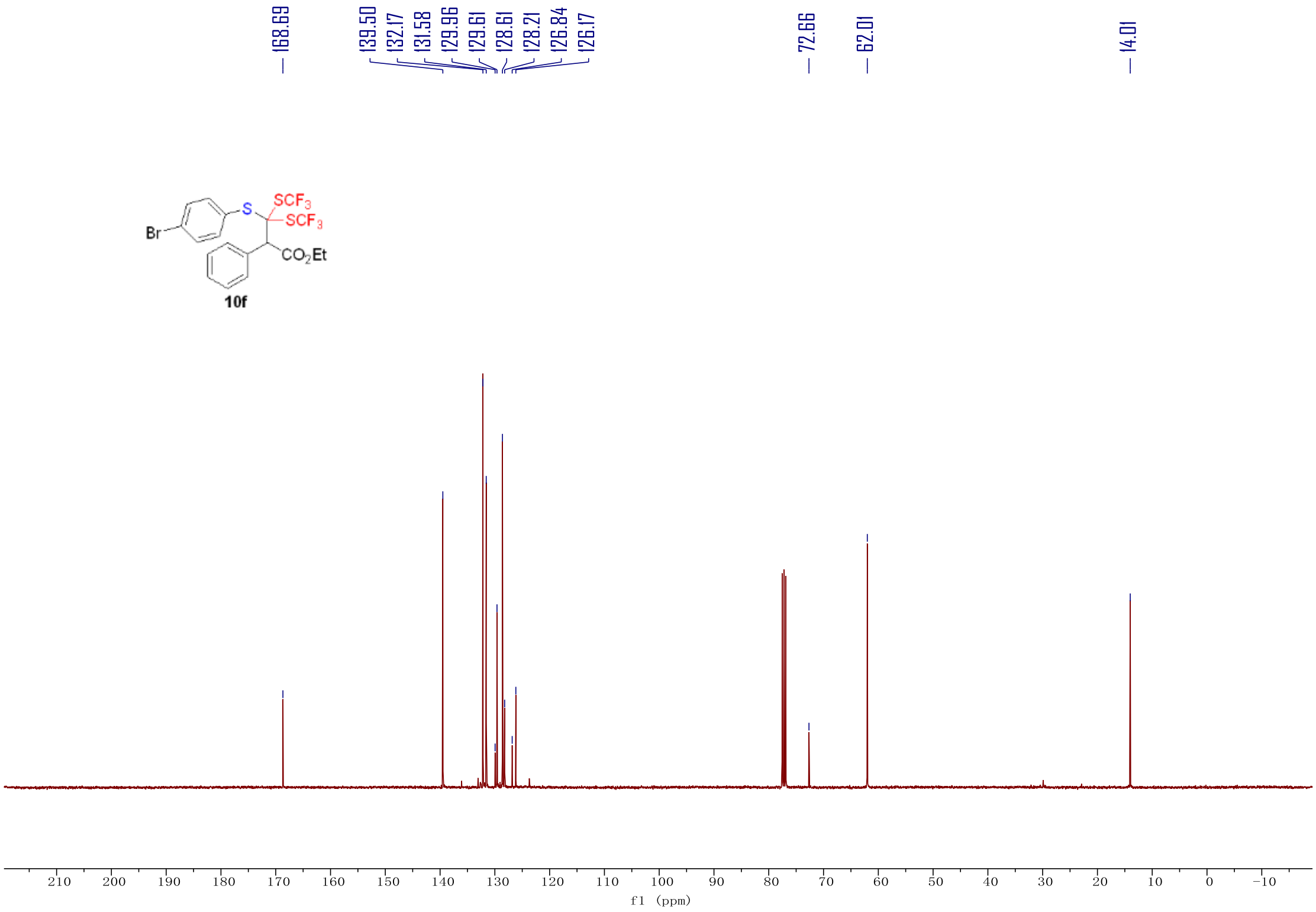

^{1}H NMR Spectrum of **10g** (400 MHz, $CDCl_3$)

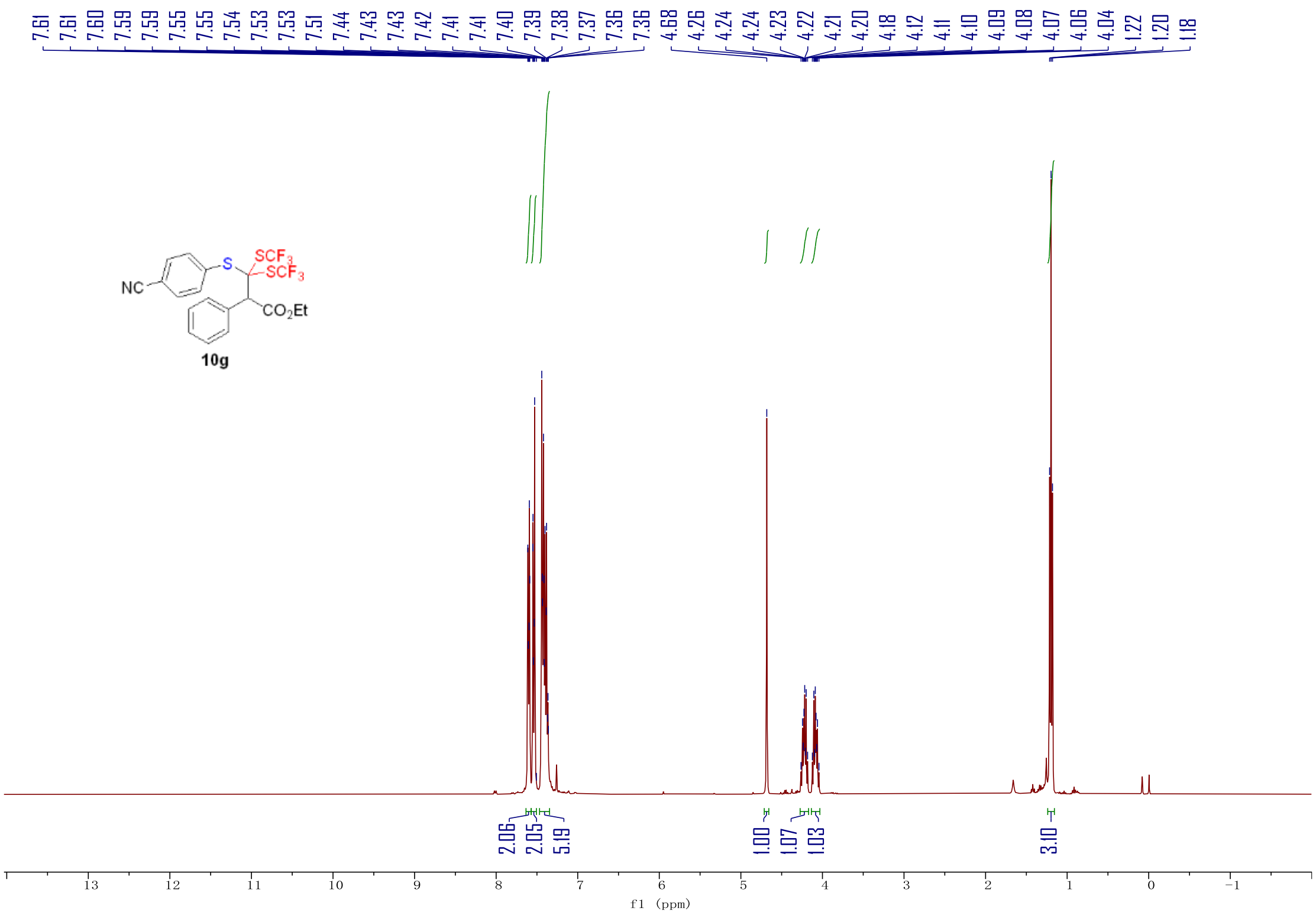

^{19}F NMR Spectrum of **10g** (376 MHz, $CDCl_3$)

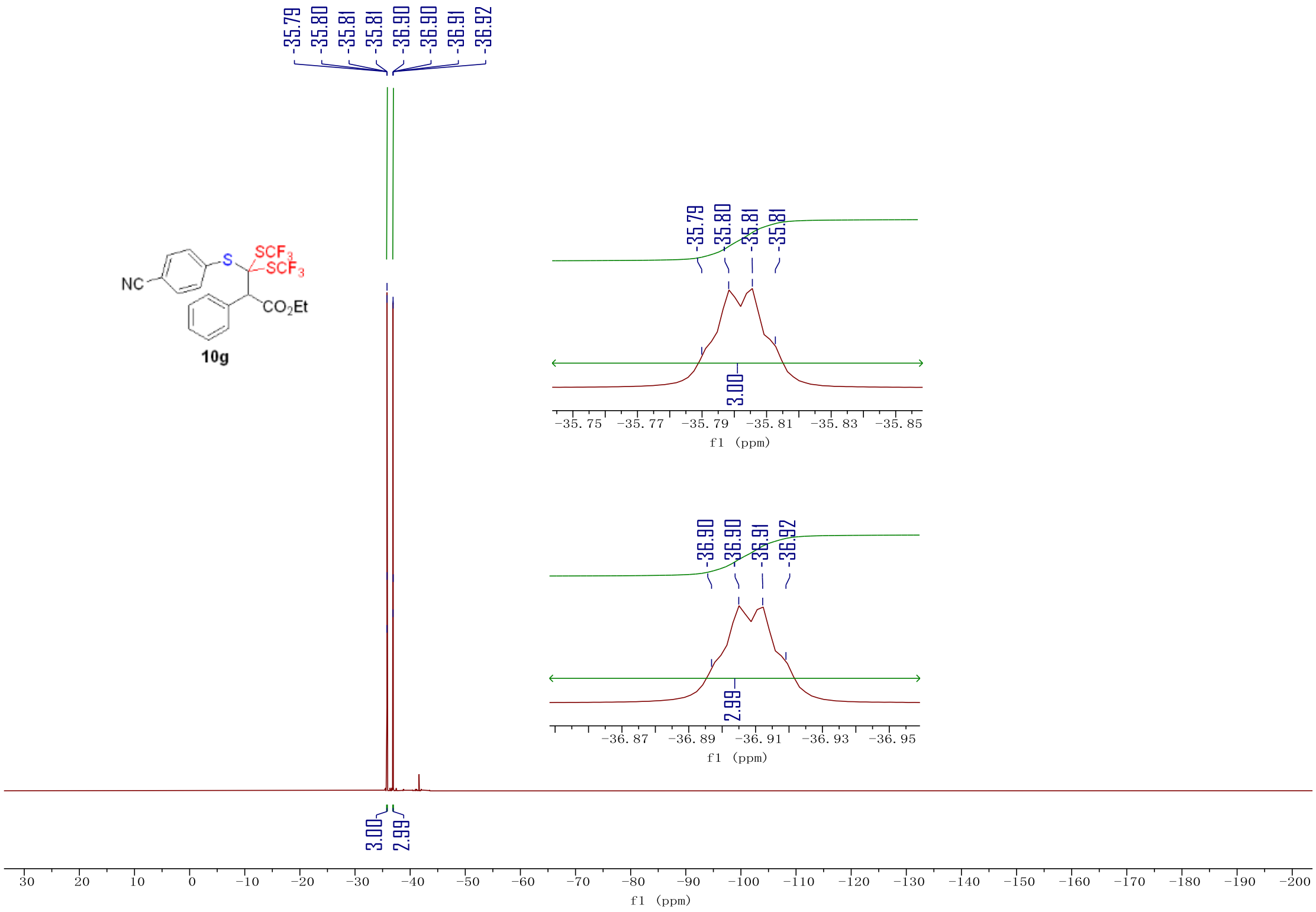

^{13}C NMR Spectrum of **10g** (101 MHz, $CDCl_3$)

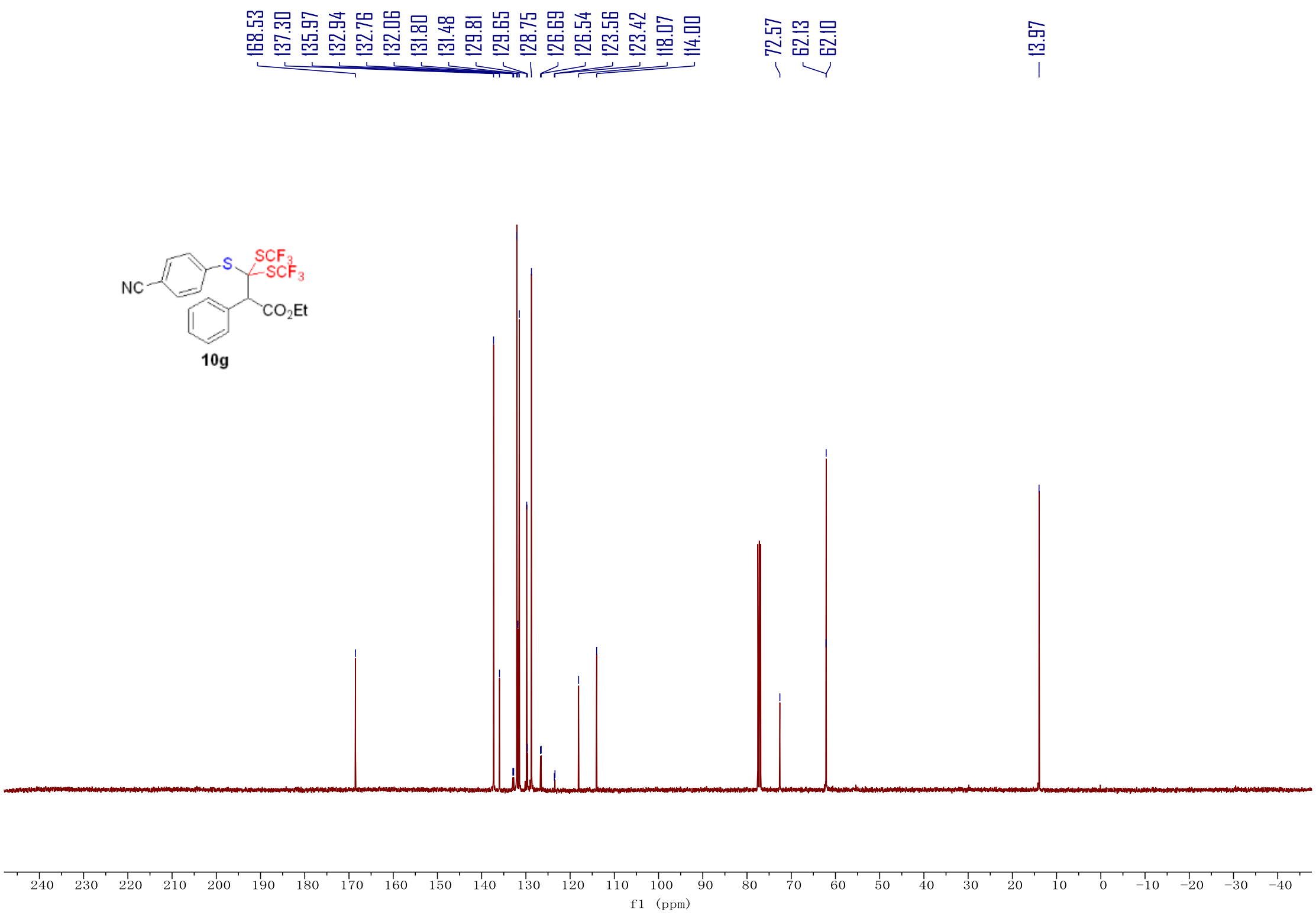

^{1}H NMR Spectrum of **10h** (400 MHz, $CDCl_3$)

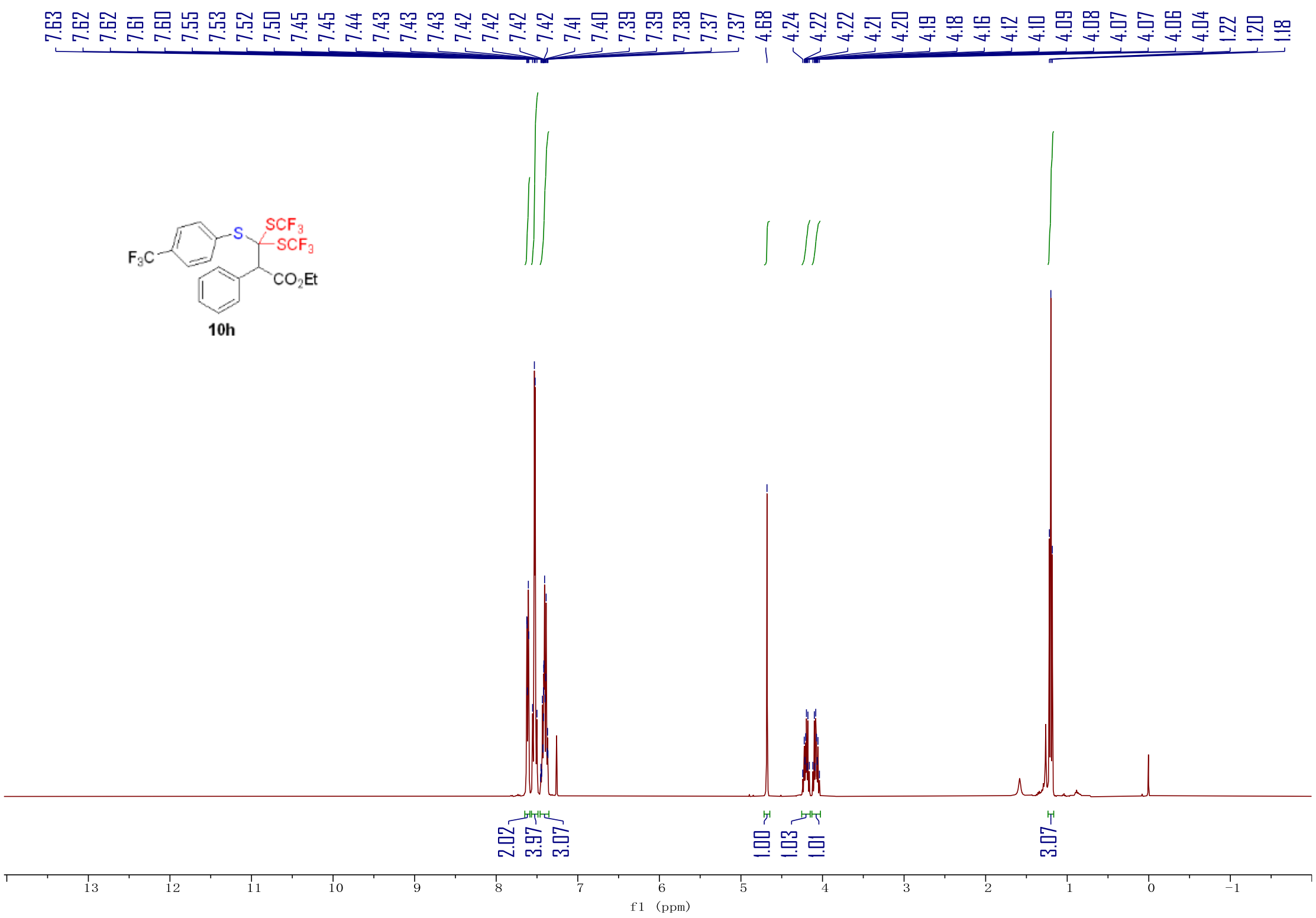

^{19}F NMR Spectrum of **10h** (376 MHz, $CDCl_3$)

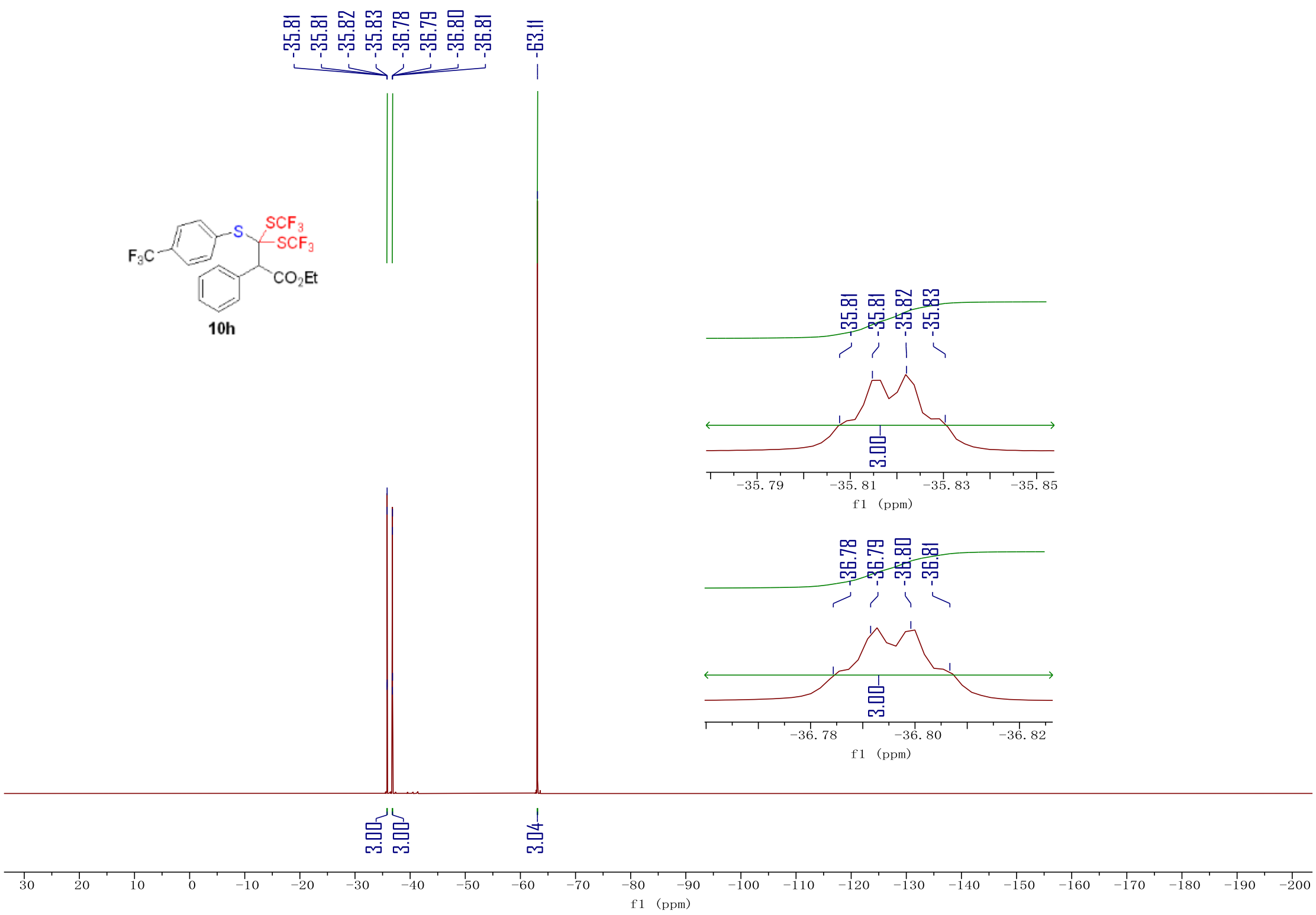

^{13}**C NMR** Spectrum of **10h** (101 MHz, $CDCl_3$)

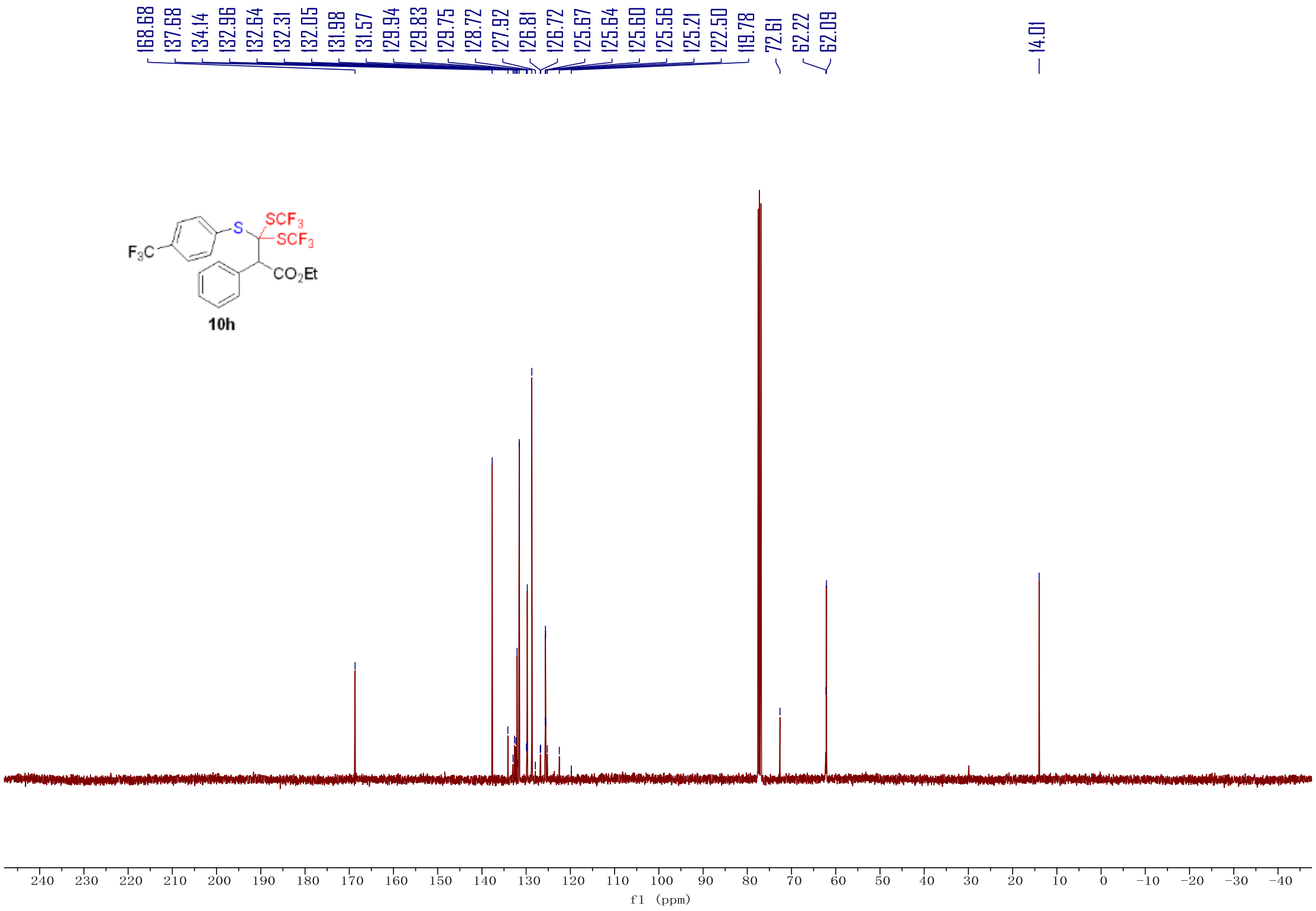

^{1}**H NMR** Spectrum of **10i** (400 MHz, $CDCl_3$)

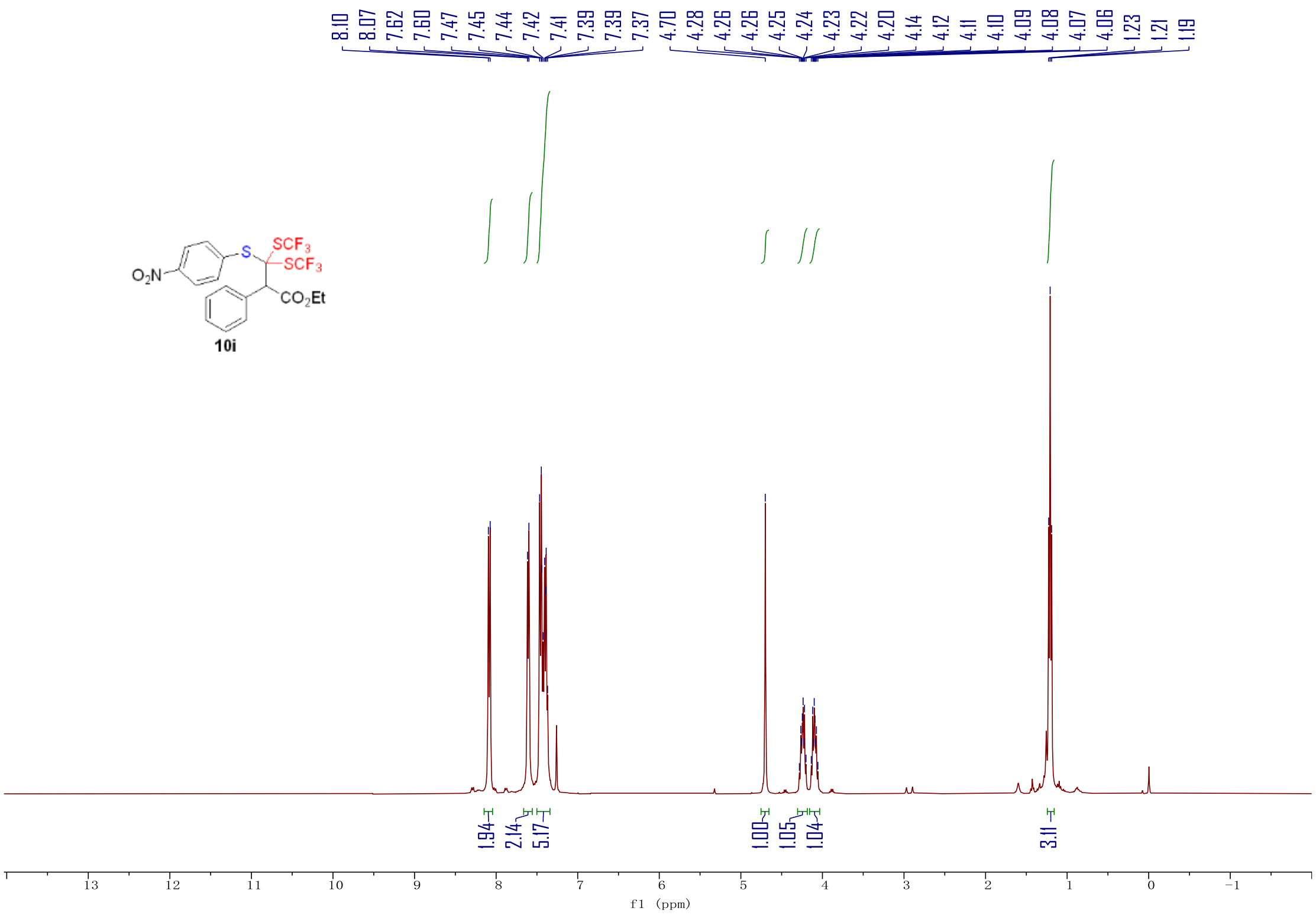

^{19}F NMR Spectrum of **10i** (376 MHz, $CDCl_3$)

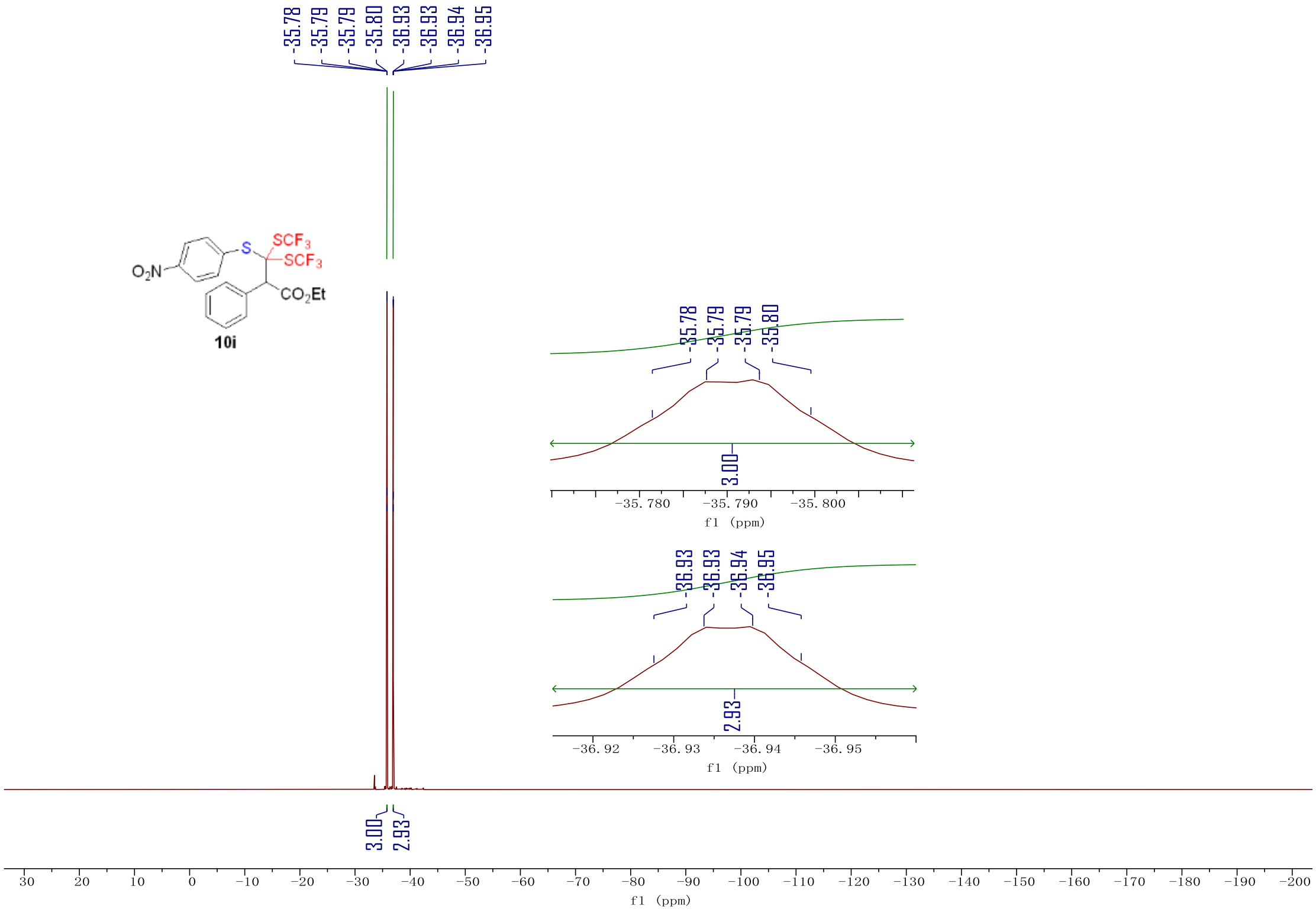

^{13}C NMR Spectrum of **10i** (101 MHz, $CDCl_3$)

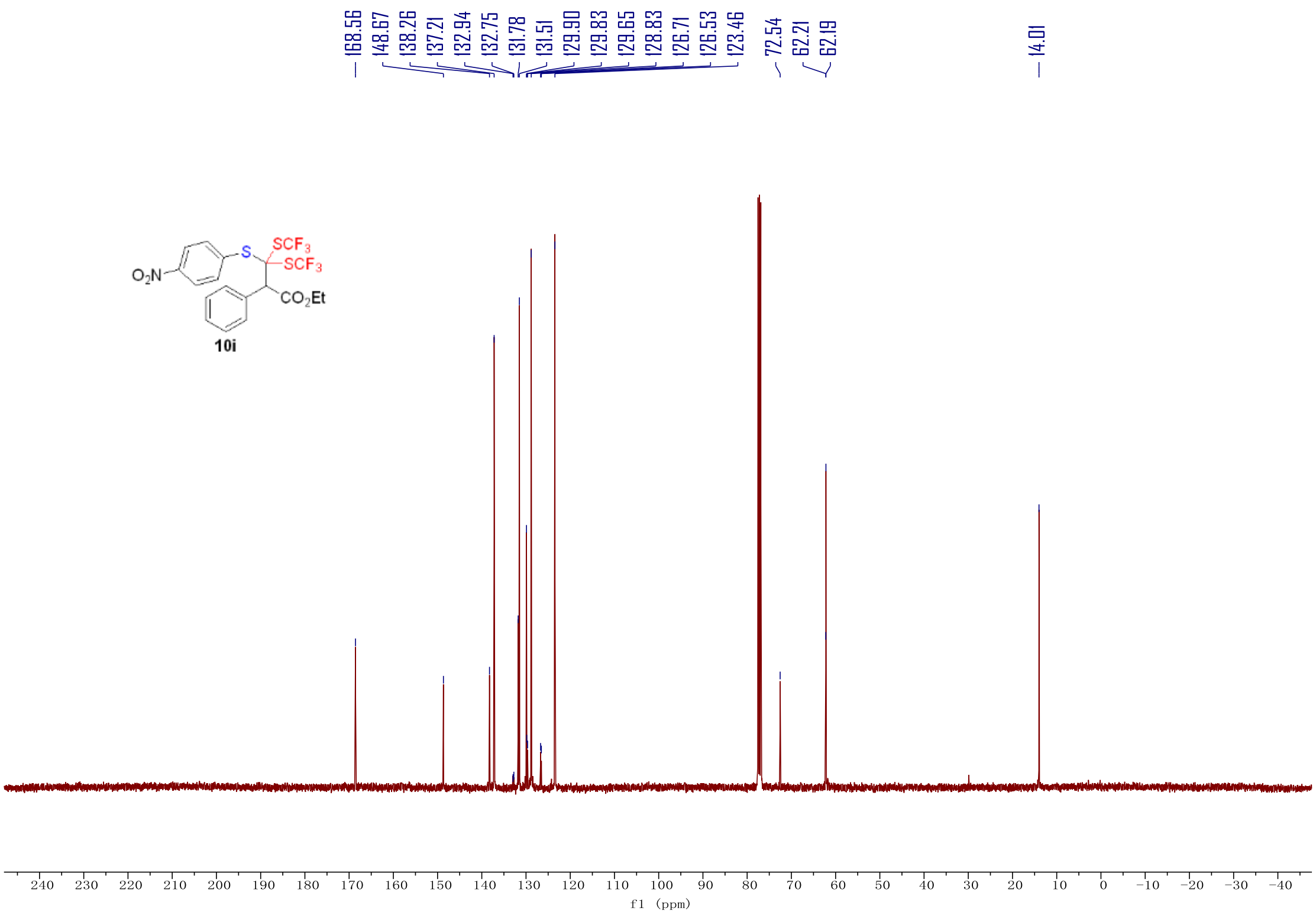

^{1}H NMR Spectrum of **10j** (400 MHz, $CDCl_3$)

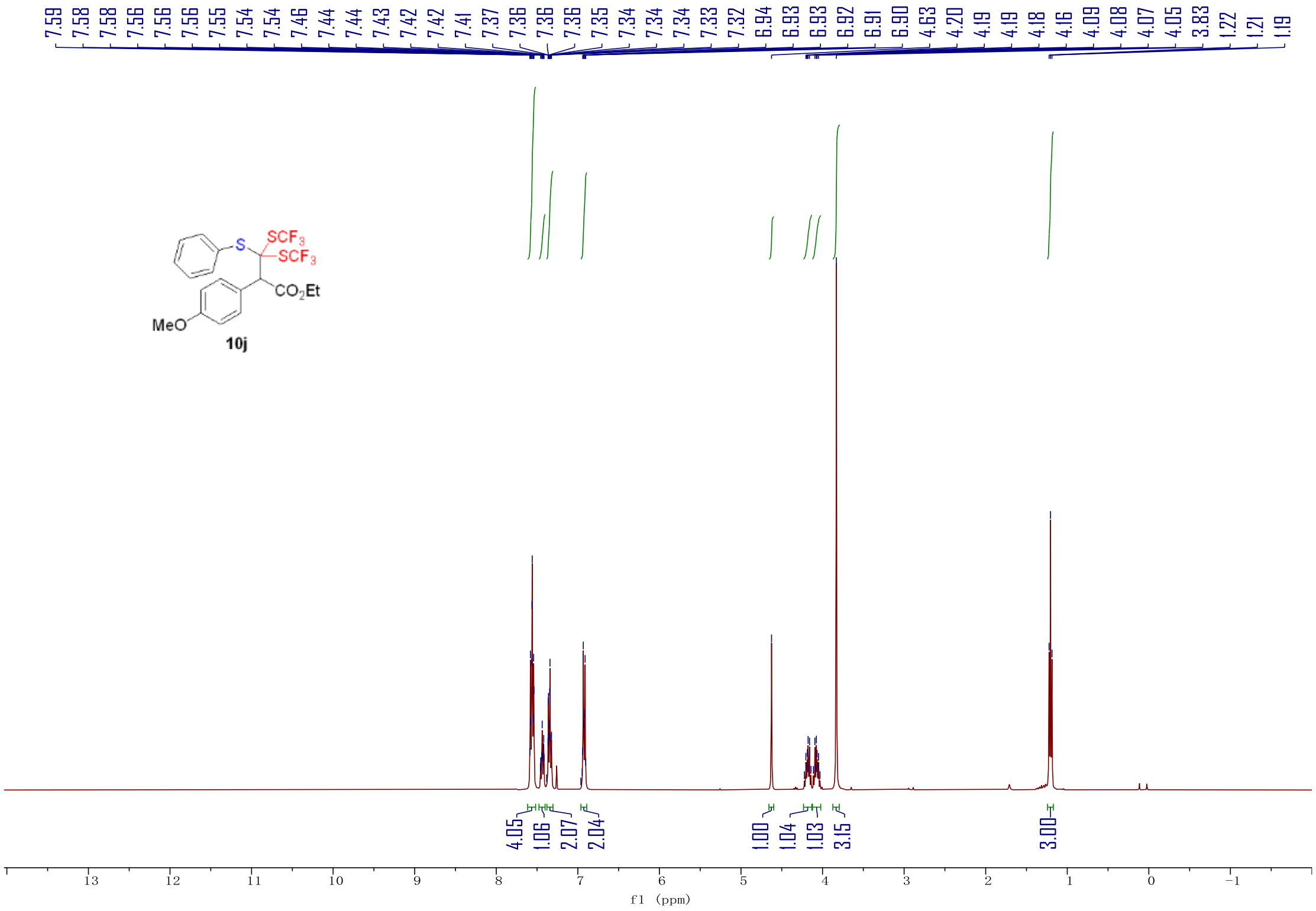

^{19}F NMR Spectrum of **10j** (376 MHz, $CDCl_3$)

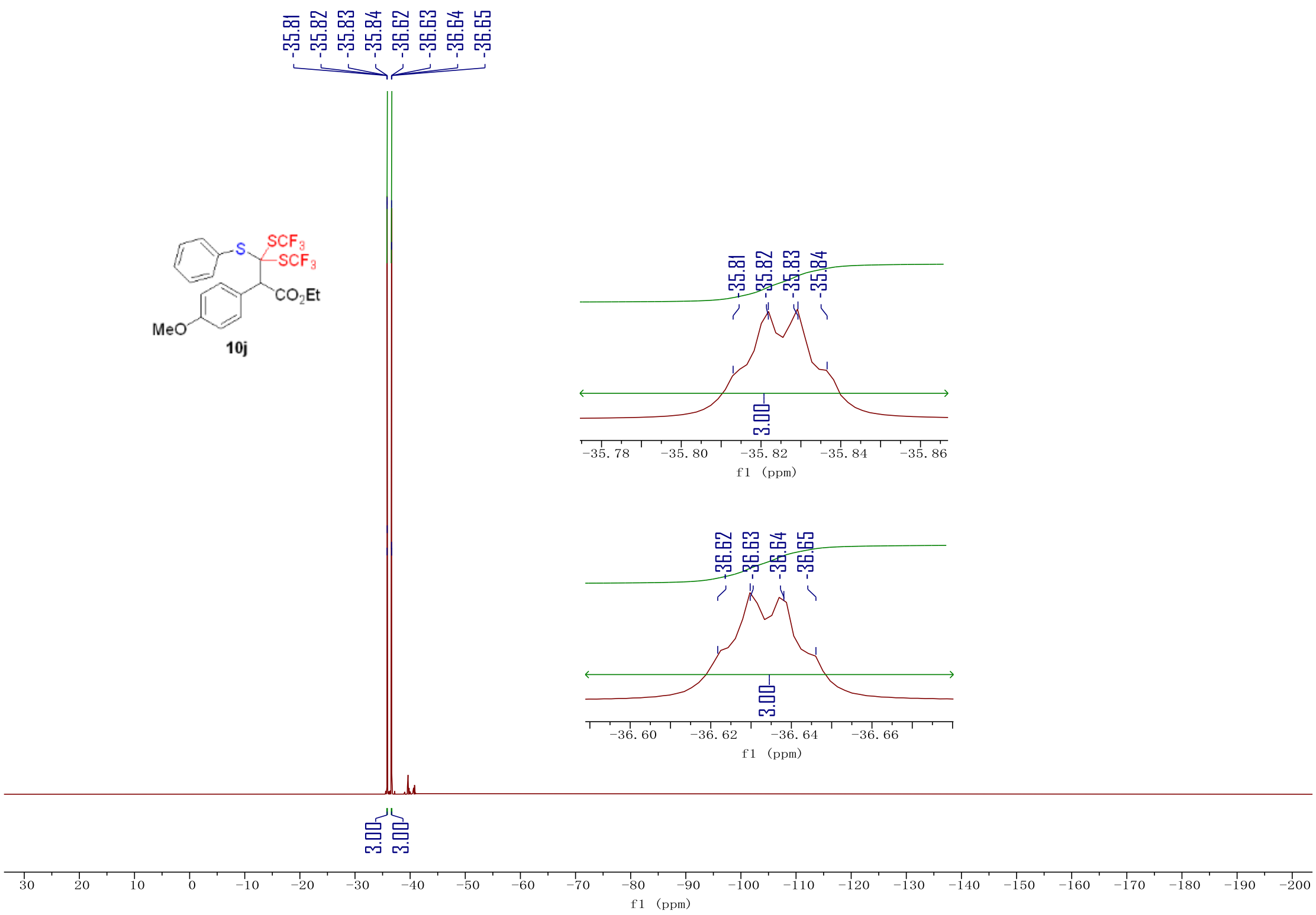

^{13}C NMR Spectrum of **10j** (101 MHz, $CDCl_3$)

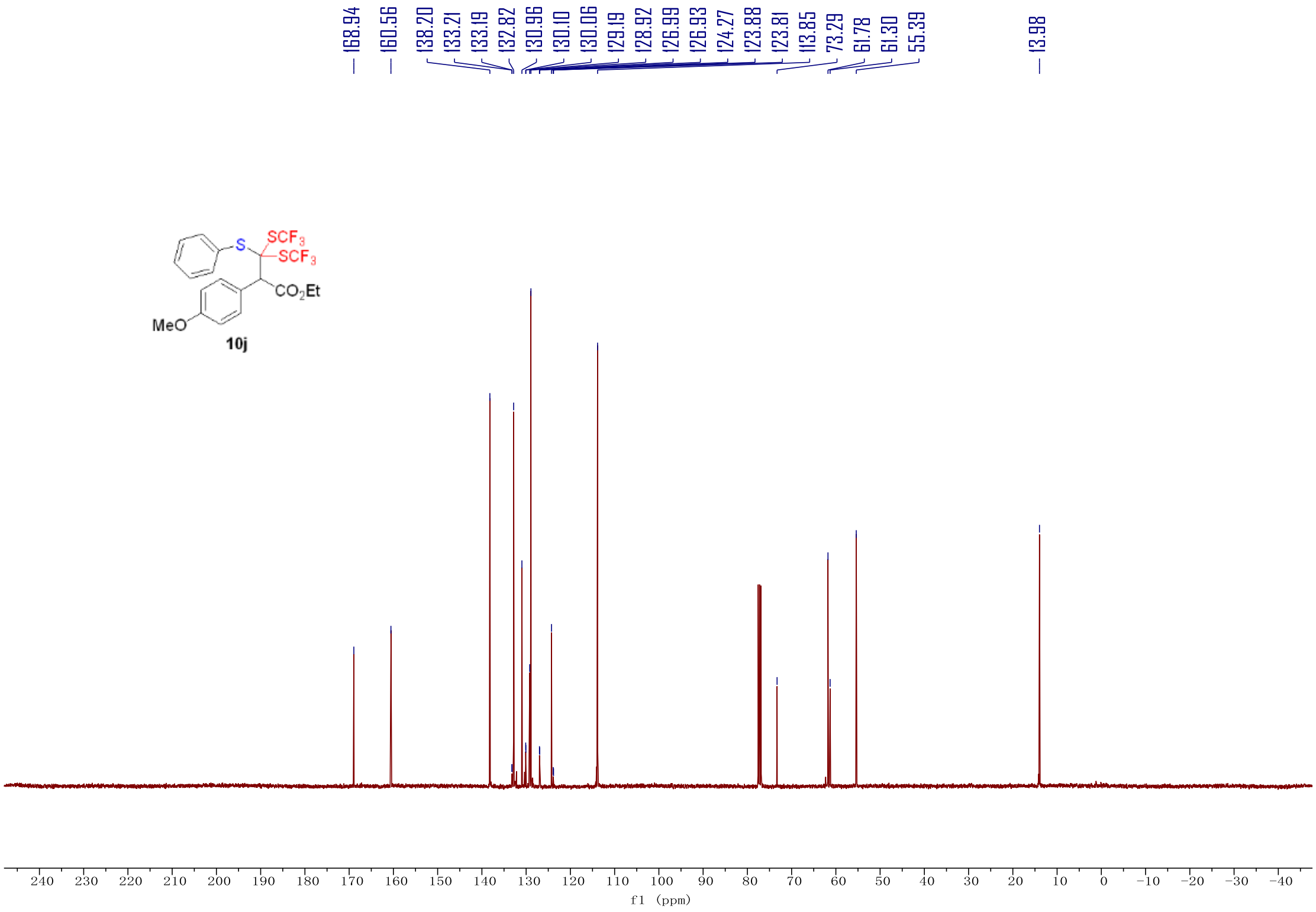

^{1}H NMR Spectrum of **10k** (400 MHz, $CDCl_3$)

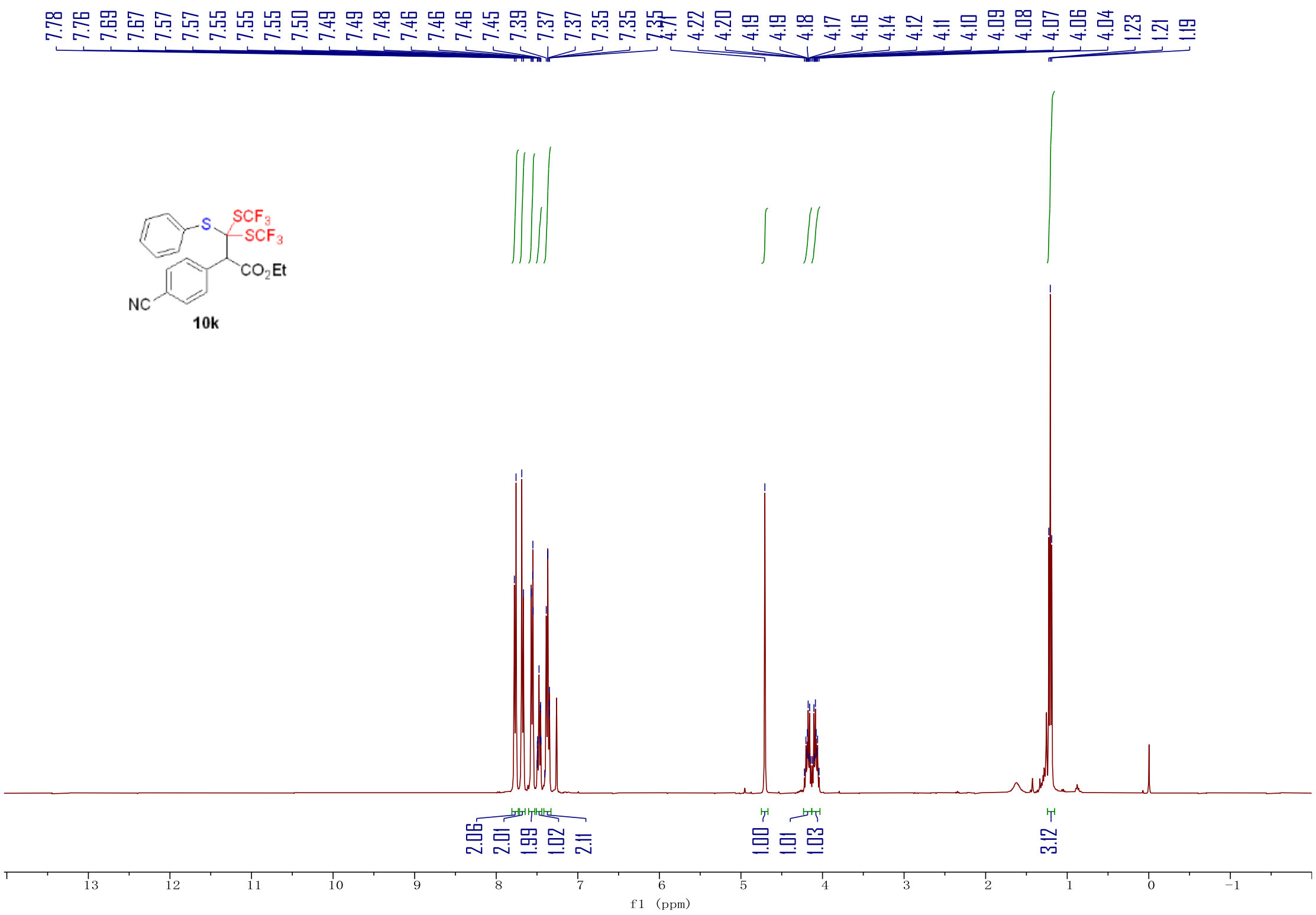

^{19}F NMR Spectrum of **10k** (376 MHz, $CDCl_3$)

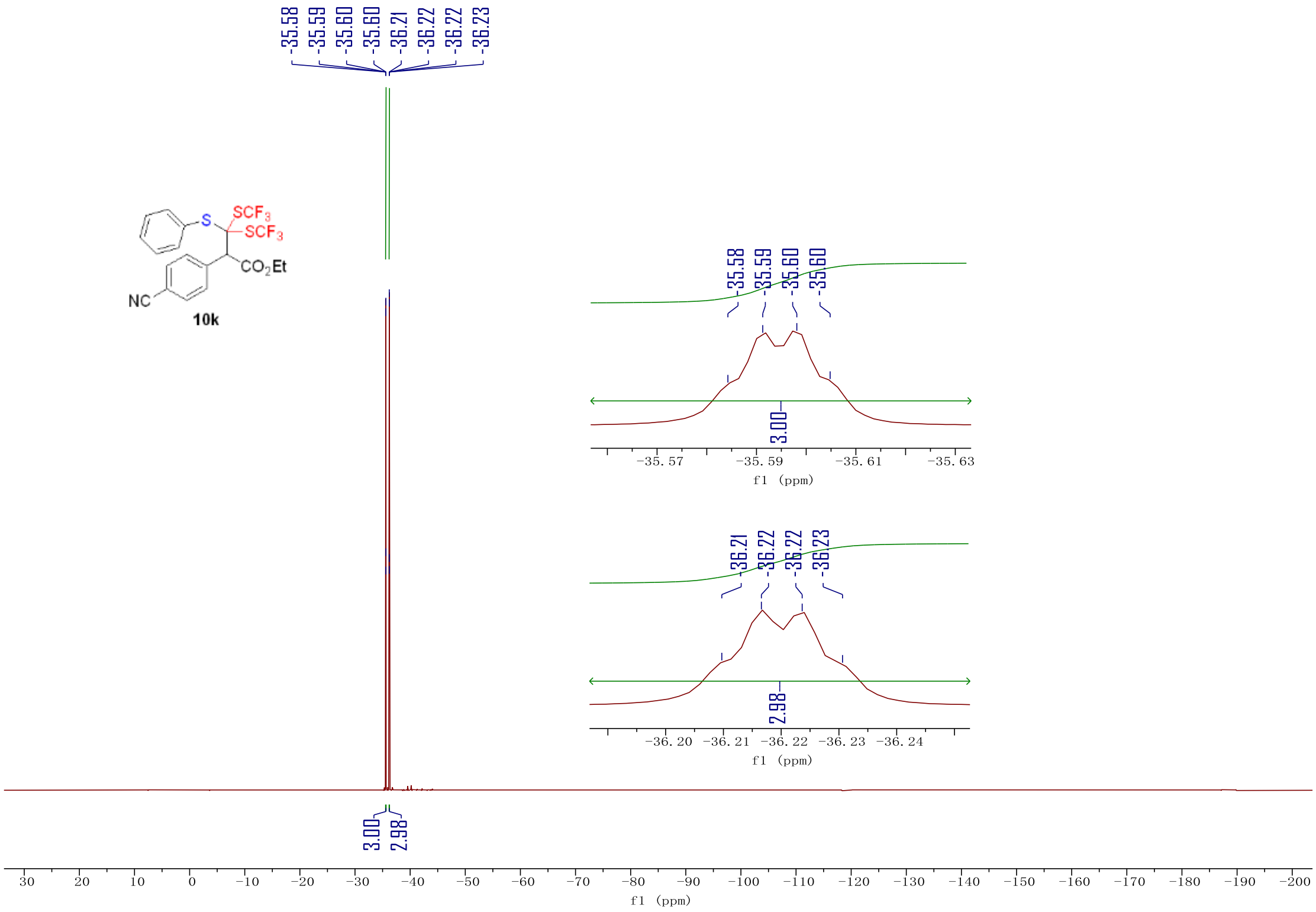

^{13}C NMR Spectrum of **10k** (101 MHz, $CDCl_3$)

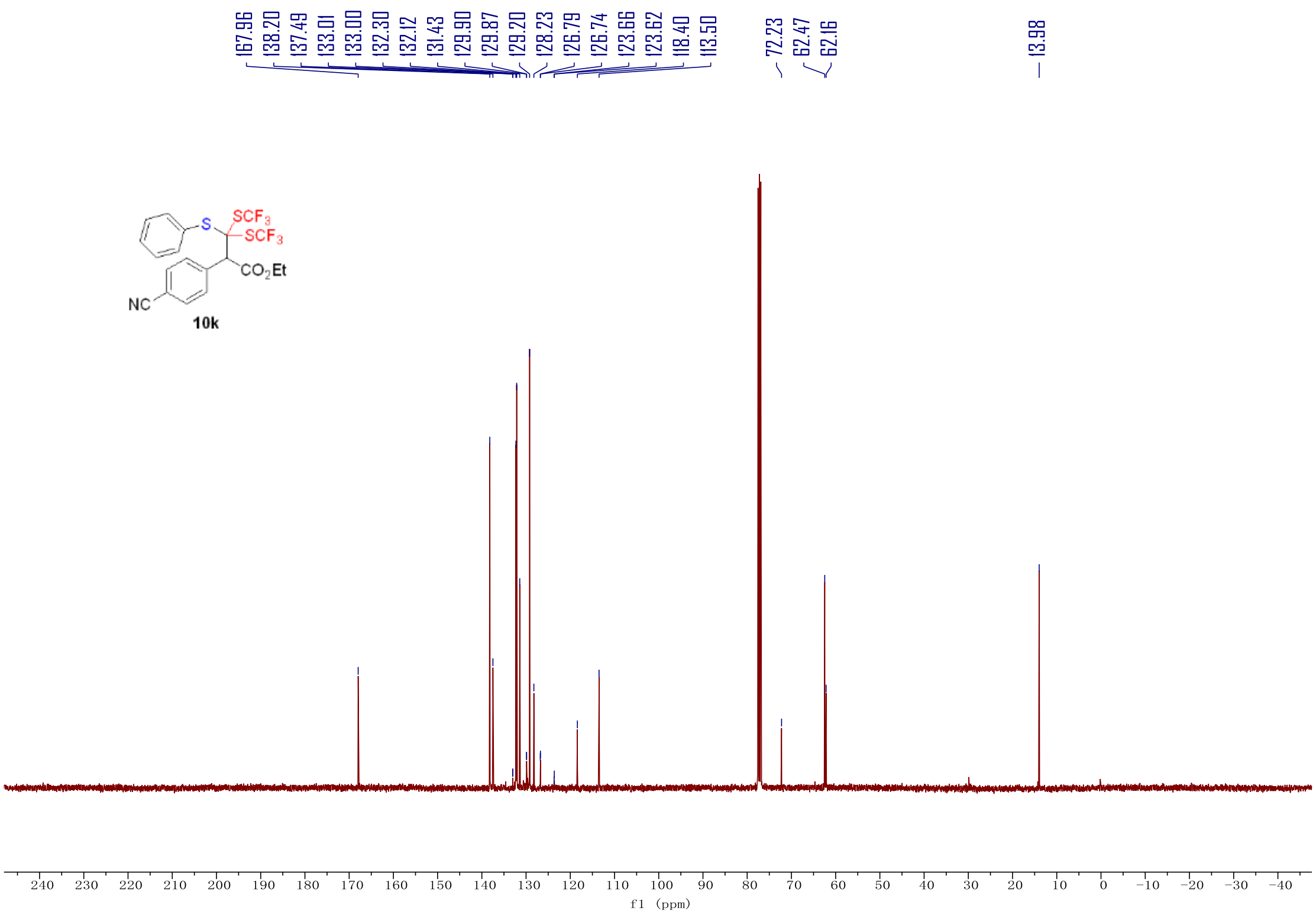

^{1}H NMR Spectrum of **10l** (400 MHz, $CDCl_3$)

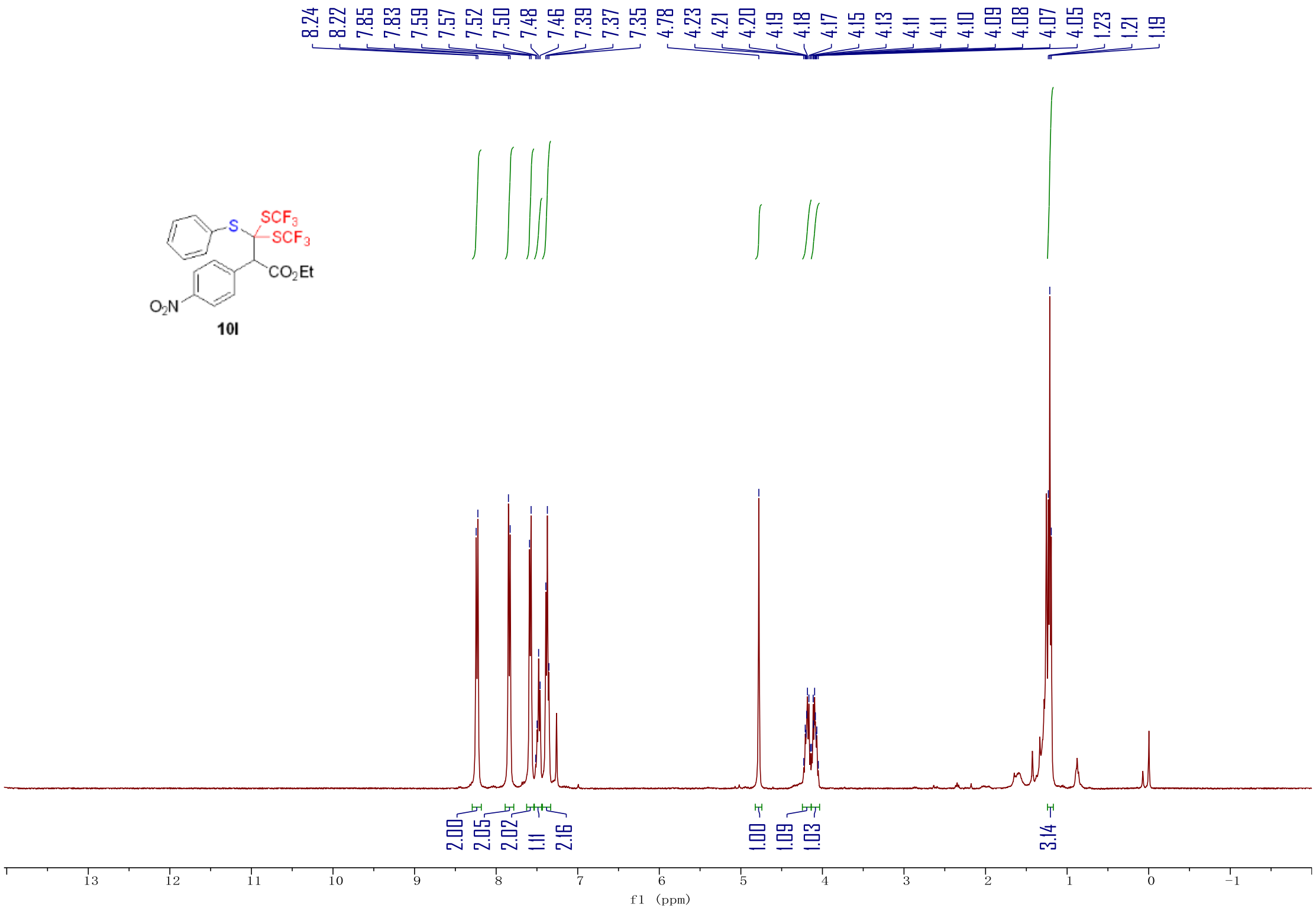

^{19}F NMR Spectrum of **10l** (376 MHz, $CDCl_3$)

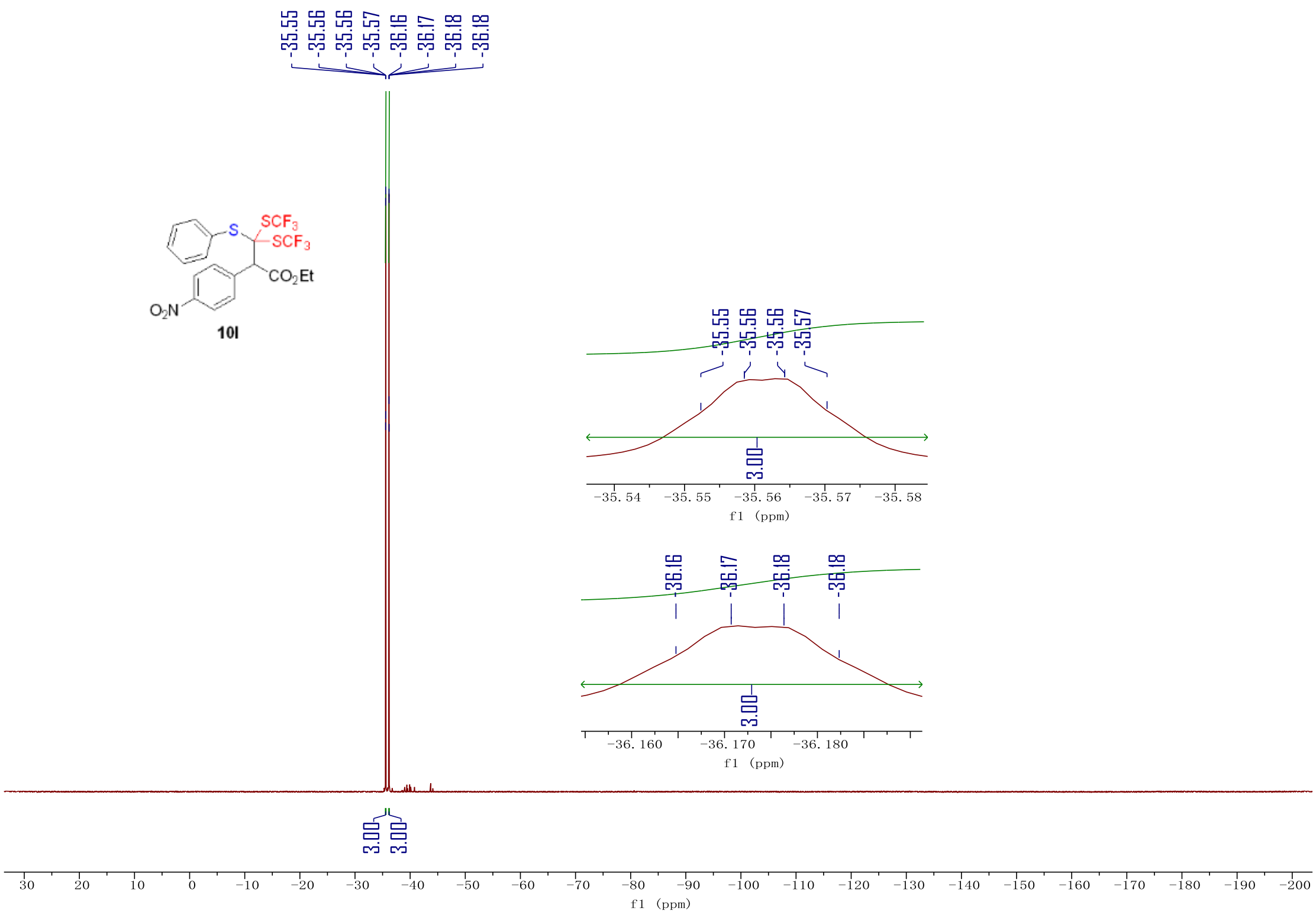

^{13}C NMR Spectrum of **10l** (101 MHz, $CDCl_3$)

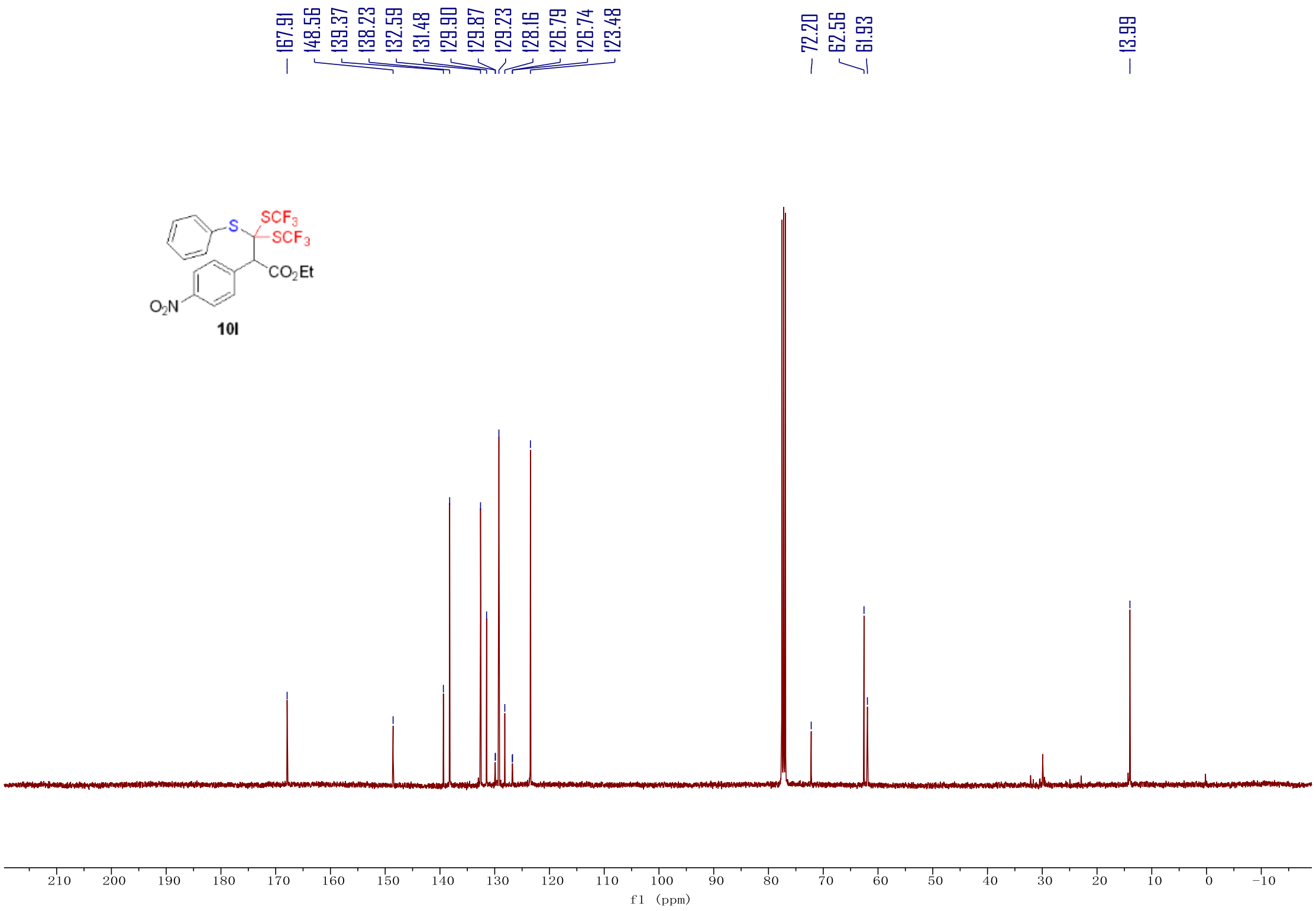

^{1}H NMR Spectrum of **10m** (400 MHz, $CDCl_3$)

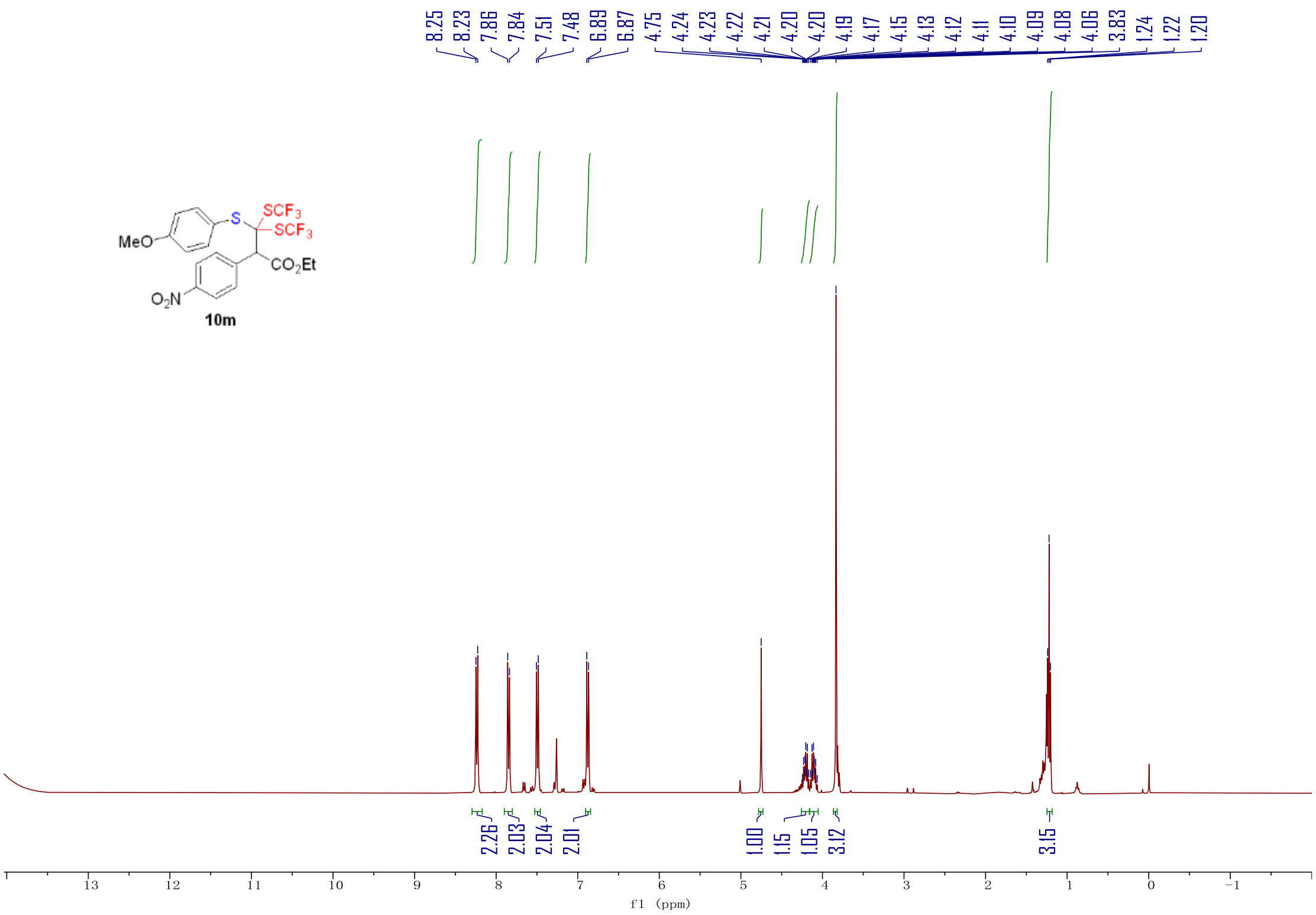

^{19}F NMR Spectrum of **10m** (376 MHz, $CDCl_3$)

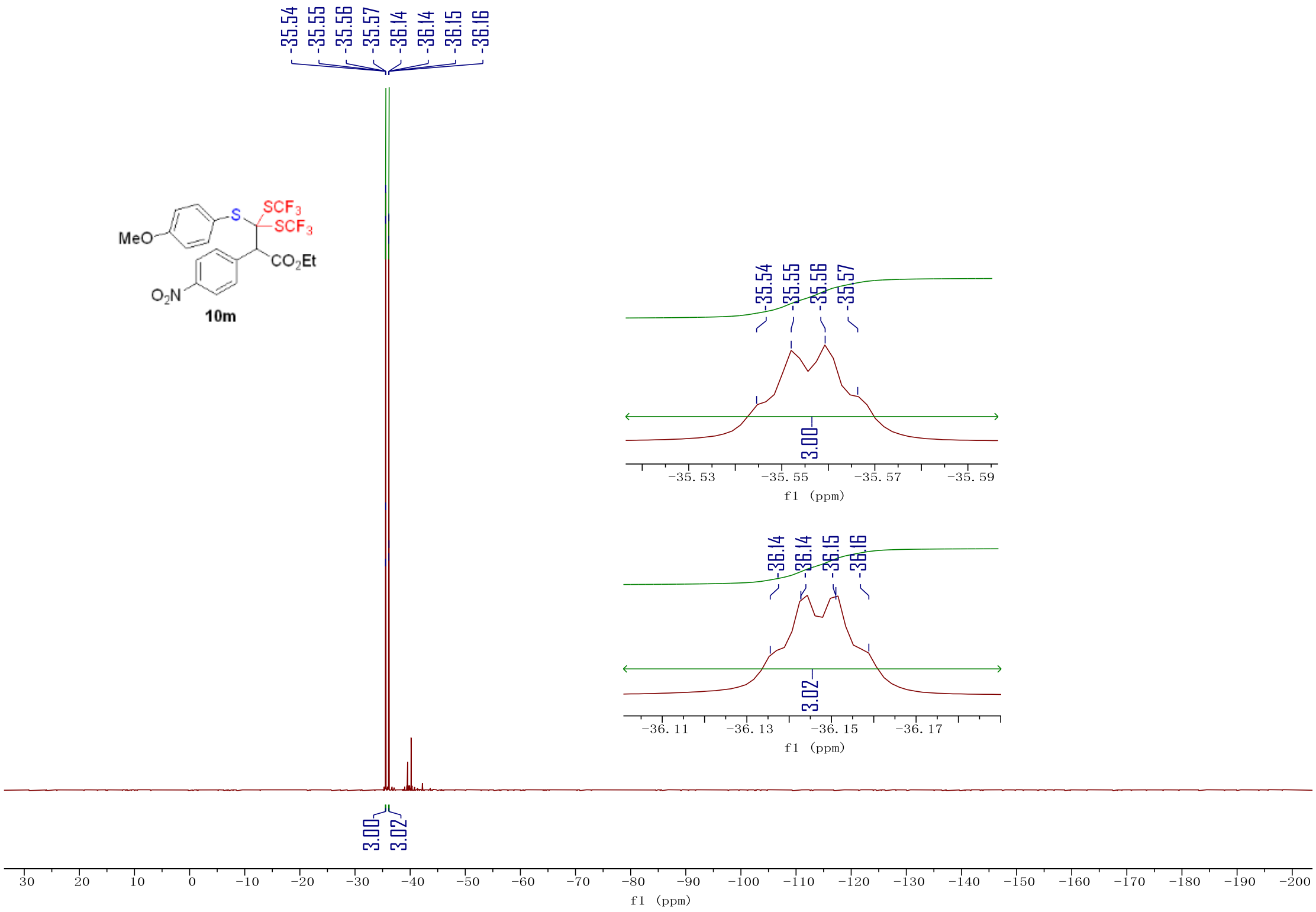

^{13}C NMR Spectrum of **10m** (101 MHz, $CDCl_3$)

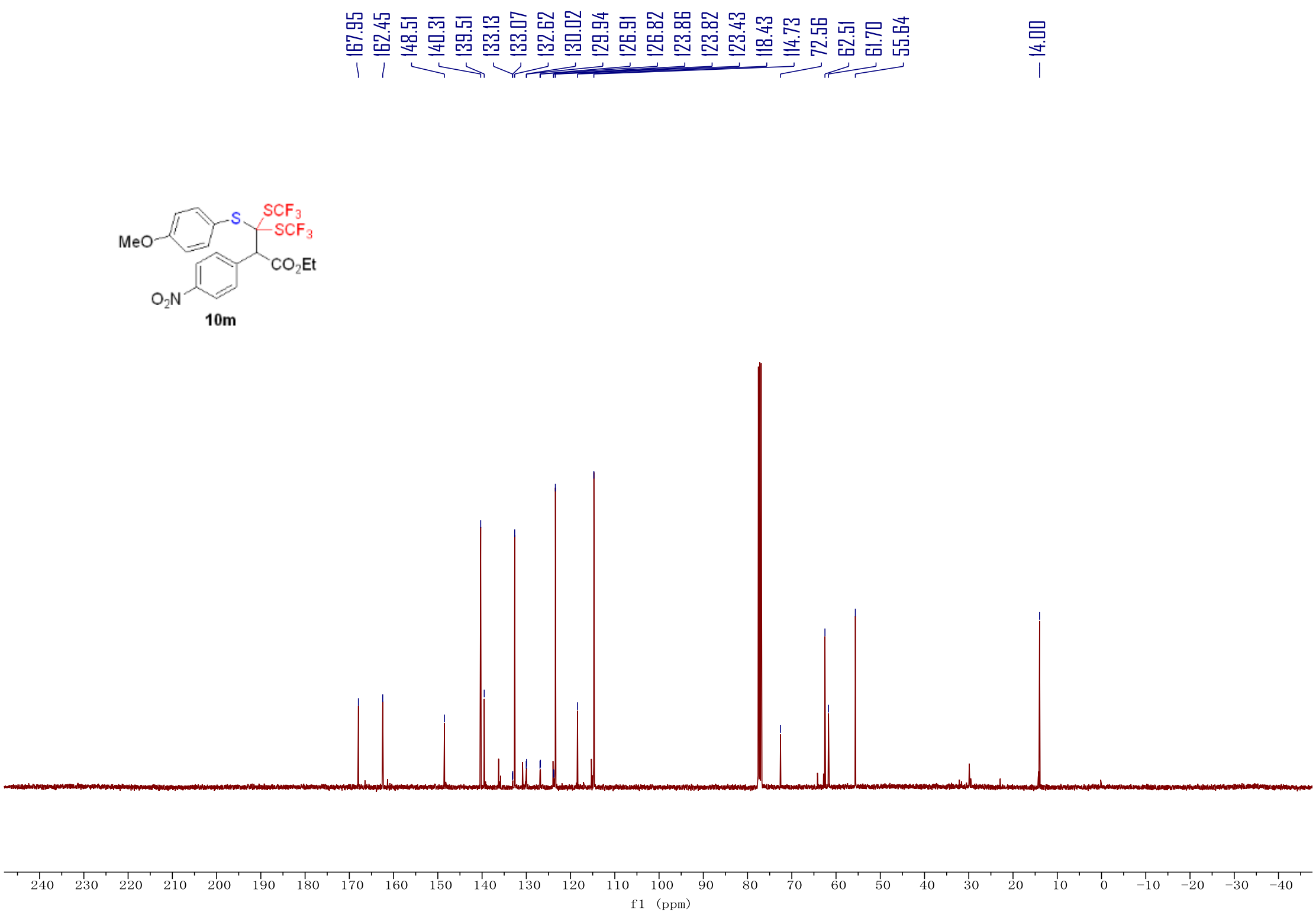

^{1}H NMR Spectrum of **10n** (400 MHz, $CDCl_3$)

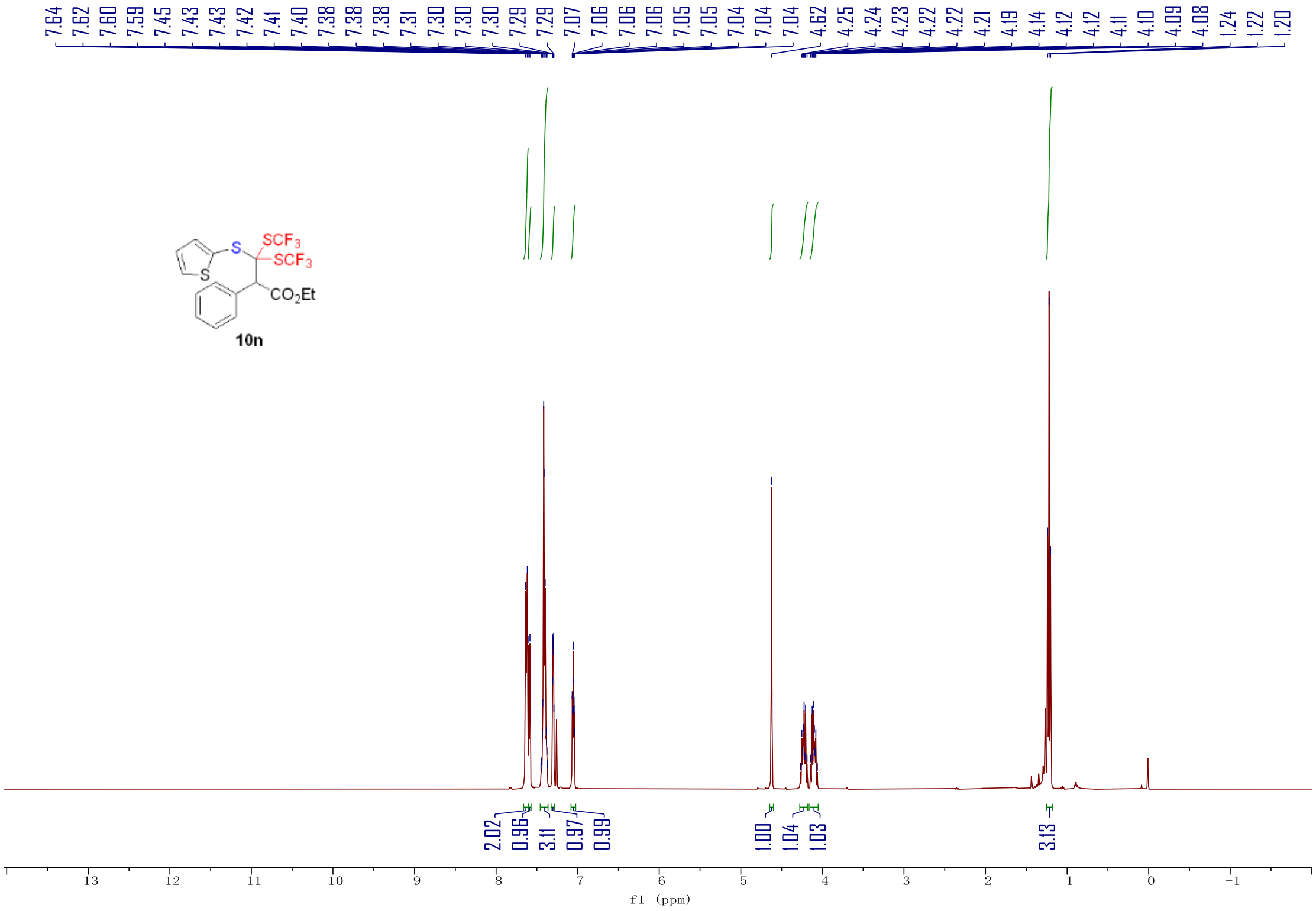

^{19}F NMR Spectrum of **10n** (376 MHz, $CDCl_3$)

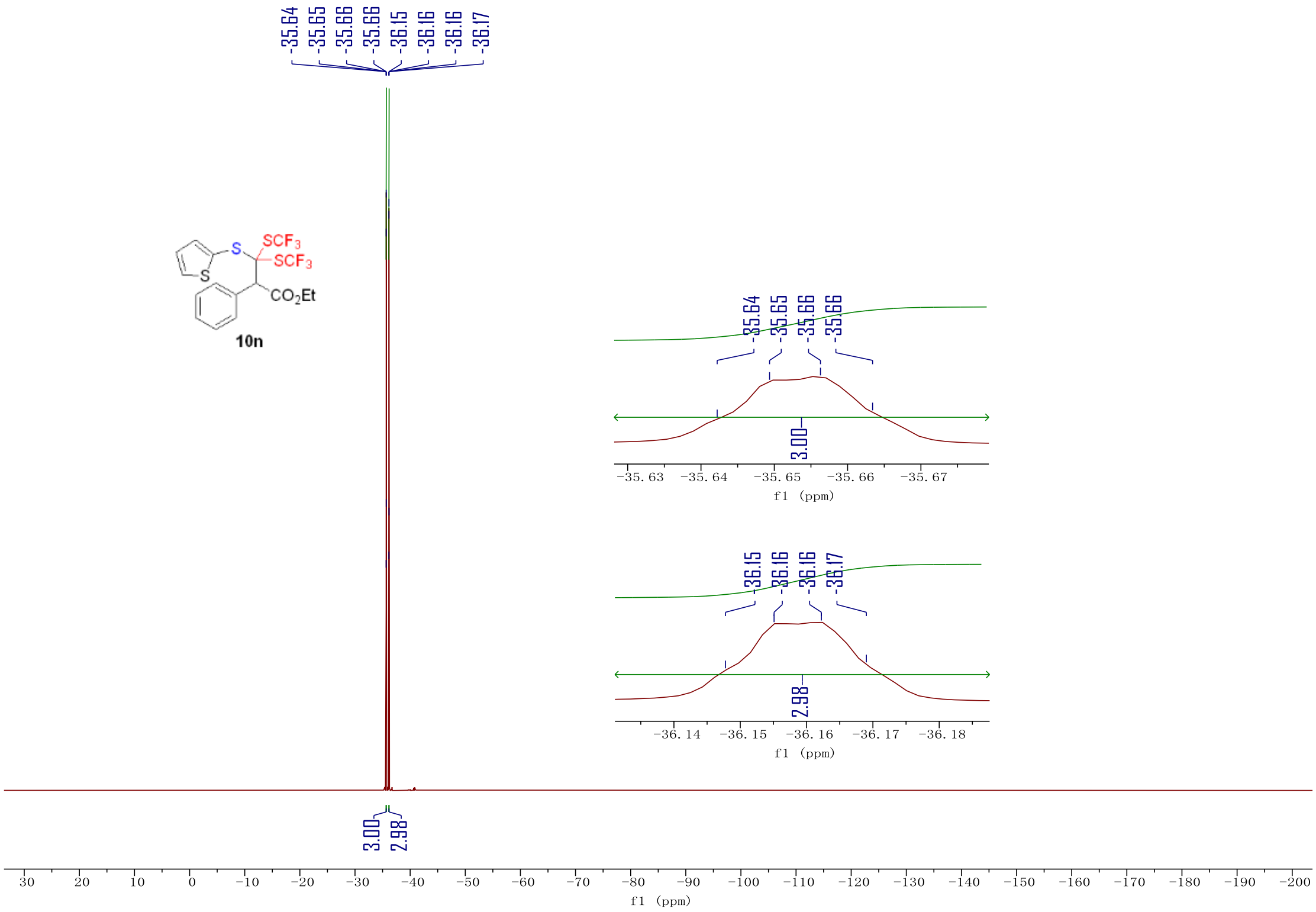

^{13}C NMR Spectrum of **10n** (101 MHz, $CDCl_3$)

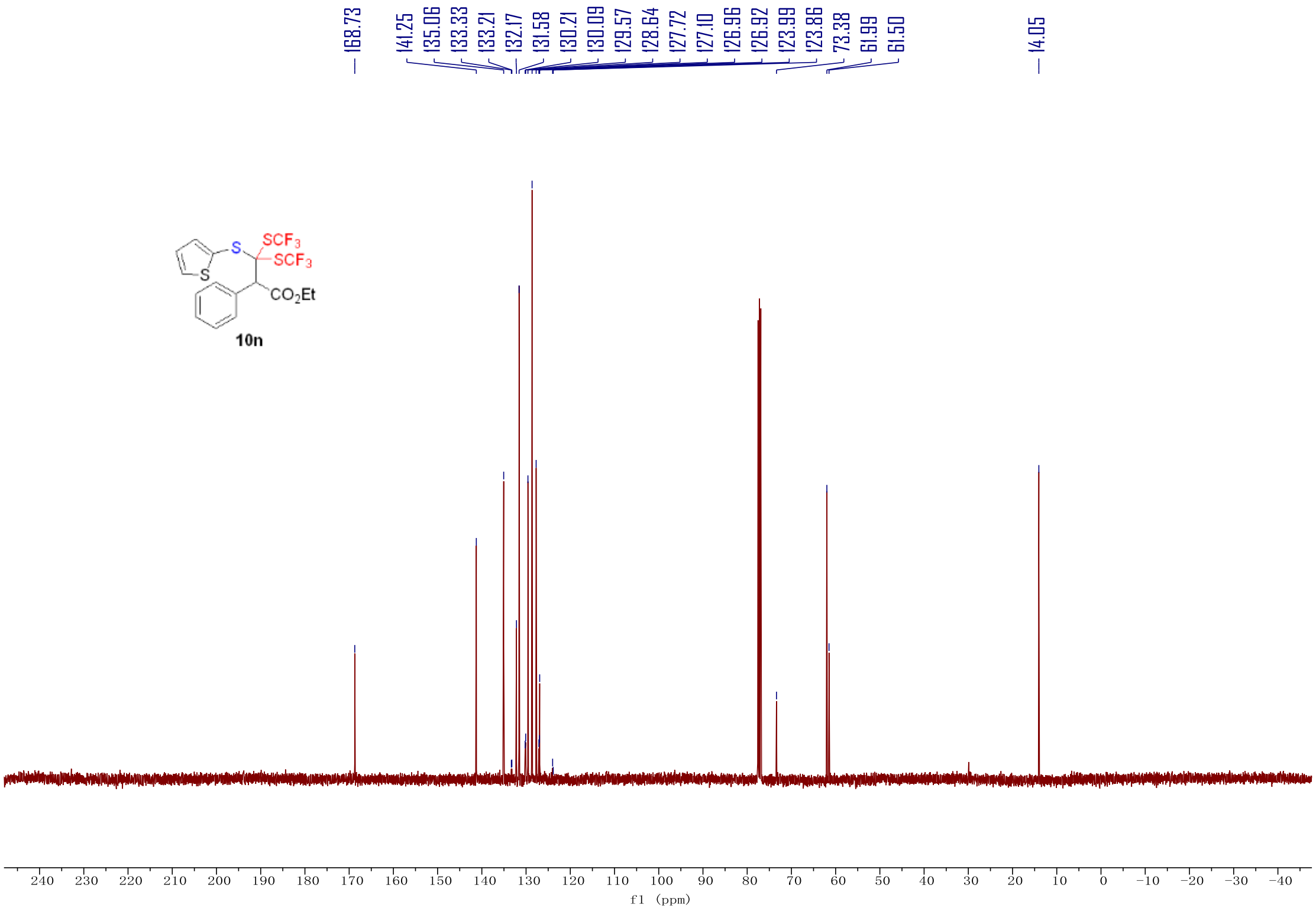

^{1}H NMR Spectrum of **10o** (400 MHz, $CDCl_3$)

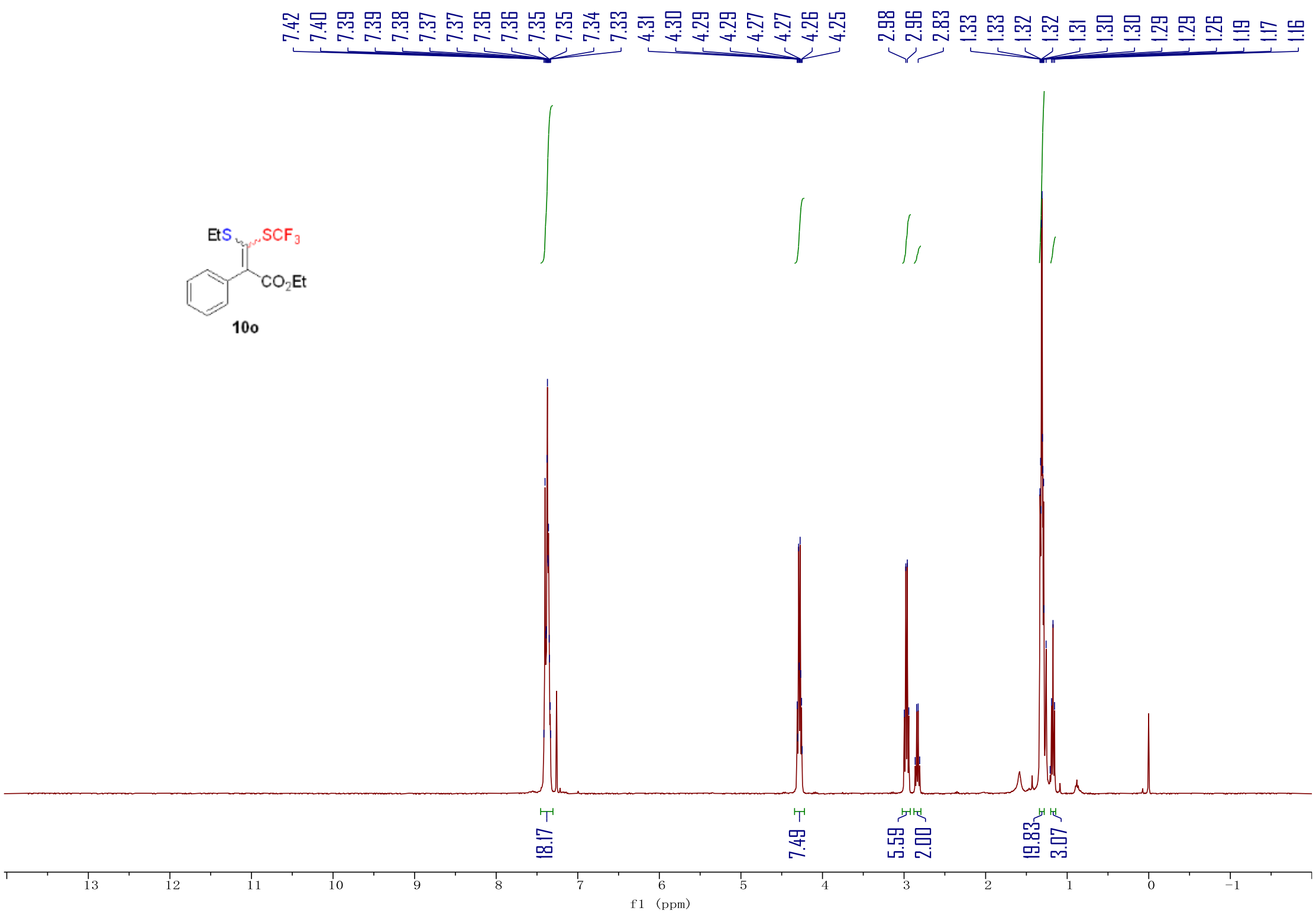

^{19}F NMR Spectrum of **10o** (376 MHz, $CDCl_3$)

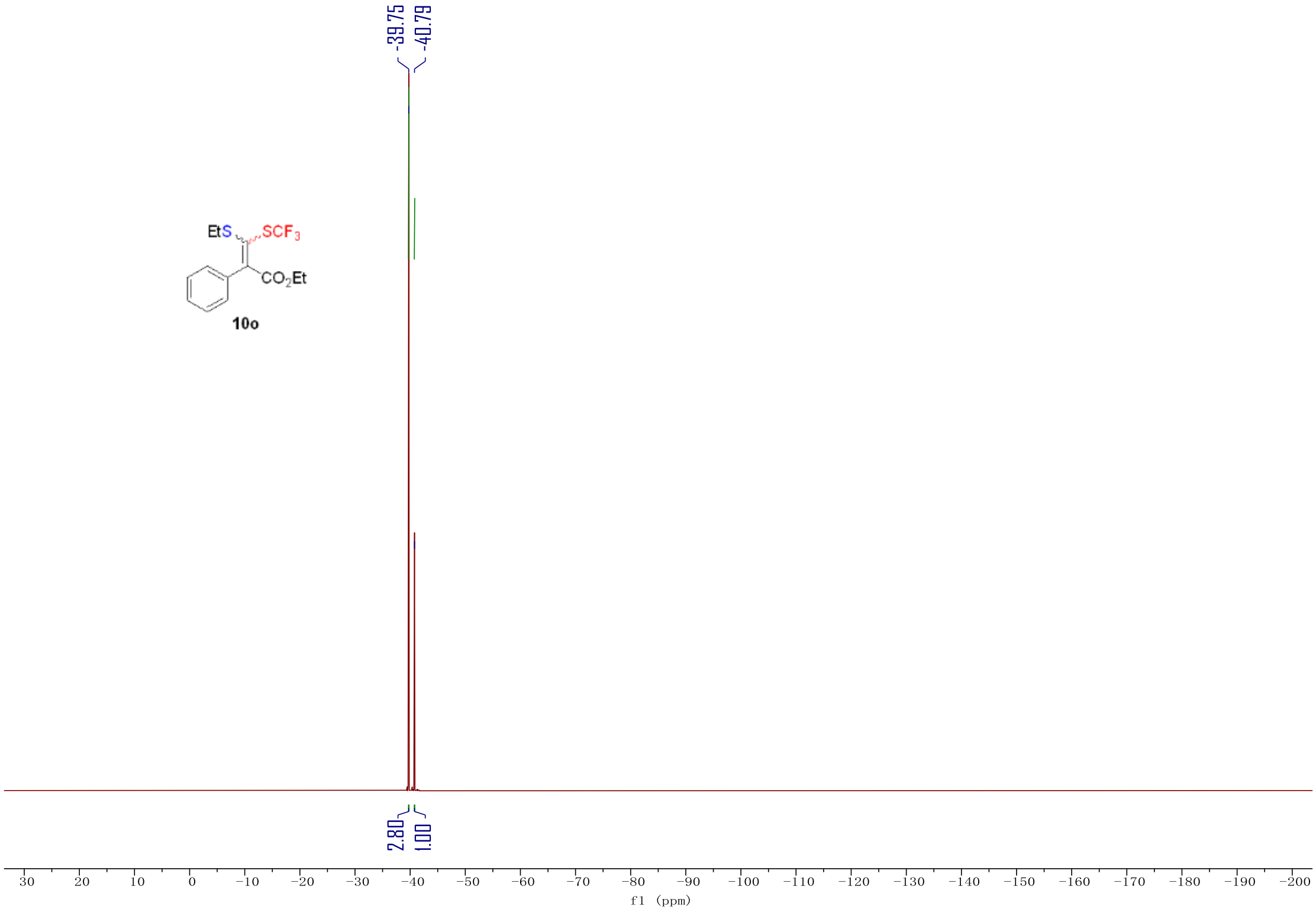

^{13}C NMR Spectrum of **10o** (101 MHz, $CDCl_3$)

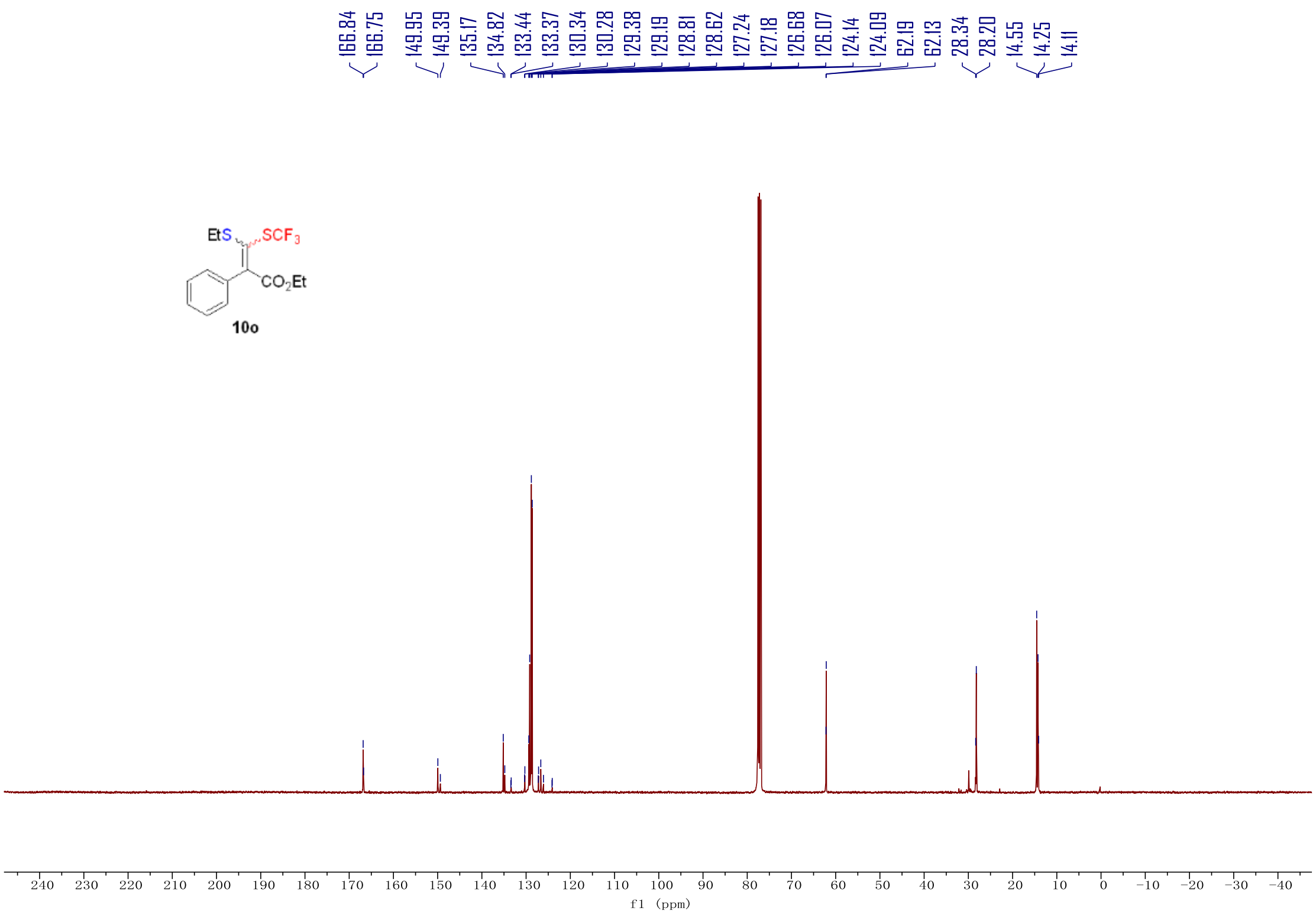

^{1}H NMR Spectrum of **10p** (400 MHz, $CDCl_3$)

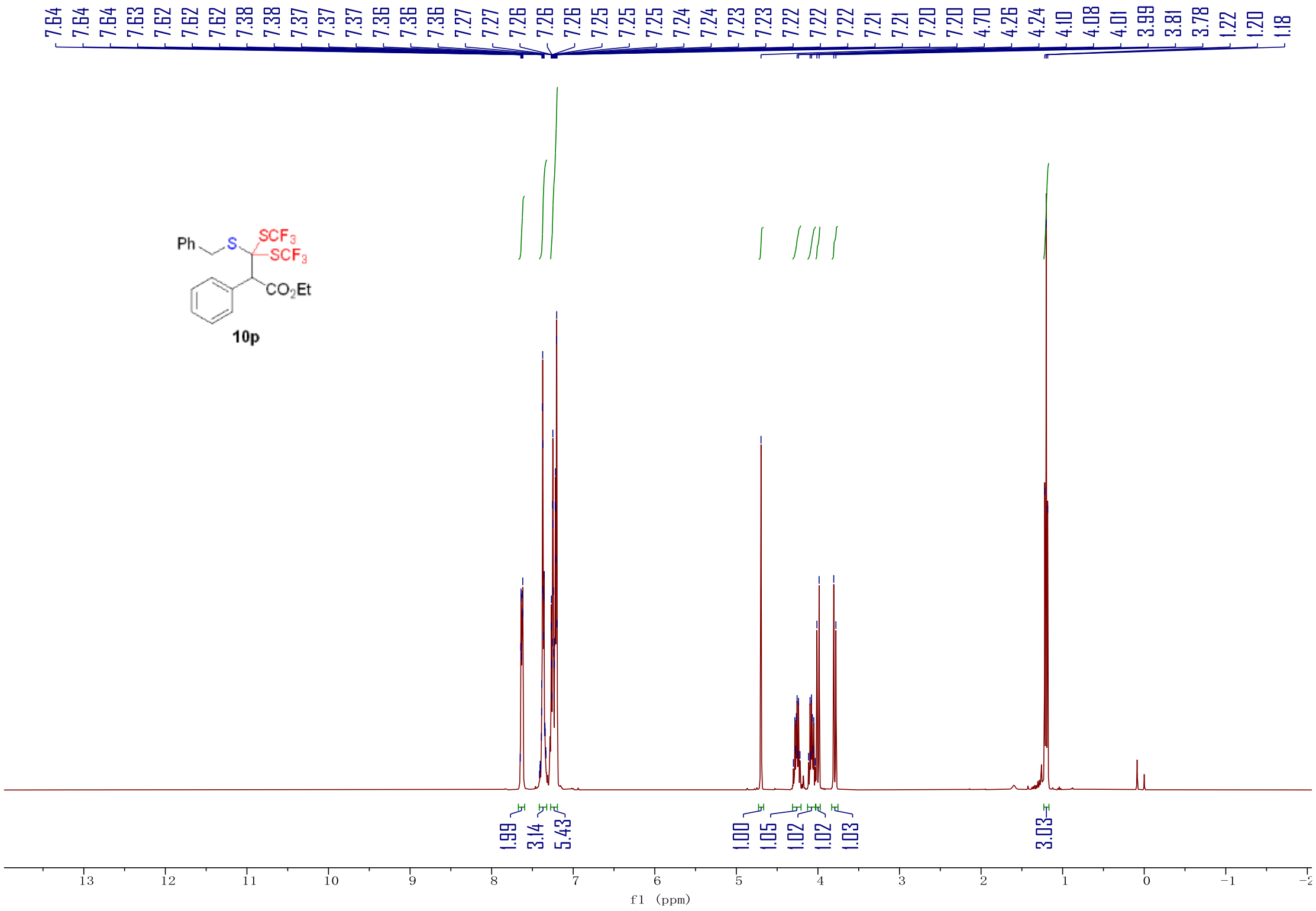

^{19}F NMR Spectrum of **10p** (376 MHz, $CDCl_3$)

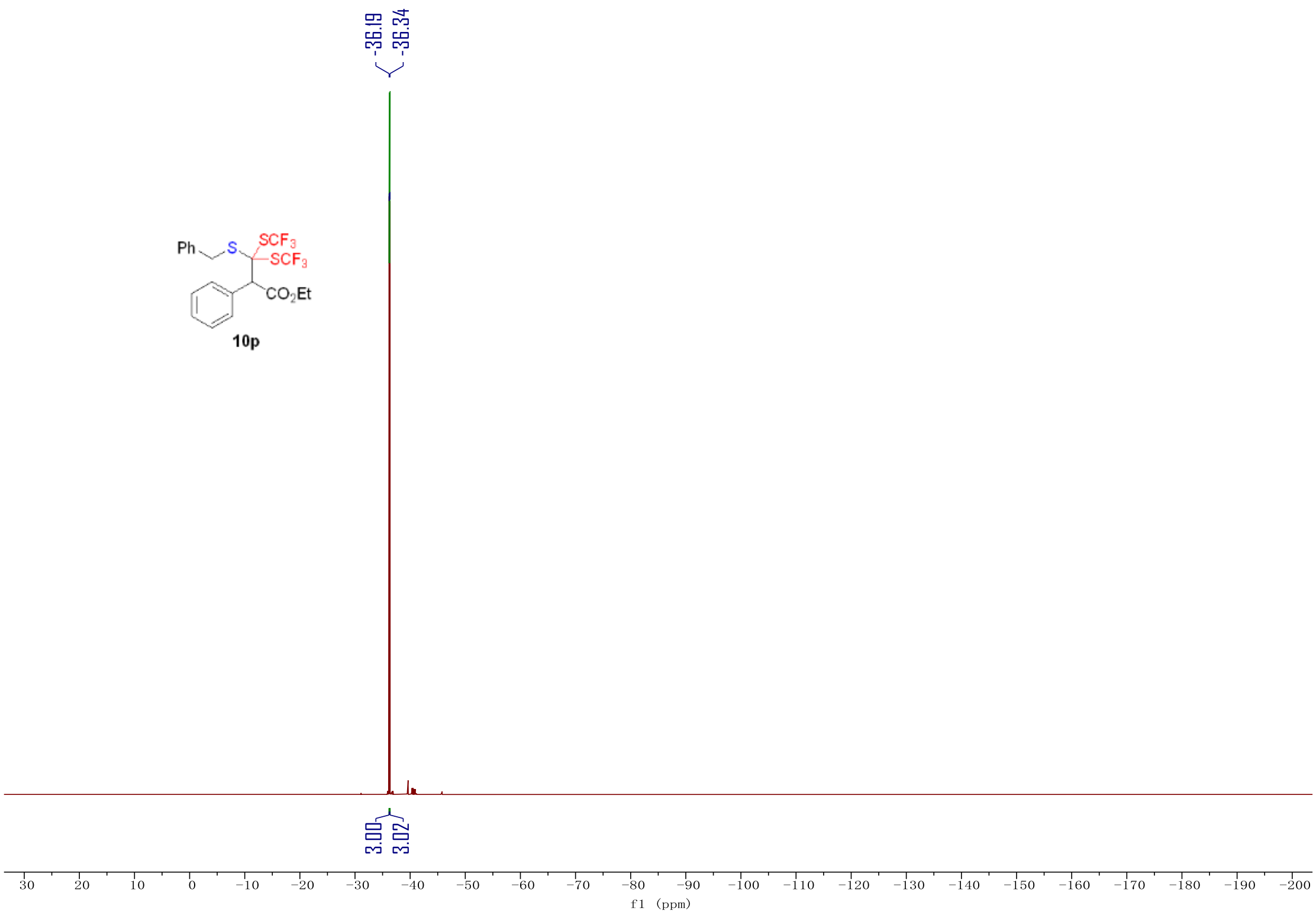

^{13}**C NMR** Spectrum of **10p** (101 MHz, $CDCl_3$)

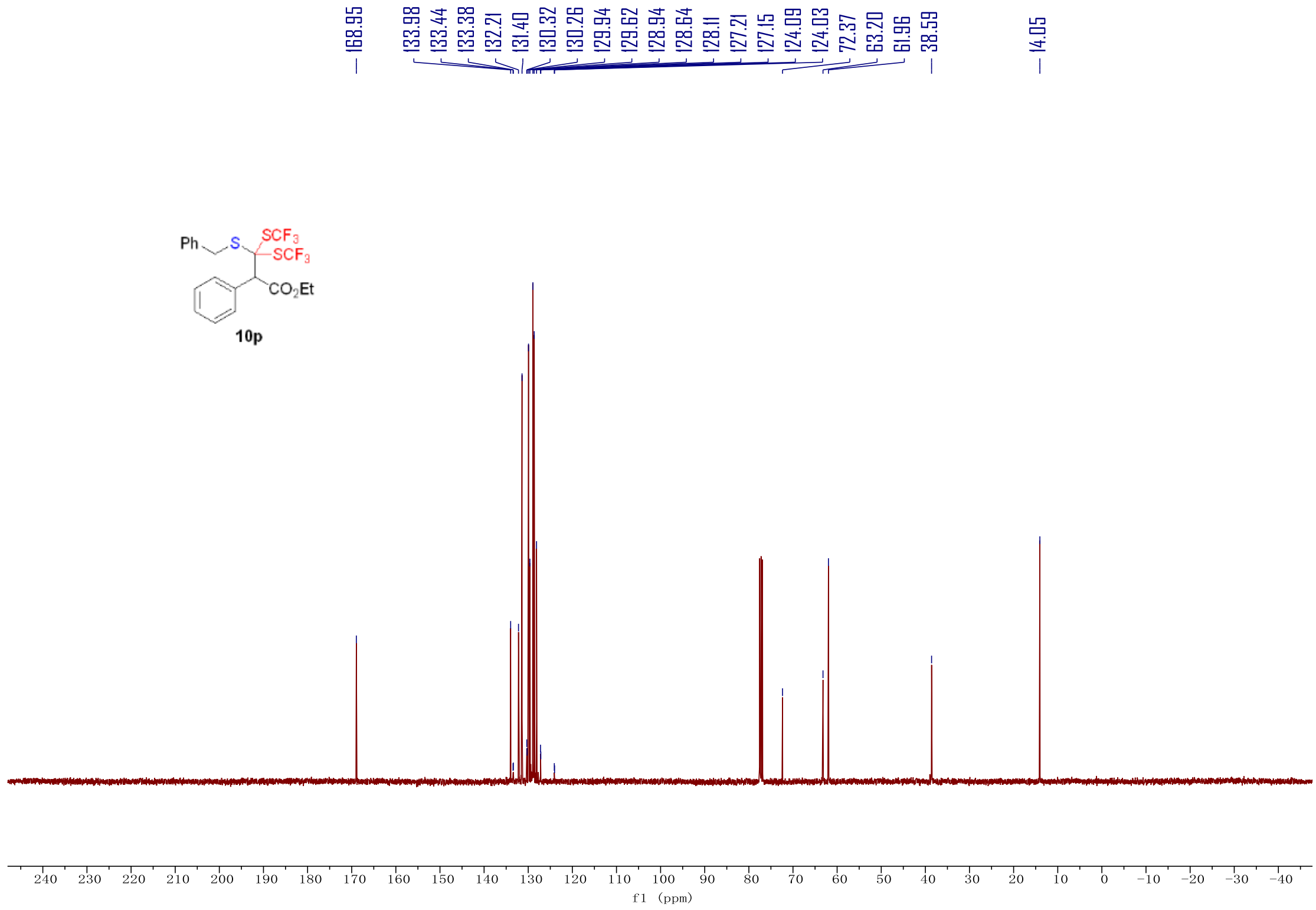

^{1}**H NMR** Spectrum of **10q** (400 MHz, $CDCl_3$)

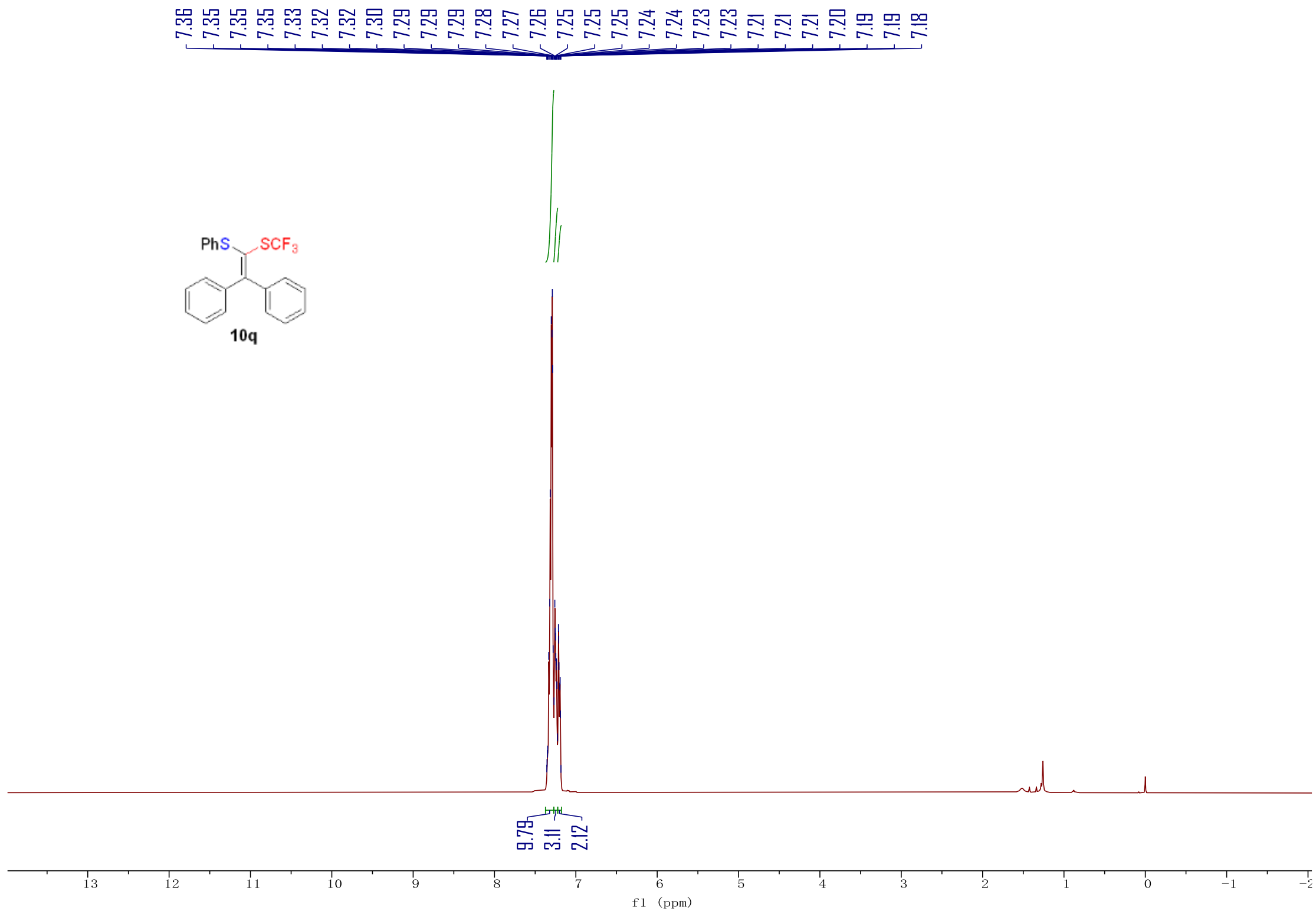

^{19}F NMR Spectrum of **10q** (376 MHz, $CDCl_3$)

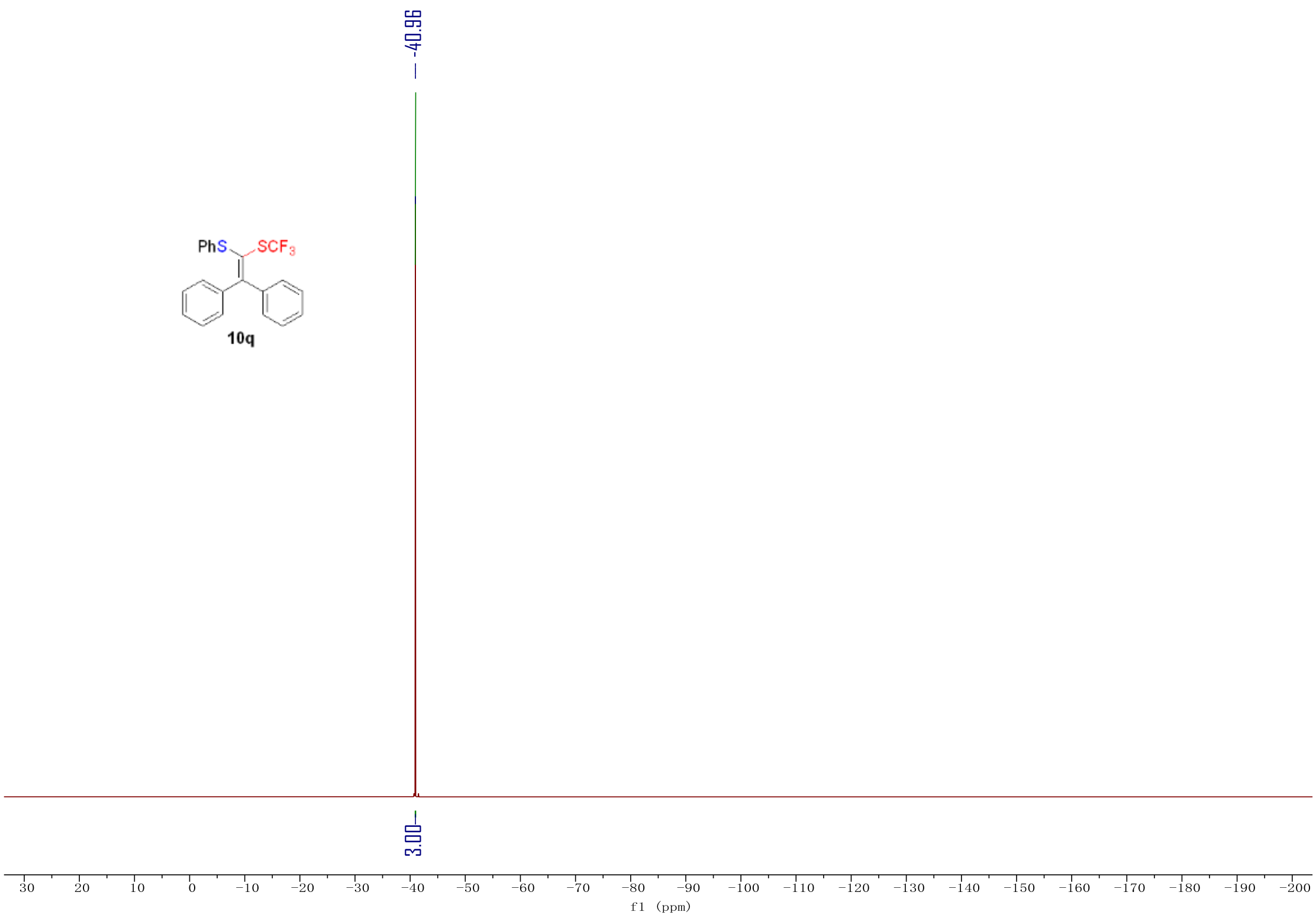

^{13}C NMR Spectrum of **10q** (101 MHz, $CDCl_3$)

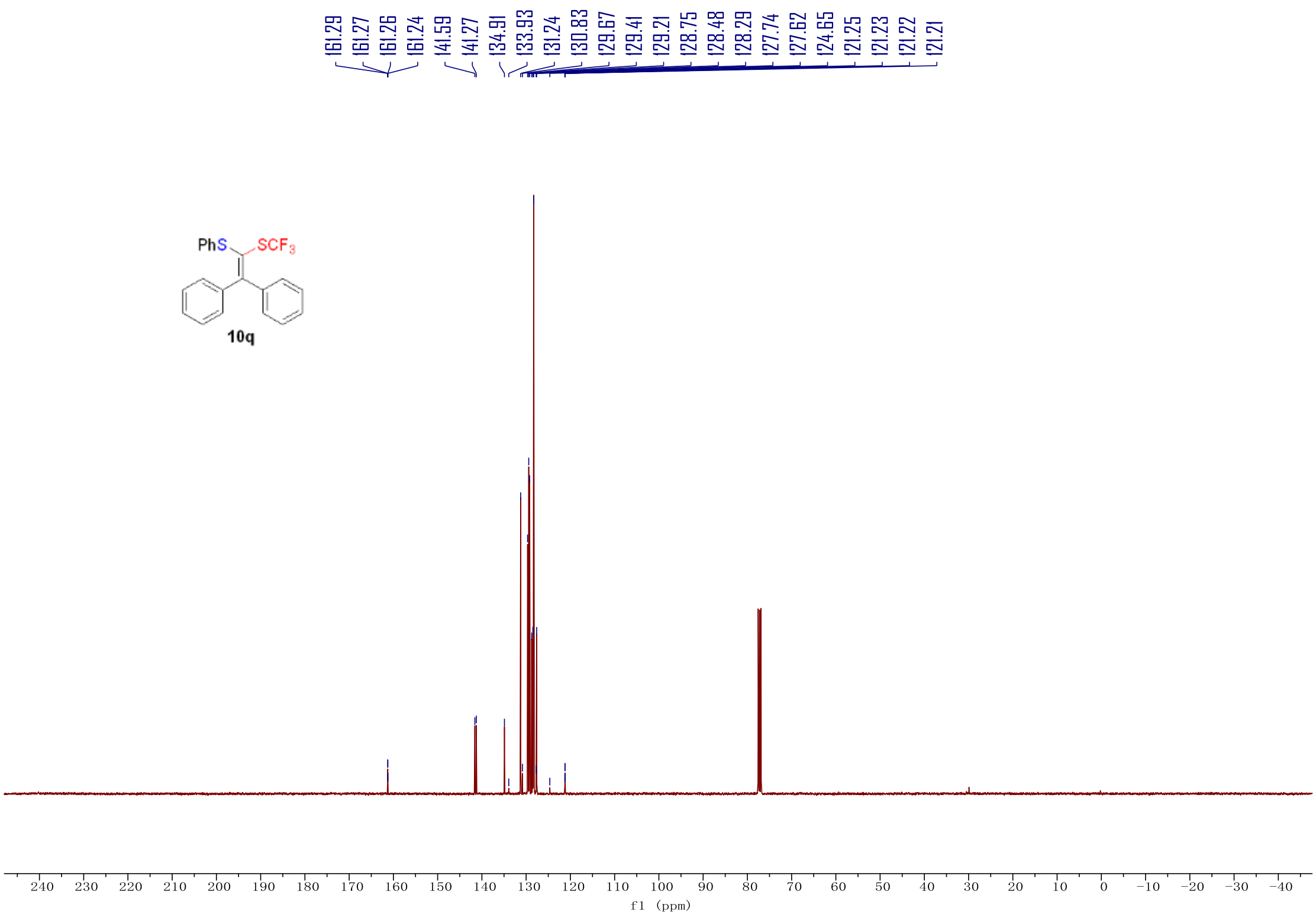

^{1}H NMR Spectrum of the mixture of **10c** and **10j** from the scrambling reaction of **9a** and **9c** (400 MHz, $CDCl_3$)

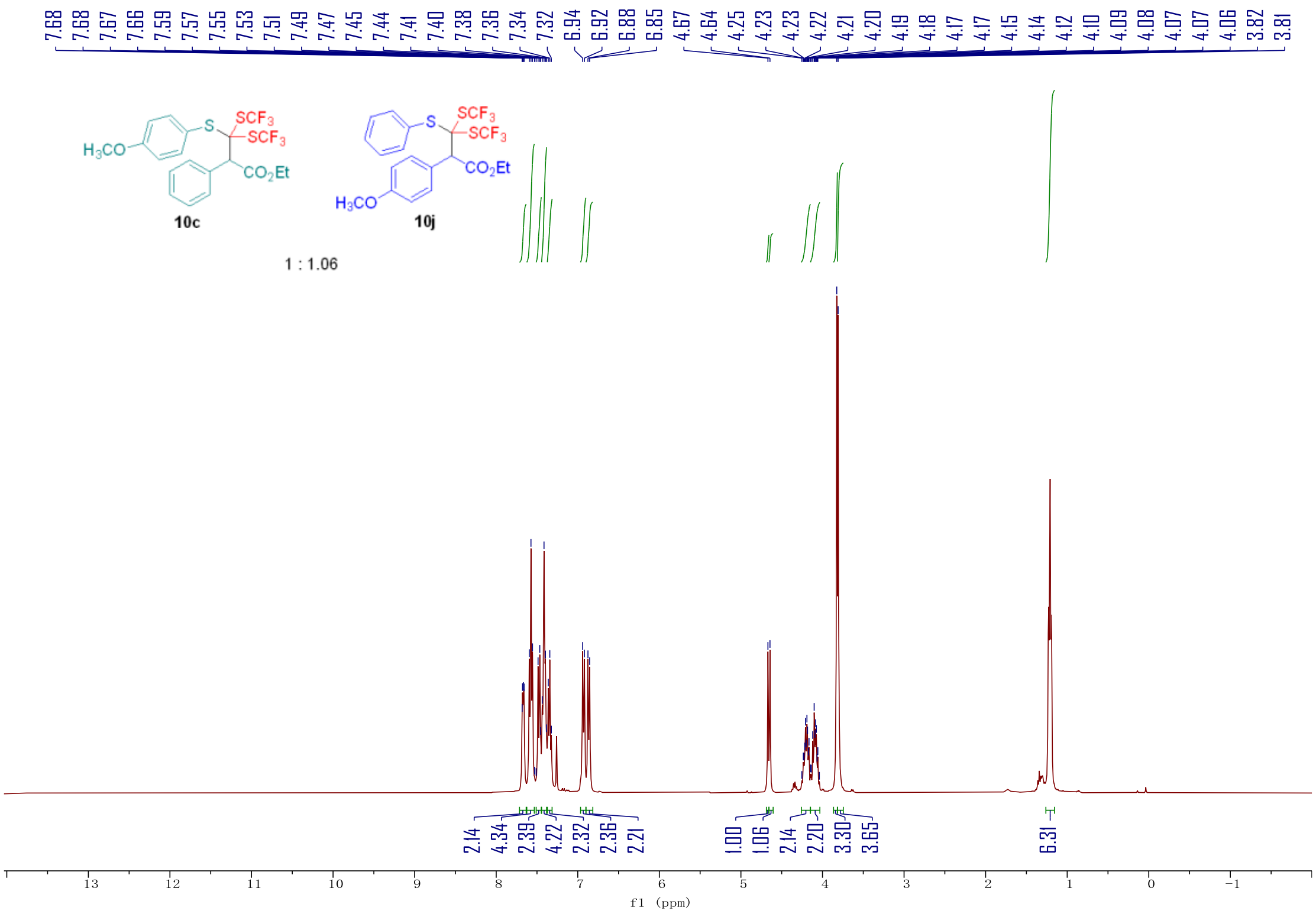

^{19}F NMR Spectrum of the mixture of **10c** and **10j** from the scrambling reaction of **9a** and **9c** (376 MHz, $CDCl_3$)

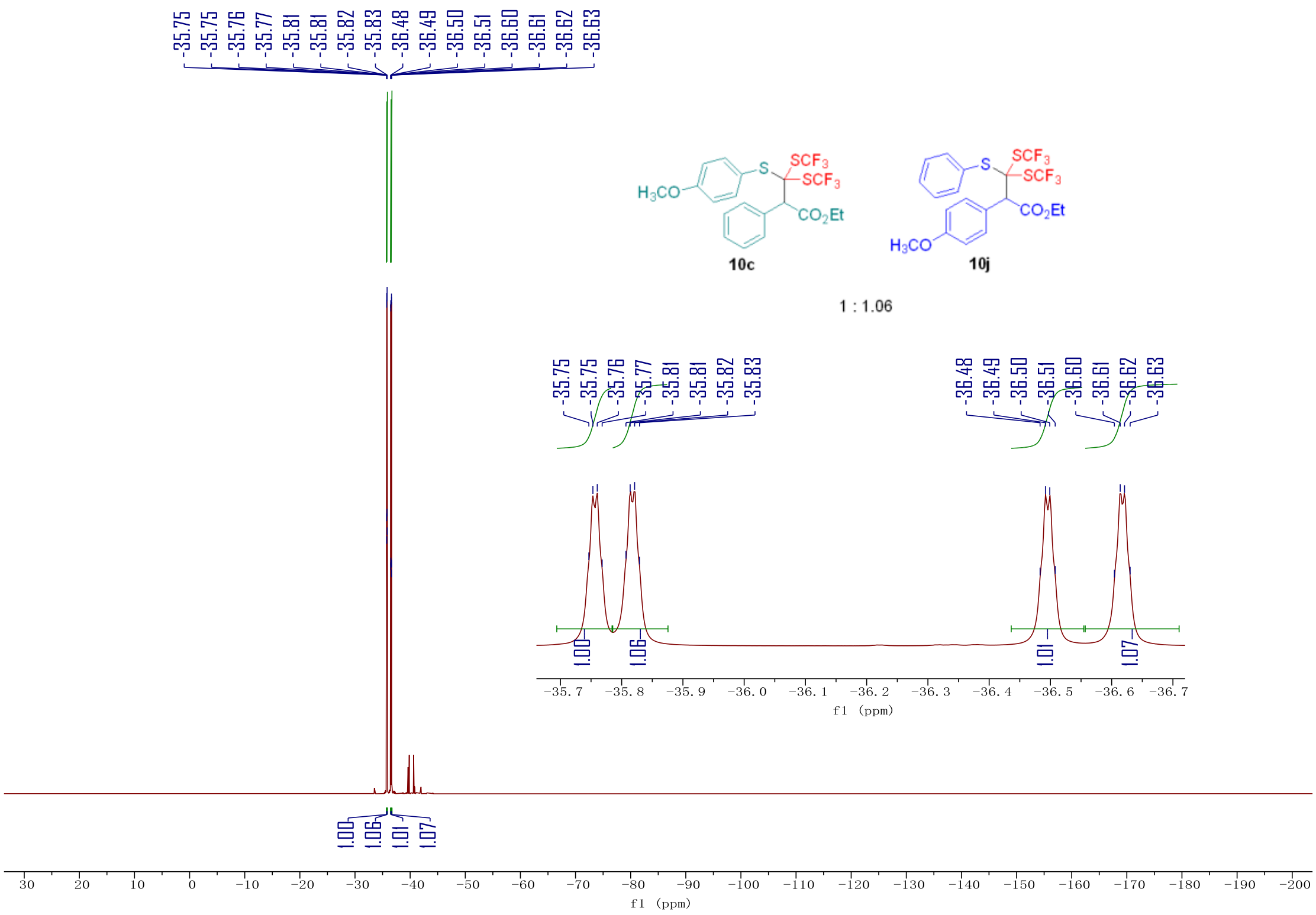

^{1}**H NMR** Spectrum of **10s** (400 MHz, $CDCl_3$)

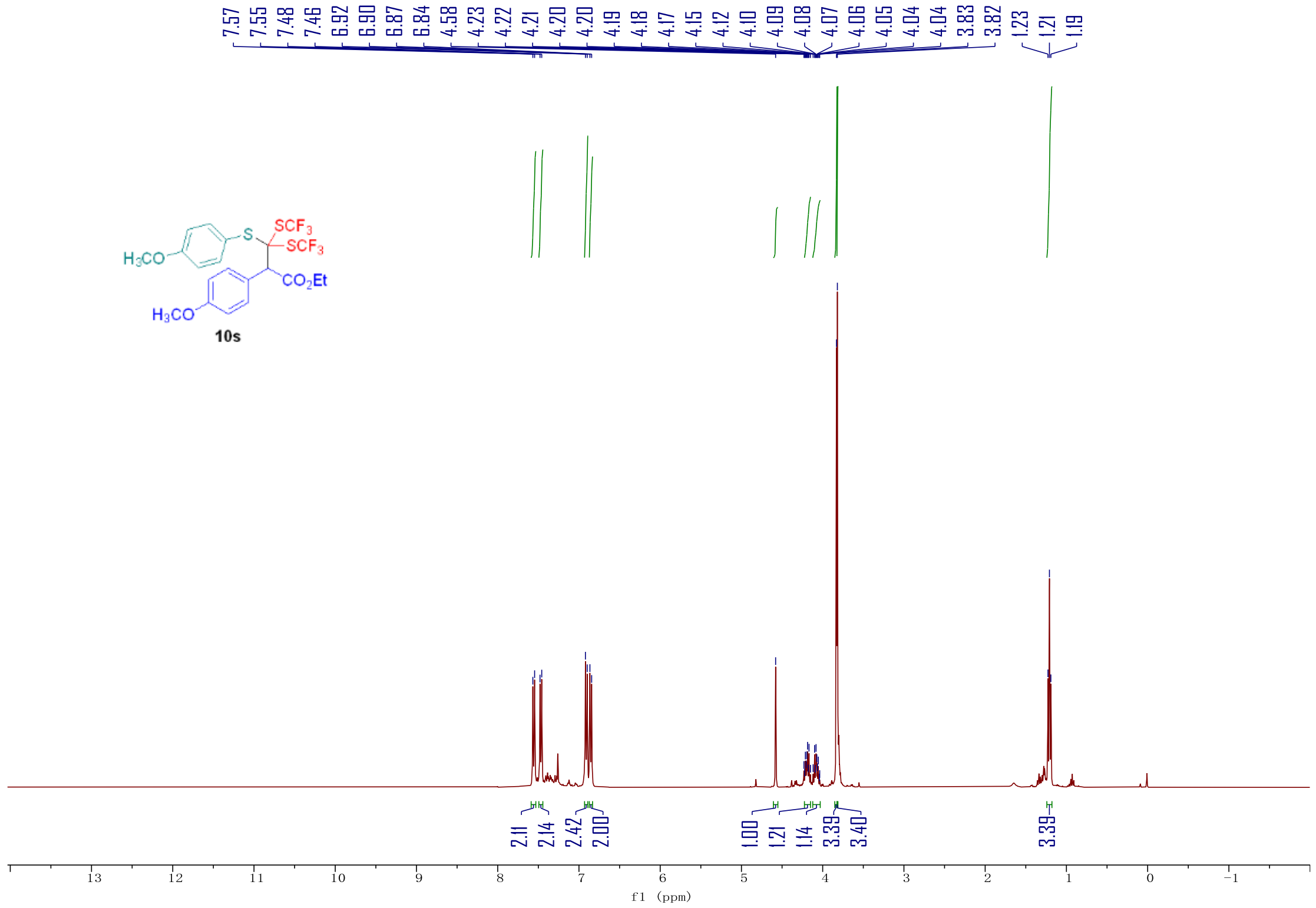

^{19}**F NMR** Spectrum of **10s** (376 MHz, $CDCl_3$)

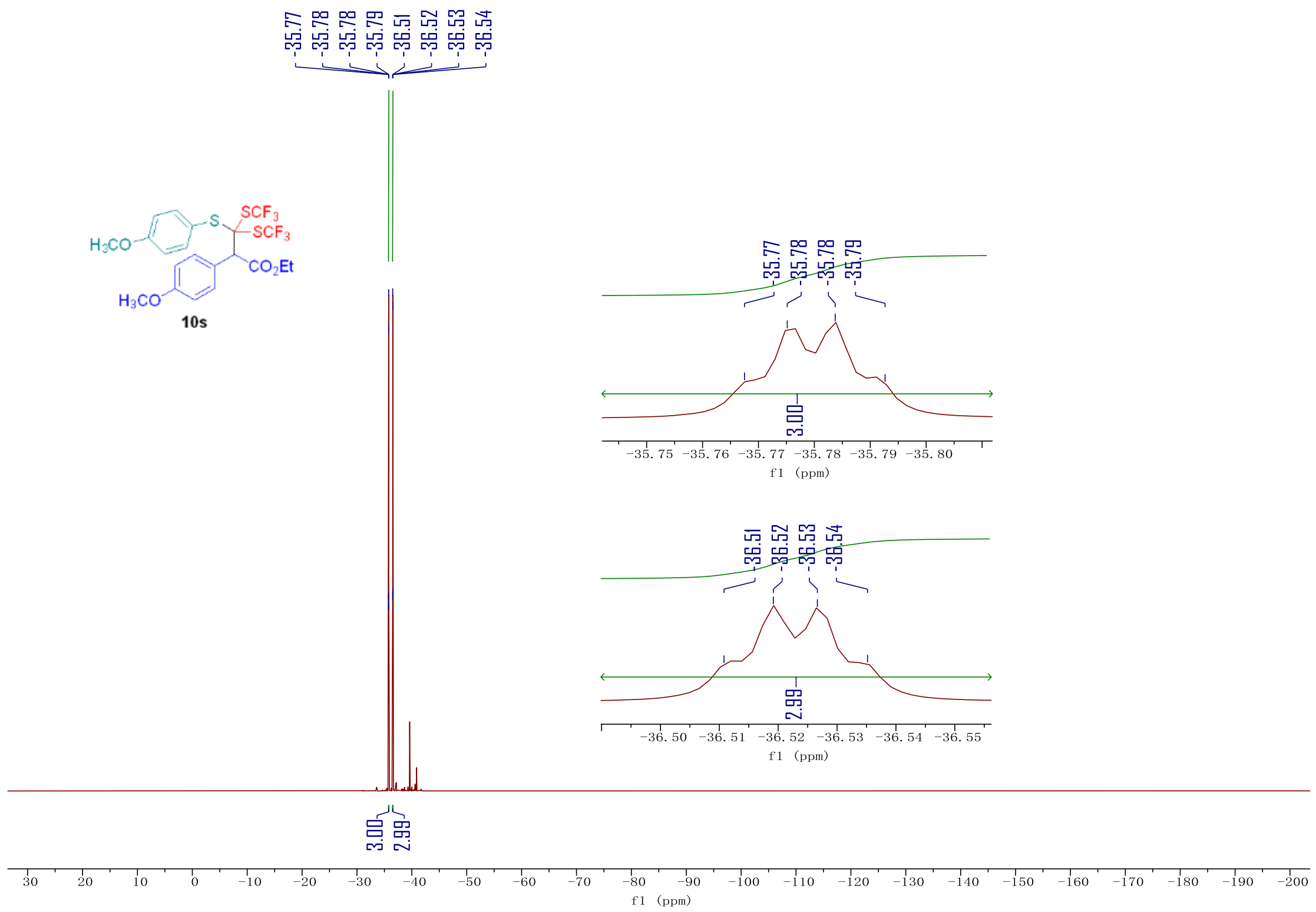

^{13}C NMR Spectrum of **10s** (101 MHz, $CDCl_3$)

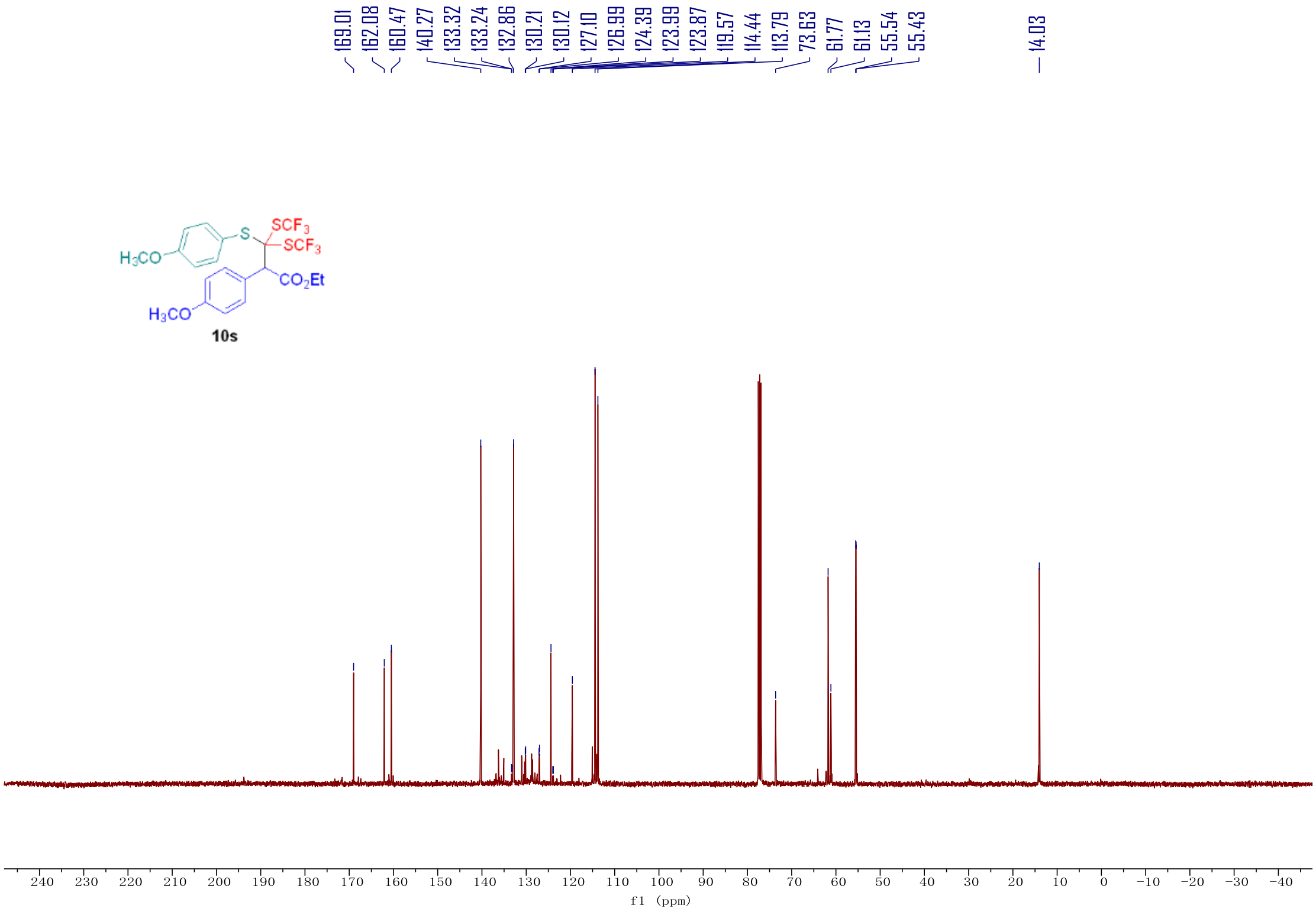

^{1}H NMR Spectrum of **12a** (400 MHz, $CDCl_3$)

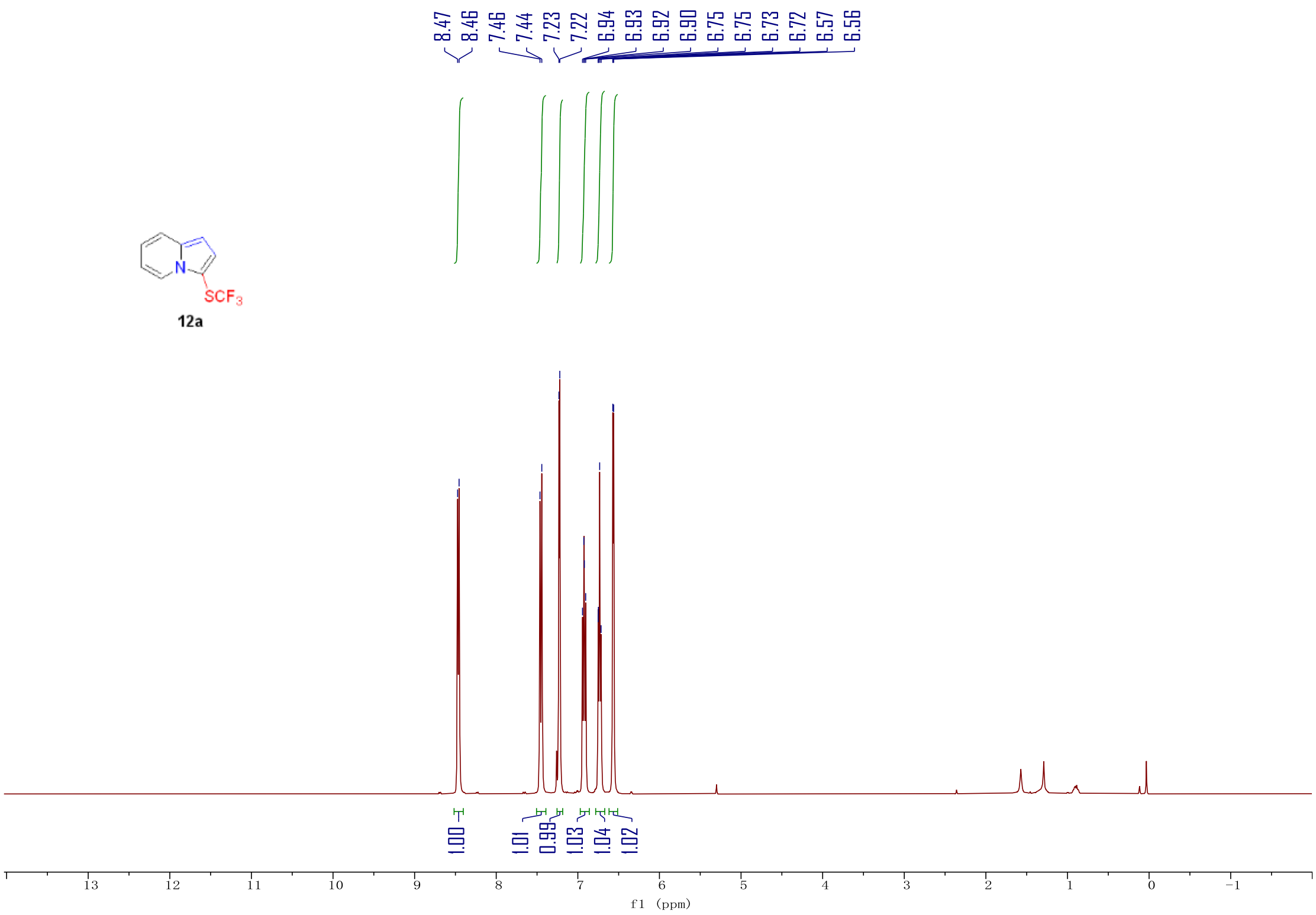

^{19}F NMR Spectrum of **12a** (376 MHz, $CDCl_3$)

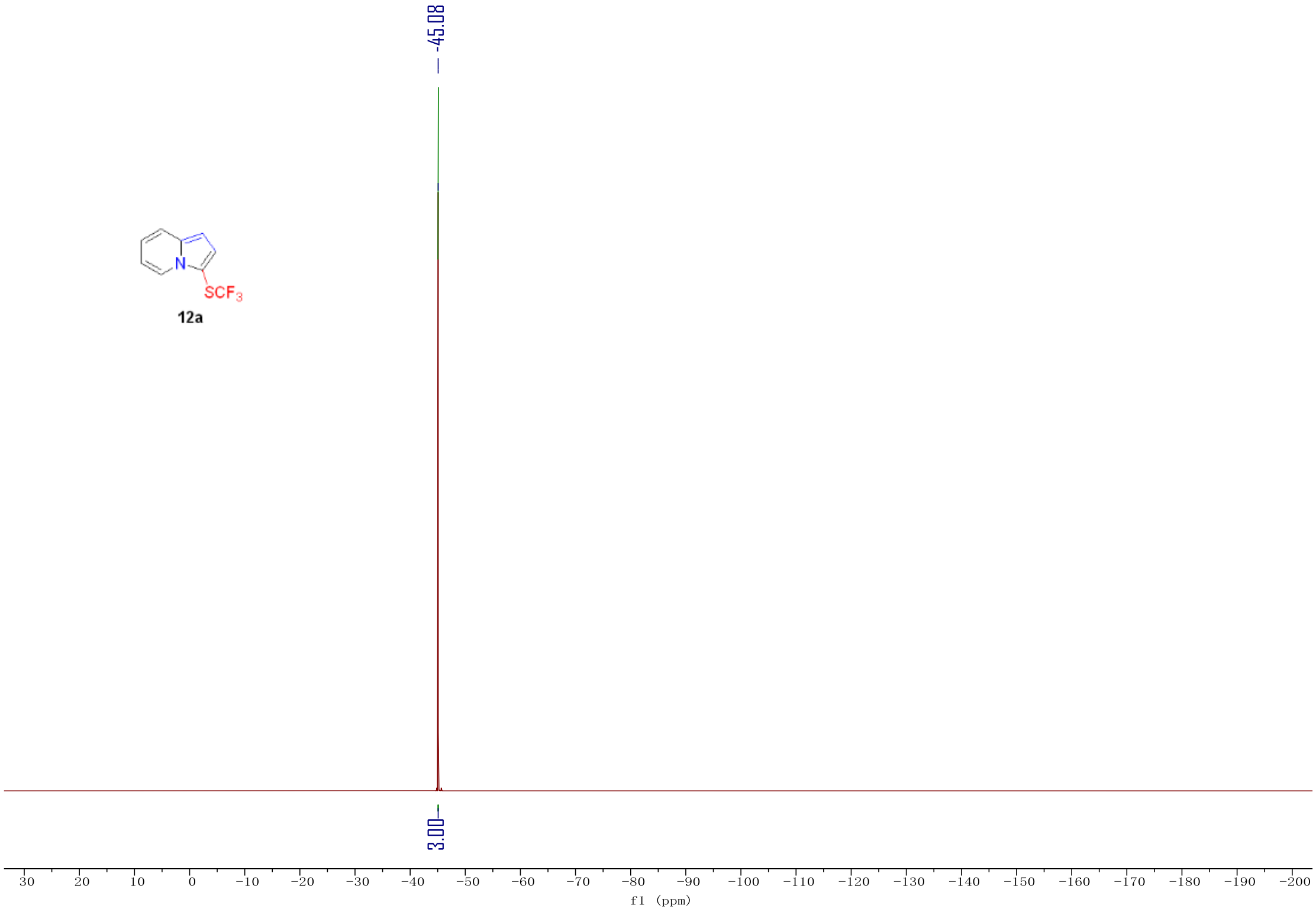

^{13}C NMR Spectrum of **12a** (101 MHz, $CDCl_3$)

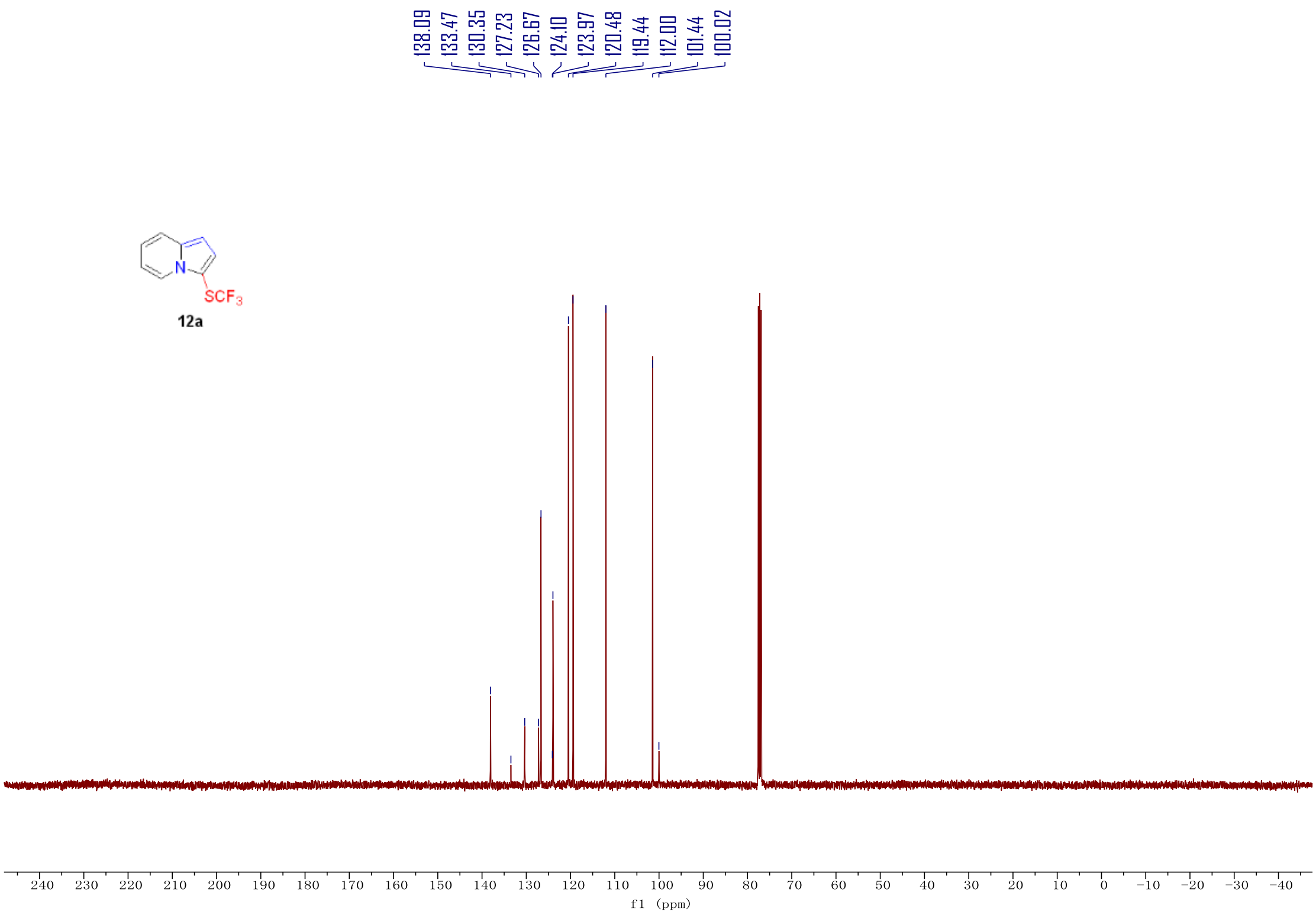

^{1}**H NMR** Spectrum of **12b** (400 MHz, $CDCl_3$)

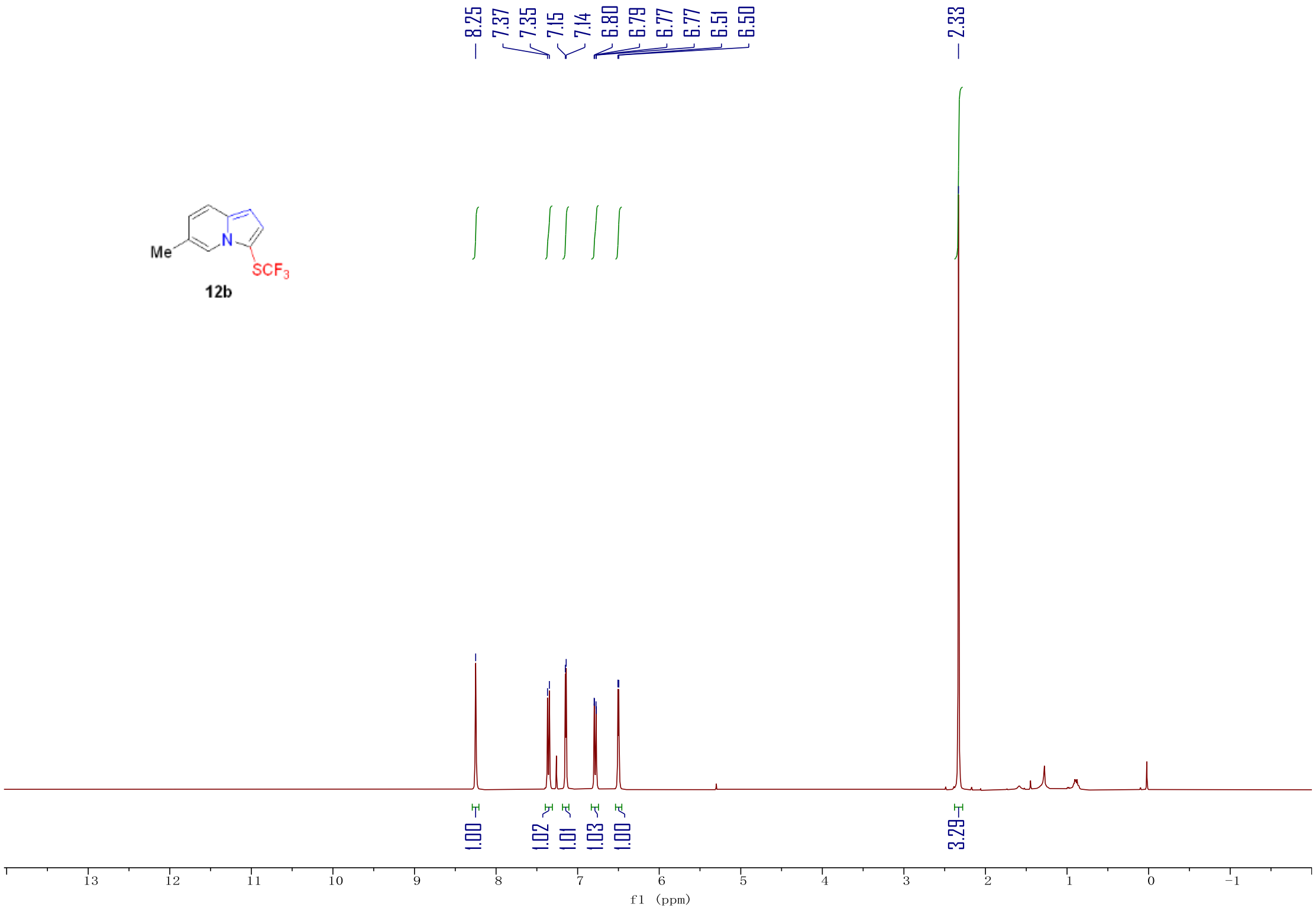

^{19}**F NMR** Spectrum of **12b** (376 MHz, $CDCl_3$)

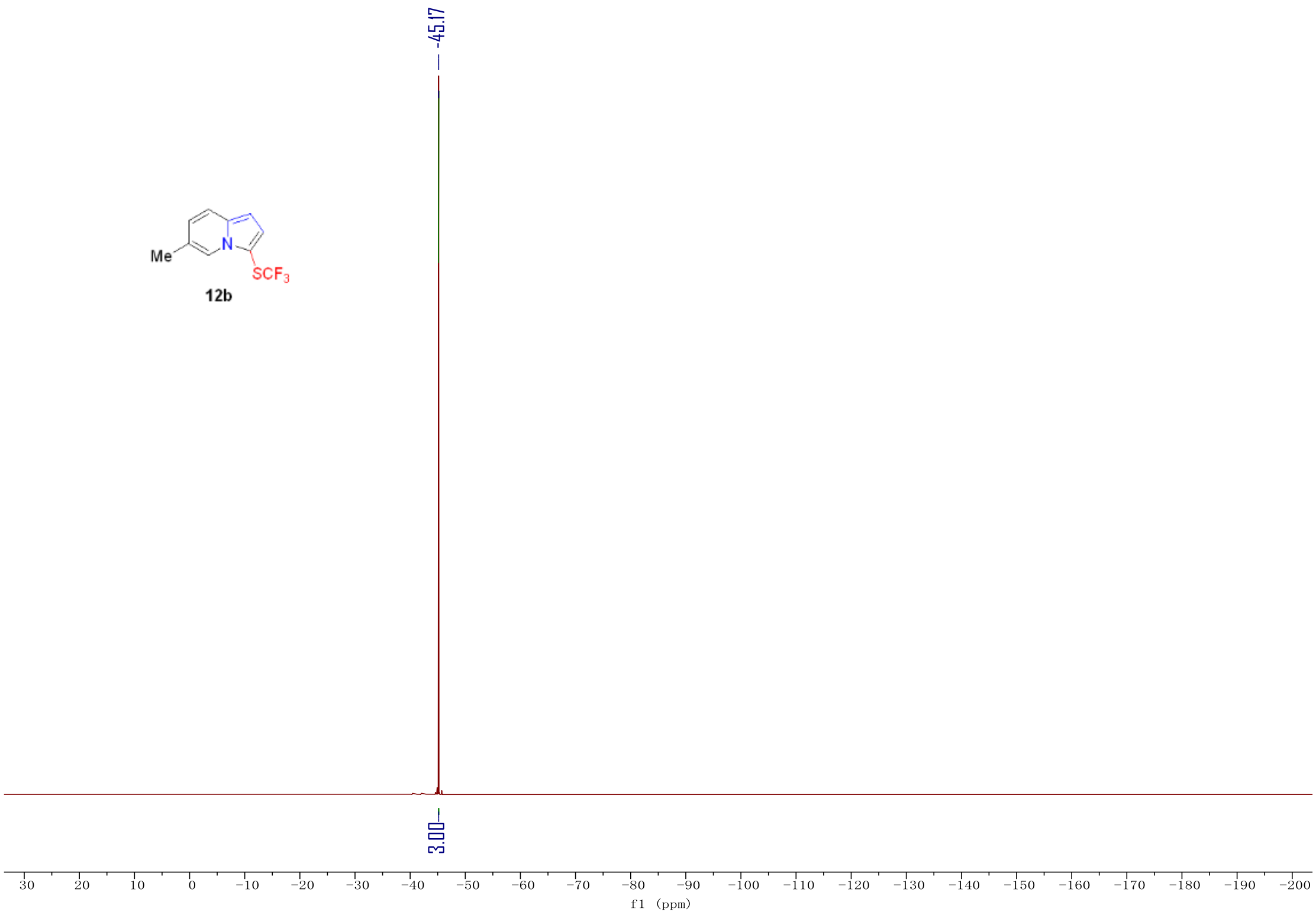

^{13}C NMR Spectrum of **12b** (101 MHz, $CDCl_3$)

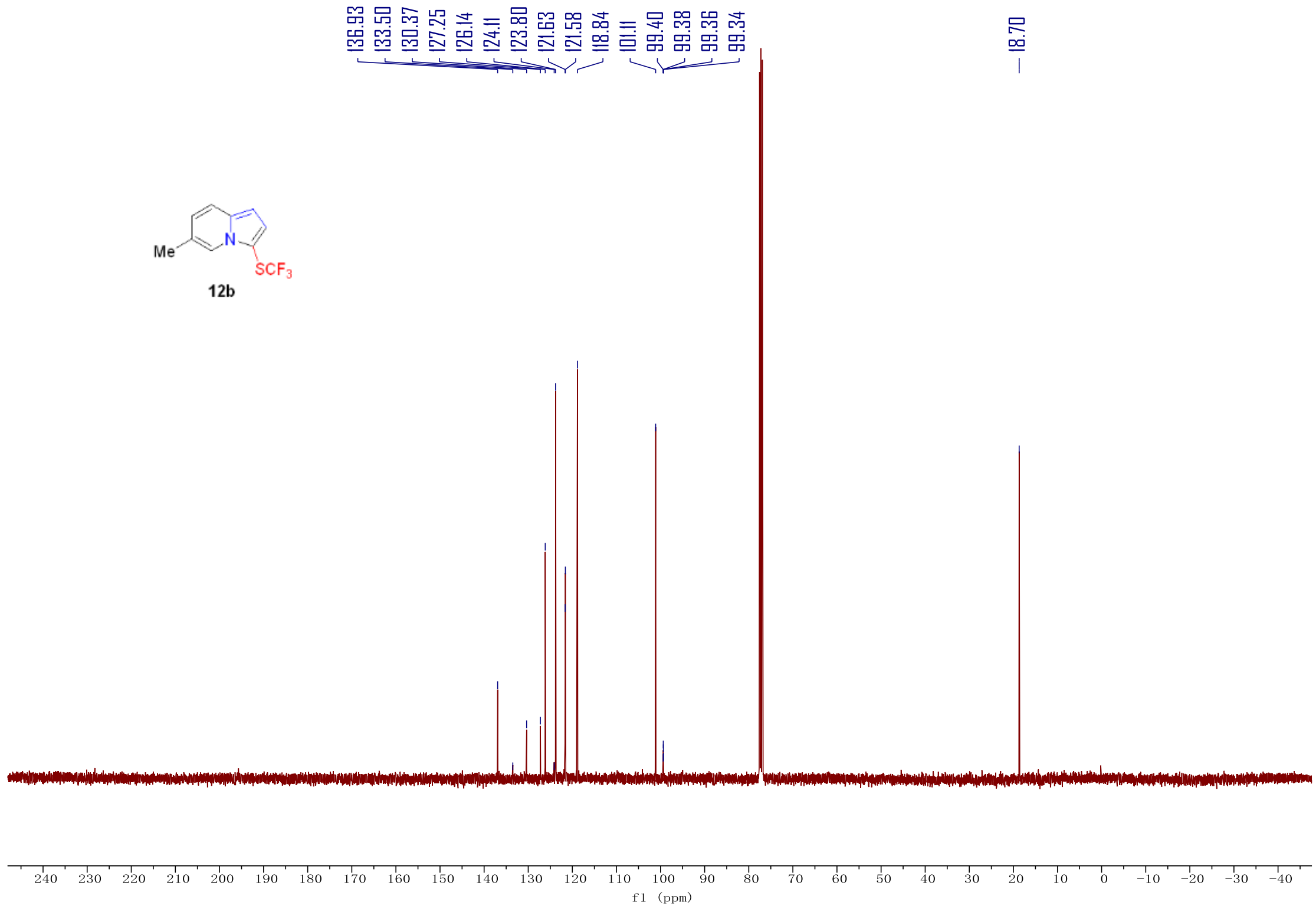

^{1}H NMR Spectrum of **12d** (400 MHz, $CDCl_3$)

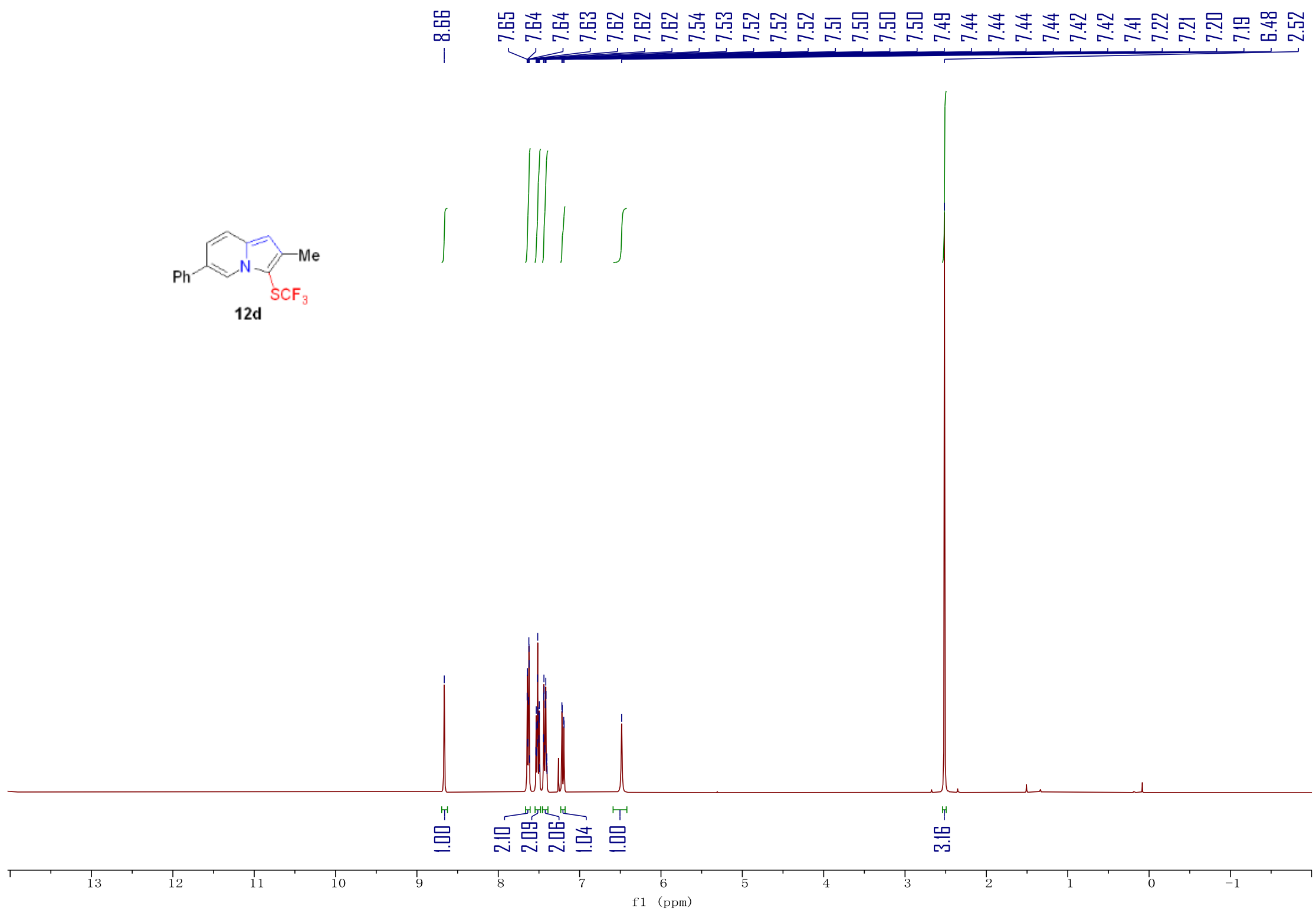

^{19}F NMR Spectrum of **12d** (376 MHz, $CDCl_3$)

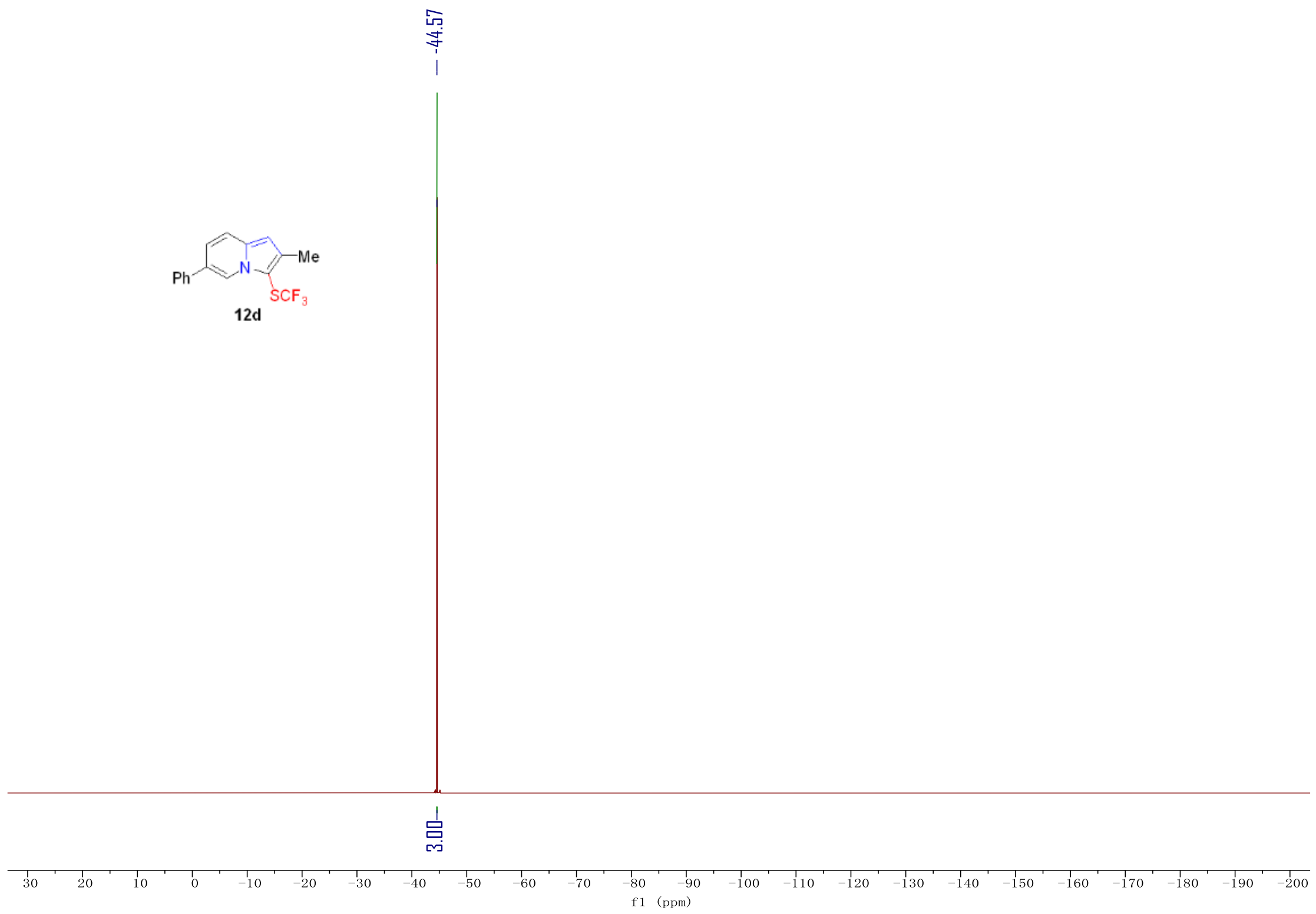

^{13}C NMR Spectrum of **12d** (101 MHz, $CDCl_3$)

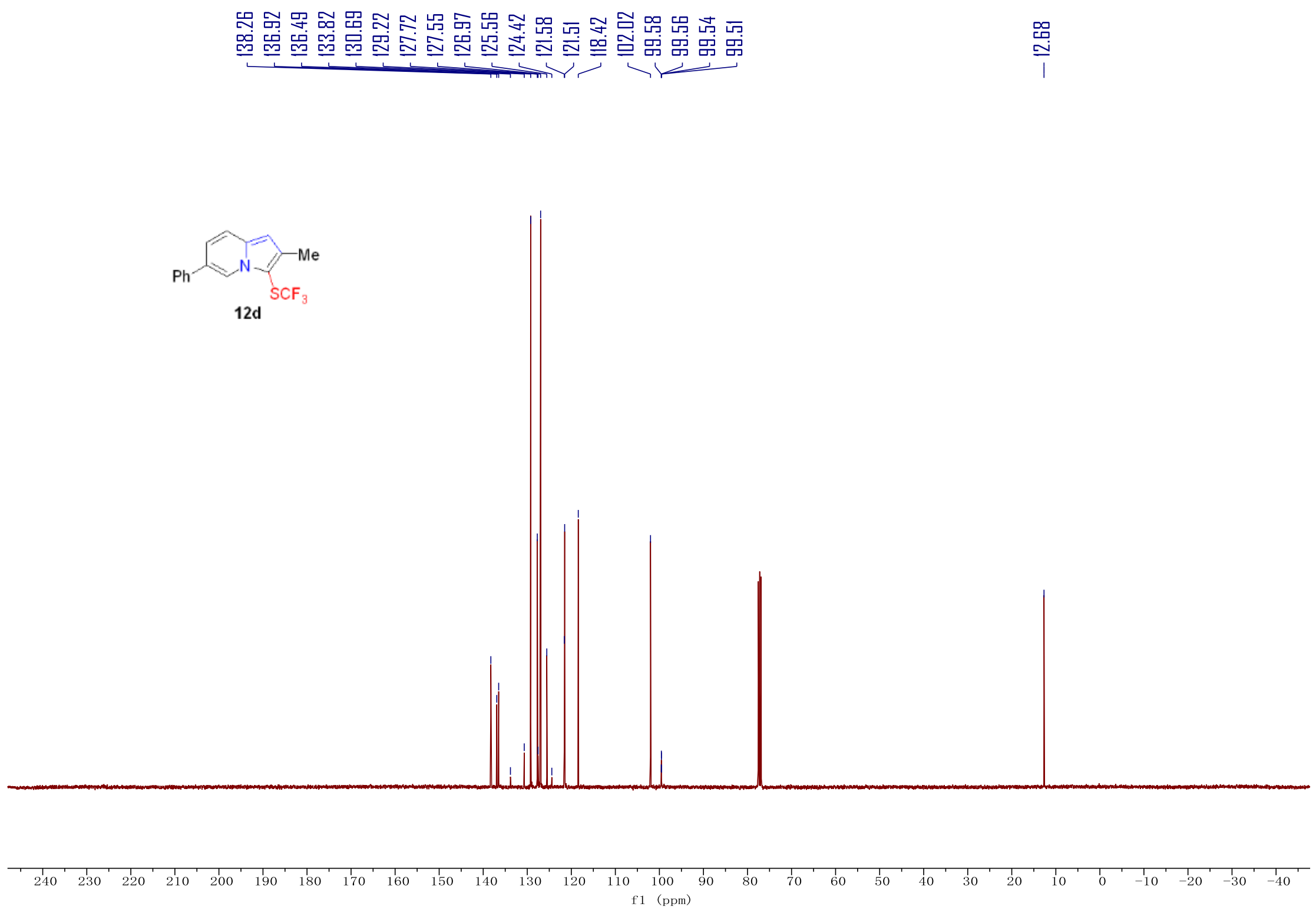

^{1}H NMR Spectrum of **12e** (400 MHz, $CDCl_3$)

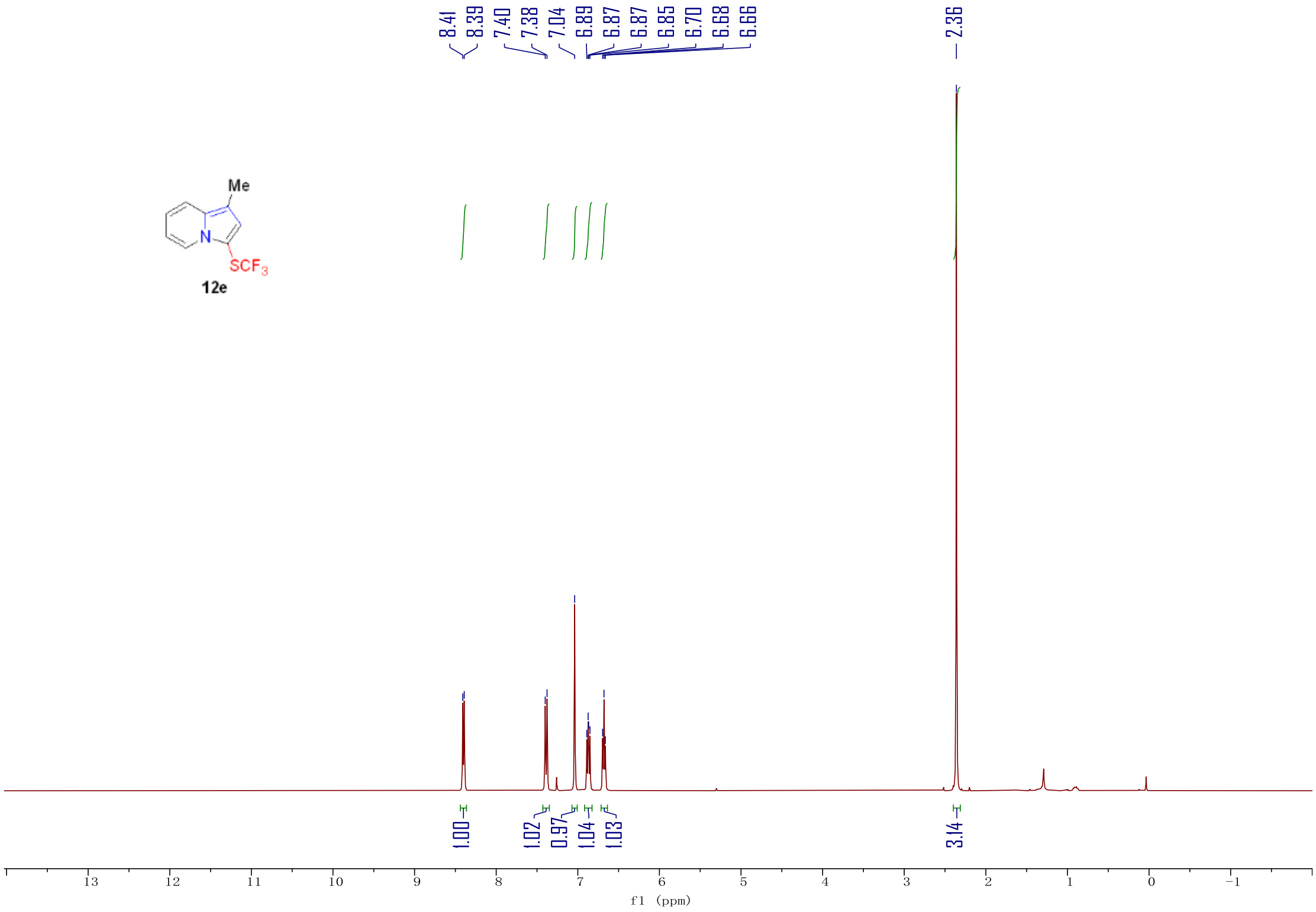

^{19}F NMR Spectrum of **12e** (376 MHz, $CDCl_3$)

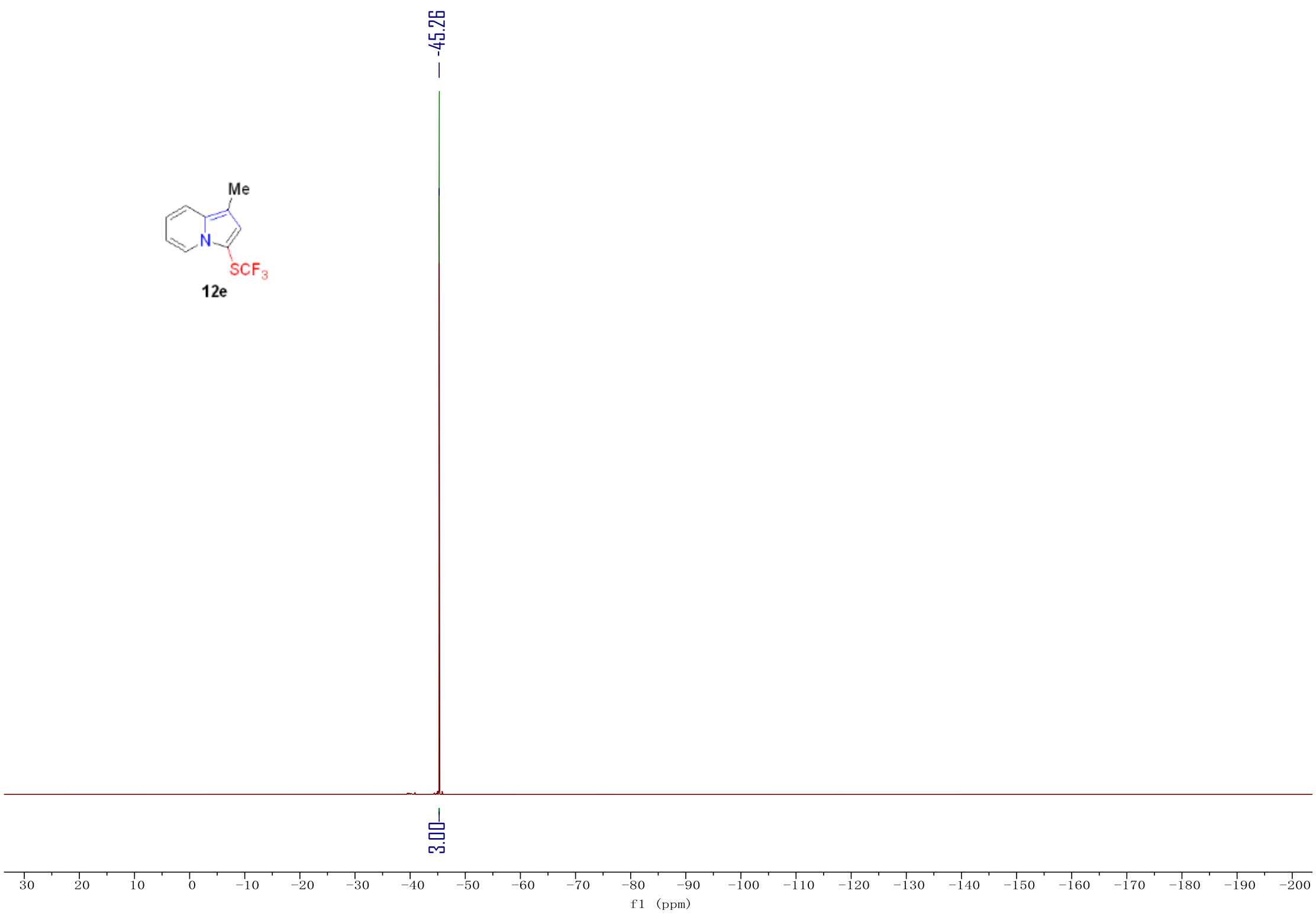

^{13}C NMR Spectrum of **12e** (101 MHz, $CDCl_3$)

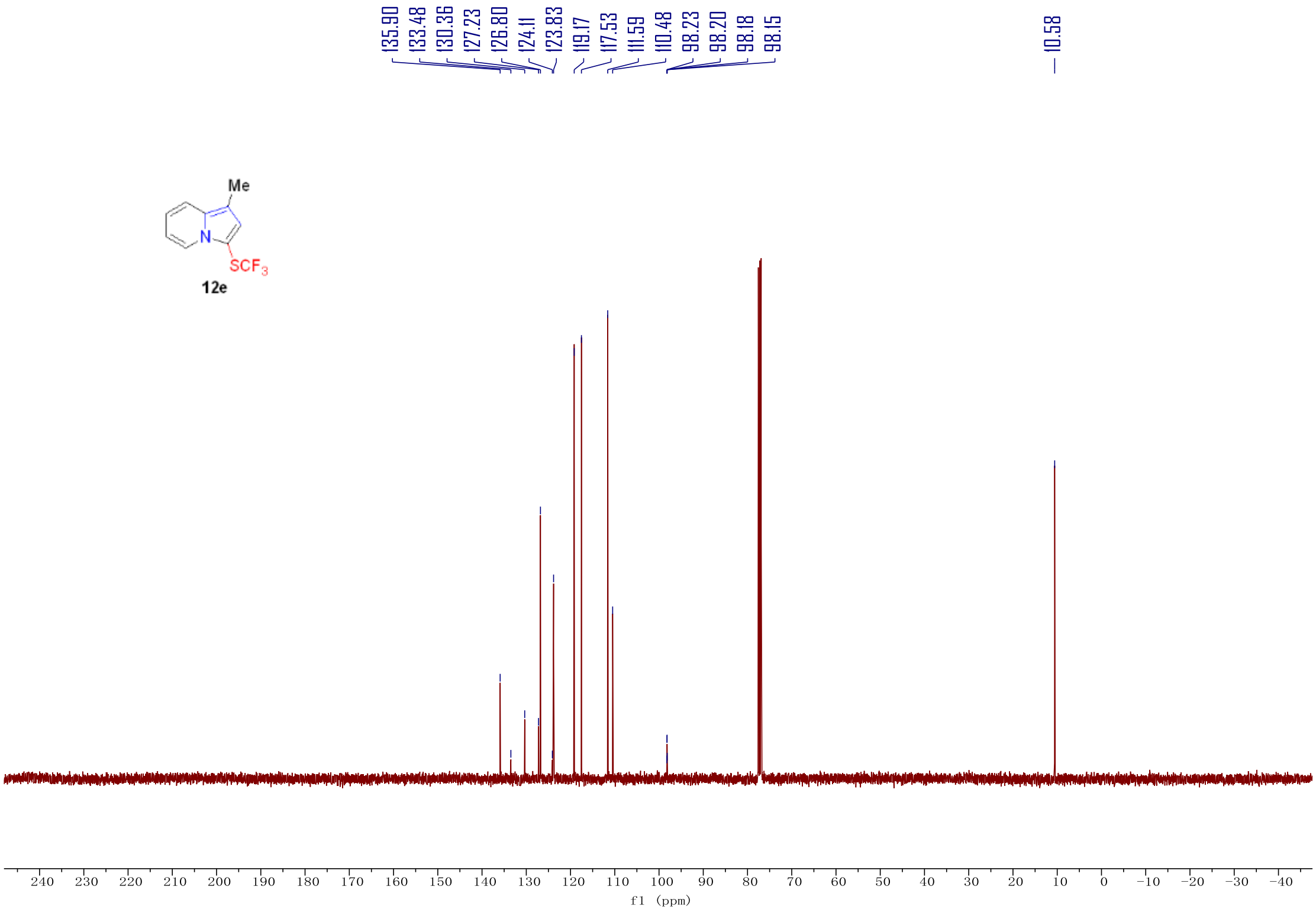

^{1}H NMR Spectrum of **12f** (400 MHz, $CDCl_3$)

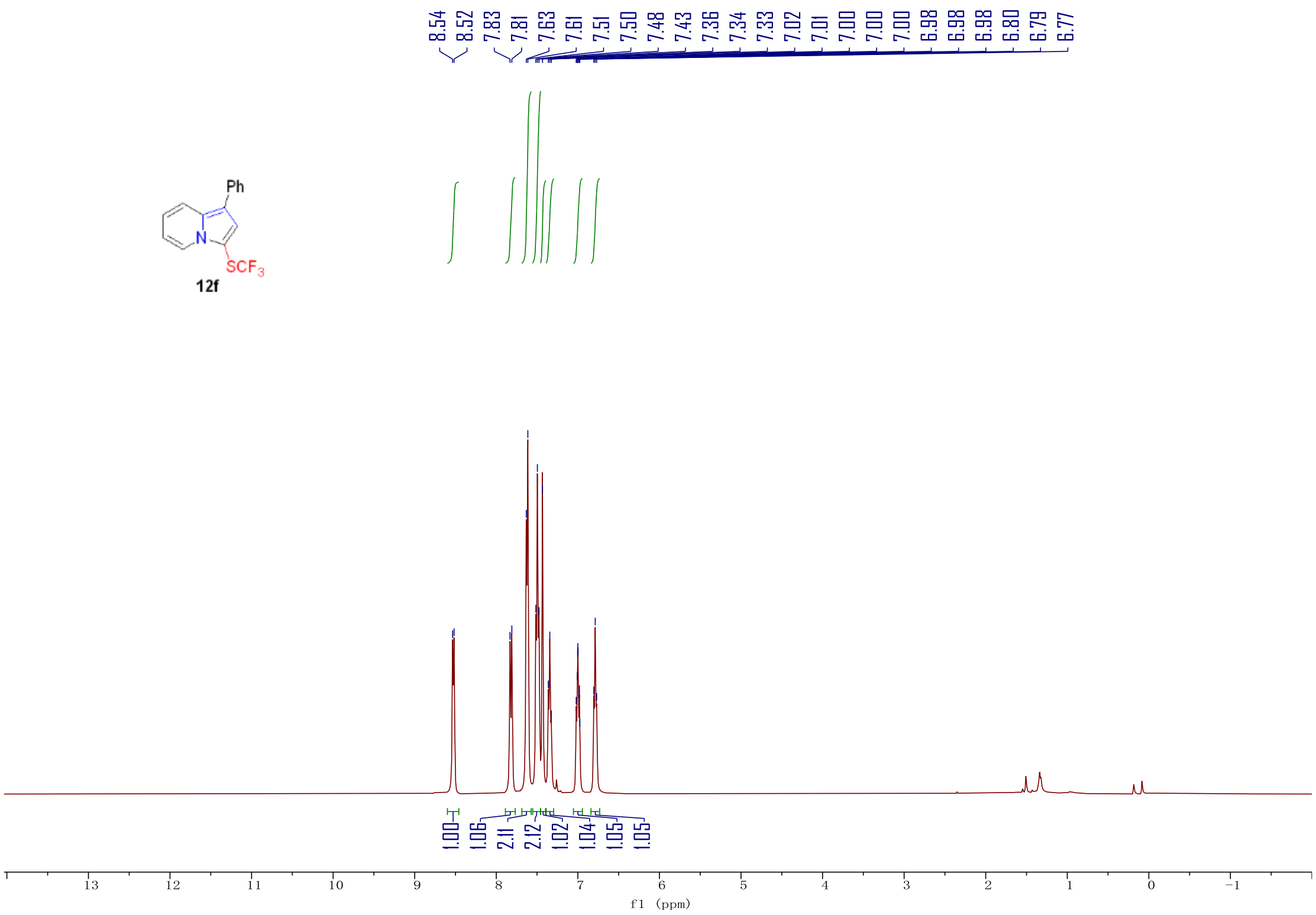

^{19}F **NMR** Spectrum of **12f** (376 MHz, $CDCl_3$)

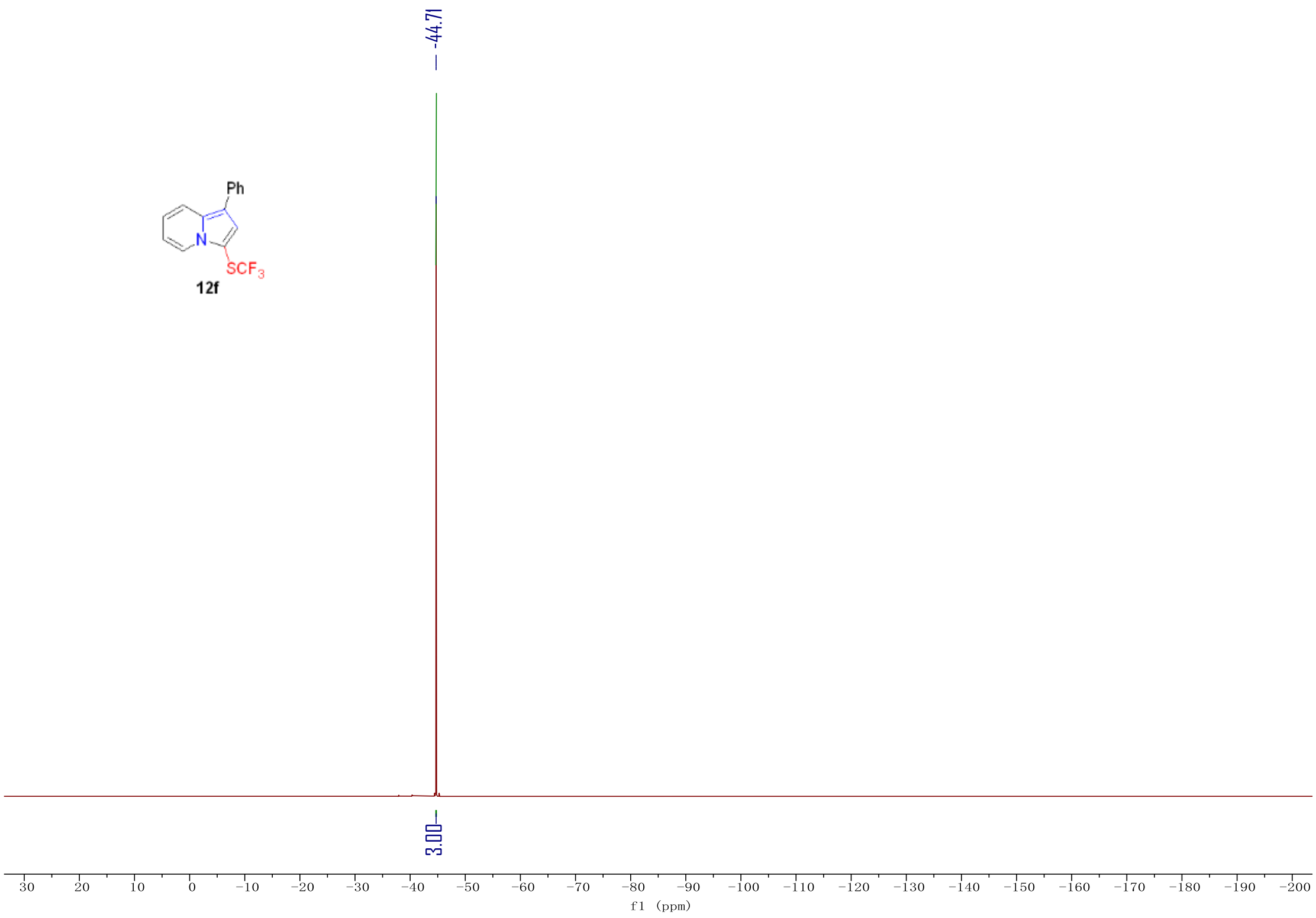

^{13}C **NMR** Spectrum of **12f** (101 MHz, $CDCl_3$)

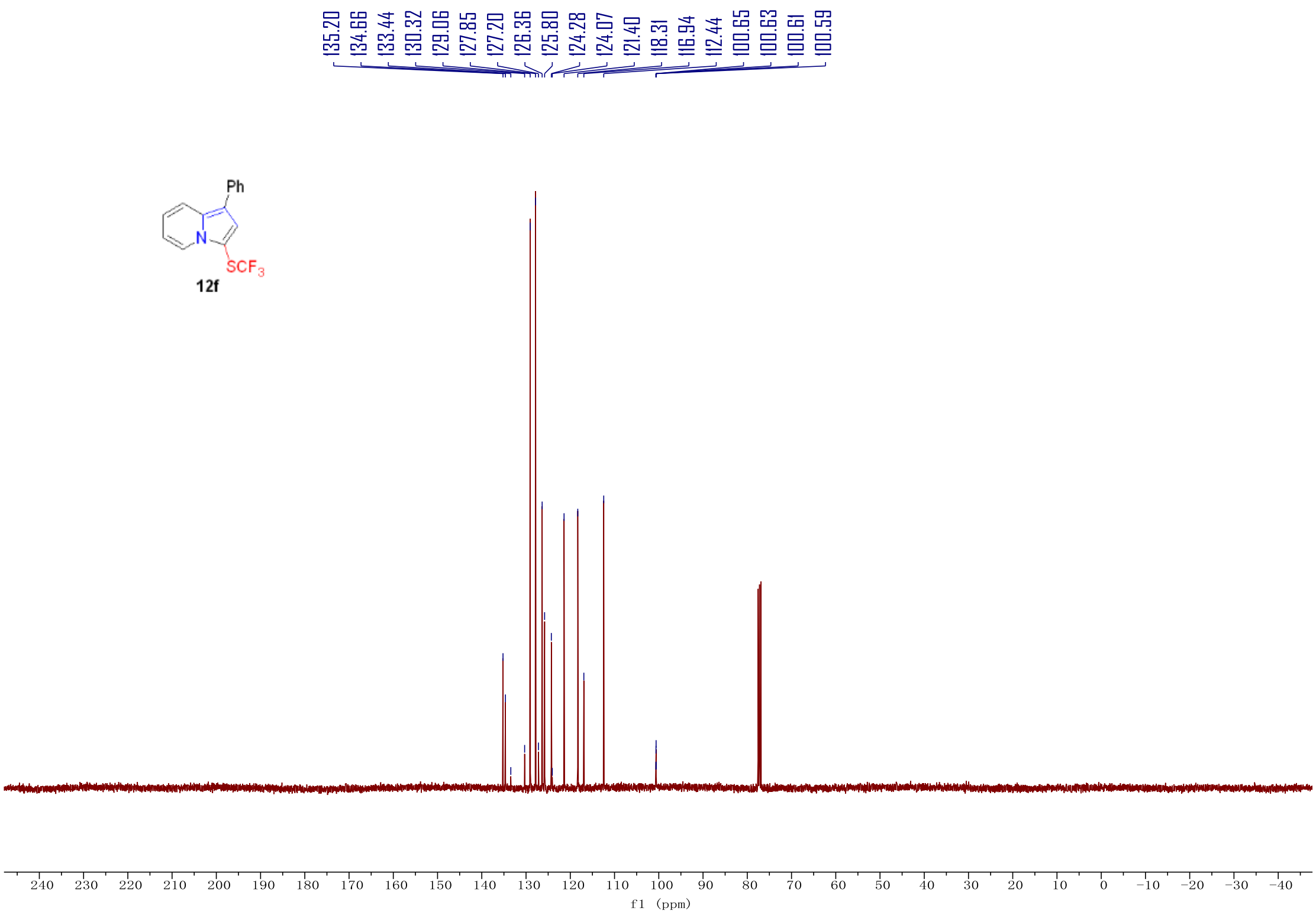

^{1}H NMR Spectrum of **12g** (400 MHz, $CDCl_3$)

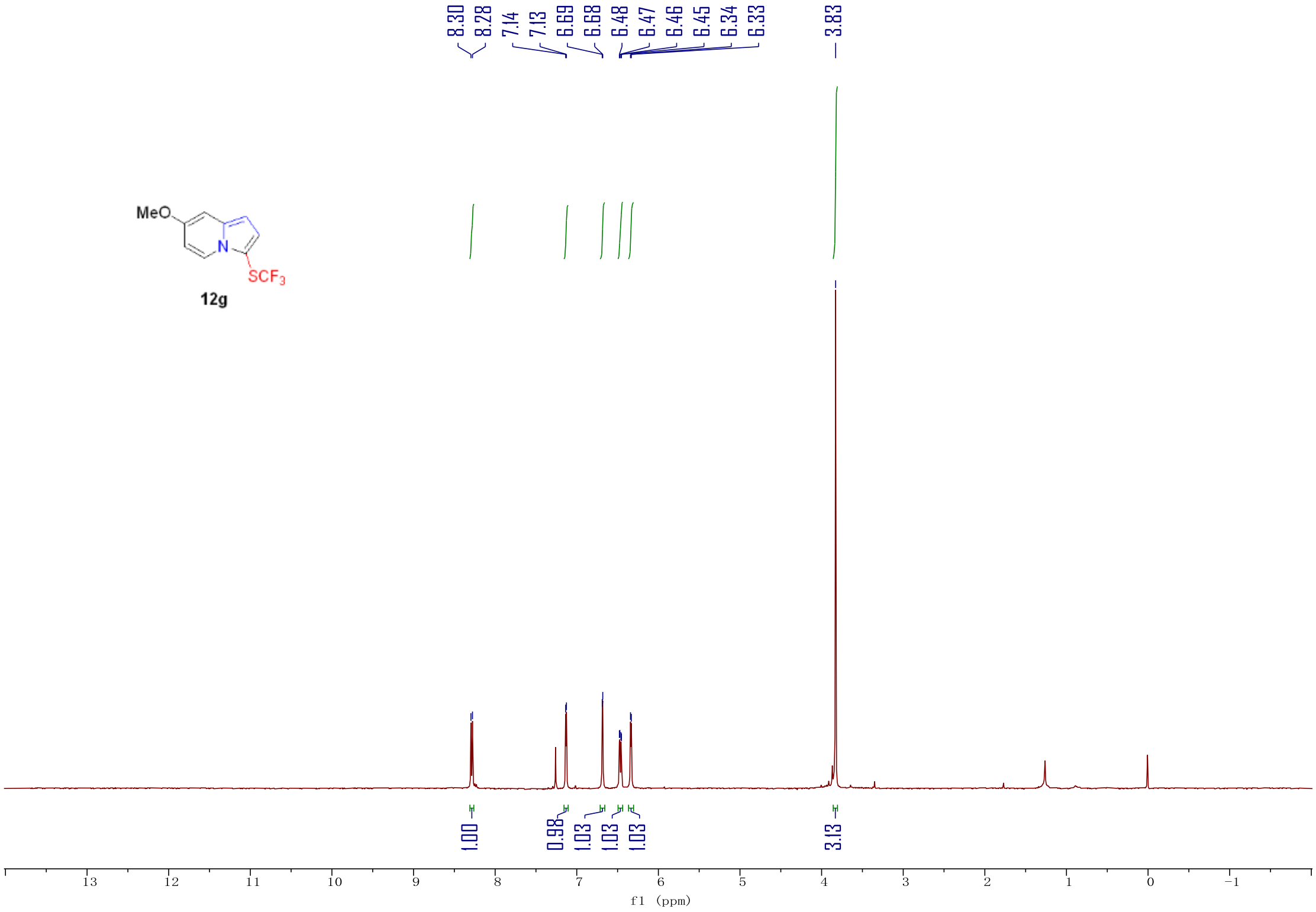

^{19}F NMR Spectrum of **12g** (376 MHz, $CDCl_3$)

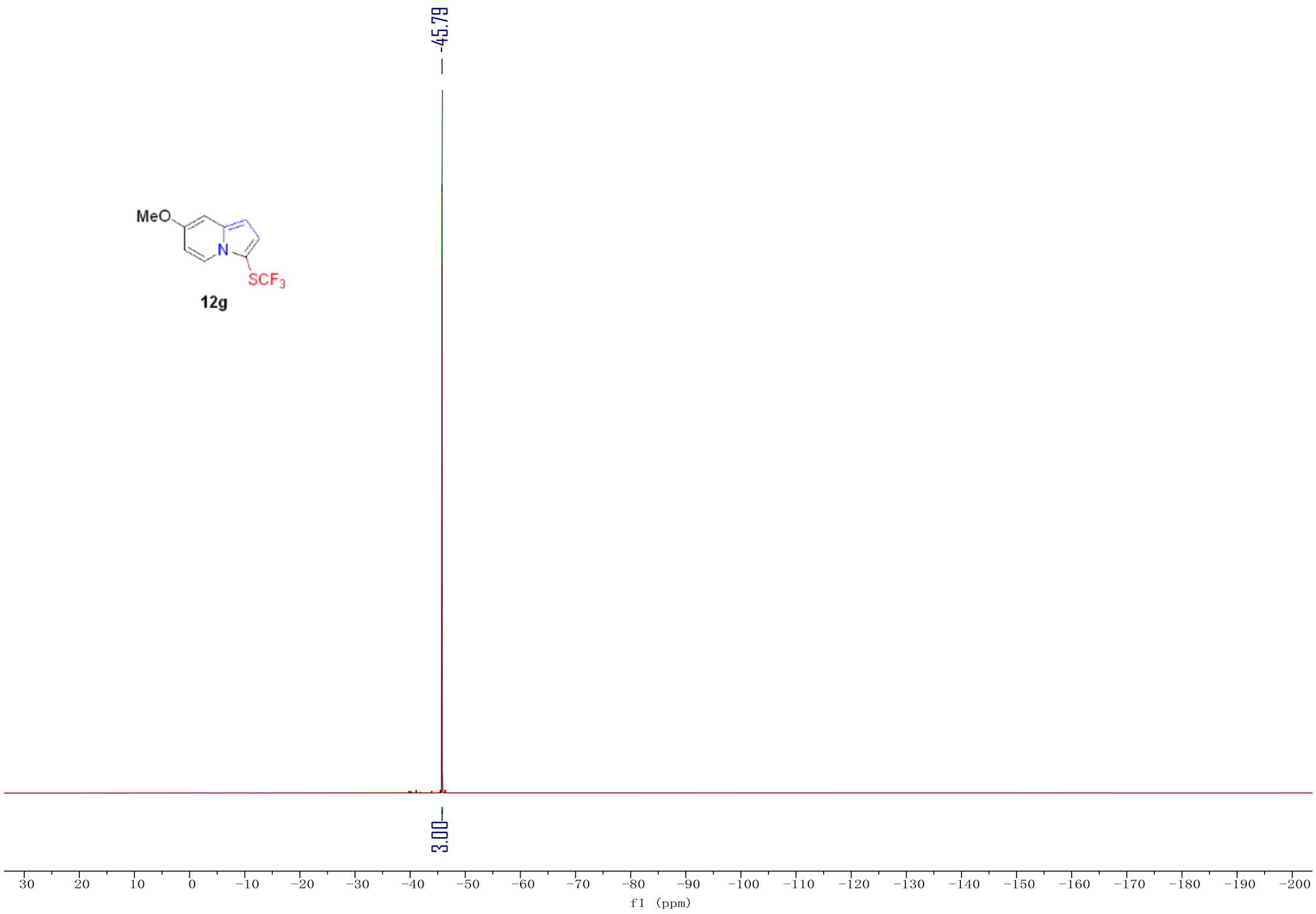

^{13}C NMR Spectrum of **12g** (101 MHz, $CDCl_3$)

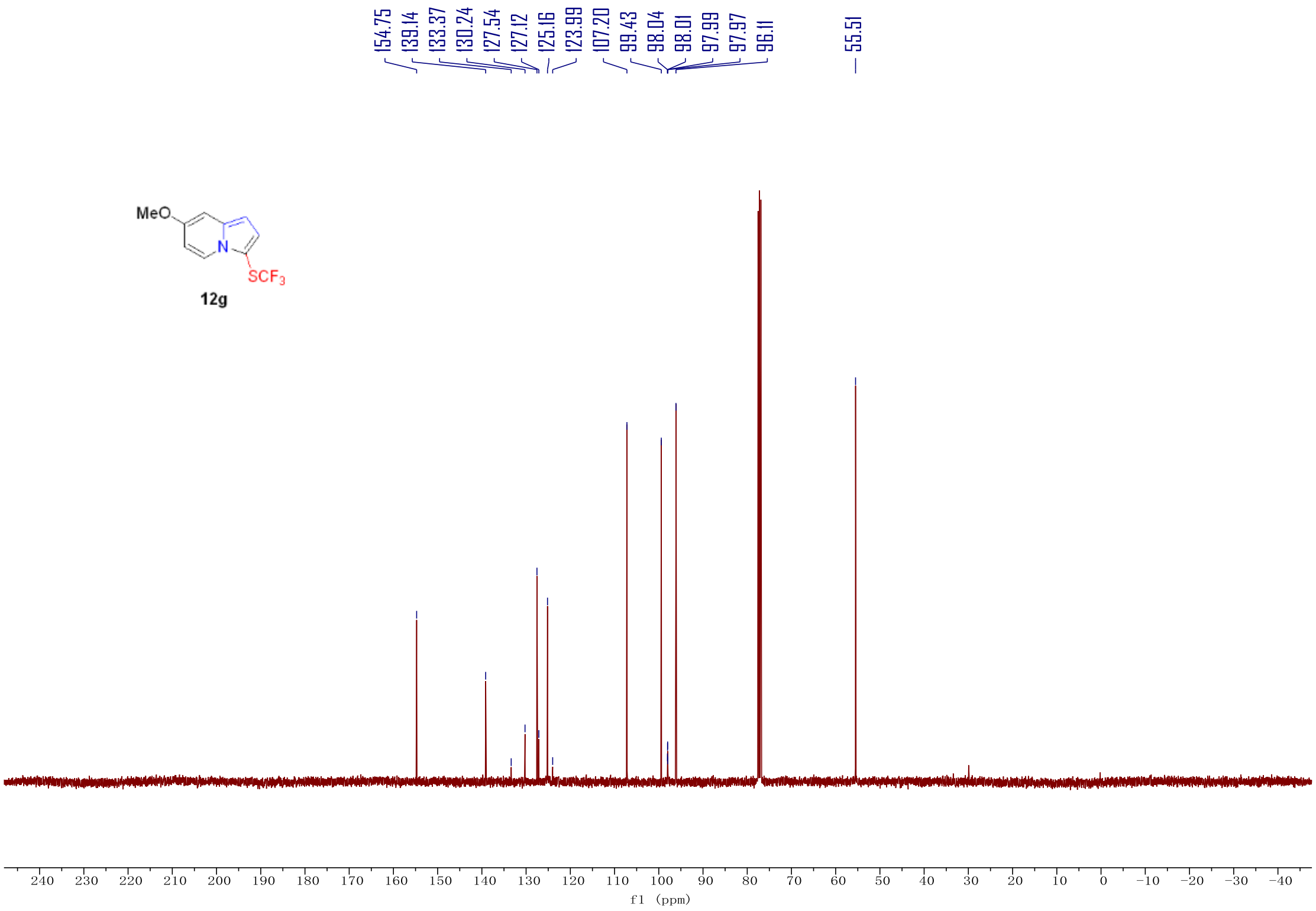

^{1}H NMR Spectrum of **12h** (400 MHz, $CDCl_3$)

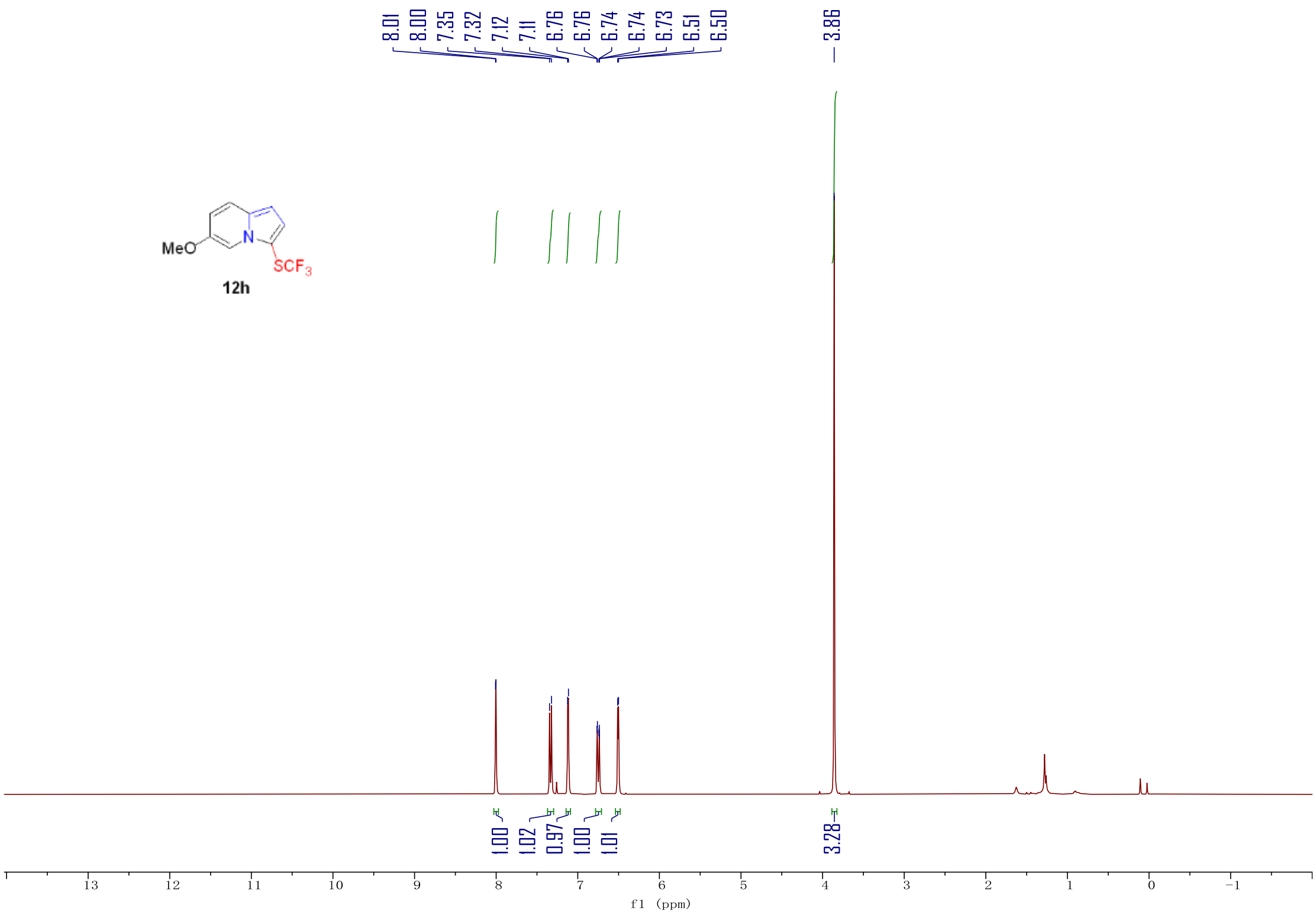

^{19}F NMR Spectrum of **12h** (376 MHz, $CDCl_3$)

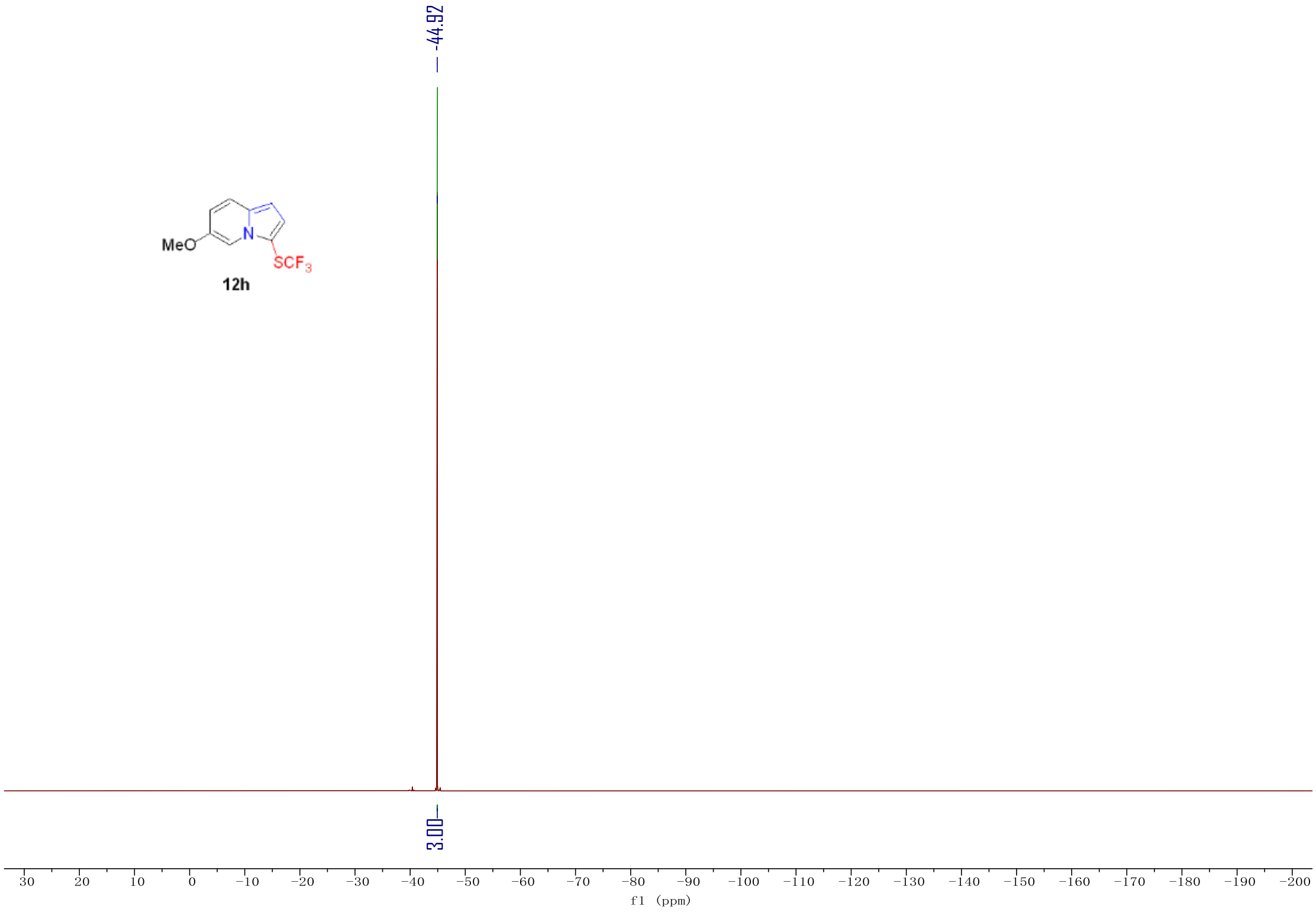

^{13}C NMR Spectrum of **12h** (101 MHz, $CDCl_3$)

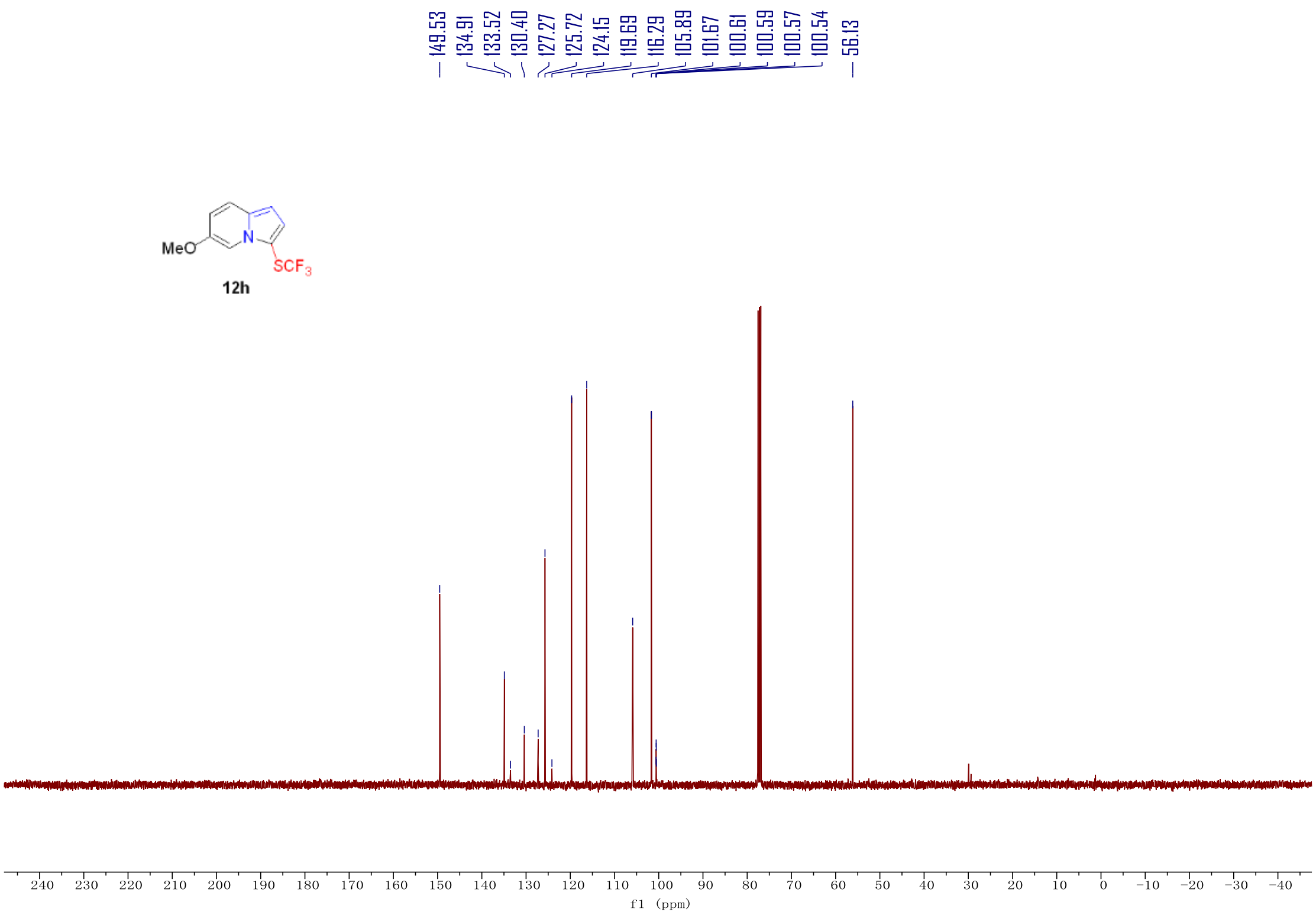

^{1}H NMR Spectrum of **12i** (400 MHz, $CDCl_3$)

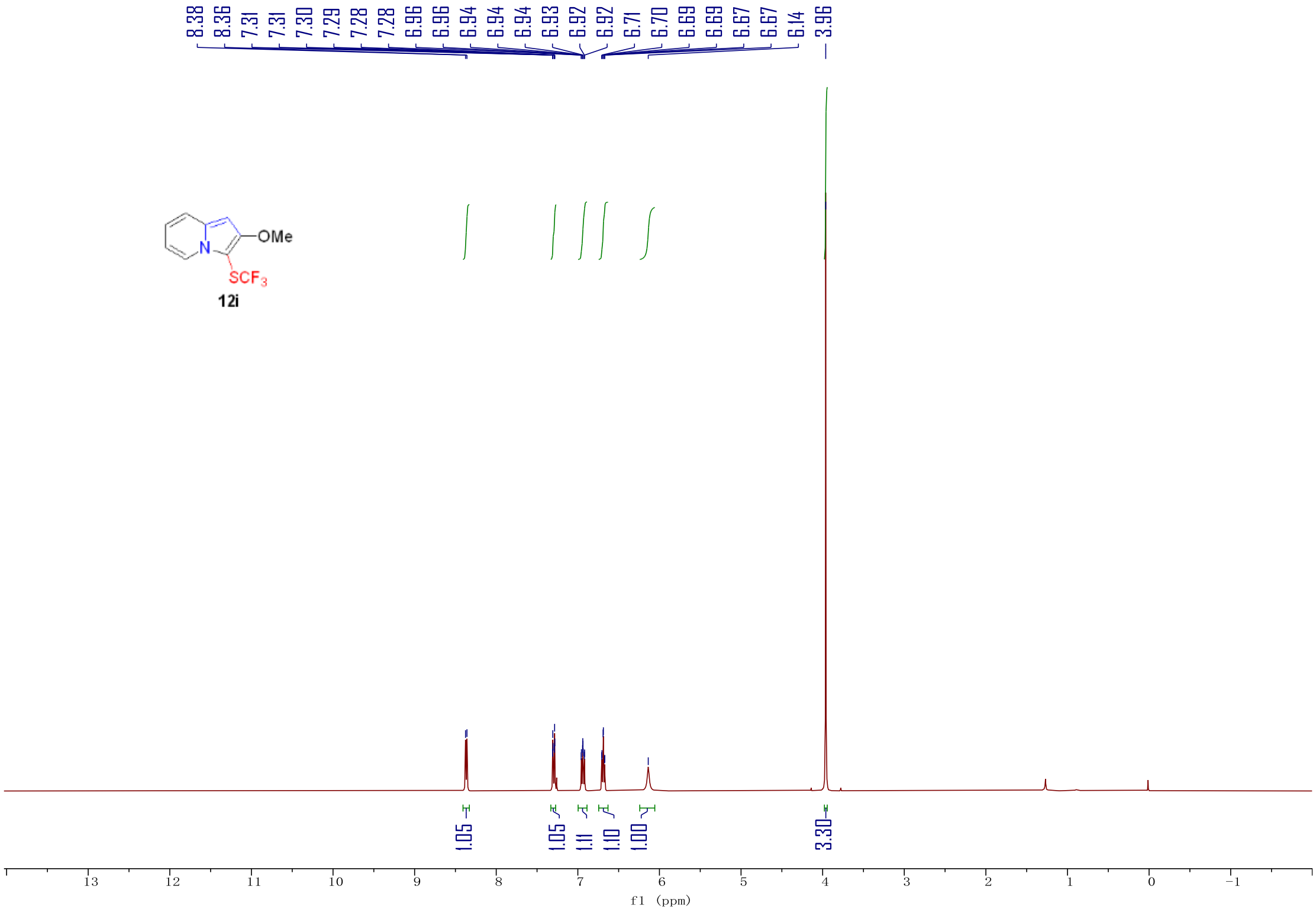

^{19}F NMR Spectrum of **12i** (376 MHz, $CDCl_3$)

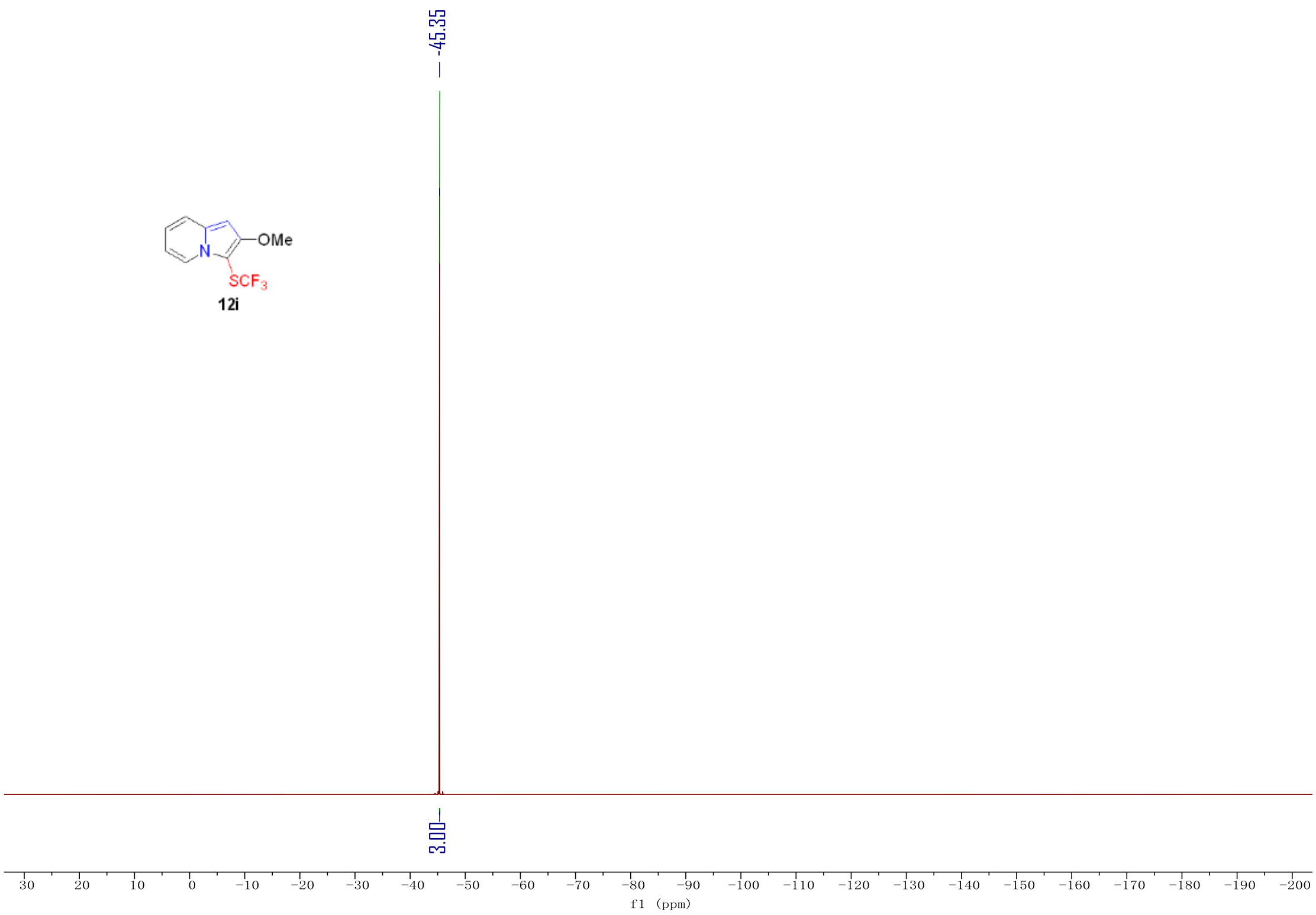

^{13}C NMR Spectrum of **12i** (101 MHz, $CDCl_3$)

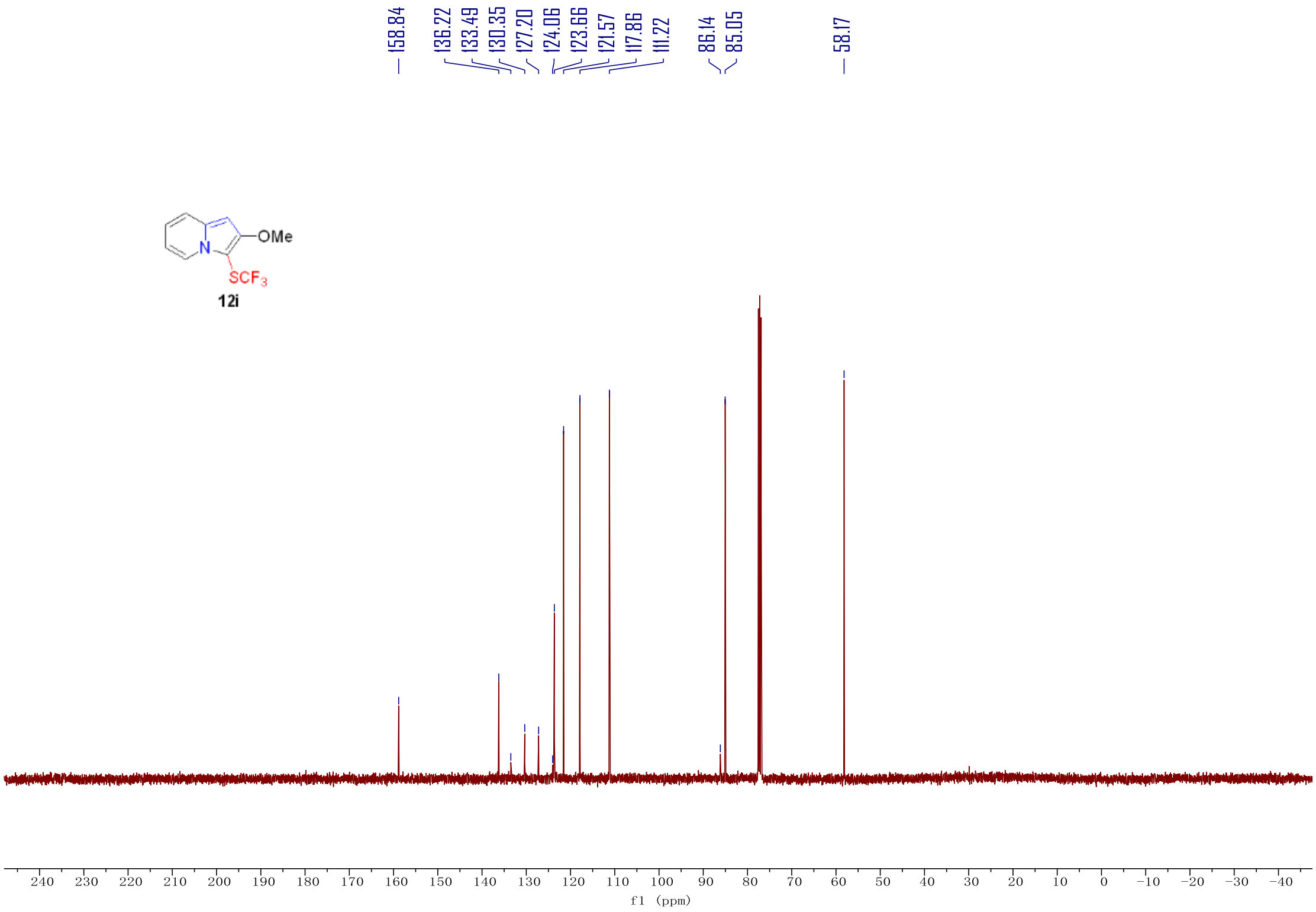

^{1}H NMR Spectrum of **12j** (400 MHz, $CDCl_3$)

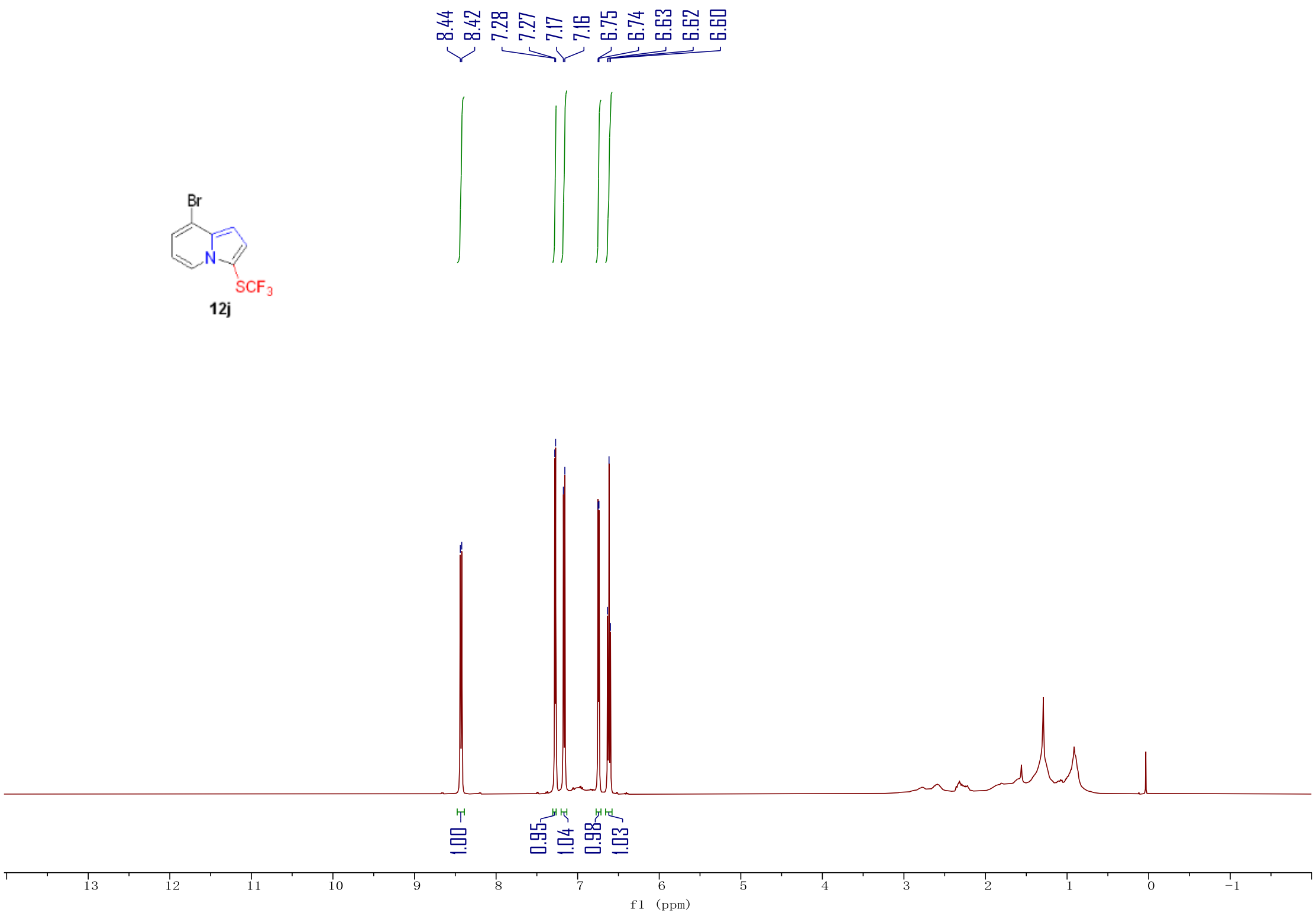

^{19}F NMR Spectrum of **12j** (376 MHz, $CDCl_3$)

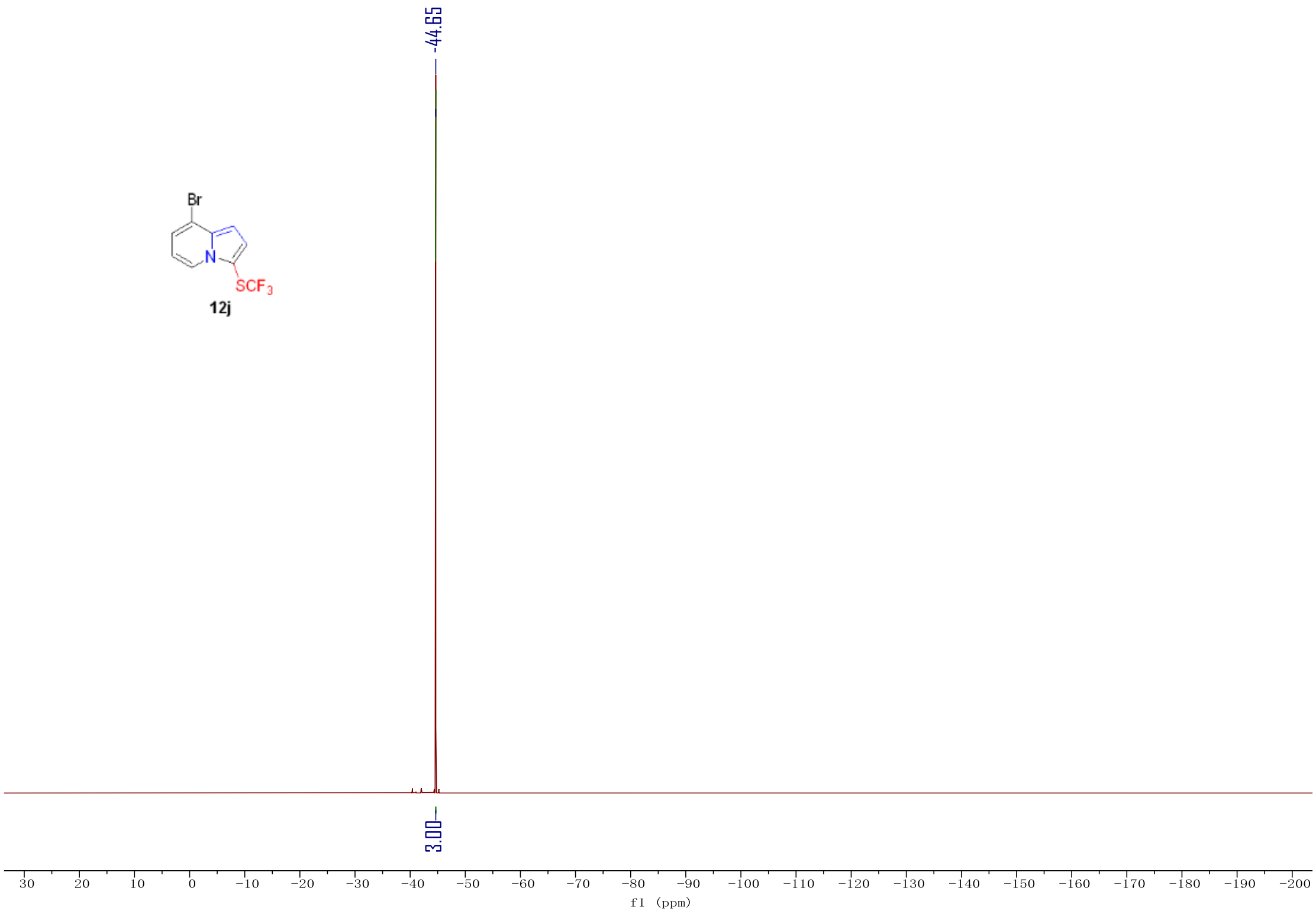

^{13}C NMR Spectrum of **12j** (101 MHz, $CDCl_3$)

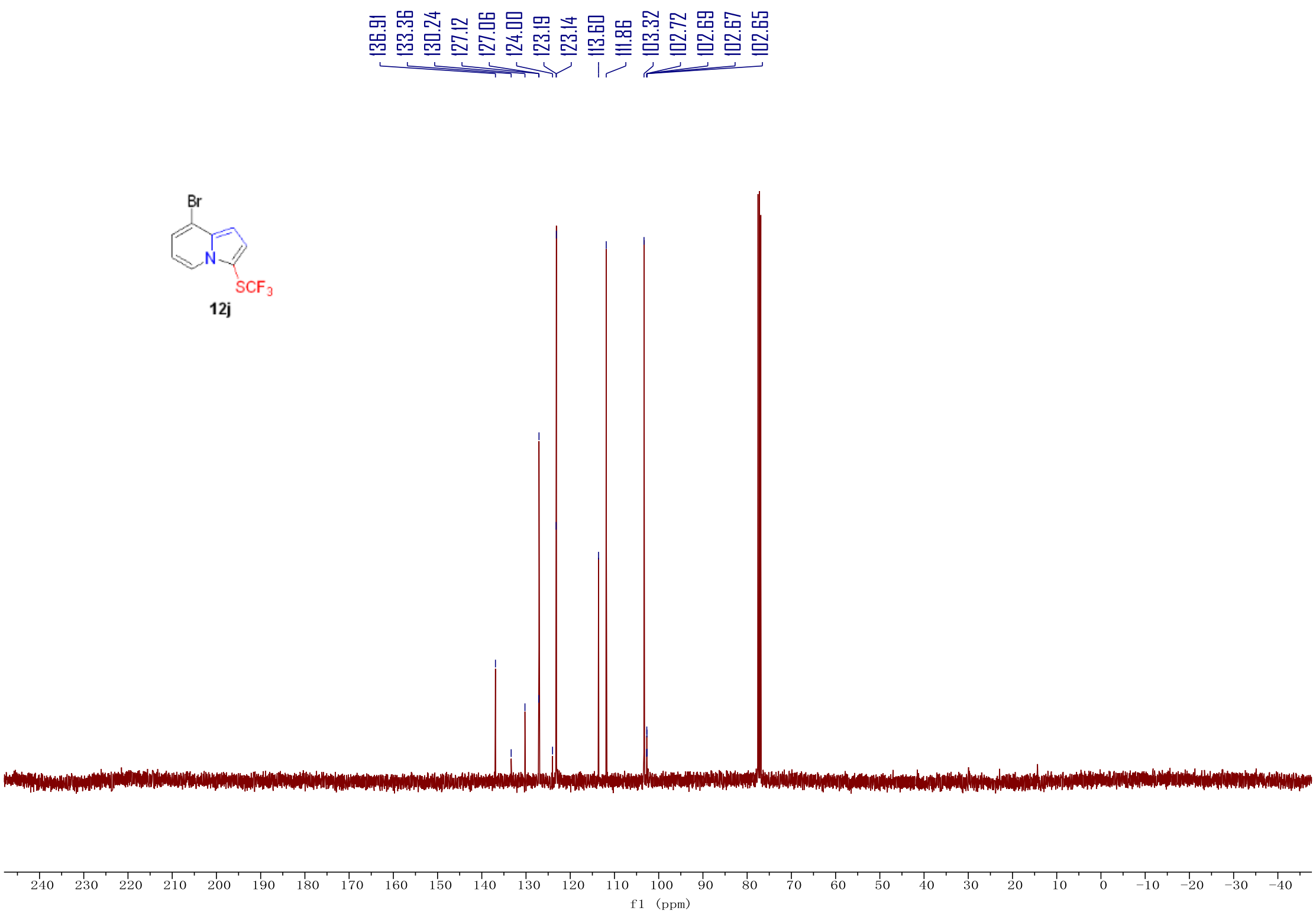

^{1}H NMR Spectrum of **12k** (400 MHz, $CDCl_3$)

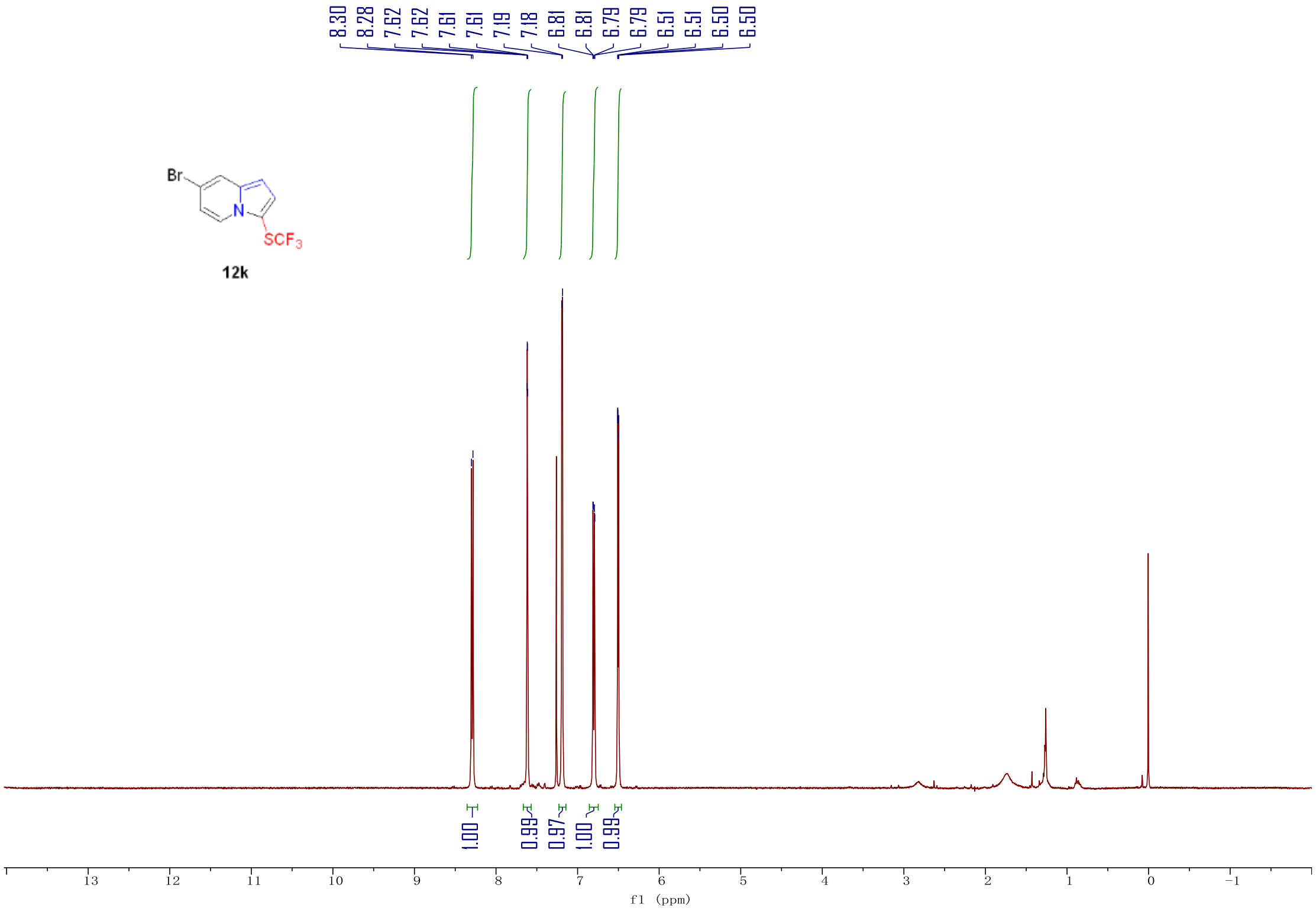

^{19}F NMR Spectrum of **12k** (376 MHz, $CDCl_3$)

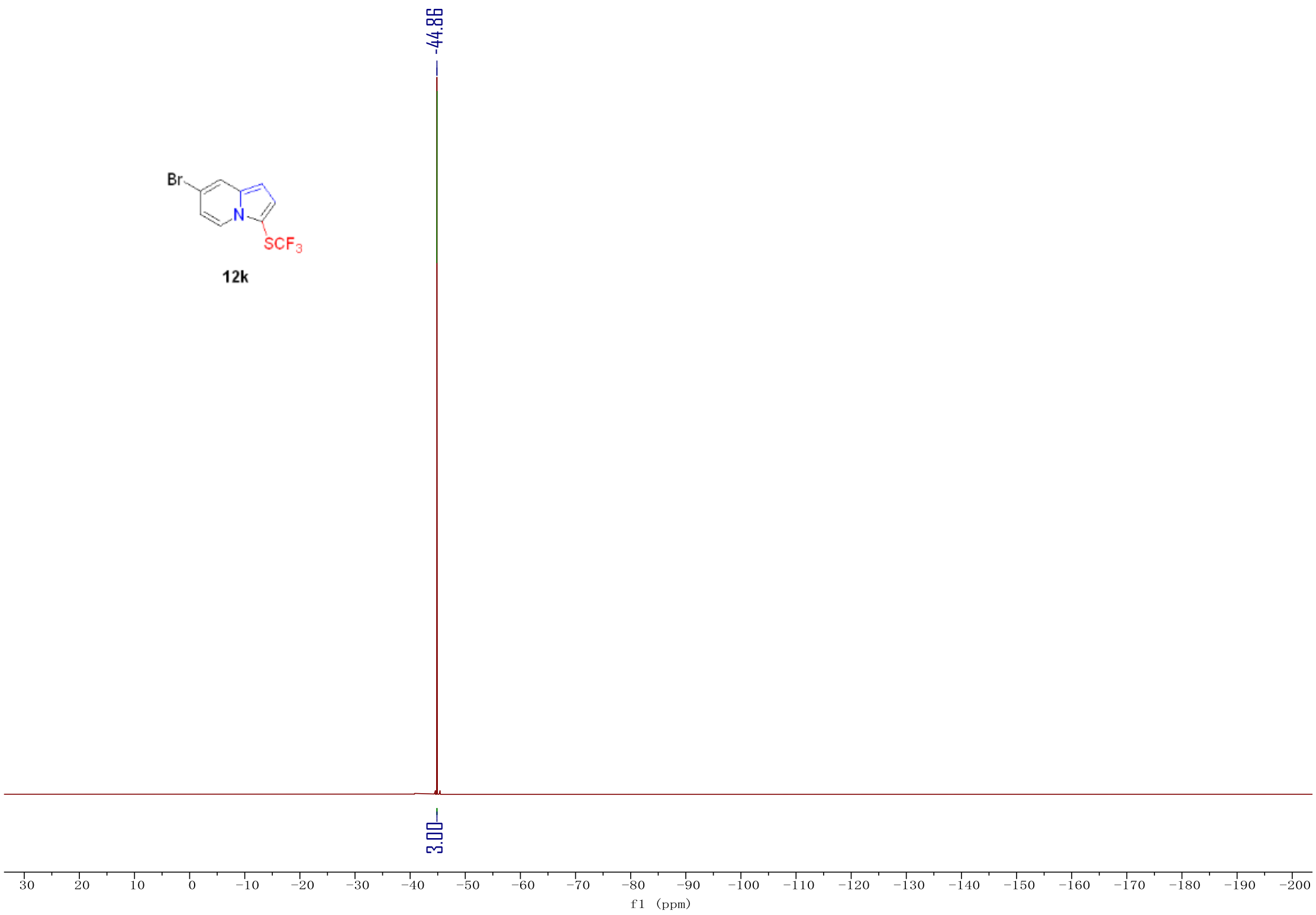

^{13}C **NMR** Spectrum of **12k** (101 MHz, $CDCl_3$)

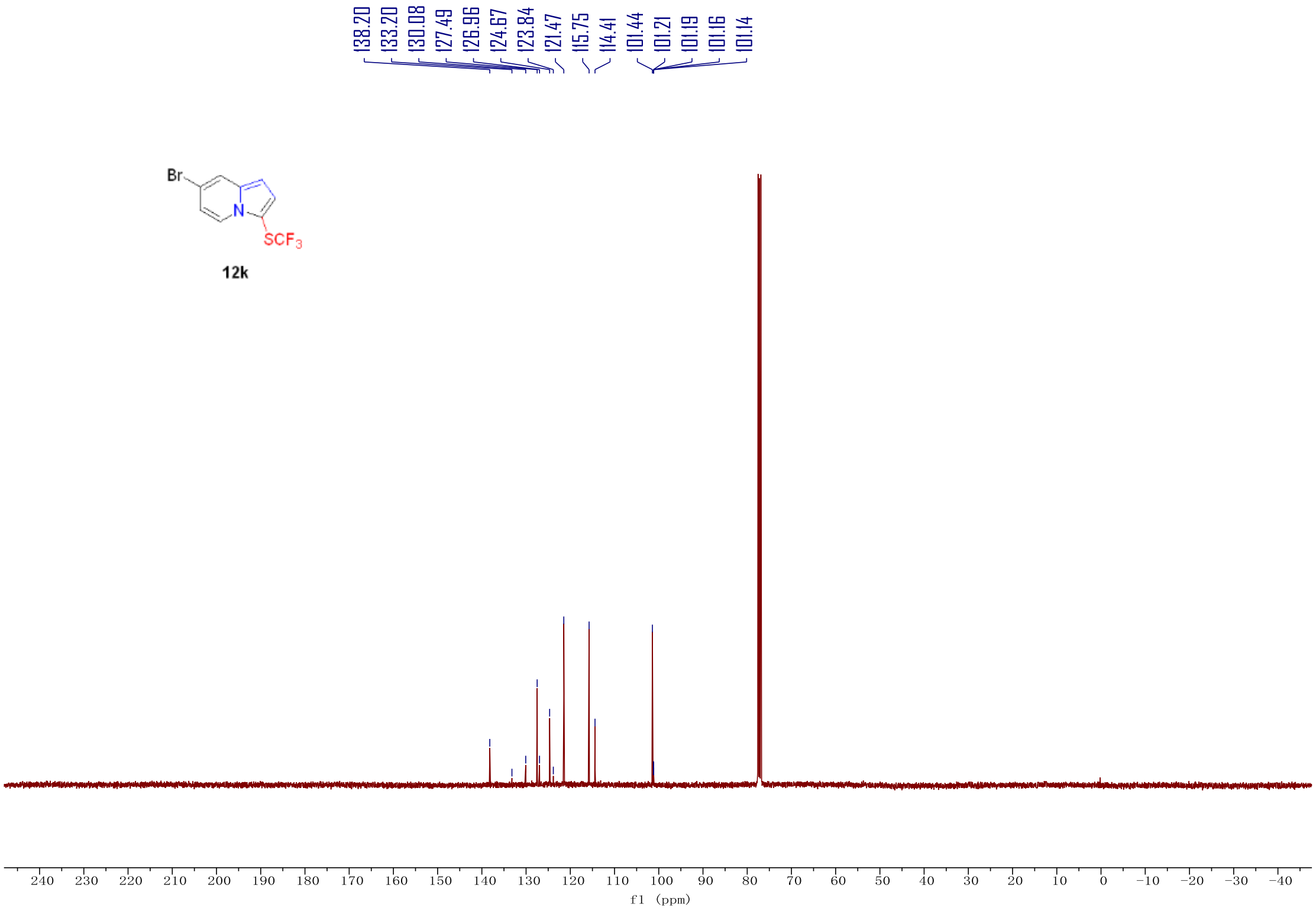

^{1}H **NMR** Spectrum of **12k'** (400 MHz, $CDCl_3$)

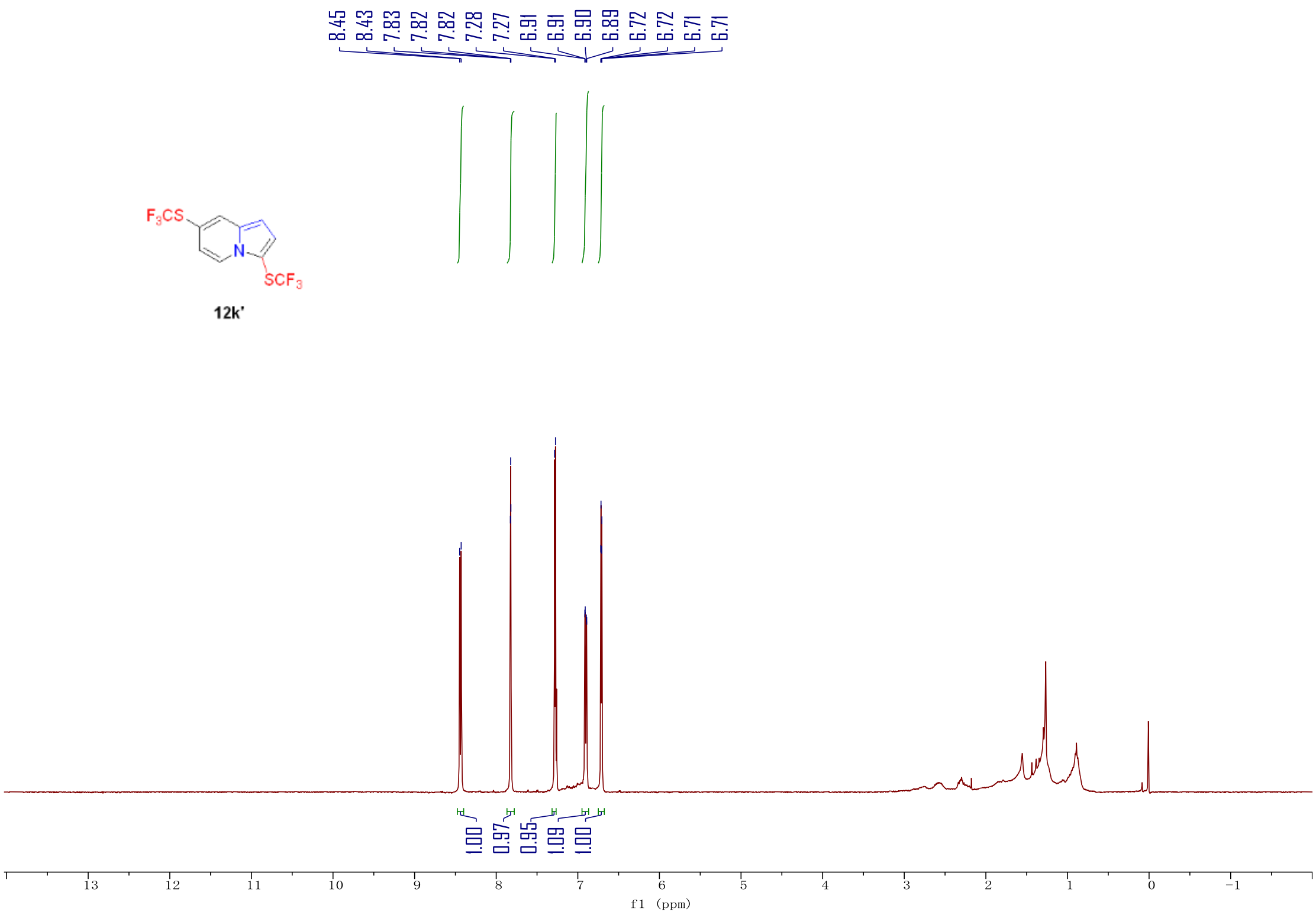

^{19}F NMR Spectrum of **12k'** (376 MHz, $CDCl_3$)

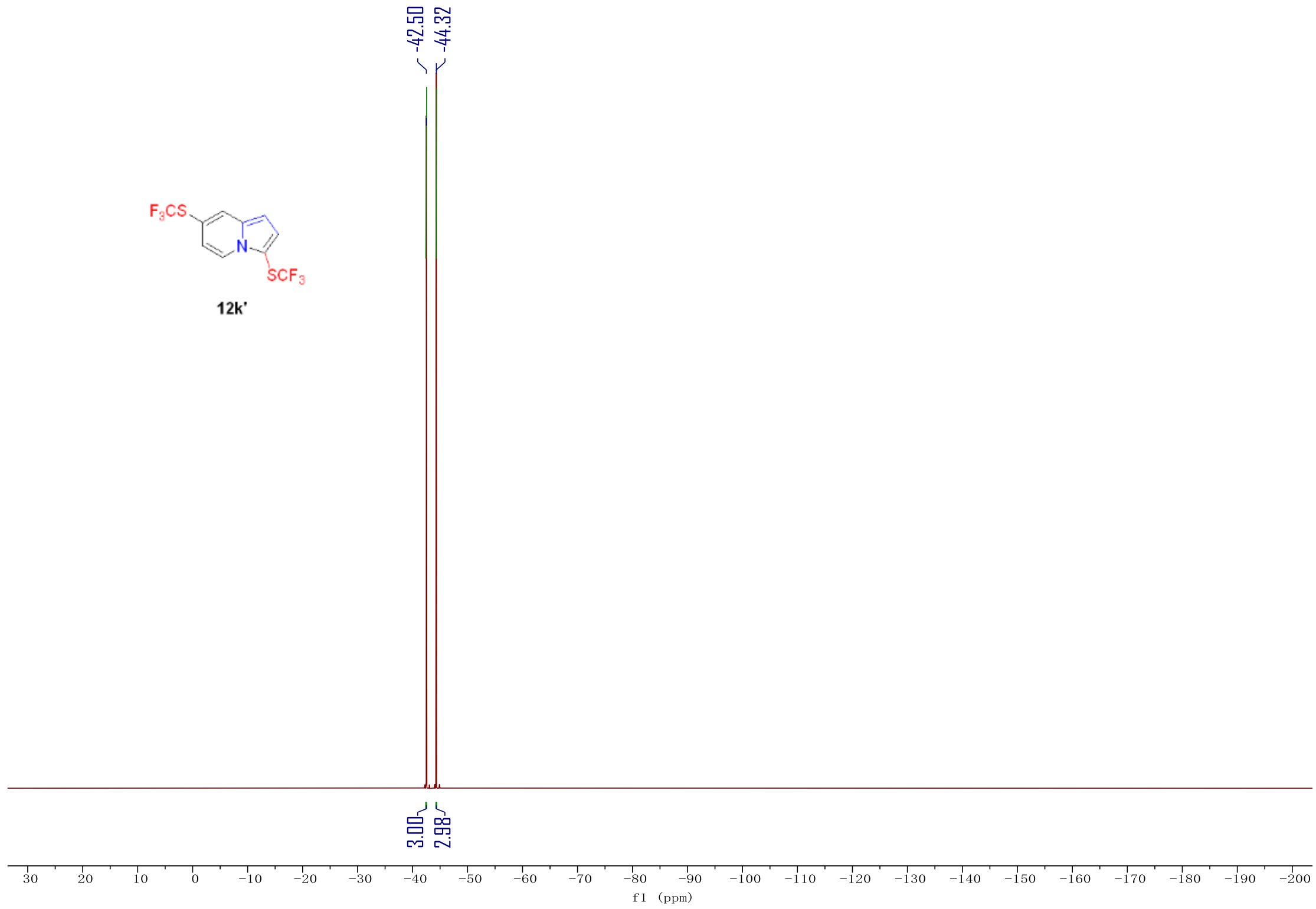

^{13}C NMR Spectrum of **12k'** (101 MHz, $CDCl_3$)

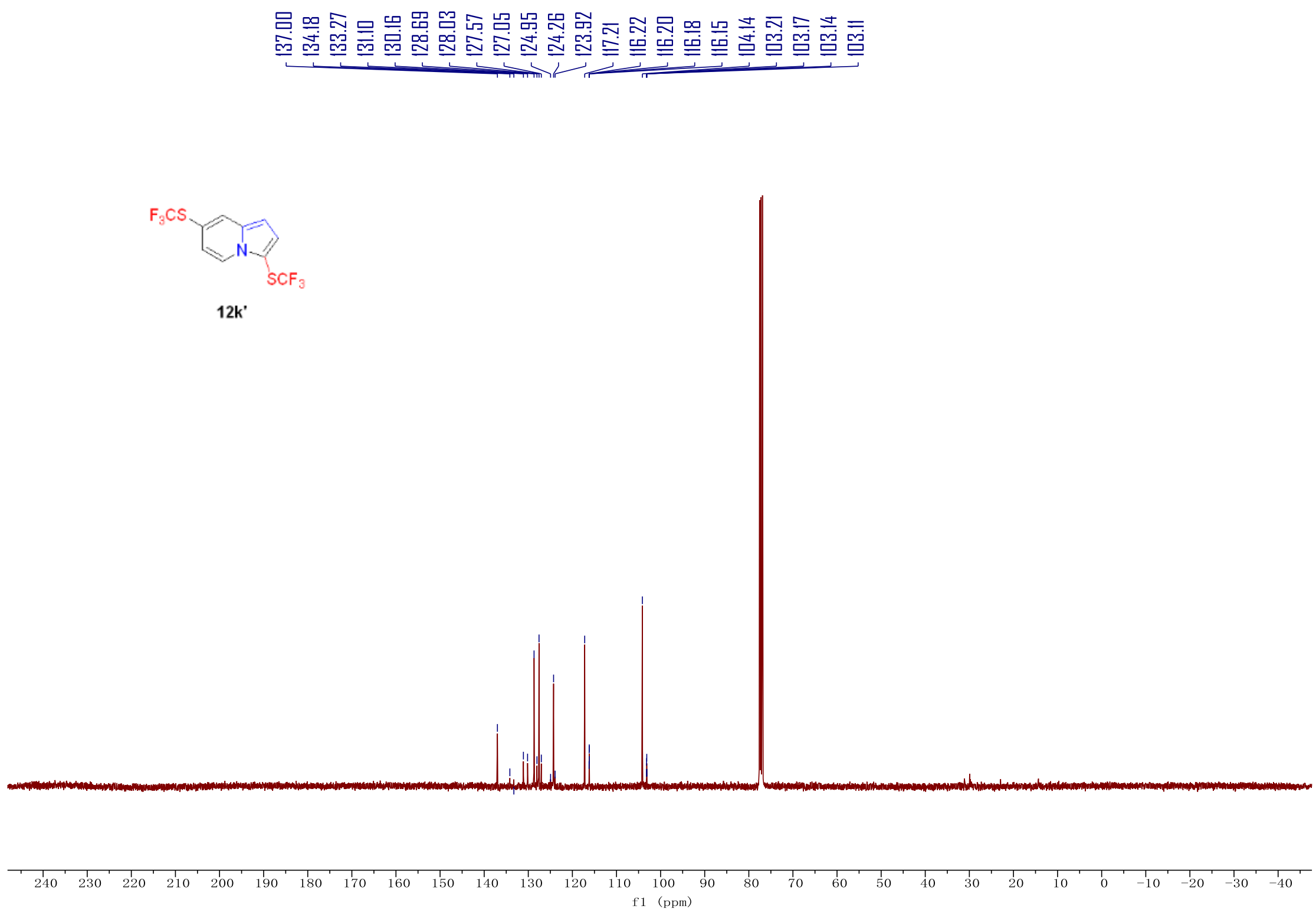

[1]**H NMR** Spectrum of **12l** (400 MHz, $CDCl_3$)

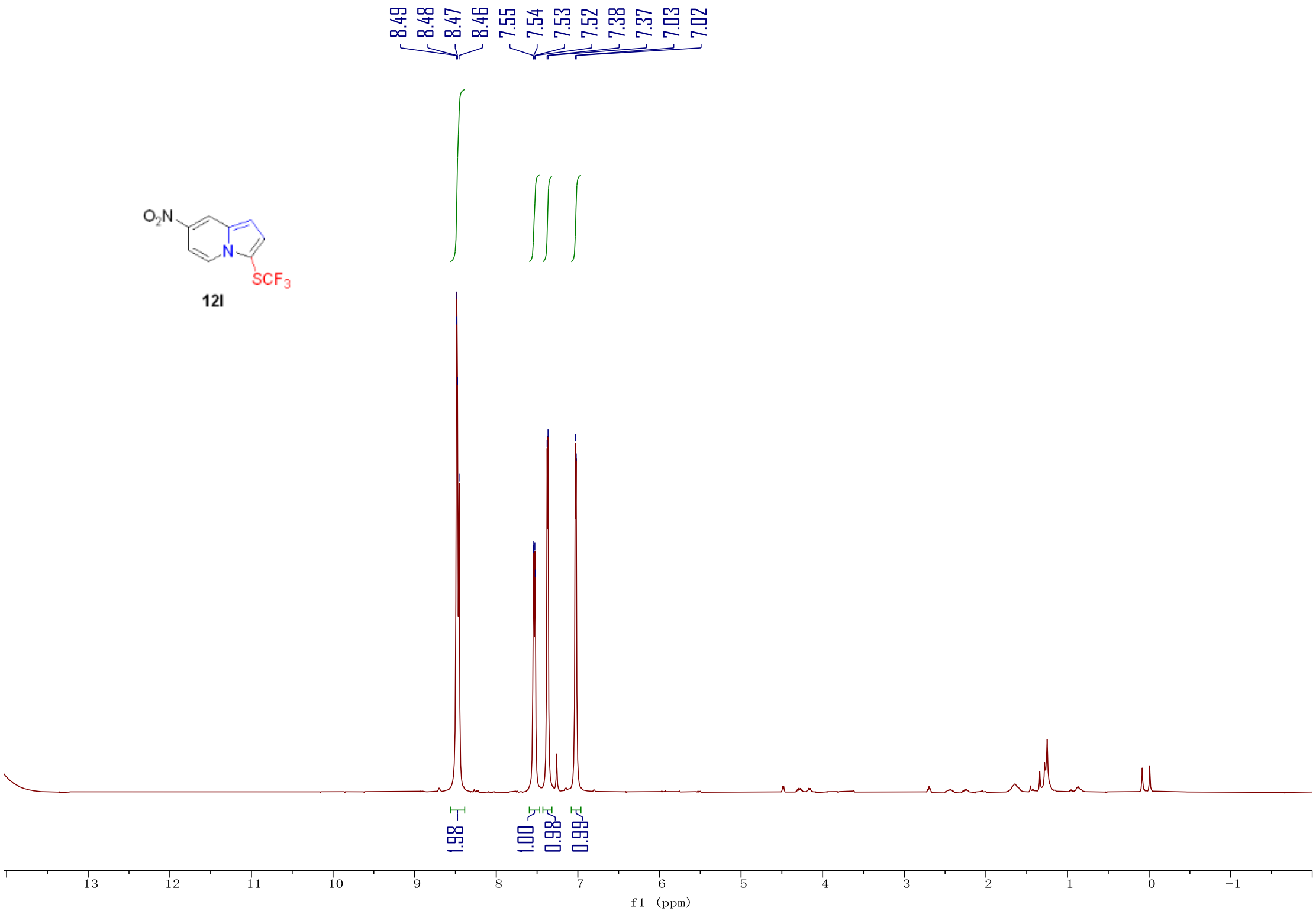

[19]**F NMR** Spectrum of **12l** (376 MHz, $CDCl_3$)

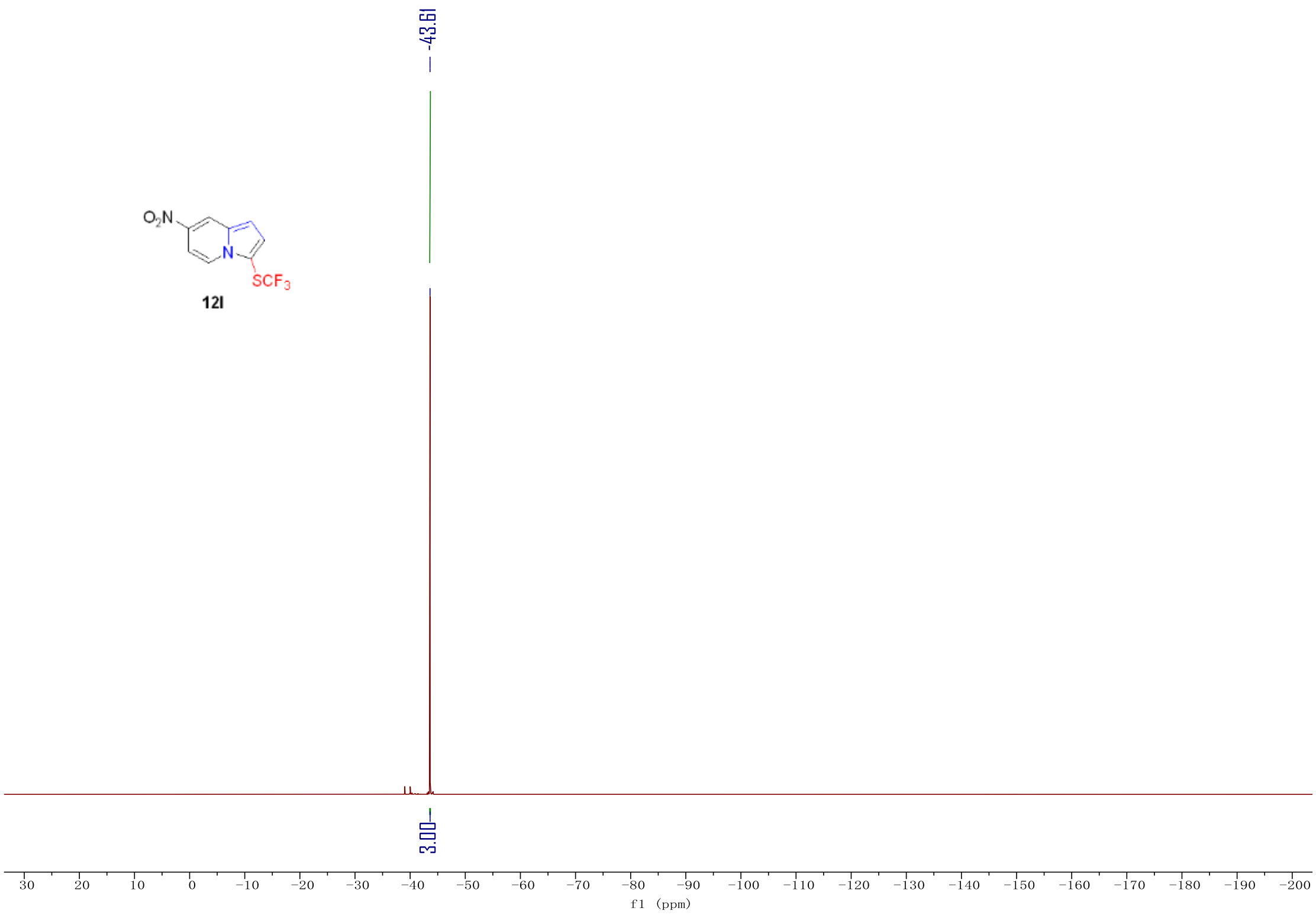

^{13}**C NMR** Spectrum of **12l** (101 MHz, $CDCl_3$)

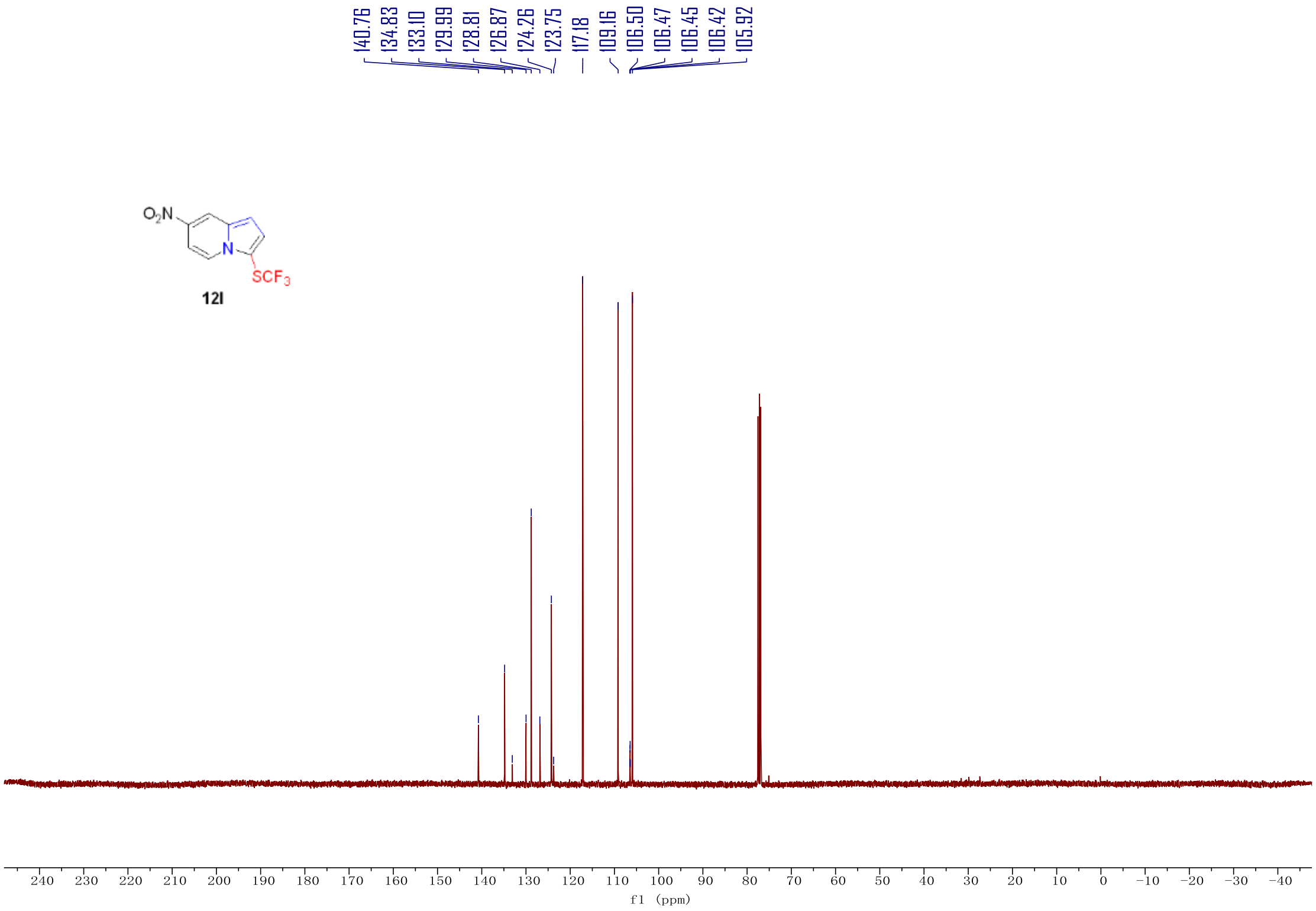

^{1}**H NMR** Spectrum of **12m** (400 MHz, $CDCl_3$)

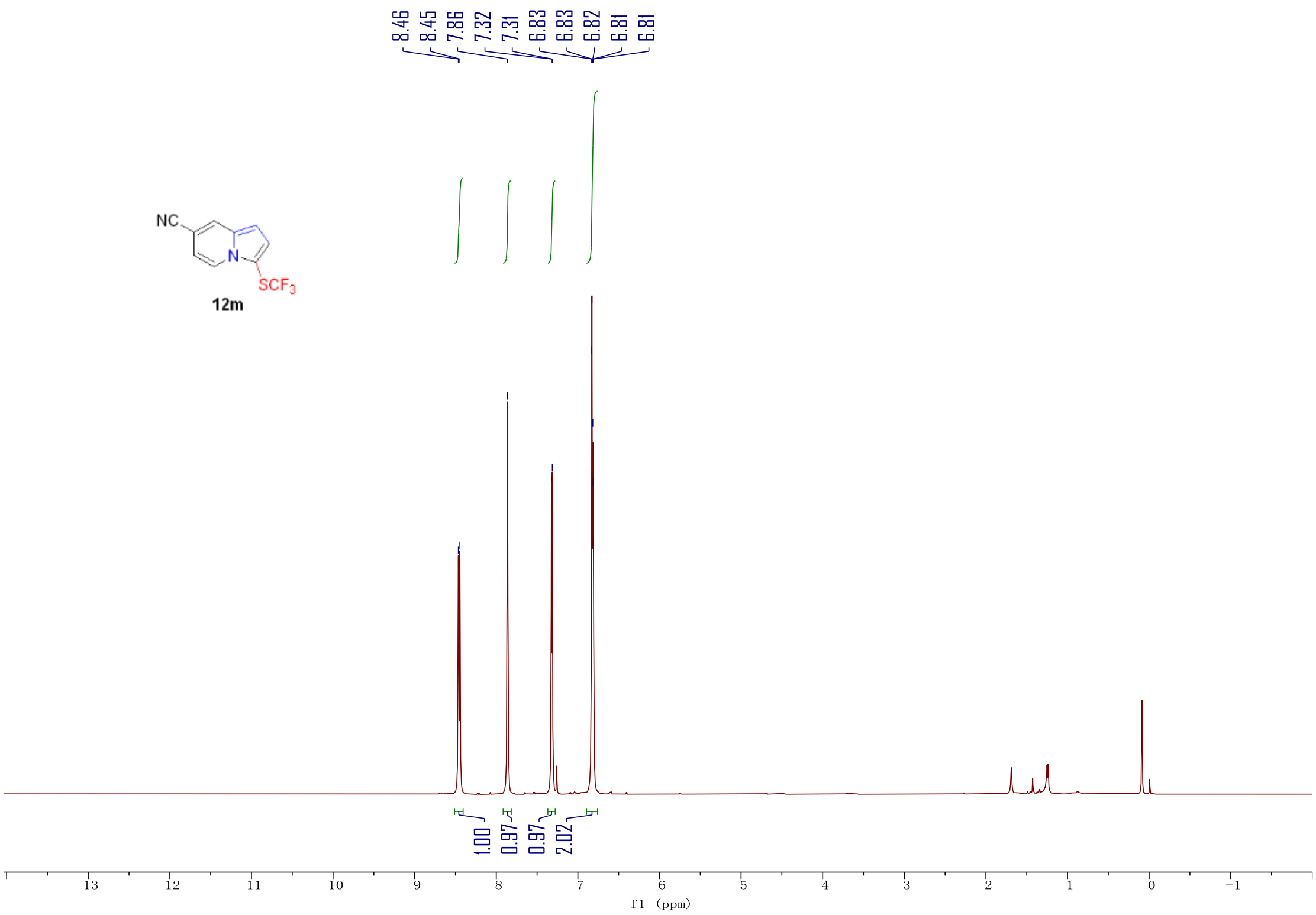

^{19}**F NMR** Spectrum of **12m** (376 MHz, $CDCl_3$)

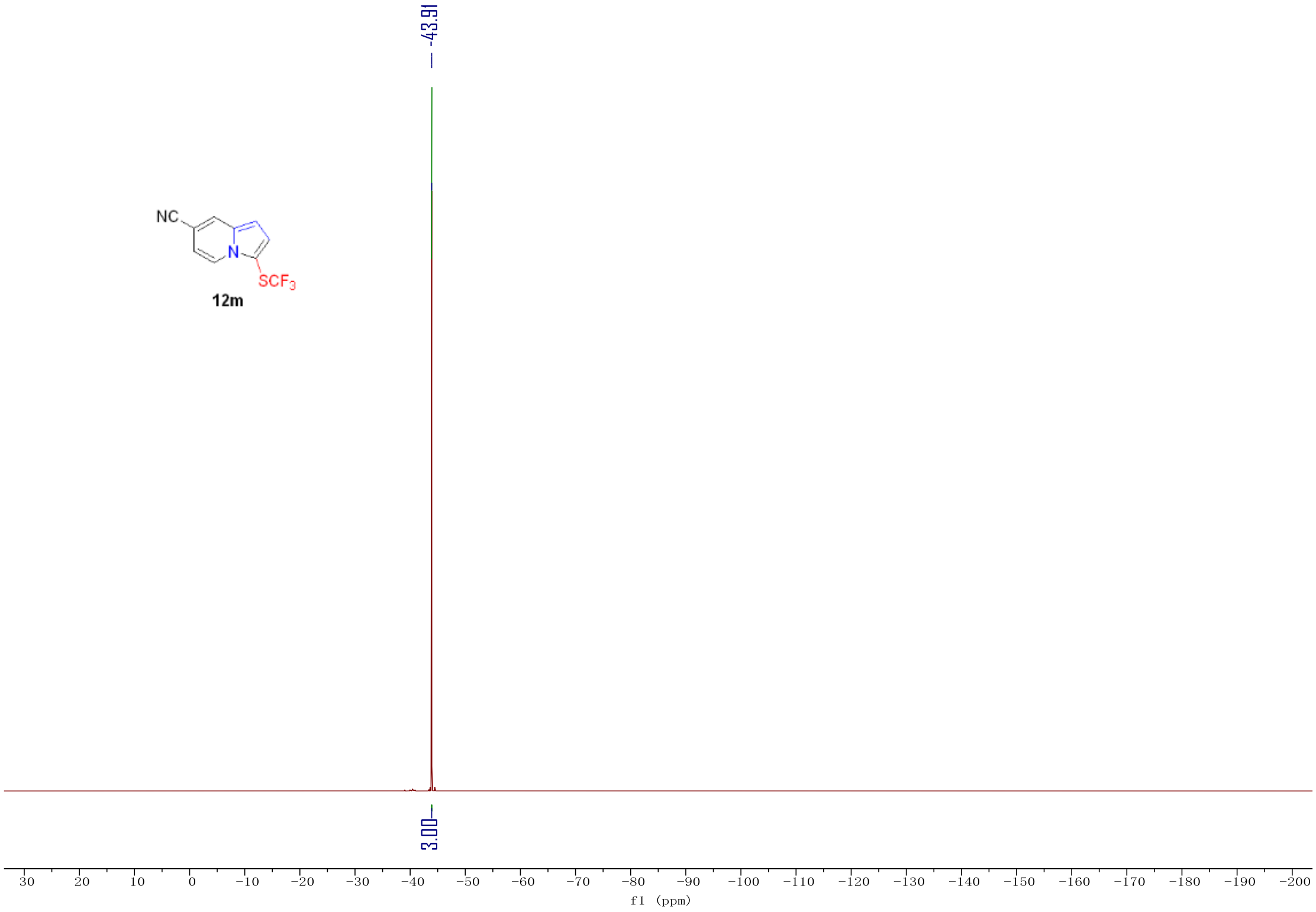

^{13}**C NMR** Spectrum of **12m** (101 MHz, $CDCl_3$)

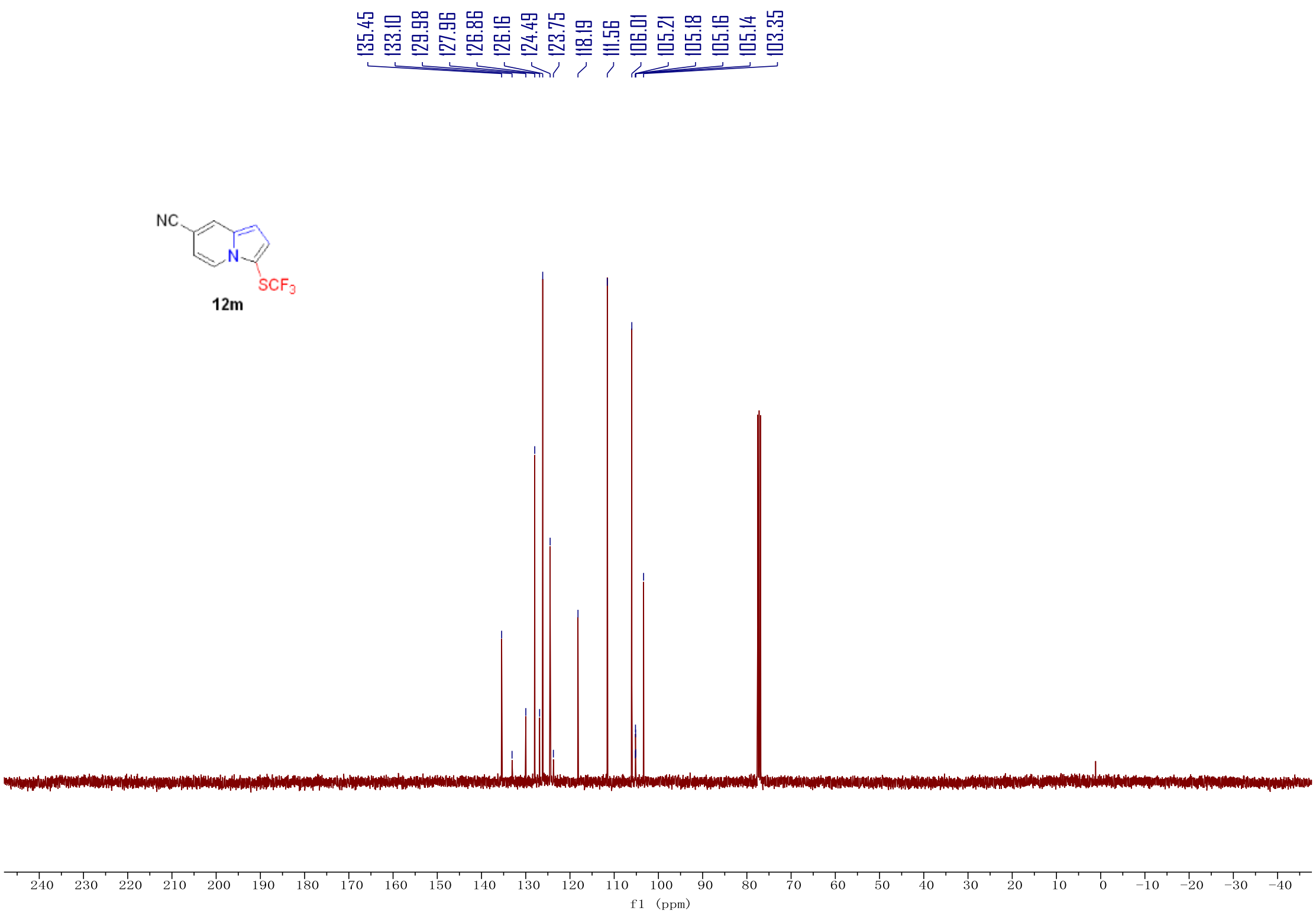

^{1}H NMR Spectrum of **12n** (400 MHz, $CDCl_3$)

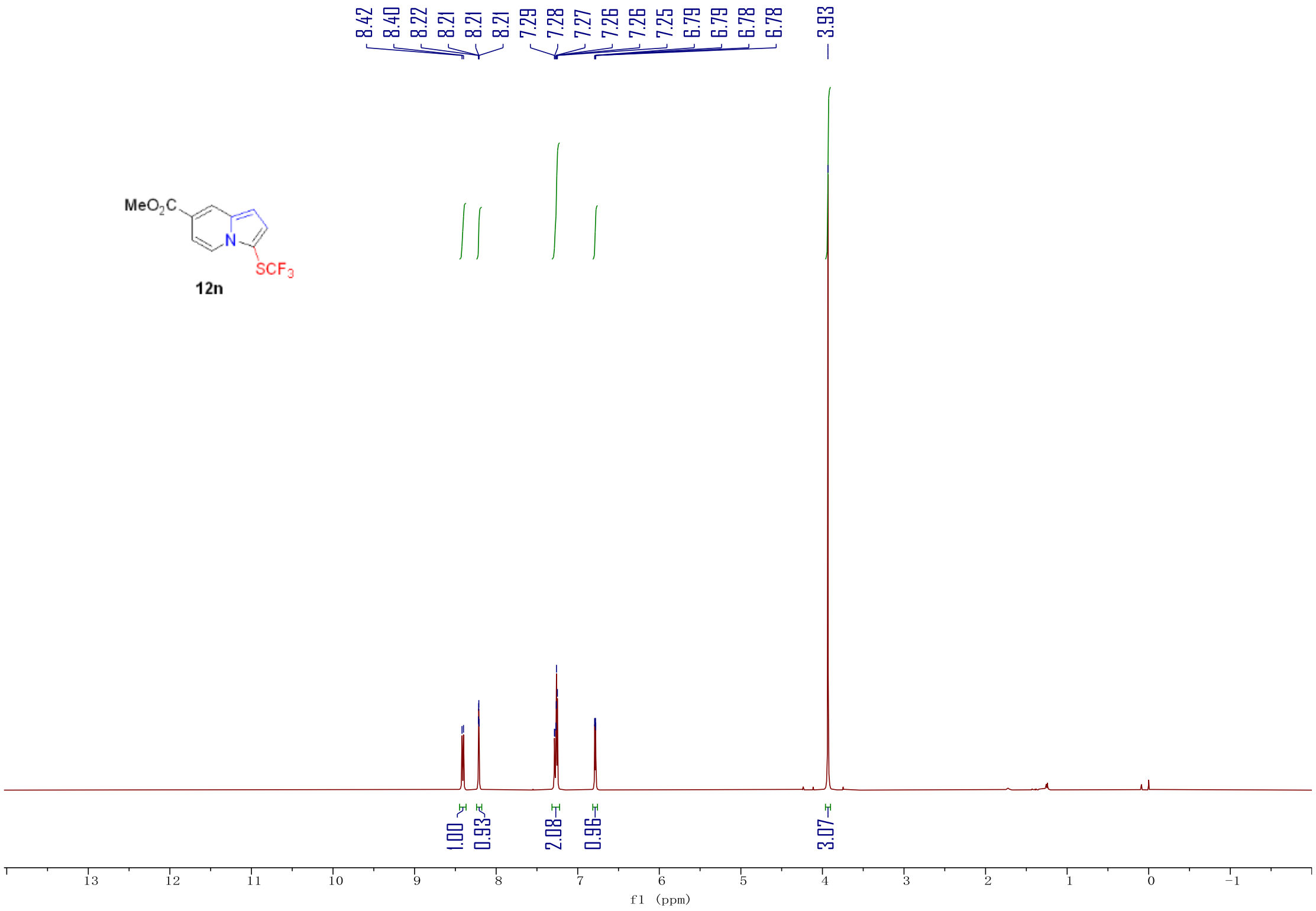

^{19}F NMR Spectrum of **12n** (376 MHz, $CDCl_3$)

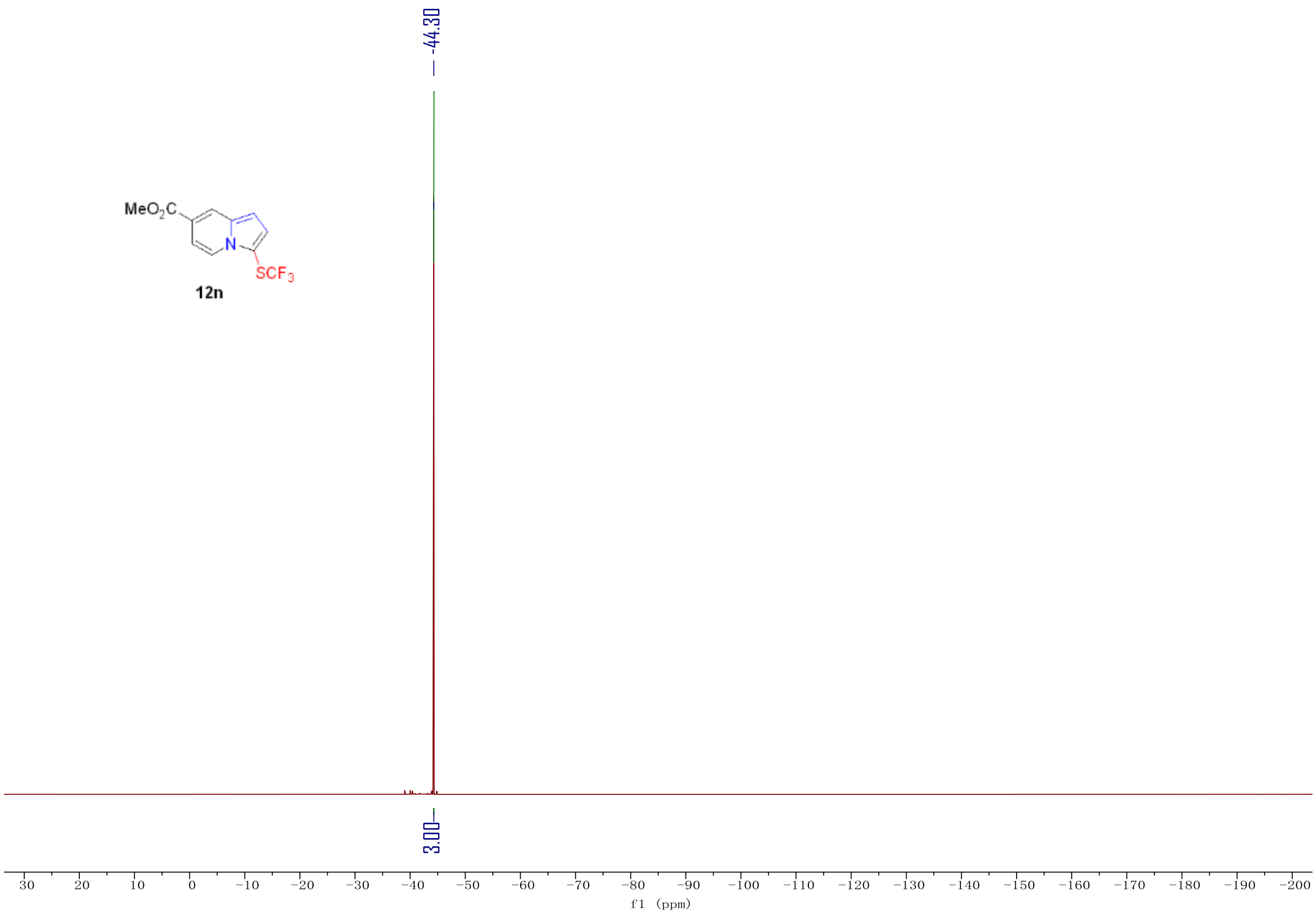

^{13}C NMR Spectrum of **12n** (101 MHz, $CDCl_3$)

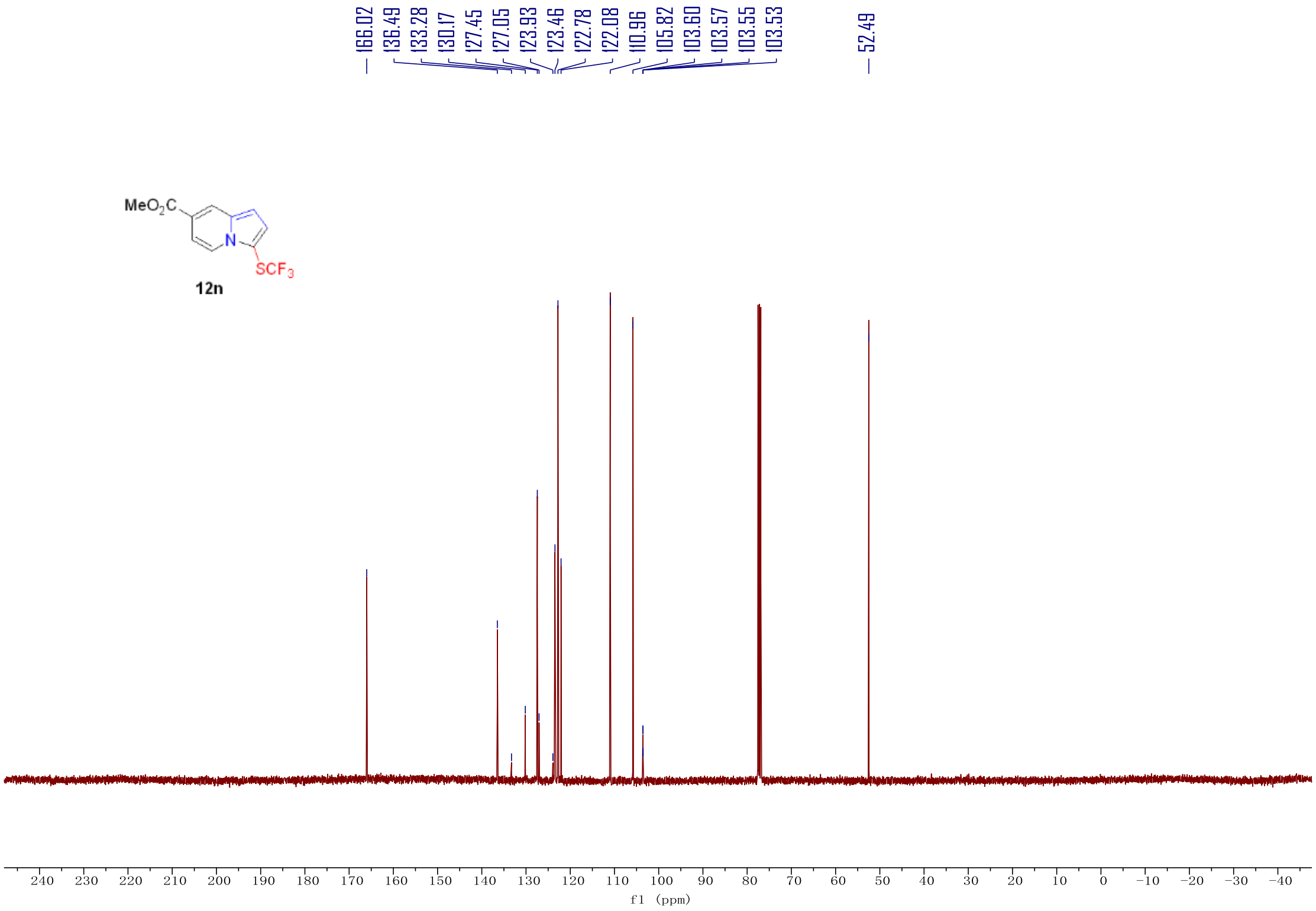

^{1}H NMR Spectrum of **12o** (400 MHz, $CDCl_3$)

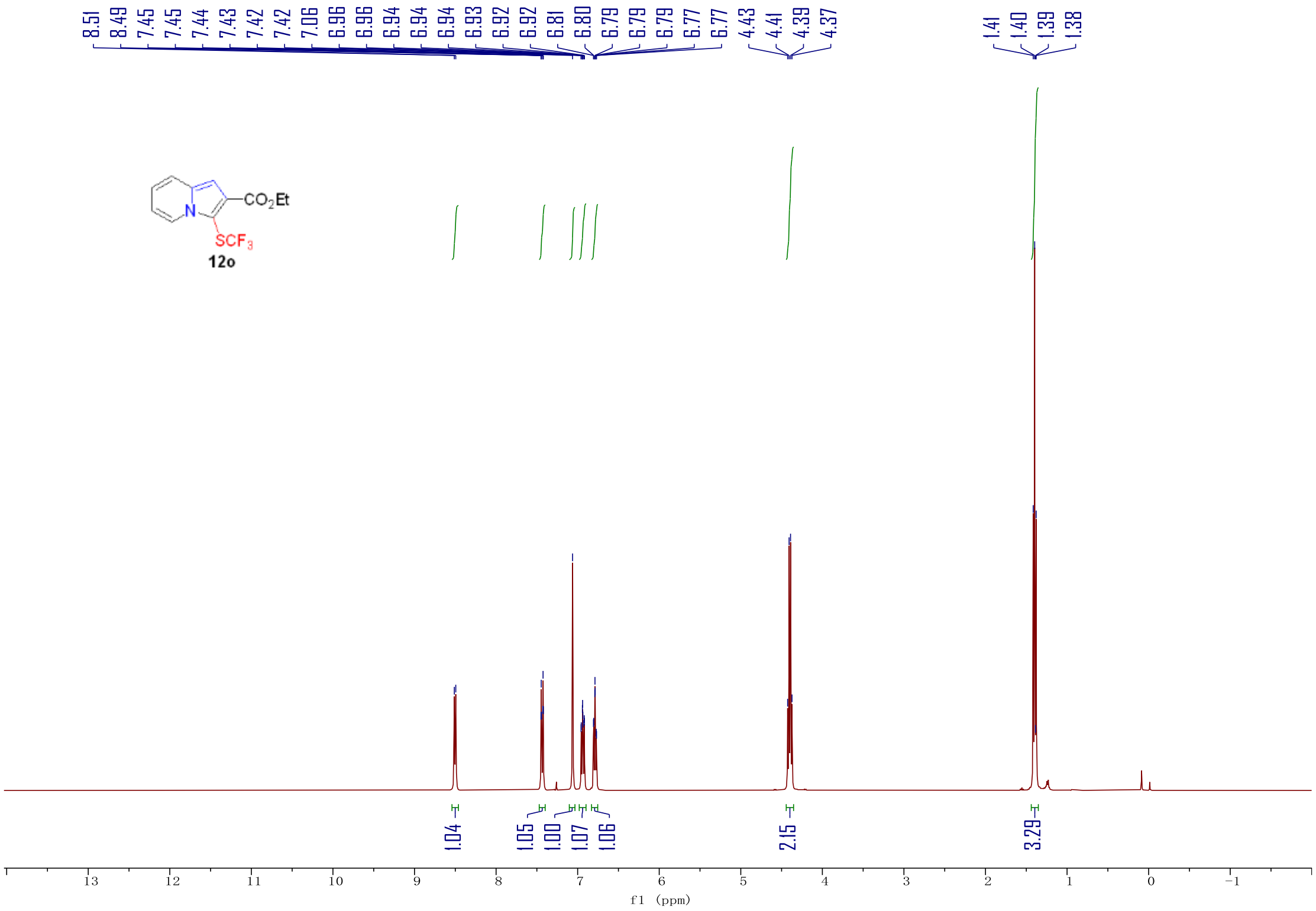

^{19}F NMR Spectrum of **12o** (376 MHz, $CDCl_3$)

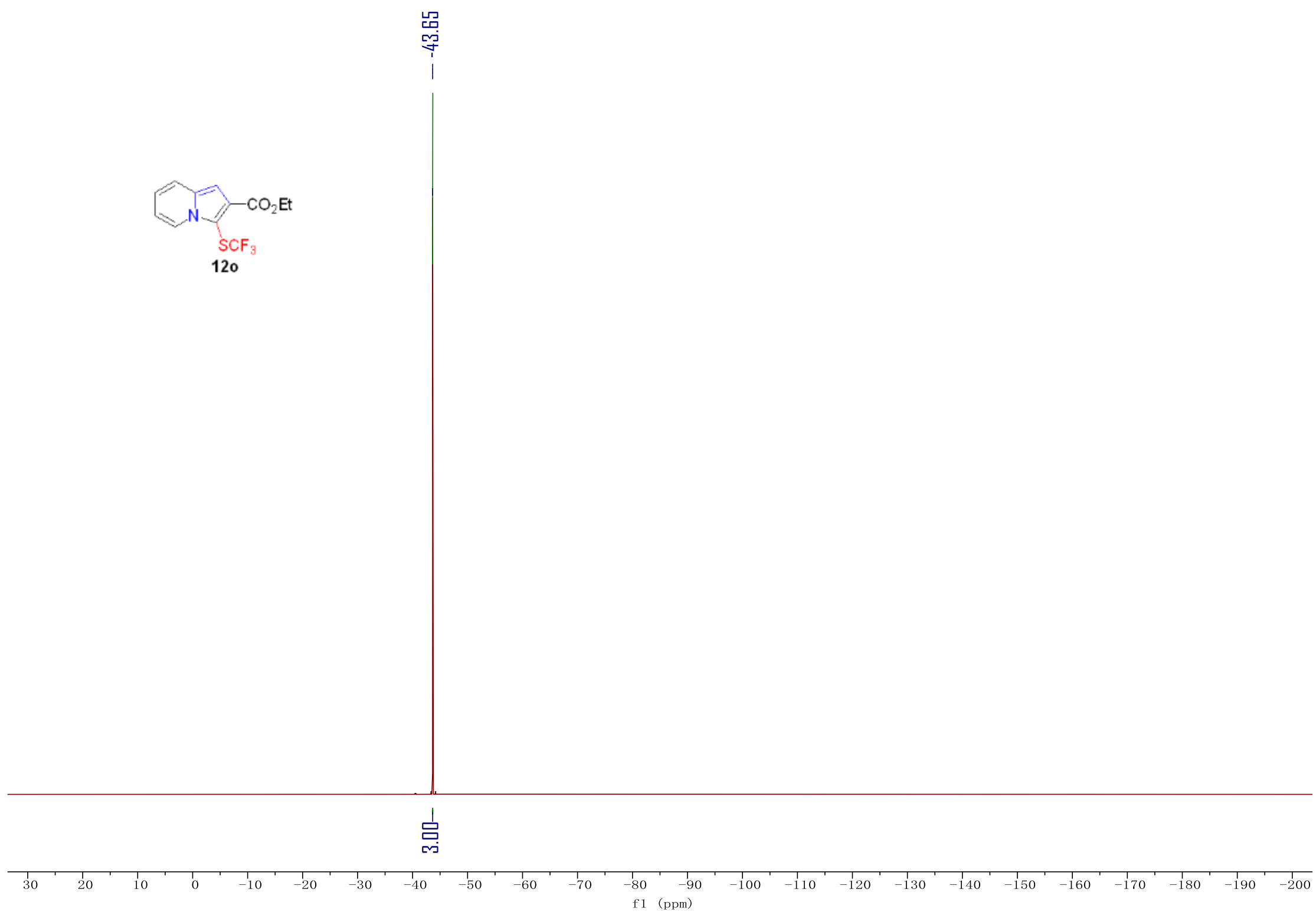

^{13}C NMR Spectrum of **12o** (101 MHz, $CDCl_3$)

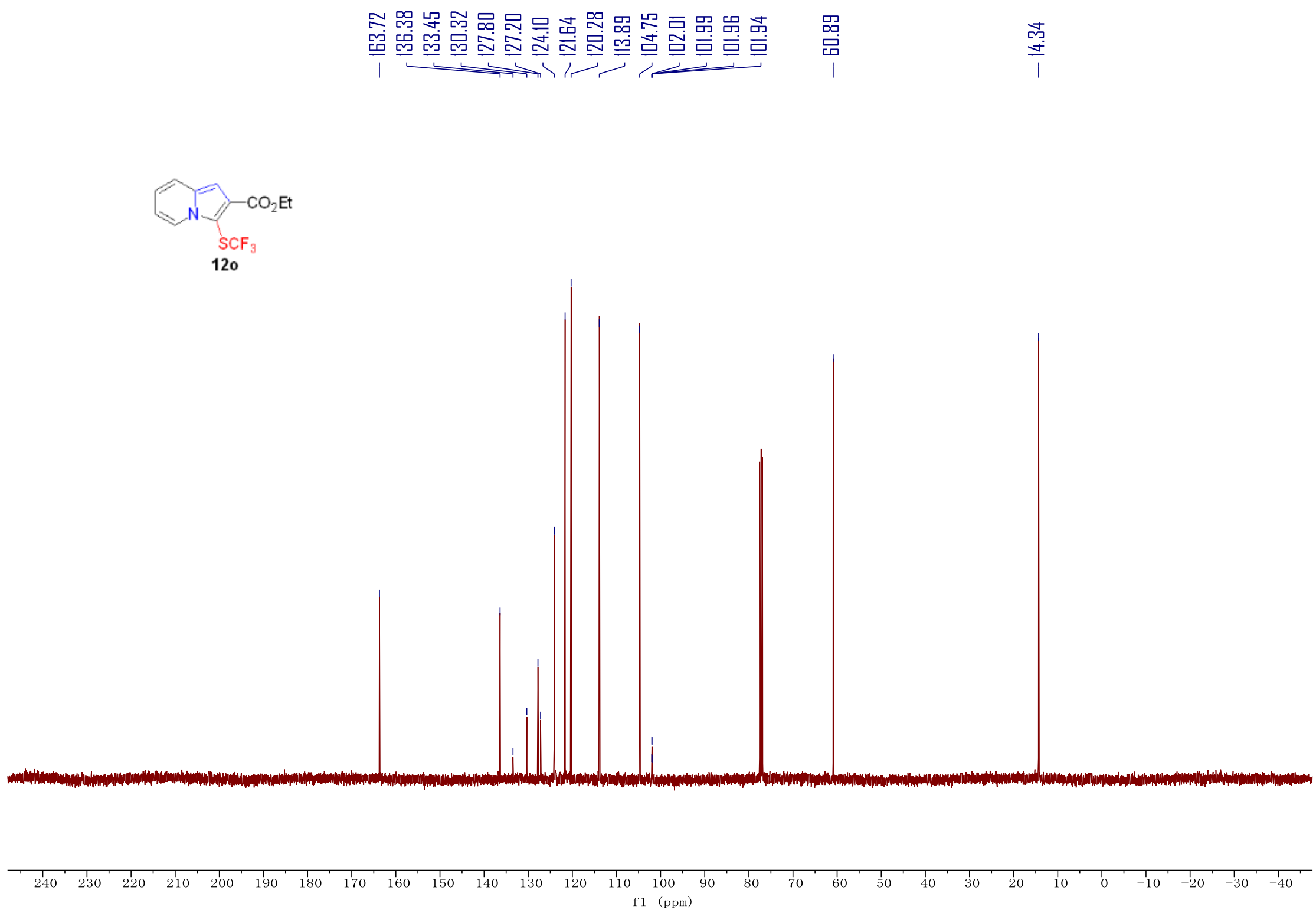

^{1}H NMR Spectrum of **12p** (400 MHz, $CDCl_3$)

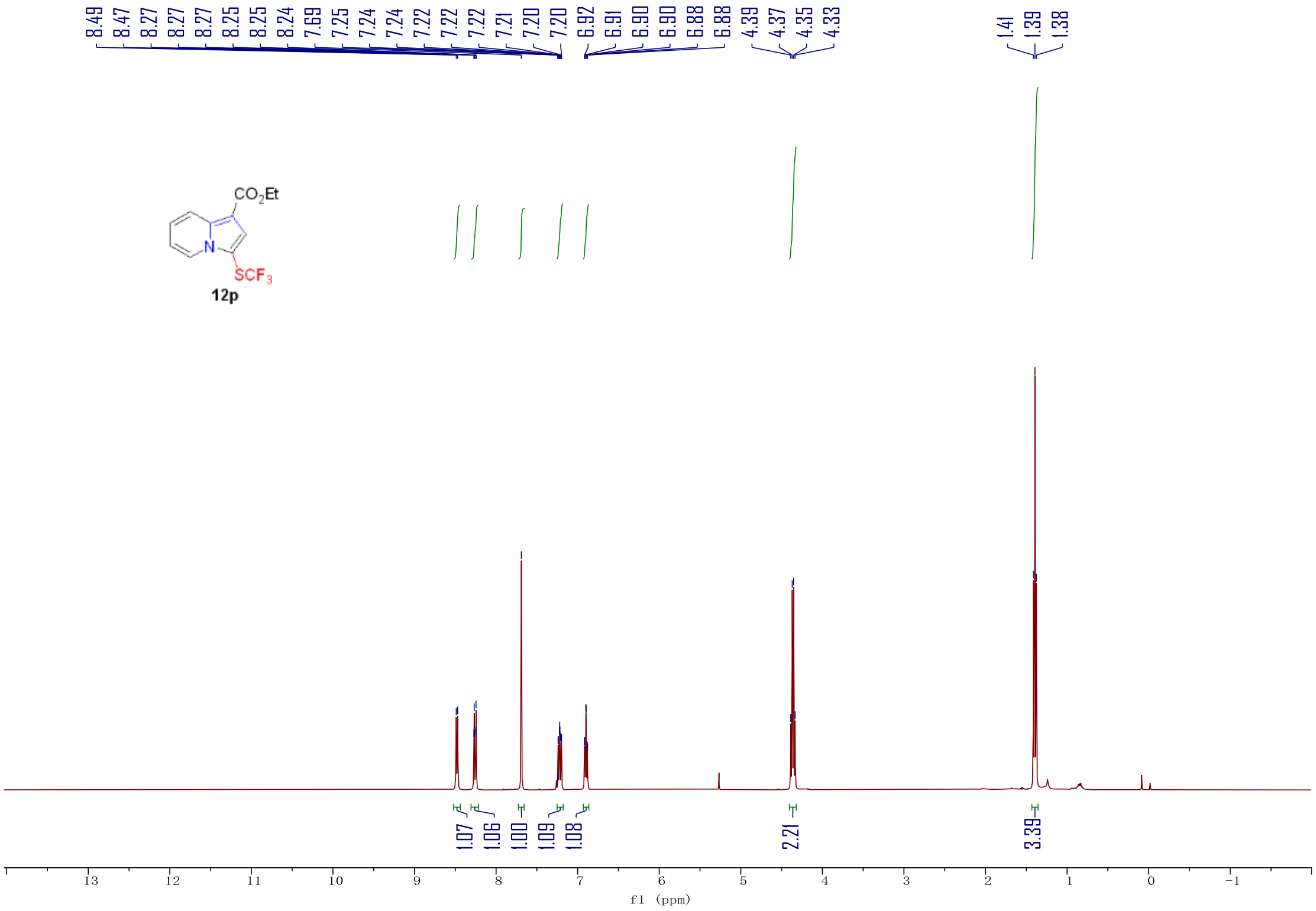

^{19}F NMR Spectrum of **12p** (376 MHz, $CDCl_3$)

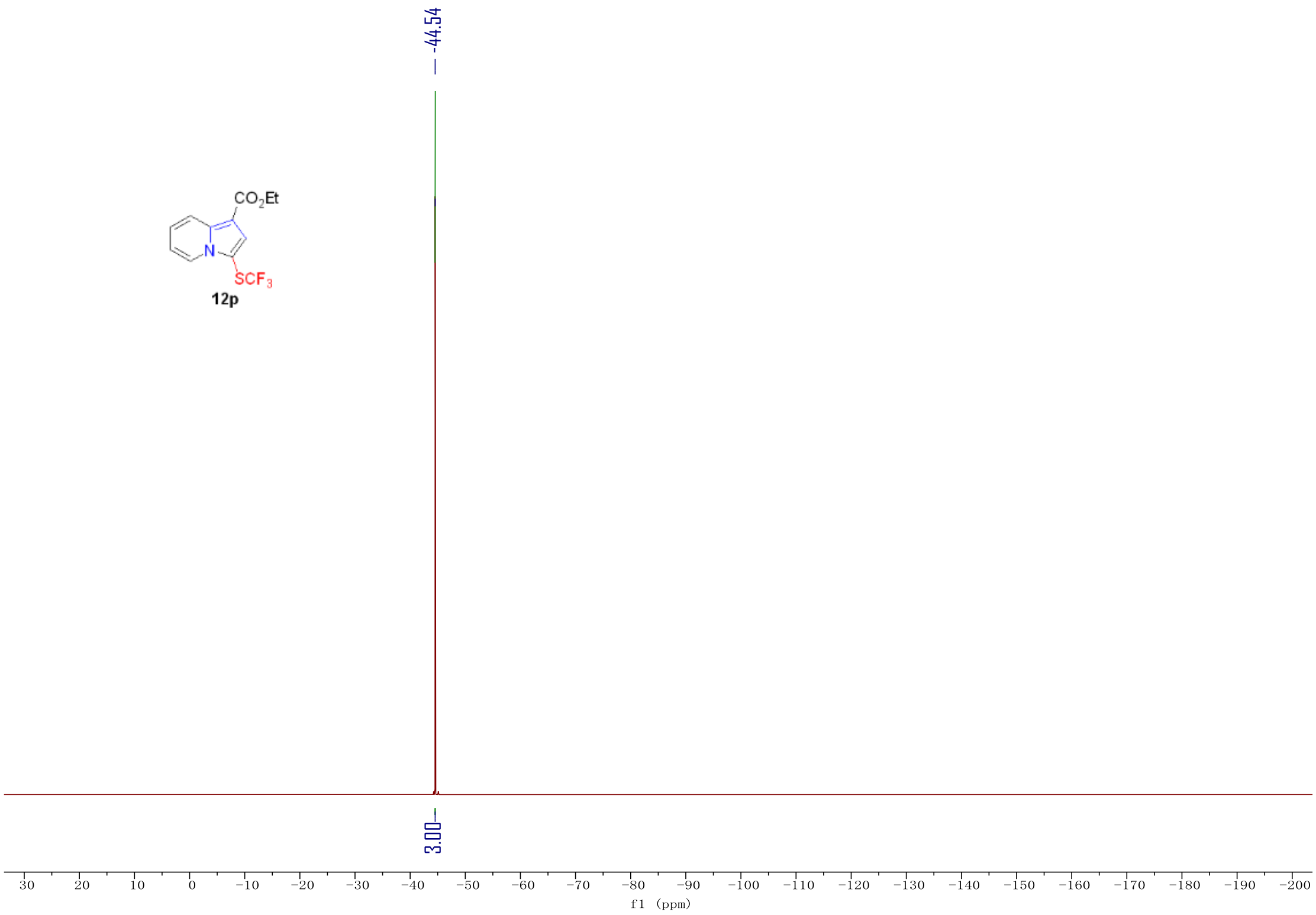

^{13}C NMR Spectrum of **12p** (101 MHz, $CDCl_3$)

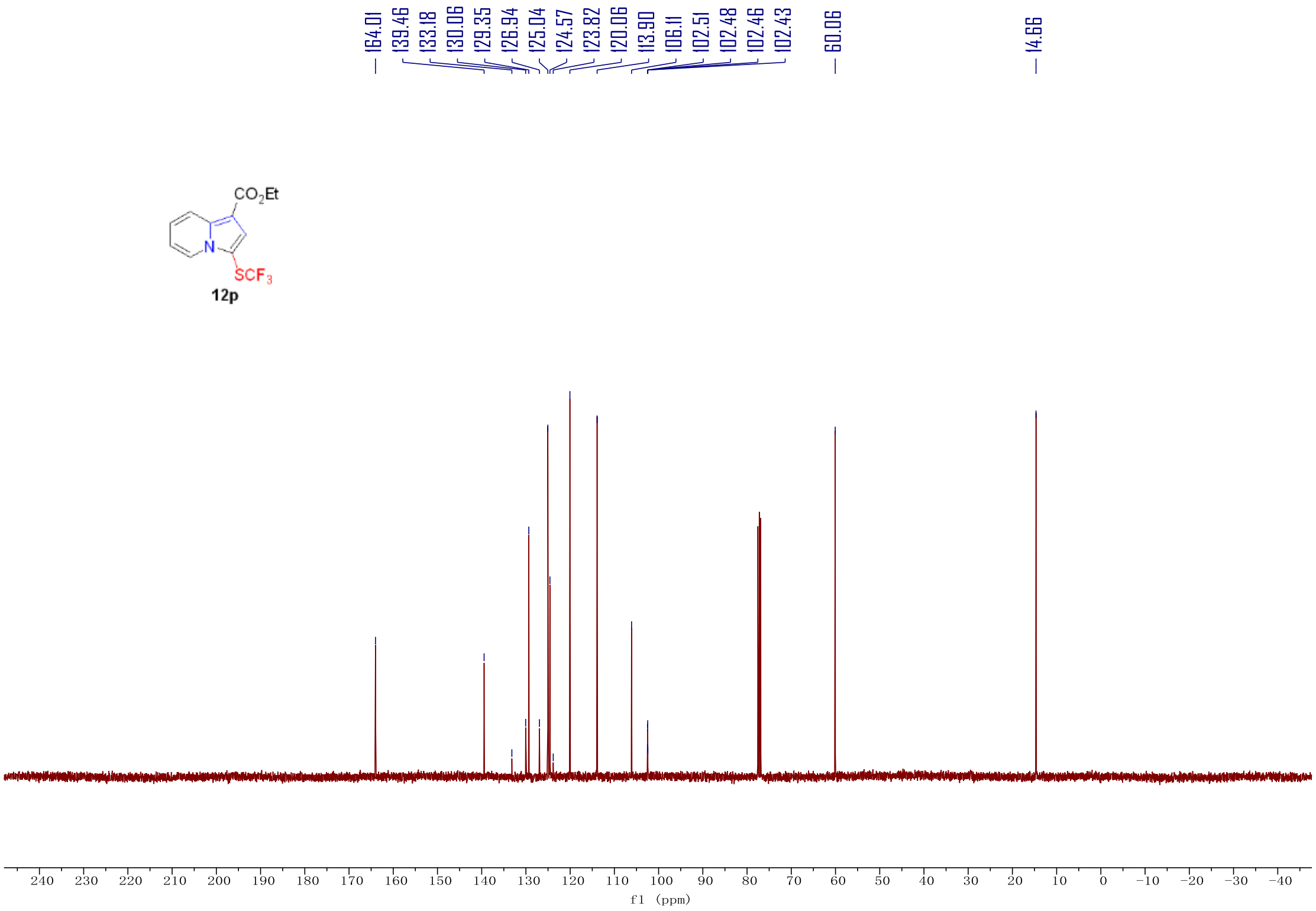

^{1}H NMR Spectrum of **12q** (400 MHz, $CDCl_3$)

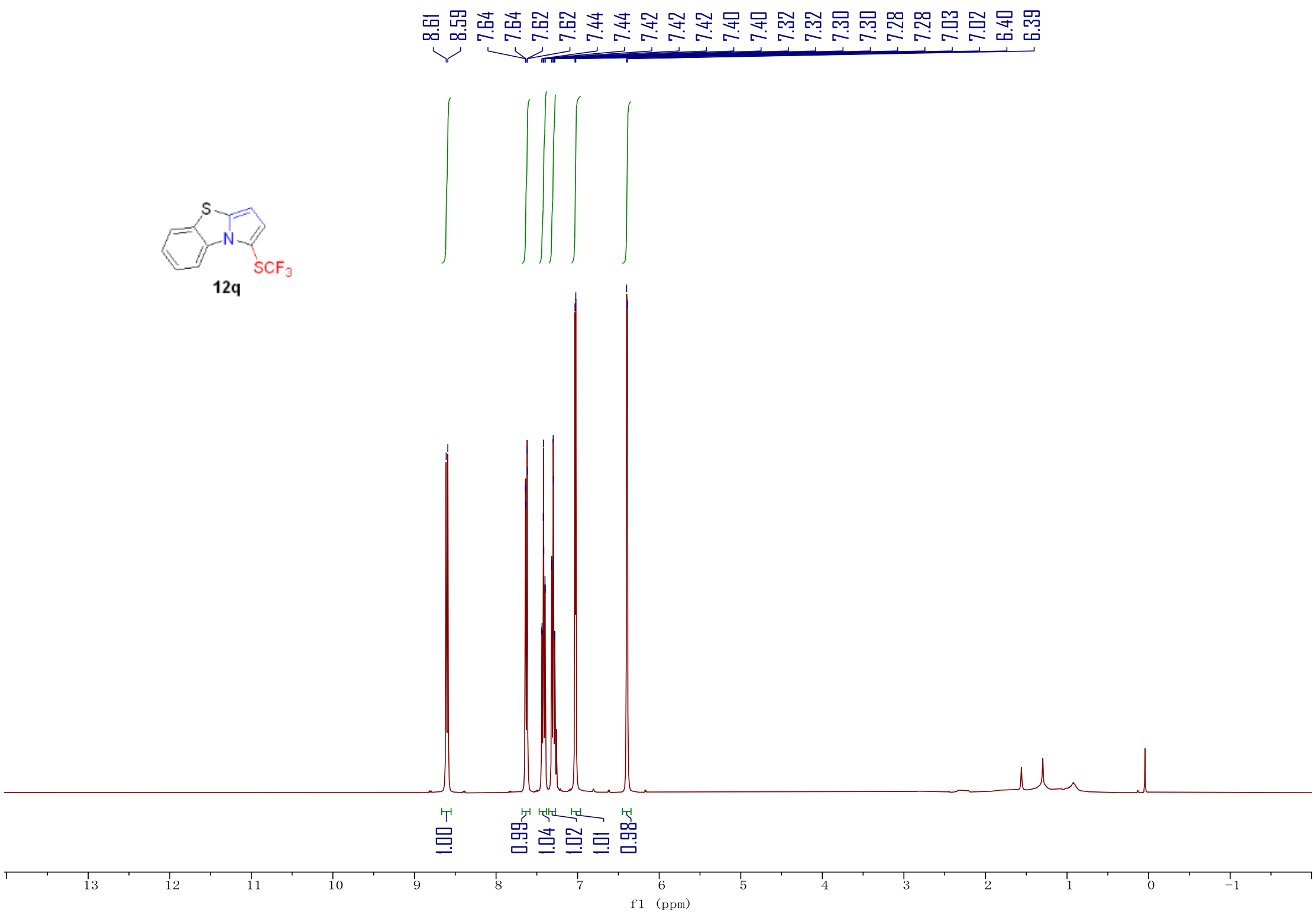

^{19}F **NMR** Spectrum of **12q** (376 MHz, $CDCl_3$)

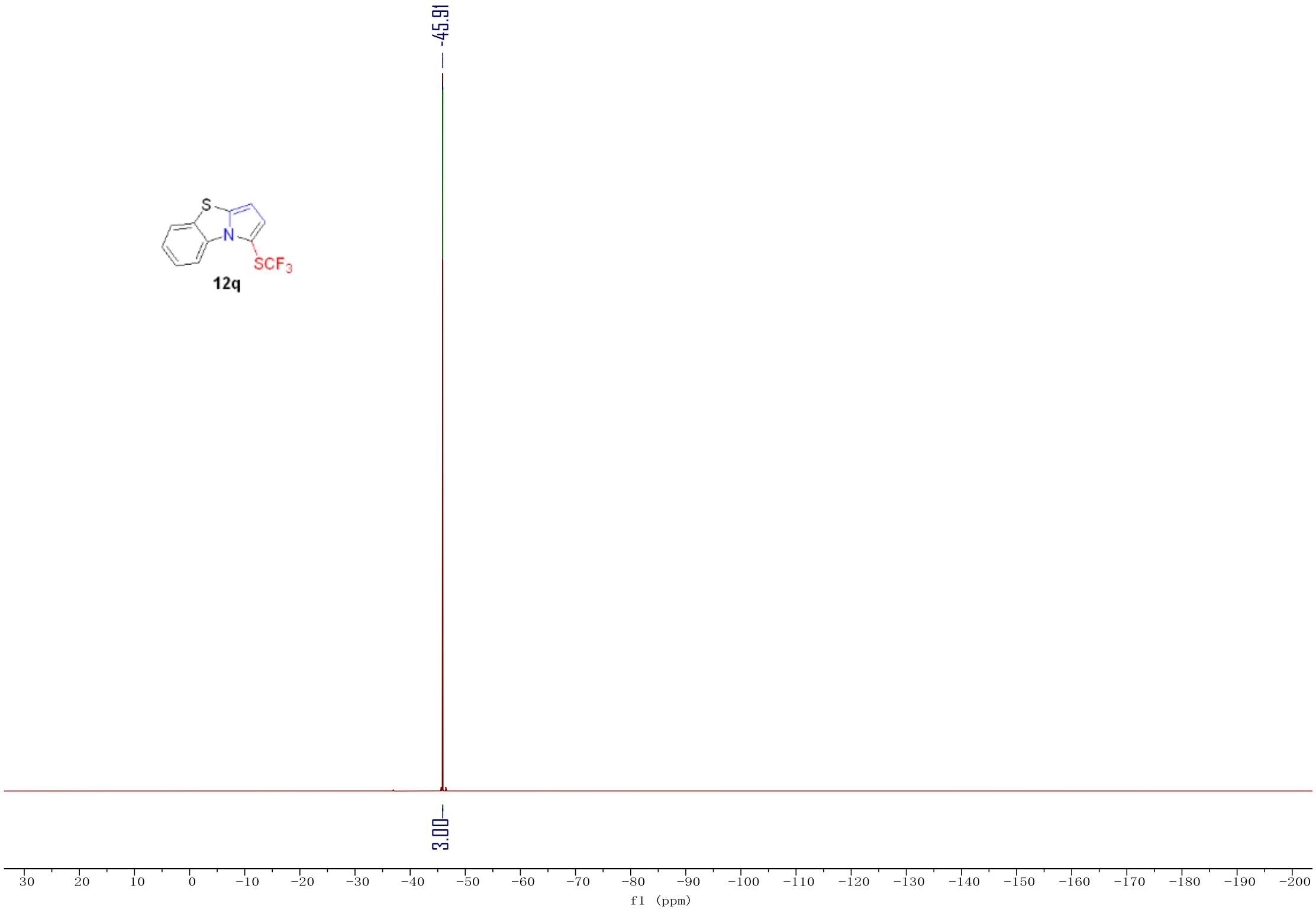

^{13}C **NMR** Spectrum of **12q** (101 MHz, $CDCl_3$)

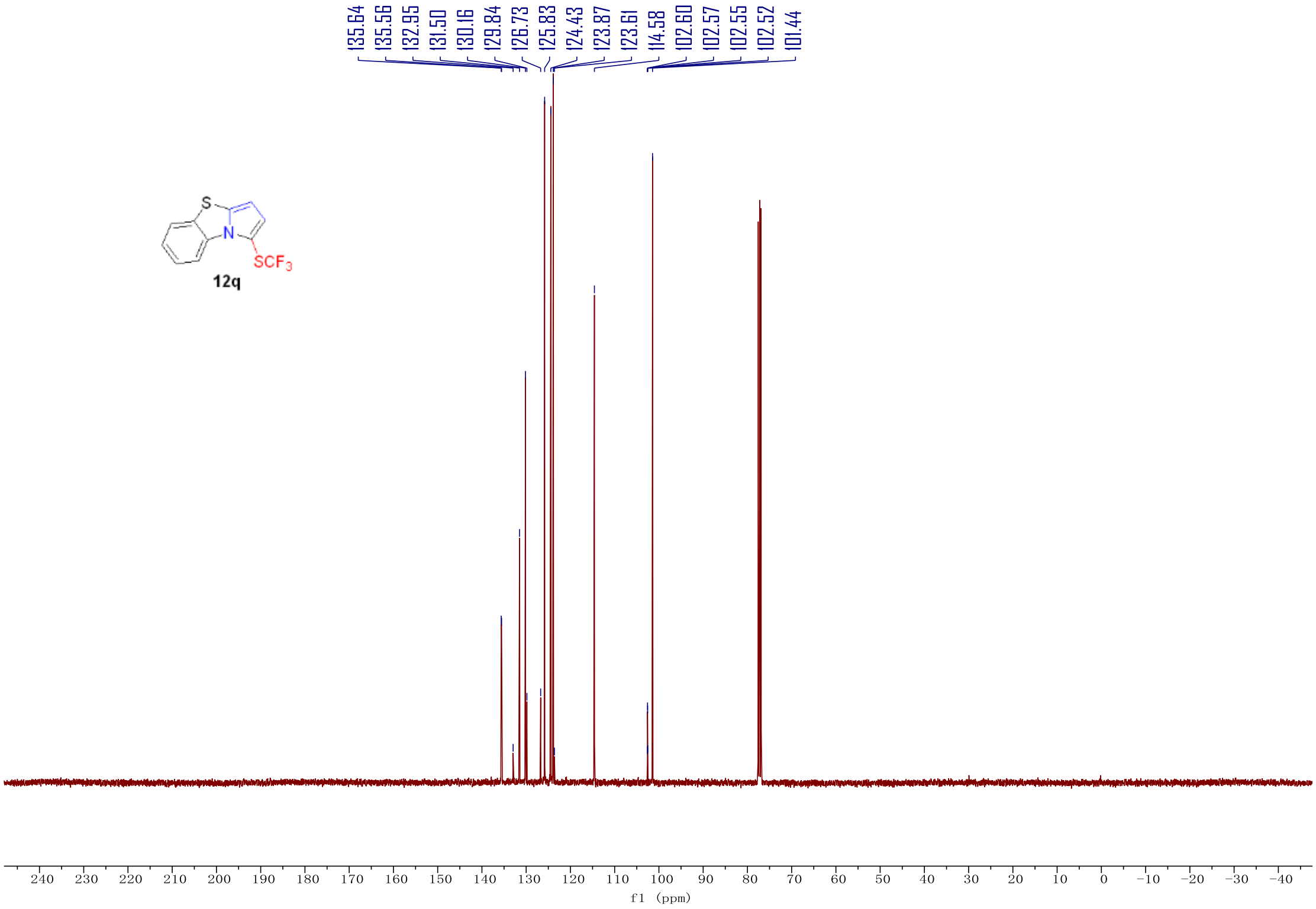

^{1}H NMR Spectrum of **12r** (400 MHz, $CDCl_3$)

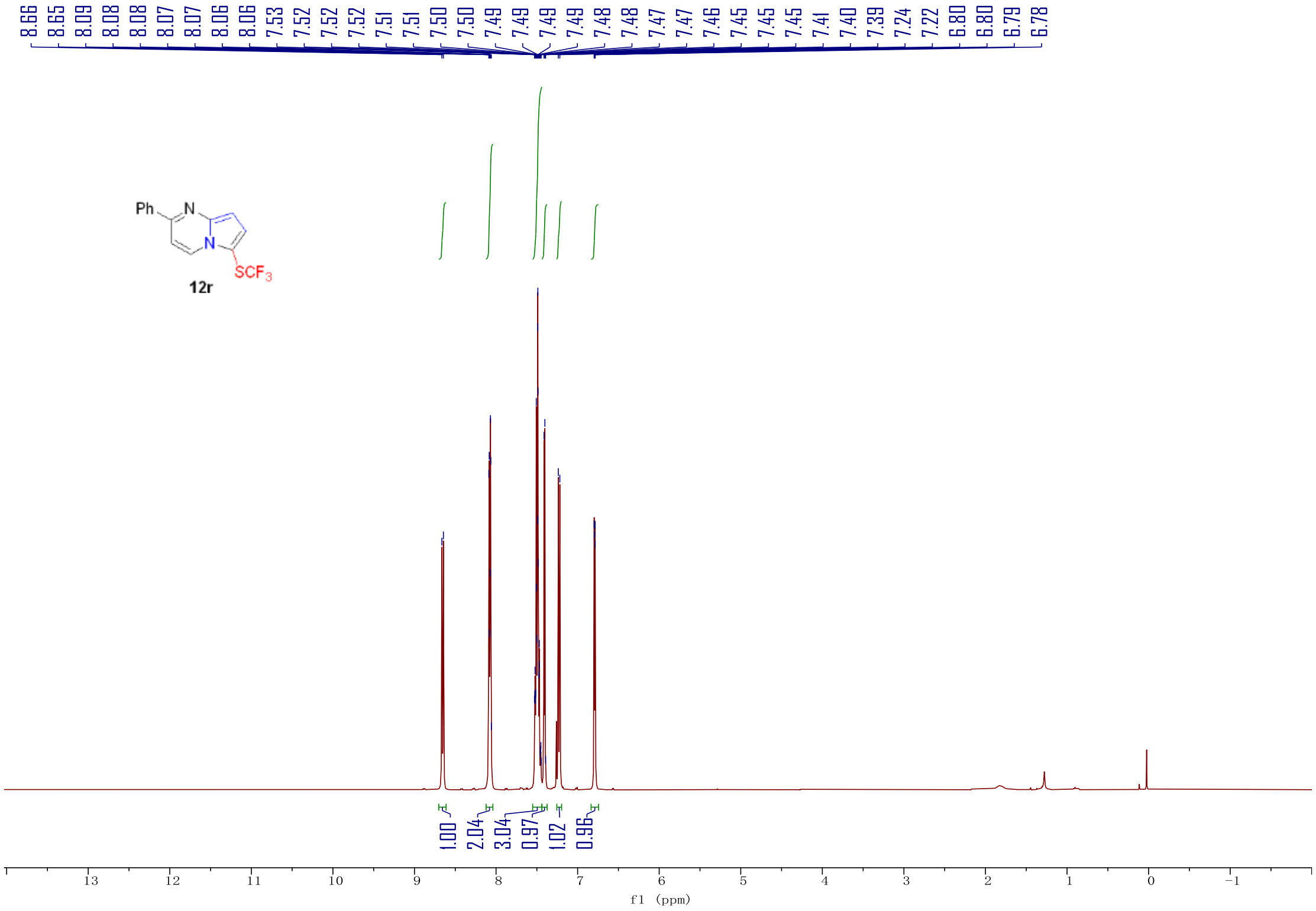

^{19}F NMR Spectrum of **12r** (376 MHz, $CDCl_3$)

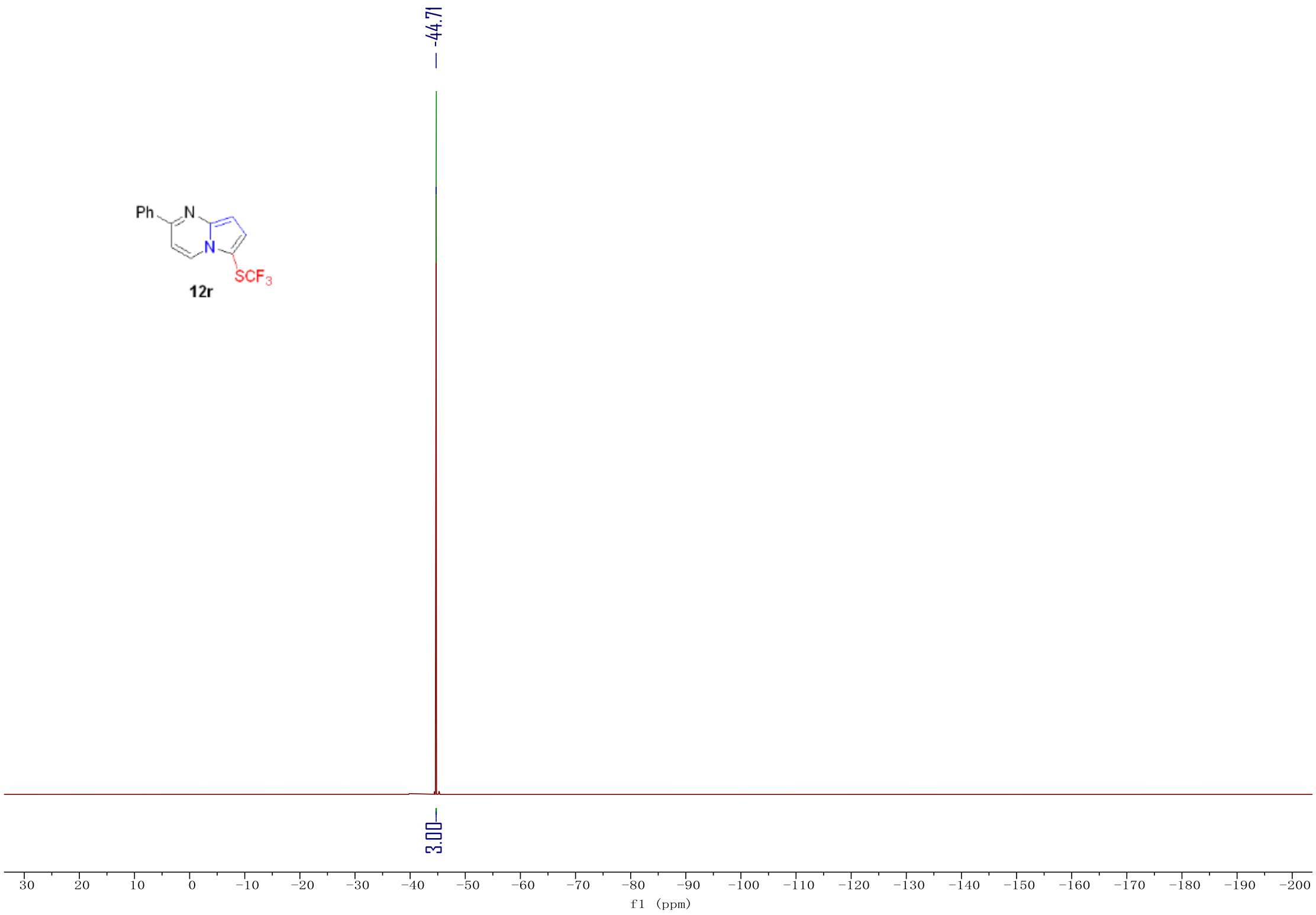

^{13}C NMR Spectrum of **12r** (101 MHz, $CDCl_3$)

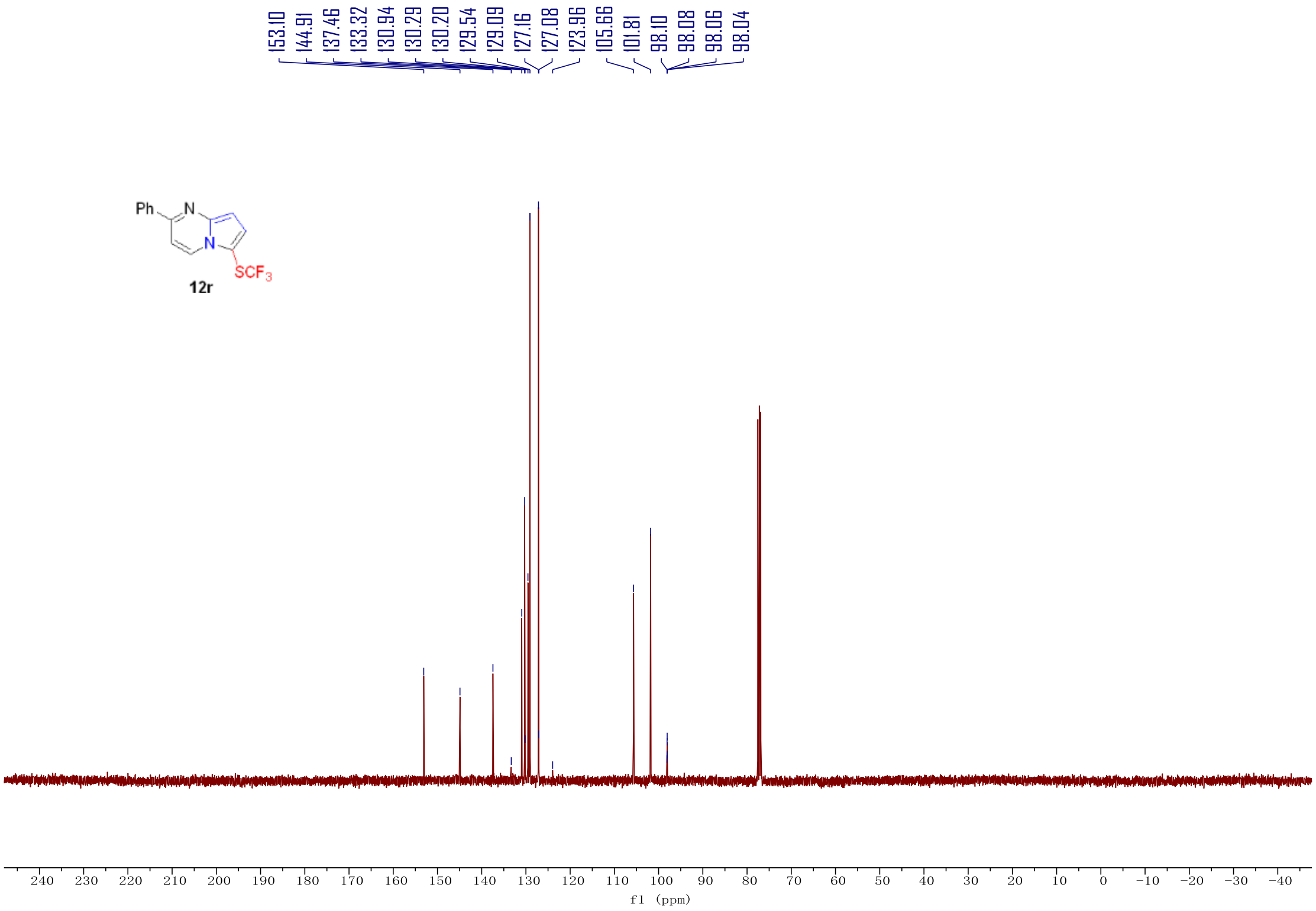

^{1}H NMR Spectrum of **12t** (400 MHz, $CDCl_3$)

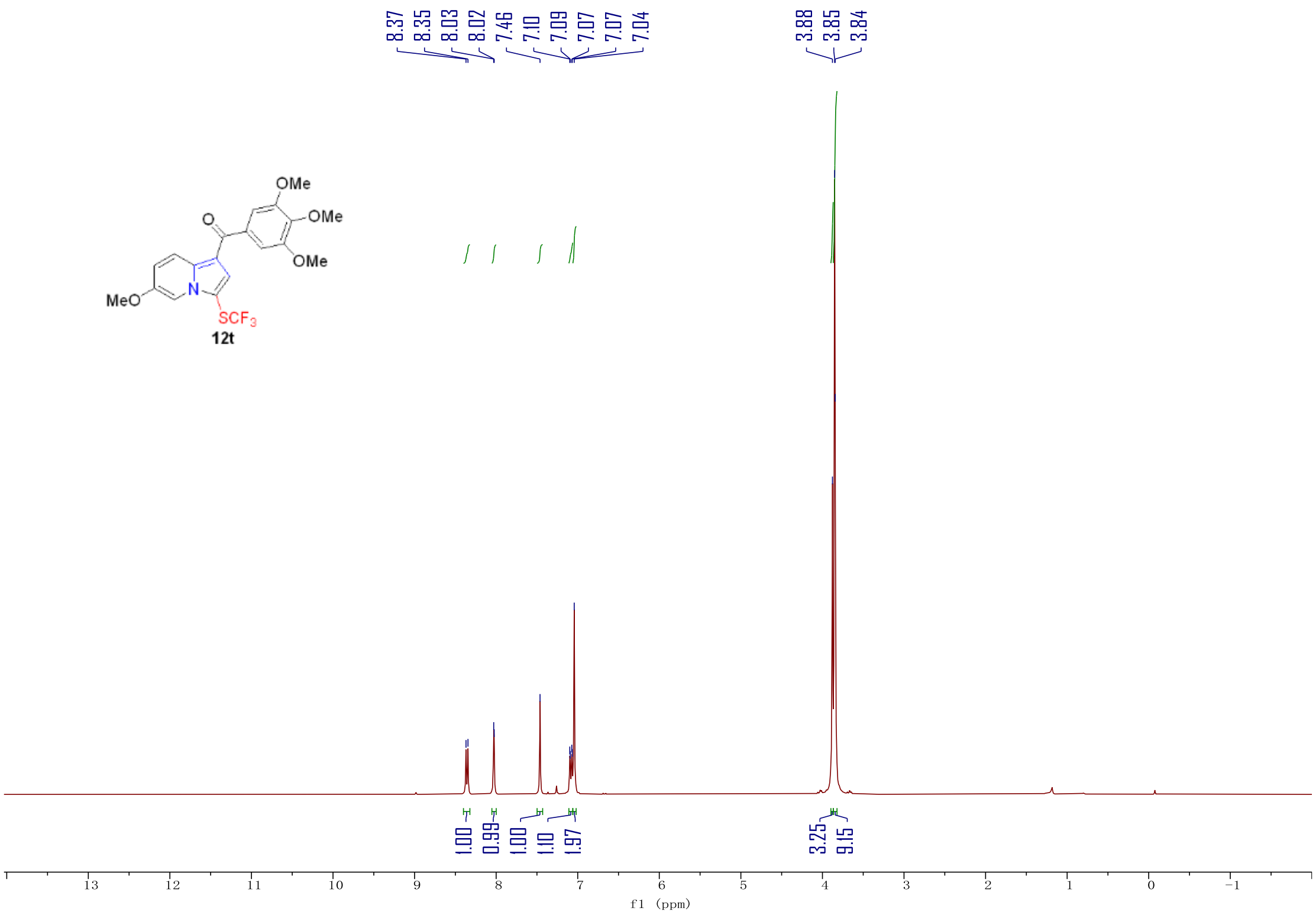

^{19}**F NMR** Spectrum of **12t** (376 MHz, $CDCl_3$)

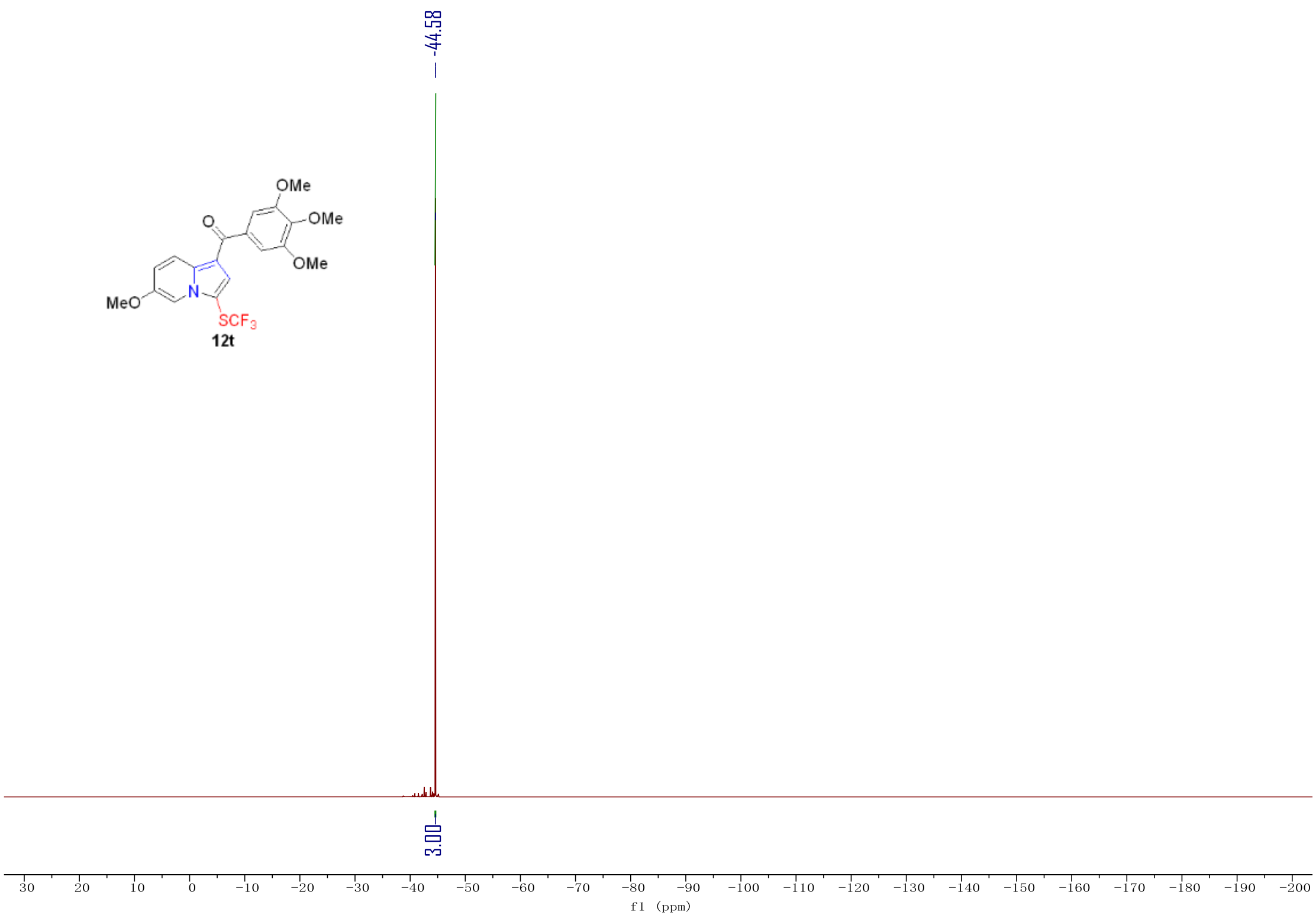

^{13}**C NMR** Spectrum of **12t** (101 MHz, $CDCl_3$)

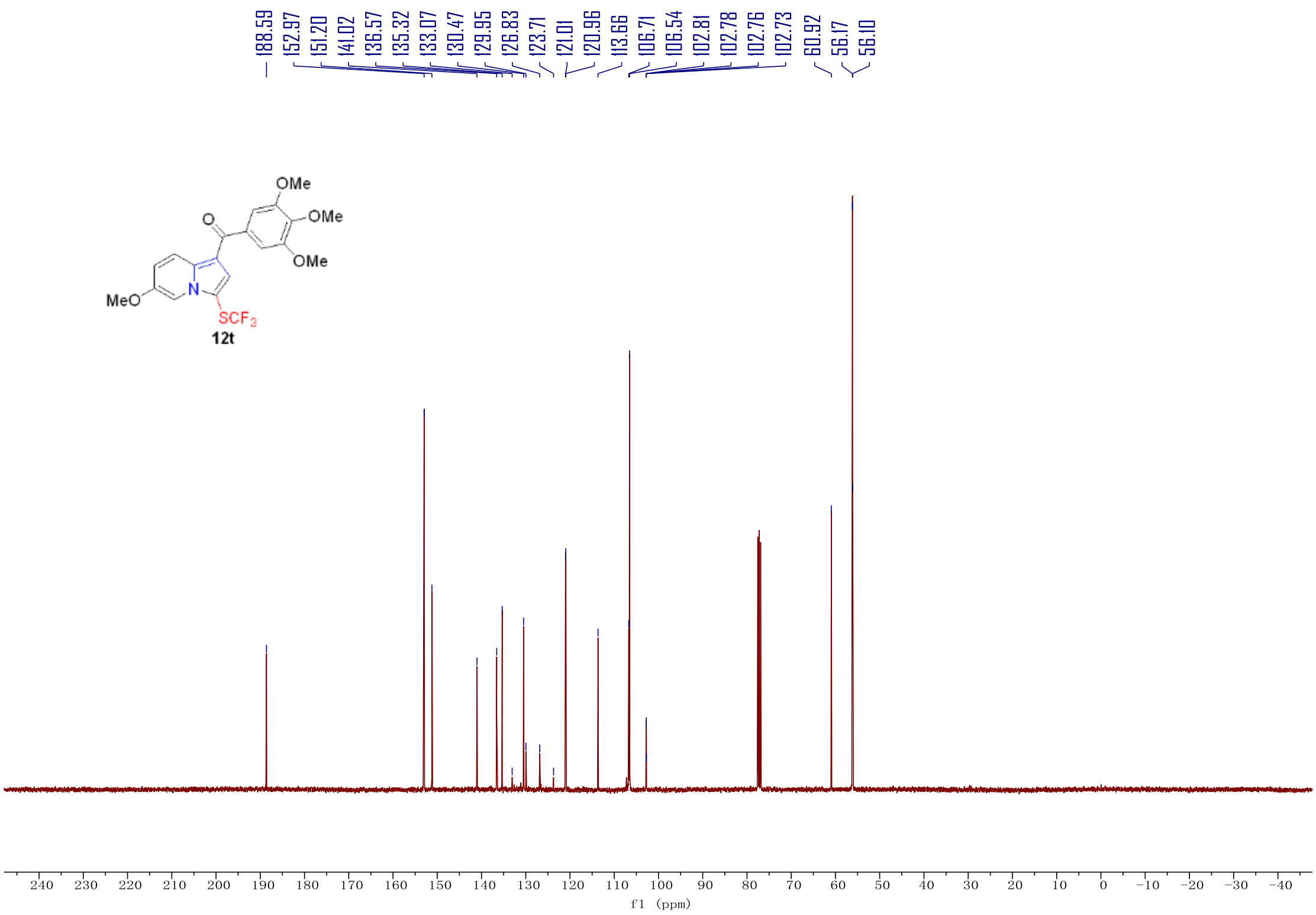

References

[1] M. J. Frisch, G. W. Trucks, H. B. Schlegel, G. E. Scuseria, M. A. Robb, J. R. Cheeseman, G. Scalmani, V. Barone, G. A. Petersson, H. Nakatsuji, X. Li, M. Caricato, A. Marenich, J. Bloino, B. G. Janesko, R. Gomperts, B. Mennucci, H. P. Hratchian, J. V. Ortiz, A. F. Izmaylov, J. L. Sonnenberg, D. Williams-Young, F. Ding, F. Lipparini, F. Egidi, J. Goings, B. Peng, A. Petrone, T. Henderson, D. Ranasinghe, V. G. Zakrzewski, J. Gao, N. Rega, G. Zheng, W. Liang, M. Hada, M. Ehara, K. Toyota, R. Fukuda, J. Hasegawa, M. Ishida, T. Nakajima, Y. Honda, O. Kitao, H. Nakai, T. Vreven, K. Throssell, J. A. Montgomery Jr., J. E. Peralta, F. Ogliaro, M. Bearpark, J. J. Heyd, E. Brothers, K. N. Kudin, V. N. Staroverov, T. Keith, R. Kobayashi, J. Normand, K. Raghavachari, A. Rendell, J. C. Burant, S. S. Iyengar, J. Tomasi, M. Cossi, J. M. Millam, M. Klene, C. Adamo, R. Cammi, J. W. Ochterski, R. L. Martin, K. Morokuma, O. Farkas, J. B. Foresman, D. J. Fox, *Gaussian 09, Revision D.01*, Gaussian, Inc., Wallingford CT, **2016**.

[2] C. Y. Legault, Université de Sherbrooke, CYLview20, **2020**, http://www.cylview.org.

[3] C. D. Díaz-Oviedo, R. Maji, B. List, *J. Am. Chem. Soc.* **2021**, *143*, 20598-20604.

[4] H. Pang, Y. Wang, F. Gallou, B. H. Lipshutz, *J. Am. Chem. Soc.* **2019**, *141*, 17117-17124.

[5] V. H. Thorat, H.-Y. Chao, P.-H. Yang, J.-C. Hsieh, *Eur. J. Org. Chem.* **2025**, *28*, e202401194.

[6] H. Guo, S. Qiu, P. Xu, *Angew. Chem. Int. Ed.* **2024**, *63*, e202317104.

[7] M. Yamaguchi, S. Fujiwara, K. Manabe, *Org. Lett.* **2019**, *21*, 6972-6977.

[8] H. Jin, F. Zhou, Z. Xiang, L. Chen, G. Liang, P. J. Walsh, J. Li, *Adv. Synth. Catal.* **2024**, *366*, 942-947.

[9] A. Parodi, S. Battaglioli, Y. Liu, M. Monari, M. Marín-Luna, C. Silva-López, M. Bandini, *Chem. Commun.* **2019**, *55*, 9669-9672.

[10] H. Vaid, A. Sharma, P. Jamwal, P. Sharma, R. Gurubrahamam, *Org. Lett.* **2024**, *26*, 2135-2140.

[11] B. Gao, T. Jiang, R. Yang, Q. Yan, X. Liu, Q. Xie, X. Zhang, *J. Org. Chem.* **2022**, *87*, 7895-7904.

[12] S. Yan, J. Rao, C.-Y. Zhou, *Org. Lett.* **2020**, *22*, 9091-9096.

[13] T. Uchikura, F. Akutsu, T. Akiyama, *Chem. Commun.* **2025**, *61*, 6328-6331.

[14] S.-B. Zhang, X. Liu, M.-Y. Gao, Z.-B. Dong, *J. Org. Chem.* **2018**, *83*, 14933-14941.

[15] X.-B. Xu, Z.-H. Lin, Y. Liu, J. Guo, Y. He, *Org. Biomol. Chem.* **2017**, *15*, 2716-2720.

[16] Ł. Woźniak, A. A. Rajkiewicz, L. Monsigny, A. Kajetanowicz, K. Grela, *Org. Lett.* **2020**, *22*, 4970-4973.

[17] J.-N. Mo, W. Han, W.-D. Liu, W. Wang, J. Zhao, *Chem. Asian J.* **2025**, *20*, e202401807.

[18] T. Ishikawa, N. Kasai, Y. Yamada, T. Hanamoto, *Tetrahedron* **2015**, *71*, 1254-1260.

[19] B. J. Ager, L. E. Bourque, K. M. Buchner, K. A. Woerpel, *J. Org. Chem.* **2010**, *75*, 5729-5732.

[20] K. Colas, R. Martín-Montero, A. Mendoza, *Angew. Chem. Int. Ed.* **2017**, *56*, 16042-16046.

[21] Y.-Z. Yang, Y. Li, G.-F. Lv, D.-L. He, J.-H. Li, *Org. Lett.* **2022**, *24*, 5115-5119.

[22] N. Alotaibi, R. Babaahmadi, S. Das, E. Richards, T. Wirth, M. Pramanik, R. L. Melen, *Chem. Eur. J.* **2025**, *31*, e202404236.

[23] H. Li, Z. Xia, L. Li, J. Zeng, J. Lv, H. Wang, S. Gu, F. Chen, *Org. Lett.* **2024**, *26*, 8405-8409.

[24] L. Marzorati, M. A. da Silva, B. Wladislaw, C. D. Vitta, *Synth. Commun.* **2003**, *33*, 3491-3495.

[25] K. Fu, K. Tian, Z. Zhang, J. Guo, C. Yuan, *Synlett* **2023**, *34*, 1791-1794.

[26] X. Jia, X. Ma, W. Feng, J.-Q. Zhang, Y. Zhao, B. Guo, L. Tang, Y.-Y. Yang, *J. Org. Chem.* **2022**, *87*, 16492-16505.

[27] L. Costantino, S. Ferrari, M. Santucci, O. M. H. Salo-Ahen, E. Carosati, S.

Franchini, A. Lauriola, C. Pozzi, M. Trande, G. Gozzi, P. Saxena, G. Cannazza, L. Losi, D. Cardinale, A. Venturelli, A. Quotadamo, P. Linciano, L. Tagliazucchi, M. G. Moschella, R. Guerrini, S. Pacifico, R. Luciani, F. Genovese, S. Henrich, S. Alboni, N. Santarem, A. da Silva Cordeiro, E. Giovannetti, G. J. Peters, P. Pinton, A. Rimessi, G. Cruciani, R. M. Stroud, R. C. Wade, S. Mangani, G. Marverti, D. D'Arca, G. Ponterini, M. P. Costi, *eLife* **2022**, *11*, e73862.

[28] R. R. Amici, C. Di Vitta, L. Marzorati, *Synthesis* **2013**, *45*, 798-802.

[29] M. Markwitz, K. Labrzycki, K. Łyczek, K. Kuciński, *J. Org. Chem.* **2025**, *90*, 9513-9518.

[30] H. Yang, E. Wang, P. Yang, H. Lv, X. Zhang, *Org. Lett.* **2017**, *19*, 5062-5065.

[31] K. Cao, S. M. Tan, R. Lee, S. Yang, H. Jia, X. Zhao, B. Qiao, Z. Jiang, *J. Am. Chem. Soc.* **2019**, *141*, 5437-5443.

[32] T. Sakamoto, Y. Kondo, M. Shiraiwa, H. Yamanaka, *Synthesis* **1984**, *1984*, 245-247.

[33] M. Hrast, R. Frlan, D. Knez, I. Zdovc, H. Barreteau, S. Gobec, *Bioorg. Med. Chem. Lett.* **2021**, *40*, 127966.

[34] A. S. Adhikari, N. Majumdar, *Org. Lett.* **2023**, *25*, 8611-8616.

[35] A. Toynton, L. Ferrins, H. L. Newson, M. L. Sykes, S. Varghese, N. Nguyen, S. Russell, R. Rahmani, J. Cheang, G. R. Flematti, B. W. Skelton, B. Zulfiqar, V. M. Avery, J. B. Baell, M. J. Piggott, *RSC Med. Chem.* **2025**, *16*, 1654-1680.

[36] H. Möhrle, M. Pycior, D. Wendisch, *Arch. Pharm.* **1994**, *327*, 445-453.

[37] Y.-F. Qiu, Y.-J. Niu, X. Wei, B.-Q. Cao, X.-C. Wang, Z.-J. Quan, *J. Org. Chem.* **2019**, *84*, 4165-4178.

[38] X.-Y. Zhou, M. Zhang, Z. Liu, J.-H. He, X.-C. Wang, *J. Am. Chem. Soc.* **2022**, *144*, 14463-14470.

[39] Y. Zhao, D. G. Truhlar, *Theor. Chem. Acc.* **2008**, *120*, 215-241.

[40] F. Weigend, F. Furche, R. Ahlrichs, *J. Chem. Phys.* **2003**, *119*, 12753-12762.

[41] A. A. Otlyotov, Y. Minenkov, *J. Comput. Chem.* **2023**, *44*, 1807-1816.

[42] H. Yu, J. Zheng, D. G. Truhlar, **2015** (unpublished),

https://comp.chem.umn.edu/freqscale/version3b2.htm.

[43] T. Lu, Q. Chen, *Comput. Theor. Chem.* **2021**, *1200*, 113249.

[44] C. Riplinger, B. Sandhoefer, A. Hansen, F. Neese, *J. Chem. Phys.* **2013**, *139*, 134101.

[45] F. Neese, *WIREs. Comput. Mol. Sci.* **2025**, *15*, e70019.

[46] a) T. Lu, F. Chen, *J. Comput. Chem.* **2012**, *33*, 580-592; b) T. Lu, *J. Chem. Phys.* **2024**, *161*, 082503.

[47] F. Weigend, R. Ahlrichs, *PCCP* **2005**, *7*, 3297-3305.

[48] R. A. Moss, K. Krogh-Jespersen, *Tetrahedron Lett.* **2013**, *54*, 4303-4305.

[49] P. J. Stephens, F. J. Devlin, C. F. Chabalowski, M. J. Frisch, *J. Phys. Chem.* **1994**, *98*, 11623-11627.

[50] a) R. Krishnan, J. S. Binkley, R. Seeger, J. A. Pople, *J. Chem. Phys.* **1980**, *72*, 650-654; b) A. D. McLean, G. S. Chandler, *J. Chem. Phys.* **1980**, *72*, 5639-5648; c) M. M. Francl, W. J. Pietro, W. J. Hehre, J. S. Binkley, M. S. Gordon, D. J. DeFrees, J. A. Pople, *J. Chem. Phys.* **1982**, *77*, 3654-3665; d) T. Clark, J. Chandrasekhar, G. W. Spitznagel, P. V. R. Schleyer, *J. Comput. Chem.* **1983**, *4*, 294-301; e) G. W. Spitznagel, T. Clark, P. von Ragué Schleyer, W. J. Hehre, *J. Comput. Chem.* **1987**, *8*, 1109-1116.

[51] a) W. J. Hehre, R. Ditchfield, J. A. Pople, *J. Chem. Phys.* **1972**, *56*, 2257-2261; b) P. C. Hariharan, J. A. Pople, *Theor. Chim. Acta* **1973**, *28*, 213-222; c) M. S. Gordon, J. S. Binkley, J. A. Pople, W. J. Pietro, W. J. Hehre, *J. Am. Chem. Soc.* **1982**, *104*, 2797-2803.
