# Supplementary material for: Bis(trifluoromethylthio)carbene as a toolbox for versatile carbene transformations
Source: Nat Commun. 2026 Jul 29;17:9214. doi: 10.1038/s41467-026-76124-z (PMC13522504; doi:10.1038/s41467-026-76124-z)
Supplement: Supplementary file 2 [file 41467_2026_76124_MOESM2_ESM.pdf]

nature portfolio

Peer Review File

Bis(trifluoromethylthio)carbene as a Toolbox for Versatile Carbene Transformations

Corresponding Author: Professor Ji-Chang Xiao

This file contains all reviewer reports in order by version, followed by all author rebuttals in order by version.

Version 0:

Reviewer comments:

Reviewer #1

(Remarks to the Author)
The manuscript by Xiao and co-workers describes the generation of a bis-SCF3 carbene generation reagent through a simple base mediated procedure from a parent bromide. The study is truly exceptional in the variety and scope of transformations that have been studied to investigate the various reactivity that this reagent can be used in. The work demonstrates that this approach can be used to install geminal SCF3 groups into a wide range of compounds including any different alicyclic and heterocyclic compounds. The versatility of the reagent is described in cyclopropanation, skeletal editing, X-H insertion, rearrangements and migrations. All of these classes of reactions are of interest to the synthetic organic community and could offer interesting pathways to include this moiety into small molecules for a variety of purposes. The potential impact of this approach could be within medicinal chemistry and materials design as there are not many methods to install a single SCF3 let alone a two on the same carbon. The paper is very well put together and the authors have done a very good job in conveying the scope of the reactivity in combination with insights into its mechanism. The supporting information has been put together well and is of high standard. This manuscript would be of interest to the general readership of Nature Communications and the study has been conducted to the highest level I believe that it should be published following some minor corrections in how some of the manuscript is phrased. Please see below for a list of suggested corrections.

1) Introduction "from carbon centre" should be change to "from a single carbon centre"
2) Introduction "conductive to maintaining carbene's electrophilicity" should be changed to "conductive to maintaining a carbene's electrophilicity"
3) Capitalisation of the titles in Scheme 1A/1B is not consistent.
4) Can the yield of the BrCH(SCF3)2 be added to the discussion in addition to the reaction scale.
5) "affords" should be changed to "afforded"
6) "presence of olefin substrate" should be changed to "presence of an olefin substrate"

Reviewer #2

(Remarks to the Author)
Xiao et al. introduce a unique new player in carbene-type chemistry by developing a tailor-made reactant, enabling the control of reactivity and selectivity. To this end, they individuate two pivotal characteristics governing the general chemical behavior, namely the installation of a powerful EW functionality and an adequate steric element. Both features are constitutively present on SCF3 groups thus, resulting in generating a C1-donor which is therefore thoroughly applied for solving a variety of transformations, hitherto regarded as elusive. This new carbene – easily accessible via standard chemistry – documents remarkable stability, an aspect per se highly interesting emerged by theoretical calculations. Undoubtedly, a key element of the present work is the flexibility to install the functionalized C1-unit in a series of recipient, while no significant modification is required to the standard conditions. This is a fact – which in the opinion of this reviewer – novel in carbene chemistry where a strong relationship between the desired chemical transformation and the employed experimental conditions is observed. This is – in my opinion – the key element justifying the publication in Nat Commun. I would recommend, however, the refinement of the English which is somewhere redundant and, if I may say, not purely scientific. Also - within the minor revisions - Authors are invited to comment about the direct selection of (CF3S)2C=S for their study and the reasons accounting for not having considered eventual alternatives.

Reviewer #3

(Remarks to the Author)
In this manuscript, Sun and colleagues report the rational design, synthesis, and comprehensive reactivity study of bis(trifluoromethylthio)carbene as a versatile platform for carbene-mediated transformations. The authors convincingly demonstrate that this electronically tuned carbene functions as a thermally and kinetically stabilized analogue of classical dihalocarbenes, while maintaining broad synthetic utility. Through extensive experimental and computational studies, they establish that the carbene is a ground-state singlet species with balanced reactivity and improved chemoselectivity. Overall, the study is conceptually innovative, experimentally comprehensive, and makes a meaningful contribution to carbene chemistry and reactive intermediate design. The range of transformations demonstrated—including cyclopropanation, skeletal editing, X–H insertion, sulfur-ylide-mediated rearrangements, and N-ylide-triggered cyclizations—is impressive. The gram-scale synthesis of the carbene precursor further highlights its practical potential. Despite these strengths, several conceptual, mechanistic, and practical issues would benefit from clarification or deeper discussion. I recommend publication after major revisions addressing the points outlined below.

Major Strengths:

1) The work exemplifies rational carbene design. Compared to dihalocarbenes:
• The bis(trifluoromethylthio)carbene is thermally stabilized (sulfur substitution) and sterically protected.
• It exists as a ground-state singlet, avoiding undesired radical pathways typical of triplet carbenes.
• Computational analysis (electrophilicity index, chemical hardness, stabilization energies) effectively rationalizes its reactivity profile.

The comparison with classical carbenes such as $:CF_2$ and :CFCl provides valuable conceptual grounding. The use of conceptual DFT indices is commendable; however, foundational contributions by de Proft and Geerlings (Chem. Rev. 2003, 103, 1793–1874) should be cited when discussing electrophilicity and hardness descriptors.
Control experiments (radical traps, selective cyclopropanation) are well-designed and convincingly support the singlet nature of the carbene.

2) The carbene precursor is applied across multiple major classes of carbene chemistry. It is particularly impressive that a single carbene platform performs efficiently across such diverse reactivity manifolds. The S-ylide-mediated rearrangements are especially strong, delivering high yields and notable chemoselectivity (e.g., absence of competing cyclopropanation). This highlights the carbene's controlled reactivity relative to many transition-metal-derived analogues.
Points for Clarification and Improvement:

Practical Considerations and Limitations:

1. Although scalable, precursor synthesis involves generating stoichiometric HCN and requires NaH for carbene generation. These factors may limit adoption in medicinal chemistry, particularly for substrates with sensitive functional groups, or in industrial settings. A brief discussion of safety considerations and operational practicality would be valuable.

2. The manuscript describes photolysis as “harsh,” yet photochemical carbene generation is widely accepted in modern synthesis. This comparison should be presented more objectively, especially since the authors use a very strong base, sodium hydride.

3. Unlike halogens, the $–SCF_3$ group does not obviously serve as a versatile synthetic handle for downstream diversification. The authors should discuss:
o Medicinal and agrochemical relevance of the $–SCF_3$ motif.
o Whether limited post-functionalization potential affects its utility for skeletal editing or late-stage modification.

4. While the scope is broad, limitations are not systematically analyzed. A critical discussion of substrate restrictions, yield variability, and functional group incompatibilities would strengthen the manuscript.

5. The authors repeatedly highlight exceptional chemoselectivity. Explicit competition experiments—particularly in substrates containing both an alkene and an X–H bond—would provide compelling support to see preferential reactivity towards alkene or X-H insertion. This would also provide insights into the nature of carbene.

6. Cyclopropanation Electronic Effects: Comparable yields for –OMe and $–NO_2$ substituted alkenes (e.g., 2c vs 2j) warrant mechanistic discussion.

7. Skeletal Editing Scope:
o Pyrrole expansion appears limited to 2,5-disubstituted systems. Are 2,3- or 3,4-substituted variants viable?
o For unsymmetrical substrates (e.g., 4q), moderate yields are reported. What are the byproducts?
o Could competing N–H insertion be occurring in heterocycles as observed with imidazoles? Why don’t imidazoles undergo ring expansions, as shown in the recent work published in J. Am. Chem. Soc. 2025, 147, 16, 13824–13832, https://doi.org/10.1021/jacs.5c02012

8. X–H Insertion Scope: In the X–H insertion section, the substrate scope appears to be limited primarily to aromatic

alcohols, amines, and thiols. It would be valuable for the authors to clarify whether aliphatic counterparts (amines and thiols) were evaluated. Notably, in example 6g, phenol appears to react more efficiently than an aliphatic alcohol. As the authors point out, the softness of this carbene, I would like to see an experiment comparing it with a substrate bearing both OH and SH groups to determine which reacts preferentially. A mechanistic rationale or discussion addressing these observations would strengthen the manuscript. Low yields (6p–6r) require clarification—are competing insertions, ring expansion, or decomposition pathways involved? To gain further mechanistic insights, I suggest that authors synthesize deuterium-labeled carbene precursors. This will help determine whether the X-H insertion proceeds via a carbene or a direct substitution reaction.

9. In most of the transformations described, NaH is employed in excess, but its amount varies from substrate to substrate and can reach as high as 8.0 equivalents. The authors are encouraged to clarify the rationale behind this difference in stoichiometry. A brief explanation regarding the role of the base in each transformation would improve clarity and reproducibility. This change in the amount of base also raises an important question about the efficient generation of carbenes, and therefore, the synthesis of a deuterium-labeled carbene precursor is further warranted.

10. The observation that tBu outperforms SMe in migration reactions contradicts expected trends in donor ability and should be rationalized.

11. N-Ylide [4+1] Cyclization Mechanism: The mechanism invokes proton-driven C–SCF_3 bond cleavage under basic conditions. Deuterium labeling of the terminal alkene (=CHD) in vinyl-pyridine 11 will provide insights into the KH/KD for SCF3 elimination.

12. Also, in the introduction, cite other carbene derivatives and their trend. The introduction uses strong language that is inconsistent with the experimental results. The authors claim that this carbene, due to its sulfur atoms, favors reactions with soft substrates, whereas most results on heteroatom insertion indicate the opposite trend. Please clarify or revise the language. I also encourage authors to compare with other sulfenylcarbene in Scheme 1a, which have one side sulfur atom and the other group being electron donating or withdrawing (see recent review, and cite it. Chem. Soc. Rev., 2025, 54, 10344 - 10362, DOI: 10.1039/d5cs00278h)

Minor Comments:
o Absolute configurations should be explicitly shown for stereogenic products.
o E/Z ratios (e.g., 10o, 10q) should be clearly reported.
o Standardize notation of the trifluoromethylthio group (SCF_3 vs –SCF_3).
o Correct minor typographical inconsistencies (e.g., bold font in yield for 10m).
o Ensure proper alignment of CF_3 labels in figures (e.g., 8q, 8r).
o Use “chemical shift” instead of “chemical migration” (SI).
o Supporting Information and Reproducibility: Specify NaH formulation (e.g., 60% dispersion in mineral oil). Include Rf values for all compounds. Resolve discrepancies in solvent reporting (e.g., toluene vs 1,4-dioxane in synthesis of 1n).

Overall Recommendation:

This manuscript presents a thoughtfully designed and experimentally validated carbene platform with broad applicability across classical carbene reaction classes. The balance between stability and reactivity, combined with detailed computational analysis and practical-scale synthesis, makes this a significant contribution to carbene chemistry. While certain mechanistic clarifications, scope expansions, and presentation refinements are warranted, these issues can be addressed through revision. I therefore recommend publication in Nature Communications after major revision addressing the points above.

Version 1:

Reviewer comments:

Reviewer #3

(Remarks to the Author)
The authors have done an excellent job addressing all of my suggestions and comments. I appreciate their careful and comprehensive responses to the review points. In my opinion, the manuscript is now suitable for publication in Nature Communications.

Open Access This Peer Review File is licensed under a Creative Commons Attribution 4.0 International License, which permits use, sharing, adaptation, distribution and reproduction in any medium or format, as long as you give appropriate credit to the original author(s) and the source, provide a link to the Creative Commons license, and indicate if changes were made.
In cases where reviewers are anonymous, credit should be given to 'Anonymous Referee' and the source.
The images or other third party material in this Peer Review File are included in the article's Creative Commons license, unless indicated otherwise in a credit line to the material. If material is not included in the article's Creative Commons license and your intended use is not permitted by statutory regulation or exceeds the permitted use, you will need to obtain permission directly from the copyright holder.
To view a copy of this license, visit https://creativecommons.org/licenses/by/4.0/

SHANGHAI INSTITUTE OF ORGANIC CHEMSTRY
CHINESE ACADEMY OF SCIENCES

==

Response to Reviewers

Dear respectable reviewers:

Thank you very much for your valuable suggestions. We have addressed all comments by revising the Manuscript and the Supplementary Information and by providing a point-by-point response listed as follows. The Manuscript with changes highlighted by a yellow background or by using the track changes mode in MS Word have also been uploaded.

To Reviewer 1:

We appreciate Reviewer 1's support for the publication of this paper after minor revisions.

Advice 1: Introduction "from carbon centre" should be change to "from a single carbon centre"

Response: The error has been corrected.

Advice 2: Introduction "conductive to maintaining carbene's electrophilicity" should be changed to "conductive to maintaining a carbene's electrophilicity"

Response: The error has been corrected.

Advice 3: Capitalisation of the titles in Scheme 1A/1B is not consistent.

Response: We thank the reviewer for pointing out this inconsistency. We have now carefully revised the titles of Scheme 1A and Scheme 1B to ensure consistent capitalization throughout (both titles now follow the same format, using headline-style capitalization).

Advice 4: Can the yield of the $BrCH(SCF_3)_2$ be added to the discussion in addition to the reaction scale.

Response: We thank the reviewer for this constructive suggestion. We have now added the yields for the two-step preparation of $BrCH(SCF_3)_2$ in the main text, alongside the reaction scale. Specifically, the hydrogen cyanide addition step afforded $(CF_3S)_2CHSCN$ in 63% yield, and the subsequent bromination step gave $BrCH(SCF_3)_2$ in 67% yield.

Advice 5: "affords" should be changed to "afforded"

Response: The error has been corrected.

Advice 6: "presence of olefin substrate" should be changed to "presence of an olefin substrate"

Response: The error has been corrected.

To Reviewer 2:

We appreciate Reviewer 2's support for the publication of this paper after minor revisions.

Advice 1: The refinement of the English which is somewhere redundant and, if I may say, not purely scientific.

Response: We thank the reviewer for this valuable suggestion. We have carefully re-examined the entire manuscript and refined the language throughout to make it more concise and scientifically rigorous.

Advice 2: Authors are invited to comment about the direct selection of $(CF_3S)_2C=S$ for their study and the reasons accounting for not having considered eventual alternatives.

Response: We thank the reviewer for this insightful question. We have added a brief comment in the revised manuscript to clarify the rationale for selecting $(CF_3S)_2C=S$ as follows: "We reason that an alternative route to this carbene can rely on base-promoted α-elimination, requiring bis(trifluoromethylthio)halomethane as precursor, However, direct double substitution on polyhalomethanes suffers from poor efficiency and generates complex mixtures. Instead, we turned to an existing bis(trifluoromethylthio) building block. Building upon our previously established hundred-gram-scale synthesis of bis(trifluoromethylthio)thiocarbonate ($(CF_3S)_2C=S$), we successfully prepared the key reagent, bis(trifluoromethylthio)bromomethane ($BrCH(SCF_3)_2$, >30 g per batch)."

To Reviewer 3:

We thank Reviewer 3 for the thorough and constructive evaluation. All raised concerns have been addressed in the revised manuscript, and detailed point-by-point responses are presented below.

Advice 1: Foundational contributions by de Proft and Geerlings (*Chem. Rev.* **2003**, *103*, 1793–1874) should be cited when discussing electrophilicity and hardness descriptors.

Response: We have now added the recommended citation (ref. 59) in the discussion of electrophilicity and hardness descriptors. We thank the reviewer for pointing out this important reference.

Advice 2: Although scalable, precursor synthesis involves generating stoichiometric HCN and requires NaH for carbene generation. These factors may limit adoption in medicinal chemistry, particularly for substrates with sensitive functional groups, or in industrial settings. A brief discussion of safety considerations and operational practicality would be valuable.

Response: We thank the reviewer for this practical consideration. In the revised manuscript, we have added brief operational comments in the Results and discussion section. Specifically, during the two-step synthesis of $BrCH(SCF_3)_2$, we now note: "All operations were performed in a well-ventilated fume hood due to HCN generation." Regarding carbene generation, we state: "NaH was weighed under nitrogen due to its moisture sensitivity." These standard precautions, already common in synthetic laboratories, ensure safe handling and reproducibility.

Advice 3: The manuscript describes photolysis as "harsh," yet photochemical carbene generation is widely accepted in modern synthesis. This comparison should be presented more objectively, especially since the authors use a very strong base, sodium hydride.

Response: We thank the reviewer for this constructive criticism. We agree that the original wording "harsh" was subjective. To present a more objective comparison, we have revised the relevant sentence in the Introduction about Hass's work: "In 1994, Hass et al. reported the generation of bis(trifluoromethylthio)carbene ($:C(SCF_3)_2$) via low-temperature photolysis of bis(trifluoromethylthio)ketene. While this photochemical method is effective, its application was limited by

the relatively laborious synthesis of the precursor.”

Advice 4: Unlike halogens, the –SCF_3 group does not obviously serve as a versatile synthetic handle for downstream diversification. The authors should discuss:

o Medicinal and agrochemical relevance of the –SCF_3 motif.

o Whether limited post-functionalization potential affects its utility for skeletal editing or late-stage modification.

Response: We thank the reviewer for this constructive comment. In response, we have revised the Introduction to explicitly address both points:

Regarding the medicinal and agrochemical relevance of the –SCF_3 motif, we have added a brief summary of the importance of the SCF_3 group in drug discovery and agrochemistry, citing representative bioactive molecules and commercial agrochemicals in the Introduction: “On the other hand, the SCF_3 motif has proven valuable in drug discovery and agrochemistry, with numerous bioactive molecules bearing this group showing promising therapeutic potential, and agrochemicals such as Flupentiofenox and Vaniliprole already in practical use. Given this background, we proposed that the bis(trifluoromethylthio)carbene (:$C(SCF_3)_2$), leveraging the synergistic electronic and steric effects of its two –SCF_3 groups, could enable efficient construction of a range of SCF_3-containing molecules.” This underscores the demand for efficient methods to incorporate SCF_3 group into diverse scaffolds.

For the post-functionalization potential and utility in skeletal editing/late-stage modification, we agree that the –SCF_3 group is not a traditional synthetic handle for further diversification. In the present work, the SCF_3 group is primarily installed as a terminal functional moiety that directly confers desirable physicochemical properties (lipophilicity, metabolic stability, electron-withdrawing character). Given that the introduction of the SCF_3 group itself is already valuable for medicinal and agrochemical applications, we did not explore further downstream derivatization of this group. That said, we recognize that exploring potential transformations of the –SCF_3 moiety under appropriate conditions could be an interesting direction for future studies.

Advice 5: While the scope is broad, limitations are not systematically analyzed. A critical discussion of substrate restrictions, yield variability, and functional group incompatibilities would strengthen the manuscript.

Response: We thank the reviewer for this constructive suggestion. In the revised manuscript, we have added a critical discussion of limitations, yield variability, and functional group incompatibilities for each of the six reaction types presented in this work. For example, we have added a specific limitation at the end of the cyclopropanation subsection: “Strongly electron-deficient alkene **1t** gave no conversion, as electron-withdrawing substituent disfavors electrophilic cycloaddition.”

Advice 6: The authors repeatedly highlight exceptional chemoselectivity. Explicit competition experiments—particularly in substrates containing both an alkene and an X–H bond—would provide compelling support to see preferential reactivity towards alkene or X-H insertion. This would also provide insights into the nature of carbene.

Response: We have performed a competition experiment using 2-vinylphenol (**5f**), which bears both an alkene and an O–H bond. The carbene exclusively inserted into the O–H bond without any cyclopropanation, confirming its preferential interaction with the O-H group. This result is now included in the revised manuscript.

Advice 7: Cyclopropanation Electronic Effects: Comparable yields for –OMe and $–NO_2$ substituted alkenes (e.g., **2c** vs **2j**) warrant mechanistic discussion.

Response: We thank the reviewer for carefully noticing this apparent inconsistency. In our initially reported data, the electron-rich styrene **1c** (*p*-OMe) gave a lower yield of cyclopropanation product **2c** than the electron-deficient **1j** (*p*-NO_2), which would indeed contradict the expected trend for an electrophilic carbene. Upon re-examining our laboratory records, we found that the **1c** reagent used in the original experiment had been stored in its bottle for a prolonged period. This extended storage may have caused partial spontaneous reactions, such as self-polymerization, leading to a decrease in its purity. When the reaction was repeated using freshly purchased **1c**, the desired cyclopropane **2c** was obtained in 87% yield, which is now consistent with the anticipated higher reactivity of electron-rich alkenes toward the electrophilic carbene. Accordingly, the yield of **2c** has been corrected in the revised manuscript.

Furthermore, we identified a similar yield anomaly for the X–H insertion product **6k** (revised numbering; originally **6j** in the initial submission). Compound **5k** also contains a methoxy substituent on an electron-rich aromatic ring. Using the originally aged sample of **5k** gave an unusually low yield; repetition with freshly obtained **5k** afforded **6k** in 68% yield. This correction has also been made in the revised manuscript.

We apologize for the oversight and thank the reviewer for prompting this re-examination, which has improved the accuracy and reliability of our reported data.

Advice 8: Skeletal Editing Scope:

o Pyrrole expansion appears limited to 2,5-disubstituted systems. Are 2,3- or 3,4-substituted variants viable?

o For unsymmetrical substrates (e.g., **4q**), moderate yields are reported. What are the byproducts?

o Could competing N–H insertion be occurring in heterocycles as observed with imidazoles? Why don't imidazoles undergo ring expansions, as shown in the recent work published in *J. Am. Chem. Soc.* **2025**, *147*, 16, 13824–13832, https://doi.org/10.1021/jacs.5c02012

Response: We thank the reviewer for these insightful comments. We have attempted to address each question separately as follows.

1. On the viability of 2,3- or 3,4-disubstituted pyrroles (pyrrole expansion):

We thank the reviewer for this question. We examined 3,4-dimethylpyrrole as a representative substrate. The substrate proved difficult to convert under the standard conditions, presumably because the bis(trifluoromethylthio)carbene is more sensitive to steric hindrance at the C3 position of the pyrrole, which impede the initial cycloaddition step, thereby compromising the skeletal editing efficiency. This result has been described in the revised manuscript.

2. On the moderate yield of product 4q (now 4r in the revised manuscript) and byproduct identification:

For this product, we performed a crude yield analysis using 0.5 mmol of $PhOCF_3$ as an internal standard, which exhibited a yield of 56%. ^{19}F NMR and GC-MS of the crude mixture did not reveal any clear byproduct signals; TLC showed some faint additional spots, but they appeared to be minor and attempts to isolate them were not successful. We therefore attribute the moderate isolated yield primarily to material loss during purification, likely due to the relatively low molecular weight and moderate volatility of the product.

3. On the observation of competing N-H insertion, absence of ring-expanded products from

imidazoles and comparison with the literature (J. Am. Chem. Soc. 2025, 147, 13824–13832):

We performed the reaction with imidazole **5t** (revised numbering; originally **5q** in the initial submission) under the standard conditions. ^{19}F NMR and GC-MS analysis of the crude reaction mixture suggested the formation of a single N–H insertion product, and no ring-expanded product was detected after repeated purification attempts. We thank the reviewer for bringing this interesting work to our attention.

We attribute the lack of imidazole ring expansion to two factors. First, our bis(trifluoromethylthio)carbene is much more sterically hindered than the monothiocarbenes used in the cited work, disfavoring the [2+1] cycloaddition to the imidazole C=C bond. Second, the literature employs mild bases (Cs_2CO_3 or *t*-BuOK), under which imidazole likely remains neutral, whereas our strong base NaH deprotonates imidazole. The carbene then preferentially reacts with the resulting nitrogen center rather than with the C=C double bond, thereby preventing the cycloaddition required for ring expansion.

Advice 9: X–H Insertion Scope: In the X–H insertion section, the substrate scope appears to be limited primarily to aromatic alcohols, amines, and thiols. It would be valuable for the authors to clarify whether aliphatic counterparts (amines and thiols) were evaluated. Notably, in example **6g**, phenol appears to react more efficiently than an aliphatic alcohol. As the authors point out, the softness of this carbene, I would like to see an experiment comparing it with a substrate bearing both OH and SH groups to determine which reacts preferentially. A mechanistic rationale or discussion addressing these observations would strengthen the manuscript. Low yields (**6p**–**6r**) require clarification—are competing insertions, ring expansion, or decomposition pathways involved? To gain further mechanistic insights, I suggest that authors synthesize deuterium-labeled carbene precursors. This will help determine whether the X-H insertion proceeds via a carbene or a direct substitution reaction.

Response:

1. Evaluation of aliphatic amines and thiols:

We examined benzyl mercaptan (**5q**, revised numbering) and benzyl methyl amine (**5w**, revised numbering) under the standard conditions. Both substrates gave very low conversion (12% for **5q** and no detectable product for **5w**), indicating that aliphatic thiols and amines are not suitable substrates for this carbene. These results have been added to the revised manuscript.

2. Competition between O–H and S–H bonds (substrate bearing both OH and SH):

We employed 3-mercaptophenol (**5p**, revised numbering) as a substrate containing both a phenolic OH and a thiophenolic SH. Under conditions with a modest excess of the carbene precursor, exclusive S–H insertion was observed, affording the corresponding sulfide product **6p**; no O–H insertion product was detected. When more than two equivalents of the carbene precursor were used, double insertion at both the S–H and O–H sites occurred to give **6p′**. These results has been described in the revised manuscript.

3. Low yields for azole substrates (6p–6r, original numbering; **6s–6u**, revised numbering**):**

We thank the reviewer for raising this point. GC-MS analysis of the crude reaction mixtures for azoles substrates did not reveal competing insertion or ring-expansion byproducts. However, TLC monitoring showed the formation of several uncharacterized spots, indicating the presence of decomposition pathways. The reaction mixtures appeared complex, and the putative byproducts could not be isolated or identified under the current conditions. We therefore attribute the low isolated yields primarily to such uncharacterized decomposition processes.

4. Mechanistic distinction between carbene insertion and direct substitution:

To differentiate the proposed carbene-mediated X–H insertion from a potential direct nucleophilic substitution pathway, we synthesized a deuterium-labeled carbene precursor, $BrCD(SCF_3)_2$. In the absence

of NaH, the sodium salt of substrate **5r** was treated with the deuterated precursor in THF. The product **6r** was obtained in a yield comparable to the standard reaction, but with complete loss of deuterium. This result strongly argues against a direct substitution process. Furthermore, from a steric perspective, the carbon center in $BrCH(SCF_3)_2$ is highly congested due to the two bulky $–SCF_3$ groups, making it unfavorable for a nucleophile to attack this carbon in an S_N2-type substitution.

Full experimental details about the deuterium-labeling experiment have been added to the Supporting Information, and a concise statement has been included in the revised manuscript (X–H insertion subsection).

Advice 10: In most of the transformations described, NaH is employed in excess, but its amount varies from substrate to substrate and can reach as high as 8.0 equivalents. The authors are encouraged to clarify the rationale behind this difference in stoichiometry. A brief explanation regarding the role of the base in each transformation would improve clarity and reproducibility. This change in the amount of base also raises an important question about the efficient generation of carbenes, and therefore, the synthesis of a deuterium-labeled carbene precursor is further warranted.

Response: We thank the reviewer for this question. In most reactions, we fixed the NaH:$BrCH(SCF_3)_2$ ratio at 2:1 to ensure complete carbene generation. Since 2–3 equiv of the precursor were typically used, NaH loadings naturally ranged from 4 to 6 equiv.

For diallyl sulfide (initially **7v**, now **7w**), we increased the precursor to 4 equiv (NaH 8 equiv) to probe the steric tolerance of the carbene. Even under this forcing condition, only mono-modification was observed, highlighting the pronounced steric hindrance of the bis(trifluoromethylthio)carbene.

Beyond carbene generation, excess NaH also deprotonates the substrates or intermediates, thereby promoting $HSCF_3$ elimination to drive skeletal editing and [4+1] cyclization reactions to completion. X–H insertion is an exception: here NaH was kept slightly lower than the precursor to avoid potential base-induced product decomposition.

These points have been added to the revised manuscript.

Advice 11: The observation that *t*Bu outperforms SMe in migration reactions contradicts expected trends in donor ability and should be rationalized.

Response: We thank the reviewer for the comment. The observed higher yield of product **10b** (4-*tert*-butylphenyl) compared to **10d** (4-methylthiophenyl) can be rationalized by their Hammett substituent constants, as compiled in the comprehensive survey by Hansch, Leo, and Taft (*Chem. Rev.* **1991**, *91*, 165-195). In that work, the σ_p values for SMe and *t*Bu are reported as 0.00 and –0.20, respectively. The negative σ_p value indicates that the *tert*-butyl group is a mild electron-donating substituent, whereas the SMe group, with a σ_p value of 0.00, exerts almost no net polar effect on the aromatic ring. Therefore, the stronger electron-donating character of the *tert*-butyl group increases the electron density on the sulfur atom of the ylide intermediate, enhancing its nucleophilicity and thus promoting the 1,2-migration step, which leads to the observed higher yield of **10b**.

Advice 12: N-Ylide [4+1] Cyclization Mechanism: The mechanism invokes proton-driven $C–SCF_3$ bond cleavage under basic conditions. Deuterium labeling of the terminal alkene (=CHD) in vinyl-pyridine **11** will provide insights into the k_H/k_D for SCF_3 elimination.

Response: We thank the reviewer for this thoughtful suggestion. In our proposed mechanism (revised manuscript, Scheme 5), direct deprotonation of the methine proton in intermediate **D-2** appears to be

difficult under the reaction conditions, as the substantial steric hindrance around the bis(trifluoromethylthio)methylene restricts its accessibility to the base, and the lack of strongly electron-withdrawing groups renders the proton weakly acidic. Instead, we propose that the elimination of $HSCF_3$ is initiated by rate-determining heterolytic cleavage of the C–SCF_3 bond, facilitated by the significant steric congestion of the geminal bis(trifluoromethylthio)methylene group and the strong conjugative electron donation from the indolizine nitrogen. The resulting carbocationic intermediate then undergoes rapid deprotonation. In this scenario, the proton transfer step appears not to be rate-limiting; therefore, we suspect that a deuterium isotope effect (k_H/k_D) study might not provide additional mechanistic insight in this particular case. We have now clarified this point in the revised manuscript by updating the mechanistic scheme and the accompanying discussion. We hope that this mechanistic rationale adequately addresses the reviewer's concern.

Advice 13: Also, in the introduction, cite other carbene derivatives and their trend. The introduction uses strong language that is inconsistent with the experimental results. The authors claim that this carbene, due to its sulfur atoms, favors reactions with soft substrates, whereas most results on heteroatom insertion indicate the opposite trend. Please clarify or revise the language. I also encourage authors to compare with other sulfenylcarbene in Scheme 1a, which have one side sulfur atom and the other group being electron donating or withdrawing (see recent review, and cite it. *Chem. Soc. Rev.*, **2025**, *54*, 10344 - 10362, DOI: 10.1039/d5cs00278h).

Response: We thank the reviewer for this constructive criticism. We agree that the original wording in the Introduction overemphasized the role of the carbene's softness in determining selectivity. In the revised manuscript, we have moderated these statements to present a more balanced discussion, we hope the revised language is more cautious and scientifically appropriate. On the other hand, we have now cited the recommended review (ref. 28) and have revised the Introduction to include a comparison with other sulfenylcarbene systems.

Advice 14: Absolute configurations should be explicitly shown for stereogenic products.

Response: We thank the reviewer for this suggestion. In our reaction system, no chiral reagents or chiral solvents were employed, and all reactions were carried out under racemic conditions. Consequently, all products bearing stereogenic centers were obtained as racemic mixtures. Under these circumstances, the absolute configurations cannot be assigned.

Advice 15: *E*/*Z* ratios (e.g., **10o**, **10q**) should be clearly reported.

Response: We thank the reviewer for this careful observation. Regarding compound **10q**, the two substituents on the alkene carbons are both phenyl groups; therefore, no *E*/*Z* isomerism exists for this product, and the issue of ratio does not apply.

For compound **10o**, it is a new compound without prior spectral characterization in the literature. We attempted to assign the *E*/*Z* configuration of the two alkene isomers by 2D NMR experiments (^1H-^1H COSY). Unfortunately, no diagnostic cross-peaks were observed that would allow unambiguous assignment. In addition, due to the lack of alternative synthetic methods to prepare each pure stereoisomer independently, we are currently unable to determine which isomer corresponds to *E* or *Z* geometry. Nevertheless, the product was obtained as a mixture of the two alkene isomers with a ratio of 2.8 : 1.

Advice 16: Standardize notation of the trifluoromethylthio group (SCF_3 vs –SCF_3).

Response: We thank the reviewer for this suggestion. We have now standardized the notation of the trifluoromethylthio group throughout the manuscript as $–SCF_3$.

Advice 17: Correct minor typographical inconsistencies (e.g., bold font in yield for **10m**).

Response: We have carefully reviewed the manuscript and corrected the minor typographical inconsistencies, including the bold font for the yield of **10m** and any similar issues elsewhere.

Advice 18: Ensure proper alignment of CF_3 labels in figures (e.g., **8q**, **8r**).

Response: We have corrected the alignment of the CF_3 labels in the figures for compounds **8q** and **8r** to ensure proper presentation.

Advice 19: Use "chemical shift" instead of "chemical migration" (SI).

Response: We have corrected the terminology in the Supporting Information, replacing "chemical migration" with the standard term "chemical shift" throughout.

Advice 20: Supporting Information and Reproducibility: Specify NaH formulation (e.g., 60% dispersion in mineral oil). Include R_f values for all compounds. Resolve discrepancies in solvent reporting (e.g., toluene vs 1,4-dioxane in synthesis of **1n**).

Response: We thank the reviewer for these important practical suggestions. The following modifications have been made in the revised manuscript and Supporting Information:

NaH formulation: We used purified NaH (free-flowing powder) obtained by washing commercial 60% dispersion in mineral oil with anhydrous THF.

***R_f* values:** R_f values for all compounds have been added in the Supporting Information.

Solvent discrepancy: The synthesis of **1n** was re-examined; the correct solvent is toluene, and the inconsistent entry (previously 1,4-dioxane) has been corrected throughout the Supporting Information.

These revisions have been highlighted in the updated Supporting Information. We believe these changes improve the clarity and reproducibility of our experimental procedures.

Thank you very much for your kind consideration.

Best Regards.

Sincerely yours,

Ji-Chang Xiao
